# NOTCH2 disrupts the synovial fibroblast identity and the inflammatory response of epiphyseal chondrocytes

**DOI:** 10.1016/j.jbc.2025.110206

**Published:** 2025-05-08

**Authors:** Ernesto Canalis, Rosa Guzzo, Lauren Schilling, Emily Denker

**Affiliations:** 1 Department of Orthopaedic Surgery, UConn Health, Farmington, Connecticut, USA; 2 Department of Medicine, UConn Health, Farmington, Connecticut, USA; 3 UConn Musculoskeletal Institute, UConn Health, Farmington, Connecticut, USA; 4 Department of Neuroscience, UConn Health, Farmington, Connecticut, USA

**Keywords:** chondrocyte, inflammation, NOTCH2, Notch receptor, osteoarthritis, transcriptome, synovial fibroblast

## Abstract

Notch signaling plays a fundamental role in the inflammatory response and has been linked to the pathogenesis of osteoarthritis in murine models of disease and humans. To address how Notch signaling modifies transcriptomes and cell populations, we examined the effects of NOTCH2 in chondrocytes from mice harboring a NOTCH2 gain-of-function mutation (*Notch2*^*tm1.1Ecan*^) and a conditional NOTCH2 gain-of-function model expressing the NOTCH2 intracellular domain (NICD2) from the *Rosa26* locus (*R26-NICD2* mice). Bulk RNA-Sequencing (RNA-Seq) of primary epiphyseal cells from both gain-of-function models established increased expression of pathways associated with the phagosome, genes linked to osteoclast activity in rheumatoid arthritis signaling, and pulmonary fibrosis signaling. Expression of genes linked to collagen degradation was enhanced in *Notch2*^*tm1.1Ecan*^ cells, while genes related to osteoarthritis pathways were increased in NICD2-expressing cells. Single cell (sc)RNA-Seq of cultured *Notch2*^*tm1.1Ecan*^ cells revealed clusters of cells related to limb mesenchyme, chondrogenic cells, and fibroblasts, including articular synovial fibroblasts. Pseudotime trajectory revealed close associations among clusters in control cultures, but the cluster of articular/synovial fibroblasts was disrupted in cells from *Notch2*^*tm1.1Ecan*^ mice. ScRNA-Seq showed similarities in the cluster distributions and pseudotime trajectories of NICD2-expressing and control cells, except for altered progression in a cluster of NICD2-expressing cells. In conclusion, NOTCH2 enhances the activity of pathways associated with inflammation in epiphyseal chondrocytes and disrupts the transcriptome profile of articular/synovial fibroblasts.

Notch receptors (Notch 1–4) have an established role in the differentiation, fate and function of a variety of cell lineages and play an important regulatory role in the response to inflammation (1, 2). This function of Notch signaling has been associated with the pathogenesis of osteoarthritis, a chronic degenerative and inflammatory disease that affects multiple tissues of the joint including the synovium cartilage and bone (3, 4, 5, 6, 7, 8, 9). Although there is a degenerative component to osteoarthritis, the disease is a complex disorder affecting the joint and driven by a host of proinflammatory signals (7, 10, 11, 12). These are often released by immune cells infiltrating synovium although articular chondrocytes are also a source of inflammatory molecules that contribute to the pathogenesis of the disease (6, 12).

Notch receptors are activated following interactions with ligands of the Jagged and Delta-like families. These interactions lead to the proteolytic cleavage of Notch at the junction of its extracellular and transmembrane domains and to the subsequent release of the Notch intracellular domain (NICD) (1, 13). Following translocation to the nucleus, the NICD forms a complex with recombination signal-binding protein for Ig of κ (RBPJκ) and mastermind-like (MAML) to activate canonical signaling and induce target gene transcription (14, 15, 16, 17). *Notch1*, *2*, *3* and *4* transcripts are detected in chondrocytes; however, the expression of *Notch2* is greater than that of other Notch receptors in chondrogenic ATDC5 cell lines, epiphyseal and costal chondrocytes (4). Since the activity of each Notch receptor depends on the cellular environment where it is expressed, the findings make NOTCH2 the most likely Notch receptor with a function in cartilage tissue.

Sustained supraphysiological activation of Notch signaling is associated with the development of osteoarthritis and the suppression of chondrogenesis, and the deletion of either *Rbpj* or the Notch target gene *Hes1* prevents the osteoarthritis that follows the surgical destabilization of the medial meniscus (DMM) in mice (3, 4, 18, 19, 20, 21). Our laboratory created a knock-in mouse model harboring a *Notch2*^*6955C>T*^ mutation in exon 34 of *Notch2* and termed *Notch2*^*tm1.1Ecan*^ (22). The mutation replicates the pathogenic variant associated with Hajdu Cheney Syndrome (HCS) and leads to the premature termination of the protein product and the loss of the PEST domain, which is necessary for the proteasomal degradation of the NICD (23, 24, 25). As a result, the NOTCH2 NICD is stable, resulting in a gain-of-NOTCH2 function (22). In accordance with the proposed role of NOTCH2 in inflammation, *Notch2*^*tm1.1Ecan*^ mice are sensitized to the osteoarthritis that follows DMM surgeries and to the osteolytic actions of tumor necrosis factor α (TNFα) (23, 26, 27). Although one case of osteoarthritis in HCS has been reported, its association with the NOTCH2 gain-of-function was not established, and the prevalence of osteoarthritis cannot be estimated due to the rarity of the syndrome (28).

Chondrocytes are cells of mesenchymal origin present in cartilage tissue. The proliferation and differentiation of epiphyseal chondrocytes present in the growth plate lead to the longitudinal growth of bones (29, 30). Chondrocytes synthesize proteins that form the extracellular matrix of cartilage, including type II collagen, and proteoglycans like aggrecan. Chondrocytes from *Notch2*^*tm1.1Ecan*^ mice display increased expression of interleukin (IL) six and are sensitized to the actions of TNFα on the inflammatory response, an effect attributed to the activity of the NOTCH2 NICD (27, 30, 31). In an initial experiment, we demonstrated that in the presence of TNFα, chondrogenic cells from *Notch2*^*tm1.1Ecan*^ display enhanced phagosome formation and the osteoarthritis pathway (31). While these observations indicate that Notch signaling itself can influence the expression and activity of cytokines on the inflammatory response, the direct effects of NOTCH2 in the absence of TNFα and the cell transcriptome profile and mechanisms involved were not elucidated. Moreover, the transcriptome profile of the joint environment and of epiphyseal chondrocytes is heterogeneous, and as a consequence, the cell subtypes and genes responsible for the actions of NOTCH2 need to be defined at a single-cell resolution (32, 33).

To have a better understanding of the mechanisms responsible for the NOTCH2-dependent inflammatory response in the chondrocyte environment, the transcriptome profile affected by NOTCH2 at a global and single cell resolution in murine epiphyseal chondrocytes was investigated. For this purpose, transcriptomes of epiphyseal chondrocytes with chondroblast-like properties were obtained from newborn *Notch2*^*tm1.1Ecan*^ mice and from *R26-NICD2* conditional mice, where sequences coding for the NICD are cloned into the *Rosa26* locus downstream of a loxP flanked STOP cassette (34). Exposure of these cells to the Cre recombinase results in the expression of the NOTCH2 NICD and NOTCH2 activation. Epiphyseal chondrocytes from *Notch2*^*tm1.1Ecan*^ and *R26-NICD2* mice were examined using bulk as well as single cell (sc)RNA-Sequencing (Seq) approaches.

## Results

### The Notch2^tm1.1Ecan^ mutation inhibits chondrogenesis, enhances the phagosome formation, collagen degradation, and the role of the osteoclasts in rheumatoid arthritis pathway

**Figure 1:**
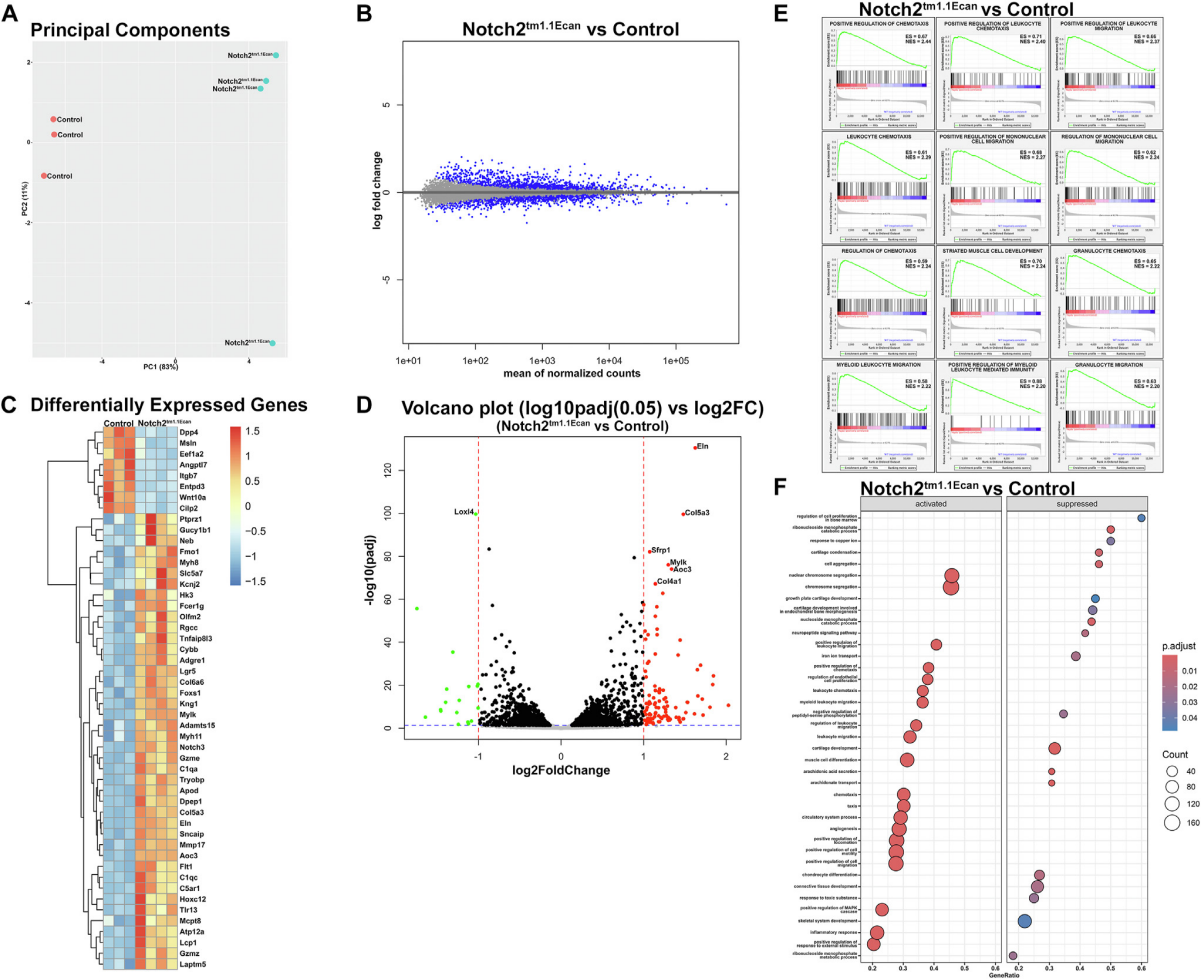
**RNA-Seq profile of *Notch2*^*tm1.1Ecan*^ chondrocytes.** Chondrocyte-enriched cells from newborn *Notch2*^*tm1.1Ecan*^ and control littermate mice were cultured to confluence and RNA was extracted and analyzed by RNA-Seq. *A*, principal component analysis of RNA-Seq technical replicates (n = 4 for *Notch2*^*tm1.1Ecan*^ and n = 3 for control). *B*, differentially expressed genes as log fold change over means of normalized counts. *C*, heat map of the 50 most differentially regulated genes between *Notch2*^*tm1.1Ecan*^ and control chondrocytes and *D*, corresponding volcano plots; log2 FoldChange, false discovery rate *p* adjusted value < 0.05 for *C* and *D*. *E*. Gene set enrichment analysis of biological pathways in epiphyseal chondrocytes from *Notch2*^*tm1.1Ecan*^ cells. Enrichment scores (ES) and normalized ES (NES) of pathways affected by NOTCH2 at a false discovery rate (FDR) of <0.05 are shown. *F*, visualization of gene enrichment analysis by clusterProfiler, as a lollipop chart. Gene count and pathways affected by *Notch2*^*tm1.1Ecan*^ at a *p* adjusted <0.05 are shown.

To determine the direct role of the *Notch2*^*tm1.1Ecan*^ mutation in epiphyseal chondrocytes, cells from newborn mice were cultured and RNA was extracted and analyzed by bulk RNA-Seq. Principal component analysis verified appropriate segregation between cells from control and *Notch2*^*tm1.1Ecan*^ mice. There were 123 ≥ log2Fold Change (FC)1 and *p* adjusted <0.05 differentially regulated genes between *Notch2*^*tm1.1Ecan*^ and control chondrocytes; 107 genes, including *Notch3* were upregulated and 16 were downregulated (Fig. 1, *A*–*D*). The 50 top dysregulated genes are shown in Figure S1. *Hes1* and *Hey1* were induced by a log2FC of 0.5 and *Notch3* by a log2FC of 1.4 in *Notch2*^*tm1.1Ecan*^ cells, confirming activation of Notch signaling and induction of *Notch3*. *Hey2* and *Heyl* were not detected. Biological pathways affected were analyzed by Gene Set Enrichment Analysis (GSEA) and by using the R Package clusterProfiler. These analyses revealed the inhibition of genes associated with chondrocyte differentiation and cartilage development, confirming the suppressive role of NOTCH2 in chondrogenesis and an enhancement of cell replication, chemotaxis, and migration (Fig. 1, *E* and *F*) (31).

**Figure 2:**
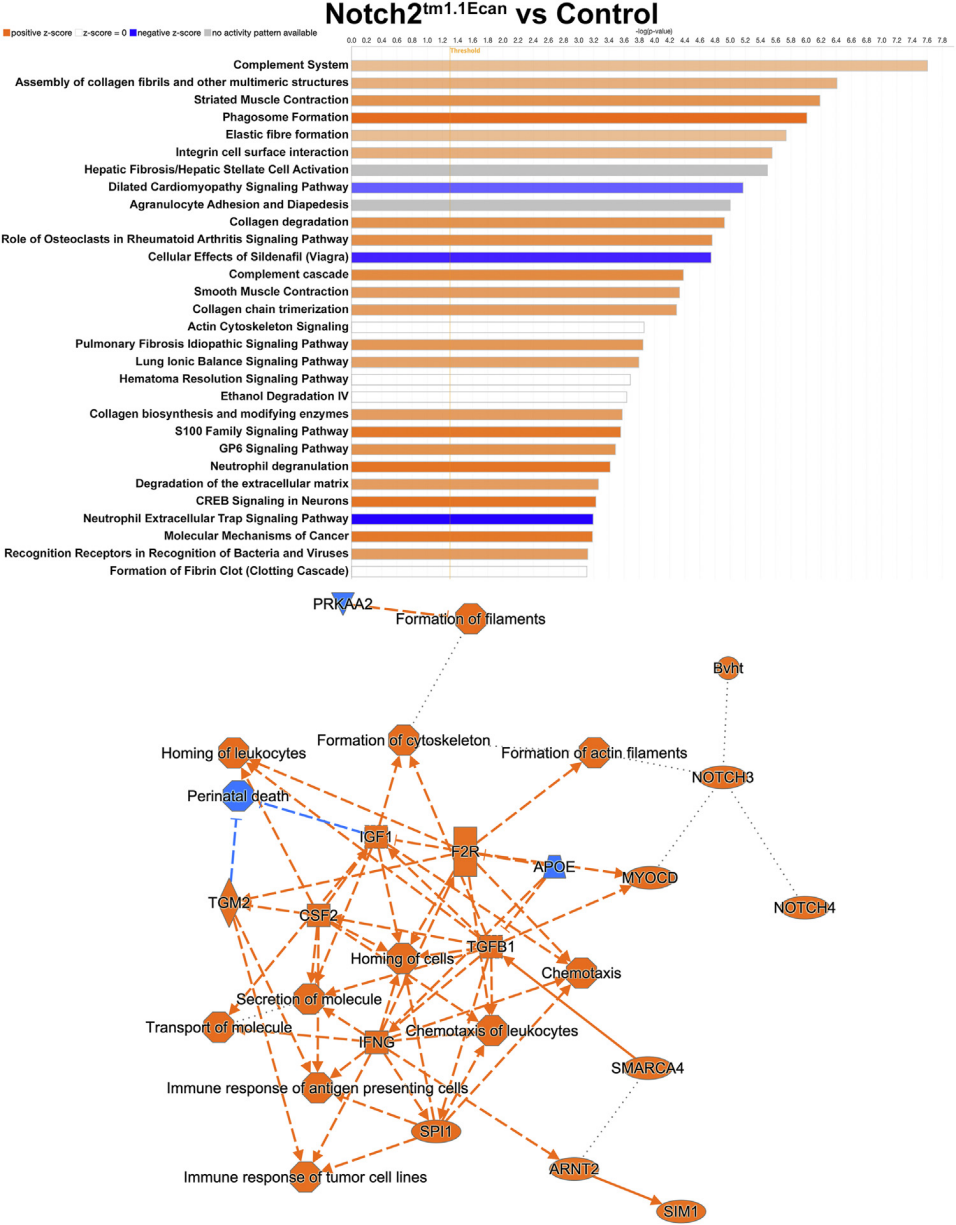
**The phagosome formation, collagen degradation, the role of osteoclasts in rheumatoid arthritis signaling and pulmonary fibrosis signaling are enhanced in chondrocytes from *Notch2*^*tm1.1Ecan*^ mice.** Chondrocyte-enriched cells from newborn *Notch2*^*tm1.1Ecan*^ and control littermate mice were cultured to confluence and RNA was extracted and processed for RNA-Seq. Ingenuity Pathway Analysis (IPA) of canonical pathways under the genes and chemicals category was performed. Genes affected by *Notch2*^*tm1.1Ecan*^ at log2FC1 *p* adjusted value < 0.1 are shown. Graphical summary of the data input showing activated (*orange*) and inhibited (*blue*) pathways. Solid lines lead to activation or inhibition, dotted lines point to inferred relationships, dashed lines to indirect and solid lines to direct interactions.

**Figure 3:**
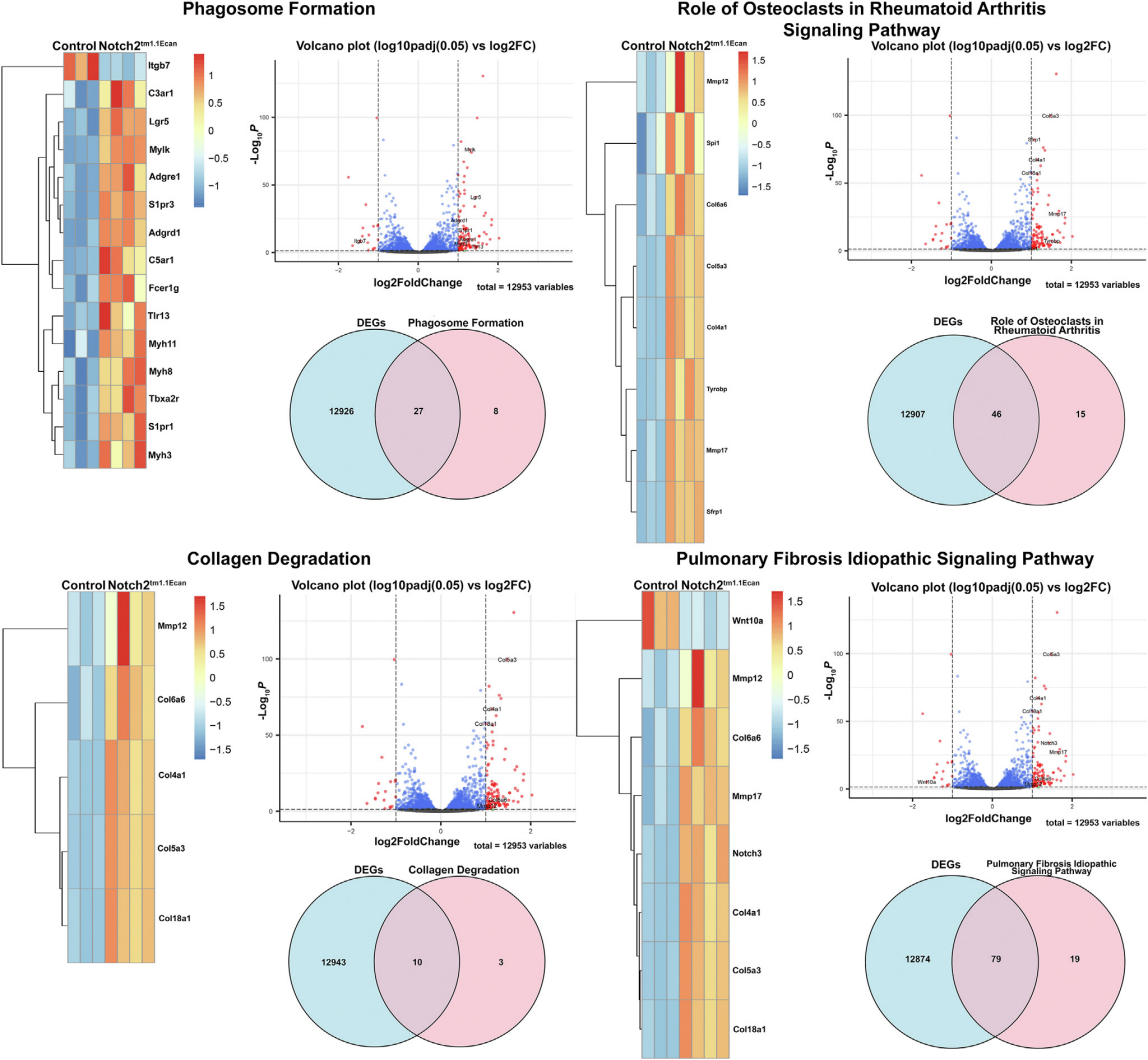
**The phagosome formation, collagen degradation, the role of osteoclasts in rheumatoid arthritis and pulmonary fibrosis signaling are enhanced in chondrocytes from *Notch2*^*tm1.1Ecan*^ mice.** Chondrocyte-enriched cells from newborn *Notch2*^*tm1.1Ecan*^ and control littermate mice were cultured to confluence and RNA was extracted and processed for RNA-Seq. Ingenuity Pathway Analysis (IPA) of canonical pathways under the genes and chemical category was performed. Genes affected by NOTCH2 at a log2FC1, *p* < 0.05, *p* adjusted value < 0.1 are shown. Heat maps, volcano plots and VENN diagrams of differentially expressed genes (DEG) between *Notch2*^*tm1.1Ecan*^ and control of the indicated signaling pathways are shown.

Ingenuity Pathway Analysis (IPA) of bulk RNA-Seq data revealed activation of phagosome formation, collagen degradation, and the role of osteoclasts in rheumatoid arthritis, and idiopathic fibrosis signaling pathways (Fig. 2). A graphical summary of multiple pathways and molecules affected by the *Notch2*^*tm1.1Ecan*^ mutation and their interrelationships is shown in Figure 2 (lower panel). Previously, we documented that the phagosome formation and osteoarthritis pathway were influenced in chondrocytes from the *Notch2*^*tm1.1Ecan*^ mice in the context of TNFα exposure (31). Whereas the phagosome formation was enhanced, the osteoarthritis pathway was not substantially modified by the *Notch2*^*tm1.1Ecan*^ mutation in the absence of TNFα (Fig. 2). Heat maps and volcano plots of the pathways affected by the *Notch2*^*tm1.1Ecan*^ mutation are shown in Figure 3, and VENN diagrams revealed that a significant number of genes present in the pathways described were affected by the NOTCH2 gain-of-function.

### NOTCH2 activation enhances pulmonary fibrosis signaling and osteoarthritis pathways, phagosome formation, and rheumatoid arthritis signaling in epiphyseal chondrocytes

**Figure 4:**
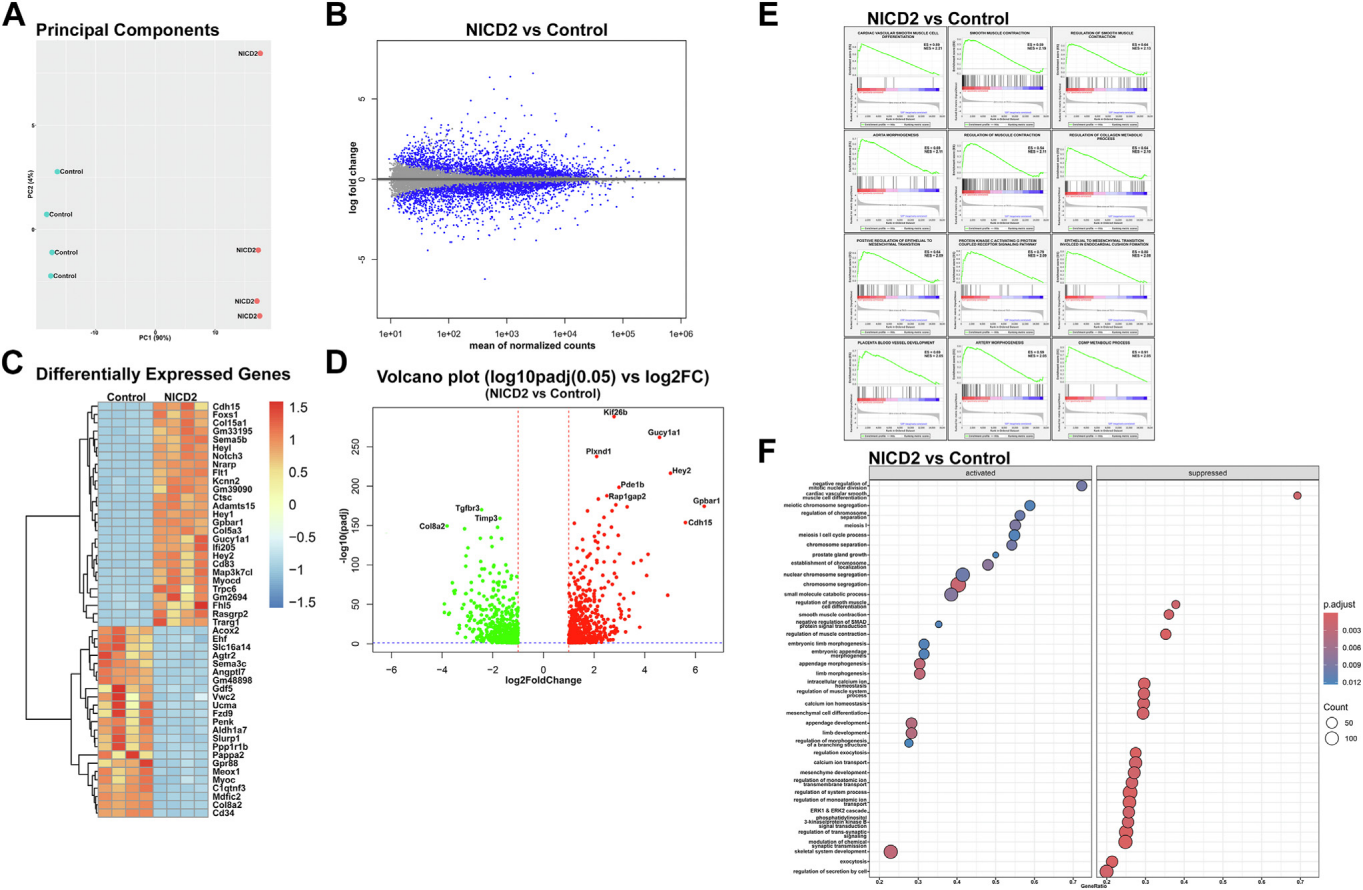
**RNA-Seq profile of NOTCH2 activated chondrocytes.** Chondrocyte-enriched cells from newborn *R26-NICD2* mice were cultured to ∼70% confluence and transfected with Ad-CMV-Cre to induce NOTCH2 NICD or Ad-CMV-GFP as a control and cultured for 24 h. Cells were collected, and total RNA was extracted and analyzed by RNA-Seq. *A*, principal component analysis of RNA-Seq technical replicates (n = 4) for NICD2 and control. *B*, differentially expressed genes as log fold change over means of normalized counts. *C*, heat map of the 50 most differentially regulated genes between NICD2 and control chondrocytes. *D*, corresponding volcano plots; log2FC, false discovery rate *p* adjusted value < 0.05 for *C* and *D*. *E*, gene set enrichment analysis of biological pathways in epiphyseal chondrocytes from activated NICD2 cells. Enrichment scores (ES) and normalized ES (NES) of pathways affected by activated NOTCH2 NICD at a false discovery rate (FDR) of <0.05 are shown. *F*, visualization of gene enrichment analysis by clusterProfiler, as a lollipop chart. Gene count and pathways affected by NICD2 at a *p* adjusted <0.05 are shown.

To establish the role of the NOTCH2 NICD in epiphyseal chondrocytes, cells from *R26-NICD2* mice were transfected with an adenoviral vector expressing Cre recombinase under the control of the cytomegalovirus promoter (Ad-CMV-Cre) so that the biologically active NOTCH2 NICD would be expressed in the cell environment. Control cultures were transfected with an analogous vector expressing green fluorescent protein (GFP), termed Ad-CMV-GFP. RNA extracted from epiphyseal chondrocytes was examined by bulk RNA-Seq analysis. Principal component analysis revealed good segregation between control and NOTCH2 NICD-expressing samples. There were 913 ≥ log2FC1, *p* adjusted <0.05 differentially regulated genes between NICD2-expressing and control chondrocytes; 468 genes were upregulated, including *Hes1*, *Hey1*, *Hey2*, and *Heyl* and 445 were downregulated by NICD2 (Fig. 4, *A*–*D*). The 50 top dysregulated genes are shown in Figure S2 and included *Hey1*, *Hey2*, and *Heyl* demonstrating activation of canonical signaling by the NICD2. *Notch3* was increased by a log2FC 4.1 confirming the *Notch3* induction by Notch signaling observed in *Notch2*^*tm1.1Ecan*^ cells. Biological pathways analyzed by GSEA and the R Package clusterProfiler revealed inhibition of genes associated with mesenchymal and smooth muscle cell differentiation and contraction and muscle development, calcium ion transport, ERK1 and ERK2 signaling and with the activation of negative Smad signal regulation. The epithelial to mesenchymal transition, collagen metabolic processes and activation of limb development and morphogenesis were enhanced in NICD2-expressing chondrocytes (Fig. 4, *E* and *F*).

**Figure 5:**
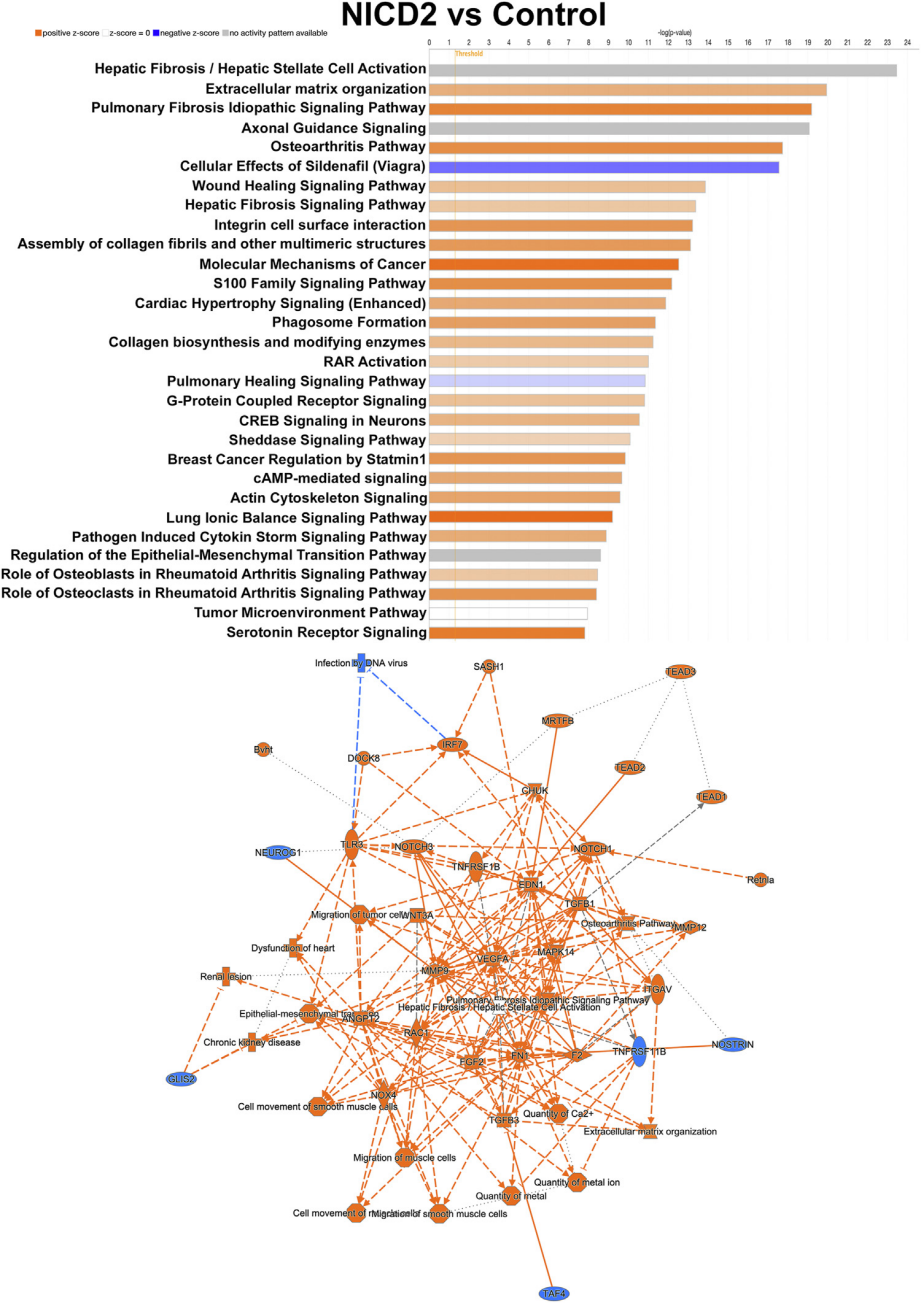
**The pulmonary fibrosis idiopathic signaling, osteoarthritis pathway, phagosome formation, and role of osteoclasts in rheumatoid arthritis are enhanced in NOTCH2 activated chondrocytes.** Chondrocytes from newborn *R26-NICD2* mice were cultured to 70% confluence and transfected with Ad-CMV-Cre (NOTCH2 activated) or Ad-CMV-GFP (control) and cultured. Cells were collected and total RNA was extracted and analyzed by RNA-Seq. Ingenuity Pathway Analysis (IPA) of canonical pathways under the genes and chemical category was performed. Genes affected by NOTCH2 at a log2FC1, *p* < 0.05, *p* adjusted of 0.1 are shown. Graphical summary of the data input showing activated (*orange*) and inhibited (*blue*) pathways. Solid lines lead to activation or inhibition, dotted lines point to inferred relationships, dashed lines to indirect, and solid lines to direct interactions.

**Figure 6:**
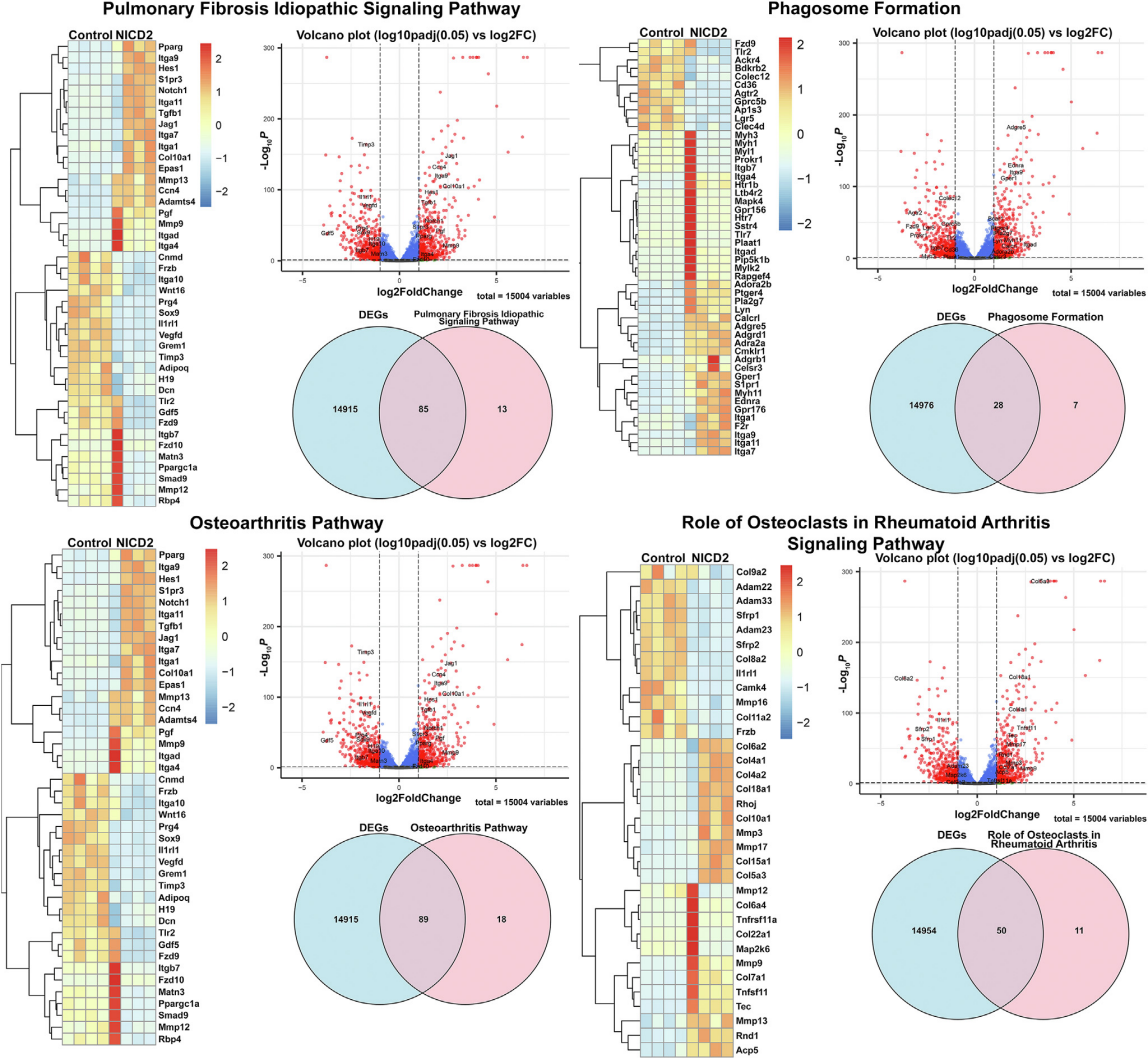
**NOTCH2 activation induces pulmonary fibrosis idiopathic signaling, the osteoarthritis pathway, phagosome formation, and the role of osteoclasts in rheumatoid arthritis pathway in chondrocytes.** Chondrocyte-enriched cells from newborn *R26-NICD2* mice were cultured to ∼70% confluence and transfected with Ad-CMV-Cre to activate NOTCH2 or Ad-CMV-GFP as a control and cultured for an additional 24 h. Cells were collected, and total RNA was extracted and analyzed by RNA-Seq. Ingenuity Pathway Analysis (IPA) of canonical pathways under the genes and chemical category was performed. Genes affected by NOTCH2 at a log2FC1, *p* < 0.05, *p* adjusted value < 0.1 are shown. Heat maps, volcano plots, and VENN diagrams of differentially expressed genes (DEG) between NICD2 and control of the individualized signaling pathways are shown.

IPA of bulk RNA-Seq data revealed activation of the pulmonary fibrosis signaling and osteoarthritis pathway, the phagosome formation, and the role of osteoclasts in rheumatoid arthritis signaling pathway in NICD2 expressing cells (Fig. 5). A graphical summary of multiple pathways and molecules and their interactions in the context of NICD2 activation is shown in Figure 5 (lower panel). Heat maps and volcano plots of the pathways affected by the NICD2 are shown in Figure 6, and VENN diagrams revealed that a significant number of genes influenced by NOTCH2 were involved in the indicated pathways, confirming the role of NOTCH2 in signals related to inflammation.

### scRNA-Seq of epiphyseal chondrocytes from Notch2^tm1.1Ecan^ chondrocytes reveals disruption of the articular/synovial transcriptome

**Figure 7:**
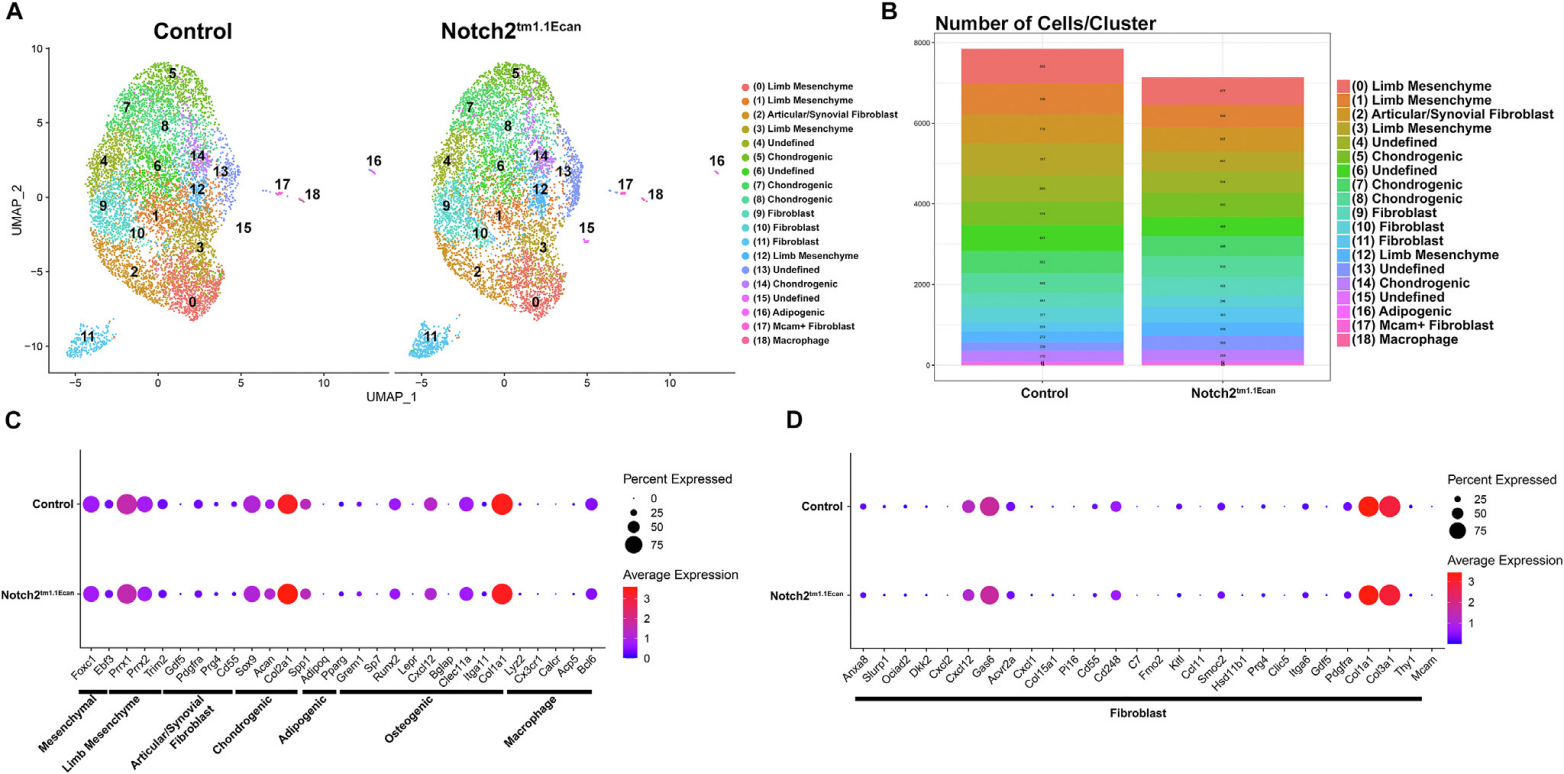
**Uniform manifold approximation and projection (UMAP) for dimension reduction of scRNA-Seq data of epiphyseal chondrocytes from *Notch2*^*tm1.1Ecan*^ and control newborn mice reveal modest alterations in cell clusters.***A*, UMAP visualization of 19 cell clusters of normalized independent data from epiphyseal chondrocytes from *Notch2*^*tm1.1Ecan*^ and control littermate newborn mice cultured to confluence. *B*, bar graph demonstrates the cell distribution present in each individual cluster from *Notch2*^*tm1.1Ecan*^ and control cells. Dot plot displaying the differential expression of genes in control and in *Notch2*^*tm1.1Ecan*^ cells associated in (*C*) with mesenchymal cells, limb mesenchyme articular/synovial, chondrogenic, adipogenic and osteogenic cells and macrophages, and in (*D*) with fibroblast cell clusters present in epiphyseal chondrocytes. *Red* denotes higher and blue denotes lower than average expression, and the size of the circle represents the percentage of cells expressing each gene. The control culture used for the UMAP visualization was shared with a study on the effect of TNFα in chondrocytes (33).

Epiphyseal chondrocytes from control and *Notch2*^*tm1.1Ecan*^ expressing cells were processed in a Chromium iX using a 3′ library kit (10x Genomics). An estimated 8120 control and 7365 *Notch2*^*tm1.1Ecan*^ cells were recovered representing a 75% and 69% recovery, respectively and 24,320 genes were detected in control and 24,747 in *Notch2*^*tm1.1Ecan*^ cells. Following normalization, cells with ≥10% mitochondrial RNA, <250 transcripts and >10,000 genes/cells were excluded. Normalization and doublet filtering and exclusion reduced the number of cells to be analyzed to 7845 control and 7141 *Notch2*^*tm1.1Ecan*^ cells. Clustering analysis of epiphyseal chondrocytes (pooled data from control and *Notch2*^*tm1.1Ecan*^ cells) using the uniform manifold approximation and projection (UMAP) non-linear dimensionality reduction algorithm, accurately distinguished 19 different cell clusters (Fig. 7, Table 1). This is consistent with clusters found in studies reporting the effect of TNFα in epiphyseal chondrocytes, sharing the same control cell population (33). The more prevalent clusters were constituted by cells with a transcriptome profile of chondrogenic cells, limb mesenchyme and fibroblasts. An articular/synovial cluster was identified by the expression of *Prg4* and *Pdgfra* and its cellularity was more sparse in *Notch2*^*tm1.1Ecan*^ than in control cells (Fig. 7, Tables 1 and S1). NOTCH2 was the prevalent Notch receptor and *Notch2* transcripts were detected in all cell clusters (Fig. S3). *Notch1* was detected but only low levels of *Notch3* and *Notch4* expression were found. The canonical Notch target gene *Hes1* was present in all cell clusters, and its level of expression was substantially higher than that of *Hey1*, *Hey2* and *Heyl* (Fig. S3). There was a modest effect of the *Notch2*^*tm1.1can*^ mutation on cluster distribution and on the expression of gene profiles used to classify the 19 cell clusters (Fig. 7, Tables 1 and S2).

**Table 1:** Cell cluster distribution of epiphyseal chondrocytes from control and *Notch2*^*tm1.1Ecan*^ mice

A.	Cell number/Cluster		% Cell/Cluster		Cluster percent		Cluster total
Cluster	Control	*Notch2*^*tm1.1Ecan*^	Control	*Notch2*^*tm1.1Ecan*^	Control	*Notch2*^*tm1.1Ecan*^	
Limb mesenchyme	862	677	10.99%	9.48%	5.75%	4.52%	10.27%
Limb mesenchyme	766	566	9.76%	7.93%	5.11%	3.78%	8.89%
Articular/Synovial	716	587	9.13%	8.22%	4.78%	3.92%	8.69%
Limb mesenchyme	787	491	10.03%	6.88%	5.25%	3.28%	8.53%
Undefined	665	544	8.48%	7.62%	4.44%	3.63%	8.07%
Chondrogenic	574	592	7.32%	8.29%	3.83%	3.95%	7.78%
Undefined	641	489	8.17%	6.85%	4.28%	3.26%	7.54%
Chondrogenic	552	495	7.04%	6.93%	3.68%	3.30%	6.99%
Chondrogenic	503	510	6.41%	7.14%	3.36%	3.40%	6.76%
Fibroblast	341	455	4.35%	6.37%	2.28%	3.04%	5.31%
Fibroblast	377	296	4.81%	4.15%	2.52%	1.98%	4.49%
Fibroblast	223	383	2.84%	5.36%	1.49%	2.56%	4.04%
Limb bud	272	328	3.47%	4.59%	1.82%	2.19%	4.00%
Undefined	209	353	2.66%	4.94%	1.39%	2.36%	3.75%
Chondrogenic	270	263	3.44%	3.68%	1.80%	1.75%	3.56%
Undefined	27	63	0.34%	0.88%	0.18%	0.42%	0.60%
Adipogenic	27	15	0.34%	0.21%	0.18%	0.10%	0.28%
Mcam + Fibroblast	18	21	0.23%	0.29%	0.12%	0.14%	0.26%
Macrophage	15	13	0.19%	0.18%	0.10%	0.09%	0.19%
Total	7845	7141			52.35%	47.65%	100.00%

Epiphyseal chondrocytes from *Notch2^tm1.1Ecan^* and control littermates were cultured to confluence. RNA-Seq data were aligned to the mouse genome and gene and cell counting and cluster distribution were performed using Cell Ranger and Seurat after normalization. Cell number/cluster, % cells/cluster and cluster percent distribution are shown for epiphyseal chondrocytes from control and experimental cells.

**Figure 8:**
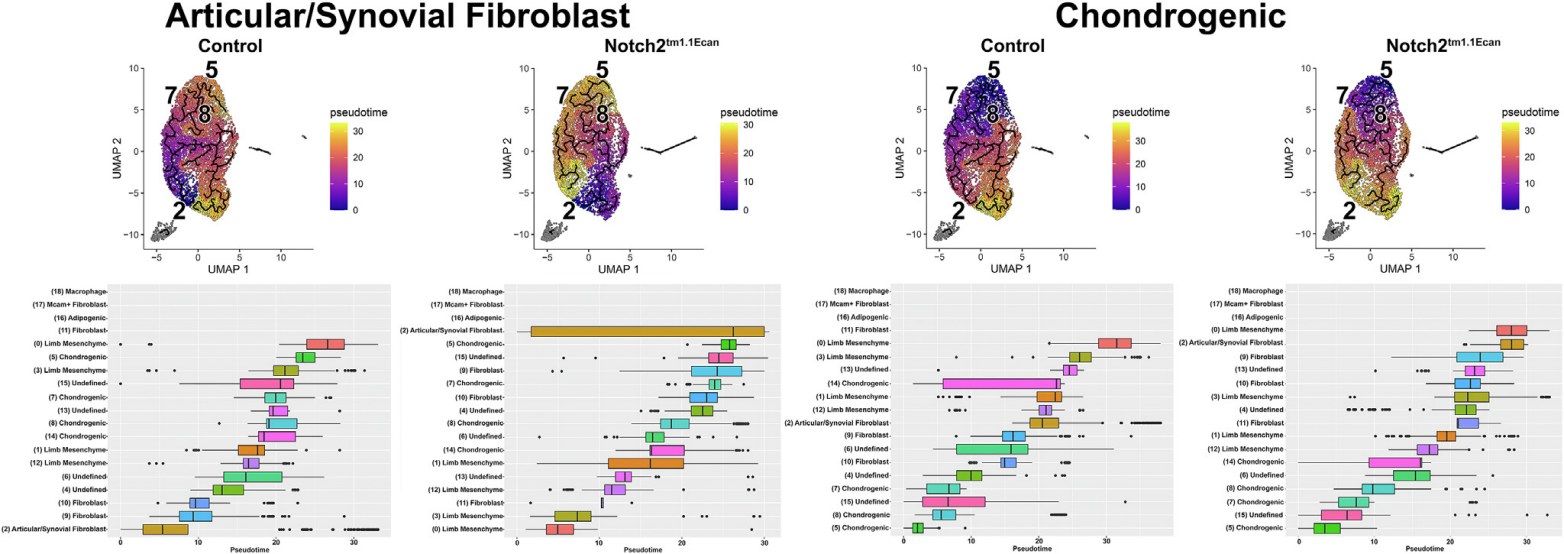
**Trajectory and pseudotime analysis reveal that the progression of articular/synovial fibroblasts is disrupted in epiphyseal chondrocytes from *Notch2*^*tm1.1Ecan*^ mice.** Pseudotime analysis of 19 cellular clusters from *Notch2*^*tm1.1Ecan*^ and control chondrocytes identified by uniform manifold approximation and projection (UMAP). On the *left*, using the articular/synovial fibroblast cluster and on the *right* using the chondrogenic cluster as a root node. An alternative visualization of pseudotime as a progression boxplot of pseudotime values is shown. The control culture used for the UMAP visualization and pseudotime analysis was shared with a study on the effect of TNFα in chondrocytes (33).

Pseudotime trajectory findings were constructed with Monocle 3 and used to predict the differentiation trajectory among clusters present in epiphyseal chondrocytes. Monocle 3 defines the trajectories and performs the pseudotime analysis applying an algorithm to learn the sequence of gene expression changes placing each cell at its proper position in the trajectory. The differential analysis toolkit was used to find genes that change as a function of pseudotime. Branched trajectories correspond to cellular “decisions” and were analyzed in the trajectories of control and experimental cultures. Pseudotime trajectory analysis selecting clusters expressing articular/synovial fibroblasts or chondrogenic cells as a root node revealed an association and good progression among the clusters from control cells (Fig. 8). In contrast, the articular/synovial fibroblast cluster from *Notch2*^*tm1.1Ecan*^ cells was fragmented, indicating an interruption of the ordinate cluster progression. IPA of canonical pathways conducted in the articular/synovial cell cluster revealed greater enhancement in the osteoarthritis pathways in cells from *Notch2*^*tm1.1Ecan*^ mice (Fig. S4). Gene analysis of the 50 mostly expressed genes in the articular/synovial cluster from *Notch2*^*tm1.1Ecan*^ cultures revealed that *Pla1a*, *Pla2g2e* and *Dpt* were upregulated ≥25% and *Clec3b*, *Dpp4*, *Agtr2*, *Pdzk1p1*, *Col6a6*, *Cd55*, *Adgrd1* and *Xdh* were downregulated ≥25% and these genes have been associated with inflammatory conditions (Table S3) (35, 36, 37, 38).

### scRNA-Req of epiphyseal chondrocytes from NICD2-expressing cells reveals modest alterations in cluster distribution and progression

**Figure 9:**
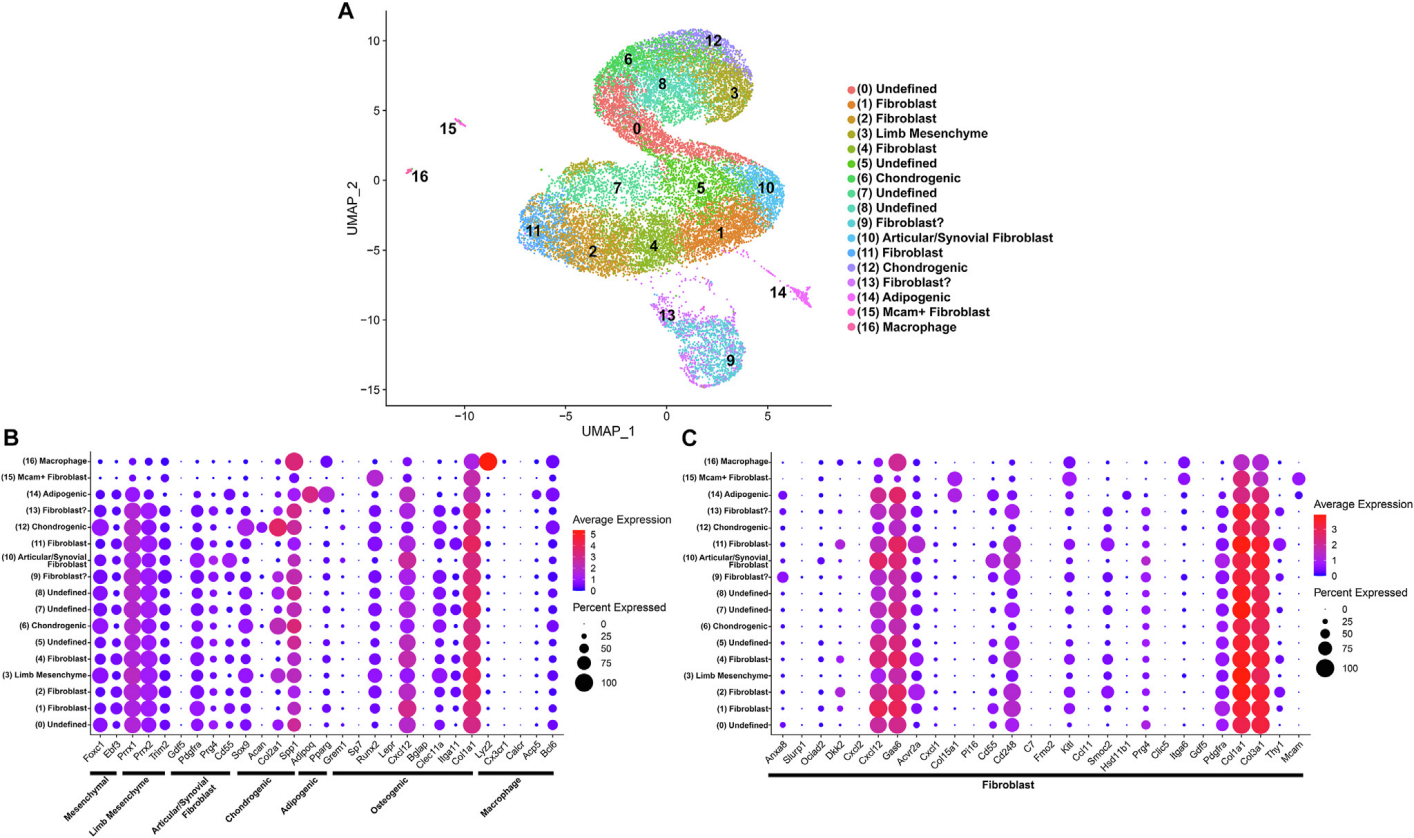
**Uniform manifold approximation and projection (UMAP) for dimension reduction of scRNA-Seq data of control and NOTCH2 activated epiphyseal chondrocytes from newborn mice identify cell clusters.***A*, UMAP visualization of cell clusters of normalized pooled data from epiphyseal chondrocytes from newborn *R26-NICD2* mice cultured to ∼70% confluence and transfected with either Ad-CMV-Cre (NOTCH2 activation) or Ad-CMV-GFP (control) and cultured for an additional 24-48 h. Dot plot displaying the expression of genes (*B*) associated with mesenchymal cells, limb mesenchyme, articular/synovial cells, chondrogenic, adipogenic, and osteogenic cells and macrophages and (*C*) associated with fibroblasts both in 17 cellular clusters from epiphyseal chondrocytes from newborn mice identified by UMAP. *Red* denotes higher and *blue* denotes lower than average expression, and the size of the circle represents the percentage of cells expressing each gene.

**Figure 10:**
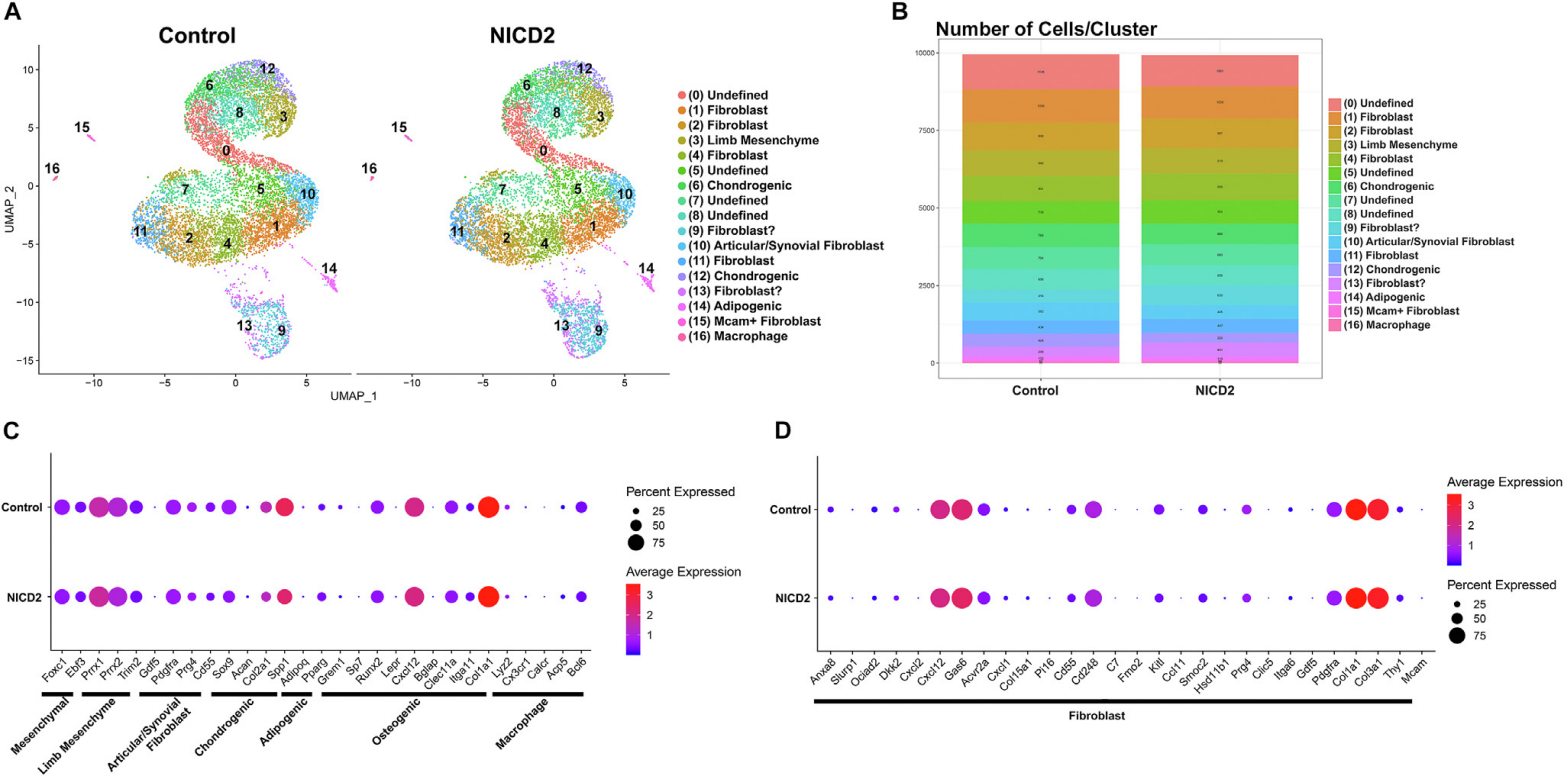
**Uniform manifold approximation and projection (UMAP) for dimension reduction of scRNA-Seq data reveals no differences in cell clusters from control and NOTCH2 activated epiphyseal chondrocytes from newborn mice.***A*, UMAP visualization of cell clusters of normalized independent data from epiphyseal chondrocytes from newborn *R26-NICD2* mice cultured to ∼70% confluence and transfected with either Ad-CMV-Cre (NOTCH2 activation) or Ad-CMV-GFP (control) and cultured for an additional 24-48 h. *B*, bar graph demonstrating the cell distribution present in each individual cluster from control and NICD2-expressing cells. Dot plot displaying the expression of genes associated (*C*) with mesenchymal cells, limb mesenchyme, articular/synovial cells, chondrogenic, adipogenic and osteogenic cells and macrophages, and (*D*) with fibroblasts in 17 cellular clusters from epiphyseal chondrocytes from newborn mice identified by UMAP. *Red* denotes higher and *blue* denotes lower than average expression, and the size of the circle represents the percentage of cells expressing each gene.

Epiphyseal chondrocytes from *R26-NICD2* transfected with Ad-CMV-GFP (control) or Ad-CMV-Cre (NICD2-expressing) cells were processed in a Chromium iX using a 3′ library kit. An estimated 10,049 control and 10,077 NICD2-expressing cells were recovered, representing an ∼60% recovery and 25,581 and 25,468 genes in control and NICD2-expressing cells detected. Following normalization, using the same criteria described for *Notch2*^*tm1.1Ecan*^ and control cells, the number of cells analyzed was reduced to 9957 control and 9924 NICD2-expressing cells (Table 2). Cluster analysis of epiphyseal chondrocytes (pooled data from control and NICD2-expressing cells) using the UMAP linear dimensional reduction algorithm, identified 17 different cell clusters (Fig. 9, Table 2). NOTCH2 was the prevalent Notch receptor and *Hes1* the prevalent target gene detected in the various clusters and there was greater expression of *Notch2* and of the Notch target genes *Hes1*, *Hey1*, *Hey2* and *Heyl* in NICD2-expressing than in control cells (Fig. S3), confirming activation of Notch signaling. Selected clusters were constituted by cells with a transcriptome profile of chondrogenic cells, limb mesenchyme, and articular/synovial cells, although the more prevalent clusters exhibited a gene profile associated with fibroblasts (Tables 2 and S4). UMAP linear dimensional reduction did not reveal substantial differences in cluster distribution between control and NICD2-expressing cells (Fig. 10, Tables 2 and S5).

**Table 2:** Cell cluster distribution of epiphyseal chondrocytes from control and NICD2-expressing cells

B.	Cell number/Cluster		% Cell/Cluster		Cluster percent		Cluster total
Cluster	Control	NICD2	Control	NICD2	Control	NICD2	
Undefined	1136	1001	11.4%	10.1%	5.71%	5.03%	10.75%
Fibroblast	1056	1030	10.6%	10.4%	5.31%	5.18%	10.49%
Fibroblast	898	967	9.0%	9.7%	4.52%	4.86%	9.38%
Limb bud	848	819	8.5%	8.3%	4.27%	4.12%	8.38%
Fibroblast	801	859	8.0%	8.7%	4.03%	4.32%	8.35%
Undefined	718	751	7.2%	7.6%	3.61%	3.78%	7.39%
Chondrogenic	766	662	7.7%	6.7%	3.85%	3.33%	7.18%
Undefined	704	693	7.1%	7.0%	3.54%	3.49%	7.03%
Undefined	656	635	6.6%	6.4%	3.30%	3.19%	6.49%
Fibroblast?	414	635	4.2%	6.4%	2.08%	3.19%	5.28%
Articular/synovial	582	445	5.8%	4.5%	2.93%	2.24%	5.17%
Fibroblast	434	447	4.4%	4.5%	2.18%	2.25%	4.43%
Chondrogenic	424	325	4.3%	3.3%	2.13%	1.63%	3.77%
Fibroblast?	298	451	3.0%	4.5%	1.50%	2.27%	3.77%
Adipogenic	132	113	1.3%	1.1%	0.66%	0.57%	1.23%
Mcam + Fibroblast	57	59	0.6%	0.6%	0.29%	0.30%	0.58%
Macrophage	33	32	0.3%	0.3%	0.17%	0.16%	0.33%
Total	9957	9924			50.08%	49.92%	100.00%

Epiphyseal chondrocytes from *R26-NICD2* mice were cultured to 70% confluence and transfected with Ad-CMV-Cre (expressing NICD2) and Ad-CMV-GFP (control) and cultured for an additional 24-48 h period. RNA-Seq data were aligned to the mouse genome and gene and cell counting and cluster distribution were performed using Cell Ranger and Seurat after normalization. Cell number/cluster, % cells/cluster and cluster percent distribution are shown for epiphyseal chondrocytes from control and experimental cells.

**Figure 11:**
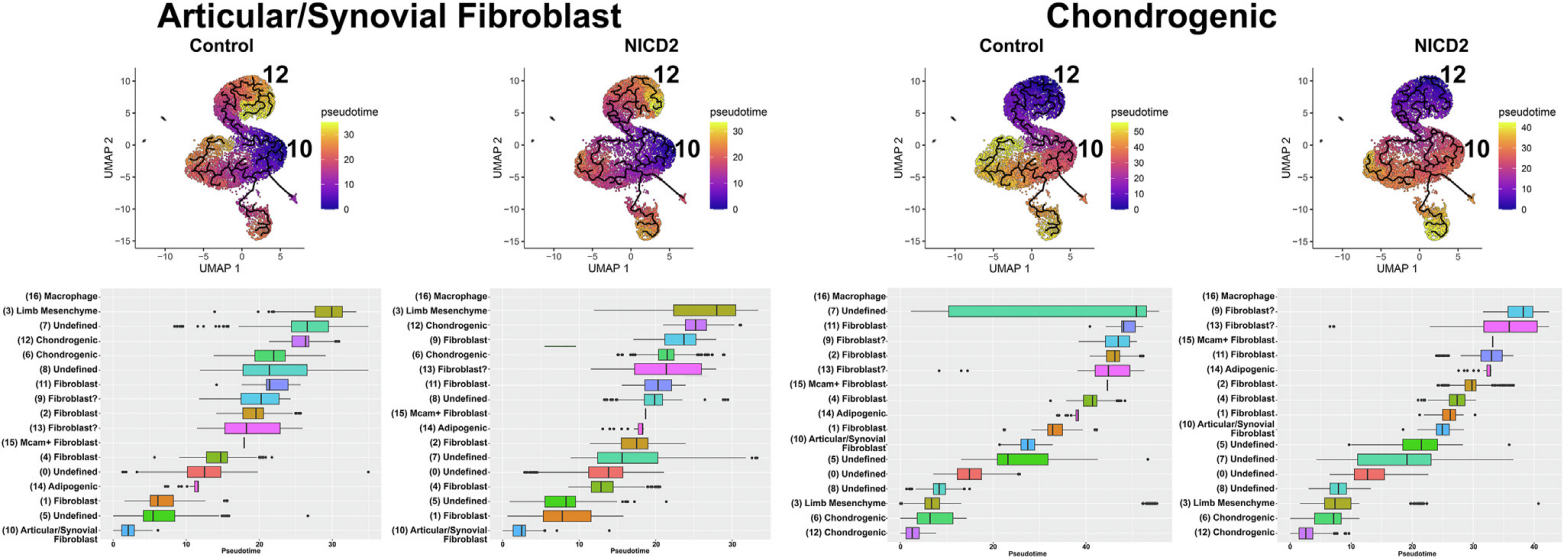
**Trajectory and pseudotime analysis reveal a redirection in the progression of articular/synovial fibroblasts by NOTCH2.** Trajectory and pseudotime analysis using Monocle 3, was performed in epiphyseal chondrocytes from *R26-NICD2* mice cultured to ∼70% confluence transfected with either Ad-CMV-Cre (NOTCH2 activated) or Ad-CMV-GFP (control) and cultured for 24-48 h. Pseudotime trajectory analysis of 17 cellular clusters from control and NICD2-expressing cells identified by uniform manifold approximation and projection (UMAP). On the *left* using articular/synovial fibroblast cluster and on the *right* using the chondrogenic cluster as a root node. An alternative visualization of the pseudotime UMAP as a progression boxplot of pseudotime values is shown.

Pseudotime trajectory findings, constructed with Monocle 3, were conducted selecting the articular/synovial fibroblasts or the chondrogenic cluster as a root node. In accordance with the results in control and *Notch2*^*tm1.1Ecan*^ cells, it demonstrated an association between clusters and good progression among the clusters from control cells (Fig. 11). In contrast to results obtained with *Notch2*^*tm1.1Ecan*^ cells, the expression of the NICD2 did not alter the pseudotime trajectory of articular/synovial fibroblasts although it modified the trajectory of an indeterminate or ill-identified cluster (Fig. 11). Genes modified by NICD2 in this cluster are shown in Table S6.

## Discussion

Previous work demonstrated that a NOTCH2 gain-of-function mutation sensitizes mice to the development of arthritis following DMM surgeries and to the osteolytic actions of TNFα (26, 27, 31). The present study extends previous findings demonstrating that NOTCH2 enhances the inflammatory response induced by TNFα in epiphyseal chondrocytes. It explores the direct effects of a mutation harbored by *Notch2*^*tm1.1Ecan*^ mice, causing a NOTCH2 gain-of-function and the induction of the NICD2 in epiphyseal chondrocytes. The transcriptome profile of epiphyseal chondrocytes was examined at the bulk and single-cell resolution. scRNA-Seq and bulk RNA-Seq were used as complementary approaches. Bulk RNA-Seq was used to provide a general landscape of the transcriptome profile and signaling pathways affected by NOTCH2, whereas scRNA-Seq was useful in the analysis of the transcriptome profile related to individual cell populations present in epiphyseal chondrocytes and their influence by NOTCH2.

Bulk RNA-Seq confirmed that NOTCH2 enhanced signaling pathways associated with the inflammatory response, arthritis, fibrosis, and collagen degradation. Activation of similar pathways was found in chondrocytes from *Notch2*^*tm1.1Ecan*^ mutants and from cells expressing the NICD2. This would indicate that the changes observed were a direct result of an effect of the NOTCH2 ICD and are in agreement with previous observations showing that the NICD2 replicates the effects of the NOTCH2 gain-of-function harbored by *Notch2*^*tm1.1Ecan*^ cells (31). Although Notch target genes associated with the activation of canonical signaling, such as *Hes1*, *Hey1*, *Hey2*, and *Heyl* were induced by NOTCH2, this does not exclude possible additional interactions of the NICD with non-canonical signals that could influence the response to inflammation.

The present work confirms that NOTCH2 is the prevalent Notch receptor expressed by cells present in cartilage (4). Whereas *Notch1* transcripts were detectable, the level of *Notch3* and *Notch4* expression was low, suggesting that, under basal conditions, these Notch receptors do not play a physiological or pathological role in this tissue environment. However, *Notch3* transcripts were induced by the NOTCH2 gain-of-function, and as a consequence, NOTCH3 could contribute to the phenotype observed. The results confirm the induction of *Notch3* by Notch signaling, an effect that has been attributed to the presence of RBPJᴋ binding sites in intron two of the *Notch3* gene (39, 40). Since *Hes1* is the canonical target gene mostly expressed in chondrocytes, it likely plays a role in mediating Notch effects in this cell environment. This would be in agreement with previous work demonstrating important roles of HES1 and HES5 in chondrogenesis and cartilage development and of HES1 in preclinical models of osteoarthritis (21, 41). Therefore, it is likely that HES1 is responsible for the actions of NOTCH2 in cartilage, as it has been shown for the effects of NOTCH2 in osteoclastogenesis (42).

scRNA-Seq analysis confirmed cellular heterogeneity in epiphyseal chondrocyte cultures and also observed in the epiphyseal growth plate and articular cartilage (32, 33, 43, 44, 45). The cell composition of each identified cluster is defined by its transcriptome profile and is not homogeneous making the identity of each cluster not absolute. Indeed, there is a continuum among cells from epiphyseal chondrocytes and pseudotime trajectory analysis revealed an association between clusters expressing gene markers identified in fibroblasts and chondrogenic cells. Some clusters could not be identified with certainty since they were constituted by genes expressed in multiple cell lineages. This was particularly evident in the experiment analyzing the effect of the NICD2, making the impact of the NICD2 on cluster distribution and progression more difficult to assess.

Cluster distribution and its potential progression were affected in cells from *Notch2*^*tm1.1Ecan*^ mice. The articular/synovial cluster was fragmented in *Notch2*^*tm1.1Ecan*^ cells, revealing an interruption in the normal cluster trajectory. Cells spread along a continuum according to their expression profile, and alterations in the pseudotime trajectory may represent gains or losses along a cell lineage or changes in gene expression (46, 47). These alterations in the trajectory and pseudotime often represent crucial decision points in a biological process, such as cell lineage commitment or disease onset. This is an important finding since alterations in pseudotime trajectories across conditions may represent gains or losses along a cell lineage, changes in the abundance of cells along a cell lineage, or in gene expression itself along the pseudotime across conditions (48). Cluster distribution and pseudotime trajectory did not reveal changes in the articular/synovial cluster from NICD2-expressing cells. The difference between these results and those obtained with cells from *Notch2*^*tm1.1Ecan*^ mice may reflect differences between treatment and control, naturally occurring sample-level variations, unwanted technical variations, and uncertainties in the inferred trajectory and pseudotime. The latter is possible because cluster identity was often not as clear in cultures from NICD2-expressing cells as it was in cultures from *Notch2*^*tm1.1Ecan*^ mice.

Analysis of genes influenced by the *Notch2*^*tm1.1Ecan*^ mutation in the articular/synovial fibroblast cluster revealed dysregulation of genes that have been associated with inflammation (35, 36, 37, 38). This would suggest that dysregulation of these genes could be responsible to some extent for the effects of NOTCH2. Since most of the genes affected were downregulated, the effect could be mediated by HES1, a known inhibitor of transcription (49, 50, 51).

A limitation of this work is the fact that data were restricted to the determination of gene profiles and changes were not confirmed at the protein level. We recognize that epiphyseal chondrocytes from newborn mice have properties of chondroblasts, are not fully mature and are not entirely representative of events occurring in the joint *in vivo*, and that osteoarthritis is a cartilage disorder of mature joints. Although the current work offered an initial understanding of the role of NOTCH2 in cartilage tissue homeostasis, it did not explore the role of NOTCH2 in osteoarthritis. Consequently, the signaling pathways affected by NOTCH2 may or may not have relevance to the pathogenesis of this disorder. The present studies are limited by a lack of functional follow up on either the cells or the pathways influenced by the NOTCH2 gain-of-function in epiphyseal chondrocytes.

In conclusion, NOTCH2 enhances the activity of pathways associated with inflammation and disrupts the transcriptome profile of articular/synovial fibroblasts in murine epiphyseal chondrocytes.

## Experimental procedures

### Genetically modified mice

*Notch2*^*tm1.1Ecan*^ mice harboring a 6955C>T substitution in the *Notch2* locus were backcrossed into a C57BL/6 background for ≥ 8 generations and have been characterized previously (22). *R26-NICD2* mice, created by Ryuichi Nishinakamura (Kumamoto University, Japan), were kindly provided by Fanxin Long (Philadelphia, PA) in a C57BL/6 background (34, 52). In *R26-NICD2* mice, sequences coding for the NOTCH2 NICD are cloned into the *Rosa26* locus downstream of a Neo-STOP cassette flanked by *loxP* sequences. Following the excision of the cassette by Cre recombination, the NOTCH2 NICD is expressed under the control of *Rosa26*. Genotyping was conducted by polymerase chain reaction (PCR) in DNA from tail extracts using specific primers from Integrated DNA Technologies (IDT). All animal studies were limited to tissue harvest following euthanasia and were approved by the Institutional Animal Care and Use Committee of UConn Health.

### Chondrocyte cultures

Epiphyseal cartilage cells were isolated from the proximal and distal joints of the tibiae of 4-day-old *Notch2*^*tm1.1Ecan*^, *R26-NICD2* and control mice, following the dissection of surrounding tissues under a Unitron Z850 stereo microscope. Epiphyseal cartilage was placed in high glucose Dulbecco’s modified Eagle medium (DMEM) and digested with 0.25% trypsin, 0.9 mM EDTA (Life Technologies) and then 200 U/ml of type II collagenase (Worthington Biochemical Corporation, Lakewood, NJ) in DMEM at 37 °C (30). Following digestions, the tissue was strained through a 70 μm membrane and the cells collected by centrifugation were cultured in DMEM supplemented with 10% heat inactivated fetal bovine serum (FBS, Atlanta Biologics) at 37 °C in a humidified 5% CO_2_ atmosphere until reaching ∼70% to 80% confluence (20, 30). Cultures were digested with trypsin once and cells were seeded at a density of one million cells/56.7 cm^2^, and for experiments using *Notch2*^*tm1.1Ecan*^, cells were cultured to confluency. To induce the NOTCH2 NICD, *R26-NICD2* chondrocytes were cultured until they reached 70 to 80% confluency, transferred to DMEM in the absence of serum for 1 h, and exposed overnight to 300 to 600 multiplicity of infection of replication-defective recombinant adenoviruses. An Ad-CMV-Cre (Vector Biolabs) was used to excise the STOP cassette in *R26-NICD2* cells, and Ad-CMV-GFP was used as a control. Following infection, cells were allowed to recover for 24 h in the presence of DMEM containing 10% FBS, digested with trypsin, and seeded as described for *Notch2*^*tm1.1Ecan*^ cells. Cells were deprived of serum for 8 h or overnight before being processed for bulk or scRNA-Seq (30). Cells were seeded as a pool for bulk RNA-Seq experiments and maintained as biological replicates (n = 4) for scRNA-Seq and pooled immediately before microfluidic partitioning.

### Bulk RNA sequencing and bioinformatics analysis

A NanoDrop 2000 spectrophotometer (Thermo Fisher Scientific) was used to determine purity ratios of total RNA, and an Agilent TapeStation 4200 (Agilent Technologies, Santa Clara, CA) was used to determine RNA quality using the RNA High Sensitivity assay. Samples used for library preparation had an RNA Integrity Number ≥ 9.0 and were processed for mRNA-sequencing at the UConn Center for Genome Innovation. This was conducted using the Illumina TruSeq Stranded mRNA sample preparation kit following manufacturer’s instructions (Illumina), as described previously (31, 33). Libraries were validated for length and adapter dimer removal using the Agilent TapeStation 4200 D1000 High Sensitivity assay (Agilent Technologies), quantified and normalized using the dsDNA High Sensitivity Assay for Qubit 3.0 (Thermo Fisher Scientific), as previously reported (31, 33). Sample libraries were prepared for sequencing by denaturing and diluting the libraries per manufacturer’s protocol (Illumina). Samples were pooled for sequencing, normalized, and run across an Illumina Next-Seq 500 using version 2.5 chemistry. Target read depth was achieved for each sample with paired end 75 bp reads. Sequencing raw reads were trimmed with Sickle (Version 1.33), with a respective quality and length threshold of 30 and 45 and mapped to the *Mus musculus* genome (GRCm39 ensembl release 105) with HISAT2 (version 2.1.0) (53). The generated SAM files were transformed into a BAM format using samtools (version 1.9), and PICARD was used to remove PCR duplicates (54). The counts were generated against the features with HTSeq-count, and the differential gene expression between control and experimental samples was determined using DESeq2 (55, 56). To increase accuracy of the results, covariates were introduced in the DESeq2 analysis and genes showing less than ten counts across the compared samples were excluded from further analysis. A false discovery rate (FDR) adjusted *p* value < 0.05 was considered significant and used for downstream analysis, as indicated in text and legends. Highly variable functions were identified, and linear dimensional reduction conducted by principal component analysis. The processed RNA-Seq results were analyzed by IPA (Qiagen, Redwood City, CA), GSEA and the R package clusterProfiler enrichment tool (57, 58, 59). The parameters chosen for IPA were a *p* value of < 0.05, an FDR *p* adjusted value of 0.1 and fold changes (FC) set at log2FC ≥ 1 analyzed using canonical pathways under the Genes and Chemicals category. For GSEA, the parameters chosen were m5.go.bp.v2024.1.Mm.symbols.gmt permutation gene set and Chip platform Mouse_Ensembl_Gene_ID_MSigDB.v2024.1.Mm.chip. Gene sets larger than 500 genes and lesser than 10 genes were excluded.

### scRNA-Seq and computational analysis

To analyze transcriptome profiles on a cell-by-cell basis, live cells from control and experimental cultures of epiphyseal chondrocytes were pooled at the completion of the culture, counted on a Cellometer K2 (Nexceleron) and processed on a Chromium iX instrument using a Chromium Single Cell 3′ library and Gel Bead Kit v3.1 (10x Genomics) to prepare barcoded libraries, as previously reported (33, 60). The single cell gene expression kit used has a cell capture efficiency of ∼65% to 75% and equal number of control and experimental cells were partitioned aimed at a recovery of ∼10,000 cells/group. Following reverse transcription, libraries were sequenced at the UConn Center for Genome Innovation. cDNAs from each single cell had unique barcodes, allowing the sequencing reads to be mapped backed to the cell of origin (61).

FASTQ files were generated using reference genome mm10 to 2020-A and analyzed using Cell Ranger v7.0 or v8.0 (10x Genomics) for sample demultiplexing, barcode processing, transcript counting and output HTMLs for data quality assessment and sample visualization. Secondary analyses of output files were conducted using the Seurat R Package (v5.2.1). These included dimensionality reduction, cell clustering and differential gene expression (62, 63). Cells with <250 transcripts, >10% mitochondrial RNA or >10,000 genes/cell were excluded manually, and the NormalizeData function in Seurat was used to normalize the data set. Highly variable functions were identified, and linear dimensional reduction conducted by principal component analysis and non-linear dimensionality reduction performed by UMAP. Doublets were identified and excluded, and cell clusters were classified according to their gene profile and gene ontology (GO). For this purpose, the FindMarkers function in Seurat was chosen to select the 50 most expressed genes in each cluster. GO was determined in the National Center for Biotechnology Information database. Pseudotime trajectory analysis of pooled and independent data from control and experimental cells was constructed by analysis of the Seurat object in Monocle 3 (64). Monocle 3 cell data set was constructed and the learn principal graph from the reduced dimensional space was applied using learn_graph, as described previously (60). Cells were organized along their trajectory using Monocle plot_cells and order_cells; marker genes for each cluster were identified using the Seurat’s FindAllMarkers function. Gene ontology and IPA of differentially expressed genes was performed on a per-cluster basis, and genes prioritized based on ontology, and relevance to transcriptional control and signaling pathways (57, 58, 59). IPA was performed to analyze canonical pathways under the Genes and Chemicals category.

### Statistics

Data are presented as means ± standard deviations (SD) and individual values. Statistical differences were determined by unpaired *t* test. Values beyond 2 SD from the mean are considered outliers.

## Data availability

All data are available from the corresponding author upon a reasonable request. The raw data for bulk and scRNA-Seq were deposited in Gene Expression Omnibus (GEO) under GSE292806 and GSE292807.

## Supporting information

This article contains supporting information.

## Conflict of interest

The authors declare no conflicts of interest with the contents of this article.
