# Supplementary material for: NOTCH2 disrupts the synovial fibroblast identity and the inflammatory response of epiphyseal chondrocytes
Source: J Biol Chem. 2025 May 8;301(6):110206. doi: 10.1016/j.jbc.2025.110206 (PMC12179613; doi:10.1016/j.jbc.2025.110206)

NOTCH2 disrupts the synovial fibroblast identity and the inflammatory response of epiphyseal chondrocytes

Ernesto Canalis[1,2,3*], Rosa Guzzo[4], Lauren Schilling[3] and Emily Denker[3]

From the Departments of [1]Orthopaedic Surgery, [4]Neuroscience and [2]Medicine, and the [3]UConn Musculoskeletal Institute, UConn Health, Farmington, CT 06030

Supporting Information:

- **Table S1.** Differentially expressed genes (log2FC) between the pooled limb mesenchyme (clusters 0, 1, 3 and 12), pooled chondrogenic (clusters 5, 7, 8 and 14), pooled fibroblast (clusters 9, 10 and 11), undefined (clusters 4, 6, 13) and articular/synovial fibroblasts (cluster 2) and all other cell clusters from epiphyseal chondrocytes from *Notch2*$^{tm1.1Ecan}$ and littermate control mice.
- **Table S2**. Differentially expressed genes between *Notch2*$^{tm1.1Ecan}$ and control littermates in mesenchymal, limb mesenchyme, articular/synovial fibroblast, chondrogenic, adipogenic, osteogenic, macrophage and fibroblasts shown in Figure 7C and 7D. The number of cells expressing each gene and the intensity or level of expression is provided.
- **Table S3**. Differentially expressed genes in the articular/synovial fibroblast cluster between *Notch2*$^{tm1.1Ecan}$ and control littermate mice. The number of cells expressing each gene in the cluster and the intensity or level of expression is provided.
- **Table S4.** Differentially expressed genes (log2FC) between the limb mesenchyme (cluster 3), pooled chondrogenic (clusters 6 and 12), pooled fibroblast (clusters 1, 2, 4 and 11), fibroblasts? (clusters 9 and 13), undefined (clusters 0, 5, 7 and 8) and articular/synovial fibroblast (cluster 10) and all other cell clusters from epiphyseal chondrocytes from *R26-NICD2* mice transfected with Ad-CMV-Cre (NOTCH2 activated) or Ad-CMV-GFP (control).
- **Table S5.** Differentially expressed genes between NICD2-expressing and control cells associated with mesenchymal, limb mesenchyme, articular/synovial fibroblast, chondrogenic, adipogenic, osteogenic, macrophages and fibroblast cells shown in Figure 9B and 9C. The number of cells expressing each gene and the intensity or level of expression is provided.
- **Table S6.** Differentially expressed genes in the undefined cluster between NICD2-expressing and control cells. The number of cells expressing each gene in the cluster and the intensity or level of expression is provided.
- **Table S7.** Primers used for genotyping.
- **Figure S1.** Differential gene expression by the *Notch2*$^{tm1.1Ecan}$ mutation in epiphyseal chondrocytes determined by bulk RNA-Seq.
- **Figure S2.** Differential gene expression by NICD2 activation in epiphyseal chondrocytes determined by bulk RNA-Seq.
- **Figure S3.** Dot plot displaying the expression of *Notch1*, *2*, *3* and *4*, *Hes1*, *Hey1*, *2* and *l* and *Jagged1*.
- **Figure S4.** Ingenuity pathway analysis (IPA) of the articular/synovial fibroblast cluster from chondrocyte-enriched cells from *Notch2*$^{tm1.1Ecan}$ and control mice.

Table S1. Differentially expressed genes (log2FC) between the pooled limb mesenchyme (clusters 0, 1, 3 and 12), pooled chondrogenic (clusters 5, 7, 8 and 14), pooled fibroblast (clusters 9, 10 and 11), undefined (clusters 4, 6, 13) and articular/synovial fibroblasts (cluster 2) and all other cell clusters from epiphyseal chondrocytes from *Notch2*$^{tm1.1Ecan}$ and littermate control mice.

Limb Mesenchyme					
Control			*Notch2*$^{tm1.1Ecan}$		
Gene	*p*	FC	Gene	*p*	FC
Tnmd	4E-218	4.1	Sfrp1	2E-108	3.7
Sfrp1	2E-140	3.8	Egfl6	2E-194	3.7
Pappa2	7E-65	3.4	Tnmd	1E-221	3.7
Thbs4	3E-68	3.4	Crabp1	2E-80	3.5
Dkk2	1E-64	3.3	Lgr5	3E-199	3.1
Mfap4	2E-239	3.1	Igfbp3	6E-98	2.9
Egfl6	1E-279	3.1	Mfap4	3E-179	2.9
Lgr5	5E-219	2.7	Serping1	6E-144	2.8
Slc1a3	4E-146	2.7	Dkk2	8E-38	2.8
Steap4	1E-78	2.6	Ccl7	8E-120	2.7
Cxcl5	9E-52	2.6	Ptn	3E-254	2.7
Ptn	0E+00	2.6	Tbxa2r	1E-72	2.6
Igfbp3	2E-112	2.6	Slit2	6E-150	2.5
Slit2	7E-140	2.5	Slc1a3	1E-129	2.5
Crabp2	2E-136	2.5	Igf1	6E-182	2.5
Serping1	3E-159	2.4	Steap4	2E-41	2.4
Igf1	9E-204	2.4	Col8a1	1E-128	2.4
Ccl7	8E-96	2.4	Cxcl5	3E-47	2.3
En1	1E-68	2.4	Fgf10	7E-59	2.3
Il1rn	4E-151	2.4	Crabp2	1E-151	2.3
Vat1l	7E-41	2.3	Thy1	6E-104	2.3
Apod	5E-114	2.2	Fbln2	9E-306	2.1
Lurap1l	4E-207	2.2	Lurap1l	4E-229	2.0
Itga2	5E-30	2.1	Postn	2E-242	2.0
Thy1	5E-120	2.1	Vcam1	9E-167	2.0
Tagln	7E-112	2.1	Aqp5	6E-40	2.0
Postn	6E-297	2.1	Il1rn	3E-119	2.0
Lbp	8E-89	2.1	Pcdh19	9E-136	2.0
Meox1	1E-198	2.0	Wnt16	4E-49	2.0
Myl9	9E-109	2.0	Apod	1E-103	2.0
Kitl	2E-127	2.0	Sfrp2	4E-192	2.0
Pcdh19	6E-170	2.0	En1	3E-51	1.9
Vcam1	5E-167	2.0	Acvrl1	1E-54	1.9
Cacna2d3	2E-48	1.9	Apbb1ip	7E-97	1.9
Kcnip4	4E-155	1.9	Rspo2	1E-205	1.9
Fgf10	2E-80	1.9	Hgf	2E-92	1.9
Sncaip	7E-55	1.9	Scara5	1E-73	1.9
Col14a1	1E-129	1.9	Nhsl2	1E-58	1.9
Wnt16	3E-82	1.9	Agmo	2E-70	1.9
Apbb1ip	1E-117	1.8	Arhgap20	7E-144	1.9
Hgf	3E-135	1.8	Meox1	1E-108	1.8
F3	3E-41	1.8	Tagln	1E-145	1.8
Agmo	1E-66	1.8	Nav3	5E-41	1.8
Sfrp2	7E-291	1.8	Myl9	2E-129	1.8
Arhgap20	2E-191	1.8	Mid1	3E-141	1.8
Fbln2	0E+00	1.8	Epha3	8E-98	1.7
Egr3	2E-44	1.8	Ltbp2	1E-75	1.7
Gpr39	3E-17	1.8	C1qtnf3	6E-24	1.7
Slc8a1	1E-142	1.8	Basp1	4E-196	1.7
Epha4	1E-111	1.8	Itga11	5E-115	1.7
Cdh2	2E-249	1.8	Hs6st3	3E-32	1.7
Acvrl1	1E-48	1.8	Ifi211	7E-35	1.6
Mid1	7E-193	1.7	Slc8a1	2E-95	1.6
Ltbp2	1E-75	1.7	Megf6	2E-91	1.6
Hs6st3	1E-48	1.7	Fgf7	2E-154	1.6
Epha7	2E-94	1.7	Cacna2d3	8E-24	1.6
Pax1	1E-26	1.7	Mfap5	2E-175	1.6
Col8a1	4E-77	1.7	Kitl	2E-53	1.6
Rspo2	3E-276	1.7	Cdh2	2E-193	1.6
Megf6	4E-63	1.7	Atp8b1	8E-74	1.6

Chondrogenic					
Control			*Notch2*$^{tm1.1Ecan}$		
Gene	*p*	FC	Gene	*p*	FC
Snorc	9E-280	5.2	Matn1	5E-238	4.6
Matn1	2E-185	4.9	Snorc	5E-275	4.0
Pantr1	7E-134	4.2	Pantr1	1E-124	4.0
Meltf	3E-171	4.2	Scin	2E-302	4.0
Scin	2E-266	4.0	Ncmap	1E-267	3.9
Col25a1	9E-151	3.9	Meltf	2E-218	3.8
Mmp13	2E-58	3.8	Col25a1	9E-154	3.8
Col9a1	0E+00	3.7	Zfp385c	1E-86	3.6
Pou3f3	2E-102	3.6	Fxyd3	2E-69	3.5
Ncmap	6E-249	3.5	Col9a2	6E-293	3.4
Col9a2	3E-289	3.5	Col9a1	0E+00	3.4
Acan	0E+00	3.4	Col9a3	0E+00	3.4
Cmtm5	1E-110	3.4	Cmtm5	2E-149	3.3
Mdga2	2E-64	3.4	Acan	0E+00	3.2
Col9a3	0E+00	3.3	Col11a2	0E+00	3.2
Fxyd3	7E-72	3.3	Chad	2E-77	3.2
Col11a2	0E+00	3.2	Ninj2	9E-74	3.2
Adgrv1	4E-108	3.2	Adgrv1	8E-82	3.1
Epyc	5E-141	3.0	Fgfr3	2E-279	3.1
Proser2	6E-81	3.0	Grem1	4E-170	2.9
Susd5	0E+00	2.9	Susd5	0E+00	2.8
Fgfr3	9E-219	2.8	Col27a1	0E+00	2.8
Col2a1	0E+00	2.8	Dhx58os	8E-157	2.8
Cidea	3E-89	2.8	Ppp1r1b	1E-260	2.7
Lef1	8E-246	2.7	Cidea	2E-114	2.7
Dhx58os	3E-108	2.5	Cpm	2E-54	2.7
Cnmd	0E+00	2.5	Bfsp2	7E-96	2.7
Enpp2	6E-218	2.5	Efcab1	3E-179	2.6
Ppp1r1b	5E-229	2.4	Tenm2	3E-45	2.6
Col27a1	0E+00	2.4	Dusp15	4E-47	2.6
Chst11	2E-284	2.4	Epyc	4E-86	2.6
Efcab1	7E-163	2.3	Col2a1	0E+00	2.5
Fzd9	0E+00	2.3	Mgat4a	6E-161	2.5
Papss2	0E+00	2.3	Chst11	1E-303	2.5
Rflna	2E-66	2.3	Gm13944	1E-65	2.5
G0s2	1E-251	2.3	Extl1	2E-89	2.5
Sh3tc2	1E-126	2.2	Frzb	2E-211	2.5
Tenm2	5E-59	2.2	Papss2	0E+00	2.5
Sorbs2os	7E-157	2.2	Proser2	3E-77	2.4
Mia	0E+00	2.2	Lef1	8E-180	2.4
Ucma	0E+00	2.2	Cd24a	4E-208	2.4
Mgat4a	3E-145	2.2	Mdga2	1E-45	2.4
Cpm	6E-43	2.2	Cnmd	0E+00	2.4
Sdk2	0E+00	2.2	Steap1	3E-192	2.4
Tspan13	5E-53	2.1	Sdk2	0E+00	2.3
Wif1	7E-186	2.1	G0s2	1E-280	2.3
4930431P03Rik	6E-51	2.1	Hapln1	0E+00	2.3
Scrg1	0E+00	2.1	Enpp2	3E-202	2.3
Nebl	7E-173	2.1	Zfp385b	8E-257	2.2
Bfsp2	6E-64	2.1	Gm28153	2E-163	2.2
Gm13944	6E-62	2.1	Gal3st1	1E-45	2.2
Spp1	5E-30	2.1	Scrg1	4E-302	2.2
Hapln1	0E+00	2.0	Sh3tc2	3E-114	2.2
Cd200	4E-31	2.0	Mmp13	5E-24	2.2
Gm13919	3E-93	2.0	Mia	0E+00	2.2
Fibin	0E+00	2.0	Nebl	1E-173	2.1
Frzb	2E-147	2.0	Gsg1l	3E-47	2.1
Steap1	1E-159	2.0	B4galnt2	3E-117	2.1
B4galnt2	8E-129	2.0	Slc16a14	3E-93	2.1
St3gal6	0E+00	2.0	Gm17173	2E-44	2.1

Fibroblast					
Control			*Notch2*$^{tm1.1Ecan}$		
Gene	*p*	FC	Gene	*p*	FC
Pbk	1E-235	7.9	Esco2	7E-217	7.0
Hmmr	1E-224	6.5	Plk1	2E-233	6.9
Esco2	3E-167	6.4	Bub1	2E-235	6.9
Neil3	5E-161	6.3	Ska1	6E-208	6.8
Cenpf	4E-189	6.2	Mxd3	4E-207	6.7
Bub1	3E-189	6.1	Kif18b	5E-163	6.6
Kif18b	5E-137	6.1	Cenpf	1E-249	6.5
Pimreg	9E-138	6.1	Pimreg	9E-200	6.5
Ska1	7E-163	5.8	Kif14	2E-166	6.4
Kif4	1E-191	5.8	Neil3	1E-194	6.4
Iqgap3	8E-141	5.8	Hmmr	1E-295	6.4
Plk1	2E-141	5.7	Kif4	7E-264	6.3
Mki67	4E-191	5.6	Ccna2	3E-283	6.2
Pclaf	3E-164	5.6	Pbk	1E-269	6.2
Ccna2	2E-206	5.6	Mki67	0E+00	6.2
Aurkb	6E-150	5.6	E2f7	1E-136	5.9
Nusap1	4E-149	5.6	Aspm	1E-213	5.8
Nuf2	2E-180	5.4	Sgo2a	6E-205	5.8
Birc5	3E-185	5.4	Melk	3E-205	5.8
Aspm	7E-141	5.3	Cep55	1E-171	5.7
Cep55	3E-122	5.3	Kif2c	3E-173	5.7
Ube2c	2E-137	5.3	Depdc1a	1E-208	5.6
Knl1	1E-175	5.3	Pclaf	1E-245	5.6
Mxd3	2E-128	5.2	Prc1	5E-195	5.4
Kif2c	1E-115	5.1	Top2a	5E-184	5.4
Ttk	1E-146	5.0	Ttk	6E-157	5.4
Dlgap5	2E-155	5.0	Aurka	2E-156	5.4
Anln	1E-163	5.0	Ckap2l	4E-247	5.4
Cenpe	4E-128	4.9	Ube2c	1E-208	5.4
Top2a	4E-98	4.8	Nusap1	3E-195	5.4
Sgo2a	3E-131	4.8	Knl1	7E-229	5.3
Ckap2l	3E-165	4.8	Iqgap3	6E-209	5.3
Shcbp1	2E-143	4.8	Sgo1	2E-204	5.3
Melk	1E-151	4.8	Troap	2E-122	5.3
Prc1	2E-101	4.8	Ndc80	7E-204	5.3
Depdc1a	6E-136	4.7	Dlgap5	2E-217	5.3
Cdca3	1E-146	4.7	Birc5	2E-264	5.3
Ccnb1	3E-113	4.6	Nuf2	9E-251	5.2
Tpx2	5E-106	4.6	Shcbp1	5E-210	5.2
Ndc80	1E-142	4.6	Ccnb1	1E-200	5.2
Sgo1	3E-147	4.4	Anln	3E-213	5.2
Aurka	3E-93	4.4	Espl1	2E-108	5.2
Ect2	1E-129	4.4	Cenpe	7E-224	5.2
Kif15	6E-126	4.4	Ect2	4E-187	5.1
Cdca8	9E-126	4.3	Kif23	3E-227	4.9
Knstrn	3E-108	4.3	Tk1	8E-185	4.9
Kif23	1E-139	4.3	Cdc20	1E-190	4.9
Fbxo5	9E-109	4.3	Kif15	8E-191	4.8
Cdc20	4E-94	4.3	Mis18bp1	2E-172	4.8
Asf1b	2E-123	4.3	Asf1b	1E-178	4.8
Tk1	2E-117	4.2	Tpx2	1E-189	4.8
Cenpa	3E-91	4.2	Rad51ap1	3E-171	4.7
Rad51ap1	6E-114	4.1	Aurkb	2E-172	4.7
Mis18bp1	3E-96	4.1	Fbxo5	5E-172	4.7
Ncaph	5E-114	4.1	Ncapg	9E-170	4.7
Ckap2	6E-70	4.1	Ccnf	4E-136	4.7
Cenpi	3E-96	4.1	Cdca2	9E-183	4.7
Ccnf	2E-96	4.0	Kif20b	9E-184	4.6
Clspn	6E-95	4.0	Bub1b	6E-215	4.5
Kntc1	4E-115	4.0	Cenpi	2E-117	4.5

Undefined					
Control			*Notch2*$^{tm1.1Ecan}$		
Gene	*p*	FC	Gene	*p*	FC
Snorc	6E-141	3.6	Matn1	1E-83	3.3
Matn1	5E-93	3.6	Snorc	3E-129	3.3
Meltf	7E-75	2.8	Fxyd3	4E-37	2.8
Cmtm5	2E-38	2.4	Cmtm5	3E-47	2.6
Cidea	1E-55	2.3	Meltf	3E-73	2.5
Scin	9E-71	2.3	Proser2	4E-35	2.5
Col25a1	3E-41	2.3	Col9a2	1E-85	2.3
Col9a3	9E-193	2.2	Scin	7E-77	2.2
Col9a2	7E-89	2.2	Cidea	8E-46	2.2
Ncmap	9E-81	2.1	Col9a3	2E-163	2.2
Enpp2	2E-119	2.1	Enpp2	1E-86	2.2
Ppp1r1b	5E-120	2.1	Dhx58os	3E-54	2.1
Acan	1E-164	2.1	Grem1	5E-55	2.1
Gm26917	6E-01	2.0	Ppp1r1b	5E-102	2.1
Dhx58os	5E-64	2.0	Cd24a	1E-67	2.0
Cytl1	4E-51	2.0	Acan	6E-139	2.0
Col11a2	6E-190	1.9	Col11a2	5E-161	1.9
Syt1	4E-26	1.9	Ncmap	2E-50	1.9
Proser2	1E-30	1.9	Fgfr3	2E-71	1.9
Fgfr3	4E-75	1.8	Gal3st1	4E-20	1.9
Col2a1	5E-281	1.8	Ntrk2	1E-27	1.8
Col9a1	8E-143	1.7	Frzb	6E-73	1.8
Tenm2	5E-18	1.7	Steap1	2E-53	1.8
Wif1	1E-94	1.7	Gm28153	1E-77	1.8
Matn3	2E-86	1.6	Col25a1	3E-31	1.8
Stc2	6E-19	1.6	Cytl1	5E-27	1.8
Gm28153	6E-91	1.6	G0s2	2E-73	1.8
Papss2	1E-121	1.6	Ostn	3E-37	1.7
Ucma	1E-214	1.6	Col9a1	4E-127	1.6
Steap1	1E-58	1.6	Susd5	1E-67	1.6
Crispld1	6E-193	1.6	Mia	3E-189	1.6
Fry	8E-181	1.5	Papss2	2E-87	1.6
Cd24a	1E-50	1.5	Scrg1	1E-130	1.6
Cnmd	8E-214	1.5	Cnmd	8E-206	1.6
Mia	2E-199	1.5	Bfsp2	4E-22	1.6
G0s2	7E-45	1.5	Wif1	3E-44	1.5
Grem1	7E-34	1.5	Col2a1	2E-214	1.5
Scrg1	6E-147	1.5	Crispld1	2E-136	1.5
Vwc2	3E-28	1.5	Fzd9	7E-84	1.5
Mgat4a	9E-41	1.5	Trpv4	6E-49	1.5
Sh3tc2	2E-31	1.5	Col27a1	6E-75	1.5
Gm28905	6E-08	1.5	Hapln1	1E-115	1.5
Xylt1	2E-180	1.5	S100b	2E-104	1.5
Gdf10	3E-104	1.5	Ndrg2	8E-17	1.5
Nebl	5E-73	1.5	Zfp385b	1E-48	1.5
Loxl4	1E-34	1.4	Stk26	5E-54	1.5
Col27a1	6E-81	1.4	Soat1	8E-31	1.4
Svopl	1E-21	1.4	Chchd10	1E-26	1.4
Fzd9	2E-98	1.4	Mgat4a	3E-35	1.4
Susd5	1E-78	1.4	Gramd2	6E-10	1.4
Fibin	8E-115	1.4	Slc16a14	1E-12	1.4
Bfsp2	1E-21	1.4	Matn3	4E-42	1.4
Msmp	1E-22	1.4	Cxadr	3E-12	1.4
Frzb	4E-53	1.4	Sh3tc2	1E-17	1.4
Epyc	1E-19	1.4	Pcolce2	9E-16	1.4
Zfp385b	1E-75	1.4	Pir	6E-23	1.4
Ostn	4E-23	1.4	Efcab1	5E-21	1.4
B4galnt2	1E-41	1.4	Cth	1E-26	1.4
St3gal6	2E-114	1.4	B4galnt2	5E-29	1.4
Atp6v0a4	7E-64	1.3	Fibin	7E-83	1.4

Articular/Synovial Fibroblast					
Control			*Notch2*$^{tm1.1Ecan}$		
Gene	*p*	FC	Gene	*p*	FC
Htra4	2E-130	5.7	Htra4	6E-123	5.7
Gm48053	3E-158	4.6	Serpinb5	2E-152	4.2
Serpinb5	2E-208	4.5	Tnxb	2E-270	4.1
Tnxb	0E+00	3.9	Clec3b	8E-273	3.9
Clec3b	0E+00	3.9	Dpp4	3E-101	3.8
Galnt16	7E-240	3.8	Gm48053	1E-86	3.8
Gm15675	8E-165	3.7	Gda	2E-142	3.8
Cd55	3E-284	3.6	Galnt16	5E-142	3.7
Pla1a	3E-130	3.6	Tspan15	3E-76	3.5
Efhd1	2E-133	3.5	Mlana	3E-104	3.4
Gda	6E-287	3.5	Pamr1	9E-162	3.3
Pamr1	2E-250	3.5	Ntn1	1E-136	3.3
Tspan15	1E-133	3.4	Gm15675	3E-65	3.2
Adgrd1	2E-145	3.4	Cd34	1E-211	3.2
Fez1	5E-208	3.3	Pla1a	4E-125	3.2
Ly6a	0E+00	3.3	Pla2g2e	9E-51	3.1
Dpp4	3E-120	3.2	Htr2a	9E-55	3.1
Htr2a	9E-91	3.2	Cd55	8E-137	3.1
Ly6c1	0E+00	3.2	Ly6a	1E-200	3.0
Mlana	2E-81	3.2	Ly6c1	4E-162	3.0
Cd34	0E+00	3.1	Fez1	9E-117	3.0
Xdh	4E-82	3.1	Inhbb	1E-33	2.8
Olfml2a	3E-133	3.1	Emilin2	3E-92	2.8
1700019D03Rik	4E-47	3.0	A330102I10Rik	3E-33	2.7
Tek	3E-68	2.9	Rarres2	2E-172	2.7
Creb5	4E-286	2.9	Prg4	4E-78	2.7
Ntn1	8E-184	2.9	Dpt	2E-150	2.7
Nova1	7E-62	2.9	Efhd1	6E-42	2.6
Emilin2	7E-208	2.9	Creb5	2E-206	2.6
Lrrn4cl	2E-106	2.9	Agtr2	3E-69	2.5
Col5a3	8E-232	2.8	Adam33	7E-89	2.5
Pla2g2e	8E-50	2.8	Lrrn4cl	2E-44	2.5
Rarres2	8E-233	2.7	Gm26740	4E-38	2.5
Myo16	8E-49	2.7	Kif1a	1E-109	2.5
Kif1a	3E-167	2.7	Eln	8E-116	2.5
Dpt	1E-142	2.6	Cd248	1E-196	2.4
Prg4	3E-72	2.6	Apcdd1	1E-29	2.4
Gm20125	6E-45	2.6	Gm16083	1E-39	2.4
Procr	2E-65	2.6	Plpp3	2E-132	2.4
Cd248	2E-304	2.6	Fam124a	8E-43	2.4
AW551984	1E-29	2.5	Col5a3	6E-161	2.4
Itgb3	3E-57	2.5	Pdzk1ip1	2E-46	2.3
Agtr2	1E-122	2.5	3300005D01Rik	5E-34	2.3
Plpp3	1E-218	2.5	Medag	2E-140	2.3
Gm16083	2E-75	2.5	Gsta4	3E-73	2.3
Mfap5	7E-217	2.4	Vit	1E-29	2.2
Tmem26	4E-34	2.4	Gm47710	2E-50	2.2
Nat8f3	9E-36	2.4	Olfml2a	4E-55	2.2
Slurp1	5E-38	2.4	Adm	3E-93	2.2
Maob	4E-43	2.4	Adamts5	9E-145	2.2
Pdzk1ip1	4E-80	2.3	Rbpms	8E-46	2.2
Thbd	6E-127	2.3	Ntrk3	4E-91	2.2
Efemp1	3E-166	2.3	Thbd	3E-65	2.1
Bmp6	3E-47	2.3	Adgrd1	1E-49	2.1
Adamts5	2E-229	2.3	Efemp1	5E-78	2.1
Adm	7E-153	2.3	Sema3c	1E-45	2.1
Gm47710	8E-64	2.3	Serpina3n	3E-39	2.0
Ucp2	8E-48	2.2	Nox4	3E-62	2.0
Tnik	1E-57	2.2	Sema3d	4E-30	2.0
Apcdd1	2E-42	2.2	Ebf1	8E-194	2.0

Limb Mesenchyme					
Control			*Notch2*$^{tm1.1Ecan}$		
Gene	*p*	FC	Gene	*p*	FC
Nhsl2	2E-48	1.7	Igfbp2	3E-103	1.5
Qpct	8E-33	1.7	Dpyd	2E-29	1.5
Nav3	6E-37	1.7	Sorcs2	1E-59	1.5
Nmnat2	6E-30	1.6	Lbp	3E-48	1.5
Slc25a21	4E-34	1.6	Acvr2a	3E-87	1.5
Cxcl12	2E-149	1.6	Acta2	7E-114	1.5
Epha3	6E-76	1.6	Sncaip	4E-30	1.4
Fgf7	2E-183	1.6	Ramp2	1E-66	1.4
Acta2	1E-97	1.6	Fst	7E-72	1.4
Aqp5	2E-27	1.6	Nmnat2	6E-32	1.4
Cacnb4	2E-39	1.6	Igfbp6	4E-164	1.4
Iqgap2	2E-33	1.5	Akap12	1E-123	1.4
C1qtnf3	5E-15	1.5	Ptx3	5E-130	1.4
Ptx3	1E-112	1.5	Jag1	1E-74	1.4
Dpyd	4E-39	1.5	Col14a1	2E-104	1.4
Ramp2	2E-115	1.5	Samd5	2E-47	1.4
Igfbp2	8E-122	1.5	Kcnip4	4E-74	1.4
Gm26771	2E-115	1.5	Cpxm2	9E-64	1.4
Astn2	2E-21	1.5	Inhba	1E-56	1.4
Cyp7b1	2E-48	1.5	Serpine1	4E-51	1.3
Gsc	2E-33	1.5	Chd3	1E-109	1.3
Ifi211	1E-45	1.5	Tnfaip6	3E-40	1.3
Samd5	4E-52	1.5	Galnt18	4E-45	1.3
Epha5	3E-61	1.5	C1qtnf7	2E-23	1.3
Atp8b1	3E-65	1.5	Egr3	3E-26	1.3
Apobec3	4E-28	1.5	Ccn3	3E-166	1.3
Tnfaip6	7E-37	1.4	Epha4	4E-85	1.3
Lrrn3	3E-24	1.4	Eya2	3E-18	1.3
Adam12	1E-58	1.4	Col1a1	0E+00	1.3
Gng8	1E-53	1.4	Cacnb3	8E-29	1.3
Acvr2a	9E-77	1.4	Hmcn1	5E-52	1.3
Pax9	4E-26	1.4	Gm26771	1E-67	1.3
Fst	5E-83	1.4	Tmem47	7E-52	1.3
Jag1	9E-38	1.4	Selenop	1E-95	1.3
Cdh11	0E+00	1.4	Nrg1	2E-37	1.3
Aplp1	8E-62	1.4	Nnmt	2E-139	1.3
Igfbp6	4E-190	1.4	Flnc	1E-81	1.3
Inhba	1E-49	1.4	Cdh11	3E-269	1.2
Pcdh18	2E-87	1.4	Gng8	6E-40	1.2
Sorcs2	1E-54	1.3	Fam227b	2E-26	1.2
Serpine1	5E-37	1.3	Pcdh18	5E-78	1.2
Col1a1	0E+00	1.3	Serpinf1	0E+00	1.2
Ksr1	2E-26	1.3	Gstm2	1E-88	1.2
Selenop	4E-131	1.3	Rassf5	4E-23	1.2
Prrg3	2E-54	1.3	Adam23	2E-49	1.2
Podn	3E-54	1.3	Thsd7a	1E-39	1.2
Bmper	2E-12	1.3	Gpr176	4E-42	1.2
Carmn	2E-59	1.3	Gsc	9E-29	1.2
Fam227b	5E-36	1.3	Prrg3	5E-30	1.2
Nnmt	1E-173	1.3	Cxcl14	2E-16	1.2
Chd3	1E-118	1.3	Ebf1	5E-118	1.2
Itga11	1E-65	1.3	Il1rap	9E-40	1.2
Crlf1	8E-160	1.3	Jcad	7E-77	1.1
Shroom3	4E-10	1.3	Ncald	2E-17	1.1
Dysf	2E-28	1.3	Pdgfd	2E-21	1.1
Penk	9E-71	1.3	Vcan	5E-106	1.1
Cacna1g	6E-35	1.3	Mmp23	6E-115	1.1
Tmem119	1E-60	1.3	Crlf1	1E-129	1.1
Gas6	6E-202	1.2	Agtr1a	1E-24	1.1
Nrg1	1E-44	1.2	Epha7	5E-48	1.1
Mfap2	3E-131	1.2	Prrx2	4E-209	1.1
Galnt18	4E-45	1.2	Mical2	9E-24	1.1
Ebf1	7E-137	1.2	Aplp1	2E-39	1.1
Adam23	1E-52	1.2	F3	3E-19	1.1

Chondrogenic					
Control			*Notch2*$^{tm1.1Ecan}$		
Gene	*p*	FC	Gene	*p*	FC
Stk26	3E-246	2.0	Fzd9	7E-297	2.1
Kcna6	5E-65	1.9	Ucma	0E+00	2.1
Extl1	2E-53	1.9	Galnt3	1E-90	2.1
Cytl1	7E-78	1.9	St3gal6	1E-302	2.1
Syt1	2E-34	1.9	Stk26	3E-251	2.1
Cd24a	2E-139	1.9	Soat1	3E-204	2.0
Gm17173	2E-57	1.9	Crispld1	2E-283	2.0
Matn3	6E-137	1.9	Cth	3E-96	2.0
Gm28153	5E-147	1.9	Cnnm1	2E-27	2.0
Kctd4	5E-53	1.9	Pir	5E-91	2.0
Tfrc	3E-48	1.9	Trpv4	1E-192	1.9
Plxnb1	3E-62	1.9	Kctd14	2E-41	1.9
Kcnq5	0E+00	1.9	Itga6	1E-151	1.9
Sorbs2	3E-291	1.9	Matn3	1E-108	1.9
Parm1	3E-85	1.8	Wwp2	2E-294	1.9
Wnk2	8E-36	1.8	Kctd4	6E-47	1.9
Runx3	2E-62	1.8	Pdzrn4	2E-205	1.9
C530008M17Rik	5E-67	1.8	Rflna	2E-43	1.9
Xylt1	0E+00	1.8	Cp	6E-173	1.9
Soat1	9E-204	1.8	Ostn	1E-102	1.8
Itga6	1E-164	1.8	Acox2	4E-69	1.8
Galnt3	2E-129	1.8	Ifitm10	3E-130	1.8
Trpv4	2E-159	1.8	Loxl4	4E-32	1.8
Bmp2	2E-29	1.8	Fibin	2E-285	1.8
Crispld1	4E-248	1.8	Fry	3E-271	1.8
Grem1	1E-53	1.8	Kcna6	4E-54	1.8
Fry	3E-283	1.8	Chadl	1E-85	1.8
Tspan2	2E-77	1.7	Gm16070	3E-133	1.8
Nt5e	1E-61	1.7	Smoc2	7E-160	1.8
Zfp385b	1E-211	1.7	Xylt1	0E+00	1.8
Usp29	1E-119	1.7	Ntrk2	2E-47	1.8
Slc16a14	3E-44	1.7	Cytl1	7E-61	1.8
Gm16070	1E-131	1.7	Sorbs1	9E-190	1.8
Sorbs1	4E-175	1.7	Sorbs2os	7E-108	1.8
Stc2	5E-56	1.7	Mei4	1E-76	1.8
Prkcz	2E-69	1.7	Tspan13	2E-34	1.8
Zim1	1E-144	1.7	Prkcz	3E-68	1.7
Gdf10	6E-132	1.7	Itga10	1E-106	1.7
Acox2	1E-79	1.7	Prune2	8E-77	1.7
Gm10371	1E-49	1.7	Wnk2	3E-35	1.7
Wwp2	1E-265	1.7	Ppp1r3c	2E-45	1.7
Rab11fip4	2E-143	1.7	Etv5	2E-87	1.7
Cth	2E-75	1.6	Usp29	1E-107	1.7
Cdkn1c	3E-215	1.6	Cdkn1c	2E-212	1.7
Gm15414	3E-34	1.6	Cxadr	2E-62	1.7
Cox4i2	4E-91	1.6	Wif1	1E-105	1.7
Slc40a1	3E-19	1.6	Sorbs2	3E-259	1.7
Ostn	2E-94	1.6	Gm973	4E-31	1.7
Ldlrad3	2E-262	1.6	Slc26a2	7E-129	1.7
2310040G24Rik	2E-29	1.6	Ldlrad3	1E-266	1.7
Cp	4E-175	1.6	S100b	1E-255	1.7
Faah	4E-44	1.6	Tfrc	6E-47	1.7
Loxl4	8E-48	1.6	Cox4i2	7E-109	1.6
Has2os	9E-101	1.6	Tspan2	4E-79	1.6
Mlip	4E-88	1.6	Kcnq5	6E-278	1.6
Ifitm10	2E-87	1.6	Pde7b	2E-149	1.6
Pdzrn4	1E-182	1.6	Shroom2	6E-36	1.6
Mdfi	2E-72	1.5	Chchd10	8E-99	1.6
Peg3	0E+00	1.5	Vwc2	1E-35	1.6
Mei4	2E-65	1.5	Stc2	7E-39	1.6
Chchd10	2E-96	1.5	Dlg2	1E-81	1.6
S100b	6E-222	1.5	Mdfi	4E-91	1.6
Fjx1	4E-55	1.5	Slc6a9	4E-91	1.6
Kcnma1	1E-99	1.5	Plxnb1	7E-52	1.6

Fibroblast					
Control			*Notch2*$^{tm1.1Ecan}$		
Gene	*p*	FC	Gene	*p*	FC
Exo1	5E-93	3.9	Cdca3	2E-202	4.5
Kif20b	9E-111	3.9	Knstrn	2E-192	4.5
Bub1b	1E-127	3.9	Racgap1	7E-197	4.4
Kif20a	2E-84	3.8	Cit	1E-152	4.4
Cdca2	3E-104	3.8	Cdc25c	3E-133	4.4
Spc24	5E-115	3.8	Cdca8	3E-204	4.4
Racgap1	1E-102	3.8	Ckap2	1E-138	4.4
Cenpm	2E-118	3.7	Ncaph	8E-146	4.4
Uhrf1	4E-94	3.7	Cenpa	8E-173	4.3
Spdl1	1E-83	3.6	Diaph3	8E-154	4.3
Cdkn3	9E-99	3.6	Gas2l3	6E-109	4.2
Diaph3	5E-66	3.6	Kifc1	2E-124	4.2
Spc25	7E-74	3.6	Spc25	9E-148	4.2
Brip1	2E-82	3.6	Clspn	2E-141	4.2
Parpbp	9E-76	3.5	Nek2	2E-104	4.2
Ccnb2	5E-77	3.5	Brip1	1E-117	4.1
Ncapg	3E-99	3.5	Kntc1	5E-141	4.1
Kif11	4E-92	3.5	Kif20a	4E-175	4.1
Tacc3	3E-92	3.4	Spc24	1E-190	4.1
Kifc1	5E-72	3.4	Cdkn3	2E-158	4.0
Cit	2E-97	3.3	Kif22	3E-125	4.0
Kif22	9E-95	3.3	Polq	3E-87	4.0
Eldr	3E-65	3.3	Prr11	2E-107	3.9
Gas2l3	9E-62	3.3	Spdl1	6E-128	3.9
Rrm2	4E-69	3.3	Kif11	2E-149	3.9
Ercc6l	3E-70	3.2	Tacc3	4E-171	3.8
Cenph	1E-73	3.1	Arhgap11a	2E-135	3.8
Gen1	4E-65	3.1	Hist1h1b	1E-98	3.8
Bard1	4E-66	3.0	Cenpm	7E-183	3.8
Slfn9	5E-47	2.9	Lockd	3E-153	3.7
Hist1h1b	9E-47	2.9	Bard1	4E-81	3.7
Ncapg2	2E-62	2.9	Ccnb2	5E-150	3.7
Arhgap11a	8E-58	2.9	Gen1	3E-100	3.7
Efcab11	7E-57	2.9	Mns1	1E-132	3.7
Lockd	3E-65	2.8	Ska3	5E-91	3.6
Cks2	4E-46	2.8	Foxm1	2E-104	3.6
Ccne2	1E-34	2.8	Uhrf1	1E-102	3.6
Brca1	6E-54	2.8	Efcab11	2E-105	3.5
Smc2	5E-38	2.7	Rrm2	7E-142	3.4
Rad54b	2E-48	2.7	Parpbp	2E-89	3.4
Incenp	3E-47	2.7	Eldr	6E-93	3.4
Rad51	4E-61	2.7	Ercc6l	3E-102	3.4
Lmnb1	4E-63	2.6	Cks2	1E-93	3.3
Cdk1	7E-39	2.6	Incenp	3E-110	3.3
Mcm5	3E-63	2.6	Cenph	5E-135	3.3
Gmnn	9E-55	2.6	Ncapd2	5E-127	3.2
Trip13	3E-52	2.6	Cenpn	2E-95	3.2
Ncapd2	1E-65	2.5	Mastl	3E-94	3.2
Cenpn	1E-68	2.5	Ncapg2	3E-101	3.2
Tcf19	9E-48	2.5	Rad51	9E-89	3.2
Fgf14	1E-17	2.5	Cenpu	3E-78	3.1
Cip2a	3E-53	2.5	Slfn9	9E-88	3.1
Mns1	1E-63	2.5	Cdk1	6E-92	3.1
Stmn1	2E-47	2.4	Cip2a	2E-102	3.1
Hist1h2ap	9E-36	2.3	Fam83d	5E-82	3.0
Cdc45	7E-33	2.3	Ccdc18	2E-79	3.0
Dbf4	1E-50	2.3	Smc2	3E-97	3.0
Fignl1	1E-45	2.3	Brca1	1E-84	3.0
Ccdc18	2E-49	2.3	Stil	5E-86	3.0
Mastl	9E-53	2.3	Mcm5	1E-95	2.9
Chaf1b	4E-35	2.2	Trip13	3E-84	2.9
Rrm1	8E-44	2.2	Hist1h2ap	3E-55	2.8
Ube2t	3E-41	2.2	Fgf14	2E-54	2.8
Mad2l1	4E-39	2.2	Lmnb1	2E-100	2.8

Undefined					
Control			*Notch2*$^{tm1.1Ecan}$		
Gene	*p*	FC	Gene	*p*	FC
Gm16070	7E-79	1.3	Gdf10	3E-61	1.3
Hapln1	3E-139	1.3	Gm13919	6E-16	1.3
Cox4i2	2E-59	1.3	Fry	8E-95	1.3
Hrct1	3E-25	1.3	Ucma	8E-164	1.3
Sdk2	1E-117	1.3	Extl1	5E-16	1.3
S100b	2E-111	1.3	Lcn2	3E-21	1.3
Parm1	2E-23	1.3	Gm16070	2E-37	1.3
Stk26	3E-70	1.3	Upb1	3E-14	1.3
Myom1	3E-27	1.3	Cox4i2	4E-52	1.3
Soat1	3E-46	1.2	Acox2	6E-19	1.3
Upb1	6E-29	1.2	Loxl4	2E-15	1.3
Rab11fip4	2E-47	1.2	Sdk2	5E-77	1.2
Acox2	3E-37	1.2	Prkcz	4E-14	1.2
Itga10	7E-63	1.2	Itga10	2E-37	1.2
Syn3	6E-86	1.2	Chst11	8E-31	1.2
Ccn2	1E-119	1.2	Rab11fip4	7E-34	1.2
Raph1	5E-84	1.2	Car6	7E-06	1.2
Omd	1E-103	1.2	Tubb2a	1E-25	1.2
Chst11	4E-48	1.2	Dlg2	6E-14	1.2
Sorbs2os	2E-31	1.2	Xylt1	6E-100	1.2
Pir	8E-23	1.2	Spint2	3E-04	1.2
Eps8l2	2E-75	1.2	Mei4	2E-15	1.1
Nrip2	3E-16	1.2	Sorbs2os	2E-16	1.1
Trpv4	5E-64	1.2	Slc7a3	2E-11	1.1
Prkcb	4E-15	1.2	Metrn	2E-19	1.1
Cth	4E-24	1.1	Nebl	1E-28	1.1
Kcna6	2E-20	1.1	Syn3	4E-24	1.1
Kcnq5	3E-138	1.1	Pcsk6	2E-07	1.1
Itga6	4E-24	1.1	Gdf15	2E-11	1.1
Pcsk6	5E-11	1.1	Hsd17b1	4E-07	1.1
Vps37b	3E-44	1.1	Wwp2	6E-58	1.1
Extl1	2E-15	1.1	Klf15	2E-10	1.1
Adm2	2E-10	1.1	Sorbs1	5E-23	1.1
Chchd10	3E-19	1.1	Smoc2	6E-18	1.1
Ppp1r3c	4E-18	1.1	Slc24a5	1E-01	1.1
Efcab1	2E-23	1.1	St3gal6	1E-61	1.1
Slc16a14	8E-08	1.1	Chadl	1E-09	1.1
Abcb9	1E-08	1.1	Dock8	4E-33	1.1
Thsd4	5E-09	1.1	2610035D17Rik	7E-18	1.1
Wwp2	2E-86	1.1	Satb1	4E-07	1.0
Npas3	4E-107	1.1	Tmem154	3E-06	1.0
Lmcd1	4E-45	1.1	Gm10371	4E-06	1.0
Galnt3	1E-20	1.1	Abcc4	6E-09	1.0
Lars2	7E-01	1.1	Psph	3E-15	1.0
Dlg2	8E-17	1.1	P3h2	3E-25	1.0
Ldlrad3	4E-76	1.1	Itga6	5E-15	1.0
Map3k5	7E-33	1.1	Fjx1	3E-08	1.0
Sorbs2	4E-78	1.0	Rpgrip1	7E-03	1.0
Brwd3	4E-08	1.0	Ppp1r3c	1E-11	1.0
Gdf15	3E-18	1.0	Ldlrad3	1E-49	1.0
D630045J12Rik	1E-45	1.0	Stbd1	7E-05	1.0
Nt5e	3E-20	1.0	Ninj1	5E-47	1.0
P3h2	3E-42	1.0	Vps37b	9E-18	1.0
Isg20	2E-28	1.0	Kank1	5E-17	1.0
Spint2	2E-18	1.0	Ss18l2	6E-14	1.0
Klf5	8E-08	1.0	Cdsn	2E-17	1.0
Gramd2	6E-11	1.0	Gng13	2E-08	1.0
Crybg1	1E-08	1.0	B3galt1	1E-30	1.0
Stxbp2	2E-05	1.0	Dcaf12l1	1E-07	1.0
Vill	7E-15	1.0	Syt1	3E-03	1.0
Mei4	2E-18	1.0	Adm2	1E-04	1.0
Sorbs1	3E-35	1.0	Nrip2	6E-11	1.0
Rorc	1E-15	1.0	Has2os	3E-16	0.9
Spsb4	6E-15	1.0	Pla2g12a	2E-08	0.9

Articular/Synovial Fibroblast					
Control			*Notch2*$^{tm1.1Ecan}$		
Gene	*p*	FC	Gene	*p*	FC
Apol9a	3E-49	2.2	Nkain4	5E-31	2.0
Arsj	6E-118	2.2	Adamtsl5	1E-86	2.0
Scara5	8E-66	2.2	Ucp2	7E-37	2.0
Inhbb	4E-42	2.2	Ednra	3E-58	1.9
Nkain4	5E-45	2.1	Sparcl1	5E-47	1.9
C1qtnf7	8E-49	2.1	C1qtnf7	5E-27	1.9
Plekha2	2E-71	2.1	Add3	7E-103	1.9
Eln	5E-114	2.1	Gm12158	9E-31	1.9
Tmem140	2E-71	2.1	Mfap5	7E-123	1.9
Pdgfd	4E-48	2.1	Plekha2	2E-39	1.9
Col6a6	3E-24	2.1	Slc7a7	3E-15	1.9
Adamtsl5	5E-170	2.1	Tmem140	9E-52	1.9
Add3	4E-176	2.1	Fstl1	2E-206	1.9
A330102I10Rik	6E-42	2.1	Arhgap20	1E-115	1.9
Gas7	4E-88	2.1	Lama2	8E-51	1.9
Sema3d	7E-56	2.1	Itih5	5E-44	1.9
Sbsn	3E-53	2.1	Loxl1	6E-174	1.9
Sema3c	3E-104	2.1	Tnik	2E-38	1.9
Gm26740	1E-37	2.1	Slurp1	2E-26	1.8
Rcan2	1E-141	2.1	Plac9b	2E-21	1.8
Adam33	4E-121	2.1	Itgb3	1E-18	1.8
Fam124a	2E-55	2.1	Rragd	2E-34	1.8
Tmem158	1E-109	2.0	Osr2	3E-107	1.8
Rbpms	4E-85	2.0	Kcnab1	1E-17	1.8
3300005D01Rik	4E-37	2.0	Arsj	6E-55	1.8
Rad51b	2E-128	2.0	Selenbp1	3E-54	1.8
Itih5	3E-74	2.0	Gm12153	4E-23	1.7
Fstl1	7E-283	2.0	Rspo2	2E-103	1.7
Slc16a10	1E-29	2.0	Arhgap6	6E-117	1.7
Sparcl1	7E-67	2.0	Entpd2	1E-44	1.7
Osr1	3E-98	2.0	Osr1	4E-66	1.7
Ebf1	9E-236	2.0	Hgf	1E-43	1.7
Selenbp1	2E-97	1.9	Phf11d	4E-16	1.7
Rspo2	3E-148	1.9	Fam126a	6E-38	1.7
Gsta4	2E-86	1.9	Maob	7E-20	1.7
Sdk1	2E-76	1.9	Bmp6	1E-16	1.7
Ston2	3E-21	1.9	Rcan2	1E-87	1.7
Gngt2	2E-24	1.9	Rasl12	1E-29	1.7
Vav3	1E-26	1.9	Sirpa	2E-58	1.7
Loxl1	2E-185	1.9	Ntn4	4E-48	1.7
Entpd2	2E-59	1.9	Igfbp6	1E-51	1.7
Medag	8E-152	1.9	Rad51b	2E-79	1.7
Flrt2	7E-92	1.9	Ebf2	2E-65	1.7
Serpina3n	3E-25	1.8	Adamts1	2E-63	1.6
Nox4	4E-65	1.8	Fbn1	9E-145	1.6
Hgf	3E-72	1.8	Bcl3	1E-08	1.6
Rab7b	1E-52	1.8	Cd44	2E-53	1.6
Mitf	4E-115	1.8	Cdh13	4E-46	1.6
Ntrk3	5E-80	1.8	Rgs2	4E-11	1.6
Spsb1	6E-57	1.8	Clec2d	8E-61	1.6
Pcolce2	2E-74	1.8	Cdr1os	1E-21	1.6
Ifih1	8E-28	1.8	C1qtnf2	1E-27	1.6
Lama2	4E-53	1.7	Lrp4	3E-20	1.6
Fbn1	2E-170	1.7	Trim16	8E-26	1.6
Vit	2E-26	1.7	Plcxd2	5E-21	1.6
Snhg11	2E-22	1.7	Cxcl12	1E-70	1.6
Osr2	8E-139	1.7	Bace2	3E-27	1.6
Mrgprf	4E-30	1.7	Itga9	2E-31	1.6
Vegfd	8E-84	1.7	Ramp1	4E-22	1.6
Sned1	4E-118	1.7	Sv2c	1E-14	1.6
Phex	1E-22	1.7	Ano5	1E-27	1.6
Phf11d	1E-29	1.7	Plekho2	2E-44	1.6
Trim16	8E-43	1.7	Ahnak2	1E-112	1.6
C1qtnf2	6E-52	1.7	Ramp2	3E-37	1.6

Limb Mesenchyme					
Control			$Notch2^{tm1.1Ecan}$		
Gene	*p*	FC	Gene	*p*	FC
Prrx2	3E-282	1.2	Cacna1a	8E-52	1.1
Nr2f2	9E-67	1.2	Il17rd	1E-20	1.1
Olfml3	2E-209	1.2	Marcks	2E-297	1.1
Akap12	9E-111	1.2	Cacna1c	5E-66	1.1
Serpinf1	0E+00	1.2	Shroom3	7E-09	1.1
Dlk1	4E-19	1.2	Adam12	5E-31	1.1
Ncald	1E-28	1.2	Erc2	3E-16	1.1
Rspo3	4E-147	1.2	Epha5	4E-42	1.1
Plcb4	4E-36	1.2	Podn	2E-30	1.1
Jcad	9E-69	1.2	Dclk1	2E-166	1.1
Olfml1	8E-35	1.2	Kazn	8E-40	1.1
Hmcn1	3E-43	1.1	Meox2	2E-54	1.1
Meox2	2E-81	1.1	S100a4	3E-201	1.1
Gstm2	3E-75	1.1	Rspo3	8E-123	1.1
Scara5	2E-41	1.1	Col1a2	0E+00	1.1
Gpm6b	3E-160	1.1	Cxcl12	2E-78	1.1
Ccn3	5E-174	1.1	Entpd2	4E-26	1.1
Angptl4	6E-28	1.1	Plcb4	9E-31	1.1
Kctd12	4E-117	1.1	Mgst1	1E-100	1.1
Entpd2	4E-28	1.1	Socs2	3E-18	1.0
Mfap5	7E-133	1.1	Rnase4	3E-89	1.0
Cacnb3	6E-22	1.1	Cpa6	1E-31	1.0
Gstt1	5E-49	1.1	Cyp7b1	3E-24	1.0
Eya2	3E-14	1.1	Tmem119	1E-42	1.0
Prr16	3E-76	1.1	Adamts5	3E-116	1.0
Gm2115	3E-23	1.1	Vegfd	3E-43	1.0
Eda	7E-46	1.1	Peli2	3E-35	1.0
Ifitm3	7E-227	1.1	Rab32	5E-31	1.0
Peli2	1E-44	1.1	Fbn1	1E-125	1.0
Marcks	0E+00	1.1	Kif26b	3E-18	1.0
Tmem47	2E-59	1.1	Fndc1	4E-107	1.0
Gm28875	4E-23	1.1	Cercam	4E-69	1.0
Thsd7a	4E-23	1.1	Gas6	4E-117	1.0
Kazn	3E-31	1.1	B4galnt1	3E-20	1.0
Dclk1	3E-175	1.1	Rbms3	4E-175	1.0
Gm12158	7E-21	1.1	St8sia2	6E-20	1.0
Dock10	3E-25	1.1	Gpc4	9E-132	1.0
Perp	2E-16	1.1	Trpm3	3E-12	1.0
Ank3	4E-82	1.1	P4ha3	8E-56	1.0
Aqp1	3E-57	1.1	Cacna1g	3E-25	1.0
Ebf2	2E-33	1.1	Ifi204	4E-16	1.0
Kcnab1	8E-11	1.1	Fzd5	3E-21	1.0
Cacna1c	2E-54	1.1	St3gal1	3E-29	1.0
Lgals9	1E-46	1.1	Pde1a	2E-20	1.0
Tsc22d3	1E-41	1.1	Nfatc4	1E-38	1.0
Rgs17	4E-31	1.0	Rgs17	5E-23	1.0
Pde1a	2E-35	1.0	Agtr2	2E-26	1.0
Cpxm1	2E-95	1.0	Fbn2	4E-103	1.0
Slc5a3	7E-22	1.0	Cdh13	6E-46	1.0
Lgi2	3E-18	1.0	Adam19	5E-33	1.0
Ptges	2E-33	1.0	Adam33	2E-27	1.0
Lifr	6E-20	1.0	Aqp1	4E-37	0.9
Ereg	7E-17	1.0	Mgll	4E-30	0.9
Vstm4	4E-26	1.0	Ifitm3	3E-173	0.9
Basp1	9E-101	1.0	Col8a2	2E-67	0.9
Cxcl14	1E-09	1.0	Fbln5	3E-148	0.9
Col1a2	0E+00	1.0	Kctd11	1E-14	0.9
Slc1a6	2E-31	1.0	Carmn	2E-41	0.9
Agtr1a	3E-31	1.0	Irak3	9E-21	0.9
Tbc1d16	7E-19	1.0	4931406P16Rik	2E-56	0.9
Il1rap	7E-39	1.0	Tbc1d16	1E-15	0.9
Isg15	5E-20	1.0	Plcl1	3E-43	0.9
Cpxm2	5E-23	1.0	Palmd	2E-57	0.9
Cttnbp2	4E-23	1.0	Calhm5	7E-32	0.9

Chondrogenic					
Control			$Notch2^{tm1.1Ecan}$		
Gene	*p*	FC	Gene	*p*	FC
Itga10	3E-105	1.5	Zim1	4E-105	1.6
Rnf180	5E-26	1.5	Tex14	4E-188	1.6
Prkcb	9E-32	1.5	Gramd2	6E-28	1.6
Spsb4	1E-46	1.4	Gm13919	5E-64	1.6
Smoc2	3E-95	1.4	Nexmif	2E-27	1.5
C230038L03Rik	2E-40	1.4	C530008M17Rik	3E-42	1.5
Pir	2E-55	1.4	Aif1l	3E-37	1.5
Cxadr	3E-53	1.4	Syt1	3E-33	1.5
Raph1	6E-205	1.4	Has2os	6E-99	1.5
Ppp1r3c	6E-42	1.4	Prkcb	3E-40	1.5
Etv5	2E-66	1.4	Nrip2	2E-57	1.5
Camk4	9E-198	1.4	2610035D17Rik	1E-80	1.5
Vps37b	6E-114	1.4	Pitpnc1	6E-140	1.5
Tex14	3E-176	1.4	Slc40a1	9E-12	1.5
Msmp	5E-18	1.4	Sema6a	7E-57	1.5
Vwc2	6E-44	1.4	Rab11fip4	3E-123	1.5
Map3k5	1E-86	1.4	Kcnma1	1E-77	1.5
Pdgfc	1E-78	1.4	Kcnt2	7E-85	1.5
Gm10848	4E-26	1.4	Pcolce2	6E-67	1.5
Arhgef3	2E-116	1.4	Stk32b	4E-81	1.5
Foxd1	9E-59	1.4	Nacad	3E-19	1.5
Slc7a3	2E-45	1.4	Camk4	3E-180	1.5
Col11a1	0E+00	1.4	Peg3	0E+00	1.5
Kctd14	2E-28	1.4	Raph1	2E-183	1.5
Stk32b	1E-72	1.4	Tox	7E-248	1.4
Hsd17b1	1E-55	1.4	Nt5e	2E-52	1.4
Prune2	3E-46	1.3	Vps37b	1E-91	1.4
Fabp5	2E-02	1.3	Slc17a9	2E-21	1.4
Adm2	1E-36	1.3	Ndrg2	3E-33	1.4
Ninj1	8E-266	1.3	Adm2	3E-37	1.4
Gm973	5E-25	1.3	Gdf10	1E-116	1.4
Zdbf2	3E-30	1.3	Fjx1	1E-50	1.4
Bend6	4E-27	1.3	Col11a1	0E+00	1.4
Hrct1	1E-27	1.3	C230038L03Rik	9E-29	1.4
Pitpnc1	3E-103	1.3	Slc7a3	3E-52	1.4
Slc6a9	3E-64	1.3	Cyb5r2	1E-67	1.4
Slc26a2	3E-77	1.3	Sobp	1E-152	1.4
Enah	1E-187	1.3	Kdm1b	7E-35	1.4
Igf2	7E-226	1.3	Upb1	2E-51	1.4
Klf15	2E-46	1.3	Kctd16	1E-47	1.4
Prtg	3E-36	1.3	Rnf144b	9E-48	1.4
Plcg2	1E-17	1.3	Nipal2	1E-24	1.4
Gm13269	1E-27	1.3	Ss18l2	3E-145	1.4
Dlg2	2E-63	1.3	Parm1	6E-56	1.4
Ctdspl	3E-276	1.3	D630003M21Rik	2E-25	1.4
Nrep	2E-57	1.3	P3h2	8E-84	1.4
Spata13	2E-22	1.3	Cd200	4E-16	1.3
Scube1	3E-16	1.3	Adhfe1	1E-68	1.3
Klf5	8E-40	1.3	Pde4dip	8E-182	1.3
Gm49708	1E-40	1.3	Faah	8E-22	1.3
Dlx5	3E-37	1.2	Abcb9	2E-28	1.3
Arhgef28	9E-132	1.2	Arhgef3	1E-99	1.3
Car6	5E-22	1.2	Has2	1E-55	1.3
Kcnc3	1E-33	1.2	Gdf15	2E-53	1.3
Shroom2	1E-29	1.2	Gm15414	1E-32	1.3
Npas3	7E-229	1.2	Msi2	0E+00	1.3
Kcnt2	2E-79	1.2	Hsd17b1	6E-57	1.3
Gm41556	1E-22	1.2	Scube1	2E-20	1.3
Fat3	6E-24	1.2	Pdgfc	2E-63	1.3
Tenm4	8E-216	1.2	Tenm4	9E-216	1.3
Crybg1	3E-18	1.2	Tubb2a	6E-110	1.3
Bmp5	4E-138	1.2	Peg10	3E-15	1.3
Glt28d2	9E-19	1.2	Metrn	4E-77	1.3
Net1	1E-39	1.2	Ninj1	9E-231	1.3

Fibroblast					
Control			$Notch2^{tm1.1Ecan}$		
Gene	*p*	FC	Gene	*p*	FC
Pole	2E-41	2.1	Rrm1	1E-91	2.7
Fam111a	2E-23	2.0	Stmn1	5E-94	2.7
Slurp1	2E-37	2.0	Ccne2	4E-47	2.7
Blm	2E-34	2.0	Gmnn	2E-79	2.7
Cenpk	1E-40	2.0	Fignl1	2E-75	2.6
Zwilch	7E-40	2.0	Fam111a	7E-64	2.5
Cenpq	2E-43	2.0	Mad2l1	4E-87	2.5
Fam83d	9E-37	2.0	Chaf1b	2E-57	2.5
Plk4	6E-41	1.9	Tcf19	1E-53	2.5
Lig1	5E-33	1.9	Dbf4	3E-80	2.5
St6galnac2	3E-21	1.9	Gtse1	9E-55	2.4
2810408I11Rik	2E-27	1.9	Spag5	2E-76	2.4
Pask	2E-27	1.9	Cep128	1E-64	2.3
Mybl1	1E-36	1.8	Rad54b	2E-63	2.3
Fen1	3E-29	1.8	Cdc45	3E-55	2.3
Spag5	2E-36	1.8	Zwilch	4E-63	2.3
Ntrk3	4E-75	1.8	Ube2t	2E-78	2.3
Csmd3	1E-11	1.8	Cenpq	1E-77	2.3
Dhfr	3E-21	1.8	Lig1	6E-61	2.3
Bora	4E-18	1.8	Cenpl	9E-59	2.3
Cep128	6E-40	1.7	Nrm	2E-50	2.3
Kpna2	5E-17	1.7	Dhfr	3E-55	2.3
Lin9	4E-34	1.7	Kpna2	4E-35	2.2
Dpp4	4E-37	1.7	Kif24	5E-41	2.2
Nkain4	1E-34	1.7	Hmgb2	6E-73	2.2
Tyms	6E-27	1.7	Hist1h2ae	8E-27	2.1
Rfc5	3E-32	1.7	Plk4	6E-63	2.1
Rad18	1E-29	1.6	Cenpk	1E-64	2.1
Plekha7	2E-37	1.6	2810408I11Rik	8E-48	2.1
Prim1	3E-30	1.6	Pkmyt1	3E-38	2.0
Stil	1E-22	1.6	Ccne1	2E-24	2.0
Syne2	2E-29	1.6	Mcm8	8E-39	2.0
Hist1h2ae	9E-11	1.6	Pole	2E-51	2.0
Chaf1a	3E-27	1.6	Cenpp	3E-69	1.9
Ccne1	7E-13	1.6	Bora	2E-35	1.9
Serpinb8	7E-29	1.6	Rad18	1E-50	1.9
Bend5	2E-30	1.5	H2afx	3E-46	1.9
Wdhd1	3E-19	1.5	Tyms	4E-45	1.9
Hist2h2ac	1E-13	1.5	Wdr62	4E-35	1.8
Nek3	6E-15	1.5	Wdhd1	1E-27	1.8
Atad2	5E-19	1.5	Cdkn2d	7E-39	1.8
Hmgb2	2E-47	1.5	Blm	2E-44	1.8
Hells	7E-13	1.5	Nek3	3E-16	1.8
Ccn5	4E-155	1.5	Reep4	3E-35	1.8
Slc4a10	6E-13	1.5	Mybl1	4E-41	1.8
Cenpp	4E-32	1.5	Lmnb2	6E-37	1.8
Kif24	2E-16	1.4	Serpinb8	2E-39	1.8
Pola1	4E-17	1.4	St6galnac2	4E-26	1.8
Cdkn2d	2E-22	1.4	Dck	2E-29	1.8
Zgrf1	4E-18	1.4	Ncapd3	4E-47	1.8
Anxa8	2E-47	1.4	Brca2	4E-43	1.7
Atad5	4E-19	1.4	Mboat1	3E-27	1.7
Rcan2	2E-78	1.4	G2e3	1E-41	1.7
C1ql3	1E-17	1.4	Syne2	6E-50	1.7
Ano5	2E-26	1.4	Fen1	2E-33	1.7
Slc9a3r1	2E-36	1.4	Cdc25b	8E-24	1.7
Eln	3E-17	1.4	Kif18a	3E-38	1.7
Brca2	6E-19	1.4	Chaf1a	1E-47	1.7
Cep152	6E-23	1.4	Atad2	7E-47	1.7
Mcm3	4E-21	1.4	Hist2h2ac	7E-26	1.7
Mms22l	7E-23	1.3	Trim59	2E-48	1.7
Npr3	1E-91	1.3	Mms22l	6E-42	1.7
Serpinb1a	3E-56	1.3	Cenps	4E-39	1.6
Rangap1	3E-22	1.3	Slurp1	8E-14	1.6

Undefined					
Control			$Notch2^{tm1.1Ecan}$		
Gene	*p*	FC	Gene	*p*	FC
Gm45025	2E-06	1.0	Raph1	6E-33	0.9
Bmp5	2E-45	1.0	Isg20	3E-16	0.9
4930523C07Rik	2E-39	1.0	Bend6	5E-06	0.9
Smad9	5E-13	0.9	Rnf144b	4E-04	0.9
Mlip	4E-15	0.9	Stc2	1E-08	0.9
Cybrd1	2E-06	0.9	Has2	8E-11	0.9
Car6	4E-05	0.9	Igf2	3E-42	0.9
Dock8	7E-40	0.9	Pde4dip	3E-37	0.9
Dcaf12l1	4E-12	0.9	Nprl2	1E-01	0.9
Edar	1E-08	0.9	Dlk1	4E-03	0.9
Gm10371	2E-11	0.9	Cpe	1E-104	0.9
Ooep	1E-03	0.9	Parm1	2E-07	0.9
Ecm2	4E-11	0.9	Atp6v0a4	1E-17	0.9
Cmtm8	2E-06	0.9	Ern1	8E-08	0.9
Prkcz	5E-17	0.9	Ccn2	2E-30	0.9
Timp3	1E-92	0.9	Map3k5	1E-11	0.9
Slc7a3	8E-09	0.9	Cox6a2	9E-01	0.9
Ninj1	2E-71	0.9	Tex14	3E-21	0.9
Plxnb1	2E-12	0.9	Smad9	2E-07	0.9
Dusp14	5E-17	0.9	Nt5e	2E-05	0.9
Ctdspl	2E-94	0.9	Pdrg1	4E-20	0.9
Cxadr	3E-14	0.9	Nqo1	4E-08	0.9
B3galt1	5E-49	0.9	Cyb5r2	4E-11	0.9
Fbxo7	1E-21	0.9	Fbxo7	8E-13	0.9
Klf15	3E-18	0.9	C030006K11Rik	1E+00	0.9
Metrn	1E-18	0.9	Kcnq5	3E-56	0.9
Arhgef3	1E-29	0.9	Dlx5	4E-03	0.9
Cp	4E-20	0.9	Abcb9	2E-05	0.9
Usp6nl	2E-16	0.9	S100a1	3E-31	0.9
Jph1	1E-05	0.9	Omd	1E-24	0.8
Lctl	3E-05	0.9	Ifitm10	5E-12	0.8
Slc6a9	7E-12	0.9	Mlip	2E-06	0.8
Platr22	4E-13	0.9	Sorbs2	1E-37	0.8
Ell2	3E-54	0.9	Il17d	5E-08	0.8
Abtb2	5E-43	0.9	Kcnt2	2E-06	0.8
Stk32b	4E-31	0.9	Col11a1	1E-140	0.8
Esr1	4E-16	0.9	Comp	4E-75	0.8
Fjx1	4E-12	0.9	Adhfe1	2E-08	0.8
Slc20a1	1E-26	0.9	Klc3	2E-02	0.8
Cpe	7E-118	0.9	Arhgef3	9E-14	0.8
Kank1	4E-39	0.9	Chac1	1E-04	0.8
Tubb2a	1E-20	0.9	C1qtnf4	1E-04	0.8
Msi2	4E-121	0.9	Slc6a9	2E-08	0.8
Mtus2	2E-12	0.9	Tprg	4E-05	0.8
Cpne8	8E-103	0.9	Kdm1b	2E-02	0.8
Rbp4	8E-49	0.9	Timp3	8E-32	0.8
Fxyd6	2E-32	0.9	Acot1	1E-03	0.8
2610035D17Rik	4E-20	0.9	Sgip1	2E-04	0.8
Shroom2	8E-09	0.8	Prtg	8E-05	0.8
Pitpnc1	2E-16	0.8	Kcna6	7E-06	0.8
Gm11146	2E-03	0.8	Tuba1c	3E-08	0.8
Dmd	3E-55	0.8	Npas3	2E-34	0.8
Ak4	3E-08	0.8	Plxnb1	2E-04	0.8
Satb1	6E-10	0.8	Pde7b	7E-25	0.8
Serinc5	4E-12	0.8	Pitpnc1	2E-08	0.8
Pde4dip	2E-53	0.8	Mdfi	2E-05	0.8
Has2os	9E-17	0.8	Enho	1E-03	0.8
Sap30	1E-25	0.8	Got1	1E-02	0.8
Tspan2	6E-10	0.8	Msi2	5E-52	0.8
Col11a1	1E-147	0.8	Pam	5E-98	0.7
Ets1	2E-09	0.8	D630045J12Rik	2E-08	0.7
Pdrg1	2E-30	0.8	Msmp	3E-05	0.7
Myo1d	5E-65	0.8	Tmem117	4E-09	0.7
Kcnt2	7E-13	0.8	Cpne8	1E-42	0.7

Articular/Synovial Fibroblast					
Control			$Notch2^{tm1.1Ecan}$		
Gene	*p*	FC	Gene	*p*	FC
Axl	8E-103	1.7	Vegfd	1E-52	1.6
Anxa8	1E-56	1.7	1500009L16Rik	9E-40	1.6
Adamts1	3E-104	1.7	Tbx18	2E-69	1.5
Aldh1l1	3E-34	1.7	Mocos	5E-15	1.5
Rragd	2E-32	1.7	Gpnmb	3E-86	1.5
Serpinb8	2E-41	1.7	Zeb2	2E-120	1.5
Vcan	9E-102	1.7	Sipa1l2	2E-17	1.5
Galm	7E-55	1.7	Mgst1	1E-92	1.5
Ampd3	1E-34	1.7	Pdpn	2E-82	1.5
Fam126a	4E-66	1.7	Ugcg	5E-38	1.5
Cd44	8E-91	1.7	Gsn	3E-122	1.5
Nsdhl	1E-67	1.7	Gbp7	3E-13	1.5
Gpnmb	4E-145	1.7	Ldb2	3E-71	1.5
Gm12158	1E-32	1.7	Procr	7E-18	1.5
Mustn1	5E-42	1.7	Flrt2	1E-47	1.5
Ifi204	1E-33	1.6	Serpinb8	1E-23	1.5
Crip1	1E-110	1.6	Nbl1	8E-70	1.5
Arhgap6	7E-143	1.6	Vav3	3E-20	1.5
Bend5	4E-42	1.6	Pdgfd	2E-15	1.5
Zeb2	2E-164	1.6	Mitf	3E-58	1.5
Arhgap22	3E-67	1.6	Sbsn	1E-18	1.5
Mgst1	1E-120	1.6	Tmem158	2E-41	1.5
Cdc42ep2	3E-50	1.6	Sema3b	3E-42	1.5
Gsn	6E-168	1.6	Eepd1	2E-12	1.5
Cfb	9E-26	1.6	Nsdhl	3E-43	1.5
Sirpa	2E-89	1.6	Ror1	1E-86	1.5
Mvd	3E-45	1.6	Selenop	1E-61	1.5
Ror1	5E-113	1.6	Gas2	6E-98	1.5
Olfml2b	1E-96	1.6	Nnmt	1E-76	1.4
Gm28535	3E-21	1.6	Prrg3	9E-25	1.4
S100a4	4E-108	1.6	Kazn	5E-37	1.4
Ccdc9b	3E-18	1.6	Lbp	2E-26	1.4
Ramp1	7E-28	1.6	Idi1	9E-49	1.4
Cyp1b1	3E-97	1.6	Lgals9	9E-31	1.4
Ramp2	3E-78	1.6	Ssc5d	2E-57	1.4
Igfbp6	2E-62	1.6	Ackr3	1E-41	1.4
Gsdmd	9E-21	1.6	Cdc42ep2	2E-29	1.4
Fgf10	1E-28	1.6	Mrgprf	4E-19	1.4
Irak3	8E-54	1.6	2310001H17Rik	2E-15	1.4
Pdpn	9E-126	1.6	Gas7	8E-26	1.4
Ahnak2	2E-175	1.6	Sned1	2E-57	1.4
Larp6	6E-25	1.6	Celf2	5E-109	1.4
Plac9b	2E-18	1.6	Crip1	7E-87	1.4
Tm7sf2	4E-33	1.6	Fbln1	6E-47	1.4
Slfn5	8E-22	1.5	S100a4	7E-71	1.4
Arhgap20	2E-142	1.5	Olfml2b	7E-52	1.4
Rgs2	2E-10	1.5	Gm15261	1E-30	1.4
Gm10125	1E-23	1.5	Gm20559	7E-30	1.4
Gm15261	9E-44	1.5	Mvd	2E-30	1.4
2310001H17Rik	3E-21	1.5	Rab7b	8E-24	1.4
Fbln1	2E-73	1.5	Gab2	1E-34	1.4
Ugdh	9E-89	1.5	Gm11266	2E-15	1.4
Hmcn1	1E-45	1.5	Spsb1	6E-26	1.4
Anxa3	3E-88	1.5	Cyp1b1	1E-43	1.4
Clec2d	9E-58	1.5	Npr3	6E-23	1.4
Plin4	3E-34	1.5	Scara5	1E-13	1.4
Bdh2	7E-35	1.5	Col3a1	2E-193	1.4
B3galnt1	7E-36	1.5	B4galt5	1E-25	1.4
Gca	2E-18	1.5	Bdh2	1E-25	1.4
Lefty1	2E-44	1.5	Sgip1	2E-21	1.4
Ildr2	2E-21	1.5	Akap6	2E-14	1.3
Plekho2	5E-47	1.5	St3gal1	3E-37	1.3
Akap12	6E-102	1.5	Zyx	1E-63	1.3
Lrp4	1E-39	1.5	Arhgap22	3E-30	1.3

Limb Mesenchyme					
Control			$Notch2^{tm1.1Ecan}$		
Gene	*p*	FC	Gene	*p*	FC
Bicc1	1E-202	1.0	Frmd4a	2E-34	0.9
Sh3bp1	3E-15	1.0	Slc1a6	2E-48	0.9
Il17rd	2E-19	1.0	Angptl4	2E-27	0.9
Flnc	6E-51	1.0	Lrrc75a	2E-16	0.9
Vcan	8E-91	1.0	Mmp19	1E-24	0.9
S100a4	3E-208	1.0	Lamb1	5E-57	0.9
Gm13052	4E-10	1.0	Ang	9E-16	0.9
Synm	3E-46	1.0	Lgals9	2E-33	0.9
Tmcc3	6E-22	1.0	Tnfaip2	1E-56	0.9
Cdc42ep5	7E-76	1.0	Nradd	2E-45	0.9
Pdlim1	8E-27	1.0	Nr2f2	2E-47	0.9
Cyp1b1	6E-30	1.0	Cavin2	5E-42	0.9
Fndc1	1E-81	0.9	Celf2	4E-99	0.9
Cacna1a	4E-46	0.9	Penk	3E-23	0.9
Ebf3	3E-38	0.9	Peak1	1E-86	0.9
Fzd4	6E-21	0.9	Cdc42ep5	1E-82	0.9
Lamb1	3E-53	0.9	Col4a1	5E-84	0.9
Gas2	1E-116	0.9	Olfml1	1E-16	0.9
Mmp19	4E-20	0.9	Pdlim1	3E-39	0.9
Cox6b2	2E-07	0.9	Mar3	9E-50	0.9
Pygb	4E-32	0.9	Plk2	2E-63	0.9
Mgst1	1E-114	0.9	Pdlim2	5E-100	0.9
Negr1	9E-20	0.9	Col5a1	3E-210	0.9
Nfatc4	2E-34	0.9	Il13ra1	2E-16	0.9
Fbn1	1E-106	0.9	Cacnb4	1E-11	0.9
Mmp23	5E-100	0.9	Tmeff2	3E-14	0.9
Rnase4	8E-85	0.9	Il3ra	3E-35	0.9
Adam19	3E-27	0.9	Map1b	1E-49	0.9
Tnfaip2	1E-41	0.9	Cavin4	2E-17	0.9
Nradd	3E-39	0.9	Nexn	9E-37	0.9
Wnt5b	6E-16	0.9	Cdc42ep1	1E-23	0.9
Rtn4r	3E-12	0.9	Dock10	2E-20	0.9
Fhod1	1E-10	0.9	Notch3	7E-15	0.8
Gm16083	4E-15	0.9	Dpysl3	3E-33	0.8
Itga1	9E-59	0.9	Tsc22d3	7E-30	0.8
Pdlim2	2E-112	0.9	Asxl3	9E-15	0.8
Adamts3	2E-16	0.9	Ptges	1E-16	0.8
Trpm3	1E-14	0.9	Trim16	9E-19	0.8
Gm4117	4E-28	0.9	Lsp1	5E-48	0.8
Cdh13	6E-56	0.9	Ccn4	2E-56	0.8
Rbms3	5E-203	0.9	Olfml3	5E-123	0.8
Bcl11a	3E-15	0.9	Tmcc3	1E-12	0.8
Adgra2	3E-22	0.9	F2r	8E-28	0.8
Cavin2	4E-55	0.9	Vstm4	2E-12	0.8
Fbln5	5E-148	0.9	Rftn1	1E-19	0.8
Gpr176	3E-24	0.9	Tmeff1	2E-26	0.8
F2r	4E-20	0.9	Arxes2	3E-31	0.8
Prr5	5E-17	0.9	Nid1	1E-61	0.8
Atp2b4	1E-10	0.9	Mgst3	3E-52	0.8
Map1b	3E-57	0.9	Nhsl1	1E-23	0.8
Gpc4	3E-94	0.9	Cdc42ep2	4E-20	0.8
Vegfd	6E-44	0.9	Lgals1	7E-288	0.8
Acss3	3E-15	0.9	Thbs2	9E-105	0.8
Csf1	2E-73	0.8	Slc6a6	2E-36	0.8
Ssc5d	7E-53	0.8	Epb41l3	1E-55	0.8
Celf2	3E-114	0.8	Kctd12	5E-68	0.8
Galnt13	3E-26	0.8	Mfap2	3E-73	0.8
Adam33	1E-37	0.8	Plpp1	9E-148	0.8
Mar3	1E-43	0.8	Cebpd	2E-31	0.8
Pkdcc	6E-18	0.8	Gm17501	7E-33	0.8
Col8a2	2E-73	0.8	Fam149a	1E-17	0.8
Snx7	4E-98	0.8	Gm2115	4E-18	0.8
Agtr2	1E-35	0.8	Gpm6b	2E-76	0.8
Plcl1	5E-37	0.8	Bicc1	2E-126	0.8

Chondrogenic					
Control			$Notch2^{tm1.1Ecan}$		
Gene	*p*	FC	Gene	*p*	FC
Atp6v0a4	5E-81	1.2	Gfpt2	2E-26	1.3
Map7d3	2E-60	1.2	Usp6nl	4E-77	1.3
Gprc5c	3E-30	1.2	Cybrd1	9E-18	1.3
Abcb9	7E-32	1.2	Gpc6	6E-237	1.3
Hmgcll1	7E-53	1.2	Nqo1	4E-41	1.3
Spats2l	3E-65	1.2	Dock8	6E-88	1.3
Adhfe1	3E-62	1.2	Fgfr1	8E-199	1.3
Slc17a9	3E-19	1.2	Hmgcll1	7E-39	1.3
Svopl	7E-22	1.2	Prtg	8E-40	1.3
Eps8l2	2E-96	1.2	Spp1	3E-24	1.3
Col24a1	2E-31	1.2	B3galt1	3E-85	1.3
Cobl	4E-140	1.2	Mtus1	1E-47	1.3
Nexmif	5E-19	1.2	Chac1	2E-72	1.3
Spint2	2E-40	1.2	Mlip	1E-60	1.3
Slc20a1	7E-87	1.2	Gprc5c	3E-21	1.2
Ern1	1E-61	1.2	Reps2	1E-15	1.2
Nos1ap	7E-38	1.2	Ern1	2E-67	1.2
Msi2	0E+00	1.2	Tmem117	2E-35	1.2
Ncam1	1E-76	1.2	Arhgef28	2E-101	1.2
Tox	6E-209	1.2	Tgfbi	1E-12	1.2
Rorc	5E-15	1.1	Tuba1c	4E-89	1.2
Gdf15	5E-49	1.1	Runx3	3E-36	1.2
Pde4dip	9E-152	1.1	Letm2	2E-36	1.2
Sema6a	8E-35	1.1	Enho	1E-55	1.2
Nrip2	2E-44	1.1	Ncam1	5E-74	1.2
Abtb2	1E-105	1.1	Car6	5E-19	1.2
C430049B03Rik	1E-78	1.1	Lrrc8c	2E-58	1.2
Kcnk1	1E-19	1.1	Bend6	2E-28	1.2
Ss18l2	1E-114	1.1	Spsb4	1E-32	1.2
Fgfr1	6E-207	1.1	Sort1	2E-32	1.2
Abcc4	7E-43	1.1	Il17d	2E-50	1.2
Samd4	1E-244	1.1	Abtb2	2E-101	1.2
Gramd2	7E-18	1.1	Enah	2E-166	1.2
Metrn	4E-49	1.1	Map3k5	1E-57	1.2
Nipal2	8E-29	1.1	Dlx5	3E-29	1.2
Pth1r	6E-18	1.1	Gpt2	3E-44	1.2
Sobp	7E-139	1.1	Dusp14	4E-50	1.2
Gm15834	3E-15	1.1	Net1	1E-34	1.2
Pcsk6	1E-11	1.1	B930036N10Rik	2E-73	1.2
Enho	9E-48	1.1	Rnf180	5E-20	1.2
Fgfrl1	8E-118	1.1	Abcc4	9E-42	1.2
Rbp4	7E-128	1.1	Msmp	4E-09	1.2
Usp6nl	1E-66	1.1	Gm15834	1E-17	1.2
Paqr3	5E-57	1.1	Dcaf12l1	3E-37	1.2
Gpc6	6E-226	1.1	Adgra3	8E-92	1.2
Pde7b	6E-76	1.1	Nrep	2E-53	1.1
B3galt1	5E-81	1.1	Tpd52l1	2E-28	1.1
Upb1	2E-40	1.1	Psph	2E-97	1.1
Gm12811	2E-23	1.1	Sox9	6E-211	1.1
4930545L23Rik	3E-16	1.1	Srebf1	4E-62	1.1
Rnf144b	1E-32	1.1	Pcdh9	2E-160	1.1
Cyb5r2	1E-54	1.1	Eps8l2	4E-80	1.1
D630003M21Rik	3E-14	1.1	Paqr3	8E-51	1.1
Chadl	2E-32	1.1	Eps8	3E-118	1.1
Nqo1	1E-27	1.1	Gm49708	3E-32	1.1
Lrrc8c	4E-56	1.1	Npas3	3E-172	1.1
Srebf1	1E-70	1.1	Slc20a1	2E-56	1.1
Pcdh9	1E-157	1.1	Fbxl2	8E-45	1.1
Pdrg1	4E-139	1.1	Gcnt1	3E-16	1.1
Adgra3	9E-81	1.0	Atp6v0a4	2E-50	1.1
Sox9	1E-212	1.0	Ctdspl	7E-207	1.1
Chac1	1E-61	1.0	C430049B03Rik	2E-61	1.1
1700113A16Rik	1E-17	1.0	Gm10371	2E-17	1.1
Ccn2	1E-153	1.0	Spata13	1E-17	1.1

Fibroblast					
Control			$Notch2^{tm1.1Ecan}$		
Gene	*p*	FC	Gene	*p*	FC
Wdr62	1E-19	1.3	Hells	7E-31	1.6
Zfp367	3E-22	1.3	Hjurp	4E-38	1.6
Ncapd3	2E-17	1.3	4930452B06Rik	6E-27	1.6
Mis18a	2E-17	1.3	Dtl	1E-33	1.6
Zdhhc2	2E-21	1.3	Lin9	3E-32	1.6
Hpgd	2E-19	1.3	Rangap1	5E-47	1.6
Dpt	5E-25	1.3	Prim1	1E-43	1.6
Rpa2	8E-18	1.3	Mis18a	2E-29	1.6
H2afx	3E-08	1.3	Slc26a7	3E-17	1.6
Pla1a	2E-19	1.3	Cep152	3E-27	1.6
G2e3	2E-18	1.3	Atad5	7E-28	1.5
Gins1	2E-15	1.2	Pola1	1E-24	1.5
4930452B06Rik	3E-18	1.2	Rad21	5E-55	1.5
Cenps	1E-22	1.2	Mcm3	3E-41	1.5
Gins2	1E-19	1.2	Cdk2	3E-22	1.5
Gm8739	2E-10	1.2	Rfc5	2E-41	1.5
Sh2d4a	3E-36	1.2	Gm8739	6E-18	1.4
Slc22a4	2E-19	1.2	Wee1	4E-27	1.4
Medag	1E-59	1.2	Tubb6	9E-42	1.4
Kif18a	4E-18	1.2	Ezh2	2E-48	1.4
Mcm6	2E-24	1.2	Bok	3E-25	1.4
Rbl1	3E-16	1.2	H1fx	7E-36	1.4
P2rx5	1E-12	1.2	Slc43a3	3E-26	1.4
Hjurp	5E-20	1.2	Pxylp1	1E-26	1.4
Haus6	2E-14	1.2	H2afz	6E-39	1.4
Ezh2	2E-23	1.2	Cdt1	4E-18	1.4
Gprc5a	2E-32	1.2	Sdk1	1E-25	1.4
Rasl12	3E-19	1.2	Pola2	2E-15	1.3
Zcwpw1	1E-13	1.2	Rcan2	4E-73	1.3
Tinagl1	6E-19	1.2	Rpa2	1E-23	1.3
Pdzk1ip1	6E-15	1.2	Suv39h1	3E-21	1.3
Sntg2	3E-38	1.2	Slc9a3r1	4E-39	1.3
Aldh1a1	2E-77	1.1	Zfp367	6E-31	1.3
Rfc4	2E-20	1.1	Rad51b	2E-74	1.3
Bok	1E-11	1.1	Ccdc34	5E-66	1.3
Creb5	2E-106	1.1	Ano5	9E-22	1.3
Tuba1b	5E-47	1.1	Cenpw	8E-39	1.3
Dck	5E-13	1.1	Zgrf1	3E-22	1.3
Cdt1	6E-12	1.1	Arhgap18	9E-64	1.3
Haus4	5E-19	1.1	Tuba1b	9E-59	1.3
Tex30	1E-15	1.1	Bend5	6E-26	1.3
Ndc1	3E-17	1.1	Ntrk3	5E-48	1.3
Morrbid	3E-25	1.1	Tex30	7E-34	1.3
Tubb6	4E-16	1.1	Smc4	3E-98	1.3
Cdc25b	1E-11	1.1	Rbl1	4E-22	1.3
Tmtc4	2E-12	1.1	Mcm6	7E-28	1.3
Sass6	2E-17	1.1	Sass6	1E-19	1.3
Itga7	6E-11	1.1	Mrnip	6E-20	1.3
Lbr	8E-18	1.1	Tubb4b	8E-26	1.3
Ccdc34	2E-38	1.1	Haus4	2E-31	1.3
H2afz	1E-09	1.1	Eln	2E-32	1.3
Slc26a7	1E-10	1.1	Rfc4	2E-33	1.3
Cdca7	4E-11	1.1	Cntrob	4E-20	1.2
Fkbp5	3E-12	1.1	Prim2	3E-28	1.2
Reep4	2E-14	1.1	Dpt	8E-36	1.2
Hirip3	1E-14	1.1	Vrk1	6E-32	1.2
Lctl	1E-16	1.1	Rerg	2E-25	1.2
Tubg1	1E-18	1.0	Pold1	2E-20	1.2
Trim59	2E-21	1.0	Cdkn2c	1E-54	1.2
Slc43a3	7E-14	1.0	Psmc3ip	8E-24	1.2
Sox11	1E-04	1.0	Polh	7E-29	1.2
Jpt1	6E-26	1.0	Arl6ip1	2E-26	1.2
Donson	2E-07	1.0	Hist1h1d	5E-18	1.2
Pstpip2	1E-15	1.0	Hirip3	5E-22	1.2

Undefined					
Control			$Notch2^{tm1.1Ecan}$		
Gene	*p*	FC	Gene	*p*	FC
Ss18l2	2E-20	0.8	9630028H03Rik	3E-01	0.7
Nos1ap	6E-11	0.8	Fxyd6	8E-07	0.7
Cyb5r2	9E-11	0.8	Tspan2	5E-07	0.7
Tox	5E-66	0.8	Map7d3	9E-03	0.7
Kdm1b	1E-05	0.8	Eps8l2	7E-14	0.7
Comp	2E-71	0.8	Vill	1E-01	0.7
Pde7b	1E-29	0.8	Flrt1	4E-02	0.7
Pla2g12a	5E-10	0.8	Kcnc3	8E-04	0.7
Samd4	3E-88	0.8	Cp	2E-14	0.7
Gm26911	1E-05	0.8	Slc26a2	1E-09	0.7
Pam	4E-153	0.8	Apmap	7E-02	0.7
Gm13919	2E-16	0.8	Sox9	8E-33	0.7
B230217O12Rik	6E-02	0.8	Maged2	2E-14	0.7
Slc7a2	4E-37	0.8	Zim1	4E-06	0.7
Kcnc3	5E-06	0.8	Slc25a33	2E-01	0.7
Rerg	1E-08	0.8	Abtb2	7E-15	0.7
Igf2	3E-51	0.8	Slc2a1	1E-03	0.7
Emb	2E-62	0.8	Rbm48	2E-01	0.7
Slc22a4	2E-05	0.8	Ncam1	7E-03	0.7
Armh4	2E-12	0.8	Id1	1E-40	0.7
Reck	3E-21	0.8	Osbpl6	9E-04	0.7
Map7d3	2E-19	0.8	Pim3	6E-06	0.7
Ern1	2E-08	0.8	Tox	9E-32	0.7
Glt28d2	7E-07	0.8	Galnt3	4E-04	0.7
Atp10a	3E-23	0.8	Camk1d	1E-01	0.7
Slc17a9	1E-05	0.8	AY036118	9E-01	0.7
Tex14	3E-32	0.8	Emb	2E-22	0.7
Gpank1	6E-01	0.8	Paqr3	2E-07	0.7
Ifitm10	2E-11	0.7	Tinagl1	8E-01	0.7
Adhfe1	2E-19	0.7	Slc7a2	1E-12	0.7
Erg	3E-23	0.7	Gpc6	3E-38	0.7
Gpc6	1E-78	0.7	Fgfrl1	6E-13	0.7
Psph	5E-13	0.7	Vldlr	3E-07	0.7
Smoc2	8E-07	0.7	Ecm2	3E-01	0.7
Rnf144b	6E-08	0.7	Btg1	5E-08	0.7
Pcgf5	3E-07	0.7	Mtcl1	7E-02	0.7
Chac1	7E-07	0.7	Ctdspl	3E-29	0.7
Pde3a	4E-29	0.7	Fbxo2	4E-03	0.7
Gm973	1E-10	0.7	Stk32b	4E-15	0.7
Slc26a2	3E-14	0.7	Dusp14	1E-06	0.7
Slc12a2	7E-19	0.7	Ltv1	1E-03	0.7
Gm44686	2E-02	0.7	Adi1	2E-01	0.7
Nrtn	9E-05	0.7	Lrrc17	6E-03	0.7
Stxbp6	4E-30	0.7	Sap30	2E-07	0.7
C1qtnf4	5E-09	0.7	Pcdh9	3E-25	0.7
Klc3	4E-09	0.7	Stk38l	4E-05	0.7
Sft2d2	5E-05	0.7	Cdkl5	3E-02	0.7
Clcn3	5E-14	0.7	Phtf2	6E-01	0.7
BC006965	3E-03	0.7	Usp29	3E-08	0.7
Chdh	3E-07	0.7	Ccdc47	6E-08	0.7
Sh3rf2	1E-13	0.7	Rrp9	8E-02	0.6
Samd12	7E-12	0.7	Lmcd1	6E-04	0.6
Tprg	1E-06	0.7	Sema6a	3E-04	0.6
Adgrg6	7E-38	0.7	1190007I07Rik	3E-03	0.6
Pcdh9	2E-49	0.7	Lgals3	2E-21	0.6
Sema3e	2E-10	0.7	Gfpt2	5E-04	0.6
Tspyl4	4E-18	0.7	Phyh	3E-07	0.6
Tiparp	2E-45	0.7	4930523C07Rik	3E-09	0.6
Sntg2	3E-03	0.7	Gm37240	2E-02	0.6
Tgfa	1E-05	0.7	Reck	3E-02	0.6
Sox5	3E-71	0.7	Cobl	1E-09	0.6
Lrrc17	2E-06	0.7	Gpt2	1E-03	0.6
Sox9	5E-52	0.7	Tspyl4	5E-04	0.6
Bend6	8E-06	0.7	Ppip5k1	8E-01	0.6

Articular/Synovial Fibroblast					
Control			$Notch2^{tm1.1Ecan}$		
Gene	*p*	FC	Gene	*p*	FC
St3gal1	1E-48	1.5	Lrrc32	5E-26	1.3
Sash1	9E-163	1.5	Anxa3	5E-43	1.3
Plcxd2	1E-17	1.5	Filip1l	5E-41	1.3
Radil	7E-26	1.5	Epb41l3	2E-46	1.3
Serpinb1a	3E-86	1.5	Axl	2E-43	1.3
Rasl12	4E-22	1.5	Cavin2	1E-13	1.3
Fbln2	1E-101	1.5	Lipa	9E-47	1.3
Zyx	2E-94	1.5	Gstm2	1E-46	1.3
Zfp385a	2E-48	1.4	Ifih1	4E-14	1.3
Tbx18	1E-66	1.4	Metrnl	6E-39	1.3
Gbp7	3E-18	1.4	Tmod2	2E-15	1.3
Tnfaip2	4E-77	1.4	Pla2r1	2E-20	1.3
Gab2	2E-45	1.4	Col4a1	2E-47	1.3
Slc25a21	3E-07	1.4	Wnt16	2E-09	1.3
Ldb2	4E-65	1.4	Il4ra	4E-12	1.3
Nbl1	1E-112	1.4	Vat1	2E-93	1.3
Pde8a	1E-77	1.4	Zfp503	4E-59	1.3
Ackr3	4E-58	1.4	Pde8b	1E-10	1.3
Sema3b	2E-51	1.4	Psmb8	5E-15	1.3
Col3a1	1E-220	1.4	Disp1	1E-31	1.3
Ptk2b	1E-27	1.4	Acvrl1	6E-15	1.3
B4galt5	4E-35	1.4	Sash1	5E-94	1.3
Selenop	2E-80	1.4	Ildr2	2E-14	1.3
Gm12153	6E-12	1.4	Fbln2	6E-77	1.3
Rasa3	5E-41	1.4	Hsd3b7	2E-13	1.3
Ednra	8E-27	1.4	Pi15	1E-33	1.3
Ifit1	2E-24	1.4	Limk1	8E-07	1.3
Lgals9	2E-47	1.4	Plin4	1E-14	1.3
Serpinb6a	2E-190	1.4	Zdhhc14	1E-14	1.3
Gm10638	1E-11	1.4	Rasa3	2E-31	1.3
Ntn4	3E-33	1.4	Mustn1	9E-22	1.3
Mmp11	5E-17	1.4	Serpinb1a	2E-39	1.3
Slco2a1	2E-12	1.4	Cited2	3E-72	1.3
Wnt16	3E-21	1.4	Pde8a	5E-46	1.3
Arhgap18	2E-49	1.4	Svep1	4E-108	1.3
Sh3pxd2b	1E-50	1.4	Sfrp2	2E-76	1.2
Psmb8	1E-18	1.4	Ugdh	5E-31	1.2
Sgip1	4E-22	1.4	Cd80	2E-08	1.2
Metrnl	1E-58	1.4	Arhgap18	6E-26	1.2
Ptpre	3E-18	1.4	Radil	9E-13	1.2
Idi1	4E-73	1.3	Lrmda	2E-18	1.2
Aif1l	3E-12	1.3	Sh2d4a	1E-14	1.2
Acss2	1E-29	1.3	Ifit1	9E-13	1.2
Pla2r1	1E-29	1.3	Fbln5	2E-86	1.2
Nav1	6E-58	1.3	Nhsl1	2E-07	1.2
Nlgn1	9E-08	1.3	Ptk2b	8E-13	1.2
Epas1	5E-27	1.3	Stom	3E-14	1.2
Cdh13	8E-54	1.3	Mturn	6E-12	1.2
A4galt	5E-25	1.3	Hmcn1	2E-22	1.2
Srgap1	2E-68	1.3	Lpin3	5E-15	1.2
Zdhhc14	2E-34	1.3	Acss2	7E-19	1.2
AA467197	1E-17	1.3	Jag1	2E-25	1.2
Acvrl1	1E-28	1.3	Lox	2E-109	1.2
Filip1l	3E-46	1.3	Nrn1	1E-05	1.2
Vat1	5E-144	1.3	Capg	3E-71	1.2
Ugcg	5E-31	1.3	Cyp4v3	4E-16	1.2
Gm16364.1	3E-20	1.3	Nav1	1E-32	1.2
Csmd3	3E-12	1.3	Akap12	1E-49	1.2
Bdnf	3E-24	1.3	Insig1	3E-36	1.2
Ephb6	1E-34	1.3	Fap	3E-27	1.2
Fdps	6E-86	1.3	Gca	3E-07	1.2
Tnnt3	7E-39	1.3	Serpinb6a	5E-107	1.2
Smurf2	4E-57	1.3	Jph1	3E-09	1.2
Il13ra1	4E-18	1.3	Anxa1	2E-103	1.2

Limb Mesenchyme

Control Gene	p	FC	*Notch2 tm1.1Ecan* Gene	p	FC
Dnm3os	2E-68	0.8	Cnrip1	3E-34	0.8
Peak1	1E-74	0.8	Rasa4	6E-14	0.8
Socs2	4E-14	0.8	Eda	3E-18	0.8
Cebpd	1E-27	0.8	Ntng1	1E-17	0.8
Arrdc4	3E-19	0.8	Il6st	1E-103	0.8
Col5a1	3E-199	0.8	Mapk13	7E-15	0.8
Calhm5	7E-22	0.8	Adamts3	2E-09	0.8
St3gal1	6E-33	0.8	Lifr	3E-16	0.8
Cnrip1	7E-29	0.8	Prr5	1E-22	0.8
Il3ra	2E-30	0.8	Ccdc102a	8E-49	0.8
Ldb2	1E-61	0.8	Ereg	1E-12	0.8
Lrp4	5E-12	0.8	Gm41724	3E-42	0.8
Cdc42ep2	1E-22	0.8	Man1c1	4E-49	0.8
Lgals1	0E+00	0.8	Gm28875	6E-16	0.8
Ephb3	2E-14	0.8	Lama2	2E-37	0.8
A830012C17Rik	5E-11	0.8	Kif1a	8E-22	0.8
Tmeff2	5E-14	0.8	Cyp1b1	9E-38	0.8
Mdk	2E-26	0.8	Gm20559	6E-15	0.8
Fras1	9E-24	0.8	Ephb2	9E-25	0.8
Fbn2	2E-85	0.8	Dusp5	6E-09	0.8
Lrrc75a	1E-17	0.8	Ank3	4E-58	0.8
Dtx3l	1E-06	0.8	Fstl1	3E-169	0.8
Il6st	4E-124	0.8	Kcne4	2E-07	0.8
Adgrl2	4E-65	0.8	Morrbid	4E-31	0.7
Frmd4a	1E-18	0.8	Snx7	1E-78	0.7
Mkx	4E-31	0.8	Ptgfrn	2E-31	0.7
Adamts1	2E-44	0.8	Ttyh3	1E-36	0.7
Ptgfrn	4E-27	0.8	Ebf3	3E-30	0.7
Rasa4	1E-15	0.8	Ndnf	8E-43	0.7
Xaf1	3E-24	0.8	Scx	3E-35	0.7
Man1c1	8E-50	0.8	Bmp4	6E-54	0.7
Creb3l1	4E-83	0.8	Tgfb1i1	1E-57	0.7
P4ha3	2E-30	0.8	Galnt13	2E-17	0.7
Adgrg2	1E-28	0.8	Il34	5E-15	0.7
Mcc	7E-43	0.8	Ece1	5E-34	0.7
Igfbp4	1E-87	0.8	Ebf2	3E-21	0.7
Rab3d	5E-09	0.8	Gstt1	6E-21	0.7
Rassf5	1E-11	0.8	Astn2	1E-08	0.7
Nexn	4E-26	0.8	Ptgr1	5E-19	0.7
Akr1b8	8E-12	0.7	Nxn	6E-46	0.7
Tbc1d2b	2E-22	0.7	Stk17b	7E-28	0.7
Cdc42ep1	2E-14	0.7	Lbh	2E-23	0.7
Cbr3	8E-28	0.7	Wnt5a	2E-27	0.7
Bst2	9E-59	0.7	Galnt17	2E-23	0.7
Syne3	2E-23	0.7	Pced1b	2E-14	0.7
Srpx	5E-46	0.7	Rflnb	9E-17	0.7
Col4a1	2E-69	0.7	Adamts1	2E-34	0.7
Htra3	3E-29	0.7	Lrp4	1E-15	0.7
Dusp5	3E-10	0.7	Mmp14	1E-120	0.7
Tlcd2	7E-13	0.7	Meis2	1E-41	0.7
Ndrg4	2E-14	0.7	Stard9	3E-18	0.7
Capn1	4E-21	0.7	Ndrg4	3E-19	0.7
4931406P16Rik	6E-40	0.7	Ephb3	3E-19	0.7
Kif26b	4E-06	0.7	Itga1	9E-37	0.7
Dpysl3	4E-25	0.7	Slc44a2	1E-31	0.7
Matn2	2E-27	0.7	Cpxm1	4E-36	0.7
Ifi204	2E-14	0.7	Tln2	3E-54	0.7
Arxes2	5E-19	0.7	Akr1b8	8E-23	0.7
Dact1	8E-08	0.7	Arxes1	6E-12	0.7
Plpp1	2E-151	0.7	Bnc2	1E-51	0.7
Thbs2	2E-85	0.7	B3galnt1	4E-16	0.7
Ednra	3E-11	0.7	Lyn	1E-13	0.7
Gm17501	8E-23	0.7	Lgi2	2E-09	0.7
Il13ra1	4E-09	0.7	Arsi	5E-13	0.7

Chondrogenic

Control Gene	p	FC	*Notch2 tm1.1Ecan* Gene	p	FC
Mboat2	1E-50	1.0	Shtn1	2E-15	1.1
Acot1	5E-21	1.0	Btg1	4E-137	1.1
Lrrc17	4E-42	1.0	Nos1ap	2E-22	1.1
Has2	7E-40	1.0	Gm15663	3E-20	1.1
Zfp612	3E-19	1.0	Trp53cor1	3E-48	1.1
Cnnm4	7E-18	1.0	Adcy9	1E-52	1.1
Psph	2E-81	1.0	Ascc2	2E-56	1.1
Smad9	4E-23	1.0	Gm12811	8E-17	1.1
Tafa5	1E-61	1.0	Fbxo7	3E-61	1.1
Nsun4	3E-41	1.0	Rdm1	1E-52	1.1
Il17d	4E-40	1.0	Klf5	6E-14	1.1
Tgfbi	4E-13	1.0	C030037D09Rik	8E-15	1.1
D630045J12Rik	5E-75	1.0	Orai1	2E-47	1.1
Mtus1	3E-32	1.0	Fam160a1	2E-14	1.1
Orai1	2E-57	1.0	Cobl	8E-109	1.1
Deptor	3E-29	1.0	Foxd1	2E-32	1.0
2610035D17Rik	4E-45	1.0	Pla2g12a	1E-54	1.0
P3h2	7E-69	1.0	Pth1r	3E-21	1.0
Angptl6	1E-55	1.0	Cdv3	2E-165	1.0
Trib3	4E-87	1.0	Arl4a	4E-68	1.0
Atf3	5E-86	1.0	Cpe	5E-240	1.0
B930036N10Rik	1E-58	1.0	Pdrg1	2E-144	1.0
Tiam1	1E-14	1.0	Trib3	5E-88	1.0
Sox5	3E-229	1.0	Rbp4	3E-89	1.0
Ciapin1	5E-45	1.0	Atf3	4E-73	1.0
Prkg2	9E-22	1.0	Tiam1	7E-19	1.0
Col4a4	5E-13	1.0	Col24a1	4E-20	1.0
Nrp2	3E-163	1.0	Map7d3	2E-39	1.0
Comp	2E-140	1.0	Angptl6	1E-47	1.0
Cpne8	1E-205	1.0	Smad9	5E-20	1.0
Capn6	3E-76	1.0	Ptchd4	2E-18	1.0
Cacnb2	1E-88	1.0	Cpne8	6E-196	1.0
Dcaf12l1	3E-35	1.0	Sox5	4E-188	1.0
Slc12a2	3E-81	1.0	Fgfrl1	4E-92	1.0
Reps2	7E-05	1.0	Prkg2	2E-15	1.0
Dock8	9E-60	1.0	S100a1	2E-132	1.0
Gm15663	8E-11	1.0	Bmp5	6E-77	1.0
Satb1	9E-21	1.0	Epb41l4b	4E-15	1.0
Slc7a11	3E-17	1.0	Pcsk6	5E-12	1.0
Letm2	2E-33	0.9	Capn6	6E-78	1.0
Gm49959	2E-22	0.9	Zfp612	2E-16	1.0
Tubb2a	3E-72	0.9	Kcnc3	1E-23	1.0
Oxr1	1E-48	0.9	BC006965	1E-20	1.0
Masp1	1E-13	0.9	Miga1	5E-14	1.0
Kctd16	5E-22	0.9	Cnnm4	2E-15	1.0
Runx2os1	5E-11	0.9	Dach2	6E-08	1.0
Gpt2	7E-32	0.9	Mboat2	3E-36	1.0
Tspan6	2E-115	0.9	Samd4	3E-159	1.0
Stbd1	6E-34	0.9	Rps6ka2	4E-08	1.0
Pla2g12a	1E-63	0.9	Slc16a2	2E-77	1.0
Shisa2	1E-18	0.9	Sgip1	1E-16	1.0
4930444A19Rik	2E-09	0.9	Igf2	5E-163	1.0
Fgd6	5E-14	0.9	Kank1	8E-74	1.0
Fbxo7	7E-37	0.9	Zbtb7c	2E-39	1.0
Trp53cor1	2E-41	0.9	Cdsn	1E-45	1.0
Arid5a	2E-19	0.9	Masp1	3E-14	0.9
Rdm1	1E-45	0.9	Hoxa3	4E-24	0.9
4930556M19Rik	6E-25	0.9	Clcn3	4E-49	0.9
Cpe	2E-219	0.9	Deptor	7E-20	0.9
Serinc5	4E-32	0.9	Ltv1	9E-48	0.9
Vill	9E-18	0.9	4930581F22Rik	9E-20	0.9
Kdm1b	6E-21	0.9	Slc7a11	2E-18	0.9
Slc16a2	7E-73	0.9	Slc8a3	5E-16	0.9
Gm4258	3E-36	0.9	Comp	6E-134	0.9

Fibroblast

Control Gene	p	FC	*Notch2 tm1.1Ecan* Gene	p	FC
Haus3	2E-16	1.0	Gins2	3E-24	1.2
Vrk1	2E-22	1.0	9630013D21Rik	6E-18	1.2
Tubb4b	2E-14	1.0	Osr2	4E-46	1.2
Prim2	2E-14	1.0	Wdr76	2E-16	1.2
Arhgap18	1E-28	1.0	Plekha7	2E-18	1.2
Mcm7	6E-13	1.0	Fkbp5	4E-08	1.2
Dtl	6E-10	1.0	L3mbtl2	2E-14	1.2
Dnmt1	7E-18	1.0	Pmf1	2E-30	1.2
2610307P16Rik	3E-11	1.0	Gm17092	4E-18	1.2
Osr2	2E-43	1.0	Zdhhc2	7E-17	1.2
Pold1	1E-09	1.0	Tmpo	5E-53	1.1
Nav2	5E-29	1.0	Selenoh	4E-44	1.1
Gm17092	3E-13	1.0	Sipa1	1E-15	1.1
Cenpw	2E-12	1.0	Dnajc9	1E-37	1.1
Il16	2E-35	1.0	Poc1a	3E-17	1.1
Bdnf	1E-13	1.0	Pcnt	1E-30	1.1
Skp2	2E-10	1.0	Morrbid	8E-43	1.1
Lox	2E-117	1.0	Zcwpw1	2E-17	1.1
Thbd	1E-46	1.0	Pcna	9E-26	1.1
Tnfrsf11b	5E-22	1.0	Lin54	4E-34	1.1
Suv39h1	9E-12	1.0	D030056L22Rik	2E-09	1.1
Bmp4	4E-50	1.0	Dsn1	6E-12	1.1
Lin54	2E-13	1.0	Jpt1	8E-43	1.1
Tmpo	8E-43	0.9	Nkain4	2E-29	1.1
Arhgap6	2E-44	0.9	Creb5	9E-100	1.1
Epas1	1E-21	0.9	Ctc1	1E-21	1.1
Chek1	2E-15	0.9	Cep89	9E-17	1.1
Rad51b	3E-31	0.9	Gemin6	1E-20	1.1
Casp7	1E-10	0.9	Stac	4E-16	1.1
Dhrs9	3E-38	0.9	Nav2	1E-52	1.1
Selenoh	9E-14	0.9	Skp2	1E-17	1.1
Gm15261	8E-17	0.9	Sntg2	9E-19	1.1
Nsd2	3E-21	0.9	Gm15261	4E-19	1.1
Dkk3	2E-51	0.9	Ska2	5E-25	1.1
Nxt1	3E-19	0.9	Ube2s	7E-43	1.1
Tmeff2	5E-05	0.9	Gm26917	3E-01	1.1
Pmf1	3E-16	0.9	Ccn5	3E-130	1.1
Mtss1	3E-15	0.9	Cchcr1	5E-16	1.1
Wee1	2E-19	0.9	Dnph1	5E-19	1.1
Enkd1	5E-11	0.9	Rccd1	6E-13	1.0
Timp3	6E-81	0.9	Dnmt1	2E-25	1.0
Wdr76	5E-12	0.9	Ppih	2E-22	1.0
Sdk1	2E-04	0.9	Hat1	3E-24	1.0
Ndufa4l2	3E-32	0.9	Aldh1a1	3E-39	1.0
Rerg	1E-12	0.9	Sned1	2E-26	1.0
a	3E-16	0.9	Haus6	7E-20	1.0
Smc4	1E-54	0.9	Nde1	8E-24	1.0
Cited2	1E-58	0.9	Sh2d4a	2E-19	1.0
Cdon	5E-80	0.9	Lbr	2E-25	1.0
Cables1	8E-13	0.9	Tmtc4	4E-19	1.0
Hat1	1E-13	0.9	Casp7	1E-17	1.0
Zranb3	1E-11	0.9	Ndc1	2E-25	1.0
Barx1	5E-53	0.9	Pkia	1E-19	1.0
Ecm2	6E-26	0.9	Cep295	3E-20	1.0
Slc16a3	4E-09	0.9	Cenpt	6E-22	1.0
Slc22a23	7E-11	0.9	Hpgd	4E-07	1.0
Fgf18	1E-08	0.9	Sema3e	1E-21	1.0
Psmc3ip	4E-11	0.9	Psrc1	5E-12	1.0
Lama5	1E-11	0.9	Myom1	2E-21	1.0
Pcna	5E-08	0.9	4930503L19Rik	1E-20	1.0
Lmf2	1E-17	0.9	Nup133	2E-18	1.0
Dnajc9	5E-16	0.9	Mcm7	4E-18	1.0
Mcm2	7E-12	0.9	Jpt2	3E-21	1.0
Dnph1	2E-12	0.9	Tubb5	1E-61	1.0

Undefined

Control Gene	p	FC	*Notch2 tm1.1Ecan* Gene	p	FC
Aff3	2E-67	0.7	4930517O19Rik	4E-01	0.6
Peg3	3E-83	0.7	Slc16a4	1E-01	0.6
Orai1	2E-09	0.7	Slc22a15	6E-01	0.6
Sort1	4E-11	0.7	Cystm1	2E-01	0.6
Efna1	2E-01	0.7	Smpdl3b	5E-01	0.6
Aig1	1E-36	0.7	N4bp2l1	6E-02	0.6
Shtn1	1E-08	0.7	Rbp4	1E-17	0.6
Prkaa2	1E-08	0.7	Nrtn	2E-03	0.6
Gli2	4E-08	0.7	Pcgf5	1E-03	0.6
Zfp612	1E-07	0.7	Serinc5	5E-04	0.6
Gm14164	7E-01	0.7	Arl4a	2E-03	0.6
Cobl	1E-30	0.7	Ggnbp1	2E-01	0.6
Cmbl	8E-02	0.7	Phf10	3E-03	0.6
Mttp	3E-04	0.7	Tafa5	2E-02	0.6
Il1rapl1	6E-28	0.7	Ddit3	2E-05	0.6
Prdm6	1E-05	0.7	Ecrg4	2E-51	0.6
Kcnma1	6E-08	0.7	Herpud1	1E-07	0.6
Atf3	4E-20	0.7	Sdr39u1	2E-01	0.6
Ddb2	7E-04	0.7	Asns	1E-06	0.6
Fhl1	1E-27	0.7	Usp6nl	2E-04	0.6
Camk4	6E-24	0.7	Cspg4	2E-01	0.6
Arhgef28	8E-24	0.7	Wfs1	2E-02	0.6
Fchsd2	5E-39	0.7	Sephs2	2E-04	0.6
Ccdc115	1E-04	0.7	Tspan6	3E-08	0.6
Epb41	3E-07	0.7	Trib3	5E-03	0.6
Id1	1E-55	0.7	Ptchd4	7E-04	0.6
Nuak2	4E-05	0.7	2700046G09Rik	5E-01	0.6
Dcun1d2	3E-01	0.7	B930036N10Rik	3E-03	0.6
Hotairm1	2E-08	0.7	Iqsec1	2E-02	0.6
Has2	2E-09	0.6	Rbm15	6E-04	0.6
C030006K11Rik	7E-02	0.6	Slc20a1	2E-03	0.6
Il16	1E-12	0.6	Thyn1	9E-02	0.6
Gm28054	7E-06	0.6	Tenm4	3E-11	0.6
Osgin2	7E-17	0.6	Slc1a5	1E-02	0.6
Carmil1	6E-25	0.6	Plagl1	4E-12	0.6
Faah	4E-05	0.6	Nos1ap	1E-02	0.6
Abcc4	8E-08	0.6	Lars2	2E-01	0.6
Angptl6	1E-07	0.6	Kcnma1	8E-04	0.6
Trib3	5E-11	0.6	Clcn3	5E-05	0.6
Npr2	3E-12	0.6	Cdpf1	3E-01	0.6
Ciapin1	6E-09	0.6	Lyrm4	3E-01	0.6
Pdzrn4	2E-31	0.6	Slc7a11	2E-01	0.6
Mylk	3E-04	0.6	Plcd1	1E-02	0.6
Sgip1	4E-04	0.6	Tead4	3E-01	0.6
Gm16133	9E-05	0.6	Arpc5l	4E-05	0.6
4930517O19Rik	1E-06	0.6	Gm42418	5E-16	0.6
Paqr3	3E-11	0.6	Tiparp	4E-08	0.6
Lin7a	3E-05	0.6	Rfesd	8E-01	0.6
Slc7a11	7E-04	0.6	Lonrf1	1E+00	0.6
C230038L03Rik	2E-06	0.6	Mtmr12	8E-01	0.6
Gm17056	8E-02	0.6	Fbxl18	5E-02	0.6
Phtf2	6E-10	0.6	Dcaf11	3E-01	0.6
Hbegf	3E-17	0.6	Hmox1	9E-01	0.6
Gm49969	4E-02	0.6	Sft2d2	7E-01	0.6
Rpgrip1	1E-01	0.6	Glis1	6E-04	0.6
Arpc5l	2E-18	0.6	Tnfrsf11b	2E-06	0.6
Tead4	3E-04	0.6	Samd4	8E-23	0.6
Pex1	9E-01	0.6	Orai1	2E-04	0.6
Nqo1	3E-06	0.6	Wdr46	6E-01	0.6
Fbxo2	1E-07	0.6	Samd12	6E-03	0.6
Mdfi	6E-11	0.6	Igfbp5	3E-09	0.6
Tuba1c	2E-11	0.6	Asrgl1	2E-02	0.6
Fgfrl1	2E-15	0.6	Rsph9	1E-01	0.6
4930556M19Rik	2E-05	0.6	Prss36	6E-01	0.6

Articular/Synovial Fibroblast

Control Gene	p	FC	*Notch2 tm1.1Ecan* Gene	p	FC
Itpr3	2E-45	1.3	Sdk1	8E-17	1.2
Cited2	4E-109	1.3	Gm10125	1E-13	1.2
Efs	1E-36	1.3	Pygl	3E-18	1.2
Dhcr24	1E-40	1.3	Ifi204	9E-06	1.2
Ctsk	2E-80	1.3	Lasp1	3E-35	1.2
Ppp1r14b	2E-169	1.3	Mill2	1E-23	1.2
Fgf14	1E-02	1.3	Ampd3	9E-19	1.2
Capg	7E-106	1.3	Dhcr24	1E-19	1.2
Stat2	2E-34	1.3	Srgap1	2E-39	1.2
Pygl	9E-26	1.3	Hsd17b7	4E-14	1.2
Ebf2	2E-55	1.3	Itpr3	8E-20	1.1
Bace2	1E-24	1.3	Cmtm8	2E-07	1.1
Ptrh1	2E-28	1.3	Arvcf	7E-08	1.1
Nectin1	3E-15	1.3	Steap3	3E-63	1.1
Prrg3	4E-22	1.3	Dock11	6E-16	1.1
Olfml1	6E-29	1.3	Pdzd2	2E-24	1.1
1500009L16Rik	1E-33	1.3	Zfp385a	8E-18	1.1
Hsd3b7	1E-27	1.3	Capn5	4E-13	1.1
Zeb2os	4E-14	1.2	Fbxo32	3E-08	1.1
Fbln5	1E-122	1.2	Irak3	8E-20	1.1
Cd80	9E-07	1.2	Il13ra1	1E-10	1.1
Rftn1	2E-16	1.2	Atp9a	4E-17	1.1
Flnc	2E-30	1.2	Parp9	8E-17	1.1
Arhgap42	8E-79	1.2	Cemip	2E-03	1.1
Mturn	8E-15	1.2	Bend5	1E-10	1.1
Epb41l3	2E-65	1.2	Lefty1	3E-16	1.1
Gm20559	1E-26	1.2	Isg15	4E-18	1.1
Scara3	4E-53	1.2	Sqle	3E-31	1.1
Sfrp2	2E-91	1.2	Lama4	5E-63	1.1
Hsd17b7	1E-26	1.2	Galnt17	8E-14	1.1
Stom	1E-23	1.2	Ctsk	4E-45	1.1
Celf2	2E-115	1.2	Dtx3l	2E-09	1.1
Lox	6E-134	1.2	Galm	2E-23	1.1
Col18a1	4E-47	1.2	Fads2	5E-16	1.1
Lss	4E-17	1.2	Insyn1	1E-10	1.1
Lama4	2E-84	1.2	Ptgs1	2E-10	1.1
Nnmt	3E-76	1.2	Anxa8	2E-17	1.1
Anxa1	6E-160	1.2	Acat2	2E-13	1.1
Acat2	6E-24	1.2	Tnfaip2	4E-33	1.1
Mvk	1E-43	1.2	Coro2b	6E-10	1.1
Lasp1	5E-45	1.2	Nid1	4E-31	1.1
Disp1	1E-54	1.2	Cnrip1	2E-21	1.1
Maoa	6E-47	1.2	Stat2	4E-14	1.1
Dync1i1	1E-10	1.2	Svil	1E-52	1.1
Insig1	6E-59	1.2	Arhgap24	2E-49	1.1
Slc22a18	5E-14	1.2	Tm7sf2	4E-16	1.1
Ifi211	2E-14	1.2	Xaf1	6E-19	1.1
Cnrip1	7E-24	1.2	Pltp	7E-45	1.1
Pde1a	1E-19	1.2	Hmgcs1	7E-43	1.1
Ssc5d	3E-51	1.2	Manba	2E-06	1.1
Fdft1	1E-57	1.2	Lrrc27	3E-07	1.1
Prag1	3E-11	1.2	Tbc1d2b	9E-16	1.1
Gstm2	4E-57	1.2	Ereg	6E-06	1.1
Rnase4	4E-61	1.2	Gm12216	2E-08	1.1
Lipa	1E-55	1.2	Epas1	5E-19	1.1
Slc16a9	3E-10	1.2	Arsi	4E-14	1.1
Cxcl12	1E-57	1.2	Plce1	3E-47	1.1
Kdsr	7E-34	1.2	Ddah2	1E-64	1.1
Il4ra	2E-11	1.2	Ndufa4l2	1E-33	1.1
Lsp1	5E-77	1.2	Galnt18	2E-21	1.1
Rab40b	5E-24	1.2	Vcan	1E-38	1.1
Cdkn2a	5E-36	1.2	Gabarapl1	1E-29	1.1
Ptgs1	2E-17	1.2	Lss	7E-13	1.1
Akap6	9E-23	1.2	Adam19	2E-21	1.1

**Limb Mesenchyme**					
**Control**			***Notch2***$^{tm1.1Ecan}$		
**Gene**	***p***	**FC**	**Gene**	***p***	**FC**
Ptk7	9E-35	0.7	Prr16	3E-38	0.7
Plce1	5E-79	0.7	Pmepa1	2E-85	0.7
Gas1	4E-127	0.7	Tm7sf2	1E-11	0.7
Slco2a1	6E-14	0.7	Gem	2E-22	0.7
Zfp185	1E-06	0.7	Igfbp4	3E-90	0.7
Scx	4E-27	0.7	Hsd17b7	4E-15	0.7
Wnt5a	9E-25	0.7	Pabpc4l	2E-10	0.7
Fmnl2	4E-53	0.7	Gas2	1E-74	0.7
Plk2	3E-43	0.7	Tmem159	6E-28	0.7
Adamts5	4E-112	0.7	Timp1	4E-61	0.7
Pcp4l1	3E-05	0.7	Isg15	1E-18	0.7
Ano3	2E-08	0.7	Bmper	8E-06	0.7
Ang	5E-11	0.7	Bmpr1b	2E-25	0.7
Grasp	9E-11	0.7	Slc38a4	9E-14	0.7
Fzd5	5E-16	0.7	Gask1b	2E-36	0.7
Ccdc102a	1E-39	0.7	Cd248	7E-98	0.7
Mmp14	7E-145	0.7	Wnt5b	9E-10	0.7
Cers4	4E-23	0.7	Gm9844	9E-14	0.7
Mgst3	1E-52	0.7	Creb3l1	9E-70	0.7
Clec14a	8E-13	0.7	Sh3bp5	2E-18	0.7
Prag1	6E-10	0.7	Glipr2	1E-29	0.7
Bmpr1b	2E-20	0.7	Ano3	2E-12	0.7
Lbh	2E-14	0.7	Apba1	1E-17	0.7
Frat2	2E-10	0.7	Adgra2	3E-22	0.7
Ephb2	3E-20	0.7	Msx1	2E-03	0.7
Gm20559	2E-12	0.7	Rhob	8E-49	0.7
Inka1	3E-13	0.7	Cdkn2b	6E-44	0.7
Lgals3bp	1E-35	0.7	Adamts2	1E-45	0.7
AC103362.1	8E-10	0.7	Fzd4	2E-16	0.7
Tmem173	7E-07	0.7	Fabp3	1E-31	0.7
Morrbid	3E-17	0.7	Synm	3E-14	0.7
Gask1b	3E-12	0.7	Pkdcc	2E-14	0.7
Cercam	2E-38	0.7	Tmsb10	2E-174	0.7
Kctd11	3E-09	0.7	Plce1	4E-65	0.6
F8a	1E-06	0.7	Rab29	2E-10	0.6
Gm41724	9E-21	0.7	Matn2	2E-35	0.6
Rab32	8E-15	0.7	Cox6b2	5E-12	0.6
Asxl3	2E-10	0.7	Slit3	2E-63	0.6
Arsi	4E-13	0.7	Mvd	6E-18	0.6
Cmtm3	4E-44	0.6	Ldb2	2E-41	0.6
Golm1	7E-14	0.6	Dnm3os	4E-55	0.6
Klf7	2E-38	0.6	Igf2bp1	3E-09	0.6
Mn1	5E-25	0.6	Irx3	3E-07	0.6
Zfp984	2E-12	0.6	Rab9	2E-49	0.6
Homer2	8E-08	0.6	Capn1	5E-19	0.6
Nid1	2E-43	0.6	Cnn2	7E-69	0.6
Pdgfra	2E-43	0.6	Cblb	2E-35	0.6
1500009L16Rik	1E-02	0.6	Wipi1	3E-51	0.6
Tmem159	2E-23	0.6	Dubr	8E-19	0.6
Slc6a6	2E-24	0.6	Mmp2	1E-58	0.6
Irak3	2E-16	0.6	Rbms1	1E-64	0.6
Gm13832	2E-11	0.6	Trnp1	8E-40	0.6
Pdgfd	4E-20	0.6	Adamts12	4E-41	0.6
Car13	2E-07	0.6	Adgrl2	3E-42	0.6
Emc2	3E-73	0.6	Sh3rf3	8E-47	0.6
Garnl3	2E-11	0.6	Csf1	5E-36	0.6
Rbms1	1E-95	0.6	Zdhhc14	2E-11	0.6
Nhsl1	4E-22	0.6	Col4a2	3E-47	0.6
Ano6	2E-60	0.6	Ptk7	2E-29	0.6
Mical2	1E-08	0.6	Ddah1	3E-24	0.6
Picalm	4E-46	0.6	Mmp11	6E-22	0.6
Pwwp3b	9E-09	0.6	Dhcr24	4E-16	0.6
Rab9	2E-55	0.6	Rab7b	1E-09	0.6
A4galt	3E-05	0.6	Garnl3	3E-08	0.6

**Chondrogenic**					
**Control**			***Notch2***$^{tm1.1Ecan}$		
**Gene**	***p***	**FC**	**Gene**	***p***	**FC**
9630028H03Rik	6E-17	0.9	Igfbp5	6E-74	0.9
Plagl1	2E-125	0.9	Lpcat1	2E-28	0.9
Tspyl4	7E-50	0.9	Spats2l	1E-40	0.9
Gm26827	3E-13	0.9	Glis1	3E-33	0.9
Btg1	8E-124	0.9	Spata1	3E-41	0.9
Ascc2	1E-45	0.9	Serinc5	5E-27	0.9
Snhg4	2E-52	0.9	D630045J12Rik	4E-54	0.9
Prkg1	7E-206	0.9	Tmem154	5E-15	0.9
Unc5b	1E-20	0.9	Id1	7E-121	0.9
Kank1	6E-60	0.9	C1qtnf4	4E-25	0.9
Cybrd1	3E-16	0.9	Tspan6	4E-112	0.9
Emb	4E-119	0.9	Gramd1b	2E-18	0.9
Isg20	4E-47	0.9	Vldlr	4E-55	0.9
BC006965	3E-21	0.9	Tll1	2E-06	0.9
Eps8	1E-73	0.9	Lrrc75b	3E-19	0.9
Clcn3	1E-59	0.9	Spint2	4E-21	0.9
Abca8b	2E-11	0.9	Flnb	1E-125	0.9
Ets1	2E-29	0.9	Ciapin1	3E-34	0.9
Ntrk2	2E-17	0.9	Plagl1	1E-113	0.9
Ak4	3E-22	0.9	Gm45025	4E-16	0.9
Ltv1	6E-44	0.9	Dipk2a	6E-59	0.9
Tpd52l1	1E-25	0.9	Nsun4	3E-29	0.9
Slc8a3	1E-09	0.9	Prkg1	8E-176	0.9
Mtus2	5E-20	0.9	Crlf3	2E-16	0.9
Runx2	4E-21	0.9	Fat3	2E-20	0.9
Hoxa3	6E-21	0.8	Tgfa	2E-18	0.9
Fchsd2	2E-114	0.8	Ldhb	6E-87	0.9
Mknk1	4E-26	0.8	Cacnb2	1E-55	0.9
Lsamp	1E-27	0.8	Tafa5	2E-50	0.9
Fign	2E-61	0.8	Epb41	3E-34	0.9
Rnf32	1E-10	0.8	Slc7a2	4E-60	0.9
Cdv3	2E-159	0.8	Slc7a1	2E-73	0.9
Stxbp6	1E-64	0.8	Plcg2	3E-08	0.8
Arl4a	4E-63	0.8	B230217O12Rik	4E-12	0.8
Tll1	2E-08	0.8	Fign	2E-48	0.8
Slc25a33	1E-27	0.8	Tspyl4	2E-44	0.8
Spata1	1E-34	0.8	Osgin2	6E-42	0.8
Sort1	4E-27	0.8	Klf15	4E-23	0.8
Stard13	6E-58	0.8	Shroom4	2E-24	0.8
Tuba1c	3E-41	0.8	Mknk1	2E-23	0.8
Ntng2	1E-07	0.8	Satb1	4E-16	0.8
Slc7a1	5E-90	0.8	Tom1l2	2E-74	0.8
Tchh	2E-13	0.8	Slc35d1	2E-17	0.8
Lrrc75b	3E-20	0.8	Pim3	2E-24	0.8
Slc25a38	2E-26	0.8	Airn	4E-152	0.8
Hist1h2ac	2E-11	0.8	Emb	2E-98	0.8
Flnb	3E-114	0.8	Acot1	7E-16	0.8
Phlda1	2E-43	0.8	Slc12a2	5E-58	0.8
Plxna2	7E-14	0.8	Lrrc17	2E-37	0.8
Phtf2	5E-21	0.8	Loxl3	1E-25	0.8
Adcy9	9E-38	0.8	Usp53	1E-13	0.8
S100a1	6E-107	0.8	Aldh1l2	6E-63	0.8
Zbtb46	4E-10	0.8	Stard13	1E-75	0.8
Sap30	1E-51	0.8	Cryzl2	7E-08	0.8
Crlf3	5E-16	0.8	Fchsd2	5E-93	0.8
Isyna1	6E-19	0.8	Ctnnal1	8E-28	0.8
Gadd45a	3E-48	0.8	Carmil1	2E-48	0.8
N4bp2l1	1E-17	0.8	Dync1i1	3E-21	0.8
Miga1	1E-11	0.8	Maged2	7E-89	0.8
Slc7a2	4E-59	0.8	4930556M19Rik	5E-13	0.8
Epha2	2E-18	0.8	Gm49959	3E-11	0.8
Plcb1	2E-119	0.8	Slc1a5	8E-27	0.8
Tmem56	2E-05	0.8	Mthfd1l	2E-32	0.8
Gm26911	1E-12	0.8	Kcnrg	4E-11	0.8

**Fibroblast**					
**Control**			***Notch2***$^{tm1.1Ecan}$		
**Gene**	***p***	**FC**	**Gene**	***p***	**FC**
Vsig10	9E-09	0.9	Twist2	2E-13	1.0
Polh	4E-07	0.9	Gprc5a	2E-14	1.0
Tbx18	2E-39	0.9	Tinf2	3E-14	1.0
Topbp1	2E-12	0.9	Anxa8	3E-27	1.0
Pim1	9E-20	0.9	Rbbp8	1E-28	1.0
Rad21	5E-16	0.9	Dhrs9	7E-39	1.0
Syce2	7E-12	0.9	Dut	4E-24	1.0
Cdkn2c	8E-30	0.9	Brip1os	1E-31	1.0
Prkcg	4E-17	0.9	Ttf2	5E-15	0.9
Eri1	1E-09	0.9	Cmc2	8E-24	0.9
Khdrbs3	5E-11	0.9	Chek1	8E-14	0.9
Anxa3	2E-60	0.9	Hmgb3	3E-29	0.9
Grb14	2E-37	0.8	Plcxd2	5E-15	0.9
Ephb6	3E-16	0.8	Nucks1	3E-54	0.9
S100a6	2E-100	0.8	Terf1	4E-22	0.9
Cav3	1E-10	0.8	Slc25a10	3E-15	0.9
Pola2	3E-09	0.8	Mcm4	1E-16	0.9
Sema3e	5E-18	0.8	Zranb3	3E-18	0.9
Ripk3	3E-07	0.8	Cks1b	8E-27	0.9
H1fx	5E-12	0.8	Rfc3	3E-19	0.9
Ppih	3E-12	0.8	Hmgn2	3E-33	0.9
Ptk2b	8E-10	0.8	Haus3	8E-19	0.9
C1galt1	2E-31	0.8	Usp1	2E-26	0.9
Clip4	3E-23	0.8	Haus1	4E-21	0.9
Rpa1	5E-16	0.8	Serpinb1a	6E-31	0.9
Nuak2	2E-15	0.8	Nsg1	9E-29	0.9
Hspa4l	2E-12	0.8	Rttn	3E-13	0.9
Gab3	2E-04	0.8	Ada	2E-11	0.9
Klhdc8a	2E-19	0.8	Npr3	6E-37	0.9
Rbbp8	1E-22	0.8	Rpa1	6E-27	0.9
Cep89	1E-09	0.8	Ckap5	1E-27	0.9
Ube2s	3E-19	0.8	Itga8	2E-04	0.9
Fzr1	1E-07	0.8	Hist1h4d	2E-13	0.9
Stk10	5E-10	0.8	C1ql3	2E-11	0.9
Pak4	4E-05	0.8	Raet1e	1E-13	0.9
Haus1	2E-09	0.8	Gm13483	4E-16	0.9
4933406I18Rik	1E-20	0.8	Eri1	3E-13	0.9
Idi1	9E-29	0.8	Smoc1	1E-31	0.9
Il1rapl1	2E-32	0.8	Cep192	2E-21	0.9
Styk1	2E-08	0.8	Arhgap6	2E-54	0.9
Ska2	3E-11	0.8	Tm4sf1	1E-55	0.9
Fxyd6	3E-29	0.8	Eri2	8E-16	0.9
Cep135	4E-10	0.8	Faap24	6E-08	0.9
Cacna1g	1E-06	0.8	Donson	1E-10	0.9
Rttn	2E-11	0.8	Stard8	2E-11	0.9
Dut	3E-13	0.8	Pold3	3E-14	0.9
Msh6	7E-08	0.8	Fgf18	3E-08	0.9
Gm15222	1E-05	0.8	Hmgn5	3E-25	0.9
Herc6	4E-11	0.8	Rdh11	2E-10	0.9
2900026A02Rik	2E-19	0.8	Dkk3	1E-54	0.9
Hmgn5	3E-21	0.8	Tmtc1	5E-43	0.9
2200002D01Rik	7E-15	0.8	Uevld	3E-10	0.9
Disp1	1E-30	0.8	Topbp1	2E-18	0.9
Arl6ip1	2E-04	0.8	Sox11	2E-06	0.9
Fhl1	1E-31	0.8	Cep70	2E-18	0.9
Gm12153	1E-06	0.8	Cyp39a1	6E-17	0.9
Nde1	2E-14	0.8	Lama5	8E-12	0.9
Rfwd3	2E-12	0.8	Rfc2	2E-26	0.9
Cenpj	2E-10	0.8	Epas1	2E-20	0.9
Pygl	2E-06	0.8	Tbx18	5E-35	0.9
Rin1	6E-09	0.8	Ephb6	2E-13	0.9
Dnaaf2	3E-09	0.8	Mtmr14	4E-11	0.8
Dsn1	4E-08	0.8	9530026P05Rik	1E-07	0.8
Hmgn2	2E-16	0.8	2610307P16Rik	2E-15	0.8

**Undefined**					
**Control**			***Notch2***$^{tm1.1Ecan}$		
**Gene**	***p***	**FC**	**Gene**	***p***	**FC**
4933400C23Rik	1E-08	0.6	Rnaset2a	1E+00	0.6
Srebf1	4E-08	0.6	Camk4	3E-09	0.6
Crybg3	6E-08	0.6	Slc39a1	4E-13	0.6
Lrrc51	4E-02	0.6	Aig1	7E-14	0.6
Acot1	2E-03	0.6	Per2	2E-01	0.6
Lrrc8c	4E-09	0.6	Atf3	8E-05	0.6
Cox6a2	4E-01	0.6	Etv5	3E-04	0.6
Tspan6	2E-24	0.6	Prkaa2	3E-02	0.6
Ltv1	3E-09	0.6	Peg3	1E-46	0.6
Prtg	1E-04	0.6	Lsm14b	4E-06	0.5
Stk38l	2E-11	0.6	Macrod1	9E-01	0.5
Bcl6	5E-21	0.6	Mtrf1	9E-02	0.5
Dhrs3	7E-25	0.6	Ets2	2E-01	0.5
Nuak1	1E-10	0.6	Boc	7E-09	0.5
Tmem246	3E-05	0.6	Dipk2a	7E-03	0.5
Gm37240	3E-08	0.6	Fam20b	2E-01	0.5
Cdkn1c	1E-06	0.6	Pdzrn4	2E-21	0.5
Cnnm4	6E-04	0.6	Nrbp2	4E-02	0.5
Prkg1	2E-71	0.6	Lpcat1	6E-04	0.5
S100a1	2E-25	0.6	Deptor	3E-02	0.5
Elmod3	1E+00	0.6	Aff3	7E-21	0.5
Ccdc68	5E-04	0.6	Prune2	5E-02	0.5
Slc2a1	5E-12	0.6	Epb41	2E-02	0.5
Nalcn	1E-01	0.6	Usp11	5E-01	0.5
Ica1	2E-05	0.6	Cep126	4E-01	0.5
Cyb5d2	9E-01	0.6	Fahd2a	4E-02	0.5
Tpd52l1	2E-05	0.6	Cmss1	7E-01	0.5
Il17d	3E-07	0.6	Id4	2E-06	0.5
Crim1	4E-20	0.6	Avpi1	5E-01	0.5
Deptor	2E-05	0.6	Spa17	8E-01	0.5
Cdsn	4E-13	0.6	B3galt6	4E-01	0.5
Sox6	3E-58	0.6	Crybg3	4E-02	0.5
Retsat	8E-01	0.6	Cdkn1c	6E-03	0.5
Ets2	6E-09	0.6	Mrpl44	5E-01	0.5
Csgalnact1	6E-48	0.6	Ero1l	2E-01	0.5
Got1	1E-04	0.6	Platr22	3E-01	0.5
Larp1b	6E-11	0.6	Myo1d	2E-10	0.5
Rbmx2	5E-01	0.6	2310010J17Rik	7E-01	0.5
Maged2	1E-13	0.6	Spsb4	8E-02	0.5
Fign	2E-21	0.6	Ncoa3	9E-01	0.5
Col4a5	1E-06	0.6	Cyb5r1	3E-03	0.5
Atoh8	3E-04	0.6	Fchsd2	1E-08	0.5
Aldh1a1	1E-06	0.6	Cgrefl	1E-01	0.5
Ptchd4	6E-10	0.6	Osgin2	2E-03	0.5
Hoxa5	1E-02	0.6	Mogs	3E-03	0.5
Snhg4	3E-09	0.6	Cdv3	3E-08	0.5
Pim3	7E-04	0.6	Pdgfrl	9E-01	0.5
Slc35e3	2E-02	0.6	Baiap2l1	1E-01	0.5
Atg16l2	1E-06	0.6	Pop5	4E-01	0.5
Nthl1	4E-01	0.6	Ptgis	2E-02	0.5
Tbx5	1E-04	0.6	Dctd	3E-01	0.5
Tmem154	6E-07	0.6	Jak2	5E-01	0.5
Plod2	3E-44	0.6	Ldhb	1E-02	0.5
Plekha7	2E-02	0.6	Prkn	1E-03	0.5
Nedd4l	2E-09	0.6	Brd3os	2E-01	0.5
Rab39b	5E-03	0.6	Dus4l	2E-01	0.5
Tmem117	5E-04	0.6	Carmil1	7E-04	0.5
Sdr39u1	5E-02	0.6	Ccdc115	8E-02	0.5
Lpar4	5E-12	0.6	Dnajc15	2E-02	0.5
Mrpl44	6E-01	0.6	Atg16l2	9E-01	0.5
Ascc2	1E-06	0.6	Sobp	5E-07	0.5
Arl4a	1E-12	0.6	Ascc2	2E-03	0.5
Herpud1	1E-16	0.6	Mid1ip1	7E-02	0.5
Tnfrsf10b	6E-01	0.5	Abcb1b	2E-01	0.5

**Articular/Synovial Fibroblast**					
**Control**			***Notch2***$^{tm1.1Ecan}$		
**Gene**	***p***	**FC**	**Gene**	***p***	**FC**
Ndrg4	7E-19	1.2	Efs	4E-20	1.1
Sipa1l2	2E-13	1.2	Dsel	2E-28	1.1
Anks1b	3E-08	1.2	Lgmn	6E-40	1.1
Tlnrd1	5E-26	1.1	Bdnf	3E-16	1.0
Dtx2	4E-21	1.1	Arhgap42	7E-37	1.0
Bcl3	3E-11	1.1	Lpl	2E-47	1.0
Btbd11	1E-24	1.1	Tshz3	2E-25	1.0
Trpm3	2E-10	1.1	Mn1	6E-19	1.0
Ass1	2E-24	1.1	Pfkfb4	4E-04	1.0
Cdkn2b	2E-64	1.1	Clip1	5E-45	1.0
Nid1	8E-42	1.1	Mmp14	2E-72	1.0
Arnt2	1E-25	1.1	Pde1a	1E-20	1.0
Gm46218	8E-09	1.1	Palm	1E-16	1.0
Ddah2	3E-89	1.1	Cmya5	2E-08	1.0
Galnt17	2E-16	1.1	Ldlr	3E-37	1.0
Palm	2E-30	1.1	Csf1	1E-42	1.0
Cavin2	6E-29	1.1	Arhgap21	2E-40	1.0
Gdpd1	6E-10	1.1	Gm26771	5E-15	1.0
Polm	7E-09	1.1	Btbd11	8E-16	1.0
Basp1	1E-35	1.1	Plat	2E-10	1.0
Ano5	2E-07	1.1	Rnase4	3E-37	1.0
Galnt18	3E-30	1.1	Cdkn2b	3E-37	1.0
Ecm1	1E-98	1.1	Twist2	6E-09	1.0
Tlr3	5E-09	1.1	Dkk3	2E-53	1.0
Sec22c	1E-08	1.1	B4galt1	1E-42	1.0
Ppp1r9a	1E-35	1.1	Sh3pxd2b	2E-22	1.0
Itga9	2E-19	1.1	Pcdh19	2E-21	1.0
Arvcf	3E-20	1.1	Cpne2	1E-17	1.0
Nrn1	1E-10	1.1	Hdac7	3E-20	1.0
Fam111a	5E-49	1.1	Msmo1	3E-33	1.0
Tmod2	7E-16	1.1	Fdft1	1E-32	1.0
Manba	9E-13	1.1	Zcchc24	6E-43	1.0
Gm26771	1E-39	1.1	Gas1	3E-78	1.0
Pcdh19	6E-35	1.1	Fdps	3E-41	1.0
Man1a	8E-48	1.1	B3galnt1	5E-10	1.0
Lrrc32	2E-26	1.1	Bicd2	8E-21	1.0
Atp8b1	1E-15	1.1	Serpine2	6E-63	1.0
Lpgat1	9E-50	1.1	Gja1	5E-48	1.0
Sqor	6E-31	1.1	Phldb1	5E-18	1.0
Dock11	6E-23	1.1	Htra3	3E-23	1.0
Rnf144a	3E-13	1.1	Apbb1ip	3E-11	1.0
Acly	4E-43	1.1	Alms1	3E-15	1.0
Ptgr1	3E-23	1.1	Kdsr	2E-25	1.0
Bicd2	1E-43	1.1	Dusp3	7E-22	1.0
Sulf1	2E-45	1.1	Pomk	1E-04	1.0
Plat	1E-15	1.1	Rab40b	2E-12	1.0
Cyp4v3	1E-16	1.1	Thbs2	4E-57	1.0
Rab3il1	2E-24	1.1	Ano6	4E-42	1.0
Tspo	2E-81	1.1	Ptpre	3E-08	1.0
Slit3	1E-52	1.1	C4b	9E-12	1.0
Ralgds	1E-25	1.1	Smurf2	1E-32	1.0
Heg1	3E-23	1.1	Ahr	3E-20	1.0
Cgnl1	9E-54	1.0	Gla	1E-10	1.0
Bin1	4E-47	1.0	Stard9	3E-10	1.0
Trerf1	2E-51	1.0	Scara3	2E-17	1.0
Arhgap24	2E-54	1.0	Notch3	9E-09	1.0
Zfp503	2E-47	1.0	Lama5	5E-10	1.0
Gstm1	5E-71	1.0	Tln2	5E-35	1.0
Lama5	3E-14	1.0	Ppp1r14b	7E-76	1.0
Zc3h12a	8E-10	1.0	Gm32618	8E-08	1.0
Mill2	1E-33	1.0	C1galt1	5E-28	1.0
Dkk3	1E-65	1.0	Plscr4	1E-07	1.0
Slc41a3	3E-15	1.0	Ociad2	1E-07	1.0
Cmya5	8E-21	1.0	Gstm1	7E-50	1.0

Limb Mesenchyme					
Control			*Notch2*$^{tm1.1Ecan}$		
Gene	*p*	FC	Gene	*p*	FC
Epb41l3	9E-43	0.6	Fam20a	6E-47	0.6
Icam1	9E-12	0.6	Mcc	1E-19	0.6
Nfkbia	2E-28	0.6	Ptges3l	4E-09	0.6
Trim16	3E-08	0.6	Ednra	2E-15	0.6
Dubr	1E-14	0.6	Pid1	2E-30	0.6
Zfp449	2E-10	0.6	Srpx	2E-29	0.6
Enc1	3E-09	0.6	Peg13	5E-11	0.6
Lsp1	5E-28	0.6	Grasp	1E-15	0.6
Tgfb1i1	9E-43	0.6	2700069I18Rik	1E-16	0.6
Erc2	7E-09	0.6	Nek6	9E-22	0.6
Tgfbr3	2E-41	0.6	Ltbp4	5E-11	0.6
Bgn	2E-264	0.6	Sqle	6E-22	0.6
Hoxc11	1E-06	0.6	Sh3kbp1	9E-24	0.6
Slc38a4	2E-09	0.6	Hdac7	1E-26	0.6
Kcne4	8E-09	0.6	Arpc1b	4E-85	0.6
Fgf2	3E-13	0.6	Fdps	2E-41	0.6
Twist2	1E-06	0.6	Gcnt2	2E-10	0.6
Meis2	3E-26	0.6	Plxna3	2E-06	0.6
Vegfc	2E-14	0.6	Adamtsl3	2E-36	0.6
Car9	9E-04	0.6	Fras1	3E-11	0.6
Fkbp11	3E-47	0.6	Apobr	1E-10	0.6
Tmsb10	3E-178	0.6	Bgn	5E-202	0.6
Rab29	2E-06	0.6	Flrt2	4E-51	0.6
Dnajb1	1E-20	0.6	Cers4	6E-20	0.6
Rhob	9E-44	0.6	Bdh2	5E-16	0.6
Amot	7E-12	0.6	Svil	4E-52	0.6
Irs1	1E-34	0.6	Ccdc71l	5E-08	0.6
Arpc1b	1E-111	0.6	Picalm	4E-56	0.6
Ece1	3E-30	0.6	Pdlim7	1E-41	0.6
Fhl2	3E-24	0.6	Itsn1	3E-38	0.6
Plppr2	6E-07	0.6	Ccdc74a	2E-05	0.6
Cd47	5E-104	0.6	Adamts6	9E-24	0.6
Zfp618	1E-17	0.6	Dram1	1E-12	0.6
Amer1	7E-17	0.6	Plekhg1	4E-23	0.6
Mapk13	7E-10	0.6	Zbtb8a	2E-06	0.6
Mettl24	8E-05	0.6	Fkbp11	7E-42	0.6
Plxna3	3E-05	0.6	Slc16a7	2E-07	0.6
Pfn2	1E-27	0.6	Gpr27	4E-09	0.6
Rgs10	7E-28	0.6	Mapk7	3E-14	0.6
Adamtsl3	1E-50	0.6	Amot	3E-17	0.6
Bace2	6E-11	0.6	Ccnd1	3E-45	0.6
Mapk7	1E-10	0.6	Sh3pxd2b	3E-32	0.6
Efs	7E-13	0.6	Afap1	2E-53	0.6
Fstl1	9E-164	0.6	Snhg12	2E-53	0.6
B4galt2	8E-15	0.6	Irs1	2E-32	0.6
Prdx4	2E-87	0.6	Kcnab1	2E-11	0.6
Fam149a	2E-11	0.6	Tnc	5E-15	0.6
Tmem37	2E-11	0.6	Tmsb4x	3E-112	0.6
Rps6ka5	4E-13	0.6	Hs6st1	1E-07	0.6
Trim12a	1E-08	0.6	Adgrg2	7E-16	0.6
Morc4	2E-17	0.6	Camk2n1	6E-06	0.6
Mgll	4E-18	0.6	Ssc5d	9E-31	0.6
Mmp2	1E-43	0.6	Csrp2	3E-26	0.6
Mrps6	8E-19	0.6	Pygb	1E-15	0.6
Abi3bp	4E-105	0.6	Ust	2E-21	0.6
Ppfibp2	6E-14	0.6	Adm	2E-29	0.6
Timp1	3E-45	0.6	Ttc39c	3E-10	0.6
Wasf1	1E-09	0.6	Gamt	9E-34	0.6
Svep1	6E-95	0.6	Inka1	1E-10	0.6
Sh3pxd2b	1E-22	0.6	Ror1	2E-33	0.6
Tshz2	4E-09	0.6	Emc2	5E-57	0.6
Apba1	9E-16	0.6	Cttnbp2	1E-06	0.6
Ecm1	2E-62	0.6	Wasf1	5E-12	0.6
Gem	2E-19	0.6	Synj2	2E-12	0.6

Chondrogenic					
Control			*Notch2*$^{tm1.1Ecan}$		
Gene	*p*	FC	Gene	*p*	FC
Phyh	2E-69	0.8	Gng13	8E-21	0.8
Hoxa5	2E-12	0.8	Hrct1	1E-12	0.8
Cfap74	7E-12	0.8	1700023F06Rik	3E-09	0.8
Ccdc115	2E-21	0.8	Hoxa5	7E-11	0.8
Il18	5E-21	0.8	Gm42439	6E-11	0.8
Gm43948	2E-13	0.8	Herpud1	3E-89	0.8
Plscr2	2E-13	0.8	Runx2	5E-17	0.8
Usp53	7E-14	0.8	Gtf2f2	9E-31	0.8
Got1	8E-32	0.8	Cryl1	7E-15	0.8
Cpa6	6E-07	0.8	Asrgl1	1E-32	0.8
Fbln7	1E-64	0.8	Sox6	1E-136	0.8
Adat1	3E-15	0.8	Hist1h2ac	3E-09	0.8
Gm45025	2E-08	0.8	Gfpt1	3E-94	0.8
Gcnt1	3E-11	0.8	Ets1	8E-20	0.8
Fbxl2	5E-28	0.8	Mtmr12	2E-22	0.8
Ldhb	3E-65	0.8	Nrp2	2E-71	0.8
Herpud1	4E-96	0.8	Stk39	2E-42	0.7
Slc25a13	4E-22	0.8	Prickle1	8E-33	0.7
Hotairm1	6E-28	0.8	Gm43948	4E-08	0.7
Lpcat1	2E-17	0.8	Col4a5	3E-28	0.7
Tiparp	4E-103	0.8	Agpat4	5E-35	0.7
Mtmr12	2E-28	0.8	Sdc4	9E-128	0.7
Peg10	1E-05	0.8	Cep126	9E-09	0.7
Airn	1E-142	0.8	Gm16124	4E-12	0.7
Ctnnal1	2E-37	0.7	Rps6ka1	1E-08	0.7
Cdk6	6E-28	0.7	Phlda1	2E-32	0.7
Prkaa2	1E-18	0.7	Fam20b	1E-23	0.7
C1qtnf4	1E-19	0.7	Dcn	1E-135	0.7
Epb41l4b	4E-07	0.7	Ube2cbp	3E-10	0.7
Gramd1c	1E-08	0.7	Phtf2	2E-21	0.7
Shtn1	3E-11	0.7	Acaca	2E-61	0.7
BC065397	9E-14	0.7	Slc38a2	5E-78	0.7
Dclre1c	1E-02	0.7	Cdkl5	8E-31	0.7
Ptchd4	1E-24	0.7	Jak2	3E-15	0.7
Eno1	2E-122	0.7	Mcu	3E-50	0.7
Gfpt1	6E-98	0.7	Stbd1	1E-20	0.7
Id1	3E-87	0.7	Slc35e3	7E-13	0.7
Prdm6	2E-16	0.7	Slc16a1	3E-14	0.7
Asrgl1	8E-26	0.7	Lrrc4	3E-21	0.7
Ddit3	4E-71	0.7	Patj	2E-18	0.7
Fam160a1	7E-10	0.7	Gm26827	3E-12	0.7
Atoh8	1E-18	0.7	Fam131a	3E-06	0.7
4930581F22Rik	3E-16	0.7	Sorl1	1E-17	0.7
3110021N24Rik	3E-15	0.7	Nr4a3	3E-08	0.7
Grin3a	3E-05	0.7	Ddit3	5E-74	0.7
Sdc4	2E-142	0.7	H19	2E-65	0.7
Maged2	2E-60	0.7	Ankrd44	6E-38	0.7
Loxl3	3E-25	0.7	Jade2	1E-09	0.7
Gm19522	2E-09	0.7	Rcc2	5E-64	0.7
Dusp14	5E-34	0.7	Meg3	4E-116	0.7
Rcc2	4E-70	0.7	Stxbp6	4E-42	0.7
Agpat4	2E-37	0.7	Gnb4	1E-15	0.7
Npnt	1E-10	0.7	Gli2	3E-28	0.7
Gm9929	3E-09	0.7	Slc25a38	1E-17	0.7
Shroom4	7E-20	0.7	D3Ertd751e	3E-16	0.7
Stxbp2	2E-15	0.7	Slc19a1	2E-08	0.7
Hist1h4i	1E-07	0.7	Ddb2	7E-06	0.7
Gm42439	2E-13	0.7	Phyh	9E-55	0.7
Ttc39b	8E-34	0.7	Gm16599	4E-08	0.7
Glis1	1E-11	0.7	Pex11a	3E-06	0.7
Pfkl	6E-34	0.7	Fmod	1E-103	0.7
Cdkl5	3E-35	0.7	Sap30	3E-32	0.7
Arhgdig	2E-07	0.7	Epha2	2E-10	0.7
Tmem117	3E-16	0.7	0610009E02Rik	1E-05	0.7

Fibroblast					
Control			*Notch2*$^{tm1.1Ecan}$		
Gene	*p*	FC	Gene	*p*	FC
Plcxd2	6E-07	0.8	Pla1a	2E-16	0.8
Cchcr1	9E-09	0.8	Cdon	4E-62	0.8
Pkia	5E-17	0.8	Nptxr	3E-10	0.8
Ccdc181	6E-07	0.8	Nup160	6E-12	0.8
Haus8	4E-15	0.8	Mtss1	1E-14	0.8
Ntn4	4E-12	0.8	H2afv	1E-69	0.8
Raet1e	8E-13	0.8	Alg13	3E-16	0.8
Car5b	2E-13	0.8	Chek2	1E-12	0.8
Ccdc80	4E-95	0.8	Tubg1	1E-20	0.8
Usp1	5E-13	0.7	Syce2	4E-17	0.8
Erg	5E-22	0.7	Barx1	7E-49	0.8
Ttf2	2E-07	0.7	Dctpp1	6E-23	0.8
Terf1	3E-12	0.7	Pim1	2E-15	0.8
Gm36033	4E-07	0.7	Il16	6E-16	0.8
Foxo1	8E-23	0.7	Nsd2	6E-16	0.8
Pole2	3E-07	0.7	Lsm3	5E-25	0.8
Tubb5	9E-25	0.7	Sun2	2E-17	0.8
Rbmx2	1E-06	0.7	Notch3	9E-06	0.8
Cep192	3E-09	0.7	Fap	2E-22	0.8
Pde3b	2E-24	0.7	Rnf26	7E-12	0.8
Cntrob	3E-06	0.7	Nup107	8E-17	0.8
Nptxr	2E-04	0.7	Dnaaf2	3E-10	0.8
Cep57l1	6E-11	0.7	Ppp2r5d	3E-15	0.8
Plb1	2E-08	0.7	Rfwd3	2E-13	0.8
Ppil1	4E-05	0.7	Cep135	3E-14	0.8
Klc3	3E-10	0.7	1700024B18Rik	9E-11	0.8
Gm41764	9E-06	0.7	Miip	2E-09	0.8
Mcm4	2E-10	0.7	Fgfr1op	5E-15	0.8
Rccd1	6E-08	0.7	Afmid	2E-12	0.8
Magi3	5E-35	0.7	Pradc1	3E-15	0.8
Nalcn	2E-05	0.7	Tagln2	1E-57	0.8
Gm12216	5E-05	0.7	Pak4	1E-06	0.8
Itga3	2E-06	0.7	Synm	2E-13	0.8
Ablim1	8E-18	0.7	Foxo1	3E-26	0.8
Lrrc27	9E-08	0.7	Cenpj	2E-13	0.8
Tm4sf1	2E-30	0.7	Gpsm2	4E-31	0.8
Plekhg3	9E-12	0.7	Phactr1	3E-14	0.8
Catspere2	1E-05	0.7	Medag	1E-37	0.8
Lpin3	2E-08	0.7	Nup155	4E-15	0.8
Sema5a	7E-13	0.7	Mcm9	3E-13	0.8
Tspan12	2E-12	0.7	Pdss1	2E-08	0.8
Ccdc77	3E-06	0.7	Myh10	2E-24	0.8
Ifit2	1E-16	0.7	Ecm2	5E-28	0.8
Pomgnt2	5E-05	0.7	Nemp1	9E-15	0.8
Gm42702	5E-06	0.7	Rbmx2	6E-09	0.8
Zfp101	2E-09	0.7	Nxt1	5E-16	0.8
Umps	1E-08	0.7	Cenpo	2E-09	0.8
Anp32e	2E-19	0.7	Umps	1E-13	0.8
Colec12	8E-41	0.7	Ywhah	9E-36	0.8
Klhl23	4E-06	0.7	Anp32e	4E-30	0.8
Nucks1	4E-28	0.7	Nt5dc2	3E-23	0.8
Nup133	1E-05	0.7	Fzr1	2E-18	0.8
Lcn2	1E-05	0.7	Mcph1	7E-15	0.8
Inpp4b	3E-01	0.7	Pparg	5E-03	0.8
Nup107	1E-07	0.7	Nelfcd	4E-09	0.8
Mlana	2E-06	0.7	Nmral1	7E-08	0.8
Sulf1	2E-20	0.7	Plekhg3	1E-22	0.8
Cmc2	5E-15	0.7	Hpf1	3E-25	0.8
Rdh11	5E-10	0.7	Bmp4	4E-38	0.8
Pcnt	4E-11	0.7	Esr1	2E-14	0.8
Fgfr1op	9E-10	0.7	Lsm2	6E-21	0.8
Tprg	3E-09	0.7	Thbd	1E-23	0.8
Noct	8E-08	0.7	Ccdc77	1E-13	0.8
Fsd1l	7E-06	0.7	Mtbp	1E-10	0.8

Undefined					
Control			*Notch2*$^{tm1.1Ecan}$		
Gene	*p*	FC	Gene	*p*	FC
Boc	2E-18	0.5	Lrrc51	8E-01	0.5
Pdzrn3	1E-34	0.5	Kif21a	1E-02	0.5
Prune2	3E-05	0.5	Gnpnat1	7E-01	0.5
Sdc3	2E-06	0.5	Plb1	2E-02	0.5
Me1	1E-14	0.5	Larp1b	6E-02	0.5
Gpt2	7E-04	0.5	C530008M17Rik	9E-01	0.5
Gm14966	1E-02	0.5	Dnajb9	6E-01	0.5
Plagl1	2E-26	0.5	Dmd	1E-08	0.5
Agpat5	4E-07	0.5	Csgalnact1	2E-15	0.5
Gne	4E-01	0.5	Epha2	7E-01	0.5
Dhrs9	1E-16	0.5	Ccpg1os	4E-01	0.5
Egfl7	2E-01	0.5	Atp10a	1E-02	0.5
Shroom4	5E-07	0.5	Myom1	2E-02	0.5
Cmss1	2E-02	0.5	Phlda1	1E-01	0.5
Cep126	5E-04	0.5	Emilin1	2E-02	0.5
N4bp2l1	2E-04	0.5	Dhrs7	8E-02	0.5
Jade1	8E-07	0.5	Eef1e1	4E-01	0.5
Mtcl1	8E-06	0.5	Loxl3	3E-01	0.5
Nr2c2ap	4E-01	0.5	Slc22a17	3E-03	0.5
Usp29	9E-14	0.5	Nupr1	8E-20	0.5
Pcdh11x	1E-20	0.5	Fbxl2	3E-01	0.5
1110002L01Rik	8E-02	0.5	Stk39	1E-03	0.5
Acot2	7E-01	0.5	0610043K17Rik	7E-01	0.5
Sdc4	4E-39	0.5	Dhrs3	4E-06	0.5
Phlda1	3E-06	0.5	Srebf1	1E-02	0.5
Jpx	5E-01	0.5	Fh1	3E-10	0.5
B930036N10Rik	7E-09	0.5	Bmp5	1E-05	0.5
Barx1	5E-17	0.5	Slc7a1	3E-04	0.5
Tenm4	7E-15	0.5	Nfu1	2E-01	0.5
Pde10a	7E-01	0.5	Nufip1	4E-04	0.5
Hdac9	1E-01	0.5	Dand5	5E-02	0.5
Map3k20	1E-10	0.5	Creld2	3E-01	0.5
Chd1l	4E-02	0.5	Ell2	2E-06	0.5
Ecrg4	4E-49	0.5	Mtus1	4E-03	0.5
Ttc39b	2E-07	0.5	Ctf1	9E-02	0.5
4933406I18Rik	2E-05	0.5	Esr1	2E-01	0.5
Zim1	4E-14	0.5	Tpd52l1	2E-02	0.5
Thap1	8E-01	0.5	Sorl1	6E-02	0.5
Dixdc1	9E-07	0.5	Psmg4	6E-01	0.5
Pex11a	6E-02	0.5	Golph3	8E-02	0.5
Dusp8	6E-03	0.5	Gas5	1E-58	0.5
Csmd1	4E-11	0.5	5430416N02Rik	6E-01	0.5
Tnfrsf11b	5E-21	0.5	Ifrd2	5E-01	0.5
Prdm16	4E-07	0.5	Stard10	3E-01	0.5
Gale	4E-03	0.5	Slc41a2	7E-01	0.5
Hsd17b1	5E-04	0.5	Agpat5	5E-01	0.5
Ctsh	1E-16	0.5	Cnot11	4E-03	0.5
Pdgfrl	1E-03	0.5	Gpx3	7E-01	0.5
Leng9	6E-01	0.5	Il16	1E-03	0.5
Pik3cb	1E-03	0.5	Retsat	7E-01	0.5
Wars	5E-02	0.5	Jam2	7E-01	0.5
Dach2	2E-01	0.5	Mtus2	5E-03	0.5
Mthfd1l	2E-02	0.5	Gstm5	4E-03	0.5
Rrp9	8E-02	0.5	Npr2	8E-01	0.5
Haus8	5E-05	0.5	Sox6	3E-26	0.5
Gm43948	2E-05	0.5	Dnajc4	9E-02	0.5
Epas1	5E-11	0.5	Dus3l	6E-04	0.5
Patj	1E-07	0.5	Lrrc75b	4E-02	0.5
Ctf1	4E-05	0.5	Ppm1h	1E-01	0.5
Kctd15	6E-01	0.5	Ciapin1	1E-01	0.5
Igfbp5	3E-15	0.5	Ccdc62	9E-01	0.4
Rcl1	4E-01	0.5	Meg3	1E-30	0.4
Stbd1	1E-02	0.5	Sort1	5E-02	0.4
Gm42418	1E-07	0.5	8430429K09Rik	9E-01	0.4

Articular/Synovial Fibroblast					
Control			*Notch2*$^{tm1.1Ecan}$		
Gene	*p*	FC	Gene	*p*	FC
Lrmda	1E-19	1.0	Vgll3	8E-41	1.0
Nqo2	1E-17	1.0	Ccn5	4E-62	1.0
Stard9	6E-15	1.0	Fam171a1	9E-12	1.0
Syne2	8E-18	1.0	Timp2	1E-137	1.0
Mt2	4E-83	1.0	Rnf144a	6E-14	1.0
Gng13	5E-06	1.0	Akap13	1E-64	1.0
Nsg1	3E-31	1.0	Nqo2	8E-11	1.0
Hspa12b	4E-11	1.0	Heg1	1E-14	1.0
Pdzd2	9E-17	1.0	Nod1	2E-09	1.0
B4galt1	1E-60	1.0	Bmp2k	6E-19	1.0
Xaf1	2E-13	1.0	Ggt5	6E-09	1.0
Tubb2b	1E-18	1.0	Pkia	1E-18	0.9
Akap13	1E-100	1.0	A4galt	1E-14	0.9
Adam19	4E-20	1.0	Adam9	2E-27	0.9
Zcchc24	1E-55	1.0	Man1a	4E-25	0.9
Pwwp2b	5E-07	1.0	Tlnrd1	7E-13	0.9
Pltp	1E-45	1.0	Ephb6	4E-11	0.9
Dpyd	2E-18	1.0	Elmo1	2E-19	0.9
Rras2	2E-12	1.0	Tspo	9E-45	0.9
Kcnab1	8E-08	1.0	Fam171a2	5E-06	0.9
Sqle	1E-45	1.0	Slit3	1E-37	0.9
H2-K1	2E-44	1.0	Taok3	4E-29	0.9
Cdr1os	2E-12	1.0	Dock9	2E-21	0.9
Serpine2	3E-88	1.0	Pdlim1	4E-18	0.9
Adam9	2E-37	1.0	Flnc	5E-15	0.9
Stk10	3E-09	1.0	Erg28	1E-38	0.9
Rtn4r	2E-08	1.0	Nectin1	5E-06	0.9
Kazn	4E-22	1.0	Tap1	6E-03	0.9
Svep1	3E-110	1.0	Ptgr1	6E-09	0.9
Gstt1	6E-26	1.0	Runx1t1	5E-66	0.9
Castor2	2E-14	1.0	Fgf14	2E-05	0.9
C4b	1E-16	1.0	Atp8b1	4E-11	0.9
Nod1	5E-10	1.0	Ptrh1	2E-09	0.9
Plce1	5E-54	1.0	Adamtsl4	4E-17	0.9
Timp2	1E-201	1.0	Gm44148	8E-05	0.9
Lynx1	6E-14	1.0	Lhfpl2	5E-26	0.9
Ksr1	3E-09	1.0	Sord	6E-17	0.9
Vgll3	3E-62	1.0	Bin1	7E-31	0.9
Hmgcs1	4E-46	1.0	Gstt1	4E-12	0.9
Msmo1	1E-42	1.0	H2-T23	5E-10	0.9
Svil	4E-53	1.0	Mmp11	4E-08	0.9
Ldlr	2E-42	1.0	Rab6b	5E-07	0.9
Cemip	5E-03	1.0	Mettl7a1	3E-14	0.9
Fam171a1	5E-23	1.0	Lpgat1	4E-28	0.9
Dapk1	2E-48	1.0	Angptl2	2E-07	0.9
Alms1	8E-21	1.0	Bst2	2E-23	0.9
Steap3	2E-61	1.0	Nmnat2	1E-04	0.9
Ano6	4E-53	1.0	Ecm1	1E-54	0.9
St8sia2	5E-08	1.0	Ndrg4	6E-10	0.9
S100a13	7E-95	1.0	Man1c1	6E-19	0.9
Map6	1E-32	1.0	Cdkn2a	1E-15	0.9
Fbxo32	9E-11	1.0	Dlgap4	2E-32	0.9
Nrg1	2E-04	1.0	Serac1	8E-03	0.9
Ang	9E-09	1.0	Ass1	1E-08	0.9
Thbs2	3E-54	1.0	S100a6	8E-89	0.9
Ropn1l	3E-12	1.0	Stard4	1E-08	0.9
Arsi	6E-13	1.0	Ifitm3	6E-77	0.9
Ece1	6E-34	1.0	S100a13	2E-58	0.9
Adam23	6E-12	1.0	Adgre5	2E-08	0.9
Hvcn1	1E-08	1.0	Gm16364.1	1E-07	0.9
Gas2	2E-60	1.0	Ralgds	2E-17	0.9
Ifit3	4E-15	1.0	St8sia2	3E-04	0.9
Atp9a	4E-15	1.0	Arl15	7E-34	0.9
Sord	3E-24	1.0	Slc36a1	2E-07	0.9

Limb Mesenchyme					
Control			*$Notch2^{tm1.1Ecan}$*		
Gene	*p*	FC	Gene	*p*	FC
Angptl1	5E-10	0.6	Nsdhl	9E-13	0.5
Itih5	1E-13	0.5	Syne3	2E-12	0.5
Rnd3	1E-65	0.5	Porcn	3E-06	0.5
Id4	1E-01	0.5	Cachd1	4E-21	0.5
B4galt5	5E-10	0.5	Tle2	4E-09	0.5
Itsn1	5E-38	0.5	Ric8a	2E-16	0.5
Sord	7E-14	0.5	Efnb1	1E-12	0.5
Nkd1	3E-14	0.5	Ano6	7E-45	0.5
Rnf150	1E-38	0.5	Amer1	2E-13	0.5
Itprid2	5E-16	0.5	Mn1	2E-16	0.5
Bmp4	1E-59	0.5	Ecm1	6E-77	0.5
Cilp2	2E-04	0.5	Cbx6	4E-28	0.5
Chst2	5E-16	0.5	Idi1	4E-15	0.5
Slc44a2	2E-18	0.5	Zdhhc9	2E-10	0.5
Gpr27	2E-07	0.5	Zfp810	4E-11	0.5
Fbxo32	1E-04	0.5	Tlcd2	2E-11	0.5
Fndc4	1E-11	0.5	Gadd45g	1E-19	0.5
Adcy6	7E-05	0.5	Tpm1	2E-70	0.5
Ntng1	4E-13	0.5	Shank1	2E-11	0.5
Iah1	5E-29	0.5	Lgals3bp	1E-27	0.5
Cbx6	1E-19	0.5	Xaf1	6E-15	0.5
Hdac7	3E-20	0.5	Col5a3	3E-38	0.5
Nectin2	4E-05	0.5	Zfp618	3E-19	0.5
Tstd3	4E-07	0.5	Npc2	2E-134	0.5
Afap1	3E-53	0.5	Fads3	8E-29	0.5
Il1r1	6E-01	0.5	Fhl2	4E-34	0.5
Adamts2	2E-36	0.5	Klf7	1E-33	0.5
Trib2	2E-08	0.5	Alcam	4E-21	0.5
Asap3	2E-10	0.5	Zcchc24	2E-37	0.5
Map4k3	9E-39	0.5	Morc4	9E-13	0.5
Nek6	2E-16	0.5	Dsel	4E-15	0.5
Sh3kbp1	2E-22	0.5	Pik3r3	6E-12	0.5
Ttyh3	2E-21	0.5	Mfsd3	1E-06	0.5
Nrbp2	3E-08	0.5	Pros1	1E-29	0.5
Dram1	2E-11	0.5	Stat2	2E-10	0.5
Zfhx4	4E-59	0.5	Dennd4a	4E-35	0.5
Sh3rf3	2E-41	0.5	Ablim1	2E-25	0.5
Il11ra1	1E-72	0.5	Meis3	1E-09	0.5
Sema3d	9E-03	0.5	Igsf3	1E-10	0.5
Hebp2	6E-14	0.5	Svep1	6E-85	0.5
Snx9	8E-63	0.5	Baiap2	5E-30	0.5
Fat4	2E-52	0.5	Ncs1	2E-13	0.5
Cacna1d	2E-10	0.5	Cbr3	6E-21	0.5
Naprt	2E-04	0.5	St5	3E-24	0.5
Cachd1	1E-25	0.5	Bst2	6E-39	0.5
Gcnt2	5E-07	0.5	Eif4e3	2E-06	0.5
Plekhg1	7E-17	0.5	Cavin1	6E-53	0.5
Pros1	2E-18	0.5	Idh1	2E-42	0.5
Prkab2	6E-07	0.5	Ggta1	8E-08	0.5
Zbtb8a	2E-08	0.5	Twist1	1E-50	0.5
Glipr2	7E-17	0.5	Hccs	4E-11	0.5
Ndnf	6E-30	0.5	Gm4117	1E-16	0.5
Zfp36l1	1E-06	0.5	Negr1	8E-09	0.5
Npc2	2E-141	0.5	Myadm	1E-21	0.5
Tmsb4x	8E-109	0.5	Gm13832	2E-09	0.5
Cnn2	5E-41	0.5	Igsf10	3E-53	0.5
Zfp810	7E-06	0.5	Npas2	3E-07	0.5
C1qtnf1	8E-12	0.5	Pqlc3	2E-12	0.5
Gsta4	3E-32	0.5	Ier5l	1E-17	0.5
Gamt	2E-29	0.5	Arsj	4E-26	0.5
Slc36a1	2E-03	0.5	Tnfrsf12a	6E-23	0.5
Stard9	2E-13	0.5	Fbln1	9E-20	0.5
Efemp2	5E-82	0.5	Efna2	2E-06	0.5
Plp2	1E-24	0.5	Flna	4E-43	0.5

Chondrogenic					
Control			*$Notch2^{tm1.1Ecan}$*		
Gene	*p*	FC	Gene	*p*	FC
Dync1i1	7E-15	0.7	Immp2l	5E-38	0.7
Platr22	6E-12	0.7	Ogt	2E-77	0.7
Bpgm	2E-08	0.7	Fabp5	1E-01	0.7
Carmil1	2E-44	0.7	Dop1b	1E-22	0.7
Ivns1abp	3E-27	0.7	Prdm6	7E-11	0.7
Slc2a1	4E-36	0.7	Hyal1	6E-07	0.7
Gramd1b	1E-08	0.7	Prkaa2	3E-14	0.7
Agrn	2E-14	0.7	Haus8	7E-15	0.7
Pde3a	2E-61	0.7	Mtcl1	1E-12	0.7
Dach2	2E-05	0.7	Dlk1	4E-06	0.7
Gm38560	3E-07	0.7	Zbtb20	7E-133	0.7
Slc7a5	1E-38	0.7	Ak4	6E-10	0.7
Mthfd1l	4E-28	0.7	Slc2a1	1E-27	0.7
Dcn	3E-148	0.7	Ppp1r9a	5E-14	0.7
Vldlr	1E-30	0.7	Il18	3E-12	0.7
Gt(ROSA)26Sor	4E-18	0.7	Gm37240	4E-27	0.7
Sipa1l1	1E-57	0.7	Psd3	2E-55	0.7
Pag1	1E-33	0.7	Limd1	1E-27	0.7
Epb41	7E-33	0.7	Chsy3	2E-33	0.7
Ahi1	5E-38	0.7	Lrrc8d	2E-06	0.7
H19	1E-55	0.7	Celf4	7E-18	0.7
4930517O19Rik	3E-11	0.7	Stxbp2	1E-11	0.7
Edil3	3E-75	0.7	Baiap2l1	4E-18	0.7
Arpc5l	2E-91	0.7	Spred1	1E-39	0.7
Enpp1	1E-16	0.7	Arpc5l	5E-76	0.7
Fam180a	1E-17	0.7	Tprg	4E-07	0.7
Osgin2	4E-38	0.7	Bcat1	1E-31	0.7
Mtcl1	9E-14	0.7	Slc25a13	5E-19	0.7
Gli2	5E-29	0.7	Kctd15	1E-07	0.7
Zfp469	1E-10	0.7	Nek1	2E-18	0.7
Immp2l	7E-46	0.7	Dixdc1	3E-12	0.7
Lrrc4	3E-22	0.7	Ahi1	4E-34	0.7
Sgk3	2E-25	0.7	Gt(ROSA)26Sor	1E-14	0.7
Hk2	4E-40	0.7	Hk2	2E-31	0.7
Trp53inp1	6E-24	0.7	Slc25a33	1E-19	0.7
Mtss2	4E-39	0.7	N4bp2l1	3E-12	0.7
Ogt	7E-78	0.7	Sgk3	3E-22	0.7
Aldh1l2	5E-55	0.7	Fbxo2	1E-15	0.7
Gm15050	6E-10	0.7	Tiparp	1E-69	0.7
Abhd6	8E-10	0.7	Lrrc51	8E-14	0.7
Chdh	9E-09	0.7	Ggnbp1	4E-13	0.6
Gtf2f2	2E-27	0.7	Dok1	1E-10	0.6
Tpbg	3E-18	0.7	Taf13	2E-14	0.6
Pdgfrl	1E-08	0.7	Unc5b	4E-08	0.6
Dctd	2E-10	0.6	Tnrc6b	6E-89	0.6
Bcat1	1E-29	0.6	Golph3	4E-49	0.6
Sox6	2E-114	0.6	Thrb	1E-04	0.6
Hoxa7	1E-14	0.6	Prkn	1E-52	0.6
1600002K03Rik	4E-23	0.6	Cspg4	3E-15	0.6
Ets2	1E-24	0.6	4930481A15Rik	6E-11	0.6
Tom1l2	3E-58	0.6	Sipa1l1	4E-52	0.6
Chsy1	3E-40	0.6	Megf9	5E-15	0.6
Atg16l2	5E-19	0.6	Rarg	5E-35	0.6
Gm37768	3E-05	0.6	Yipf2	7E-10	0.6
Cdsn	3E-33	0.6	Ank	6E-11	0.6
Aig1	5E-82	0.6	Ivns1abp	1E-17	0.6
Papss1	3E-43	0.6	Sesn3	2E-59	0.6
Plscr1	4E-09	0.6	Aldh6a1	2E-17	0.6
Psd3	6E-55	0.6	Samd12	8E-18	0.6
Adgrg6	2E-55	0.6	Snhg4	3E-23	0.6
Lman2l	3E-18	0.6	Srpx2	3E-68	0.6
Gm28375	3E-02	0.6	Rhbdd1	5E-33	0.6
Omd	6E-67	0.6	Fgd6	1E-08	0.6
Hist2h3c2	4E-05	0.6	Nim1k	6E-10	0.6

Fibroblast					
Control			*$Notch2^{tm1.1Ecan}$*		
Gene	*p*	FC	Gene	*p*	FC
Hes1	8E-35	0.7	Ndrg1	1E-31	0.8
Angptl1	3E-25	0.7	Uaca	4E-14	0.8
Cep295	4E-11	0.7	Rasa3	4E-23	0.8
Cep70	7E-11	0.7	Etaa1	2E-17	0.7
Adm	1E-22	0.7	Hmgb1	3E-99	0.7
Crip2	2E-51	0.7	Nedd1	8E-12	0.7
Palmd	4E-13	0.7	Angptl1	4E-29	0.7
Scarb1	7E-08	0.7	Exosc8	5E-18	0.7
Mical2	9E-10	0.6	Vrk2	9E-10	0.7
Stac	6E-05	0.6	Gm28875	9E-08	0.7
Slc1a6	7E-08	0.6	Klhl23	2E-08	0.7
Mturn	2E-07	0.6	Cx3cl1	5E-07	0.7
Rab23	4E-13	0.6	Nin	7E-10	0.7
Ttbk2	1E-02	0.6	Csmd1	3E-13	0.7
Poc5	4E-05	0.6	Ccdc15	2E-10	0.7
Fbln1	2E-16	0.6	Sema5a	8E-14	0.7
P4htm	1E-05	0.6	Lox	6E-87	0.7
P4ha2	1E-38	0.6	Gucy1a2	5E-13	0.7
Slbp	6E-09	0.6	Grb14	3E-28	0.7
Cks1b	2E-09	0.6	Nasp	4E-22	0.7
Nsg1	4E-14	0.6	Cdca4	2E-12	0.7
Pxdc1	2E-17	0.6	Gm9844	2E-07	0.7
Loxl1	5E-40	0.6	Gjc1	7E-18	0.7
Gemin6	2E-06	0.6	Tgfb2	4E-59	0.7
Pomt1	3E-06	0.6	Pms2	7E-09	0.7
Lsm3	8E-13	0.6	Mdc1	3E-12	0.7
Ehd2	8E-46	0.6	Lctl	6E-08	0.7
Anapc15	7E-10	0.6	Lrch2	1E-06	0.7
Ddx39	5E-12	0.6	Atp2b4	1E-09	0.7
Pradc1	5E-08	0.6	Tead2	6E-11	0.7
Atp6v0e2	3E-02	0.6	a	1E-15	0.7
Cdca4	9E-11	0.6	Lmf2	2E-17	0.7
Sqle	4E-21	0.6	Gm12153	1E-08	0.7
Dctpp1	2E-09	0.6	Tubd1	5E-05	0.7
Fdps	3E-25	0.6	Prag1	8E-09	0.7
Ckap5	1E-12	0.6	Ntn4	8E-12	0.7
Dusp8	1E-06	0.6	Mpi	3E-07	0.7
Cx3cl1	1E-03	0.6	Colec12	4E-41	0.7
Spata24	4E-08	0.6	Zfp948	3E-12	0.7
Pold3	4E-08	0.6	Dock5	3E-11	0.7
Etaa1	3E-05	0.6	Cep112	1E-14	0.7
Mis12	3E-07	0.6	Rnaseh2c	1E-22	0.7
App	4E-82	0.6	Rasl12	8E-06	0.7
Cav1	4E-32	0.6	Pold2	2E-12	0.7
Fads2	2E-12	0.6	Lrig1	2E-12	0.7
Ift140	2E-03	0.6	Ddit4l	2E-10	0.7
Ahnak2	5E-45	0.6	Pcp4l1	3E-05	0.7
Cobll1	1E-23	0.6	Alg8	5E-14	0.7
Vgll3	3E-39	0.6	Fsd1l	1E-05	0.7
Nasp	9E-11	0.6	Nudt1	1E-12	0.7
H2afv	1E-34	0.6	Fancm	2E-07	0.7
Ccdc57	8E-05	0.6	Herc6	2E-11	0.7
Cenpt	4E-10	0.6	Zfp111	3E-05	0.7
Tex9	3E-05	0.6	Tpm4	3E-55	0.7
Ccn4	8E-05	0.6	Ranbp17	2E-14	0.7
Itgb3bp	4E-13	0.6	Ccp110	1E-11	0.7
Tspo	3E-34	0.6	Gm49692	2E-04	0.7
Sept10	2E-10	0.6	Bub3	4E-25	0.7
Hmgb3	2E-09	0.6	Pole2	4E-08	0.7
Pxdn	8E-20	0.6	Stk10	9E-10	0.7
B930095G15Rik	1E-06	0.6	Spata24	1E-09	0.7
Rap1gap2	2E-06	0.6	Ift140	2E-07	0.7
Slf1	8E-12	0.6	Xaf1	3E-01	0.7
Haus7	3E-10	0.6	Rin1	9E-06	0.7

Undefined					
Control			*$Notch2^{tm1.1Ecan}$*		
Gene	*p*	FC	Gene	*p*	FC
Wdr45	9E-01	0.5	Cryl1	4E-01	0.4
Med12l	9E-04	0.5	4933400C23Rik	8E-01	0.4
Synpo	4E-06	0.5	Sdc3	1E-01	0.4
Ppm1h	3E-13	0.5	Cd46	2E-01	0.4
2700097O09Rik	6E-01	0.5	Alg2	3E-01	0.4
Slc16a4	8E-03	0.5	Crebl2	6E-01	0.4
Gprc5c	6E-03	0.5	Spata1	2E-01	0.4
Tarbp2	6E-01	0.5	Ric3	7E-01	0.4
Per2	1E-03	0.5	Pts	8E-01	0.4
Zpbp	2E-01	0.5	Ssr2	2E-03	0.4
Btg1	3E-15	0.5	Bhlhb9	9E-03	0.4
Tsacc	8E-03	0.5	Prkg1	1E-30	0.4
Fam20b	5E-05	0.5	Avil	4E-01	0.4
Etv5	4E-08	0.5	Nudcd1	5E-01	0.4
5930430L01Rik	3E-06	0.5	Hrct1	1E-02	0.4
Fbxl2	1E-04	0.5	Siae	6E-04	0.4
Pag1	3E-06	0.5	Haus8	1E-01	0.4
Foxc1	2E-21	0.5	Eml2	2E-01	0.4
Psd3	3E-17	0.5	Lonp1	2E-01	0.4
Hk2	8E-04	0.5	Me1	7E-02	0.4
Gm15834	1E-02	0.5	Cutc	3E-01	0.4
Glcci1	3E-04	0.5	Shroom4	2E-01	0.4
Pcolce2	1E-08	0.5	Calml4	3E-02	0.4
Taco1	7E-01	0.5	Klhl21	4E-01	0.4
Sobp	3E-11	0.5	Hspa5	8E-24	0.4
Cap2	7E-02	0.5	Wdr48	2E-01	0.4
Zfp141	5E-02	0.5	Armh4	5E-02	0.4
Wdr35	3E-06	0.5	Malt1	6E-01	0.4
4930581F22Rik	2E-06	0.5	Rbm4b	3E-04	0.4
Zbtb46	1E-01	0.5	Nt5c3	1E+00	0.4
Csf2ra	1E+00	0.5	Srpx2	2E-06	0.4
Prxl2c	3E-08	0.5	B4galt4	2E-02	0.4
Isyna1	2E-01	0.5	Nsun4	1E-01	0.4
Uck2	6E-03	0.5	Txndc11	9E-01	0.4
Baiap2l1	2E-12	0.5	Nuak2	1E-03	0.4
Tchh	1E-02	0.5	Col4a5	2E-02	0.4
Sgk3	3E-11	0.5	Ccnq	4E-01	0.4
Pard6a	3E-01	0.5	Aldh6a1	5E-02	0.4
Lef1	6E-08	0.5	Zdhhc12	3E-01	0.4
Lncppara	2E-06	0.5	Angptl6	3E-02	0.4
Pdcd4	8E-23	0.5	Stxbp6	1E-04	0.4
D3Ertd751e	1E-01	0.5	Sdc4	1E-08	0.4
Pfkl	5E-05	0.5	Sypl	6E-01	0.4
App	1E-69	0.5	Hbegf	4E-02	0.4
Slc7a1	9E-13	0.5	Eps8	3E-02	0.4
Srpx2	8E-21	0.5	Rdm1	2E-01	0.4
Gpx3	9E-06	0.5	Tsr2	1E-02	0.4
Slc1a5	3E-03	0.5	Neurl2	4E-08	0.4
Qrsl1	2E-01	0.5	Epyc	1E-02	0.4
Stard10	2E-02	0.5	Arhgap44	3E-03	0.4
Slc19a2	1E+00	0.5	Acot8	3E-02	0.4
Hmgn5	2E-01	0.5	Nek4	2E-02	0.4
Camk1d	2E-01	0.5	B4galt3	6E-03	0.4
Aff2	2E-12	0.5	Ccdc73	2E-02	0.4
Fzd6	7E-05	0.5	Slc25a38	6E-01	0.4
Rnaset2b	4E-02	0.5	Smim26	2E-01	0.4
Slc7a5	1E-03	0.5	4930430F08Rik	4E-03	0.4
Sox11	2E-03	0.5	Birc2	3E-04	0.4
AY036118	7E-01	0.5	Tmem246	9E-01	0.4
Phyh	1E-15	0.5	Lrrc8c	2E-02	0.4
Dock6	1E+00	0.5	Sdhaf3	2E-02	0.4
Flnb	2E-19	0.5	1500011B03Rik	6E-01	0.4
Oxld1	5E-01	0.5	Lins1	3E-01	0.4
Tiam1	1E-01	0.5	4933431E20Rik	5E-01	0.4

Articular/Synovial Fibroblast					
Control			*$Notch2^{tm1.1Ecan}$*		
Gene	*p*	FC	Gene	*p*	FC
Cpne2	3E-20	1.0	Abcg2	4E-04	0.9
Vwa5a	2E-39	1.0	Fsd1l	4E-05	0.9
Ccng2	2E-09	1.0	Sgsm2	2E-13	0.9
Agtr1a	3E-12	1.0	Arhgap29	1E-37	0.9
Glul	7E-17	1.0	Myadm	2E-25	0.9
Gja1	2E-58	1.0	Trim12a	2E-08	0.9
Sema3f	2E-16	1.0	Dnase2a	2E-13	0.9
Gabarapl1	6E-38	1.0	Trpm3	1E-03	0.9
Sulf2	2E-35	1.0	1110046J04Rik	1E-06	0.9
Tnfaip6	8E-18	1.0	Arhgef10l	5E-18	0.9
Ifitm3	1E-114	1.0	Mmp23	1E-30	0.9
Gyg	1E-29	1.0	Man2a1	2E-22	0.9
S100a6	4E-119	1.0	Rras2	5E-06	0.9
Dlgap4	1E-51	1.0	Tspan14	2E-05	0.9
Ralgps1	2E-11	1.0	Mettl27	2E-06	0.9
Clip1	2E-69	1.0	Nrp1	8E-45	0.9
Zfp820	3E-10	1.0	Trim2	7E-20	0.9
Trnp1	2E-40	1.0	Ahnak	5E-79	0.9
Lgals3bp	4E-28	0.9	Rab3il1	2E-12	0.9
Mmp14	2E-85	0.9	Dapk1	1E-26	0.9
Csf1	5E-56	0.9	Pla2g15	6E-08	0.9
Gm10373	3E-08	0.9	Klhl13	3E-17	0.9
Dock9	1E-25	0.9	Acly	1E-24	0.9
Dsel	9E-33	0.9	Mt2	6E-53	0.8
Kank2	8E-44	0.9	Ece1	5E-15	0.8
Mettl7a1	1E-30	0.9	Prkcg	8E-06	0.8
Coro2b	7E-08	0.9	Trim12c	3E-06	0.8
Gla	2E-14	0.9	H2-D1	9E-33	0.8
Carhsp1	2E-37	0.9	Angptl4	2E-07	0.8
Gm11266	6E-12	0.9	Maoa	4E-17	0.8
Gprc5a	6E-11	0.9	Pard3b	1E-47	0.8
Mmp16	6E-17	0.9	Angptl1	6E-15	0.8
Mt1	3E-108	0.9	Cachd1	4E-19	0.8
Dhcr7	5E-09	0.9	Tsga10	2E-05	0.8
Kctd9	4E-13	0.9	Plcl1	9E-29	0.8
Akr1b8	1E-11	0.9	Kank2	8E-27	0.8
Arhgef10l	5E-32	0.9	Sulf1	2E-17	0.8
Tmod1	9E-08	0.9	Amdhd2	1E-15	0.8
H2-D1	3E-50	0.9	Car13	1E-06	0.8
Myadm	4E-45	0.9	Samd9l	6E-16	0.8
Tmem50b	6E-08	0.9	Zeb1	1E-28	0.8
Fer	8E-45	0.9	Sptbn1	3E-57	0.8
Dmxl2	3E-08	0.9	Ttyh2	3E-10	0.8
Naaa	1E-16	0.9	A330023F24Rik	4E-08	0.8
Casp9	8E-06	0.9	Fat3	2E-01	0.8
Hdac7	3E-25	0.9	Wdr91	2E-05	0.8
9130008F23Rik	2E-05	0.9	Ptgfrn	4E-07	0.8
Rgs17	2E-14	0.9	Olfml1	1E-06	0.8
Bmp2k	4E-26	0.9	C1qtnf1	5E-08	0.8
Cmtm3	2E-37	0.9	Thsd7a	1E-09	0.8
Bst2	1E-33	0.9	Tmem104	4E-02	0.8
Gpc3	2E-21	0.9	Magi2	3E-50	0.8
Mical2	1E-06	0.9	Vsig10	3E-06	0.8
Jph1	3E-15	0.9	Mvk	2E-19	0.8
Lrrc27	2E-10	0.9	Plekhg3	1E-09	0.8
Mmp23	4E-45	0.9	Otud1	1E-08	0.8
Creld1	1E-15	0.9	Osbpl3	4E-06	0.8
Slc43a2	2E-07	0.9	Arnt2	3E-14	0.8
Epdr1	4E-26	0.9	Mt1	3E-78	0.8
Trim2	4E-33	0.9	Map6	1E-16	0.8
Cdkl2	3E-06	0.9	Parp12	3E-04	0.8
Dusp3	9E-33	0.9	Slc41a3	6E-10	0.8
Pbx1	7E-68	0.9	Nln	6E-16	0.8
Taok3	1E-38	0.9	Ugp2	5E-28	0.8

Limb Mesenchyme

Control Gene	p	FC	$Notch2^{tm1.1Ecan}$ Gene	p	FC
Jph2	2E-07	0.5	Lama4	4E-40	0.5
Snhg12	6E-59	0.5	Hpcal1	5E-18	0.5
Cd248	2E-93	0.5	Msrb1	9E-27	0.5
Cyp51	7E-20	0.5	Rnf150	5E-37	0.5
Pparg	9E-11	0.5	Emilin2	3E-20	0.5
Nfil3	5E-12	0.5	Pla2g15	6E-07	0.5
Socs1	7E-07	0.5	Rap2a	1E-17	0.5
Pid1	2E-28	0.5	Mex3b	6E-04	0.5
Arhgef25	3E-28	0.5	Pear1	1E-06	0.5
Adipor2	4E-13	0.5	Fscn1	2E-47	0.5
Gm16201	8E-05	0.5	Kras	2E-29	0.5
Fabp3	2E-15	0.5	Ap2a2	3E-37	0.5
Vcl	1E-26	0.5	Atp2b4	3E-09	0.5
Tom1l1	8E-05	0.5	Rgs10	2E-16	0.5
Gcat	4E-08	0.5	Snhg16	1E-10	0.5
Dact3	1E-10	0.5	Pmm1	5E-30	0.5
Kctd1	1E-20	0.5	Cd44	4E-27	0.5
Mmp11	8E-14	0.5	Hmgcs1	4E-21	0.5
Gm15222	9E-06	0.5	Cyp51	8E-24	0.5
Gpc1	4E-16	0.5	Dnm3	2E-20	0.5
Helb	7E-07	0.5	P2ry10b	1E-11	0.5
Aldh3a2	1E-08	0.5	Adgre5	1E-10	0.5
Prkar2b	2E-14	0.5	Mfhas1	2E-15	0.5
Sertad4	2E-09	0.5	Vegfc	2E-09	0.5
Mvb12b	2E-13	0.5	Traf4	1E-10	0.5
Plec	1E-53	0.5	Sntb1	1E-21	0.5
Grik5	2E-16	0.5	Fmnl2	1E-23	0.5
Efna2	2E-04	0.5	Tmem123	3E-16	0.5
Tent5a	3E-17	0.5	Gxylt2	9E-36	0.5
Adamts6	3E-16	0.5	Sgpp1	1E-12	0.5
Lama4	1E-34	0.5	Nrp1	5E-31	0.5
Dnm3	4E-19	0.5	Fads2	1E-19	0.5
Dsel	4E-14	0.5	Ptprd	9E-25	0.5
Snhg17	1E-04	0.5	Ly6a	2E-46	0.5
Col4a2	5E-25	0.5	Adamtsl4	3E-16	0.5
Tap2	4E-07	0.5	Clmp	2E-30	0.5
Adamts12	2E-22	0.5	Rai14	1E-27	0.5
Clu	7E-09	0.5	Vcl	5E-24	0.5
Sh3bp5	1E-12	0.5	Plpp5	5E-18	0.5
Fgf13	4E-01	0.5	Cd47	2E-55	0.5
Cavin1	6E-62	0.5	Bmp1	5E-40	0.5
Purg	1E-14	0.5	Eif4ebp1	2E-62	0.5
Pth1r	3E-07	0.5	Actn1	2E-55	0.5
Synj2	6E-09	0.5	Homer2	4E-06	0.5
Mpp1	8E-17	0.5	Pdp1	8E-23	0.5
Gpr173	7E-08	0.5	Git1	2E-10	0.5
Sri	8E-53	0.5	Qpctl	3E-04	0.5
Adgrl3	2E-30	0.5	Lipa	2E-27	0.5
Snhg1	2E-48	0.5	Ugcg	5E-15	0.5
Slc9a9	3E-25	0.5	Usb1	2E-05	0.5
Nxn	7E-32	0.5	Bcl2l11	1E-10	0.5
Tle5	4E-94	0.5	Kalrn	1E-08	0.5
Elovl1	2E-20	0.5	Pcsk5	4E-10	0.5
Fam20a	3E-21	0.5	Plec	5E-50	0.5
Hebp1	4E-21	0.5	Insyn1	5E-05	0.5
Stat2	2E-07	0.5	Pfn2	2E-26	0.5
Bdh2	5E-12	0.5	Rgs19	3E-14	0.5
Gadd45g	2E-11	0.5	Mdk	1E-18	0.5
B2m	4E-144	0.5	Tshz2	4E-05	0.5
Pqlc3	2E-10	0.5	Sigmar1	2E-26	0.5
Palmd	2E-10	0.5	Hebp2	8E-13	0.5
Flrt3	9E-05	0.5	Aspn	1E-90	0.5
Igsf10	4E-48	0.5	Gas1	3E-49	0.5
Baiap2	5E-29	0.5	Arhgap29	2E-34	0.5

Chondrogenic

Control Gene	p	FC	$Notch2^{tm1.1Ecan}$ Gene	p	FC
Tspan18	2E-17	0.6	Isg20	2E-27	0.6
Me1	9E-59	0.6	Uba7	8E-05	0.6
Pcsk5	2E-11	0.6	Map3k1	3E-12	0.6
Gm16124	5E-09	0.6	Tmtc2	5E-36	0.6
Gm47271	5E-08	0.6	Tiam2	4E-54	0.6
Ero1l	2E-38	0.6	Got1	2E-24	0.6
Slc35e3	1E-10	0.6	Alg2	3E-15	0.6
Pcgf5	9E-14	0.6	Papss1	3E-38	0.6
Chsy3	3E-29	0.6	Lars	1E-67	0.6
D3Ertd751e	2E-12	0.6	Dus4l	6E-06	0.6
Tenm3	3E-44	0.6	Iqsec1	3E-34	0.6
Cdk17	5E-33	0.6	Tpbg	2E-10	0.6
0610043K17Rik	4E-08	0.6	4930523C07Rik	2E-19	0.6
Gnb4	5E-17	0.6	Crybg3	1E-15	0.6
Thyn1	4E-30	0.6	Tpmt	3E-14	0.6
Frs2	3E-30	0.6	Smim3	2E-12	0.6
Zbtb20	3E-136	0.6	Rtkn	4E-08	0.6
Acaca	4E-48	0.6	Fzd6	8E-12	0.6
Megf9	4E-16	0.6	Thyn1	2E-38	0.6
Nupr1	1E-83	0.6	Islr	3E-54	0.6
4930481A15Rik	4E-07	0.6	A930015D03Rik	5E-13	0.6
Cyb5r1	1E-56	0.6	Ppp1r10	1E-22	0.6
Dixdc1	7E-13	0.6	Kif21a	3E-26	0.6
Srpx2	1E-70	0.6	Gm4221	3E-08	0.6
Skap2	2E-48	0.6	Ero1l	4E-34	0.6
4930523C07Rik	3E-27	0.6	Pts	7E-30	0.6
Acot7	3E-23	0.6	Cyb5r1	2E-62	0.6
A430018G15Rik	1E-04	0.6	Selenos	2E-127	0.6
Nr4a2	9E-20	0.6	Bbs2	2E-04	0.6
Ube2cbp	1E-07	0.6	Slc7a5	6E-37	0.6
Cep126	2E-06	0.6	Phf10	4E-54	0.6
Pim3	9E-19	0.6	Gadd45a	3E-20	0.6
Tmx1	1E-38	0.6	Ccn2	7E-61	0.6
Slc16a1	5E-12	0.6	Mttp	3E-10	0.6
Dipk2a	1E-39	0.6	Adi1	4E-19	0.6
Eef1e1	3E-21	0.6	Stk38l	1E-16	0.6
Ifrd2	4E-09	0.6	Mtmr10	1E-11	0.6
Rhbdd1	4E-27	0.6	Ccdc47	1E-40	0.6
Cmtm8	2E-11	0.6	Edil3	4E-56	0.6
Osbpl6	1E-15	0.6	Gm47271	8E-05	0.6
Mpp6	1E-23	0.6	Aplp2	1E-106	0.6
Unc5c	2E-47	0.6	Nfat5	8E-67	0.6
Tmem246	7E-17	0.6	Fbln7	2E-50	0.6
Per2	1E-12	0.6	Rgmb	2E-11	0.6
Slc1a5	1E-10	0.6	Trp53inp1	1E-23	0.6
Lars	2E-67	0.6	Mtmr3	2E-49	0.6
Samd12	1E-14	0.6	Sesn2	2E-23	0.6
Dok1	7E-11	0.6	Csgalnact1	3E-61	0.6
Hdac9	3E-04	0.6	Shisa2	1E-08	0.6
Mindy4	8E-10	0.6	Enpp5	1E-17	0.6
Znhit6	1E-23	0.6	Ppp1r15a	1E-39	0.6
Iqsec1	1E-28	0.6	Slc7a6os	3E-08	0.6
Taco1	4E-01	0.6	Nfatc1	5E-14	0.6
Asns	1E-64	0.6	Cdk17	2E-25	0.6
Phf10	1E-47	0.6	Cyp4f13	7E-07	0.6
Golph3	2E-48	0.6	Polg2	1E-07	0.6
Mef2c	5E-50	0.6	Nedd4l	5E-20	0.6
Gm16845	6E-07	0.6	Oxr1	1E-24	0.6
Creb3l2	9E-53	0.6	Fam135a	3E-22	0.6
Bcl6	4E-34	0.6	Cyth1	5E-09	0.6
Smim3	2E-11	0.6	Ppip5k2	8E-19	0.6
Lrrc8d	4E-05	0.6	Zbtb18	6E-06	0.6
Selenos	5E-133	0.6	Nupr1	4E-70	0.6
B230118H07Rik	2E-41	0.6	Plscr1	2E-09	0.6

Fibroblast

Control Gene	p	FC	$Notch2^{tm1.1Ecan}$ Gene	p	FC
Tnfrsf12a	3E-23	0.6	Fads2	9E-21	0.7
Sema3c	1E-26	0.6	Nup85	5E-09	0.7
Sgk3	1E-13	0.6	Cntrl	6E-09	0.7
Clip1	3E-39	0.6	Ddx39	9E-13	0.7
Chrdl1	2E-11	0.6	C1galt1	6E-22	0.7
Ldlrad4	5E-45	0.6	Fhl1	4E-36	0.7
Bicd2	4E-22	0.6	Cdk5rap2	2E-16	0.7
Tmtc1	7E-22	0.6	Mettl15	7E-09	0.7
Ccdc68	6E-09	0.6	Cav1	2E-32	0.7
Rfx1	5E-03	0.6	Bcl2l12	7E-12	0.7
Kat2b	5E-12	0.6	Atm	4E-13	0.7
9530026P05Rik	1E-10	0.6	Aaas	3E-06	0.7
Pdlim4	3E-12	0.6	Hist1h1e	4E-09	0.7
Jpt2	2E-10	0.6	Fbxw17	1E-19	0.7
Rasl11a	4E-05	0.6	Kremen1	6E-11	0.7
Aspn	3E-21	0.6	Arhgap22	5E-10	0.7
Tgfb2	3E-38	0.6	Ifit3	5E-06	0.7
1700025G04Rik	2E-11	0.6	Khdrbs3	4E-12	0.7
Fbxw17	1E-16	0.6	Tti2	3E-02	0.7
Junos	5E-02	0.6	Cdo1	8E-37	0.7
Poc1a	6E-07	0.6	Psip1	6E-31	0.7
Gm10125	2E-03	0.6	Ehd4	4E-16	0.7
Arsi	4E-07	0.6	Pcbp3	1E-05	0.7
Gm13470	2E-08	0.6	Crip1	1E-25	0.7
Hpf1	5E-18	0.6	Uhrf2	4E-18	0.7
Endod1	2E-07	0.6	Lefty1	3E-07	0.7
Tagln2	8E-35	0.6	Enkd1	8E-07	0.7
Stard4	5E-07	0.6	Cklf	1E-09	0.7
Rnaseh2c	2E-13	0.6	Sept10	4E-19	0.7
Rfc2	1E-08	0.6	Pdlim4	1E-16	0.7
B3galnt1	2E-06	0.6	Ppil1	3E-17	0.7
Mtr	2E-06	0.6	Sap18b	9E-07	0.6
Ccdc15	1E-05	0.6	Tbx4	9E-16	0.6
Plat	2E-05	0.6	Olfml2a	3E-14	0.6
Phactr1	1E-11	0.6	Slbp	1E-11	0.6
Hist1h1e	1E-06	0.6	Slc1a6	5E-14	0.6
Crip1	4E-10	0.6	Dtymk	7E-29	0.6
Sun2	9E-12	0.6	Srrt	2E-17	0.6
Glrx	8E-08	0.6	Zfp101	2E-10	0.6
Sipa1	8E-06	0.6	Lrrc49	8E-13	0.6
Mylk	6E-04	0.6	Gm43462	1E-04	0.6
Mast2	2E-19	0.6	Syne3	6E-13	0.6
Cspg4	2E-17	0.6	2900026A02Rik	1E-10	0.6
Siah1b	2E-05	0.6	Cep57l1	6E-14	0.6
Cep162	4E-11	0.6	Pknox2	8E-16	0.6
Gab2	4E-13	0.6	Rap1gap2	2E-07	0.6
Fap	7E-05	0.6	Magi3	1E-23	0.6
Tpm4	2E-44	0.6	Robo1	2E-22	0.6
Ccp110	9E-08	0.6	Tubgcp6	3E-09	0.6
Add3	3E-21	0.6	Nup93	6E-15	0.6
Ctc1	4E-08	0.6	Lrrc27	4E-06	0.6
Ywhah	2E-16	0.6	Xpo1	1E-23	0.6
Gjc1	2E-12	0.6	Nup37	1E-08	0.6
Syn3	1E-39	0.6	Zbtb45	7E-04	0.6
Rnf141	7E-05	0.6	Pi15	9E-17	0.6
Csrp1	5E-19	0.6	Notch1	8E-11	0.6
Nup93	4E-09	0.6	Mcm2	5E-07	0.6
Tuba1a	6E-46	0.6	Igf2bp1	5E-07	0.6
Sema3f	2E-05	0.6	Cdc23	2E-08	0.6
Kctd9	9E-11	0.6	S100a6	8E-80	0.6
Gpr137b	3E-05	0.6	Mafb	3E-15	0.6
Mafk	1E-08	0.6	Il1rapl1	2E-22	0.6
Cenpc1	5E-11	0.6	Ncaph2	5E-18	0.6
B3gnt2	4E-13	0.6	Dek	1E-21	0.6

Undefined

Control Gene	p	FC	$Notch2^{tm1.1Ecan}$ Gene	p	FC
Phf10	7E-05	0.5	Sec11c	7E-01	0.4
Pxdc1	1E-04	0.4	Dop1b	3E-01	0.4
Ccar2	7E-01	0.4	Gfpt1	2E-01	0.4
Kctd6	9E-01	0.4	Bcl2l1	1E+00	0.4
Rtkn	5E-02	0.4	Abhd6	4E-01	0.4
Tafa5	1E-06	0.4	Pacsin2	9E-01	0.4
Fbln7	8E-13	0.4	Trabd	7E-05	0.4
Cog8	1E-01	0.4	Emc6	3E-01	0.4
Rsph9	4E-01	0.4	Gramd1b	9E-01	0.4
Rnf167	1E-01	0.4	Mknk1	3E-01	0.4
Bcat1	2E-03	0.4	Barx1	1E-03	0.4
Per3	4E-01	0.4	Plod2	1E-11	0.4
Mtus1	1E-07	0.4	Ppp1r10	1E+00	0.4
Foxd1	1E-03	0.4	Ckb	3E-03	0.4
Ulk4	4E-01	0.4	Atp1b3	7E-01	0.4
Glis1	9E-06	0.4	Gm16124	9E-01	0.4
Polg2	6E-02	0.4	Nsun3	6E-02	0.4
Enpp1	5E-07	0.4	1700084C06Rik	2E-01	0.4
Mindy4	2E-01	0.4	Tgfa	8E-03	0.4
Ncam1	8E-04	0.4	Ctu2	5E-02	0.4
Yju2	5E-01	0.4	Riox2	1E-02	0.4
Meg3	1E-55	0.4	Fmod	6E-09	0.4
Sil1	2E-07	0.4	Coil	1E-02	0.4
Mpp6	5E-03	0.4	Mgp	3E-01	0.4
Myo9b	3E-02	0.4	Fbf1	5E-04	0.4
Gm12811	9E-02	0.4	Fzd6	1E+00	0.4
Firre	3E-01	0.4	Timm8a1	5E-01	0.4
Dnaja3	1E-01	0.4	Shmt2	3E-02	0.4
B4galt3	7E-01	0.4	Prxl2c	1E-01	0.4
Vldlr	6E-04	0.4	Mef2c	3E-01	0.4
Cdkl5	2E-08	0.4	Mkrn2	3E-04	0.4
Pop5	1E-05	0.4	Tiam1	9E-01	0.4
Apmap	1E-02	0.4	Arhgef28	6E-03	0.4
Gramd1b	6E-01	0.4	Bcat1	6E-01	0.4
Cystm1	6E-01	0.4	Pdcd4	8E-04	0.4
Gfpt1	3E-13	0.4	Phf1	7E-01	0.4
Rpgr	4E-01	0.4	Saraf	7E-01	0.4
Tsen54	1E+00	0.4	Rcc2	1E-01	0.4
Tns3	1E-12	0.4	Them4	6E-01	0.4
Lpcat1	3E-04	0.4	Crlf3	8E-02	0.4
Tmem129	2E-01	0.4	Phactr2	3E-01	0.4
Ssx2ip	8E-01	0.4	Aldh1l2	3E-01	0.4
Efnb2	3E-04	0.4	Gm13056	1E-02	0.4
Rab4a	8E-01	0.4	Kantr	1E-01	0.4
Clip4	1E-01	0.4	Slc38a2	1E-01	0.4
Nob1	5E-01	0.4	Cab39	1E-01	0.4
Zbtb20	2E-51	0.4	Rps6ka2	3E-01	0.4
Mef2c	5E-10	0.4	Ccl27a	2E-01	0.4
Id4	2E-10	0.4	Pwp1	2E-05	0.4
4933431E20Rik	3E-03	0.4	Gm20721	5E-04	0.4
Ero1l	1E-02	0.4	Pycr1	8E-01	0.4
Fam131a	7E-01	0.4	Sox5	2E-14	0.4
Gm19705	2E-01	0.4	Enah	8E-02	0.4
Mlh1	6E-01	0.4	Adgrg6	2E-02	0.4
B230118H07Rik	4E-06	0.4	Psd3	3E-02	0.4
Zfp87	1E-01	0.4	Flnb	4E-03	0.4
Ppip5k2	8E-03	0.4	Pus3	8E-01	0.4
Spata1	8E-05	0.4	Dnaja3	8E-01	0.4
Enah	5E-07	0.4	Snai1	1E+00	0.4
Isy1	9E-01	0.4	Pcdh11x	4E-03	0.4
Slc19a1	7E-01	0.4	Jpx	3E-01	0.4
Hoxd9	4E-02	0.4	Ogfod3	4E-01	0.4
Mir22hg	8E-03	0.4	Acbd4	4E-03	0.4
Hist1h4d	4E-02	0.4	Yod1	6E-02	0.4

Articular/Synovial Fibroblast

Control Gene	p	FC	$Notch2^{tm1.1Ecan}$ Gene	p	FC
Mrgbp	4E-16	0.9	Nudt4	3E-34	0.8
Ccn5	1E-47	0.9	Apoe	3E-12	0.8
Phka2	8E-13	0.9	Il17rd	3E-05	0.8
Vsig10	3E-06	0.9	Clcn6	2E-07	0.8
Pkia	1E-16	0.9	Adra1b	2E-03	0.8
Fads2	2E-20	0.9	Wdr81	1E-09	0.8
Smpdl3a	2E-57	0.9	Lifr	3E-05	0.8
Dennd2a	1E-17	0.9	Ehd2	1E-37	0.8
Ndufa4l2	7E-18	0.9	Cped1	4E-57	0.8
Adamtsl4	6E-24	0.9	Lamc1	2E-28	0.8
Zdhhc3	6E-44	0.9	Dag1	8E-44	0.8
Thsd7a	2E-14	0.9	Pbx1	2E-52	0.8
Lgmn	1E-50	0.9	Mvp	9E-15	0.8
Irgm1	2E-05	0.9	Adgrg2	5E-12	0.8
Fnip2	5E-34	0.9	Syne3	2E-06	0.8
Dtx3l	8E-11	0.9	Slc22a18	4E-09	0.8
Cdo1	8E-29	0.9	Adam23	3E-09	0.8
Sgsm2	1E-21	0.9	Gm20707	7E-06	0.8
Dag1	7E-87	0.9	Gem	4E-13	0.8
Erg28	8E-40	0.9	Daam1	4E-14	0.8
Zfp36l1	4E-40	0.9	Rassf8	3E-23	0.8
Npr3	3E-17	0.9	Gyg	4E-19	0.8
Grk5	5E-24	0.9	Mrgbp	1E-06	0.8
Lbp	7E-20	0.9	Dync1i1	3E-02	0.8
Pik3ip1	3E-22	0.9	Renbp	8E-15	0.8
Lpin3	4E-08	0.9	Adamts2	4E-21	0.8
Slc1a6	5E-10	0.9	Gamt	4E-20	0.8
Tstd3	3E-09	0.9	Elovl6	9E-11	0.8
Plscr4	5E-10	0.9	Cdon	9E-35	0.8
Fyn	5E-27	0.9	Marcks	5E-76	0.8
Hnrnpll	2E-21	0.9	Jcad	2E-10	0.8
Nmnat2	2E-06	0.9	Prkd1	6E-17	0.8
Gem	3E-11	0.9	Pcdh18	2E-17	0.8
Col4a1	1E-18	0.9	Gm14296	4E-02	0.8
Ppm1l	2E-13	0.9	Mical2	4E-07	0.8
Ano3	9E-05	0.9	Sytl2	4E-05	0.8
Sbno2	2E-17	0.9	Hebp1	2E-17	0.8
Stard4	2E-10	0.9	Tmem43	3E-08	0.8
Cyb5a	2E-89	0.9	Snn	7E-08	0.8
Car13	2E-07	0.9	Colec12	5E-48	0.8
Adamts2	2E-38	0.9	Col4a2	3E-21	0.8
Ugp2	9E-42	0.9	Gdpd1	2E-09	0.8
Pim1	5E-09	0.9	Col18a1	7E-29	0.8
Mgst3	3E-25	0.9	Kctd12	3E-21	0.8
Csnk1e	3E-35	0.9	Pced1b	8E-03	0.8
Podxl2	5E-22	0.9	Lamb2	2E-20	0.8
Fam20c	9E-13	0.9	Hook2	1E-02	0.8
Ino80c	2E-08	0.9	Retreg1	6E-08	0.8
Prickle3	2E-04	0.9	Sc5d	2E-16	0.8
Tln2	3E-35	0.9	Vwa5a	7E-18	0.8
Sc5d	4E-31	0.8	Gpc3	1E-08	0.8
Man1c1	1E-27	0.8	Raet1e	4E-04	0.8
Tapbpl	2E-02	0.8	Rnf213	9E-07	0.8
C1galt1	7E-28	0.8	Ppp1r9a	4E-11	0.8
Trim47	2E-24	0.8	Plcl2	5E-06	0.8
Hebp2	6E-25	0.8	Synj2	2E-08	0.8
Mn1	3E-17	0.8	Aacs	1E-13	0.8
Apbb1ip	1E-12	0.8	Lacc1	2E-04	0.8
Ap3m2	1E-05	0.8	Cyp51	5E-22	0.8
Ahnak	7E-119	0.8	Dennd2a	6E-08	0.8
Crppa	2E-11	0.8	Large1	1E-43	0.8
Bmp1	5E-37	0.8	AI413582	1E-21	0.8
Anpep	1E-08	0.8	Peli2	3E-07	0.8
Gprc5b	4E-08	0.8	Uap1l1	6E-39	0.8

Limb Mesenchyme

Control Gene	*p*	FC	*Notch2*[tm1.1Ecan] Gene	*p*	FC
Apobr	2E-07	0.5	Zfp449	5E-06	0.5
Fbln1	4E-19	0.5	Ahnak2	2E-59	0.5
Dpy19l1	4E-07	0.5	Blcap	7E-14	0.5
Wipi1	3E-40	0.5	Tspo	1E-45	0.5
Idh1	3E-40	0.5	Mtch1	6E-62	0.5
Nlgn2	2E-08	0.5	Map4k3	1E-29	0.5
Foxp1	5E-91	0.5	Mkx	6E-09	0.5
Blvrb	4E-20	0.5	Gpr173	3E-09	0.5
Plscr3	2E-20	0.5	Bace2	9E-07	0.5
Slc27a3	1E-09	0.5	Dhcr7	3E-04	0.5
Hpcal1	4E-10	0.5	Nfil3	8E-16	0.5
Dennd4a	5E-29	0.5	Fgf2	7E-08	0.5
Mecom	2E-13	0.5	Snx16	7E-09	0.4
Tln2	3E-36	0.5	Sema3f	3E-10	0.4
Efnb1	1E-08	0.5	Lox	2E-74	0.4
Klf2	2E-47	0.5	Bcl11a	8E-08	0.4
Castor1	6E-12	0.5	Asap1	2E-54	0.4
Cers6	9E-19	0.5	Snx9	4E-41	0.4
Ifi27l2a	1E-16	0.5	Gatd1	3E-18	0.4
Tmeff1	1E-12	0.4	Fdft1	4E-18	0.4
Gatb	4E-09	0.4	Lasp1	4E-23	0.4
Fads2	7E-13	0.4	Ephb4	1E-05	0.4
Sertad3	1E-09	0.4	Prag1	5E-09	0.4
Psat1	4E-15	0.4	Ehd1	8E-28	0.4
Fdps	2E-25	0.4	Rasal2	1E-23	0.4
Slc16a7	4E-05	0.4	Hdac4	4E-13	0.4
L3hypdh	2E-09	0.4	Klf2	1E-41	0.4
Plekhf1	2E-14	0.4	Efs	3E-15	0.4
Igf2bp1	1E-06	0.4	Ebp	4E-29	0.4
Lama2	4E-24	0.4	Mvp	2E-19	0.4
Egr2	2E-03	0.4	Cad	1E-14	0.4
Prelp	1E-18	0.4	Mcat	4E-08	0.4
Clmp	2E-34	0.4	Chst14	1E-13	0.4
Pold4	4E-33	0.4	Akt1	2E-35	0.4
Ehd1	1E-23	0.4	Iah1	2E-30	0.4
Arhgap24	1E-28	0.4	Vat1	8E-46	0.4
Dhx33	2E-03	0.4	Tle5	2E-68	0.4
Esyt1	6E-09	0.4	Msmo1	3E-12	0.4
Plxdc2	2E-64	0.4	Mob3a	3E-08	0.4
Flot1	3E-26	0.4	Adipor2	2E-14	0.4
Pknox2	9E-12	0.4	B2m	4E-85	0.4
Gfra4	5E-05	0.4	Plat	2E-11	0.4
Porcn	7E-10	0.4	Gnao1	7E-08	0.4
Plcb3	1E-09	0.4	Eif3e	2E-76	0.4
Trnp1	2E-23	0.4	Cav1	1E-34	0.4
Pdp1	4E-21	0.4	Eya1	2E-12	0.4
Elovl6	2E-16	0.4	Cib1	3E-20	0.4
Ust	9E-20	0.4	Ahnak	5E-75	0.4
Fads3	1E-19	0.4	Efemp2	5E-60	0.4
Palm	7E-12	0.4	B4galt5	2E-06	0.4
Hs3st3a1	4E-07	0.4	Plcb3	6E-08	0.4
Ttc39c	1E-07	0.4	Renbp	2E-19	0.4
Clec11a	3E-23	0.4	Fat4	9E-37	0.4
Hmgcs1	2E-14	0.4	Phlda3	7E-48	0.4
Wipf1	3E-20	0.4	Pmvk	9E-13	0.4
Plekhg2	9E-08	0.4	Samd9l	5E-14	0.4
Tmem168	3E-13	0.4	Gucy1a2	9E-12	0.4
Ifi35	1E-08	0.4	Zfhx3	4E-39	0.4
Sigmar1	1E-24	0.4	Adgrl3	5E-28	0.4
Gsto1	2E-26	0.4	Prelp	3E-33	0.4
Ctsk	1E-80	0.4	Ahr	5E-15	0.4
Galns	9E-05	0.4	Cemip2	2E-20	0.4
Calml4	3E-06	0.4	Dbn1	3E-23	0.4
Arap2	1E-03	0.4	Arhgef25	1E-31	0.4

Chondrogenic

Control Gene	*p*	FC	*Notch2*[tm1.1Ecan] Gene	*p*	FC
Crybg3	3E-10	0.6	Igdcc4	2E-17	0.6
Ttll7	2E-10	0.6	Zbtb46	7E-08	0.6
Ppp1r9a	1E-10	0.6	Mmp28	1E-05	0.6
Fam20b	2E-16	0.6	Hotairm1	1E-11	0.6
Rwdd3	2E-04	0.6	Pdgfrl	1E-05	0.6
Tead4	9E-09	0.6	Rin2	2E-25	0.6
Anxa11	3E-14	0.6	Gm4258	3E-22	0.6
Rrs1	8E-22	0.6	Plscr2	1E-10	0.6
1700023F06Rik	5E-08	0.6	Bcl6	2E-28	0.6
Agpat5	9E-21	0.6	Arid5a	3E-08	0.6
Stk39	3E-29	0.6	Asns	3E-66	0.6
Cox6a2	3E-06	0.6	Gm10516	1E-07	0.6
Ctsh	5E-43	0.6	Taf15	7E-43	0.6
Zfr2	1E-12	0.6	BC049715	6E-06	0.6
Txndc11	2E-13	0.6	Txndc11	7E-14	0.6
E030030I06Rik	1E-08	0.6	Osbpl6	9E-14	0.6
Ctf1	4E-15	0.5	Agpat5	2E-31	0.6
Haus8	2E-15	0.5	Eno1	1E-83	0.6
Nedd4l	5E-24	0.5	Trib1	2E-13	0.6
Ppip5k2	4E-13	0.5	Mtus2	2E-10	0.6
Ooep	3E-16	0.5	Efna5	1E-62	0.6
Ecrg4	5E-71	0.5	Vill	9E-08	0.6
Gm10516	6E-05	0.5	Nat8f1	5E-05	0.6
Sft2d2	1E-15	0.5	Rnaset2b	2E-10	0.6
Ddit4	1E-08	0.5	Cfap74	1E-08	0.6
Fam135a	2E-30	0.5	Kcnq1ot1	6E-50	0.6
Zbtb7c	1E-21	0.5	Usp24	4E-26	0.6
Yrdc	2E-23	0.5	Jph1	6E-10	0.6
Rps6ka1	3E-06	0.5	Spa17	2E-05	0.6
Slc35d1	5E-13	0.5	Wdr35	9E-24	0.6
Pacrg	1E-05	0.5	Dnajb9	6E-21	0.6
Ell2	1E-61	0.5	0610043K17Rik	5E-07	0.6
Mcu	1E-30	0.5	Tpd52	2E-03	0.6
Foxc1	4E-61	0.5	Eml6	3E-08	0.5
Lyrm4	4E-12	0.5	Platr22	1E-05	0.5
Flrt1	1E-03	0.5	Ctsd	3E-130	0.5
Sorl1	2E-13	0.5	Mindy4	7E-09	0.5
Gm14966	1E-06	0.5	Hadh	3E-34	0.5
D130020L05Rik	2E-04	0.5	Ric3	7E-08	0.5
Tiam2	2E-55	0.5	Prxl2c	1E-28	0.5
Mlh3	5E-09	0.5	Ing2	1E-13	0.5
Fap	8E-05	0.5	Sfxn2	7E-06	0.5
Them4	3E-07	0.5	Zhx2	5E-09	0.5
4933431E20Rik	2E-08	0.5	Chsy1	2E-27	0.5
Jade2	2E-08	0.5	E030030I06Rik	7E-08	0.5
Pam16	2E-46	0.5	1110002L01Rik	2E-07	0.5
Igf2r	2E-62	0.5	Id4	1E-19	0.5
Whrn	6E-04	0.5	Igf2r	6E-64	0.5
Brd3os	5E-06	0.5	Slc16a4	6E-11	0.5
Nudt14	1E-08	0.5	Unc5c	1E-35	0.5
Ppat	2E-08	0.5	Fam110a	3E-08	0.5
Sdc2	4E-28	0.5	Camk1d	1E-18	0.5
Gm19705	2E-06	0.5	Myo1d	1E-41	0.5
Map3k1	2E-09	0.5	Rgs7bp	2E-03	0.5
Myo1b	3E-08	0.5	Plcb1	2E-57	0.5
Aff2	1E-31	0.5	Creb3l2	1E-46	0.5
Gas5	3E-117	0.5	Pdss2	3E-19	0.5
Gm11146	2E-07	0.5	Adamts12	8E-01	0.5
Taf13	1E-13	0.5	Aff2	3E-27	0.5
Mttp	2E-06	0.5	Ctf1	1E-11	0.5
Pdzrn3	2E-71	0.5	6330418K02Rik	2E-03	0.5
Spred1	7E-33	0.5	Med22	3E-05	0.5
Tnrc6b	2E-76	0.5	AW146154	1E-03	0.5
2810410L24Rik	7E-05	0.5	Rrs1	3E-16	0.5

Fibroblast

Control Gene	*p*	FC	*Notch2*[tm1.1Ecan] Gene	*p*	FC
Zeb1	2E-26	0.6	Pde3b	7E-18	0.6
Ppfibp1	1E-33	0.6	Wdr47	3E-08	0.6
B130055M24Rik	3E-05	0.6	Cables1	3E-09	0.6
Ccdc82	1E-08	0.6	Cep83os	1E-03	0.6
Nrip1	3E-17	0.6	Add3	2E-25	0.6
Carhsp1	2E-15	0.6	Parp9	5E-08	0.6
Csrp2	2E-25	0.6	Csel1	7E-18	0.6
Eri2	1E-05	0.6	Ripk3	2E-12	0.6
Mdga1	1E-07	0.6	Nalcn	1E-06	0.6
Rorc	8E-06	0.6	Top3a	2E-05	0.6
Cdr1os	5E-05	0.6	1500009L16Rik	2E-13	0.6
Pold2	1E-08	0.6	Ampd3	1E-05	0.6
Ncaph2	3E-12	0.6	Ndufa4l2	1E-24	0.6
Lrrc40	3E-06	0.6	Cap2	2E-04	0.6
Nup85	1E-07	0.6	Mre11a	4E-06	0.6
Cklf	2E-05	0.6	Hacd4	4E-28	0.6
Orc6	4E-10	0.6	Erg	1E-28	0.6
Cgnl1	2E-23	0.6	2700049A03Rik	1E-10	0.6
Chek2	6E-05	0.6	Disp1	2E-18	0.6
Svep1	2E-45	0.6	Ccdc82	2E-08	0.6
Osbpl3	5E-05	0.5	Ifit2	3E-09	0.6
Myom1	1E-05	0.5	Efs	9E-05	0.6
Rbm43	2E-06	0.5	Klhdc8a	3E-16	0.6
Raly	2E-42	0.5	Slx4	5E-07	0.6
Axl	7E-14	0.5	Atxn1l	3E-05	0.6
Csel1	2E-11	0.5	Ift80	4E-16	0.6
Mdc1	2E-09	0.5	Trabd2b	2E-30	0.6
Ccdc138	7E-08	0.5	Arsi	2E-06	0.6
Plxna4	8E-02	0.5	5730522E02Rik	3E-05	0.6
Rassf7	1E-05	0.5	Rgs12	1E-09	0.6
Gm13483	2E-08	0.5	Nuak2	1E-10	0.6
Cyp39a1	3E-05	0.5	Sord	5E-11	0.6
Nin	2E-08	0.5	Odf2l	8E-07	0.6
Ppm1d	7E-10	0.5	Usp13	5E-07	0.6
Hist1h1d	2E-07	0.5	Lpin3	1E-04	0.6
Atp10a	7E-17	0.5	Cenpc1	7E-13	0.6
Dmd	6E-28	0.5	Vgll3	2E-34	0.6
Snrpa1	6E-15	0.5	Fam110a	4E-07	0.6
Col3a1	2E-79	0.5	Fdps	3E-26	0.6
Exosc8	3E-06	0.5	Slc35b4	3E-09	0.6
Dtymk	2E-12	0.5	Carhsp1	9E-21	0.6
Tgfbr2	4E-29	0.5	Dynlt1f	3E-10	0.6
Hacd4	1E-23	0.5	Ino80c	2E-05	0.6
Dhx32	2E-10	0.5	F2r	4E-08	0.6
Nup160	2E-05	0.5	Spidr	2E-11	0.6
Nedd1	1E-07	0.5	Ptpn3	2E-04	0.6
Lefty1	2E-04	0.5	Pomt1	3E-07	0.6
Mphosph9	3E-05	0.5	Mdga1	4E-09	0.6
Armt1	3E-04	0.5	9330159M07Rik	2E-04	0.6
Aff3	1E-37	0.5	Abca9	1E-04	0.6
Mthfd1	1E-09	0.5	Fut10	2E-07	0.6
Hipk2	8E-21	0.5	Ccdc138	3E-09	0.6
Fancm	4E-05	0.5	Gltp	6E-15	0.6
Dlc1	6E-67	0.5	4933406I18Rik	4E-12	0.6
Ell2	3E-29	0.5	Tex9	7E-06	0.6
Mef2a	2E-30	0.5	Uchl5	1E-12	0.6
Taf5	1E-04	0.5	Eya4	5E-40	0.6
Sap18	4E-27	0.5	Zbtb8a	8E-06	0.6
Rtel1	2E-05	0.5	Pot1b	3E-07	0.6
Hmgb1	1E-46	0.5	Pdik1l	8E-05	0.6
Dnajc27	1E-02	0.5	Gm10125	1E-02	0.6
Mre11a	5E-05	0.5	Clip4	8E-12	0.6
Myh10	3E-11	0.5	Msh6	2E-04	0.6
Zdhhc15	6E-05	0.5	Itgbl1	3E-35	0.6

Undefined

Control Gene	*p*	FC	*Notch2*[tm1.1Ecan] Gene	*p*	FC
Sypl	9E-09	0.4	Tns2	9E-01	0.4
Otud1	2E-01	0.4	Rgcc	1E-03	0.4
D930016D06Rik	9E-02	0.4	Adat1	8E-01	0.4
Gsdme	8E-01	0.4	Gm5617	3E-01	0.4
Harbi1	3E-01	0.4	Pigf	6E-02	0.4
Ahcyl2	1E-05	0.4	Ing2	4E-01	0.4
Epb41l4b	5E-02	0.4	Tnfrsf10b	4E-01	0.4
Cdk17	4E-05	0.4	Dolk	4E-03	0.4
Tmem238	7E-03	0.4	Gt(ROSA)26Sor	9E-01	0.4
Idua	3E-01	0.4	Smim12	2E-07	0.4
Tinagl1	4E-02	0.4	3110056K07Rik	4E-02	0.4
B230217C12Rik	2E-01	0.4	Bscl2	8E-01	0.4
Rian	2E-13	0.4	Dixdc1	2E-01	0.4
Daglb	8E-01	0.4	Atr	3E-02	0.4
Nfatc1	2E-03	0.4	Rbbp9	3E-02	0.4
Slc25a33	1E-02	0.4	Pard6a	5E-03	0.4
Zw10	8E-01	0.4	Ccdc57	4E-02	0.4
Enho	7E-05	0.4	Ank	2E-01	0.4
Zfp932	4E-01	0.4	Emc1	7E-05	0.4
Rbm45	9E-01	0.4	Rhbdd1	2E-01	0.4
Sp4	9E-01	0.4	Gin1	2E-03	0.4
Trp53cor1	1E-03	0.4	Sat1	6E-01	0.4
Mbnl3	1E-01	0.4	Gale	1E-02	0.4
Pde4b	2E-07	0.4	Dbi	3E-21	0.4
Pigf	9E-01	0.4	Sgk3	1E-01	0.4
Asrgl1	5E-03	0.4	Ears2	8E-01	0.4
Sat1	5E-06	0.4	Ciao3	1E-03	0.4
Gstm5	4E-09	0.4	Tgoln1	4E-01	0.4
Celf4	1E-01	0.4	Nol9	5E-05	0.4
Mknk1	5E-02	0.4	Adck1	3E-03	0.4
Xpr1	3E-02	0.4	Ube2g2	2E-03	0.4
Ogfod3	1E-04	0.4	Acad10	8E-01	0.4
Pycr1	5E-01	0.4	Imp3	8E-01	0.4
Adpgk	5E-01	0.4	Kbtbd4	8E-04	0.4
Sec14l1	7E-05	0.4	Pde3a	4E-02	0.4
Tmem17	3E-01	0.4	Sod2	6E-01	0.4
Gas5	3E-45	0.4	Krr1	3E-01	0.4
Mtmr12	3E-04	0.4	Col24a1	2E-04	0.4
Smim26	5E-01	0.4	Ppib	3E-42	0.4
Osbpl6	6E-03	0.4	Vps11	2E-02	0.4
Ldhb	9E-05	0.4	Il1rapl1	2E-04	0.4
Cdv3	1E-10	0.4	Bcl6	2E-02	0.4
Mgp	5E-02	0.4	Rmdn3	1E-02	0.4
Mterf2	2E-01	0.4	Mipol1	2E-01	0.4
Rgmb	5E-02	0.4	Mob1b	3E-08	0.4
Mettl5	7E-02	0.4	Sntg2	6E-01	0.4
Fam241a	1E-02	0.4	Hyou1	7E-01	0.4
Ddit3	4E-03	0.4	Fign	7E-03	0.4
Iqsec1	2E-05	0.4	Fgfr1	1E-02	0.4
Snhg9	9E-01	0.4	Marf1	3E-05	0.4
Spred1	1E-04	0.4	Arhgef1	3E-01	0.4
Ninl	1E+00	0.4	Tbc1d7	2E-01	0.4
Mageh1	3E-04	0.4	Zfp958	1E-04	0.4
Gm20721	9E-01	0.4	Rpf1	5E-06	0.4
6330418K02Rik	3E-01	0.4	1110038B12Rik	3E-02	0.4
Slc25a38	4E-03	0.4	Mlh3	1E-01	0.4
Ebna1bp2	6E-03	0.4	Selenos	9E-16	0.4
Kti12	5E-01	0.4	Lysmd4	1E-03	0.4
Ctnnal1	1E-04	0.4	Gng7	1E-01	0.4
C530008M17Rik	4E-02	0.4	Sertad3	2E-07	0.4
Twnk	9E-01	0.4	Ptrhd1	4E-03	0.4
Loxl3	7E-02	0.4	Ppp1r13b	2E-02	0.4
Agrn	3E-01	0.4	Mrto4	7E-04	0.4
Cryl1	9E-04	0.4	Morn2	5E-01	0.4

Articular/Synovial Fibroblast

Control Gene	*p*	FC	*Notch2*[tm1.1Ecan] Gene	*p*	FC
H2-T23	9E-12	0.8	Nampt	9E-16	0.8
Limk1	9E-06	0.8	Rdh11	2E-08	0.8
Fabp3	5E-14	0.8	Pdgfrb	9E-21	0.8
Sytl2	9E-10	0.8	Rfx5	1E-04	0.8
Gm44148	5E-08	0.8	Supt7l	1E-02	0.8
Gm12216	3E-05	0.8	Lgr4	2E-09	0.8
Tbc1d2b	1E-17	0.8	Mllt6	8E-03	0.8
Htra3	2E-12	0.8	Bmp4	2E-12	0.8
Sptbn1	2E-97	0.8	Nudt18	1E-05	0.8
Rab11fip5	7E-19	0.8	Acap3	2E-07	0.7
F2r	2E-05	0.8	Creld1	5E-11	0.7
Cyp51	1E-38	0.8	Agfg2	7E-08	0.7
Ttyh2	2E-13	0.8	Bid	4E-03	0.7
Il17rc	3E-17	0.8	Ccng2	1E-04	0.7
Zfp874b	1E-05	0.8	St3gal2	2E-15	0.7
Runx1t1	7E-75	0.8	Fads1	2E-12	0.7
Fzd4	3E-13	0.8	Sulf2	6E-17	0.7
Prkag2	6E-16	0.8	Vstm4	1E-03	0.7
Rab6b	5E-12	0.8	Mmp2	3E-28	0.7
Tnks1bp1	4E-29	0.8	Lamb1	5E-11	0.7
Aldh3a2	4E-06	0.8	Bmp1	4E-27	0.7
Srebf2	6E-32	0.8	Dtx2	2E-10	0.7
Arhgap21	3E-35	0.8	Rdh5	2E-05	0.7
Lpl	3E-36	0.8	Lrsam1	5E-05	0.7
Aldh3b1	6E-10	0.8	Tlr2	7E-05	0.7
Zeb1	4E-51	0.8	Gba	6E-13	0.7
Samd9l	1E-19	0.8	Bicd1	1E-05	0.7
Thy1	5E-05	0.8	Dhcr7	9E-06	0.7
Klhl13	5E-19	0.8	Tacc1	5E-21	0.7
Dagla	2E-03	0.8	Tbc1d9	2E-04	0.7
Npc2	1E-116	0.8	Cyb5a	1E-50	0.7
Bid	4E-07	0.8	Slc25a23	4E-03	0.7
Adam15	1E-37	0.8	Ncald	4E-04	0.7
Nhsl1	2E-06	0.8	Smpdl3a	1E-21	0.7
1500015L24Rik	8E-24	0.8	Lsp1	3E-34	0.7
Sh2d4a	2E-14	0.8	Man2b1	2E-22	0.7
Mknk2	5E-14	0.8	Cbr3	1E-09	0.7
Adgrg2	8E-25	0.8	Tgfbr3	1E-23	0.7
Abca3	2E-05	0.8	Pid1	6E-11	0.7
Ccdc74a	5E-06	0.8	S100a16	1E-24	0.7
Gpd1l	3E-03	0.8	Lrrc8b	2E-03	0.7
Snn	1E-07	0.8	Txnrd3	1E-07	0.7
1110046J04Rik	1E-09	0.8	Atg7	2E-11	0.7
Gm13470	3E-08	0.8	Mpv17	1E-12	0.7
Atg7	3E-13	0.8	Diaph1	3E-18	0.7
Tspan5	2E-32	0.8	Ino80c	5E-06	0.7
Sncaip	4E-06	0.8	Cgnl1	6E-22	0.7
Pcsk6	3E-29	0.8	Trerf1	1E-18	0.7
Cmtm8	1E-05	0.8	Dusp10	2E-05	0.7
Slc38a4	2E-04	0.8	Csnk1e	1E-18	0.7
Apoe	3E-23	0.8	Hsd17b11	3E-08	0.7
Arap1	3E-08	0.8	Serpine1	8E-09	0.7
Phldb1	9E-19	0.8	Pdlim2	5E-33	0.7
Idh1	3E-50	0.8	Nr3c2	3E-06	0.7
Pced1b	1E-05	0.8	Tspan17	1E-14	0.7
Psrc1	5E-06	0.8	Podxl2	2E-14	0.7
Magi2	3E-70	0.8	Nagpa	1E-04	0.7
Arhgap29	5E-40	0.8	Lynx1	6E-07	0.7
Pdlim1	4E-11	0.8	Ebf3	1E-18	0.7
Arhgap44	2E-08	0.8	Zfp36l1	9E-22	0.7
Emc2	7E-53	0.8	Arsa	2E-11	0.7
Foxo1	1E-25	0.8	Aspn	8E-28	0.7
Colec12	5E-66	0.8	Twf2	7E-11	0.7
Atp6v0e2	4E-06	0.8	Pcsk6	2E-12	0.7

Limb Mesenchyme

Control			$Notch2^{tm1.1Ecan}$		
Gene	*p*	FC	Gene	*p*	FC
Traf5	3E-07	0.4	Nfkbia	2E-16	0.4
Mycbp2	1E-17	0.4	Asap3	6E-08	0.4
Psrc1	1E-03	0.4	Palm	1E-18	0.4
Csrp1	2E-14	0.4	Six1	5E-18	0.4
Ctso	5E-07	0.4	Ctsk	4E-57	0.4
Hccs	2E-09	0.4	Apba3	6E-09	0.4
Tpm1	2E-49	0.4	Rnd3	6E-42	0.4
Eif4ebp1	7E-53	0.4	Ptges2	3E-08	0.4
Fkbp7	1E-51	0.4	Mif4gd	5E-13	0.4
Cpeb2	1E-05	0.4	Ccdc12	1E-24	0.4
Ccnd1	1E-41	0.4	Ass1	1E-12	0.4
Trim12c	4E-05	0.4	A4galt	5E-07	0.4
Gyg	8E-20	0.4	Tpst2	3E-17	0.4
Sema3a	3E-09	0.4	Foxp1	3E-61	0.4
Slc38a10	3E-24	0.4	Magi1	1E-17	0.4
Hsd17b7	8E-07	0.4	Plxdc2	6E-51	0.4
Ror1	2E-20	0.4	Grik5	3E-11	0.4
Ccn4	3E-13	0.4	AI413582	3E-16	0.4
Cavin3	8E-64	0.4	Nkd1	5E-13	0.4
Emilin2	7E-13	0.4	Rab21	2E-32	0.4
Amdhd2	2E-12	0.4	Castor1	1E-10	0.4
Nme4	3E-13	0.4	Map7d1	1E-28	0.4
Srxn1	8E-05	0.4	S100a10	2E-71	0.4
Itga2b	4E-04	0.4	Ppp1r2	4E-18	0.4
Lmo1	2E-11	0.4	Kctd10	1E-15	0.4
Lxn	4E-19	0.4	2410006H16Rik	1E-57	0.4
Twist1	9E-44	0.4	Mycbp2	6E-19	0.4
Pcdh7	3E-45	0.4	Srebf2	1E-21	0.4
Txnip	4E-22	0.4	Sri	4E-43	0.4
Ier3	3E-15	0.4	Thrb	4E-11	0.4
Tmem123	1E-08	0.4	Prkab2	1E-07	0.4
Mfhas1	3E-12	0.4	Rbms2	3E-31	0.4
Rap2b	2E-10	0.4	Snhg1	1E-36	0.4
Rnf227	2E-02	0.4	Dmpk	2E-09	0.4
Sirt6	5E-04	0.4	Ten1	2E-12	0.4
Laptm4b	3E-09	0.4	Aldh3b1	4E-09	0.4
Glis2	1E-11	0.4	L3hypdh	4E-11	0.4
Col16a1	9E-09	0.4	Mest	3E-44	0.4
Ric8a	4E-11	0.4	Garem1	8E-11	0.4
Lasp1	3E-17	0.4	Tmem168	4E-12	0.4
Trim46	9E-05	0.4	Dpy19l1	1E-09	0.4
Mvd	2E-07	0.4	Tead1	8E-43	0.4
Slc50a1	2E-39	0.4	Igfbp7	3E-51	0.4
Cln6	7E-05	0.4	Jph2	2E-08	0.4
1700123M08Rik	1E-04	0.4	Abracl	5E-45	0.4
Gucy1a2	3E-09	0.4	Ccdc85b	1E-19	0.4
Ltbp4	1E-03	0.4	S100a11	7E-95	0.4
Zcchc24	4E-27	0.4	B230307C23Rik	5E-08	0.4
Rai14	1E-25	0.4	Tmem41a	7E-05	0.4
Rac3	6E-10	0.4	Tent5a	2E-19	0.4
Pdlim7	2E-20	0.4	Frrs1	9E-12	0.4
Arsj	6E-21	0.4	Prkcd	3E-07	0.4
Prkcd	3E-09	0.4	Zfhx4	6E-36	0.4
Svil	3E-34	0.4	Hebp1	2E-15	0.4
Gm9844	1E-04	0.4	1110012L19Rik	2E-14	0.4
2610002M06Rik	2E-04	0.4	Plekho2	9E-11	0.4
Wrn	9E-11	0.4	Pknox2	2E-12	0.4
Snn	1E-05	0.4	Tead2	7E-12	0.4
Stk17b	2E-10	0.4	Tbl2	1E-04	0.4
Ncs1	6E-09	0.4	Slc38a10	2E-26	0.4
Aprt	7E-59	0.4	Cers6	5E-23	0.4
Map2	4E-08	0.4	Blvrb	2E-16	0.4
Wscd2	3E-04	0.4	Il11ra1	7E-32	0.4
Dmpk	3E-12	0.4	Sh3bgrl3	9E-74	0.4

Chondrogenic

Control			$Notch2^{tm1.1Ecan}$		
Gene	*p*	FC	Gene	*p*	FC
Ppm1k	3E-04	0.5	Tmem129	2E-04	0.5
Efna5	1E-57	0.5	Ptch1	8E-08	0.5
Pdss2	5E-24	0.5	Mmp16	9E-28	0.5
Zhx2	1E-13	0.5	Cbx4	1E-09	0.5
Csgalnact1	6E-57	0.5	Tsacc	3E-08	0.5
Camk1d	1E-18	0.5	1600002K03Rik	6E-14	0.5
Taf15	4E-45	0.5	Wfs1	3E-09	0.5
Apmap	5E-13	0.5	Cst6	7E-12	0.5
Fzd6	3E-13	0.5	Flvcr1	4E-03	0.5
Il1rapl1	9E-55	0.5	Morn4	2E-02	0.5
Celf4	7E-12	0.5	Slc39a11	9E-11	0.5
Dnaja3	6E-17	0.5	Ccdc115	6E-15	0.5
Esr1	4E-12	0.5	Pfkl	1E-15	0.5
Pts	6E-24	0.5	Tns2	3E-12	0.5
Rps6ka2	4E-05	0.5	Map3k20	4E-30	0.5
Hoxa11os	1E-06	0.5	Tfb1m	4E-04	0.5
Lrrc51	1E-10	0.5	Wdr48	7E-13	0.5
Phlpp1	1E-09	0.5	Aig1	3E-54	0.5
Dnph1	4E-07	0.5	Npnt	2E-05	0.5
Dnajb9	3E-21	0.5	Dnajc15	5E-30	0.5
Tsc22d1	4E-86	0.5	Per2	7E-08	0.5
Bzw2	3E-38	0.5	Afap1l2	1E-05	0.5
Lmo4	2E-74	0.5	Hmox1	3E-31	0.5
Arl14ep	3E-23	0.5	Mterf3	1E-07	0.5
Gm37240	1E-19	0.5	Cep63	7E-11	0.5
Islr	2E-36	0.5	Pcsk5	9E-09	0.5
Gm32618	5E-03	0.5	Zfr2	5E-09	0.5
Nim1k	7E-12	0.5	Klc2	3E-04	0.5
Fbxo2	3E-13	0.5	Neat1	2E-80	0.5
Gpat3	1E-05	0.5	Atp1a1	2E-47	0.5
Rgcc	6E-06	0.5	Gas5	6E-99	0.5
Zfp566	3E-04	0.5	Pop5	7E-25	0.5
Eaf2	5E-04	0.5	B230118H07Rik	1E-31	0.5
Ggnbp1	1E-06	0.5	Arl10	2E-07	0.5
1500011B03Rik	2E-19	0.5	Btg3	1E-39	0.5
Pex11a	2E-08	0.5	Tmub1	2E-04	0.5
B230216N24Rik	3E-04	0.5	Ecrg4	4E-61	0.5
Tmem64	8E-06	0.5	Taf4b	2E-04	0.5
Slc22a15	1E-04	0.5	Mpp6	2E-17	0.5
Tns3	7E-40	0.5	1700029J07Rik	5E-07	0.5
Sesn2	3E-16	0.5	Adam22	1E-07	0.5
Dop1b	2E-12	0.5	Pkp4	5E-18	0.5
Snrpn	1E-05	0.5	Snx24	2E-26	0.5
Rassf3	5E-07	0.5	Ttc39b	3E-16	0.5
Mmp28	7E-04	0.5	Frs2	6E-24	0.5
Adi1	7E-13	0.5	Angpt1	4E-16	0.5
1700029J07Rik	1E-06	0.5	Bpnt1	5E-04	0.5
Kcnk2	2E-24	0.5	Znhit6	8E-19	0.5
Gng5	6E-181	0.5	Osgepl1	6E-03	0.5
Ssr2	3E-74	0.5	Them4	4E-12	0.5
Mak16	3E-24	0.5	Hax1	3E-37	0.5
Prxl2c	2E-27	0.5	Tbx5	3E-03	0.5
Enpp5	4E-12	0.5	Zfp692	1E-03	0.5
Myo1d	2E-55	0.5	2300009A05Rik	2E-16	0.5
Atp10a	7E-25	0.5	Tgoln1	1E-30	0.5
Hist1h1c	2E-33	0.5	Phyhd1	4E-41	0.5
Cst6	9E-07	0.5	Phactr2	2E-16	0.5
Acyp1	4E-23	0.5	Acot7	6E-12	0.5
Slc25a36	2E-13	0.5	Mirg	3E-09	0.5
A930005H10Rik	6E-08	0.5	Retsat	9E-06	0.5
Klc2	6E-06	0.5	Nt5c3	1E-10	0.5
Ptpn4	2E-17	0.5	Gsdme	2E-06	0.5
8430429K09Rik	1E-09	0.5	Epb41l1	3E-08	0.5
Slc35e2	6E-03	0.5	Pdzrn3	4E-57	0.5

Fibroblast

Control			$Notch2^{tm1.1Ecan}$		
Gene	*p*	FC	Gene	*p*	FC
Bcl2l12	2E-06	0.5	App	2E-71	0.6
Pmepa1	3E-13	0.5	Ccdc80	2E-74	0.6
Fosl1	3E-03	0.5	Zfp52	6E-04	0.6
Tipin	2E-15	0.5	Tnk2	9E-04	0.6
Rpp30	2E-07	0.5	Npat	7E-10	0.6
Sh3bgrl	1E-41	0.5	Camk2n2	1E-09	0.6
Lsm2	3E-09	0.5	Eepd1	2E-03	0.6
Tatdn2	4E-05	0.5	A930037H05Rik	6E-05	0.6
Gm4876	5E-08	0.5	Mad1l1	2E-13	0.6
Lgr4	1E-16	0.5	Pfas	2E-07	0.6
Recql	3E-04	0.5	Alyref	8E-29	0.6
Dock5	2E-08	0.5	Rnaseh2b	6E-10	0.6
Nemp1	4E-06	0.5	Ssrp1	5E-20	0.6
Psrc1	8E-04	0.5	Cntln	8E-20	0.6
Dynlt1f	2E-06	0.5	Ccdc112	5E-10	0.6
Msn	3E-35	0.5	Traf2	1E-04	0.6
Selenbp1	3E-08	0.5	Cdk19	1E-10	0.6
Fam20a	2E-08	0.5	Poc5	4E-05	0.6
Map6	9E-16	0.5	Nup205	3E-10	0.6
Osr1	3E-05	0.5	Lin7a	2E-03	0.6
Tinf2	8E-04	0.5	Snx11	2E-05	0.6
Psip1	4E-17	0.5	Als2	1E-07	0.6
Hoxd9	2E-04	0.5	Parp2	4E-12	0.6
Zfp948	2E-09	0.5	Slf1	2E-10	0.6
Saal1	3E-04	0.5	Pom121	7E-09	0.6
Baz1a	3E-05	0.5	Tnfrsf11b	4E-08	0.6
Rfc3	2E-06	0.5	Fdft1	2E-23	0.6
Dbi	9E-54	0.5	Phip	8E-34	0.6
Gm47167	3E-03	0.5	Idi1	1E-17	0.6
Dhrs3	1E-21	0.5	Sh3bgrl	3E-51	0.6
Miip	3E-04	0.5	Ninl	2E-07	0.6
Plcd3	7E-08	0.5	Mvk	1E-15	0.6
Cpsf4	1E-05	0.5	Cbx5	1E-29	0.6
Nsmce4a	8E-11	0.5	Pgp	2E-17	0.6
Ildr2	5E-03	0.5	Cep57	3E-14	0.6
Cavin2	3E-04	0.5	Cited2	5E-32	0.6
Htra1	2E-18	0.5	Phldb2	5E-40	0.6
Tgfb3	1E-12	0.5	Rif1	2E-14	0.6
Insig1	2E-15	0.5	Cdr1os	3E-06	0.6
1700024B18Rik	5E-07	0.5	Cped1	1E-43	0.6
Tpm1	4E-36	0.5	Ablim1	5E-15	0.6
Steap3	5E-17	0.5	Cgnl1	1E-21	0.6
Mapk3	5E-16	0.5	Chd1l	2E-07	0.6
Nmral1	6E-04	0.5	Specc1	7E-11	0.6
Pms1	2E-05	0.5	Tinagl1	1E-09	0.6
Frmd8	4E-04	0.5	Poc1b	2E-05	0.6
Sephs2	5E-07	0.5	Soga1	1E-12	0.6
Col5a2	1E-48	0.5	Tmem50b	3E-03	0.6
Cyp51	4E-14	0.5	Panx1	1E-04	0.6
Pgp	1E-10	0.5	Myof	5E-31	0.6
Pvr	7E-06	0.5	Fbln1	2E-15	0.6
Gm4890	1E-03	0.5	Suz12	1E-12	0.6
Ptpn3	4E-06	0.5	Orc6	2E-10	0.5
Pik3cb	6E-07	0.5	Mef2a	5E-27	0.5
Abca9	2E-03	0.5	Klhdc4	4E-07	0.5
Brip1os	1E-08	0.5	Glis3	5E-31	0.5
Nup205	2E-04	0.5	Zdhhc15	5E-06	0.5
Mcub	2E-08	0.5	1700025G04Rik	1E-14	0.5
Efnb2	2E-10	0.5	Ldlrad4	9E-39	0.5
Zbtb8a	6E-05	0.5	Dnase1l1	2E-06	0.5
Rif1	6E-08	0.5	Ccdc68	4E-09	0.5
Cnksr3	2E-11	0.5	Glrx	1E-10	0.5
Kremen1	3E-08	0.5	Ubr7	1E-10	0.5
Pi15	5E-07	0.5	Smc6	1E-31	0.5

Undefined

Control			$Notch2^{tm1.1Ecan}$		
Gene	*p*	FC	Gene	*p*	FC
Gm12353	9E-01	0.4	Cetn3	1E-02	0.4
Fam174a	3E-03	0.4	Ppp1r15a	3E-01	0.4
Aopep	2E-30	0.4	Slc6a8	3E-04	0.4
Timmdc1	7E-01	0.4	Higd1a	4E-06	0.4
Ccpg1os	7E-01	0.4	Tmem222	7E-04	0.3
Aldh6a1	9E-06	0.4	Isyna1	6E-01	0.3
Asns	4E-04	0.4	Plekha3	2E-02	0.3
Otud6b	5E-03	0.4	Trim47	3E-01	0.3
Zbtb18	3E-01	0.4	Lef1	4E-01	0.3
Miga1	2E-03	0.4	Cpeb3	7E-01	0.3
Epha2	9E-04	0.4	Tsen15	4E-05	0.3
Stk39	6E-05	0.4	1300002E11Rik	4E-03	0.3
Abhd6	6E-02	0.4	Usp24	2E-01	0.3
Veph1	2E-01	0.4	Gm15417	9E-01	0.3
Icmt	1E-02	0.4	Atp6v1g1	8E-07	0.3
Exoc8	2E-01	0.4	Eno1	4E-02	0.3
Pdcl	6E-01	0.4	Fhl1	5E-01	0.3
Sdf2l1	4E-05	0.4	Enpp5	8E-01	0.3
Gm35188	3E-01	0.4	Znrd1as	1E-01	0.3
Rida	6E-01	0.4	Wdr27	5E-01	0.3
Emc6	4E-03	0.4	Rgp1	6E-02	0.3
Saraf	1E-05	0.4	Erg	1E-01	0.3
Ggnbp1	3E-02	0.4	Cerk	1E-03	0.3
Ptrh2	7E-02	0.4	Cox17	2E-01	0.3
Slc24a5	2E-01	0.4	Gpn1	7E-02	0.3
0610009E02Rik	2E-02	0.4	Pigh	2E-02	0.3
Hspb6	2E-02	0.4	B230217O12Rik	3E-01	0.3
Cab39	2E-05	0.4	Cep104	6E-04	0.3
Rap2c	9E-01	0.4	Eef1akmt2	8E-05	0.3
Nrep	1E-02	0.4	Ndufa12	4E-01	0.3
3110021N24Rik	2E-02	0.4	Hk2	8E-01	0.3
Rdm1	4E-03	0.4	Pam16	3E-01	0.3
0610043K17Rik	7E-01	0.4	Mfsd4a	2E-04	0.3
Eps8	4E-05	0.4	Abcf2	4E-06	0.3
Rgcc	1E-01	0.4	Itm2c	9E-05	0.3
Nubpl	1E-01	0.4	Lipo3	1E+00	0.3
Pum3	8E-02	0.4	Brwd3	1E-02	0.3
Kdm6b	2E-01	0.4	Ipo8	7E-08	0.3
Rsph3b	2E-01	0.4	Tsc22d4	3E-01	0.3
Mfsd4a	7E-01	0.4	Ebna1bp2	8E-02	0.3
8430429K09Rik	5E-02	0.4	Pdia6	1E-09	0.3
Klf12	2E-09	0.4	Arl14ep	4E-02	0.3
Gprc5a	8E-02	0.4	Arl16	2E-07	0.3
2210408F21Rik	4E-01	0.4	Hax1	5E-01	0.3
Dcn	7E-43	0.4	Ndufab1	1E+00	0.3
Lonrf1	1E-01	0.4	Med31	2E-02	0.3
Vamp5	3E-09	0.4	Pebp1	1E-11	0.3
Hist1h1c	4E-06	0.4	Rab28	4E-01	0.3
Tgoln1	2E-06	0.4	Gabarapl2	7E-01	0.3
2610306M01Rik	2E-01	0.4	Rpa3	4E-01	0.3
Smo	9E-08	0.4	1110002L01Rik	7E-01	0.3
Rpap2	6E-01	0.4	Unc50	2E-01	0.3
Nomo1	4E-03	0.4	Rnf167	6E-02	0.3
Ppie	3E-01	0.4	Kazald1	2E-02	0.3
Tm2d3	2E-02	0.4	Kctd16	3E-01	0.3
Dnajc15	3E-03	0.4	Otud6b	2E-01	0.3
Mmp28	7E-01	0.4	Gtf2h4	2E-02	0.3
Pld1	9E-04	0.4	Cd9	3E-02	0.3
Rangrf	6E-01	0.4	Asnsd1	1E-03	0.3
Gnl3l	6E-01	0.4	Gng5	2E-29	0.3
Pdlim4	2E-01	0.4	Rragb	7E-01	0.3
Nipal2	5E-03	0.4	Nedd4l	4E-01	0.3
Fgfr2	2E-17	0.4	B830012L14Rik	4E-01	0.3
Zfp51	2E-01	0.4	Agrn	6E-01	0.3

Articular/Synovial Fibroblast

Control			$Notch2^{tm1.1Ecan}$		
Gene	*p*	FC	Gene	*p*	FC
Gan	7E-14	0.8	Slc1a6	4E-04	0.7
Jag1	3E-14	0.8	Tmem123	8E-07	0.7
Aacs	4E-13	0.8	Naaa	2E-08	0.7
Tmod3	3E-38	0.8	Chrdl1	1E-03	0.7
Rdh11	5E-08	0.8	Hspb8	2E-11	0.7
Cachd1	3E-25	0.8	Fer	3E-20	0.7
Pax9	1E-05	0.8	Dmpk	4E-12	0.7
Peg10	2E-07	0.8	Mgst3	1E-15	0.7
Plaat3	4E-44	0.8	Sh3bp5	1E-04	0.7
Cyp4f16	5E-05	0.8	Ostm1	9E-08	0.7
Fam117a	5E-05	0.8	Il1r1	5E-17	0.7
Tspan17	4E-24	0.8	H2-K1	3E-26	0.7
Edem2	7E-11	0.8	Robo2	6E-14	0.7
Plxna1	6E-14	0.8	Pcolce2	6E-14	0.7
Gm13052	2E-04	0.8	Ralgps1	3E-05	0.7
Raet1e	1E-05	0.8	Cmtm3	1E-19	0.7
C1qtnf1	2E-08	0.8	Errfi1	2E-09	0.7
Hebp1	1E-19	0.8	Zfp449	8E-04	0.7
Renbp	1E-16	0.8	Cog7	7E-08	0.7
AI413582	3E-29	0.8	Snx9	2E-34	0.7
Peli2	1E-16	0.8	Fyn	7E-10	0.7
Lrrc75a	1E-08	0.8	Trnp1	5E-20	0.7
Adam17	1E-18	0.8	Slc24a3	6E-24	0.7
Ptgfrn	2E-13	0.8	Tnks1bp1	7E-18	0.7
Glis3	2E-51	0.8	Mpdz	1E-23	0.7
Isg15	6E-09	0.8	Gucy1a2	2E-07	0.7
Litaf	2E-19	0.8	Gm10373	1E-03	0.7
Tshz3	6E-14	0.8	Arsk	2E-09	0.7
Ehd3	1E-07	0.8	Lgals3	3E-37	0.7
Nln	8E-23	0.8	Chrd	3E-05	0.7
Nrk	4E-01	0.8	Plaat3	1E-20	0.7
Car5b	3E-07	0.8	Hspa2	7E-03	0.7
Mvp	9E-19	0.8	Ldlrad4	2E-31	0.7
Arsk	1E-12	0.8	Myzap	5E-04	0.7
Ggta1	2E-06	0.8	Emc2	5E-28	0.7
Lrp11	1E-05	0.8	Il16	3E-06	0.7
Tom1	1E-18	0.8	Rftn1	2E-03	0.7
Slc12a9	4E-06	0.8	Vasn	2E-18	0.7
Parp9	3E-12	0.8	Nsmf	9E-06	0.7
Rhou	1E-10	0.8	Arsb	1E-10	0.7
Ggt7	1E-08	0.8	Thy1	5E-04	0.7
Lrrc8b	2E-05	0.8	Traf6	2E-05	0.7
Pard3b	3E-58	0.8	Hipk2	2E-13	0.7
Plekhg3	3E-09	0.8	Eef2k	2E-12	0.7
2200002D01Rik	2E-09	0.8	Il17rc	3E-12	0.7
Vcam1	4E-14	0.8	2200002D01Rik	5E-07	0.7
Zswim6	3E-25	0.8	Nfatc4	4E-11	0.7
Klhdc8a	3E-12	0.8	Atp7a	4E-07	0.7
Acaa2	8E-29	0.8	Tead3	3E-02	0.7
Diaph1	2E-31	0.8	Olfm1	5E-10	0.7
Eef2k	2E-25	0.8	Rab29	1E-04	0.7
Ehd2	6E-37	0.8	Pip5k1c	3E-05	0.7
A330023F24Rik	2E-09	0.7	Rtp4	8E-04	0.7
Mmp19	2E-08	0.7	Mef2a	5E-39	0.7
Fat3	6E-02	0.7	Zdhhc3	3E-16	0.7
Jam2	1E-10	0.7	Pi4kb	6E-10	0.7
Agpat3	3E-34	0.7	Srebf2	2E-14	0.7
Ahr	8E-18	0.7	Cndp2	4E-07	0.7
Plin3	8E-41	0.7	Carhsp1	2E-10	0.7
Bmp4	1E-18	0.7	Sema3f	1E-06	0.7
Plin2	1E-32	0.7	Kctd9	2E-06	0.7
Tlr4	6E-17	0.7	Idh1	2E-28	0.7
Il17ra	2E-12	0.7	Cdc14b	1E-06	0.7
Fads1	4E-20	0.7	Junos	1E-03	0.7

Limb Mesenchyme					
Control			$Notch2^{tm1.1Ecan}$		
Gene	*p*	FC	Gene	*p*	FC
Add1	2E-26	0.4	Parva	4E-43	0.4
Lgalsl	9E-06	0.4	Asah1	4E-41	0.4
Gm48742	8E-08	0.4	Evi5	3E-21	0.4
Bcl2l11	1E-11	0.4	Ppp1r9b	3E-11	0.4
Camkk2	1E-12	0.4	Crip1	2E-35	0.4
Cdr1os	4E-04	0.4	Plp2	8E-19	0.4
Naaa	5E-07	0.4	Rpain	5E-10	0.4
Phlda3	7E-36	0.4	Vmp1	9E-45	0.4
Rbms2	9E-24	0.4	Tpm4	2E-46	0.4
Bnc2	3E-26	0.4	Csrp1	2E-16	0.4
Prkar1b	2E-05	0.4	Ripk3	2E-08	0.4
Med13l	2E-30	0.4	Acat2	6E-07	0.4
Gatd1	6E-11	0.4	2410131K14Rik	4E-06	0.4
Ipmk	6E-04	0.4	Ddah2	5E-35	0.4
Cblb	4E-24	0.4	Itprid2	1E-12	0.4
Sgpp1	1E-05	0.4	Plekha4	5E-08	0.4
Cox6a2	6E-09	0.4	Efna4	4E-09	0.4
Parp9	5E-10	0.4	Cd34	6E-28	0.4
Mfsd10	5E-13	0.4	Fkbp7	3E-48	0.4
Bmt2	2E-14	0.4	Tle6	5E-05	0.4
Pmvk	3E-07	0.4	Vegfa	5E-12	0.4
Tbl2	1E-04	0.4	AU022252	2E-08	0.4
Mgmt	6E-19	0.4	Laptm4b	7E-14	0.4
Galk2	7E-23	0.4	Ggt7	2E-09	0.4
Ralb	6E-13	0.4	Glis2	6E-12	0.4
Cd1d1	2E-07	0.4	Med13l	2E-26	0.4
Scn2a	1E-07	0.4	Plscr3	6E-17	0.4
Abracl	2E-40	0.4	Eef2k	3E-19	0.4
Zdhhc15	6E-03	0.4	Bid	3E-07	0.4
Tesk2	9E-04	0.4	Zfp385a	2E-04	0.4
S100a10	1E-74	0.4	Pld2	7E-06	0.4
Sh3bgrl3	2E-70	0.4	Arhgap24	1E-31	0.4
Clk3	6E-06	0.4	Trim12c	3E-07	0.4
Arhgef9	1E-04	0.4	Fam171a2	5E-06	0.4
6330403K07Rik	2E-06	0.4	Adam9	5E-18	0.4
Pdgfrb	2E-19	0.4	Ppp1r14b	2E-82	0.4
Tle2	7E-06	0.4	Gdi1	2E-12	0.4
Mfsd3	3E-02	0.4	Snhg6	1E-31	0.4
5430416N02Rik	6E-09	0.4	Mgmt	8E-16	0.4
Cemip2	2E-15	0.4	Fbxo6	2E-10	0.4
Pld2	3E-03	0.4	Pdha1	1E-29	0.4
Ptgr1	6E-08	0.4	S100a6	5E-86	0.4
Smco4	5E-14	0.4	Gsto1	8E-22	0.4
2410006H16Rik	4E-62	0.4	Tmem256	1E-58	0.4
Prr7	7E-07	0.4	Zswim6	8E-16	0.4
Evi5	1E-16	0.4	Tcf7l2	4E-28	0.4
Erg28	1E-26	0.4	Esyt1	8E-17	0.4
2510009E07Rik	5E-09	0.4	Zfp984	7E-10	0.4
Oat	3E-29	0.4	Cadm2	9E-08	0.4
Cdkn2b	2E-16	0.4	Ptrh1	6E-13	0.4
B4galt1	1E-14	0.4	Ar	8E-11	0.4
Ppp1r2	3E-13	0.4	Wipf1	7E-15	0.4
Alcam	1E-09	0.4	Tnk2	4E-07	0.4
Lmx1b	3E-08	0.4	Pdia5	6E-16	0.4
Rpia	6E-03	0.4	Aprt	3E-55	0.4
Gpr153	2E-06	0.4	Fgf13	3E-01	0.4
Paxx	2E-13	0.4	Ing5	1E-08	0.4
Heg1	2E-02	0.4	Mmab	4E-09	0.4
Cybc1	8E-06	0.4	Tmtc3	1E-07	0.4
Layn	7E-08	0.4	Pex5	3E-09	0.4
Klhdc10	3E-09	0.4	Cmtm3	2E-24	0.4
Sytl2	2E-05	0.4	Fam89b	6E-22	0.4
Fscn1	4E-33	0.4	Snhg15	1E-07	0.4
Actn1	1E-34	0.4	Htra3	7E-23	0.4

Chondrogenic					
Control			$Notch2^{tm1.1Ecan}$		
Gene	*p*	FC	Gene	*p*	FC
5930430L01Rik	1E-13	0.5	Emilin1	1E-36	0.5
Adam22	1E-07	0.5	Klhdc1	8E-05	0.5
Uck2	5E-13	0.5	Mef2c	6E-35	0.5
Xpot	1E-27	0.5	Mylip	1E-10	0.5
Imp3	4E-46	0.5	Gm10762	8E-06	0.5
Aplp2	1E-90	0.5	Rsph3b	2E-03	0.5
Ogfod3	2E-07	0.5	Saraf	1E-40	0.5
Tcf4	3E-102	0.5	Hspa9	5E-69	0.5
Matn4	6E-29	0.5	A930007I19Rik	2E-03	0.5
Fzd3	2E-04	0.5	Sppl2a	5E-21	0.5
Rin2	4E-16	0.5	Mlh3	7E-07	0.5
Prkn	2E-33	0.5	Cpeb3	2E-14	0.5
Pfkfb3	3E-07	0.5	Lmo4	5E-52	0.5
Dnajc15	8E-30	0.5	Lipo3	1E-05	0.5
Rarg	3E-21	0.5	Pycr1	5E-24	0.5
Hspa9	2E-76	0.5	3110021N24Rik	2E-05	0.5
Ppm1h	2E-16	0.5	Mphosph9	4E-09	0.5
Trmt61b	3E-08	0.5	Rfesd	2E-14	0.5
Nek1	3E-11	0.5	Det1	2E-02	0.5
Ankrd44	2E-21	0.5	Tead4	2E-05	0.5
Sesn3	3E-42	0.5	Adcy2	7E-24	0.5
Prss36	5E-07	0.5	BC065397	4E-05	0.5
Pop5	1E-24	0.5	Lin7a	2E-03	0.5
Npr2	1E-19	0.5	Limk2	4E-07	0.5
Tbx15	7E-63	0.5	Wdr92	1E-03	0.5
Ing2	1E-12	0.5	Sdr39u1	4E-14	0.5
Slc37a4	1E-06	0.5	Me1	2E-33	0.5
Snai1	2E-05	0.5	Mgat5	4E-06	0.5
Pkp4	6E-13	0.5	Atf6	5E-15	0.5
Tcfl5	3E-07	0.5	Bach2	3E-04	0.5
Ndufaf4	6E-20	0.5	Plag1	2E-08	0.5
Map3k20	2E-26	0.5	Ap1g1	1E-18	0.5
Ppp3ca	5E-64	0.5	Nr4a2	7E-10	0.5
E230016M11Rik	1E-02	0.5	Jmy	2E-16	0.5
Tmem86a	9E-06	0.5	Cox6a2	1E-05	0.5
Bach2	5E-08	0.5	Pdk1	7E-08	0.5
Tmem154	3E-08	0.5	Nrk	8E-28	0.5
Tmtc2	3E-29	0.5	Mbnl2	2E-80	0.5
Tut1	6E-05	0.5	4930517O19Rik	3E-05	0.5
Slc39a11	2E-08	0.5	Tubb2b	7E-06	0.5
Slc9a3r2	6E-09	0.5	Cd46	9E-03	0.5
Gfpt2	3E-09	0.5	Tecpr2	3E-07	0.5
Sec11c	2E-22	0.5	Tbx15	1E-49	0.5
2810001G20Rik	5E-08	0.5	Pcgf5	9E-07	0.5
Igfbp5	2E-20	0.5	Boc	2E-23	0.4
Mtmr3	1E-27	0.5	Rnaset2a	3E-05	0.4
Slc38a2	6E-38	0.5	Tmx1	1E-26	0.4
Ubxn8	1E-05	0.5	Sh3rf2	5E-12	0.4
Phyhd1	3E-42	0.5	Ripk2	1E-05	0.4
Nubpl	4E-09	0.5	Dmac2l	2E-06	0.4
Rnaset2b	1E-06	0.5	Zfp516	3E-20	0.4
5031425E22Rik	9E-25	0.5	Mthfd2	9E-35	0.4
Stk38l	6E-13	0.5	1700012D14Rik	9E-06	0.4
Sh3rf2	9E-13	0.5	Abca1	3E-11	0.4
Bnip3	7E-23	0.5	Apmap	1E-11	0.4
A930015D03Rik	3E-11	0.5	Mrps6	9E-25	0.4
Tgfa	1E-06	0.5	Gng5	1E-123	0.4
Gm4221	5E-08	0.5	Zfp469	8E-05	0.4
Pak1	3E-09	0.5	Id2	1E-23	0.4
Plag1	5E-07	0.5	Mtss2	5E-23	0.4
Dancr	2E-05	0.5	A930005H10Rik	8E-05	0.4
Arv1	1E-04	0.5	Adgrg6	8E-27	0.4
Ccdc47	2E-24	0.5	Fam20c	1E-05	0.4
Grwd1	4E-05	0.5	Xpr1	2E-13	0.4

Fibroblast					
Control			$Notch2^{tm1.1Ecan}$		
Gene	*p*	FC	Gene	*p*	FC
Tead2	1E-05	0.5	Lrrcc1	2E-13	0.5
Sema3b	4E-10	0.5	Mlf1	7E-14	0.5
Pms2	1E-05	0.5	Adcy7	1E-07	0.5
Mmab	3E-07	0.5	Arhgap42	1E-19	0.5
Ifit3	5E-07	0.5	Spcs3	3E-18	0.5
Il15ra	3E-04	0.5	Ackr3	7E-07	0.5
Alyref	2E-15	0.5	Gm11266	7E-06	0.5
Nup62	3E-08	0.5	Pla2r1	4E-04	0.5
Uchl5	1E-08	0.5	Anxa3	6E-26	0.5
Mn1	4E-08	0.5	Enpp1	2E-08	0.5
Tfdp1	1E-08	0.5	Nrip1	1E-17	0.5
Tbc1d31	6E-05	0.5	Abhd10	4E-07	0.5
Xpo1	4E-17	0.5	Zfp449	2E-03	0.5
Spata6	1E-12	0.5	Slc35d2	4E-06	0.5
Sned1	3E-17	0.5	Wdpcp	4E-12	0.5
S100a13	9E-35	0.5	Dab2	1E-23	0.5
Kif13b	6E-14	0.5	Sacs	3E-07	0.5
Zswim9	2E-02	0.5	Ano8	4E-04	0.5
Gtpbp8	3E-09	0.5	Tuba1a	9E-44	0.5
Stard8	2E-03	0.5	Dock9	5E-07	0.5
Trabd2b	5E-16	0.5	Rtel1	2E-11	0.5
Acsl5	8E-07	0.5	Foxd2os	3E-08	0.5
4930523C07Rik	3E-25	0.5	Msh2	7E-06	0.5
Tubgcp6	2E-03	0.5	Steap3	2E-19	0.5
Parp12	5E-04	0.5	Sipa1l2	3E-05	0.5
Nrtn	8E-03	0.5	Lrrc40	6E-06	0.5
Smoc1	9E-13	0.5	Rpgrip1l	1E-04	0.5
Lats2	2E-23	0.5	Nfia	5E-50	0.5
Gm41724	4E-09	0.5	Platr25	5E-10	0.5
Gas2	9E-19	0.5	Kpnb1	7E-12	0.5
Ifih1	4E-03	0.5	Pxdn	4E-23	0.5
Tmem43	2E-04	0.5	Xrcc1	1E-09	0.5
Mindy3	1E-09	0.5	Pear1	4E-04	0.5
Dock11	3E-08	0.5	Kat2b	7E-12	0.5
Ift80	9E-07	0.5	Cav3	3E-07	0.5
L3mbtl3	5E-13	0.5	Fads1	4E-16	0.5
Slc38a1	3E-15	0.5	Itgb3bp	1E-11	0.5
Gm15581	1E-04	0.5	Vegfc	6E-04	0.5
Lrrc8b	2E-06	0.5	Ehd2	1E-31	0.5
Ubr7	7E-07	0.5	Sae1	5E-17	0.5
Itgbl1	3E-20	0.5	E2f1	3E-08	0.5
Thsd4	5E-02	0.5	Casp3	3E-07	0.5
Stip1	7E-12	0.5	Rad50	4E-21	0.5
Eogt	1E-04	0.5	Pml	1E-06	0.5
Thbs1	6E-18	0.5	Sart3	1E-11	0.5
Srrt	4E-10	0.5	Rcc1	6E-06	0.5
Nup155	5E-06	0.5	Mphosph9	2E-10	0.5
Unc119b	6E-05	0.5	Baz1a	4E-13	0.5
Tmem132a	4E-10	0.5	Loxl1	8E-44	0.5
Pmp22	2E-34	0.5	Rpgrip1	6E-06	0.5
Cib2	1E-06	0.5	Ptms	9E-64	0.5
Mical3	4E-13	0.5	Tbc1d31	5E-07	0.5
Nr1d1	1E-03	0.5	Ran	1E-25	0.5
Sae1	8E-10	0.5	Akr1b10	7E-05	0.5
Lsm6	1E-16	0.5	Sgsh	5E-03	0.5
Idh2	7E-25	0.5	Ctxn1	8E-06	0.5
Aaas	9E-04	0.5	Rab7b	6E-09	0.5
Sh2b3	2E-02	0.5	Lsm6	1E-21	0.5
Poc1b	6E-05	0.5	Selenbp1	6E-07	0.5
Acox2	2E-03	0.5	Svep1	4E-44	0.5
Col4a5	2E-10	0.5	Ell2	1E-21	0.5
Ampd3	2E-07	0.5	Nicn1	3E-05	0.5
Ccdc61	2E-05	0.5	Gm42047	8E-06	0.5
Gpsm2	4E-12	0.5	Pot1a	2E-06	0.5

Undefined					
Control			$Notch2^{tm1.1Ecan}$		
Gene	*p*	FC	Gene	*p*	FC
Lcn2	1E-09	0.4	Tsc22d1	2E-05	0.3
Thyn1	1E-01	0.4	Prorsd1	9E-04	0.3
Id2	5E-07	0.4	Hspe1	4E-21	0.3
Hoxa11	2E-02	0.4	Cebpzos	8E-02	0.3
Lman2l	7E-02	0.4	Per3	6E-01	0.3
Coa7	6E-02	0.4	Ccdc90b	4E-03	0.3
Gm19522	1E-01	0.4	Lars	4E-01	0.3
Pkp4	7E-04	0.4	Ivns1abp	6E-03	0.3
Malt1	4E-02	0.4	Gm15050	2E-01	0.3
Flad1	3E-01	0.4	Foxo3	4E-04	0.3
Lrrc75b	5E-02	0.4	Clu	1E-01	0.3
P4ha1	1E-23	0.4	Lrrc8d	9E-01	0.3
Lgals3	1E-12	0.4	Sumf2	6E-03	0.3
Chic1	3E-01	0.4	Psmg2	8E-02	0.3
Arid5a	1E-03	0.4	Glce	7E-02	0.3
Bhlhe40	1E-05	0.4	Ctnnal1	9E-01	0.3
Lipo3	5E-01	0.4	Xylt2	5E-04	0.3
Neat1	5E-27	0.4	Hmgcll1	3E-01	0.3
Sorl1	5E-05	0.4	Slc35a1	3E-05	0.3
Ric3	1E-02	0.4	Hoxc6	2E-03	0.3
9530026P05Rik	1E-01	0.4	Ctsh	6E-01	0.3
Arhgef2	3E-01	0.3	Erlin1	3E-01	0.3
Pygo2	6E-01	0.3	Park7	3E-05	0.3
Dek	9E-14	0.3	Mylk	5E-02	0.3
Lyrm4	2E-02	0.3	Sh3rf2	5E-01	0.3
Selenos	2E-30	0.3	Fzd3	5E-02	0.3
Fmod	2E-19	0.3	Slc15a4	1E-03	0.3
Mtm1	7E-01	0.3	Vps39	3E-04	0.3
1700029J07Rik	9E-01	0.3	Smo	6E-01	0.3
Zscan26	7E-01	0.3	Ndufa1	8E-01	0.3
Golph3	1E-05	0.3	Saysd1	1E-02	0.3
Nupr1	1E-14	0.3	Mboat2	8E-01	0.3
Crebl2	6E-01	0.3	Plpbp	4E-03	0.3
Nacc2	3E-04	0.3	Synpo	3E-01	0.3
2810001G20Rik	5E-02	0.3	Isoc2b	2E-06	0.3
Slc38a2	2E-06	0.3	Sppl2a	9E-01	0.3
Rcc2	4E-05	0.3	Slc12a2	8E-01	0.3
Mir99ahg	3E-25	0.3	Icam1	5E-01	0.3
Pde3b	5E-06	0.3	Fbxo31	3E-04	0.3
Ppp1r9a	2E-01	0.3	Eif1	2E-49	0.3
Mcts2	4E-01	0.3	Ghitm	4E-01	0.3
Kantr	4E-02	0.3	Xpot	2E-01	0.3
Pgrmc2	2E-06	0.3	Gtf2b	5E-09	0.3
Usp50	1E+00	0.3	Tom1l2	7E-01	0.3
B830012L14Rik	5E-05	0.3	Cstf1	2E-03	0.3
Eml6	7E-02	0.3	Tmem238	5E-01	0.3
Shq1	2E-01	0.3	Thnsl2	4E-01	0.3
Abi2	2E-01	0.3	Ctsd	4E-17	0.3
Tbc1d4	6E-01	0.3	Mfap1b	3E-02	0.3
Yrdc	1E-01	0.3	Senp8	3E-02	0.3
Ankrd28	4E-04	0.3	Ogt	2E-01	0.3
Cpox	7E-03	0.3	Mageh1	2E-01	0.3
Cbx4	5E-01	0.3	Cox18	7E-03	0.3
Mirg	5E-04	0.3	Pum3	2E-02	0.3
Zbtb11os1	6E-01	0.3	Nifk	2E-02	0.3
Tbc1d1	6E-01	0.3	Vamp5	4E-01	0.3
Adal	4E-01	0.3	Rps24	1E-55	0.3
Nedd9	3E-05	0.3	Prpf31	1E-04	0.3
Cpsf1	6E-01	0.3	H2-DMa	3E-03	0.3
Zfp839	8E-01	0.3	Zfp994	2E-01	0.3
Gt(ROSA)26Sor	2E-02	0.3	2810403D21Rik	7E-02	0.3
Dctd	1E-01	0.3	Gtpbp2	1E-03	0.3
Gng13	6E-03	0.3	Ism1	1E-01	0.3
Lars	8E-05	0.3	Zfp81	5E-06	0.3

Articular/Synovial Fibroblast					
Control			$Notch2^{tm1.1Ecan}$		
Gene	*p*	FC	Gene	*p*	FC
Poglut3	1E-19	0.7	Ggta1	2E-03	0.7
Fsd1l	6E-06	0.7	Cers4	3E-09	0.7
Rassf8	5E-19	0.7	Aida	6E-12	0.7
Slc31a2	3E-11	0.7	Ddit4l	6E-08	0.7
Hsdl1	9E-04	0.7	Zfp90	4E-06	0.7
Tcn2	1E-29	0.7	Ifit3	6E-08	0.7
Mgll	8E-16	0.7	Lgalsl	1E-05	0.7
Tap1	8E-07	0.7	Gm49359	7E-02	0.7
Mmp2	8E-27	0.7	Npc2	7E-61	0.7
B2m	4E-49	0.7	Pcyt2	3E-08	0.7
Large1	7E-58	0.7	Rbms1	7E-53	0.7
Fbn2	1E-19	0.7	Ifit2	3E-08	0.7
Cndp2	6E-08	0.7	Mr1	3E-03	0.7
2900026A02Rik	6E-12	0.7	Prag1	1E-04	0.7
Lgals3	1E-52	0.7	Fbn2	4E-16	0.7
Hipk2	2E-36	0.7	Ropn1l	2E-03	0.7
Chst7	2E-05	0.7	Agtrap	1E-05	0.7
Dmpk	1E-11	0.7	D130040H23Rik	4E-05	0.7
S100a16	1E-25	0.7	Gm26881	5E-07	0.7
Eepd1	8E-04	0.7	Gprc5a	5E-03	0.7
Arl15	6E-39	0.7	Rbms3	4E-43	0.7
Pla2g15	2E-04	0.7	Prss23	6E-21	0.6
Ccdc141	2E-05	0.7	Fn1	2E-57	0.6
Cers4	5E-11	0.7	Cyth3	2E-22	0.6
Mat2a	2E-38	0.7	Khdrbs3	6E-04	0.6
Mocs1	9E-06	0.7	Tcn2	4E-18	0.6
St3gal2	5E-21	0.7	Tns1	3E-16	0.6
Cped1	3E-55	0.7	Slco3a1	2E-06	0.6
Sh3pxd2a	4E-34	0.7	Fxyd1	2E-27	0.6
Itga5	9E-21	0.7	Cltb	6E-18	0.6
D130040H23Rik	4E-08	0.7	Mmp19	1E-05	0.6
Pgpep1	1E-06	0.7	Gpsm2	5E-14	0.6
Pqlc3	4E-12	0.7	Dtnbp1	1E-26	0.6
Msx1	3E-05	0.7	Myo7a	8E-06	0.6
Fxyd1	6E-37	0.7	G6pdx	3E-07	0.6
Slco3a1	1E-09	0.7	Dact1	5E-05	0.6
Pdgfra	5E-14	0.7	Lrig1	7E-03	0.6
Lamb1	5E-15	0.7	Gxylt2	3E-09	0.6
Birc3	5E-05	0.7	Rpia	6E-04	0.6
Sh3bp5	2E-09	0.7	Cav3	8E-06	0.6
Ifi35	7E-13	0.7	Glis3	5E-33	0.6
Dnase2a	7E-09	0.7	1500015L24Rik	7E-13	0.6
Ebp	8E-16	0.7	Mat2a	3E-20	0.6
Vcl	2E-41	0.7	Cyb5r3	2E-41	0.6
4732440D04Rik	1E-05	0.7	Vps13b	2E-28	0.6
Pi4kb	3E-16	0.7	Sbno2	4E-10	0.6
Prr13	2E-36	0.7	Apba1	3E-06	0.6
Plcl1	2E-17	0.7	Tmem41a	1E-04	0.6
Cercam	1E-18	0.7	Rabl2	6E-03	0.6
Rab23	4E-10	0.7	Arhgap44	2E-07	0.6
Marcks	6E-80	0.7	Meox2	2E-10	0.6
Vegfb	4E-26	0.7	Nckap5l	7E-02	0.6
Dpysl3	7E-14	0.7	Nhsl2	1E-05	0.6
Cyb5r3	8E-65	0.7	Dlc1	3E-47	0.6
Rab32	8E-05	0.7	Litaf	3E-13	0.6
Uap1l1	8E-36	0.7	Unc93b1	1E-07	0.6
Atp6v0a1	5E-21	0.7	Epha4	1E-06	0.6
Nat8f1	1E-03	0.7	Ifi35	7E-06	0.6
Calhm2	6E-08	0.7	Tecpr1	9E-05	0.6
Laptm4b	1E-11	0.7	Hebp2	2E-10	0.6
Dact1	1E-06	0.7	Faxc	3E-03	0.6
S100a10	9E-68	0.7	Agtr1a	6E-04	0.6
Cd1d1	7E-07	0.7	Arhgdib	2E-08	0.6
L3mbtl3	6E-16	0.7	Itga7	5E-06	0.6

Limb Mesenchyme

Control			*Notch2*$^{tm1.1Ecan}$		
Gene	*p*	FC	Gene	*p*	FC
Kras	3E-24	0.4	Plekha2	1E-05	0.4
1600014C10Rik	2E-03	0.4	Itga9	2E-06	0.4
Setd4	2E-03	0.4	Pml	8E-02	0.4
Zfp524	2E-06	0.4	Tshz3	1E-15	0.4
Lyn	4E-06	0.4	Brsk1	6E-04	0.4
Parp12	4E-05	0.4	Rab30	2E-15	0.4
Zfp992	1E-03	0.4	Cyb5r3	1E-50	0.4
Tspan4	1E-34	0.4	Gyg	6E-23	0.4
Tlr2	3E-03	0.4	Nme2	2E-116	0.4
Kif1a	3E-11	0.4	Ikzf2	5E-05	0.4
Greb1l	3E-02	0.4	Cald1	2E-60	0.4
Ifngr1	3E-06	0.4	Bhlhe41	1E-04	0.4
Pmm1	3E-14	0.4	Erg28	1E-23	0.4
Ptges3l	8E-04	0.4	Macrod2	9E-14	0.4
Cdk5	1E-04	0.4	Cavin3	5E-50	0.4
Eif3e	3E-84	0.4	Clec11a	5E-27	0.4
Smarcd2	7E-07	0.4	Oaf	5E-16	0.4
Six1	3E-13	0.4	Ankrd29	3E-09	0.4
Pik3r3	5E-06	0.4	Galt	1E-09	0.4
2310022B05Rik	1E-13	0.4	Trib2	3E-04	0.4
Nln	7E-14	0.4	2510009E07Rik	3E-09	0.4
Syde1	5E-07	0.4	Mxra7	1E-39	0.4
Gse1	7E-08	0.4	Klhdc10	2E-12	0.4
Plekha4	8E-06	0.4	Ier3	3E-13	0.4
Pck2	7E-23	0.4	Man1a	1E-13	0.4
Lrrc42	1E-16	0.4	Adgrl1	3E-13	0.4
Tmod2	9E-05	0.4	Chrd	3E-07	0.4
Gpx7	2E-26	0.4	Cdr2l	5E-09	0.4
2700069I18Rik	6E-09	0.4	Slc5a3	5E-03	0.4
Tnfsf12	7E-16	0.4	Pold4	4E-23	0.4
Ablim1	9E-20	0.4	Lama3	1E-04	0.4
Maml2	1E-19	0.4	2310022B05Rik	5E-18	0.4
Ier5l	2E-09	0.4	Gipc1	1E-15	0.4
Blcap	1E-08	0.4	5730409E04Rik	3E-07	0.4
Dipk1a	2E-04	0.4	Abcg2	7E-07	0.4
Rhoj	4E-14	0.4	Kctd17	1E-10	0.4
Prcp	2E-10	0.4	Cdk14	3E-37	0.4
Ahr	2E-07	0.4	Flot1	2E-15	0.4
Pbx3	6E-12	0.4	Abi3bp	8E-30	0.4
Eva1b	8E-28	0.4	Tomm40l	4E-05	0.4
Lrp6	5E-30	0.4	Hdac3	2E-14	0.4
Eef2k	5E-14	0.4	Zfp524	3E-10	0.4
Selenom	2E-63	0.4	LTO1	3E-06	0.4
Asap1	6E-47	0.4	Tpm2	1E-25	0.4
Zfp365	7E-03	0.4	Paox	3E-05	0.4
Pmepa1	1E-29	0.4	Sema3a	1E-07	0.4
Ndn	2E-15	0.4	Nlgn2	7E-12	0.4
Tmem151a	3E-05	0.4	Prss35	2E-04	0.4
Ak1	2E-13	0.4	Gpc1	6E-15	0.4
Ap2a2	2E-26	0.4	Mdfic	2E-23	0.4
Tnk2	1E-03	0.4	Map2	3E-09	0.4
Ten1	6E-07	0.4	Cask	1E-27	0.4
Paox	1E-02	0.4	Prss23	2E-50	0.4
Nrp1	3E-21	0.4	Nme4	1E-18	0.4
Ddx58	3E-05	0.4	Selenom	2E-62	0.4
Plekho2	3E-07	0.4	Trim8	5E-11	0.4
Akt1	9E-21	0.4	Stard3nl	2E-22	0.4
Nsdhl	6E-09	0.4	Fbxo25	3E-07	0.4
Acap3	3E-07	0.4	Akap13	3E-39	0.4
Rap2a	2E-12	0.4	Arhgap28	1E-22	0.4
Bmp1	1E-25	0.4	Shisa5	2E-23	0.4
1700017B05Rik	7E-03	0.4	Cthrc1	2E-16	0.4
Tm7sf2	3E-04	0.4	Mark4	7E-07	0.4
Spryd7	9E-06	0.4	Irf9	2E-07	0.4

Chondrogenic

Control			*Notch2*$^{tm1.1Ecan}$		
Gene	*p*	FC	Gene	*p*	FC
Lin7a	6E-05	0.5	Sec11c	3E-21	0.4
Ccpg1os	8E-10	0.5	Mphosph10	9E-14	0.4
Spa17	2E-05	0.5	Sft2d2	1E-14	0.4
Rfesd	4E-14	0.5	Slc19a2	2E-05	0.4
Nmnat3	6E-06	0.5	Sec14l1	2E-19	0.4
Yipf2	1E-05	0.5	Cep85	2E-05	0.4
Pdcd4	2E-60	0.5	Ppat	2E-07	0.4
Ears2	5E-05	0.5	Atg16l2	3E-08	0.4
4732471J01Rik	1E-04	0.5	Nmnat3	2E-06	0.4
Slc1a4	8E-13	0.4	Nol8	3E-06	0.4
Mpped2	3E-08	0.4	Agrn	2E-08	0.4
Gsdme	3E-11	0.4	Dclre1c	4E-02	0.4
Cbx4	7E-11	0.4	Usp37	1E-13	0.4
Ctsd	6E-107	0.4	Kirrel3	8E-07	0.4
Zfp423	7E-12	0.4	Pecr	6E-06	0.4
Slc16a4	1E-09	0.4	Mtfr1	2E-10	0.4
Wasf3	1E-06	0.4	Xpot	2E-30	0.4
Mllt11	5E-07	0.4	Ptpre	3E-03	0.4
Sil1	4E-23	0.4	Snrpb2	1E-26	0.4
Wdr73	1E-02	0.4	Klhl22	1E-08	0.4
Gar1	1E-14	0.4	Tsc22d4	2E-47	0.4
Tmub1	1E-06	0.4	Wars	9E-07	0.4
Ccdc8	2E-05	0.4	Acad10	3E-04	0.4
Polr3h	1E-11	0.4	Ppm1h	5E-21	0.4
Med12l	6E-03	0.4	Zfp239	2E-05	0.4
Ric3	2E-06	0.4	Ccdc57	9E-02	0.4
Patj	6E-15	0.4	Slc25a36	4E-11	0.4
Zdhhc12	2E-09	0.4	Ptrh2	7E-10	0.4
Mthfd2	1E-36	0.4	Clint1	1E-30	0.4
Sgf29	2E-06	0.4	Atp2b1	4E-44	0.4
Tpmt	1E-09	0.4	Ralgapa2	6E-13	0.4
Hax1	2E-32	0.4	Ift88	4E-11	0.4
Rtkn	4E-06	0.4	Sybu	5E-08	0.4
Acad10	3E-04	0.4	Prdm15	3E-02	0.4
Rrp12	8E-06	0.4	Sik3	7E-27	0.4
Arl10	7E-07	0.4	Mipol1	1E-17	0.4
Nudcd1	5E-07	0.4	Ifrd2	1E-02	0.4
Hyou1	5E-10	0.4	Isyna1	1E-06	0.4
Snx24	3E-23	0.4	mt-Nd6	8E-09	0.4
Rab27b	4E-05	0.4	5930430L01Rik	5E-06	0.4
Eml6	6E-07	0.4	Amacr	3E-04	0.4
Mtfr1	3E-11	0.4	Zfas1	7E-35	0.4
Cd320	2E-16	0.4	B830012L14Rik	9E-14	0.4
Lpar4	1E-29	0.4	Bcat2	9E-05	0.4
Meg3	1E-52	0.4	Gfod2	2E-04	0.4
Taf1d	3E-25	0.4	Bcl2l1	1E-15	0.4
Kirrel3	8E-07	0.4	Aldh18a1	2E-12	0.4
Epb41l4aos	6E-20	0.4	Bnip3	4E-17	0.4
Resf1	6E-09	0.4	Trmt10a	2E-06	0.4
Rbak	3E-03	0.4	Ssr2	4E-56	0.4
Wars	8E-11	0.4	Rab28	5E-29	0.4
Aen	5E-12	0.4	Atoh8	8E-05	0.4
Glrb	6E-05	0.4	Tns3	9E-42	0.4
Actr6	2E-06	0.4	Arhgef5	1E-09	0.4
Col4a5	4E-15	0.4	A730081D07Rik	4E-03	0.4
Pam	7E-94	0.4	Alkbh1	2E-12	0.4
Zbtb18	5E-06	0.4	Ifi30	3E-27	0.4
Gm20300	2E-02	0.4	Rad9b	2E-04	0.4
Sdc3	1E-16	0.4	Tmem246	2E-06	0.4
Dek	7E-60	0.4	Ngrn	1E-05	0.4
Hypk	2E-20	0.4	Slc1a4	5E-11	0.4
Rnaset2a	1E-07	0.4	Lpar4	7E-26	0.4
Tsc22d4	9E-48	0.4	Ahcyl2	9E-17	0.4
Rrp9	1E-08	0.4	2210408F21Rik	9E-13	0.4

Fibroblast

Control			*Notch2*$^{tm1.1Ecan}$		
Gene	*p*	FC	Gene	*p*	FC
Gltp	2E-06	0.5	C2cd3	6E-10	0.5
Cdk5rap2	2E-07	0.5	Smc1a	3E-34	0.5
Nck2	3E-05	0.5	Plxna2	1E-10	0.5
Ndrg1	1E-18	0.5	Gsta4	8E-01	0.5
Rcc1	5E-04	0.5	Haus7	4E-09	0.5
Clcc1	4E-12	0.5	Timp3	2E-23	0.5
Gm1673	2E-10	0.5	St3gal2	5E-14	0.5
Apol8	7E-06	0.5	Oscp1	7E-07	0.5
Pacsin2	4E-19	0.5	Arnt2	1E-05	0.5
Ttl	6E-03	0.5	Mbnl3	1E-07	0.5
Cyp27a1	6E-04	0.5	Cdkn2a	2E-17	0.5
Afmid	3E-05	0.5	Mkx	2E-11	0.5
C2cd3	5E-06	0.5	C2cd2	6E-05	0.5
Gm16133	2E-05	0.5	Olfml2b	1E-13	0.5
Acat2	2E-06	0.5	Lats2	5E-25	0.5
Fam129b	1E-09	0.5	Cdc25a	2E-08	0.5
Lrwd1	5E-02	0.5	Raly	3E-48	0.5
Slc4a7	4E-09	0.5	Gins4	3E-08	0.5
Cdc25a	6E-04	0.5	Htra1	2E-23	0.5
Phip	5E-28	0.5	Rnf144a	2E-10	0.5
Grk5	2E-11	0.5	Sbno2	2E-09	0.5
Rhno1	1E-04	0.5	Vgll4	6E-27	0.5
Fam110a	1E-03	0.5	Fam20a	3E-15	0.5
Tcirg1	6E-03	0.5	Pitx1	6E-34	0.5
Dek	1E-06	0.5	Lmo1	2E-06	0.5
Ptgr1	3E-02	0.5	Rasl11a	2E-05	0.5
Sgms2	4E-09	0.5	Kctd9	1E-09	0.5
Cep112	2E-04	0.5	Aspn	1E-22	0.5
Pdpn	4E-21	0.5	Anapc15	8E-12	0.5
Ldlr	1E-19	0.5	Snrpd1	3E-37	0.5
Capg	2E-21	0.5	Tipin	2E-09	0.5
Dcbld1	3E-02	0.5	Cc2d1a	1E-03	0.5
Erlin1	1E-09	0.5	Tmem140	7E-07	0.5
Higd1a	3E-34	0.5	Dlc1	3E-54	0.5
E2f1	2E-05	0.5	Eif2ak2	1E-13	0.5
Pfkp	4E-09	0.5	Mettl14	9E-03	0.5
Prkd1	6E-15	0.5	Olfm1	1E-10	0.5
Sugp2	1E-07	0.5	Naa40	5E-07	0.5
Prkci	2E-12	0.5	Mitf	8E-12	0.5
Dgcr8	2E-07	0.5	Zfp40	4E-04	0.5
Prnp	2E-45	0.5	Lrrc8b	3E-04	0.5
Fdft1	1E-13	0.5	Ncoa7	8E-14	0.5
Agfg2	3E-08	0.5	Stom	1E-06	0.5
Ccdc84	2E-01	0.5	Gm1673	1E-18	0.5
Plekhh2	1E-22	0.5	Lmbrd2	3E-06	0.5
G6pdx	4E-05	0.4	Fgf13	6E-15	0.5
Rexo5	7E-04	0.4	A430033K04Rik	2E-04	0.5
Ssx2ip	5E-06	0.4	Nup62	3E-10	0.5
Csmd1	2E-07	0.4	Adamtsl4	1E-11	0.5
Ptbp3	2E-15	0.4	Ctdspl2	2E-13	0.5
Zfp668	6E-02	0.4	Rbmx	1E-10	0.5
Tram2	4E-17	0.4	Zc3h4	4E-08	0.5
Igf2bp1	3E-03	0.4	Adarb1	3E-03	0.5
Eftud2	3E-07	0.4	Mettl27	5E-05	0.5
Eng	7E-07	0.4	Lsm8	3E-10	0.5
Rassf2	2E-05	0.4	Ocel1	3E-04	0.5
Lynx1	2E-08	0.4	Casp8ap2	8E-09	0.5
Cst6	2E-13	0.4	Tspo	4E-35	0.5
Pitx1	8E-29	0.4	Katnb1	9E-06	0.5
Ess2	3E-03	0.4	Ccn4	4E-12	0.5
Sorbs3	2E-05	0.4	A630089N07Rik	1E-04	0.5
Podxl2	4E-04	0.4	Larp7	1E-14	0.5
Arfgap2	2E-05	0.4	Slc16a3	5E-05	0.5
Mvk	3E-10	0.4	Manba	3E-02	0.5

Undefined

Control			*Notch2*$^{tm1.1Ecan}$		
Gene	*p*	FC	Gene	*p*	FC
1810024B03Rik	4E-01	0.3	Fahd1	5E-01	0.3
Uxs1	1E-03	0.3	Mrpl9	3E-06	0.3
Nit1	8E-02	0.3	Trp53cor1	2E-01	0.3
Osbpl9	3E-07	0.3	Pxdc1	9E-01	0.3
2300009A05Rik	9E-01	0.3	Rpl7l1	3E-06	0.3
Dlx5	1E-01	0.3	Polr2g	5E-02	0.3
Plcd1	2E-03	0.3	Trim32	3E-07	0.3
Gm47271	3E-01	0.3	Celf4	6E-01	0.3
Nfxl1	9E-01	0.3	Mtln	1E-01	0.3
Slc25a26	5E-02	0.3	Rab39b	4E-01	0.3
4930430F08Rik	2E-01	0.3	Pkp4	7E-01	0.3
Agpat4	3E-01	0.3	Rpl9-ps6	7E-04	0.3
Ccdc47	2E-02	0.3	Wdr35	7E-02	0.3
Sept1	4E-01	0.3	Mzt1	3E-06	0.3
Mettl17	4E-01	0.3	Mrpl39	2E-03	0.3
Chadl	4E-02	0.3	Cox11	3E-03	0.3
Adgra3	3E-02	0.3	Zfp606	6E-08	0.3
Jam2	2E-01	0.3	B130055M24Rik	1E-02	0.3
Tada2a	2E-01	0.3	Gtpbp4	1E-01	0.3
Strbp	9E-06	0.3	1600002K03Rik	1E+00	0.3
Bcl2l1	6E-03	0.3	Zmym1	4E-05	0.3
Yars2	9E-01	0.3	Il15ra	2E-02	0.3
Nsun4	8E-05	0.3	Slc38a1	5E-03	0.3
Gm36975	1E+00	0.3	Tm9sf2	4E-01	0.3
Hyou1	7E-02	0.3	Nucb2	6E-02	0.3
Kif21a	5E-05	0.3	B230216N24Rik	7E-01	0.3
Abcc1	3E-01	0.3	Tmem250-ps	2E-05	0.3
Cspg4	8E-01	0.3	Fam136a	3E-05	0.3
Tsc22d1	1E-17	0.3	1700021F05Rik	8E-07	0.3
Ddit4	9E-01	0.3	2010001A14Rik	4E-02	0.3
Slc35d1	1E-01	0.3	Tmem181a	5E-08	0.3
Mboat2	1E-05	0.3	Rpl36a-ps1	9E-03	0.3
Lonp1	9E-01	0.3	Id2	7E-02	0.3
Gng5	2E-42	0.3	Chrdl1	8E-01	0.3
Tmx1	8E-03	0.3	Adgra3	8E-01	0.3
Mief1	2E-01	0.3	Zfyve21	2E-02	0.3
Skap2	4E-07	0.3	Mtm1	8E-02	0.3
Flrt1	2E-01	0.3	Naa80	6E-02	0.3
Slc22a15	3E-02	0.3	Nfe2l1	7E-01	0.3
Fam43a	2E-02	0.3	Sdf2l1	1E+00	0.3
Sema6a	1E-01	0.3	Inpp5b	6E-05	0.3
Gcsh	4E-01	0.3	Rnaset2b	4E-01	0.3
Tpbg	4E-02	0.3	Tk2	6E-03	0.3
Ints5	3E-01	0.3	Pdk1	1E-01	0.3
Tsc22d4	2E-06	0.3	Ankrd9	2E-01	0.3
Pno1	4E-01	0.3	Phgdh	6E-01	0.3
Xrcc6	5E-01	0.3	Nim1k	2E-01	0.3
Tspan18	1E-03	0.3	Ier2	2E-07	0.3
Hspa5	3E-31	0.3	Atg2a	2E-02	0.3
Cyb5r1	2E-03	0.3	Hspb1	3E-03	0.3
Adamts9	8E-02	0.3	Blzf1	1E-03	0.3
Gm16124	4E-02	0.3	Ulk4	9E-06	0.3
Ppp1r13b	4E-01	0.3	Phyhd1	8E-02	0.3
Rpl36a-ps1	4E-01	0.3	Wdr61	4E-04	0.3
Prkn	5E-04	0.3	Pno1	4E-01	0.3
Pam16	2E-04	0.3	Rida	1E-02	0.3
Mef2b	7E-01	0.3	Rabac1	1E-05	0.3
Thtpa	7E-01	0.3	4732471J01Rik	4E-03	0.3
Tbx15	3E-15	0.3	Tma16	9E-03	0.3
Rnaseh1	2E-01	0.3	Tmx1	4E-01	0.3
Pebp1	1E-19	0.3	Idua	1E-01	0.3
Bag4	5E-02	0.3	2210408F21Rik	1E-01	0.3
2310010J17Rik	6E-01	0.3	E2f5	1E-02	0.3
Stxbp5	2E-03	0.3	Rsl24d1	4E-06	0.3

Articular/Synovial Fibroblast

Control			*Notch2*$^{tm1.1Ecan}$		
Gene	*p*	FC	Gene	*p*	FC
Gm6710	1E-03	0.7	Glul	8E-06	0.6
Elovl6	6E-20	0.7	L3mbtl3	1E-09	0.6
Adam12	3E-03	0.7	Sestd1	9E-08	0.6
Peg13	1E-05	0.7	Sh3bp1	4E-05	0.6
Elmo2	2E-07	0.7	Rnf150	6E-15	0.6
Gas1	2E-62	0.7	Adam15	1E-15	0.6
Elmo1	2E-14	0.7	Ndrg1	5E-14	0.6
Fn1	9E-89	0.7	Zfp984	2E-02	0.6
Tnfsf12	1E-21	0.7	Numb	1E-12	0.6
Rbms1	9E-78	0.7	Pik3ip1	2E-11	0.6
Bicd1	2E-07	0.7	Cdo1	2E-12	0.6
Carns1	5E-06	0.7	Rab32	3E-05	0.6
A330076H08Rik	2E-10	0.7	Capn1	4E-06	0.6
Cand2	3E-04	0.7	P2rx4	8E-10	0.6
Dbn1	1E-18	0.7	Casp12	2E-09	0.6
Asap2	6E-13	0.7	Nrip1	2E-15	0.6
Mfsd1	4E-19	0.7	Asah1	9E-42	0.6
Il18	3E-06	0.7	Ccdc181	7E-02	0.6
Wbp1	6E-15	0.7	Plin3	4E-18	0.6
Cbr3	4E-11	0.7	Car5b	1E-07	0.6
Mfsd6	3E-03	0.7	Mpp2	6E-04	0.6
Osbpl3	3E-03	0.7	Mapkbp1	3E-02	0.6
Fam102b	2E-20	0.7	Tlr4	1E-08	0.6
Abca2	8E-05	0.7	Fgd4	1E-08	0.6
Phactr1	3E-14	0.7	Aldh1a1	2E-13	0.6
Nudt4	3E-28	0.7	Eya4	8E-33	0.6
Amdhd2	1E-16	0.7	Mapk7	4E-05	0.6
Dynlt1f	1E-07	0.7	Vcam1	5E-04	0.6
Spata6	6E-21	0.7	Grk5	8E-09	0.6
Sorcs2	5E-06	0.7	Vegfb	2E-12	0.6
Slc25a24	2E-13	0.7	Tnfaip6	4E-08	0.6
Slc25a1	9E-23	0.7	Cttnbp2	2E-06	0.6
Tmem123	2E-10	0.7	St5	1E-20	0.6
Rbm43	7E-08	0.7	Mid1ip1	1E-03	0.6
Gm28875	6E-07	0.7	Fzd4	1E-06	0.6
Comt	6E-37	0.7	Gypc	1E-04	0.6
Vps13b	9E-41	0.7	Nr6a1	2E-07	0.6
Scd2	4E-38	0.7	Cdkl2	4E-04	0.6
Lacc1	1E-04	0.7	Rras	6E-22	0.6
Atg4d	1E-03	0.7	Acaa2	1E-19	0.6
Zbtb4	2E-20	0.7	Lpar1	2E-14	0.6
Hs6st3	1E-03	0.7	Gng13	1E-04	0.6
Ppp1r18	3E-10	0.7	Accs	2E-03	0.6
Il3ra	3E-10	0.7	Pmp22	2E-26	0.6
Hspa12a	4E-04	0.7	Dnm3os	5E-19	0.6
Pdlim2	4E-34	0.7	Cdc42bpb	2E-11	0.6
Igsf8	6E-17	0.7	Depdc5	8E-09	0.6
Nampt	1E-15	0.7	Mamld1	3E-05	0.6
Arsa	1E-13	0.7	Lgals3bp	3E-13	0.6
Endod1	3E-08	0.7	Serping1	3E-09	0.6
G6pdx	1E-11	0.7	Hnrnpll	2E-08	0.6
Stat6	3E-07	0.7	Morc4	6E-03	0.6
Lamc1	5E-21	0.7	Rbm43	2E-06	0.6
Apba1	8E-11	0.7	Scrn1	8E-08	0.6
Rnf213	2E-09	0.7	Tgfbr2	3E-18	0.6
Efemp2	5E-60	0.7	Sfxn3	2E-12	0.6
Dtnbp1	1E-37	0.7	Mnt	6E-10	0.6
Serping1	2E-19	0.7	Cercam	3E-14	0.6
Asl	3E-09	0.6	Vegfc	1E-02	0.6
Herc6	1E-05	0.6	Tspan5	7E-13	0.6
Rdh5	6E-03	0.6	Wasl	7E-18	0.6
Triobp	1E-11	0.6	Slc15a4	7E-04	0.6
Dusp10	1E-06	0.6	Dnm1	2E-11	0.6
Suox	6E-05	0.6	Arhgef10	5E-11	0.6

Limb Mesenchyme						Chondrogenic						Fibroblast						Undefined						Articular/Synovial Fibroblast					
Control			$Notch2^{tm1.1Ecan}$			Control			$Notch2^{tm1.1Ecan}$			Control			$Notch2^{tm1.1Ecan}$			Control			$Notch2^{tm1.1Ecan}$			Control			$Notch2^{tm1.1Ecan}$		
Gene	*p*	FC	Gene	*p*	FC	Gene	*p*	FC	Gene	*p*	FC	Gene	*p*	FC	Gene	*p*	FC	Gene	*p*	FC	Gene	*p*	FC	Gene	*p*	FC	Gene	*p*	FC
Hdgfl3	4E-15	0.3	Svbp	1E-37	0.4	Dock4	6E-02	0.4	Slc22a15	1E-04	0.4	Atp11c	5E-07	0.4	Hspa4l	1E-07	0.5	Dhrs11	3E-01	0.3	Nr2c2ap	9E-01	0.3	Mettl27	2E-06	0.6	Zfp995	3E-03	0.6
Il34	2E-04	0.3	C2cd2	2E-03	0.4	Nacc2	2E-16	0.4	Paxbp1	2E-08	0.4	Nans	1E-05	0.4	Nup35	2E-10	0.5	Tom1l2	6E-05	0.3	Slc33a1	9E-02	0.3	Tlr2	2E-04	0.6	Rasl11a	4E-02	0.6
Ccdc711	3E-03	0.3	Stambpl1	1E-09	0.4	Thumpd3	5E-07	0.4	6030443J06Rik	1E-07	0.4	Enpp4	6E-04	0.4	Fkbp2	1E-25	0.5	Eef1e1	2E-01	0.3	Mrps28	2E-01	0.3	Clcn6	6E-06	0.6	Ap3m2	5E-05	0.6
Nme2	2E-127	0.3	Hdgfl3	4E-18	0.4	Mif	9E-50	0.4	Nr2c2ap	1E-07	0.4	Trim16	4E-04	0.4	Lacc1	5E-06	0.5	Hmgcll1	2E-03	0.3	Hoxa3	2E-01	0.3	Serac1	1E-06	0.6	Rgs17	3E-03	0.6
Ahnak	8E-73	0.3	Elovl1	7E-16	0.4	Sfxn2	1E-06	0.4	Tcea1	2E-51	0.4	Cep57	1E-08	0.4	Gas1	1E-36	0.5	Upf3b	4E-01	0.3	Usp2	5E-01	0.3	Platr25	2E-05	0.6	Gan	1E-04	0.6
Cldnd1	1E-18	0.3	Zdhhc7	2E-05	0.4	Gng11	2E-03	0.4	Mar5	2E-36	0.4	1500009L16Rik	1E-09	0.4	Mex3d	3E-03	0.5	Gm15050	7E-02	0.3	Dcn	2E-22	0.3	Chrd	2E-04	0.6	Fabp3	4E-08	0.6
Cthrc1	1E-08	0.3	Tstd3	5E-03	0.4	Nfatc1	5E-13	0.4	Srprb	2E-10	0.4	Cys1	1E-06	0.4	Ece2	1E-06	0.5	Ears2	3E-01	0.3	Nudt16l1	9E-06	0.3	Rab3d	9E-05	0.6	Hoxd8	2E-04	0.6
Epn2	4E-09	0.3	Pgpep1	7E-08	0.4	Twnk	3E-06	0.4	Prr7	2E-05	0.4	Gnb1l	8E-02	0.4	Slc26a11	3E-03	0.5	Tnfaip8	8E-03	0.3	Uhrf1bp1	1E-02	0.3	Aox1	2E-04	0.6	Ezh1	3E-04	0.6
Jun	2E-43	0.3	Schip1	6E-06	0.4	Mets2	4E-06	0.4	BC005561	2E-05	0.4	Stom	4E-04	0.4	Btbd8	5E-03	0.5	Slc41a2	2E-01	0.3	Cxxc1	2E-05	0.3	St5	2E-19	0.6	Efemp2	9E-37	0.6
Tspo	7E-46	0.3	Gcc1	3E-05	0.4	B230217O12Rik	1E-06	0.4	Ptpn4	6E-12	0.4	Glis2	1E-11	0.4	Zfp958	9E-04	0.5	Zfp316	2E-01	0.3	Ecsit	3E-04	0.3	Twf2	6E-10	0.6	Brpf3	5E-02	0.6
Sept6	1E-14	0.3	Numbl	2E-13	0.4	Flvcr1	1E-02	0.4	Dusp8	4E-04	0.4	St5	6E-10	0.4	Hdgf	2E-44	0.5	Ndufa4l2	1E-05	0.3	Ndor1	1E-01	0.3	Tollip	5E-07	0.6	Prkx	3E-04	0.6
Csk	3E-12	0.3	Dnajb1	4E-11	0.3	Foxc2	1E-04	0.4	Cdk6	7E-12	0.4	Taf1	5E-12	0.4	Gm4876	1E-07	0.5	Slc33a1	3E-01	0.3	Daam2	1E+00	0.3	2900005J15Rik	5E-03	0.6	Psen2	2E-05	0.6
Fam129a	8E-38	0.3	Comt	6E-24	0.3	2310010J17Rik	2E-13	0.4	Tm9sf2	1E-34	0.4	Ipo11	5E-08	0.4	Lpar6	2E-03	0.5	Gm11944	2E-02	0.3	B230118H07Rik	4E-01	0.3	Angptl2	5E-09	0.6	Prkag2	5E-05	0.6
Trim3	2E-04	0.3	Pten	7E-33	0.3	BC005561	2E-09	0.4	Ormdl3	2E-18	0.4	Hsp90aa1	6E-27	0.4	Specc1l	3E-10	0.5	Zswim4	2E-01	0.3	Fxyd5	5E-02	0.3	Nipa1	2E-05	0.6	Acyp2	3E-17	0.6
Ar	6E-07	0.3	Dock4	8E-15	0.3	Smim4	5E-18	0.4	Pigf	1E-06	0.4	Gm16364.1	6E-02	0.4	Kif6	8E-03	0.5	Zkscan8	3E-01	0.3	Pgam1	2E-01	0.3	Pld2	4E-06	0.6	Fbxw17	1E-08	0.6
Idi1	8E-09	0.3	Unc5b	2E-08	0.3	Sdr39u1	7E-13	0.4	Pde3a	2E-31	0.4	Tfdp2	6E-10	0.4	Ssna1	1E-19	0.5	Snx25	4E-01	0.3	Cep95	1E-03	0.3	Ppp1r9b	1E-05	0.6	Fbxo25	5E-09	0.6
Irf1	2E-01	0.3	Klf13	8E-24	0.3	Rab39b	2E-06	0.4	Acot8	2E-02	0.4	Ran	2E-14	0.4	Rab23	4E-10	0.5	Zfp869	5E-01	0.3	Gpx4	3E-22	0.3	Ikbke	3E-04	0.6	Zfyve26	3E-07	0.6
Pxk	4E-06	0.3	Zeb2	6E-33	0.3	Lin52	2E-06	0.4	Nomo1	2E-13	0.4	Lrrk2	3E-06	0.4	Hmgcs1	6E-16	0.5	Ppat	7E-01	0.3	Map3k20	5E-01	0.3	Heca	3E-08	0.6	Tnfsf12	3E-12	0.6
Copz2	4E-50	0.3	Plekhf1	1E-12	0.3	4921511C10Rik	1E-04	0.4	Pam16	5E-38	0.4	Svil	1E-15	0.4	Fxyd6	5E-16	0.5	Nr4a2	1E-02	0.3	C87436	3E-02	0.3	Mcub	9E-13	0.6	Dpysl2	5E-18	0.6
Anxa6	1E-20	0.3	Id4	4E-02	0.3	Sat1	1E-21	0.4	Gm16759	2E-04	0.4	Pcgf5	1E-05	0.4	Tspan12	1E-08	0.5	Fgfr1	6E-06	0.3	Tex2	4E-01	0.3	Casp3	4E-09	0.6	Cobll1	3E-08	0.6
Dyrk2	9E-05	0.3	Enc1	1E-06	0.3	Ahcyl2	2E-16	0.4	Krba1	1E-02	0.4	Mtmr11	5E-03	0.4	Brwd3	1E-06	0.5	Ptgis	7E-02	0.3	Pdzrn3	5E-04	0.3	Gm14636	6E-04	0.6	Ppm1f	2E-03	0.6
Shf	2E-03	0.3	Ada	1E-06	0.3	Nt5c3	2E-07	0.4	Ogg1	2E-03	0.4	Larp7	2E-08	0.4	Gsc	9E-05	0.5	Hivep2	1E-01	0.3	Ubxn2b	2E-02	0.3	Cep170	9E-21	0.6	Ap5z1	5E-03	0.6
Ggta1	4E-03	0.3	Ccdc25	8E-15	0.3	Rbm48	4E-05	0.4	Gm26797	1E-01	0.4	Cbx5	1E-16	0.4	Btbd11	3E-07	0.5	Zbtb38	1E-04	0.3	Ssx2ip	7E-02	0.3	Casp6	4E-07	0.6	Rab9	1E-22	0.6
Ankrd27	5E-08	0.3	Abhd11	3E-09	0.3	Ormdl3	1E-27	0.4	Slc3a2	2E-36	0.4	Nek8	4E-03	0.4	Lsm5	2E-19	0.5	Gm43672	3E-02	0.3	Prdx6	3E-03	0.3	Acap3	1E-04	0.6	Slc35d2	4E-02	0.6
Hspb1	2E-08	0.3	Gfra4	3E-05	0.3	Zfp598	2E-04	0.4	Prkdc	5E-10	0.4	Tab3	2E-04	0.4	Lss	2E-05	0.5	Cisd2	2E-03	0.3	Kiz	3E-02	0.3	Ifit2	9E-07	0.6	Scd2	1E-22	0.6
Wwp1	8E-18	0.3	Trip6	7E-16	0.3	Utp14a	1E-11	0.4	Hist1h1c	3E-22	0.4	Myof	9E-21	0.4	Taf4	4E-03	0.5	Psmg4	2E-01	0.3	Cnpy3	2E-01	0.3	Gm47071	8E-05	0.6	Rap2a	8E-12	0.6
Zc3hc1	4E-05	0.3	Scn2a	6E-05	0.3	5330438D12Rik	2E-11	0.4	Gnao1	1E-02	0.4	Sptbn1	5E-44	0.4	Tubgcp4	2E-09	0.5	Ppid	9E-01	0.3	Pacsin3	1E-02	0.3	Gpam	1E-02	0.6	Ercc4	2E-03	0.6
Flcn	1E-03	0.3	Smarcd2	6E-09	0.3	Riiad1	2E-04	0.4	Atg2b	1E-04	0.4	Nfyb	4E-06	0.4	Cnksr3	7E-09	0.5	Lgr4	7E-05	0.3	Klhdc1	5E-01	0.3	Capn1	6E-08	0.6	Inpp4b	1E-01	0.6
Dcun1d3	1E-05	0.3	Snhg17	7E-06	0.3	Paxbp1	4E-11	0.4	Enpp1	1E-09	0.4	Usp13	3E-04	0.4	Smad6	7E-14	0.5	Gm4876	4E-02	0.3	Hoxa11	2E-01	0.3	Mr1	7E-04	0.6	Foxo1	7E-11	0.6
Zfp120	8E-04	0.3	Tbc1d2b	2E-09	0.3	C030006K11Rik	3E-08	0.4	Mtg1	9E-03	0.4	Phldb1	3E-06	0.4	Pms1	4E-06	0.5	Dgke	3E-01	0.3	Ddt	2E-01	0.3	Pros1	6E-13	0.6	C1ra	2E-13	0.6
Pusl1	4E-07	0.3	Prdx4	7E-42	0.3	Nfu1	3E-38	0.4	Khk	1E-03	0.4	Rnf168	1E-06	0.4	Armc10	2E-03	0.5	a	7E-01	0.3	Snrpn	6E-01	0.3	Insyn1	3E-06	0.6	F2r	9E-04	0.6
Dnpep	2E-09	0.3	Rara	7E-15	0.3	Cpox	2E-10	0.4	Dleu2	3E-16	0.4	Dpysl2	1E-20	0.4	Phka2	7E-07	0.5	Pigb	1E-01	0.3	Hagh	5E-01	0.3	Dusp16	7E-13	0.6	Rmc1	3E-04	0.6
Elac2	4E-02	0.3	Runx2	6E-24	0.3	Cryzl2	2E-04	0.4	Slc41a2	1E-08	0.4	Tyw1	4E-03	0.4	Ppm1d	9E-08	0.5	Lrp5	9E-01	0.3	Papss1	6E-02	0.3	Fig4	4E-10	0.6	Gm11084	2E-04	0.6
Mtch1	1E-40	0.3	Mtx2	3E-21	0.3	Fmod	6E-51	0.4	Arhgef1	8E-14	0.4	Zfp626	5E-02	0.4	Synj2	8E-04	0.5	Entpd7	7E-01	0.3	Tdp1	6E-05	0.3	Aida	5E-23	0.6	Stk38	1E-16	0.6
Parva	6E-32	0.3	Aldh16a1	1E-04	0.3	Lrrc1	1E-06	0.4	Mdn1	5E-07	0.4	Cped1	3E-28	0.4	Stk35	1E-06	0.5	Men1	6E-03	0.3	Fkbp4	2E-05	0.3	Hivep1	2E-09	0.6	2900026A02Rik	2E-05	0.6
Slc41a3	2E-07	0.3	Zfp36l1	3E-10	0.3	Larp1b	8E-21	0.4	Ell2	2E-39	0.4	Lrrfip1	2E-17	0.4	Adgrl2	5E-21	0.5	Eno1	9E-07	0.3	Fam20c	4E-02	0.3	Ptprj	1E-09	0.6	Slc31a2	5E-05	0.6
Ifngr2	7E-12	0.3	Gdap2	2E-07	0.3	Tmem230	2E-19	0.4	Mageh1	8E-13	0.4	Itpr3	1E-08	0.4	Gstcd	1E-05	0.5	Ppp1r10	2E-03	0.3	Acox1	7E-01	0.3	Hmgcr	3E-11	0.6	Slc25a1	1E-17	0.6
Vangl1	1E-07	0.3	Gclm	1E-10	0.3	Gnpnat1	2E-13	0.4	5430416N02Rik	2E-07	0.4	Tmem98	4E-08	0.4	Snrpa1	7E-16	0.5	Hspa1a	1E-01	0.3	Rad1	2E-01	0.3	Tbc1d24	2E-03	0.6	Fasn	3E-12	0.6
Snhg16	5E-04	0.3	F8a	2E-04	0.3	1810009A15Rik	8E-06	0.4	Gars	4E-35	0.4	Noc4l	8E-02	0.4	Tdp2	2E-07	0.5	Fam126b	8E-02	0.3	Impa1	1E-04	0.3	Ankrd9	1E-02	0.6	Tle5	5E-40	0.6
Cav1	4E-29	0.3	Itih5	1E-06	0.3	Srm	4E-24	0.4	Tcf4	1E-61	0.4	Elmo2	1E-04	0.4	Zbed3	8E-08	0.5	Kazald1	6E-01	0.3	Tpbg	2E-02	0.3	Ociad2	9E-04	0.6	Basp1	6E-09	0.6
Zfp503	6E-15	0.3	Aldh3a2	4E-07	0.3	Limd1	4E-12	0.4	Tor3a	3E-05	0.4	Nme7	6E-06	0.4	Nsmce4a	2E-13	0.5	Crlf2	1E+00	0.3	Brix1	8E-02	0.3	Aph1b	7E-05	0.6	Shisa5	6E-17	0.6
Tpm4	2E-31	0.3	Ralb	6E-10	0.3	Arhgef2	2E-20	0.4	Fgfr2	7E-55	0.4	Mad2l1bp	1E-02	0.4	Wdr5	7E-03	0.5	Unk	2E-01	0.3	Chchd4	6E-02	0.3	Rp2	3E-05	0.6	Slc16a3	9E-02	0.6
Gm26802	2E-11	0.3	Plrg1	1E-06	0.3	Gm16599	5E-07	0.4	Prdm16	2E-11	0.4	Cd80	2E-05	0.4	Rab13	2E-10	0.5	Pdss2	3E-01	0.3	Bysl	1E-03	0.3	Slc25a42	2E-03	0.6	Nmi	1E-06	0.6
Ctsz	4E-50	0.3	Slc30a7	5E-14	0.3	Sgip1	2E-07	0.4	Neo1	3E-28	0.4	Pycard	7E-03	0.4	Zeb1	2E-22	0.5	P4ha2	3E-08	0.3	Dph6	4E-02	0.3	Nrip1	2E-22	0.6	Cys1	3E-05	0.6
Sema3f	2E-05	0.3	Psrc1	3E-05	0.3	Gm42726	1E-01	0.4	Npr2	3E-12	0.4	Sac3d1	8E-07	0.4	Plekha8	5E-03	0.5	E030030I06Rik	3E-01	0.3	Uchl3	2E-01	0.3	Jak1	3E-42	0.6	Fbxo30	1E-03	0.6
Prdm4	1E-02	0.3	Kctd13	5E-03	0.3	Trmt10a	3E-07	0.4	Tmem238	9E-14	0.4	Uaca	2E-04	0.4	Haus8	7E-11	0.5	St3gal4	6E-03	0.3	Borcs8	1E-04	0.3	Fgd4	2E-13	0.6	Col6a2	2E-38	0.6
Ppp1r12a	4E-28	0.3	Naaa	4E-06	0.3	Syn3	8E-27	0.4	Anxa11	2E-08	0.4	Sdf2l1	4E-06	0.4	Ccdc22	3E-05	0.5	Bola1	4E-02	0.3	Ncl	9E-05	0.3	Thrb	3E-06	0.6	Tmod3	4E-12	0.6
Garem1	5E-05	0.3	Snhg5	3E-07	0.3	Limk2	7E-05	0.4	Xrcc6	2E-06	0.4	Esr1	1E-06	0.4	Col3a1	6E-77	0.5	1600002K03Rik	1E-02	0.3	Mir99ahg	3E-07	0.3	Sdccag8	7E-21	0.6	Meis1	3E-06	0.6
Tax1bp3	7E-17	0.3	Sertad4	1E-09	0.3	Sox11	9E-11	0.4	Ndufaf4	2E-12	0.4	Slc25a53	3E-02	0.4	Ldlr	2E-17	0.5	Lpin2	9E-01	0.3	Gm4221	6E-02	0.3	Trio	9E-25	0.6	Prxl2a	2E-05	0.6
Ggt7	1E-07	0.3	Rap1gap2	4E-03	0.3	Fam131a	9E-04	0.4	Stard5	2E-06	0.4	Ptpre	2E-04	0.4	Golm1	3E-08	0.5	Tmed1	1E-02	0.3	Ndufb3	2E-03	0.3	Slc9a1	2E-07	0.6	Jak1	9E-26	0.6
Msrb1	2E-14	0.3	Stat6	5E-09	0.3	Bcl2l1	2E-10	0.4	Kantr	2E-10	0.4	Shisa4	9E-13	0.4	Elmo2	9E-06	0.5	Ppan	1E+00	0.3	2410006H16Rik	2E-07	0.3	Cyp2j6	2E-05	0.6	Gm49692	9E-02	0.6
Klf13	4E-19	0.3	Zfp503	1E-14	0.3	Banp	4E-05	0.4	Bzw2	3E-25	0.4	Bub3	2E-06	0.4	Col6a2	6E-45	0.5	Smim27	4E-02	0.3	Oxld1	2E-01	0.3	Nrp1	3E-35	0.6	Rps6kc1	4E-09	0.6
Gstt3	1E-03	0.3	Esyt2	3E-15	0.3	Neo1	3E-34	0.4	Siah2	1E-06	0.4	Lgals3	4E-28	0.4	Mrps11	6E-11	0.5	Coq3	2E-01	0.3	Hsd17b10	1E-01	0.3	S100a11	2E-93	0.6	Evi5	9E-12	0.6
Pabpc4l	8E-05	0.3	Utp23	6E-08	0.3	Loxl2	1E-09	0.4	Larp1b	8E-09	0.4	Lss	6E-04	0.4	Pik3c3	2E-07	0.5	Armcx6	2E-01	0.3	Dhtkd1	3E-01	0.3	Klf3	3E-28	0.6	Ggt7	2E-04	0.6
Rras	7E-17	0.3	Sytl2	7E-05	0.3	Hadh	5E-28	0.4	9630028H03Rik	5E-07	0.4	Mmgt1	3E-04	0.4	Eno2	2E-02	0.5	Etl4	1E-05	0.3	Exosc3	3E-01	0.3	Dpp3	4E-11	0.6	Sntb2	1E-30	0.6
Bak1	5E-08	0.3	Prelid1	4E-46	0.3	Cmtm4	2E-07	0.4	Mrps28	1E-25	0.4	Hsd17b7	4E-06	0.4	Rpa3	9E-06	0.5	Lrrc4	2E-01	0.3	Cyp4f13	3E-01	0.3	Frrs1	2E-08	0.6	Agpat3	5E-16	0.6
Zfp46	2E-03	0.3	Dnajc14	9E-09	0.3	Oard1	4E-07	0.4	Timmdc1	4E-09	0.4	Wbp1l	9E-06	0.4	Tap2	7E-05	0.5	Lrba	3E-01	0.3	Mtmr3	5E-02	0.3	Tbc1d9	1E-05	0.6	Tstd3	6E-03	0.6
1110046J04Rik	1E-06	0.3	Cast	8E-26	0.3	Rfc3	4E-06	0.4	Gm19705	1E-01	0.4	Gpr19	8E-03	0.4	Zmym1	1E-10	0.5	Tvp23b	3E-01	0.3	Coq2	5E-02	0.3	Rras	2E-27	0.6	Galk2	4E-20	0.6
Junb	5E-24	0.3	Abcb10	6E-04	0.3	Nol3	3E-07	0.4	Tanc2	4E-19	0.4	Hmgn3	2E-27	0.4	Itih2	5E-07	0.5	Apbb2	5E-08	0.3	Neat1	3E-11	0.3	Wdr81	2E-03	0.6	Traf5	6E-02	0.6
Hoxd8	1E-04	0.3	Slc39a13	2E-19	0.3	Foxo3	2E-12	0.4	Lonp1	2E-15	0.4	Samd9l	3E-08	0.4	Inip	4E-04	0.5	Creb3l2	7E-05	0.3	Bphl	2E-03	0.3	Lamb2	1E-21	0.6	Mfsd1	3E-14	0.6
Prickle1	3E-03	0.3	Slc9a9	7E-13	0.3	Hspe1	3E-108	0.4	Nfu1	2E-35	0.4	Pknox2	1E-05	0.4	Nelfe	2E-10	0.5	Sppl2a	5E-04	0.3	Klf12	8E-02	0.3	Ddit4l	1E-04	0.6	Casp7	6E-03	0.6
Mospd3	2E-05	0.3	Olfml2b	1E-25	0.3	Nprl3	7E-06	0.4	Clpb	8E-08	0.4	Creld1	5E-03	0.4	Gab2	3E-14	0.5	Hspe1	3E-21	0.3	Borcs6	4E-07	0.3	Cavin3	3E-57	0.6	Cavin3	1E-36	0.6
Washc3	1E-19	0.3	Fkbp1b	1E-04	0.3	Slc25a37	2E-05	0.4	Sar1b	4E-30	0.4	Mettl21a	1E-03	0.4	Rassf3	4E-08	0.5	Ptcd3	7E-01	0.3	Dcun1d2	9E-05	0.3	Ostm1	3E-07	0.6	Clip4	6E-07	0.6
Arhgap29	6E-20	0.3	Rffl	3E-03	0.3	Gm10762	2E-04	0.4	Tmem230	4E-16	0.4	Prpf31	2E-06	0.4	Myo1b	8E-09	0.5	Klhl21	2E-01	0.3	Slc7a5	2E-01	0.3	Man2b2	3E-10	0.6	Rhbdf1	3E-08	0.6

Limb Mesenchyme					
Control			*Notch2*$^{tm1.1Ecan}$		
Gene	*p*	FC	Gene	*p*	FC
Tbc1d10b	7E-06	0.3	Arrdc4	1E-08	0.3
Wdr55	4E-03	0.3	Fstl3	5E-05	0.3
Nudt7	1E-04	0.3	Cttn	2E-22	0.3
Mpv17	6E-11	0.3	Nthl1	6E-09	0.3
Fpgs	3E-03	0.3	Col5a2	2E-69	0.3
Lox	3E-74	0.3	Ostf1	5E-34	0.3
Prlr	2E-02	0.3	Mpp1	4E-17	0.3
St5	2E-06	0.3	Dact3	5E-10	0.3
Tmem150a	2E-08	0.3	Parp3	4E-24	0.3
Flna	3E-22	0.3	Syde1	3E-11	0.3
Lpar6	8E-03	0.3	Puf60	4E-18	0.3
Rcn3	4E-53	0.3	Hoxd8	1E-07	0.3
Phgdh	3E-25	0.3	Aida	4E-14	0.3
Fdft1	1E-15	0.3	Col16a1	1E-09	0.3
Cc2d1a	2E-03	0.3	Angptl1	3E-01	0.3
Ripor1	3E-07	0.3	Layn	2E-10	0.3
Zfp286	2E-03	0.3	Hmga2	1E-28	0.3
Fancf	3E-04	0.3	Ripor1	6E-10	0.3
Nsmf	3E-04	0.3	Rnf149	4E-08	0.3
Mest	2E-30	0.3	Klf6	8E-27	0.3
Cad	6E-04	0.3	Capn2	1E-20	0.3
Mlf1	2E-06	0.3	Mrps11	1E-09	0.3
Mar9	1E-04	0.3	Wwp1	2E-14	0.3
Timm10	5E-08	0.3	Sept6	8E-11	0.3
Trip6	5E-11	0.3	Gm14296	1E-03	0.3
S100a11	4E-75	0.3	Clec2d	1E-11	0.3
Glis3	6E-07	0.3	Tgds	7E-08	0.3
Eif4e3	7E-04	0.3	Zfp281	3E-05	0.3
Ap5s1	6E-04	0.3	Pdgfra	2E-17	0.3
Hs6st2	7E-17	0.3	Txnip	4E-17	0.3
Snhg6	3E-20	0.3	B4galt2	9E-11	0.3
Mtpn	1E-37	0.3	Zfp467	9E-03	0.3
Coa4	2E-01	0.3	Dusp19	1E-04	0.3
Rara	2E-13	0.3	Dipk1a	6E-06	0.3
Selenon	1E-10	0.3	Mypop	2E-07	0.3
Cask	2E-22	0.3	Kank2	8E-15	0.3
Mpdz	2E-17	0.3	Tmod2	2E-05	0.3
Klf6	9E-24	0.3	Gab2	6E-11	0.3
Comt	1E-20	0.3	Myo7a	5E-07	0.3
Map1a	2E-10	0.3	Plag1	1E-03	0.3
Tmem185b	2E-02	0.3	Filip1l	1E-10	0.3
Pycr1	2E-17	0.3	Myof	1E-23	0.3
Samd9l	2E-06	0.3	Dpagt1	1E-08	0.3
Mzt2	1E-06	0.3	Slc22a18	6E-05	0.3
Tmem14a	9E-07	0.3	Galk2	2E-19	0.3
Nagpa	3E-03	0.3	Imp4	6E-09	0.3
Myd88	4E-05	0.3	Cd59a	3E-02	0.3
Cyb5r3	3E-35	0.3	Rhoc	2E-45	0.3
Fam171a1	1E-09	0.3	Tsta3	6E-11	0.3
Ccndbp1	6E-06	0.3	Fbxl6	3E-09	0.3
Sept7	8E-61	0.3	Smco4	2E-15	0.3
Jak1	5E-25	0.3	Lztr1	3E-08	0.3
Abhd14b	3E-07	0.3	B4galt1	2E-22	0.3
Mat2a	3E-17	0.3	Jak1	2E-29	0.3
Elovl5	2E-07	0.3	Csk	3E-18	0.3
Cnppd1	2E-06	0.3	St3gal5	4E-14	0.3
Clip3	5E-08	0.3	Tspan4	3E-29	0.3
Rab21	4E-27	0.3	Prcp	1E-06	0.3
Orai3	5E-05	0.3	Prelid2	1E-05	0.3
Gm14325	3E-09	0.3	Ttc21b	3E-03	0.3
Itm2b	4E-83	0.3	Mettl22	1E-04	0.3
Pi15	6E-09	0.3	Nagk	7E-17	0.3
Gtpbp10	5E-02	0.3	Flcn	5E-05	0.3
Aida	1E-12	0.3	Tmem258	1E-40	0.3

Chondrogenic					
Control			*Notch2*$^{tm1.1Ecan}$		
Gene	*p*	FC	Gene	*p*	FC
Mipol1	3E-13	0.4	Ppp3ca	2E-50	0.4
Mtln	3E-17	0.4	Slc25a23	4E-03	0.4
Mbnl2	1E-77	0.4	Usp11	2E-07	0.4
Pycr1	4E-18	0.4	Epb41l4aos	8E-16	0.4
Gm17056	2E-05	0.4	Igip	2E-04	0.4
Gfod2	2E-02	0.4	Fbxl12	1E-05	0.4
Saraf	2E-40	0.4	Lrrc28	3E-08	0.4
Snrnp35	7E-03	0.4	Sat1	1E-25	0.4
Hprt	4E-20	0.4	Ears2	2E-04	0.4
Dusp12	3E-12	0.4	Ebna1bp2	2E-16	0.4
Wars2	1E-13	0.4	Prss36	2E-09	0.4
2300009A05Rik	5E-16	0.4	Ptpn14	1E-21	0.4
Gm35188	7E-09	0.4	Arhgef2	1E-16	0.4
Cdh19	1E-71	0.4	Mdm2	3E-13	0.4
Pik3cb	8E-09	0.4	Abhd6	3E-06	0.4
Ruvbl2	1E-06	0.4	Orai2	2E-03	0.4
Xpr1	3E-11	0.4	4933431E20Rik	7E-06	0.4
E530011L22Rik	1E-03	0.4	Smim8	3E-12	0.4
Btg3	8E-29	0.4	Ivd	3E-09	0.4
Auts2	4E-49	0.4	Mir99ahg	4E-49	0.4
Jph1	6E-07	0.4	Hprt	2E-22	0.4
Arhgef1	9E-10	0.4	Tcaim	4E-04	0.4
2210408F21Rik	9E-09	0.4	Smpdl3b	6E-05	0.4
Atp6v1g1	3E-65	0.4	Rab39b	3E-04	0.4
P4ha1	2E-54	0.4	Foxc1	4E-37	0.4
Polr2d	8E-15	0.4	Enc1	2E-04	0.4
Mid2	1E-05	0.4	Eprs	1E-67	0.4
Slc3a2	3E-32	0.4	Mafg	4E-14	0.4
Stx3	3E-06	0.4	Dek	1E-45	0.4
Smo	3E-24	0.4	Ficd	3E-03	0.4
Cdk2ap1	4E-14	0.4	Bambi	4E-03	0.4
4930550C14Rik	5E-04	0.4	Clmn	6E-06	0.4
Adpgk	2E-05	0.4	Fxyd6	4E-11	0.4
Alg3	3E-04	0.4	Tmsb15b2	2E-04	0.4
Adamts9	8E-12	0.4	Nfxl1	1E-02	0.4
Pdk1	4E-07	0.4	Gm11944	2E-03	0.4
Mafg	5E-14	0.4	Elmsan1	5E-11	0.4
Neat1	3E-51	0.4	Fam180a	6E-10	0.4
Veph1	3E-07	0.4	Il1rapl1	3E-33	0.4
Mphosph10	1E-13	0.4	Pigv	2E-03	0.4
Gm15952	1E-02	0.4	Ica1	3E-05	0.4
Snhg3	9E-17	0.4	Arl14ep	1E-15	0.4
Slc41a2	1E-12	0.4	Pdcd4	1E-35	0.4
Ganab	4E-14	0.4	Ror2	4E-07	0.4
Vamp5	2E-22	0.4	Ddrgk1	8E-30	0.4
Cab39	2E-14	0.4	Banp	7E-08	0.4
Rtl3	1E-13	0.4	Skap2	4E-20	0.4
Ebna1bp2	2E-23	0.4	Ecpas	6E-26	0.4
1110002L01Rik	9E-07	0.4	Utp4	3E-03	0.4
Atf6	1E-14	0.4	Stard10	2E-11	0.4
Kantr	8E-07	0.4	Rian	3E-20	0.4
Nt5dc2	2E-02	0.4	Lman2l	9E-03	0.4
Lmcd1	8E-17	0.4	Gnl3l	2E-09	0.4
Hs2st1	2E-19	0.4	Btg2	3E-36	0.4
Tcea1	2E-51	0.4	Gm15050	2E-02	0.4
Gpr153	1E-05	0.4	Mapre3	1E-04	0.4
Angpt1	7E-18	0.4	Mccc2	8E-05	0.4
Psmg4	1E-20	0.4	Adat1	3E-05	0.4
Sept9	8E-15	0.4	Tspan18	2E-09	0.4
Ptch1	2E-10	0.4	Emc6	4E-24	0.4
Ggct	1E-03	0.4	Znhit3	1E-03	0.4
Pgam1	2E-44	0.4	Sept9	2E-12	0.4
Atp1a1	1E-29	0.4	Ehd3	1E-05	0.4
Znrf3	6E-09	0.4	Klf12	2E-20	0.4

Fibroblast					
Control			*Notch2*$^{tm1.1Ecan}$		
Gene	*p*	FC	Gene	*p*	FC
Cdkn2a	7E-08	0.4	Zfyve1	7E-07	0.5
Zdhhc13	4E-04	0.4	Snrnp25	9E-09	0.5
Pot1a	5E-05	0.4	Rab40b	8E-04	0.5
Tbc1d9	1E-02	0.4	Dis3	1E-07	0.5
Tm7sf2	3E-06	0.4	Smc3	2E-17	0.5
Fam219b	3E-01	0.4	Dapk1	7E-12	0.5
Trim37	3E-08	0.4	Uvrag	3E-20	0.5
Cavin3	3E-33	0.4	Mrpl49	1E-06	0.5
Platr25	5E-06	0.4	Fkbpl	1E-05	0.5
Kat7	9E-07	0.4	Lrrk2	5E-04	0.5
Heg1	3E-05	0.4	Gas2	3E-34	0.5
Trp63	1E-03	0.4	Ankrd29	3E-10	0.5
Man1c1	2E-07	0.4	Acad9	1E-01	0.5
Specc1l	6E-09	0.4	Camkmt	5E-07	0.5
Casp8ap2	2E-05	0.4	Arsj	6E-07	0.5
Slx4	3E-03	0.4	Tgfb3	3E-18	0.5
Msmo1	9E-15	0.4	Cd80	1E-05	0.5
Wdr47	2E-04	0.4	Lncppara	3E-19	0.5
Baiap2l1	3E-04	0.4	Rpap1	1E-04	0.5
Cyb5r3	9E-29	0.4	Pbx3	5E-15	0.5
Cav2	5E-07	0.4	Cyth3	7E-25	0.5
Adamtsl4	2E-09	0.4	Zw10	2E-06	0.5
Ubald2	3E-04	0.4	Trim37	2E-11	0.5
Syt11	3E-07	0.4	Plekhg2	2E-09	0.5
Gas1	5E-23	0.4	Nudc	5E-20	0.5
Nectin1	1E-03	0.4	Hdac6	1E-04	0.5
Cd44	6E-08	0.4	Ankrd26	8E-06	0.5
Jup	2E-03	0.4	Crip2	3E-30	0.5
Katnbl1	2E-05	0.4	Ctps	6E-11	0.5
Paxip1	2E-03	0.4	Clcc1	4E-13	0.5
Cyth3	2E-19	0.4	Ptbp3	8E-12	0.5
Arhgap29	1E-15	0.4	Ermp1	4E-04	0.5
Diaph1	2E-11	0.4	Catsper2	5E-03	0.5
Zmynd19	4E-04	0.4	Vps36	8E-16	0.5
Smc6	3E-16	0.4	Bst2	1E-02	0.5
Col6a2	5E-30	0.4	Cyp51	3E-20	0.5
Cnot6l	4E-09	0.4	Eng	6E-14	0.5
Ipo9	4E-06	0.4	Gm44686	4E-02	0.5
Smad6	4E-17	0.4	Ebf2	5E-09	0.5
Hist1h4d	1E-02	0.4	Aldh16a1	5E-05	0.5
Zfp385a	6E-06	0.4	Stip1	7E-16	0.5
Dxo	1E-03	0.4	Phf6	2E-11	0.5
Mcph1	2E-06	0.4	Elovl5	1E-06	0.5
Uevld	1E-03	0.4	Nap1l1	2E-29	0.5
Nudt18	9E-05	0.4	Zcchc8	2E-07	0.4
Il20rb	1E-02	0.4	Mis12	4E-06	0.4
Olfm1	1E-06	0.4	Cby1	7E-09	0.4
Gstm1	2E-14	0.4	Ighmbp2	2E-03	0.4
Ptpdc1	5E-03	0.4	Rnf168	2E-10	0.4
Lmcd1	4E-12	0.4	Mturn	6E-06	0.4
Ccdc167	6E-07	0.4	Ptpdc1	1E-04	0.4
Spidr	2E-04	0.4	Lyst	2E-14	0.4
Dag1	8E-31	0.4	Abcc1	3E-13	0.4
Hmgcs1	9E-13	0.4	Ahcyl1	5E-22	0.4
Parp9	5E-04	0.4	Osr1	2E-08	0.4
Arhgef10	2E-04	0.4	2310014F06Rik	2E-02	0.4
Adamtsl5	1E-08	0.4	Zmym3	6E-03	0.4
Taf6l	2E-02	0.4	Tfdp1	6E-06	0.4
Cdo1	6E-17	0.4	Nr6a1os	1E-02	0.4
Actn4	2E-25	0.4	Trp63	1E-04	0.4
Myo7a	3E-02	0.4	Cnih4	2E-20	0.4
2810402E24Rik	1E-02	0.4	Elf4	5E-11	0.4
Ogn	9E-23	0.4	Tnfrsf12a	6E-13	0.4
Rapgef1	2E-10	0.4	Sgms2	4E-10	0.4

Undefined					
Control			*Notch2*$^{tm1.1Ecan}$		
Gene	*p*	FC	Gene	*p*	FC
Slc39a1	3E-07	0.3	Rab11fip3	3E-01	0.3
Fancc	2E-01	0.3	Ccdc77	2E-02	0.3
Pdia6	1E-23	0.3	Cab39l	2E-01	0.3
Gde1	7E-01	0.3	Tns3	3E-02	0.3
Ube2g2	1E+00	0.3	BC031181	6E-01	0.3
Cutc	9E-01	0.3	Ganab	1E-02	0.3
Nrp2	1E-15	0.3	Eif2b5	3E-07	0.3
Gnpnat1	3E-01	0.3	Zfp507	2E-07	0.3
Cep63	2E-01	0.3	Smim19	7E-03	0.3
Txndc11	2E-01	0.3	Naa10	5E-04	0.3
Btg2	9E-12	0.3	Wasf3	1E-02	0.3
Ddx21	2E-03	0.3	Vdac3	5E-01	0.3
B230216N24Rik	1E-01	0.3	Cd151	5E-01	0.3
Mrps31	7E-02	0.3	Prlr	1E-01	0.3
Msrb2	7E-01	0.3	Tvp23b	6E-02	0.3
1810026B05Rik	9E-02	0.3	Wdr74	2E-03	0.3
Sft2d3	8E-01	0.3	2610020C07Rik	9E-03	0.3
Card19	5E-02	0.3	Anxa4	1E-01	0.3
Wdr34	8E-01	0.3	Pgrmc2	2E-02	0.3
Rpa1	3E-01	0.3	Zfp365	2E-03	0.3
Phpt1	1E-01	0.3	Foxc1	5E-01	0.3
Jak2	2E-01	0.3	Ptpn2	3E-05	0.3
Gstcd	3E-01	0.3	Zfyve27	2E-02	0.3
Stau2	4E-01	0.3	Nol3	1E-01	0.3
Lncpint	7E-02	0.3	Atg3	2E-04	0.3
Hist1h4i	3E-01	0.3	Nars	8E-01	0.3
Dipk2a	6E-03	0.3	Pde10a	6E-06	0.3
Actr6	5E-01	0.3	Tubb2b	3E-01	0.3
Cxcl16	2E-01	0.3	Rps27a	6E-59	0.3
Zfp329	2E-01	0.3	Nit1	1E-05	0.3
Slc39a11	2E-01	0.3	Sipa1l1	4E-01	0.3
Rpl9-ps6	8E-01	0.3	Rnf181	3E-03	0.3
Bloc1s1	4E-02	0.3	Ggact	6E-03	0.3
Hoxa9	2E-03	0.3	Tmem60	1E-01	0.3
Mtln	2E-02	0.3	Psma2	2E-04	0.3
Zrsr1	7E-01	0.3	Adam22	2E-01	0.3
Aldh1l2	3E-01	0.3	Rcbtb1	3E-07	0.3
Dnajc9	3E-01	0.3	Pccb	5E-05	0.3
1600010M07Rik	9E-01	0.3	Dbp	1E-02	0.3
Usp24	2E-02	0.3	Timm10	2E-04	0.3
Snhg20	7E-01	0.3	Mdh1	4E-04	0.3
Usp53	3E-01	0.3	P4ha1	2E-02	0.3
Siah2	3E-01	0.3	Qsox2	3E-02	0.3
Ncoa3	6E-01	0.3	Tefm	4E-01	0.3
Mrps27	2E-01	0.3	Kmt5a	3E-04	0.3
Ssr3	1E-11	0.3	Tnfaip8	2E-02	0.3
Traf3ip2	3E-02	0.3	Skp1a	1E-01	0.3
Gpsm2	3E-01	0.3	Nop10	5E-02	0.3
B4galt7	8E-01	0.3	Acaa1a	3E-02	0.3
Lage3	3E-01	0.3	Atp5g1	3E-01	0.3
Fzd3	7E-01	0.3	Ppa1	4E-03	0.3
1700024B18Rik	3E-03	0.3	Prrg2	4E-03	0.3
Tomm7	5E-12	0.3	Zbtb8os	2E-01	0.3
Castor1	1E-02	0.3	Spred1	3E-02	0.3
5031425E22Rik	9E-05	0.3	Phykpl	2E-06	0.3
Ube4b	4E-02	0.3	Mab21l2	4E-01	0.3
Jmjd8	1E+00	0.3	Jade2	9E-01	0.3
Atpsckmt	4E-01	0.3	Alpk1	2E-06	0.3
Ptpn4	3E-03	0.3	Sil1	1E-01	0.3
Slc25a40	4E-01	0.3	Hmces	4E-04	0.3
Cisd3	5E-01	0.3	4930581F22Rik	1E-01	0.3
Alkbh1	5E-01	0.3	Ecpas	3E-01	0.3
Adsl	5E-01	0.3	Pik3ip1	2E-06	0.3
Ocel1	3E-01	0.3	Zfp330	4E-03	0.3

Articular/Synovial Fibroblast					
Control			*Notch2*$^{tm1.1Ecan}$		
Gene	*p*	FC	Gene	*p*	FC
Tgfbr2	6E-37	0.6	Kcnd2	2E-04	0.6
Eya4	1E-39	0.6	Dubr	5E-05	0.6
Nr6a1	8E-13	0.6	Slc52a2	4E-02	0.6
Cavin1	3E-43	0.6	Hif1a	6E-39	0.6
S1pr2	7E-10	0.6	Nhlrc3	1E-04	0.6
Gm12743	1E-07	0.6	Pnpla7	5E-02	0.6
Pcyt2	2E-16	0.6	Ccdc22	4E-04	0.6
Zdhhc7	6E-05	0.6	Cpt1a	6E-05	0.6
Fgf18	5E-04	0.6	Txndc16	2E-05	0.6
Rgs12	1E-06	0.6	Adprh	1E-11	0.6
Daam1	5E-20	0.6	Prr13	7E-15	0.6
Sestd1	7E-12	0.6	Prnp	1E-33	0.6
Cd109	3E-15	0.6	Tmed8	2E-02	0.6
Tle5	1E-61	0.6	Itgb1bp1	7E-12	0.6
Tecpr1	2E-08	0.6	Sgsh	3E-03	0.6
Gpr137b	9E-11	0.6	Plxna1	1E-04	0.6
Eif3e	1E-66	0.6	Orai3	4E-03	0.6
Tgfbr3	5E-21	0.6	Map1a	1E-08	0.6
Sfxn3	2E-08	0.6	Neu1	7E-10	0.6
Maml3	9E-07	0.6	Pdxk	7E-06	0.6
Pnp	4E-27	0.6	Zmiz2	2E-04	0.6
Arsb	3E-12	0.6	Kif1c	3E-08	0.6
Rbms3	1E-50	0.6	Vps4b	9E-15	0.6
Antxr1	1E-38	0.6	Eda2r	5E-02	0.6
Mpdz	3E-27	0.6	Ier3	1E-09	0.6
Sh3bp1	3E-04	0.6	Retreg3	8E-04	0.6
Lpar1	2E-20	0.6	Psrc1	1E-02	0.6
Tmem65	4E-17	0.6	Il17ra	1E-04	0.6
Gamt	5E-16	0.6	P4htm	4E-03	0.6
Tns1	9E-31	0.6	Cep170	4E-13	0.6
Tmem43	2E-07	0.6	Pik3r2	5E-08	0.6
Txnrd3	3E-06	0.6	Ppp2r5b	8E-03	0.6
Hacl1	1E-04	0.6	Grik5	5E-05	0.6
Cpt1a	2E-11	0.6	Abr	2E-03	0.6
Bmper	9E-03	0.6	Mex3b	1E-01	0.6
Tnfrsf1b	1E-02	0.6	Irf9	4E-03	0.5
Tmem184b	3E-08	0.6	Wdr47	1E-04	0.5
Prdx5	1E-36	0.6	Epn2	8E-10	0.5
Klc1	2E-25	0.6	Hivep1	1E-04	0.5
Iqsec2	3E-12	0.6	Mmab	4E-04	0.5
Asah1	8E-48	0.6	Tmem126b	3E-03	0.5
Cacna2d1	1E-11	0.6	Nxpe4	5E-12	0.5
Zfp760	1E-04	0.6	Lrp1	4E-50	0.5
Arhgap10	1E-19	0.6	Ephb3	2E-06	0.5
Lgals1	6E-85	0.6	Mfhas1	4E-04	0.5
Pcdh18	8E-13	0.6	Ccdc80	4E-32	0.5
Smurf1	2E-09	0.6	Il3ra	2E-05	0.5
Gdap2	4E-05	0.6	Sdccag8	1E-13	0.5
Flt3l	1E-03	0.6	Il6st	2E-24	0.5
Zfp704	6E-15	0.6	Stxbp1	9E-05	0.5
Snap29	9E-04	0.6	Nudt7	1E-03	0.5
Pfkp	1E-06	0.6	Rap1gap2	3E-03	0.5
Dnm1	6E-17	0.6	Zfp518a	3E-02	0.5
Nfatc4	7E-08	0.6	Ankrd29	1E-02	0.5
Rnf150	1E-16	0.6	Nectin2	8E-03	0.5
Cast	2E-42	0.6	AC149090.1	3E-08	0.5
Rab29	2E-03	0.6	Pim1	9E-04	0.5
Tnfrsf11b	7E-01	0.6	Gm13470	1E-02	0.5
Vim	3E-100	0.6	Syne2	1E-04	0.5
Galnt4	3E-05	0.6	Ccdc141	2E-02	0.5
Gypc	2E-10	0.6	Bclaf3	5E-06	0.5
Klhl8	1E-02	0.6	Ptpn3	9E-04	0.5
A230057D06Rik	1E-09	0.6	Gm9801	3E-04	0.5
Agfg2	8E-06	0.6	Magee1	1E-04	0.5

Limb Mesenchyme

Control Gene	p	FC	*Notch2*$^{tm1.1Ecan}$ Gene	p	FC
Hdac4	2E-07	0.3	Tbc1d13	1E-04	0.3
Crip1	2E-35	0.3	Calm1	7E-55	0.3
Lynx1	1E-04	0.3	Selenon	1E-13	0.3
Chpf	5E-12	0.3	Comtd1	3E-11	0.3
Mdga1	4E-06	0.3	Coa5	6E-10	0.3
Pycrl	4E-04	0.3	Purg	1E-08	0.3
Tcf7l2	4E-16	0.3	Fam207a	2E-06	0.3
Irx3	3E-07	0.3	Insig1	2E-09	0.3
Aspn	2E-60	0.3	Dnpep	7E-20	0.3
Gnao1	3E-03	0.3	Tax1bp3	1E-18	0.3
Pgpep1	1E-06	0.3	Inf2	3E-09	0.3
Pdha1	8E-23	0.3	Agps	3E-11	0.3
Zfp800	1E-04	0.3	Trerf1	3E-11	0.3
Oaf	6E-11	0.3	S1pr2	2E-04	0.3
Fbxo6	7E-07	0.3	Parp12	3E-05	0.3
Sfxn3	5E-09	0.3	Loxl2	2E-08	0.3
Ssbp4	3E-10	0.3	Nav1	2E-07	0.3
Pced1b	2E-04	0.3	Gm47863	6E-03	0.3
Flrt2	1E-37	0.3	Wwc2	9E-26	0.3
Smurf1	1E-08	0.3	Rras	2E-19	0.3
Prss23	9E-42	0.3	Tuba1a	2E-40	0.3
AU022252	3E-05	0.3	Sept8	8E-15	0.3
Lipa	3E-17	0.3	Enox1	4E-12	0.3
Zfp27	5E-03	0.3	Pex16	6E-08	0.3
Ptprm	3E-23	0.3	Med10	2E-13	0.3
Slc22a18	8E-03	0.3	Spred2	7E-07	0.3
Myadm	2E-12	0.3	Abhd14b	4E-09	0.3
Adgre5	5E-05	0.3	Serpinb6a	1E-37	0.3
Frk	5E-07	0.3	Nt5c	2E-22	0.3
Meis3	5E-08	0.3	Mcoln1	1E-04	0.3
Nudt16	3E-02	0.3	Capza2	1E-29	0.3
Sqle	4E-10	0.3	Socs5	6E-09	0.3
Armcx2	5E-09	0.3	Ppp1r18	7E-13	0.3
Unc93b1	9E-06	0.3	Bdnf	4E-07	0.3
Yif1a	4E-18	0.3	Lima1	7E-25	0.3
Pten	8E-28	0.3	Coa4	1E-03	0.3
Asna1	5E-10	0.3	Maml2	4E-17	0.3
Zfp354c	1E-07	0.3	Nanp	1E-04	0.3
Acot2	9E-18	0.3	Atp8b2	5E-08	0.3
Git1	7E-06	0.3	Pusl1	8E-12	0.3
Mxra7	4E-26	0.3	Stimate	4E-09	0.3
Rsu1	8E-31	0.3	Tmem150a	9E-13	0.3
Imp4	6E-04	0.3	Nsg1	6E-10	0.3
Pcolce	4E-60	0.3	Mdga1	2E-03	0.3
Fkbp1b	3E-03	0.3	Mr1	6E-04	0.3
Herpud2	4E-08	0.3	Leprot	7E-20	0.3
Tmem256	1E-45	0.3	Gnpda1	3E-06	0.3
Pias3	7E-03	0.3	Mvk	3E-10	0.3
Sgpl1	2E-05	0.3	Gba	1E-08	0.3
Slc31a2	5E-04	0.3	Gnpda2	3E-10	0.3
Pigc	1E-06	0.3	Pdk3	9E-11	0.3
Ostf1	3E-23	0.3	Rassf2	1E-04	0.3
Ankrd29	1E-06	0.3	H2-K1	1E-19	0.3
Tpm2	4E-10	0.3	Elmod2	2E-05	0.3
Zfp532	7E-06	0.3	Myc	2E-06	0.3
Klf4	1E-28	0.3	Xpc	9E-04	0.3
Tspan17	8E-09	0.3	Bet1l	6E-18	0.3
Fat1	2E-28	0.3	Vasp	3E-11	0.3
AI413582	2E-13	0.3	Idnk	7E-10	0.3
Eepd1	5E-03	0.3	Ankrd27	3E-11	0.3
Ccdc12	4E-11	0.3	Tspan17	2E-17	0.3
Hmga2	6E-17	0.3	1700017B05Rik	3E-07	0.3
Galt	2E-04	0.3	Pfn1	2E-53	0.3
Igsf3	2E-08	0.3	Tmem219	4E-16	0.3

Chondrogenic

Control Gene	p	FC	*Notch2*$^{tm1.1Ecan}$ Gene	p	FC
Dnaaf4	3E-03	0.4	Gtf2i	3E-29	0.4
Ccdc51	9E-04	0.4	4632427E13Rik	7E-09	0.4
Dkc1	7E-13	0.4	2310010J17Rik	1E-09	0.4
Kcnq1ot1	4E-28	0.4	Avil	4E-02	0.4
Gtf2i	1E-27	0.4	Nfe2l1	2E-38	0.4
Lgi2	3E-03	0.4	Flrt1	2E-03	0.4
Elmsan1	5E-13	0.4	Hoxa11	2E-07	0.4
Zcchc14	7E-08	0.4	Ppa1	2E-15	0.4
Mogs	1E-04	0.4	Sil1	2E-19	0.4
Gm15614	5E-02	0.4	Sgf29	9E-05	0.4
Ptpn2	3E-11	0.4	E4f1	6E-02	0.4
Ecpas	1E-29	0.4	Hspe1	5E-80	0.4
Mlh1	1E-01	0.4	Malat1	2E-64	0.4
Yae1d1	2E-12	0.4	Sertad2	4E-19	0.4
Atrip	3E-02	0.4	Cpox	3E-05	0.4
Plod2	1E-50	0.4	Amy1	1E-02	0.4
Kctd15	5E-06	0.4	Tst	5E-05	0.4
6330418K02Rik	6E-03	0.4	Pex3	8E-09	0.4
Dusp6	2E-07	0.4	Hint2	7E-17	0.4
Ror2	1E-09	0.4	Snd1	3E-28	0.4
Gm28905	8E-03	0.4	Hspe1-rs1	1E-04	0.4
Alg2	1E-08	0.4	Ganab	8E-18	0.4
D11Wsu47e	4E-02	0.4	Dnaja3	2E-08	0.4
Ppan	9E-09	0.4	Zfp598	6E-03	0.4
Ptpn14	4E-22	0.4	Usp49	3E-03	0.4
Shox2	1E-16	0.4	Pam	5E-64	0.4
Ddx55	7E-02	0.4	Snhg8	5E-29	0.4
Snd1	5E-34	0.4	Fbxo9	7E-13	0.4
Krt10	2E-09	0.4	Zkscan1	1E-10	0.4
Cbx1	1E-47	0.4	Dnajc7	2E-19	0.4
Ccdc62	7E-05	0.4	Hlcs	2E-03	0.4
Wdr74	6E-08	0.4	BC031181	8E-36	0.4
Tm9sf2	9E-30	0.4	Tsc22d1	1E-51	0.4
Rbks	8E-04	0.4	Mtif2	7E-11	0.4
Gm26797	1E-02	0.4	Spry2	1E-05	0.4
Nuak1	3E-14	0.4	Prmt3	4E-07	0.4
Mar5	3E-29	0.4	Col16a1	4E-11	0.4
Mtmr10	8E-05	0.4	Acyp1	1E-15	0.4
Baiap2l1	9E-12	0.4	Scarb2	5E-14	0.4
Zfp788	8E-03	0.4	Rai1	9E-10	0.4
Gnl3	1E-27	0.4	Plekha5	6E-10	0.4
Clcn5	1E-09	0.4	Sft2d3	1E-06	0.4
Fut8	6E-05	0.4	AW209491	4E-03	0.4
Ktn1	1E-31	0.4	Mbnl3	2E-03	0.4
Rtn2	7E-02	0.4	2700046G09Rik	3E-03	0.4
Iars	4E-20	0.4	Zdhhc12	1E-04	0.4
Pcolce2	1E-18	0.4	Tmco3	2E-08	0.4
Setd6	2E-03	0.4	Mrpl44	2E-07	0.4
Lonp1	6E-11	0.4	Slc5a3	2E-12	0.4
A530072M11Rik	5E-04	0.4	Arhgap32	4E-04	0.4
Bbs4	7E-02	0.4	Iars	5E-15	0.4
Gm43713	5E-01	0.4	Mecr	2E-08	0.4
Gars	3E-33	0.4	Gar1	8E-08	0.4
Smoc1	3E-01	0.4	Shox2	2E-13	0.4
Ddb2	5E-03	0.4	C1qbp	2E-29	0.4
Creld2	1E-14	0.4	Atp10a	8E-17	0.4
Amacr	5E-03	0.4	Eif2s2	3E-57	0.4
Snhg8	5E-31	0.4	5031425E22Rik	4E-18	0.4
Taz	3E-07	0.4	Lrrc1	2E-05	0.4
Prdm16	4E-09	0.4	Ddit4	5E-04	0.4
Uxs1	7E-16	0.4	Sp4	9E-04	0.4
Rgs7bp	2E-05	0.4	Mak16	8E-14	0.4
Ostc	2E-49	0.4	Klhl21	5E-04	0.4
Shank1	5E-03	0.4	8430429K09Rik	2E-06	0.4

Fibroblast

Control Gene	p	FC	*Notch2*$^{tm1.1Ecan}$ Gene	p	FC
Scx	3E-06	0.4	Idh2	1E-26	0.4
Snrnp25	4E-07	0.4	Hexdc	1E-03	0.4
Dbr1	2E-02	0.4	Trim12a	1E-03	0.4
Cd109	3E-09	0.4	Rab35	6E-06	0.4
Rap2a	4E-08	0.4	Csrp2	6E-24	0.4
Exosc2	2E-02	0.4	Nox4	1E-03	0.4
Pgm2	6E-05	0.4	Usp39	9E-08	0.4
Gspt2	1E-02	0.4	Plat	1E-04	0.4
Pop4	4E-05	0.4	Gtf3c5	2E-04	0.4
Hexdc	6E-03	0.4	Mast2	1E-17	0.4
Pkm	7E-36	0.4	Zdhhc7	4E-02	0.4
Nsdhl	1E-07	0.4	Hsp90aa1	4E-35	0.4
Tlr4	2E-04	0.4	Emp2	3E-12	0.4
Ints1	3E-04	0.4	Tmem129	2E-04	0.4
Nbl1	4E-12	0.4	Mical3	3E-09	0.4
Sept11	8E-31	0.4	Pdgfra	9E-08	0.4
Srsf3	1E-18	0.4	Sdf2l1	1E-06	0.4
Kcnc3	1E-02	0.4	Rnf157	4E-05	0.4
Mitf	4E-04	0.4	Mcub	1E-12	0.4
Rab7b	6E-06	0.4	Ipo11	2E-07	0.4
Chrd	1E-03	0.4	Ptpn1	2E-20	0.4
Hsf2	1E-04	0.4	Acsl5	4E-03	0.4
Gabarapl1	2E-10	0.4	Mn1	1E-08	0.4
Riiad1	1E-02	0.4	Rtp4	8E-03	0.4
Adam33	2E-07	0.4	B3galnt1	5E-06	0.4
Nr6a1os	6E-03	0.4	Cdc27	3E-09	0.4
Stxbp1	5E-05	0.4	Fam76b	8E-10	0.4
Ptms	2E-35	0.4	Hp1bp3	2E-20	0.4
B9d1	8E-06	0.4	Lcorl	9E-10	0.4
Alg8	6E-05	0.4	Mmgt1	2E-06	0.4
Sfmbt2	4E-03	0.4	Trim2	4E-06	0.4
Ormdl2	2E-06	0.4	Plekha2	8E-04	0.4
Farp1	7E-20	0.4	Nop9	1E-07	0.4
Nav1	4E-07	0.4	Ess2	2E-04	0.4
Eya4	2E-20	0.4	Mob3a	3E-02	0.4
Zfp385b	4E-08	0.4	Ttll1	4E-04	0.4
Pde4b	3E-10	0.4	Fat4	3E-18	0.4
Mxd1	2E-02	0.4	Zfp74	2E-02	0.4
Crtc3	4E-10	0.4	1700109K24Rik	5E-02	0.4
Lncppara	1E-16	0.4	Msn	8E-28	0.4
Tusc1	6E-03	0.4	Mfsd8	7E-04	0.4
Glis3	8E-23	0.4	Nectin1	1E-05	0.4
Orc5	1E-02	0.4	Eif4ebp3	5E-03	0.4
Acot9	1E-11	0.4	S100a16	1E-14	0.4
Twist2	4E-03	0.4	Rab3ip	5E-04	0.4
Fbln7	1E-05	0.4	Sestd1	4E-13	0.4
Fhl2	2E-07	0.4	Tes	3E-07	0.4
Pla2g15	3E-01	0.4	Pih1d1	6E-13	0.4
Cramp1l	2E-03	0.4	Ccng2	6E-04	0.4
Dcbld2	1E-07	0.4	Tmem17	3E-03	0.4
Dhcr24	3E-06	0.4	Ipmk	1E-06	0.4
Btd	2E-06	0.4	Wdfy1	6E-10	0.4
Helq	9E-03	0.4	Rnf24	5E-05	0.4
Nfia	5E-26	0.4	Ppp1cc	2E-16	0.4
Mzt1	1E-07	0.4	Senp1	5E-07	0.4
Gramd4	7E-03	0.4	Lmna	2E-45	0.4
Zc3h7a	4E-11	0.4	Runx1	4E-15	0.4
Eda2r	5E-02	0.4	Aldh3a2	5E-02	0.4
Usp39	2E-07	0.4	Taf1	3E-11	0.4
Tpm2	2E-20	0.4	Heg1	3E-06	0.4
Ercc1	6E-04	0.4	Pxdc1	6E-09	0.4
Atl2	5E-05	0.4	Carns1	4E-03	0.4
Fam220a.1	3E-02	0.4	Prelp	2E-15	0.4
Fads1	2E-08	0.4	1810055G02Rik	5E-04	0.4

Undefined

Control Gene	p	FC	*Notch2*$^{tm1.1Ecan}$ Gene	p	FC
Airn	4E-07	0.3	Mrpl41	6E-06	0.3
Dhtkd1	3E-01	0.3	Ppip5k2	6E-01	0.3
Slc1a4	1E-01	0.3	Emc4	4E-03	0.3
Hist3h2a	6E-01	0.3	Snhg4	6E-01	0.3
Eml2	6E-01	0.3	Exosc5	2E-03	0.3
Adi1	2E-02	0.3	9130401M01Rik	3E-05	0.3
1700084C06Rik	3E-01	0.3	Aptx	4E-03	0.3
Mid2	7E-01	0.3	Ccdc130	1E-04	0.3
Ecpas	4E-06	0.3	Hspb6	7E-01	0.3
Ddrgk1	1E-02	0.3	Ubap1	6E-04	0.3
Dpy30	7E-01	0.3	Cmc4	4E-05	0.3
D16Ertd472e	5E-01	0.3	Lgr4	5E-01	0.3
Hipk1	1E-01	0.3	Bcar3	4E-01	0.3
Ttll7	2E-02	0.3	Camk1	6E-04	0.3
Ssr2	2E-04	0.3	Rpl36al	3E-08	0.3
Ndor1	9E-01	0.3	Sybu	6E-01	0.3
Hadh	5E-02	0.3	Rraga	8E-04	0.3
Rrs1	4E-02	0.3	H19	9E-02	0.3
Hoxa3	7E-02	0.3	Sec14l1	9E-01	0.3
Fitm2	1E-01	0.3	Gsto1	5E-05	0.3
Alg2	9E-01	0.3	Wars	4E-02	0.3
Gm15867	9E-01	0.3	Grwd1	1E-02	0.3
Ndufaf4	2E-01	0.3	Mcee	4E-03	0.3
Fkbp4	8E-01	0.3	Eif2s2	6E-07	0.3
Ehd4	7E-02	0.3	Sesn2	9E-02	0.3
Bcar3	6E-03	0.3	Lrrc8a	1E-04	0.3
Mphosph10	1E-01	0.3	Rrnad1	7E-02	0.3
Plcb1	1E-11	0.3	Bhlhe40	5E-02	0.3
Hist1h2ae	6E-01	0.3	Hfe	2E-02	0.3
Papss1	2E-02	0.3	Mrps14	3E-01	0.3
Tchp	3E-01	0.3	Adprm	2E-02	0.3
Maff	6E-01	0.3	Mpst	9E-04	0.3
Scarb2	3E-02	0.3	Nol7	2E-01	0.3
Timm8a1	6E-01	0.3	Dcaf12	5E-05	0.3
Xndc1	8E-01	0.3	Acot7	2E-02	0.3
Tmem161b	4E-01	0.3	Ttc4	2E-02	0.3
N6amt1	1E-02	0.3	St6galnac4	8E-08	0.3
Ormdl3	4E-01	0.3	Tmem176a	3E-03	0.3
BC003965	2E-01	0.3	Cops6	3E-08	0.3
Smc4	6E-02	0.3	Arhgef5	8E-02	0.3
Zmat1	6E-01	0.3	Igbp1	3E-06	0.3
Btg3	7E-02	0.3	Nacc2	4E-01	0.3
Lrrc28	6E-01	0.3	Hdac9	1E-01	0.3
Thumpd1	3E-02	0.3	Spcs2	4E-01	0.3
Hint2	2E-02	0.3	Adpgk	8E-02	0.3
Rps6ka2	1E+00	0.3	Fundc1	5E-04	0.3
Exosc3	1E-01	0.3	Mcrip2	6E-03	0.3
Zbtb21	4E-01	0.3	Fam43a	4E-02	0.3
Rarg	6E-03	0.3	3110040N11Rik	3E-03	0.3
Nme6	1E+00	0.3	Snhg20	4E-02	0.3
Fam135a	3E-02	0.3	Kti12	2E-03	0.3
Gatc	1E-01	0.3	Acot2	1E-03	0.3
Cebpb	1E-02	0.3	Lnpep	3E-01	0.3
Ncl	9E-15	0.3	Mccc1	3E-02	0.3
Tmem60	4E-01	0.3	Tmem53	1E-07	0.3
Pygo1	5E-01	0.3	Erp44	5E-02	0.3
Ism1	5E-01	0.3	Gm15867	1E-03	0.3
Sec11c	3E-01	0.3	Nup43	2E-02	0.3
Gtpbp4	2E-02	0.3	Hspa9	6E-02	0.2
Zfp346	9E-01	0.3	Tmed1	6E-06	0.2
Nt5c3	2E-01	0.3	Rdh5	3E-03	0.2
Tmem128	9E-01	0.3	Gtpbp8	3E-05	0.2
Gnb1l	8E-01	0.3	Borcs5	9E-03	0.2
Sybu	1E-01	0.3	Mrps31	1E-06	0.2

Articular/Synovial Fibroblast

Control Gene	p	FC	*Notch2*$^{tm1.1Ecan}$ Gene	p	FC
C1ra	8E-17	0.6	Hivep3	2E-05	0.5
Eno2	3E-03	0.6	Arhgap10	2E-11	0.5
Atp2b4	5E-04	0.6	Trio	2E-11	0.5
Lgr4	1E-14	0.6	Kctd17	3E-08	0.5
Fgf13	8E-03	0.6	S1pr2	1E-05	0.5
Tmem38a	1E-04	0.6	Prcp	4E-06	0.5
Ctso	3E-07	0.6	Rad51d	1E-03	0.5
0610010F05Rik	6E-10	0.6	Ephx1	4E-17	0.5
Gpsm2	4E-15	0.6	Lima1	3E-33	0.5
Parp12	4E-07	0.6	Polg	2E-03	0.5
Prss23	1E-23	0.6	Gm14636	5E-04	0.5
Plekhb2	3E-05	0.6	Tmem65	2E-10	0.5
Pdxk	4E-07	0.6	Ccnyl1	1E-01	0.5
Trak1	4E-12	0.6	Itga5	3E-07	0.5
Ttll11	1E-05	0.6	Atl2	6E-04	0.5
Plcb3	2E-05	0.6	Tkfc	2E-02	0.5
Sntb2	1E-34	0.6	Zfp286	5E-02	0.5
Slk	1E-19	0.6	Cpped1	2E-08	0.5
Cdon	3E-29	0.6	Fnip2	3E-08	0.5
Cpd	5E-15	0.6	Nr1d1	9E-05	0.5
Wdfy1	1E-07	0.6	2310022B05Rik	2E-09	0.5
Cbx6	2E-09	0.6	Tap2	5E-05	0.5
Gstt3	5E-07	0.6	Endod1	9E-04	0.5
Pgd	7E-11	0.6	Irgq	7E-04	0.5
Grasp	2E-05	0.6	Zswim6	2E-15	0.5
Arhgap26	2E-02	0.6	Garnl3	3E-03	0.5
Pvr	3E-04	0.6	Paox	3E-02	0.5
Map3k8	2E-04	0.6	Baz1a	7E-07	0.5
Sgsh	1E-03	0.6	Zhx1	2E-06	0.5
Sh3d19	4E-26	0.6	Glmp	5E-26	0.5
Rab9	2E-20	0.6	Heatr5b	4E-05	0.5
Gusb	3E-15	0.6	H2-DMa	7E-03	0.5
Lmbr1	4E-13	0.6	Aebp1	1E-29	0.5
Tap2	1E-08	0.6	Vcl	5E-24	0.5
Vegfc	6E-04	0.6	Ndst2	2E-03	0.5
Adamts12	1E-09	0.6	Zbtb2	2E-01	0.5
Cldn34c1	2E-03	0.6	Syngr2	4E-07	0.5
Tpcn1	1E-06	0.6	Dennd5b	4E-04	0.5
Naglu	2E-10	0.6	Dbn1	1E-10	0.5
Nin	9E-07	0.6	Selenon	3E-09	0.5
Retreg3	3E-05	0.6	Dpysl3	4E-04	0.5
Synj2	5E-06	0.6	Lactb2	8E-07	0.5
Gucy1a2	2E-07	0.6	Mef2d	5E-12	0.5
Tle6	9E-04	0.6	Tango2	3E-04	0.5
Dlc1	1E-46	0.6	Cast	9E-19	0.5
Myo9a	6E-26	0.6	Klf4	2E-11	0.5
Zfp583	8E-04	0.6	Ccdc28b	5E-06	0.5
Rnh1	3E-31	0.6	Znfx1	1E-04	0.5
Epb41l2	1E-31	0.6	D230025D16Rik	3E-02	0.5
Enox2	1E-14	0.6	Plec	1E-20	0.5
Zcchc2	7E-05	0.6	Ralb	1E-05	0.5
Ckb	4E-22	0.6	Rnf217	3E-07	0.5
Man2a1	7E-17	0.6	Vim	2E-58	0.5
4933407K13Rik	2E-02	0.6	Zfp704	1E-12	0.5
Iah1	3E-18	0.6	Ago1	9E-02	0.5
Scrn1	1E-11	0.6	Il34	1E-03	0.5
Fam149a	2E-06	0.6	Thra	2E-27	0.5
Dbi	2E-73	0.6	Ccdc74a	4E-02	0.5
Cerk	6E-05	0.6	Atp6v1b2	7E-10	0.5
Bak1	3E-08	0.6	Suox	5E-04	0.5
Arl6ip5	7E-31	0.6	Rnd2	2E-03	0.5
Ccdc82	2E-04	0.6	Alpk1	1E-06	0.5
Hacd4	1E-16	0.6	Sept8	9E-11	0.5
Fap	3E-08	0.6	Adgrl2	7E-18	0.5

Limb Mesenchyme

Control			*Notch2*[tm1.1Ecan]		
Gene	*p*	FC	Gene	*p*	FC
Zeb2	3E-30	0.3	Trim46	3E-07	0.3
Myzap	9E-06	0.3	Klf4	4E-22	0.3
Ccdc85b	4E-15	0.3	Nt5dc3	5E-05	0.3
St3gal5	9E-08	0.3	Maml3	3E-11	0.3
Hmox1	1E-14	0.3	Cdr2	6E-06	0.3
Lamb2	7E-10	0.3	Ift27	3E-14	0.3
Rcn1	3E-34	0.3	Ntpcr	7E-09	0.3
H2-T23	2E-04	0.3	Rcn1	5E-29	0.3
Mdfic	6E-15	0.3	Hilpda	2E-06	0.3
Magi1	2E-07	0.3	Dctn2	2E-27	0.3
Fubp3	7E-03	0.3	Bin1	5E-14	0.3
Tomm40l	7E-03	0.3	Amdhd2	2E-13	0.3
Zhx1	2E-08	0.3	Myd88	1E-04	0.3
Emp3	2E-51	0.3	Acsl3	2E-07	0.3
Zfhx3	8E-24	0.3	Lxn	1E-16	0.3
Rabl2	4E-03	0.3	Mtpn	1E-31	0.3
Bambi	2E-03	0.3	Wasl	1E-15	0.3
Adamtsl4	2E-09	0.3	Trim3	2E-07	0.3
Fam102a	1E-01	0.3	Eef1akmt4	6E-04	0.3
Tead1	4E-28	0.3	Pck2	5E-19	0.3
Map7d1	5E-19	0.3	Ifngr2	2E-14	0.3
Mcfd2	4E-07	0.3	Rbpms	2E-02	0.3
Nrk	4E-04	0.3	Ptpn9	7E-13	0.3
Acap2	3E-14	0.3	Tbrg4	1E-04	0.3
Atp5o	2E-41	0.3	Adcy6	3E-03	0.3
Zfp512b	9E-03	0.3	Pard3b	1E-18	0.3
Pofut2	2E-18	0.3	Usp22	2E-11	0.3
Shkbp1	2E-02	0.3	Acly	3E-13	0.3
Atf4	8E-29	0.3	Arpc1a	7E-18	0.3
Asah1	2E-38	0.3	Ostm1	2E-07	0.3
Pafah1b3	1E-10	0.3	Phf13	1E-05	0.3
Klhl42	8E-02	0.3	Adarb1	1E-05	0.3
Il10rb	5E-06	0.3	Cox6a1	5E-33	0.3
Zfp518b	9E-03	0.3	Rcn3	2E-43	0.3
Slc38a6	7E-03	0.3	Socs1	2E-05	0.3
Socs5	3E-04	0.3	Mfsd1	1E-11	0.3
Nipsnap3b	4E-05	0.3	Mfsd10	4E-12	0.3
Ech1	9E-22	0.3	Rsu1	3E-23	0.3
Rcor2	9E-06	0.3	Ehmt2	3E-13	0.3
Prelid1	2E-33	0.3	Pik3r2	7E-09	0.3
Stard3nl	9E-13	0.3	Dlk1	1E-02	0.3
Mrps30	6E-04	0.3	Zfc3h1	5E-25	0.3
Nt5c	2E-15	0.3	Aph1b	1E-06	0.3
Dhcr24	1E-05	0.3	Nhs	5E-08	0.3
Eya4	9E-30	0.3	Gpkow	2E-02	0.3
Alg11	5E-04	0.3	Marcksl1	1E-12	0.3
Rad51d	6E-03	0.3	Pcdh7	9E-32	0.3
Renbp	3E-06	0.3	Snn	4E-07	0.3
Clec2d	1E-08	0.3	Ralgds	9E-05	0.3
Ddah2	4E-20	0.3	Zhx1	4E-07	0.3
Dgka	8E-03	0.3	Pskh1	1E-03	0.3
C030014I23Rik	7E-03	0.3	Rap1gds1	1E-15	0.3
Btbd3	1E-03	0.3	Kdelr3	8E-19	0.3
Cstf1	5E-02	0.3	Tulp4	5E-23	0.3
Dtd2	2E-05	0.3	Ak1	1E-13	0.3
Ogfrl1	1E-05	0.3	Mpdu1	3E-07	0.3
Arpc4	2E-09	0.3	Rab3ip	8E-04	0.3
Kdelr1	7E-37	0.3	Gpsm1	8E-06	0.3
Ndufa3	6E-32	0.3	Tnfaip1	4E-11	0.3
Leprot	1E-16	0.3	Lpar1	2E-18	0.3
Zfc3h1	4E-23	0.3	Anxa2	2E-45	0.3
Dpf2	2E-05	0.3	Zfp319	4E-05	0.3
Srd5a3	9E-03	0.3	Ppfibp2	1E-03	0.3
Pcsk5	2E-02	0.3	2610002M06Rik	1E-07	0.3

Chondrogenic

Control			*Notch2*[tm1.1Ecan]		
Gene	*p*	FC	Gene	*p*	FC
Zfp516	9E-22	0.4	Thada	3E-09	0.4
Mageh1	9E-10	0.4	Gm10561	8E-03	0.4
Tmem201	7E-03	0.4	Atp6v1a	3E-25	0.4
Nedd9	1E-16	0.4	Yars	2E-12	0.3
Tanc2	5E-16	0.4	Ermard	1E-03	0.3
Nars	2E-46	0.4	Ogfod3	1E-04	0.3
Wdr35	4E-11	0.4	Tmem94	4E-02	0.3
4930533B01Rik	1E-02	0.4	Lnpep	1E-12	0.3
Acot8	1E-02	0.4	Zfp566	1E-02	0.3
Myom1	3E-08	0.4	Chuk	2E-05	0.3
Coq2	3E-09	0.4	Zscan21	3E-05	0.3
Dhtkd1	8E-04	0.4	Nacc2	2E-12	0.3
Lekr1	2E-05	0.4	2810001G20Rik	2E-06	0.3
Atp2b1	4E-33	0.4	Farsb	2E-10	0.3
Wdr27	7E-04	0.4	Txlng	1E-06	0.3
Hhat	1E-04	0.4	Glcci1	1E-08	0.3
1810024B03Rik	8E-06	0.4	Phlpp1	2E-04	0.3
Psmg2	2E-11	0.4	Atp6v1g1	1E-47	0.3
Ddx21	2E-22	0.4	Cdh19	5E-66	0.3
Plekha3	2E-08	0.3	Yrdc	4E-11	0.3
Ggact	2E-08	0.3	Auts2	2E-41	0.3
Dnhd1	3E-02	0.3	Araf	6E-11	0.3
Fgfr2	1E-49	0.3	Rmnd5a	6E-09	0.3
Pole2	2E-02	0.3	Gm16845	1E-01	0.3
Etl4	5E-14	0.3	Ppib	2E-85	0.3
Epm2a	2E-01	0.3	Aen	4E-12	0.3
Ica1	1E-07	0.3	Gnpnat1	1E-12	0.3
Sh3rf1	1E-07	0.3	Syne1	1E-26	0.3
Sypl	1E-20	0.3	Usp2	4E-04	0.3
Tmem238	5E-12	0.3	Scrn3	4E-05	0.3
Gprin3	8E-06	0.3	Trp53rka	6E-02	0.3
Ncl	2E-53	0.3	Rdh10	2E-02	0.3
Mrps28	4E-15	0.3	Dnajb14	6E-07	0.3
Saysd1	8E-11	0.3	Gmds	7E-35	0.3
Surf6	5E-06	0.3	Spout1	6E-04	0.3
Hspd1	9E-46	0.3	Nog	5E-04	0.3
Rbm4b	3E-08	0.3	Ier2	3E-43	0.3
Orai2	1E-03	0.3	Dusp12	2E-08	0.3
Kdm5b	6E-24	0.3	Utp14a	3E-10	0.3
Nr2c2ap	3E-07	0.3	Taf5l	6E-04	0.3
Pno1	3E-10	0.3	Thumpd3	4E-05	0.3
Mbd6	2E-02	0.3	Cdc42ep3	2E-16	0.3
Snhg12	6E-24	0.3	Kif16b	5E-09	0.3
Gna12	3E-13	0.3	Osbpl9	1E-22	0.3
Zfas1	1E-30	0.3	Gys1	8E-05	0.3
Ap1g1	2E-12	0.3	Stx3	1E-03	0.3
Paip2b	2E-04	0.3	Dhtkd1	2E-04	0.3
Synpo	4E-12	0.3	Gne	6E-04	0.3
Tm2d3	7E-14	0.3	Bscl2	5E-17	0.3
Gys1	3E-06	0.3	Dhrs11	6E-04	0.3
Eml2	3E-05	0.3	Tars2	2E-01	0.3
Eif2b3	1E-10	0.3	Pyroxd1	7E-03	0.3
Prpsap2	1E-03	0.3	Ctsh	3E-17	0.3
0610009E02Rik	1E-02	0.3	Zbtb11os1	7E-04	0.3
Nip7	6E-08	0.3	Kdm5b	6E-20	0.3
Ccdc97	8E-03	0.3	Hoxa9	1E-13	0.3
Dbp	5E-09	0.3	Fam185a	2E-03	0.3
Nat10	3E-05	0.3	Hoxa11os	2E-07	0.3
Hoxa9	1E-23	0.3	Gm14966	1E-02	0.3
Mia2	6E-22	0.3	Pla2g4a	8E-05	0.3
Amd1	8E-11	0.3	Ptk2	1E-14	0.3
Enox1	1E-08	0.3	Bicral	7E-10	0.3
Mtif2	4E-11	0.3	Sec62	7E-58	0.3
Mphosph9	3E-04	0.3	Snai2	2E-13	0.3

Fibroblast

Control			*Notch2*[tm1.1Ecan]		
Gene	*p*	FC	Gene	*p*	FC
Creld2	3E-05	0.4	Pvr	8E-05	0.4
Btbd19	2E-01	0.4	Cep162	6E-08	0.4
Srsf2	5E-16	0.4	Slx4ip	1E-06	0.4
Ppp1r18	3E-06	0.4	Lrrc32	5E-02	0.4
Fam107b	9E-06	0.4	Ppm1f	2E-01	0.4
Fbxo6	8E-05	0.4	A4galt	5E-03	0.4
Rad50	5E-11	0.4	Mtr	1E-04	0.4
Wdr35	6E-12	0.4	1700086O06Rik	3E-03	0.4
Cnpy4	1E-08	0.4	Prkcg	1E-04	0.4
Ccdc112	6E-06	0.4	Zfp229	1E-03	0.4
Arhgap42	2E-11	0.4	Alms1	2E-04	0.4
Mustn1	6E-12	0.4	Pawr	1E-12	0.4
Ddr1	2E-04	0.4	Zfp664	9E-10	0.4
Spcs3	1E-07	0.4	Tfdp2	4E-11	0.4
Vamp5	8E-14	0.4	Ttl	1E-03	0.4
Kank2	8E-12	0.4	Smc5	4E-09	0.4
Plekhg2	4E-05	0.4	Zfp410	5E-06	0.4
Tbx4	2E-06	0.4	Stn1	8E-03	0.4
Cand1	4E-12	0.4	Car5b	4E-03	0.4
Ehd3	9E-05	0.4	Spata6	2E-09	0.4
Abcg2	4E-01	0.4	Map2	1E-08	0.4
Sms	8E-06	0.4	Nxt2	1E-03	0.4
St3gal2	1E-08	0.4	2700054A10Rik	3E-03	0.4
Msra	1E-03	0.4	Ldb2	7E-08	0.4
Lmna	3E-36	0.4	Dazap1	1E-16	0.4
Cntrl	1E-05	0.4	Sh3pxd2a	6E-15	0.4
Cerk	7E-04	0.4	Primpol	6E-03	0.4
Atp6v0a4	2E-13	0.4	Cep19	9E-05	0.4
Etfdh	3E-05	0.4	Hoxa11	3E-05	0.4
Arhgap22	5E-08	0.4	Cc2d2a	5E-08	0.4
Maoa	5E-05	0.4	Fbxo30	2E-06	0.4
Fxn	2E-04	0.4	B930095G15Rik	4E-04	0.4
Ajuba	3E-04	0.4	Resf1	5E-09	0.4
Baz1b	3E-12	0.4	Pmvk	7E-07	0.4
Ssrp1	4E-11	0.4	Cys1	2E-08	0.4
Nelfe	5E-09	0.4	Simc1	9E-07	0.4
Isg20	7E-06	0.4	Zbtb17	7E-05	0.4
Slc24a3	7E-17	0.4	Aldh3b1	2E-05	0.4
Myl12a	1E-35	0.4	Hmgn3	1E-29	0.4
Zfp687	6E-02	0.4	Vps33b	6E-04	0.4
Phldb2	1E-18	0.4	Me2	4E-06	0.4
2810006K23Rik	3E-05	0.4	Lypla2	1E-02	0.4
Rfx2	1E-02	0.4	Trim16	3E-04	0.4
Zhx3	7E-06	0.4	Snta1	4E-10	0.4
Usp28	4E-03	0.4	Dhx32	2E-08	0.4
Nup37	7E-05	0.4	Runx1t1	2E-27	0.4
Lsm8	7E-07	0.4	Clic1	1E-28	0.4
Dstn	3E-30	0.4	Nrde2	9E-04	0.4
Mfsd14b	9E-09	0.4	Rnf219	1E-04	0.4
Me2	3E-05	0.4	Uba2	7E-11	0.4
Mcm9	5E-04	0.4	Acsf2	4E-03	0.4
Pdlim7	8E-08	0.4	Gemin2	1E-05	0.4
Twsg1	2E-11	0.4	1810037I17Rik	8E-17	0.4
S100a11	9E-50	0.4	Gpr19	2E-04	0.4
Mrs2	8E-05	0.4	Ptma	2E-64	0.4
Slc25a23	1E-01	0.4	Cstf2	1E-03	0.4
Fam171a2	3E-05	0.4	Brd9	7E-14	0.4
Rdh5	1E-02	0.4	Adgrl3	2E-05	0.4
Gm9844	1E-03	0.4	Adss	1E-09	0.4
Tnfrsf1b	2E-02	0.4	Zfp37	2E-01	0.4
Cnot9	2E-04	0.4	Gtf3c4	2E-03	0.4
Met	6E-02	0.4	Pank1	1E-06	0.4
Deps	9E-04	0.4	Gcnt2	1E-06	0.4
Med23	1E-03	0.4	Gabpb1	1E-04	0.4

Undefined

Control			*Notch2*[tm1.1Ecan]		
Gene	*p*	FC	Gene	*p*	FC
Thnsl2	4E-01	0.3	Nphp1	4E-04	0.2
Coq2	2E-01	0.3	Eml6	2E-03	0.2
Dohh	3E-01	0.3	Anks1	4E-03	0.2
Sod1	7E-01	0.3	Uqcrq	2E-03	0.2
Zfp142	3E-01	0.3	Gadd45a	4E-03	0.2
Mras	1E-01	0.3	Slc39a11	8E-03	0.2
Nim1k	8E-01	0.3	Tmco4	3E-04	0.2
Sehl1	6E-01	0.3	Gm15283	3E-10	0.2
Tcf4	9E-29	0.3	Slc19a1	2E-01	0.2
Slc37a4	8E-01	0.3	Ssr3	8E-01	0.2
Sdad1	7E-01	0.3	Ndufaf8	8E-05	0.2
Mzt1	7E-01	0.3	Krtcap2	4E-06	0.2
Znrd1as	8E-01	0.3	Smim8	1E-01	0.2
AW209491	2E-01	0.3	Mrpl46	2E-05	0.2
Epb41l4aos	3E-01	0.3	Slain2	3E-06	0.2
Pde4a	9E-01	0.3	Selenbp1	2E-01	0.2
Bbs9	6E-01	0.3	E2f6	4E-05	0.2
Mab21l2	7E-03	0.3	Bnip3	8E-01	0.2
Tmem230	8E-01	0.3	Ezh1	4E-04	0.2
Klf10	4E-02	0.3	Ndufs3	1E-07	0.2
Lmo4	2E-05	0.3	St3gal4	3E-02	0.2
Kcnk2	2E-03	0.3	Zcchc3	1E-03	0.2
A430018G15Rik	3E-01	0.3	2010320M18Rik	3E-03	0.2
Aasdhppt	6E-01	0.3	Kdelr2	3E-07	0.2
Grsf1	4E-01	0.3	Ahcyl2	6E-01	0.2
Rnaset2a	3E-01	0.3	Gpr180	3E-05	0.2
Tmub1	5E-02	0.3	Ndufaf6	5E-03	0.2
Hax1	6E-01	0.3	Ddit4	4E-02	0.2
Hmox1	4E-01	0.3	Ndufa4l2	2E-01	0.2
Ndufaf6	4E-01	0.3	Taldo1	4E-02	0.2
Dcp1b	9E-01	0.3	Ndufa5	2E-03	0.2
Shld2	4E-01	0.3	Bpnt1	5E-02	0.2
Tmem38b	3E-01	0.3	Mthfd1l	2E-01	0.2
Nol7	8E-05	0.3	Mpp7	3E-03	0.2
Cebpzos	2E-01	0.3	Mirg	4E-01	0.2
Ldlrap1	8E-01	0.3	Hps5	4E-04	0.2
Prkab1	3E-01	0.3	Sfxn5	7E-06	0.2
Sipa1l1	1E-03	0.3	Fgfr2	8E-04	0.2
Tmem161a	7E-01	0.3	Snhg8	5E-02	0.2
Atp1b3	2E-02	0.3	Zfp869	9E-02	0.2
Lrrc14	5E-02	0.3	Gm16740	9E-02	0.2
Pacsin2	1E-04	0.3	Sf3b6	3E-01	0.2
Yipf2	2E-02	0.3	Ssbp1	1E-03	0.2
Gm15952	5E-01	0.3	Srpr	1E-02	0.2
Tmem39b	7E-01	0.3	Gmppb	2E-08	0.2
A930015D03Rik	6E-01	0.3	Pigb	3E-02	0.2
Erp29	1E-05	0.3	Slc35d1	9E-02	0.2
Gsk3a	5E-03	0.3	Hprt	1E-01	0.2
Unc5b	7E-01	0.3	Insr	1E-04	0.2
Cep41	7E-02	0.3	Gtf2a1	1E-03	0.2
Gm26887	9E-01	0.3	Mtmr10	6E-01	0.2
Aifm2	5E-01	0.3	Hspd1	5E-02	0.2
Clcc1	8E-01	0.3	Ndufaf4	1E-01	0.2
Ficd	5E-01	0.3	5430405H02Rik	5E-04	0.2
Trafd1	1E-01	0.3	Ruvbl2	2E-03	0.2
Krba1	1E-02	0.3	Micos13	2E-01	0.2
Slc39a10	1E-01	0.3	2310061I04Rik	2E-02	0.2
Ppp1r15a	2E-02	0.3	Ldha	7E-01	0.2
Nr4a3	1E-01	0.3	Coro7	6E-04	0.2
Rtl3	8E-01	0.3	Mettl4	3E-03	0.2
Ndufa12	4E-02	0.3	Zfp87	1E-04	0.2
Farsb	9E-01	0.3	Pithd1	6E-01	0.2
Lsm8	9E-01	0.3	Aplp2	8E-04	0.2
Tspan12	5E-01	0.3	Eef1akmt1	3E-05	0.2

Articular/Synovial Fibroblast

Control			*Notch2*[tm1.1Ecan]		
Gene	*p*	FC	Gene	*p*	FC
Gm32618	2E-05	0.6	Paqr4	2E-04	0.5
Fasn	7E-16	0.6	Zdhhc21	4E-02	0.5
Abr	2E-05	0.6	Erlin2	6E-05	0.5
Lrrc49	3E-07	0.6	Sorcs2	2E-03	0.5
Dffa	2E-03	0.6	Acadl	6E-22	0.5
Dst	2E-34	0.6	Gm47071	3E-05	0.5
Ifi27l2a	2E-12	0.6	Fbxl15	1E-04	0.5
Aebp1	3E-45	0.6	S100a10	1E-34	0.5
Mar2	5E-16	0.6	Dpp7	1E-08	0.5
Tmem192	7E-15	0.6	Epdr1	2E-08	0.5
Zfp286	1E-03	0.6	Adam17	1E-08	0.5
Sgpp1	2E-07	0.6	Lgals1	2E-51	0.5
Mapkbp1	3E-02	0.6	Naprt	4E-02	0.5
Olfml1	3E-09	0.6	Tpcn1	2E-03	0.5
Micall1	1E-02	0.6	Zfp36l2	6E-17	0.5
Ndrg1	5E-20	0.6	Shf	2E-02	0.5
Ripor1	2E-07	0.6	Fam102b	8E-12	0.5
Bclaf3	3E-07	0.6	Htra1	7E-16	0.5
Hoxd8	5E-05	0.6	Otud3	2E-02	0.5
Fam171a2	2E-05	0.5	Nfia	6E-29	0.5
Rtl8b	3E-08	0.5	Galnt2	1E-10	0.5
Tpcn2	4E-03	0.5	Dock5	6E-06	0.5
Lrp1	6E-75	0.5	Tmem185b	3E-01	0.5
Ereg	8E-05	0.5	Prkaca	2E-06	0.5
Adprh	6E-12	0.5	Spata6	2E-08	0.5
Snx9	2E-31	0.5	Ppm1l	3E-04	0.5
Clcn7	4E-05	0.5	Dhx32	3E-05	0.5
Taco1os	3E-02	0.5	Src	3E-04	0.5
Twist2	5E-03	0.5	Rab30	1E-05	0.5
Tmem94	6E-05	0.5	Col5a2	3E-44	0.5
Gcnt2	2E-05	0.5	Gcnt2	2E-04	0.5
Fam214a	2E-10	0.5	Mblac2	4E-02	0.5
Slc11a2	3E-06	0.5	Zbtb4	2E-06	0.5
Vps4b	5E-20	0.5	Ttll11	3E-01	0.5
Evc	9E-06	0.5	Dnajb4	2E-12	0.5
Tifa	8E-02	0.5	Sh3pxd2a	5E-15	0.5
Pmvk	1E-07	0.5	Cxxc5	2E-09	0.5
H2-T22	1E-08	0.5	Jarid2	2E-09	0.5
Angptl1	1E-08	0.5	Dnajc13	4E-11	0.5
Ano8	4E-03	0.5	Carmn	4E-05	0.5
Tsga10	3E-06	0.5	Mocs1	7E-03	0.5
Trim12c	1E-05	0.5	Morrbid	3E-07	0.5
Uevld	4E-04	0.5	Casp3	5E-06	0.5
Ago4	1E-03	0.5	Ebp	3E-10	0.5
Xylt2	3E-03	0.5	Nin	4E-03	0.5
Elf4	4E-10	0.5	Man2b2	7E-05	0.5
Dpysl2	1E-27	0.5	A330076H08Rik	4E-04	0.5
Src	8E-05	0.5	Klf13	9E-17	0.5
Kat2b	2E-10	0.5	Mmp16	5E-03	0.5
Fkbpl	7E-02	0.5	Trak1	1E-06	0.5
Ccnyl1	2E-05	0.5	Rhoq	4E-21	0.5
Morrbid	3E-10	0.5	Igsf8	5E-08	0.5
Ly96	9E-05	0.5	Dst	9E-23	0.5
Mospd1	4E-04	0.5	Cd1d1	2E-04	0.5
Ccdc93	6E-07	0.5	Naaladl2	7E-21	0.5
Lgalsl	1E-04	0.5	B2m	3E-26	0.5
Prkd1	2E-15	0.5	Ubtd2	1E-12	0.5
Casp12	2E-07	0.5	Jup	1E-01	0.5
Mef2a	1E-31	0.5	Clstn1	1E-16	0.5
Capn2	2E-25	0.5	Sntg2	4E-04	0.5
Mfsd8	2E-04	0.5	Fez2	9E-03	0.5
Ehbp1l1	8E-14	0.5	Fam111a	5E-15	0.5
Grik5	4E-06	0.5	Pex5	3E-01	0.5
Cdc42bpb	1E-15	0.5	Akr1b10	2E-03	0.5

Limb Mesenchyme					
Control			$Notch2^{tm1.1Ecan}$		
Gene	*p*	FC	Gene	*p*	FC
Nenf	2E-52	0.3	Rhog	7E-11	0.3
Runx2	1E-23	0.3	Pdgfrb	2E-25	0.3
Rnf219	7E-02	0.3	Vwa5a	5E-12	0.3
Vwa5a	5E-11	0.3	Galk1	2E-16	0.3
Gclm	2E-11	0.3	Slc25a1	6E-16	0.3
Hcfc1r1	1E-36	0.3	Cdkn2a	5E-13	0.3
Ppp1r9b	2E-04	0.3	Rfng	1E-04	0.3
Samd1	8E-06	0.3	Itm2b	8E-51	0.3
Slc38a7	3E-03	0.3	Kars	1E-06	0.3
1700123O20Rik	1E-07	0.3	Mrm2	2E-04	0.3
Vangl2	7E-04	0.3	Naa25	4E-04	0.3
Cdc14a	4E-04	0.3	Hivep1	2E-03	0.3
Xxylt1	3E-05	0.3	Prkd1	7E-12	0.3
Chrd	1E-02	0.3	Apbb1	2E-04	0.3
Tprkb	3E-08	0.3	Mpdz	2E-15	0.3
Hmg20b	7E-16	0.3	Pofut2	4E-17	0.3
4930402H24Rik	1E-09	0.3	Fam3a	9E-09	0.3
Nkiras2	1E-04	0.3	Csf2ra	2E-05	0.3
Pcsk7	1E-03	0.3	Rrp1b	4E-07	0.3
Rgs19	3E-07	0.3	Vps26b	3E-05	0.3
Tradd	3E-04	0.3	Lyrm1	3E-05	0.3
Plpp5	4E-07	0.3	Ctps	3E-10	0.3
Gtpbp6	4E-03	0.3	Kctd5	2E-05	0.3
Ift27	3E-13	0.3	Fzd1	8E-10	0.3
Stard5	1E-05	0.3	Pip4k2a	5E-10	0.3
1110012L19Rik	1E-05	0.3	Tep1	8E-08	0.3
Ppp1r18	4E-05	0.3	Zfp52	5E-03	0.3
Jarid2	2E-03	0.3	Pygo2	3E-05	0.3
Rab13	9E-07	0.3	Atf4	8E-32	0.3
Mrps11	3E-06	0.3	Eef2	9E-59	0.3
Fbxl6	2E-05	0.3	Hmg20b	2E-21	0.3
Camk2n1	1E-02	0.3	Arl8a	1E-15	0.3
Calm3	1E-24	0.3	Bmt2	1E-07	0.3
Emc10	3E-23	0.3	Tnfsf12	1E-13	0.3
Akip1	1E-03	0.3	0610030E20Rik	7E-04	0.3
Mif4gd	2E-04	0.3	Slc41a3	2E-06	0.3
Myo7a	3E-02	0.3	Ids	2E-06	0.3
Mthfd2	3E-21	0.3	Kremen1	2E-06	0.3
Mindy2	3E-08	0.3	Cpeb2	2E-05	0.3
Agpat1	8E-02	0.3	Mex3a	9E-05	0.3
Gnb2	2E-45	0.3	Il10rb	5E-08	0.3
Prkra	1E-08	0.3	Qdpr	3E-10	0.3
Mtx2	6E-14	0.3	Dennd2a	3E-07	0.3
Sgce	1E-11	0.3	Zfp428	9E-07	0.3
Aldh16a1	3E-02	0.3	Elf4	4E-11	0.3
Fads1	4E-07	0.3	Smurf1	3E-12	0.3
Abcg2	2E-03	0.3	Fads1	2E-10	0.3
Parp3	1E-11	0.3	Nipsnap3b	6E-10	0.3
Ahnak2	4E-41	0.3	Dyrk2	2E-05	0.3
Pik3c3	7E-03	0.3	Zfp820	7E-04	0.3
Ugcg	2E-04	0.3	Thap11	2E-07	0.3
Npdc1	7E-22	0.3	Mad2l2	9E-06	0.3
Isoc2b	5E-06	0.3	Ndn	7E-17	0.3
2610008E11Rik	7E-03	0.3	Ubtd2	2E-10	0.3
Ikbke	4E-02	0.3	Impdh1	8E-09	0.3
Dtx2	2E-02	0.3	Satb2	5E-08	0.3
Cast	2E-18	0.3	Ccdc6	7E-10	0.3
Mrtfa	3E-12	0.3	Igf2bp3	4E-10	0.3
Atxn1	2E-05	0.3	Mcfd2	1E-10	0.3
Impdh1	9E-06	0.3	Mar9	3E-04	0.3
Ass1	8E-05	0.3	Golt1b	3E-09	0.3
Hykk	2E-02	0.3	2200002D01Rik	1E-04	0.3
Manea	2E-07	0.3	Ctsz	8E-37	0.3
C2cd2	3E-01	0.3	Rbfa	1E-10	0.3

Chondrogenic					
Control			$Notch2^{tm1.1Ecan}$		
Gene	*p*	FC	Gene	*p*	FC
Ppp1r10	1E-12	0.3	Cab39	4E-14	0.3
2410002F23Rik	2E-05	0.3	Gatd3a	3E-05	0.3
Glce	1E-07	0.3	Sacm1l	6E-09	0.3
Zbtb38	2E-14	0.3	1500011B03Rik	2E-10	0.3
Avpi1	5E-15	0.3	Atl1	8E-02	0.3
Scrn3	6E-05	0.3	Rbm4	9E-07	0.3
Ifi30	1E-18	0.3	Amd1	4E-06	0.3
Vezt	1E-05	0.3	Tnfaip8	2E-08	0.3
2410006H16Rik	7E-36	0.3	Dus2	1E-02	0.3
Pus3	3E-03	0.3	Wars2	3E-08	0.3
Rgl1	7E-02	0.3	Mras	3E-09	0.3
Sybu	2E-08	0.3	Micu1	2E-15	0.3
Tpi1	5E-21	0.3	Ktn1	6E-26	0.3
Chd1l	1E-03	0.3	Cdkn1a	1E-22	0.3
Zfp239	1E-02	0.3	Fam71e1	2E-03	0.3
Eprs	1E-59	0.3	Pcdh11x	4E-18	0.3
Ghitm	9E-31	0.3	Ubap2	5E-21	0.3
Gm4876	1E-08	0.3	Satb2	3E-01	0.3
Smim8	1E-11	0.3	Pak1	4E-06	0.3
Pfkm	1E-05	0.3	2810013P06Rik	2E-06	0.3
Nomo1	4E-10	0.3	Ankrd28	2E-18	0.3
Mdm2	2E-13	0.3	Pithd1	2E-04	0.3
Dusp8	2E-03	0.3	Eefsec	1E-05	0.3
Ly6e	7E-01	0.3	Vamp8	2E-29	0.3
Dph6	5E-14	0.3	Senp2	3E-10	0.3
Slc4a4	6E-04	0.3	Acot2	6E-12	0.3
Catspere2	7E-02	0.3	Alg3	1E-02	0.3
Dus4l	6E-04	0.3	Zfp609	9E-13	0.3
Vamp8	2E-32	0.3	Rap2c	5E-08	0.3
2810403D21Rik	2E-07	0.3	Uhrf1bp1	4E-03	0.3
Ddrgk1	1E-23	0.3	Gm19710	1E-04	0.3
Eif2s2	4E-54	0.3	Nars	2E-36	0.3
Smim26	2E-14	0.3	Micall1	1E-02	0.3
Tbc1d10a	7E-03	0.3	Pgam1	4E-39	0.3
Mbnl3	7E-05	0.3	Sepsecs	1E-03	0.3
N4bp1	2E-16	0.3	Gtpbp4	6E-26	0.3
Pcdh11x	3E-23	0.3	Foxo3	6E-10	0.3
Kti12	9E-06	0.3	Tsr3	3E-10	0.3
1810034E14Rik	2E-03	0.3	Oard1	9E-06	0.3
Arhgap32	4E-08	0.3	Gprin3	4E-03	0.3
Slc9a8	6E-07	0.3	Ttll7	4E-04	0.3
2700046G09Rik	3E-03	0.3	Trmt61b	5E-05	0.3
Rwdd4a	2E-07	0.3	Lpin2	2E-03	0.3
AW146154	3E-02	0.3	Mfap1b	8E-07	0.3
Hspa5	9E-48	0.3	Myo6	2E-08	0.3
Ehd3	4E-08	0.3	Mccc1	1E-06	0.3
Armcx6	1E-03	0.3	Alg1	2E-05	0.3
Srpr	6E-27	0.3	Tbc1d1	1E-05	0.3
B230217C12Rik	1E-04	0.3	Lca5	1E-01	0.3
Tmsb15b1	6E-03	0.3	Sgms1	1E-10	0.3
Clint1	2E-26	0.3	Smim10l1	3E-24	0.3
Irak2	6E-02	0.3	Qrsl1	6E-02	0.3
Ivd	4E-11	0.3	Tmem63a	2E-03	0.3
Mettl1	9E-17	0.3	Myo9b	2E-13	0.3
Gm5617	3E-07	0.3	E130311K13Rik	1E-04	0.3
Rai1	1E-07	0.3	Sypl	6E-19	0.3
Emc6	7E-22	0.3	Sdc3	2E-11	0.3
Ctsf	5E-11	0.3	Eif5	4E-36	0.3
Rsl1d1	2E-25	0.3	Ring1	5E-06	0.3
Ankrd28	8E-18	0.3	Ly6e	4E-04	0.3
Trub2	9E-07	0.3	Dpy30	5E-16	0.3
Nop56	1E-12	0.3	Timm8a1	6E-13	0.3
Aldh6a1	1E-08	0.3	Pus3	2E-03	0.3
Sik3	2E-18	0.3	Slc33a1	1E-09	0.3

Fibroblast					
Control			$Notch2^{tm1.1Ecan}$		
Gene	*p*	FC	Gene	*p*	FC
Gm6712	1E-02	0.4	Zfp287	7E-03	0.4
S100a16	2E-12	0.4	Srbd1	2E-06	0.4
Nox4	2E-02	0.4	Hspb6	1E-07	0.4
Ahctf1	3E-07	0.4	Rcan1	4E-06	0.4
Inip	3E-02	0.4	Fbxo34	2E-07	0.4
Tpcn1	9E-04	0.4	Srsf3	6E-26	0.4
Clic1	7E-22	0.4	Nbl1	8E-12	0.4
Alg12	4E-02	0.4	Inpp4b	9E-03	0.4
Ptgis	3E-11	0.4	Dag1	3E-29	0.4
Ehbp1l1	6E-07	0.4	Phf13	8E-02	0.4
Npat	2E-09	0.4	Myef2	3E-10	0.4
Tmem237	1E-06	0.4	Arid4a	1E-14	0.4
Cyld	1E-09	0.4	Rhno1	6E-08	0.4
Pom121	2E-04	0.4	Zc3h7a	4E-12	0.4
Eif1ad	1E-05	0.4	Smad3	6E-14	0.4
Phtf1	1E-08	0.4	Saal1	3E-04	0.4
Pcdh11x	5E-12	0.4	Zfp513	3E-02	0.4
Rbm28	9E-10	0.4	2200002D01Rik	3E-04	0.4
Slc25a24	6E-05	0.4	Taf5	6E-05	0.4
Snrpd1	2E-11	0.4	Crat	1E-06	0.4
Myadm	9E-11	0.4	Scai	4E-09	0.4
Mavs	3E-04	0.4	Hnrnpa3	9E-24	0.4
Arpin	1E-03	0.4	Pld1	3E-04	0.4
Kbtbd2	5E-04	0.4	Slc24a3	1E-15	0.4
Cnnm3	4E-03	0.4	Clec16a	1E-04	0.4
Faap100	6E-02	0.4	Fem1a	5E-03	0.4
Nup43	2E-04	0.4	Tatdn2	8E-04	0.4
Il17rc	5E-03	0.4	Sulf1	2E-12	0.4
Zhx2	5E-04	0.4	Bdnf	3E-08	0.4
Zkscan5	8E-02	0.4	Podxl2	7E-05	0.4
Trmt10b	2E-02	0.4	Meaf6	5E-07	0.4
Pcbp3	9E-03	0.4	Fbxl8	2E-03	0.4
Nampt	1E-10	0.4	Nfatc4	5E-06	0.4
Wdpcp	2E-05	0.4	Ltn1	3E-04	0.4
Zfp90	1E-02	0.4	Lmtk2	1E-06	0.4
Casp9	6E-03	0.4	Taok2	8E-06	0.4
Fancf	4E-05	0.4	Sap18	1E-17	0.4
Traf3ip2	2E-03	0.4	Dmd	1E-14	0.4
Ahcyl1	7E-17	0.4	Grk6	3E-07	0.4
Slc39a3	2E-03	0.4	Supv3l1	1E-04	0.4
Vegfc	2E-03	0.4	Zfat	4E-02	0.4
Tamm41	5E-03	0.4	Il15ra	1E-05	0.4
Ints10	7E-04	0.3	Nudt4	2E-22	0.4
Lclat1	8E-05	0.3	Aida	2E-09	0.4
Armcx4	6E-03	0.3	Cyld	7E-13	0.4
Stk24	2E-07	0.3	Casp12	3E-08	0.4
Sh3pxd2a	1E-10	0.3	Dhrs13	2E-02	0.4
Naa40	8E-03	0.3	H2afy2	7E-09	0.4
Hras	5E-12	0.3	Hnrnpab	1E-17	0.4
Mxi1	1E-05	0.3	Cobll1	3E-12	0.4
Sec14l1	5E-13	0.3	Myo7a	3E-04	0.4
Setd7	1E-08	0.3	Dbndd2	7E-05	0.4
5031439G07Rik	7E-06	0.3	Parp12	9E-04	0.4
Vgll4	3E-15	0.3	Antxr2	2E-07	0.4
Fkbp2	4E-12	0.3	Clip1	6E-18	0.4
0610010F05Rik	5E-08	0.3	Arl6	2E-05	0.4
Uhmk1	5E-04	0.3	Pdzd2	3E-05	0.4
Emp1	4E-19	0.3	Wdr35	7E-14	0.4
Rasa3	1E-07	0.3	Sema3f	1E-06	0.4
Gpx4	6E-34	0.3	Srsf2	1E-17	0.4
Sipa1l2	4E-02	0.3	Pld2	8E-04	0.4
Kcnd2	2E-03	0.3	Sfpq	6E-21	0.4
Abl2	5E-10	0.3	Rasal2	2E-17	0.4
Ehd4	8E-12	0.3	Emsy	1E-08	0.4

Undefined					
Control			$Notch2^{tm1.1Ecan}$		
Gene	*p*	FC	Gene	*p*	FC
2310011J03Rik	8E-02	0.3	Gm4876	9E-01	0.2
Gemin2	1E+00	0.3	Veph1	4E-02	0.2
Lrrc1	6E-01	0.3	Slc37a4	3E-01	0.2
Alpk1	2E-02	0.3	Sub1	3E-03	0.2
Sept9	3E-01	0.3	Cbx4	8E-03	0.2
Slc25a13	9E-01	0.3	Nr4a3	2E-01	0.2
Cdk2ap1	5E-01	0.3	Tmbim4	5E-02	0.2
Clmn	4E-01	0.3	Dap	7E-02	0.2
Fndc3b	2E-16	0.3	Endod1	1E-05	0.2
Ncoa4	1E-01	0.3	Rps27rt	6E-03	0.2
Atp6v1g1	1E-08	0.3	Chmp2b	3E-04	0.2
Tsr3	3E-01	0.3	Clip4	1E-02	0.2
Mtx1	6E-01	0.2	Nfat5	3E-01	0.2
Mphosph9	1E-01	0.2	Desi1	1E-02	0.2
Hspa9	1E-04	0.2	Rom1	1E-02	0.2
Ing2	6E-01	0.2	Syvn1	4E-04	0.2
Fmc1	6E-01	0.2	Lrrc4	2E-03	0.2
Trim24	1E+00	0.2	Uqcrh	4E-14	0.2
Eif2s3x	6E-01	0.2	2610301B20Rik	5E-07	0.2
Yod1	9E-01	0.2	Ndufb2	3E-02	0.2
Runx1	6E-02	0.2	Armcx6	2E-03	0.2
Cog6	5E-01	0.2	Phpt1	6E-03	0.2
Nfu1	3E-01	0.2	Lpar4	2E-01	0.2
Vamp8	4E-01	0.2	Wdr37	6E-06	0.2
Sulf1	2E-03	0.2	Pfdn4	3E-05	0.2
1700109H08Rik	4E-01	0.2	Pnrc2	4E-05	0.2
Slc38a1	5E-03	0.2	Bap1	7E-07	0.2
Acot11	5E-02	0.2	Odc1	2E-01	0.2
G6pc3	5E-01	0.2	2700062C07Rik	6E-04	0.2
Polr2d	6E-02	0.2	Stard5	3E-02	0.2
Ddx56	1E-01	0.2	Selenok	2E-01	0.2
Tmem80	4E-01	0.2	Hint1	6E-08	0.2
BC065397	3E-01	0.2	Mospd3	6E-05	0.2
Ddt	1E-01	0.2	BC065397	7E-02	0.2
Bcat2	5E-01	0.2	Nfatc1	2E-01	0.2
Gab1	2E-01	0.2	Cops9	9E-02	0.2
Twsg1	6E-01	0.2	Fnta	4E-03	0.2
Immp2l	7E-02	0.2	Hypk	2E-02	0.2
Dok1	5E-01	0.2	Hist1h1c	2E-01	0.2
Gadd45a	8E-02	0.2	Paip2b	2E-02	0.2
Hspe1-rs1	1E-01	0.2	Zfp24	7E-03	0.2
Slc30a9	7E-01	0.2	Clint1	4E-02	0.2
Arhgef6	2E-02	0.2	Wdr89	1E-03	0.2
Fbxl6	2E-02	0.2	Mrpl55	2E-04	0.2
Mthfd2	7E-01	0.2	Erp29	3E-01	0.2
Taf13	9E-01	0.2	Atp1a1	8E-01	0.2
Spire1	8E-01	0.2	Higd2a	3E-03	0.2
Il17ra	1E-02	0.2	Taf15	1E-02	0.2
Cd320	1E-01	0.2	Aen	2E-05	0.2
Trp53inp1	9E-01	0.2	Epb41l4aos	8E-02	0.2
Rab28	4E-01	0.2	Cebpb	4E-01	0.2
Polr3e	3E-01	0.2	Gys1	1E-03	0.2
Pakap	4E-01	0.2	Rarg	1E+00	0.2
Pttg1	7E-01	0.2	Mob2	7E-05	0.2
Alad	1E-01	0.2	Ddx10	2E-08	0.2
Ogt	4E-03	0.2	Mettl23	2E-06	0.2
Polr2m	6E-02	0.2	Mthfd2	1E-01	0.2
Cables1	6E-01	0.2	Selenoo	1E-02	0.2
Zfp566	3E-01	0.2	Nbdy	1E-03	0.2
Avpi1	1E-01	0.2	Serpinh1	2E-18	0.2
Gm26749	7E-01	0.2	Tomm20	8E-01	0.2
Bcl2	4E-01	0.2	Hint2	1E-01	0.2
Usp37	2E-01	0.2	Zyg11b	2E-03	0.2
Atp1a1	2E-01	0.2	Atf6	1E-02	0.2

Articular/Synovial Fibroblast					
Control			$Notch2^{tm1.1Ecan}$		
Gene	*p*	FC	Gene	*p*	FC
Lima1	6E-33	0.5	Lysmd2	2E-04	0.5
Trim25	9E-03	0.5	Arid5b	8E-22	0.5
Clip4	1E-09	0.5	Mtmr11	1E-01	0.5
Man2b1	6E-11	0.5	Smurf1	2E-04	0.5
Dennd4c	3E-13	0.5	Mafb	9E-08	0.5
Tmem241	1E-09	0.5	Mfsd8	1E-03	0.5
Dennd5b	3E-07	0.5	Ttll1	4E-03	0.5
C1s1	2E-07	0.5	Klf3	3E-11	0.5
Ank2	1E-25	0.5	Rab43	2E-05	0.5
Ddr2	2E-40	0.5	Snta1	3E-05	0.5
Abcg2	1E-07	0.5	Fgf13	8E-02	0.5
Abhd11	2E-04	0.5	Ctso	6E-04	0.5
Tnks	7E-15	0.5	Pofut1	3E-04	0.5
Exosc7	2E-09	0.5	C1s1	2E-04	0.5
Mthfs	1E-04	0.5	Pqlc1	2E-02	0.5
Bach1	9E-09	0.5	Fam13c	3E-08	0.5
Mapkapk3	7E-02	0.5	Ssbp2	2E-11	0.5
Mblac2	3E-03	0.5	Ttyh3	8E-07	0.5
H19	3E-01	0.5	Pqlc3	2E-02	0.5
Ctps2	2E-10	0.5	Rpgr	3E-01	0.5
Klf4	2E-27	0.5	Zc3h3	4E-02	0.5
Abl2	1E-14	0.5	Gns	1E-16	0.5
Meis2	1E-14	0.5	Mknk2	2E-07	0.5
Galnt2	7E-16	0.5	Ifi30	3E-16	0.5
Snx29	2E-11	0.5	Ifngr1	1E-04	0.5
Xpnpep1	3E-12	0.5	Insig2	2E-09	0.5
Abl1	2E-09	0.5	Tle2	5E-02	0.5
Pfkfb4	2E-07	0.5	Aifm2	1E-01	0.5
Arid5b	6E-39	0.5	Grn	2E-31	0.5
Ezh1	2E-09	0.5	Cc2d2a	5E-05	0.5
Ankrd29	8E-05	0.5	Casp9	2E-02	0.5
Myzap	1E-04	0.5	Trim47	5E-06	0.5
Cys1	4E-10	0.5	Laptm4b	7E-06	0.5
Il17rd	2E-03	0.5	Usp42	2E-02	0.5
Pxmp4	3E-08	0.5	Zfp651	2E-03	0.5
Aldh1a1	8E-21	0.5	Wdfy1	3E-06	0.5
D6Wsu163e	1E-03	0.5	Pik3r4	6E-05	0.5
Ank3	7E-09	0.5	Ubl7	5E-03	0.5
Efr3b	1E-03	0.5	Myo9a	3E-15	0.5
Map2k6	3E-04	0.5	Papolg	6E-01	0.5
Smcr8	1E-03	0.5	S100a11	1E-45	0.5
Magee1	9E-04	0.5	Cacna2d1	6E-11	0.5
Akr1b10	3E-03	0.5	Secisbp2l	6E-07	0.5
Myl12b	1E-24	0.5	H2-T22	2E-05	0.5
Atrnl1	7E-18	0.5	Prps2	2E-02	0.5
Glmp	3E-28	0.5	Vps18	2E-03	0.5
Tacc1	8E-16	0.5	Vps13c	4E-14	0.5
Ipmk	4E-04	0.5	Col6a1	3E-37	0.5
Wbp11	3E-13	0.5	Nectin3	5E-07	0.5
Insig2	1E-11	0.5	Klc1	2E-08	0.5
Nptxr	5E-03	0.5	Ttl	1E-01	0.5
Adgrl2	2E-21	0.5	Eng	2E-05	0.5
Igf2bp3	4E-14	0.5	Dapp1	5E-03	0.5
Col4a3bp	6E-15	0.5	Cav1	4E-14	0.5
Kcnd2	2E-06	0.5	Cep162	1E-04	0.5
Rtn4	1E-47	0.5	Prkca	7E-14	0.5
Macrod2	2E-07	0.5	Elf4	2E-08	0.5
Fbxo30	6E-08	0.5	Iah1	1E-09	0.5
Acadl	3E-25	0.5	Frmd6	1E-11	0.5
Abhd14b	8E-09	0.5	Tmem184b	1E-04	0.5
Nhlrc2	5E-12	0.5	Plekhb2	2E-03	0.5
Ttyh3	4E-07	0.5	Gpm6b	1E-16	0.5
Myo1c	1E-14	0.5	Cyp2j6	1E-02	0.5
Pikfyve	5E-10	0.5	Pip5k1b	2E-02	0.5

Limb Mesenchyme

Control			*Notch2* $^{tm1.1Ecan}$		
Gene	*p*	FC	Gene	*p*	FC
Mcur1	1E-06	0.3	Pex11g	2E-03	0.3
Commd9	3E-08	0.3	Ssbp3	5E-18	0.3
Irf2bpl	2E-10	0.3	Pafah1b3	6E-09	0.3
Arhgap28	2E-16	0.3	Oat	1E-21	0.3
Slc2a10	1E-01	0.3	Gpx7	3E-23	0.3
Pfn1	6E-50	0.3	Fer	2E-13	0.3
Plod3	2E-11	0.3	Rps2	7E-85	0.3
Fech	1E-06	0.3	Tlnrd1	4E-07	0.3
Atf5	3E-27	0.3	Gmpr2	5E-04	0.3
Mrpl22	1E-06	0.3	Slc31a2	4E-06	0.3
Evc2	1E-06	0.3	Mxd4	1E-30	0.3
Ifi27	3E-34	0.3	Rexo4	2E-04	0.3
Wwc2	7E-17	0.3	Zfp617	1E-03	0.3
Ccnd3	2E-07	0.3	Coasy	5E-05	0.3
Ccdc32	5E-04	0.3	Trim12a	2E-07	0.3
Eef1aknmt	5E-01	0.3	Bckdhb	5E-04	0.3
Selenow	7E-32	0.3	Wsb2	2E-14	0.3
Tle6	6E-04	0.3	Rabif	4E-08	0.3
Cdkn2aip	2E-05	0.3	Fam189b	2E-05	0.3
Zfpl1	2E-09	0.3	Npdc1	2E-27	0.3
Capns1	1E-36	0.3	Phf23	2E-10	0.3
Tmtc3	4E-05	0.3	Glis3	2E-12	0.3
Siae	2E-02	0.3	Samd1	7E-10	0.3
S1pr2	4E-03	0.3	Dusp23	1E-04	0.3
Itga9	7E-02	0.3	Atp6v0d1	5E-22	0.3
Shisa5	8E-15	0.3	Camkk2	4E-05	0.3
Rala	1E-24	0.3	Esd	4E-34	0.3
Sulf2	3E-07	0.3	Fnbp1	2E-06	0.3
Fam171b	1E-06	0.3	Rhoj	2E-10	0.3
Ankrd50	1E-03	0.3	Atp5o	2E-34	0.3
Stx2	5E-05	0.3	Eva1b	7E-23	0.3
Actg1	7E-34	0.3	Alg11	2E-06	0.3
Ubl4a	7E-10	0.3	Mboat7	7E-07	0.3
Ube2d1	5E-06	0.3	Fam118a	6E-04	0.3
Dip2c	2E-27	0.3	Sord	2E-09	0.3
Chmp6	3E-05	0.3	Tmem121	2E-06	0.3
Tacc1	8E-06	0.3	Ankrd50	1E-06	0.3
Bet1l	3E-14	0.3	Nox4	6E-25	0.3
Tmem43	1E-05	0.3	Smyd5	3E-03	0.3
Satb2	5E-04	0.3	Dpp7	5E-11	0.3
Htatip2	1E-04	0.3	Bloc1s1	2E-06	0.3
Ddx28	1E-03	0.3	Npepl1	3E-10	0.3
Atp13a3	2E-08	0.3	Rac3	2E-07	0.3
Hs1bp3	1E-02	0.3	Scd2	3E-16	0.3
Ak5	8E-10	0.3	Bak1	1E-13	0.3
Ptpn9	8E-12	0.3	Plxna1	9E-07	0.3
Macrod2	4E-06	0.3	Dpp3	2E-08	0.3
Jrkl	2E-03	0.3	Mthfd2	2E-21	0.3
Nsg1	4E-07	0.3	Col3a1	1E-60	0.3
Mark4	3E-04	0.3	Rala	5E-23	0.3
Cdk14	2E-28	0.3	Mfsd5	4E-10	0.3
Myo9a	4E-14	0.3	Commd8	3E-13	0.3
Apba3	5E-02	0.3	Mdp1	4E-09	0.3
Traf4	2E-04	0.3	Heg1	7E-02	0.3
Pelo	5E-06	0.3	Zfp593	2E-09	0.3
Dctn3	1E-16	0.3	Stx4a	2E-13	0.3
Kat5	3E-03	0.3	Syngr2	7E-17	0.3
Csrp2	5E-06	0.3	Tab2	2E-12	0.3
Czib	2E-05	0.3	Pycr1	6E-13	0.3
Esd	8E-36	0.3	Sars	8E-35	0.3
Rab34	1E-19	0.3	Fam8a1	3E-07	0.3
Cadm2	9E-04	0.3	Samhd1	9E-15	0.3
1500015L24Rik	1E-09	0.3	Fndc4	8E-06	0.3
Mob3a	8E-03	0.3	Noa1	2E-02	0.3

Chondrogenic

Control			*Notch2* $^{tm1.1Ecan}$		
Gene	*p*	FC	Gene	*p*	FC
Tgoln1	6E-20	0.3	Rpp40	1E-03	0.3
Eefsec	1E-04	0.3	Hbs1l	1E-10	0.3
Hmgb3	4E-02	0.3	Acp6	3E-02	0.3
Ppp1r15a	3E-15	0.3	Gm15952	4E-01	0.3
Sar1b	8E-24	0.3	Hint3	2E-12	0.3
Nfe2l1	2E-40	0.3	Trib2	6E-05	0.3
Rhbdd3	2E-02	0.3	Bbx	3E-18	0.3
Wfs1	7E-05	0.3	Rnf41	6E-03	0.3
Nfat5	4E-27	0.3	Ccdc88a	3E-23	0.3
Syne1	4E-27	0.3	Abca5	7E-06	0.3
Ubqln4	3E-04	0.3	Ctsf	2E-11	0.3
Tbc1d1	7E-05	0.3	Eaf2	2E-02	0.3
Gtf2ird2	7E-02	0.3	Ptpn2	6E-11	0.3
Jmy	2E-10	0.3	Snx25	1E-01	0.3
Rap2c	1E-07	0.3	Zfp423	2E-05	0.3
Rpp40	7E-04	0.3	Cldn12	2E-03	0.3
Cdc37l1	4E-14	0.3	Dph6	2E-11	0.3
Ift20	4E-36	0.3	Mpped2	5E-05	0.3
Ngrn	1E-08	0.3	Cd151	2E-09	0.3
Snrpb2	1E-19	0.3	Snai1	2E-04	0.3
Pacs2	2E-01	0.3	Gm44686	1E-02	0.3
Ank	2E-11	0.3	St3gal3	7E-07	0.3
Zfat	7E-02	0.3	Dbp	4E-09	0.3
Pgm3	4E-08	0.3	0610040B10Rik	3E-02	0.3
Glcci1	6E-09	0.3	Cd320	2E-09	0.3
AY036118	4E-02	0.3	Sod2	2E-25	0.3
Apoo	9E-04	0.3	Lins1	3E-02	0.3
Gtpbp4	6E-24	0.3	Hist1h4i	2E-02	0.3
Oxnad1	7E-03	0.3	Gls	1E-16	0.3
Spcs2	2E-32	0.3	Cep85l	3E-02	0.3
BC031181	6E-31	0.3	Fam98c	1E-02	0.3
Cpeb3	1E-09	0.3	Nubpl	1E-05	0.3
Pebp1	2E-69	0.3	Sox12	1E-01	0.3
Rpp38	3E-05	0.3	6430590A07Rik	3E-02	0.3
Aldh18a1	8E-10	0.3	1810024B03Rik	3E-04	0.3
Mto1	2E-04	0.3	Zbtb8os	2E-08	0.3
Atpsckmt	3E-06	0.3	Gm34455	4E-02	0.3
Rab28	8E-16	0.3	Tmem33	3E-08	0.3
6030443J06Rik	4E-08	0.3	Cnpy3	2E-08	0.3
Sdad1	1E-07	0.3	Gm15417	2E-10	0.3
Tmem101	1E-06	0.3	Fth1	4E-86	0.3
Ccdc88a	2E-14	0.3	Trim35	1E-16	0.3
2810402E24Rik	5E-03	0.3	Krr1	6E-05	0.3
Srsf9	5E-28	0.3	Map3k3	2E-10	0.3
Fahd1	3E-06	0.3	Csf2ra	2E-04	0.3
Aasdh	7E-03	0.3	2610044O15Rik8	8E-02	0.3
1700084C06Rik	3E-03	0.3	Tmem161a	9E-06	0.3
Mad1l1	4E-04	0.3	Mbd6	2E-03	0.3
Armh4	2E-13	0.3	Abraxas1	1E-02	0.3
Tsacc	3E-06	0.3	Tbc1d22a	4E-05	0.3
Taf1a	2E-02	0.3	Snhg3	1E-09	0.3
Boc	2E-12	0.3	E130102H24Rik	4E-03	0.3
Phactr2	4E-10	0.3	Cdc14a	1E-01	0.3
Rab33b	3E-05	0.3	Uri1	6E-15	0.3
Fam174a	1E-16	0.3	Shld1	3E-02	0.3
Usp24	6E-09	0.3	Spcs2	3E-32	0.3
Ppa1	3E-15	0.3	Zfp827	2E-06	0.3
B3galnt2	2E-03	0.3	Dcbld1	3E-02	0.3
Malat1	1E-63	0.3	Polr3f	4E-01	0.3
Ssr3	3E-41	0.3	Cisd2	2E-17	0.3
Tomm20	6E-41	0.3	Stau2	3E-05	0.3
Pura	3E-25	0.3	Uchl3	3E-13	0.3
Tmed9	1E-57	0.3	Gmpr	7E-03	0.3
Nolc1	6E-11	0.3	Dym	2E-09	0.3

Fibroblast

Control			*Notch2* $^{tm1.1Ecan}$		
Gene	*p*	FC	Gene	*p*	FC
Coro1c	1E-05	0.3	Pde4d	2E-13	0.4
Smc1a	3E-17	0.3	D930016D06Rik	2E-03	0.4
Runx1t1	2E-21	0.3	St5	8E-14	0.4
1600010M07Rik	8E-05	0.3	Zhx3	3E-10	0.4
Abhd10	4E-05	0.3	Gm16133	1E-04	0.4
Arhgap10	8E-11	0.3	Sp3	3E-09	0.4
Zfp871	6E-08	0.3	Erap1	2E-02	0.4
Gm32618	5E-03	0.3	Adck5	2E-03	0.4
Abcc1	2E-07	0.3	Csnk1e	8E-09	0.4
Ntn1	1E-03	0.3	Lynx1	3E-03	0.4
Trim2	2E-06	0.3	Gpm6b	2E-11	0.4
Rhog	9E-06	0.3	Dcps	2E-06	0.4
Adss	5E-10	0.3	Ilf2	2E-12	0.4
Bcl7a	5E-03	0.3	Dnajc10	1E-18	0.4
C330018D20Rik	2E-01	0.3	E130308A19Rik	4E-02	0.4
Top3a	3E-02	0.3	Usp54	5E-06	0.4
Agpat3	5E-08	0.3	Dhcr7	3E-05	0.4
Pcp4l1	3E-03	0.3	Samd14	2E-03	0.4
Lrrc32	2E-02	0.3	Ttyh3	1E-06	0.4
4930503L19Rik	9E-03	0.3	Utp6	1E-06	0.4
Zfp568	4E-03	0.3	Kat8	2E-04	0.4
Ets2	1E-05	0.3	Ogfrl1	1E-04	0.4
Gipc1	1E-06	0.3	Adm	4E-09	0.4
Hspa2	6E-04	0.3	Armcx4	2E-05	0.4
Ccnyl1	1E-03	0.3	Zcchc24	8E-12	0.4
Ermp1	1E-04	0.3	Htra3	2E-09	0.4
Itga2b	9E-02	0.3	Lgals1	2E-42	0.4
Zdhhc16	2E-03	0.3	Thop1	7E-05	0.4
Soga1	3E-04	0.3	Farp1	2E-22	0.4
Cep170b	2E-02	0.3	3300002I08Rik	1E-03	0.4
Fut10	4E-04	0.3	Cinp	3E-07	0.4
Rab3b	6E-03	0.3	Me3	1E-07	0.4
1700086O06Rik	3E-03	0.3	Pdpr	2E-02	0.4
Rassf8	3E-06	0.3	Mllt1	9E-02	0.4
Mfge8	2E-17	0.3	Ankrd42	1E-03	0.4
Sema3a	2E-05	0.3	Zfp808	5E-03	0.4
Lrrk1	2E-12	0.3	Mblac2	1E-02	0.4
Gli3	4E-17	0.3	Fbxl14	4E-03	0.4
Pgd	8E-06	0.3	Smtn	5E-08	0.4
Macf1	7E-27	0.3	Dpysl2	4E-19	0.4
Aarsd1	3E-07	0.3	Zfp385a	4E-04	0.4
Iqgap1	2E-17	0.3	Pds5b	1E-13	0.4
Dgkh	1E-04	0.3	Twsg1	5E-13	0.4
Rp2	2E-03	0.3	P4ha2	3E-20	0.4
Nup35	2E-03	0.3	Dapp1	4E-04	0.4
Dip2a	2E-01	0.3	Nectin2	9E-03	0.4
Suco	8E-05	0.3	Agfg2	6E-05	0.4
Slc35a4	4E-05	0.3	Bicd2	7E-13	0.4
Loxl4	7E-03	0.3	Paxip1	8E-05	0.4
Msh2	6E-04	0.3	Tgfbr2	6E-19	0.4
Scara3	2E-12	0.3	Crtc3	3E-15	0.4
Hdgf	7E-24	0.3	Cd109	3E-10	0.4
Alg14	4E-05	0.3	Ckb	4E-06	0.4
Otud1	8E-05	0.3	Lama3	6E-03	0.4
Sf3a3	3E-05	0.3	Scoc	1E-16	0.4
Agps	1E-07	0.3	Kank2	5E-12	0.4
Fam110b	3E-05	0.3	Rnaseh2a	9E-07	0.4
Zfp729a	1E-01	0.3	Zyx	3E-17	0.4
Platr22	1E-02	0.3	Tubgcp2	3E-06	0.4
Manba	2E-01	0.3	Lsm4	7E-14	0.4
Sacs	1E-03	0.3	Galm	2E-04	0.4
Brat1	1E-03	0.3	Mov10	3E-04	0.4
2900089D17Rik	4E-03	0.3	1600010M07Rik	3E-05	0.4
Slc22a21	6E-03	0.3	Tpm2	4E-25	0.4

Undefined

Control			*Notch2* $^{tm1.1Ecan}$		
Gene	*p*	FC	Gene	*p*	FC
Hprt	8E-02	0.2	Fem1c	5E-05	0.2
Impact	2E-01	0.2	Tomm7	6E-03	0.2
Dzip1l	7E-01	0.2	Hotairm1	8E-02	0.2
Brd3os	8E-02	0.2	Nomo1	4E-01	0.2
Noc3l	9E-01	0.2	Malsu1	1E-05	0.2
Desi2	4E-03	0.2	Tm9sf4	3E-03	0.2
Zfp26	1E-04	0.2	Mcfd2	5E-06	0.2
Tmbim1	2E-01	0.2	Wbp2	2E-04	0.2
Cep83os	1E+00	0.2	Tmem199	5E-02	0.2
Ggact	1E+00	0.2	Emc7	2E-01	0.2
Srm	6E-01	0.2	Heatr1	3E-02	0.2
Brcc3	1E-01	0.2	Ica1	2E-01	0.2
Ttc27	5E-01	0.2	Ascc1	1E-02	0.2
Ahi1	1E-01	0.2	Zrsr2	8E-06	0.2
Whamm	6E-01	0.2	Mif	6E-01	0.2
Nudt14	8E-02	0.2	Slc25a14	2E-02	0.2
Sod2	4E-02	0.2	Cd109	2E-02	0.2
Gm14296	2E-01	0.2	Mrps10	7E-06	0.2
Mcm2	5E-01	0.2	Vhl	3E-03	0.2
Tgfb3	4E-02	0.2	Diablo	2E-03	0.2
Mrps2	3E-03	0.2	Aldh18a1	9E-02	0.2
Cers2	6E-01	0.2	Tceal1	2E-02	0.2
Park7	5E-06	0.2	Rps21	3E-58	0.2
3110082I17Rik	7E-01	0.2	Prickle1	7E-03	0.2
C430049B03Rik	4E-03	0.2	Taf13	9E-05	0.2
Nop10	4E-05	0.2	Tmem230	2E-01	0.2
Bpnt1	1E-01	0.2	Rap2c	8E-04	0.2
Bckdk	8E-01	0.2	Cep57	4E-03	0.2
Fam185a	7E-01	0.2	Cdipt	1E-05	0.2
Gys1	5E-01	0.2	M6pr	4E-09	0.2
Daam2	9E-02	0.2	Mis12	4E-06	0.2
Gphn	3E-01	0.2	Rps7	5E-33	0.2
Chd2	7E-01	0.2	Tmbim1	3E-02	0.2
Usp2	3E-01	0.2	Gm19705	2E-01	0.2
Ippk	2E-01	0.2	Tmem179b	1E-03	0.2
Dkc1	1E-01	0.2	Zfp692	4E-02	0.2
Slc16a1	2E-01	0.2	Spry2	1E-01	0.2
Sesn3	1E-02	0.2	Sec23b	2E-03	0.2
Eif1	4E-33	0.2	Shisa2	1E-02	0.2
Anp32b	7E-04	0.2	Tor3a	4E-03	0.2
Gfod2	3E-01	0.2	Ppt1	9E-04	0.2
Pitpnm2	3E-01	0.2	Ndufaf7	2E-05	0.2
Pja2	8E-02	0.2	Ibtk	6E-07	0.2
Irx3	5E-05	0.2	Xndc1	3E-03	0.2
Arhgef18	8E-01	0.2	Trp53i13	5E-04	0.2
A530072M11Rik	1E-01	0.2	Agpat1	4E-08	0.2
Smim10l1	8E-01	0.2	Alkbh7	1E-02	0.2
Mrpl38	7E-01	0.2	Bola1	1E-02	0.2
Med19	6E-01	0.2	Slc4a3	4E-03	0.2
Tmed9	4E-12	0.2	Smim10l1	2E-02	0.2
B3gnt2	1E-01	0.2	Atf5	8E-01	0.2
Taldo1	9E-02	0.2	Igip	6E-01	0.2
Mtif2	9E-02	0.2	Rab33b	2E-03	0.2
Tmem267	3E-01	0.2	Rasl11a	4E-02	0.2
Gin1	3E-01	0.2	Ccl25	3E-03	0.2
Gpx4	2E-26	0.2	Grpel1	3E-06	0.2
Tmem185a	4E-01	0.2	Rpl32	6E-27	0.2
Acaca	2E-02	0.2	Pfkl	2E-03	0.2
Dop1b	4E-01	0.2	Tmed10	2E-03	0.2
Ndc1	3E-01	0.2	Naa50	3E-05	0.2
Gabarapl2	4E-02	0.2	2310033P09Rik	2E-02	0.2
Chmp2b	5E-01	0.2	A930015D03Rik	2E-02	0.2
2410006H16Rik	8E-08	0.2	Zfp469	6E-02	0.2
Mak16	6E-01	0.2	Hspa1a	6E-03	0.2

Articular/Synovial Fibroblast

Control			*Notch2* $^{tm1.1Ecan}$		
Gene	*p*	FC	Gene	*p*	FC
Plec	1E-27	0.5	Ikbkg	7E-03	0.5
Stx2	1E-04	0.5	Sqor	1E-04	0.5
Tnfrsf1a	2E-14	0.5	Gsc	4E-03	0.5
Pik3r2	5E-08	0.5	Ro60	2E-06	0.5
Synj1	8E-08	0.5	Ptpn21	6E-07	0.5
Pml	2E-03	0.5	Glb1l	4E-03	0.5
Tmsb15b1	8E-03	0.5	Tuba1a	9E-24	0.5
Slc39a6	2E-14	0.5	Hipk3	2E-06	0.5
Lrsam1	6E-06	0.5	Mark1	9E-01	0.5
Zfp449	3E-02	0.5	Sft2d1	8E-04	0.5
Acyp2	2E-19	0.5	Lgi2	1E-02	0.5
Zmiz2	7E-05	0.5	Mcc	3E-16	0.5
Ephx1	2E-20	0.5	Mex3c	6E-06	0.5
Fam53b	1E-05	0.5	Lpin1	5E-03	0.5
Fam172a	1E-27	0.5	Slc9a9	3E-16	0.5
Prkx	2E-05	0.5	Mical1	1E-06	0.5
Klhl20	4E-02	0.5	Rnh1	1E-18	0.5
Slc24a3	5E-20	0.5	Btd	1E-05	0.5
Prnp	2E-37	0.5	Togaram1	2E-04	0.5
Efna4	4E-02	0.5	Grb14	3E-13	0.5
Atp6v1b2	5E-10	0.5	Crem	2E-04	0.5
Cep97	5E-02	0.5	Parp1	3E-06	0.5
P2ry10b	1E-01	0.5	Xpnpep1	6E-09	0.5
Antxr2	2E-13	0.5	Mex3d	2E-02	0.5
Syngr2	2E-11	0.5	Btbd3	2E-06	0.5
Klhl42	1E-02	0.5	Exoc1	2E-04	0.5
Fam241b	8E-02	0.5	Lrrc49	7E-05	0.5
Eif2ak2	4E-12	0.5	Plekhm1	5E-02	0.5
Slc26a11	5E-03	0.5	Tbc1d32	1E-04	0.5
Mical1	2E-04	0.5	Gm12743	8E-05	0.5
Gm16201	1E-04	0.5	Pacs2	5E-06	0.5
P4htm	4E-03	0.5	Tram2	9E-09	0.5
Trib1	4E-07	0.5	Zfp667	2E-02	0.5
Slc35a5	5E-04	0.5	Tmtc1	6E-09	0.5
Flii	5E-12	0.5	Smim1	8E-10	0.5
Lgals8	3E-10	0.5	Naglu	1E-08	0.5
Abhd4	3E-07	0.5	Pde10a	3E-07	0.5
Coa5	8E-06	0.5	Eif3e	1E-30	0.5
Zfyve26	2E-05	0.5	Cyld	2E-08	0.5
Shisa5	8E-20	0.5	Chst12	2E-04	0.5
Map1a	5E-13	0.5	Scpep1	2E-19	0.5
Cltb	2E-15	0.5	Phactr1	5E-07	0.5
Wasf2	1E-34	0.5	Zfp983	2E-02	0.5
Wasl	5E-15	0.5	Flot2	2E-08	0.5
Ndn	3E-16	0.5	Parp3	8E-13	0.5
Rnf13	5E-20	0.5	Echdc3	7E-02	0.5
Ssh1	4E-04	0.5	Tspyl2	2E-02	0.5
Pmp22	2E-29	0.5	Esrra	2E-01	0.5
Dnajc13	2E-15	0.5	Fbxl14	1E-01	0.5
Ccnd1	1E-12	0.5	Dennd4c	1E-08	0.5
Znrf2	2E-05	0.5	Tent2	4E-12	0.5
Zfp90	5E-04	0.5	Rcan1	3E-03	0.5
Rasa4	1E-04	0.5	Simc1	2E-02	0.5
Tmem106b	1E-13	0.5	Smug1	1E-02	0.5
Unc93b1	1E-03	0.5	Snap29	5E-03	0.5
Taf6l	3E-02	0.5	Comt	4E-16	0.5
Gba	1E-07	0.5	E430024I08Rik	3E-03	0.5
Meis3	1E-02	0.5	Gm48678	2E-01	0.5
Acsl4	4E-05	0.5	Impdh1	8E-03	0.5
Il16	8E-08	0.5	Ap2a2	1E-08	0.5
Ccdc80	4E-19	0.5	Golm1	5E-04	0.5
Rcan1	3E-04	0.5	Enpp5	5E-03	0.5
Stn1	6E-03	0.5	Fancf	5E-03	0.5
Robo2	1E-13	0.5	Rtca	7E-05	0.5

Limb Mesenchyme					
Control			*Notch2*$^{tm1.1Ecan}$		
Gene	*p*	FC	Gene	*p*	FC
Lactb	3E-05	0.3	Tagln2	2E-33	0.3
Fcgrt	7E-24	0.3	Ctnnbip1	3E-10	0.3
Kctd13	2E-02	0.3	Fkbp1a	4E-26	0.3
Dennd2a	6E-07	0.3	Pkd2	7E-15	0.3
Cryab	6E-19	0.3	Rps20	3E-103	0.3
Cd276	2E-03	0.3	Plekhg2	1E-10	0.3
Yif1b	6E-15	0.3	Dmap1	1E-03	0.3
Ebp	3E-07	0.3	Tmed5	7E-13	0.3
Rps20	1E-133	0.3	Emp1	5E-20	0.3
Capn2	2E-15	0.3	Wasf2	2E-23	0.3
Arap1	3E-03	0.3	Sirt6	5E-04	0.3
Vat1	3E-26	0.3	Klhl42	7E-04	0.3
Mrpl16	2E-02	0.3	Pde12	2E-02	0.3
Rhog	4E-06	0.3	Cacna1d	2E-03	0.3
Tuba1a	5E-30	0.3	Ccndbp1	2E-07	0.3
Adm	3E-15	0.3	Cactin	2E-04	0.3
Gxylt2	3E-16	0.3	Unc93b1	7E-11	0.3
Ppp2r3c	5E-02	0.3	1700037H04Rik	8E-07	0.3
Emp2	8E-11	0.3	Itpripl2	2E-13	0.3
Ethe1	5E-05	0.3	Stradb	1E-03	0.3
Prcc	4E-03	0.3	Psat1	4E-14	0.3
Numbl	3E-10	0.3	Gtf2e1	4E-03	0.3
Ints12	1E-01	0.3	Yif1a	2E-14	0.3
Ddah1	6E-03	0.3	Zfp358	6E-06	0.3
Alg8	8E-02	0.3	Golm1	5E-10	0.3
Efr3a	2E-11	0.2	Fdx1	3E-11	0.3
Arhgdia	6E-24	0.2	Fcgrt	5E-29	0.3
Mpg	1E-06	0.2	Rpl9-ps6	8E-12	0.3
Gopc	4E-03	0.2	Rab5c	9E-15	0.3
Cmtm6	3E-02	0.2	Gpx8	7E-24	0.2
Ifitm2	7E-39	0.2	Trim26	2E-07	0.2
Heca	6E-06	0.2	Gjc1	1E-13	0.2
Gpx8	2E-22	0.2	Il17rc	5E-08	0.2
Msmo1	6E-07	0.2	Eif2b2	1E-09	0.2
Arsa	2E-04	0.2	Rangrf	5E-06	0.2
Ercc1	9E-06	0.2	Cdr1os	2E-02	0.2
Tshz3	3E-09	0.2	Map2k1	5E-11	0.2
Atp6v0d1	3E-14	0.2	Vrk3	1E-05	0.2
Cpa6	1E-03	0.2	Emc10	3E-25	0.2
Cyba	6E-18	0.2	Sptbn1	4E-31	0.2
Chst14	4E-06	0.2	H2-T22	2E-09	0.2
Dbnl	7E-09	0.2	Hivep3	2E-04	0.2
D6Wsu163e	8E-02	0.2	Copz2	3E-31	0.2
Itpripl2	1E-07	0.2	Ciao2a	4E-11	0.2
Eef2	1E-59	0.2	Mex3d	3E-06	0.2
Dctn2	2E-21	0.2	Paxx	7E-09	0.2
Cdk19	1E-04	0.2	Eif1a	8E-23	0.2
Fkbp1a	1E-29	0.2	Mlycd	2E-03	0.2
Psmg3	5E-05	0.2	Armcx3	3E-13	0.2
Mboat7	4E-03	0.2	Lmna	4E-46	0.2
C1qtnf2	4E-02	0.2	Pdhx	9E-03	0.2
Uap1l1	8E-11	0.2	Cep170	2E-13	0.2
Vps26b	2E-03	0.2	Pbx1	6E-23	0.2
Vamp4	8E-09	0.2	Anxa1	2E-34	0.2
5730409E04Rik	4E-05	0.2	Bcor	1E-07	0.2
Acat2	5E-03	0.2	Sfxn3	2E-13	0.2
Snhg5	2E-06	0.2	Gpr153	1E-05	0.2
Rhbdf1	3E-05	0.2	Map1a	4E-11	0.2
Chst12	1E-03	0.2	Wdr55	1E-03	0.2
Osbpl5	7E-05	0.2	Adam15	1E-10	0.2
Atf7ip	2E-04	0.2	Rpl28	2E-88	0.2
Dzip1	4E-09	0.2	Agl	1E-07	0.2
Zfp251	1E-01	0.2	Pdzd4	3E-04	0.2
Gnpda2	2E-06	0.2	Bmp2k	3E-07	0.2

Chondrogenic					
Control			*Notch2*$^{tm1.1Ecan}$		
Gene	*p*	FC	Gene	*p*	FC
Ficd	7E-03	0.3	Mid2	3E-03	0.3
Pigf	5E-06	0.3	Rida	4E-08	0.3
Ppp1r13b	3E-04	0.3	Timm8b	9E-27	0.3
2610507I01Rik	6E-02	0.3	Zyg11b	1E-12	0.3
Rab11fip3	8E-07	0.3	Erp29	1E-37	0.3
Unc119	8E-09	0.3	Spata5	6E-09	0.3
Coq9	3E-04	0.3	Gm16740	6E-04	0.3
Tmem199	8E-04	0.3	Lncpint	2E-12	0.3
Foxk1	3E-03	0.3	Asf1a	2E-05	0.3
5730522E02Rik	2E-02	0.3	1110019D14Rik	3E-06	0.3
Wdr92	3E-03	0.3	Tnfrsf10b	4E-03	0.3
Pyroxd1	4E-03	0.3	Zscan12	5E-01	0.3
Sema5a	3E-05	0.3	Nckap5	1E-14	0.3
Fam162a	5E-19	0.3	2410002F23Rik	8E-07	0.3
Xrcc6	5E-06	0.3	Sdad1	4E-06	0.3
Slc20a2	1E-05	0.3	Tefm	2E-02	0.3
Isoc1	2E-10	0.3	Pebp1	7E-57	0.3
Zfp523	1E-03	0.3	Tomm20	1E-35	0.3
Hist1h1d	2E-02	0.3	1810062O18Rik	8E-02	0.3
Clk4	2E-05	0.3	Ccdc58	3E-08	0.3
Srp14	5E-49	0.3	Ccser1	1E-09	0.3
Zfp329	4E-06	0.3	Minpp1	8E-05	0.3
Lncppara	3E-14	0.3	Plpbp	1E-07	0.3
Scarb2	8E-12	0.3	A230057D06Rik	1E-06	0.3
Dmac2	2E-03	0.3	Plod2	2E-35	0.3
Cdc42bpa	7E-24	0.3	2010320M18Rik	2E-04	0.3
Cpq	4E-28	0.3	Gbe1	8E-06	0.3
Zranb3	4E-03	0.3	Hoxa7	1E-03	0.3
D130017N08Rik	6E-03	0.3	Zfr	2E-25	0.3
Cyp4f13	2E-03	0.3	Srpr	2E-24	0.3
Mkrn1	7E-09	0.3	Aff3	3E-32	0.3
Cebpg	2E-10	0.3	Odc1	5E-14	0.3
Rnf181	9E-06	0.3	Uxs1	3E-08	0.3
Rdh10	2E-03	0.3	Cdc42bpa	1E-16	0.3
Dgke	6E-02	0.3	Fam126b	9E-04	0.3
Fxyd6	1E-09	0.3	Gpd2	2E-06	0.3
2610020C07Rik	4E-03	0.3	D330023K18Rik	1E-02	0.3
Mettl26	5E-08	0.3	Aga	7E-21	0.3
Tmem60	1E-14	0.3	Ncbp1	5E-04	0.3
E4f1	1E-02	0.3	Igf2bp2	4E-07	0.3
Tbc1d4	5E-02	0.3	Znrd1as	1E-04	0.3
Magoh	4E-19	0.3	Ift20	1E-31	0.3
Erp29	6E-43	0.3	Ubxn2b	8E-03	0.3
Nifk	3E-10	0.3	Hyou1	9E-07	0.3
Cdpf1	7E-07	0.3	Trmt10c	3E-04	0.3
Plekha5	9E-08	0.3	Ghitm	8E-25	0.3
Dnajc7	8E-19	0.3	Wrap73	4E-03	0.3
Otud6b	1E-07	0.3	Clic4	2E-26	0.3
Alkbh6	3E-04	0.3	Ccdc157	8E-04	0.3
Wdr77	3E-04	0.3	Serpinh1	5E-57	0.3
Cep85	6E-04	0.3	Gm50240	1E-03	0.3
Vps51	6E-03	0.3	Ippk	8E-02	0.3
Dmd	2E-23	0.3	B230217C12Rik	6E-03	0.3
Prorsd1	2E-13	0.3	Lyrm4	3E-08	0.3
C1qbp	5E-24	0.3	Vamp5	3E-16	0.3
Cisd1	1E-19	0.3	G6pc3	9E-06	0.3
Gne	5E-07	0.3	Usp38	9E-03	0.3
Pgrmc2	5E-20	0.3	Exosc1	1E-07	0.3
Cux1	9E-13	0.3	Coq3	5E-02	0.3
Crcp	6E-04	0.3	Snhg5	4E-06	0.3
Sp4	7E-04	0.3	Yae1d1	3E-07	0.3
1810021B22Rik	4E-03	0.3	Ccdc62	9E-05	0.3
Usp11	1E-07	0.3	Vwa8	2E-07	0.3
Eif3c	4E-53	0.3	Zscan26	2E-05	0.3

Fibroblast					
Control			*Notch2*$^{tm1.1Ecan}$		
Gene	*p*	FC	Gene	*p*	FC
Insig2	1E-04	0.3	Ints10	2E-04	0.4
Dab2	4E-09	0.3	Hoxa11os	2E-04	0.4
Entpd5	4E-03	0.3	Rfx3	7E-05	0.4
Cpd	2E-08	0.3	Isg15	3E-03	0.4
Vrk2	6E-04	0.3	Itga5	7E-10	0.4
Rdh10	4E-03	0.3	Zfp212	3E-03	0.4
Carns1	3E-03	0.3	Bud13	3E-02	0.4
Tmem185b	1E-02	0.3	Dele1	9E-05	0.4
Zbed3	2E-06	0.3	Fbxw5	4E-02	0.4
Vps13b	1E-16	0.3	Sap30bp	2E-09	0.4
Sh3rf1	6E-07	0.3	Pcyt2	3E-09	0.4
Lnpk	2E-06	0.3	Ncald	2E-03	0.4
Mpp7	1E-06	0.3	Clasrp	6E-04	0.4
Tdp2	2E-04	0.3	Snn	2E-01	0.4
Fosl2	3E-09	0.3	Phactr4	1E-08	0.4
Lrrc75a	3E-03	0.3	Map2k3	5E-10	0.4
Stk35	3E-05	0.3	Dgka	2E-03	0.4
C2cd5	5E-06	0.3	Dzip1l	2E-03	0.4
Gask1b	2E-04	0.3	Ints2	2E-06	0.4
Ptrh1	7E-03	0.3	Zfp703	7E-08	0.4
Sc5d	2E-07	0.3	Rbm43	2E-06	0.4
Cdkn1b	2E-12	0.3	Tti1	2E-04	0.4
1810055G02Rik	2E-03	0.3	Rell1	8E-04	0.4
Vat1	1E-14	0.3	Galnt4	1E-02	0.4
Man2a2	1E-03	0.3	Mapre1	7E-13	0.4
Mfn2	2E-07	0.3	Sfmbt1	4E-07	0.4
Sowahc	1E-03	0.3	Rpusd1	1E-03	0.4
Atp2b4	4E-03	0.3	Klhl18	2E-04	0.4
Amotl2	8E-16	0.3	Tmem237	4E-08	0.4
Nt5dc2	4E-05	0.3	Edrf1	2E-03	0.4
Adgre5	1E-03	0.3	Cdc42ep2	1E-06	0.4
Sf3a2	3E-02	0.3	Dhcr24	1E-07	0.4
Prelp	7E-11	0.3	Mme	2E-17	0.4
Thoc3	5E-06	0.3	Birc3	4E-02	0.4
Alg13	4E-05	0.3	Tbc1d1	1E-03	0.4
Zfp703	6E-07	0.3	Orc2	3E-05	0.4
Lnx2	2E-02	0.3	Atp11c	2E-07	0.4
Sestd1	2E-07	0.3	Nbn	1E-07	0.4
Sbno2	3E-04	0.3	Tspan9	3E-07	0.4
Aida	3E-06	0.3	Akap6	5E-04	0.4
Myo1c	3E-10	0.3	Arhgef11	4E-07	0.4
Bcar1	2E-04	0.3	Dcbld2	2E-11	0.4
9330159M07Rik	6E-03	0.3	Casp9	4E-02	0.4
Tshz3	2E-06	0.3	Atl2	1E-03	0.4
Lrrcc1	1E-07	0.3	Fhl2	1E-11	0.4
Cltb	2E-09	0.3	Aff3	7E-19	0.4
Pak3	3E-11	0.3	Arsb	6E-07	0.4
Tmem106c	2E-05	0.3	Upf3b	1E-09	0.4
Dhx9	1E-08	0.3	Ist1	4E-03	0.4
Chst12	1E-05	0.3	Ccdc61	4E-06	0.4
Tubgcp4	3E-04	0.3	Hes1	3E-18	0.4
Tubd1	3E-02	0.3	Ranbp1	5E-17	0.4
Rfc1	1E-05	0.3	Klc3	6E-07	0.4
Cep290	1E-04	0.3	Nup153	7E-10	0.4
Actr1a	4E-06	0.3	Smchd1	5E-13	0.4
Uba2	3E-05	0.3	Cln6	1E-04	0.4
Fancc	3E-08	0.3	Igsf8	4E-12	0.4
Gm44686	4E-03	0.3	Adcy3	1E-03	0.4
Paip2b	1E-03	0.3	Ranbp3	1E-04	0.4
Cpeb4	4E-07	0.3	Nudt18	7E-06	0.4
Fxyd5	2E-06	0.3	Jph2	5E-02	0.4
Nab1	1E-03	0.3	Snapc2	1E-03	0.4
Pld1	2E-03	0.3	Svil	3E-13	0.4
Dhrs13	2E-03	0.3	Zfp934	3E-05	0.4

Undefined					
Control			*Notch2*$^{tm1.1Ecan}$		
Gene	*p*	FC	Gene	*p*	FC
Cdon	4E-01	0.2	Mrpl27	7E-08	0.2
Mark1	3E-03	0.2	Fbln7	4E-01	0.2
Rrp15	4E-01	0.2	Gnasas1	3E-03	0.2
Bbip1	6E-01	0.2	Noc3l	4E-04	0.2
Brix1	7E-01	0.2	Polr2h	1E-05	0.2
Usp11	8E-01	0.2	Rpl35a	3E-26	0.2
Nfkb2	5E-03	0.2	Tfam	2E-04	0.2
Letm2	1E+00	0.2	Lancl1	4E-04	0.2
Zfp637	2E-01	0.2	Hspe1-rs1	6E-01	0.2
Trub2	6E-01	0.2	Sar1b	6E-01	0.2
Sf3b6	5E-03	0.2	Tmem161a	6E-04	0.2
Mrps28	6E-01	0.2	Bloc1s4	9E-07	0.2
Spout1	1E+00	0.2	Cbwd1	1E-03	0.2
Amd1	5E-02	0.2	Ube2v2	1E-05	0.2
Gm15417	6E-01	0.2	Rplp0	8E-38	0.2
Btaf1	2E-01	0.2	Gm19710	5E-02	0.2
Rmdn3	5E-02	0.2	Tmed2	2E-01	0.2
Arl14ep	3E-01	0.2	Tet3	6E-05	0.2
1190007I07Rik	2E-01	0.2	Rrs1	2E-01	0.2
Taf1d	2E-01	0.2	Cox20	1E-03	0.2
Fuom	8E-02	0.2	Taf5l	3E-02	0.2
Igf2r	9E-01	0.2	Cyb5d2	1E-01	0.2
3110040N11Rik	9E-01	0.2	2300009A05Rik	2E-01	0.2
Tcea1	9E-04	0.2	Pigx	3E-05	0.2
Gmpr	5E-01	0.2	Mtg2	4E-02	0.2
Sh3bp5l	8E-01	0.2	Yipf6	2E-03	0.2
Pigp	5E-02	0.2	Mpp6	6E-02	0.2
9630028H03Rik	7E-01	0.2	Cdc42se1	8E-04	0.2
Camsap2	2E-01	0.2	Rpl13a	4E-27	0.2
Dolk	9E-01	0.2	Abt1	1E-03	0.2
Abcd3	7E-01	0.2	Gm47071	1E-01	0.2
Mrpl3	2E-01	0.2	Mthfd1	6E-02	0.2
Plekhh2	5E-01	0.2	Sdhb	2E-06	0.2
Gm30054	2E-01	0.2	Erf	3E-03	0.2
Oscp1	1E-01	0.2	Ppie	5E-02	0.2
Taf15	4E-01	0.2	Polr3h	5E-06	0.2
Lsm5	6E-01	0.2	Sft2d3	9E-04	0.2
BC005561	1E+00	0.2	Hspb11	2E-05	0.2
Scrn3	6E-01	0.2	Daglb	2E-02	0.2
Arl2	2E-01	0.2	Clba1	3E-03	0.2
Marveld1	5E-02	0.2	Crnkl1	4E-13	0.2
Pdpr	1E-01	0.2	Alg5	1E-05	0.2
Irs2	9E-01	0.2	Cops7a	9E-06	0.2
Stard13	7E-05	0.2	Nbr1	7E-07	0.2
Higd1a	1E-10	0.2	Fmc1	2E-07	0.2
Hbs1l	9E-01	0.2	Snx25	5E-03	0.2
Phyhd1	3E-02	0.2	Mthfsl	3E-03	0.2
Pts	5E-01	0.2	Cpox	2E-02	0.2
Pnrc2	2E-02	0.2	Slc41a1	3E-02	0.2
Ccdc86	1E+00	0.2	Tpt1	6E-49	0.2
Slc25a17	6E-01	0.2	Pak1ip1	5E-03	0.2
Tm9sf2	4E-04	0.2	Sema3e	2E-01	0.2
Smad6	1E-01	0.2	Rps6	5E-24	0.2
Champ1	3E-02	0.2	Ndufs1	3E-09	0.2
Abca5	5E-01	0.2	Gcsh	6E-04	0.2
Mtg2	7E-01	0.2	Rgs7bp	3E-02	0.2
Pacsin3	9E-01	0.2	Hspb8	2E-03	0.2
Wdr53	6E-02	0.2	Sec22b	1E-06	0.2
Acox1	6E-02	0.2	Commd6	2E-04	0.2
Cd109	9E-01	0.2	Polr2k	2E-04	0.2
Qsox2	1E+00	0.2	Tsacc	4E-01	0.2
Ctsd	1E-12	0.2	Mettl26	1E-03	0.2
Stk11	6E-01	0.2	Etl4	4E-02	0.2
Wfs1	7E-01	0.2	Txnrd2	2E-04	0.2

Articular/Synovial Fibroblast					
Control			*Notch2*$^{tm1.1Ecan}$		
Gene	*p*	FC	Gene	*p*	FC
Ubtd2	5E-12	0.5	Nr2f2	4E-06	0.5
Cstf1	7E-03	0.5	Heca	4E-05	0.5
Mef2d	2E-13	0.5	Cers6	3E-07	0.5
Arpc1b	8E-28	0.5	Antxr2	6E-12	0.5
Pon3	8E-08	0.5	Dpyd	2E-03	0.4
P2rx4	7E-14	0.5	Ddx58	6E-03	0.4
Pip4p1	4E-08	0.5	Hoxd9	4E-03	0.4
Arhgef10	2E-10	0.5	Ocel1	2E-01	0.4
Eng	7E-07	0.5	Mterf2	5E-03	0.4
Hk1	1E-10	0.5	Spg11	8E-04	0.4
Arntl2	7E-03	0.5	Hpcal1	4E-02	0.4
Baz1a	8E-06	0.5	Atrnl1	1E-08	0.4
Rassf5	2E-01	0.5	Ttpal	4E-01	0.4
Cpped1	5E-09	0.5	Mxd4	3E-18	0.4
Gm44899	4E-02	0.5	Fam214a	8E-05	0.4
Cog7	2E-05	0.5	Rit1	2E-03	0.4
Tuba1a	1E-34	0.5	Lurap1l	1E-13	0.4
Esrra	2E-02	0.5	Trpc1	6E-03	0.4
Traf5	2E-03	0.5	Klhl26	7E-04	0.4
Tead3	1E-03	0.5	Atp2b4	3E-03	0.4
Sms	2E-06	0.5	Sept11	6E-22	0.4
Igf2bp1	9E-04	0.5	Rtn4	9E-26	0.4
Extl3	5E-06	0.5	Dlg4	2E-05	0.4
Agtrap	7E-05	0.5	Plin2	1E-09	0.4
Chrdl1	2E-06	0.5	Mfn2	1E-04	0.4
Cdr2l	1E-02	0.5	Zhx3	4E-06	0.4
Cxxc5	7E-12	0.5	Klf10	3E-03	0.4
Ier3	6E-14	0.5	1700037H04Rik	3E-01	0.4
Tank	9E-11	0.5	Fbxo6	2E-05	0.4
Tomm40l	3E-02	0.5	Mdga1	2E-03	0.4
Cst6	7E-07	0.5	Vps13d	1E-10	0.4
Cnnm2	9E-06	0.5	Matn2	1E-05	0.4
Cstb	3E-38	0.5	Fmnl2	2E-10	0.4
Psmf1	7E-03	0.5	Ctsb	2E-27	0.4
Wdr7	7E-13	0.5	F8a	5E-02	0.4
Naaladl2	1E-27	0.5	Mxra7	3E-18	0.4
Ighmbp2	5E-02	0.5	Stat1	1E-04	0.4
Cyth3	2E-11	0.5	Antxr1	3E-21	0.4
Vps13c	1E-13	0.5	Capn2	6E-12	0.4
Nectin3	7E-08	0.5	H6pd	2E-06	0.4
Rtl8a	3E-08	0.5	Tank	6E-04	0.4
Dusp8	8E-04	0.5	Dclk1	1E-15	0.4
1700025G04Rik	1E-08	0.5	Dock10	3E-06	0.4
Ddx58	6E-06	0.5	Kremen1	3E-04	0.4
Il1r1	4E-10	0.5	Ehmt2	3E-08	0.4
Lpin1	3E-06	0.5	Gm16201	3E-03	0.4
Chfr	2E-10	0.5	Sms	6E-03	0.4
Tirap	2E-03	0.5	Map3k2	5E-08	0.4
Plcl2	3E-03	0.5	Mapk3	3E-09	0.4
BC029722	2E-07	0.5	Arl6ip5	1E-10	0.4
Inpp4b	6E-01	0.5	Mdk	8E-03	0.4
Tgfa	2E-01	0.5	Sat2	7E-04	0.4
Flcn	2E-04	0.5	Mcur1	2E-04	0.4
Usp24	4E-14	0.5	Poglut3	8E-08	0.4
Stk38	1E-11	0.5	Pxmp4	7E-03	0.4
Matn2	1E-05	0.5	Rapgef1	4E-04	0.4
Smarcd2	5E-06	0.5	Fam107b	2E-01	0.4
Trim12a	4E-04	0.5	Yju2	5E-01	0.4
Acsl5	2E-06	0.5	Cstb	4E-24	0.4
Hspb8	1E-09	0.5	Tpp1	1E-14	0.4
Mamld1	3E-06	0.5	Snx2	4E-12	0.4
Atp7a	2E-07	0.5	Pvr	2E-02	0.4
Kremen1	3E-07	0.5	Irak2	1E-01	0.4
Sergef	2E-03	0.5	Cib2	1E-04	0.4

Limb Mesenchyme					
Control			*Notch2* $^{tm1.1Ecan}$		
Gene	*p*	FC	Gene	*p*	FC
Vasp	2E-05	0.2	2900026A02Rik	8E-08	0.2
Kdelr3	2E-11	0.2	Rrp36	1E-05	0.2
Nfkbiz	2E-06	0.2	Fam219a	2E-06	0.2
F420014N23Rik	9E-02	0.2	D830050J10Rik	2E-02	0.2
Zfp593	9E-04	0.2	Thoc6	2E-06	0.2
Trerf1	1E-07	0.2	Kdelr1	3E-29	0.2
Tcea3	1E-19	0.2	B4galt6	4E-05	0.2
Mypop	6E-03	0.2	Acap2	3E-13	0.2
H2-T22	2E-06	0.2	Add1	8E-20	0.2
B3galnt1	7E-05	0.2	Myl6	8E-34	0.2
Smg9	2E-02	0.2	Gramd1a	2E-05	0.2
Lima1	3E-18	0.2	Dgkz	5E-04	0.2
Hes6	4E-03	0.2	Zfp738	1E-04	0.2
Slit3	1E-24	0.2	Arhgef9	2E-02	0.2
Bdnf	2E-02	0.2	Fancf	9E-04	0.2
Zfp9	3E-07	0.2	Rtn4	1E-39	0.2
Hdac3	8E-07	0.2	Dcun1d3	4E-07	0.2
Elf4	9E-07	0.2	Zmat3	8E-12	0.2
Mettl22	3E-03	0.2	Stox2	4E-07	0.2
2410131K14Rik	9E-03	0.2	Map6	2E-12	0.2
Mios	7E-04	0.2	Arid5b	3E-17	0.2
Rft1	4E-05	0.2	Rps26	1E-62	0.2
B3gnt9	9E-06	0.2	Pik3c2a	4E-06	0.2
Tubb5	6E-59	0.2	Bcl7c	1E-18	0.2
Gjc1	2E-07	0.2	Anxa6	1E-16	0.2
Rspry1	6E-07	0.2	Czib	5E-11	0.2
Abhd11	1E-04	0.2	Ptpn12	2E-08	0.2
Ttc21b	7E-04	0.2	Gsta4	5E-32	0.2
Spidr	3E-03	0.2	Zpr1	1E-08	0.2
Actb	4E-34	0.2	Cdk5	4E-08	0.2
Mfsd5	1E-06	0.2	Elovl5	1E-06	0.2
Mxd4	2E-23	0.2	Zfp9	4E-07	0.2
Grn	2E-33	0.2	Cnih1	1E-17	0.2
Lpar1	3E-15	0.2	Apex1	1E-12	0.2
Zswim6	5E-05	0.2	Arhgdia	3E-30	0.2
Ppox	4E-02	0.2	Cep170b	4E-05	0.2
Rgs2	2E-02	0.2	Gtdc1	2E-08	0.2
Myl6	2E-24	0.2	Emp3	3E-31	0.2
Copz1	2E-11	0.2	Iqsec2	1E-07	0.2
Sars	5E-27	0.2	Gcat	9E-06	0.2
Ncln	8E-04	0.2	Pelo	3E-08	0.2
Nmrk1	3E-04	0.2	Cant1	1E-05	0.2
Pla2g4a	5E-05	0.2	Sharpin	9E-06	0.2
Fkbpl	1E-01	0.2	Pstk	3E-08	0.2
Slc29a3	1E-03	0.2	Umps	2E-04	0.2
Il17rc	2E-07	0.2	Gatb	1E-03	0.2
Tor2a	2E-04	0.2	Fam117b	4E-07	0.2
Twf2	5E-05	0.2	Trpm4	3E-03	0.2
Arf2	1E-01	0.2	Irf2bpl	8E-10	0.2
Pigs	1E-04	0.2	Cfl1	2E-41	0.2
Abca9	7E-03	0.2	Ap5m1	2E-02	0.2
Cpt1a	3E-03	0.2	Abhd4	2E-07	0.2
Ap1s1	2E-11	0.2	Eif4e2	2E-22	0.2
Eif2d	8E-03	0.2	Ank2	2E-16	0.2
Stambpl1	1E-07	0.2	Plekha1	4E-04	0.2
Pik3r2	8E-08	0.2	Vgll3	2E-22	0.2
Pik3r1	6E-10	0.2	Slc2a8	4E-04	0.2
Tmem258	5E-26	0.2	Fbxl7	6E-22	0.2
Dpagt1	3E-02	0.2	Atxn7l3	2E-08	0.2
Gtf2e1	4E-03	0.2	Ddit4	3E-07	0.2
Lamc1	1E-07	0.2	Zfas1	2E-18	0.2
Tep1	8E-04	0.2	Cndp2	1E-03	0.2
Ppp2r1b	7E-03	0.2	1700123O20Rik	5E-09	0.2
Eln	8E-09	0.2	Dbnl	6E-12	0.2

Chondrogenic					
Control			*Notch2* $^{tm1.1Ecan}$		
Gene	*p*	FC	Gene	*p*	FC
4933434E20Rik	2E-09	0.3	Ice2	5E-03	0.3
Dele1	1E-04	0.3	Kctd3	7E-03	0.3
Grsf1	2E-06	0.3	Imp3	2E-29	0.3
Ndufa12	1E-24	0.3	Micos13	8E-30	0.3
Igdcc4	6E-06	0.3	Cmtm8	2E-04	0.3
Tbx5	6E-02	0.3	Nop10	2E-32	0.3
Kif6	6E-01	0.3	Ddx20	5E-02	0.3
Optn	5E-04	0.3	Tspan3	7E-39	0.3
Exosc5	9E-10	0.3	Hs2st1	5E-10	0.3
Timm8b	2E-27	0.3	Oxnad1	2E-01	0.3
Adcy2	2E-10	0.3	Crot	6E-06	0.3
Exosc1	2E-06	0.3	Chd1l	3E-01	0.3
Tmed10	7E-53	0.3	Traf3ip1	2E-01	0.3
Hoxa11	8E-07	0.3	Gm12905	4E-01	0.3
Yars	5E-09	0.3	Vdac3	2E-26	0.3
Nop10	4E-36	0.3	Lonrf1	4E-02	0.3
Dusp18	3E-02	0.3	Klf9	4E-10	0.3
Txndc12	5E-13	0.3	Pgm3	9E-07	0.3
Klhl11	3E-02	0.3	Nop14	1E-10	0.3
Tnfaip8	6E-07	0.3	Smim26	1E-09	0.3
1700016P03Rik	1E-02	0.3	Hspb6	2E-05	0.3
Zcchc4	4E-01	0.3	Glce	3E-06	0.3
Atp6v1a	4E-18	0.3	Adpgk	5E-03	0.3
Clpb	7E-05	0.3	Apex2	2E-06	0.3
Med22	3E-02	0.3	Fam229b	1E-05	0.3
Rpp14	9E-05	0.3	Gnl3	1E-17	0.3
Rida	1E-08	0.3	Zfp839	6E-02	0.3
Rsph3b	2E-02	0.3	Pik3cb	2E-03	0.3
Dap	5E-31	0.3	B4galt7	1E-06	0.3
Rmnd5a	5E-09	0.3	Surf6	3E-04	0.3
Nle1	1E-01	0.3	Gm26782	1E-06	0.3
Tmx4	7E-03	0.3	Upf3b	3E-06	0.3
Jade1	4E-11	0.3	Akap8l	3E-08	0.3
Gstk1	4E-04	0.3	Igsf3	1E-02	0.3
Zrsr1	1E-02	0.3	1110004F10Rik	3E-29	0.3
Nol8	4E-05	0.3	Tra2b	5E-19	0.3
Mccc1	7E-04	0.3	Qtrt2	1E-01	0.3
Dad1	6E-48	0.3	Atg13	2E-04	0.3
Gm15417	7E-08	0.3	Ostc	1E-35	0.3
Daam2	7E-07	0.3	Orc2	2E-04	0.3
Surf2	5E-08	0.3	Ttc5	2E-02	0.3
2310039H08Rik	1E-10	0.3	Rpl13a	1E-66	0.3
Emilin1	1E-11	0.3	Pno1	2E-08	0.3
Tspan3	7E-35	0.3	Daam2	3E-07	0.3
Bcl9	8E-09	0.3	Chchd4	1E-02	0.3
Mdn1	3E-05	0.3	Zfp248	7E-01	0.3
Desi1	1E-07	0.3	Gpatch4	2E-03	0.3
Gm17106	3E-03	0.3	Galnt11	7E-05	0.3
Vdac3	3E-26	0.3	Sfxn1	2E-13	0.3
Crot	8E-07	0.3	Lcn2	2E-05	0.3
Gm19409	4E-02	0.3	Nhlrc3	1E-01	0.3
Nr4a3	6E-04	0.3	Gng7	9E-07	0.3
Zyg11b	3E-10	0.3	Vps37a	3E-07	0.3
Lins1	3E-03	0.3	Cab39l	7E-09	0.3
Tfb1m	3E-02	0.3	Cd9	4E-21	0.3
Gm43672	3E-03	0.3	Ttc7	4E-03	0.3
Fra10ac1	2E-07	0.3	Gtf2ird2	3E-03	0.3
Tmem251	1E-05	0.3	Cep44	2E-02	0.3
Hbegf	3E-11	0.3	Leo1	1E-05	0.3
Id3	2E-22	0.3	Eif1	2E-74	0.3
Cyth1	1E-04	0.3	Pck2	3E-13	0.3
Spg20	6E-08	0.3	Pnpla7	1E-02	0.3
Alkbh1	2E-11	0.3	Ppm1b	2E-06	0.3
Ccnq	7E-06	0.3	Desi1	6E-05	0.3

Fibroblast					
Control			*Notch2* $^{tm1.1Ecan}$		
Gene	*p*	FC	Gene	*p*	FC
Zfp995	8E-03	0.3	Osbpl10	9E-04	0.4
Nrf1	1E-04	0.3	Ints7	5E-07	0.4
Zfp974	2E-02	0.3	Ddx58	2E-04	0.4
Tmem97	6E-06	0.3	Il20rb	2E-03	0.4
Acsl3	7E-04	0.3	Rbm41	2E-04	0.4
Schip1	1E-04	0.3	Ggt7	1E-05	0.4
Tmem109	2E-05	0.3	Snrpc	8E-14	0.4
Nap1l1	3E-14	0.3	Prpf38b	5E-12	0.4
Ezr	2E-05	0.3	Wdr91	4E-03	0.4
Ppp2r5d	5E-03	0.3	Pank2	4E-09	0.4
Dnajc10	1E-11	0.3	Zfp729b	5E-03	0.4
Itga5	2E-05	0.3	Efr3b	1E-04	0.4
Ipo5	5E-07	0.3	Tlr4	3E-04	0.4
Mettl27	5E-02	0.3	Dvl2	4E-03	0.4
Rnf24	6E-05	0.3	Capg	1E-22	0.4
Clp1	1E-01	0.3	Hsf1	1E-04	0.4
Uvrag	2E-14	0.3	Adgre5	1E-03	0.4
Sart3	4E-05	0.3	Actl6a	3E-08	0.4
Ptpn1	2E-12	0.3	Tsen34	5E-15	0.4
Rab11fip5	3E-04	0.3	Glis2	5E-12	0.4
Sgk1	1E-07	0.3	Rel	6E-06	0.4
Lmbr1	5E-04	0.3	Pknox1	2E-04	0.4
Zcchc24	8E-09	0.3	L3mbtl3	3E-09	0.4
Rnf26	3E-03	0.3	Hipk2	3E-12	0.4
Pml	3E-02	0.3	Gas8	6E-07	0.4
Mme	3E-13	0.3	Sms	2E-06	0.4
Polr3f	2E-02	0.3	Srsf1	1E-11	0.4
Pgk1	7E-15	0.3	Fam110b	2E-15	0.4
M6pr	2E-08	0.3	Usp42	1E-03	0.4
Zfp664	5E-06	0.3	Dbr1	2E-03	0.4
Prcp	2E-03	0.3	Nsmce1	8E-12	0.4
Psmd8	6E-23	0.3	Kbtbd2	1E-02	0.4
B4gat1	7E-06	0.3	Ppm1g	6E-11	0.4
Mettl4	4E-02	0.3	Acss2	8E-04	0.4
Itm2a	4E-24	0.3	Tmem209	1E-04	0.4
Ncbp3	7E-03	0.3	Gm45669	3E-02	0.4
Lysmd4	5E-05	0.3	Gpatch1	2E-01	0.4
Ryk	5E-17	0.3	Tmem106c	1E-07	0.4
Mvd	1E-02	0.3	Map6	5E-09	0.4
Elof1	3E-07	0.3	Ccdc57	4E-06	0.4
Ssna1	8E-09	0.3	Hdac9	8E-04	0.4
Ror1	1E-10	0.3	Ahnak2	3E-21	0.4
Xrcc1	3E-03	0.3	Hnrnpul1	2E-13	0.4
Pot1b	2E-03	0.3	Pphln1	1E-05	0.4
Rpia	8E-03	0.3	Mapre2	3E-13	0.4
Tmem154	3E-02	0.3	Wrn	7E-04	0.4
Rnaseh2b	8E-03	0.3	E2f6	2E-05	0.4
Senp1	1E-04	0.3	Patl1	3E-06	0.4
Ahr	1E-04	0.3	Mrpl18	2E-14	0.4
Plxnb2	6E-10	0.3	Ednra	3E-03	0.4
Timm50	2E-05	0.3	Rftn2	3E-06	0.4
Tmod3	3E-09	0.3	Sept11	9E-31	0.4
Kdm6a	3E-05	0.3	Mrps27	8E-03	0.4
Lyst	1E-08	0.3	Fnbp1l	2E-04	0.4
Kif6	4E-02	0.3	Recql	5E-03	0.4
Syne3	4E-04	0.3	Nmi	2E-05	0.4
Cstf2	4E-02	0.3	Srebf2	5E-10	0.4
Katnb1	2E-02	0.3	Zkscan5	4E-04	0.4
Gpnmb	2E-10	0.3	Zfp61	8E-03	0.4
Chd1l	8E-04	0.3	Ccdc130	4E-02	0.4
Tbl2	4E-03	0.3	Rarres2	3E-09	0.4
Zbtb46	1E-01	0.3	Nrf1	7E-13	0.4
Map2	4E-09	0.3	Ing1	1E-08	0.4
Lrch2	2E-01	0.3	Mettl25	8E-04	0.4

Undefined					
Control			*Notch2* $^{tm1.1Ecan}$		
Gene	*p*	FC	Gene	*p*	FC
Map3k8	4E-02	0.2	Acaca	4E-02	0.2
Pnpla6	2E-01	0.2	Zcchc10	1E-03	0.2
Gm1976	5E-01	0.2	Plekhf2	4E-04	0.2
Tecpr2	7E-01	0.2	Prpf8	3E-07	0.2
Rbks	1E+00	0.2	Glrx5	1E-08	0.2
Sacs	1E-03	0.2	Fam219b	3E-01	0.2
Dph6	9E-01	0.2	Prdm16	4E-01	0.2
Plb1	4E-01	0.2	Parl	5E-06	0.2
Erlin1	6E-02	0.2	Srp14	4E-01	0.2
Cobll1	7E-01	0.2	Nol8	1E-02	0.2
Snx15	1E+00	0.2	Card19	1E-01	0.2
Herc1	4E-01	0.2	Ift80	7E-05	0.2
Tef	5E-01	0.2	Mrps18b	3E-04	0.2
Ddx18	5E-01	0.2	Dnajb2	2E-05	0.2
Znrd1	1E-01	0.2	Btg3	2E-01	0.2
Rabac1	2E-06	0.2	Slc1a4	3E-03	0.2
Chmp7	8E-04	0.2	Pdss2	1E-03	0.2
Mrps26	4E-02	0.2	Rpl21	1E-30	0.2
Mtmr3	7E-02	0.2	Taf6	3E-06	0.2
Atr	1E-01	0.2	Taf4b	5E-01	0.2
Farp1	5E-01	0.2	Cox7a2	2E-01	0.2
Gm34455	4E-01	0.2	Sdf2	4E-04	0.2
Tiprl	6E-01	0.2	Tusc1	3E-08	0.2
Tubgcp3	9E-01	0.2	Cox7c	5E-15	0.2
Polr2g	6E-01	0.2	Igf2r	3E-02	0.2
BC031181	2E-02	0.2	Tm2d3	2E-02	0.2
4933434E20Rik	5E-01	0.2	Ing3	6E-03	0.2
Homer1	9E-01	0.2	Nck2	2E-06	0.2
Id3	4E-06	0.2	Gnl3l	2E-02	0.2
5330438D12Rik	8E-01	0.2	Sugct	7E-04	0.2
Clba1	3E-01	0.2	Rwdd4a	1E-03	0.2
Dnal4	2E-01	0.2	Surf1	1E-03	0.2
Skil	6E-01	0.2	Srm	1E-04	0.2
Pogz	7E-01	0.2	F8	7E-02	0.2
Setd7	6E-01	0.2	Ndufs5	7E-01	0.2
Pdzd2	1E+00	0.2	Ufl1	7E-03	0.2
Wdr12	2E-01	0.2	Rbx1	4E-01	0.2
BC049715	2E-02	0.2	Acyp1	2E-02	0.2
Mlh3	2E-01	0.2	Zfp821	3E-04	0.2
Unc50	2E-01	0.2	Ubb	4E-05	0.2
Adat1	1E-01	0.2	Smyd2	3E-10	0.2
Mcat	1E-01	0.2	Nars2	1E-02	0.2
Alg3	7E-01	0.2	Thap3	7E-07	0.2
4932438A13Rik	6E-01	0.2	Mrpl23	8E-06	0.2
Cox11	8E-01	0.2	Anp32b	1E+00	0.2
Fam162a	1E-01	0.2	Gm26887	2E-03	0.2
Tipin	2E-01	0.2	Trmt61b	4E-01	0.2
Spcs2	3E-04	0.2	Rps27	1E-24	0.2
Snhg8	1E-01	0.2	Magoh	1E-03	0.2
Bscl2	3E-01	0.2	Prnp	5E-02	0.2
Secisbp2	2E-01	0.2	Vps28	5E-04	0.2
Aplp2	2E-07	0.2	Txnrd3	2E-04	0.2
Cep95	7E-02	0.2	Ddrgk1	5E-02	0.2
Dnajb9	2E-01	0.2	Os9	2E-04	0.2
Lyar	7E-01	0.2	Eif4e	5E-05	0.2
Slc25a39	5E-01	0.2	Zbtb25	2E-05	0.2
Selenoo	1E+00	0.2	Nudt1	2E-04	0.2
Rps27a	2E-39	0.2	Hmgn5	1E-02	0.2
Crtc1	4E-02	0.2	Fuca2	7E-05	0.2
Ttll12	4E-02	0.2	Dad1	9E-03	0.2
Arhgef1	1E-01	0.2	Sdf4	1E-01	0.2
Desi1	7E-01	0.2	Nol12	4E-06	0.2
Elmsan1	4E-01	0.2	Gas2l1	9E-12	0.2
Ccdc138	7E-01	0.2	Mrps6	2E-02	0.2

Articular/Synovial Fibroblast					
Control			*Notch2* $^{tm1.1Ecan}$		
Gene	*p*	FC	Gene	*p*	FC
Gm9801	5E-03	0.5	Pcyox1	9E-06	0.4
Cers6	5E-12	0.5	Myl12b	3E-16	0.4
Ldlrad4	8E-22	0.5	Wbp1	4E-08	0.4
Fcgrt	3E-29	0.5	Arhgap28	5E-10	0.4
Cdc14b	4E-09	0.5	Smarcd2	4E-05	0.4
Plekho1	4E-06	0.5	Wdr7	3E-09	0.4
Pkn1	2E-07	0.5	Sri	3E-18	0.4
Elovl5	2E-06	0.5	Dclre1a	3E-02	0.4
Ptpn3	6E-03	0.5	Igf1r	4E-17	0.4
Lnx2	2E-02	0.5	Ctsl	1E-35	0.4
Lbh	2E-06	0.5	Cyb5r4	2E-05	0.4
Zfp263	1E-04	0.5	Fam20c	2E-04	0.4
Zc3h3	5E-03	0.5	Twsg1	2E-05	0.4
Jpt1	7E-18	0.5	Hexa	9E-20	0.4
Fam214b	3E-04	0.5	Sos2	3E-06	0.4
Dclre1a	4E-04	0.5	Spry2	2E-05	0.4
Wipf2	4E-05	0.5	Pgpep1	5E-05	0.4
Ttc39c	2E-01	0.5	Mapre2	2E-15	0.4
Rnd2	3E-04	0.5	Myo1c	2E-08	0.4
Hsd17b11	8E-06	0.5	Tmem50b	8E-03	0.4
Gcfc2	1E-01	0.5	Sorbs3	2E-03	0.4
Vasn	2E-12	0.5	Baz2a	4E-05	0.4
Dusp1	9E-21	0.5	Rcan3	2E-01	0.4
Saal1	3E-03	0.5	Tnfrsf1a	5E-07	0.4
Col8a1	3E-05	0.5	Zc3h6	2E-02	0.4
Inpp5a	1E-05	0.5	Rap1b	2E-19	0.4
Spry2	3E-05	0.5	Fam172a	7E-16	0.4
Phlda1	5E-01	0.5	Bmt2	1E-07	0.4
Hs1bp3	3E-05	0.5	Dusp16	5E-04	0.4
Stk24	6E-12	0.5	Eif4ebp2	1E-06	0.4
Gadd45g	9E-05	0.5	Gusb	2E-06	0.4
Ankrd27	7E-06	0.5	Nipsnap3b	1E-01	0.4
Pik3r3	9E-07	0.5	Pxdn	2E-10	0.4
Pip5k1b	5E-03	0.5	Rtl8b	8E-05	0.4
Mtmr11	5E-03	0.5	Cnpy4	6E-03	0.4
Cotl1	3E-03	0.5	Slc38a6	5E-03	0.4
Nr6a1os	1E-01	0.5	Tmem45a	2E-07	0.4
Gns	1E-16	0.5	Cpeb1	2E-09	0.4
Flot1	1E-12	0.5	Fbxo21	3E-02	0.4
Lactb2	2E-09	0.5	Spcs3	1E-04	0.4
Slc25a44	3E-04	0.5	Sh3d19	2E-11	0.4
Prkca	1E-22	0.5	Rhoj	1E-12	0.4
Ttll1	2E-03	0.5	Fcgrt	2E-20	0.4
Cd164	2E-18	0.5	Zfp438	2E-02	0.4
Samd4b	1E-09	0.5	2310015A10Rik	3E-01	0.4
3300002I08Rik	3E-03	0.5	Purg	2E-03	0.4
Supt7l	3E-02	0.5	Sun1	1E-04	0.4
Ccdc171	2E-04	0.5	Rab23	3E-03	0.4
Tapbp	8E-13	0.5	Slc44a1	4E-06	0.4
Pisd	4E-03	0.5	Sptlc2	2E-06	0.4
Lifr	1E-03	0.5	Pnrc1	5E-16	0.4
Spcs3	1E-05	0.4	Lnx2	8E-02	0.4
Ptn	9E-08	0.4	Dbi	3E-32	0.4
Plp2	4E-10	0.4	Ctns	5E-04	0.4
2900089D17Rik	2E-02	0.4	Crtc3	6E-10	0.4
Flot2	7E-09	0.4	Irf2	1E-10	0.4
Btd	4E-06	0.4	Tmem192	1E-06	0.4
Tns2	1E-07	0.4	Rab3gap2	6E-03	0.4
Tmem41a	1E-02	0.4	Wbp11	4E-06	0.4
Mapk13	7E-02	0.4	Herc6	2E-02	0.4
Wdtc1	2E-04	0.4	Ctsz	3E-22	0.4
Glt8d2	1E-05	0.4	Fndc4	4E-03	0.4
Echdc3	4E-02	0.4	Nudt16	5E-01	0.4
Sri	3E-21	0.4	Tada2b	1E+00	0.4

Limb Mesenchyme						Chondrogenic						Fibroblast						Undefined						Articular/Synovial Fibroblast					
Control			$Notch2^{tm1.1Ecan}$			Control			$Notch2^{tm1.1Ecan}$			Control			$Notch2^{tm1.1Ecan}$			Control			$Notch2^{tm1.1Ecan}$			Control			$Notch2^{tm1.1Ecan}$		
Gene	*p*	FC	Gene	*p*	FC	Gene	*p*	FC	Gene	*p*	FC	Gene	*p*	FC	Gene	*p*	FC	Gene	*p*	FC	Gene	*p*	FC	Gene	*p*	FC	Gene	*p*	FC
Wdr81	2E-02	0.2	Ppp1r12a	6E-19	0.2	Shmt2	8E-06	0.3	Tfeb	8E-02	0.3	1810037I17Rik	2E-14	0.3	Pmepa1	8E-14	0.4	Sptssa	7E-01	0.2	Tceal	4E-01	0.2	Tbl3	6E-03	0.4	Stk24	2E-07	0.4
Syngr2	3E-08	0.2	Gm48742	1E-05	0.2	Aopep	2E-48	0.3	Tm9sf4	2E-15	0.3	Ddah2	2E-10	0.3	Cep290	1E-08	0.4	Cox17	4E-02	0.2	BC029722	3E-06	0.2	Mex3d	6E-03	0.4	Aldh3b1	5E-04	0.4
Dffa	2E-01	0.2	Zdhhc13	2E-02	0.2	Zfp672	1E-04	0.3	E230016M11Rik	1E-01	0.3	Serp2	5E-03	0.3	Aste1	3E-01	0.4	Uri1	9E-02	0.2	Trappc6a	6E-04	0.2	Dock5	1E-05	0.4	Slk	5E-08	0.4
Ppp1r14b	4E-48	0.2	Sap30l	3E-13	0.2	Pde3b	2E-07	0.3	Pfkm	7E-04	0.3	Wdfy1	3E-04	0.3	Pde4b	1E-09	0.4	Myg1	2E-01	0.2	Fastk	3E-06	0.2	Cacul1	1E-07	0.4	Tns2	4E-04	0.4
Vmp1	7E-20	0.2	Mvb12b	4E-09	0.2	Ccdc137	3E-02	0.3	Snrpn	6E-03	0.3	Rpusd1	5E-02	0.3	Gask1b	1E-04	0.4	Fem1c	5E-01	0.2	Drg2	7E-05	0.2	Clip3	2E-08	0.4	Cavin1	1E-18	0.4
Klhl29	2E-10	0.2	Ftl1-ps1	7E-08	0.2	Upf3b	2E-07	0.3	Abcb7	3E-03	0.3	Slc52a2	4E-02	0.3	Arid5b	9E-22	0.3	Ift57	7E-01	0.2	Pi4k2a	1E-05	0.2	Dubr	6E-04	0.4	Lrp3	2E-02	0.4
Scamp2	1E-05	0.2	Mpg	8E-10	0.2	Orc2	5E-05	0.3	Ncl	3E-29	0.3	Huwe1	2E-13	0.3	Tmem183a	4E-05	0.3	Rpl13a	2E-33	0.2	Skap2	2E-03	0.2	Inafm2	3E-02	0.4	Fam53c	1E-02	0.4
Ube3b	1E-03	0.2	Dram2	4E-05	0.2	Tm9sf4	1E-14	0.3	Srp14	2E-38	0.3	Nudt1	2E-03	0.3	Baz1b	5E-12	0.3	Bloc1s6	9E-01	0.2	Ywhaq	1E-03	0.2	Exoc1	3E-02	0.4	Slc35f5	4E-06	0.4
Rbfa	1E-05	0.2	Ttll12	2E-05	0.2	B830012L14Rik	3E-05	0.3	Mettl1	1E-11	0.3	Fntb	1E-04	0.3	Lmcd1	8E-07	0.3	Syngap1	2E-01	0.2	Zfp143	2E-02	0.2	Patl1	9E-06	0.4	Ank2	6E-14	0.4
Olfml2b	3E-13	0.2	Coq6	2E-04	0.2	Sft2d3	3E-06	0.3	Efna1	4E-02	0.3	Zdhhc14	2E-01	0.3	Klhl5	4E-04	0.3	Usf3	9E-01	0.2	2510002D24Rik	7E-02	0.2	Tmem131l	2E-02	0.4	LTO1	1E-01	0.4
Sccpdh	1E-03	0.2	Commd9	1E-09	0.2	Lrrc28	1E-04	0.3	Ptp4a2	2E-32	0.3	Runx1	2E-12	0.3	Actr1a	3E-10	0.3	Nmnat3	5E-01	0.2	Tmem141	2E-05	0.2	Rhoq	3E-21	0.4	Abcc5	4E-10	0.4
Fzd1	7E-05	0.2	Col6a2	4E-24	0.2	Ppp1r35	4E-04	0.3	Prkca	4E-07	0.3	Mtmr9	7E-04	0.3	Kctd18	2E-02	0.3	Ppp5c	7E-03	0.2	Mrpl57	1E-02	0.2	Evi5	1E-13	0.4	Dusp1	4E-11	0.4
Gba	4E-05	0.2	Hacd4	2E-12	0.2	Unc50	9E-14	0.3	Ccl25	1E-02	0.3	Tgfb1	4E-06	0.3	Grik5	6E-03	0.3	Bpgm	3E-01	0.2	Yae1d1	1E-03	0.2	Aspn	2E-11	0.4	Ptbp3	7E-09	0.4
H2-DMa	4E-02	0.2	Snap29	3E-06	0.2	Ppib	1E-66	0.3	Zfp329	5E-05	0.3	Tmem245	3E-09	0.3	Rassf2	5E-05	0.3	Canx	3E-06	0.2	Mrpl2	5E-05	0.2	Sumf1	1E-06	0.4	Dlg3	3E-01	0.4
Hscb	1E-03	0.2	Sept11	4E-30	0.2	Tprg	5E-04	0.3	Cisd1	2E-15	0.3	Sh3d19	3E-13	0.3	Prpf3	1E-05	0.3	Slc25a25	9E-01	0.2	Nek8	5E-03	0.2	Sept8	3E-08	0.4	9330159M07Rik	4E-02	0.4
Ppa2	2E-05	0.2	Tmem205	6E-14	0.2	Mettl5	2E-05	0.3	Foxc2	3E-02	0.3	Lcorl	1E-05	0.3	Ankrd28	3E-09	0.3	Agfg1	3E-01	0.2	Tmsb15b2	5E-02	0.2	Stradb	5E-02	0.4	Zfp655	9E-03	0.4
Usp45	1E-03	0.2	Smim1	7E-10	0.2	Kif21a	2E-08	0.3	Zfp777	1E-01	0.3	Mxra7	7E-18	0.3	Bbs9	7E-04	0.3	Timm21	2E-01	0.2	Pigk	4E-06	0.2	Il6st	2E-27	0.4	Zc2hc1a	1E-06	0.4
Srrd	1E-01	0.2	Chpf	5E-12	0.2	Igsf3	2E-01	0.3	Rps8	2E-87	0.3	Nudc	2E-10	0.3	Fut11	2E-07	0.3	CAAA01118383.1	3E-01	0.2	2310009A05Rik	7E-06	0.2	Psen2	3E-06	0.4	A230057D06Rik	6E-04	0.4
Man1a	3E-07	0.2	Ppcs	2E-04	0.2	Anp32b	8E-41	0.3	Scamp5	7E-03	0.3	Bcar3	6E-06	0.3	Lamc1	2E-12	0.3	Ap4s1	4E-01	0.2	Ppid	7E-05	0.2	4930522L14Rik	5E-02	0.4	Atp6v0a1	4E-09	0.4
Tmem121	6E-02	0.2	Zfp553	6E-03	0.2	Nme1	1E-26	0.3	Rogdi	5E-05	0.3	Bmpr1a	4E-16	0.3	Rmdn2	2E-03	0.3	Cyp26b1	3E-03	0.2	Eprs	1E-02	0.2	Rapgefl1	4E-08	0.4	Vps13a	4E-05	0.4
Commd8	2E-05	0.2	Aip	3E-08	0.2	Senp6	2E-22	0.3	Hagh	6E-09	0.3	Rpa3	3E-03	0.3	Slf2	2E-05	0.3	Rybp	9E-02	0.2	Mak16	9E-03	0.2	Negr1	8E-08	0.4	Triobp	4E-07	0.4
Calm1	3E-45	0.2	Capns1	1E-30	0.2	Apbb2	3E-35	0.3	Hspa5	3E-34	0.3	Mex3a	6E-02	0.3	Kif13b	1E-09	0.3	Hypk	4E-02	0.2	Ccdc107	4E-02	0.2	Stat1	3E-07	0.4	Zmynd8	2E-17	0.4
Gtf3c4	5E-02	0.2	Rpl22l1	8E-48	0.2	Id2	2E-14	0.3	Ddx21	2E-13	0.3	Ndrg4	9E-02	0.3	Clec2d	5E-07	0.3	Dyrk1a	7E-01	0.2	Mycbp	2E-04	0.2	Ints10	4E-01	0.4	Stradb	2E-01	0.4
Lrsam1	6E-03	0.2	Pappa	3E-21	0.2	Pgk1	7E-15	0.3	Magoh	2E-15	0.3	Adgrg6	7E-14	0.3	Prpf19	2E-07	0.3	Phactr1	7E-01	0.2	Armt1	6E-06	0.2	Dipk1b	5E-06	0.4	Extl3	8E-04	0.4
Gga1	2E-05	0.2	Lrrc42	9E-13	0.2	Abi2	5E-05	0.3	Rxylt1	8E-12	0.3	Dazap1	2E-09	0.3	Dbi	8E-30	0.3	Abcf3	9E-01	0.2	Fem1a	8E-04	0.2	Fryl	4E-06	0.4	Psap	1E-26	0.4
Sept8	1E-08	0.2	Mmgt2	8E-06	0.2	Trim44	3E-12	0.3	Tvp23b	4E-11	0.3	Med24	1E-02	0.3	Helq	1E-02	0.3	C1qbp	9E-02	0.2	Mbtps1	3E-06	0.2	Strn	4E-05	0.4	Calhm2	3E-03	0.4
Rab3ip	4E-02	0.2	Tnks1bp1	1E-09	0.2	Sod2	6E-22	0.3	P4ha1	3E-30	0.3	Tex2	2E-01	0.3	Trappc1	3E-09	0.3	2610020C07Rik	9E-01	0.2	Ptcd3	7E-05	0.2	Csk	5E-09	0.4	Klhl7	2E-03	0.4
Scyl1	4E-05	0.2	Txndc5	3E-17	0.2	Mark3	2E-13	0.3	B230216N24Rik	5E-02	0.3	Snx18	1E-08	0.3	Col5a2	5E-27	0.3	Sertad2	2E-01	0.2	Zfr2	2E-01	0.2	Meis1	3E-03	0.4	Wasf2	3E-20	0.4
Tulp4	1E-15	0.2	Ints12	3E-03	0.2	Tmem243	4E-03	0.3	Pias3	1E-01	0.3	Pdss1	5E-02	0.3	Rpain	6E-04	0.3	4933421O10Rik	7E-01	0.2	Fbxl4	1E-05	0.2	Ei24	4E-13	0.4	Npdc1	7E-18	0.4
Nagk	1E-08	0.2	Fat1	1E-20	0.2	Aasdhppt	2E-07	0.3	Tut1	5E-03	0.3	Cby1	3E-04	0.3	Prpf4	2E-06	0.3	Vgll4	4E-01	0.2	Plekhm1	3E-03	0.2	Col6a1	2E-44	0.4	Dip2b	9E-11	0.4
Ctns	3E-02	0.2	Plin2	2E-18	0.2	Fan1	3E-02	0.3	4933406I18Rik	9E-04	0.3	Smyd4	5E-04	0.3	S100a11	6E-48	0.3	Dop1a	6E-01	0.2	3110021N24Rik	2E-01	0.2	Rtp4	3E-02	0.4	Setd4	1E-02	0.4
Mad2l2	4E-02	0.2	Rab40b	1E-05	0.2	Prr7	1E-02	0.3	Dkc1	3E-06	0.3	Mterf4	4E-03	0.3	Cyp1b1	9E-09	0.3	Dbi	4E-07	0.2	Lsg1	1E-03	0.2	Cpa6	7E-03	0.4	Pde4d	8E-05	0.4
Ank2	2E-15	0.2	Dnm2	1E-11	0.2	Sirt3	7E-05	0.3	Wdr34	8E-03	0.3	Clasp1	9E-07	0.3	Proser1	6E-03	0.3	Rps8	1E-36	0.2	Ttc39b	1E-02	0.2	Ehd1	2E-11	0.4	Mcub	7E-05	0.4
Slc30a4	2E-02	0.2	Pnkp	5E-04	0.2	Clmn	8E-03	0.3	Fuca2	1E-06	0.3	Gm11266	1E-02	0.3	Sh3rf1	1E-08	0.3	Dusp6	6E-02	0.2	Zfp512	2E-04	0.2	Col6a3	4E-21	0.4	Abhd17c	2E-02	0.4
Fbxo25	3E-05	0.2	Mtmr11	3E-06	0.2	Odc1	6E-13	0.3	Dctd	8E-03	0.3	Fam118b	1E-03	0.3	Pip5k1b	6E-05	0.3	Ndufb3	8E-01	0.2	Myo9b	1E-01	0.2	Smyd3	2E-15	0.4	Myo5a	1E-08	0.4
Srebf2	2E-09	0.2	Lrch2	9E-03	0.2	1700012D14Rik	7E-02	0.3	Ccdc59	1E-12	0.3	Gm49692	6E-02	0.3	Josd1	2E-03	0.3	Hoxa10	6E-01	0.2	Isg20l2	4E-07	0.2	Glb1l	6E-04	0.4	Sugp1	2E-01	0.4
Glb1	1E-08	0.2	Eml4	1E-09	0.2	Gm4924	1E-03	0.3	Polr1a	3E-03	0.3	S100a10	1E-20	0.3	E2f3	3E-07	0.3	Cluh	3E-02	0.2	Tkt	6E-08	0.2	Inpp5k	8E-03	0.4	Arntl2	9E-02	0.4
Scpep1	6E-19	0.2	Gng10	4E-23	0.2	Aldh4a1	2E-01	0.3	Brix1	1E-09	0.3	Gtpbp3	4E-01	0.3	Rapgef4	2E-03	0.3	Tpt1	1E-51	0.2	Uqcrb	1E-01	0.2	Dcn	9E-22	0.4	Zfp369	2E-02	0.4
Svbp	5E-14	0.2	Smpd4	1E-03	0.2	Sfxn1	2E-14	0.3	1700084C06Rik	3E-04	0.3	Gng12	3E-13	0.3	Zmat1	2E-06	0.3	Pfkfb3	1E-01	0.2	Dhrs9	9E-01	0.2	Lclat1	2E-04	0.4	Akr1b8	2E-02	0.4
Gmppa	1E-03	0.2	Sys1	7E-15	0.2	Mettl2	6E-03	0.3	Gna12	1E-07	0.3	Exosc10	3E-04	0.3	Msmo1	2E-08	0.3	Trappc6a	5E-01	0.2	Efnb2	1E-03	0.2	Pcgf1	7E-02	0.4	Inpp5f	8E-02	0.4
Fgd1	5E-05	0.2	Ssh1	9E-06	0.2	Gde1	1E-14	0.3	Ssr3	5E-29	0.3	Pxn	1E-04	0.3	Atp10a	1E-09	0.3	Qtrt2	8E-01	0.2	Urod	3E-06	0.2	E130308A19Rik	2E-03	0.4	Nhlrc2	3E-05	0.4
Fam126a	1E-04	0.2	Uap1l1	3E-15	0.2	Mirg	4E-04	0.3	Wdr43	1E-10	0.3	Akip1	2E-04	0.3	H1f0	2E-07	0.3	Dnmbp	3E-01	0.2	P4ha2	1E-02	0.2	Plekha4	8E-04	0.4	Sgms2	8E-02	0.4
Ppp2r5b	5E-03	0.2	Litaf	6E-09	0.2	6430590A07Rik	2E-02	0.3	Mlh1	1E-01	0.3	Tmem140	9E-04	0.3	Mark2	5E-08	0.3	Nr4a1	2E-01	0.2	Hadh	1E-01	0.2	Plekhm2	4E-09	0.4	Plekho1	8E-03	0.4
Mpdu1	3E-02	0.2	Fam43a	9E-05	0.2	Slc39a7	3E-14	0.3	Bcl7a	6E-02	0.3	Tubgcp2	1E-03	0.3	B4galt5	1E-02	0.3	Bcl7b	8E-02	0.2	Gtpbp6	9E-06	0.2	Itgb1bp1	6E-09	0.4	Elovl1	2E-08	0.4
Fam189b	2E-03	0.2	Gstp1	2E-07	0.2	Hagh	7E-07	0.3	Morn2	2E-07	0.3	Cry2	6E-09	0.3	Rnf141	4E-03	0.3	Zfp646	6E-01	0.2	Nrk	8E-01	0.2	Sprtn	2E-02	0.4	Cyb5b	5E-07	0.4
Dbr1	1E-01	0.2	Lrrc14	1E-03	0.2	Acot11	9E-03	0.3	Kbtbd3	1E-01	0.3	Zfp212	3E-02	0.3	Rhbdd2	1E-04	0.3	Gmds	6E-05	0.2	Snhg12	1E-01	0.2	Cpeb1	1E-09	0.4	Ehbp1l1	6E-05	0.4
Rhoc	1E-32	0.2	Ppp1r7	2E-06	0.2	Gprasp2	1E-05	0.3	Sclt1	7E-06	0.3	Tmco4	1E-04	0.3	Slc41a3	9E-03	0.3	Creld2	3E-02	0.2	Rpl30	2E-26	0.2	Zfp992	5E-02	0.4	Iffo1	2E-02	0.4
Cryz	7E-04	0.2	Icmt	4E-05	0.2	Adck1	3E-03	0.3	Smox	8E-02	0.3	Cdr2l	2E-02	0.3	Dhx15	1E-09	0.3	Thbs3	8E-03	0.2	Gm35188	4E-03	0.2	Oxsm	2E-02	0.4	Notch1	2E-01	0.4
Aacs	9E-05	0.2	Pth1r	3E-08	0.2	Wdr48	8E-05	0.3	Twnk	1E-02	0.3	Vmac	3E-01	0.3	Med14	1E-08	0.3	Mre11a	8E-01	0.2	Atg2b	7E-02	0.2	Snx33	4E-04	0.4	Grina	1E-13	0.4
Arpc3	3E-28	0.2	Tesk2	7E-04	0.2	Sec14l1	3E-11	0.3	Rpa3	2E-03	0.3	Cipc	6E-02	0.3	Fmr1	1E-12	0.3	Hgsnat	2E-02	0.2	Rpl36a	2E-20	0.2	Abhd18	2E-04	0.4	Bak1	3E-04	0.4
Sptlc2	3E-04	0.2	Helb	3E-05	0.2	Tnc	8E-23	0.3	Tmem161b	7E-03	0.3	Mipep	1E-02	0.3	Ergic2	6E-11	0.3	Prnp	2E-08	0.2	Poglut2	2E-03	0.2	Washc2	7E-13	0.4	Plxdc2	2E-17	0.4
Nol3	1E-01	0.2	Dpf2	5E-08	0.2	Parp8	3E-06	0.3	Gnptg	2E-05	0.3	Man2a1	1E-07	0.3	Pea15a	3E-07	0.3	Tmem181a	1E-03	0.2	Ctsf	7E-02	0.2	Trpm4	1E-01	0.4	Srfbp1	2E-05	0.4
Clcn6	1E-01	0.2	Lta4h	1E-06	0.2	Uspl1	6E-03	0.3	Lemd2	2E-04	0.3	Cntln	3E-05	0.3	Zfp825	4E-02	0.3	Rplp0	1E-37	0.2	Cln5	4E-10	0.2	Higd1a	2E-09	0.4	Tmem131l	8E-02	0.4
Iqcc	4E-02	0.2	Actg1	2E-30	0.2	Wdr43	6E-14	0.3	Nr1d2	7E-05	0.3	Gss	1E-02	0.3	Trerf1	4E-06	0.3	Camk1	7E-01	0.2	Hspbap1	3E-05	0.2	Ttc21b	3E-04	0.4	Rfng	1E-01	0.4
Acox3	3E-01	0.2	St3gal2	2E-07	0.2	Fbxo42	9E-05	0.3	Homer1	2E-03	0.3	Dars2	4E-03	0.3	Zfp503	2E-07	0.3	Srsf10	6E-02	0.2	Pdcd6	1E-06	0.2	Rmc1	1E-02	0.4	Ctsa	1E-16	0.4
Gng10	1E-10	0.2	Stard4	1E-02	0.2	Erall	1E-01	0.3	Akap8	3E-02	0.3	3110082I17Rik	5E-05	0.3	Rapgef6	2E-09	0.3	Habp4	5E-02	0.2	Pnkd	6E-03	0.2	Frmd6	2E-13	0.4	Kdm5d	3E-04	0.4
Acot9	1E-09	0.2	Tprkb	5E-06	0.2	G6pc3	1E-06	0.3	Plod1	3E-11	0.3	Zbed4	3E-03	0.3	Tbc1d19	4E-08	0.3	Nr2c1	2E-02	0.2	Fam45a	1E-07	0.2	Gm20342	7E-02	0.4	Pdgfra	7E-08	0.4
Noc4l	2E-01	0.2	Kif3c	5E-07	0.2	Tbpl1	6E-06	0.3	Gm5617	3E-09	0.3	Lrfn4	7E-03	0.3	Msh3	1E-03	0.3	Acad10	2E-01	0.2	Patj	6E-01	0.2	Slc9a3r1	2E-02	0.4	Nipa1	9E-02	0.4
Tmem68	8E-03	0.2	Pqlc1	8E-05	0.2	Snhg15	2E-04	0.3	Slc9a6	3E-02	0.3	Robo1	1E-07	0.3	Zfp53	1E-02	0.3	Lcor	5E-01	0.2	Aldh1a1	9E-01	0.2	Rab43	1E-06	0.4	Wipf1	3E-05	0.4
Golph3l	2E-02	0.2	Dcp2	8E-05	0.2	Hint2	7E-14	0.3	Zfp317	2E-01	0.3	Cc2d2a	1E-04	0.3	Gm13986	4E-04	0.3	Rmnd5a	2E-02	0.2	Psmb4	3E-07	0.2	Spg21	3E-12	0.4	Pgd	3E-06	0.4
Ppp1ca	5E-24	0.2	Gemin8	2E-02	0.2	Apobr	1E-03	0.3	Polr2g	1E-15	0.3	Zc3h12a	2E-02	0.3	Dhx9	2E-11	0.3	Mettl26	1E+00	0.2	Eef1b2	1E-15	0.2	Ppm1f	5E-02	0.4	Acsl5	2E-02	0.4
Tmem219	1E-07	0.2	Lpp	9E-19	0.2	Wdyhv1	3E-02	0.3	Ino80	6E-07	0.3	Txnrd1	1E-07	0.3	Noct	1E-03	0.3	Rragb	7E-01	0.2	Strbp	5E-01	0.2	Nhej1	1E-02	0.4	Spag9	1E-20	0.4
Trip10	4E-02	0.2	Colgalt1	2E-16	0.2	Fancc	2E-08	0.3	F8	4E-05	0.3	Slc7a6	2E-03	0.3	9230114K14Rik	5E-04	0.3	Glt8d2	2E-01	0.2	Aasdhppt	3E-05	0.2	Gm14305	1E-02	0.4	Cipc	5E-02	0.4
Coa5	1E-04	0.2	Maoa	9E-07	0.2	Lyplal1	3E-02	0.3	Zfp330	3E-07	0.3	Scoc	2E-08	0.3	Phf5a	3E-10	0.3	Mark3	3E-01	0.2	Hacl1	8E-03	0.2	Prps2	2E-03	0.4	Twist1	3E-08	0.4

Limb Mesenchyme

Control Gene	p	FC	$Notch2^{tm1.1Ecan}$ Gene	p	FC
Rex1bd	2E-24	0.2	Pop1	4E-03	0.2
Brf2	2E-01	0.2	Ifi35	3E-09	0.2
Stard4	2E-01	0.2	Aacs	6E-05	0.2
Cdkn1a	1E-14	0.2	Tcf3	2E-07	0.2
Smarca1	3E-02	0.2	Herpud2	9E-10	0.2
Rbbp5	1E-01	0.2	Aph1a	2E-12	0.2
Ehmt2	2E-10	0.2	C030014I23Rik	3E-04	0.2
Dnm2	1E-11	0.2	Pdss1	2E-02	0.2
Taf8	1E-02	0.2	Jun	2E-20	0.2
Rflnb	5E-07	0.2	Mcur1	9E-08	0.2
Mvk	6E-05	0.2	Ccnyl1	5E-05	0.2
Gskip	3E-03	0.2	Rnpepl1	1E-05	0.2
Ccdc22	2E-02	0.2	Cpne2	6E-06	0.2
Tmx3	7E-07	0.2	Msrb3	2E-11	0.2
Tns1	4E-08	0.2	H13	2E-14	0.2
Bace1	7E-05	0.2	Rab11a	7E-16	0.2
Tmem97	6E-04	0.2	Pcgf2	8E-06	0.2
Mad2l1bp	7E-03	0.2	Tcf7l1	1E-07	0.2
Rpl28	2E-102	0.2	Parp9	6E-05	0.2
9430038I01Rik	7E-02	0.2	Katnal1	6E-03	0.2
Inf2	3E-04	0.2	Ntmt1	1E-10	0.2
Tmem9	2E-04	0.2	Crls1	4E-09	0.2
Zfp467	2E-02	0.2	Map1lc3a	3E-26	0.2
Pkd2	2E-09	0.2	Tusc1	1E-06	0.2
Ctnnbip1	8E-07	0.2	Gnai1	2E-04	0.2
Nosip	3E-03	0.2	Sept7	8E-33	0.2
Gstp1	3E-02	0.2	Tubb2b	9E-07	0.2
Znfx1	2E-01	0.2	Cdo1	7E-06	0.2
Aip	2E-05	0.2	Tmem97	8E-08	0.2
Nid2	1E-08	0.2	Ssbp4	5E-09	0.2
Atf7	6E-08	0.2	Col6a3	1E-19	0.2
Tesk1	2E-03	0.2	Frk	1E-08	0.2
Urod	1E-03	0.2	Trim56	4E-04	0.2
Pisd	5E-02	0.2	Mt2	9E-18	0.2
Pltp	5E-15	0.2	Tmem115	8E-09	0.2
Zfp655	2E-03	0.2	Lamc1	1E-09	0.2
Ece2	1E-03	0.2	Rusc1	1E-04	0.2
S100a16	9E-12	0.2	Snx32	2E-03	0.2
Eif3f	1E-33	0.2	Mettl1	4E-11	0.2
H1f0	3E-07	0.2	Kctd6	1E-03	0.2
Dnajc25	1E-06	0.2	Calm3	3E-25	0.2
Pip5k1c	4E-02	0.2	Ptpn13	1E-04	0.2
Ap2m1	1E-12	0.2	Arl15	1E-16	0.2
Rab40b	9E-05	0.2	Atp6v0b	3E-18	0.2
Plpp3	5E-29	0.2	Snrk	5E-07	0.2
Fam89b	3E-06	0.2	Sh3bp5l	1E-04	0.2
Lmna	4E-36	0.2	2810433D01Rik	2E-03	0.2
Get4	6E-06	0.2	Cep164	9E-09	0.2
Adam15	6E-05	0.2	Sgpl1	2E-11	0.2
Mmab	7E-03	0.2	Piezo1	4E-13	0.2
Dusp19	1E-01	0.2	Wbp1l	2E-06	0.2
Ecd	3E-01	0.2	Acaa2	5E-17	0.2
Ggh	2E-10	0.2	Eif4ebp3	2E-02	0.2
Hacd2	2E-04	0.2	Ubald1	3E-09	0.2
Glrx3	2E-21	0.2	Hspb1	6E-08	0.2
Gria3	1E-13	0.2	Nectin2	4E-04	0.2
Zdhhc7	8E-02	0.2	Prkar2b	4E-08	0.2
Enoph1	7E-01	0.2	4930402H24Rik	8E-09	0.2
Smarca2	2E-12	0.2	Nenf	1E-35	0.2
Pip4k2a	3E-03	0.2	Fkbp8	2E-13	0.2
Nt5dc3	5E-04	0.2	Unc119	1E-07	0.2
Aph1b	1E-01	0.2	Rpsa	3E-55	0.2
Rnf144a	7E-02	0.2	Rab34	3E-22	0.2
Upf1	2E-03	0.2	Htatip2	2E-08	0.2

Chondrogenic

Control Gene	p	FC	$Notch2^{tm1.1Ecan}$ Gene	p	FC
Jak2	6E-04	0.3	Zfp24	7E-06	0.3
Mrpl44	1E-06	0.3	Zrsr2	5E-13	0.3
Gmeb1	4E-03	0.3	Kcnk2	2E-08	0.3
Hmox2	1E-08	0.3	Snhg12	4E-20	0.3
Tfb2m	2E-03	0.3	Zswim1	3E-01	0.3
Smim10l1	2E-23	0.3	Nup43	8E-04	0.3
Ap4s1	1E-07	0.3	Cdyl2	4E-02	0.3
Mafb	5E-01	0.3	Tmed9	2E-34	0.3
Gmds	2E-29	0.3	Tmed10	7E-40	0.3
E130102H24Rik	8E-03	0.3	Iffo2	2E-06	0.3
Rad1	4E-03	0.3	Wasf3	6E-04	0.3
Tnfrsf11b	2E-32	0.3	Rsl1d1	1E-18	0.3
Slc7a6os	6E-04	0.3	Zfpm2	5E-19	0.3
Acp6	4E-02	0.3	Fbxo33	2E-01	0.3
Ccnd2	9E-35	0.3	2810403D21Rik	2E-05	0.3
Impa2	4E-06	0.3	Mef2b	3E-02	0.3
Gpatch4	8E-05	0.3	B3glct	3E-09	0.3
Pogz	4E-02	0.3	Fut8	7E-05	0.3
Cdk5rap2	1E-05	0.3	Patz1	2E-01	0.3
Bhlhe40	2E-03	0.3	Bccip	7E-10	0.3
Mrpl57	8E-26	0.3	Plekha3	3E-09	0.3
Casc4	8E-14	0.3	Bpgm	5E-03	0.3
2810013P06Rik	3E-04	0.3	Dele1	3E-03	0.3
Btbd1	6E-15	0.3	Matn4	2E-15	0.3
Mecr	9E-06	0.3	Wdr4	3E-03	0.3
Zrsr2	4E-11	0.3	2410006H16Rik	2E-22	0.3
Atg14	2E-02	0.3	Snhg15	3E-03	0.3
Elmo1	1E-04	0.3	Wdcp	5E-01	0.3
Zfp738	1E-01	0.3	Sf3b6	1E-26	0.3
Trim39	1E-02	0.3	Fhit	3E-12	0.3
Tefm	7E-03	0.3	Clcn5	7E-09	0.3
Klf12	2E-11	0.3	Tpk1	7E-06	0.3
2410018L13Rik	6E-04	0.3	Pacsin2	1E-10	0.3
Eif5	3E-26	0.3	Casc4	9E-12	0.3
Odf2l	3E-03	0.3	Ubr4	1E-08	0.3
Timm9	3E-07	0.3	Bysl	3E-04	0.3
Dph7	8E-02	0.3	Tmbim4	1E-19	0.3
Rbm4	4E-08	0.3	Srsf9	2E-17	0.3
Dalrd3	5E-03	0.3	Lman1	2E-31	0.3
Zbtb5	1E-01	0.3	Rufy3	8E-06	0.3
Inpp4b	1E-19	0.3	Sppl2b	1E-02	0.3
Slc25a23	5E-02	0.3	Cepg1os	1E-03	0.3
Dnajc11	4E-03	0.3	Bcar3	3E-04	0.3
Cnpy3	5E-09	0.3	Arfgap1	2E-08	0.3
Phf7	7E-04	0.3	Ppp1r13b	3E-04	0.3
Osbpl9	3E-23	0.3	Ndufaf3	4E-07	0.3
Eif4e	5E-13	0.3	Slc35e2	4E-01	0.3
Pitpnm2	9E-03	0.3	Dimt1	7E-04	0.3
Thrb	1E-01	0.3	Ooep	2E-05	0.3
Prkcsh	7E-17	0.3	Lin52	2E-04	0.3
Pex2	9E-06	0.3	Tmem101	2E-03	0.3
Ptp4a2	8E-36	0.3	Hspd1	5E-28	0.3
Ring1	4E-07	0.3	Dancr	3E-01	0.3
Tbc1d22a	3E-06	0.3	Mllt11	3E-04	0.3
Mtg2	5E-04	0.3	Zfp952	7E-02	0.3
Uchl3	5E-08	0.3	Ppdpf	3E-09	0.3
Ccdc173	8E-03	0.3	Gm4285	6E-03	0.3
Pcid2	6E-01	0.3	Ggct	8E-03	0.3
Tpt1	8E-143	0.3	Sehl1	2E-04	0.3
Sppl2a	3E-09	0.3	Clybl	2E-08	0.3
Tmco3	6E-08	0.3	Secisbp2	1E-06	0.3
Itfg2	5E-05	0.3	Nfkbib	5E-02	0.3
Fbxo9	4E-08	0.3	Phf1	1E-03	0.3
Chchd7	1E-14	0.3	Nifk	7E-07	0.3

Fibroblast

Control Gene	p	FC	$Notch2^{tm1.1Ecan}$ Gene	p	FC
Gm49969	5E-04	0.3	Thoc6	6E-05	0.3
Pole3	2E-05	0.3	S100a10	3E-34	0.3
Igsf8	1E-03	0.3	Gk5	1E-05	0.3
Zfp180	3E-01	0.3	Sephs1	6E-05	0.3
Mrpl10	9E-04	0.3	Supt16	7E-15	0.3
Rpp21	4E-06	0.3	Gabarapl1	2E-08	0.3
Mbnl3	2E-02	0.3	Emc8	9E-08	0.3
Zcchc2	9E-02	0.3	Ift22	1E-05	0.3
Stk38l	7E-05	0.3	Slco3a1	2E-05	0.3
Faap24	3E-03	0.3	3110082I17Rik	1E-04	0.3
Uxs1	5E-07	0.3	Tmem161b	2E-03	0.3
Fuom	1E-05	0.3	Cenpb	6E-15	0.3
Wrap73	1E-02	0.3	Hdac1	4E-14	0.3
Vbp1	4E-05	0.3	Ifi35	1E-03	0.3
Rel	2E-07	0.3	Dusp1	7E-15	0.3
Lrch3	8E-08	0.3	Coro1c	4E-09	0.3
Mtrf1	7E-01	0.3	Ano6	1E-10	0.3
Stk17b	5E-03	0.3	Dnah7b	4E-03	0.3
Msmp	1E-06	0.3	Actn4	1E-18	0.3
Itgb5	3E-14	0.3	Ago2	3E-11	0.3
1500004A13Rik	1E-01	0.3	Ints5	6E-04	0.3
Cdc42ep2	2E-04	0.3	Dock11	1E-05	0.3
Dusp1	6E-15	0.3	Esyt2	8E-12	0.3
Hoxd8	3E-02	0.3	Ralgds	4E-05	0.3
Arhgap23	6E-12	0.3	Plp2	1E-07	0.3
Slf2	4E-04	0.3	Brd8	9E-10	0.3
Vps13d	5E-10	0.3	Plxnb2	4E-10	0.3
Wbp1	1E-05	0.3	Pxmp4	1E-05	0.3
Acer3	7E-05	0.3	Hsd17b12	2E-11	0.3
Aopep	4E-30	0.3	Otud1	5E-02	0.3
Agtpbp1	2E-06	0.3	Dars2	1E-03	0.3
Cep104	1E-02	0.3	Zswim1	8E-02	0.3
Parp1	5E-04	0.3	Fndc4	6E-05	0.3
Acsl4	5E-04	0.3	Zfp933	2E-03	0.3
Pdzrn3	2E-12	0.3	Yes1	9E-04	0.3
Orc2	3E-04	0.3	Cdr2l	4E-01	0.3
Atp8b2	2E-01	0.3	Sac3d1	3E-07	0.3
Hnrnpab	2E-10	0.3	Tmem109	8E-10	0.3
Pon3	1E-05	0.3	2610020C07Rik	8E-04	0.3
Dip2b	2E-05	0.3	Mfn2	2E-06	0.3
Clcn6	4E-03	0.3	Psme2	2E-16	0.3
Ago2	3E-08	0.3	Dzip3	2E-05	0.3
Srbd1	1E-04	0.3	Tmem132a	3E-07	0.3
Rusc2	1E-07	0.3	Sept11	2E-02	0.3
Zfp219	2E-01	0.3	Dis3l	7E-04	0.3
Rhbdd2	2E-01	0.3	Tgfb1	4E-08	0.3
Tmem18	2E-01	0.3	Gzf1	7E-04	0.3
Rasal2	2E-10	0.3	Gstm1	4E-13	0.3
Gins4	5E-03	0.3	Ruvbl2	8E-06	0.3
Ctdspl2	2E-05	0.3	Agps	3E-07	0.3
Map3k20	2E-08	0.3	Lhx9	1E-03	0.3
Zbtb40	1E-01	0.3	Fancf	3E-04	0.3
Lca5	6E-03	0.3	Opa3	3E-05	0.3
Fbn2	5E-07	0.3	Stk24	5E-10	0.3
Lrrc17	7E-02	0.3	Pwwp3a	4E-07	0.3
Pitpnm2	4E-03	0.3	Ybx1	1E-43	0.3
Hnrnpa1	2E-18	0.3	Brpf1	4E-03	0.3
2700049A03Rik	1E-03	0.3	Smpd4	1E-02	0.3
Zdhhc7	6E-03	0.3	Lrrfip1	2E-15	0.3
Ephb4	3E-01	0.3	Nln	3E-06	0.3
Rab13	3E-04	0.3	Zc3hav1	1E-06	0.3
Ilf2	5E-07	0.3	Spata7	8E-05	0.3
Zfp692	5E-02	0.3	Pelp1	3E-03	0.3
Dst	8E-17	0.3	Fam126a	1E-06	0.3

Undefined

Control Gene	p	FC	$Notch2^{tm1.1Ecan}$ Gene	p	FC
Ghitm	1E+00	0.2	Ramac	2E-06	0.2
Rabepk	4E-01	0.2	Pecr	2E-02	0.2
Sppl2b	1E-01	0.2	Idh3a	6E-04	0.2
Ptp4a2	1E-04	0.2	Jrkl	7E-04	0.2
Vps72	8E-01	0.2	Vps37c	6E-05	0.2
Tsen15	7E-01	0.2	2410002F23Rik	3E-04	0.2
Cep57	8E-01	0.2	Gpaa1	6E-07	0.2
Pnpla7	3E-02	0.2	Poglut1	4E-07	0.2
Morn2	2E-01	0.2	Btg2	2E-01	0.2
Pex7	4E-01	0.2	Zfp428	1E-05	0.2
Dars2	5E-01	0.2	Mia2	8E-03	0.2
Rpl10-ps3	6E-01	0.2	Nkiras1	1E-02	0.2
Micos13	3E-01	0.2	Ssr1	2E-09	0.2
Mrpl55	6E-01	0.2	Slc35b1	3E-02	0.2
2810004N23Rik	2E-01	0.2	Pag1	5E-01	0.2
Ttc5	5E-02	0.2	Farsb	4E-03	0.2
Ehd3	5E-01	0.2	Rps3a1	7E-29	0.2
Ppp1r21	3E-03	0.2	Ing4	3E-06	0.2
Rab11fip3	5E-01	0.2	Rian	1E+00	0.2
Krr1	4E-01	0.2	Osbpl9	4E-01	0.2
Actl6a	6E-01	0.2	Mtif3	1E-02	0.2
Tmem201	6E-01	0.2	Limk2	3E-02	0.2
Dele1	5E-01	0.2	Vta1	3E-08	0.2
Sgms2	7E-01	0.2	Rpn2	3E-03	0.2
Bcl9	8E-01	0.2	Nudt8	2E-05	0.2
Wdr43	1E+00	0.2	Zmym2	1E-04	0.2
Zfp467	2E-01	0.2	Gm20275	4E-03	0.2
Crlf3	9E-01	0.2	Nup35	1E-04	0.2
Zc3h4	3E-01	0.2	Id3	6E-01	0.2
Vdac3	2E-01	0.2	Rpl22	2E-15	0.2
Hspd1	9E-02	0.2	Osgepl1	5E-01	0.2
Prelid3b	2E-02	0.2	Kif1bp	6E-09	0.2
Tmed10	1E-10	0.2	Rsl1d1	3E-03	0.2
Ppib	2E-19	0.2	Plscr2	5E-02	0.2
Eif2s2	2E-02	0.2	Zfp945	6E-03	0.2
Ctnna1	3E-01	0.2	Mettl5	3E-02	0.2
Trps1	1E-08	0.2	Urm1	1E-05	0.2
Spry2	5E-01	0.2	Clmn	5E-02	0.2
Nup35	4E-03	0.2	5330438D12Rik	5E-05	0.2
mt-Nd6	8E-01	0.2	Llph	2E-04	0.2
1500011B03Rik	5E-01	0.2	Cdk2ap2	8E-04	0.2
Hibch	2E-01	0.2	Plpp2	2E-05	0.2
Mrpl57	3E-01	0.2	Nme1	1E-01	0.2
Cdk8	3E-03	0.2	Zbtb20	1E-06	0.2
Neo1	9E-02	0.2	D3Ertd751e	6E-01	0.2
Cnpy3	4E-01	0.2	Mrpl10	5E-06	0.2
Zdhhc12	6E-01	0.2	Fam199x	3E-06	0.2
Vhl	6E-01	0.2	Tmem176b	2E-04	0.2
Mid1ip1	4E-02	0.2	Tmco3	1E-03	0.2
Trim35	5E-01	0.2	Anxa7	7E-04	0.2
Pigh	3E-01	0.2	Rpl23	9E-32	0.2
Uba52	1E+00	0.2	Copb1	5E-11	0.2
Slc25a20	3E-01	0.2	Prdx3	3E-04	0.2
Map9	7E-01	0.2	Rtraf	4E-02	0.2
Ppm1b	4E-01	0.2	Ndufc2	5E-02	0.2
Mrpl20	7E-01	0.2	Nupl2	1E-02	0.2
2900076A07Rik	4E-01	0.2	Mecp2	2E-05	0.2
Ift81	3E-01	0.2	Cisd1	3E-02	0.2
Ankra2	7E-01	0.2	Nuak1	6E-03	0.2
Uros	3E-01	0.2	Tmem38b	3E-05	0.2
Smpd1	5E-02	0.2	Gar1	2E-02	0.2
Zfr	1E-02	0.2	Rpl26	6E-26	0.2
Nme1	1E-01	0.2	Rpl9	1E-20	0.2
Neurl4	7E-01	0.2	Uxt	2E-04	0.2

Articular/Synovial Fibroblast

Control Gene	p	FC	$Notch2^{tm1.1Ecan}$ Gene	p	FC
LTO1	3E-04	0.4	Pik3r3	7E-05	0.4
Rbpj	3E-19	0.4	Bach1	3E-04	0.4
Zfp53	3E-02	0.4	BC002059	5E-01	0.4
Pi15	1E-09	0.4	Dcn	2E-13	0.4
Acer3	5E-10	0.4	Ecm2	2E-05	0.4
Tdrd7	4E-04	0.4	Abl1	5E-07	0.4
Ctsb	3E-34	0.4	Dipk1a	2E-02	0.4
Fdxr	1E-02	0.4	Mavs	3E-02	0.4
Wwox	1E-12	0.4	Pitrm1	1E-04	0.4
Tacc2	6E-06	0.4	Ncoa7	2E-05	0.4
Slc44a1	2E-14	0.4	Aco1	1E-04	0.4
Tbc1d17	7E-05	0.4	Ppp2r1b	5E-03	0.4
Tram2	4E-08	0.4	Idnk	5E-02	0.4
Fbxw11	5E-13	0.4	Mgll	2E-03	0.4
Zfp800	3E-04	0.4	Spsb2	1E-04	0.4
Rap1b	2E-27	0.4	Tmem256	2E-18	0.4
Ift22	5E-05	0.4	Pfkp	2E-04	0.4
A430033K04Rik	5E-02	0.4	Natd1	1E-02	0.4
Rps6kc1	6E-08	0.4	Ly96	1E-05	0.4
Adam10	1E-08	0.4	Fbrs	5E-02	0.4
Rtn3	3E-24	0.4	Chmp1b	1E-01	0.4
Clstn1	3E-13	0.4	Slc22a15	2E-01	0.4
Atl2	1E-02	0.4	Ehd3	3E-03	0.4
Ebf3	8E-12	0.4	Zfp949	1E-01	0.4
Pafah2	1E-03	0.4	Gramd3	4E-04	0.4
Dlg3	2E-02	0.4	Taf4	5E-02	0.4
Ccdc181	2E-02	0.4	Rnf13	3E-09	0.4
Hsf2	7E-04	0.4	Zfp397	7E-06	0.4
Fam129b	3E-05	0.4	Slc2a8	2E-02	0.4
Wdpcp	2E-07	0.4	Layn	1E-02	0.4
Kbtbd2	2E-03	0.4	Snx29	7E-06	0.4
Psap	6E-37	0.4	Chfr	2E-05	0.4
Nfia	2E-23	0.4	Epb41l2	7E-13	0.4
Amer1	6E-04	0.4	Sik2	1E-04	0.4
Ccdc85b	7E-10	0.4	Ripor1	7E-04	0.4
Elovl1	2E-09	0.4	Flt3l	5E-02	0.4
Pdgfrb	2E-11	0.4	Stk10	5E-03	0.4
Rb1cc1	2E-18	0.4	Atrip	4E-02	0.4
Jarid2	9E-06	0.4	Ppp1r9b	2E-03	0.4
Ccdc57	6E-03	0.4	Sbf2	1E-12	0.4
Mex3c	1E-05	0.4	Poc5	7E-02	0.4
Scpep1	2E-22	0.4	Firre	5E-02	0.4
H6pd	7E-04	0.4	Abhd13	1E-01	0.4
Ctns	5E-06	0.4	Arhgef12	8E-14	0.4
R74862	4E-02	0.4	Bcap29	7E-04	0.4
Slc35d2	2E-03	0.4	Cd164	9E-11	0.4
Pitrm1	1E-04	0.4	Edem2	4E-03	0.4
Cln3	1E-01	0.4	Pip5k1a	1E-03	0.4
Hsd17b12	3E-13	0.4	Scn2a	9E-03	0.4
Pcdh7	4E-08	0.4	Atxn1	5E-15	0.4
Snx32	1E-04	0.4	Acox3	4E-02	0.4
Dennd1a	6E-17	0.4	Kif1b	6E-15	0.4
Slc38a7	1E-02	0.4	D830050J10Rik	2E-04	0.4
Tead2	2E-03	0.4	Egfr	4E-11	0.4
Nr3c2	5E-04	0.4	Esyt2	6E-07	0.4
Meox2	9E-08	0.4	Arnt	1E-06	0.4
Pank3	3E-05	0.4	Mrpl16	2E-04	0.4
Rpain	3E-04	0.4	Cpd	4E-06	0.4
Pcmtd2	7E-06	0.4	Ablim1	5E-05	0.4
Hif1a	2E-28	0.4	Kctd11	8E-02	0.4
Kif1b	2E-25	0.4	Hbp1	9E-06	0.4
Ralb	7E-09	0.4	Evc2	3E-02	0.4
Ptbp3	2E-08	0.4	Rrm2b	2E-02	0.4
Asah2	7E-07	0.4	Asap2	1E-05	0.4

Limb Mesenchyme

Control			*Notch2* $^{tm1.1Ecan}$		
Gene	*p*	FC	Gene	*p*	FC
Gm28198	1E-01	0.2	Plin3	2E-11	0.2
Dpp7	8E-06	0.2	Pcolce	1E-29	0.2
Ccdc136	4E-04	0.2	Mat2a	6E-15	0.2
Vegfb	6E-07	0.2	Natd1	3E-04	0.2
Fam177a	4E-01	0.2	Tead3	1E-04	0.2
Cox6a1	1E-20	0.2	Idh3b	1E-09	0.2
Nup37	1E-02	0.2	Ufm1	5E-12	0.2
Ccdc6	2E-03	0.2	Arpc3	3E-27	0.2
Eif3d	2E-11	0.2	Tent2	3E-11	0.2
Col5a2	6E-42	0.2	Zfp532	1E-08	0.2
Dtl	1E-01	0.2	H2-D1	3E-18	0.2
Mtmr14	3E-02	0.2	Atxn1	8E-07	0.2
Mier3	3E-01	0.2	Smim13	3E-05	0.2
Pip4p1	5E-06	0.2	Mcrs1	5E-06	0.2
Thap4	2E-03	0.2	Ccnd2	1E-19	0.2
Pabpc1	4E-34	0.2	Gapdh	6E-28	0.2
Rab1b	5E-07	0.2	Ttbk2	5E-03	0.2
Ostm1	3E-04	0.2	Ankrd24	2E-04	0.2
Rp2	6E-02	0.2	Plod3	2E-10	0.2
Daxx	1E-01	0.2	Prmt5	4E-04	0.2
Fam171a2	3E-04	0.2	Appl1	6E-13	0.2
Psme1	2E-14	0.2	Rab13	2E-07	0.2
Acaa2	4E-10	0.2	D16Ertd472e	5E-03	0.2
Alms1	1E-02	0.2	Atf7ip	2E-08	0.2
Polk	4E-05	0.2	Slc50a1	1E-17	0.2
Gstk1	4E-01	0.2	Wdr77	1E-03	0.2
Cnih1	5E-13	0.2	Eif3f	9E-30	0.2
Anxa1	1E-27	0.2	Tomm5	6E-22	0.2
Gdi1	1E-03	0.2	Pdpn	1E-12	0.2
Hmgcr	5E-05	0.2	Nol6	1E-03	0.2
Mospd1	3E-03	0.2	Trappc1	3E-10	0.2
Klf8	2E-02	0.2	Tmem9	1E-08	0.2
Kctd5	3E-02	0.2	Rnh1	8E-20	0.2
Cept1	2E-05	0.2	Cyren	2E-05	0.2
Arf5	4E-33	0.2	Acvr1	7E-10	0.2
Arhgef40	8E-06	0.2	Mospd1	3E-05	0.2
Tsen34	5E-11	0.2	Gfer	3E-09	0.2
Zfp820	3E-01	0.2	Fam160b1	3E-05	0.2
Tcf7l1	7E-09	0.2	Nsmf	1E-06	0.2
Tab2	6E-08	0.2	Il1r1	6E-01	0.2
Nus1	1E-05	0.2	Zmynd19	6E-05	0.2
Llgl1	5E-03	0.2	Msantd3	4E-08	0.2
Reep4	2E-02	0.2	Zfp318	9E-07	0.2
Tgds	6E-04	0.2	Med20	5E-04	0.2
Rcan3	1E-01	0.2	Timp2	1E-48	0.2
Ccdc107	3E-09	0.2	Xxylt1	1E-06	0.2
Mcrip2	7E-02	0.2	Rab22a	8E-13	0.2
Rtn4	9E-30	0.2	Ccdc136	1E-04	0.2
Pigh	3E-02	0.2	Ahsa2	2E-06	0.2
Ssbp3	3E-12	0.2	Rab4b	8E-11	0.2
Abhd4	7E-04	0.2	Kidins220	2E-08	0.2
Esyt2	3E-08	0.2	Mfsd14b	2E-04	0.2
Fxyd1	8E-11	0.2	Aars	2E-19	0.2
Cdc42ep3	9E-08	0.2	Ndufv1	5E-10	0.2
Gm30025	2E-02	0.2	Nbl1	7E-21	0.2
Hip1	3E-06	0.2	Commd7	3E-07	0.2
B3galt6	1E-01	0.2	Mrpl45	2E-06	0.2
Prkd3	3E-04	0.2	Lamb2	9E-12	0.2
Tmed5	1E-07	0.2	Dhx33	8E-03	0.2
Arl1	2E-20	0.2	Tbc1d17	2E-05	0.2
Rilpl1	1E-05	0.2	Ttll5	5E-06	0.2
Dlg5	8E-03	0.2	Dnajc25	1E-10	0.2
Dapk1	6E-12	0.2	Tgfb3	1E-08	0.2
Tmem209	3E-02	0.2	Rab35	7E-03	0.2

Chondrogenic

Control			*Notch2* $^{tm1.1Ecan}$		
Gene	*p*	FC	Gene	*p*	FC
Dpy30	9E-13	0.3	Slc9a3r2	5E-04	0.3
Nudt5	3E-04	0.3	Tmem189	3E-04	0.3
Antxr2	2E-01	0.3	Mif	7E-27	0.3
Nudt9	2E-09	0.3	Pramef8	6E-02	0.3
Pdia6	4E-35	0.3	Gm12353	1E-03	0.3
Trim35	4E-17	0.3	Nol7	1E-16	0.2
Gm12905	9E-02	0.3	Bcl9	2E-06	0.2
Hspe1-rs1	1E-02	0.3	Chchd7	6E-12	0.2
Tent4b	3E-03	0.3	Otud6b	2E-05	0.2
Polr2g	1E-13	0.3	AW554918	1E-05	0.2
AW209491	3E-04	0.3	Snapc5	2E-06	0.2
Bud31	3E-11	0.3	Bola1	5E-11	0.2
Serpinh1	4E-57	0.3	Shank1	2E-01	0.2
Rps27a	1E-104	0.3	Pex13	4E-06	0.2
Mrps18b	9E-08	0.3	Senp6	6E-19	0.2
Bicral	7E-05	0.3	Rnf167	8E-06	0.2
Ubap2	2E-13	0.3	Alkbh2	2E-02	0.2
Cbwd1	4E-04	0.3	Kri1	2E-01	0.2
Uri1	2E-11	0.3	Dnmt3a	6E-06	0.2
Ndufab1	5E-31	0.3	Smo	2E-12	0.2
Zfp428	5E-04	0.3	Pogz	1E-03	0.2
Cryl1	1E-03	0.3	Zadh2	3E-02	0.2
Rpl36a-ps1	4E-04	0.3	Eml2	7E-05	0.2
Smpdl3b	5E-02	0.3	Ube2e1	1E-12	0.2
P3h1	2E-10	0.3	Taf1d	1E-08	0.2
Itpkb	2E-02	0.3	Rhod	1E-03	0.2
Zfp609	1E-09	0.3	Slc35e1	5E-02	0.2
Msrb2	7E-02	0.3	Traf3	3E-05	0.2
Nop2	3E-04	0.3	Dtna	1E-03	0.2
Fkbp4	8E-13	0.3	Mmaa	8E-03	0.2
Dnajc9	9E-09	0.3	Slc25a37	4E-02	0.2
Col16a1	5E-10	0.3	Taz	7E-04	0.2
Micos13	2E-35	0.3	Mia2	2E-15	0.2
Rpl13a	2E-83	0.3	Mettl22	3E-02	0.2
Eef1g	2E-52	0.3	Uros	1E-03	0.2
Usp54	1E-01	0.3	Ric8b	2E-02	0.2
Atp1b3	3E-12	0.3	Xndc1	3E-01	0.2
Pramef8	2E-02	0.3	Coasy	4E-04	0.2
Bbx	8E-18	0.3	Esf1	1E-09	0.2
D5Ertd579e	1E-04	0.3	Fra10ac1	3E-05	0.2
Prmt3	2E-05	0.3	Timm9	4E-06	0.2
Serp1	3E-16	0.3	Sub1	3E-28	0.2
Akap1	6E-03	0.3	Fnta	5E-16	0.2
Qrsl1	8E-02	0.3	Tmem86a	2E-02	0.2
Igip	1E-03	0.3	Atp6v0a2	3E-03	0.2
Iffo2	2E-03	0.3	Nck2	4E-03	0.2
Rxylt1	4E-15	0.3	Atpsckmt	3E-04	0.2
Coasy	1E-03	0.3	Sertad4	7E-04	0.2
Mylip	6E-04	0.3	Tmx2	1E-01	0.2
Gm47071	2E-04	0.3	Wdr27	4E-04	0.2
Sfi1	8E-03	0.3	Psmg2	1E-06	0.2
Atpaf2	6E-01	0.3	Kat6b	8E-14	0.2
Gtf2a2	9E-17	0.3	mt-Co1	7E-79	0.2
1810062O18Rik	9E-03	0.3	Gm14305	6E-01	0.2
Hmox1	4E-07	0.3	Avpi1	2E-13	0.2
Irx3	8E-03	0.3	Dbr1	2E-01	0.2
Rian	8E-09	0.3	Btaf1	3E-11	0.2
Map9	2E-03	0.3	Fzd3	5E-05	0.2
Zfp408	2E-01	0.3	Rbp1	5E-02	0.2
Rab40c	5E-04	0.3	5330438D12Rik	1E-04	0.2
Prkab1	3E-02	0.3	Rbak	4E-02	0.2
Tet3	6E-07	0.3	Slc30a9	2E-05	0.2
Ube2e1	3E-13	0.3	Pak1ip1	1E-11	0.2
Bccip	1E-13	0.3	Gin1	2E-03	0.2

Fibroblast

Control			*Notch2* $^{tm1.1Ecan}$		
Gene	*p*	FC	Gene	*p*	FC
Xpnpep1	2E-07	0.3	Siva1	2E-09	0.3
Hnrnpa3	4E-11	0.3	Sqle	6E-12	0.3
Vim	3E-40	0.3	Syngr2	4E-05	0.3
Sox6	2E-19	0.3	Tmed5	7E-09	0.3
Smchd1	2E-07	0.3	Foxd1	3E-07	0.3
3110056K07Rik	3E-02	0.3	Unk	1E-06	0.3
Memo1	4E-07	0.3	Cavin3	4E-28	0.3
Manf	3E-12	0.3	Ipo9	2E-06	0.3
Micall1	5E-02	0.3	Dedd	3E-01	0.3
Slc10a7	4E-06	0.3	Pdcd7	1E-05	0.3
Rarres2	7E-09	0.3	Arpin	3E-03	0.3
Kpnb1	2E-03	0.3	Npas2	1E-02	0.3
Smc3	1E-09	0.3	Il1r1	3E-12	0.3
Rnf217	3E-04	0.3	Tm9sf1	2E-06	0.3
Scd2	1E-09	0.3	Diaph1	2E-09	0.3
Wdr59	2E-02	0.3	Rubcn	4E-06	0.3
Iffo1	5E-03	0.3	Fbxo45	3E-02	0.3
Pxmp2	3E-02	0.3	Tacc1	1E-07	0.3
Mrpl18	2E-12	0.3	Vim	2E-54	0.3
Snapc3	7E-04	0.3	Setx	1E-06	0.3
Kras	5E-06	0.3	Slc27a3	2E-02	0.3
Retreg2	7E-04	0.3	Nme6	3E-03	0.3
Rpap1	2E-02	0.3	Cep83	2E-08	0.3
Echdc3	2E-01	0.3	Cav2	4E-06	0.3
Cryab	9E-17	0.3	Eftud2	3E-06	0.3
Grwd1	1E-02	0.3	Stk3	6E-12	0.3
Brwd3	2E-02	0.3	Daglb	6E-01	0.3
Ranbp1	1E-09	0.3	Calm2	1E-15	0.3
Pea15a	3E-06	0.3	Hnrnpa1	3E-19	0.3
Zdhhc18	4E-03	0.3	Zc2hc1a	1E-11	0.3
Klf10	9E-05	0.3	Rbbp4	7E-14	0.3
Satb1	8E-03	0.3	Tle5	5E-28	0.3
Hectd2	2E-01	0.3	Tprg	5E-03	0.3
Magee1	9E-04	0.3	Ubald2	2E-07	0.3
Atp1b3	1E-12	0.3	Scara3	5E-11	0.3
Tmem9b	4E-06	0.3	Ell	1E-03	0.3
Tmem243	1E-01	0.3	Palmd	4E-10	0.3
Rad51d	2E-01	0.3	Htt	4E-06	0.3
Mtbp	9E-03	0.3	Rfx1	6E-03	0.3
Pde10a	3E-03	0.3	Rbm10	4E-04	0.3
Ppip5k1	2E-03	0.3	Rpp21	2E-06	0.3
Tspan14	5E-02	0.3	AW554918	2E-04	0.3
Pfas	1E-02	0.3	Ddx23	2E-05	0.3
Ankrd29	9E-03	0.3	Klc1	1E-08	0.3
Clu	2E-07	0.3	Tuba4a	1E-02	0.3
Slx4ip	4E-02	0.3	Gja1	4E-13	0.3
Rbm15	3E-03	0.3	Tnfaip1	2E-05	0.3
Alg6	3E-03	0.3	Wnt5b	4E-05	0.3
Camk2n2	5E-04	0.3	Ccdc28b	2E-04	0.3
Zfp710	1E-03	0.3	Prkaca	8E-06	0.3
Atg3	6E-11	0.3	Tmem68	5E-03	0.3
Arl6ip6	2E-03	0.3	Gle1	3E-04	0.3
Oscp1	1E-02	0.3	Tcp11l1	2E-03	0.3
Omd	5E-19	0.3	Parp1	2E-06	0.3
Tmtc3	8E-02	0.3	Fig4	3E-01	0.3
Dapk1	2E-05	0.3	Gxylt1	2E-05	0.3
Fmr1	9E-11	0.3	Slc25a25	5E-04	0.3
D130040H23Rik	1E-01	0.3	Ctr9	1E-06	0.3
Nudt21	1E-08	0.3	Tent4a	5E-06	0.3
Kpna3	4E-07	0.3	Rgl2	3E-02	0.3
Ecm1	5E-18	0.3	Cotl1	2E-04	0.3
Mbnl1	6E-16	0.3	Cep97	6E-03	0.3
Tmem107	4E-04	0.3	Sf3a3	8E-08	0.3
Ranbp17	2E-02	0.3	S100a13	5E-22	0.3

Undefined

Control			*Notch2* $^{tm1.1Ecan}$		
Gene	*p*	FC	Gene	*p*	FC
Sec62	1E-13	0.2	Zdhhc16	2E-06	0.2
Ring1	6E-01	0.2	Pcbp4	4E-08	0.2
Oser1	1E-02	0.2	Pdzd2	5E-05	0.2
Iffo2	5E-01	0.2	Dctn4	3E-06	0.2
Sec22b	2E-01	0.2	Ccnk	1E-07	0.2
Mtif3	2E-01	0.2	Mrpl20	2E-01	0.2
Mipol1	9E-01	0.2	Sppl2b	6E-02	0.2
Ylpm1	3E-02	0.2	Oxsr1	9E-07	0.2
Magoh	4E-01	0.2	Styx	9E-04	0.2
Gm49267	2E-01	0.2	Pycrl	1E-05	0.2
Nudcd1	3E-01	0.2	Sgf29	9E-02	0.2
Usp13	9E-01	0.2	Ndufb11	4E-03	0.2
Emilin1	4E-02	0.2	Cst3	3E-04	0.2
Dph5	8E-01	0.2	Txn2	5E-03	0.2
Phactr2	4E-01	0.2	Cwc15	2E-04	0.2
Pgam1	1E+00	0.2	Usp20	1E-02	0.2
Trim41	6E-01	0.2	Rpl37	6E-19	0.2
Endog	2E-02	0.2	Slc9a3r2	3E-03	0.2
Fra10ac1	6E-01	0.2	Hsdl2	1E-01	0.2
Pgk1	3E-01	0.2	2810001G20Rik	6E-02	0.2
Mettl22	2E-02	0.2	Cul4b	5E-11	0.2
Ppa1	5E-01	0.2	Nop16	6E-05	0.2
Catsper2	9E-01	0.2	Alg14	4E-04	0.2
BC005537	5E-01	0.2	Iars	9E-04	0.2
Tubb4b	3E-03	0.2	Guk1	1E-04	0.2
Ndrg1	8E-01	0.2	Psmb7	3E-07	0.2
Nanp	8E-05	0.2	Adcy9	2E-01	0.2
Tbc1d23	3E-01	0.2	Slc25a17	8E-07	0.2
Eya3	4E-03	0.2	Xrcc6	2E-02	0.2
Ccdc62	6E-01	0.2	Ttc33	3E-03	0.2
Ubap1	7E-01	0.2	Ndufb10	2E-04	0.2
Ccl27a	2E-01	0.2	Aldh2	7E-04	0.2
Nphp3	3E-01	0.2	Psmb5	9E-03	0.2
Tma16	6E-01	0.2	Gprasp1	1E-05	0.2
Ciao2b	1E-02	0.2	Ssr4	3E-02	0.2
Dnajc3	7E-03	0.2	Cox14	2E-02	0.2
Ttc32	7E-01	0.2	Zscan26	5E-03	0.2
Cbx1	3E-02	0.2	Rpl18	4E-23	0.2
Hoxc9	1E-01	0.2	Bud31	3E-07	0.2
Uqcrq	2E-04	0.2	Dtwd1	4E-04	0.2
Fahd2a	6E-01	0.2	Tle4	2E-02	0.2
Pds5b	3E-03	0.2	Gmds	2E-01	0.2
Fndc3a	6E-01	0.2	Dek	7E-01	0.2
Zbtb8os	2E-01	0.2	Slx1b	8E-04	0.2
Wdr61	5E-01	0.2	Rpl38	6E-18	0.2
Alg14	6E-01	0.2	Bloc1s5	4E-03	0.2
Ercc4	1E-02	0.2	H2afj	1E-03	0.2
Tor3a	1E-01	0.2	Eif5	4E-01	0.2
Mafk	1E-01	0.2	Tspan31	6E-05	0.2
Tpra1	4E-01	0.2	Bccip	4E-03	0.2
Snta1	3E-01	0.2	Nr4a2	1E-02	0.2
Snrnp70	8E-01	0.2	Rabepk	2E-03	0.2
Rack1	5E-21	0.2	Ndufa9	3E-10	0.2
Nkap	2E-01	0.2	Nup85	5E-08	0.2
Gatad1	5E-05	0.2	Taz	6E-03	0.2
Trib1	2E-02	0.2	Itih2	3E-05	0.2
Csel1	2E-01	0.2	Scyl3	1E-03	0.2
Utp14a	1E-01	0.2	Fth1	5E-26	0.2
Usf2	4E-01	0.2	Rps10	7E-21	0.2
Rufy3	8E-01	0.2	Ptrh2	2E-05	0.2
Sltm	2E-01	0.2	Ncoa4	1E-04	0.2
Mcu	2E-01	0.2	Clpb	8E-02	0.2
Tmtc4	7E-02	0.2	Tcp11l2	5E-02	0.2
Txn2	7E-01	0.2	Ppp1r9a	3E-02	0.2

Articular/Synovial Fibroblast

Control			*Notch2* $^{tm1.1Ecan}$		
Gene	*p*	FC	Gene	*p*	FC
C2cd2	2E-02	0.4	Kmt2d	5E-02	0.4
Naprt	4E-02	0.4	Gdap2	1E-02	0.4
Mafb	7E-02	0.4	Cds2	3E-03	0.4
Cryz	3E-04	0.4	Rab11fip5	3E-03	0.4
Rock2	1E-21	0.4	Ppp1r18	2E-05	0.4
Nr1d1	1E-02	0.4	Sgcd	2E-10	0.4
Iffo1	7E-03	0.4	Frmd8	7E-02	0.4
Cpt1c	2E-04	0.4	Myof	3E-10	0.4
Ciao1	4E-04	0.4	Alkbh8	6E-02	0.4
Uaca	6E-03	0.4	Fech	5E-05	0.4
Grn	2E-26	0.4	Plekhg2	9E-04	0.4
Tmem126b	4E-05	0.4	Slc12a6	1E-02	0.4
Zfp995	2E-02	0.4	Prdx5	2E-17	0.4
Selenow	1E-29	0.4	Tvp23a	3E-01	0.4
Mcoln1	2E-03	0.4	Rassf2	1E-02	0.4
Rap2b	5E-06	0.4	Pqlc2	1E-01	0.4
Mfsd3	5E-02	0.4	Sgpp1	9E-04	0.4
Chst12	1E-08	0.4	Usp45	1E-03	0.4
Selenon	1E-05	0.4	Ddx56	6E-01	0.4
Pigs	1E-05	0.4	Wdpcp	8E-05	0.4
Cep162	3E-06	0.4	Pet100	8E-09	0.4
Myof	2E-16	0.4	Eya3	2E-04	0.4
Uap1	3E-05	0.4	Gm15283	2E-07	0.4
Por	7E-04	0.4	Gfra4	8E-02	0.4
Zmynd8	2E-16	0.4	Frrs1	6E-04	0.4
Cyfip1	3E-17	0.4	Tomm40l	1E-01	0.4
Lypla2	5E-03	0.4	Sspn	1E-06	0.4
Cav1	3E-15	0.4	Plekhm2	8E-07	0.4
Osbpl11	1E-04	0.4	Lrp10	3E-08	0.4
Slc29a3	7E-03	0.4	Prcc	5E-02	0.4
Col6a2	4E-25	0.4	Rreb1	2E-05	0.4
Rnd3	6E-15	0.4	Nfya	2E-02	0.4
Ltbp1	9E-03	0.4	Itpr2	2E-09	0.4
Pmm1	1E-07	0.4	Numbl	5E-03	0.4
Mpv17	2E-08	0.4	Sptan1	2E-14	0.4
Twsg1	3E-08	0.4	Stk3	2E-10	0.4
Gm12905	1E-01	0.4	Mrps18a	2E-08	0.4
Fam76a	4E-16	0.4	Col4a3bp	1E-05	0.4
Dbndd2	3E-02	0.4	Med23	2E-01	0.4
Rap2a	2E-06	0.4	Cst6	1E-03	0.4
Bivm	5E-02	0.4	Snx10	2E-03	0.4
Atp9b	2E-07	0.4	Ankrd10	4E-03	0.4
Nagk	1E-10	0.4	Hsd17b4	9E-08	0.4
Arhgap17	7E-09	0.4	Rsu1	4E-16	0.4
Abhd17c	3E-02	0.4	Lmtk2	8E-03	0.4
Fyco1	2E-06	0.4	Prelp	3E-03	0.4
Zfp715	1E-01	0.4	Smyd3	2E-10	0.4
Psmd8	3E-16	0.4	Klhl28	4E-02	0.4
Sept5	5E-03	0.4	Nlgn2	2E-03	0.4
Pnpla7	6E-03	0.4	Taf1	3E-06	0.4
Rabgap1l	3E-15	0.4	Selenow	7E-18	0.4
Ammecr1	1E-05	0.4	Ocrl	4E-03	0.4
Mfn2	6E-06	0.4	Gltp	9E-03	0.4
Ipo8	2E-04	0.4	Evc	2E-01	0.4
Cyld	8E-09	0.4	2410131K14Rik	2E-01	0.4
Cramp1l	2E-04	0.4	Snx30	2E-02	0.4
Siae	3E-03	0.4	Synrg	3E-03	0.4
Fndc4	3E-03	0.4	Fam219a	2E-03	0.4
Brsk1	3E-01	0.4	Abl2	3E-07	0.4
Ttc30b	4E-02	0.4	Pkn1	2E-04	0.4
Igf1r	4E-19	0.4	Pnp	1E-09	0.4
Klhl29	1E-07	0.4	Rdh10	4E-02	0.4
Ctsa	1E-18	0.4	Col5a1	2E-22	0.4
Parp3	1E-13	0.4	Glrx	1E-04	0.4

Limb Mesenchyme

Control			*Notch2*$^{tm1.1Ecan}$		
Gene	*p*	FC	Gene	*p*	FC
Tmem45a	4E-04	0.2	Ctso	8E-08	0.2
Tagln2	2E-17	0.2	Dlgap4	2E-12	0.2
Kars	8E-05	0.2	Map1s	2E-05	0.2
Plagl2	7E-01	0.2	Cryab	8E-13	0.2
Vps8	2E-03	0.2	Cdk9	4E-05	0.2
Stam2	2E-05	0.2	Prkx	3E-02	0.2
Pja1	1E-01	0.2	Fastkd2	6E-04	0.2
Mt2	6E-14	0.2	Podxl2	3E-10	0.2
Tarsl2	1E-02	0.2	Ice1	3E-06	0.2
Cyfip1	2E-09	0.2	Spns1	1E-08	0.2
Fibp	8E-07	0.2	Cpt2	4E-05	0.2
Cmc4	4E-03	0.2	Arf5	3E-31	0.2
Fdx1	3E-06	0.2	Eda2r	3E-02	0.2
Dipk1b	1E-05	0.2	Armc1	6E-08	0.2
Ydjc	8E-02	0.2	Maff	4E-04	0.2
Tmem205	4E-06	0.2	Ly6c1	3E-11	0.2
Tmem127	1E-04	0.2	Slc35f6	1E-03	0.2
Cnbd2	3E-02	0.2	Mrtfa	3E-10	0.2
Ptges2	8E-03	0.2	Abhd5	4E-07	0.2
Cttn	4E-11	0.2	Copz1	4E-09	0.2
Dcaf4	3E-03	0.2	Slc38a6	4E-03	0.2
Nbea	1E-10	0.2	Arl1	2E-24	0.2
Lrfn4	9E-02	0.2	Psme3	8E-08	0.2
Anapc16	1E-06	0.2	0610010K14Rik	1E-11	0.2
Slc2a8	7E-03	0.2	Bbs5	8E-04	0.2
Slx1b	3E-01	0.2	Scamp2	3E-11	0.2
Puf60	5E-07	0.2	Gm30025	3E-03	0.2
Cyb5r1	4E-12	0.2	Otub1	3E-10	0.2
Cald1	9E-26	0.2	Cul1	5E-10	0.2
Pdia5	4E-06	0.2	Dvl2	9E-04	0.2
Loxl2	2E-01	0.2	Anapc15	9E-04	0.2
Eif2ak2	1E-04	0.2	Sertad3	5E-09	0.2
Bckdhb	4E-02	0.2	Pycrl	4E-04	0.2
Wsb2	5E-07	0.2	Trim25	1E-02	0.2
Hipk3	2E-04	0.2	Anapc16	1E-12	0.2
Accs	6E-02	0.2	Trappc3	2E-12	0.2
Ptpn12	1E-03	0.2	Zfp277	1E-07	0.2
Evc	9E-03	0.2	Ttl	4E-03	0.2
Arhgap21	2E-06	0.2	Chchd5	4E-06	0.2
Rhoa	4E-33	0.2	Ppp1ca	9E-26	0.2
Ptdss2	6E-04	0.2	Tor2a	4E-07	0.2
Hars	4E-03	0.2	Prkd3	1E-05	0.2
Ap1s2	6E-03	0.2	Dcp1b	3E-04	0.2
Fez2	1E-03	0.2	Arpc5	1E-14	0.2
Timp2	4E-64	0.2	Ap1m1	2E-08	0.2
Itgb1bp1	7E-07	0.2	Evl	1E-08	0.2
Bag5	9E-07	0.2	Tdrd7	3E-05	0.2
Trim8	9E-03	0.2	Zxdc	2E-04	0.2
Prelid2	1E-04	0.2	Agtrap	2E-04	0.2
Pcgf2	2E-01	0.2	Rhbdd3	2E-05	0.2
Phb2	4E-10	0.2	Clns1a	2E-07	0.2
Cenpb	7E-12	0.2	Snapc2	2E-02	0.2
Eif4e2	4E-13	0.2	Gm49359	3E-03	0.2
Nfkb2	4E-04	0.2	2310011J03Rik	5E-06	0.2
Sft2d1	2E-04	0.2	Pafah1b2	5E-12	0.2
Nox4	7E-16	0.2	Eya4	7E-16	0.2
Insyn1	2E-02	0.2	Dtd2	1E-04	0.2
Npepl1	7E-05	0.2	Tmem127	3E-09	0.2
Mpzl1	1E-04	0.2	Ankrd54	1E-02	0.2
Cdc16	2E-07	0.2	Mrps7	4E-09	0.2
Irf3	5E-05	0.2	Sival	5E-14	0.2
Hivep3	1E-01	0.2	Rnd2	1E-03	0.2
Ptprf	2E-03	0.2	Crlf2	7E-11	0.2
Washc1	3E-05	0.2	Tgfbr3	1E-05	0.2

Chondrogenic

Control			*Notch2*$^{tm1.1Ecan}$		
Gene	*p*	FC	Gene	*p*	FC
Snhg5	8E-05	0.3	Kif13b	5E-04	0.2
Nars2	3E-02	0.3	Traf3ip2	3E-02	0.2
Asf1a	1E-06	0.3	Prdx3	2E-08	0.2
1110038B12Rik	4E-19	0.3	Saysd1	8E-11	0.2
Mast3	3E-01	0.3	Acsf2	1E-01	0.2
Slc25a26	1E-01	0.3	Oma1	4E-03	0.2
Gcsh	1E-09	0.3	Card19	2E-14	0.2
Csf2ra	9E-06	0.3	Cutc	2E-02	0.2
St3gal3	2E-04	0.3	Mettl3	2E-01	0.2
Phykpl	6E-05	0.3	Emc4	9E-10	0.2
Rhod	2E-04	0.3	Dnah7b	8E-03	0.2
Fnta	2E-13	0.3	Fam174a	1E-11	0.2
Usf2	5E-11	0.3	Gtf2a2	3E-14	0.2
Tvp23b	2E-12	0.3	Dtnb	9E-06	0.2
Gfm2	1E-02	0.3	Mb21d2	3E-02	0.2
Esf1	3E-11	0.2	Ints5	4E-01	0.2
Gart	6E-03	0.2	Rnf4	2E-03	0.2
Mir99ahg	6E-26	0.2	Hdac9	1E+00	0.2
Ttc27	4E-02	0.2	Nol10	2E-02	0.2
Ccdc58	2E-06	0.2	Med30	2E-07	0.2
Setbp1	4E-18	0.2	Erlec1	5E-16	0.2
Ric8b	3E-04	0.2	Hypk	1E-08	0.2
Sfmbt1	4E-05	0.2	Hbegf	5E-04	0.2
Dand5	7E-03	0.2	Shmt2	8E-08	0.2
B4galt7	4E-03	0.2	Gsto1	8E-05	0.2
Taf5l	8E-04	0.2	Nars2	5E-02	0.2
Ssbp1	8E-17	0.2	Dalrd3	4E-03	0.2
Spout1	2E-03	0.2	Rps27a	1E-74	0.2
Srprb	4E-05	0.2	Rabggtb	7E-07	0.2
Snhg6	5E-11	0.2	Lrrfip2	4E-07	0.2
Gm26749	2E-01	0.2	Zbtb22	2E-02	0.2
Sf3b6	1E-22	0.2	Prkce	9E-09	0.2
Wdr6	5E-05	0.2	Cdkl3	1E-03	0.2
Snrpd3	6E-22	0.2	Fam104a	2E-17	0.2
Ercc5	2E-03	0.2	Ssbp1	1E-15	0.2
BC049715	1E-01	0.2	Nprl3	9E-03	0.2
Tmem138	2E-03	0.2	Jmjd1c	1E-11	0.2
Crim1	2E-14	0.2	Ftx	2E-04	0.2
Shq1	2E-02	0.2	Tmem38b	7E-02	0.2
Lonrf1	7E-03	0.2	Exosc3	6E-07	0.2
Clpp	3E-12	0.2	Cbx1	3E-21	0.2
Vegfa	1E-07	0.2	Smim27	7E-09	0.2
Timmdc1	9E-07	0.2	Ccdc191	6E-03	0.2
Rabggtb	1E-06	0.2	Unc119	4E-04	0.2
Nfxl1	1E-02	0.2	Cog8	6E-05	0.2
Mrpl38	3E-05	0.2	Erich1	2E-06	0.2
Mras	4E-05	0.2	Snrpd3	1E-17	0.2
Coprs	2E-08	0.2	Srrd	2E-01	0.2
Id4	6E-12	0.2	Tpt1	2E-94	0.2
Eif1	1E-65	0.2	Ap4s1	7E-06	0.2
Snrnp70	1E-15	0.2	R3hdm1	4E-12	0.2
Lsm8	2E-11	0.2	C330018D20Rik	1E-01	0.2
Mosmo	2E-04	0.2	Kcnip4	6E-09	0.2
Swap70	5E-05	0.2	Isoc1	8E-10	0.2
Krtcap2	1E-30	0.2	Ufl1	2E-08	0.2
Zfyve21	3E-06	0.2	2310033P09Rik	2E-04	0.2
Prpsap1	2E-04	0.2	St3gal4	6E-09	0.2
Ankrd46	2E-01	0.2	B9d1	8E-04	0.2
Ice2	3E-03	0.2	Dexr	2E-09	0.2
Armc9	4E-03	0.2	Gcsh	5E-09	0.2
Fam229b	2E-05	0.2	Dbt	1E-03	0.2
Tomm7	9E-40	0.2	Shq1	4E-02	0.2
Uqcc1	1E-03	0.2	Kdm6b	2E-05	0.2
D10Wsu102e	2E-05	0.2	Txn2	8E-12	0.2

Fibroblast

Control			*Notch2*$^{tm1.1Ecan}$		
Gene	*p*	FC	Gene	*p*	FC
Ulk1	2E-03	0.3	Gla	4E-03	0.3
Foxd1	3E-06	0.3	Sik1	2E-03	0.3
Slc35e2	3E-01	0.3	Mbd1	9E-02	0.3
Casp12	1E-04	0.3	Mmab	2E-04	0.3
Ppp5c	2E-03	0.3	Prss23	3E-20	0.3
Cep250	3E-04	0.3	Bclaf3	2E-06	0.3
Fam126a	1E-03	0.3	Pop4	4E-06	0.3
F2r	1E-05	0.3	Prdm4	1E-03	0.3
Map2k3	2E-06	0.3	C1s1	8E-03	0.3
Pnp	2E-15	0.3	Chrd	2E-03	0.3
Mat2b	3E-05	0.3	Phlpp2	2E-06	0.3
H3f3a	3E-45	0.3	Atr	2E-05	0.3
Ybx1	8E-33	0.3	Eogt	2E-05	0.3
Ski	7E-16	0.3	Rtn3	1E-16	0.3
Sptan1	3E-14	0.3	Acadl	1E-14	0.3
Erg28	5E-07	0.3	Pdpn	1E-14	0.3
Tspan9	8E-04	0.3	Zfp28	3E-02	0.3
Lrrc49	2E-04	0.3	Pwwp2a	1E-03	0.3
Raver2	5E-02	0.3	Ppp1r7	5E-08	0.3
Smpd4	3E-02	0.3	Kazald1	3E-10	0.3
Flvcr1	2E-02	0.3	Pspc1	1E-07	0.3
Prelid2	5E-02	0.3	Fnip1	3E-14	0.3
Ift22	2E-03	0.3	Rprd1b	5E-07	0.3
Lrp1	3E-28	0.3	Cpd	4E-10	0.3
Snta1	5E-05	0.3	Arhgap24	2E-12	0.3
Trib1	3E-05	0.3	Kpna3	5E-06	0.3
Casp8	2E-02	0.3	Nrp1	2E-18	0.3
Arhgef10l	4E-04	0.3	Msrb2	3E-01	0.3
Metrnl	3E-09	0.3	Nectin3	2E-05	0.3
Pdhx	3E-02	0.3	Ganc	3E-04	0.3
Hnrnpd	1E-08	0.3	Cramp1l	8E-06	0.3
Dcp2	2E-04	0.3	Mtmr11	2E-03	0.3
B4galt1	1E-09	0.3	Hnrnph2	5E-09	0.3
Itpr2	2E-06	0.3	Ror1	1E-14	0.3
Akap6	9E-03	0.3	Iffo1	4E-04	0.3
Parp2	2E-04	0.3	Ppia	1E-43	0.3
Tbp	6E-03	0.3	G6pdx	4E-05	0.3
Atm	2E-03	0.3	Tbc1d4	1E-03	0.3
Atg4d	5E-02	0.3	Gipc1	2E-05	0.3
C330007P06Rik	1E-03	0.3	Gadd45b	1E-07	0.3
Prkar1b	1E-01	0.3	Capn2	1E-12	0.3
Snapc2	1E-02	0.3	Rpp30	3E-07	0.3
Cpt1a	6E-04	0.3	Ethe1	4E-05	0.3
Ndufaf2	4E-08	0.3	Syt11	3E-06	0.3
Wasf2	1E-16	0.3	Def6	1E-02	0.3
Pef1	1E-06	0.3	Rnf145	6E-03	0.3
Ctps2	2E-04	0.3	Aacs	1E-02	0.3
Slc35a1	8E-04	0.3	Sh3rf2	4E-04	0.3
Hk1	1E-04	0.3	Spsb2	5E-05	0.3
Arhgdib	7E-03	0.3	Cfap410	4E-01	0.3
Edem2	1E-04	0.3	Akip1	5E-05	0.3
2310057M21Rik	4E-03	0.3	Smarcal1	9E-05	0.3
Pcyt2	1E-04	0.3	Cbr2	2E-07	0.3
Srfbp1	1E-03	0.3	Stx2	1E-04	0.3
Apmap	2E-03	0.3	Sdc1	2E-08	0.3
Srebf2	1E-09	0.3	Mrpl51	8E-13	0.3
Suz12	4E-05	0.3	Mettl6	2E-03	0.3
Dock9	2E-06	0.3	Otud4	2E-04	0.3
Celf2	2E-20	0.3	Anapc7	5E-03	0.3
D830050J10Rik	1E-01	0.3	Rpe	2E-03	0.3
Lpin1	1E-01	0.3	Fbln7	3E-04	0.3
Ift74	5E-06	0.3	Fam98b	1E-08	0.3
Sgsm2	3E-04	0.3	Tssc4	3E-06	0.3
Stxbp3	6E-05	0.3	Med19	3E-08	0.3

Undefined

Control			*Notch2*$^{tm1.1Ecan}$		
Gene	*p*	FC	Gene	*p*	FC
Tob2	6E-01	0.2	Pdcl	1E-02	0.2
Vbp1	8E-01	0.2	Mrpl15	8E-08	0.2
Tiam2	6E-02	0.2	Snx17	7E-07	0.2
Gm26532	4E-01	0.2	Atp2b1	2E-01	0.2
Uchl3	3E-01	0.2	2310039H08Rik	2E-04	0.2
Rxylt1	8E-01	0.2	Mphosph9	2E-01	0.2
Ddx3y	7E-01	0.2	Tmem126a	2E-03	0.2
Snd1	5E-02	0.2	Srsf9	1E-04	0.2
Fem1a	5E-01	0.2	Taf1b	9E-04	0.2
Gls	3E-01	0.2	Wdr83os	6E-07	0.2
Rps6	2E-21	0.2	Ift172	9E-06	0.2
C1rl	8E-01	0.2	Lman1	2E-01	0.2
Llph	1E+00	0.2	Coa6	3E-06	0.2
Rpl36a	3E-23	0.2	Usp53	5E-01	0.2
Cdc42ep3	9E-01	0.2	Dynlt3	8E-09	0.2
Fgfr1op	2E-01	0.2	1110032A03Rik	2E-02	0.2
Iqgap1	6E-01	0.2	H6pd	4E-08	0.2
Tnrc6b	6E-04	0.2	Bbs7	5E-07	0.2
Rps24	6E-23	0.2	Fbxo9	2E-02	0.2
Fth1	2E-22	0.2	Gars	1E-01	0.2
2310039H08Rik	2E-01	0.2	Mien1	1E-05	0.2
Mrps18b	6E-01	0.2	Lncppara	8E-03	0.2
Dcp1a	8E-02	0.2	Ap1b1	2E-06	0.2
Srsf9	5E-01	0.2	Klhl15	2E-03	0.2
Alkbh7	5E-02	0.2	Vti1b	5E-08	0.2
Gm48678	4E-01	0.2	Gm16845	4E-01	0.2
Plpp2	9E-01	0.2	Mars	8E-06	0.2
Snhg3	5E-01	0.2	Rpl19	5E-22	0.2
Kdm6a	1E-01	0.2	Atg4c	2E-03	0.2
Naa30	9E-02	0.2	Ankrd44	1E-01	0.2
Ifit3	5E-01	0.2	Nudt14	4E-03	0.2
Tdg	3E-02	0.2	Cops4	4E-09	0.2
Smim11	2E-01	0.2	Fam126b	7E-03	0.2
Zfas1	5E-02	0.2	E030030I06Rik	8E-01	0.2
Dad1	8E-03	0.2	Pld3	2E-10	0.2
Ccdc58	5E-01	0.2	Timm8b	3E-04	0.2
Cox10	9E-01	0.2	Slc25a36	1E-02	0.2
Ivns1abp	2E-01	0.2	Smim20	3E-09	0.2
1810034E14Rik	7E-01	0.2	Slc35a4	9E-07	0.2
Hint1	2E-07	0.2	Fam3c	2E-11	0.2
Parg	3E-02	0.2	Jph1	8E-01	0.2
Gnasas1	4E-02	0.2	Cpsf3	2E-05	0.2
Sub1	3E-02	0.2	Letm2	4E-02	0.2
Zbtb10	8E-03	0.2	Lemd2	1E-02	0.2
Cpeb3	6E-01	0.2	Clpx	6E-05	0.2
Gfm2	3E-01	0.2	Slc25a12	7E-05	0.2
Uggt2	1E-01	0.2	Mrpl42	2E-05	0.2
Dclre1c	5E-07	0.2	Sec16a	1E-08	0.2
Mrps14	3E-01	0.2	Msl3l2	5E-03	0.2
Cry2	7E-01	0.2	Slc25a11	3E-07	0.2
Mvb12a	2E-05	0.2	Vamp8	2E-02	0.2
Vps16	1E-02	0.2	Fuom	1E-05	0.2
Tpmt	7E-01	0.2	Vps26c	7E-05	0.2
Fam98a	6E-01	0.2	Nop58	2E-05	0.2
Them4	8E-01	0.2	Mppe1	3E-03	0.2
Snhg12	9E-01	0.2	Ippk	4E-02	0.2
Rmnd1	2E-01	0.2	Bag4	4E-09	0.2
Churc1	2E-01	0.2	Alkbh6	2E-01	0.2
Abhd2	4E-04	0.2	Unkl	5E-03	0.2
Exosc5	3E-01	0.2	Znhit2	1E-05	0.2
Zfp672	3E-01	0.2	Pde3b	9E-01	0.2
Aen	6E-01	0.2	Vma21	2E-10	0.2
Tomm20	2E-01	0.2	Tspan3	6E-02	0.2
Sgf29	6E-01	0.2	Atp6v0e	7E-01	0.2

Articular/Synovial Fibroblast

Control			*Notch2*$^{tm1.1Ecan}$		
Gene	*p*	FC	Gene	*p*	FC
Afap1	1E-16	0.4	Cdyl	2E-04	0.4
Prkaca	2E-07	0.4	Asap1	1E-15	0.4
2310022B05Rik	3E-09	0.4	Sumf1	1E-02	0.4
Hyal2	1E-02	0.4	Adipor2	2E-02	0.4
Asap3	6E-03	0.4	Ggh	6E-11	0.4
Prxl2b	1E-05	0.4	Cetn4	2E-01	0.4
Zfp110	3E-04	0.4	Ube2e2	2E-13	0.4
Inpp1	4E-03	0.4	Snrpn	1E-01	0.4
Scn1b	5E-08	0.4	Fam160b2	5E-03	0.4
Prmt9	2E-02	0.4	Dcbld2	2E-02	0.4
Cyb5b	1E-05	0.4	Lats2	6E-11	0.4
Mindy1	3E-04	0.4	Afg1l	3E-03	0.4
Tango2	2E-04	0.4	Auh	1E-03	0.4
Zfp397	3E-06	0.4	Mrrf	3E-02	0.4
Fnip1	6E-19	0.4	Slc7a6	8E-01	0.4
Slc15a4	3E-02	0.4	Zbtb7a	7E-06	0.4
Mgmt	5E-08	0.4	Dnah7b	6E-02	0.4
Tshz2	6E-07	0.4	Dynlt1f	4E-02	0.4
Med24	7E-03	0.4	Diaph2	9E-10	0.4
Mroh1	6E-07	0.4	Lbh	4E-03	0.4
Pgap1	5E-03	0.4	Sipa1	4E-02	0.4
Il1rap	9E-06	0.4	Crip2	3E-08	0.4
Cc2d2a	3E-06	0.4	Pknox1	3E-02	0.4
Rps6ka3	1E-11	0.4	Mtf1	1E-02	0.4
Wdr37	3E-05	0.4	Slc38a10	1E-06	0.4
Fbxo31	3E-04	0.4	Fam122a	5E-04	0.4
Zfp442	2E-02	0.4	Unkl	2E-02	0.4
Sgcd	7E-16	0.4	Cd63	1E-39	0.4
Galnt1	4E-14	0.4	Gadd45g	4E-04	0.4
Bcl2	1E-02	0.4	Rtl8a	9E-06	0.4
Ggh	2E-16	0.4	Lmbrd1	9E-08	0.4
Nav2	1E-06	0.4	Tbc1d22b	2E-01	0.4
Cln8	1E-01	0.4	Ahdc1	2E-05	0.4
Klf10	3E-03	0.4	Pls3	3E-07	0.4
Esyt2	2E-12	0.4	Cc2d1a	1E-01	0.4
AI987944	5E-03	0.4	Adgra2	1E-01	0.4
Nsmaf	1E-03	0.4	Dnajc10	3E-09	0.4
Thra	3E-23	0.4	Pdia5	4E-05	0.4
Klf12	5E-10	0.4	Por	3E-04	0.4
Rmdn2	5E-02	0.4	Tecr	2E-14	0.4
Adipor2	3E-04	0.4	Zfp182	1E-03	0.4
Anxa4	1E-15	0.4	Katnal1	2E-01	0.4
Tmsb15b2	9E-03	0.4	Ttc21b	8E-02	0.4
Arl16	3E-04	0.4	Fut11	8E-03	0.4
Mindy2	7E-04	0.4	Tor3a	7E-05	0.4
Klf8	4E-01	0.4	Prmt9	4E-02	0.4
Kif3c	6E-03	0.4	Tcf12	1E-15	0.4
Smug1	7E-02	0.4	Plscr3	2E-06	0.4
Ikbkg	6E-04	0.4	Casp6	2E-03	0.4
Wipi1	1E-12	0.4	Lmo1	4E-06	0.4
AC149090.1	5E-08	0.4	Ndn	6E-08	0.4
Wrn	6E-05	0.4	Mroh1	1E-04	0.4
Ndst2	5E-02	0.4	Ncs1	2E-01	0.4
Smim1	5E-09	0.4	Leng1	2E-01	0.4
Rhoc	2E-22	0.4	Taok2	6E-03	0.4
Hsd17b4	5E-09	0.4	Lyn	1E-02	0.4
Rictor	7E-05	0.4	Emp1	3E-09	0.4
Tpp1	4E-11	0.4	Clcn5	5E-03	0.4
Fez2	3E-05	0.4	Rnf157	1E-01	0.4
Rpp21	6E-06	0.4	Armc7	8E-03	0.4
Retreg1	1E-03	0.4	Rbbp5	2E-01	0.4
Acad9	2E-03	0.4	Dvl3	3E-02	0.4
Slc8a1	3E-06	0.4	Ap1m1	1E-01	0.4
Tgfb1i1	3E-09	0.4	Wdr60	2E-02	0.4

Limb Mesenchyme					
Control			$Notch2^{tm1.1Ecan}$		
Gene	*p*	FC	Gene	*p*	FC
Gstm4	2E-02	0.2	Ifi27	2E-20	0.2
Gramd3	1E-02	0.2	Pttg1ip	2E-09	0.2
Kank2	3E-06	0.2	Cln6	5E-02	0.2
Trappc3	8E-07	0.2	2310057M21Rik	2E-02	0.2
Nhlrc3	2E-01	0.2	Sptlc2	7E-12	0.2
Elk1	1E-02	0.2	Gskip	2E-07	0.2
Trim28	6E-06	0.2	Acot9	5E-10	0.2
Acd	2E-03	0.2	Rbp1	6E-06	0.2
Rab4b	4E-07	0.2	Amotl2	3E-12	0.2
Aco1	3E-02	0.2	Polr3a	2E-02	0.2
Mrps36	2E-11	0.2	Man2a1	8E-08	0.2
Timm13	4E-17	0.2	Cyfip1	3E-13	0.2
Otub1	1E-09	0.2	Zbed3	8E-07	0.2
Cyp4f17	7E-02	0.2	Fam114a1	2E-17	0.2
Ankrd24	1E-02	0.2	Lgalsl	4E-06	0.2
Hmbs	7E-03	0.2	5430416N02Rik	2E-07	0.2
Ptov1	3E-16	0.2	Hscb	1E-04	0.2
Pycard	7E-03	0.2	Zfp251	6E-03	0.2
Usp21	9E-02	0.2	Ydjc	5E-07	0.2
Tnfrsf12a	2E-03	0.2	Cdca4	8E-02	0.2
Far1	2E-04	0.2	Wscd2	5E-05	0.2
Frrs1	6E-03	0.2	Rhbdf1	3E-07	0.2
Spryd3	1E-02	0.2	Sulf2	7E-09	0.2
Rps2	5E-69	0.2	Cdc42	5E-32	0.2
Ccdc93	2E-02	0.2	Galnt10	6E-11	0.2
Nit2	6E-05	0.2	Nln	5E-08	0.2
Mrps18a	8E-07	0.2	Taco1os	5E-02	0.2
Mtmr6	6E-05	0.2	Pbx2	2E-05	0.2
Dhodh	7E-02	0.2	Gmppb	2E-08	0.2
Dapk3	4E-04	0.2	Cldnd1	2E-13	0.2
Tpst2	2E-04	0.2	AU041133	2E-05	0.2
Mpnd	8E-08	0.2	Ybx3	7E-14	0.2
Nfix	2E-40	0.2	Mlxip	1E-04	0.2
Ppcs	9E-03	0.2	Ptprm	4E-11	0.2
Capn10	3E-02	0.2	Itgav	2E-10	0.2
Wasl	7E-09	0.2	Jdp2	8E-13	0.2
Gla	2E-01	0.2	Cnppd1	1E-03	0.2
Nav1	2E-04	0.2	Tmx3	8E-09	0.2
Lhx9	6E-02	0.2	Pbxip1	5E-11	0.2
Trp53rka	2E-03	0.2	Eif2ak2	9E-04	0.2
Capza2	3E-14	0.2	Txndc17	9E-20	0.2
Socs3	3E-03	0.2	Pabpc1	1E-26	0.2
St3gal2	1E-02	0.2	Uckl1	6E-05	0.2
Trappc2l	2E-12	0.2	Nudt16	2E-03	0.2
Shank1	2E-01	0.2	Acot2	3E-11	0.2
Apip	2E-06	0.2	Cdc42ep3	5E-06	0.2
Sybu	2E-02	0.2	Klhl2	6E-04	0.2
Tmed8	1E-02	0.2	Ddb1	1E-14	0.2
Ptpmt1	6E-08	0.2	Zfp809	1E-02	0.2
Cd44	7E-17	0.2	Rhoa	2E-29	0.2
Comtd1	5E-03	0.2	Rps4x	2E-50	0.2
Lgals8	1E-04	0.2	Golga7	2E-06	0.2
Notch1	8E-01	0.2	Cct4	2E-17	0.2
Thap11	4E-03	0.2	Serf2	5E-51	0.2
Nab2	1E-05	0.2	Fig4	1E-06	0.2
Tnfaip1	1E-05	0.2	Rap2b	8E-10	0.2
Exoc7	5E-03	0.2	Ptdss2	3E-05	0.2
Aimp2	2E-03	0.2	Nrbp1	4E-09	0.2
Rab35	3E-02	0.2	Ankrd39	1E-04	0.2
Neurl2	2E-01	0.2	Rce1	5E-07	0.2
Naxe	3E-09	0.2	Rpl10a	2E-48	0.2
Pbx1	4E-23	0.2	Fam110b	5E-09	0.2
Smim15	3E-04	0.2	Cept1	4E-08	0.2
Eif3l	6E-07	0.2	Rps6ka5	3E-04	0.2

Chondrogenic					
Control			$Notch2^{tm1.1Ecan}$		
Gene	*p*	FC	Gene	*p*	FC
Sertad2	2E-09	0.2	Alg14	3E-05	0.2
Zswim1	1E-01	0.2	Dnajb11	2E-11	0.2
Cep83os	1E-01	0.2	Fkbp4	1E-14	0.2
Nup210l	7E-04	0.2	Alkbh6	8E-03	0.2
Catsper2	4E-01	0.2	Ddx3y	3E-09	0.2
Senp2	7E-04	0.2	Prep	6E-03	0.2
Tmed1	1E-08	0.2	Rad1	2E-03	0.2
Med19	5E-06	0.2	Omd	7E-21	0.2
Nt5c2	3E-03	0.2	Krtcap2	2E-28	0.2
Nsun2	2E-05	0.2	Zfp512	6E-03	0.2
Lpin2	8E-04	0.2	Ggact	1E-03	0.2
Ankzf1	7E-01	0.2	Ggps1	3E-06	0.2
Ublcp1	1E-06	0.2	Xpnpep3	5E-03	0.2
Mar6	1E-05	0.2	Dhrs7	6E-10	0.2
Hist3h2a	3E-03	0.2	Pdia6	2E-25	0.2
Fkbp9	3E-18	0.2	Zcrb1	5E-18	0.2
5430416N02Rik	7E-05	0.2	S100pbp	1E-01	0.2
Strbp	7E-19	0.2	Rab4a	1E-02	0.2
Agap1	4E-13	0.2	Cebpg	1E-06	0.2
Nop58	1E-14	0.2	Prdm10	2E-03	0.2
Txn2	2E-15	0.2	Srpk2	1E-11	0.2
Blzf1	4E-04	0.2	Slc39a7	5E-13	0.2
Krr1	4E-04	0.2	Ubap1	8E-04	0.2
Ogg1	1E-01	0.2	Rora	1E-18	0.2
Eef1akmt1	8E-07	0.2	Gabarapl2	2E-23	0.2
Timm8a1	2E-06	0.2	Nkiras1	4E-05	0.2
Cars	4E-07	0.2	Adprm	4E-03	0.2
Rps8	1E-90	0.2	B3galt6	9E-07	0.2
Ift88	7E-06	0.2	Plcd1	5E-06	0.2
Crlf2	4E-08	0.2	St7	2E-07	0.2
2810002D19Rik	6E-02	0.2	Itfg2	3E-02	0.2
Fam185a	4E-03	0.2	Ndufa12	1E-18	0.2
Gm16740	2E-02	0.2	Utp25	2E-02	0.2
Ngdn	5E-04	0.2	Znrf3	2E-04	0.2
Rrp8	5E-05	0.2	Tiprl	2E-08	0.2
Fam71e1	1E-02	0.2	Zfp335os	4E-01	0.2
Ptk2	3E-07	0.2	Suclg2	1E-06	0.2
Gm50240	3E-02	0.2	Paip2b	7E-03	0.2
Zscan26	5E-05	0.2	Dvl1	1E-01	0.2
Polg2	2E-03	0.2	Sdf2l1	1E-08	0.2
4632427E13Rik	2E-04	0.2	Nob1	1E-04	0.2
Bcl2	2E-04	0.2	Firre	4E-02	0.2
Gemin5	6E-02	0.2	Strbp	4E-15	0.2
Fahd2a	5E-03	0.2	Abi2	3E-05	0.2
Hint1	2E-38	0.2	Tet3	5E-07	0.2
Cmpk1	9E-17	0.2	Sema5a	3E-05	0.2
Zbtb22	7E-05	0.2	Atf4	1E-22	0.2
Rgp1	5E-01	0.2	Sec16b	3E-03	0.2
Atp6v0e2	1E-03	0.2	Os9	9E-12	0.2
Gm48678	2E-01	0.2	Jpx	1E-03	0.2
Cisd2	1E-14	0.2	Aasdh	3E-02	0.2
Ripk2	6E-04	0.2	Eif3c	6E-30	0.2
Pcgf6	6E-03	0.2	Pura	9E-13	0.2
Dnajb11	2E-13	0.2	Zfp131	5E-06	0.2
Gm13056	1E-02	0.2	Phgdh	3E-19	0.2
Fndc3b	2E-32	0.2	2610020C07Rik	7E-03	0.2
Fbxl18	3E-01	0.2	Ppie	6E-04	0.2
Ywhaq	2E-19	0.2	Stim2	1E-02	0.2
Mtfr1l	1E-06	0.2	Pag1	2E-08	0.2
Cisd3	5E-07	0.2	Dad1	4E-29	0.2
Gm44686	4E-05	0.2	Cgrrf1	1E-04	0.2
Cdc42ep3	3E-14	0.2	Arl5b	5E-03	0.2
Rsbn1	9E-03	0.2	Mettl2	9E-02	0.2
Srsf10	1E-12	0.2	Ptcd3	6E-03	0.2

Fibroblast					
Control			$Notch2^{tm1.1Ecan}$		
Gene	*p*	FC	Gene	*p*	FC
Fam120c	4E-04	0.3	Sptbn1	7E-27	0.3
Galnt1	1E-10	0.3	Dusp8	2E-03	0.3
Pla2r1	2E-02	0.3	Antxr1	2E-11	0.3
Rhbdf1	2E-02	0.3	Ssx2ip	2E-04	0.3
Pcnx	2E-03	0.3	Epb41l2	4E-15	0.3
Btbd8	5E-02	0.3	Prkar2a	2E-05	0.3
Anxa2	3E-27	0.3	Depdc5	3E-06	0.3
Zmiz1	1E-11	0.3	Lrch4	8E-04	0.3
Mcm3ap	5E-03	0.3	Serp2	1E-03	0.3
Ankrd54	9E-02	0.3	Ski	8E-23	0.3
Glipr2	5E-04	0.3	Mfsd11	2E-04	0.3
Zcchc8	5E-04	0.3	Ift57	1E-05	0.3
5730522E02Rik	4E-01	0.3	Pxn	1E-03	0.3
Pcyox1l	3E-02	0.3	Dzip1	5E-07	0.3
Zc2hc1a	9E-09	0.3	Dbn1	1E-08	0.3
Uhrf2	4E-04	0.3	Nab2	1E-07	0.3
Fam124a	4E-03	0.3	Cbfb	9E-12	0.3
Vps36	2E-06	0.3	Cnot6l	2E-08	0.3
Brd9	3E-08	0.3	Zfp568	2E-06	0.3
Klhl28	5E-01	0.3	Spop	5E-12	0.3
Myef2	5E-05	0.3	Col6a1	4E-26	0.3
Snrpb	5E-14	0.3	Numa1	2E-10	0.3
Pigl	2E-02	0.3	Vps13b	2E-13	0.3
Cfl2	6E-17	0.3	Zfp975	3E-03	0.3
Paics	1E-10	0.3	Lrrc57	3E-02	0.3
Smarca4	7E-13	0.3	Tmem65	2E-10	0.3
Piga	2E-03	0.3	Prelid2	2E-03	0.3
Lsm5	1E-06	0.3	Polr3c	6E-04	0.3
Pih1d1	1E-02	0.3	Nudt13	1E-03	0.3
Gm28875	9E-03	0.3	Ak6	3E-08	0.3
Magohb	1E-06	0.3	Cib2	3E-04	0.3
Pcbp4	2E-05	0.3	Actb	5E-32	0.3
Zfp84	1E-01	0.3	Mfge8	2E-18	0.3
Exo5	7E-01	0.3	0610010K14Rik	4E-09	0.3
Cyb5b	1E-05	0.3	Erlin1	1E-06	0.3
Lpar6	6E-03	0.3	Endod1	5E-07	0.3
Sox4	4E-06	0.3	Rfc1	2E-08	0.3
Wdcp	2E-01	0.3	Ahr	2E-07	0.3
Gucy1a2	2E-02	0.3	Ppm1l	3E-02	0.3
BC052040	2E-05	0.3	Ppox	3E-02	0.3
Rnf167	5E-04	0.3	Ly96	3E-04	0.3
Cnep1r1	1E-07	0.3	Ctif	2E-07	0.3
Col6a1	1E-18	0.3	Slc35a5	8E-03	0.3
Fxyd1	6E-04	0.3	5930430L01Rik	6E-05	0.3
Nphp1	3E-03	0.3	Stat1	7E-04	0.3
Rapgef4	6E-05	0.3	Pithd1	1E-03	0.3
Opa3	6E-06	0.3	Rassf1	3E-06	0.3
Vps13a	1E-03	0.3	Tpst1	4E-09	0.3
Hp1bp3	3E-09	0.3	Gmeb1	3E-04	0.3
Pmvk	5E-05	0.3	Thap11	2E-01	0.3
Dnlz	4E-05	0.3	Erg28	2E-12	0.3
Nploc4	2E-03	0.3	Pole3	3E-07	0.3
Esyt1	4E-04	0.3	Ncor2	3E-09	0.3
Plod2	5E-12	0.3	Msra	1E-05	0.3
Dapp1	1E-03	0.3	Gulp1	9E-08	0.3
Als2	3E-02	0.3	Pdgfrb	2E-09	0.3
Cers4	3E-03	0.3	Prickle2	2E-08	0.3
Mettl14	4E-02	0.3	Rbck1	8E-05	0.3
Tsen34	1E-09	0.3	Ptgis	2E-09	0.3
Tjp1	2E-04	0.3	Cand2	1E-03	0.3
B4galt4	1E-01	0.3	Wdfy3	7E-09	0.3
Med12l	5E-02	0.3	Tln1	1E-21	0.3
Ahnak	3E-25	0.3	Crim1	3E-11	0.3
Ext1	4E-13	0.3	Nsmce2	3E-11	0.3

Undefined					
Control			$Notch2^{tm1.1Ecan}$		
Gene	*p*	FC	Gene	*p*	FC
Gadd45b	2E-03	0.2	Anapc10	6E-07	0.2
Rbm48	5E-01	0.2	Exosc8	7E-06	0.2
Apol8	8E-01	0.2	Triqk	3E-05	0.2
Fbxl18	1E+00	0.2	Ftsj1	9E-04	0.2
Snrpd3	4E-01	0.2	Usf2	1E-08	0.2
Itpr2	2E-01	0.2	Zfp523	8E-02	0.2
Nfkbil1	4E-02	0.2	Lcmt1	9E-06	0.2
Gnl3	5E-01	0.2	Spg20	2E-05	0.2
Ndufab1	3E-01	0.2	Trdmt1	6E-03	0.2
Slc49a4	5E-01	0.2	Ergic3	4E-03	0.2
Slc25a53	2E-01	0.2	Psma1	9E-06	0.2
Cbwd1	6E-02	0.2	Malat1	2E-08	0.2
Mar6	6E-01	0.2	Cat	6E-12	0.2
Lsm1	3E-01	0.2	Laptm4a	8E-08	0.2
Ric1	5E-03	0.2	Pak3	3E-04	0.2
Ubqln4	5E-01	0.2	Leprotl1	3E-06	0.2
Caap1	2E-01	0.2	Tmem107	6E-05	0.2
Pfdn4	8E-02	0.2	Flad1	6E-03	0.2
Bbs2	9E-02	0.2	Slu7	4E-04	0.2
Nop56	5E-01	0.2	Cfap97	3E-04	0.2
Ddx49	5E-01	0.2	Rps17	5E-12	0.2
Nfat5	2E-02	0.2	Iffo2	4E-04	0.2
Itih2	5E-01	0.2	Fbxl6	2E-06	0.2
Zbtb7b	4E-01	0.2	Yrdc	6E-02	0.2
Trappc2	8E-01	0.2	Prdm10	2E-03	0.2
Cep85	1E+00	0.2	Itpr3	3E-02	0.2
Tgif1	4E-01	0.2	Gstp1	4E-03	0.2
Cdr2	1E-02	0.2	Hnrnpk	2E-03	0.2
Dcaf13	2E-01	0.2	Rpl39	6E-20	0.2
Rnf126	3E-02	0.2	Gsk3a	1E-03	0.2
Kctd3	2E-01	0.2	Mterf4	9E-04	0.2
Zfp592	1E-02	0.2	Chchd6	1E-03	0.2
1110032A03Rik	4E-01	0.2	Gabpb2	2E-06	0.2
Mar5	9E-01	0.2	G6pc3	3E-02	0.2
Spata5	7E-01	0.2	AI506816	2E-16	0.2
Limk2	8E-02	0.2	Gtf2f2	4E-03	0.2
Ftx	1E-01	0.2	Ogfod1	2E-04	0.2
Afmid	9E-02	0.2	Gls	3E-03	0.2
Klhl22	1E-04	0.2	Osbpl2	2E-06	0.2
2010320M18Rik	3E-01	0.2	Zfp706	1E-01	0.2
Ccdc107	8E-01	0.2	4930556M19Rik	9E-01	0.2
Toe1	7E-01	0.2	Trim39	1E-03	0.2
Pfdn1	1E-01	0.2	Nfs1	3E-02	0.2
Srp9	1E-02	0.2	Cd320	1E-03	0.2
Rasl11a	1E-01	0.2	Naglu	2E-15	0.2
Trabd2b	9E-01	0.2	0610009B22Rik	1E-04	0.2
Prdx6	9E-01	0.2	Maip1	5E-07	0.2
Mical3	3E-04	0.2	Hspa4l	1E-07	0.2
Mrpl12	4E-03	0.2	Polr3k	2E-05	0.2
Fam229b	7E-01	0.2	Washc1	2E-10	0.2
1110004F10Rik	2E-01	0.2	Zmat5	8E-07	0.2
A930005H10Rik	6E-01	0.2	Mkrn1	4E-05	0.2
Rps21	1E-40	0.2	Hint3	3E-02	0.2
Bcs1l	5E-01	0.2	Polr2d	2E-05	0.2
Drap1	3E-01	0.2	Vars	9E-08	0.2
Zfp654	9E-01	0.2	Nol10	1E-02	0.2
Usp54	1E+00	0.2	Amacr	6E-03	0.2
Utp18	8E-02	0.2	Grpel2	5E-05	0.2
Phkg2	4E-01	0.2	Phactr1	2E-03	0.2
Cops7a	8E-01	0.2	Fiz1	4E-04	0.2
Cep290	5E-01	0.2	Zfp950	7E-07	0.2
Nars	3E-01	0.2	Capn6	4E-02	0.2
Zfp1	5E-02	0.2	Zfp952	2E-03	0.2
Erf	1E-01	0.2	Dmac2l	2E-01	0.2

Articular/Synovial Fibroblast					
Control			$Notch2^{tm1.1Ecan}$		
Gene	*p*	FC	Gene	*p*	FC
Wdr47	1E-02	0.4	Scn1b	3E-08	0.4
Rbm41	2E-02	0.4	Flot1	1E-04	0.4
Dcaf4	1E-02	0.4	Inpp5a	4E-05	0.4
Fbxo25	6E-06	0.4	Peli1	1E-02	0.4
Wdr24	2E-02	0.4	Asap3	2E-02	0.4
Uhmk1	9E-04	0.4	Pot1a	2E-01	0.4
Tbck	7E-08	0.4	Vps50	2E-03	0.4
Snx2	2E-11	0.4	Tprkb	2E-04	0.4
Fam118a	4E-01	0.4	Rnpepl1	5E-02	0.4
Srfbp1	2E-03	0.4	Aldh3a2	6E-02	0.4
Washc1	6E-05	0.4	Wrn	6E-05	0.4
Gpd2	5E-05	0.4	Avil	2E-01	0.4
Git2	7E-05	0.4	Pip4p1	3E-04	0.4
Emc9	3E-03	0.4	Atp8b2	5E-02	0.4
Col28a1	8E-07	0.4	Itgb5	3E-13	0.4
Dhx32	6E-04	0.4	Usp33	3E-04	0.4
Tmem37	1E-01	0.4	5730409E04Rik	1E-02	0.4
Vps52	1E-03	0.4	Abi1	1E-08	0.4
Tbc1d32	5E-04	0.4	Pi4ka	2E-07	0.4
Fam13c	2E-08	0.4	Rora	2E-16	0.4
Unc119b	5E-02	0.4	Ist1	1E-04	0.4
Ap2a2	1E-12	0.4	Zfp30	2E-02	0.4
Cd99l2	1E-06	0.4	Cant1	7E-03	0.4
Nicn1	3E-03	0.4	Washc2	1E-08	0.4
Ephb2	9E-04	0.4	Wipf2	1E-01	0.4
Cbr1	2E-09	0.4	Erap1	6E-01	0.4
Atp13a2	2E-01	0.4	Gpr137b	1E-02	0.4
Trip10	2E-03	0.4	Fbxo28	1E-02	0.4
Cat	8E-09	0.4	Wipi1	7E-11	0.4
Arhgef12	2E-16	0.4	Ddr2	1E-11	0.4
Tmem168	2E-03	0.4	Hdac5	6E-03	0.4
Gspt2	6E-02	0.4	Hs1bp3	1E-02	0.4
Zfyve1	5E-05	0.4	Bcl2	4E-02	0.4
Gjc1	1E-05	0.4	Zcchc3	4E-01	0.4
Add1	1E-11	0.4	Kif3b	4E-02	0.4
Tango6	1E-02	0.4	Siae	2E-03	0.4
Ccn3	1E-13	0.4	Ski	4E-13	0.4
Snx18	8E-09	0.4	Xab2	8E-02	0.4
Abcb10	1E-02	0.4	Ankib1	7E-07	0.4
Gosr1	5E-04	0.4	Sertad1	1E-01	0.4
Tle2	9E-02	0.4	Egr3	6E-02	0.4
Hexa	3E-15	0.4	Lgals8	7E-05	0.4
Gli3	4E-18	0.4	Ankrd52	2E-02	0.3
Ssbp2	1E-06	0.4	Elmo2	1E-02	0.3
Acsl3	4E-05	0.4	C330007P06Rik	1E-04	0.3
Rassf2	2E-05	0.4	Tyk2	2E-01	0.3
Ankrd39	3E-02	0.4	Nab2	3E-04	0.3
Mnt	2E-04	0.4	Srf	2E-03	0.3
Sh3gl1	8E-09	0.4	Trp53bp2	4E-05	0.3
Atg4b	4E-04	0.4	Rab5b	1E-03	0.3
Mcc	4E-16	0.4	Fam149a	1E-02	0.3
Myo7a	3E-02	0.4	Pank3	6E-04	0.3
Syne3	2E-04	0.4	Gnpda2	5E-03	0.3
Rragb	8E-03	0.4	Tbck	3E-03	0.3
Llgl1	2E-02	0.4	Mgat1	1E-03	0.3
Ankrd12	3E-20	0.4	Ermp1	1E-01	0.3
Mmab	4E-04	0.4	Sirt5	3E-02	0.3
Ier5l	6E-04	0.4	Rictor	2E-04	0.3
Faxc	4E-02	0.4	Tsc1	1E-04	0.3
Neu1	1E-05	0.4	Tdp1	3E-02	0.3
Pnpt1	5E-04	0.4	Rab10os	1E-02	0.3
Rsu1	5E-15	0.4	Arhgap17	1E-07	0.3
Orai3	4E-03	0.4	Stat5b	2E-04	0.3
Mapk7	6E-03	0.4	Nsun6	4E-02	0.3

Limb Mesenchyme

Control			*Notch2*$^{tm1.1Ecan}$		
Gene	*p*	FC	Gene	*p*	FC
Prrx1	1E-20	0.2	Xbp1	4E-11	0.2
Serf2	1E-61	0.2	Vapb	4E-11	0.2
Fam118a	5E-01	0.2	Zfp46	2E-05	0.2
Akap13	2E-16	0.2	Tns1	6E-05	0.2
Stat6	6E-05	0.2	Acadl	3E-12	0.2
Ddx54	2E-05	0.2	S100a16	3E-14	0.2
Ddx19a	1E-02	0.2	Cenpv	6E-05	0.2
Dmwd	3E-04	0.2	Mrpl21	8E-09	0.2
Arl15	9E-11	0.2	Ajuba	2E-03	0.2
Med10	1E-07	0.2	Scpep1	7E-19	0.2
Foxn3	1E-22	0.2	Pip4k2b	1E-03	0.2
Nmd3	2E-03	0.2	Tor1b	4E-07	0.2
Creb3	2E-04	0.2	Axl	7E-13	0.2
Rab5c	7E-08	0.2	Tmem11	1E-08	0.2
Aldh3b1	3E-02	0.2	Bad	7E-11	0.2
Pex11b	6E-03	0.2	Aldoa	6E-25	0.2
Ids	4E-02	0.2	Mrpl54	2E-16	0.2
Ssr4	4E-30	0.2	Gas8	3E-04	0.2
Ing5	4E-02	0.2	Polr1e	4E-03	0.2
Erbb2	9E-03	0.2	Rap1b	5E-19	0.2
Mir100hg	1E-05	0.2	Lpgat1	9E-14	0.2
Zmat3	1E-05	0.2	Ssna1	3E-08	0.2
H2-K1	7E-13	0.2	Ak5	5E-11	0.2
Fer	2E-08	0.2	Washc2	7E-11	0.2
Hs6st1	4E-02	0.2	AI987944	5E-03	0.2
Dhx38	4E-02	0.2	Brf2	2E-03	0.2
Ino80b	1E-02	0.2	Itgb1bp1	6E-10	0.2
Tmem192	1E-05	0.2	Ppp2r3d	3E-03	0.2
Sgcb	8E-05	0.2	Tesk1	6E-07	0.2
Mthfd2l	1E-04	0.2	Lsm10	7E-09	0.2
Top1mt	5E-02	0.2	Sirt1	2E-03	0.2
Lnx2	1E+00	0.2	Cenpb	3E-12	0.2
Bin1	4E-09	0.2	Capzb	7E-17	0.2
Gmeb2	9E-03	0.2	Mbd6	5E-03	0.2
Kctd10	4E-03	0.2	Zfp329	9E-04	0.2
Bahcc1	1E-01	0.2	Arfip2	3E-07	0.2
Ncdn	3E-01	0.2	Ddx19a	1E-03	0.2
Mtmr9	2E-02	0.2	Ick	2E-06	0.2
Bad	2E-07	0.2	Noc2l	3E-08	0.2
Cct4	4E-14	0.2	Azi2	7E-05	0.2
Scn1b	3E-09	0.2	Pdcd2	3E-07	0.2
Psmd9	6E-05	0.2	Fzd7	1E-03	0.2
Bcl7c	5E-09	0.2	Lrp6	7E-12	0.2
Retreg3	1E-02	0.2	Usp25	7E-09	0.2
Tead3	1E-02	0.2	Aggf1	3E-05	0.2
Map1lc3a	2E-19	0.2	Anapc2	2E-08	0.2
Cnpy2	2E-26	0.2	2610001J05Rik	3E-10	0.2
Ctdnep1	4E-07	0.2	Cybc1	1E-05	0.2
Orc4	1E-03	0.2	Capg	2E-17	0.2
Fbxl7	3E-16	0.2	Mea1	7E-09	0.2
Pip4k2b	2E-02	0.2	Stk25	5E-06	0.2
C1qtnf6	7E-05	0.2	Foxn3	6E-20	0.2
Wrb	1E-01	0.2	Far1	6E-08	0.2
Ankrd39	4E-03	0.2	Lrrc47	3E-03	0.2
Pias4	6E-02	0.2	Gla	2E-04	0.2
Gm10373	1E-01	0.2	Rps15a	1E-57	0.2
Lztfl1	2E-08	0.2	Pcnx3	4E-05	0.2
Ufc1	2E-13	0.2	Pde7a	2E-06	0.2
Fosb	3E-17	0.2	Thumpd2	8E-03	0.2
Usp46	8E-04	0.2	Aarsd1	2E-07	0.2
Adra1b	2E-01	0.2	Tacc1	4E-08	0.2
Cul7	4E-04	0.2	C1qtnf12	7E-06	0.2
Surf1	8E-05	0.2	Ctif	3E-08	0.2
Ptdss1	2E-02	0.2	Lmx1b	4E-03	0.2

Chondrogenic

Control			*Notch2*$^{tm1.1Ecan}$		
Gene	*p*	FC	Gene	*p*	FC
Trappc6a	2E-10	0.2	Impa2	6E-04	0.2
Slc35e1	2E-02	0.2	Fancl	2E-02	0.2
Slc2a13	1E-01	0.2	Apbb2	1E-20	0.2
Os9	7E-11	0.2	Yipf4	1E-14	0.2
Ccdc77	5E-03	0.2	Triqk	1E-04	0.2
Nol7	1E-14	0.2	Nol3	3E-03	0.2
1110004F10Rik	8E-22	0.2	Tle4	4E-01	0.2
Bet1	7E-08	0.2	Kti12	3E-01	0.2
Clasp2	2E-04	0.2	Rab11fip3	3E-06	0.2
Snhg20	1E-03	0.2	Tmem138	1E-01	0.2
Rbmx	2E-02	0.2	Mtrf1	1E-01	0.2
Smim19	1E-11	0.2	Wwtr1	2E-13	0.2
Ptrh2	6E-07	0.2	Tusc2	6E-03	0.2
Dnajc12	1E-02	0.2	Pcgf6	3E-02	0.2
Brix1	2E-07	0.2	Slc35b1	1E-08	0.2
Dmac2l	2E-02	0.2	Dap	9E-25	0.2
Pithd1	4E-03	0.2	Utp20	5E-03	0.2
Sub1	9E-34	0.2	Rexo2	2E-23	0.2
Arhgef5	1E-03	0.2	Polr3e	2E-01	0.2
Stard10	6E-06	0.2	Atp1b3	1E-09	0.2
Pex13	5E-07	0.2	Rev1	2E-05	0.2
Polr3a	1E+00	0.2	Cox16	2E-14	0.2
Ptar1	2E-03	0.2	Nudcd1	4E-04	0.2
Btg2	4E-19	0.2	Acbd5	3E-05	0.2
Idua	2E-03	0.2	Clk1	2E-09	0.2
Mpzl1	5E-04	0.2	Setdb1	1E-02	0.2
Uso1	3E-08	0.2	Gtf2a1	6E-05	0.2
Ecsit	3E-03	0.2	Cox17	2E-15	0.2
Ubap1	1E-02	0.2	Arhgef6	3E-02	0.2
Usp37	5E-06	0.2	Diablo	1E-03	0.2
Uvssa	1E-02	0.2	Park7	1E-29	0.2
B3glct	7E-06	0.2	Alg5	3E-10	0.2
Ctxn1	4E-02	0.2	Rars2	2E-04	0.2
H2afj	2E-15	0.2	Mtm1	8E-02	0.2
Zfp24	2E-06	0.2	B930095G15Rik	2E-02	0.2
Lman1	2E-27	0.2	Eef1e1	8E-07	0.2
Wdr46	1E-02	0.2	Phpt1	6E-04	0.2
Tmco1	1E-15	0.2	Katna1	8E-06	0.2
Plcl2	3E-03	0.2	Pgrmc2	6E-17	0.2
Cers2	5E-08	0.2	Rab33b	2E-03	0.2
Homer1	6E-03	0.2	Btbd1	1E-09	0.2
Ppie	1E-06	0.2	Cdc14b	2E-03	0.2
Tmem42	1E-05	0.2	Fndc3b	9E-19	0.2
Akap7	2E-02	0.2	Rapgef4	2E-01	0.2
Sdf2l1	7E-08	0.2	Mark3	2E-09	0.2
Igf2bp2	1E-08	0.2	Zbtb24	3E-02	0.2
Hnrnph3	9E-09	0.2	Hnrnph3	1E-10	0.2
Pi4k2a	2E-04	0.2	Ccdc127	6E-06	0.2
Tubb4b	1E-25	0.2	Cog6	1E-02	0.2
Triqk	5E-04	0.2	Rpap3	3E-04	0.2
Lmo7	1E-14	0.2	P3h1	2E-05	0.2
Nup43	4E-03	0.2	Eif2s3y	7E-05	0.2
Pex3	3E-04	0.2	Tfb2m	4E-02	0.2
Farsb	2E-05	0.2	Appbp2	2E-09	0.2
Erg	3E-08	0.2	Gemin2	1E-01	0.2
Mycbp	1E-05	0.2	Kansl1l	2E-05	0.2
Rsph9	2E-05	0.2	Zmym5	2E-03	0.2
Sema3e	1E-03	0.2	Lrrc20	4E-01	0.2
Zbtb8os	7E-08	0.2	Smim4	5E-07	0.2
C1qtnf6	3E-03	0.2	Aopep	2E-18	0.2
Rpl9-ps6	1E-02	0.2	Oxld1	5E-02	0.2
AW554918	2E-04	0.2	Nle1	1E+00	0.2
Alg5	1E-08	0.2	Gucd1	1E+00	0.2
Cox14	1E-22	0.2	Slirp	2E-11	0.2

Fibroblast

Control			*Notch2*$^{tm1.1Ecan}$		
Gene	*p*	FC	Gene	*p*	FC
Nsun6	6E-03	0.3	Erc1	4E-08	0.3
Slc26a11	9E-02	0.3	Coq6	1E-02	0.3
Ttll1	6E-03	0.3	Smarcd2	5E-03	0.3
Tsen15	4E-04	0.3	Hist1h4i	1E-04	0.3
Kpna4	3E-13	0.3	Tvp23a	2E-04	0.3
Fbxl14	6E-02	0.3	Man1a2	6E-13	0.3
Acly	4E-07	0.3	Fmnl3	2E-06	0.3
Arl6ip5	8E-10	0.3	Uspl1	5E-04	0.3
Hspa14	1E-03	0.3	Adamts9	2E-03	0.3
Arsj	2E-04	0.3	Tm7sf2	8E-03	0.3
Tank	2E-05	0.3	Zbtb41	9E-07	0.3
Nab2	1E-04	0.3	Myl12a	3E-29	0.3
Itgb1	2E-34	0.3	Prps2	3E-05	0.3
Mrpl14	4E-11	0.3	Sash1	5E-14	0.3
Gfm1	6E-01	0.3	Myadm	2E-06	0.3
Pdzd2	2E-04	0.3	Cacng7	5E-03	0.3
Six1	1E-02	0.3	Sf3a1	2E-06	0.3
Tbx5	3E-04	0.3	Ezr	7E-07	0.3
Tmem121	7E-02	0.3	C330007P06Rik	9E-07	0.3
Flna	1E-10	0.3	Fam210a	2E-02	0.3
Tmbim1	1E-05	0.3	Ube2z	3E-06	0.3
Map3k1	3E-01	0.3	Nxf1	1E-04	0.3
Pfkfb4	8E-02	0.3	2810006K23Rik	1E-05	0.3
Ncs1	3E-03	0.3	Hnrnpdl	4E-13	0.3
Zfp438	2E-02	0.3	Dyrk1b	4E-03	0.3
Zfp653	5E-02	0.3	Ecm1	4E-20	0.3
Fundc2	8E-14	0.3	Plec	3E-12	0.3
Dhcr7	2E-02	0.3	Amer1	2E-04	0.3
2310015A10Rik	1E-01	0.3	Syncrip	2E-14	0.3
Ftsj1	7E-02	0.3	Hyal2	5E-03	0.3
Mical1	5E-04	0.3	Dot1l	4E-05	0.3
Zpbp	1E-01	0.3	Shisa4	2E-08	0.3
Ahdc1	2E-04	0.3	Arhgef10l	4E-07	0.3
Tpp2	8E-12	0.3	Flna	7E-19	0.3
Polr2l	2E-06	0.3	Hmbox1	5E-04	0.3
Trp53bp2	2E-02	0.3	Xpo4	4E-05	0.3
E2f5	1E-01	0.3	Filip1l	3E-04	0.3
Enpp1	7E-03	0.3	Nacc1	1E-03	0.3
Heatr1	8E-03	0.3	Smurf2	3E-16	0.3
Chid1	2E-04	0.3	Daxx	2E-02	0.3
Taok2	7E-04	0.3	Fnbp4	2E-05	0.3
Cdyl	2E-04	0.3	Stard7	3E-03	0.3
Sh3rf3	1E-08	0.3	Dyrk2	1E-01	0.3
Rock1	7E-16	0.3	Fbxw11	4E-08	0.3
Pawr	1E-05	0.3	Ddx55	4E-03	0.3
Plekhb2	4E-02	0.3	Nav1	7E-09	0.3
Phactr4	1E-05	0.3	Cdk10	8E-03	0.3
Myo5a	4E-10	0.3	Pnp	1E-18	0.3
Slc10a3	3E-01	0.3	Dgcr8	8E-05	0.3
Armh4	5E-11	0.3	Tars2	6E-06	0.3
Flii	8E-08	0.3	Macf1	4E-19	0.3
Cxxc5	2E-09	0.3	Tmem18	8E-04	0.3
Slc35d2	2E-03	0.3	Whamm	7E-02	0.3
Gatad2a	5E-09	0.3	Calm3	2E-09	0.3
Gm16759	2E-01	0.3	Pitpnm2	4E-02	0.3
Smc5	3E-03	0.3	Clp1	1E-03	0.3
Hspa1a	2E-01	0.3	Tmod3	3E-09	0.3
Rhobtb2	7E-01	0.3	Slc24a5	3E-03	0.3
Ofd1	4E-04	0.3	Zfp26	1E-02	0.3
Tln1	1E-18	0.3	Cdkn1b	2E-09	0.3
Nipsnap1	1E-02	0.3	Castor2	3E-03	0.3
Usf3	5E-02	0.3	Slc4a7	6E-09	0.3
Il18	6E-01	0.3	Smarca5	1E-15	0.3
Mettl15	3E-02	0.3	Mat2b	5E-09	0.3

Undefined

Control			*Notch2*$^{tm1.1Ecan}$		
Gene	*p*	FC	Gene	*p*	FC
Pappa	1E-01	0.2	Ankzf1	4E-04	0.2
Borcs6	5E-02	0.2	Crim1	4E-01	0.2
Mtmr10	4E-01	0.2	Tmem101	6E-02	0.2
C1d	5E-01	0.2	Khk	5E-01	0.2
Proser1	2E-01	0.2	Psmb2	3E-07	0.2
1700030K09Rik	2E-03	0.2	Snrpb2	6E-07	0.2
Med7	2E-01	0.2	Nr3c2	1E-02	0.2
Manf	1E-02	0.2	Txndc12	5E-04	0.2
Sdc1	3E-03	0.2	Cebpg	6E-06	0.2
Oma1	9E-01	0.2	Ddx47	3E-08	0.2
Aasdh	4E-01	0.2	Polr2l	4E-07	0.2
Pnpt1	5E-03	0.2	Mrpl17	1E-05	0.2
Coro7	2E-02	0.2	Cops5	1E-07	0.2
Gnb4	7E-01	0.2	Ogn	6E-01	0.2
Mia2	6E-01	0.2	Ddx21	1E-04	0.2
Scrn2	1E-01	0.2	Chic1	5E-04	0.2
Cisd1	3E-01	0.2	Utp14a	1E-03	0.2
Aktip	1E-01	0.2	Fopnl	2E-12	0.2
Wdr92	1E-01	0.2	Slc7a6os	3E-01	0.2
Sdhaf4	1E-01	0.2	Trmu	1E-05	0.2
Rab24	3E-02	0.2	Rpl10	3E-19	0.2
Ptpn14	1E+00	0.2	Dock6	1E-04	0.2
Ganab	4E-01	0.2	Tcp1	4E-07	0.2
Pkig	7E-03	0.2	Prmt1	2E-06	0.2
Ank	1E-01	0.2	Creg1	7E-04	0.2
Ube2a	9E-07	0.2	1810026B05Rik	4E-05	0.2
Txndc16	3E-06	0.2	Rexo2	4E-01	0.2
Fyttd1	2E-01	0.2	Tsen2	3E-05	0.2
Vwa8	2E-01	0.2	Prorp	2E-04	0.2
Rrp12	7E-01	0.2	Pabpc4	5E-07	0.2
Dap	2E-01	0.2	Rps11	4E-14	0.2
Oard1	9E-02	0.2	Rpl34	1E-15	0.2
Mrto4	6E-01	0.2	F420014N23Rik	2E-03	0.2
Dhx32	6E-01	0.2	Wsb1	4E-06	0.2
Elp2	1E-01	0.2	Cst6	4E-03	0.2
Trappc11	1E-01	0.2	Ssb	1E-03	0.2
Cox14	1E-02	0.2	Tbrg1	6E-02	0.2
Lyst	1E-04	0.2	Dcxr	7E-05	0.2
Mettl1	5E-01	0.2	Phb	3E-06	0.2
Usp36	5E-01	0.2	Kat2a	4E-07	0.2
Fktn	8E-02	0.2	Rpp30	8E-04	0.2
Nudt22	2E-02	0.2	Rtf2	6E-11	0.2
Odc1	3E-01	0.2	Mrpl50	2E-08	0.2
Zcrb1	7E-01	0.2	Nubpl	6E-02	0.2
Macrod1	8E-01	0.2	Slc3a2	6E-03	0.2
Arl16	9E-03	0.2	Rnasek	5E-04	0.2
Rsl1d1	4E-02	0.2	Serp1	1E-04	0.2
Primpol	1E-01	0.2	Mbnl3	3E-02	0.2
Rrp8	3E-01	0.2	Ikbip	8E-04	0.2
Txndc12	1E-01	0.2	Slc39a7	8E-05	0.2
Gm26782	1E+00	0.2	Ggct	4E-01	0.2
Smox	3E-01	0.2	Ndrg3	6E-04	0.2
Rpp40	7E-01	0.2	Nae1	8E-05	0.2
Rabl3	2E-01	0.2	Ube2q2	1E-03	0.2
Smim19	2E-01	0.2	Rpl14	2E-11	0.2
Clptm1l	1E-02	0.2	Tsr3	3E-03	0.2
Ctsf	5E-01	0.2	Chchd7	4E-09	0.2
Dnajb11	5E-01	0.2	Psmb1	2E-05	0.2
Eef1g	9E-08	0.2	Dctpp1	7E-06	0.2
Gtf2f2	9E-01	0.2	Myd88	1E-05	0.2
Tdp2	5E-02	0.2	Map3k1	7E-02	0.2
Cnksr3	7E-01	0.2	Coq5	2E-06	0.2
Rps3a1	2E-26	0.2	Naa30	5E-08	0.2
Dus4l	8E-01	0.2	Fam174a	2E-04	0.2

Articular/Synovial Fibroblast

Control			*Notch2*$^{tm1.1Ecan}$		
Gene	*p*	FC	Gene	*p*	FC
Zfp949	4E-01	0.4	Zfp808	1E-01	0.3
Fam117b	6E-04	0.4	Tom1	6E-04	0.3
Gm7072	4E-02	0.4	Tulp4	3E-10	0.3
Camkmt	1E-05	0.4	Nlrx1	5E-02	0.3
Acox3	5E-03	0.4	Rft1	5E-02	0.3
Hgs	1E-01	0.4	Ccdc136	7E-04	0.3
Dpp7	3E-07	0.4	Hk1	1E-05	0.3
Vrk2	7E-03	0.4	Ripk1	5E-05	0.3
Lrp10	4E-10	0.4	Galnt4	1E-03	0.3
Atp6ap1	1E-16	0.4	Ldb1	5E-02	0.3
Ap3s1	1E-17	0.4	Mtss1	2E-01	0.3
Zadh2	4E-06	0.4	Lmbr1	5E-04	0.3
Psmb10	7E-05	0.4	Rnd3	3E-11	0.3
Spag9	3E-23	0.4	Sgce	3E-07	0.3
Mark2	7E-06	0.4	Nagk	9E-05	0.3
Rnf157	7E-04	0.4	Gtpbp3	3E-01	0.3
Secisbp2l	3E-07	0.4	Chd9	2E-10	0.3
Pid1	9E-06	0.4	Maml2	8E-14	0.3
Plppr2	2E-02	0.4	Six1	5E-01	0.3
Taf4	1E-01	0.4	Agmo	1E-01	0.3
Ndst1	2E-06	0.4	Git2	8E-03	0.3
Txndc16	2E-05	0.4	Ftsj1	1E+00	0.3
Cnnm3	1E-03	0.4	Ipo9	2E-01	0.3
Slc7a6	2E-03	0.4	Rps6ka3	6E-07	0.3
Ptp4a3	2E-04	0.4	9430038I01Rik	5E-02	0.3
Cib1	5E-05	0.4	Dram2	6E-04	0.3
Mta3	2E-05	0.4	Cpt2	4E-01	0.3
Stat5b	1E-03	0.4	Tor1b	6E-04	0.3
Tle4	1E-01	0.4	Dnm3	3E-02	0.3
Aldh4a1	1E-02	0.4	Ptpdc1	1E-01	0.3
Ralbp1	3E-16	0.4	Arpc1b	2E-11	0.3
Oip5os1	4E-11	0.4	Dok5	5E-03	0.3
Ctsz	3E-20	0.4	Erlin1	5E-02	0.3
Sptlc2	6E-08	0.4	Nfkbiz	2E-05	0.3
Appl2	3E-05	0.4	Tacc2	6E-05	0.3
Errfi1	8E-05	0.4	Oip5os1	5E-07	0.3
Fhl1	3E-07	0.4	Usp31	5E-01	0.3
Cab39l	4E-06	0.4	Wwox	2E-09	0.3
Tmem41b	2E-04	0.4	Gm12905	6E-03	0.3
Usp21	3E-01	0.4	Rabgap1l	3E-07	0.3
Zfp948	8E-03	0.4	Pisd	5E-03	0.3
Dync1h1	3E-12	0.4	Pikfyve	4E-04	0.3
Fbxo6	1E-04	0.4	Tmem241	7E-04	0.3
Ppp2r1b	4E-01	0.4	Tmem135	1E-05	0.3
Glrx	7E-04	0.4	Aph1b	1E-01	0.3
Cryab	2E-15	0.4	Calml4	4E-01	0.3
Fuca2	1E-04	0.3	Cd109	6E-07	0.3
Aldh16a1	7E-02	0.3	Usp25	4E-04	0.3
Rnf130	5E-08	0.3	Hmgcr	8E-04	0.3
Sgpl1	5E-05	0.3	Hacd4	4E-05	0.3
Klhl2	1E-03	0.3	Slc9a8	2E-02	0.3
Zc3h12b	7E-06	0.3	Atox1	1E-20	0.3
Pofut1	5E-04	0.3	Lrrk2	1E-01	0.3
Extl2	1E-01	0.3	Itm2b	2E-21	0.3
Ctsl	2E-24	0.3	Nrg1	3E-01	0.3
Fam199x	1E-01	0.3	3110082I17Rik	9E-01	0.3
Vps13d	1E-09	0.3	Plekhg5	2E-04	0.3
Pradc1	1E-02	0.3	Fdxr	3E-02	0.3
Tmx3	1E-09	0.3	Kctd13	4E-02	0.3
Cxxc1	7E-02	0.3	Herc4	8E-04	0.3
Slc35f5	2E-05	0.3	Scai	3E-03	0.3
Tyk2	4E-03	0.3	Fam53b	2E-06	0.3
Lhfpl2	5E-08	0.3	Rb1cc1	3E-08	0.3
Pde10a	1E-05	0.3	Dnmbp	1E-02	0.3

Limb Mesenchyme					
Control			*$Notch2^{tm1.1Ecan}$*		
Gene	*p*	FC	Gene	*p*	FC
Atp11c	3E-03	0.2	Cyth2	4E-07	0.2
Gipc1	1E-04	0.2	Prkra	3E-09	0.2
Dhps	1E-03	0.2	Cers5	6E-10	0.2
Ankrd54	3E-01	0.2	Josd1	1E-03	0.2
Fam102b	3E-05	0.2	Gabpa	2E-04	0.2
Ccdc74a	3E-02	0.2	Rcor1	5E-07	0.2
Pex19	2E-04	0.2	Rexo1	2E-05	0.2
S100a6	2E-59	0.2	Fam210b	6E-03	0.2
Rab11a	4E-12	0.2	Prelid3b	2E-06	0.2
Tmem242	2E-03	0.2	4921524J17Rik	5E-09	0.2
Psmg1	9E-04	0.2	Eipr1	3E-02	0.2
Zdhhc9	3E-02	0.2	Glrx3	1E-20	0.2
Magohb	1E-05	0.2	Carm1	2E-09	0.2
Rps16	2E-73	0.2	Ppp3cb	8E-07	0.2
Zdhhc13	6E-01	0.2	Pycard	6E-03	0.2
Grk6	5E-03	0.2	Atpif1	4E-26	0.2
Cgref1	9E-03	0.2	Nras	1E-10	0.2
Abraxas2	1E-04	0.2	Tap2	8E-04	0.2
Zfp719	4E-03	0.2	9430038I01Rik	3E-05	0.2
Siva1	1E-13	0.2	Egln2	3E-08	0.2
Cfl1	1E-28	0.2	Slc25a4	2E-32	0.2
Oxa1l	6E-03	0.2	Bcl10	2E-08	0.2
Rab30	8E-07	0.2	Igf1r	4E-12	0.2
Zfp52	2E-03	0.2	Snx10	8E-05	0.2
Diaph2	7E-06	0.2	Zfp511	3E-05	0.2
Tmem126b	2E-03	0.2	Twist2	7E-03	0.2
Plekhh3	2E-02	0.2	Gm26802	2E-05	0.2
Txndc5	4E-11	0.2	Lrrc32	2E-06	0.2
Ghdc	4E-01	0.2	Zfp286	1E-03	0.2
Tti2	2E-02	0.2	Bag2	8E-05	0.2
Vps18	4E-01	0.2	Rft1	1E-02	0.2
Pced1a	2E-01	0.2	Cep78	8E-03	0.2
Lama3	3E-02	0.2	Gnpat	2E-03	0.2
Tsta3	2E-05	0.2	Pip5k1c	5E-03	0.2
Kpnb1	2E-05	0.2	Lims1	4E-14	0.2
Nectin3	7E-05	0.2	Ak2	3E-07	0.2
Aldh1l2	3E-11	0.2	Hacd2	2E-06	0.2
Sin3b	5E-08	0.2	Arsk	4E-05	0.2
Dpp9	2E-05	0.2	Paxip1	7E-03	0.2
Zfp414	9E-03	0.2	Kirrel	2E-08	0.2
Fig4	2E-03	0.2	Rab5b	2E-07	0.2
Prmt7	1E-02	0.2	Dlat	2E-10	0.2
Spns1	8E-04	0.2	Ptpmt1	5E-09	0.2
Sat2	3E-02	0.2	AI837181	1E-05	0.2
Col6a3	7E-10	0.2	Crcp	4E-04	0.2
Samhd1	2E-03	0.2	Txn1	8E-24	0.2
Cdo1	1E-06	0.2	Mpnd	3E-10	0.2
Rps15a	4E-65	0.2	Med8	1E-08	0.2
Spred2	4E-04	0.2	Cyth3	5E-04	0.2
Scara3	3E-03	0.2	Trp53rka	2E-02	0.2
Sptbn1	8E-27	0.2	Cope	3E-15	0.2
Anapc11	4E-10	0.2	Sbf1	1E-03	0.2
Pcnx3	6E-02	0.2	Ndufaf1	1E-02	0.2
Etfb	2E-18	0.2	Zfp267	3E-02	0.2
Ociad2	1E-02	0.2	Polk	2E-05	0.2
Ggcx	6E-05	0.2	Bud13	8E-02	0.2
Myo1c	7E-08	0.2	Gosr2	7E-06	0.2
Zfp111	7E-02	0.2	Lpar6	9E-03	0.2
Appl1	2E-05	0.2	Clcn6	5E-01	0.2
Ccdc171	2E-01	0.2	Tmem19	5E-07	0.2
Glmp	3E-11	0.2	Clcn4	3E-06	0.2
Commd4	6E-03	0.2	Cct5	4E-18	0.2
Adck2	1E-01	0.2	Srxn1	2E-06	0.2
Gdpd1	1E-02	0.2	Gna13	1E-04	0.2

Chondrogenic					
Control			*$Notch2^{tm1.1Ecan}$*		
Gene	*p*	FC	Gene	*p*	FC
Cep68	8E-03	0.2	Rabac1	3E-26	0.2
Uxt	4E-03	0.2	Prrc1	5E-08	0.2
Zfp317	4E-01	0.2	Zbtb38	2E-06	0.2
2010315B03Rik	3E-02	0.2	Zfp395	2E-02	0.2
Phgdh	2E-18	0.2	Ttc32	4E-02	0.2
Bckdha	2E-03	0.2	Taf2	4E-03	0.2
Cog6	3E-03	0.2	Sp3os	1E-06	0.2
Bbs9	9E-04	0.2	Tent5a	3E-11	0.2
Smim27	1E-08	0.2	Lhfp	1E-08	0.2
Abca5	3E-02	0.2	Ppan	3E-04	0.2
Ttc7	6E-01	0.2	Pja2	2E-12	0.2
Thada	5E-05	0.2	Leng9	2E-01	0.2
Zzz3	1E-06	0.2	Eif2b1	7E-01	0.2
Marveld1	6E-06	0.2	Phc2	5E-03	0.2
Mbtd1	2E-06	0.2	Zbtb11	3E-06	0.2
Mapre3	1E-04	0.2	Kdm7a	2E-04	0.2
Eef1akmt4	1E-02	0.2	Pcca	2E-06	0.2
Tpd52	7E-03	0.2	Snhg6	2E-10	0.2
Yipf6	5E-03	0.2	Rptor	2E-05	0.2
Fam104a	4E-17	0.2	Gng11	1E+00	0.2
Cdc42se1	2E-04	0.2	Sugct	6E-03	0.2
Atg13	1E-03	0.2	Ddt	4E-15	0.2
Plod1	1E-06	0.2	Kmt5b	2E-05	0.2
Usp50	2E-03	0.2	Marveld1	2E-04	0.2
Letmd1	3E-02	0.2	1700025G04Rik	2E-03	0.2
Ttc32	3E-02	0.2	Armt1	9E-01	0.2
Nkiras1	3E-03	0.2	D10Wsu102e	3E-04	0.2
Shld1	1E-01	0.2	Nudt13	7E-01	0.2
Fem1a	4E-01	0.2	Higd1a	6E-19	0.2
Sars	2E-26	0.2	Cops9	2E-15	0.2
Snai2	6E-08	0.2	Ddhd1	7E-01	0.2
Snx25	1E-04	0.2	Gm32618	2E-01	0.2
1110032A03Rik	9E-05	0.2	Bbs9	2E-05	0.2
Pawr	6E-11	0.2	Inpp5a	3E-03	0.2
Prkce	5E-09	0.2	Phkb	3E-06	0.2
2010320M18Rik	4E-04	0.2	Ptar1	1E-01	0.2
3110009E18Rik	1E-01	0.2	Atp6v0e	3E-24	0.2
Mcrip1	2E-11	0.2	Foxp4	1E-03	0.2
Ddt	2E-12	0.2	Tcfl5	3E-03	0.2
Pum3	8E-08	0.2	Gfm2	3E-02	0.2
Cd151	4E-10	0.2	Pole2	1E-02	0.2
Pgm1	2E-03	0.2	Cox10	1E-03	0.2
Slc24a5	8E-04	0.2	2610306M01Rik	8E-02	0.2
Gtf2e2	2E-06	0.2	Iars2	1E-01	0.2
Gmpr	3E-02	0.2	Rab2b	3E-04	0.2
Plpbp	5E-04	0.2	Zbtb5	2E-01	0.2
Tsfm	1E-05	0.2	Unc50	1E-12	0.2
Dyrk1a	3E-05	0.2	Gpcpd1	2E-02	0.2
Zscan21	1E-03	0.2	Prpsap2	9E-03	0.2
Irf2bp1	3E-04	0.2	B3gat3	6E-04	0.2
Wdr12	2E-06	0.2	Cpa6	5E-01	0.2
4933421O10Rik	2E-03	0.2	mt-Nd5	7E-18	0.2
A930007I19Rik	9E-02	0.2	Malt1	3E-02	0.2
4833439L19Rik	6E-05	0.2	Rrp15	6E-04	0.2
B9d2	2E-02	0.2	Fbxo36	2E-03	0.2
Polr1a	4E-02	0.2	Tulp3	5E-05	0.2
Polr2m	3E-19	0.2	4933434E20Rik	2E-04	0.2
Arl4c	4E-02	0.2	Zfp523	6E-02	0.2
Npm3	2E-08	0.2	Coq7	5E-06	0.2
Hacd1	1E-10	0.2	Ttc4	3E-05	0.2
Gab1	3E-04	0.2	Top1	3E-16	0.2
Serf1	5E-16	0.2	Dph5	4E-02	0.2
Gm10561	2E-01	0.2	Cfap97	4E-06	0.2
Nr1d2	6E-03	0.2	Emc7	2E-13	0.2

Fibroblast					
Control			*$Notch2^{tm1.1Ecan}$*		
Gene	*p*	FC	Gene	*p*	FC
Usp40	7E-03	0.3	Psen1	4E-03	0.3
Dusp19	2E-02	0.3	Rassf8	1E-05	0.3
Dnajb4	5E-06	0.3	Cep250	1E-06	0.3
Ttyh3	7E-05	0.3	Sprtn	2E-02	0.3
Epb41l2	3E-10	0.3	Cnst	9E-02	0.3
Gm44899	9E-02	0.3	Abraxas1	4E-01	0.3
Il34	3E-01	0.3	Gab1	2E-06	0.3
Hsd17b12	8E-08	0.3	Ppwd1	2E-06	0.3
Spata7	1E-03	0.3	Snrpb	1E-18	0.3
Ybx3	7E-11	0.3	Fam129b	5E-07	0.3
Gemin2	3E-04	0.3	Prepl	8E-01	0.3
Rnf144a	2E-02	0.3	Ints13	3E-03	0.3
Cep85	4E-02	0.3	Ptpa	7E-09	0.3
Tln2	7E-07	0.3	Rp2	9E-05	0.3
Abcb10	4E-02	0.3	Elk4	6E-06	0.3
Gm17018	2E-02	0.3	Optn	9E-06	0.3
Afdn	9E-08	0.3	Ccdc97	3E-03	0.3
B4galt3	1E-01	0.3	Akt3	1E-09	0.3
Calm3	3E-07	0.3	Brms1	2E-04	0.3
Elp6	6E-02	0.3	Slc25a28	1E-02	0.3
Steap2	3E-03	0.3	Nudt21	1E-10	0.3
Arvcf	1E-01	0.3	Aox1	1E-02	0.3
Aifm1	4E-07	0.3	Tnrc18	2E-14	0.3
Ndst2	2E-02	0.3	Nab1	1E-05	0.3
Actb	1E-23	0.3	Plcl1	2E-11	0.3
Ccdc28b	9E-02	0.3	Tpm1	2E-19	0.3
Adat2	1E-01	0.3	Eefsec	1E-01	0.3
Med30	2E-03	0.3	H3f3a	3E-52	0.3
Ap4b1	2E-02	0.3	Prkd1	7E-07	0.3
Rtn3	4E-12	0.3	Zfp438	1E-03	0.3
Svopl	2E-03	0.3	Mindy3	4E-05	0.3
Mpv17l2	2E-03	0.3	Men1	3E-02	0.3
Lgals1	5E-18	0.3	Pbx1	5E-11	0.3
D6Wsu163e	3E-02	0.3	Snx18	6E-07	0.3
Hdac7	1E-03	0.3	Ppfibp1	2E-11	0.3
Smurf2	9E-13	0.3	Dcun1d2	2E-02	0.3
Lin7c	7E-04	0.3	Dtnbp1	2E-15	0.3
Stn1	1E-02	0.3	Vasn	1E-08	0.3
Gm34455	7E-02	0.3	Tmem43	1E-06	0.3
Lcmt1	3E-04	0.3	Zfp120	2E-02	0.3
Lrig1	5E-02	0.3	Pakap	8E-08	0.3
Ergic2	2E-07	0.3	Cry2	5E-08	0.3
Sall2	1E-01	0.3	Efna1	6E-02	0.3
Hdac2	1E-04	0.3	Calhm2	6E-03	0.3
Jph2	4E-02	0.3	Egfr	2E-12	0.3
Mtpap	3E-02	0.3	Fam92a	7E-13	0.3
Eif4enif1	1E-04	0.3	Iqcb1	3E-03	0.3
Sfpq	2E-10	0.3	Dhrs7b	3E-05	0.3
Tnpo1	1E-08	0.3	Adcy6	2E-02	0.3
Epn2	6E-04	0.3	Cd2bp2	2E-04	0.3
Mgp	2E-06	0.3	Mat2a	2E-07	0.3
Mprip	6E-13	0.3	Cplane1	6E-06	0.3
Zw10	4E-02	0.3	Dhrs3	4E-08	0.3
Nid2	3E-06	0.3	Zcchc2	3E-01	0.3
Wrb	5E-01	0.3	Rbm27	2E-06	0.3
Zmat1	9E-03	0.3	Grk5	5E-08	0.3
Gla	6E-02	0.3	Bckdk	2E-06	0.3
Ranbp10	3E-02	0.3	Kcnd2	3E-04	0.3
Pa2g4	1E-04	0.3	2900076A07Rik	7E-03	0.3
Wdr53	3E-03	0.3	Capn10	7E-03	0.3
Lmtk2	3E-03	0.3	Acot9	2E-07	0.3
Ptpn21	9E-04	0.3	Insig1	7E-12	0.3
C1ra	6E-07	0.3	Cnot9	4E-04	0.3
Klc1	1E-06	0.3	Pgm1	1E-04	0.3

Undefined					
Control			*$Notch2^{tm1.1Ecan}$*		
Gene	*p*	FC	Gene	*p*	FC
Trmt10c	4E-01	0.2	Serhl	3E-07	0.2
Pim1	4E-01	0.2	Trp53inp1	3E-05	0.2
Psmg2	8E-01	0.2	Ift57	1E-04	0.2
Gm4924	7E-01	0.2	Cfl2	9E-04	0.2
Ywhaq	9E-01	0.2	Pign	3E-08	0.2
Sh2d4a	8E-01	0.2	Ndufa13	1E+00	0.2
1110038B12Rik	6E-01	0.2	Foxd1	3E-01	0.2
Ankzf1	7E-02	0.2	Zfp672	2E-04	0.2
Magi3	1E-05	0.2	Csel1	2E-08	0.2
Ints13	9E-01	0.2	Bin3	6E-06	0.2
Mon2	2E-01	0.2	Eed	6E-05	0.2
Mrps9	4E-03	0.2	Rnf44	9E-03	0.2
Zfp768	8E-03	0.2	Vbp1	1E-06	0.2
Ifrd2	6E-01	0.2	Phospho2	7E-06	0.2
Plpbp	2E-01	0.2	Zbtb33	4E-04	0.2
Vps37a	1E-01	0.2	Ttc37	1E-06	0.2
Rpe	2E-01	0.2	Tcfl5	2E-04	0.2
Satb2	3E-02	0.2	Rfxap	6E-05	0.2
Nol10	8E-02	0.2	Ndufb5	3E-04	0.2
Skp1a	1E-02	0.2	Fam149b	4E-07	0.2
Bmpr1a	4E-01	0.2	Hspa14	4E-06	0.2
Rdh14	6E-01	0.2	Ndufaf3	4E-02	0.2
Prmt3	3E-01	0.2	Cars	2E-06	0.2
Fam204a	7E-01	0.2	Gm48678	9E-02	0.2
Taf5l	1E-01	0.2	Pprc1	2E-06	0.2
Nbr1	2E-01	0.2	Ccdc171	6E-05	0.2
Rhou	2E-04	0.2	Rps18	2E-13	0.1
Ice2	3E-01	0.2	Pdlim4	2E-04	0.1
Gnl1	1E-02	0.2	Supt4a	5E-11	0.1
Fahd1	1E-01	0.2	5031425E22Rik	2E-02	0.1
Trim39	4E-01	0.2	Kdsr	2E-05	0.1
Imp3	4E-02	0.2	Bbs5	5E-03	0.1
Zwint	1E-01	0.2	Sfi1	7E-05	0.1
Tmem203	5E-01	0.2	Gnb1l	2E-04	0.1
Kiz	5E-01	0.2	Kpna1	2E-05	0.1
B9d2	5E-01	0.2	Gucd1	4E-03	0.1
Slc23a2	2E-01	0.2	Zfp346	2E-03	0.1
Pard3bos1	3E-02	0.2	Fam173a	7E-11	0.1
Slc25a28	9E-01	0.2	Fbl	5E-08	0.1
Slc35d2	2E-02	0.2	Psmc2	2E-08	0.1
Phykpl	7E-01	0.2	Zfpm2	5E-01	0.1
Coq4	1E-02	0.2	Cnot7	3E-05	0.1
Polr2h	2E-01	0.2	Alg1	1E-03	0.1
Srp14	6E-04	0.2	Nprl3	4E-04	0.1
Krtcap2	1E-02	0.2	Haghl	2E-04	0.1
Ndufs5	2E-01	0.2	Tmtc4	2E-03	0.1
Mmaa	6E-01	0.2	Hist1h2ac	2E-03	0.1
Cd2ap	9E-01	0.2	Tsnax	8E-08	0.1
Slc25a37	7E-01	0.2	Tmem243	7E-04	0.1
Adcy9	5E-01	0.2	Ola1	2E-10	0.1
Hagh	9E-01	0.2	Sec61g	3E-02	0.1
Cndp2	2E-05	0.2	Ncbp2	1E-08	0.1
Acadsb	3E-01	0.2	Rxrb	2E-09	0.1
Gm47071	6E-01	0.2	Fntb	7E-05	0.1
Mettl7a1	8E-02	0.2	Nmnat3	5E-02	0.1
Rwdd4a	2E-01	0.2	Mxi1	4E-11	0.1
Bccip	5E-01	0.2	Pigp	8E-03	0.1
Zhx3	1E+00	0.2	Ktn1	2E-03	0.1
Ubap2	9E-01	0.2	Zbtb38	6E-03	0.1
Bysl	4E-01	0.2	Tmem37	4E-11	0.1
Vps39	3E-01	0.2	Ormdl3	6E-03	0.1
Mrps12	4E-03	0.2	Dync2li1	2E-02	0.1
Spsb2	2E-03	0.2	Manf	2E-01	0.1
Slain2	8E-01	0.2	Aff2	4E-01	0.1

Articular/Synovial Fibroblast					
Control			*$Notch2^{tm1.1Ecan}$*		
Gene	*p*	FC	Gene	*p*	FC
Epn2	2E-04	0.3	Ehd1	3E-05	0.3
Zkscan6	1E-01	0.3	Klhl23	2E-01	0.3
Znfx1	3E-03	0.3	Extl2	9E-03	0.3
Nectin2	3E-02	0.3	Itgb8	3E-03	0.3
Nudt18	4E-04	0.3	Lonp2	4E-06	0.3
Suds3	2E-06	0.3	Lrrc57	1E-01	0.3
Eif5a2	8E-02	0.3	Calhm5	9E-02	0.3
Cd81	1E-36	0.3	Zfhx4	7E-10	0.3
Dzip1	2E-04	0.3	Ppp6r2	6E-05	0.3
Lats2	3E-08	0.3	Cln5	4E-04	0.3
Glud1	7E-17	0.3	Fam193a	7E-09	0.3
Dnpep	4E-08	0.3	Lpcat3	1E-05	0.3
Sh3bgrl3	2E-23	0.3	Ifngr2	2E-04	0.3
Snta1	2E-04	0.3	Znrf2	4E-02	0.3
Ccpg1	3E-05	0.3	Sh3bgrl3	4E-17	0.3
2610001J05Rik	3E-08	0.3	Arhgap12	2E-04	0.3
Adgre5	5E-02	0.3	Spg21	2E-08	0.3
Cdyl	9E-08	0.3	Cdc42se1	3E-03	0.3
Atox1	3E-28	0.3	Jam2	7E-03	0.3
Idh2	2E-08	0.3	Sh3bgrl	1E-11	0.3
Hccs	2E-02	0.3	Prr16	1E-01	0.3
Cdc42se1	1E-03	0.3	Anxa4	2E-09	0.3
Tspan14	2E-02	0.3	Usf3	3E-01	0.3
Tbc1d19	2E-03	0.3	Ext1	5E-08	0.3
Lmtk2	1E-05	0.3	Commd4	1E-05	0.3
Zfp369	2E-02	0.3	Calcoco1	2E-03	0.3
Tet2	3E-04	0.3	Lrch3	2E-04	0.3
Dnajb4	1E-07	0.3	Macrod1	5E-08	0.3
Ints7	5E-05	0.3	Eif2ak2	3E-05	0.3
Nudt7	2E-02	0.3	Fnip1	2E-11	0.3
Tmem231	3E-02	0.3	Git1	5E-02	0.3
Lrch2	2E-01	0.3	Dgkh	1E-04	0.3
Ccdc25	3E-02	0.3	Rbm41	2E-02	0.3
Tecr	5E-19	0.3	Snx13	3E-02	0.3
Klhl24	2E-07	0.3	2510009E07Rik	2E-03	0.3
Gnpda2	1E-04	0.3	Ndst1	1E-04	0.3
Atp8b2	1E-02	0.3	Abat	4E-03	0.3
Nup133	1E-01	0.3	Dhrs11	5E-02	0.3
Rad51d	7E-04	0.3	Tubgcp3	2E-02	0.3
Itm2b	2E-33	0.3	Mxi1	1E-03	0.3
Sos2	9E-07	0.3	Usp24	2E-04	0.3
Tmem256	3E-20	0.3	Arhgef40	4E-04	0.3
Atg10	1E-08	0.3	Camkk2	7E-04	0.3
Sspn	1E-06	0.3	Wdr55	5E-02	0.3
Zc3hav1	3E-05	0.3	Rilpl1	2E-03	0.3
Lpp	8E-27	0.3	Ap3m1	2E-01	0.3
Ccdc102a	2E-06	0.3	Amer1	9E-04	0.3
Kif1c	8E-04	0.3	D11Wsu47e	1E-01	0.3
B4galt3	1E+00	0.3	Hsd17b12	7E-07	0.3
Mxd4	3E-18	0.3	Gask1b	2E-01	0.3
Zfp182	2E-04	0.3	Snx18	3E-06	0.3
Zc3h6	2E-04	0.3	Pcyt1a	2E-06	0.3
Zxdc	4E-03	0.3	Ccdc171	1E-03	0.3
Ssbp4	5E-05	0.3	Fdx1	2E-05	0.3
Gcdh	3E-02	0.3	Sdc2	6E-15	0.3
Usp28	2E-01	0.3	Specc1	3E-05	0.3
Crip2	4E-07	0.3	Zfyve1	6E-03	0.3
Zfp30	3E-04	0.3	Zc3h12a	2E-03	0.3
Rara	2E-03	0.3	Hoxc5	9E-03	0.3
Mapk6	3E-06	0.3	Brf1	8E-02	0.3
Scn2a	2E-02	0.3	Hsf2	5E-03	0.3
Klf13	7E-13	0.3	Klhl29	6E-05	0.3
Nxpe4	1E-06	0.3	Ap3s1	2E-10	0.3
Prkcg	5E-03	0.3	Lum	7E-04	0.3

Limb Mesenchyme

Control			*Notch2*$^{tm1.1Ecan}$		
Gene	*p*	FC	Gene	*p*	FC
Rpl10a	5E-50	0.2	Nsa2	8E-19	0.2
Dusp10	2E-01	0.2	Mical1	1E-04	0.2
Rcc1l	8E-02	0.2	Hmgcr	6E-07	0.2
Abhd5	8E-07	0.2	Vamp3	4E-13	0.2
Ckap4	5E-18	0.2	Osbpl1a	2E-07	0.2
Nans	4E-04	0.2	Gtf3a	4E-10	0.2
Fam114a1	2E-15	0.2	Fam171a1	2E-07	0.2
Serpinb9	6E-01	0.2	Slco3a1	6E-05	0.2
Robo2	1E-10	0.2	Nelfa	9E-06	0.2
Gabarap	2E-41	0.2	Arfrp1	3E-06	0.2
Vim	2E-22	0.2	Csnk1d	3E-08	0.2
Plat	6E-02	0.2	Csnk2b	2E-12	0.2
Aldh9a1	2E-03	0.2	Lrsam1	5E-04	0.2
Ogfod2	3E-01	0.2	Fam91a1	2E-06	0.2
Arf6	8E-06	0.2	Nfkb2	1E-04	0.2
Rps4x	5E-54	0.2	Ndufa3	2E-23	0.2
Armc10	2E-01	0.2	Pxdn	1E-09	0.2
Spin1	2E-03	0.2	Dip2b	2E-09	0.2
Toe1	2E-02	0.2	Vkorc1	2E-14	0.2
Kidins220	3E-07	0.2	Skil	4E-05	0.2
Fkbp8	9E-09	0.2	Socs3	1E-04	0.2
Cdk9	1E-05	0.2	Coq8b	5E-04	0.2
Sh3glb1	5E-20	0.2	Grn	9E-24	0.2
Zfas1	2E-14	0.2	Jazf1	2E-02	0.2
Dctn5	2E-02	0.2	Srm	2E-10	0.2
Vps9d1	1E-01	0.2	Arntl	1E-02	0.2
Hsd3b7	3E-01	0.2	Piezo2	4E-03	0.2
Slc35a2	5E-04	0.2	Tmem185a	2E-03	0.2
Lpp	2E-13	0.2	Rps5	2E-44	0.2
Unc119	3E-02	0.2	Rictor	1E-05	0.2
Zbtb12	4E-01	0.2	Mtmr9	2E-05	0.2
Pard3b	7E-10	0.2	Ilvbl	3E-08	0.2
Eif3h	7E-30	0.2	Abca3	3E-02	0.2
Map3k14	8E-01	0.2	Fam76a	2E-08	0.2
Esrra	1E-03	0.2	Fam214b	7E-05	0.2
Qdpr	5E-05	0.2	Tmod3	6E-11	0.2
Lrrc32	4E-02	0.2	Capn10	9E-04	0.2
Sar1a	8E-12	0.2	Tap1	2E-04	0.2
Pld3	2E-07	0.2	Ctxn1	7E-04	0.2
Pelp1	4E-03	0.2	Ap1s1	2E-14	0.2
LTO1	1E-01	0.2	Ndufs8	9E-15	0.2
Crtap	2E-12	0.2	Rgs3	3E-09	0.2
Dnajc18	6E-03	0.2	Ptprs	3E-10	0.2
Rbfox1	2E-01	0.2	Ccnd3	2E-11	0.2
Cd81	1E-20	0.2	Sft2d1	2E-05	0.2
0610010K14Rik	1E-05	0.2	Mier3	5E-06	0.2
Rnf166	7E-03	0.2	Arf6	5E-09	0.2
Mfsd1	2E-05	0.2	Arpc2	3E-19	0.2
Flot2	3E-05	0.2	Chst2	2E-04	0.2
Snrk	9E-04	0.2	Mrps18a	4E-09	0.2
Mea1	3E-09	0.2	Cc2d1a	6E-03	0.2
Emp1	4E-12	0.2	Cars	2E-11	0.2
Pde5a	1E-02	0.2	Nmrk1	6E-02	0.2
Rpsa	2E-51	0.2	Tradd	4E-05	0.2
Cct5	9E-11	0.2	Ube2a	4E-06	0.2
Rpl29	7E-51	0.2	Dapk1	2E-06	0.2
Egln2	4E-04	0.2	Actb	6E-24	0.2
Gga2	2E-04	0.2	Zfp800	1E-03	0.2
Tmem86a	1E-02	0.2	Gnai2	4E-24	0.2
Usp25	4E-06	0.2	Arap1	4E-06	0.2
Fam53c	6E-02	0.2	Msn	1E-09	0.2
Fbxl14	1E-01	0.2	Rxrb	2E-07	0.2
Cacng7	1E+00	0.2	Kctd20	6E-04	0.2
Psap	6E-26	0.2	Slc39a3	2E-04	0.2

Chondrogenic

Control			*Notch2*$^{tm1.1Ecan}$		
Gene	*p*	FC	Gene	*p*	FC
Clba1	4E-03	0.2	Evi5l	5E-03	0.2
Ppm1b	4E-04	0.2	Gm20275	9E-02	0.2
Mterf3	6E-03	0.2	Slc35a3	2E-01	0.2
Llph	1E-11	0.2	2810004N23Rik	2E-09	0.2
Gm4285	4E-01	0.2	Llph	3E-12	0.2
Mepce	3E-03	0.2	Armcx5	8E-02	0.2
Manf	7E-23	0.2	Cpsf6	2E-06	0.2
Mccc2	1E-02	0.2	Rab40c	7E-04	0.2
Tra2b	4E-15	0.2	Nme1	4E-19	0.2
Tsen15	1E-04	0.2	Susd6	1E-11	0.2
Fth1	8E-71	0.2	Lzic	4E-04	0.2
Tsr3	1E-05	0.2	Cul2	3E-04	0.2
Trib1	5E-03	0.2	Galnt7	3E-02	0.2
Me3	3E-02	0.2	Usf3	1E-01	0.2
Riox2	2E-01	0.2	Mtln	2E-06	0.2
Bex3	2E-15	0.2	Tmx4	1E-01	0.2
Prmt1	3E-13	0.2	Pde4a	1E-01	0.2
Gm26542	6E-01	0.2	Mrtfb	9E-05	0.2
Rab2b	3E-04	0.2	Zfp398	2E-01	0.2
Srbd1	6E-02	0.2	Gm47071	3E-03	0.2
Gpbp1	2E-09	0.2	Tmem11	8E-08	0.2
Cdyl2	2E-01	0.2	Uqcrq	8E-26	0.2
Mgat5	2E-03	0.2	Tmco1	5E-11	0.2
Erlec1	2E-13	0.2	Ube4b	4E-06	0.2
2700049A03Rik	4E-03	0.2	Pex2	1E-06	0.2
Hbs1l	2E-06	0.2	Acad11	1E-01	0.2
Vars	2E-05	0.2	Dusp5	3E-01	0.2
Tecpr2	2E-02	0.2	Slc37a4	1E-01	0.2
Usp2	2E-03	0.2	Mrpl24	7E-10	0.2
Tmem11	6E-06	0.2	Tbrg1	2E-16	0.2
Fastk	5E-07	0.2	Nuak1	1E-04	0.2
Leprotl1	2E-11	0.2	Ubxn7	1E-02	0.2
Dym	4E-05	0.2	Uvssa	1E-03	0.2
Myo9b	7E-06	0.2	Apobr	6E-02	0.2
Phc2	2E-04	0.2	Spg20	3E-06	0.2
Zfp827	2E-05	0.2	Ndufaf6	2E-02	0.2
Ing4	9E-07	0.2	Ddx18	2E-07	0.2
Parl	3E-08	0.2	Pttg1	2E-02	0.2
Ccdc28a	4E-02	0.2	Coq2	3E-07	0.2
Bscl2	6E-07	0.2	Cnot7	3E-04	0.2
Erh	8E-08	0.2	Zfyve9	1E-04	0.2
Phf5a	5E-08	0.2	Uso1	4E-04	0.2
Gnl3l	4E-08	0.2	Uck2	2E-02	0.2
Slc25a40	9E-01	0.2	Ccnq	4E-06	0.2
Tgfb2	9E-11	0.2	Ndufa6	4E-20	0.2
Exosc3	2E-06	0.2	Zfat	3E-02	0.2
Rflnb	3E-02	0.2	Ndufab1	4E-21	0.2
Rnf114	3E-02	0.2	2310039H08Rik	1E-08	0.2
Fubp1	7E-13	0.2	Gfer	2E-04	0.2
Erich1	1E-02	0.2	Cbwd1	4E-02	0.2
Tmem164	3E-04	0.2	Ercc5	4E-02	0.2
Rnps1	1E-09	0.2	Usp54	2E-01	0.2
Nat8f1	8E-03	0.2	Ddx50	2E-12	0.2
Klc4	5E-02	0.2	C030006K11Rik	2E-04	0.2
Rpf2	3E-03	0.2	Cars	2E-05	0.2
Srp72	2E-13	0.2	Mogs	3E-03	0.2
Tmem223	9E-12	0.2	Rnf138	1E-03	0.2
Primpol	1E-01	0.2	Trappc2	1E-02	0.2
Zfp975	7E-01	0.2	Tmem60	1E-09	0.2
Ube2e3	3E-07	0.2	Zfp146	8E-03	0.2
Set	1E-26	0.2	Trim47	2E-02	0.2
Fermt2	2E-09	0.2	Tbccd1	1E+00	0.2
Card19	2E-13	0.2	Adamts9	2E-05	0.2
Hipk1	2E-03	0.2	Rcl1	3E-02	0.2

Fibroblast

Control			*Notch2*$^{tm1.1Ecan}$		
Gene	*p*	FC	Gene	*p*	FC
Rnf157	4E-02	0.3	Hdac7	2E-04	0.3
Plec	3E-09	0.3	Nfyb	3E-06	0.3
Smim12	9E-02	0.3	Uggt1	7E-08	0.3
Morn4	6E-02	0.3	Plekhh2	1E-10	0.3
Irf2	2E-04	0.2	Hnrnpl	2E-10	0.3
Apool	1E-03	0.2	Atp11b	4E-04	0.3
Supt16	1E-06	0.2	Arhgap23	2E-10	0.3
Nxt2	3E-02	0.2	Icmt	3E-01	0.3
Rbm14	3E-03	0.2	Nipa2	1E-04	0.3
Uchl1	9E-12	0.2	Cep350	9E-07	0.3
Cstf3	2E-07	0.2	Maoa	2E-03	0.3
Fzd1	1E-03	0.2	Sphk2	8E-02	0.3
Prrc2a	6E-05	0.2	Rfx5	5E-02	0.3
Numb	8E-04	0.2	Pdlim1	5E-08	0.3
Tgfbr1	4E-04	0.2	Kdm6a	3E-04	0.3
Cyren	3E-01	0.2	Itih5	2E-04	0.3
Pomk	3E-02	0.2	Ap2m1	1E-14	0.3
Rragb	3E-02	0.2	Plaat3	2E-10	0.3
Rassf3	3E-02	0.2	Map3k20	1E-08	0.3
Csnk1e	5E-05	0.2	Recql5	5E-02	0.3
U2af1	2E-08	0.2	Dcaf17	1E-03	0.3
Pdik1l	4E-03	0.2	Igf1r	1E-21	0.3
Ptma	1E-27	0.2	Dhrs4	6E-08	0.3
Gadd45b	2E-05	0.2	Morn1	4E-04	0.3
Synm	2E-01	0.2	Rab43	1E-03	0.3
Bicd1	6E-02	0.2	Syn3	5E-10	0.3
AW554918	4E-03	0.2	Ttc13	3E-03	0.3
Rmnd5b	3E-02	0.2	Map9	7E-04	0.3
Gemin8	1E-02	0.2	Dennd2a	2E-02	0.3
Tmsb10	3E-18	0.2	Aldh9a1	5E-01	0.3
Mpp1	1E-02	0.2	Sik2	2E-08	0.3
Sfmbt1	5E-03	0.2	Cyb5r3	4E-23	0.3
Dok5	4E-01	0.2	Rcor2	8E-02	0.3
Acyp2	5E-09	0.2	Rnf217	4E-06	0.3
Sardh	3E-03	0.2	Paics	2E-11	0.3
Vps33b	5E-02	0.2	Ccdc167	5E-07	0.3
Nrp1	4E-07	0.2	Cbl	3E-05	0.3
Apeh	2E-02	0.2	Helb	4E-04	0.3
Crat	7E-04	0.2	Nup188	1E-03	0.3
Mnat1	1E-11	0.2	Anapc11	2E-11	0.3
Tmem143	2E-01	0.2	Rngtt	4E-07	0.3
Tbc1d4	6E-04	0.2	Frg1	5E-08	0.3
Dennd4c	1E-03	0.2	Tmsb10	1E-26	0.3
Pigf	1E-03	0.2	Pfkp	9E-07	0.3
Me3	2E-02	0.2	Hivep2	4E-11	0.3
Wdr60	3E-02	0.2	Nubp2	9E-03	0.3
Med14	4E-04	0.2	Glipr2	2E-06	0.3
Arnt2	9E-02	0.2	Itpr2	2E-04	0.3
Timp1	5E-05	0.2	Mpp1	5E-04	0.3
Adam9	2E-05	0.2	Gpn3	6E-04	0.3
Ggt7	1E-02	0.2	Smarca4	1E-10	0.3
Stradb	9E-01	0.2	Cep85	2E-04	0.3
Lactb2	3E-04	0.2	Pde6d	7E-09	0.3
Depdc5	7E-03	0.2	Snx1	1E-07	0.3
Ttc26	3E-01	0.2	Ctcf	1E-10	0.3
Sgcd	1E-15	0.2	Chac2	3E-03	0.3
Usp4	1E-04	0.2	Gtf2ird1	5E-06	0.3
Lap3	2E-05	0.2	Nvl	2E-04	0.3
Bbs7	2E-03	0.2	Caml	5E-04	0.3
Tob1	2E-07	0.2	Lancl2	3E-03	0.3
Nras	3E-06	0.2	Exoc2	1E-04	0.3
Phrf1	1E-04	0.2	Odf2	5E-05	0.3
Nudt4	2E-07	0.2	Bahcc1	5E-03	0.3
Arid5b	3E-12	0.2	Nsdhl	7E-05	0.3

Undefined

Control			*Notch2*$^{tm1.1Ecan}$		
Gene	*p*	FC	Gene	*p*	FC
Med20	8E-02	0.2	Tmem98	4E-07	0.1
Nfe2l1	3E-01	0.2	Commd1	2E-11	0.1
Dhdds	2E-01	0.2	C1d	2E-08	0.1
Aifm1	1E+00	0.2	Tor2a	2E-08	0.1
Gfer	6E-04	0.2	Eif3i	3E-03	0.1
Crebzf	4E-01	0.2	Dtd1	9E-06	0.1
Mepce	2E-01	0.2	Ice2	2E-06	0.1
Cyp4f13	1E+00	0.2	Mrpl53	9E-09	0.1
Gid4	6E-02	0.2	Anxa11	6E-04	0.1
2810403D21Rik	2E-01	0.2	Ndufb4	1E-03	0.1
Psmb5	9E-01	0.2	Cisd2	3E-02	0.1
Pdik1l	1E-01	0.2	C4b	2E-01	0.1
Zranb2	4E-01	0.2	Rab11fip2	1E-03	0.1
Ssbp2	3E-01	0.2	Utp18	4E-06	0.1
Uqcrh	2E-09	0.2	Arl3	1E-04	0.1
Tmem216	5E-01	0.2	Arl2bp	3E-06	0.1
Vegfa	1E-01	0.2	BC051226	4E-03	0.1
Tapt1	1E-01	0.2	Alg6	6E-03	0.1
Mif	7E-01	0.2	Zfp106	4E-04	0.1
Nle1	8E-01	0.2	Exosc1	1E-04	0.1
Nup210l	6E-01	0.2	Gid4	5E-07	0.1
2810032G03Rik	1E-01	0.2	Lman2l	2E-02	0.1
Prpf3	9E-02	0.2	Agfg1	6E-08	0.1
Tmem126a	4E-01	0.2	1110004F10Rik	4E-03	0.1
Cdpf1	2E-01	0.2	Rab24	3E-08	0.1
Tbpl1	2E-01	0.2	Fra10ac1	4E-05	0.1
Prlr	4E-01	0.2	Prune1	7E-06	0.1
Plscr2	6E-01	0.2	Atp5f1	4E-05	0.1
Wdfy2	3E-02	0.2	Rpl13	2E-18	0.1
Mbd3	3E-02	0.2	Triap1	6E-06	0.1
Trim23	5E-05	0.2	Pmpcb	7E-08	0.1
Igip	4E-01	0.2	Sec61b	1E-02	0.1
Sdcbp	2E-01	0.2	Optn	5E-03	0.1
Rad1	5E-01	0.2	Abca5	3E-02	0.1
Ptrhd1	6E-01	0.2	Sod1	6E-04	0.1
Nprl2	2E-02	0.2	Eif3g	4E-07	0.1
Dab2	8E-01	0.2	Kri1	3E-02	0.1
Hipk2	6E-01	0.2	Tmem218	1E-07	0.1
Nop58	3E-01	0.2	Etf1	4E-05	0.1
Sesn2	2E-01	0.2	Zmat1	2E-03	0.1
Plscr4	1E-02	0.1	Sesn3	3E-01	0.1
Ccdc59	2E-01	0.1	Chmp2a	7E-06	0.1
Tob1	8E-01	0.1	Dact1	8E-07	0.1
Ogn	1E-02	0.1	Otulin	2E-12	0.1
Mamld1	9E-02	0.1	Gnl3	2E-04	0.1
Zscan29	1E+00	0.1	Ttll7	4E-04	0.1
Immp1l	2E-01	0.1	Kmt2c	4E-12	0.1
Slirp	2E-01	0.1	Derl1	1E-04	0.1
Cwc22	1E-01	0.1	Decr1	6E-09	0.1
Ttyh2	5E-01	0.1	P4htm	1E-02	0.1
Arl6	3E-02	0.1	Rpap1	3E-02	0.1
Rtraf	2E-02	0.1	Acbd5	3E-04	0.1
Klc2	3E-01	0.1	Tnrc6b	1E-01	0.1
Ndufaf8	2E-01	0.1	Rpl7	5E-14	0.1
Cdkal1	5E-02	0.1	Ttpal	1E-04	0.1
Eef1akmt4	4E-01	0.1	Amn1	2E-05	0.1
Mtfr1	9E-01	0.1	Aga	2E-02	0.1
Eef1b2	2E-14	0.1	Rpl27	3E-05	0.1
Vps26c	5E-02	0.1	Mri1	1E-07	0.1
Ssbp1	4E-01	0.1	Churc1	9E-10	0.1
Ndufa6	8E-01	0.1	Mybbp1a	3E-09	0.1
E230016M11Rik	4E-01	0.1	Bcl7b	7E-07	0.1
Zmym5	1E-01	0.1	Fpgs	8E-07	0.1
Timm8b	9E-01	0.1	Mar5	2E-05	0.1

Articular/Synovial Fibroblast

Control			*Notch2*$^{tm1.1Ecan}$		
Gene	*p*	FC	Gene	*p*	FC
Cdk19	3E-05	0.3	Armcx6	2E-01	0.3
Fhit	2E-05	0.3	Slc9a1	3E-03	0.3
Arnt	1E-07	0.3	Pced1a	3E-01	0.3
Zfp426	4E-02	0.3	Npepps	8E-09	0.3
Nsmf	1E-02	0.3	Acsl3	2E-03	0.3
Nudt16	2E-01	0.3	Vps33a	2E-01	0.3
Rab30	5E-06	0.3	Fam76a	1E-09	0.3
Depdc5	9E-04	0.3	Soga1	9E-03	0.3
Zc2hc1a	1E-06	0.3	Eif4e3	3E-01	0.3
Zbtb43	5E-02	0.3	Glmn	2E-03	0.3
Abcb1b	7E-02	0.3	1810024B03Rik	5E-01	0.3
Optn	1E-05	0.3	Mindy3	3E-03	0.3
Zhx1	3E-04	0.3	Gm12353	3E-01	0.3
Rtca	2E-02	0.3	Rragc	6E-06	0.3
Zbtb7a	3E-08	0.3	Cttnbp2nl	2E-02	0.3
Prcp	8E-03	0.3	Sav1	1E-01	0.3
Npdc1	4E-15	0.3	Scrn3	9E-02	0.3
Ctbs	3E-02	0.3	Zdhhc13	2E-02	0.3
Emp1	7E-15	0.3	Ago4	6E-02	0.3
Morn2	3E-04	0.3	AU022252	1E-02	0.3
Mospd2	1E-05	0.3	Commd8	3E-04	0.3
Pcyox1l	2E-01	0.3	Cyfip1	1E-08	0.3
Galk2	2E-12	0.3	Coq10a	6E-04	0.3
Ppp1r7	3E-05	0.3	Nfkbia	1E-02	0.3
Stk3	8E-12	0.3	Arhgap35	3E-05	0.3
Isoc2b	3E-02	0.3	Brdt	2E-01	0.3
Ppp1r16a	6E-01	0.3	Zxdc	1E-02	0.3
Zfp984	5E-02	0.3	Rlf	8E-07	0.3
Zhx3	9E-06	0.3	Rbms2	2E-08	0.3
Dcbld2	7E-07	0.3	Ranbp10	3E-02	0.3
Afdn	1E-09	0.3	Adamts10	7E-03	0.3
Abcc5	9E-12	0.3	Map2k3	3E-06	0.3
Il10rb	1E-02	0.3	AI987944	2E-02	0.3
Map1lc3b	2E-27	0.3	Nhej1	5E-02	0.3
Washc5	2E-07	0.3	0610010F05Rik	2E-04	0.3
Cetn4	5E-03	0.3	Ypel3	6E-13	0.3
Slc38a6	3E-04	0.3	Flii	7E-05	0.3
Pip5k1c	6E-03	0.3	Gnptab	9E-03	0.3
Ube2e2	1E-12	0.3	Ccn3	2E-11	0.3
Abi1	2E-11	0.3	Cd99l2	2E-03	0.3
Atp11c	2E-04	0.3	Camkmt	1E-03	0.3
Nfkbiz	4E-08	0.3	Qdpr	7E-03	0.3
Zfp810	2E-02	0.3	Sap18	1E-05	0.3
Tent2	4E-08	0.3	Pkd1	5E-03	0.3
Ripk1	2E-05	0.3	Dusp8	2E-01	0.3
Clic1	4E-27	0.3	Washc4	7E-05	0.3
Ifi27	2E-21	0.3	Mad2l2	8E-02	0.3
Nbn	2E-02	0.3	Nbea	2E-05	0.3
Katnb1	2E-01	0.3	BC034090	1E-01	0.3
Fam189b	2E-04	0.3	Pros1	4E-04	0.3
Dlg4	3E-04	0.3	Atg4a	5E-02	0.3
Tnip1	7E-02	0.3	Sh3rf3	3E-11	0.3
Prkab2	3E-01	0.3	Ephb2	2E-01	0.3
Baz2a	6E-04	0.3	Smyd4	3E-02	0.3
Nipsnap3b	2E-03	0.3	Ssbp4	1E-05	0.3
Slain2	3E-06	0.3	Slc39a10	2E-02	0.3
Zmiz1	9E-12	0.3	Gigyf2	6E-10	0.3
Atp6v1e1	7E-21	0.3	Zfp668	2E-01	0.3
Snx8	3E-02	0.3	Zfp976	3E-02	0.3
Fam151b	4E-02	0.3	Maml3	1E-03	0.3
Zfp942	7E-03	0.3	Btbd10	4E-03	0.3
Wee1	3E-02	0.3	Tmem63b	6E-05	0.3
Hspg2	6E-15	0.3	Gmcl1	1E-02	0.3
Slc25a30	2E-01	0.3	Dhx33	5E-01	0.3

Limb Mesenchyme

Control Gene	p	FC	*Notch2*$^{tm1.1Ecan}$ Gene	p	FC
Cep170	2E-08	0.2	Akirin2	2E-11	0.2
Rffl	2E-01	0.2	Eif3l	1E-13	0.2
Slc39a1	9E-06	0.2	Plekhh3	2E-04	0.2
Bfar	3E-05	0.2	Ube2w	1E-11	0.2
Arpc5	2E-11	0.2	Clip3	3E-09	0.2
Rpl38	4E-69	0.2	Cep83	4E-07	0.2
Pecr	2E-01	0.2	Zfp90	5E-03	0.2
Hgs	7E-04	0.2	Rps6ka3	1E-07	0.2
Ttc38	2E-01	0.2	Mpzl1	3E-07	0.2
Phldb2	2E-18	0.2	Atp23	7E-03	0.2
Dnajc30	8E-04	0.2	Phgdh	6E-16	0.2
Tapbp	3E-05	0.2	Mecom	1E-05	0.2
Lsm10	3E-05	0.2	Serp1	1E-12	0.2
Zfp267	2E-01	0.2	3300002I08Rik	9E-02	0.2
Kalrn	3E-03	0.2	Ercc1	5E-06	0.2
Yipf3	2E-10	0.2	Nus1	5E-06	0.2
Ubac1	4E-03	0.2	Tecr	4E-16	0.2
Glce	3E-03	0.2	Cnn3	4E-14	0.2
Rap1b	8E-13	0.2	Trappc2l	7E-15	0.2
Gaa	2E-07	0.2	Tbcd	1E-05	0.2
Crls1	1E-02	0.2	Vangl1	3E-06	0.2
Ints6l	1E-01	0.2	Fech	9E-08	0.2
Exosc4	1E-04	0.2	Maml1	8E-04	0.2
Klhl23	3E-02	0.2	Spryd7	2E-03	0.2
Slc4a3	3E-01	0.2	Lmo1	6E-07	0.2
E430024I08Rik	2E-01	0.2	Socs6	4E-03	0.2
Col3a1	7E-57	0.2	Gm14326	2E-02	0.2
Rxrb	4E-03	0.2	Zfp948	7E-05	0.2
Sgta	8E-06	0.2	Scrn1	5E-07	0.2
Aars	6E-11	0.2	Esrra	9E-04	0.2
2610044O15Rik8	7E-02	0.2	Ndufs6	1E-17	0.2
Rps19	8E-57	0.2	Rpl27	2E-47	0.2
Acsl5	5E-03	0.2	Hnrnpul2	7E-09	0.2
Tmem120b	2E-02	0.2	Triobp	1E-04	0.2
Stx4a	4E-04	0.2	Rps19	4E-45	0.2
Mtmr11	1E-02	0.2	Pnp	3E-11	0.2
Lhfp	4E-15	0.2	Usf1	7E-06	0.2
Rpl37a	2E-57	0.2	Rps18	1E-43	0.2
Ofd1	2E-03	0.2	Gabarap	2E-34	0.2
Stom	3E-01	0.2	Relb	4E-03	0.2
Mrpl9	4E-04	0.2	Ypel5	2E-06	0.2
Shprh	2E-05	0.2	Dnlz	3E-07	0.2
Aldh18a1	2E-03	0.2	Scn1b	1E-09	0.2
Casp6	8E-02	0.2	Selenow	5E-23	0.2
Rcor1	8E-07	0.2	Stx17	1E-04	0.2
Rnh1	5E-13	0.2	Sik1	5E-04	0.2
Txn1	1E-22	0.2	Get4	2E-07	0.2
Zfp385a	1E-01	0.2	Stard5	1E-03	0.2
Cpt2	5E-04	0.2	Mrpl16	1E-04	0.2
Nsf	6E-06	0.2	Cln8	2E-02	0.2
Tdrd7	1E-01	0.2	Rps29	2E-60	0.2
Smim1	2E-03	0.2	Nid2	3E-07	0.2
Urm1	2E-03	0.2	Hras	2E-13	0.2
Casp8	9E-03	0.2	Sumo3	9E-06	0.2
Atp6v0b	5E-12	0.2	Snhg8	3E-16	0.2
Irf9	1E-02	0.2	Dusp18	2E-04	0.2
Ywhah	2E-10	0.2	Actr1a	2E-06	0.2
Npepps	3E-09	0.2	Ptov1	2E-14	0.2
Trpc1	4E-02	0.2	Mpv17	8E-10	0.2
Rab36	4E-01	0.2	Hddc2	3E-04	0.2
Cxxc5	2E-04	0.2	E2f4	5E-05	0.2
Plrg1	7E-03	0.2	Sall2	5E-03	0.2
Glmn	2E-02	0.2	Fam129a	3E-16	0.2
9230114K14Rik	6E-02	0.2	Polr3d	4E-09	0.2

Chondrogenic

Control Gene	p	FC	*Notch2*$^{tm1.1Ecan}$ Gene	p	FC
Slc30a9	3E-06	0.2	Nudt9	3E-06	0.2
R3hdm1	1E-08	0.2	Fitm2	2E-01	0.2
Nop16	2E-04	0.2	Nupl2	5E-01	0.2
Anks1	7E-04	0.2	Gm37494	1E-02	0.2
Polr3e	2E-02	0.2	Slc9a8	9E-02	0.2
Btaf1	1E-07	0.2	Crcp	1E-01	0.2
Tmem98	7E-03	0.2	Phf12	1E-02	0.2
Foxp4	2E-02	0.2	Gm20342	4E-01	0.2
2900076A07Rik	3E-02	0.2	Ttc9c	7E-03	0.2
Pcyox1l	7E-02	0.2	Ccdc28a	6E-01	0.2
Fam53a	7E-04	0.2	Ylpm1	3E-05	0.2
Zcrb1	9E-17	0.2	Eif3g	2E-09	0.2
Usp49	5E-01	0.2	Clpp	9E-09	0.2
Ncoa3	7E-04	0.2	Naa38	9E-10	0.2
Epb41l1	3E-03	0.2	Nfs1	4E-03	0.2
Alg1	7E-03	0.2	Gde1	1E-07	0.2
Noc3l	2E-03	0.2	Pum3	6E-06	0.2
Ppdpf	1E-08	0.2	Bex3	4E-16	0.2
Atf6b	3E-03	0.2	Swap70	9E-05	0.2
Luc7l	3E-08	0.2	Gm4876	1E-03	0.2
Prickle1	8E-11	0.2	Bivm	1E-01	0.2
Ccl25	4E-03	0.2	Rsbn1	3E-03	0.2
Mrto4	8E-05	0.2	Dmtf1	4E-02	0.2
Slc38a1	3E-05	0.2	Tdg	3E-02	0.2
Efna1	1E-01	0.2	Zfp608	8E-07	0.2
Cpsf6	2E-07	0.2	Nop2	2E-02	0.2
Hsd17b10	1E-10	0.2	Pnrc2	7E-09	0.2
Susd6	3E-08	0.2	Itpr3	4E-04	0.2
Coq7	3E-04	0.2	Itpkb	6E-02	0.2
Acbd5	1E-02	0.2	Sephs2	1E-01	0.2
Rom1	2E-01	0.2	1110032A03Rik	3E-06	0.2
Ide	7E-07	0.2	Creld2	6E-08	0.2
Ppic	1E-19	0.2	Ambra1	4E-09	0.2
Gm34455	4E-03	0.2	Lgi2	7E-03	0.2
Trmu	5E-01	0.2	Rabl3	1E-01	0.2
Ibtk	3E-03	0.2	N4bp1	7E-07	0.2
Gng7	2E-02	0.2	Acaa1a	4E-08	0.2
Trmt11	9E-02	0.2	Zfp788	1E-01	0.2
Rplp0	5E-65	0.2	Mthfsl	1E-01	0.2
Cep63	4E-05	0.2	Ccng1	6E-08	0.2
Gbe1	8E-03	0.2	Rdh14	6E-03	0.2
Pck2	1E-11	0.2	Slc35c2	6E-04	0.2
Myo10	2E-09	0.2	Cmpk1	4E-13	0.2
Ints4	1E-02	0.2	Rps25	5E-36	0.2
Eif1ad	7E-03	0.2	Ttc17	7E-03	0.2
Sec62	4E-27	0.2	Per3	3E-04	0.2
Adamts12	5E-02	0.2	Rwdd4a	1E-04	0.2
Bola1	7E-09	0.2	Ppp2r5a	5E-04	0.2
Rexo2	9E-22	0.2	Zfp994	1E-01	0.2
Cox17	9E-16	0.2	Tm2d3	8E-07	0.2
Cnot7	3E-05	0.2	Eef1g	1E-25	0.2
Sp3os	2E-05	0.2	Mrps10	2E-03	0.2
2810004N23Rik	8E-11	0.2	Supt3	2E-06	0.2
Sympk	2E-02	0.2	Cdc37l1	5E-09	0.2
Dus1l	2E-03	0.2	Rnf14	7E-04	0.2
Nol11	4E-04	0.2	Abitram	5E-02	0.2
Atp6v0e	6E-22	0.2	Tmed1	1E-09	0.2
Kat6b	3E-07	0.2	Dhrs3	2E-17	0.2
Lemd2	5E-04	0.2	Mrpl20	2E-15	0.2
Dhrs13	4E-01	0.2	Agap1	3E-07	0.2
Nob1	6E-04	0.2	Zfp113	8E-01	0.2
Rchy1	8E-05	0.2	Psmg4	2E-07	0.2
Rab10os	8E-03	0.2	Nip7	4E-06	0.2
Plcd1	4E-03	0.2	Dnal4	3E-02	0.2

Fibroblast

Control Gene	p	FC	*Notch2*$^{tm1.1Ecan}$ Gene	p	FC
2310009B15Rik	2E-07	0.2	Lin37	7E-03	0.3
Zc3h18	1E-04	0.2	Sardh	6E-06	0.3
Gas8	6E-02	0.2	Fuom	2E-08	0.3
Plp2	3E-06	0.2	Cnot1	1E-07	0.3
Golm1	6E-03	0.2	Mipep	9E-03	0.3
Aacs	5E-03	0.2	Lims1	2E-13	0.3
Wdr19	3E-01	0.2	Magohb	1E-04	0.3
Gng13	1E-01	0.2	Fosl2	6E-09	0.3
Rab1b	1E-03	0.2	Scly	7E-02	0.3
Cetn2	1E-06	0.2	Traf3ip2	3E-01	0.3
Hnrnpll	2E-06	0.2	Hoxa10	2E-11	0.3
Specc1	6E-06	0.2	Numb	1E-03	0.3
Crem	4E-02	0.2	Pfdn6	4E-05	0.3
Lyar	1E-04	0.2	AI506816	2E-10	0.3
Osbpl10	2E-01	0.2	Dnajb4	3E-08	0.3
Oip5os1	6E-06	0.2	Rrp12	1E-02	0.3
Kif1b	4E-12	0.2	Hnrnpd	2E-11	0.3
Plpp2	7E-04	0.2	Dcaf1	3E-06	0.3
Itih5	1E-01	0.2	Tcerg1	2E-10	0.3
Caprin2	8E-03	0.2	Gcfc2	6E-03	0.3
E2f6	7E-03	0.2	Cycs	6E-06	0.3
Ifnar1	7E-02	0.2	Pcnx	1E-06	0.3
Fbxw11	9E-07	0.2	Ttll5	2E-04	0.3
Pqbp1	2E-04	0.2	Gfra4	2E-02	0.3
Mpdz	6E-08	0.2	Rock1	1E-13	0.3
Lmbrd2	2E-03	0.2	Pls3	4E-13	0.3
Smg6	1E-09	0.2	Amotl2	1E-10	0.3
Tmx2	9E-03	0.2	Agap3	6E-03	0.3
Ano6	6E-06	0.2	Nr2c2	1E-05	0.3
Ppp1r37	3E-02	0.2	Dennd5b	8E-05	0.3
Malt1	3E-02	0.2	Mpdz	2E-09	0.3
Nbn	4E-03	0.2	Pacsin2	4E-09	0.3
Slc35e4	8E-04	0.2	Eipr1	2E-02	0.3
Dnajb11	3E-07	0.2	Zfp760	1E-03	0.3
Cnih4	3E-09	0.2	Twist1	2E-09	0.3
Mtf1	3E-02	0.2	Abracl	2E-11	0.3
Rtca	7E-03	0.2	2310022B05Rik	4E-06	0.3
Gab1	1E-06	0.2	1110059E24Rik	5E-06	0.3
Tbc1d1	1E-04	0.2	Arl6ip6	9E-05	0.3
E2f3	6E-04	0.2	Cand1	4E-08	0.3
Rnf219	1E-02	0.2	Zfp251	3E-02	0.3
Ppia	5E-22	0.2	Aarsd1	1E-04	0.3
Tpk1	8E-05	0.2	Usp20	9E-03	0.3
Ola1	9E-09	0.2	Hdgfl3	1E-03	0.3
Heatr5b	2E-03	0.2	Tnpo3	3E-08	0.3
Klhl18	3E-03	0.2	0610010F05Rik	2E-06	0.3
Psmd5	8E-03	0.2	Atrnl1	1E-07	0.3
Calhm2	5E-02	0.2	Hnrnpu	3E-19	0.3
Dennd5b	3E-03	0.2	Dusp16	2E-03	0.3
Cdc42se2	7E-05	0.2	Scrib	9E-05	0.3
Cycs	4E-07	0.2	Thbs1	5E-06	0.3
Zbtb41	4E-01	0.2	Letm1	4E-04	0.3
Eed	5E-04	0.2	Jup	2E-03	0.3
Ckap4	7E-09	0.2	Mus81	1E-01	0.3
Cnp	2E-02	0.2	Snrnp40	2E-04	0.3
Ier5	3E-05	0.2	Fam118b	2E-03	0.3
Tle5	3E-16	0.2	Sorbs3	8E-05	0.3
Rmdn3	1E-03	0.2	Ilk	1E-09	0.3
Kctd10	9E-03	0.2	Ttll11	1E-01	0.3
Egfr	4E-06	0.2	Tab1	9E-02	0.3
Slain2	1E-04	0.2	Hnrnpul2	1E-08	0.3
Hspg2	5E-11	0.2	1810026B05Rik	3E-08	0.3
Ppp3cc	2E-01	0.2	Cers6	5E-07	0.3
Cdc27	6E-04	0.2	Srpk1	2E-06	0.3

Undefined

Control Gene	p	FC	*Notch2*$^{tm1.1Ecan}$ Gene	p	FC
Usp10	8E-01	0.1	Elp2	4E-05	0.1
Tpi1	7E-01	0.1	Tomm6	3E-08	0.1
Znrf3	1E-01	0.1	Eef1g	2E-03	0.1
Slc7a6os	5E-01	0.1	Fubp1	7E-07	0.1
Gars	7E-01	0.1	Gm10076	2E-03	0.1
Clic4	8E-03	0.1	Cln3	3E-05	0.1
Itpr1	5E-01	0.1	2010315B03Rik	4E-01	0.1
Btbd19	1E-01	0.1	Gid8	2E-09	0.1
Rps25	4E-18	0.1	Clic4	2E-02	0.1
Cacnb2	4E-08	0.1	Bzw2	1E-09	0.1
Dstn	2E-02	0.1	Hoxc5	8E-06	0.1
Gm30025	6E-02	0.1	Taf2	2E-04	0.1
Bbx	9E-01	0.1	Zfas1	8E-02	0.1
Paip2	9E-01	0.1	Nadk	7E-13	0.1
Ap1g1	1E+00	0.1	Gli2	9E-01	0.1
Ikbip	4E-01	0.1	Dnajb11	6E-08	0.1
Ptpn1	1E-01	0.1	BC004004	5E-11	0.1
Zyg11b	1E+00	0.1	Dexi	1E-09	0.1
Uggt1	1E-02	0.1	Rps8	2E-16	0.1
Hist1h2ac	3E-01	0.1	Rad23a	1E-05	0.1
Nudt8	3E-01	0.1	Atp6ap1	2E-06	0.1
Gm16740	2E-01	0.1	Znrd1	2E-09	0.1
Itgb8	7E-01	0.1	Thumpd1	1E-05	0.1
Rsl24d1	4E-02	0.1	Btaf1	4E-06	0.1
Nup214	2E-01	0.1	Uap1	9E-05	0.1
Eif3g	1E-01	0.1	Chd1l	2E-01	0.1
Fbxo31	3E-01	0.1	Tet2	4E-07	0.1
C4b	3E-04	0.1	Yme1l1	5E-07	0.1
Eif4ebp3	1E-01	0.1	Cstf2t	1E-07	0.1
C87436	1E-01	0.1	Rps23	6E-15	0.1
Dpy19l3	1E-02	0.1	Lrwd1	2E-08	0.1
Maf1	1E-02	0.1	Chchd1	1E-07	0.1
Esco1	6E-01	0.1	Minpp1	2E-03	0.1
Atad1	1E-02	0.1	Ensa	2E-07	0.1
Tfb2m	5E-01	0.1	Ciao2b	1E-06	0.1
Wdr77	5E-01	0.1	Socs7	1E-05	0.1
Ppip5k1	8E-01	0.1	Jade1	2E-01	0.1
Bicral	6E-01	0.1	Ostc	2E-03	0.1
Rbbp9	5E-01	0.1	Nip7	5E-08	0.1
Tsfm	6E-02	0.1	Fam135a	1E-03	0.1
Slc45a4	5E-01	0.1	Tm2d1	9E-06	0.1
Dbt	9E-02	0.1	Yipf4	8E-05	0.1
Eif2b3	6E-01	0.1	Hmox2	4E-05	0.1
Dnajb14	2E-01	0.1	Lamtor2	1E-05	0.1
Mtrex	6E-02	0.1	Mrpl24	9E-05	0.1
Cog3	7E-01	0.1	Rabggtb	3E-04	0.1
Zhx2	5E-01	0.1	Prmt3	9E-06	0.1
Chchd1	2E-03	0.1	Ppp1r11	4E-04	0.1
Prorp	5E-01	0.1	Suclg1	2E-08	0.1
Zfp933	9E-01	0.1	Pik3r4	1E-09	0.1
Ier2	4E-03	0.1	Rpl18a	6E-14	0.1
Bbs7	2E-02	0.1	Zswim1	6E-05	0.1
Nifk	8E-01	0.1	Ttyh2	3E-04	0.1
Rpl35a	7E-20	0.1	Rpl11	1E-06	0.1
Traf3	7E-01	0.1	Dcbld1	8E-03	0.1
Rnf7	3E-01	0.1	Timmdc1	2E-03	0.1
Rpl7l1	2E-01	0.1	Ddost	3E-05	0.1
Rgs7bp	1E-01	0.1	Rad17	5E-06	0.1
Rpn2	8E-01	0.1	Mplkip	2E-10	0.1
Asb3	8E-02	0.1	Bex3	3E-05	0.1
Neu1	5E-02	0.1	E130307A14Rik	1E-04	0.1
Appbp2	4E-01	0.1	Swi5	1E-01	0.1
Zdhhc6	2E-02	0.1	Usp50	4E-05	0.1
Guk1	4E-01	0.1	Fxyd1	3E-04	0.1

Articular/Synovial Fibroblast

Control Gene	p	FC	*Notch2*$^{tm1.1Ecan}$ Gene	p	FC
Myh10	9E-08	0.3	Shld2	3E-01	0.3
Aldh9a1	6E-04	0.3	Dab2ip	4E-06	0.3
Limch1	1E-03	0.3	Slc9a6	6E-01	0.3
Rab13	3E-04	0.3	Galns	5E-02	0.3
Mcm9	4E-03	0.3	Ralbp1	4E-07	0.3
Sbf2	6E-10	0.3	Lmna	2E-19	0.3
Blvrb	7E-05	0.3	Hspb2	1E-02	0.3
Sbds	4E-07	0.3	Rnf145	5E-03	0.3
B4galt6	2E-03	0.3	Cyp39a1	1E-01	0.3
Gxylt2	6E-04	0.3	Timm21	4E-01	0.3
Ankib1	9E-09	0.3	Nfs1	4E-02	0.3
Irgq	6E-02	0.3	Enoph1	3E-01	0.3
Zfp944	7E-02	0.3	Rerg	2E-01	0.3
Heatr5b	1E-01	0.3	Lrrfip1	6E-06	0.3
Tprkb	5E-04	0.3	Gm14326	2E-01	0.3
Arrdc3	3E-04	0.3	Pbxip1	6E-08	0.3
1110012L19Rik	7E-04	0.3	Patl1	5E-02	0.3
Zfp46	3E-02	0.3	Maco1	3E-03	0.3
Rhoj	1E-10	0.3	R3hcc1l	4E-02	0.3
Mast4	2E-05	0.3	Gga2	5E-04	0.3
Cttnbp2	1E-05	0.3	Zfp407	2E-05	0.3
Hbp1	7E-07	0.3	Tmem106b	6E-06	0.3
Dlg5	2E-03	0.3	Rap1gds1	4E-04	0.3
Igfbp5	3E-01	0.3	Mier3	2E-02	0.3
Ftl1-ps1	4E-02	0.3	Shkbp1	3E-01	0.3
Ranbp10	2E-03	0.3	A930037H05Rik	3E-01	0.3
Ncstn	6E-04	0.3	Afap1	3E-09	0.3
Sept1	6E-01	0.3	Aar2	2E-02	0.3
Pcyt1a	2E-06	0.3	Fbxw11	3E-05	0.3
Zfp667	2E-01	0.3	Qars	2E-03	0.3
Mapk3	7E-07	0.3	Afdn	2E-06	0.3
Hivep3	4E-03	0.3	Kat2b	2E-04	0.3
Mxi1	1E-03	0.3	Optn	2E-03	0.3
Vps35l	1E-06	0.3	Etfdh	3E-03	0.3
1700037H04Rik	1E-02	0.3	Rhoc	7E-13	0.3
Sirt2	3E-07	0.3	Scarb1	4E-02	0.3
Zcchc3	4E-01	0.3	Rsrp1	3E-12	0.3
Rufy1	3E-03	0.3	Tsc2	8E-02	0.3
Phc1	1E-01	0.3	Atg4c	2E-01	0.3
Gas8	2E-03	0.3	Sugct	3E-02	0.3
Stx18	3E-04	0.3	Tapbp	4E-07	0.3
Lrrfip1	2E-09	0.3	Pradc1	6E-02	0.3
Stx4a	1E-05	0.3	Zfp87	9E-02	0.3
Abtb1	4E-02	0.3	Col6a3	6E-12	0.3
Inf2	6E-02	0.3	Nynrin	3E-02	0.3
Traf7	5E-03	0.3	Ppp3cc	1E-01	0.3
Stxbp1	7E-03	0.3	Dennd1a	6E-09	0.3
Zfp703	3E-03	0.3	Hes1	7E-08	0.3
Pdia5	5E-07	0.3	Nptn	1E-14	0.3
Sdc2	2E-13	0.3	Dtwd2	3E-01	0.3
Zfp808	1E-01	0.3	Csk	6E-05	0.3
Cdadc1	1E-02	0.3	Eif2ak4	1E-01	0.3
Ankrd24	3E-02	0.3	Kat14	9E-03	0.3
Cds2	6E-03	0.3	Ccdc85b	5E-06	0.3
Kidins220	1E-08	0.3	Dnpep	2E-04	0.3
Crtc3	2E-07	0.3	Gm20342	2E-01	0.3
Rhobtb1	7E-03	0.3	1700021F05Rik	2E-03	0.3
Ccdc22	5E-02	0.3	Tollip	8E-02	0.3
Bcorl1	3E-02	0.3	Zfp931	4E-01	0.3
Fut10	6E-03	0.3	Taf1b	7E-01	0.3
Pabpc4l	1E+00	0.3	Ddx6	1E-06	0.3
Pard3bos1	2E-02	0.3	Tcp11l1	5E-01	0.3
Zfp40	2E-02	0.3	Dock1	2E-08	0.3
Pxn	2E-03	0.3	Zc3h12b	3E-04	0.3

Limb Mesenchyme					
Control			*Notch2*$^{tm1.1Ecan}$		
Gene	*p*	FC	Gene	*p*	FC
Pdcd2	1E-03	0.2	Etfb	5E-16	0.2
Col6a2	5E-16	0.2	Ulk1	2E-03	0.2
Tceal8	5E-12	0.2	Lgals8	2E-07	0.2
Ubc	1E-29	0.2	Spidr	3E-04	0.2
5730455P16Rik	3E-02	0.2	Elovl6	1E-07	0.2
Ilvbl	1E-03	0.2	Ckap4	2E-16	0.2
Thra	2E-13	0.2	Sc5d	1E-06	0.2
H13	1E-06	0.2	Ascc3	1E-06	0.2
Ybx3	1E-11	0.2	Cstb	2E-18	0.2
Tbcd	2E-02	0.2	Rps16	5E-46	0.2
Elp6	9E-01	0.2	Tmem43	1E-05	0.2
Fbxl15	2E-02	0.2	Haus2	4E-03	0.2
Smc6	2E-15	0.2	Atp5d	4E-23	0.2
Ap2s1	6E-16	0.2	Akt1s1	7E-14	0.2
Galnt17	2E-07	0.2	Greb1l	1E-01	0.2
Plin3	2E-05	0.2	Gxylt1	3E-02	0.2
Lias	3E-03	0.2	Rpl27a	1E-46	0.2
Rps26	3E-43	0.2	Wrn	2E-06	0.2
Krtcap2	3E-26	0.2	Mrrf	8E-05	0.2
Rab22a	1E-08	0.2	Ech1	4E-15	0.2
Map2k1	3E-05	0.2	Nynrin	2E-03	0.2
Tpgs1	7E-05	0.2	Ube2v1	1E-13	0.2
Zdhhc14	3E-02	0.2	Bfar	1E-06	0.2
Carm1	5E-03	0.2	Sgta	3E-08	0.2
Rrp36	2E-02	0.2	Hdac2	9E-09	0.2
Fzd8	4E-05	0.2	Stx18	1E-08	0.2
Nadk2	6E-02	0.2	Prrx1	2E-15	0.2
Inpp5f	2E-02	0.2	Pdxk	2E-02	0.2
Eif3k	5E-22	0.2	Ubtd1	3E-07	0.2
Zfp36	6E-04	0.2	Tmed3	5E-21	0.2
Rps29	8E-69	0.2	Kptn	6E-03	0.2
Armc1	1E-05	0.2	Atp13a3	1E-08	0.2
Asb7	2E-01	0.2	Faap100	1E-03	0.2
Tomm5	5E-11	0.2	Nfe2l2	1E-13	0.2
Mr1	1E-01	0.2	Ifnar1	3E-04	0.2
Rpl32	1E-55	0.2	Actr2	3E-10	0.2
Rgs3	3E-04	0.2	Atp13a1	2E-03	0.2
Hddc2	6E-04	0.2	Zfp354c	6E-06	0.2
Slc25a4	9E-33	0.2	Prr13	3E-13	0.2
Ptpn13	6E-03	0.2	Rpl36	1E-39	0.2
Hps3	1E-02	0.2	Vegfb	2E-06	0.2
Mcoln1	8E-02	0.2	Tsen34	1E-10	0.2
Ppp2r1a	2E-04	0.2	Antxr1	7E-20	0.2
Ctps	3E-04	0.2	Rilpl1	2E-05	0.2
Sidt2	2E-02	0.2	Washc3	4E-10	0.2
Cstb	3E-16	0.2	Mustn1	5E-06	0.2
1110038B12Rik	9E-18	0.2	Pwwp3b	3E-03	0.2
Dram2	5E-04	0.2	Ccm2	3E-09	0.2
Rit1	2E-04	0.2	Mrps18b	9E-08	0.2
Slc30a7	8E-04	0.2	Gopc	6E-05	0.2
Obsl1	7E-02	0.2	Dync1h1	2E-12	0.2
Mrps15	9E-09	0.2	Sgce	1E-10	0.2
Pstk	9E-02	0.2	Nipa1	2E-02	0.2
Gm14326	7E-02	0.2	Dctn6	1E-06	0.2
Magi2	3E-10	0.2	Map2k3	3E-09	0.2
Akirin2	3E-04	0.2	Ypel2	1E-04	0.2
Napa	2E-06	0.2	Ddr2	3E-13	0.2
Trappc8	3E-03	0.2	Calhm2	3E-03	0.2
Keap1	9E-04	0.2	Cd302	2E-07	0.2
Ppp3cb	8E-06	0.2	Plaat3	9E-11	0.2
Nog	9E-01	0.2	Rpl41	3E-52	0.2
Slc17a5	2E-02	0.2	Derl2	9E-08	0.2
Tbcb	1E-07	0.2	Ranbp3	1E-03	0.2
Cdc42	4E-21	0.2	Eif4a1	4E-22	0.2

Chondrogenic					
Control			*Notch2*$^{tm1.1Ecan}$		
Gene	*p*	FC	Gene	*p*	FC
Gstcd	2E-02	0.2	Mkrn1	3E-04	0.2
Hlcs	1E-03	0.2	Ccny	2E-08	0.2
Rpl39l	2E-01	0.2	Synpo	2E-04	0.2
Tfam	5E-04	0.2	Prkcsh	2E-09	0.2
Phf1	3E-02	0.2	Eif5b	3E-15	0.2
Ddx27	5E-04	0.2	Cmss1	1E-13	0.2
Fbxo36	3E-02	0.2	Coro7	4E-01	0.2
Pde4a	1E-01	0.2	Fbxo42	4E-03	0.2
Stim2	2E-03	0.2	Mrps14	2E-15	0.2
Dnajc17	6E-02	0.2	Terf2ip	5E-02	0.2
Hist1h4d	7E-04	0.2	Apopt1	2E-04	0.2
Ubac1	7E-06	0.2	Pvt1	8E-09	0.2
Trmt13	3E-01	0.2	AU040320	2E-05	0.2
Kdm6b	9E-07	0.2	Rab3a	2E-03	0.2
Stau2	5E-04	0.2	Tsr1	9E-02	0.2
Tbl1x	1E-08	0.2	Tbc1d8b	4E-01	0.2
Gabarapl2	2E-18	0.2	D430042O09Rik	1E-01	0.2
Riok2	4E-02	0.2	Zfp869	2E-02	0.2
Pdss1	4E-01	0.2	Clasp2	5E-06	0.2
Wdr4	7E-02	0.2	Ubqln4	4E-01	0.2
Pard6g	6E-02	0.2	Srm	8E-14	0.2
Jam2	1E-02	0.2	Kiz	2E-07	0.2
Uqcrq	9E-28	0.2	Zfp821	6E-01	0.2
Ctu2	7E-01	0.2	Ip6k2	6E-03	0.2
Limd2	2E-05	0.2	Gm4924	7E-02	0.2
5430405H02Rik	2E-03	0.2	Eif2b3	1E-03	0.2
Sclt1	6E-04	0.2	Nup210l	2E-01	0.2
Tasor2	7E-04	0.2	H2afj	1E-12	0.2
Tmem33	1E-06	0.2	Dera	6E-01	0.2
Bin3	8E-02	0.2	Pygo1	3E-02	0.2
Rpl7l1	7E-06	0.2	Optn	3E-02	0.2
Diablo	7E-05	0.2	Trappc6a	1E-07	0.2
Prpf4b	8E-10	0.2	Eral1	6E-01	0.2
Ftsj1	2E-01	0.2	Rragb	2E-01	0.2
Pabpc4	1E-12	0.2	Tmem199	1E-02	0.2
Fam234a	8E-03	0.2	Jam2	1E-05	0.2
Klhl22	2E-04	0.2	Mrpl57	7E-15	0.2
Nap1l3	3E-02	0.2	Zfp637	3E-08	0.2
Tma16	4E-03	0.2	Zfp938	3E-03	0.2
Zbtb11	1E-06	0.2	Mapkap1	2E-08	0.2
Rps21	4E-86	0.2	Rhot1	3E-03	0.2
Fli1	2E-04	0.2	Bmi1	4E-04	0.2
Fam114a2	3E-06	0.2	Slc38a7	2E-01	0.2
Rps3a1	4E-74	0.2	Tarbp2	2E-01	0.2
Narf	8E-04	0.2	Kdm3b	1E-02	0.2
Secisbp2	4E-04	0.2	Blzf1	8E-04	0.2
Cab39l	5E-07	0.2	Gm26887	1E-02	0.2
Jmjd1c	3E-08	0.2	Trim44	9E-08	0.2
Zbtb10	7E-03	0.2	Rps3a1	1E-55	0.2
Pigb	8E-02	0.2	Ppp1r35	1E-02	0.2
Traf3ip2	4E-02	0.2	Lncppara	1E-06	0.2
Aldh2	8E-15	0.2	Golgb1	4E-14	0.2
Zfp637	3E-12	0.2	Gabpb1	1E-01	0.2
Mtif3	6E-02	0.2	Ccdc91	2E-03	0.2
Higd1a	1E-21	0.2	Zfp280d	1E-05	0.2
Polr2c	4E-07	0.2	Grwd1	3E-03	0.2
Dnajb14	3E-06	0.2	Dusp6	6E-02	0.2
Morf4l1	5E-46	0.2	Nkapd1	2E-02	0.2
Cep41	1E-04	0.2	Bet1	6E-06	0.2
Rab11fip2	8E-06	0.2	Cep41	2E-02	0.2
Cwc22	3E-03	0.2	D16Ertd472e	7E-03	0.2
Atf4	4E-14	0.2	Lancl1	1E-04	0.2
Mapk8ip3	2E-01	0.2	Tmem185a	2E-02	0.2
Pde4b	2E-07	0.2	Ddx42	1E-07	0.2

Fibroblast					
Control			*Notch2*$^{tm1.1Ecan}$		
Gene	*p*	FC	Gene	*p*	FC
Ranbp3	2E-05	0.2	Hnrnpa2b1	1E-22	0.3
Zfpm2	7E-14	0.2	Setd2	9E-08	0.3
Tpd52	1E-02	0.2	Slc39a3	4E-02	0.3
BC051226	9E-01	0.2	Midn	2E-08	0.3
Fam92a	3E-09	0.2	Fancc	2E-06	0.3
Spata2	2E-01	0.2	Prrc2a	1E-09	0.3
Dtnbp1	1E-06	0.2	Pbrm1	2E-16	0.3
Ino80c	1E-01	0.2	Cenpx	3E-11	0.3
Spice1	3E-02	0.2	Zfp770	2E-02	0.3
Adarb1	8E-02	0.2	Abat	1E-02	0.3
Plekha2	7E-03	0.2	Mamld1	1E-01	0.3
Pdk2	1E-01	0.2	Axl	2E-10	0.3
Tbl3	5E-02	0.2	Gmps	3E-08	0.3
Derl2	3E-07	0.2	Galnt10	3E-06	0.3
Ncor2	3E-05	0.2	Ptges3	3E-13	0.3
Dtx2	1E-02	0.2	Blvrb	9E-07	0.3
Retreg1	2E-04	0.2	Mustn1	6E-07	0.3
Plcd1	2E-04	0.2	Nmrk1	1E-01	0.3
Rubcn	9E-05	0.2	Cnot6	2E-06	0.3
Kirrel	1E-08	0.2	Txn1	5E-17	0.3
Gm47283	1E-03	0.2	Itpkb	4E-03	0.3
Polg	2E-03	0.2	Ckap4	6E-13	0.3
Agbl3	2E-03	0.2	Il6st	1E-18	0.3
Flot2	3E-05	0.2	Lrba	5E-09	0.3
1700037H04Rik	1E-02	0.2	Ints1	7E-04	0.3
Crim1	4E-09	0.2	Pias2	2E-03	0.3
Snx13	7E-05	0.2	Atn1	4E-06	0.3
Zfp808	2E-02	0.2	Ddah2	6E-14	0.3
Lgmn	2E-07	0.2	Foxp2	1E-07	0.3
Dnajb2	1E-03	0.2	Dnal1	7E-03	0.3
Cpe	6E-12	0.2	Lca5	6E-04	0.3
Mapre2	2E-06	0.2	Casp6	3E-04	0.3
2700054A10Rik	1E-01	0.2	Heatr6	9E-04	0.3
Esyt2	3E-07	0.2	Wasf2	1E-13	0.3
Hoxa11	4E-02	0.2	B3gnt2	7E-06	0.3
Tnfsf12	1E-03	0.2	Gypc	2E-02	0.3
Traf7	3E-04	0.2	Casp8	2E-04	0.3
Dusp3	2E-04	0.2	Unc45a	2E-03	0.3
Haus2	4E-01	0.2	Creld2	8E-09	0.3
Ccdc39	8E-02	0.2	Cbx1	1E-13	0.3
Cops7b	7E-01	0.2	Fiz1	4E-02	0.3
Vps13c	1E-04	0.2	Tmtc3	6E-04	0.3
Psme2	9E-08	0.2	Pop7	1E-03	0.3
Pkig	4E-07	0.2	Serpinb9	6E-03	0.3
Psmb7	8E-08	0.2	Trip10	4E-03	0.3
Smg9	6E-02	0.2	Tram2	3E-09	0.3
Prkaa1	4E-02	0.2	Dync2h1	1E-07	0.3
Utp23	7E-02	0.2	Gpkow	4E-04	0.3
Zyx	2E-07	0.2	Deaf1	5E-05	0.3
Fan1	3E-01	0.2	G3bp1	2E-09	0.3
Mtmr14	7E-03	0.2	Irf1	4E-03	0.3
Cggbp1	5E-07	0.2	Tmem123	3E-06	0.3
Calr	1E-10	0.2	Irf2	3E-06	0.3
Gdpd1	1E-01	0.2	Bcl2l2	1E-01	0.3
Cox20	9E-08	0.2	Trps1	1E-16	0.3
Terf2	3E-03	0.2	Ddx19a	2E-03	0.3
Ppp1r2	3E-08	0.2	Slc10a7	4E-05	0.3
Tpi1	3E-13	0.2	Ehbp1l1	2E-05	0.3
Tle4	2E-02	0.2	Arhgap31	4E-07	0.3
Akap13	1E-12	0.2	Aifm2	7E-02	0.3
Srpk1	1E-03	0.2	Mad2l1bp	2E-04	0.3
Ppm1l	2E-02	0.2	Tnfsf12	1E-04	0.3
Hivep2	1E-06	0.2	Mpp7	2E-05	0.3
Rita1	7E-01	0.2	Memo1	7E-06	0.3

Undefined					
Control			*Notch2*$^{tm1.1Ecan}$		
Gene	*p*	FC	Gene	*p*	FC
Ccdc28b	7E-01	0.1	Atpaf2	3E-02	0.1
Rpl30	2E-17	0.1	Eif4a2	7E-04	0.1
Efl1	1E-02	0.1	Mbtps2	8E-06	0.1
Fundc1	2E-01	0.1	Pcmtd2	2E-05	0.1
Lrrc8a	7E-04	0.1	Rdh10	3E-02	0.1
Trmt12	1E-01	0.1	Islr	2E-03	0.1
Malat1	2E-15	0.1	Srsf10	2E-06	0.1
Mbnl2	1E-07	0.1	Ndufb6	9E-10	0.1
Ldha	2E-02	0.1	Cnbp	3E-06	0.1
Gtpbp3	9E-04	0.1	Eif2a	3E-09	0.1
Nelfcd	9E-04	0.1	Pex19	5E-11	0.1
Psip1	7E-01	0.1	Luc7l	4E-11	0.1
Mapk1ip1	5E-01	0.1	Mat2b	9E-13	0.1
Prss36	9E-01	0.1	Tmem14a	2E-05	0.1
Fuz	7E-01	0.1	App	1E-08	0.1
Surf2	9E-01	0.1	Ubald2	7E-10	0.1
Rexo2	9E-02	0.1	Rps29	1E-13	0.1
Mrpl24	9E-01	0.1	Ndufb1-ps	8E-01	0.1
Dgcr6	2E-03	0.1	Maea	3E-09	0.1
B930095G15Rik	2E-01	0.1	Eif2b3	5E-05	0.1
Wdr83os	1E-01	0.1	Ufsp2	2E-08	0.1
Snrpn	9E-01	0.1	Ddb2	6E-02	0.1
Pmf1	6E-01	0.1	Lsm5	7E-08	0.1
Gm16599	9E-01	0.1	Secisbp2	7E-05	0.1
Ccdc66	3E-01	0.1	Rplp1	2E-19	0.1
Nt5c2	4E-01	0.1	Recql	1E-04	0.1
Mapkap1	9E-01	0.1	Psmg3	9E-11	0.1
Zfp706	7E-01	0.1	Iscu	1E-10	0.1
Dcaf5	2E-01	0.1	Heatr3	3E-03	0.1
Yae1d1	9E-01	0.1	Fgfr1op2	2E-09	0.1
Timm9	1E+00	0.1	Rpl35	4E-08	0.1
Plk3	5E-01	0.1	Eid2	3E-07	0.1
Paxbp1	6E-01	0.1	Zfp423	3E-02	0.1
Ostc	5E-01	0.1	Dis3	3E-05	0.1
Rpl21	3E-17	0.1	Pde4b	1E-02	0.1
Mynn	3E-02	0.1	Vasp	9E-09	0.1
Trim37	9E-01	0.1	Sars	3E-03	0.1
Araf	9E-01	0.1	Mrps35	9E-10	0.1
Rab33b	7E-01	0.1	Rps13	2E-11	0.1
Mrpl18	1E-01	0.1	Fam118b	1E-03	0.1
Polr2c	8E-01	0.1	Polr2c	7E-07	0.1
Stk25	8E-03	0.1	Phf8	2E-07	0.1
Rps27rt	4E-01	0.1	Rpl7a	9E-05	0.1
Ift20	1E-02	0.1	Gm14966	7E-02	0.1
Rpl36al	1E-05	0.1	Snhg6	2E-06	0.1
Slc35e2	4E-01	0.1	Cul2	4E-04	0.1
Pithd1	6E-01	0.1	Naa16	4E-05	0.1
Diablo	5E-01	0.1	Gadd45gip1	4E-08	0.1
Rplp1	1E-17	0.1	Nrep	4E-01	0.1
Uba5	5E-02	0.1	Wdr70	2E-08	0.1
Ubxn6	3E-02	0.1	B3gnt2	1E-03	0.1
Zfpm2	6E-03	0.1	2900076A07Rik	4E-05	0.1
Mrpl53	5E-04	0.1	Psmb3	3E-07	0.1
Gxylt1	2E-07	0.1	Cct6a	3E-09	0.1
Gpn3	2E-01	0.1	Yars	2E-06	0.1
Micu1	2E-01	0.1	Itgb3bp	2E-04	0.1
Ndufaf7	2E-03	0.1	Csf2ra	4E-02	0.1
Gtf2i	9E-01	0.1	Uqcc1	6E-05	0.1
Bola3	5E-01	0.1	Lmo4	3E-02	0.1
Dnajc16	2E-02	0.1	Ip6k1	3E-07	0.1
Wars2	6E-01	0.1	Fam204a	2E-05	0.1
Rpap3	1E-03	0.1	Selenof	2E-01	0.1
Cux1	5E-01	0.1	Nadk2	3E-02	0.1
Gypc	3E-02	0.1	Mrpl36	5E-09	0.1

Articular/Synovial Fibroblast					
Control			*Notch2*$^{tm1.1Ecan}$		
Gene	*p*	FC	Gene	*p*	FC
Klhl28	6E-02	0.3	Phip	1E-05	0.3
Sorbs3	2E-02	0.3	Uvrag	2E-06	0.3
Tesk2	4E-02	0.3	Malt1	8E-01	0.3
Alpk1	3E-02	0.3	Inip	2E-01	0.3
Layn	2E-03	0.3	Dhrs9	6E-03	0.3
Tmem219	6E-05	0.3	Zfp944	2E-02	0.3
Calm3	2E-13	0.3	Eno2	1E-01	0.3
Fut11	2E-04	0.3	Entpd5	3E-01	0.3
Rftn2	6E-05	0.3	Fam110b	2E-04	0.3
Numb	5E-06	0.3	Nckap1	1E-06	0.3
Mapre2	5E-11	0.3	Gmpr	7E-01	0.3
Pip4k2b	3E-01	0.3	Blvrb	5E-05	0.3
Rgl2	3E-02	0.3	Arhgap23	3E-03	0.3
Sirt1	9E-03	0.3	Plcb3	1E-03	0.3
Slc36a1	7E-02	0.3	Ccpg1	5E-05	0.3
Il6ra	2E-02	0.3	Pot1b	7E-01	0.3
Actr8	6E-01	0.3	Gli3	7E-10	0.3
Spg11	3E-03	0.3	Champ1	1E-01	0.3
Oxsr1	6E-02	0.3	Slc25a24	8E-03	0.3
Rbms2	1E-07	0.3	Alas1	1E-02	0.3
Fbxo34	6E-02	0.3	Atg10	3E-07	0.3
Champ1	8E-03	0.3	Pafah2	1E-02	0.3
Pdha1	2E-11	0.3	Gm14325	7E-04	0.3
Samd8	1E-02	0.3	Mpv17l2	6E-04	0.3
Nckap1	4E-13	0.3	Stam	1E-02	0.3
Ydjc	2E-01	0.3	Cnppd1	4E-03	0.3
Emp3	4E-26	0.3	Gm26532	1E-02	0.3
Gemin8	2E-01	0.3	Rtn3	6E-08	0.3
Ppm1m	9E-02	0.3	Coa5	1E-01	0.3
Atp6v0d1	6E-12	0.3	Trp53inp2	3E-03	0.3
Arntl	8E-02	0.3	Trim24	3E-02	0.3
Pacs2	4E-05	0.3	Adcy6	4E-01	0.3
Mmut	1E-02	0.3	Clcn7	3E-03	0.3
Exoc2	4E-05	0.3	2700081O15Rik	8E-03	0.3
Zfp553	3E-01	0.3	Zfp846	1E-01	0.3
Strip1	7E-02	0.3	Tle3	4E-01	0.3
Tcf12	2E-13	0.3	Gstt3	5E-03	0.3
Arhgdib	9E-06	0.3	Ckb	2E-08	0.3
Gm2a	1E-06	0.3	Sp1	3E-02	0.3
Coa4	2E-02	0.3	Gm17501	2E-02	0.3
Bag1	3E-13	0.3	Glt8d2	1E-01	0.3
Khk	3E-02	0.3	Mapkapk3	2E-01	0.3
Trp53bp2	3E-02	0.3	Pom121	6E-02	0.3
Otud1	2E-03	0.3	Gm2a	1E-05	0.3
Cenpv	2E-02	0.3	Ncoa2	2E-04	0.3
Tsc1	4E-05	0.3	Wnt5b	5E-01	0.3
Dyrk1b	7E-02	0.3	Ercc3	8E-03	0.3
Apc	7E-09	0.3	Ppox	8E-01	0.3
Klhl7	1E-03	0.3	Zfp809	9E-02	0.3
Trim11	1E-01	0.3	Gzf1	9E-02	0.3
Piezo1	9E-05	0.3	Dis3l2	1E-03	0.3
Ermp1	8E-02	0.3	Rasa1	2E-05	0.3
Zfp52	3E-01	0.3	Lyrm1	2E-01	0.3
Etfdh	2E-01	0.3	Zfp810	7E-02	0.3
Exoc4	2E-12	0.3	Mar2	1E-03	0.3
Ptpn21	3E-03	0.3	Scyl3	1E-01	0.3
5730409E04Rik	4E-03	0.3	Ick	2E-05	0.3
Herc4	4E-06	0.3	Ankrd12	4E-11	0.3
Tpst2	8E-03	0.3	Calm3	4E-09	0.3
Irf9	5E-02	0.3	Smad3	8E-05	0.3
Htra1	4E-08	0.3	Trappc9	8E-04	0.3
Mpv17l2	1E-03	0.3	Ppard	4E-03	0.3
Ski	7E-15	0.3	Fryl	2E-03	0.3
Cant1	5E-03	0.3	Jund	2E-15	0.3

Limb Mesenchyme					
Control			*Notch2*$^{tm1.1Ecan}$		
Gene	*p*	FC	Gene	*p*	FC
Dpm2	9E-05	0.2	Socs4	4E-03	0.2
Cyb561d2	2E-02	0.2	Nhp2	5E-07	0.2
Rmi1	3E-01	0.2	Smarca1	1E-06	0.2
Adam9	4E-04	0.2	Gm15867	5E-05	0.2
Tmed3	2E-17	0.2	Gars	2E-16	0.2
Lztr1	2E-03	0.2	Ttc23	5E-05	0.2
Zfp931	1E-01	0.2	Abraxas2	2E-08	0.2
Rps5	9E-42	0.2	Cltb	5E-07	0.2
Dis3l	3E-02	0.2	Wiz	2E-03	0.2
Sgcd	4E-07	0.2	Slc3a2	3E-15	0.2
R3hdm4	4E-04	0.2	Mrps36	9E-10	0.2
Cyth2	4E-04	0.2	Pbx3	3E-06	0.2
Tbrg1	1E-17	0.2	Mettl2	2E-03	0.2
Anapc2	4E-04	0.2	Trappc5	2E-04	0.2
Acadl	2E-13	0.2	St6galnac6	5E-06	0.2
Tex264	6E-07	0.2	Rcor2	2E-03	0.2
Sfr1	2E-23	0.2	Erbin	1E-09	0.2
Mfap3	7E-02	0.2	Dlst	2E-05	0.2
Gm49692	2E-01	0.2	Fibp	4E-08	0.2
Rps10	6E-54	0.2	Sike1	7E-06	0.2
Washc2	1E-06	0.2	Med29	4E-06	0.2
Cmas	2E-02	0.2	Xpa	2E-09	0.2
Gm10076	3E-24	0.2	Myo1c	1E-09	0.2
Mlx	5E-03	0.2	Pih1d1	2E-06	0.2
Gltp	7E-02	0.2	Dr1	6E-03	0.2
St6galnac6	2E-03	0.2	Serinc1	6E-16	0.2
Col5a3	3E-17	0.2	Nfix	9E-17	0.2
H2afy2	7E-06	0.2	Mrps24	3E-09	0.2
Fam3a	9E-04	0.2	H2afy2	2E-08	0.2
Mocs2	2E-05	0.2	Ppp2r5b	7E-03	0.2
Polr3d	2E-04	0.2	Lrrc59	7E-08	0.2
AI597479	8E-01	0.2	Rps28	1E-47	0.2
Slc39a13	3E-06	0.2	Smarca2	1E-10	0.2
Maged1	2E-16	0.2	Stam2	3E-09	0.2
Tram1	5E-07	0.2	Cript	3E-08	0.2
Vps29	5E-10	0.2	Gm2000	4E-11	0.2
Rnf215	4E-02	0.2	Slc25a16	7E-06	0.2
Rps18	5E-46	0.2	Rpl12	4E-32	0.2
Hacd4	9E-06	0.2	Mapk3	9E-10	0.2
Birc3	8E-02	0.2	Arhgap42	3E-07	0.2
Rce1	2E-02	0.2	Fbh1	3E-03	0.2
Tmem50a	5E-16	0.2	Rnf2	4E-03	0.2
Atpif1	4E-23	0.2	Acss2	4E-02	0.2
Mars	3E-03	0.2	Ncor2	3E-08	0.2
Epg5	2E-01	0.2	Tanc1	7E-06	0.2
Gpatch11	3E-03	0.2	Dync1li1	2E-07	0.2
Apool	2E-02	0.2	Hgs	3E-04	0.2
Ipo4	2E-02	0.2	Rbm14	1E-02	0.2
Zfp651	8E-02	0.2	Cfdp1	1E-17	0.2
Sall2	8E-02	0.2	Cpeb1	5E-06	0.2
Derl1	3E-08	0.2	Ccdc82	2E-04	0.2
Rpl22l1	2E-25	0.2	Glce	1E-07	0.2
Ltbr	3E-03	0.2	Ap4b1	4E-02	0.2
Tmod3	1E-04	0.2	Smarcc2	1E-06	0.2
Lca5	7E-02	0.2	Fn1	1E-21	0.2
Sike1	2E-03	0.2	Tcea3	3E-08	0.2
Srp19	3E-13	0.2	Gm14305	7E-03	0.2
Eml3	6E-02	0.2	Nr2f6	4E-10	0.2
Sart3	4E-01	0.2	Timm13	3E-17	0.2
Nsa2	1E-17	0.2	Plk3	4E-02	0.2
Usp42	4E-02	0.2	B230354K17Rik	2E-04	0.2
Bcl9l	2E-05	0.2	Abhd13	3E-02	0.2
Erbin	2E-05	0.2	Fam120b	1E-02	0.2
Atp13a1	3E-01	0.2	Gmppa	4E-07	0.2

Chondrogenic					
Control			*Notch2*$^{tm1.1Ecan}$		
Gene	*p*	FC	Gene	*p*	FC
Mrpl20	1E-14	0.2	Pomt1	1E+00	0.2
Dvl1	6E-02	0.2	Akt2	2E-05	0.2
Jpt2	3E-01	0.2	Med26	8E-03	0.2
Ttc5	7E-06	0.2	Dcaf12	2E-02	0.2
Ttyh2	3E-02	0.2	Decr1	1E-05	0.2
Lncpint	1E-03	0.2	Ddx46	3E-06	0.2
Gm19710	2E-02	0.2	Ccdc90b	2E-08	0.2
Ptprg	4E-06	0.2	Tomm7	1E-21	0.2
Tmem161b	1E-01	0.2	Prkab1	8E-02	0.2
Map3k3	5E-04	0.2	Micu3	4E-03	0.2
H3f3b	2E-32	0.2	Zranb2	1E-08	0.2
Kbtbd3	7E-02	0.2	Cwc22	7E-04	0.2
Xndc1	2E-02	0.2	Tars	2E-06	0.2
Elp1	4E-04	0.2	Myg1	7E-04	0.2
Snu13	2E-13	0.2	Tbl1x	5E-10	0.2
Fundc1	9E-06	0.2	Slc25a40	8E-02	0.2
Pak1ip1	1E-09	0.2	Taf1a	2E-01	0.2
Cnbp	4E-13	0.2	Mycbp	3E-03	0.2
Zfp398	4E-02	0.2	Coq9	1E-03	0.2
Zfp266	2E-05	0.2	Vezt	5E-02	0.2
Btbd8	5E-01	0.2	Rsrc2	7E-13	0.2
Snhg18	2E-18	0.2	Pafah2	8E-01	0.2
Gm26887	1E-02	0.2	Ypel2	1E-01	0.2
Zfp113	8E-03	0.2	Gripap1	3E-04	0.2
Hnrnph1	2E-06	0.2	Capn15	1E-01	0.2
Gtpbp2	9E-03	0.2	Xpo5	1E-02	0.2
Cirbp	2E-03	0.2	Vapa	2E-14	0.2
6030458C11Rik	3E-01	0.2	Tmem87a	5E-02	0.2
Slc33a1	5E-05	0.2	Ssx2ip	9E-03	0.2
Nfya	2E-01	0.2	Fam234a	2E-02	0.2
Amfr	5E-04	0.2	Rpl30	3E-43	0.2
Naa25	9E-02	0.2	Ide	5E-06	0.2
1810026B05Rik	3E-07	0.2	Txndc12	6E-07	0.2
Klc3	3E-01	0.2	Ebag9	8E-03	0.2
Prdx6	3E-07	0.2	Crim1	6E-10	0.2
Lrrfip2	2E-05	0.2	Cux1	3E-05	0.2
Ints13	1E-02	0.2	1810009A15Rik	1E-02	0.2
Morn2	7E-05	0.2	Ints6	1E-02	0.2
Hspa14	4E-03	0.2	Tatdn1	2E-03	0.2
Acbd6	3E-06	0.2	Klhl20	6E-01	0.2
Tars2	1E-01	0.2	Wdfy2	3E-03	0.2
Taf4b	1E-02	0.2	Pnpla8	1E-05	0.2
Dtnb	3E-04	0.2	Rbm48	2E-03	0.2
Prdx3	2E-04	0.2	Ppip5k1	4E-03	0.2
Commd3	6E-15	0.2	Rnmt	2E-02	0.2
Osbpl10	5E-01	0.2	Eif4e	7E-10	0.2
Evi5l	2E-01	0.2	Zgpat	9E-02	0.2
Tgif1	4E-03	0.2	Rcn2	6E-08	0.2
Dcaf12	7E-05	0.2	Slc30a5	4E-03	0.2
Paip2	2E-19	0.2	Rpl36a	1E-28	0.2
Prps1	7E-02	0.2	Tmem98	3E-02	0.2
Mettl9	3E-12	0.2	Jade1	2E-04	0.2
Nub1	8E-06	0.2	Hgsnat	2E-02	0.2
Ddx19b	2E-01	0.2	Pds5b	3E-03	0.2
Rbm22	2E-04	0.2	BC002059	2E-01	0.2
4833420G17Rik	4E-03	0.2	mt-Co3	6E-32	0.2
Tmbim4	5E-10	0.2	Rps24	1E-39	0.2
Fbxl12	8E-03	0.2	Il16	2E-06	0.2
Gm10563	3E-01	0.2	Sap130	1E-01	0.2
Ppp2r3a	8E-02	0.2	Ppp1r11	3E-09	0.2
Pex6	2E-02	0.2	Recql5	2E-02	0.2
Smox	1E-03	0.2	Sirt3	3E-02	0.2
Cox7c	1E-46	0.2	Zmynd11	1E-08	0.2
Vps37a	1E-07	0.2	Strada	2E-02	0.2

Fibroblast					
Control			*Notch2*$^{tm1.1Ecan}$		
Gene	*p*	FC	Gene	*p*	FC
Kifc3	2E-01	0.2	Slc25a27	1E-03	0.3
Twist1	8E-06	0.2	Thoc3	1E-04	0.3
Tent4a	6E-02	0.2	Pou2f1	4E-03	0.3
Mapk8ip1	6E-01	0.2	Anapc5	3E-14	0.3
Ppm1g	9E-05	0.2	Tmem184c	3E-03	0.3
Nono	6E-07	0.2	Ppp1ca	3E-20	0.3
Ugdh	3E-06	0.2	Grwd1	7E-03	0.3
Nlk	3E-03	0.2	6430590A07Rik	1E-02	0.3
Zfp934	1E-03	0.2	Gm49969	4E-03	0.3
C2cd2	1E-01	0.2	Zfp687	6E-03	0.3
Pofut1	2E-02	0.2	Smg6	1E-09	0.3
Rara	7E-04	0.2	Ap2s1	5E-14	0.3
Fh1	4E-04	0.2	Vrk3	5E-02	0.3
Selenon	8E-03	0.2	Dstn	1E-16	0.3
Zmiz2	5E-02	0.2	Ppp1r9b	2E-02	0.3
Impa1	3E-04	0.2	Emc9	2E-03	0.3
Htra2	4E-05	0.2	Rad1	7E-02	0.3
Tmem141	3E-01	0.2	Bcar1	2E-05	0.3
Zc3h4	2E-05	0.2	Fscn1	5E-06	0.3
Idh3a	3E-05	0.2	Nkap	5E-07	0.3
Bivm	1E-01	0.2	Glt8d2	2E-03	0.3
Zbtb18	1E-01	0.2	Cwc22	7E-04	0.3
Kdm2b	4E-02	0.2	Prpf38a	7E-06	0.3
Spag9	1E-14	0.2	Fam189b	5E-03	0.3
Rbm41	5E-02	0.2	Ppme1	8E-03	0.3
B230216N24Rik	1E-01	0.2	Asah2	3E-02	0.3
Tnfaip8	4E-04	0.2	Hace1	3E-03	0.3
Sephs1	2E-02	0.2	Mrpl28	6E-09	0.3
Polb	4E-04	0.2	Ipo5	1E-06	0.3
Msl2	2E-02	0.2	Fam118a	1E-02	0.3
Wdr37	3E-03	0.2	Fryl	3E-04	0.3
Pop7	6E-03	0.2	Cep68	1E-04	0.3
Calm2	3E-06	0.2	Itm2a	2E-20	0.3
Snx1	2E-05	0.2	Fntb	4E-04	0.3
Ruvbl1	4E-04	0.2	Luc7l3	1E-13	0.3
Ilf3	8E-04	0.2	Pdia5	6E-08	0.3
Ints3	1E-02	0.2	Psmd14	2E-08	0.3
Slc4a2	1E-03	0.2	Ap2a1	2E-07	0.3
Zfp958	3E-01	0.2	Cenpv	3E-04	0.3
Oga	3E-06	0.2	Megf8	2E-03	0.3
Mrpl33	4E-13	0.2	Rps6kb2	3E-03	0.3
Bpnt1	4E-01	0.2	Prnp	4E-15	0.3
Nudt13	6E-02	0.2	Myo18a	1E-06	0.3
Hace1	1E-02	0.2	Irf2bpl	3E-09	0.3
Zfp821	1E-01	0.2	Klhl12	3E-03	0.3
Dleu2	1E-04	0.2	Dlg4	5E-04	0.3
Mus81	1E-01	0.2	Ythdf2	1E-08	0.3
Qtrt2	4E-01	0.2	Prdx4	6E-12	0.3
Bms1	2E-04	0.2	Eif2d	4E-03	0.3
Tmem65	2E-04	0.2	Pakap.1	1E-07	0.3
Kif3b	2E-02	0.2	Magt1	1E-12	0.3
Eaf1	1E-01	0.2	Mrpl13	5E-06	0.3
Fndc3b	8E-15	0.2	Cstf3	3E-06	0.3
Tmem29	2E-02	0.2	Endov	2E-01	0.3
Eno3	6E-03	0.2	Srsf4	5E-07	0.3
Anapc2	4E-04	0.2	Rpap2	1E-01	0.3
Adgrl2	7E-12	0.2	Hoxc10	1E-08	0.3
Cyb5r4	1E-03	0.2	Vps13d	8E-08	0.3
Trmt12	6E-01	0.2	Tfeb	2E-01	0.3
Nmt2	5E-03	0.2	Pias1	4E-07	0.3
Wnk1	9E-08	0.2	Notch2	2E-07	0.3
Tst	2E-03	0.2	Cpeb4	1E-05	0.3
Nelfcd	1E-02	0.2	Bcl2	5E-03	0.3
Coq4	6E-02	0.2	Nfic	1E-12	0.3

Undefined					
Control			*Notch2*$^{tm1.1Ecan}$		
Gene	*p*	FC	Gene	*p*	FC
Pin1	1E-01	0.1	Pcca	3E-05	0.1
1700016P03Rik	7E-01	0.1	Ndufa11	9E-02	0.1
Pycr2	2E-01	0.1	Abhd14a	1E-09	0.1
Khnyn	5E-03	0.1	Mzt2	2E-06	0.1
Mdh1	6E-01	0.1	Btd	2E-07	0.1
Tmem101	2E-01	0.1	Ift20	2E-02	0.1
Wdr70	2E-02	0.1	Tatdn3	8E-05	0.1
Polh	2E-03	0.1	Rps19	2E-09	0.1
Stx3	3E-01	0.1	Rps14	3E-11	0.1
Limd1	2E-02	0.1	Rnpc3	6E-07	0.1
Faf1	3E-02	0.1	Eif1ax	4E-11	0.1
Chac2	1E-02	0.1	Cops3	1E-07	0.1
Bzw2	7E-01	0.1	Prepl	9E-04	0.1
Xpot	7E-01	0.1	Atic	4E-06	0.1
Wtap	4E-02	0.1	Nphp3	7E-05	0.1
Rubcn	4E-01	0.1	Mar6	3E-07	0.1
Cpq	5E-03	0.1	4933406I18Rik	2E-01	0.1
Rfesd	8E-01	0.1	Rbm8a	5E-09	0.1
Mrpl11	1E-02	0.1	Lamtor3	1E-08	0.1
Polg	1E-01	0.1	Ythdf1	1E-10	0.1
Slc25a36	1E+00	0.1	Znrd2	4E-07	0.1
Cbr2	1E-01	0.1	Cbx3	5E-11	0.1
Pfkm	5E-01	0.1	Dbt	8E-03	0.1
Pus3	7E-01	0.1	Trib1	2E-01	0.1
Gpr89	6E-01	0.1	Tmem39b	2E-04	0.1
Rabgef1	9E-03	0.1	Mtfr1	1E-02	0.1
Sdhaf1	7E-01	0.1	Slc27a4	7E-04	0.1
Sec61b	7E-05	0.1	Pitpnm2	4E-03	0.1
6030443J06Rik	3E-01	0.1	H2-Ke6	5E-05	0.1
Xpo5	6E-01	0.1	Tusc2	1E-04	0.1
Anxa4	1E+00	0.1	Lmbrd1	4E-08	0.1
Atp2b1	6E-03	0.1	Rpl8	1E-08	0.1
Lancl2	5E-02	0.1	Nf2	9E-09	0.1
Pitx1	1E-01	0.1	Bola3	7E-05	0.1
Pgm1	9E-01	0.1	Zfp780b	2E-06	0.1
Slc35e1	5E-01	0.1	Dctn3	9E-18	0.1
Strada	5E-01	0.1	Utp15	8E-04	0.1
Coprs	7E-01	0.1	Cpeb4	5E-07	0.1
Dcaf11	4E-01	0.1	Cycs	2E-06	0.1
Per1	9E-01	0.1	Limd2	6E-07	0.1
Man1b1	4E-02	0.1	Ost4	4E-02	0.1
Pigg	3E-01	0.1	Ndufa7	9E-01	0.1
Ap5m1	2E-02	0.1	4933434E20Rik	8E-04	0.1
Tex9	2E-01	0.1	Pdgfa	7E-04	0.1
Tenm1	4E-01	0.1	Nt5c3b	4E-08	0.1
Cfl2	2E-01	0.1	Commd3	1E-06	0.1
Ngdn	9E-02	0.1	Cdk17	2E-02	0.1
Atg16l1	3E-02	0.1	Zcchc4	3E-04	0.1
Mesd	2E-01	0.1	Mlh1	2E-02	0.1
Ttc14	2E-01	0.1	Zmpste24	4E-09	0.1
Ndufa1	1E+00	0.1	Twsg1	3E-03	0.1
Cox7a2l	3E-02	0.1	Tmem67	8E-05	0.1
Vps51	9E-01	0.1	Fkbp9	2E-06	0.1
Zfp239	7E-01	0.1	Ndufa2	9E-01	0.1
Phip	5E-02	0.1	Stxbp3	8E-15	0.1
Phf7	3E-01	0.1	Eps15	4E-06	0.1
Mybbp1a	8E-01	0.1	Ndufaf2	4E-08	0.1
Nars2	6E-01	0.1	Zfr	3E-04	0.1
Ubl3	4E-03	0.1	Lrrfip2	6E-05	0.1
Polr1c	7E-02	0.1	Akap8l	5E-04	0.1
Gm15261	2E-01	0.1	Psmd12	8E-09	0.1
Klc4	2E-01	0.1	Trmt10b	7E-05	0.1
Wdr27	5E-01	0.1	Mras	5E-04	0.1
P3h4	9E-02	0.1	Cers2	1E-07	0.1

Articular/Synovial Fibroblast					
Control			*Notch2*$^{tm1.1Ecan}$		
Gene	*p*	FC	Gene	*p*	FC
Tgfbr1	1E-04	0.3	Zfp358	7E-02	0.3
1110019D14Rik	7E-03	0.3	Pon3	8E-04	0.3
Fam219a	2E-04	0.3	Pou6f1	1E-01	0.3
Spata24	2E-04	0.3	Nt5dc3	1E-03	0.3
Oxct1	9E-16	0.3	Lamp2	4E-19	0.3
Vps26a	3E-05	0.3	Wdtc1	2E-03	0.3
Prelp	7E-04	0.3	Pml	2E-02	0.3
Hadhb	6E-04	0.3	Stx2	7E-03	0.3
Gga2	2E-05	0.3	Tgfbr1	1E-03	0.3
Angptl4	2E-02	0.3	Zfp994	1E-02	0.3
Ncoa7	4E-04	0.3	Stat5a	3E-01	0.3
Sat2	3E-01	0.3	Szt2	1E-01	0.3
Rhno1	6E-03	0.3	Fbxl3	2E-04	0.3
Ccdc32	1E-02	0.3	Tnk2	6E-01	0.3
Dclk1	6E-12	0.3	Kif26b	1E-01	0.3
Kpna6	3E-03	0.3	Mrc2	1E-08	0.3
Atxn1l	1E-01	0.3	Ccdc9	3E-02	0.3
Tnip2	2E-01	0.3	Ttf1	2E-02	0.3
Fdx1	3E-05	0.3	Zmiz1	2E-09	0.3
Tbc1d13	9E-03	0.3	Abcc1	2E-03	0.3
Rrm2b	8E-03	0.3	Dcaf10	7E-03	0.3
E130307A14Rik	2E-03	0.3	Slc48a1	3E-03	0.3
Gpcpd1	7E-04	0.3	Zbtb37	8E-02	0.3
Ice1	1E-01	0.3	Smc5	5E-02	0.3
Tbx5	1E-01	0.3	Tbx4	9E-03	0.3
Vps13a	1E-05	0.3	Slc36a4	6E-01	0.3
Aar2	1E-01	0.3	Smad6	8E-04	0.3
Ifngr1	5E-05	0.3	Limch1	1E-02	0.3
Cep104	3E-02	0.3	Synm	7E-02	0.3
Lrwd1	1E-01	0.3	Amotl2	3E-06	0.3
Lrig1	1E-01	0.3	Clip3	2E-05	0.3
Armc7	2E-01	0.3	Bmpr2	4E-06	0.3
Slc25a23	5E-02	0.3	Tmem50a	9E-13	0.3
Tmem50a	1E-16	0.3	Tubb2b	8E-03	0.3
Nlgn2	1E-01	0.3	Phka2	1E-02	0.3
Prkaa1	1E-02	0.3	Dazap2	5E-06	0.3
Ppp1cc	3E-09	0.3	Acvr2a	2E-05	0.3
Kif3a	1E-04	0.3	Gm17056	5E-01	0.3
Natd1	7E-02	0.3	Abcb10	2E-01	0.3
Fbxo28	3E-02	0.3	Uaca	7E-03	0.3
Pvt1	2E-09	0.3	Cd81	6E-20	0.3
Synm	2E-01	0.3	Ppfibp2	1E-01	0.3
Lzts2	7E-07	0.3	Rad52	3E-01	0.3
Spidr	4E-05	0.3	Ccdc50	2E-05	0.3
Hpcal1	3E-03	0.3	Meis2	3E-04	0.3
Txnip	1E-04	0.3	Appl2	1E-05	0.3
Pbxip1	8E-11	0.3	Slc29a3	5E-02	0.3
Slc6a8	4E-02	0.3	Wdr59	7E-01	0.3
Plekha5	9E-03	0.3	Sgpl1	6E-03	0.3
Sirt7	3E-02	0.3	Vps9d1	1E-01	0.3
Scly	1E-01	0.3	Parp6	5E-02	0.3
Dapp1	3E-01	0.3	Cic	4E-02	0.3
Nrde2	1E-02	0.3	Prkra	8E-05	0.3
Fam53c	2E-01	0.3	Plp2	6E-04	0.3
Vps29	2E-11	0.3	Smad1	1E-02	0.3
Gm49359	7E-03	0.3	Platr25	6E-03	0.3
Cebpd	6E-04	0.3	Rock2	3E-09	0.3
Cln5	2E-04	0.3	Gas6	3E-09	0.3
Taf8	4E-02	0.3	Cdk19	3E-02	0.3
Psme2	3E-10	0.3	Slc49a4	1E-02	0.3
Tbc1d14	3E-02	0.3	Htra2	3E-02	0.3
Col5a1	6E-16	0.3	Add1	4E-08	0.3
Grb14	5E-11	0.3	Mfap2	3E-02	0.3
Rgmb	3E-03	0.3	Ttc39c	3E-01	0.3

Limb Mesenchyme					
Control			*Notch2*$^{tm1.1Ecan}$		
Gene	*p*	FC	Gene	*p*	FC
Fzd2	4E-07	0.2	Fam160b2	3E-02	0.2
Phb	2E-03	0.2	Efr3a	2E-10	0.2
Alas1	2E-04	0.2	Dazap1	2E-11	0.2
Ikzf2	4E-02	0.2	Nudt3	4E-07	0.2
Golt1b	4E-06	0.2	Nubp2	5E-03	0.2
Zfp606	5E-01	0.2	Elp5	6E-07	0.2
Rpl27a	3E-52	0.2	Mmp28	5E-03	0.2
Gm14305	9E-03	0.2	Rpl18a	2E-39	0.2
Ptprd	3E-02	0.2	Cebpg	2E-10	0.2
AI480526	2E-01	0.2	Prkar1a	2E-11	0.2
Ampd2	2E-02	0.2	Mthfd2l	1E-04	0.2
Gadd45gip1	1E-06	0.2	Eid1	3E-13	0.2
Fam228b	3E-01	0.2	Stat3	2E-09	0.2
Fbxo10	7E-02	0.2	Cwc25	8E-06	0.2
D030056L22Rik	3E-01	0.2	Dpp9	3E-04	0.2
Usf1	1E-02	0.2	Map2k7	1E-04	0.2
Hspbp1	2E-02	0.2	Josd2	5E-07	0.2
Tars	5E-05	0.2	Nbea	2E-07	0.2
Arfip2	2E-03	0.2	Irak1	6E-07	0.2
Mdp1	1E-03	0.2	Psmg3	8E-08	0.2
2810433D01Rik	2E-01	0.2	Nfib	2E-08	0.2
Evl	7E-03	0.2	Bola2	4E-13	0.2
Rpl39	1E-57	0.2	Ifnar2	4E-07	0.2
Mlxip	9E-02	0.2	Nectin3	4E-09	0.2
Ifnar2	3E-07	0.2	2510039O18Rik	3E-07	0.2
Tyw5	2E-01	0.2	Ndufa9	7E-10	0.1
Man2a1	9E-06	0.2	Lysmd2	2E-03	0.1
Prrg1	2E-02	0.2	Ap2s1	3E-12	0.1
Mcat	5E-02	0.2	Iqce	2E-02	0.1
Zfp580	4E-02	0.2	Scamp3	3E-06	0.1
Tatdn1	1E-04	0.2	Grina	2E-12	0.1
Rnpepl1	3E-02	0.2	Itgb1	1E-23	0.1
Sox12	1E-01	0.2	Pank3	8E-05	0.1
Edem1	1E-04	0.2	Mfn1	3E-03	0.1
Jazf1	6E-02	0.2	Zyx	6E-14	0.1
Mrpl45	2E-03	0.2	Hes1	8E-07	0.1
Sertad1	1E-07	0.1	Cdk4	8E-17	0.1
Maz	2E-04	0.1	Ankrd42	2E-02	0.1
Ubtf	4E-05	0.1	Hs6st2	1E-10	0.1
Myof	2E-05	0.1	Epn2	3E-06	0.1
Igfbp7	6E-29	0.1	Flywch1	1E-03	0.1
Cand2	4E-01	0.1	Acads	2E-06	0.1
Dusp7	6E-02	0.1	Bag5	9E-09	0.1
Egfr	5E-09	0.1	Sar1a	1E-13	0.1
Slc25a32	2E-02	0.1	Tubb5	8E-47	0.1
Dusp23	2E-02	0.1	Pus1	2E-05	0.1
Prxl2b	3E-04	0.1	Pef1	4E-07	0.1
Egfl7	1E-01	0.1	Gnas	4E-11	0.1
Gmpr2	8E-02	0.1	Prpf4	5E-02	0.1
Eml4	1E-03	0.1	Atf5	5E-17	0.1
Gm13483	5E-01	0.1	Smarcd3	7E-06	0.1
Kbtbd2	2E-01	0.1	Acsl4	1E-03	0.1
Zmym3	2E-01	0.1	Plgrkt	8E-08	0.1
3300002I08Rik	3E-02	0.1	Mar2	9E-08	0.1
Mvp	4E-04	0.1	Gadd45b	5E-05	0.1
Xpa	7E-04	0.1	Ext1	1E-13	0.1
Dok5	1E-01	0.1	Fam102b	3E-09	0.1
Thbs3	6E-04	0.1	Tfpt	2E-04	0.1
Acvr1	1E-04	0.1	Nin	9E-06	0.1
Cd34	6E-20	0.1	Ccdc97	3E-01	0.1
Zfp358	6E-02	0.1	Tmbim6	2E-09	0.1
Serpinh1	1E-19	0.1	Klhl5	5E-06	0.1
Chka	5E-04	0.1	Matn4	6E-01	0.1
Rpl35	3E-38	0.1	Egfr	4E-12	0.1

Chondrogenic					
Control			*Notch2*$^{tm1.1Ecan}$		
Gene	*p*	FC	Gene	*p*	FC
Aff3	5E-19	0.2	Slc22a21	5E-01	0.2
Porcn	7E-01	0.2	Wsb1	9E-05	0.2
Vwa8	2E-03	0.2	Actl6a	9E-06	0.2
Zranb2	7E-13	0.2	Kctd6	3E-02	0.2
Aox1	7E-01	0.2	Zfp770	1E-01	0.2
Ercc6l2	7E-03	0.2	Prdx6	6E-08	0.2
Nupl2	7E-02	0.2	Khdc4	1E-03	0.2
Dnmt3a	6E-06	0.2	Hipk1	4E-02	0.2
Mrpl47	6E-03	0.2	Ccdc66	8E-01	0.2
Rtraf	3E-21	0.2	Usp36	7E-03	0.2
Rab4a	3E-03	0.2	Fam53a	2E-02	0.2
Erlin1	1E-01	0.2	Mon2	2E-04	0.2
Fkbp10	1E-09	0.2	Loxl2	3E-02	0.2
Cgrrf1	1E-04	0.2	Brd3os	1E-02	0.2
Arl2bp	7E-06	0.2	Tmem201	3E-02	0.2
Canx	6E-18	0.2	Cd59a	2E-01	0.2
Gpr180	6E-05	0.2	Msl2	2E-02	0.2
Ccdc59	2E-08	0.2	Ppp1r12b	1E-03	0.2
Pygo1	2E-03	0.2	Uqcrh	2E-26	0.2
Spire1	9E-05	0.2	Trim24	1E-04	0.2
Fxr2	5E-02	0.2	Rnf166	7E-03	0.2
Eef1b2	4E-43	0.2	Esco1	9E-04	0.2
Zfr	2E-11	0.2	Atp5g1	2E-12	0.2
Ccny	4E-06	0.2	Poglut2	1E-02	0.2
Tob2	6E-02	0.2	Grpel2	1E+00	0.2
Hist1h2bc	1E-07	0.2	Nt5c2	4E-04	0.2
Pcif1	7E-04	0.2	Anks1	9E-05	0.2
Fam126b	5E-02	0.2	Srgap2	2E-03	0.2
Cemip	5E-11	0.2	Tgfbrap1	2E-01	0.2
Gm16133	2E-02	0.2	B130055M24Rik	4E-02	0.2
Fam204a	6E-04	0.2	Gpc1	3E-02	0.2
Tmem216	1E-01	0.2	Cdk5rap2	4E-05	0.2
Mapk1ip1	1E-02	0.2	Nolc1	5E-05	0.2
Dctn4	2E-06	0.2	Tigd2	1E-03	0.2
2310015A10Rik	5E-02	0.2	Tbpl1	4E-03	0.2
Wdfy2	1E-02	0.2	Rraga	2E-07	0.2
Ift122	5E-03	0.2	Sars	4E-16	0.2
Zfp706	2E-14	0.2	Tpi1	2E-07	0.2
Bcl7b	2E-05	0.2	Bud31	1E-06	0.2
Prelid3b	7E-05	0.2	Dido1	8E-02	0.2
Mrps14	2E-13	0.2	Eif4a2	4E-10	0.2
Noa1	2E-02	0.2	Atr	5E-03	0.2
Mrpl2	1E-05	0.2	Prmt1	1E-11	0.2
Rdh14	1E-04	0.2	Ift122	4E-02	0.2
1700109H08Rik	9E-01	0.2	Ankhd1	3E-03	0.2
Rabepk	4E-01	0.2	mt-Nd4	1E-26	0.2
Metap1d	5E-05	0.2	Usf2	1E-07	0.2
Sertad4	1E-02	0.2	Acadsb	3E-06	0.2
2310033P09Rik	9E-04	0.2	Taldo1	1E-12	0.2
Cwf19l2	1E-04	0.2	Cox14	9E-16	0.2
Bivm	8E-02	0.2	Ndufb3	2E-11	0.2
Ftsj3	2E-02	0.2	Mrps18b	7E-05	0.2
Kansl3	4E-02	0.2	Ywhaq	2E-10	0.2
Fastkd2	3E-02	0.2	Abcd3	2E-04	0.2
Cmss1	6E-11	0.2	Tyw1	8E-02	0.2
Ccdc157	9E-02	0.2	Synj2bp	3E-03	0.2
Snupn	3E-02	0.2	Zkscan8	3E-02	0.2
0610040B10Rik	2E-02	0.2	Tapt1	3E-02	0.2
Rrnad1	2E-01	0.2	Pdcl	3E-02	0.2
Gtf2b	7E-04	0.2	Fmn1	1E-04	0.2
Cops9	2E-17	0.2	Fkbp10	6E-09	0.2
Zkscan1	6E-05	0.2	1110038B12Rik	1E-14	0.2
Bcl7a	5E-02	0.2	Spire1	5E-04	0.2
Cyb5d2	2E-03	0.2	Srp9	1E-14	0.2

Fibroblast					
Control			*Notch2*$^{tm1.1Ecan}$		
Gene	*p*	FC	Gene	*p*	FC
Mtfmt	3E-02	0.2	Frmd4a	7E-05	0.3
Thrap3	6E-11	0.2	Hdgfl2	3E-04	0.3
Adra1b	1E-01	0.2	Lasp1	8E-04	0.3
Togaram1	1E-02	0.2	Rtca	2E-05	0.3
Pam	2E-19	0.2	Bcl7a	3E-03	0.3
Elf4	4E-05	0.2	Cnep1r1	4E-07	0.3
Fnip1	2E-08	0.2	Hras	4E-09	0.3
Stx2	7E-03	0.2	Fbxl15	2E-02	0.3
Slc39a6	6E-05	0.2	Hnrnph1	7E-07	0.3
Wdr12	2E-02	0.2	Peli1	2E-05	0.3
Pet100	2E-05	0.2	Col4a5	4E-06	0.3
Baz2a	8E-03	0.2	Lrig2	4E-02	0.3
Acp1	5E-10	0.2	Zfp930	3E-02	0.3
Cd151	2E-03	0.2	Cox6b2	4E-02	0.3
Tmem123	3E-03	0.2	Rhog	5E-04	0.3
Ctr9	7E-03	0.2	Anapc2	7E-07	0.3
Nup153	5E-05	0.2	Kctd5	3E-02	0.3
Mark2	6E-04	0.2	Flot2	5E-09	0.3
Rras2	4E-03	0.2	Pdlim5	2E-09	0.3
Cenpb	1E-05	0.2	Sptan1	4E-12	0.3
Itpa	2E-02	0.2	Dgkd	2E-04	0.3
Lrrc57	2E-02	0.2	Trim28	8E-07	0.3
Adcy3	5E-02	0.2	Anxa2	9E-28	0.3
Zfp65	2E-01	0.2	Mgat2	9E-07	0.3
Hdac1	9E-06	0.2	Anp32b	3E-09	0.3
Galnt2	5E-05	0.2	Aff4	2E-11	0.3
Ulk2	5E-04	0.2	Gatad1	2E-10	0.3
Exoc2	4E-04	0.2	Pop1	7E-03	0.3
Irf2bpl	9E-06	0.2	Etfdh	5E-05	0.3
Zfand2b	1E-02	0.2	Fbxo4	2E-03	0.3
Med12	4E-03	0.2	Hdac3	1E-02	0.3
Gtf3c4	4E-02	0.2	Slc25a26	5E-02	0.3
Fam98b	8E-06	0.2	Asf1a	9E-04	0.3
Tep1	1E-01	0.2	Bdp1	7E-05	0.3
Hnrnph2	9E-05	0.2	Actg1	1E-13	0.3
Wls	2E-16	0.2	Plcd3	7E-02	0.3
Cyb5a	2E-11	0.2	Hmg20b	2E-11	0.3
Ankrd13a	1E-02	0.2	Ssc5d	1E-04	0.3
Plekhg5	5E-05	0.2	Emp1	3E-14	0.3
Kif26b	9E-01	0.2	Cep170	5E-06	0.3
Mrpl51	4E-06	0.2	Dus1l	6E-02	0.3
Sfxn3	7E-04	0.2	Bmpr1a	1E-14	0.3
Tgfa	4E-02	0.2	1600012H06Rik	5E-02	0.3
Sumo2	4E-15	0.2	Sgf29	2E-03	0.3
Tmed5	1E-03	0.2	Vac14	2E-03	0.3
Igf1r	2E-08	0.2	Tcf12	2E-12	0.3
Uimc1	4E-04	0.2	Fam210b	1E-02	0.3
Nubp2	2E-01	0.2	Lemd3	2E-03	0.3
Cog7	2E-02	0.2	Nfib	6E-21	0.3
Leng1	3E-03	0.2	Zeb2	2E-20	0.3
Dhx29	1E-03	0.2	Nup50	8E-04	0.3
Pcmt1	2E-05	0.2	Dgke	1E-02	0.3
Sap30	2E-03	0.2	Acox3	1E-01	0.3
Hspbap1	2E-02	0.2	Hdac5	6E-03	0.3
Vps9d1	1E-01	0.2	Cpne2	1E-02	0.3
Ctsh	2E-10	0.2	Hspb8	1E-03	0.3
Acadl	3E-07	0.2	Kif2a	1E-07	0.3
Ap3m1	4E-03	0.2	Suds3	2E-03	0.3
Abhd17c	9E-02	0.2	Snrpe	7E-20	0.3
Ndufaf7	6E-04	0.2	Mylk	8E-01	0.3
Spsb2	3E-03	0.2	Gm47283	4E-06	0.3
Tanc1	3E-05	0.2	Caprin2	6E-04	0.3
Ebag9	1E-02	0.2	Pnrc1	1E-11	0.3
Csgalnact1	7E-12	0.2	Ctps2	9E-06	0.3

Undefined					
Control			*Notch2*$^{tm1.1Ecan}$		
Gene	*p*	FC	Gene	*p*	FC
Jtb	7E-02	0.1	Ndufaf5	1E-02	0.1
Znhit3	9E-01	0.1	Bmyc	1E-05	0.1
Gm4258	4E-01	0.1	Prkaa1	3E-09	0.1
Fam45a	1E-01	0.1	Rps5	1E-07	0.1
Tmsb15b2	7E-01	0.1	Snapc5	3E-04	0.1
Etnk1	3E-02	0.1	Ptk2	1E-06	0.1
Rpl19	1E-18	0.1	Tbl1x	3E-04	0.1
Gm49959	8E-01	0.1	Wdr83	1E-05	0.1
Zfp579	3E-03	0.1	Fam71e1	2E-01	0.1
Itpr3	8E-01	0.1	Tmem216	2E-03	0.1
Cdc14b	1E-02	0.1	B9d2	1E-05	0.1
Arfgef2	4E-02	0.1	Clybl	5E-05	0.1
Gtf2a2	4E-01	0.1	Cd82	1E-04	0.1
Fbf1	2E-01	0.1	Cdkn2aip	2E-06	0.1
Ints4	9E-02	0.1	Ccdc88a	2E-03	0.1
Plekha3	8E-01	0.1	Pin4	2E-09	0.1
Zcchc4	8E-02	0.1	Gm47283	4E-03	0.1
Efhd2	8E-02	0.1	Adipor1	2E-08	0.1
Ptbp1	3E-04	0.1	Aopep	8E-01	0.1
Ints11	1E-02	0.1	Trnau1ap	2E-05	0.1
Syf2	9E-02	0.1	Bcs1l	7E-03	0.1
Ciao3	4E-01	0.1	Ift52	1E-03	0.1
Tns2	4E-01	0.1	Gm16759	7E-04	0.1
Prdx3	9E-01	0.1	Bmi1	2E-02	0.1
Rai1	3E-01	0.1	Cct8	5E-10	0.1
Apoo	1E+00	0.1	Bloc1s2	2E-09	0.1
Hs2st1	8E-01	0.1	Atf4	4E-06	0.1
Fkbp3	2E-01	0.1	Rab3gap1	8E-09	0.1
Malsu1	4E-02	0.1	Stoml2	7E-05	0.1
Eprs	3E-03	0.1	Gphn	2E-05	0.1
Lnpep	7E-01	0.1	Zbtb10	4E-06	0.1
Fam107b	1E-01	0.1	2700054A10Rik	3E-03	0.1
Tomm40	9E-03	0.1	Tmem64	5E-11	0.1
Saysd1	3E-01	0.1	Ppp1r8	4E-04	0.1
Atg4c	3E-02	0.1	Zcrb1	2E-02	0.1
Ell	5E-03	0.1	Obsl1	8E-10	0.1
Steap2	6E-01	0.1	Ankmy2	1E-08	0.1
Ktn1	9E-02	0.1	Aamp	3E-08	0.1
Lrig2	1E-03	0.1	Zfp622	1E-13	0.1
Kcnq1ot1	9E-01	0.1	Cables1	3E-03	0.1
Tmem251	4E-01	0.1	Trmt10a	6E-05	0.1
Ndufb2	4E-01	0.1	1810062O18Rik	7E-03	0.1
Lekr1	2E-01	0.1	Snupn	4E-06	0.1
Atpaf1	3E-02	0.1	Tbc1d4	2E-02	0.1
Sugp1	5E-01	0.1	Casc3	2E-04	0.1
Pole3	4E-02	0.1	Psma3	1E-04	0.1
Smtn	6E-01	0.1	Akap11	3E-10	0.1
Cmtr1	4E-03	0.1	Zfpl1	1E-08	0.1
Pex11g	2E-01	0.1	Ccdc127	6E-05	0.1
Rbak	4E-01	0.1	Adamts9	2E-04	0.1
Taf4b	5E-01	0.1	Creb3l2	3E-03	0.1
Kbtbd4	8E-01	0.1	Vamp4	3E-07	0.1
Krt10	3E-01	0.1	Dcakd	3E-08	0.1
Pex3	1E-01	0.1	Eif3d	1E-16	0.1
Zmiz1	7E-01	0.1	Trmt10c	8E-02	0.1
Cd151	3E-01	0.1	Gm11808	1E-08	0.1
Rhot1	8E-04	0.1	Sarnp	8E-05	0.1
Spcs3	4E-02	0.1	Mllt11	2E-04	0.1
Ddx50	3E-01	0.1	Appbp2	4E-09	0.1
Sfxn2	9E-01	0.1	Btf3	7E-01	0.1
Ralgapb	6E-01	0.1	Tob1	2E-05	0.1
Fam136a	4E-03	0.1	Pfdn2	1E-08	0.1
Shmt2	9E-01	0.1	Tmed3	7E-05	0.1
AU040320	4E-02	0.1	Zfp788	8E-03	0.1

Articular/Synovial Fibroblast					
Control			*Notch2*$^{tm1.1Ecan}$		
Gene	*p*	FC	Gene	*p*	FC
Faim	2E-02	0.3	D030056L22Rik	3E-01	0.3
Prkcd	1E-01	0.3	Pef1	5E-02	0.3
Ergic1	3E-05	0.3	Ptprj	1E-03	0.3
Grina	2E-13	0.3	Kdm4b	4E-01	0.3
Atg14	1E-02	0.3	Pink1	4E-02	0.3
Fuca1	8E-13	0.3	Tmem184c	2E-04	0.3
Slc30a4	6E-02	0.3	9530026P05Rik	2E-02	0.3
Cic	1E-02	0.3	Spata24	5E-03	0.3
Rock1	2E-12	0.3	Wdr3	9E-02	0.3
Plscr3	1E-02	0.3	Fosl2	5E-06	0.3
Rora	6E-16	0.3	Pygb	6E-02	0.3
Wdr60	1E-01	0.3	Zfp62	1E-01	0.3
Zmat3	1E-02	0.3	3110009E18Rik	3E-01	0.3
Dnm3os	2E-07	0.3	Ikbke	1E-01	0.3
Exd2	2E-01	0.3	Zfp945	2E-02	0.3
Smad3	1E-04	0.3	Snrnp25	2E-01	0.3
Tm2d2	2E-09	0.3	Fam214b	8E-02	0.3
Abcd1	1E-01	0.3	Eci2	3E-06	0.3
Nudt16l1	1E-01	0.3	Gm37494	2E-02	0.3
Gnpda1	3E-03	0.3	Rab26os	7E-02	0.3
Ehmt2	8E-06	0.3	Pknox2	1E-01	0.3
Rufy2	2E-04	0.3	Gm30025	6E-02	0.3
Ifngr2	8E-05	0.3	Fam32a	6E-03	0.3
Inpp4a	3E-02	0.3	Scd1	9E-24	0.3
Fbxl3	7E-04	0.3	Dzip1	7E-04	0.3
Pten	6E-12	0.3	Cep68	2E-01	0.3
Bad	3E-08	0.3	Asah2	3E-01	0.3
Sertad1	1E-01	0.3	Ei24	1E-04	0.3
Tex264	5E-08	0.3	Ccdc93	2E-02	0.3
Pcnx4	3E-03	0.3	Slc30a4	8E-03	0.3
Nfkbia	9E-06	0.3	Ilvbl	2E-02	0.3
Wdr55	6E-01	0.3	Rbpj	4E-06	0.3
Dmxl1	7E-06	0.3	Slc10a7	9E-03	0.3
Kctd17	3E-04	0.3	Stx18	2E-05	0.3
Armcx3	1E-07	0.3	Fhl1	2E-04	0.3
H2-DMa	2E-01	0.3	Pdhx	6E-02	0.3
Slc27a4	3E-02	0.3	Dync2h1	2E-02	0.3
Arpc4	2E-06	0.3	Rubcn	3E-02	0.3
Kdm3a	3E-04	0.3	Eif4g3	1E-07	0.3
Smad1	6E-06	0.3	Tut7	3E-05	0.3
Mettl21a	1E-03	0.3	Ctdsp2	1E-07	0.3
Tbc1d16	3E-02	0.3	Eif2d	8E-02	0.3
Papolg	1E-01	0.3	Rnf219	2E-01	0.3
Dennd6a	3E-05	0.3	Mtch2	2E-07	0.3
Nceh1	5E-02	0.3	C2cd2	1E-01	0.3
Rusc2	2E-04	0.3	Abhd18	8E-03	0.3
Btbd10	4E-02	0.3	Ulk2	3E-05	0.3
Apba3	1E-01	0.3	Oxct1	1E-11	0.3
Cyb5r4	2E-04	0.3	Ypel1	6E-02	0.3
Ap3b1	9E-08	0.3	Rufy2	8E-04	0.3
Katnal1	1E-01	0.3	Ammecr1	2E-02	0.3
Npc1	3E-02	0.3	Evi5l	2E-01	0.3
Cep250	6E-04	0.3	Casd1	1E-01	0.3
Ranbp3	2E-01	0.3	Bicra	5E-02	0.3
Vps33a	9E-02	0.3	Nr3c1	7E-07	0.3
Garnl3	2E-02	0.3	Stat3	1E-07	0.3
Bag6	3E-05	0.3	Lypla1	1E-01	0.3
Rab22a	2E-06	0.3	Aox1	3E-02	0.3
Cyp39a1	8E-02	0.3	Irak4	9E-01	0.3
Helq	4E-02	0.3	Vcpip1	6E-05	0.3
Glmn	5E-03	0.3	Gpatch8	5E-08	0.3
Zfp518a	3E-03	0.3	Nt5dc1	8E-04	0.3
Leprot	2E-08	0.3	Taco1os	1E-01	0.3
Arid2	8E-04	0.3	Fbxo8	2E-01	0.3

Limb Mesenchyme

Control			*Notch2* $^{tm1.1Ecan}$		
Gene	*p*	FC	Gene	*p*	FC
Slc26a11	2E-02	0.1	Fbxo22	1E-02	0.1
Smad4	5E-06	0.1	Rpl31	1E-27	0.1
Spats2	1E-02	0.1	Shprh	1E-06	0.1
Csnk2b	7E-09	0.1	Upf1	9E-05	0.1
Slc3a2	8E-13	0.1	Tram1	2E-09	0.1
Med12	5E-02	0.1	Cox7b	2E-15	0.1
Rpl18a	2E-45	0.1	Washc1	4E-08	0.1
Nop9	2E-02	0.1	Pinx1	3E-04	0.1
Gm2a	2E-03	0.1	Rpl29	9E-38	0.1
Dnaja2	1E-09	0.1	Creb3	4E-07	0.1
Eif4a1	5E-15	0.1	Glmp	1E-11	0.1
Gars	2E-11	0.1	Gm14325	1E-06	0.1
Kctd2	1E-01	0.1	Ogfod2	3E-03	0.1
Nfib	1E-08	0.1	Rps3	2E-34	0.1
Klhl5	4E-03	0.1	BC034090	5E-03	0.1
Lsm7	2E-05	0.1	Gstm4	4E-03	0.1
Lysmd3	1E-03	0.1	Ppia	2E-29	0.1
Gfer	6E-04	0.1	Faf2	7E-06	0.1
Dbn1	1E-04	0.1	Thbs3	1E-07	0.1
Pxmp4	1E-04	0.1	Ldlrad4	9E-11	0.1
Psmc4	3E-05	0.1	Ddx54	2E-07	0.1
Mrps24	4E-09	0.1	Cdkn2aip	9E-03	0.1
Galk1	2E-05	0.1	Nosip	7E-05	0.1
Ttll12	3E-02	0.1	Rpl32	1E-39	0.1
Cmya5	2E-01	0.1	Gaa	2E-08	0.1
Tmem11	6E-05	0.1	Exoc5	2E-08	0.1
Phf13	1E-01	0.1	Chmp1a	1E-06	0.1
Psma6	1E-05	0.1	Ndufs4	1E-15	0.1
Rpl36	5E-45	0.1	Eif3k	5E-19	0.1
Dnajc13	9E-05	0.1	Gnptab	4E-02	0.1
Etfbkmt	1E-01	0.1	E230016M11Rik	3E-06	0.1
Cpeb1	2E-02	0.1	Rcan3	5E-04	0.1
Nrbf2	4E-04	0.1	Tsc22d2	1E-10	0.1
Slc27a4	1E-01	0.1	Six4	2E-02	0.1
Gfpt2	2E-01	0.1	Mrps6	5E-04	0.1
Snx4	6E-07	0.1	Aar2	5E-03	0.1
Msrb3	4E-06	0.1	Mad2l1bp	2E-03	0.1
Sparcl1	4E-01	0.1	Tbrg1	2E-17	0.1
Chmp5	5E-06	0.1	Gramd3	3E-05	0.1
Stt3a	4E-07	0.1	Eif3h	3E-19	0.1
Sec61g	1E-27	0.1	Ptbp1	2E-04	0.1
Pik3c2a	6E-03	0.1	Tmem64	3E-07	0.1
Serpine2	1E-09	0.1	Spats2	5E-04	0.1
Sh3pxd2a	2E-09	0.1	Col6a1	9E-13	0.1
Cyp2j6	2E-02	0.1	6330403K07Rik	8E-03	0.1
Armcx3	1E-04	0.1	Asb7	9E-05	0.1
Anapc7	4E-01	0.1	Hcfc1r1	1E-13	0.1
Zfand1	6E-03	0.1	Farp2	9E-07	0.1
Arhgap1	2E-02	0.1	Nip7	2E-05	0.1
Snx8	1E-02	0.1	Twistnb	3E-07	0.1
Snx2	8E-08	0.1	Numb	7E-05	0.1
Agps	2E-03	0.1	Dtx3	2E-04	0.1
Rexo1	9E-03	0.1	Ier5	1E-04	0.1
Xab2	2E-02	0.1	Ypel3	9E-10	0.1
Epdr1	3E-02	0.1	Atg101	2E-04	0.1
Ykt6	1E-03	0.1	Timm10b	1E-12	0.1
Naa20	3E-05	0.1	1500015L24Rik	9E-10	0.1
Rnf149	3E-01	0.1	Pakap.1	2E-08	0.1
Gm46430	2E-01	0.1	Klhl23	1E-02	0.1
Plxna1	3E-02	0.1	Arf3	4E-05	0.1
Nop16	3E-03	0.1	Psmb10	2E-05	0.1
Snx33	1E-01	0.1	Ints1	1E-01	0.1
Tent2	1E-06	0.1	Strn4	2E-05	0.1
Zfp263	9E-01	0.1	Nab1	1E-05	0.1

Chondrogenic

Control			*Notch2* $^{tm1.1Ecan}$		
Gene	*p*	FC	Gene	*p*	FC
Mrpl24	1E-09	0.2	Slc41a1	2E-01	0.2
Echdc3	3E-01	0.2	Pnkd	2E-08	0.2
Traf3	4E-04	0.2	Mar6	9E-06	0.2
Ndufa6	5E-19	0.2	Adar	2E-01	0.2
Gorasp1	9E-02	0.2	Metap1d	4E-06	0.2
Chordc1	3E-05	0.2	Crebzf	4E-02	0.2
Qtrt1	4E-02	0.2	Mettl16	3E-03	0.2
Polr2l	2E-04	0.2	Pprc1	1E-02	0.2
Cct2	4E-17	0.2	Rwdd3	2E-02	0.2
Zfp692	2E-01	0.2	Rfc3	4E-02	0.2
Kmt5b	2E-01	0.2	Polr2m	3E-11	0.2
Sf3b5	2E-13	0.2	Slc35a2	2E-03	0.2
Ip6k2	4E-02	0.2	Snrnp48	5E-03	0.2
Papola	2E-11	0.2	Cox11	2E-03	0.2
Dtna	2E-05	0.2	Elp1	3E-03	0.2
Ldha	1E-12	0.2	Nat10	1E-02	0.2
Slc35b1	2E-03	0.2	Ndrg3	6E-03	0.2
Fzd7	1E-01	0.2	Lsm8	3E-07	0.2
Eif4g2	4E-20	0.2	Asb3	3E-03	0.2
Mtx1	1E-02	0.2	Nucb2	1E-06	0.2
Srr	3E-02	0.2	Zbtb10	9E-04	0.2
Znrd2	4E-03	0.2	Rpl10-ps3	4E-02	0.2
Sec61b	1E-23	0.2	Sec23b	8E-04	0.2
Noc2l	3E-02	0.2	Prr3	3E-01	0.2
Pfdn6	2E-05	0.2	Rab27b	6E-02	0.2
Tmem39a	7E-06	0.2	Armc9	8E-02	0.2
4933406I18Rik	4E-03	0.2	Wdr12	5E-03	0.2
Tmed2	9E-17	0.2	H3f3b	3E-22	0.2
Jagn1	7E-03	0.2	Snhg20	3E-02	0.2
Slc25a39	8E-09	0.2	Wdr74	2E-04	0.2
Slc30a5	2E-03	0.2	Ankra2	3E-05	0.2
Foxj3	3E-02	0.2	Rabepk	4E-03	0.2
Tulp3	4E-04	0.2	Dap3	7E-07	0.2
Fbxo10	2E-01	0.2	Msantd2	8E-02	0.2
Mipep	9E-02	0.2	Dstyk	1E-03	0.2
Rnf220	6E-02	0.2	Akap9	3E-08	0.2
Mrps16	1E-09	0.2	Sec61b	1E-15	0.2
Naa16	1E-02	0.2	A330076H08Rik	7E-02	0.2
Gid4	3E-03	0.2	Limd2	5E-03	0.2
Alg14	8E-04	0.2	Gbf1	8E-09	0.2
Nop14	8E-06	0.2	Snhg18	2E-12	0.2
Pomt2	8E-01	0.2	Aatf	8E-04	0.2
Rnf113a2	1E-02	0.2	Phka1	4E-02	0.2
Gle1	4E-02	0.2	Akap1	3E-01	0.2
Msh2	6E-02	0.2	Zrsr1	2E-01	0.2
Pld1	8E-03	0.2	Cnot8	3E-05	0.2
Araf	8E-05	0.2	Slc35b3	7E-02	0.2
Usp10	3E-03	0.2	Ankrd11	8E-09	0.2
Gm17092	4E-01	0.2	mt-Co2	1E-26	0.2
Lum	2E-06	0.2	Tmem164	2E-03	0.2
Tomm70a	5E-03	0.2	Rchy1	6E-04	0.2
Usp36	9E-02	0.2	Zfp707	9E-02	0.2
Rbbp9	7E-04	0.2	Fahd2a	2E-04	0.2
Chchd6	7E-04	0.2	Pi4k2a	4E-04	0.2
Emc4	2E-05	0.2	Gm43672	2E-02	0.2
Micu1	5E-07	0.2	Gsk3a	6E-04	0.2
Ube4b	7E-04	0.2	Odf2l	1E-02	0.2
Zfyve9	1E-05	0.2	Impa1	3E-03	0.2
Tspan31	8E-07	0.2	Cox18	1E-03	0.2
St7	4E-05	0.2	Mphosph8	1E-05	0.2
Khdc4	3E-02	0.2	Jagn1	8E-03	0.2
Jam3	4E-02	0.2	Tbp	2E-01	0.2
Kpna1	1E-03	0.2	Dtwd1	3E-02	0.2
Dnajc4	1E-03	0.2	Csad	2E-06	0.2

Fibroblast

Control			*Notch2* $^{tm1.1Ecan}$		
Gene	*p*	FC	Gene	*p*	FC
Klf13	5E-05	0.2	Vwa5a	8E-06	0.3
Gk5	3E-02	0.2	Ogn	2E-10	0.3
Reck	2E-09	0.2	Apbb2	5E-09	0.3
Slk	2E-06	0.2	Erh	1E-07	0.3
Gin1	2E-02	0.2	Emd	1E-04	0.3
Abca3	2E-01	0.2	Acyp2	8E-09	0.3
Chkb	7E-02	0.2	Msl3	8E-07	0.3
Srsf4	3E-04	0.2	Cryab	2E-20	0.3
Katnal1	3E-02	0.2	Hdac2	8E-06	0.3
Ube2n	3E-06	0.2	Clasp2	4E-05	0.3
Prkcd	6E-01	0.2	Pde5a	3E-06	0.3
Uggt1	2E-05	0.2	Map1a	1E-04	0.3
Mgat1	5E-05	0.2	Polr2a	2E-10	0.3
Pik3ip1	1E-01	0.2	Ttc28	4E-11	0.3
Morn2	1E-03	0.2	Acsl4	9E-05	0.3
B4galt7	7E-03	0.2	C4b	3E-03	0.3
Bclaf3	2E-02	0.2	Setd7	6E-08	0.3
Ercc8	8E-02	0.2	Nsun6	3E-03	0.3
Naaladl2	4E-08	0.2	Chd1	4E-06	0.3
Tjap1	2E-03	0.2	Pwp1	4E-03	0.3
Pigu	2E-02	0.2	Sin3a	4E-04	0.3
Gmpr2	1E-01	0.2	Slc25a14	1E-01	0.3
Msantd4	1E-04	0.2	Smyd4	2E-04	0.3
Cdkn2aip	2E-01	0.2	Hif1an	2E-03	0.3
Hdac5	3E-02	0.2	Rbbp7	7E-16	0.3
Clba1	5E-02	0.2	Srgap3	4E-09	0.3
Mkks	1E-01	0.2	Zfp976	7E-03	0.3
Pmm2	1E-01	0.2	Chd4	1E-18	0.3
Pank2	3E-03	0.2	Tcof1	2E-07	0.3
Trim12c	3E-02	0.2	Cdadc1	1E-03	0.3
Tnrc18	3E-09	0.2	1700037H04Rik	9E-04	0.2
Pdxk	8E-02	0.2	Mtmr6	9E-04	0.2
Upf3b	2E-04	0.2	Zfp868	3E-01	0.2
Lmbr1l	4E-01	0.2	Fbxo21	8E-04	0.2
Patj	6E-04	0.2	2010001A14Rik	2E-01	0.2
Pias2	2E-05	0.2	Man1b1	2E-02	0.2
Prss23	1E-04	0.2	Txnrd1	2E-05	0.2
R3hcc1l	6E-02	0.2	6720427I07Rik	1E-05	0.2
Pxk	1E-03	0.2	Rybp	9E-02	0.2
Xpo4	2E-04	0.2	Ap4e1	9E-03	0.2
Ckb	2E-04	0.2	Crebzf	7E-03	0.2
Hif1an	4E-01	0.2	Cxcl12	7E-28	0.2
Ttc32	2E-01	0.2	Pskh1	8E-03	0.2
Arl6	7E-03	0.2	Patz1	4E-02	0.2
Gtf2f1	1E-02	0.2	Cpt1a	1E-03	0.2
Nup88	2E-02	0.2	Slk	6E-07	0.2
Nsmce1	2E-03	0.2	Anxa1	2E-23	0.2
Mtcl1	8E-02	0.2	Prr14l	4E-05	0.2
Slc20a1	9E-05	0.2	Pcmt1	6E-09	0.2
Efna1	5E-02	0.2	Tcf20	6E-06	0.2
Slc39a10	2E-03	0.2	Wdsub1	1E-01	0.2
Dnase2a	3E-02	0.2	Tcf4	3E-18	0.2
Fmnl3	6E-02	0.2	Cggbp1	9E-08	0.2
Grk2	6E-03	0.2	Tmx2	5E-02	0.2
Qars	4E-03	0.2	Med26	5E-02	0.2
Gm42726	2E-01	0.2	Acp1	5E-11	0.2
Ctps	1E-04	0.2	Large1	3E-12	0.2
Ddx55	6E-03	0.2	Vill	3E-03	0.2
Hcfc1	4E-03	0.2	Vps13a	2E-05	0.2
Bbs5	1E+00	0.2	Cars2	9E-03	0.2
Fbxo4	1E-02	0.2	Naa20	4E-06	0.2
Rasa1	2E-06	0.2	Zfp949	1E-01	0.2
Cab39l	7E-03	0.2	Eid2b	3E-02	0.2
Mad1l1	4E-03	0.2	Ap1s1	3E-07	0.2

Undefined

Control			*Notch2* $^{tm1.1Ecan}$		
Gene	*p*	FC	Gene	*p*	FC
Pex11b	1E-01	0.1	Kctd15	5E-01	0.1
Rars	2E-01	0.1	Furin	9E-06	0.1
Cep44	4E-01	0.1	Ap1g1	6E-05	0.1
Rpl23	9E-19	0.1	Stard13	1E-02	0.1
Setx	4E-05	0.1	Paip1	8E-09	0.1
Adamtsl1	2E-01	0.1	Pja2	1E-05	0.1
Hsph1	9E-03	0.1	Tm7sf3	1E-18	0.1
Dhrs7	3E-01	0.1	Eef1d	1E-01	0.1
Emc4	6E-02	0.1	Ccar2	2E-03	0.1
H19	7E-01	0.1	Cog5	3E-11	0.1
Crbn	1E-02	0.1	Nfatc3	1E-09	0.1
Piezo2	9E-03	0.1	Clstn1	1E-10	0.1
Hoxc5	3E-01	0.1	Tmed9	8E-01	0.1
Kctd20	6E-06	0.1	Pigq	7E-03	0.1
Isoc1	7E-01	0.1	B3galnt2	4E-03	0.1
Rbm8a	4E-01	0.1	Stim1	8E-10	0.1
H2afz	1E-02	0.1	Airn	5E-01	0.1
Tmem260	1E-01	0.1	Ttc1	3E-08	0.1
Tfb1m	1E+00	0.1	Vmac	2E-02	0.1
Echs1	4E-03	0.1	Slc39a10	7E-07	0.1
Snupn	6E-01	0.1	Slc4a1ap	5E-08	0.1
Ptprg	3E-02	0.1	Slc16a2	2E-02	0.1
Dnajb6	3E-02	0.1	1110008P14Rik	2E-04	0.1
Rora	1E-01	0.1	Slc12a9	3E-09	0.1
Rps23	9E-16	0.1	Rpp14	3E-04	0.1
Rpl18	8E-15	0.1	Gsr	6E-08	0.1
Usp38	4E-02	0.1	Trnt1	7E-12	0.1
Lsm14a	1E-02	0.1	Immp2l	1E-01	0.1
Dcaf17	2E-01	0.1	Akt2	5E-03	0.1
Pigyl	5E-01	0.1	Ssu72	6E-07	0.1
Nek1	5E-01	0.1	mt-Co1	1E-19	0.1
Dot1l	2E-02	0.1	Tob2	2E-03	0.1
Ankhd1	2E-02	0.1	Dnmt3a	6E-05	0.1
Gtf2h4	5E-02	0.1	Rdh14	3E-05	0.1
Tanc2	2E-01	0.1	Gatc	4E-02	0.1
Trpc4ap	5E-02	0.1	Inppl1	2E-10	0.1
Pus7	2E-01	0.1	Wdr82	3E-08	0.1
Cox7c	2E-09	0.1	Pgm3	5E-06	0.1
Rpain	8E-02	0.1	Eif3c	9E-01	0.1
Wdcp	6E-01	0.1	Tmed4	1E-06	0.1
Ampd2	3E-04	0.1	Rgmb	4E-02	0.1
Rpl26	3E-18	0.1	Sec62	4E-03	0.1
Ccl25	9E-01	0.1	Sptssa	2E-05	0.1
Prpsap2	4E-01	0.1	Spg7	1E-10	0.1
Tsnax	5E-02	0.1	Cacfd1	1E-11	0.1
Mpv17l	1E-01	0.1	Nop2	9E-03	0.1
Ccdc91	3E-02	0.1	Aimp2	1E-09	0.1
Anks3	1E-02	0.1	Mib2	4E-03	0.1
Ivd	9E-01	0.1	Gm2a	8E-12	0.1
Ccdc90b	5E-01	0.1	Ube2d3	2E-07	0.1
Zfp560	1E-02	0.1	Rab1b	1E-17	0.1
Cdc42se1	6E-01	0.1	Rab4a	2E-02	0.1
Dzip3	3E-02	0.1	Psma5	6E-11	0.1
Hmox2	7E-02	0.1	Adh5	1E-05	0.1
Sdhb	2E-01	0.1	Amfr	8E-07	0.1
Jdp2	2E-02	0.1	Leng1	6E-06	0.1
Slc20a2	6E-01	0.1	Ipo4	2E-10	0.1
Nip7	2E-01	0.1	Krt10	3E-03	0.1
Serpinh1	9E-08	0.1	Etfrf1	7E-05	0.1
Ndufc2	2E-01	0.1	Tmem29	3E-08	0.1
Hnrnph3	1E+00	0.1	Hsf2	6E-08	0.1
Zfp64	2E-02	0.1	Farsa	1E-09	0.1
Lmtk2	1E-02	0.1	Rsrc2	4E-05	0.1
Ttc38	4E-01	0.1	Slc16a1	7E-02	0.1

Articular/Synovial Fibroblast

Control			*Notch2* $^{tm1.1Ecan}$		
Gene	*p*	FC	Gene	*p*	FC
Col4a2	8E-03	0.3	Usp13	6E-02	0.3
Zfp738	2E-02	0.3	Rap2b	1E-03	0.3
Cuedc2	1E-08	0.3	P4ha2	1E-07	0.3
Phf13	3E-02	0.3	Pcnx4	6E-02	0.3
Rnf217	8E-09	0.3	Gcfc2	1E-01	0.3
Zdhhc21	4E-03	0.3	Cldnd1	3E-03	0.3
Xpa	4E-06	0.3	Sec23ip	7E-03	0.3
Cdk5rap1	3E-01	0.3	Ddx55	2E-01	0.3
Arpc3	1E-17	0.3	B3gntl1	1E-01	0.3
Cep85l	7E-02	0.3	2700054A10Rik	8E-02	0.3
Mad2l1bp	2E-01	0.3	Zfp426	9E-02	0.3
Fam89b	4E-04	0.3	Ctif	1E-03	0.3
Ube2d1	5E-03	0.3	Hmgxb3	7E-01	0.3
Rab5b	3E-03	0.3	Vps41	6E-07	0.3
Rere	2E-15	0.3	Galc	2E-01	0.3
Dock1	4E-10	0.3	Fxyd5	2E-01	0.3
Uhrf2	5E-05	0.3	Gpatch1	3E-02	0.3
Tulp4	1E-06	0.3	Mypop	3E-02	0.3
1110051M20Rik	8E-08	0.3	Dhx16	6E-02	0.3
Adat2	7E-01	0.3	Ptn	1E-02	0.3
Zfp729a	5E-02	0.3	Wdr19	2E-01	0.3
Pxk	4E-03	0.3	Pomt2	8E-01	0.3
Ptpn12	3E-05	0.3	Galnt1	1E-05	0.3
Calml4	2E-01	0.3	Cbx6	3E-02	0.3
Nr3c1	5E-10	0.3	Arpc1a	8E-08	0.3
Armcx4	1E-01	0.3	Tmem19	1E-01	0.3
2610002M06Rik	8E-03	0.3	Wls	2E-11	0.3
Ncoa2	4E-08	0.3	Mast4	7E-04	0.3
Apbb1	5E-02	0.3	Trib1	7E-04	0.3
Lamp1	9E-29	0.3	Kctd18	1E-01	0.3
Col5a2	6E-17	0.3	Sh2b3	2E-02	0.3
Trappc11	3E-02	0.3	Ifi27	1E-09	0.3
Ccdc50	4E-07	0.3	Ubn1	2E-08	0.3
Mapk14	1E-03	0.3	Abhd5	9E-03	0.3
Snx6	7E-10	0.3	Arpc4	3E-03	0.3
Mdga1	2E-01	0.3	Pias2	7E-05	0.3
Fundc2	9E-15	0.3	Rnpep	2E-03	0.3
Pdcd6ip	5E-08	0.3	Klf8	7E-02	0.3
Rab1b	1E-04	0.3	Zmym3	5E-01	0.3
Cdkn1a	3E-05	0.3	Ccnd1	4E-01	0.3
Anapc1	2E-03	0.3	Slain2	2E-06	0.3
Ptpn23	1E-02	0.3	Cryab	4E-11	0.3
Eif4e3	5E-02	0.3	Bcl7a	4E-01	0.3
Arpc1a	4E-07	0.3	Snx6	7E-09	0.3
Arhgef9	7E-02	0.3	Rgs12	7E-03	0.3
Elf1	7E-05	0.3	Map1lc3b	3E-16	0.3
Abracl	2E-12	0.3	Tmem219	9E-04	0.3
Vps8	1E-03	0.3	Dnajc18	1E-02	0.3
Msra	9E-01	0.3	Zc3hav1	7E-05	0.3
Sesn1	1E-02	0.3	Abca3	3E-02	0.3
Asap1	1E-11	0.3	Per3	8E-03	0.3
Anxa7	3E-08	0.3	Bcr	1E-01	0.3
Gpatch2l	2E-01	0.3	Zup1	8E-01	0.3
Dhx33	4E-02	0.3	Psmf1	4E-01	0.3
Ro60	8E-03	0.3	Lamp1	1E-19	0.3
Tmem45a	1E-05	0.3	Arap1	1E-02	0.3
Traf6	3E-02	0.3	Rffl	8E-01	0.3
Tvp23a	5E-01	0.3	Mtmr4	4E-01	0.3
Ephb3	3E-02	0.3	D6Wsu163e	2E-01	0.3
Cobll1	2E-04	0.3	Orc5	5E-01	0.3
Fbh1	1E-02	0.3	Tprgl	2E-05	0.3
Psmd10	3E-02	0.3	Oxsm	1E-02	0.3
Ikbkb	4E-03	0.3	Mark2	6E-04	0.3
2810032G03Rik	3E-01	0.3	3300002I08Rik	2E-01	0.3

Limb Mesenchyme					
Control			Notch2 $^{tm1.1Ecan}$		
Gene	p	FC	Gene	p	FC
Setd1b	2E-02	0.1	Tmem165	2E-08	0.1
Iqsec2	1E-04	0.1	Mlf2	1E-10	0.1
Thoc6	1E-01	0.1	Mettl17	3E-04	0.1
Atp5e	8E-30	0.1	Traf5	2E-03	0.1
Rpl17	3E-38	0.1	Plxnb2	2E-06	0.1
Zbtb14	6E-01	0.1	Rpl35	6E-30	0.1
Zfp318	7E-03	0.1	Eid2b	3E-02	0.1
Xylt2	2E-01	0.1	Pip4p1	7E-07	0.1
Rnpep	5E-04	0.1	Fosb	2E-10	0.1
Cebpg	5E-06	0.1	Rrp7a	4E-04	0.1
R3hcc1	8E-02	0.1	Eaf1	5E-02	0.1
Ralgds	2E-02	0.1	Hhat	4E-03	0.1
Cd46	8E-02	0.1	Wdr45	7E-04	0.1
Cep83	5E-05	0.1	Gtpbp6	1E-04	0.1
Atxn7l3b	2E-10	0.1	Yif1b	1E-10	0.1
Eif2b2	2E-03	0.1	Yipf3	1E-10	0.1
Uxt	2E-02	0.1	Ncln	1E-05	0.1
Ppia	2E-28	0.1	Dyrk1b	3E-02	0.1
Plag1	2E-02	0.1	Elmo2	3E-03	0.1
Nhs	6E-05	0.1	Rpl37a	2E-39	0.1
Dusp11	2E-07	0.1	Gse1	5E-04	0.1
Pank3	4E-03	0.1	Pcgf3	2E-03	0.1
Tbc1d17	2E-03	0.1	Rnf121	1E-03	0.1
Igf2bp3	5E-04	0.1	Tbc1d12	1E-05	0.1
Cars	8E-04	0.1	Lrrc45	6E-03	0.1
Hspb11	5E-07	0.1	Dcaf4	1E-03	0.1
2210016F16Rik	2E-04	0.1	Adamtsl5	9E-09	0.1
Cenpw	4E-06	0.1	Alas1	8E-07	0.1
Mib2	1E-01	0.1	Fancc	1E-05	0.1
Aph1a	7E-05	0.1	Napa	8E-10	0.1
Crppa	2E-01	0.1	Gm10076	1E-25	0.1
Grpel2	2E-01	0.1	Dynlrb1	8E-16	0.1
Rbm7	3E-04	0.1	Eif5a	4E-13	0.1
Zfp868	1E-01	0.1	Twf1	2E-08	0.1
Fam160b1	3E-02	0.1	Msl1	2E-05	0.1
Gm2000	3E-06	0.1	Snf8	5E-13	0.1
Igdcc4	9E-03	0.1	Mapk8ip1	5E-02	0.1
Cfdp1	3E-12	0.1	Crk	3E-07	0.1
Dynll1	1E-19	0.1	Tfb2m	5E-04	0.1
Isca2	1E-02	0.1	Reep3	1E-07	0.1
Sra1	2E-08	0.1	Snx8	1E-03	0.1
Eid1	2E-11	0.1	Anapc11	7E-12	0.1
Eml1	8E-02	0.1	Emp2	5E-08	0.1
Arf1	2E-10	0.1	Acap3	1E-03	0.1
Anxa2	4E-18	0.1	Steap3	2E-09	0.1
Hras	4E-05	0.1	Ubfd1	1E-05	0.1
Mapkbp1	2E-02	0.1	Chordc1	1E-07	0.1
Sys1	3E-05	0.1	Rpl14	6E-31	0.1
Cks1b	1E-10	0.1	Rmi1	2E-04	0.1
Cope	2E-08	0.1	Ube3b	3E-03	0.1
Il6ra	3E-01	0.1	Orc4	8E-07	0.1
Cr1l	1E-04	0.1	Itgb5	7E-09	0.1
Lpgat1	3E-09	0.1	Gsn	1E-17	0.1
Fam210b	4E-01	0.1	Epb41l2	5E-08	0.1
Rad52	9E-02	0.1	Fam126a	1E-03	0.1
4921524J17Rik	1E-04	0.1	Rps9	6E-27	0.1
Glb1l	3E-01	0.1	Zfp866	1E-01	0.1
Commd6	7E-05	0.1	Rfk	1E-02	0.1
Ap1m1	6E-03	0.1	Cap1	3E-04	0.1
Scand1	9E-13	0.1	Mrpl52	3E-16	0.1
Mmgt2	5E-03	0.1	Fbxo10	2E-01	0.1
Rpl11	7E-43	0.1	Atp11c	8E-03	0.1
Reep3	1E-05	0.1	Nsmce3	1E-04	0.1
Kdelr2	6E-17	0.1	Dcaf6	1E-10	0.1

Chondrogenic					
Control			Notch2 $^{tm1.1Ecan}$		
Gene	p	FC	Gene	p	FC
Slc25a28	4E-02	0.2	Mcrip1	5E-10	0.2
Gatd3a	4E-03	0.2	Uty	1E-02	0.1
Arhgap5	2E-10	0.2	Rpn2	6E-10	0.1
Tmem161a	2E-02	0.2	Il17ra	4E-02	0.1
Ccdc43	3E-01	0.2	Dand5	7E-02	0.1
Pnrc2	3E-05	0.2	Gmeb1	3E-01	0.1
Ninl	1E-01	0.2	Cisd3	2E-01	0.1
Isy1	9E-02	0.2	Desi2	5E-07	0.1
Mfap1a	3E-02	0.2	Tdp2	2E-01	0.1
Mmp16	2E-10	0.2	Tbc1d4	1E-01	0.1
Tle3	7E-03	0.2	Zfyve16	6E-02	0.1
Dcaf11	2E-02	0.2	Gcn1	6E-04	0.1
Rps24	2E-50	0.2	Snapc1	1E-02	0.1
Dzip1l	3E-03	0.2	Med27	4E-02	0.1
Klhdc1	5E-03	0.2	Pde3b	3E-03	0.1
Ier2	2E-17	0.2	Leprotl1	8E-08	0.1
Gps1	7E-04	0.2	Tmed2	4E-17	0.1
Nudt19	1E-03	0.2	Ikbip	7E-09	0.1
Osgepl1	2E-01	0.2	Ppic	2E-13	0.1
Birc2	1E-02	0.2	Fbxw9	7E-01	0.1
Lrpprc	7E-03	0.2	Ssbp2	4E-08	0.1
Ddx18	2E-08	0.2	Med17	3E-02	0.1
Scrn2	2E-02	0.2	Hddc2	2E-02	0.1
Psmb5	9E-12	0.2	Naa80	4E-02	0.1
Cep85l	2E-02	0.2	Rbm26	5E-05	0.1
BC004004	8E-06	0.2	Ets2	5E-06	0.1
Trp53bp1	1E-05	0.2	Hmox2	1E-04	0.1
Med27	7E-04	0.2	Sec61a1	4E-05	0.1
Mrpl43	1E-07	0.2	Itm2c	4E-12	0.1
Pecr	6E-03	0.2	Rps21	8E-48	0.1
Rpgr	4E-02	0.2	Son	3E-13	0.1
Tbc1d7	9E-03	0.2	Taf7	4E-03	0.1
Mphosph6	1E-02	0.2	Rnf114	4E-02	0.1
Ddx3y	9E-08	0.2	Idua	4E-02	0.1
Lzic	3E-03	0.2	Sdc2	2E-02	0.1
Siah2	6E-04	0.2	Sec22b	2E-10	0.1
Arhgef17	2E-03	0.2	Zmiz1	1E-07	0.1
Znrd1as	6E-03	0.2	Steap2	5E-02	0.1
Gm14164	3E-02	0.2	Zmym6	6E-01	0.1
Skp1a	6E-14	0.2	Pacs2	5E-01	0.1
Hnrnpab	5E-11	0.2	Klhdc3	2E-01	0.1
Etv1	6E-01	0.2	Gstcd	1E-01	0.1
Brd8	2E-05	0.2	Asap2	2E-02	0.1
Slc19a2	3E-02	0.2	4932438A13Rik	5E-07	0.1
B3gat3	1E-03	0.2	Gclc	7E-01	0.1
Bhlhe41	8E-03	0.2	Rnf139	4E-02	0.1
Rerg	2E-03	0.2	Tmem248	2E-02	0.1
Wdr34	1E-02	0.2	Gcat	7E-02	0.1
Poglut2	8E-03	0.2	Mettl6	7E-02	0.1
Mettl25	8E-02	0.2	Dnajc9	6E-05	0.1
Ralgapa2	6E-05	0.2	Higd2a	1E-06	0.1
1300002E11Rik	6E-02	0.2	Rab11fip2	8E-04	0.1
Kansl1l	9E-04	0.2	Nktr	5E-06	0.1
Ndufaf6	1E-02	0.2	Rrp12	8E-02	0.1
Pvt1	2E-07	0.2	1500004A13Rik	3E-02	0.1
Sdhaf3	4E-01	0.2	Cemip2	3E-02	0.1
Ttc4	1E-03	0.2	Atxn2l	1E-02	0.1
Acaa1a	1E-05	0.2	Hfe	9E-02	0.1
Fkbp3	3E-13	0.2	Plcl2	7E-02	0.1
Uqcrh	8E-27	0.2	Paip2	3E-13	0.1
Cemip2	1E-02	0.2	Phf7	2E-01	0.1
Nfs1	4E-04	0.2	Smim14	1E-11	0.1
Rpl36a	1E-34	0.2	Mbtd1	5E-05	0.1
Rpp30	1E-02	0.2	Mettl26	3E-02	0.1

Fibroblast					
Control			Notch2 $^{tm1.1Ecan}$		
Gene	p	FC	Gene	p	FC
Psmc1	8E-07	0.2	Zfp553	1E-01	0.2
Erbin	2E-09	0.2	Polr2d	4E-04	0.2
Phka2	1E-02	0.2	Vcan	3E-05	0.2
Triobp	1E-03	0.2	Myh9	4E-15	0.2
Med19	2E-03	0.2	Tnfrsf1a	2E-05	0.2
Prpf3	2E-03	0.2	Alg9	1E-03	0.2
Dis3	6E-04	0.2	Itgb1	1E-23	0.2
Bbs9	6E-02	0.2	Kdm3a	6E-04	0.2
Snx33	2E-01	0.2	Senp2	2E-04	0.2
Rb1cc1	8E-05	0.2	Ttc8	3E-03	0.2
Gtf3c5	4E-02	0.2	Bms1	7E-03	0.2
Ppwd1	2E-03	0.2	Otud5	8E-05	0.2
Ldha	3E-08	0.2	Tut7	4E-07	0.2
U2af2	4E-05	0.2	Haus2	3E-02	0.2
Bhlhe40	3E-04	0.2	Osbp	2E-03	0.2
Cast	1E-10	0.2	Lin52	2E-03	0.2
D030056L22Rik	1E-02	0.2	Traf7	6E-07	0.2
Ppp1cc	4E-07	0.2	Dpy19l1	2E-03	0.2
Fndc3a	5E-06	0.2	Tmem107	9E-04	0.2
Calml4	1E-01	0.2	Gpr137b	4E-01	0.2
Mbd1	8E-02	0.2	Ephb4	2E-01	0.2
Bcor	7E-03	0.2	2310057M21Rik	1E-02	0.2
Usp47	5E-06	0.2	Magee1	3E-03	0.2
Thoc1	3E-04	0.2	Ccdc9	5E-02	0.2
Med22	1E-01	0.2	Efna4	1E-01	0.2
Rps6kc1	7E-02	0.2	Sema3a	3E-05	0.2
Slc4a1ap	2E-02	0.2	Dcp1a	4E-03	0.2
Rell1	7E-03	0.2	Bivm	5E-02	0.2
Pdzd4	2E-01	0.2	Hmgn1	2E-22	0.2
Tmem128	7E-03	0.2	Glyr1	1E-06	0.2
Utp6	3E-02	0.2	Ptbp2	1E-04	0.2
Syngr2	3E-05	0.2	Smg1	6E-08	0.2
Fry	2E-17	0.2	Slc39a14	2E-03	0.2
Ccdc51	8E-02	0.2	Akap13	3E-14	0.2
Tdp1	5E-02	0.2	Ift74	9E-11	0.2
Myg1	8E-04	0.2	Iqgap1	6E-10	0.2
Cnot6	3E-05	0.2	Stard4	1E-04	0.2
Ywhae	2E-15	0.2	Prkd2	4E-03	0.2
Hoxc8	2E-04	0.2	Hira	2E-07	0.2
Clpb	7E-03	0.2	Ppp2r3c	7E-03	0.2
Edem3	5E-02	0.2	Arhgap29	2E-10	0.2
Tssc4	4E-03	0.2	Nom1	2E-03	0.2
Gfod2	1E-02	0.2	Mfn1	2E-02	0.2
Ralbp1	3E-05	0.2	Hgs	5E-03	0.2
Arl16	2E-02	0.2	Stk17b	8E-05	0.2
Pced1b	7E-01	0.2	Uhmk1	1E-03	0.2
Trabd	3E-01	0.2	Usp14	5E-10	0.2
Far1	1E-03	0.2	Naa15	1E-07	0.2
Zbtb38	5E-05	0.2	Lgmn	1E-08	0.2
Cdk5rap1	1E-01	0.2	Stard3nl	6E-04	0.2
Sirpa	2E-05	0.2	Sc5d	3E-03	0.2
Zbtb17	2E-01	0.2	Idh3g	4E-07	0.2
Mto1	2E-01	0.2	Sh3rf3	3E-09	0.2
Pls3	1E-07	0.2	Alg3	5E-02	0.2
Pop1	2E-02	0.2	Elof1	4E-08	0.2
Smad3	4E-04	0.2	Rtf1	4E-08	0.2
Emp3	6E-15	0.2	Smad1	8E-05	0.2
Parp3	1E-04	0.2	Pycard	5E-02	0.2
Usp12	1E-02	0.2	Adamts10	7E-07	0.2
Sept2	9E-03	0.2	Tle1	1E-04	0.2
Gpr89	3E-02	0.2	Fus	4E-13	0.2
Mau2	2E-03	0.2	Vps13c	1E-04	0.2
Tnks1bp1	2E-05	0.2	Slc7a6	2E-03	0.2
Man1a2	2E-09	0.2	Ssh2	1E-06	0.2

Undefined					
Control			Notch2 $^{tm1.1Ecan}$		
Gene	p	FC	Gene	p	FC
5530601H04Rik	8E-02	0.1	Tapt1	3E-01	0.1
Atp5g1	9E-01	0.1	Wdr11	6E-13	0.1
Jmy	5E-01	0.1	Snrnp27	2E-04	0.1
2610301B20Rik	4E-02	0.1	B4galt7	7E-05	0.1
Ngrn	7E-01	0.1	Trmt112	5E-07	0.1
Selenbp1	8E-01	0.1	Ndufa6	5E-04	0.1
Ankrd44	3E-01	0.1	Sertad2	2E-03	0.1
Pkia	3E-03	0.1	Elp6	5E-06	0.1
Acyp1	7E-01	0.1	Tpcn1	2E-04	0.1
Sgk1	1E-01	0.1	Klhdc8a	1E-08	0.1
Itpkb	7E-01	0.1	Atp5j2	5E-02	0.1
Nsrp1	4E-03	0.1	Tmco1	4E-06	0.1
Riok1	7E-04	0.1	Mrps9	1E-05	0.1
Rps7	1E-13	0.1	Ggcx	2E-06	0.1
2810013P06Rik	2E-02	0.1	Ccdc58	4E-04	0.1
Pfdn2	7E-02	0.1	Slc30a6	3E-05	0.1
Map3k3	1E-01	0.1	Sirt7	1E-02	0.1
Vps4a	1E-03	0.1	C1galt1c1	4E-04	0.1
Dbp	3E-01	0.1	Bcap31	1E-06	0.1
Thumpd3	6E-01	0.1	Shld1	1E-03	0.1
Otud3	6E-01	0.1	Acp6	6E-02	0.1
Naxd	2E-03	0.1	Cryz	6E-06	0.1
Maea	2E-01	0.1	Ssbp2	1E-04	0.1
Ppp1r8	5E-02	0.1	Arhgap39	5E-05	0.1
Arl3	3E-01	0.1	Rpl15	1E-11	0.1
Mrpl19	6E-03	0.1	Aqr	7E-09	0.1
Sdhaf3	7E-01	0.1	Aars	1E-08	0.1
Fbxo9	8E-01	0.1	Rpl6	4E-12	0.1
Txlng	6E-02	0.1	Polr1c	3E-07	0.1
Rnps1	3E-02	0.1	Rps3	2E-05	0.1
Hspa4	8E-01	0.1	BC005561	2E-04	0.1
Rpl7	2E-11	0.1	Syf2	1E-10	0.1
Eefsec	8E-01	0.1	Arf2	6E-05	0.1
Ascc1	9E-01	0.1	Eif2s3x	3E-07	0.1
Use1	5E-01	0.1	Pdia3	8E-01	0.1
Usp9x	3E-01	0.1	Cct2	1E-08	0.1
Cmc1	5E-04	0.1	Eif2s3y	2E-07	0.1
Nudt6	7E-01	0.1	Txnl1	8E-08	0.1
Mosmo	2E-01	0.1	Sdhaf4	4E-08	0.1
Rtf2	2E-01	0.1	Ubl5	3E-02	0.1
Cwc15	4E-02	0.1	Tada1	1E-07	0.1
Nbn	4E-03	0.1	Lsm14a	1E-09	0.1
Snhg18	1E-01	0.1	Ppp1r12c	2E-10	0.1
Rpl8	5E-10	0.1	Ubap2	8E-04	0.1
Mettl2	6E-02	0.1	Pold3	9E-04	0.1
Golim4	9E-01	0.1	Gtf2h2	1E-02	0.1
Tbrg4	1E-02	0.1	Psma6	2E-11	0.1
Rpf2	7E-01	0.1	Snx27	3E-10	0.1
Cbx3	4E-03	0.1	Cog2	4E-09	0.1
Apbb1	9E-03	0.1	Gde1	7E-06	0.1
Set	8E-01	0.1	Ap4s1	2E-03	0.1
Gtf2e2	4E-01	0.1	Gm4924	5E-04	0.1
Slf1	3E-02	0.1	Ublcp1	8E-05	0.1
Nfkbib	4E-02	0.1	Iqgap1	8E-07	0.1
Btbd1	2E-01	0.1	Snd1	3E-05	0.1
Pigm	1E-01	0.1	Rack1	3E-10	0.1
Ppil1	8E-01	0.1	Fkrp	2E-06	0.1
Pak1ip1	9E-01	0.1	Ncdn	3E-02	0.1
Snrpb2	2E-02	0.1	Ccdc167	1E-08	0.1
Hint3	2E-01	0.1	Rcn2	2E-04	0.1
Klf9	5E-01	0.1	Npm1	1E-04	0.1
Snrpd1	3E-01	0.1	Smap2	5E-12	0.1
Arhgef5	1E+00	0.1	Ak4	4E-01	0.1
Nelfe	7E-03	0.1	Gtf2e2	8E-08	0.1

Articular/Synovial Fibroblast					
Control			Notch2 $^{tm1.1Ecan}$		
Gene	p	FC	Gene	p	FC
1700021F05Rik	6E-03	0.3	Gjc1	2E-03	0.3
Kctd11	3E-01	0.3	Tmem131	1E-02	0.3
Prmt2	4E-02	0.3	Pou2f1	1E-01	0.3
Dync1li1	7E-05	0.3	Clip2	7E-01	0.3
Deaf1	5E-04	0.3	Grhpr	1E-02	0.3
Eci2	1E-07	0.3	Fig4	7E-02	0.3
Commd4	9E-07	0.3	Rock1	2E-08	0.3
Fbxo4	4E-03	0.3	Pusl1	2E-01	0.3
Tnfrsf12a	1E-04	0.3	Chm	3E-04	0.3
9630014M24Rik	1E-01	0.3	Pxn	7E-04	0.3
Borcs5	5E-02	0.3	Eea1	2E-04	0.3
Ddx19b	3E-01	0.3	Coro7	4E-01	0.3
Stam	9E-03	0.3	Azi2	6E-04	0.3
Eif4ebp2	9E-06	0.3	Snx16	1E-01	0.3
Dnase1l1	2E-02	0.3	Bhlhe41	2E-01	0.3
Atp2b1	1E-07	0.3	Saal1	8E-01	0.3
Eml3	3E-02	0.3	Zbed4	2E-01	0.3
Ly6e	4E-12	0.3	Hdgfl3	3E-04	0.3
Zfp36l2	9E-10	0.3	Pik3c3	7E-02	0.3
Pnrc1	1E-11	0.3	Bbc3	2E-01	0.3
Aggf1	1E-04	0.3	Txnip	1E-04	0.3
Tgfbrap1	2E-02	0.3	Fbxo38	2E-02	0.3
Ubc	2E-25	0.3	Fnbp1	1E-03	0.3
Herc2	1E-06	0.3	Usp28	2E-01	0.3
Gopc	2E-01	0.3	Alg13	7E-01	0.3
M6pr	2E-05	0.3	Btbd19	3E-01	0.3
Thoc6	6E-03	0.3	Pgm1	2E-01	0.3
Dgat2	3E-03	0.3	Atf2	6E-05	0.3
Plcd3	8E-02	0.3	Scyl2	9E-02	0.3
Tmc6	1E-02	0.3	Cdk6	3E-01	0.3
Hoxc6	1E-02	0.3	Far1	2E-03	0.3
Irak4	4E-02	0.3	Ccdc71l	5E-01	0.3
Vstm4	4E-01	0.3	Dcaf4	1E-01	0.3
Xkr6	7E-02	0.3	Psme2	2E-06	0.3
Hspb2	6E-02	0.3	Dennd6a	7E-04	0.3
Snapc3	1E-04	0.3	Birc3	9E-02	0.3
Esd	2E-14	0.3	Oxsr1	8E-02	0.3
Lrp3	5E-03	0.3	Pmvk	3E-02	0.3
Gm14326	5E-03	0.3	Cryzl1	1E-03	0.3
AW146154	2E-01	0.3	Narf	2E-02	0.3
Npepps	2E-08	0.3	Ttll12	2E-01	0.3
Ywhag	1E-09	0.3	Chst14	7E-01	0.3
Mrtfb	2E-02	0.3	Zfp384	3E-01	0.3
Arl6	2E-02	0.3	Itgb1	1E-16	0.3
Tmem151a	3E-02	0.3	Eaf2	5E-01	0.3
Spice1	2E-02	0.3	Castor2	6E-02	0.3
Tcaf1	9E-07	0.3	Tk2	2E-01	0.3
Lekr1	2E-02	0.3	Tcf7l1	7E-08	0.3
Vapb	2E-07	0.3	Carns1	3E-02	0.3
Lrrc17	1E-06	0.3	Adam10	1E-05	0.3
Bbs7	1E-02	0.3	Stx4a	3E-05	0.3
Rnf115	6E-04	0.3	Cryz	3E-02	0.3
Btbd3	8E-04	0.3	Stoml1	8E-02	0.3
Nudt13	6E-02	0.3	Ptpn23	3E-02	0.3
Camkk2	8E-05	0.3	Mapk6	8E-03	0.3
Coq10a	2E-01	0.3	Prrg1	6E-02	0.3
Blcap	2E-02	0.3	Apba3	2E-01	0.3
Ppt2	3E-04	0.3	Rgl2	5E-01	0.3
Snrpn	4E-02	0.3	Dipk1b	2E-04	0.3
Snx4	1E-04	0.3	Erbin	3E-08	0.3
Sptan1	5E-14	0.3	Atg2a	2E-01	0.3
Stard7	7E-02	0.3	Mar8	6E-02	0.3
3110009E18Rik	2E-01	0.3	Tmem120a	5E-02	0.3
Slc35e4	7E-02	0.3	Atp13a1	8E-01	0.3

Limb Mesenchyme

Control Gene	p	FC	*Notch2* [tm1.1Ecan] Gene	p	FC
Samm50	9E-05	0.1	Dtd1	2E-06	0.1
Ntpcr	1E-03	0.1	Fau	1E-34	0.1
Rps11	3E-45	0.1	Dennd4c	2E-08	0.1
Fam219a	2E-01	0.1	Psme1	8E-11	0.1
Chmp1a	8E-03	0.1	Ctdnep1	1E-06	0.1
Efr3b	2E-04	0.1	Ccn5	6E-17	0.1
Taf12	7E-04	0.1	Mob1a	1E-06	0.1
Ddit3	1E-08	0.1	Nat9	2E-02	0.1
Rrp1b	8E-02	0.1	Fez2	3E-03	0.1
Mapk3	3E-04	0.1	Lrwd1	2E-04	0.1
Cript	6E-05	0.1	Castor2	7E-03	0.1
Atp5h	4E-17	0.1	Rex1bd	1E-10	0.1
Dtx3	1E-03	0.1	Thnsl2	3E-01	0.1
Acadm	7E-10	0.1	Toe1	1E-01	0.1
Rps6ka3	1E-03	0.1	Rpl39	1E-40	0.1
Amacr	1E-01	0.1	Dxo	3E-03	0.1
Ak6	8E-03	0.1	Smad4	5E-07	0.1
BC002059	5E-01	0.1	1110038B12Rik	5E-13	0.1
Pskh1	9E-01	0.1	Vps35l	2E-04	0.1
Vapb	3E-05	0.1	Clec3b	3E-12	0.1
Lyrm1	9E-02	0.1	Nectin1	1E-02	0.1
Tor1b	8E-03	0.1	Nop9	2E-03	0.1
Psmd7	1E-07	0.1	Armcx2	7E-09	0.1
Bag2	2E-02	0.1	Nck1	5E-07	0.1
Pepd	9E-02	0.1	1190005I06Rik	8E-04	0.1
Ccdc15	2E-01	0.1	Srp19	6E-12	0.1
Thrb	2E-03	0.1	1600014C10Rik	3E-02	0.1
Tecr	3E-12	0.1	Exoc8	5E-04	0.1
Tbc1d31	4E-01	0.1	Pofut1	5E-03	0.1
Mpc2	1E-18	0.1	Rnf41	2E-04	0.1
Apbb1	1E-02	0.1	Wdr4	4E-02	0.1
Rnf213	6E-02	0.1	Rplp1	9E-32	0.1
Atp5d	2E-16	0.1	Iqcc	2E-02	0.1
Zfp281	6E-02	0.1	Surf2	2E-04	0.1
Rmdn2	2E-02	0.1	Rpl13	4E-33	0.1
Gpsm1	2E-02	0.1	Sh3pxd2a	2E-13	0.1
Tmem53	2E-01	0.1	Mrps15	6E-11	0.1
Ubl7	3E-03	0.1	Asl	5E-06	0.1
Psmc3	1E-10	0.1	Ppfibp1	2E-12	0.1
Scamp3	4E-04	0.1	Phb2	1E-12	0.1
Lmbr1l	7E-02	0.1	Trmt12	3E-02	0.1
Pnp	2E-04	0.1	Asah2	4E-02	0.1
Ddost	7E-10	0.1	Rpe	8E-04	0.1
1190005I06Rik	3E-02	0.1	Vps8	7E-05	0.1
Usp16	7E-06	0.1	C1qtnf1	8E-05	0.1
Rpl24	4E-38	0.1	Ddx52	3E-06	0.1
Tlnrd1	1E-02	0.1	Tceal8	3E-13	0.1
Dvl2	3E-01	0.1	Rpl38	1E-43	0.1
Aarsd1	4E-03	0.1	Rps11	1E-35	0.1
Ubqln2	2E-01	0.1	Rbm18	3E-05	0.1
Vasn	3E-03	0.1	Zdhhc4	3E-04	0.1
Cd99l2	1E-01	0.1	Tpgs1	3E-08	0.1
Cep164	2E-04	0.1	Ykt6	1E-06	0.1
Olfm1	1E-03	0.1	Nr1h2	1E-05	0.1
Ccdc82	1E-01	0.1	Urm1	2E-07	0.1
Slc24a3	3E-05	0.1	Preb	1E-03	0.1
Rps28	2E-44	0.1	Ift43	2E-07	0.1
Rpl13	6E-40	0.1	Mlec	3E-10	0.1
Ppp2r5c	1E-05	0.1	Pias4	7E-04	0.1
Rps27	4E-33	0.1	Dohh	2E-05	0.1
C1ra	1E-01	0.1	Tbc1d10b	1E-02	0.1
Ssna1	1E-07	0.1	Manea	3E-06	0.1
Umps	2E-01	0.1	Camta1	3E-05	0.1
Ripk1	4E-04	0.1	Tars	4E-07	0.1

Chondrogenic

Control Gene	p	FC	*Notch2* [tm1.1Ecan] Gene	p	FC
Rere	5E-04	0.2	Psenen	1E-07	0.1
Gspt1	2E-11	0.2	Lgals3	1E-06	0.1
Prpf4	6E-02	0.2	Creb1	3E-02	0.1
Gtf2h4	6E-01	0.2	Zbtb12	2E-01	0.1
Tmem214	4E-04	0.2	Rbbp7	3E-05	0.1
Bbip1	4E-05	0.2	Srbd1	2E-02	0.1
Abce1	8E-05	0.2	Grsf1	1E-04	0.1
Rack1	7E-37	0.2	Bbip1	1E-06	0.1
D330023K18Rik	2E-02	0.2	Gtf2e2	1E-02	0.1
Ipo13	7E-01	0.2	Prorsd1	9E-07	0.1
Morn4	2E-01	0.2	Atpaf2	5E-01	0.1
Ddx20	8E-02	0.2	Pycr2	5E-02	0.1
Cox16	1E-09	0.2	Odf2	8E-03	0.1
Rhbdd2	4E-02	0.2	5430405H02Rik	3E-03	0.1
Apex2	1E-02	0.2	Rbm17	7E-06	0.1
Tigd2	2E-03	0.2	Zfp719	2E-02	0.1
Sltm	1E-06	0.2	Ubac1	2E-04	0.1
Sec23b	3E-03	0.2	Kmt5a	6E-07	0.1
Kcmf1	6E-06	0.2	Prorp	5E-03	0.1
Cpeb4	2E-02	0.2	Mre11a	4E-01	0.1
Fam98a	3E-05	0.2	Zzz3	3E-04	0.1
Cfap97	3E-05	0.2	Mfsd4a	6E-01	0.1
Actl6a	6E-05	0.2	Tmem39a	1E-05	0.1
Dzip3	1E-04	0.2	Rbm5	4E-05	0.1
Akap8	2E-01	0.2	Pus10	1E-01	0.1
Srp9	1E-16	0.2	Lekr1	1E-01	0.1
Phka1	3E-01	0.2	Rsrc1	7E-07	0.1
Mtg1	2E-01	0.2	Mrpl19	4E-03	0.1
Stam	7E-03	0.2	Aldh7a1	2E-05	0.1
Pacsin2	2E-05	0.2	Pdia4	2E-07	0.1
D16Ertd472e	2E-02	0.2	Pdgfa	2E-05	0.1
Cd2ap	4E-08	0.2	Rack1	6E-26	0.1
Ube2v2	4E-07	0.2	Zfp1	1E-01	0.1
Cdkl3	4E-03	0.2	Snrnp27	5E-09	0.1
2700097O09Rik	2E-02	0.2	Trim41	8E-02	0.1
Med30	1E-07	0.2	Wdr41	2E-01	0.1
Chchd4	2E-02	0.2	Fyn	9E-03	0.1
Hdhd2	6E-03	0.2	Magt1	5E-05	0.1
Gm26782	3E-03	0.2	Mars	2E-03	0.1
Zfp101	2E-01	0.2	Nop58	4E-08	0.1
Ambra1	9E-05	0.2	Sbno1	2E-05	0.1
Polr3d	3E-03	0.2	Rps27	1E-26	0.1
Uba5	3E-09	0.2	Lmo7	7E-05	0.1
Gm15283	2E-01	0.2	Rmdn1	2E-02	0.1
B130055M24Rik	2E-01	0.2	Zer1	2E-02	0.1
Ggps1	2E-02	0.2	Tnpo2	8E-02	0.1
Ddx49	1E-02	0.2	Cep57	3E-03	0.1
2310061I04Rik	2E-01	0.2	Hif1an	7E-01	0.1
Arl3	3E-09	0.2	Kdm5a	3E-04	0.1
Trim37	4E-03	0.2	Rpf2	4E-02	0.1
Appbp2	1E-05	0.2	Gstm5	1E-07	0.1
Ddx42	1E-05	0.2	Dctn4	1E-08	0.1
Acad11	2E-02	0.2	Mar7	2E-02	0.1
Rab31	4E-02	0.2	Gphn	3E-07	0.1
Phf12	6E-03	0.2	Ddx31	3E-01	0.1
Abca1	2E-05	0.2	Bbc3	2E-01	0.1
Rps27l	8E-20	0.2	Pabpc4	9E-08	0.1
Maea	2E-05	0.2	Tial1	2E-02	0.1
Tbca	1E-13	0.2	Dolk	7E-03	0.1
Tial1	4E-02	0.2	Anp32b	5E-17	0.1
Fgfr1op2	4E-08	0.2	5530601H04Rik	2E-01	0.1
Pspc1	7E-02	0.2	Ndufb9	1E-16	0.1
Atf5	2E-12	0.2	Rnf38	1E-02	0.1
Trmt10c	6E-03	0.2	C87436	7E-03	0.1

Fibroblast

Control Gene	p	FC	*Notch2* [tm1.1Ecan] Gene	p	FC
Jade1	6E-03	0.2	Atg4a	1E-03	0.2
Nomo1	8E-03	0.2	Pmp22	2E-14	0.2
Sh3rf2	7E-04	0.2	Mpst	5E-02	0.2
Otud4	2E-04	0.2	Qsox2	2E-02	0.2
Xab2	6E-02	0.2	Rbm15b	5E-04	0.2
Cavin1	3E-10	0.2	Elmod2	5E-02	0.2
Pdzd11	1E-03	0.2	Taf12	3E-07	0.2
Desi1	2E-04	0.2	Samd9l	8E-03	0.2
Ikzf5	7E-03	0.2	Slc12a6	2E-04	0.2
Ppme1	2E-03	0.2	Rtl8a	1E-03	0.2
Thop1	9E-03	0.2	Pcdh18	4E-02	0.2
Ammecr1l	4E-03	0.2	Tarsl2	4E-01	0.2
Tnfaip1	4E-02	0.2	Clu	3E-07	0.2
Rc3h1	8E-04	0.2	Dicer1	5E-04	0.2
Ogfod1	8E-02	0.2	Mrpl14	9E-10	0.2
Adcy6	7E-02	0.2	Smad7	3E-08	0.2
Znhit3	4E-02	0.2	Fnbp1	5E-06	0.2
Serpinb9	4E-02	0.2	Dpp9	5E-04	0.2
Kbtbd4	2E-01	0.2	Cdc5l	1E-05	0.2
Opa1	5E-03	0.2	Slc27a1	2E-01	0.2
Olfml2b	1E-02	0.2	Slc9a1	4E-05	0.2
Klhl2	1E-01	0.2	Ppp5c	2E-05	0.2
Zbtb7b	1E-01	0.2	B4galt1	4E-09	0.2
Hoxc10	2E-05	0.2	Fgd1	1E-03	0.2
Fuz	2E-01	0.2	Tbl1xr1	3E-05	0.2
Cep97	7E-02	0.2	Umad1	2E-06	0.2
Col6a3	5E-09	0.2	Arhgef40	4E-04	0.2
Dhx15	2E-04	0.2	Chfr	6E-05	0.2
Pde4d	3E-06	0.2	Gng12	7E-13	0.2
Anp32b	8E-06	0.2	Pomgnt1	9E-03	0.2
Zfp397	3E-02	0.2	Sgsm3	2E-02	0.2
Wdr34	9E-02	0.2	D830050J10Rik	2E-02	0.2
Zfp503	3E-03	0.2	Gabpb2	9E-06	0.2
Ugp2	2E-06	0.2	Itgb5	5E-14	0.2
Lipa	5E-05	0.2	Taco1	7E-02	0.2
Ipo7	4E-03	0.2	Dcbld1	4E-02	0.2
Cnot1	1E-05	0.2	Abl1	3E-05	0.2
Dusp16	3E-02	0.2	Thrap3	2E-13	0.2
Slc18b1	9E-02	0.2	Col6a3	3E-11	0.2
Slc25a25	3E-02	0.2	Uba6	2E-03	0.2
Tpgs2	9E-03	0.2	Tgfb1i1	6E-05	0.2
Tecr	1E-09	0.2	Snap23	1E-04	0.2
Snrpe	3E-16	0.2	Ppt2	4E-04	0.2
Aph1b	5E-02	0.2	Hmgcr	5E-05	0.2
Nckap1	5E-04	0.2	Eya3	5E-03	0.2
Mapre1	1E-07	0.2	Mxra7	2E-14	0.2
Cabin1	3E-02	0.2	Ahctf1	2E-05	0.2
Papolg	3E-01	0.2	Camk2g	3E-04	0.2
Bdh2	6E-01	0.2	Ugp2	8E-08	0.2
Qsox1	1E-04	0.2	4930453N24Rik	5E-05	0.2
Armc7	5E-01	0.2	Scd2	3E-08	0.2
Myl6	9E-22	0.2	Rara	2E-05	0.2
Fbf1	1E-01	0.2	Emc3	9E-06	0.2
Nap1l3	8E-02	0.2	Mrps22	1E-04	0.2
Akt3	3E-06	0.2	Psat1	1E-03	0.2
Tsn	9E-11	0.2	Golph3l	1E-02	0.2
Gja1	6E-06	0.2	Dis3l2	3E-03	0.2
Trmt2b	1E-01	0.2	Elp5	4E-02	0.2
Cers5	3E-04	0.2	Git2	3E-05	0.2
Nmi	7E-03	0.2	Hars	5E-05	0.2
Ppp1cb	2E-08	0.2	Sumo2	3E-22	0.2
Map3k4	9E-04	0.2	Mapk1ip1	3E-01	0.2
Elk4	5E-03	0.2	Phldb1	2E-04	0.2
Cs	4E-04	0.2	Kmt2b	1E-02	0.2

Undefined

Control Gene	p	FC	*Notch2* [tm1.1Ecan] Gene	p	FC
Pwwp3a	1E-06	0.1	6430590A07Rik	2E-01	0.1
Chuk	6E-01	0.1	Mical1	3E-07	0.1
Hspb11	4E-02	0.1	Htatip2	1E-06	0.1
Osbpl10	2E-01	0.1	Mapk1ip1l	3E-14	0.1
Slc35b3	2E-02	0.1	Tmem147	5E-07	0.1
Rpl37	6E-12	0.1	Armc8	9E-07	0.1
Usp3	2E-01	0.1	Xpnpep3	6E-05	0.1
Birc2	3E-01	0.1	Phkg2	3E-05	0.1
Rtcb	1E-02	0.1	Slc25a3	1E-01	0.1
Zbtb11	3E-01	0.1	Ahi1	2E-02	0.1
Dynlt3	4E-02	0.1	Smpd2	7E-09	0.1
Cox7a2	2E-01	0.1	Pank1	1E-05	0.1
Cab39l	1E+00	0.1	Rps25	3E-11	0.1
Ccdc191	3E-01	0.1	Mtx1	4E-08	0.1
Srp68	6E-06	0.1	Esco1	3E-03	0.1
Mbnl1	8E-01	0.1	Nek7	1E-07	0.1
Ctdsp1	3E-03	0.1	Gm1976	1E-04	0.1
P3h1	3E-01	0.1	Cdk11b	2E-07	0.1
Rbl2	7E-02	0.1	Rpl17	7E-11	0.1
Kri1	1E-02	0.1	Xrcc5	5E-06	0.1
Hsd17b10	9E-01	0.1	Epas1	2E-01	0.1
Fbl	2E-01	0.1	Kdm7a	5E-02	0.1
Trmt10a	9E-01	0.1	Mphosph10	7E-04	0.1
Nif3l1	3E-01	0.1	Exoc7	2E-08	0.1
Nudt9	2E-02	0.1	Snw1	8E-11	0.1
Acot13	2E-02	0.1	Mecr	1E-01	0.1
Slc15a4	1E-03	0.1	AU040320	9E-04	0.1
Pawr	9E-03	0.1	Atg13	1E-02	0.1
Txlna	1E-03	0.1	Rpl4	1E-03	0.1
Tmem39a	3E-01	0.1	Las1l	5E-03	0.1
Polr2k	4E-02	0.1	Tctex1d2	7E-06	0.1
Srgap3	7E-01	0.1	Sdcbp	1E-07	0.1
Ice1	1E-04	0.1	Ncln	1E-08	0.1
Smim8	7E-01	0.1	Uri1	7E-07	0.1
Faap20	5E-03	0.1	Bag6	6E-15	0.1
Ost4	4E-02	0.1	Lrba	2E-04	0.1
Vezf1	4E-01	0.1	Xrcc4	2E-06	0.1
B3glct	4E-01	0.1	Zfp579	8E-05	0.1
Nfatc3	6E-03	0.1	Dcps	4E-08	0.1
Tfe3	1E-01	0.1	Gtf2a2	3E-06	0.1
Mthfd1	6E-01	0.1			
Cmpk1	1E-01	0.1			
Jam3	9E-02	0.1			
Fam219b	4E-01	0.1			
Usp20	1E-02	0.1			
Adh5	9E-01	0.1			
Nap1l4	4E-04	0.1			
Pdhb	4E-02	0.1			
Bri3bp	2E-01	0.1			
Lpar6	7E-03	0.1			
Fubp1	7E-01	0.1			
Gcnt1	7E-03	0.1			
Mcrip1	2E-02	0.1			
Pex13	1E-02	0.1			
Gm26672	1E-01	0.1			
Taz	9E-01	0.1			
Osmr	1E-01	0.1			
Fxyd5	5E-01	0.1			
Bcas2	1E-01	0.1			
Atxn1l	9E-02	0.1			
Creb1	7E-02	0.1			
Mafg	5E-03	0.1			
Nucb2	5E-01	0.1			
Mcee	3E-02	0.1			

Articular/Synovial Fibroblast

Control Gene	p	FC	*Notch2* [tm1.1Ecan] Gene	p	FC
Cpeb3	2E-03	0.3	Fuca2	7E-06	0.3
Parp6	9E-03	0.3	Rbl2	7E-02	0.3
Slc4a3	2E-01	0.3	Calu	8E-11	0.3
Gm16759	6E-02	0.3	Atg4d	1E-01	0.3
Trpc1	1E-02	0.3	Fam117b	6E-03	0.3
Ablim1	4E-06	0.3	Idh2	2E-09	0.2
Golm1	2E-02	0.3	Snrk	4E-02	0.2
Pi4ka	4E-05	0.3	Plcd3	7E-02	0.2
Mme	7E-08	0.3	Map2k5	3E-04	0.2
Kif9	2E-01	0.3	Mpnd	9E-05	0.2
Clcn5	6E-03	0.3	Zfp974	5E-02	0.2
Atg4a	3E-04	0.3	Mtx2	1E-02	0.2
Coq8b	5E-02	0.3	Msn	1E-06	0.2
Crem	1E-02	0.3	Slc25a53	2E-01	0.2
Erap1	9E-03	0.3	Zdhhc8	2E-02	0.2
Gm39469	3E-02	0.3	Dnlz	6E-06	0.2
D11Wsu47e	3E-01	0.3	Rab11a	9E-05	0.2
Pms1	7E-02	0.3	Zc3h7a	5E-04	0.2
Nptn	2E-12	0.3	Vps26a	1E-05	0.2
Comtd1	1E-01	0.3	Kctd20	3E-01	0.2
Dok5	1E-01	0.3	Mms19	2E-01	0.2
Actr1a	2E-04	0.3	Rab13	7E-04	0.2
Dnah7b	3E-03	0.3	Ppp2r5c	6E-04	0.2
Fech	1E-03	0.3	Atp6v0d1	1E-06	0.2
Tob1	4E-05	0.3	Klf12	7E-04	0.2
Plekha8	2E-01	0.3	Cbfa2t2	6E-03	0.2
Wdr91	7E-02	0.3	Olfml3	3E-09	0.2
Echdc1	4E-03	0.3	Dcaf13	1E-01	0.2
Cmtm6	3E-01	0.3	Il10rb	2E-01	0.2
Fem1b	2E-02	0.3	Fam189b	5E-03	0.2
Sun1	2E-02	0.3	Pdcd7	1E-01	0.2
Arhgap28	1E-05	0.3	Tbk1	3E-02	0.2
Dram2	1E-03	0.3	Fkbp15	7E-03	0.2
BC052040	1E-02	0.3	Gpx8	9E-12	0.2
Dhrs11	9E-02	0.3	Slc25a20	6E-02	0.2
Poc1b	5E-01	0.3	Stat6	1E-02	0.2
Ppp2r5b	2E-01	0.3	Acvr1	2E-04	0.2
Wdr20	8E-03	0.3	Mtor	5E-03	0.2
Ethe1	3E-02	0.3	Ppp1r21	9E-04	0.2
Sugp2	9E-02	0.3	Ubtf	3E-06	0.2
Ahdc1	3E-06	0.3	Ppp1r7	3E-03	0.2
Apool	3E-03	0.3	Rab22a	4E-04	0.2
Ifrd1	2E-04	0.3	Xpo4	9E-02	0.2
Zmym3	9E-02	0.3	Prkci	1E-02	0.2
Hdac5	3E-03	0.3	Zfp512b	6E-01	0.2
Arrdc4	2E-01	0.3	AW146154	2E-01	0.2
Dip2b	3E-08	0.3	Sqstm1	2E-07	0.2
Ick	1E-04	0.3	Asna1	2E-03	0.2
Mtor	5E-02	0.3	Tmx3	2E-04	0.2
Tmem9b	5E-05	0.3	Klhl2	3E-01	0.2
Sav1	7E-05	0.3	Rnf130	2E-05	0.2
Ppard	2E-03	0.3	Tefm	4E-01	0.2
Afg1l	9E-03	0.3	Tbc1d17	2E-02	0.2
Tubgcp4	3E-02	0.3	Bcl2l2	2E-01	0.2
4930402H24Rik	1E-03	0.3	Acot13	7E-05	0.2
Nmi	6E-02	0.3	Abcd1	7E-02	0.2
Tfip11	2E-01	0.3	Lix1l	8E-05	0.2
Myo5a	8E-06	0.3	Wdsub1	1E-02	0.2
Scmh1	2E-05	0.3	Cd47	7E-07	0.2
Neurl2	4E-01	0.3	Gm7072	3E-02	0.2
Exoc5	1E-05	0.3	Ankrd40	1E-05	0.2
Kri1	8E-01	0.3	Fam118b	7E-01	0.2
Mtmr1	5E-02	0.3	Elac2	2E-01	0.2
Zfp867	5E-01	0.3	Tbc1d14	1E-01	0.2

Limb Mesenchyme

Control			$Notch2^{tm1.1Ecan}$		
Gene	p	FC	Gene	p	FC
Tceal1	5E-02	0.1	Armc10	1E-03	0.1
1700037H04Rik	3E-02	0.1	Ppp1r16a	7E-02	0.1
Calu	5E-15	0.1	Atp5e	2E-24	0.1
Cib1	5E-03	0.1	Rpl3	5E-25	0.1
Brpf3	6E-01	0.1	Nrbf2	2E-06	0.1
Fbxo33	1E+00	0.1	Apool	7E-06	0.1
Auh	5E-04	0.1	Palld	7E-11	0.1
Nup62	4E-02	0.1	Dnaja4	2E-01	0.1
2610001J05Rik	6E-04	0.1	Rpl34	2E-35	0.1
Osbpl1a	2E-05	0.1	Samd4b	3E-05	0.1
Rps3	6E-34	0.1	Sergef	1E-02	0.1
Triobp	6E-03	0.1	Sec61g	2E-25	0.1
Mtrf1l	3E-01	0.1	Dip2c	4E-07	0.1
Crat	1E+00	0.1	Vps29	2E-13	0.1
Rgl2	5E-02	0.1	Aldh1l2	9E-11	0.1
Bod1l	5E-05	0.1	Tnip1	9E-02	0.1
Ccdc50	8E-06	0.1	Bbc3	5E-04	0.1
Cradd	8E-02	0.1	Shkbp1	9E-04	0.1
Sdhd	2E-03	0.1	2410004B18Rik	3E-03	0.1
Usp28	4E-01	0.1	Gnai3	3E-07	0.1
Gtf3a	7E-03	0.1	Pigm	4E-02	0.1
Ddx39b	1E-04	0.1	Adprh	3E-07	0.1
Tceal9	1E-12	0.1	Ddx28	2E-02	0.1
Got2	6E-04	0.1	Mindy2	2E-06	0.1
Tusc1	8E-02	0.1	Tfip11	7E-03	0.1
Ift43	2E-05	0.1	Npepps	2E-10	0.1
Ndufa13	2E-18	0.1	Btbd2	5E-03	0.1
AW549877	3E-02	0.1	Mon1a	5E-02	0.1
Rbpms	3E-01	0.1	Vim	7E-15	0.1
Prkaca	1E-04	0.1	Tbc1d10a	4E-02	0.1
Thap3	1E-02	0.1	Zfp513	3E-02	0.1
Rps12	9E-30	0.1	Nif3l1	3E-03	0.1
Zfp846	2E-01	0.1	Noc4l	1E-02	0.1
Mul1	1E-01	0.1	Atp6v0a1	3E-05	0.1
Calhm2	5E-02	0.1	Rps10	3E-32	0.1
Mthfr	6E-02	0.1	Rpl17	6E-23	0.1
Rusc1	9E-02	0.1	Rpl24	7E-33	0.1
Amotl2	3E-06	0.1	Derl1	3E-11	0.1
Pdcd2l	4E-02	0.1	Zfp120	1E-03	0.1
Sec11a	7E-08	0.1	Nop16	3E-05	0.1
Chchd7	2E-04	0.1	Cmip	3E-04	0.1
Cd302	4E-04	0.1	Ago2	2E-05	0.1
Arfip1	2E-02	0.1	Glrx2	1E-09	0.1
Rasal2	3E-05	0.1	Ptprf	5E-04	0.1
Gas8	1E-01	0.1	Zdhhc3	9E-06	0.1
Zfp944	7E-02	0.1	Whamm	1E-02	0.1
Smarcc2	6E-05	0.1	Uba5	2E-06	0.1
Clta	1E-16	0.1	Taf6l	1E-01	0.1
Dtnbp1	7E-06	0.1	Cyc1	2E-08	0.1
Rer1	2E-07	0.1	Prep	3E-05	0.1
Hook2	4E-01	0.1	Iars	3E-11	0.1
Acads	2E-02	0.1	Kxd1	2E-06	0.1
Nars	2E-12	0.1	Junb	6E-06	0.1
Tfpt	6E-01	0.1	Diaph2	4E-06	0.1
Vgll3	9E-09	0.1	Rnf19b	2E-04	0.1
Vrk3	4E-02	0.1	Nfkb1	5E-07	0.1
Rpl27	1E-35	0.1	Ssr4	1E-17	0.1
Taf4	4E-01	0.1	Coq4	5E-03	0.1
Cyren	5E-02	0.1	Tusc3	1E-08	0.1
Fau	1E-41	0.1	Foxn2	2E-03	0.1
Ypel3	3E-07	0.1	Tmem192	4E-05	0.1
Mlec	2E-08	0.1	Ube2q1	3E-06	0.1
Coq6	6E-01	0.1	Sccpdh	4E-03	0.1
Cfap43	8E-01	0.1	Trmt10b	3E-02	0.1

Chondrogenic

Control			$Notch2^{tm1.1Ecan}$		
Gene	p	FC	Gene	p	FC
Ypel1	2E-01	0.2	Spryd3	2E-01	0.1
Gnl2	7E-04	0.2	Prr14	2E-02	0.1
Adh5	3E-07	0.2	Uba5	8E-04	0.1
Zfp704	5E-04	0.2	Surf2	4E-02	0.1
Banf1	2E-08	0.2	mt-Nd3	9E-10	0.1
Rpl18	6E-40	0.2	Aars	1E-05	0.1
Malt1	1E-01	0.2	Rgma	1E-01	0.1
Abcf2	2E-02	0.2	Trub1	2E-02	0.1
Slc35b3	2E-02	0.2	Tomm40	4E-04	0.1
Park7	1E-19	0.2	Tbc1d23	3E-02	0.1
Usp34	2E-05	0.2	Gli3	1E-05	0.1
Fbxl4	7E-01	0.2	Msh2	1E-01	0.1
Lsm1	2E-08	0.2	D930016D06Rik	2E-01	0.1
Sox12	2E-01	0.2	Hist1h4d	2E-02	0.1
Srpk2	7E-08	0.2	Zfp945	1E-01	0.1
Slc39a10	4E-03	0.2	Purb	1E-06	0.1
CAAA01118383.1	1E-02	0.2	Lyplal1	4E-02	0.1
Slirp	3E-09	0.2	Fam204a	1E-05	0.1
Rpf1	7E-02	0.2	Immp1l	2E-05	0.1
Rgmb	6E-01	0.2	Bphl	1E-05	0.1
Clic4	4E-10	0.2	Dynll2	2E-06	0.1
Zfp799	1E-01	0.2	Trappc11	7E-02	0.1
Ndufb6	7E-11	0.2	Ppil4	3E-03	0.1
Stard3	6E-02	0.2	Las1l	7E-03	0.1
Jkamp	7E-06	0.2	Pus7	9E-02	0.1
Vkorc1	1E-11	0.2	Ppm1m	9E-02	0.1
Ankrd16	3E-01	0.2	Xrcc5	4E-02	0.1
Ttll4	1E-01	0.2	Bnip1	1E-01	0.1
Ppp3r1	2E-03	0.2	Noa1	3E-01	0.1
2510002D24Rik	1E-02	0.2	Zfyve21	4E-06	0.1
St3gal4	2E-07	0.2	Rps6	4E-17	0.1
Rpl30	1E-44	0.2	Dmac2	6E-01	0.1
Snrnp27	5E-10	0.2	Armh4	1E-03	0.1
Mef2b	1E-03	0.2	Sdhaf1	5E-03	0.1
Spry2	1E-02	0.2	Zranb3	8E-01	0.1
Tmem107	4E-03	0.2	Hsdl2	3E-01	0.1
Ascc1	5E-04	0.2	Mapk1ip1	4E-01	0.1
Afg1l	1E-01	0.2	Sdf4	4E-13	0.1
Cct6a	8E-09	0.2	Scaf4	8E-02	0.1
Recql5	1E-01	0.2	Mtg2	4E-02	0.1
Eif2ak3	3E-02	0.2	Praf2	1E-03	0.1
H2afz	2E-48	0.2	Lrrc41	6E-02	0.1
Cdk5rap1	4E-01	0.2	Mrpl39	2E-04	0.1
Dbt	9E-02	0.2	Nsfl1c	9E-03	0.1
Csnk2a1	5E-09	0.2	Fastk	1E-05	0.1
Arel1	7E-01	0.2	Guk1	6E-08	0.1
Tti1	8E-01	0.2	Psmb5	2E-10	0.1
Prdm15	8E-01	0.2	Sept1	2E-01	0.1
3110056K07Rik	3E-01	0.2	Slc39a10	2E-02	0.1
Rabac1	1E-13	0.2	Nphp3	3E-01	0.1
Kctd3	6E-02	0.2	Rps14	4E-31	0.1
R3hcc1l	6E-02	0.2	Rpl21	9E-26	0.1
Aamp	3E-07	0.2	Naxd	1E-04	0.1
Rps25	7E-37	0.2	Letmd1	9E-01	0.1
Rab24	1E-06	0.2	Endov	5E-01	0.1
Bloc1s5	6E-02	0.2	mt-Cytb	2E-17	0.1
Desi2	4E-06	0.2	Fastkd2	8E-01	0.1
Ubald1	2E-01	0.2	Zfp955b	1E+00	0.1
Ndufaf3	7E-03	0.2	Rpl4	5E-16	0.1
Prrc1	2E-06	0.2	Trim25	9E-01	0.1
Cdk2ap2	5E-04	0.2	Nub1	2E-04	0.1
Pbdc1	6E-02	0.2	Rnf103	8E-02	0.1
Zfpm2	8E-11	0.1	Gm17106	5E-01	0.1
Amy1	4E-01	0.1	Shld2	5E-02	0.1

Fibroblast

Control			$Notch2^{tm1.1Ecan}$		
Gene	p	FC	Gene	p	FC
Rpgrip1l	6E-02	0.2	Prr14	2E-05	0.2
Rnf123	1E-01	0.2	Sehl1	2E-03	0.2
Trdmt1	6E-02	0.2	Gnai2	2E-17	0.2
Zc3h13	3E-05	0.2	Srrm1	1E-12	0.2
Cep83	9E-03	0.2	Pym1	1E-02	0.2
Gcfc2	6E-02	0.2	Epg5	4E-02	0.2
Ccar2	1E-01	0.2	B9d1	5E-06	0.2
Rcan1	6E-02	0.2	Fbn2	4E-08	0.2
Smarca5	4E-09	0.2	Mtf2	9E-06	0.2
Ocel1	8E-02	0.2	Tmem97	3E-06	0.2
Slc27a4	2E-01	0.2	Naaladl2	3E-06	0.2
Irgq	3E-01	0.2	Snrpf	9E-17	0.2
Gtf2h5	1E-07	0.2	Raf1	9E-05	0.2
Phtf2	2E-02	0.2	Hps1	5E-03	0.2
Tsc1	9E-03	0.2	Drg1	2E-04	0.2
Map3k2	1E-05	0.2	Tmem38a	4E-04	0.2
Mrps11	2E-02	0.2	Dgkh	5E-04	0.2
Cpsf2	8E-04	0.2	Anapc1	2E-06	0.2
1110059E24Rik	1E-02	0.2	Usp5	3E-05	0.2
Cdkl2	9E-02	0.2	H2afy	2E-05	0.2
Rfx5	1E-02	0.2	Afg3l1	5E-03	0.2
Nif3l1	7E-02	0.2	Numbl	2E-04	0.2
Uap1	2E-03	0.2	Taf3	2E-04	0.2
Sfswap	2E-04	0.2	Icam1	1E-02	0.2
Lamc1	4E-05	0.2	Trmt2b	7E-02	0.2
Fam241a	4E-02	0.2	Trrap	7E-02	0.2
Snrpf	2E-08	0.2	Sav1	3E-05	0.2
Arhgef40	8E-03	0.2	Lgr4	3E-06	0.2
Elovl5	3E-01	0.2	Ifrd2	4E-04	0.2
Abhd17b	3E-05	0.2	Erbin	2E-09	0.2
Alms1	7E-03	0.2	Grk2	1E-03	0.2
Kars	2E-02	0.2	Fbxo17	9E-03	0.2
Tcf12	4E-09	0.2	Sf3b2	4E-16	0.2
Rab26os	5E-01	0.2	Cd82	1E-03	0.2
Sec24b	4E-03	0.2	Eml4	3E-05	0.2
Rngtt	3E-05	0.2	Flii	1E-05	0.2
Spg7	1E-03	0.2	Gcdh	1E-01	0.2
Slc31a2	1E-01	0.2	Acap2	2E-04	0.2
Zfp518a	5E-02	0.2	Ppm1h	1E-02	0.2
Lrba	1E-07	0.2	Mpdu1	1E-03	0.2
Cemip	5E-01	0.2	BC052040	2E-03	0.2
Jam2	1E-01	0.2	Oip5os1	4E-08	0.2
Ttll7	3E-02	0.2	Kbtbd3	5E-02	0.2
Zc3h12c	8E-01	0.2	Sdccag8	2E-05	0.2
Arhgef7	5E-03	0.2	Lyar	1E-05	0.2
Tpr	6E-12	0.2	Enox2	3E-04	0.2
Ppp2cb	1E-02	0.2	Bak1	2E-05	0.2
Acox3	9E-02	0.2	Tmem128	1E-01	0.2
Polr2f	4E-08	0.2	Ndufaf2	2E-09	0.2
Tmem183a	5E-04	0.2	Aopep	2E-17	0.2
Immt	6E-09	0.2	Psme1	1E-08	0.2
Arhgef11	9E-03	0.2	Eif2b1	3E-04	0.2
Dennd1b	5E-03	0.2	U2af1	3E-10	0.2
Dnajc11	2E-02	0.2	Zfp787	6E-03	0.2
Tmem184c	9E-04	0.2	Wbp11	6E-10	0.2
Prune1	1E-01	0.2	Znhit3	2E-03	0.2
Mar9	4E-01	0.2	Ifnar1	2E-01	0.2
Spast	7E-03	0.2	Khsrp	9E-05	0.2
Rprd1b	2E-02	0.2	Zbtb43	1E-02	0.2
Iqcb1	1E-02	0.2	Mbip	9E-03	0.2
Tapbp	4E-03	0.2	Tpm3	2E-10	0.2
Timp2	2E-20	0.2	Nup214	2E-04	0.2
Midn	1E-04	0.2	Kat7	7E-04	0.2
Thoc7	3E-09	0.2	Pde4a	1E-03	0.2

Undefined

Control			$Notch2^{tm1.1Ecan}$		
Gene	p	FC	Gene	p	FC
Rpl14	3E-09	0.1			
Zmat2	9E-04	0.1			
Slc30a6	9E-01	0.1			
Sp3os	1E-01	0.1			
Rbbp4	9E-04	0.1			
Dusp1	7E-01	0.1			
Magt1	2E-01	0.1			
Cnnm3	4E-02	0.1			
Nom1	4E-01	0.1			
Borcs7	5E-02	0.1			
Zcchc14	3E-01	0.1			
Ufl1	9E-03	0.1			
Nsun2	4E-01	0.1			
Rpl34	1E-10	0.1			
Gm15614	9E-02	0.1			
Tatdn3	7E-02	0.1			
mt-Co1	3E-16	0.1			
Pibf1	7E-01	0.1			
Znrd2	6E-02	0.1			
Ccny	3E-01	0.1			
Ogfr	3E-01	0.1			
Fnta	7E-01	0.1			
Npm1	2E-07	0.1			
Psenen	4E-01	0.1			
Ndfip2	9E-03	0.1			
Naa38	2E-01	0.1			
Dnajc17	4E-01	0.1			
Gprasp1	3E-01	0.1			
Rbbp6	3E-02	0.1			
Ranbp3	3E-04	0.1			
Higd2a	2E-02	0.1			
Mettl15	9E-01	0.1			
Cdk9	2E-04	0.1			
Gm20275	1E-01	0.1			
Gbe1	8E-02	0.1			
Braf	2E-01	0.1			
Snapc1	8E-01	0.1			
2900097C17Rik	1E-01	0.1			
Rnf6	2E-01	0.1			
Unc5c	2E-01	0.1			
Stat5a	1E-01	0.1			
Frs2	4E-02	0.1			
Cln3	4E-01	0.1			
Chchd6	5E-01	0.1			
Foxj2	4E-01	0.1			
Fxn	2E-01	0.1			
Tra2b	6E-01	0.1			
Ddx46	6E-03	0.1			
Tm9sf1	5E-02	0.1			
Hoxc10	6E-01	0.1			
Ckb	4E-02	0.1			
Setbp1	5E-01	0.1			
Ddx47	1E-03	0.1			
Cdadc1	4E-01	0.1			
Ppm1g	2E-01	0.1			
Dicer1	2E-02	0.1			
Med14	1E-01	0.1			
Taf1a	6E-01	0.1			
Parl	1E-02	0.1			
Tada2b	5E-02	0.1			
Spaca6	2E-03	0.1			
Bcl2l2	5E-03	0.1			
Timm50	2E-02	0.1			
Gsto1	1E-07	0.1			

Articular/Synovial Fibroblast

Control			$Notch2^{tm1.1Ecan}$		
Gene	p	FC	Gene	p	FC
Sgce	2E-06	0.3	Zfp618	4E-01	0.2
Asna1	1E-02	0.3	Epm2a	2E-01	0.2
Tmem19	5E-02	0.3	Gin1	9E-02	0.2
Tmem209	3E-01	0.3	Ints3	1E-01	0.2
Parp1	3E-02	0.3	E130309D02Rik	7E-02	0.2
Pqlc2	6E-02	0.3	Pi4k2b	9E-01	0.2
Maco1	6E-05	0.3	Ap3d1	1E-05	0.2
Wdr19	8E-02	0.3	Gm4117	3E-01	0.2
Pcyox1	8E-03	0.3	Rbm45	5E-01	0.2
Anapc2	5E-03	0.3	Polb	2E-02	0.2
Ctdsp2	8E-08	0.3	Socs4	2E-01	0.2
Purg	5E-03	0.3	B4gat1	7E-04	0.2
Zfp780b	3E-02	0.3	Ptpn1	5E-05	0.2
Notch1	1E-01	0.3	Syt11	4E-03	0.2
Atxn1	4E-10	0.3	Zmat2	2E-05	0.2
Naa80	4E-01	0.3	Prelid2	4E-01	0.2
C330007P06Rik	9E-04	0.3	Fbxw4	7E-02	0.2
Hars2	6E-02	0.3	Dnajc5	1E-01	0.2
Dr1	5E-02	0.3	Tdrd7	7E-02	0.2
9530068E07Rik	4E-07	0.3	Serf2	2E-28	0.2
Pde4d	2E-02	0.3	Tcaf1	3E-03	0.2
Vps50	5E-03	0.3	Anapc2	8E-03	0.2
Umad1	4E-03	0.3	Bcl2l12	9E-02	0.2
Shb	3E-01	0.3	Kin	3E-01	0.2
Rreb1	1E-05	0.3	Cnksr3	2E-04	0.2
Abcd4	4E-02	0.3	Rmnd5b	4E-02	0.2
Ext1	1E-07	0.3	Enox2	2E-02	0.2
Wwp1	3E-05	0.3	Spats2	2E-02	0.2
Junos	3E-01	0.3	Bckdhb	1E-03	0.2
Oga	2E-05	0.3	Sbds	1E-03	0.2
Sptlc1	2E-01	0.3	Dmxl1	5E-04	0.2
Htt	7E-04	0.3	Prelid1	1E-06	0.2
Tent4a	3E-02	0.3	Klhdc8a	4E-02	0.2
Ypel3	7E-10	0.3	Dpp3	3E-02	0.2
Mgat1	3E-05	0.3	Cyth1	9E-02	0.2
Zscan29	2E-01	0.3	Ifitm2	9E-19	0.2
Synrg	5E-03	0.2	Dleu2	4E-05	0.2
Stx8	7E-06	0.2	Xiap	3E-02	0.2
Nfe2l2	2E-07	0.2	Arpc3	1E-10	0.2
Zfp229	3E-01	0.2	Abhd11	5E-02	0.2
Zbtb2	4E-02	0.2	Stx8	1E-05	0.2
Bbc3	1E-02	0.2	Cenpc1	1E-02	0.2
Prkci	7E-03	0.2	Ints1	2E-02	0.2
Il1rn	4E-02	0.2	Acot11	9E-01	0.2
Dleu2	2E-06	0.2	Slc8a1	5E-04	0.2
Lysmd2	5E-02	0.2	Uhrf1bp1l	7E-04	0.2
Itpr2	1E-03	0.2	Ogfrl1	6E-01	0.2
Tmem189	1E-04	0.2	Sh3gl1	2E-03	0.2
mt-Atp8	6E-02	0.2	Aste1	8E-01	0.2
C1ql3	1E-01	0.2	C2cd5	2E-02	0.2
Mrps21	8E-15	0.2	Ikbkb	7E-02	0.2
Cd63	5E-26	0.2	Ccdc62	7E-01	0.2
Ilvbl	3E-02	0.2	Pcmtd1	1E-06	0.2
Fosl2	3E-06	0.2	Bcas3	4E-06	0.2
Actr3	8E-10	0.2	Tm2d2	5E-08	0.2
Mar8	2E-02	0.2	Dbnl	8E-03	0.2
Gfm1	1E+00	0.2	Kbtbd2	7E-02	0.2
Dhtkd1	4E-01	0.2	Prpf18	3E-01	0.2
Golph3l	1E-01	0.2	Sel1l	9E-05	0.2
Dip2a	1E-01	0.2	Rex1bd	1E-06	0.2
Arfgef2	3E-05	0.2	Sat1	3E-07	0.2
2700054A10Rik	1E-01	0.2	Dennd5a	7E-04	0.2
Zfp655	4E-03	0.2	Vps29	8E-09	0.2
Pias4	1E-01	0.2	Rere	3E-10	0.2

Limb Mesenchyme					
Control			$Notch2^{tm1.1Ecan}$		
Gene	*p*	FC	Gene	*p*	FC
Jagn1	6E-02	0.1	Tmem50a	4E-15	0.1
Zbtb44	2E-01	0.1	Smc6	3E-13	0.1
Rbm19	1E+00	0.1	Snx33	3E-03	0.1
Fbxl8	2E-01	0.1	Rpp38	6E-04	0.1
Fbxw9	5E-02	0.1	Tbcb	1E-10	0.1
Mcrip1	2E-08	0.1	Bcl9l	2E-07	0.1
Btbd10	7E-02	0.1	Cpne1	4E-05	0.1
Rpl14	2E-29	0.1	Jarid2	4E-03	0.1
Dnttip2	1E-03	0.1	Mapkapk3	4E-03	0.1
Camk2n2	4E-01	0.1	Synj1	4E-05	0.1
Stat3	3E-04	0.1	Bag1	6E-13	0.1
Josd2	2E-04	0.1	Crtap	2E-10	0.1
Dnajc2	1E-02	0.1	Dmwd	4E-03	0.1
Kctd16	4E-03	0.1	D6Wsu163e	1E-01	0.1
Itga5	3E-02	0.1	Nmt1	4E-08	0.1
Snx30	5E-02	0.1	Man1b1	5E-03	0.1
Ndufa4	9E-18	0.1	Zfp518b	8E-02	0.1
Tcn2	5E-05	0.1	Chmp7	2E-03	0.1
Pigx	2E-02	0.1	Chmp3	4E-09	0.1
Sqstm1	2E-07	0.1	Sh3glb1	2E-14	0.1
Bcl10	3E-03	0.1	Rpl37	2E-31	0.1
Rragc	3E-04	0.1	Sucla2	1E-07	0.1
Hspa8	2E-20	0.1	Fam122a	1E-03	0.1
Gabpa	8E-01	0.1	Cyb5r1	1E-13	0.1
Sirt1	2E-01	0.1	Tsr2	2E-01	0.1
Coasy	2E-01	0.1	Eef1a1	5E-29	0.1
Edc4	3E-01	0.1	Zswim7	2E-02	0.1
Stat5b	4E-03	0.1	Atf7	7E-04	0.1
Bloc1s1	3E-04	0.1	Pigs	1E-05	0.1
Tatdn3	1E-01	0.1	Lrp3	2E-03	0.1
Idnk	1E-02	0.1	Mrpl22	2E-04	0.1
Pabpc4	5E-04	0.1	Cul7	1E-05	0.1
Ap4b1	5E-01	0.1	Gnb1	1E-11	0.1
Rcor3	2E-03	0.1	Adam10	2E-06	0.1
Lcn2	3E-02	0.1	Arf1	2E-16	0.1
Tada1	2E-01	0.1	Pja1	3E-05	0.1
Ddr2	2E-09	0.1	Tspan18	3E-02	0.1
Stimate	5E-02	0.1	Mtfmt	8E-02	0.1
Kat8	4E-01	0.1	Atxn7l3b	4E-10	0.1
Szrd1	2E-03	0.1	Edc4	2E-03	0.1
Rpgrip1l	2E-01	0.1	Pik3r1	5E-06	0.1
Mrps22	4E-01	0.1	Samd14	2E-02	0.1
Pes1	5E-02	0.1	Qars	2E-04	0.1
Vrk2	4E-03	0.1	Alkbh3	3E-05	0.1
Cd59a	6E-01	0.1	Zfp729a	3E-02	0.1
Zfp275	4E-01	0.1	Psap	3E-19	0.1
Arhgap42	2E-02	0.1	Chfr	1E-04	0.1
Orc6	1E-03	0.1	Lsm7	1E-09	0.1
Dhrs4	2E-04	0.1	Rpl23a	2E-16	0.1
Natd1	2E-01	0.1	Trim2	9E-07	0.1
Smap1	3E-09	0.1	Cog4	5E-04	0.1
Sc5d	2E-03	0.1	Usp16	3E-05	0.1
Prr13	4E-04	0.1	Fbxo30	5E-04	0.1
Atraid	9E-08	0.1	Pogk	1E-03	0.1
Adrm1	3E-03	0.1	Lias	8E-05	0.1
Arl5a	3E-02	0.1	Actr10	2E-06	0.1
Dnttip1	1E-02	0.1	Dnajc30	2E-03	0.1
Fam50a	9E-03	0.1	Ppp2r5c	2E-07	0.1
Rad23a	3E-03	0.1	Ywhah	2E-09	0.1
Dcbld2	4E-01	0.1	Clptm1l	5E-03	0.1
Cog1	3E-01	0.1	Gnb2	1E-19	0.1
Dync1li1	3E-05	0.1	Krtcap2	8E-19	0.1
Osbpl2	1E-01	0.1	H2-T23	7E-04	0.1
Chmp1b	4E-03	0.1	Mov10	8E-02	0.1

Chondrogenic					
Control			$Notch2^{tm1.1Ecan}$		
Gene	*p*	FC	Gene	*p*	FC
Tab1	3E-01	0.1	Ing4	3E-05	0.1
Dars	2E-03	0.1	Cdk11b	9E-05	0.1
Atg4c	1E-01	0.1	Rpap1	1E-01	0.1
Itpr1	3E-06	0.1	Zfp445	1E-01	0.1
Bmi1	9E-04	0.1	Bhlhe40	9E-02	0.1
Rbbp7	5E-06	0.1	Polr2k	2E-07	0.1
Akt2	2E-03	0.1	Vkorc1	3E-08	0.1
C87436	1E-03	0.1	Nae1	5E-04	0.1
Zmynd11	4E-05	0.1	Ppid	8E-06	0.1
Dleu2	5E-04	0.1	Rbm39	3E-14	0.1
Inpp5a	5E-02	0.1	Rpl18	5E-26	0.1
Zfp512	6E-03	0.1	Pdcd2l	7E-02	0.1
Micall1	2E-01	0.1	Eef1b2	8E-22	0.1
Suclg2	4E-05	0.1	Ezr	2E-01	0.1
Abraxas1	9E-01	0.1	Ccdc107	3E-06	0.1
Lpcat2	4E-03	0.1	Ppp2r5e	6E-02	0.1
Eif4ebp1	2E-13	0.1	BC005537	3E-06	0.1
Rps6	3E-28	0.1	Cpq	7E-11	0.1
Med21	4E-05	0.1	Snrnp40	1E-02	0.1
Trafd1	9E-03	0.1	Cradd	2E-01	0.1
Rbm10	2E-01	0.1	Rnf215	3E-01	0.1
Wscd2	7E-02	0.1	Gpr180	1E-03	0.1
Trim11	2E-01	0.1	Scaf8	2E-02	0.1
Dap3	1E-08	0.1	Rbm22	4E-04	0.1
Slc39a14	7E-02	0.1	Eif4ebp1	2E-11	0.1
Proser1	4E-03	0.1	Tmem223	6E-06	0.1
Mthfsl	1E-01	0.1	Rnf169	9E-03	0.1
Minpp1	2E-02	0.1	Srsf4	7E-03	0.1
Gm1976	1E-02	0.1	Ascc1	2E-03	0.1
Mtdh	2E-12	0.1	Gorasp2	3E-04	0.1
Rps14	1E-46	0.1	Atg9a	2E-02	0.1
Arhgap26	5E-02	0.1	Luc7l	1E-05	0.1
Eif2s3x	1E-03	0.1	Per1	1E-01	0.1
Ift81	8E-03	0.1	Fkbp9	7E-11	0.1
Lrrc20	1E-02	0.1	Rpl26	3E-30	0.1
Brd2	4E-05	0.1	A330023F24Rik	4E-01	0.1
Ube2j2	5E-03	0.1	Cops3	8E-06	0.1
Cox10	7E-02	0.1	Orc5	4E-01	0.1
Polr3b	5E-01	0.1	Gtf2e1	1E-01	0.1
Pwwp2a	3E-01	0.1	Commd3	2E-10	0.1
Bsdc1	4E-02	0.1	Mbip	9E-01	0.1
Dtwd2	2E-01	0.1	Thoc5	5E-01	0.1
Mettl8	2E-01	0.1	Gm10785	4E-03	0.1
Gstm5	1E-06	0.1	Srsf10	2E-07	0.1
Per3	5E-02	0.1	Thoc1	3E-02	0.1
Zpbp	2E-01	0.1	Dhx35	3E-01	0.1
Ranbp1	3E-13	0.1	Map9	2E-01	0.1
Fyttd1	4E-06	0.1	Mrpl32	1E-05	0.1
Zkscan3	1E-02	0.1	Smn1	2E-03	0.1
Akap9	2E-07	0.1	Noc3l	2E-02	0.1
Mrpl21	2E-04	0.1	Nphp1	1E-03	0.1
Ssb	7E-12	0.1	Knop1	2E-03	0.1
Cog8	2E-03	0.1	Ikbkg	3E-01	0.1
Ulk4	6E-05	0.1	Wdr61	3E-08	0.1
Pdcl	1E-03	0.1	Pnpla6	4E-01	0.1
Tbrg1	4E-09	0.1	Atf5	3E-13	0.1
Ino80	1E-02	0.1	Inppl1	9E-01	0.1
Cep57	5E-03	0.1	Rgcc	8E-03	0.1
Csad	6E-06	0.1	Gm39469	3E-01	0.1
Pnisr	4E-05	0.1	Rabl6	6E-05	0.1
Ss18	7E-03	0.1	Rpl36al	1E-17	0.1
Srsf11	6E-09	0.1	Slc25a39	8E-05	0.1
Cwc15	1E-06	0.1	Adipor1	3E-08	0.1
Upf2	7E-04	0.1	Trpm7	8E-06	0.1

Fibroblast					
Control			$Notch2^{tm1.1Ecan}$		
Gene	*p*	FC	Gene	*p*	FC
Zfp422	5E-02	0.2	Kptn	1E-02	0.2
Scrib	4E-02	0.2	Dyrk1a	4E-06	0.2
Supt20	3E-04	0.2	Gatad2a	6E-07	0.2
Cinp	8E-04	0.2	Vps9d1	6E-02	0.2
Brd8	9E-05	0.2	Gm49336	9E-04	0.2
Mapk6	3E-04	0.2	Fam193b	2E-03	0.2
Sdc2	2E-15	0.2	Ahdc1	8E-07	0.2
Pdlim5	5E-06	0.2	Lrrc75a	3E-02	0.2
Arhgef5	6E-03	0.2	E130309D02Rik	4E-02	0.2
Ica1	8E-03	0.2	Mafk	5E-04	0.2
Msl3	2E-05	0.2	Satb1	8E-02	0.2
Get4	2E-03	0.2	Hnrnpll	1E-04	0.2
Thap2	3E-04	0.2	Atg3	3E-10	0.2
Mef2d	1E-04	0.2	Zbtb1	1E-04	0.2
Vars	2E-02	0.2	Fam241a	2E-01	0.2
Mar8	5E-04	0.2	Map3k2	7E-08	0.2
Ogfrl1	1E-01	0.2	Higd1a	1E-15	0.2
Actr1b	3E-02	0.2	Arpp19	9E-12	0.2
Arhgap21	2E-08	0.2	Sntb2	5E-08	0.2
Tle3	1E+00	0.2	Hnrnpa0	2E-13	0.2
Cep19	8E-02	0.2	Phlpp1	2E-06	0.2
Mrpl28	8E-06	0.2	Gpnmb	9E-08	0.2
Skiv2l	5E-02	0.2	Ebf3	7E-04	0.2
Tcerg1	4E-04	0.2	Traf6	1E-02	0.2
Fbxo31	3E-02	0.2	Hnrnpm	8E-12	0.2
B230219D22Rik	4E-07	0.2	Med30	1E-06	0.2
Usp32	3E-05	0.2	Map2k4	2E-05	0.2
Ddx23	4E-03	0.2	Vps33a	3E-02	0.2
Galnt4	9E-02	0.2	Prune1	2E-02	0.2
Sergef	2E-02	0.2	Prcc	3E-03	0.2
Cul4b	4E-04	0.2	Rbm42	7E-04	0.2
Pcnx4	1E-02	0.2	Cnnm2	2E-04	0.2
Snx15	9E-04	0.2	Mprip	2E-12	0.2
Impad1	1E-08	0.2	Rad52	8E-01	0.2
Prep	9E-02	0.2	Pnpt1	3E-02	0.2
Fam13c	1E-04	0.2	Tarbp2	2E-04	0.2
Siva1	3E-02	0.2	Ccdc43	6E-02	0.2
Hnrnpm	3E-09	0.2	Mtor	7E-06	0.2
Pde6d	7E-04	0.2	Tob1	5E-07	0.2
Polr2d	1E-03	0.2	Bace2	8E-03	0.2
Nudt8	2E-02	0.2	Gm43672	4E-01	0.2
Mfn1	8E-03	0.2	Kdm2b	3E-03	0.2
Dnajc3	3E-11	0.2	Zfp955b	6E-02	0.2
Lims1	2E-07	0.2	Cacul1	2E-03	0.2
Anks1	2E-01	0.2	Kpna4	1E-09	0.2
Rab27b	3E-01	0.2	Zkscan17	3E-02	0.2
Cenpx	4E-04	0.2	Zfp335os	2E-02	0.2
Erap1	2E-01	0.2	Rbak	3E-01	0.2
Ephx1	3E-04	0.2	Qsox1	1E-06	0.2
Casp3	4E-02	0.2	Wdr12	3E-04	0.2
Scyl2	2E-02	0.2	Dlg5	2E-04	0.2
Arid4a	2E-03	0.2	Hspa13	6E-04	0.2
Slco3a1	2E-02	0.2	Stxbp1	2E-03	0.2
Yipf6	7E-02	0.2	4921524J17Rik	4E-03	0.2
Mov10	5E-01	0.2	Exoc7	3E-03	0.2
Hoxa5	4E-02	0.2	Taf6l	1E-03	0.2
Hacl1	4E-02	0.2	Piezo2	5E-08	0.2
Phf8	3E-01	0.2	Fam120c	2E-04	0.2
Vwa5a	3E-05	0.2	Nle1	2E-01	0.2
Fip1l1	3E-06	0.2	Ythdc2	3E-02	0.2
Ptpn11	7E-06	0.2	Zfp830	1E-03	0.2
Pbrm1	4E-10	0.2	Wdyhv1	5E-02	0.2
Ermard	3E-01	0.2	Morc2a	9E-04	0.2
Ptbp2	3E-05	0.2	D230025D16Rik	6E-02	0.2

Undefined					
Control			$Notch2^{tm1.1Ecan}$		
Gene	*p*	FC	Gene	*p*	FC
Laptm4a	4E-07	0.1			
Cycs	2E-02	0.1			
Zfp423	2E-01	0.1			
Mettl23	2E-01	0.1			
Cdk12	2E-01	0.1			
Flywch1	2E-03	0.1			
Fgfr1op2	5E-02	0.1			
Tbc1d10a	2E-01	0.1			
Casp12	5E-01	0.1			
Sf3b5	5E-01	0.1			
Med8	2E-04	0.1			
Gpaa1	2E-01	0.1			
Zbtb44	2E-01	0.1			
Prps1	9E-01	0.1			
Prkrip1	1E-03	0.1			
Eif5	8E-01	0.1			
5430416N02Rik	1E-02	0.1			
2510002D24Rik	8E-01	0.1			
Ndufb6	1E-02	0.1			
Aurkaip1	6E-02	0.1			
Myl12a	4E-02	0.1			
Eif5a	2E-01	0.1			
Rpl9	3E-07	0.1			
Btbd2	2E-09	0.1			
Shc1	1E-01	0.1			
Ndufaf1	7E-03	0.1			
Prr3	3E-03	0.1			
A730081D07Rik	2E-02	0.1			
Tmem208	2E-07	0.1			
Itgb3bp	4E-02	0.1			
Nphp1	6E-01	0.1			
Casc4	5E-01	0.1			
Pak3	2E-02	0.1			
Rplp2	2E-08	0.1			
Sephs2	1E-02	0.1			
Safb	1E-03	0.1			
H2afj	2E-01	0.1			
Fam216a	5E-02	0.1			
2310015A10Rik	7E-01	0.1			
Rpl15	5E-08	0.1			
Bax	2E-02	0.1			
Camta1	4E-03	0.1			
Vps53	3E-02	0.1			
Lrrk1	2E-01	0.1			
Gripap1	8E-03	0.1			
Rps17	2E-09	0.1			
Esf1	6E-01	0.1			
Msantd3	2E-02	0.1			
Arl6ip5	1E-04	0.1			
Aimp1	5E-02	0.1			
Smim14	1E-01	0.1			
Lemd2	7E-02	0.1			
Gm20732	5E-01	0.1			
Ptpn2	3E-01	0.1			

Articular/Synovial Fibroblast					
Control			$Notch2^{tm1.1Ecan}$		
Gene	*p*	FC	Gene	*p*	FC
Prxl2a	3E-03	0.2	Jph2	3E-01	0.2
Map1b	6E-06	0.2	Pus1	2E-01	0.2
5031439G07Rik	6E-03	0.2	Snhg9	4E-01	0.2
Mfap2	2E-02	0.2	Tle6	7E-02	0.2
Ccdc136	3E-03	0.2	Zfp644	1E-05	0.2
Vti1a	2E-06	0.2	Zbtb45	4E-01	0.2
Gnptab	7E-02	0.2	Psmb10	1E-02	0.2
Nt5dc3	6E-02	0.2	Sh3kbp1	1E-04	0.2
Lamp2	2E-19	0.2	Cib1	1E-03	0.2
Tmem132a	2E-02	0.2	Zdhhc5	6E-02	0.2
Adck2	5E-02	0.2	Cramp1l	3E-01	0.2
Ccdc43	7E-02	0.2	Cenpv	2E-01	0.2
Trappc1	2E-04	0.2	Tusc1	1E-01	0.2
Cpne3	2E-04	0.2	Chp1	4E-04	0.2
Babam2	2E-08	0.2	Hspg2	1E-07	0.2
Dnajc10	2E-07	0.2	Zfp932	2E-01	0.2
Dhx38	4E-01	0.2	Lipo3	1E+00	0.2
Lrch3	1E-04	0.2	Stx7	2E-03	0.2
Pot1b	5E-01	0.2	Dlg5	6E-04	0.2
Wipf1	8E-05	0.2	Noc2l	2E-01	0.2
Wbp2	2E-03	0.2	Prkab2	2E-01	0.2
Brdt	2E-01	0.2	Bad	2E-04	0.2
Mcee	4E-05	0.2	Atat1	5E-02	0.2
Itgb1	2E-19	0.2	Thumpd2	2E-01	0.2
Rasal2	2E-07	0.2	Asph	3E-03	0.2
Spsb2	6E-03	0.2	Prxl2b	2E-03	0.2
Gpx8	8E-12	0.2	Unc119b	7E-02	0.2
Bri3	6E-20	0.2	Tmem127	1E-02	0.2
Zfp111	4E-01	0.2	Rpap2	2E-01	0.2
Capzb	3E-13	0.2	Zfp933	3E-01	0.2
Lonp2	2E-05	0.2	Klhl9	3E-03	0.2
2610307P16Rik	1E-02	0.2	Higd1a	3E-04	0.2
Lysmd4	5E-02	0.2	Atp6v0b	4E-09	0.2
Hykk	2E-01	0.2	Snx19	4E-01	0.2
Gpatch8	3E-07	0.2	Gm11944	8E-01	0.2
Zswim7	1E+00	0.2	Zdhhc9	4E-02	0.2
Tle1	2E-04	0.2	Tex264	1E-03	0.2
Znrf1	6E-03	0.2	Vps35l	3E-04	0.2
Herpud2	3E-03	0.2	Ptgr2	3E-01	0.2
Ap1ar	6E-03	0.2	Elk1	1E-01	0.2
Otud4	1E-02	0.2	Anxa7	1E-04	0.2
Zbtb8a	8E-01	0.2	Amotl1	5E-04	0.2
Cacna2d3	1E-01	0.2	Ap4m1	2E-01	0.2
Jund	8E-14	0.2	Tcf20	8E-04	0.2
Zfyve27	4E-01	0.2	Fhit	6E-02	0.2
Slc12a6	6E-04	0.2	Ndnf	4E-03	0.2
Ghdc	3E-01	0.2	Selenoo	1E-01	0.2
Ypel2	1E-03	0.2	Jmjd8	7E-01	0.2
Cxcl16	1E+00	0.2	Fundc2	4E-11	0.2
Ifi30	6E-07	0.2	Eid2b	5E-01	0.2
Vps54	1E-02	0.2	Trim11	2E-01	0.2
Macrod1	4E-06	0.2	Acap2	3E-04	0.2
Macf1	8E-11	0.2	Mdc1	4E-01	0.2
Hipk3	9E-05	0.2	Mcoln1	3E-01	0.2
Rbm42	3E-03	0.2	Pwwp2a	1E-01	0.2
Uvrag	5E-09	0.2	Mettl4	3E-01	0.2
Snx16	3E-03	0.2	Cnep1r1	2E-02	0.2
Sowahc	1E-01	0.2	Spast	7E-03	0.2
Ubn1	4E-06	0.2	Arl16	2E-02	0.2
Zfp799	1E-01	0.2	Pycard	2E-02	0.2
Fbxo8	2E-01	0.2	Ankrd16	1E+00	0.2
Ndrg3	8E-03	0.2	Mafk	5E-02	0.2
Idnk	7E-03	0.2	Osbpl1a	2E-03	0.2
Polr3c	8E-02	0.2	Khsrp	9E-04	0.2

Limb Mesenchyme						Chondrogenic						Fibroblast						Undefined						Articular/Synovial Fibroblast					
Control			*Notch2*$^{tm1.1Ecan}$			Control			*Notch2*$^{tm1.1Ecan}$			Control			*Notch2*$^{tm1.1Ecan}$			Control			*Notch2*$^{tm1.1Ecan}$			Control			*Notch2*$^{tm1.1Ecan}$		
Gene	*p*	FC	Gene	*p*	FC	Gene	*p*	FC	Gene	*p*	FC	Gene	*p*	FC	Gene	*p*	FC	Gene	*p*	FC	Gene	*p*	FC	Gene	*p*	FC	Gene	*p*	FC
Gins4	1E-01	0.1	Rbfox1	3E-01	0.1	Cdkn1a	2E-06	0.1	Slc30a6	5E-01	0.1	Nup50	1E-03	0.2	Ubl7	3E-02	0.2							Htra2	2E-02	0.2	Txndc5	1E-05	0.2
Arid5b	1E-05	0.1	Cul4a	2E-04	0.1	Pogk	1E-01	0.1	Fam160a2	9E-02	0.1	Eepd1	2E-01	0.2	Arhgap32	2E-03	0.2							Hectd4	9E-02	0.2	Ccdc137	2E-01	0.2
Lix1l	1E-05	0.1	Man1a2	4E-09	0.1	Timm23	1E-05	0.1	Apaf1	6E-01	0.1	Avil	2E-01	0.2	Tlnrd1	4E-02	0.2							Itga2b	3E-01	0.2	Pacs1	2E-02	0.2
Psme2	3E-06	0.1	Dhx16	3E-02	0.1	Pex7	4E-03	0.1	Parp11	8E-02	0.1	Nhp2	1E-03	0.2	Yeats2	2E-03	0.2							Ggct	1E-01	0.2	Ankrd49	3E-01	0.2
Hexa	3E-09	0.1	Kat5	2E-02	0.1	Guk1	2E-06	0.1	Sfmbt1	4E-03	0.1	Erh	1E-04	0.2	Sp2	3E-02	0.2							Adck1	2E-02	0.2	Zfp948	9E-02	0.2
Ddit4	4E-02	0.1	Kifc3	5E-02	0.1	Nabp2	3E-04	0.1	Dnajc11	1E-01	0.1	Glud1	2E-11	0.2	Gli3	2E-08	0.2							Dcxr	2E-02	0.2	Nup153	1E-01	0.2
Bud23	3E-02	0.1	Phax	2E-06	0.1	Trub1	2E-01	0.1	Ccnd2	6E-12	0.1	Ppp2r2d	2E-04	0.2	P3h3	3E-07	0.2							Dbnl	5E-04	0.2	Ano3	6E-01	0.2
Man1a2	8E-07	0.1	Ppan	6E-04	0.1	Tmem126a	1E-10	0.1	Nbdy	2E-10	0.1	Hmgxb4	7E-04	0.2	Pdzd4	3E-02	0.2							Ddx41	4E-02	0.2	Babam2	2E-04	0.2
Ak3	1E-03	0.1	Scand1	9E-13	0.1	Tfg	8E-07	0.1	Pabpn1	4E-05	0.1	Syncrip	2E-07	0.2	Wls	2E-16	0.2							Zfp697	6E-01	0.2	Ipmk	3E-01	0.2
Bbc3	4E-02	0.1	Enoph1	2E-02	0.1	Gls	3E-03	0.1	Polr2c	2E-04	0.1	Ints11	1E-03	0.2	Rab11b	1E-09	0.2							Spire1	1E-02	0.2	Zdhhc17	2E-01	0.2
Tmem184c	4E-02	0.1	Lss	3E-02	0.1	Lyar	7E-04	0.1	Ttc14	7E-03	0.1	Mamld1	4E-02	0.2	Six4	8E-02	0.2							Crat	1E-02	0.2	Bcl2l13	3E-02	0.2
Igf1r	7E-08	0.1	Cc2d1b	3E-03	0.1	Myo6	9E-02	0.1	Trp53bp1	3E-04	0.1	Zfp335os	4E-01	0.2	Dtd2	2E-02	0.2							Rab5c	5E-05	0.2	Cdc42ep5	7E-05	0.2
AI837181	9E-02	0.1	Zfp422	2E-04	0.1	Rpl36al	2E-23	0.1	Smndc1	2E-05	0.1	Sumf1	1E-01	0.2	Hoxd9	3E-01	0.2							Map3k2	8E-05	0.2	Rasal2	2E-03	0.2
Ppp2r5d	5E-02	0.1	Psmd3	2E-08	0.1	Hist1h2ae	4E-05	0.1	Nop56	7E-05	0.1	Dab2ip	5E-04	0.2	Lpin1	2E-02	0.2							Erlin2	9E-02	0.2	Psmd8	3E-07	0.2
Tkfc	1E-01	0.1	Rps23	2E-23	0.1	Prkca	8E-02	0.1	Preb	6E-02	0.1	C1s1	1E-02	0.2	Dpy19l4	7E-04	0.2							Ap5z1	4E-01	0.2	Mthfsl	2E-01	0.2
Cln5	9E-03	0.1	Ap3b1	2E-10	0.1	Cep295	3E-01	0.1	Tmem222	4E-05	0.1	Ypel1	7E-01	0.2	Nfyc	4E-03	0.2							Ube2h	8E-06	0.2	Cln3	5E-02	0.2
Pole4	1E-02	0.1	Ammecr1	3E-07	0.1	Zfp280d	8E-07	0.1	Fam120a	4E-07	0.1	Cd2bp2	3E-03	0.2	Pip4k2a	2E-02	0.2							Aaas	2E-01	0.2	C030014I23Rik	3E-01	0.2
Chic2	9E-05	0.1	Tceal9	6E-14	0.1	Ndufb3	5E-08	0.1	Gpr27	3E-02	0.1	Pds5b	2E-05	0.2	1500004A13Rik	4E-01	0.2							Gm15417	2E-03	0.2	Trap1	8E-01	0.2
Twf1	1E-03	0.1	Nubp1	2E-05	0.1	Srsf4	5E-02	0.1	Zswim7	1E-01	0.1	Hsp90b1	1E-09	0.2	Scaf11	1E-10	0.2							Prkacb	8E-04	0.2	Ofd1	2E-01	0.2
Twistnb	5E-03	0.1	Cdkn1a	2E-07	0.1	Son	4E-10	0.1	Ppp4r1	5E-03	0.1	Pank1	7E-02	0.2	Mettl7a1	5E-03	0.2							Rcan3	5E-02	0.2	Ubc	4E-16	0.2
Commd7	2E-03	0.1	Yju2	7E-03	0.1	Gm42047	1E-01	0.1	Tnfrsf23	4E-01	0.1	Rnpep	4E-02	0.2	Egln1	3E-05	0.2							Capza1	3E-06	0.2	Lclat1	3E-02	0.2
Fam122a	6E-03	0.1	Ankrd49	3E-04	0.1	Zfp945	3E-01	0.1	Taco1	2E-01	0.1	Ube4b	2E-05	0.2	Xndc1	1E-03	0.2							Scaf1	1E-04	0.2	Colgalt1	6E-05	0.2
Kctd20	7E-05	0.1	Slc2a13	3E-03	0.1	Pigq	2E-02	0.1	Med31	3E-04	0.1	Trnau1ap	6E-04	0.2	Tango2	1E-01	0.2							Stim1	5E-03	0.2	Zcchc2	2E-01	0.2
Reep5	2E-12	0.1	Wdr18	5E-04	0.1	Camk2d	3E-07	0.1	Tada2a	3E-02	0.1	Prkaca	6E-04	0.2	Fyco1	1E-02	0.2							Akr1b3	6E-05	0.2	Mindy1	8E-03	0.2
Mrpl30	2E-07	0.1	Ikzf5	3E-03	0.1	Gm16093	7E-01	0.1	4933421O10Rik	2E-01	0.1	Abcb7	8E-02	0.2	Prpf40b	5E-02	0.2							Arel1	1E-01	0.2	Wnk1	1E-06	0.2
Borcs8	5E-03	0.1	Map2k2	4E-08	0.1	Etv6	1E-02	0.1	Morf4l1	3E-16	0.1	Tpm3	2E-11	0.2	Cs	2E-05	0.2							Ap1m1	2E-02	0.2	BC029722	5E-04	0.2
Pdzd4	5E-02	0.1	Btbd3	3E-03	0.1	Nktr	1E-07	0.1	Chchd6	1E-04	0.1	Ttpal	2E-01	0.2	Ccdc25	4E-02	0.2							Fbxo21	3E-02	0.2	Arfgef2	1E-02	0.2
Mcm3ap	6E-02	0.1	Tmem14a	2E-03	0.1	Tcaim	1E-01	0.1	Cetn4	9E-02	0.1	Galnt13	7E-01	0.2	Ext1	8E-14	0.2							Plekhg1	9E-03	0.2	Ywhag	9E-06	0.2
Anapc15	2E-02	0.1	Cryz	7E-04	0.1	Hinfp	7E-01	0.1	Tshz1	4E-01	0.1	Chd1	1E-06	0.2	Exo5	8E-02	0.2							Pomk	1E-01	0.2	Tfpt	1E+00	0.2
Recql	3E-01	0.1	Hdac5	9E-03	0.1	Snhg1	2E-10	0.1	Serp1	5E-07	0.1	Pi4k2b	3E-03	0.2	Tead1	2E-11	0.2							Dglucy	1E-01	0.2	Nmt2	3E-02	0.2
Cpne1	1E-01	0.1	Amacr	2E-04	0.1	Coq10b	6E-01	0.1	Rplp0	4E-22	0.1	Smg1	2E-04	0.2	Ints14	6E-04	0.2							Atp6v0b	1E-13	0.2	Exoc5	6E-05	0.2
Rps13	7E-31	0.1	Bicd2	3E-04	0.1	Msra	4E-01	0.1	Pgm1	6E-02	0.1	Rhobtb3	6E-07	0.2	Gtf2a2	2E-08	0.2							Rnf111	7E-03	0.2	Trappc12	2E-01	0.2
Rpl37	1E-32	0.1	Dpm2	4E-08	0.1	Mrps10	2E-03	0.1	Cwf19l2	1E-02	0.1	Btbd11	5E-02	0.2	Qser1	7E-04	0.2							Ift27	3E-05	0.2	Qrich1	8E-03	0.2
Cdr2l	1E-01	0.1	Cyp20a1	4E-06	0.1	Zfp330	2E-02	0.1	D5Ertd579e	7E-03	0.1	Cln6	4E-02	0.2	Map3k7	6E-03	0.2							Arhgef11	3E-03	0.2	Dync2li1	1E-01	0.2
Gsn	5E-17	0.1	Fzd2	4E-10	0.1	Wdr61	1E-05	0.1	Adh5	3E-05	0.1	Ctxn1	3E-01	0.2	Cast	1E-08	0.2							Tpp2	1E-05	0.2	Bri3	2E-13	0.2
Serinc1	4E-08	0.1	Pramef8	4E-02	0.1	Setd5	4E-07	0.1	Ddx55	3E-01	0.1	Prickle2	9E-03	0.2	Clptm1	3E-06	0.2							Ptges3l	1E-01	0.2	Klf9	2E-05	0.2
Med9	2E-02	0.1	Sox12	3E-03	0.1	Ezr	3E-01	0.1	Brwd1	2E-03	0.1	Pptc7	1E-02	0.2	Tmem121	4E-03	0.2							Ptgr2	2E-02	0.2	Slmap	3E-02	0.2
Bcor	4E-02	0.1	Hacd1	3E-04	0.1	Farsa	1E-01	0.1	Ubxn4	1E-06	0.1	Mrpl27	6E-02	0.2	Efnb2	2E-05	0.2							Smarcc2	7E-07	0.2	Xpo6	2E-02	0.2
Mlycd	9E-02	0.1	Ddx39b	2E-05	0.1	Polr2h	2E-04	0.1	Vps39	7E-01	0.1	Dusp7	3E-01	0.2	Dcaf7	5E-04	0.2							Zfp438	3E-01	0.2	Ube2j1	4E-04	0.2
Dcp2	2E-01	0.1	Surf4	4E-11	0.1	E130311K13Rik	2E-01	0.1	Uqcr10	3E-12	0.1	Ruvbl2	1E-02	0.2	Rab1b	5E-07	0.2							Abhd16a	3E-03	0.2	Vps52	1E-01	0.2
4930453N24Rik	3E-02	0.1	Cipc	1E-02	0.1	Bloc1s2	4E-02	0.1	Lsg1	9E-03	0.1	Bahcc1	2E-02	0.2	Ets2	1E-03	0.2							Msn	6E-08	0.2	Gcat	1E+00	0.2
Syap1	4E-02	0.1	Pard3	4E-08	0.1	Bcs1l	4E-02	0.1	Ramac	4E-03	0.1	Otud3	1E-01	0.2	Msto1	4E-02	0.2							Vps39	7E-01	0.2	Tnpo1	2E-04	0.2
Rpl22	9E-26	0.1	Tmem151a	3E-05	0.1	Rps6kb2	8E-01	0.1	Ccnl1	7E-04	0.1	Ap1ar	4E-02	0.2	Sh3d19	7E-08	0.2							Gm14296	2E-01	0.2	Cdk5	2E-02	0.2
Rabif	8E-03	0.1	Rasa2	4E-04	0.1	Zfp91	5E-08	0.1	Sh3rf1	2E-01	0.1	Rcn2	4E-09	0.2	Mxd1	4E-02	0.2							Ogdh	1E-03	0.2	Pld3	2E-08	0.2
Ncoa7	2E-01	0.1	Cinp	5E-03	0.1	Taf6	2E-01	0.1	Ptprg	8E-04	0.1	Aagab	4E-02	0.2	Cpq	4E-16	0.2							Usp20	2E-01	0.2	Samd4b	1E-03	0.2
Fam8a1	2E-02	0.1	Zfp414	2E-02	0.1	Nckap5	2E-03	0.1	Ube2g1	2E-02	0.1	Atp6v1b2	1E-01	0.2	Marcksl1	6E-04	0.2							Tmem14a	3E-01	0.2	Tbc1d19	2E-01	0.2
Golga7	2E-02	0.1	Arhgap21	8E-08	0.1	Lsm5	3E-07	0.1	Tuba4a	1E-02	0.1	Hsd17b4	3E-03	0.2	Dhrs1	4E-05	0.2							Pet100	5E-06	0.2	Smyd2	8E-03	0.2
Prps2	4E-01	0.1	Med9	1E-04	0.1	Odr4	1E-03	0.1	Cdk8	6E-03	0.1	Zbtb1	9E-04	0.2	Cxxc5	2E-06	0.2							Atat1	5E-02	0.2	Rhou	2E-01	0.2
Pbx2	6E-02	0.1	Smap1	2E-10	0.1	Cdk11b	1E-05	0.1	Nubp2	6E-01	0.1	Wdr27	3E-01	0.2	Armt1	1E-03	0.2							Pcbp3	1E-01	0.2	Rgmb	4E-02	0.2
Skiv2l	4E-01	0.1	Chmp4b	3E-08	0.1	Adprm	1E-01	0.1	Cst3	3E-02	0.1	Polr3h	4E-02	0.2	Larp4b	6E-10	0.2							Ocrl	3E-02	0.2	Zfp267	5E-02	0.2
Zfp948	5E-02	0.1	Gm17056	1E-03	0.1	Strada	4E-01	0.1	Cyp20a1	4E-04	0.1	Srprb	1E-01	0.2	Apbb1	2E-01	0.2							Camk1d	3E-02	0.2	Aktip	9E-02	0.2
Abcd4	7E-03	0.1	Vrk2	4E-04	0.1	Tdg	1E-02	0.1	Rpl7	4E-21	0.1	Pds5a	2E-04	0.2	Vps16	3E-02	0.2							Hps5	1E-01	0.2	Phf13	2E-01	0.2
Gm4285	9E-01	0.1	Tnfrsf23	4E-04	0.1	Cfap43	9E-01	0.1	Elf1	2E-02	0.1	Ing1	8E-03	0.2	Gmcl1	7E-03	0.2							Urgcp	8E-02	0.2	Cep250	8E-02	0.2
Rabep2	4E-02	0.1	Bace1	3E-05	0.1	Rsrc2	2E-08	0.1	Cdc42se1	2E-03	0.1	Sash1	2E-10	0.2	Socs7	2E-03	0.2							Pparg	1E-03	0.2	Lrrc47	9E-01	0.2
Rpp25l	3E-02	0.1	Bcs1l	5E-03	0.1	Robo1	3E-06	0.1	Nfatc3	4E-03	0.1	Pigk	2E-04	0.2	Adprh	2E-05	0.2							Clic4	3E-09	0.2	Zfp710	1E-01	0.2
Utp11	4E-05	0.1	Gm39469	9E-03	0.1	Pdcd11	3E-03	0.1	Fyttd1	1E-06	0.1	Ireb2	3E-04	0.2	Mir22hg	1E-02	0.2							Zfp651	2E-02	0.2	Kidins220	1E-04	0.2
Prkacb	3E-02	0.1	Fli1	2E-02	0.1	Ssu72	9E-06	0.1	Cfl2	4E-06	0.1	Gmps	4E-04	0.2	Fxyd1	9E-06	0.2							Gm43462	2E-01	0.2	Utp20	1E-01	0.2
Zbtb7a	6E-04	0.1	Zfp995	2E-02	0.1	Ptges3	4E-10	0.1	Rrnad1	1E-01	0.1	Anapc5	9E-10	0.2	Senp3	2E-02	0.2							Plbd2	3E-04	0.2	Tradd	6E-01	0.2
Tceanc2	5E-01	0.1	Rrp15	1E-03	0.1	Sphk2	1E-01	0.1	Rpl10	1E-19	0.1	Herc1	8E-06	0.2	Abcb7	3E-04	0.2							Efhd2	5E-04	0.2	Sgcb	1E-04	0.2
Ick	1E-03	0.1	Fkbp14	2E-05	0.1	Aven	1E-01	0.1	Bod1	4E-05	0.1	Topors	2E-03	0.2	Dynll1	2E-04	0.2							Shld1	2E-02	0.2	Ccdc138	2E-01	0.2
Capzb	4E-09	0.1	Krcc1	1E-08	0.1	Snhg17	1E-01	0.1	Tmem126a	2E-08	0.1	Tmem14c	3E-09	0.2	Kras	2E-05	0.2							Trim8	8E-04	0.2	2210016F16Rik	1E-02	0.2
Dnajc14	3E-02	0.1	Lats1	2E-03	0.1	Gpaa1	3E-02	0.1	Pigp	1E-07	0.1	Ggta1	2E-01	0.2	Man2a2	6E-03	0.2							Stam2	2E-03	0.2	Sec24b	2E-03	0.2
Klhdc3	2E-02	0.1	Tango6	2E-03	0.1	Rmdn3	3E-02	0.1	Tnfrsf11b	5E-16	0.1	Pqlc3	4E-02	0.2	Tango6	2E-02	0.2							Arhgap12	1E-02	0.2	Capzb	2E-08	0.2
Scnm1	8E-03	0.1	Usp46	1E-04	0.1	Tmem203	1E-02	0.1	Suz12	5E-02	0.1	Cnot11	4E-02	0.2	Phf7	2E-01	0.2							Gm15283	6E-05	0.2	Serinc1	1E-06	0.2

Limb Mesenchyme

Control Gene	p	FC	$Notch2^{tm1.1Ecan}$ Gene	p	FC
Ilk	9E-04	0.1	Lrrc58	2E-06	0.1
Trmu	1E-01	0.1	Znrf1	4E-03	0.1
Zdhhc4	2E-02	0.1	Nab2	2E-04	0.1
Txnrd3	2E-02	0.1	Endov	2E-01	0.1
Psmd3	3E-03	0.1	Mrps30	5E-05	0.1
Tango2	2E-04	0.1	Aspscr1	2E-03	0.1
Hdac2	3E-05	0.1	Ppp3r1	3E-06	0.1
Maoa	6E-04	0.1	Eif6	3E-07	0.1
Ntmt1	2E-02	0.1	Keap1	2E-04	0.1
Chpf2	4E-02	0.1	Csnk1e	4E-04	0.1
Telo2	4E-01	0.1	Lzts2	1E-06	0.1
Ube2j1	3E-03	0.1	Sin3b	9E-10	0.1
Snx17	2E-03	0.1	Ubl4a	2E-07	0.1
Kremen1	6E-02	0.1	Tsen2	3E-03	0.1
2310061I04Rik	2E-01	0.1	Akap1	2E-02	0.1
Apeh	5E-01	0.1	Spryd3	2E-03	0.1
Kif3c	2E-01	0.1	Mrps23	5E-06	0.1
Haghl	5E-03	0.1	Naca	3E-21	0.1
Eno2	2E-01	0.1	Itpa	6E-03	0.1
Tmem14c	5E-07	0.1	Plscr2	2E-02	0.1
Gnai2	1E-12	0.1	Isg20l2	2E-04	0.1
Fam43a	3E-01	0.1	Traf3ip1	8E-02	0.1
Nckap5l	1E-01	0.1	Necap1	5E-04	0.1
Rxra	1E-01	0.1	Klhl20	6E-01	0.1
Rpl3	3E-25	0.1	Ccnc	1E-03	0.1
Sdhc	7E-06	0.1	Gatc	2E-03	0.1
Ttbk2	6E-02	0.1	Gtf3c6	1E-06	0.1
Xbp1	7E-03	0.1	Cxxc5	4E-05	0.1
A230057D06Rik	4E-02	0.1	Mpc2	6E-13	0.1
Psen1	8E-03	0.1	Trmt6	2E-03	0.1
Gpkow	3E-01	0.1	Timm10	3E-05	0.1
Osbpl8	5E-04	0.1	Ap1s2	4E-05	0.1
Tmem67	7E-02	0.1	Ilk	4E-10	0.1
Cnn3	9E-05	0.1	Cuta	2E-07	0.1
Wasf2	6E-07	0.1	Rps13	9E-25	0.1
Nptn	2E-06	0.1	Rbm15b	2E-04	0.1
Tead2	2E-03	0.1	Gprin3	2E-01	0.1
Ufm1	6E-05	0.1	Mroh1	5E-03	0.1
Tmem70	4E-03	0.1	Upp2	1E-03	0.1
Med11	2E-01	0.1	Add3	7E-12	0.1
Irak1	7E-03	0.1	Psmc4	6E-09	0.1
Cd82	9E-02	0.1	Uqcrc2	3E-05	0.1
Vegfa	1E-01	0.1	Egln1	2E-07	0.1
Eci2	7E-06	0.1	Sqstm1	7E-08	0.1
Nab1	1E-01	0.1	Lrp5	2E-02	0.1
Snx1	2E-02	0.1	Uba7	7E-03	0.1
Ap3d1	2E-04	0.1	Rrbp1	2E-12	0.1
Myh9	4E-08	0.1	Slc39a6	3E-05	0.1
2410004B18Rik	3E-03	0.1	Ifitm2	3E-11	0.1
Dlg4	2E-02	0.1	Hsp90ab1	9E-18	0.1
Zfp608	3E-03	0.1	Zfp944	3E-02	0.1
Yars	8E-05	0.1	Tomm34	1E-04	0.1
Rnmt	3E-01	0.1	Tnfrsf1a	1E-12	0.1
Dcaf10	3E-02	0.1	Strap	1E-08	0.1
Eif2s2	2E-16	0.1	Tyw5	2E-02	0.1
Col6a1	2E-11	0.1	Slc39a9	5E-02	0.1
Morc2a	6E-02	0.1	Atp11a	5E-04	0.1
Tmem222	2E-01	0.1	Klhdc3	4E-02	0.1
Prmt5	3E-01	0.1	Col12a1	3E-09	0.1
Bola2	2E-07	0.1	Adprhl2	2E-02	0.1
Ap2a1	1E-02	0.1	Mzt2	8E-06	0.1
Chmp7	3E-03	0.1	Ttc32	2E-01	0.1
Rab8b	4E-01	0.1	Men1	3E-03	0.1
Plin2	1E-09	0.1	Cox5b	8E-16	0.1

Chondrogenic

Control Gene	p	FC	$Notch2^{tm1.1Ecan}$ Gene	p	FC
Lgr4	9E-09	0.1	Cabin1	1E-01	0.1
Arl2	2E-05	0.1	Yars2	5E-01	0.1
Slc29a1	4E-03	0.1	Kdm5d	9E-01	0.1
Cebpzos	2E-04	0.1	Foxj3	3E-02	0.1
Ccdc28b	2E-01	0.1	Hacd1	2E-05	0.1
Eloc	6E-10	0.1	Cdk2ap1	1E-05	0.1
Tnfrsf23	9E-01	0.1	1700123O20Rik	2E-03	0.1
Pdhb	6E-04	0.1	Eif2ak3	3E-01	0.1
Snapc5	9E-05	0.1	Cep290	7E-02	0.1
Ccng1	3E-05	0.1	Parl	1E-03	0.1
Mrpl53	1E-06	0.1	Hsd17b10	2E-06	0.1
Farp1	9E-08	0.1	Edem1	1E-01	0.1
Npm1	4E-23	0.1	Hist3h2a	1E-01	0.1
Rsl24d1	1E-02	0.1	Nol11	3E-01	0.1
Rab2a	5E-15	0.1	Mecp2	2E-02	0.1
Ppid	2E-05	0.1	Chordc1	2E-02	0.1
Smg5	7E-02	0.1	Mast4	1E-15	0.1
Ssrp1	6E-06	0.1	Erh	2E-04	0.1
Smim14	1E-08	0.1	AY036118	4E-04	0.1
Ndufb10	1E-10	0.1	Zcchc14	1E-03	0.1
Gm12353	1E-01	0.1	Tgif1	3E-02	0.1
Thap1	1E-01	0.1	Rnf44	8E-01	0.1
Zkscan17	3E-01	0.1	Ddx24	6E-08	0.1
Pja2	9E-05	0.1	Nfx1	5E-03	0.1
Eif5b	2E-09	0.1	Hint1	1E-12	0.1
Pigl	4E-01	0.1	Smim19	6E-06	0.1
Tusc2	2E-02	0.1	Pip4k2c	3E-01	0.1
Mzt1	5E-05	0.1	Apoo	1E-02	0.1
Tor1a	3E-03	0.1	Mesd	8E-04	0.1
Pigv	7E-01	0.1	Zfp408	6E-02	0.1
Cars2	1E-01	0.1	mt-Nd2	2E-10	0.1
Zscan12	2E-01	0.1	Ppp4r2	3E-03	0.1
Scap	2E-01	0.1	Mrps31	8E-02	0.1
Bax	3E-10	0.1	Xkr6	3E-01	0.1
Sec23a	5E-05	0.1	Hscb	5E-01	0.1
Pou6f1	8E-01	0.1	Hoxc4	2E-01	0.1
Mrps34	2E-04	0.1	Sdhb	4E-07	0.1
Tpcn1	6E-01	0.1	4732471J01Rik	9E-02	0.1
Rbm8a	1E-07	0.1	Aasdhppt	2E-04	0.1
Nup35	2E-03	0.1	Ttc3	2E-08	0.1
Ccdc107	3E-07	0.1	Larp1	3E-03	0.1
Dis3	9E-01	0.1	Casd1	6E-02	0.1
Smarca1	8E-02	0.1	Anxa4	8E-05	0.1
Ddx5	8E-19	0.1	Tob2	1E-01	0.1
1500004A13Rik	2E-01	0.1	Ophn1	3E-03	0.1
B230307C23Rik	6E-01	0.1	Rps27l	2E-09	0.1
Bzw1	6E-09	0.1	Efnb2	2E-02	0.1
Mettl17	8E-02	0.1	Tlk2	6E-04	0.1
mt-Co1	1E-23	0.1	Efcab14	4E-03	0.1
Usp39	5E-02	0.1	Ppcs	3E-01	0.1
Fmc1	2E-04	0.1	Gpank1	4E-01	0.1
Ndufb9	4E-16	0.1	Rpn1	5E-07	0.1
Gxylt2	9E-01	0.1	Prkag1	4E-02	0.1
Cdc26	2E-05	0.1	Ddhd2	8E-03	0.1
Nr3c2	4E-01	0.1	Mrpl4	7E-05	0.1
Wdpcp	2E-01	0.1	Etf1	4E-05	0.1
Ccdc186	6E-03	0.1	Nrbp2	7E-02	0.1
Etnk1	2E-02	0.1	Fam162a	5E-06	0.1
Zfp335os	2E-01	0.1	Snx14	2E-01	0.1
Fancl	5E-02	0.1	Prpf4b	1E-04	0.1
Zmym6	4E-01	0.1	Fermt2	1E-06	0.1
Utp25	4E-02	0.1	Tab3	3E-01	0.1
Mrpl39	2E-02	0.1	Acbd3	4E-05	0.1
Wdr33	1E-03	0.1	Ngly1	1E-01	0.1

Fibroblast

Control Gene	p	FC	$Notch2^{tm1.1Ecan}$ Gene	p	FC
Adk	4E-08	0.2	Zfyve26	1E-03	0.2
Casp6	2E-02	0.2	Anxa6	5E-05	0.2
Mapk1ip1l	1E-04	0.2	Snrnp200	7E-04	0.2
Hnrnpul2	3E-05	0.2	Psmd8	1E-13	0.2
Mknk2	2E-02	0.2	Tsen15	3E-04	0.2
Agfg1	2E-04	0.2	Xpnpep1	3E-06	0.2
Zbtb21	9E-03	0.2	Naa50	1E-07	0.2
Rbmxl1	2E-03	0.2	Sf3b3	2E-05	0.2
Mrpl13	1E-03	0.2	Mapk8	2E-04	0.2
E130309D02Rik	3E-02	0.2	Myo1d	9E-12	0.2
Med31	1E-02	0.2	Ric1	7E-03	0.2
Ccng2	8E-02	0.2	Dleu2	6E-08	0.2
Pym1	7E-02	0.2	Zfp667	6E-02	0.2
Antxr2	2E-02	0.2	Slc36a4	5E-02	0.2
Zmym1	1E-01	0.2	Acat2	6E-06	0.2
Mnt	2E-01	0.2	Ifitm3	3E-21	0.2
Gtpbp6	6E-01	0.2	Ythdc1	1E-05	0.2
Ankrd28	5E-06	0.2	Usp25	2E-05	0.2
Exd2	2E-01	0.2	Dgkz	2E-02	0.2
Dgcr2	4E-03	0.2	Evl	7E-04	0.2
Vps45	7E-04	0.2	Plb1	8E-06	0.2
Azin1	4E-05	0.2	Iws1	2E-05	0.2
Emp2	1E-05	0.2	Znfx1	1E-01	0.2
Zdhhc8	2E-01	0.2	Mtpn	2E-09	0.2
Pcgf3	6E-03	0.2	Manf	2E-12	0.2
Tubgcp3	5E-02	0.2	Por	3E-05	0.2
Nqo2	5E-03	0.2	Psmd3	2E-03	0.2
Trappc1	1E-02	0.2	Plrg1	7E-03	0.2
Egln1	1E-03	0.2	Scaf4	9E-03	0.2
Kif2a	5E-05	0.2	B230219D22Rik	1E-09	0.2
Arl4c	3E-02	0.2	Lrrk1	5E-10	0.2
Plekho2	9E-03	0.2	Erlin2	2E-01	0.2
Khdrbs1	1E-09	0.2	Usp37	4E-03	0.2
Tapt1	3E-02	0.2	Zfp799	1E-02	0.2
Gm26749	1E-01	0.2	Fads3	2E-06	0.2
Klhl5	6E-02	0.2	Trappc9	1E-04	0.2
Sun1	1E-02	0.2	Uimc1	2E-03	0.2
Cdc42bpb	3E-03	0.2	Rnf214	3E-03	0.2
Psmd2	3E-07	0.2	Ubn1	4E-07	0.2
Dhx30	3E-03	0.2	Csgalnact2	7E-05	0.2
Dynlt3	4E-06	0.2	Clasp1	7E-05	0.2
Mtpn	8E-08	0.2	Zbtb6	2E-03	0.2
Syvn1	7E-01	0.2	Smu1	8E-07	0.2
Trappc13	1E-02	0.2	Stxbp6	2E-06	0.2
Polr3k	4E-02	0.2	Vdac1	1E-15	0.2
Ltbp3	1E-05	0.2	Maml2	1E-11	0.2
Wdr48	6E-02	0.2	Thoc7	4E-11	0.2
Mlh1	7E-02	0.2	Prdm16	8E-03	0.2
Ercc3	2E-01	0.2	Jam2	9E-02	0.2
Gm7072	9E-02	0.2	U2af2	2E-03	0.2
Mrps23	7E-04	0.2	Fxn	2E-02	0.2
Cetn3	4E-05	0.2	Arfgap2	6E-04	0.2
Shisa2	8E-01	0.2	Hoxa9	9E-07	0.2
Babam2	4E-06	0.2	Sox4	2E-07	0.2
Med9	1E-02	0.2	C1qtnf2	6E-03	0.2
Txnrd3	6E-01	0.2	Tank	2E-05	0.2
Brf2	9E-02	0.2	Slc25a46	6E-03	0.2
Fus	3E-09	0.2	Cherp	5E-04	0.2
Hoxa10	5E-06	0.2	Zbtb2	7E-03	0.2
Capn2	1E-05	0.2	Tnpo2	2E-02	0.2
Lipo3	7E-02	0.2	Cdc123	1E-05	0.2
Ddx31	5E-01	0.2	Slc45a4	3E-02	0.2
Chchd6	5E-02	0.2	Ndfip2	3E-05	0.2
9230114K14Rik	9E-02	0.2	Itpr3	6E-05	0.2

Undefined

Control Gene	p	FC	$Notch2^{tm1.1Ecan}$ Gene	p	FC

Articular/Synovial Fibroblast

Control Gene	p	FC	$Notch2^{tm1.1Ecan}$ Gene	p	FC
Arhgap39	6E-02	0.2	Ptpn13	3E-03	0.2
Orc5	5E-02	0.2	Impact	5E-06	0.2
Cryzl1	2E-03	0.2	Cacul1	1E-02	0.2
Per3	1E-04	0.2	Slc22a21	7E-02	0.2
Trim23	2E-02	0.2	Washc5	3E-04	0.2
Zfp143	6E-01	0.2	Wdr44	6E-03	0.2
Phc3	6E-04	0.2	Snx33	8E-03	0.2
Rapgef2	2E-06	0.2	Ginm1	2E-06	0.2
Ppfibp2	3E-01	0.2	Trappc8	2E-02	0.2
Zdhhc1	3E-02	0.2	Pten	6E-07	0.2
Dgcr2	3E-02	0.2	Dip2c	3E-06	0.2
Coil	5E-01	0.2	1810034E14Rik	2E-02	0.2
Tuba4a	4E-02	0.2	Tmed5	8E-02	0.2
Hoxc4	4E-02	0.2	Foxj2	3E-02	0.2
Tmem63a	3E-03	0.2	Rp2	2E-01	0.2
Wnk1	5E-10	0.2	Lpp	2E-09	0.2
Dnaja1	4E-14	0.2	Smpd2	3E-02	0.2
Hscb	3E-02	0.2	Apool	3E-02	0.2
Psen1	2E-04	0.2	Csnk1d	1E-02	0.2
Ttl	9E-02	0.2	Dtd2	2E-01	0.2
Mthfsl	3E-01	0.2	Plxnb2	5E-02	0.2
Osbpl1a	1E-04	0.2	Gramd4	3E-01	0.2
Ints14	6E-02	0.2	Atp6ap1	6E-07	0.2
Galk1	2E-04	0.2	Telo2	6E-02	0.2
Sept8	4E-03	0.2	Wwp1	2E-01	0.2
Sh3rf3	3E-05	0.2	Lzts2	3E-03	0.2
Mtch1	2E-09	0.2	Idh3b	2E-03	0.2
Eif2ak1	6E-02	0.2	Bag1	2E-05	0.2
Cd302	2E-05	0.2	Ints10	9E-01	0.2
Bcap29	2E-04	0.2	Vti1a	7E-05	0.2
Rita1	1E-01	0.2	Jpt1	3E-08	0.2
Homer3	7E-03	0.2	Gstp1	5E-01	0.2
Hoxa7	8E-03	0.2	Tnrc6c	1E-06	0.2
Ptbp2	1E-04	0.2	Tab3	3E-01	0.2
Vps45	2E-02	0.2	Ythdf2	4E-04	0.2
Zfp120	9E-02	0.2	Aak1	3E-04	0.2
Slc22a17	8E-02	0.2	Ppp2r3c	2E-01	0.2
Tufm	3E-02	0.2	Gnb5	3E-01	0.2
Trip6	1E-02	0.2	Mrps21	5E-08	0.2
Nsmce2	6E-09	0.2	Ric8a	9E-02	0.2
Vps41	8E-05	0.2	Dcxr	5E-02	0.2
Dync1i2	2E-10	0.2	Trip4	7E-03	0.2
Gm9844	1E-01	0.2	Iws1	1E-03	0.2
Trmt6	5E-02	0.2	Nop9	2E-01	0.2
Ifitm2	5E-20	0.2	Polr2a	2E-05	0.2
Ppia	3E-29	0.2	Csgalnact2	2E-02	0.2
Zfp142	1E-01	0.2	Macf1	2E-06	0.2
1810034E14Rik	5E-02	0.2	Zfand5	2E-04	0.2
Slc4a2	2E-02	0.2	Fam234a	2E-02	0.2
Rc3h2	3E-04	0.2	Brip1os	5E-03	0.2
Dcp1b	5E-01	0.2	Mgat4b	2E-01	0.2
Mtpn	2E-08	0.2	Ccdc68	7E-02	0.2
Nup54	3E-01	0.2	Slc35a5	1E-01	0.2
Lta4h	9E-04	0.2	Mapk9	5E-01	0.2
Lrp12	9E-03	0.2	Utp23	3E-01	0.2
Caprin2	4E-02	0.2	Trip6	4E-04	0.2
Ctif	4E-04	0.2	Rpgrip1l	4E-02	0.2
Lrrfip2	3E-03	0.2	Tatdn2	5E-01	0.2
Slc25a27	7E-02	0.2	Ece2	9E-02	0.2
Mettl3	3E-01	0.2	Tmbim6	2E-05	0.2
Txndc5	2E-08	0.2	Glud1	2E-05	0.2
Tex9	1E-01	0.2	Tnfrsf10b	2E-01	0.2
Dolk	9E-01	0.2	Ppt2	2E-03	0.2
Plagl2	1E-01	0.2	Cdk10	5E-01	0.2

Limb Mesenchyme

Control			*Notch2*$^{tm1.1Ecan}$		
Gene	*p*	FC	Gene	*p*	FC
Btbd2	5E-04	0.1	Rpl8	7E-22	0.1
Grina	1E-06	0.1	Chic2	3E-06	0.1
Iars	9E-04	0.1	Dok5	9E-02	0.1
Pla2g15	8E-03	0.1	Ube2r2	1E-11	0.1
Fam76a	3E-04	0.1	Eci2	1E-05	0.1
Ndufv3	1E-09	0.1	Rps19bp1	4E-05	0.1
Rpl41	1E-40	0.1	Pgls	9E-12	0.1
2310014F06Rik	6E-01	0.1	Ghitm	8E-12	0.1
Sin3a	1E-01	0.1	Kif5b	2E-09	0.1
Ano10	2E-02	0.1	Strn	1E-02	0.1
Hnrnpul2	7E-03	0.1	Ccdc8	2E-03	0.1
Serpinb6a	8E-23	0.1	Hspa8	3E-19	0.1
Steap3	2E-10	0.1	1810055G02Rik	2E-04	0.1
Actr2	2E-04	0.1	Rps6kb2	2E-04	0.1
Rpl15	4E-30	0.1	Clcn7	7E-02	0.1
Ino80e	2E-02	0.1	Ociad1	2E-05	0.1
Dda1	2E-03	0.1	Lactb	4E-04	0.1
Slc11a2	2E-01	0.1	Stx2	1E-02	0.1
Tmem41a	2E-01	0.1	Akr7a5	2E-04	0.1
Rpl12	7E-22	0.1	Lmbr1	2E-04	0.1
Mmadhc	2E-02	0.1	Wbp1	1E-04	0.1
Ndufs8	7E-04	0.1	C1qtnf6	5E-07	0.1
Sirt7	5E-02	0.1	Rpap2	1E-02	0.1
Tusc3	1E-05	0.1	Magohb	3E-06	0.1
Txndc9	5E-04	0.1	Ppp2r1a	2E-06	0.1
Mrpl40	1E-02	0.1	Acadm	3E-09	0.1
Psme3	2E-01	0.1	Pigc	1E-03	0.1
Nrbp1	1E-02	0.1	Vps52	1E-02	0.1
Fas	1E-01	0.1	Maged1	3E-09	0.1
Scrib	2E-01	0.1	Mospd3	3E-04	0.1
Bop1	2E-01	0.1	Cfap298	4E-03	0.1
Qpctl	9E-01	0.1	Hmgn1	7E-14	0.1
Adprh	3E-03	0.1	Btbd10	4E-05	0.1
Mtch2	1E-05	0.1	Dhx38	1E-02	0.1
Prdx2	8E-11	0.1	Szrd1	3E-06	0.1
Spcs1	7E-11	0.1	Tmem138	1E-02	0.1
Rps7	9E-26	0.1	Sgcd	6E-04	0.1
Chm	6E-03	0.1	Psme2	3E-08	0.1
Zfp995	2E-01	0.1	Fam114a2	2E-04	0.1
Phf3	2E-04	0.1	Heatr5a	6E-04	0.1
Rpl34	2E-30	0.1	Dnajc21	9E-04	0.1
Mb21d2	5E-01	0.1	Cdc5l	2E-06	0.1
Aldoa	4E-11	0.1	Ndufs2	1E-10	0.1
Flt3l	3E-01	0.1	Maz	2E-04	0.1
Sucla2	1E-02	0.1	Sra1	8E-08	0.1
Prkaa1	1E-01	0.1	Glul	4E-03	0.1
Brdt	9E-02	0.1	Wdr45b	9E-04	0.1
Eif1a	2E-06	0.1	Cuedc2	8E-07	0.1
Fkbp10	1E-03	0.1	Ddost	6E-11	0.1
Dcaf6	1E-04	0.1	Vamp4	1E-07	0.1
Usb1	8E-02	0.1	Ndst1	2E-03	0.1
Zfp74	3E-02	0.1	Reep5	4E-12	0.1
Micall1	7E-02	0.1	Arhgap1	7E-05	0.1
Tbck	1E-03	0.1	Fgd1	1E-04	0.1
Rpl9	6E-26	0.1	Rps12	7E-18	0.1
Rraga	3E-03	0.1	Coa3	1E-10	0.1
Dlgap4	5E-04	0.1	Eif4b	1E-08	0.1
Abhd17a	2E-03	0.1	Cdc34	2E-07	0.1
Cyp4v3	2E-01	0.1	Zfyve19	2E-02	0.1
Rab7b	6E-03	0.1	Pdap1	6E-07	0.1
Cox4i1	5E-20	0.1	3830406C13Rik	5E-04	0.1
Dip2b	1E-05	0.1	Gm17018	5E-06	0.1
Timm8a1	1E-01	0.1	Tprgl	3E-08	0.1
Tmem134	1E-04	0.1	Uqcrc1	5E-09	0.1

Chondrogenic

Control			*Notch2*$^{tm1.1Ecan}$		
Gene	*p*	FC	Gene	*p*	FC
Rpl19	4E-40	0.1	Gm11084	2E-01	0.1
Zkscan8	1E+00	0.1	Aco2	2E-04	0.1
Sgms1	1E-03	0.1	R3hdm2	4E-04	0.1
BC003965	1E-02	0.1	1110008P14Rik	5E-07	0.1
Rps26	2E-28	0.1	Ttll4	4E-01	0.1
Ccdc141	3E-01	0.1	Ing3	6E-01	0.1
Aftph	6E-02	0.1	Arhgap39	3E-01	0.1
Ddx50	2E-09	0.1	Izumo4	5E-01	0.1
Smn1	7E-03	0.1	Akap7	2E-03	0.1
2410022M11Rik	7E-02	0.1	Pex7	2E-01	0.1
Naa80	2E-01	0.1	Rtraf	3E-10	0.1
Vgll4	2E-04	0.1	Rcbtb1	9E-02	0.1
Blmh	2E-02	0.1	Lcor	9E-04	0.1
Pfdn4	2E-05	0.1	Sirt7	4E-01	0.1
Akap8l	6E-03	0.1	B3galnt2	4E-01	0.1
Echs1	9E-06	0.1	Mrpl38	3E-02	0.1
Maip1	5E-03	0.1	Gpc3	1E+00	0.1
Qpctl	5E-01	0.1	Eps15	2E-01	0.1
Slx4ip	9E-01	0.1	Zfp704	5E-03	0.1
Clk1	1E-02	0.1	Dmd	2E-07	0.1
Itm2c	8E-06	0.1	Pdia3	1E-09	0.1
Ttc3	1E-09	0.1	Gpbp1	4E-07	0.1
Carnmt1	1E-04	0.1	Slc25a27	2E-01	0.1
BC005537	4E-05	0.1	Plxna2	3E-01	0.1
Avil	3E-01	0.1	Ssr4	9E-11	0.1
Mbd2	2E-07	0.1	Sarnp	1E-06	0.1
Tarbp2	1E-04	0.1	Dop1a	6E-02	0.1
Cnn3	1E-10	0.1	Gprasp1	1E-02	0.1
Ddx24	4E-08	0.1	Stam	2E-02	0.1
Eef1d	6E-13	0.1	Nif3l1	3E-01	0.1
Ppip5k1	7E-01	0.1	Dyrk1a	3E-04	0.1
Stard5	2E-01	0.1	Rpl19	5E-29	0.1
Emc7	1E-08	0.1	Phykpl	1E-01	0.1
Mrs2	4E-01	0.1	Invs	8E-02	0.1
Ttc13	5E-01	0.1	Tmem18	4E-01	0.1
Mrpl1	4E-02	0.1	Twsg1	3E-02	0.1
Dph5	1E-01	0.1	Tasor2	4E-02	0.1
Aars	1E-05	0.1	Gm20732	2E-01	0.1
4930430F08Rik	1E-01	0.1	Mrpl43	4E-04	0.1
Slain2	8E-03	0.1	Fam216a	2E-02	0.1
Dcaf1	1E-01	0.1	Tchp	6E-01	0.1
Rufy3	5E-02	0.1	Snap47	2E-02	0.1
Ndufs5	5E-16	0.1	Mrto4	8E-05	0.1
Dhrs7	8E-04	0.1	Utp15	8E-01	0.1
Slc25a12	8E-02	0.1	Manf	8E-12	0.1
Mars	3E-04	0.1	Tmem80	5E-02	0.1
Rbbp6	3E-04	0.1	Slc45a4	8E-01	0.1
Ccdc90b	1E-05	0.1	Rps17	1E-18	0.1
Cdipt	3E-02	0.1	U2surp	1E-04	0.1
Gpcpd1	2E-01	0.1	Snhg17	1E-01	0.1
Pdxdc1	1E-03	0.1	Ints3	9E-01	0.1
Nae1	1E-02	0.1	Gpr137b	2E-02	0.1
Mks1	6E-02	0.1	Ubxn2a	7E-03	0.1
Slc41a1	3E-01	0.1	Cttnbp2nl	3E-01	0.1
Aatf	3E-02	0.1	Prmt7	2E-01	0.1
Gm16759	1E-02	0.1	Mphosph6	2E-02	0.1
Grhpr	2E-02	0.1	Aldh2	1E-05	0.1
Rpl21	6E-31	0.1	Esr1	1E-02	0.1
Cep44	2E-01	0.1	Unk	3E-01	0.1
Higd2a	8E-06	0.1	Rpl5	2E-15	0.1
Smndc1	3E-05	0.1	Mrpl17	5E-07	0.1
1600020E01Rik	2E-01	0.1	Eci1	2E-03	0.1
Denr	3E-07	0.1	Rps26	2E-09	0.1
Sec22b	9E-08	0.1	Lgr4	3E-07	0.1

Fibroblast

Control			*Notch2*$^{tm1.1Ecan}$		
Gene	*p*	FC	Gene	*p*	FC
Ttc37	4E-02	0.2	Cd44	1E-05	0.2
Commd2	2E-03	0.2	Plekha4	5E-02	0.2
Ilk	4E-04	0.2	Col5a3	4E-04	0.2
Gnai2	2E-13	0.2	Pgk1	3E-09	0.2
Pdlim2	2E-07	0.2	Gpbp1l1	6E-06	0.2
Wdr5	7E-02	0.2	Zfp710	1E-03	0.2
AI506816	2E-03	0.2	Abhd17b	3E-05	0.2
Cox18	1E-01	0.2	Med16	3E-02	0.2
Hspb2	9E-02	0.2	Snip1	3E-03	0.2
Uba3	6E-03	0.2	Dipk1a	1E-02	0.2
Capn15	1E-02	0.2	Ppp2r5c	6E-07	0.2
Hprt	2E-05	0.2	Gnb2	6E-15	0.2
Bmpr1b	2E-03	0.2	Cluap1	6E-02	0.2
Cd99l2	4E-02	0.2	Med4	1E-03	0.2
Kdsr	5E-02	0.2	Rbbp9	2E-02	0.2
Eif2b5	4E-03	0.2	Sugp2	1E-01	0.2
Dnase1l1	6E-02	0.2	Gucd1	7E-03	0.2
Gtf2ird1	3E-05	0.2	Stxbp5	1E-05	0.2
Sgf29	1E-01	0.2	2610507B11Rik	2E-05	0.2
Dnm1l	2E-04	0.2	Plod2	1E-09	0.2
Vdac1	2E-05	0.2	Upf3a	2E-05	0.2
Mphosph8	2E-05	0.2	Nudt22	2E-01	0.2
Phc3	7E-04	0.2	Gm17018	3E-02	0.2
Sos2	7E-03	0.2	Tapt1	2E-02	0.2
Tnfrsf1a	2E-05	0.2	Rpgr	1E-01	0.2
Snx32	4E-01	0.2	Agtpbp1	3E-05	0.2
Prkag2	2E-01	0.2	Tgfa	5E-01	0.2
Psmd13	4E-06	0.2	Phf20	2E-03	0.2
Spsb3	5E-02	0.2	Trim56	2E-02	0.2
Simc1	4E-02	0.2	Ilf3	2E-05	0.2
Nup54	5E-02	0.2	Sh2b3	3E-01	0.2
Mrpl16	9E-03	0.2	Sumf2	1E-02	0.2
Apbb2	3E-07	0.2	Anapc13	3E-10	0.2
Rnps1	5E-07	0.2	Ube2n	1E-08	0.2
Ncoa7	2E-03	0.2	Adamts1	6E-05	0.2
A630089N07Rik	1E-01	0.2	Fam171a2	7E-02	0.2
Chfr	5E-04	0.2	Rab31	1E-04	0.2
Tmem184b	3E-03	0.2	Sgcd	3E-10	0.2
Odf2l	1E-01	0.2	Tagln	1E-06	0.2
Sgsm3	2E-02	0.2	Mtmr9	1E-02	0.2
Nudcd1	3E-02	0.2	Katnal1	7E-03	0.2
Fbxo22	1E-01	0.2	Rnf126	3E-02	0.2
Pnrc1	3E-05	0.2	Cipc	6E-03	0.2
Afg1l	3E-02	0.2	Prlr	6E-02	0.2
Erlin2	8E-02	0.2	Atpif1	1E-19	0.2
Oxsr1	3E-03	0.2	Oser1	3E-02	0.2
Speg	2E-01	0.2	Gigyf1	2E-01	0.2
Tia1	3E-03	0.2	Ube2a	2E-06	0.2
Wdr81	3E-01	0.2	Celf2	4E-14	0.2
Srsf1	1E-04	0.2	Slc25a24	9E-04	0.2
Gm10130	6E-02	0.2	Bcar3	3E-04	0.2
Tiam1	1E-01	0.2	Exoc1	1E-02	0.2
Skap2	2E-02	0.2	Fat1	9E-10	0.2
Rbm3	2E-09	0.2	Mthfd1	6E-03	0.2
Rgl2	4E-02	0.2	Rnps1	2E-06	0.2
Shkbp1	1E-01	0.2	Tbce	2E-03	0.2
Akr1b10	3E-02	0.2	Fam214a	1E-02	0.2
Eif4ebp2	2E-03	0.2	Prg4	1E-04	0.2
Lage3	4E-05	0.2	Yif1b	2E-11	0.2
Cd164	1E-06	0.2	Wnk1	1E-12	0.2
Ddx20	1E-01	0.2	Herc1	6E-06	0.2
Magt1	1E-06	0.2	Cep170b	3E-03	0.2
Nfyc	2E-02	0.2	Pten	2E-08	0.2
Ap3s1	5E-09	0.2	Rbm3	1E-15	0.2

Undefined

Control			*Notch2*$^{tm1.1Ecan}$		
Gene	*p*	FC	Gene	*p*	FC

Articular/Synovial Fibroblast

Control			*Notch2*$^{tm1.1Ecan}$		
Gene	*p*	FC	Gene	*p*	FC
Fbrsl1	3E-02	0.2	Rara	4E-03	0.2
Zfp287	1E-01	0.2	Zfyve19	7E-01	0.2
Wdfy3	2E-07	0.2	Pfn2	3E-01	0.2
Anapc10	9E-04	0.2	Ank3	2E-05	0.2
Smyd5	4E-01	0.2	Ttc28	6E-07	0.2
Wdr77	1E-01	0.2	Rabgef1	8E-02	0.2
Rmdn1	2E-05	0.2	Gpr137	2E-01	0.2
Sh3glb2	2E-03	0.2	Negr1	1E-01	0.2
Vps37d	2E-01	0.2	Zfp697	2E-01	0.2
Dgkh	3E-03	0.2	Ccdc102a	5E-02	0.2
Ddx6	5E-08	0.2	Inpp5k	2E-02	0.2
Sucla2	1E-04	0.2	Tbp	5E-01	0.2
Mxra7	5E-07	0.2	Krit1	1E-04	0.2
Tbc1d5	4E-07	0.2	Capza1	3E-05	0.2
Zbtb14	6E-01	0.2	Ppp1cc	1E-06	0.2
Ccdc12	1E-03	0.2	Lrp12	7E-02	0.2
Rfx7	7E-06	0.2	Rxra	2E-01	0.2
Wfs1	1E+00	0.2	Atxn3	2E-01	0.2
Psmd9	2E-03	0.2	Rnf111	3E-02	0.2
Sh3kbp1	4E-04	0.2	Ralgapa1	5E-03	0.2
Pias1	3E-06	0.2	Epha3	8E-04	0.2
Zdhhc9	6E-02	0.2	Trmt6	4E-02	0.2
Sik2	2E-05	0.2	Mif4gd	7E-01	0.2
Ccdc167	2E-03	0.2	Ahcyl1	4E-05	0.2
Aamdc	1E-04	0.2	Setd1b	6E-01	0.2
Lcmt1	6E-03	0.2	Otud4	1E-01	0.2
Mindy3	2E-03	0.2	Zadh2	1E-01	0.2
Relch	3E-05	0.2	Vps54	3E-02	0.2
Diaph2	1E-07	0.2	Fibp	5E-04	0.2
Fam222b	7E-04	0.2	Zfhx3	3E-08	0.2
Ppp1r12a	2E-05	0.2	Klhl18	5E-01	0.2
Fxyd5	6E-01	0.2	Tysnd1	1E-01	0.2
Xpo6	9E-03	0.2	Tbc1d5	4E-04	0.2
Ganc	1E-01	0.2	Med10	1E-01	0.2
Furin	6E-03	0.2	Ppp1r2	3E-03	0.2
Btbd9	7E-05	0.2	Trip10	2E-02	0.2
Zswim8	2E-04	0.2	Fdx2	3E-04	0.2
Mul1	4E-02	0.2	Fuca1	3E-07	0.2
Atraid	3E-09	0.2	Usp12	2E-01	0.2
Spa17	5E-01	0.2	Crat	3E-02	0.2
Dcaf7	5E-03	0.2	Cd302	2E-03	0.2
Crl1	4E-03	0.2	Rpp21	5E-02	0.2
Pknox1	9E-02	0.2	Ifrd1	7E-04	0.2
Carmn	6E-03	0.2	Sirt2	3E-04	0.2
Zfp809	2E-02	0.2	Nup54	2E-01	0.2
Rnf145	3E-02	0.2	Cfap410	2E-01	0.2
Leng1	3E-01	0.2	Arrdc3	6E-03	0.2
Rab36	6E-01	0.2	Zfand1	2E-01	0.2
Tkt	2E-05	0.2	Alg9	5E-01	0.2
Tatdn2	3E-01	0.2	Barx1	7E-05	0.2
Sugct	8E-04	0.2	Hes6	2E-01	0.2
Zbtb10	5E-01	0.2	Arhgef7	2E-01	0.2
Zfand2b	2E-01	0.2	Inpp1	6E-02	0.2
Usp32	1E-03	0.2	Ten1	4E-01	0.2
Pdpk1	5E-04	0.2	Atp6v1e1	2E-08	0.2
Abhd5	1E-03	0.2	Pcsk7	1E-02	0.2
Lin54	7E-02	0.2	Ino80dos	5E-01	0.2
Abat	2E-01	0.2	Nploc4	1E-02	0.2
Pacsin2	1E-02	0.2	Sucla2	3E-05	0.2
Mesd	1E-06	0.2	Rspry1	7E-03	0.2
Ift140	5E-02	0.2	Phldb2	3E-04	0.2
Avil	2E-01	0.2	Ctbs	8E-02	0.2
Smc5	1E-01	0.2	Cdk14	4E-05	0.2
Setx	7E-03	0.2	Nubp1	3E-02	0.2

Limb Mesenchyme					
Control			$Notch2^{tm1.1Ecan}$		
Gene	*p*	FC	Gene	*p*	FC
Eef1a1	4E-26	0.1	Ap3d1	7E-07	0.1
Cd2bp2	4E-03	0.1	Dhx40	3E-05	0.1
Tspan14	4E-03	0.1	Pym1	2E-02	0.1
Dtd1	4E-03	0.1	Lrfn4	7E-03	0.1
Zfp771	1E-02	0.1	Ehbp1l1	1E-04	0.1
Prkx	2E-01	0.1	Auh	5E-06	0.1
Ubald1	9E-02	0.1	Rspry1	1E-05	0.1
Ankrd42	7E-01	0.1	Mocs2	1E-05	0.1
2810032G03Rik	2E-02	0.1	Nup210l	2E-03	0.1
Rpl7a	4E-21	0.1	Rps14	4E-26	0.1
Thap7	3E-02	0.1	Ctbp1	2E-08	0.1
Ccdc9	2E-01	0.1	Ppp5c	1E-01	0.1
Actr1a	3E-03	0.1	Arfip1	7E-04	0.1
Pold2	8E-01	0.1	Mef2b	1E-01	0.1
Fut11	1E-01	0.1	Eny2	1E-10	0.1
Fam98c	7E-01	0.1	Irgq	4E-03	0.1
Dync1h1	3E-05	0.1	Ccn1	8E-08	0.1
Rpl31	2E-19	0.1	Irf3	5E-06	0.1
Stat1	2E-02	0.1	Copg1	8E-06	0.1
Psmb1	5E-10	0.1	Leng8	6E-04	0.1
Lrrc59	9E-05	0.1	Dock9	9E-05	0.1
Map4k4	2E-03	0.1	Magee1	1E-03	0.1
Runx1t1	1E-10	0.1	Ppp2r1b	5E-01	0.1
Pprc1	2E-01	0.1	Kctd2	9E-05	0.1
Stoml1	9E-02	0.1	Btbd19	7E-03	0.1
Klhl15	3E-01	0.1	Zfp110	1E-02	0.1
Cuta	3E-06	0.1	Ftsj1	3E-03	0.1
Cbr4	4E-03	0.1	Myh9	3E-08	0.1
Utp23	9E-02	0.1	Rack1	5E-16	0.1
Adprhl2	2E-02	0.1	Tmem263	1E-06	0.1
Pgam5	3E-01	0.1	Map4	3E-09	0.1
Tbx4	3E-01	0.1	Tmub1	2E-02	0.1
Caprin2	3E-01	0.1	Dnajc2	5E-06	0.1
Msantd3	5E-03	0.1	Pxk	1E-05	0.1
Rpl8	2E-24	0.1	Mrps26	4E-06	0.1
Uqcrc1	5E-04	0.1	Cpne3	9E-05	0.1
Dnaja1	3E-09	0.1	Myo9a	2E-06	0.1
Ess2	2E-01	0.1	Srp72	4E-07	0.1
Klhl7	4E-01	0.1	Srp68	8E-05	0.1
Snf8	5E-05	0.1	Galnt4	4E-02	0.1
Tnfrsf1a	5E-03	0.1	Adrm1	3E-07	0.1
Mospd2	4E-01	0.1	Llgl1	8E-03	0.1
Ajuba	4E-02	0.1	Eif4a3	2E-06	0.1
Zfp422	1E-01	0.1	Ccdc186	7E-06	0.1
Mrps7	1E-03	0.1	Gng12	2E-08	0.1
Stk25	4E-03	0.1	Slc4a2	6E-05	0.1
Eif6	3E-02	0.1	Asph	3E-06	0.1
N4bp2	3E-02	0.1	Hmox2	2E-05	0.1
Marcksl1	2E-02	0.1	Sdhaf2	3E-03	0.1
Rab7	1E-06	0.1	Plaa	7E-06	0.1
BC051226	3E-01	0.1	Rplp2	3E-19	0.1
Uba6	3E-03	0.1	Rragc	4E-07	0.1
Gm11808	2E-03	0.1	Kdelr2	2E-17	0.1
Nelfa	1E-01	0.1	Atg9a	2E-01	0.1
Ube2w	2E-05	0.1	Zfp248	8E-02	0.1
Clns1a	6E-02	0.1	Ttc28	1E-08	0.1
Thop1	9E-02	0.1	Ino80b	2E-05	0.1
Polr2i	1E-05	0.1	Vmac	2E-01	0.1
Kif1c	1E-01	0.1	Dtx2	5E-04	0.1
Cystm1	2E-01	0.1	Ddx56	6E-03	0.1
Idh3b	2E-06	0.1	Apip	1E-04	0.1
1110065P20Rik	6E-05	0.1	Zfp410	9E-03	0.1
Pak2	7E-04	0.1	Surf1	2E-05	0.1
Vps36	1E-03	0.1	Ro60	5E-05	0.1

Chondrogenic					
Control			$Notch2^{tm1.1Ecan}$		
Gene	*p*	FC	Gene	*p*	FC
Cxcl14	7E-03	0.1	Gtf2h3	6E-01	0.1
Utp20	7E-02	0.1	Rae1	2E-02	0.1
U2af1	2E-06	0.1	Wdr3	2E-01	0.1
Cep19	3E-02	0.1	Tmem42	2E-02	0.1
Tbc1d23	9E-04	0.1	Nudt5	5E-02	0.1
Arap2	1E-01	0.1	Jkamp	9E-03	0.1
Atp6v1f	3E-10	0.1	Trmt1	4E-02	0.1
Mrpl23	7E-08	0.1	Etv3	5E-01	0.1
Lnpep	7E-03	0.1	Stard3	3E-01	0.1
Ptpa	4E-02	0.1	Cyb5d2	2E-01	0.1
Dcun1d2	4E-03	0.1	Tmem175	1E-02	0.1
Ate1	4E-01	0.1	Amfr	1E-03	0.1
Coq10a	1E-01	0.1	Mta1	3E-02	0.1
Rhobtb2	7E-01	0.1	Zfp365	3E-01	0.1
Mettl15	3E-01	0.1	Creg1	6E-07	0.1
Dtwd1	5E-02	0.1	Cdk7	2E-05	0.1
Pigyl	7E-04	0.1	Ap5s1	1E+00	0.1
Grpel1	8E-06	0.1	Fubp1	3E-08	0.1
Firre	2E-01	0.1	Rprd1b	3E-01	0.1
Fiz1	4E-01	0.1	Cluh	3E-01	0.1
Crebzf	2E-02	0.1	Sf1	7E-03	0.1
Efnb2	3E-04	0.1	Brd2	3E-04	0.1
Setd3	1E-03	0.1	Pcif1	3E-02	0.1
Dhx34	9E-01	0.1	Ptdss1	4E-01	0.1
Trim24	4E-03	0.1	Yipf5	1E-04	0.1
Tomm40	4E-03	0.1	Mosmo	3E-03	0.1
A730081D07Rik	1E-01	0.1	Ubac2	7E-01	0.1
0610030E20Rik	7E-01	0.1	Heatr3	2E-01	0.1
Rint1	6E-01	0.1	Zfp654	1E-02	0.1
Ssbp2	8E-06	0.1	Id3	2E-05	0.1
Ubfd1	5E-03	0.1	Fancc	7E-01	0.1
Nudt6	3E-01	0.1	Fgfr1op2	1E-07	0.1
Insr	3E-01	0.1	Tbca	6E-10	0.1
Gm26532	5E-02	0.1	Exosc5	7E-03	0.1
Psmc2	3E-05	0.1	Coprs	1E-02	0.1
Zfp867	5E-01	0.1	Adal	8E-01	0.1
Cops3	1E-02	0.1	Zfp672	6E-02	0.1
Slc35b2	1E-04	0.1	Fkbp3	3E-07	0.1
Smpd2	6E-01	0.1	4833420G17Rik	2E-01	0.1
Dnal1	9E-01	0.1	Tra2a	2E-04	0.1
Acbd3	7E-07	0.1	3110040N11Rik	3E-04	0.1
Golgb1	4E-10	0.1	Mtfr1l	2E-02	0.1
Mbd3	6E-08	0.1	Pnn	7E-04	0.1
Immp1l	1E-04	0.1	Rbfox1	3E-01	0.1
Pdia3	5E-14	0.1	Mthfd1	2E-01	0.1
Rbm15	3E-01	0.1	Bsdc1	5E-01	0.1
Gphn	7E-04	0.1	Cdk5rap1	9E-01	0.1
Fam133b	4E-02	0.1	Mtif3	4E-01	0.1
Pon2	1E-02	0.1	Mrps22	7E-01	0.1
Rnf187	2E-04	0.1	Cfap298	2E-01	0.1
Abt1	1E-01	0.1	Sirt5	4E-01	0.1
Lipo3	9E-02	0.1	Abhd17c	2E-02	0.1
Vapa	2E-08	0.1	Zwint	9E-02	0.1
Ip6k1	2E-02	0.1	Itpr1	2E-02	0.1
Stxbp5	6E-04	0.1	Pdcd2	3E-01	0.1
Atg2b	9E-01	0.1	E2f5	7E-02	0.1
Nog	4E-03	0.1	Ubn2	5E-04	0.1
Uqcr10	1E-13	0.1	Ppp2r3a	1E-02	0.1
Trp53rka	8E-01	0.1	Acer3	2E-02	0.1
Snrnp48	4E-02	0.1	Abcb1b	5E-01	0.1
Dgkd	4E-02	0.1	Cnbd2	3E-01	0.1
Cd59a	6E-02	0.1	Cox7c	2E-20	0.1
Snrpf	5E-09	0.1	Mto1	3E-01	0.1
Lrrc8a	3E-03	0.1	Upf2	8E-02	0.1

Fibroblast					
Control			$Notch2^{tm1.1Ecan}$		
Gene	*p*	FC	Gene	*p*	FC
Daam1	4E-04	0.2	Snx25	2E-04	0.2
Hdgfl2	1E-03	0.2	Wdr44	1E-03	0.2
Atg4a	3E-03	0.2	Cdkn2aipnl	4E-03	0.2
Prpf18	5E-02	0.2	Mrs2	5E-03	0.2
Atxn3	5E-02	0.2	Pacs1	1E-03	0.2
Leo1	9E-02	0.2	Kansl1l	1E-05	0.2
Piezo2	4E-05	0.2	Znrf2	5E-02	0.2
Ppp1r7	4E-04	0.2	Nt5dc3	6E-03	0.2
Phf6	4E-03	0.2	Nphp1	2E-02	0.2
Mtrex	5E-05	0.2	Trp53bp2	4E-02	0.2
Prr3	1E-02	0.2	Myo5a	5E-09	0.2
Slc9a1	2E-01	0.2	Vps4a	6E-03	0.2
Psmd3	2E-03	0.2	Cep126	2E-01	0.2
Arl5b	3E-02	0.2	Rnf123	2E-02	0.2
Becn1	8E-03	0.2	Psmc3	9E-07	0.2
Pex11a	3E-01	0.2	Zfand2a	3E-01	0.2
Fam162a	1E-06	0.2	Adam9	1E-04	0.2
Tor1b	3E-02	0.2	Usp47	3E-08	0.2
Lamb2	8E-06	0.2	Rhobtb1	1E-01	0.2
Lin52	1E-03	0.2	Med24	5E-03	0.2
Emc3	2E-05	0.2	Pex7	2E-02	0.2
Dzip1l	1E-02	0.2	Gm17106	3E-01	0.2
Scaf11	1E-07	0.2	Cers4	1E-02	0.2
Oaz1	1E-17	0.2	Nono	1E-08	0.2
Hnrnpu	3E-10	0.2	Tpr	7E-14	0.2
Psmg2	8E-03	0.2	Dr1	1E-01	0.2
Rtf1	1E-05	0.2	Limch1	1E-05	0.2
Taf6	1E-01	0.2	Ccar1	4E-09	0.2
Gpr137	7E-02	0.2	Trim24	3E-04	0.2
Cluap1	5E-02	0.2	Mecom	2E-02	0.2
Ppp4r3a	7E-04	0.2	Pdzrn3	2E-09	0.2
Rbbp4	2E-06	0.2	Nup43	4E-03	0.2
Sdc4	2E-09	0.2	B130055M24Rik	3E-03	0.2
Gnai3	1E-05	0.2	Plekhf1	9E-03	0.2
Slc35f6	3E-01	0.2	Atp1b3	3E-09	0.2
Tm2d1	8E-05	0.2	Ddx31	2E-02	0.2
Camkmt	3E-02	0.2	Rapgef1	2E-04	0.2
Tm2d2	2E-06	0.2	Arhgef17	2E-06	0.2
Cbx1	4E-08	0.2	Mical2	2E-04	0.2
Ssb	7E-09	0.2	Mxi1	1E-03	0.2
Naa20	3E-04	0.2	Abcd1	7E-03	0.2
Ppp1ca	3E-09	0.2	Terf2	5E-05	0.2
Pknox1	2E-02	0.2	Lnpk	1E-02	0.2
Hoxc9	3E-02	0.2	Agk	3E-02	0.2
Polr2c	4E-03	0.2	Zfyve19	1E-03	0.2
Rgs12	1E-02	0.2	Pcm1	3E-08	0.2
Mrto4	1E-02	0.2	Itfg1	4E-05	0.2
Ap1b1	4E-03	0.2	Mkln1	1E-07	0.2
Eif1ax	4E-07	0.2	Rab30	1E-03	0.2
Hmgn1	3E-13	0.2	Bcl2l13	6E-02	0.2
Rtn4	6E-12	0.2	Tbl3	3E-03	0.2
Fbxo34	2E-01	0.2	Trak2	1E-03	0.2
Nkap	5E-04	0.2	Cry1	8E-03	0.2
Mettl7a1	1E-03	0.2	Babam1	2E-04	0.2
Supt7l	2E-01	0.2	Gtpbp8	8E-05	0.2
Usp14	1E-03	0.2	Ppp2r2d	8E-05	0.2
Wdsub1	9E-02	0.2	Atg12	2E-02	0.2
Timm22	4E-02	0.2	Sf3b1	1E-11	0.2
Psmd14	9E-05	0.2	Wbp1	8E-06	0.2
Hif1a	8E-12	0.2	Map4k5	6E-06	0.2
Mtor	5E-03	0.2	Mast4	3E-06	0.2
Atpif1	2E-11	0.2	Pign	9E-05	0.2
Gpatch1	2E-02	0.2	Wdr45b	1E-05	0.2
Stk16	4E-03	0.2	Sf3b4	1E-04	0.2

Undefined					
Control			$Notch2^{tm1.1Ecan}$		
Gene	*p*	FC	Gene	*p*	FC

Articular/Synovial Fibroblast					
Control			$Notch2^{tm1.1Ecan}$		
Gene	*p*	FC	Gene	*p*	FC
Ormdl2	4E-03	0.2	Map2k6	4E-01	0.2
Chac2	2E-01	0.2	Agk	1E-01	0.2
Zfp729b	2E-01	0.2	Chka	2E-02	0.2
Sp1	5E-02	0.2	Hexim1	1E-01	0.2
Kif21a	5E-03	0.2	Atg14	2E-01	0.2
Ccdc71l	4E-01	0.2	Rapgef2	5E-04	0.2
Tmem127	2E-02	0.2	Ssh1	3E-02	0.2
Slc6a6	1E-07	0.2	Gtf3c4	5E-01	0.2
Ccdc28b	1E-01	0.2	Tspan4	1E-04	0.2
Mon1a	2E-01	0.2	Slc35b3	1E-01	0.2
Rab10os	2E-01	0.2	Tnip1	6E-01	0.2
Pld1	1E-01	0.2	Vps16	5E-01	0.2
Cuta	2E-06	0.2	Cln6	8E-01	0.2
Rab8b	3E-03	0.2	Ino80d	2E-02	0.2
Ptdss2	3E-01	0.2	Rnaseh2a	5E-01	0.2
Ubxn2b	2E-01	0.2	Rad17	5E-01	0.2
Utrn	3E-08	0.2	Mettl21a	3E-02	0.2
Zranb1	9E-04	0.2	Snrnp200	6E-03	0.2
Spopl	7E-03	0.2	Dyrk2	3E-01	0.2
Atpif1	1E-11	0.2	Nsg1	2E-05	0.2
Pot1a	2E-01	0.2	Dctn1	3E-02	0.2
Dop1b	3E-02	0.2	Msra	4E-02	0.2
Slc10a7	2E-02	0.2	Wdfy3	8E-04	0.2
Tbcd	4E-03	0.2	Rnf14	3E-03	0.2
Rnf185	9E-03	0.2	Med24	6E-01	0.2
Ammecr1l	1E-02	0.2	Ccdc6	1E-02	0.2
Zbtb17	7E-01	0.2	Cox7b	3E-08	0.2
Git1	6E-02	0.2	Smg6	4E-04	0.2
Vps28	2E-03	0.2	Clec16a	4E-02	0.2
Chp1	3E-05	0.2	Piezo1	9E-03	0.2
Bmt2	5E-05	0.2	Vegfa	2E-01	0.2
Ube2v1	2E-03	0.2	Pip4k2c	4E-02	0.2
Ulk2	6E-05	0.2	Sfxn5	2E-02	0.2
Txnrd2	6E-01	0.2	Gm15952	1E-01	0.2
Ginm1	7E-04	0.2	Sergef	6E-03	0.2
Usp25	2E-04	0.2	Arl8b	6E-04	0.2
Rbl2	4E-02	0.2	Trub2	3E-01	0.2
Ints1	3E-01	0.2	Tbc1d20	3E-05	0.2
Irf2bpl	1E-02	0.2	Zbtb1	3E-02	0.2
Rnf2	7E-02	0.2	Mdfic	2E-02	0.2
Sox4	5E-04	0.2	Cog5	3E-04	0.2
Dlst	6E-03	0.2	Atp2c1	7E-02	0.2
Hmbox1	1E-02	0.2	Hace1	5E-01	0.2
AW549877	3E-02	0.2	Tpra1	9E-02	0.2
St6galnac4	1E-01	0.2	Coq3	9E-02	0.2
Gpatch1	2E-01	0.2	Gm20732	6E-01	0.2
Azi2	1E-02	0.2	Pld2	6E-01	0.2
Hdgfl3	3E-04	0.2	Gabpa	5E-01	0.2
Cenpx	1E-03	0.2	Cd2bp2	3E-02	0.2
Szt2	3E-01	0.2	Glyr1	3E-03	0.2
Brip1os	2E-03	0.2	Dnajc14	8E-02	0.2
Zfand1	1E-01	0.2	Sod3	7E-05	0.2
Epb41l1	3E-01	0.2	Smarcc2	1E-02	0.2
Pym1	2E-01	0.2	Lrrc1	6E-01	0.2
Asb8	4E-02	0.2	D330023K18Rik	2E-01	0.2
Zfp790	4E-01	0.2	Mapk8	3E-01	0.2
Pced1a	1E-01	0.2	Atp9b	1E-02	0.2
Eid2b	8E-01	0.2	Abhd4	2E-01	0.2
Irf2	2E-04	0.2	Ak1	4E-02	0.2
Zfp207	4E-05	0.2	Pex1	7E-01	0.2
Slc9a9	1E-05	0.2	Exoc7	2E-02	0.2
Chmp1b	3E-01	0.2	Strn	2E-02	0.2
Alg10b	1E-01	0.2	Tatdn3	1E-01	0.2
Prrg2	2E-01	0.2	Ube2q2	3E-01	0.2

Limb Mesenchyme

Control Gene	p	FC	*Notch2*$^{tm1.1Ecan}$ Gene	p	FC
Nfkb1	2E-02	0.1	Rchy1	4E-04	0.1
Sod3	2E-02	0.1	Mb21d2	1E-01	0.1
Nabp1	2E-02	0.1	Alms1	6E-02	0.1
Washc4	3E-04	0.1	Sec13	4E-12	0.1
Akt1s1	2E-04	0.1	Uba52	7E-08	0.1
Dexi	2E-02	0.1	A430005L14Rik	1E-02	0.1
Fam53b	1E-01	0.1	Ipo5	9E-04	0.1
Mtfl	6E-01	0.1	Ddx39	1E-07	0.1
Ttc9c	3E-01	0.1	Snx3	9E-09	0.1
Rdx	1E-09	0.1	Platr25	4E-02	0.1
Tbccd1	3E-01	0.1	Samm50	8E-06	0.1
Tor3a	2E-03	0.1	Ggh	6E-06	0.1
Mrps23	3E-01	0.1	Tollip	2E-03	0.1
Snhg8	1E-07	0.1	Ndufa10	9E-10	0.1
Naca	4E-23	0.1	Alg10b	5E-03	0.1
Ap3b1	5E-05	0.1	Lix1l	2E-07	0.1
Mri1	2E-02	0.1	Mbd3	4E-06	0.1
Cox5b	4E-13	0.1	Cggbp1	1E-05	0.1
Pigg	9E-01	0.1	Ttll11	4E-03	0.1
Meis1	2E-02	0.1	Sirt2	3E-08	0.1
Atp5c1	1E-10	0.1	Akr1b3	5E-06	0.1
Tcf3	3E-03	0.1	Lynx1	2E-03	0.1
Myc	3E-01	0.1	Ier3ip1	4E-09	0.1
Tcta	4E-01	0.1	Sod3	3E-04	0.1
Zfp30	3E-01	0.1	Hbp1	2E-04	0.1
Eapp	4E-04	0.1	Dera	4E-04	0.1
Pdk3	4E-02	0.1	Rrp8	3E-04	0.1
Borcs5	2E-01	0.1	Grb10	5E-08	0.1
Hsbp1	4E-08	0.1	Ftl1	6E-23	0.1
Nmi	3E-01	0.1	Slc39a7	2E-08	0.1
Fam199x	7E-01	0.1	Nop53	4E-04	0.1
Snx3	4E-08	0.1	Ankrd9	1E-02	0.1
Nynrin	2E-02	0.1	Zmat5	2E-05	0.1
Ppp1r15a	7E-06	0.1	Exosc4	5E-05	0.1
Isg20l2	2E-01	0.1	Endog	1E-04	0.1
2410002F23Rik	6E-02	0.1	Snx5	8E-06	0.1
Rab43	2E-02	0.1	Rpl15	4E-19	0.1
Rfng	4E-01	0.1	Gtpbp3	1E-02	0.1
Cyp26b1	3E-04	0.1			
Ncstn	2E-03	0.1			
Tmem263	8E-05	0.1			
Ints3	1E-01	0.1			
Shmt2	2E-02	0.1			
Coa3	3E-05	0.1			
Igbp1	1E-03	0.1			
Nol12	8E-03	0.1			
Trappc1	4E-03	0.1			
Rnf139	2E-01	0.1			
Klhdc1	1E+00	0.1			
Tnc	4E-01	0.1			
Ubtd2	5E-04	0.1			
Dock1	2E-03	0.1			
Aar2	8E-01	0.1			
Dnase1l1	4E-01	0.1			
Dus3l	1E-01	0.1			
Dynlrb1	9E-09	0.1			
Zfp277	3E-02	0.1			
Glrx2	4E-05	0.1			

Chondrogenic

Control Gene	p	FC	*Notch2*$^{tm1.1Ecan}$ Gene	p	FC
Rpl26	5E-34	0.1	Pole4	6E-02	0.1
Mettl16	3E-02	0.1	Pin4	1E-03	0.1
Rnf215	1E-01	0.1	5730522E02Rik	3E-01	0.1
Rmnd1	3E-01	0.1	Cirbp	3E-02	0.1
Tmem63b	5E-01	0.1	Dhdds	4E-01	0.1
Usp20	7E-01	0.1	Neurl4	4E-01	0.1
2010001A14Rik	5E-01	0.1	Ppcdc	1E-01	0.1
Pigx	7E-03	0.1	Ubp1	4E-02	0.1
Seh1l	7E-03	0.1	Gadd45gip1	1E-05	0.1
Slc31a1	2E-02	0.1	Tipin	3E-03	0.1
Ttc9c	2E-01	0.1	Tma16	2E-02	0.1
Zfp866	2E-01	0.1	Atxn7l3b	1E-06	0.1
Eif3g	2E-05	0.1	Crlf2	1E-01	0.1
Rpn1	2E-06	0.1	Setd5	1E-04	0.1
Utp18	1E-02	0.1	Zkscan3	2E-02	0.1
Bphl	5E-03	0.1	Cetn2	3E-03	0.1
Aup1	2E-02	0.1	Mob2	1E-02	0.1
Gm45669	5E-01	0.1	Ubxn6	6E-03	0.1
Rpl35a	1E-31	0.1	Camk2d	2E-06	0.1
Coil	2E-01	0.1	Dapk3	1E-01	0.1
Pigp	1E-07	0.1	Riox2	9E-02	0.1
Pnn	4E-03	0.1	Cpeb1	2E-01	0.1
Stt3b	7E-05	0.1	1110051M20Rik	3E-02	0.1
Gm15867	4E-03	0.1	Usp13	1E+00	0.1
Matr3	4E-04	0.1	Slc25a17	1E-02	0.1
Setdb1	3E-02	0.1	Skp1a	2E-10	0.1
Arid1b	3E-03	0.1	Med7	4E-03	0.1
Rangrf	1E-02	0.1	Usp12	7E-02	0.1
Ythdc2	7E-01	0.1	Bax	1E-07	0.1
Nufip1	6E-02	0.1	Iqcb1	2E-02	0.1
Polr3k	1E-04	0.1	Mab21l2	9E-03	0.1
Scarf2	2E-02	0.1	Erp44	6E-06	0.1
Trmt112	2E-06	0.1	Nucb1	1E-04	0.1
Pinx1	2E-02	0.1	Daglb	4E-04	0.1
Ppp2r5e	1E-02	0.1	Zfp639	7E-03	0.1
Klhl21	1E-01	0.1	Ube2v2	9E-05	0.1
Atp5g1	3E-06	0.1	Zfp933	2E-01	0.1
Acadsb	4E-04	0.1	Dot1l	4E-01	0.1
Creb1	1E-02	0.1	Top1mt	6E-01	0.1
Yipf4	7E-07	0.1	Wdr77	2E-02	0.1
mt-Nd5	6E-08	0.1	Exoc4	3E-07	0.1
Psma7	2E-06	0.1	Cuedc1	2E-02	0.1
Dstyk	9E-02	0.1	Stt3b	1E-02	0.1
Wrnip1	2E-01	0.1	Sdf2	2E-05	0.1
Bcat2	4E-02	0.1	Pfdn2	4E-06	0.1
Dnaja4	2E-01	0.1	Impact	5E-03	0.1
Bbc3	3E-01	0.1	Rps7	3E-19	0.1
Rnaseh2a	5E-01	0.1	Nr3c2	2E-01	0.1
Zfp445	1E-02	0.1	Ptp4a3	9E-02	0.1
Ttc1	1E-02	0.1			
Las1l	4E-02	0.1			
Tent5a	6E-09	0.1			
Wbp11	8E-05	0.1			
Msl3l2	3E-01	0.1			
Romo1	1E-14	0.1			
Stox2	1E-01	0.1			
Tet2	8E-01	0.1			
Mrpl32	4E-03	0.1			
Wdr83os	1E-08	0.1			
Tmsb15b2	2E-02	0.1			
Mesd	3E-05	0.1			
Qtrt2	2E-01	0.1			
Acot2	1E-07	0.1			
Bcar3	6E-03	0.1			

Fibroblast

Control Gene	p	FC	*Notch2*$^{tm1.1Ecan}$ Gene	p	FC
Zswim1	6E-01	0.2	Npc1	3E-02	0.2
D8Ertd738e	2E-10	0.2	Isca1	2E-02	0.2
Mrpl46	2E-02	0.2	2810001G20Rik	3E-01	0.2
Ep400	3E-03	0.2	Cyfip1	2E-08	0.2
Nek9	2E-02	0.2	Dancr	1E-02	0.2
Snx6	3E-06	0.2	Cmtm6	2E-01	0.2
Dcaf11	3E-01	0.2	Rev3l	9E-06	0.2
Zfp384	4E-02	0.2	Pde7a	6E-04	0.2
Eef1akmt2	6E-02	0.2	Arid2	4E-04	0.2
Tomm70a	9E-03	0.2	Lrrc17	1E-03	0.2
Plcl1	1E-04	0.2	Cmip	8E-05	0.2
Srrm1	1E-07	0.2	Kdm5c	2E-03	0.2
Stat1	3E-03	0.2	Bcr	2E-02	0.2
Nhej1	1E-01	0.2	Cnpy4	2E-05	0.2
Exoc3	4E-05	0.2	Atp9b	3E-06	0.2
Pex1	5E-01	0.2	Afdn	8E-05	0.2
Eif2s3x	6E-03	0.2	Tmem98	3E-05	0.2
Snapc1	5E-02	0.2	Psmf1	6E-04	0.2
Txn1	2E-06	0.2	Trpm4	5E-02	0.2
Large1	3E-08	0.2	Pcgf5	3E-02	0.2
Vill	4E-01	0.2	Dipk1b	6E-03	0.2
Agk	6E-02	0.2	Rprd1a	6E-03	0.2
Cebpzos	1E-04	0.2	Smarcd1	2E-05	0.2
Tmem30a	4E-05	0.2	Bri3bp	2E-04	0.2
Klf3	2E-03	0.2	Greb1l	1E-02	0.2
Arl2	2E-04	0.2	Bag3	2E-03	0.2
Clptm1	8E-03	0.2	Cuedc2	8E-08	0.2
Fam133b	1E-03	0.2	Cltb	5E-07	0.2
Pdia5	4E-03	0.2	Trim12c	6E-02	0.2
Hgsnat	7E-03	0.2	Tubgcp5	1E-03	0.2
Parg	2E-03	0.2	Tollip	6E-02	0.2
Gatc	2E-01	0.2	Ep400	2E-05	0.2
Dmxl1	5E-03	0.2	Smyd3	2E-05	0.2
Anapc11	1E-04	0.2	Parg	2E-04	0.2
Rev1	5E-03	0.2	Sec14l1	2E-06	0.2
Abhd18	1E-01	0.2	Bsdc1	3E-01	0.2
Tecpr1	3E-01	0.2	Zfp518b	1E-02	0.2
Ell	2E-02	0.2	Mpv17l2	2E-02	0.2
Cdkn2b	1E-04	0.2	Pcyox1l	5E-03	0.2
Zdhhc12	2E-02	0.2	Snx6	9E-10	0.2
Eif4a3	1E-05	0.2	Tead3	2E-02	0.2
Rxra	6E-02	0.2	Cyb5r4	3E-04	0.2
Il6st	3E-10	0.2	Gpatch2l	5E-02	0.2
Tcp11l1	8E-02	0.2	Cbx3	4E-11	0.2
Sprtn	2E-01	0.2	P4ha3	4E-03	0.2
Mbip	2E-01	0.2	Phtf1	3E-05	0.2
Ostm1	5E-02	0.2	Ccl27a	5E-02	0.2
Irf3	7E-01	0.2	Pkp4	2E-04	0.2
Secisbp2l	3E-03	0.2	Man1c1	1E-08	0.2
Pakap.1	2E-06	0.2	C2cd5	4E-03	0.2
Smarce1	7E-05	0.2	Fam160b1	1E-01	0.2
Nf2	1E-02	0.2	Zfp521	1E-09	0.2
Hoxc6	5E-02	0.2	Pgap2	2E-04	0.2
Nup188	2E-03	0.2	Hmgxb4	2E-02	0.2
Mpg	2E-03	0.2	Ubtd2	1E-05	0.2
Tmod2	1E-01	0.2	Cavin1	9E-13	0.2
1110051M20Rik	8E-04	0.2	Lrp10	5E-04	0.2
Ak6	2E-02	0.2	Mcl1	4E-09	0.2
Zdhhc21	3E-02	0.2	Fas	1E-05	0.2
Nr2f6	6E-03	0.2	Tulp4	1E-06	0.2
Tes	3E-02	0.2	Rbm15	3E-04	0.2
Rcor1	1E-02	0.2	Mgat1	6E-06	0.2
G3bp1	3E-03	0.2	Supt20	9E-05	0.2
Shq1	4E-01	0.2	Ccnyl1	2E-03	0.2

Undefined

Control Gene	p	FC	*Notch2*$^{tm1.1Ecan}$ Gene	p	FC

Articular/Synovial Fibroblast

Control Gene	p	FC	*Notch2*$^{tm1.1Ecan}$ Gene	p	FC
Ubtd1	3E-02	0.2	Akap10	1E-01	0.2
Zfp28	5E-01	0.2	Gaa	4E-04	0.2
Nup153	3E-04	0.2	Ammecr1l	4E-02	0.2
Gramd3	1E-01	0.2	Cystm1	5E-02	0.2
Krit1	1E-03	0.2	Tshz2	1E-02	0.2
Dctn5	2E-01	0.2	Abracl	2E-06	0.2
Tceanc2	3E-02	0.2	Mfsd11	2E-01	0.2
Mtmr9	5E-02	0.2	Ccdc97	5E-01	0.2
Zc3h12c	6E-01	0.2	Afap1l2	3E-01	0.2
Nek6	3E-01	0.2	Rusc2	7E-02	0.2
Med15	4E-03	0.2	Hoxa7	8E-02	0.2
Ttf2	4E-01	0.2	Emc1	2E-02	0.2
Txndc17	1E-08	0.2	AI597479	7E-01	0.2
Dhx35	8E-01	0.2	1110019D14Rik	4E-02	0.2
Cnksr3	6E-02	0.2	Foxk2	2E-01	0.2
2210408I21Rik	2E-02	0.2	Megf8	2E-01	0.2
Specc1	2E-03	0.2	Uap1	9E-03	0.2
Rev3l	6E-04	0.2	Ly6e	8E-05	0.2
Xrn1	2E-04	0.2	Zdhhc4	8E-02	0.2
Hexb	9E-05	0.2	Tfip11	2E-01	0.2
Mar9	2E-01	0.2	Mindy2	3E-02	0.2
Eea1	6E-08	0.2	Efcab14	3E-02	0.2
Numbl	3E-02	0.2	Stambpl1	8E-02	0.2
Pms2	1E+00	0.2	Hook3	1E-03	0.2
Rfng	2E-01	0.2	Ints9	2E-01	0.2
Man2c1	2E-01	0.2	Zfp346	3E-01	0.2
Rap1a	2E-06	0.2	Bloc1s1	3E-01	0.2
Paxip1	2E-01	0.2	Camta2	5E-01	0.2
Ccdc68	3E-01	0.2	Gtf2h3	5E-01	0.2
Snx30	7E-02	0.2	Ap1s2	1E-01	0.2
Xxylt1	2E-01	0.2	Flad1	1E-01	0.2
Rragc	8E-06	0.2	Emp3	4E-09	0.2
Pdk2	1E-01	0.2	B3gnt9	5E-02	0.2
Mfsd14a	1E-02	0.2	Homer3	4E-02	0.2
Vps36	2E-05	0.2	Usp7	1E-01	0.2
Atp2c1	2E-03	0.2	Zfp84	8E-01	0.2
Gclc	7E-02	0.2	Gng10	2E-04	0.2
Btbd6	4E-01	0.2	Gm39469	3E-01	0.2
Arhgef7	3E-02	0.2	Dctn2	6E-04	0.2
Cers5	1E-04	0.2	Fkbpl	2E-01	0.2
Tmtc3	8E-02	0.2	Exoc4	6E-05	0.2
Dancr	9E-01	0.2	Ggps1	9E-02	0.2
Adcy6	2E-01	0.2	Ap2a1	7E-03	0.2
Mtrf1l	1E-01	0.2	Poglut2	1E-01	0.2
Calcoco1	6E-02	0.2	Lsm10	3E-02	0.2
Kirrel	1E-06	0.2	Fbxo4	7E-02	0.2
Il20rb	7E-01	0.2	Tax1bp3	1E-04	0.2
Telo2	6E-02	0.2	Slc11a2	2E-01	0.2
Cystm1	7E-02	0.2	Reps1	1E-01	0.2
Mtmr4	4E-01	0.2	Smap1	2E-06	0.2
Wrb	1E-01	0.2	Nav2	4E-04	0.2
Sec24b	9E-04	0.2	Relch	1E-03	0.2
Itgb5	7E-07	0.2	Nfib	2E-07	0.2
Zfhx3	1E-08	0.2	Thrb	6E-02	0.2
Gabpa	7E-03	0.2	Epg5	8E-02	0.2
2610044O15Rik8	5E-01	0.2	Arel1	5E-01	0.2
Anapc13	5E-07	0.2	Ankrd27	7E-03	0.2
Nf1	5E-05	0.2	Mrps2	4E-01	0.2
Stmn1	3E-19	0.2	Ccndbp1	4E-02	0.2
Rngtt	8E-02	0.2	Tmem132a	1E-02	0.2
Med23	1E-01	0.2	Cuedc2	8E-06	0.2
Cep192	5E-02	0.2	Ghr	1E-05	0.2
Galc	2E-01	0.2	Mlycd	9E-01	0.2
Rufy3	3E-03	0.2	Mpp5	4E-02	0.2

Limb Mesenchyme						Chondrogenic						Fibroblast						Undefined						Articular/Synovial Fibroblast					
Control			*$Notch2^{tm1.1Ecan}$*			Control			*$Notch2^{tm1.1Ecan}$*			Control			*$Notch2^{tm1.1Ecan}$*			Control			*$Notch2^{tm1.1Ecan}$*			Control			*$Notch2^{tm1.1Ecan}$*		
Gene	*p*	FC	Gene	*p*	FC	Gene	*p*	FC	Gene	*p*	FC	Gene	*p*	FC	Gene	*p*	FC	Gene	*p*	FC	Gene	*p*	FC	Gene	*p*	FC	Gene	*p*	FC
						Tbc1d22b	8E-01	0.1				Clic4	3E-08	0.2	Lrfn4	7E-03	0.2							Brd9	6E-03	0.2	Fam129b	5E-02	0.2
						Nbdy	6E-06	0.1				Trmt2a	1E-01	0.2	Ddhd1	4E-04	0.2							Slc39a3	3E-01	0.2	Hmgn3	2E-06	0.2
						Ylpm1	7E-02	0.1				Thtpa	3E-01	0.2	Alad	2E-02	0.2							Bloc1s1	7E-01	0.2	Nfkbib	3E-01	0.2
						Ankra2	2E-03	0.1				Ttc8	5E-02	0.2	Crppa	1E-02	0.2							5730480H06Rik	1E+00	0.2	4930402H24Rik	5E-02	0.2
						Trit1	3E-01	0.1				Zfp617	7E-02	0.2	Zc3h13	7E-06	0.2							Traf3ip2	2E-01	0.2	Mthfd2l	9E-02	0.2
						Naa38	1E-06	0.1				Zfp809	6E-02	0.2	Ntn1	2E-03	0.2							Usp53	1E-01	0.2	Macrod2	2E-02	0.2
						Zbtb24	9E-01	0.1				Tmed1	4E-02	0.2	Camsap1	3E-05	0.2							Trp53inp1	1E-03	0.2	Fbrsl1	2E-01	0.2
						Pmpca	1E-01	0.1				Fbxo45	3E-02	0.2	Tpi1	2E-12	0.2							Cnpy4	1E-01	0.2	Wwc2	8E-03	0.2
						Smim13	2E-03	0.1				Anxa1	5E-14	0.2	Smarcc1	1E-07	0.2							Heatr1	7E-01	0.2	Nfkb2	3E-01	0.2
						Uros	3E-01	0.1				Pomgnt1	1E-02	0.2	Capn7	2E-02	0.2							Paics	1E-05	0.2	Dpy19l1	2E-02	0.2
						Prpf39	6E-01	0.1				Plekha1	5E-02	0.2	Nudt2	6E-04	0.2							Pycard	5E-04	0.2	Zfand2b	3E-02	0.2
						Prkag1	5E-03	0.1				Dele1	4E-02	0.2	Atg101	1E-03	0.2							Rnaseh2a	1E-01	0.2	Scamp1	8E-01	0.2
						Mrpl17	1E-04	0.1				Bri3bp	2E-02	0.2	Tiam1	6E-02	0.2							Atl3	3E-04	0.2	Ube2h	2E-04	0.2
						Ubn2	1E-05	0.1				Prkar2a	1E-02	0.2	Ier5	2E-05	0.2							Dnal1	3E-02	0.2	Ube2w	1E-03	0.2
						Atxn2l	2E-02	0.1				Plxna2	2E-03	0.2	Pmpca	1E-01	0.2							Bcl2l12	1E-01	0.2	Cep19	3E-01	0.2
						Arid4b	2E-03	0.1				Cuedc2	7E-06	0.2	Tmem243	1E-03	0.2							Fbxl14	1E-01	0.2	Rnf169	3E-01	0.2
						Gid8	6E-02	0.1				Mrpl49	1E-01	0.2	Stk38	4E-03	0.2							Erbin	1E-07	0.2	2510039O18Rik	7E-01	0.2
						Zfp952	1E-01	0.1				Plekhf2	1E-01	0.2	Mcm3ap	3E-03	0.2							Apobec1	5E-02	0.2	Dtnb	6E-02	0.2
						Rsrc1	2E-05	0.1				Zfp362	4E-02	0.2	Mvd	2E-02	0.2							Tmem205	2E-03	0.2	Akap1	2E-01	0.2
						D930016D06Rik	3E-01	0.1				Tnk2	7E-02	0.2	Csrp1	6E-06	0.2							Imp4	4E-01	0.2	Mlx	2E-01	0.2
						Nt5m	1E-01	0.1				Peli1	1E-02	0.2	Habp4	1E-03	0.2							Rabif	2E-02	0.2	Mb21d2	3E-01	0.2
						Ccnt2	3E-01	0.1				Gkap1	2E-02	0.2	Homer3	1E-04	0.2							9430038I01Rik	1E-01	0.2	Isca2	3E-01	0.2
						Erf	7E-02	0.1				Trp53	2E-03	0.2	Timp1	1E-05	0.2							Zfp606	4E-01	0.2	Vrk2	3E-02	0.2
						Aebp2	2E-01	0.1				Pdia4	3E-04	0.2	Vps4b	2E-05	0.2							Simc1	1E-01	0.2	Kansl1	4E-05	0.2
						Asxl2	4E-02	0.1				Mdfic	7E-03	0.2	Snx15	9E-04	0.2							Tmem218	5E-03	0.2	Abhd17b	5E-02	0.2
						mt-Co3	1E-13	0.1				Slc25a14	2E-01	0.2	Wac	1E-06	0.2							Czib	2E-02	0.2	Wiz	2E-01	0.2
						Brwd1	2E-03	0.1				Sh2b1	3E-01	0.2	Cnn2	6E-08	0.2							C2cd5	6E-02	0.2	Znrf1	2E-02	0.2
						Rps17	9E-22	0.1				Fam222b	2E-03	0.2	Otud6b	3E-05	0.2							Fam207a	5E-01	0.2	Fcho2	2E-03	0.2
						Ubxn2a	4E-02	0.1				Dtwd1	1E-01	0.2	Gtf2f1	1E-04	0.2							Sap18	5E-04	0.2	Mme	6E-05	0.2
						Cdkn2aipnl	4E-01	0.1				Strip1	7E-02	0.2	Armh4	3E-10	0.2							Apeh	5E-01	0.2	Adk	3E-04	0.2
						AI597479	5E-02	0.1				Ahsa2	3E-02	0.2	Hspa14	2E-05	0.2							Zfp157	3E-01	0.2	Sec24c	2E-01	0.2
						Hfe	2E-02	0.1				Rhot2	1E-01	0.2	2310033P09Rik	2E-04	0.2							Ercc5	1E-01	0.2	Hnrnph2	3E-02	0.2
						Btf3l4	5E-02	0.1				Snx27	8E-03	0.2	Aimp2	2E-04	0.2							Fkbp15	6E-03	0.2	Adprhl2	3E-01	0.2
						Wtap	3E-02	0.1				Etfa	2E-04	0.2	Sdc2	1E-13	0.2							Mrps11	2E-02	0.2	Cln8	2E-01	0.2
						Magt1	1E-04	0.1				Odf2	9E-02	0.2	Cabin1	3E-03	0.2							Tbc1d12	1E-02	0.2	Ncstn	5E-02	0.2
						Rcc1l	3E-01	0.1				Ndufb8	7E-07	0.2	Atg10	6E-05	0.2							Mtss1	8E-02	0.2	Galnt7	2E-01	0.2
						Rpl7	4E-25	0.1				Tango2	8E-02	0.2	Rhbdf1	4E-02	0.2							Bin3	2E-01	0.2	Hoxc6	8E-03	0.2
						Prdm10	1E-01	0.1				Irak4	4E-01	0.2	Usp49	6E-01	0.2							Dpy19l4	2E-01	0.2	Stx3	3E-01	0.2
						Ranbp17	5E-02	0.1				Gpx1	3E-11	0.2	Baz2b	6E-06	0.2							Rab3ip	7E-01	0.2	Rab31	4E-03	0.2
						Prpf6	1E-02	0.1				Brd4	4E-04	0.2	Pard6g	9E-02	0.2							Pde4a	5E-01	0.2	Il17d	3E-02	0.2
						Zfp608	1E-02	0.1				E2f4	4E-01	0.2	Secisbp2l	2E-03	0.2							Slc30a7	3E-03	0.2	Otub1	1E-03	0.2
						Fam49b	7E-04	0.1				Rfng	3E-01	0.2	Reck	2E-06	0.2							Pls3	4E-04	0.2	Ppip5k1	1E+00	0.2
						Rplp1	1E-29	0.1				Sclt1	6E-02	0.2	Gemin8	8E-03	0.2							Zfp65	7E-01	0.2	Ubac2	9E-03	0.2
						Pdgfa	8E-03	0.1				Atxn1l	1E-01	0.2	Prkx	1E-02	0.2							Ybx3	5E-07	0.2	Ano10	9E-02	0.2
						Utp15	2E-01	0.1				Paox	1E-01	0.2	Tyw5	8E-03	0.2							Cwc25	2E-02	0.2	Trim3	2E-01	0.2
						Zcchc10	8E-01	0.1				Kdm4b	1E-01	0.2	Txnrd2	1E-02	0.2							Dvl2	5E-02	0.2	Kif13b	1E-03	0.2
						Alkbh2	2E-02	0.1				Senp5	3E-03	0.2	Tm2d2	2E-07	0.2							Dhrs1	2E-04	0.2	Polr3c	9E-02	0.2
						Zfp236	4E-01	0.1				Gsr	2E-02	0.2	Stub1	3E-07	0.2							Sft2d1	4E-01	0.2	Ifnar2	8E-04	0.2
						A630089N07Rik	3E-01	0.1				Gale	2E-01	0.2	Rrp1b	6E-03	0.2							Arfgap3	7E-04	0.2	1700025G04Rik	3E-02	0.2
						Gnb1l	7E-02	0.1				Pin1	4E-03	0.2	Btd	6E-05	0.2							Uqcc2	6E-12	0.2	Itsn2	1E-02	0.2
						Rpl34	2E-29	0.1				Tut1	5E-01	0.2	Ccdc88a	2E-06	0.2							Map2k3	2E-03	0.2	Map7d1	5E-04	0.2
						Def6	9E-02	0.1				Vma21	1E-03	0.2	Eea1	7E-07	0.2							Tfcp2	8E-02	0.2	Mrps11	9E-02	0.2
						Selenok	3E-11	0.1				Arhgef18	1E-04	0.2	Ankfy1	2E-03	0.2							Golga1	1E-02	0.2	1110051M20Rik	2E-03	0.2
						Tpk1	9E-03	0.1				Tcp1	1E-05	0.2	Dcaf5	2E-04	0.2							Ece2	5E-02	0.2	Fbxo31	9E-02	0.2
						N4bp2l2	2E-04	0.1				Ier5l	3E-02	0.2	Prpf31	1E-04	0.2							Akr1e1	1E-01	0.2	Zfp800	4E-01	0.2
						Zfp830	1E-01	0.1				Zfp286	4E-01	0.2	Pa2g4	5E-07	0.2							Pip4k2c	4E-02	0.2	Med15	4E-02	0.2
						Dynll2	7E-04	0.1				Ube2i	7E-10	0.2	Jade1	1E-03	0.2							Cdc42ep5	9E-04	0.2	Egln1	2E-03	0.2
						Srsf6	3E-05	0.1				Atl3	2E-05	0.2	Eif4h	7E-10	0.2							Agtpbp1	7E-02	0.2	Cab39l	2E-02	0.2
						Stxbp4	2E-01	0.1				Tmem17	2E-01	0.2	Eda2r	3E-02	0.2							Gtpbp6	3E-01	0.2	Tmem9b	1E-02	0.2
						Eif2b1	8E-02	0.1				Hnrnpa0	3E-07	0.2	Dclre1a	7E-02	0.2							Ajuba	5E-01	0.2	Dync1li2	3E-03	0.2
						Fam98c	1E-01	0.1				Sec16b	9E-03	0.2	Arhgap21	2E-05	0.2							Prkar1a	5E-06	0.2	Slc30a7	2E-01	0.2
						Zfp131	1E-02	0.1				Ints8	7E-02	0.2	Bmp2k	1E-04	0.2							Dtd1	8E-03	0.2	Cstf2t	1E-01	0.2
						Zdhhc18	7E-01	0.1				Asap2	3E-02	0.2	Zfp445	5E-03	0.2							Ptpn9	9E-04	0.2	Sh2b1	1E-01	0.2
						Sdf4	2E-08	0.1				Sord	4E-02	0.2	Dip2b	6E-06	0.2							Tmtc1	4E-02	0.2	Mtch1	4E-06	0.2

Limb Mesenchyme					
Control			*Notch2*$^{tm1.1Ecan}$		
Gene	*p*	FC	Gene	*p*	FC

Chondrogenic					
Control			*Notch2*$^{tm1.1Ecan}$		
Gene	*p*	FC	Gene	*p*	FC
Fhit	3E-04	0.1			
Mast4	2E-12	0.1			
Cthrc1	1E-05	0.1			
Med4	2E-01	0.1			
Hira	7E-01	0.1			
Hoxc8	2E-01	0.1			
Aste1	4E-01	0.1			
Ddx31	9E-02	0.1			
Rhoq	2E-03	0.1			
Mcrip2	3E-02	0.1			
Prkrip1	5E-02	0.1			
Epm2aip1	2E-01	0.1			
Fam160a2	5E-01	0.1			
Timm21	3E-02	0.1			
Tmem167b	8E-02	0.1			
Rpl8	5E-23	0.1			
Gpn3	4E-02	0.1			
Chd2	2E-03	0.1			
Dnajc8	1E-06	0.1			
Pabpn1	1E-03	0.1			
Tut4	4E-03	0.1			
Nudcd3	9E-04	0.1			
Fam216a	3E-02	0.1			
Dmtf1	6E-02	0.1			
Lsg1	9E-03	0.1			
Purb	9E-05	0.1			
Inip	8E-01	0.1			
Atxn7l1	8E-02	0.1			
Terf2ip	2E-01	0.1			
Sacm1l	2E-03	0.1			
Slc12a4	1E-02	0.1			
Slc35c2	8E-03	0.1			
Dolk	1E-02	0.1			
Rps23	1E-25	0.1			
Bcas2	3E-06	0.1			
R74862	4E-01	0.1			
Tars	8E-04	0.1			
Abcf1	1E-04	0.1			
Arhgef18	6E-01	0.1			
Ccdc30	9E-01	0.1			
E2f6	9E-01	0.1			
Pnkd	1E-04	0.1			
Kdm3b	2E-02	0.1			
Tmem128	2E-03	0.1			
Zfp777	7E-01	0.1			
Rfk	8E-02	0.1			
C1d	3E-06	0.1			
Suclg1	3E-05	0.1			
Rev1	2E-02	0.1			
Tra2a	3E-05	0.1			
Rbm39	2E-10	0.1			
Rab3a	4E-02	0.1			
Kctd13	2E-01	0.1			
Nfatc3	2E-02	0.1			
2310009A05Rik	7E-05	0.1			
Cdk7	5E-02	0.1			
Rpl23	1E-32	0.1			
Cfap298	1E-01	0.1			
Tent4a	8E-01	0.1			
Dglucy	4E-01	0.1			
Gtpbp1	8E-01	0.1			
Ikbip	2E-05	0.1			
Dcun1d5	4E-06	0.1			
Ippk	4E-01	0.1			

Fibroblast					
Control			*Notch2*$^{tm1.1Ecan}$		
Gene	*p*	FC	Gene	*p*	FC
Zfp248	6E-01	0.2	Tubgcp3	3E-03	0.2
Lig3	6E-02	0.2	B9d2	1E-02	0.2
Clcn7	3E-01	0.2	Mcmbp	5E-04	0.2
Ahsa1	8E-03	0.2	Mgat4b	2E-04	0.2
Rnh1	7E-07	0.2	Tia1	7E-03	0.2
Mr1	2E-01	0.2	Zfp827	2E-02	0.2
Brf1	6E-01	0.2	Tor1b	3E-04	0.2
Psmd12	4E-05	0.2	Syt1	2E-02	0.2
2610301B20Rik	2E-02	0.2	Tshz1	8E-05	0.2
Actg1	4E-06	0.2	Ado	9E-05	0.2
Nudt5	3E-02	0.2	Rab11fip5	2E-02	0.2
Crybg3	2E-03	0.2	Stag1	2E-06	0.2
Tax1bp1	2E-07	0.2	Alkbh8	4E-04	0.2
Ankrd49	9E-02	0.2	Polr3b	4E-03	0.2
Ap1s1	1E-03	0.2	Aggf1	3E-03	0.2
Stambp	3E-02	0.2	Arhgap17	5E-04	0.2
Pwp1	3E-02	0.2	Pdxk	1E-02	0.2
Serpinb6a	4E-13	0.2	Per1	4E-02	0.2
Stxbp5	8E-04	0.2	Ocrl	1E-02	0.2
Setd1b	4E-03	0.2	Eed	2E-03	0.2
Hyou1	3E-02	0.2	Fam192a	1E-01	0.2
Exosc7	1E-02	0.2	Galnt2	7E-05	0.2
Galnt10	5E-03	0.2	Wrnip1	6E-02	0.2
Eif1b	1E-03	0.2	Nfkb2	2E-02	0.2
Zswim8	9E-04	0.2	Ncoa5	4E-02	0.2
Nipa2	2E-03	0.2	Zmynd19	1E-02	0.2
Sh3bp4	9E-02	0.2	Sema3b	6E-04	0.2
Nagpa	2E-01	0.2	Ddb1	9E-07	0.2
Cbr2	2E-03	0.2	Slc29a3	1E-01	0.2
Adam15	2E-05	0.2	Snupn	1E-02	0.2
Emd	5E-03	0.2	Ubqln4	6E-03	0.2
5730455P16Rik	2E-02	0.2	Ranbp9	9E-04	0.2
Parn	1E-02	0.2	Mef2d	6E-04	0.2
Mir22hg	6E-04	0.2	Dennd1b	5E-03	0.2
Ift57	2E-02	0.2	Adsl	1E-02	0.2
Prr13	3E-04	0.2	Chm	3E-03	0.2
L2hgdh	2E-01	0.2	Stat6	8E-04	0.2
Ccar1	5E-04	0.2	Mapkbp1	7E-04	0.2
Sufu	2E-02	0.2	Mtpap	4E-02	0.2
Inpp4a	3E-01	0.2	Cdyl	6E-05	0.2
Mrps31	3E-02	0.2	Kirrel	1E-07	0.2
Greb1l	2E-01	0.2	Epc1	1E-05	0.2
Rbm15b	1E-01	0.2	Pter	2E-02	0.2
Naa15	5E-03	0.2	Nek8	8E-02	0.2
Spsb1	3E-02	0.2	Rpp25l	4E-04	0.2
Zfp37	7E-02	0.2	Bclaf1	6E-08	0.2
Grb2	6E-04	0.2	Prkab1	2E-01	0.2
Tgs1	7E-03	0.2	Firre	1E-01	0.2
Xndc1	9E-02	0.2	Aasdh	5E-01	0.2
Znhit1	1E-03	0.2	Nipsnap3b	8E-03	0.2
Ctcf	2E-03	0.2	Lig3	4E-02	0.2
Ap5s1	9E-02	0.2	Srf	1E-02	0.2
Heatr6	1E-03	0.2	Nans	2E-06	0.2
Tango6	8E-02	0.2	Agtrap	3E-03	0.2
Adipor1	3E-05	0.2	Usp38	1E-01	0.2
Tusc2	4E-02	0.2	E130307A14Rik	2E-03	0.2
Med6	1E-02	0.2	Tmem131l	5E-02	0.2
Fancl	1E-02	0.2	Mgp	9E-11	0.2
Eif2ak2	1E-01	0.2	Dhx38	3E-02	0.2
2610507B11Rik	6E-03	0.2	Asap3	2E-03	0.2
Wdyhv1	1E-01	0.2	Zfp36l2	2E-07	0.2
Cfl1	1E-11	0.2	Gkap1	3E-06	0.2
Tnpo3	1E-03	0.2	Arhgap10	7E-06	0.2
Tmem53	3E-02	0.2	Thap12	1E-07	0.2

Undefined					
Control			*Notch2*$^{tm1.1Ecan}$		
Gene	*p*	FC	Gene	*p*	FC

Articular/Synovial Fibroblast					
Control			*Notch2*$^{tm1.1Ecan}$		
Gene	*p*	FC	Gene	*p*	FC
Ccdc61	5E-01	0.2	Msto1	5E-01	0.2
Ttll5	4E-02	0.2	Galk1	6E-04	0.2
Wsb2	5E-05	0.2	Smad5	1E-01	0.2
Gpalpp1	2E-02	0.2	Fam71e1	4E-01	0.2
Mgrn1	1E-01	0.2	2610008E11Rik	5E-02	0.2
Rab11a	7E-06	0.2	Nkiras2	7E-01	0.2
Ifnar1	5E-02	0.2	Lztr1	2E-01	0.2
Lamtor3	3E-04	0.2	Cep112	6E-02	0.2
1110059G10Rik	1E-03	0.2	Zgpat	1E-02	0.2
Alas1	2E-02	0.2	Zcchc4	2E-01	0.2
Dis3l2	1E-03	0.2	Crebrf	1E-04	0.2
Armc10	1E-01	0.2	Itfg1	3E-04	0.2
Pik3c2a	5E-02	0.2	Tpp2	3E-05	0.2
Mfsd14b	4E-03	0.2	Map3k12	4E-02	0.2
Uba1	8E-05	0.2	2610044O15Rik8	2E-01	0.2
Sgms2	1E-01	0.2	Arfgap3	1E-03	0.2
Mlx	2E-01	0.2	E130308A19Rik	4E-01	0.2
Cap1	3E-03	0.2	Ganc	4E-01	0.2
Sel1l	2E-04	0.2	Ggct	2E-01	0.2
Adprm	2E-01	0.2	Zdhhc1	3E-01	0.2
Plekhm1	9E-02	0.2	Gstm4	9E-02	0.2
Shf	1E-01	0.2	Rprd1a	3E-02	0.2
Six1	1E-01	0.2	Zswim7	3E-01	0.2
Commd10	2E-02	0.2	Gpatch11	5E-02	0.2
Azin2	1E-01	0.2	Dync1h1	5E-05	0.2
Pigu	4E-01	0.2	Pdlim7	7E-04	0.2
Etv3	3E-01	0.2	Kif16b	1E-02	0.2
Ppp1r2	2E-04	0.2	Usp32	2E-02	0.2
Mtch2	1E-05	0.2	5730480H06Rik	1E-01	0.2
Tprgl	6E-05	0.2	Aarsd1	3E-01	0.2
Hmgn3	4E-05	0.2	Dnajc25	7E-03	0.2
Borcs7	2E-03	0.2	Mertk	2E-01	0.2
Pex19	4E-01	0.2	Vma21	5E-03	0.2
Maml2	3E-08	0.2	Map4k4	4E-05	0.2
Polr3a	3E-02	0.2	Mkks	4E-01	0.2
Galnt13	3E-02	0.2	Coa7	8E-01	0.2
Lcn2	2E-01	0.2	Commd10	9E-03	0.2
Nfs1	2E-01	0.2	Micu2	6E-03	0.2
Phf20l1	2E-04	0.2	Vps11	2E-01	0.2
Ago2	6E-03	0.2	Usf1	1E-01	0.2
Cnp	4E-01	0.2	Cbr1	2E-03	0.2
Gipc1	2E-03	0.2	Cflar	4E-02	0.2
Wdr26	8E-06	0.2	Gpatch2l	7E-02	0.2
Cldnd1	1E-04	0.2	Cpsf3	1E-01	0.2
Dpy19l3	1E-01	0.2	Ssh2	2E-02	0.2
Ppp3cc	1E-01	0.2	Tbce	3E-02	0.2
Zfp638	2E-04	0.2	Armcx2	1E-01	0.2
Myc	5E-02	0.2	Hdac8	2E-03	0.2
Ap1s2	5E-02	0.2	Tnks	9E-04	0.2
Hfe	5E-01	0.2	Lrrc58	1E-03	0.2
Wls	8E-09	0.2	Prdx4	2E-06	0.2
Cpt2	1E+00	0.2	Naa20	2E-03	0.2
BC002059	2E-01	0.2	Mesd	1E-02	0.2
Iqce	2E-01	0.2	Gpam	1E-01	0.2
Trappc9	7E-04	0.2	Tmem37	2E-01	0.2
Dcaf10	2E-02	0.2	Slc4a2	5E-02	0.2
Mcm3ap	7E-01	0.2	Ddx19b	2E-01	0.2
Scamp1	2E-02	0.2	Tmem53	6E-02	0.2
Cyp4f13	4E-01	0.2	Clcn4	5E-03	0.2
Cmtr1	5E-02	0.2	Snip1	3E-01	0.2
Sbf1	4E-02	0.2	Rsph9	4E-04	0.2
Serf2	1E-21	0.2	Josd2	8E-03	0.2
Rpgr	1E+00	0.2	Upp2	2E-02	0.2
Ankrd13a	5E-02	0.2	Plekha4	3E-01	0.2

Limb Mesenchyme						Chondrogenic						Fibroblast						Undefined						Articular/Synovial Fibroblast					
Control			$Notch2^{tm1.1Ecan}$			Control			$Notch2^{tm1.1Ecan}$			Control			$Notch2^{tm1.1Ecan}$			Control			$Notch2^{tm1.1Ecan}$			Control			$Notch2^{tm1.1Ecan}$		
Gene	*p*	FC	Gene	*p*	FC	Gene	*p*	FC	Gene	*p*	FC	Gene	*p*	FC	Gene	*p*	FC	Gene	*p*	FC	Gene	*p*	FC	Gene	*p*	FC	Gene	*p*	FC
						Adprhl2	3E-01	0.1				Smarcad1	2E-02	0.2	Klhl2	1E-02	0.2							Dctn3	7E-05	0.2	Impad1	8E-04	0.2
						Abcb7	1E-01	0.1				Pqlc2	4E-02	0.2	Fbxl19	1E-02	0.2							Cyth1	5E-02	0.2	Ufc1	2E-06	0.2
						Mfsd4a	5E-01	0.1				Vasn	2E-03	0.2	Pias4	2E-02	0.2							Fancf	3E-01	0.2	Acbd4	6E-01	0.2
						Zfp326	5E-03	0.1				Ppp2r3c	4E-02	0.2	Zfp592	3E-03	0.2							Upp2	2E-03	0.2	C1qtnf12	3E-01	0.2
						Mta1	5E-03	0.1				Zfp667	9E-01	0.2	Rabgef1	2E-03	0.2							E130311K13Rik	4E-01	0.2	Hfe	7E-02	0.2
						Sec22a	3E-01	0.1				Rock2	2E-06	0.2	Pnpla6	6E-02	0.2							Adar	8E-02	0.2	Man2a2	1E-01	0.2
						Zc3h14	2E-01	0.1				Smg7	1E-03	0.2	Ralbp1	1E-08	0.2							Atg12	6E-03	0.2	Pepd	3E-03	0.2
						Phf21a	3E-02	0.1				Atp11b	6E-03	0.2	Naf1	4E-03	0.2							Dab2ip	4E-03	0.2	Ctdsp1	5E-02	0.2
						Per1	5E-01	0.1				Yes1	6E-03	0.2	Ubtf	5E-06	0.2							Tmem181a	1E-02	0.2	Mcl1	1E-05	0.2
						Chchd2	7E-18	0.1				Btbd6	4E-01	0.2	Pip5k1c	3E-03	0.2							Dctn2	3E-04	0.2	Tmem38b	1E-01	0.2
						Degs1	1E-03	0.1				Cdk2ap2	2E-02	0.2	Tpgs2	9E-03	0.2							Kdm4b	1E-01	0.2	Zswim8	4E-02	0.2
						Atic	5E-02	0.1				Ptgs1	2E-01	0.2	Robo2	3E-06	0.2							Ppip5k1	5E-01	0.2	Mfsd5	9E-02	0.2
						Cactin	2E-01	0.1				Plekha8	4E-02	0.2	Ift46	5E-03	0.2							Dazap2	5E-02	0.2	Snap23	2E-02	0.2
						Cox18	1E-02	0.1				Cdc23	7E-02	0.2	Bdh2	1E-01	0.2							Mcur1	6E-02	0.2	Atp11c	2E-01	0.2
						Cox7a2	8E-15	0.1				Abhd12	1E-03	0.2	4930523C07Rik	2E-07	0.2							Eif4g3	9E-07	0.2	Rela	4E-01	0.2
						Mybbp1a	2E-03	0.1				Bag3	3E-03	0.2	Plpp2	1E-04	0.2							Ap2a1	4E-03	0.2	Zcchc8	4E-01	0.2
						Tbc1d15	2E-02	0.1				Tmem50b	3E-01	0.2	Zfp30	2E-02	0.2							Mus81	7E-01	0.2	Xrn1	9E-03	0.2
						Sirt5	3E-01	0.1				Sharpin	5E-02	0.2	Vkorc1l1	6E-04	0.2							Zfp975	1E-01	0.2	Kif3c	2E-01	0.2
						Zgpat	1E-01	0.1				Ece2	1E-02	0.2	Ism1	2E-04	0.2							Snx10	3E-01	0.2	Wdr26	6E-04	0.2
						Zfp943	7E-01	0.1				Arpp19	2E-06	0.2	Zdhhc14	7E-02	0.2							Me2	9E-02	0.2	Prkcd	8E-01	0.2
						Gm28198	6E-01	0.1				Gfm2	4E-01	0.2	Cmtr1	4E-04	0.2							Snapin	7E-05	0.2	Smarcd3	1E-01	0.2
						Aimp2	4E-01	0.1				Nfkb2	1E-01	0.2	Lifr	4E-02	0.2							Gk5	3E-02	0.2	Rbfox2	4E-06	0.2
						Wwtr1	5E-04	0.1				Vps26a	2E-04	0.2	2410004B18Rik	4E-03	0.2							Fam32a	3E-03	0.2	Wsb2	6E-04	0.2
						Hnrnpdl	2E-05	0.1				Ddx58	2E-02	0.2	Dnajc3	3E-12	0.2							Timp1	7E-01	0.2	1700123M08Rik	2E-01	0.2
						Pcbd2	2E-04	0.1				Poglut1	3E-02	0.2	Arhgef7	5E-02	0.2							Rxrb	6E-01	0.2	Zfand3	3E-04	0.2
						Ssr4	9E-09	0.1				Fbxo30	4E-04	0.2	Pcbd2	1E-07	0.2							Mapk8ip1	6E-01	0.2	Dcaf7	1E-01	0.2
						Bysl	1E-01	0.1				Itih2	5E-01	0.2	Twistnb	3E-03	0.2							Polb	6E-03	0.2	Mrpl34	3E-03	0.2
						Ubxn4	1E-06	0.1				Rela	1E-02	0.2	Smarce1	1E-08	0.2							Dync1li2	2E-03	0.2	H19	8E-06	0.2
						Suz12	2E-02	0.1				Tnfrsf10b	7E-03	0.2	Snx7	3E-06	0.2							Zfand5	7E-06	0.2	Tbcel	4E-01	0.2
						Atp6v0c	3E-10	0.1				Rbm27	9E-03	0.2	Ccdc174	1E-04	0.2							Men1	2E-01	0.2	Tor1aip1	1E-02	0.2
						Patz1	6E-02	0.1				Tada2b	5E-01	0.2	Sept8	7E-03	0.2							Cspp1	6E-03	0.2	Tmem41b	2E-01	0.2
						Cnot9	8E-02	0.1				Psmd9	9E-03	0.2	Clip2	7E-03	0.2							Taf1	5E-04	0.2	Arid2	8E-02	0.2
						Tgfbr1	1E-01	0.1				Golga1	3E-02	0.2	Zbed4	2E-01	0.2							Xab2	4E-01	0.2	Poc1b	6E-01	0.2
						Dcun1d1	1E-01	0.1				Rad54l2	2E-02	0.2	Fam91a1	2E-01	0.2							Kdm4a	6E-02	0.2	Mast2	7E-03	0.2
						Rae1	2E-01	0.1				Ddi2	2E-02	0.2	Hsf2	2E-02	0.2							Rap1gds1	3E-03	0.2	Efl1	3E-01	0.2
						Abhd17c	1E-01	0.1				Itfg1	2E-03	0.2	Khdrbs1	2E-07	0.2							Dnajc18	1E-02	0.2	4921524J17Rik	1E-02	0.2
						Gtf2a1	1E-01	0.1				Arl2bp	3E-04	0.2	Bod1l	6E-05	0.2							Kdm7a	4E-02	0.2	Hps1	6E-01	0.2
						Ptcd3	1E-01	0.1				Xrn2	3E-06	0.2	Cwc25	8E-02	0.2							Rnpepl1	3E-02	0.2	Nid2	1E-01	0.2
						Nbr1	3E-02	0.1				Ankrd13c	3E-03	0.2	Cfl1	2E-14	0.2							F8	5E-02	0.2	Cnot6	6E-04	0.2
						Mff	5E-04	0.1				Ints9	1E-02	0.2	Impad1	4E-08	0.2							Brf1	2E-01	0.2	Phtf1	2E-02	0.2
						Gm39469	7E-01	0.1				Gm19710	5E-01	0.2	Gramd4	6E-02	0.2							Pelp1	2E-01	0.2	Cnot9	6E-01	0.2
						Kif13b	3E-01	0.1				Mitd1	5E-02	0.2	Rnf114	1E-01	0.2							Egfr	2E-04	0.2	Haus2	1E+00	0.2
						Sdhaf1	4E-03	0.1				Fktn	5E-03	0.2	Tmem164	4E-04	0.2							Uqcc3	6E-05	0.2	Klc4	3E-01	0.2
						Nprl2	5E-01	0.1				Rb1	3E-01	0.2	Crybg3	1E-04	0.2							Plxnb2	1E-03	0.2	Ywhab	1E-06	0.2
						Zfp560	4E-03	0.1				Twf2	1E-01	0.2	Rnf150	1E-09	0.2							Ankrd13c	8E-02	0.2	Washc3	2E-04	0.2
						Aqr	4E-02	0.1				Txnrd2	6E-01	0.2	Ccdc122	3E-02	0.2							Zfp653	3E-01	0.2	Rhog	1E-01	0.2
						Metap2	1E-09	0.1				Acd	5E-03	0.2	Fkbp3	6E-12	0.2							Vps51	6E-01	0.2	Ankrd24	2E-01	0.2
						Ndufc2	8E-10	0.1				Hinfp	1E-01	0.2	Msrb1	2E-05	0.2							Accs	4E-01	0.2	Lmf1	8E-01	0.2
						Timm50	1E-02	0.1				Chac2	4E-02	0.2	Phrf1	1E-04	0.2							Amn1	1E-02	0.2	Pld1	8E-03	0.2
						Morf4l2	6E-08	0.1				1810034E14Rik	3E-01	0.2	Ifngr1	4E-05	0.2							Fcho2	8E-05	0.2	Kpna6	2E-01	0.2
						Lmf1	8E-01	0.1				Ralb	1E-02	0.2	Tardbp	4E-06	0.2							Huwe1	4E-05	0.2	Rc3h1	2E-02	0.2
						Rpl37	1E-23	0.1				Trappc2	9E-01	0.2	Psmd10	1E-04	0.2							Pde7a	1E-02	0.2	Ppp3cb	3E-03	0.2
						Sec16b	4E-01	0.1				Fads3	1E-03	0.2	Polr3k	9E-06	0.2							Nhlrc3	3E-01	0.2	BC051226	3E-01	0.2
						Usp12	1E-02	0.1				Prkd2	2E-02	0.2	Med1	3E-04	0.2							Gm37494	2E-02	0.2	Herc2	3E-02	0.2
						Ext2	4E-04	0.1				Spa17	3E-01	0.2	4932438A13Rik	6E-05	0.2							Pik3r4	2E-02	0.2	Chpf	6E-01	0.2
						Zfp639	2E-03	0.1				Zfp827	1E-01	0.2	Spice1	2E-03	0.2							Rnf123	3E-01	0.2	Galt	3E-01	0.2
						Gnptg	5E-02	0.1				Ywhab	3E-08	0.2	Ddx20	2E-02	0.2							Hoxc9	2E-02	0.2	Flcn	3E-01	0.2
						Eif3j1	3E-06	0.1				Slc25a28	2E-01	0.2	Il1rn	4E-08	0.2							Sike1	2E-01	0.2	Cmtr1	3E-01	0.2
						Snx10	3E-01	0.1				Rbm8a	3E-05	0.2	Sgk3	3E-03	0.2							Dcaf8	4E-05	0.2	Mpv17l	5E-01	0.2
						Zfp869	1E-02	0.1				Psat1	3E-01	0.2	Suco	1E-03	0.2							Arhgap35	9E-04	0.2	Klf6	2E-03	0.2
						Qsox2	9E-02	0.1				Vps52	8E-01	0.2	Ppp2r1b	7E-03	0.2							Mettl4	3E-01	0.2	Arhgap31	1E-01	0.2
						Pttg1	3E-02	0.1				Hspa4	7E-05	0.2	Sspn	8E-04	0.2							Tug1	4E-02	0.2	C1qtnf6	1E-02	0.2
						Rps7	4E-22	0.1				Sdc3	2E-03	0.2	Kif1b	3E-10	0.2							Slf2	8E-03	0.2	Ndufa3	2E-06	0.2
						Bpnt1	6E-02	0.1				Slc35b4	3E-01	0.2	Lrp12	3E-02	0.2							Gpm6b	1E-05	0.2	Rmdn2	1E-01	0.2

Limb Mesenchyme

Control			*Notch2* tm1.1Ecan		
Gene	*p*	FC	Gene	*p*	FC

Chondrogenic

Control			*Notch2* tm1.1Ecan		
Gene	*p*	FC	Gene	*p*	FC
Mmaa	1E-01	0.1			
Rpap3	1E-01	0.1			
Ubp1	2E-02	0.1			
Lyrm1	9E-01	0.1			

Fibroblast

Control			*Notch2* tm1.1Ecan		
Gene	*p*	FC	Gene	*p*	FC
Pcm1	7E-06	0.2	Fam53c	5E-01	0.2
Dnajc8	9E-07	0.2	Fubp1	1E-09	0.2
Tsen2	2E-01	0.2	Tmem138	2E-02	0.2
Prr14	5E-02	0.2	Zbtb38	7E-05	0.2
Dync1i2	2E-06	0.2	Tmem216	6E-02	0.2
Mrrf	2E-01	0.2	Ulk1	5E-01	0.2
Anapc13	1E-06	0.2	Rab8b	1E-04	0.2
Mecr	1E-02	0.2	Zfp180	9E-02	0.2
Map1s	7E-02	0.2	Riiad1	5E-02	0.2
Atp13a3	2E-04	0.2	Pgs1	1E-03	0.2
Hnrnpa2b1	1E-09	0.2	Clk4	2E-03	0.2
Pcyt1a	2E-03	0.2	Ficd	5E-02	0.2
Adnp2	3E-01	0.2	Cgrrf1	6E-03	0.2
Tuba4a	1E-01	0.2	Slc25a13	4E-02	0.2
Vangl1	2E-02	0.2	Far1	4E-05	0.2
Rnf150	2E-05	0.2	Pdcl	1E-02	0.2
Wdr1	1E-04	0.2	Nipa1	2E-02	0.2
Cgref1	1E-01	0.2	Atrx	1E-06	0.2
Eif6	9E-04	0.2	Dnlz	1E-06	0.2
Csgalnact2	3E-01	0.2	Ubac2	9E-04	0.2
Clip2	1E-01	0.2	Zc3h18	9E-05	0.2
Adamts10	7E-02	0.2	Klf13	1E-04	0.2
U2af1l4	3E-03	0.2	Hsp90b1	2E-13	0.2
Cacul1	1E-02	0.2	8030462N17Rik	2E-02	0.2
Ythdf1	5E-02	0.2	Map3k4	1E-03	0.2
Gstcd	3E-02	0.2	Dst	7E-08	0.2
Smtn	4E-03	0.2	Eif1ad	1E-03	0.2
Macrod1	1E-01	0.2	Hnrnpf	4E-08	0.2
Ercc6	2E-01	0.2	Med6	1E-02	0.2
Meaf6	3E-03	0.2	Ywhab	4E-09	0.2
Stx8	1E-02	0.2	Mboat7	2E-01	0.2
Dpagt1	5E-02	0.2	Cstf1	5E-02	0.2
Gtf3c6	2E-01	0.2	Gsr	7E-03	0.2
Eif2b1	7E-02	0.2	Mettl9	9E-07	0.2
Sap30l	2E-02	0.2	Arid1a	4E-05	0.2
Lancl2	1E-02	0.2	Mterf2	2E-01	0.2
Pou2f1	7E-03	0.2	Rhoj	5E-05	0.2
Drg2	9E-02	0.2	Capzb	4E-11	0.2
Mlf2	6E-05	0.2	Apeh	6E-03	0.2
Poglut2	8E-02	0.2	Ptar1	4E-03	0.2
Ppp1r14b	3E-10	0.2	Nid2	8E-05	0.2
Gm19705	1E-01	0.2	Lpar4	4E-04	0.2
Shoc2	6E-04	0.2	Blvra	2E-03	0.2
Ergic1	1E-04	0.2	Acin1	2E-08	0.2
D630045J12Rik	2E-03	0.2	Pik3r3	6E-03	0.2
Utp3	9E-05	0.2	Pkig	5E-05	0.2
Ino80e	2E-01	0.2	Wapl	1E-04	0.2
Immp1l	3E-04	0.2	Smg7	5E-04	0.2
Arhgap31	2E-03	0.2	Slc27a4	6E-05	0.2
Ubtf	5E-04	0.2	Zfp943	9E-02	0.2
Prelid1	1E-04	0.2	Ttc27	3E-01	0.2
Pik3c3	2E-01	0.2	Ppp3cc	9E-02	0.2
Stub1	2E-03	0.2	Gtpbp10	4E-02	0.2
Smu1	4E-02	0.2	Cnnm3	9E-02	0.2
Napg	7E-02	0.2	Srfbp1	7E-03	0.2
Bclaf1	1E-06	0.2	Stx17	1E-02	0.2
Ewsr1	7E-04	0.2	Ptpn4	1E-02	0.2
Thap12	6E-03	0.2	Ewsr1	1E-05	0.2
Hoxc5	1E-01	0.2	Entr1	7E-02	0.2
Mertk	5E-01	0.2	Rfx7	5E-05	0.2
Wdr82	2E-01	0.2	Pogz	8E-03	0.2
Lin7a	2E-01	0.2	Thumpd1	9E-02	0.2
Wdr89	8E-01	0.2	Nup54	5E-04	0.2
Rbis	8E-02	0.2	Exosc10	9E-04	0.2

Undefined

Control			*Notch2* tm1.1Ecan		
Gene	*p*	FC	Gene	*p*	FC

Articular/Synovial Fibroblast

Control			*Notch2* tm1.1Ecan		
Gene	*p*	FC	Gene	*p*	FC
Cpeb4	3E-03	0.2	Eipr1	2E-01	0.2
Daglb	3E-02	0.2	Borcs7	4E-03	0.2
Atp6v1h	1E-03	0.2	2610301B20Rik	2E-02	0.2
Xpc	4E-01	0.2	Zscan29	1E-01	0.2
Morc4	2E-01	0.2	Pard3	1E-04	0.2
Rnf34	1E-01	0.2	Ndufv3	8E-07	0.2
Slc49a4	2E-02	0.2	Kif3a	3E-02	0.2
Haus2	2E-01	0.2	Tssc4	8E-01	0.2
Vipas39	1E-01	0.2	1110059G10Rik	5E-02	0.2
Dnajb1	7E-03	0.2	Ccnt2	7E-02	0.2
Zfp871	1E-01	0.2	Il15ra	3E-01	0.2
6430590A07Rik	8E-01	0.2	Nf2	2E-02	0.2
Zfp60	3E-01	0.2	4933407K13Rik	6E-01	0.2
Nup93	5E-01	0.2	Ccdc71	5E-01	0.2
Gpx1	3E-10	0.2	Bag4	2E-01	0.2
Slu7	6E-03	0.2	Dnm2	1E-02	0.2
Togaram1	3E-02	0.2	Amy1	3E-01	0.2
Map7d1	1E-04	0.2	Hexb	9E-04	0.2
Mgat4b	2E-01	0.2	Nek6	2E-01	0.2
Mapk9	1E-01	0.2	Psmc1	5E-03	0.2
Ap4b1	3E-01	0.2	Nbn	6E-01	0.2
Gm26881	2E-01	0.2	Stxbp4	3E-01	0.2
Csgalnact2	3E-02	0.2	Ech1	2E-04	0.2
Txnrd1	1E-03	0.2	Fam199x	2E-01	0.2
Slc18b1	4E-02	0.2	Vps8	5E-02	0.2
Tmem242	5E-02	0.2	Bace1	3E-03	0.2
Tmsb4x	1E-19	0.2	Prkce	9E-02	0.2
Polr2a	3E-04	0.2	Sec14l1	2E-02	0.2
Casd1	2E-02	0.2	Hmgcl	1E-01	0.2
Pacsin3	3E-01	0.2	Vgll4	3E-03	0.2
Bcr	3E-02	0.2	Ppfia1	2E-01	0.2
Ap4e1	3E-02	0.2	Fyco1	6E-02	0.2
Khdrbs3	2E-01	0.2	Zfp871	2E-02	0.2
Mark4	8E-01	0.2	Mapk14	5E-02	0.2
Prkra	6E-03	0.2	Mical3	2E-01	0.2
Tsen34	3E-05	0.2	Snx4	3E-02	0.2
Gpi1	7E-05	0.2	Anapc7	1E-01	0.2
Thap11	1E-01	0.2	Gtf3c3	2E-01	0.2
Pan3	1E-03	0.2	Spidr	4E-02	0.2
Gpkow	6E-01	0.2	Fancm	4E-01	0.2
Mov10	4E-01	0.2	H1f0	3E-03	0.2
Dpy19l1	1E-01	0.2	Tmeff1	6E-01	0.2
Phf20	2E-03	0.2	Pde12	9E-01	0.2
Epg5	2E-01	0.2	Slx1b	2E-01	0.2
Nfrkb	1E+00	0.2	Pes1	3E-01	0.2
Pak3	2E-04	0.2	Pigyl	1E-02	0.2
Ogfod2	7E-01	0.2	Exoc6b	2E-03	0.2
Srgap2	1E-05	0.2	Mtmr9	2E-01	0.2
Mtmr10	1E-01	0.2	E130102H24Rik	6E-01	0.2
Coq5	1E-01	0.2	Rnf34	2E-01	0.2
1110008P14Rik	2E-04	0.2	Npc1	3E-02	0.2
Tceal1	6E-01	0.2	Chmp3	6E-03	0.2
Bach2	5E-01	0.2	Ethe1	2E-01	0.2
Plxdc2	2E-06	0.2	Rab8b	1E-01	0.2
Nenf	6E-09	0.2	Relb	3E-02	0.2
Prelid1	3E-06	0.2	Pfkfb2	9E-01	0.2
Rxra	5E-01	0.2	Uqcc2	3E-07	0.2
Eif4ebp3	2E-01	0.2	Cyp4f13	2E-01	0.2
Cmc2	2E-01	0.2	Pnpt1	2E-01	0.2
Eya3	2E-02	0.2	Cdr2l	2E-02	0.2
B230307C23Rik	1E-02	0.2	Phf20	1E-03	0.2
Vcp	1E-09	0.2	Pcgf3	3E-02	0.2
Mfsd11	1E-03	0.2	Phlda3	9E-04	0.2
Fam8a1	1E-01	0.2	Eml4	4E-02	0.2

Limb Mesenchyme						Chondrogenic						Fibroblast						Undefined						Articular/Synovial Fibroblast					
Control			*Notch2*$^{tm1.1Ecan}$			Control			*Notch2*$^{tm1.1Ecan}$			Control			*Notch2*$^{tm1.1Ecan}$			Control			*Notch2*$^{tm1.1Ecan}$			Control			*Notch2*$^{tm1.1Ecan}$		
Gene	*p*	FC	Gene	*p*	FC	Gene	*p*	FC	Gene	*p*	FC	Gene	*p*	FC	Gene	*p*	FC	Gene	*p*	FC	Gene	*p*	FC	Gene	*p*	FC	Gene	*p*	FC
												Hectd1	1E-05	0.2	Lrp1	4E-15	0.2							Sgcb	4E-02	0.2	Capns1	9E-08	0.2
												Wsb1	2E-04	0.2	Armc5	2E-01	0.2							Kat5	4E-01	0.2	Rabl3	7E-01	0.2
												Tsr2	4E-01	0.2	Phc1	3E-01	0.2							Eid1	8E-07	0.2	Akirin2	4E-03	0.2
												Ipo8	1E-02	0.2	Slc35e4	2E-04	0.2							Zc3h7b	9E-01	0.2	Memo1	6E-03	0.2
												Zfyve26	9E-02	0.2	Cd164	6E-06	0.2							Cry1	3E-01	0.2	Unk	3E-01	0.2
												Erc1	2E-05	0.2	4933407K13Rik	3E-01	0.2							Armc9	7E-02	0.2	Ssbp3	7E-02	0.2
												Gcc2	1E-02	0.2	Baz2a	4E-04	0.2							Fam110b	9E-02	0.2	Ifnar1	3E-01	0.2
												Aak1	2E-04	0.2	Zfp68	2E-02	0.2							Trappc8	2E-02	0.2	Acads	8E-02	0.2
												Kif21a	7E-03	0.2	Rpf2	8E-02	0.2							Mfhas1	6E-02	0.2	Ice1	2E-01	0.2
												Cnbd2	3E-01	0.2	Ruvbl1	4E-03	0.2							Sepsecs	9E-01	0.2	Sntb1	2E-01	0.2
												Dph3	1E-05	0.2	Bod1	5E-02	0.2							Snx21	7E-02	0.2	Plekhf1	4E-02	0.2
												Traf6	1E-01	0.2	Ap1ar	2E-02	0.2							Zfyve19	9E-01	0.2	Herpud2	7E-02	0.2
												Ppt2	9E-02	0.2	Tbc1d14	4E-02	0.2							Ankrd40	1E-02	0.2	Plbd2	2E-04	0.2
												Myzap	7E-03	0.2	Kif1c	4E-03	0.2							Fbxo38	2E-02	0.2	Aldh9a1	3E-01	0.2
												Tctex1d2	9E-02	0.2	Cdk4	6E-11	0.2							Pbdc1	6E-03	0.2	Fkbp1a	8E-06	0.2
												Ppig	6E-05	0.2	Usp3	1E-03	0.2							Snx1	1E-03	0.2	Zfp26	8E-01	0.2
												Zfp983	4E-01	0.2	Fam162a	4E-08	0.2							Wdr82	2E-01	0.2	Rab35	9E-02	0.2
												Anxa5	5E-09	0.2	Zfp157	4E-01	0.2							Tspan4	2E-04	0.2	Ttc17	1E-01	0.2
												Rnd2	3E-01	0.2	Plekhg5	1E-02	0.2							Nelfb	5E-02	0.2	Ppil3	5E-02	0.2
												Gpd2	3E-01	0.2	Smpd1	1E-06	0.2							Serinc1	2E-06	0.2	Abcb8	5E-01	0.2
												Nsmce2	1E-06	0.2	Srek1	3E-08	0.2							Lmna	7E-09	0.2	B4galt4	1E-01	0.2
												Cdk19	1E-02	0.2	Abhd13	6E-02	0.2							Bace1	5E-04	0.2	Pgghg	1E-01	0.2
												Gsta4	5E-01	0.2	Wwox	5E-04	0.2							Pgap2	1E-02	0.2	Akip1	7E-02	0.2
												Prdx2	4E-08	0.2	Afg1l	3E-02	0.2							Zfp938	5E-01	0.2	Ubr7	2E-01	0.2
												Cmc4	2E-01	0.2	Gba	5E-02	0.2							Tada3	6E-02	0.2	Gnai3	7E-03	0.2
												Hoxa11os	6E-01	0.2	Zfp653	3E-02	0.2							Tasor	4E-02	0.2	Mrpl36	1E-02	0.2
												Ptcd3	8E-03	0.2	Sec22a	4E-03	0.2							Kdm3b	2E-01	0.2	Agfg1	6E-03	0.2
												Fut11	2E-01	0.2	Alg10b	8E-03	0.2							Rad52	4E-01	0.2	Adam12	7E-02	0.2
												Slc39a14	4E-02	0.2	Pmm2	5E-02	0.2							Cyb561d2	4E-02	0.2	Morn2	3E-02	0.2
												Ncam1	1E-01	0.2	Nap1l4	1E-07	0.2							Slc17a5	2E-01	0.2	Dnaja1	1E-06	0.2
												Rbmx	3E-02	0.2	Glb1l	1E-02	0.2							Zmpste24	9E-03	0.2	Kmt2e	1E-04	0.2
												Daam2	1E-02	0.2	Fam76a	5E-06	0.2							Dis3l	2E-01	0.2	Fam222b	3E-01	0.2
												Csnk1g3	1E-04	0.2	Ccdc181	4E-02	0.2							Wrap73	5E-01	0.2	Mgrn1	4E-01	0.2
												Gdap2	3E-02	0.2	Polr2f	3E-11	0.2							1600010M07Rik	1E-01	0.2	Rab10	1E-04	0.2
												Psd3	1E-04	0.2	Bbs5	5E-02	0.2							9330159M07Rik	2E-01	0.2	Chd4	3E-04	0.2
												Serbp1	1E-10	0.2	Cpsf6	1E-05	0.2							Rad54l2	3E-02	0.2	Washc1	3E-02	0.2
												Zranb1	1E-02	0.2	Gm15952	2E-01	0.2							Coq6	4E-01	0.2	Selenom	6E-05	0.2
												Hspa13	7E-04	0.2	Mmp14	2E-10	0.2							Snrnp35	6E-01	0.2	Arf6	4E-02	0.2
												Hspb8	9E-03	0.2	Atf7ip	2E-02	0.2							Emc1	1E-01	0.2	Elf2	7E-03	0.2
												Gm15283	5E-04	0.2	Ttc32	3E-01	0.2							Agps	1E-03	0.2	Txnrd1	8E-02	0.2
												Alkbh6	2E-01	0.2	Trappc11	4E-02	0.2							Zc3h7a	4E-02	0.2	Ptpra	2E-04	0.2
												Rmnd1	5E-03	0.2	Mcur1	1E-01	0.2							Impact	8E-06	0.2	Kif13a	4E-01	0.2
												Dynll2	2E-06	0.2	Srsf7	2E-08	0.2							Brox	3E-01	0.2	Pparg	8E-03	0.2
												Nubp1	3E-02	0.2	Zmynd8	2E-07	0.2							Ptges	9E-02	0.2	Pan3	2E-02	0.2
												Skp1a	2E-06	0.1	Nfya	6E-02	0.2							Mbip	9E-02	0.2	Dip2a	6E-02	0.2
												Cnn2	2E-05	0.1	Zfp574	1E-01	0.2							Avl9	2E-02	0.2	Mta3	4E-01	0.2
												Gtf2h2	5E-02	0.1	Ppp4r3a	9E-04	0.2							Btbd7	6E-05	0.2	Ip6k1	2E-03	0.2
												Arl6ip4	3E-03	0.1	Rabep2	1E-01	0.2							Wwc2	5E-05	0.2	Zfp263	3E-01	0.2
												Gabarapl2	5E-06	0.1	Dock6	1E-03	0.2							Stard3nl	6E-02	0.2	Vangl1	2E-01	0.2
												Luc7l3	4E-05	0.1	Arhgap28	2E-04	0.2							Snrpa	8E-03	0.2	Krt10	1E-01	0.2
												Ttc33	8E-02	0.1	Mbnl1	2E-05	0.2							Ybx1	2E-13	0.2	Ift172	4E-02	0.2
												Crk	3E-02	0.1	Eif1ax	7E-10	0.2							Lmbrd1	7E-03	0.2	Tgfb1i1	3E-05	0.2
												Trim47	1E-02	0.1	Gm14966	4E-02	0.2							Slc2a8	3E-01	0.2	Eid1	1E-03	0.2
												Slc35a3	6E-02	0.1	Ankrd13c	4E-02	0.2							Adsl	6E-01	0.2	Dgcr2	2E-02	0.2
												Nmt1	4E-04	0.1	Ralb	6E-04	0.2							Vps26c	1E-01	0.2	Ccdc32	8E-02	0.2
												Smarcc2	2E-02	0.1	Fbxo31	7E-04	0.2							Usp40	7E-02	0.2	Timm44	6E-02	0.1
												Ccdc122	4E-02	0.1	Btaf1	4E-08	0.2							Elf2	8E-03	0.2	Kirrel	3E-04	0.1
												Rheb	1E-05	0.1	Rtn4	9E-10	0.2							Uhrf1bp1l	8E-02	0.2	Abca1	3E-01	0.1
												Gzf1	5E-01	0.1	Ptgr1	1E-02	0.2							Ppp1r12c	1E-01	0.2	Stk25	3E-01	0.1
												Parp6	3E-01	0.1	Gtf3c6	2E-03	0.2							Mad2l2	3E-01	0.2	Lrif1	1E-01	0.1
												Phlpp2	2E-02	0.1	Ugdh	8E-08	0.2							Qsox2	4E-01	0.2	Shprh	7E-02	0.1
												Pbdc1	8E-04	0.1	Smarcad1	1E-03	0.2							Barx1	2E-05	0.2	Pigs	6E-02	0.1
												Thra	5E-05	0.1	Ptprs	5E-04	0.2							Gm1673	4E-03	0.2	Eaf1	6E-01	0.1
												4933400C23Rik	2E-01	0.1	Mnt	3E-02	0.2							Trim56	6E-01	0.2	Zfp553	7E-01	0.1

Limb Mesenchyme					
Control			$Notch2^{tm1.1Ecan}$		
Gene	*p*	FC	Gene	*p*	FC

Chondrogenic					
Control			$Notch2^{tm1.1Ecan}$		
Gene	*p*	FC	Gene	*p*	FC

Fibroblast					
Control			$Notch2^{tm1.1Ecan}$		
Gene	*p*	FC	Gene	*p*	FC
Exosc9	1E-02	0.1	Hectd4	1E-02	0.2
Acsf2	9E-02	0.1	Rragb	8E-02	0.2
Blvrb	1E-03	0.1	Vbp1	5E-04	0.2
Phf5a	2E-03	0.1	Rc3h2	3E-05	0.2
Ccser2	1E-04	0.1	Crtc1	4E-03	0.2
Whamm	7E-01	0.1	Tjp1	6E-04	0.2
Dbndd2	4E-01	0.1	Fam133b	2E-05	0.2
Nop56	2E-02	0.1	Dennd1a	8E-06	0.2
Thap11	8E-01	0.1	Kat6a	1E-02	0.2
Ints7	7E-03	0.1	Rad54l2	2E-03	0.2
Bmp2k	8E-03	0.1	Tnks	4E-02	0.2
Rab35	3E-03	0.1	Cdk5rap1	1E-01	0.2
Eps8l2	6E-03	0.1	Smg5	3E-01	0.2
Larp4b	2E-04	0.1	Itga1	4E-05	0.2
Tut7	2E-03	0.1	Pfkm	5E-02	0.2
Cd82	1E-01	0.1	Ptpn11	6E-05	0.2
Cep170	3E-03	0.1	Ggh	1E-06	0.2
Zfp53	3E-01	0.1	Prcp	5E-03	0.2
Cwc25	2E-01	0.1	Ints11	6E-04	0.2
Antxr1	1E-03	0.1	Snx16	7E-03	0.2
Smim7	2E-03	0.1	Brcc3	2E-02	0.2
Nup214	3E-04	0.1	Aak1	4E-06	0.2
Fyco1	8E-03	0.1	Hectd2	8E-01	0.2
Ppfia1	1E-02	0.1	Psd3	5E-06	0.2
Sepsecs	3E-01	0.1	Col4a3bp	3E-03	0.2
Gadd45g	5E-06	0.1	Cdr2	2E-02	0.2
Fopnl	5E-02	0.1	Ercc6l2	2E-03	0.2
Taf5l	3E-01	0.1	Cyb5b	5E-06	0.2
Dusp10	7E-01	0.1	Coq7	3E-03	0.2
Clasrp	1E-01	0.1	Pon3	1E-03	0.2
P3h2	5E-02	0.1	Paip2b	2E-02	0.2
Phc1	9E-01	0.1	Nudt9	2E-02	0.2
Uhrf1bp1l	7E-04	0.1	Strbp	7E-06	0.2
Mad2l2	2E-02	0.1	Fam102b	6E-04	0.2
Foxk2	1E-02	0.1	Lage3	2E-04	0.2
Ddx46	2E-04	0.1	Chd9	7E-06	0.2
Inpp5a	8E-02	0.1	Cacybp	2E-05	0.2
Hmgcr	8E-03	0.1	Hnrnph3	4E-04	0.2
Plxna1	9E-01	0.1	Pcid2	5E-02	0.2
Map4k5	3E-04	0.1	Zbtb5	2E-01	0.2
Myo9a	8E-05	0.1	Fzd1	2E-06	0.2
Sf3b1	3E-09	0.1	Fubp3	1E-01	0.2
Morn1	1E-01	0.1	Tmed8	2E-01	0.2
Nt5c3b	3E-02	0.1	Dennd6a	1E-02	0.2
Atg2a	2E-01	0.1	Agpat3	3E-05	0.2
Zfp36l2	4E-05	0.1	Clcn6	2E-02	0.2
Sdc1	7E-03	0.1	Ddx19b	3E-02	0.2
Cnnm2	9E-02	0.1	Frmd6	9E-07	0.2
Isg20l2	3E-01	0.1	Gtpbp1	2E-02	0.2
Selenoi	2E-02	0.1	Ogg1	1E-01	0.2
Prrc2b	4E-04	0.1	Tmx4	4E-07	0.2
Tlnrd1	7E-02	0.1	Jcad	1E-04	0.2
Akr1e1	2E-01	0.1	Ino80d	4E-02	0.2
Rabgap1l	1E-04	0.1	Ccm2	9E-04	0.2
Gpn3	2E-01	0.1	Borcs7	3E-03	0.2
Ubqln2	6E-01	0.1	Dglucy	5E-02	0.2
Bysl	5E-02	0.1	Axin1	6E-03	0.2
Anxa4	2E-05	0.1	Mfsd5	1E-01	0.2
Gulp1	5E-03	0.1	Pank3	1E-02	0.2
Gpkow	4E-02	0.1	Map4k3	9E-09	0.2
Pgm1	8E-02	0.1	Kin	3E-04	0.2
Hikeshi	1E-04	0.1	Sephs2	5E-04	0.2
Gart	6E-03	0.1	Morn2	2E-03	0.2
Wdr36	2E-01	0.1	Celf1	3E-07	0.2

Undefined					
Control			$Notch2^{tm1.1Ecan}$		
Gene	*p*	FC	Gene	*p*	FC

Articular/Synovial Fibroblast					
Control			$Notch2^{tm1.1Ecan}$		
Gene	*p*	FC	Gene	*p*	FC
Mettl2	5E-01	0.2	Ppm1d	4E-01	0.1
Dnlz	2E-03	0.2	Zfp1	7E-01	0.1
Prdx2	9E-08	0.2	Phkb	1E-01	0.1
Prpf39	1E-02	0.2	Mar2	4E-04	0.1
Dnajb2	2E-01	0.2	Tbc1d12	2E-02	0.1
Dync2h1	8E-03	0.2	Atp6v1h	9E-03	0.1
Oaz2	9E-03	0.2	Vcp	5E-07	0.1
Tex2	5E-01	0.2	Slc38a9	2E-01	0.1
Siah1a	5E-02	0.2	St6galnac4	5E-02	0.1
Gabbr1	1E-01	0.2	BC005624	4E-04	0.1
Kctd2	9E-01	0.2	Atxn7l1	1E-03	0.1
Ubqln2	1E-01	0.2	Fmr1	1E-01	0.1
Pef1	8E-03	0.2	Alg10b	3E-01	0.1
Kdm5d	1E-01	0.2	Ilrun	6E-02	0.1
Golga4	6E-04	0.2	Asb8	8E-02	0.1
Rab10	1E-06	0.2	Ep300	2E-01	0.1
Memo1	2E-03	0.2	Usp4	7E-02	0.1
Iqcc	3E-01	0.2	Gabarap	2E-10	0.1
Dbp	3E-02	0.2	Zfp664	2E-02	0.1
Acox1	4E-06	0.2	Rin2	2E-03	0.1
Ptpra	4E-06	0.2	Elf1	6E-03	0.1
Ncln	1E-01	0.2	Btbd7	1E-03	0.1
Mtfmt	3E-01	0.2	Smarce1	4E-04	0.1
Kctd18	9E-01	0.2	Smg1	1E-02	0.1
Ppp2r3c	3E-02	0.2	Apc	2E-03	0.1
Tmlhe	2E-01	0.2	Lamtor4	5E-03	0.1
Sf3a2	4E-01	0.2	Clic1	1E-05	0.1
Cenpc1	3E-02	0.2	Dcp1a	8E-01	0.1
Zbtb7b	1E-01	0.2	Sgta	2E-02	0.1
Zfand6	1E-04	0.2	Dync1i2	4E-04	0.1
Golim4	3E-04	0.2	Lrrc28	6E-01	0.1
Ndfip2	1E-01	0.2	Myo6	4E-03	0.1
Prmt7	1E-01	0.2	Lin37	9E-02	0.1
Kif16b	2E-04	0.2	Actr2	2E-02	0.1
Rpia	4E-01	0.2	Ftl1	2E-15	0.1
Gm49336	7E-01	0.2	Zfp934	2E-01	0.1
Rnasek	3E-05	0.2	Rufy1	4E-01	0.1
Myd88	5E-01	0.2	Pex14	3E-02	0.1
4930517O19Rik	4E-01	0.2	Pcf11	8E-01	0.1
Pgghg	6E-01	0.2	Suds3	2E-02	0.1
Spr	9E-02	0.2	Slf2	3E-02	0.1
Gnai2	2E-09	0.2	Mios	4E-01	0.1
Tmem87b	6E-04	0.2	Nenf	6E-05	0.1
Adnp	5E-03	0.2	Ypel2	2E-01	0.1
Hmgxb3	6E-01	0.2	Pacsin3	3E-01	0.1
Med12	6E-01	0.2	Dgke	6E-01	0.1
Spast	9E-03	0.2	Isca1	8E-01	0.1
Focad	1E-02	0.2	Zfat	4E-01	0.1
Gtdc1	2E-01	0.2	Jun	9E-04	0.1
Megf8	2E-01	0.2	Tex9	4E-01	0.1
Pibf1	2E-02	0.2	A630089N07Rik	4E-01	0.1
Zfp113	3E-01	0.2	Ints8	1E-01	0.1
Cfap36	2E-04	0.2	Snx1	2E-02	0.1
Elk4	8E-04	0.2	Sp3	7E-02	0.1
Zfand3	2E-05	0.2	Mrps36	2E-02	0.1
Rnf25	8E-02	0.2	Acer3	6E-02	0.1
Gna13	2E-02	0.2	Gtpbp8	4E-01	0.1
Ppp6c	5E-02	0.2	Ciao1	7E-02	0.1
Paox	3E-01	0.2	Fkbp1b	1E+00	0.1
Nmrk1	2E-01	0.2	Slc39a6	8E-02	0.1
Smpd2	3E-01	0.2	mt-Nd1	6E-08	0.1
Zfp976	6E-01	0.2	Cul1	2E-02	0.1
4632427E13Rik	8E-03	0.2	Gtf2h1	2E-02	0.1
Tut7	3E-05	0.2	Spsb3	1E+00	0.1

Limb Mesenchyme						Chondrogenic						Fibroblast						Undefined						Articular/Synovial Fibroblast					
Control			*Notch2* tm1.1Ecan			Control			*Notch2* tm1.1Ecan			Control			*Notch2* tm1.1Ecan			Control			*Notch2* tm1.1Ecan			Control			*Notch2* tm1.1Ecan		
Gene	*p*	FC	Gene	*p*	FC	Gene	*p*	FC	Gene	*p*	FC	Gene	*p*	FC	Gene	*p*	FC	Gene	*p*	FC	Gene	*p*	FC	Gene	*p*	FC	Gene	*p*	FC
												Ncoa3	1E-03	0.1	Trit1	2E-02	0.2							Utp6	4E-01	0.2	Umad1	5E-02	0.1
												Setd1a	7E-02	0.1	Rb1	2E-02	0.2							Nup35	3E-01	0.2	Gm41724	8E-01	0.1
												AI987944	1E-01	0.1	Sgk1	1E-03	0.2							Tkfc	7E-01	0.2	Naf1	6E-01	0.1
												Naglu	2E-02	0.1	Rbl2	9E-02	0.2							Tmem53	2E-01	0.2	Psmd10	7E-02	0.1
												Dpy30	7E-03	0.1	Hikeshi	5E-06	0.2							Cdc27	1E-01	0.2	Reep3	9E-02	0.1
												Sgcb	3E-02	0.1	Edc4	1E-02	0.2							Scaf11	3E-05	0.2	a	1E-01	0.1
												Ap3s2	2E-01	0.1	Ppargc1a	5E-01	0.2							Ppp1r37	1E-01	0.2	1300002E11Rik	8E-02	0.1
												Rbm10	8E-03	0.1	1700109H08Rik	3E-01	0.2							Hdac8	2E-04	0.2	Hps3	1E-01	0.1
												Rmdn2	3E-01	0.1	5031439G07Rik	1E-02	0.2							Tfpt	2E-01	0.2	Arhgef9	5E-01	0.1
												Polr2a	3E-05	0.1	Nt5c3b	5E-04	0.2							Pld3	8E-05	0.2	Camsap2	2E-01	0.1
												Hint1	4E-09	0.1	Tmem94	3E-02	0.2							Epm2a	1E-01	0.2	Hccs	3E-01	0.1
												Psma4	2E-06	0.1	Idua	4E-02	0.2							Bbs5	2E-01	0.2	Gatad2a	3E-03	0.1
												Cers6	2E-03	0.1	Mmut	1E-01	0.2							Gltp	5E-03	0.2	Sh3rf1	1E-01	0.1
												Acp6	8E-02	0.1	Csnk1g2	5E-04	0.2							Tubb6	8E-04	0.2	Samd8	3E-02	0.1
												Fra10ac1	2E-02	0.1	Tmem241	1E-02	0.2							Gm48742	1E-01	0.2	Tpd52l2	1E-02	0.1
												Psmd1	6E-06	0.1	Spsb4	3E-02	0.2							Samd14	9E-01	0.2	Pggt1b	2E-01	0.1
												Usp33	4E-02	0.1	Pitrm1	6E-03	0.2							Rheb	7E-06	0.2	Mfsd14a	2E-01	0.1
												Tbc1d22b	2E-01	0.1	Rala	4E-04	0.2							Cipc	7E-01	0.2	Atraid	5E-07	0.1
												Sf3b2	1E-06	0.1	Gmfb	1E-04	0.2							Arl8b	3E-03	0.2	Arhgap1	4E-01	0.1
												Dhrs4	1E-02	0.1	Parp11	5E-01	0.2							Zhx2	7E-01	0.2	Prrx1	2E-06	0.1
												Dyrk1a	2E-04	0.1	Ppp6r3	6E-05	0.2							Hexim1	1E-02	0.2	Cox6c	8E-11	0.1
												Elp4	4E-03	0.1	Tecr	2E-07	0.2							Ankrd49	1E-01	0.2	1700030K09Rik	5E-01	0.1
												Pwwp3a	2E-02	0.1	Nudcd3	5E-05	0.2							Gaa	4E-05	0.2	Mprip	7E-03	0.1
												Stx6	7E-02	0.1	Usp31	1E-01	0.2							Eipr1	3E-01	0.2	2810402E24Rik	6E-01	0.1
												Ccdc9	6E-02	0.1	Ptpn23	8E-03	0.2							Gak	4E-02	0.2	Traf7	3E-03	0.1
												Ttll5	8E-02	0.1	Oard1	4E-03	0.2							Reps1	4E-02	0.2	Snf8	3E-03	0.1
												Msto1	1E-01	0.1	Retreg2	1E-05	0.2							Dhx16	2E-01	0.2	Map1b	6E-04	0.1
												Rcbtb2	2E-02	0.1	Kdm4b	7E-02	0.2							H3f3a	4E-12	0.2	Upf1	2E-01	0.1
												Pex11g	5E-01	0.1	Yod1	6E-02	0.2							Gga3	6E-02	0.2	Daglb	3E-02	0.1
												Zpr1	4E-01	0.1	Bicd1	7E-02	0.2							Lmo1	4E-02	0.2	Clk2	3E-01	0.1
												Clec2d	3E-02	0.1	Vcl	1E-07	0.2							Prdx4	3E-06	0.2	Cinp	7E-01	0.1
												Smad2	7E-03	0.1	Aph1b	7E-02	0.2							Rasa1	7E-03	0.2	Tmsb15b2	9E-01	0.1
												Taf3	2E-03	0.1	U2surp	8E-08	0.2							Cmip	5E-02	0.2	Kctd2	1E-01	0.1
												Nfib	2E-08	0.1	2410018L13Rik	4E-01	0.2							Lpcat3	6E-05	0.2	Rab8a	6E-02	0.1
												Tm9sf1	6E-03	0.1	Tmem14c	2E-09	0.2							Cpne1	7E-03	0.2	Rnf113a2	1E+00	0.1
												Ssc5d	7E-03	0.1	Brd3	2E-03	0.2							Selenom	1E-06	0.2	Pwwp3a	1E-01	0.1
												Ubr1	7E-02	0.1	D130040H23Rik	3E-01	0.2							Fam120b	3E-02	0.2	Ift27	2E-02	0.1
												Vta1	3E-03	0.1	Usp7	2E-04	0.2							Csnk1d	2E-01	0.2	Leprot	1E-03	0.1
												Zfp2	2E-01	0.1	Fuca1	1E-08	0.2							Ist1	3E-02	0.2	Lcmt1	8E-02	0.1
												Bud13	7E-02	0.1	Ahnak	1E-17	0.2							Hnrnph2	2E-02	0.2	Ddhd1	2E-01	0.1
												Ethe1	1E-02	0.1	Gm19705	4E-02	0.2							Fbxo17	1E-01	0.2	Fbxl19	3E-01	0.1
												Ppp2r1b	2E-01	0.1	2310009B15Rik	2E-06	0.2							Elac2	6E-01	0.2	Lrrc17	1E-01	0.1
												Cpne3	1E-02	0.1	Trim23	3E-02	0.2							Zbed4	1E+00	0.2	Ergic1	9E-02	0.1
												Atp5g3	8E-06	0.1	Zfp275	9E-03	0.2							Tab3	1E-01	0.2	Rassf3	4E-01	0.1
												Ncbp1	2E-02	0.1	Ptcd3	2E-03	0.2							Far1	2E-03	0.2	Rngtt	3E-01	0.1
												Tbcb	3E-05	0.1	Clk2	2E-01	0.2							Pomgnt1	6E-01	0.2	Pvt1	3E-03	0.1
												Usp20	2E-02	0.1	Oaz1	1E-21	0.2							Usp31	4E-01	0.2	Ago2	8E-04	0.1
												Gtf3a	9E-03	0.1	Cdc34	9E-03	0.2							Tmed4	3E-02	0.2	Mtrf1l	2E-01	0.1
												Sec22a	7E-03	0.1	Tsc1	2E-01	0.2							Ppp3cb	4E-02	0.2	Gpr176	7E-01	0.1
												Frmd6	5E-05	0.1	Lin7c	1E-03	0.2							Micu2	4E-03	0.2	Ddx47	1E-01	0.1
												Pelp1	4E-02	0.1	Tcaf1	3E-03	0.2							Vma21	5E-03	0.2	Gatb	1E-01	0.1
												Abracl	2E-04	0.1	Kdm4c	6E-04	0.2							Coro7	5E-01	0.2	Tceanc2	4E-01	0.1
												Oard1	3E-02	0.1	Zfpm2	2E-07	0.2							Pigh	2E-01	0.2	mt-Nd2	5E-08	0.1
												Slc25a40	1E-02	0.1	Btbd10	2E-03	0.2							Nisch	5E-07	0.2	Sco2	2E-01	0.1
												Cox17	4E-03	0.1	Vps50	9E-03	0.2							Ttc27	4E-01	0.2	Tmem29	1E-02	0.1
												Efna4	3E-01	0.1	Ywhaq	1E-09	0.2							Amotl1	2E-04	0.2	Fbxl5	1E-01	0.1
												Tardbp	5E-03	0.1	Mapkap1	4E-04	0.2							Gon4l	4E-02	0.2	Ift140	1E+00	0.1
												Bola3	2E-05	0.1	Calm1	7E-09	0.2							Zfp398	3E-01	0.2	AW549877	6E-01	0.1
												Hoxa9	7E-05	0.1	Socs4	4E-03	0.2							Clec16a	2E-01	0.2	Snapc3	1E-01	0.1
												Kctd11	2E-01	0.1	Ltbp3	3E-09	0.2							Adck5	9E-01	0.2	Arpc5	3E-05	0.1
												Cbl	3E-02	0.1	Ssb	2E-11	0.2							Clock	7E-04	0.2	Smarcad1	1E-02	0.1
												Wipf2	1E-01	0.1	Pes1	3E-01	0.2							Psme1	5E-03	0.2	Fbxw7	8E-02	0.1
												Bod1l	2E-03	0.1	Lrrc59	3E-04	0.2							Xpo4	8E-03	0.2	Ufm1	2E-02	0.1
												Nmd3	5E-02	0.1	Dxo	3E-03	0.2							Trnt1	3E-01	0.2	Dusp7	3E-01	0.1

Limb Mesenchyme					
Control			*Notch2*$^{tm1.1Ecan}$		
Gene	*p*	FC	Gene	*p*	FC

Chondrogenic					
Control			*Notch2*$^{tm1.1Ecan}$		
Gene	*p*	FC	Gene	*p*	FC

Fibroblast					
Control			*Notch2*$^{tm1.1Ecan}$		
Gene	*p*	FC	Gene	*p*	FC
Fam102b	4E-03	0.1	Ppp1r2	2E-05	0.2
Aff4	5E-04	0.1	Ykt6	7E-02	0.2
Gabpb1	1E-01	0.1	2810433D01Rik	1E-01	0.2
Gnasas1	2E-03	0.1	Pcyox1	3E-02	0.2
Tada3	3E-02	0.1	Ciao3	7E-02	0.2
Camsap1	1E-01	0.1	Glul	1E-01	0.2
Nufip1	1E-02	0.1	Chid1	2E-03	0.2
Klhl15	3E-02	0.1	Apip	6E-04	0.2
Kmt5a	2E-02	0.1	Ormdl2	9E-04	0.2
Ccdc6	6E-03	0.1	Pfn1	1E-07	0.2
Utrn	7E-07	0.1	Tug1	2E-02	0.2
Ptges3l	5E-01	0.1	Gm13470	1E-01	0.2
Mast4	2E-01	0.1	Dclre1c	4E-02	0.2
Fcho2	7E-04	0.1	Timm50	2E-03	0.2
Hnrnpul1	5E-03	0.1	Mief1	3E-01	0.2
Ing3	1E-01	0.1	Klf10	1E-02	0.2
Hnrnpl	1E-04	0.1	Kctd17	3E-03	0.2
Ccdc124	1E-03	0.1	Sclt1	2E-02	0.2
Banf1	5E-03	0.1	Usp50	2E-03	0.2
Hsf1	1E-01	0.1	Gart	2E-02	0.2
Mlycd	1E-01	0.1	Pgghg	2E-01	0.2
Rrp36	3E-01	0.1	Smg8	6E-03	0.2
Eaf2	7E-02	0.1	Stx12	2E-05	0.2
Eef1akmt1	6E-02	0.1	Mta1	2E-04	0.2
Tmem219	5E-02	0.1	Prpf39	3E-04	0.2
Thada	1E-02	0.1	Jph1	1E-02	0.2
Ap2s1	2E-05	0.1	Arhgap35	1E-04	0.2
Eif2a	3E-03	0.1	Alg6	5E-02	0.2
Car9	1E-01	0.1	Furin	5E-03	0.2
St3gal4	1E-03	0.1	Huwe1	2E-07	0.2
Pacs1	7E-02	0.1	Banf1	9E-06	0.2
Chic1	1E-02	0.1	Hccs	2E-03	0.2
Wwox	4E-04	0.1	Fam216a	2E-02	0.2
Prps1	1E-01	0.1	Fggy	2E-02	0.2
Mfsd11	6E-03	0.1	Six1	8E-03	0.2
Elovl1	2E-03	0.1	Rbm28	3E-04	0.2
Kin	2E-01	0.1	Btbd9	3E-05	0.2
Actl6a	1E-02	0.1	Myo1c	2E-04	0.2
Ercc6l2	8E-03	0.1	Utp3	8E-08	0.2
Myo1d	3E-08	0.1	Fam149a	7E-02	0.2
Heca	1E-01	0.1	Tmem245	5E-04	0.2
Fez2	2E-01	0.1	Hipk1	2E-02	0.2
Rbm25	2E-08	0.1	Ints9	8E-02	0.2
Cnot3	9E-03	0.1	Grasp	3E-01	0.2
Ccnk	1E-02	0.1	Arhgdia	4E-06	0.2
Nceh1	2E-02	0.1	Zfp617	6E-02	0.2
Rbpms	7E-02	0.1	Braf	3E-06	0.2
Wapl	9E-05	0.1	Slc35a4	1E-03	0.2
Lztr1	6E-01	0.1	Wdr6	9E-04	0.2
Fbxw8	2E-02	0.1	Srsf10	5E-06	0.2
Nol11	9E-04	0.1	Pkn2	2E-06	0.2
C4b	9E-01	0.1	Zranb1	7E-04	0.2
Cds2	8E-01	0.1	Fut8	1E-02	0.2
Ubap2	6E-06	0.1	Nckap1	5E-06	0.2
D10Wsu102e	9E-02	0.1	Purb	3E-05	0.2
Ktn1	4E-04	0.1	Slc39a6	2E-01	0.2
Lypla1	5E-01	0.1	Cbfa2t2	6E-04	0.2
Pdia6	5E-06	0.1	Stx3	3E-01	0.2
Rbm19	6E-02	0.1	Nudt14	4E-02	0.2
Nxpe4	3E-02	0.1	Pkm	7E-14	0.2
Capza1	2E-03	0.1	Socs6	6E-02	0.2
Nt5m	3E-01	0.1	Cttnbp2nl	6E-01	0.2
H2afy	6E-03	0.1	Hcfc2	3E-02	0.2
Cmtr1	6E-02	0.1	Fzd2	2E-05	0.2

Undefined					
Control			*Notch2*$^{tm1.1Ecan}$		
Gene	*p*	FC	Gene	*p*	FC

Articular/Synovial Fibroblast					
Control			*Notch2*$^{tm1.1Ecan}$		
Gene	*p*	FC	Gene	*p*	FC
Pdcd2l	1E-01	0.2	Ube4a	1E-02	0.1
Acot9	2E-02	0.2	Pigu	6E-01	0.1
Rbfa	5E-02	0.2	Tmem160	8E-05	0.1
Mrc2	2E-04	0.2	Zfp780b	5E-01	0.1
Vcpip1	4E-01	0.2	Bbs9	7E-02	0.1
Lmf2	2E-01	0.2	Fam120b	1E-01	0.1
Ftl1	1E-24	0.2	Crot	1E-01	0.1
Pkd1	1E-02	0.1	Glb1	1E-02	0.1
Nup62	2E-01	0.1	Cnot4	1E-03	0.1
Phip	3E-04	0.1	Sugp2	2E-01	0.1
Mlh3	6E-01	0.1	Tnfaip1	2E-01	0.1
Atf2	4E-03	0.1	2610307P16Rik	2E-01	0.1
Slc38a10	1E-03	0.1	Jam3	1E-01	0.1
Snx12	5E-02	0.1	Bri3bp	2E-02	0.1
Hcfc2	2E-01	0.1	8030462N17Rik	6E-02	0.1
Slc4a1ap	3E-01	0.1	Sdcbp	2E-04	0.1
Cd46	5E-01	0.1	1110059E24Rik	4E-01	0.1
Cfap410	8E-01	0.1	Tstd2	3E-01	0.1
Adk	5E-05	0.1	Zfp318	2E-01	0.1
Soga1	2E-02	0.1	Atp5e	3E-10	0.1
Serpine1	3E-04	0.1	Cnnm2	9E-02	0.1
P4ha2	1E-02	0.1	Rheb	7E-04	0.1
Cops5	9E-03	0.1	Echdc1	5E-02	0.1
Tmem9	1E-01	0.1	Crlf1	7E-03	0.1
Sept11	2E-07	0.1	Ift22	1E-02	0.1
Ppp6r2	4E-02	0.1	Zfp746	3E-01	0.1
Ctdsp1	4E-02	0.1	Osbpl11	6E-02	0.1
Gm11084	3E-02	0.1	AU041133	1E+00	0.1
Tmem106c	5E-01	0.1	Bcl10	1E-01	0.1
Cul9	3E-01	0.1	Tgs1	6E-01	0.1
Rpe	4E-01	0.1	Atic	8E-02	0.1
Smarca2	6E-06	0.1	Opa1	1E-01	0.1
Npat	2E-01	0.1	Hoxc9	3E-01	0.1
Asph	2E-02	0.1	BC052040	9E-01	0.1
Rbfox2	3E-05	0.1	Chmp4b	2E-03	0.1
Chmp3	4E-04	0.1	Kdm6a	6E-02	0.1
Nup205	3E-01	0.1	Heatr6	7E-01	0.1
Chm	5E-03	0.1	Fbxl4	2E-01	0.1
Irak1	4E-02	0.1	Ubald1	2E-01	0.1
Tspan9	8E-02	0.1	Osbpl8	6E-02	0.1
Pip5k1a	9E-02	0.1	Nr2c2ap	9E-01	0.1
Elk1	3E-01	0.1	Nipsnap2	1E-01	0.1
Phkb	3E-02	0.1	Anapc13	1E-03	0.1
Eda	5E-01	0.1	E2f5	3E-01	0.1
Tgs1	2E-02	0.1	Slc44a2	5E-03	0.1
Stx16	3E-02	0.1	Marf1	6E-03	0.1
Fam122a	2E-02	0.1	mt-Nd4	4E-07	0.1
Usp15	7E-04	0.1	Dpy19l4	2E-01	0.1
Cpsf2	4E-01	0.1	Trafd1	9E-02	0.1
mt-Cytb	8E-20	0.1	Rexo4	6E-01	0.1
Cep350	6E-03	0.1	Sirt7	2E-01	0.1
Syngap1	2E-01	0.1	Rasa4	4E-01	0.1
Ptprf	5E-01	0.1	Ppp4r1	7E-02	0.1
Anxa6	1E-02	0.1	Phc3	8E-02	0.1
Ppp2r5e	2E-02	0.1	Tmem87a	1E-01	0.1
Mettl15	8E-01	0.1	Gpatch2	9E-03	0.1
Bcor	8E-03	0.1	Stx17	1E-01	0.1
Cd82	7E-01	0.1	Actr3	5E-04	0.1
Lrrc57	5E-01	0.1	Dvl2	3E-01	0.1
Man2a2	1E-01	0.1	6430590A07Rik	4E-01	0.1
Ctnnbl1	3E-01	0.1	Tmtc3	2E-01	0.1
Cspg4	3E-02	0.1	Dock4	8E-01	0.1
Acot13	7E-04	0.1	Nat9	1E+00	0.1
Cyhr1	7E-03	0.1	Mbd5	8E-03	0.1

Limb Mesenchyme

Control			*Notch2* $^{tm1.1Ecan}$		
Gene	*p*	FC	Gene	*p*	FC

Chondrogenic

Control			*Notch2* $^{tm1.1Ecan}$		
Gene	*p*	FC	Gene	*p*	FC

Fibroblast

Control			*Notch2* $^{tm1.1Ecan}$		
Gene	*p*	FC	Gene	*p*	FC
Lpar4	4E-02	0.1	Ctbp1	1E-04	0.2
Faf1	4E-05	0.1	Nsmf	1E-01	0.2
Utp25	8E-02	0.1	Casc3	1E-01	0.2
Ift52	2E-01	0.1	Tle4	1E-02	0.2
Cdsn	2E-01	0.1	Nqo2	1E-01	0.2
Ncoa5	2E-02	0.1	Tax1bp1	8E-09	0.2
Adck5	4E-02	0.1	Prrc2b	1E-05	0.2
Cpped1	2E-01	0.1	Cfl2	7E-08	0.2
Ist1	1E-02	0.1	Prkd3	2E-03	0.2
Lta4h	6E-03	0.1	Npepl1	6E-03	0.2
Ttf1	9E-02	0.1	Ap3s2	2E-01	0.2
Sypl	2E-03	0.1	Trappc5	3E-02	0.2
1810026B05Rik	8E-05	0.1	Hoxa5	4E-02	0.2
Srsf7	5E-06	0.1	Gna13	5E-04	0.2
Efcab14	3E-02	0.1	Cog1	4E-03	0.2
Zfp398	8E-02	0.1	Hk1	8E-05	0.2
Pcx	1E-03	0.1	Hivep1	1E-01	0.2
Zfp944	1E-01	0.1	Rap2a	3E-03	0.2
Glt8d2	4E-02	0.1	Sos1	2E-03	0.2
Hnrnph1	4E-04	0.1	Tut4	2E-04	0.2
Acat1	1E-04	0.1	Eif2ak1	2E-01	0.2
Vcp	2E-07	0.1	Ttll4	9E-03	0.2
Tmem176a	6E-04	0.1	Mzt1	5E-05	0.2
Capzb	2E-04	0.1	Tmem80	1E-02	0.2
Ccn3	3E-02	0.1	Rps6ka5	2E-03	0.2
Lhfpl2	4E-01	0.1	S100pbp	9E-02	0.2
Zkscan1	1E-01	0.1	Adal	3E-01	0.2
Mat2a	3E-03	0.1	Zfp91	2E-08	0.2
Ik	2E-04	0.1	Dennd5a	1E-04	0.2
Micos10	6E-07	0.1	Nudt5	4E-04	0.2
Ndnf	3E-02	0.1	Cnot3	1E-03	0.2
Atp5g2	9E-09	0.1	Aifm1	2E-04	0.2
Pip5k1c	2E-02	0.1	Cpsf7	1E-03	0.2
Cant1	5E-01	0.1	Hint1	2E-10	0.2
Pon2	8E-03	0.1	Plxdc2	1E-07	0.2
Tmem117	4E-02	0.1	2210408I21Rik	1E-03	0.2
Usp24	1E-03	0.1	Cep78	7E-02	0.2
Cep68	7E-02	0.1	Hif1a	5E-08	0.2
Zfp91	3E-04	0.1	Hspa4	8E-08	0.2
Cox6c	3E-06	0.1	Snapin	4E-03	0.2
Anapc1	9E-02	0.1	Foxc1	6E-07	0.2
D330023K18Rik	9E-01	0.1	Ammecr1	3E-03	0.2
Rit1	2E-02	0.1	Tada3	4E-03	0.2
Shisa5	1E-02	0.1	Mcts2	2E-02	0.2
Chmp6	3E-01	0.1	Pex11a	5E-02	0.2
Zfp60	6E-01	0.1	Hexim1	1E-02	0.2
Nptn	6E-06	0.1	Ammecr1l	3E-02	0.2
Gpalpp1	1E-01	0.1	Matr3	6E-06	0.2
Aqr	1E-02	0.1	Cap1	2E-04	0.2
Chtop	7E-02	0.1	Nf1	5E-05	0.2
Eif4g3	2E-04	0.1	Galnt1	7E-04	0.2
Apc	4E-02	0.1	Vopp1	1E-02	0.2
Adpgk	2E-01	0.1	Fip1l1	5E-04	0.2
Cbfb	1E-03	0.1	Zfp654	9E-03	0.2
Ddx10	9E-02	0.1	Pcx	4E-04	0.2
Tmem41b	2E-01	0.1	Dync1i2	2E-08	0.2
Zfp64	6E-03	0.1	Ankrd54	2E-01	0.2
Ccdc114	2E-01	0.1	Dusp3	8E-02	0.2
Ptpa	3E-04	0.1	Prpf40a	2E-09	0.2
Btrc	8E-03	0.1	Chrdl1	2E-01	0.2
Cbll1	7E-02	0.1	Zbtb21	1E-01	0.2
Mier1	1E-03	0.1	Rnf40	6E-02	0.2
Pip4k2b	5E-01	0.1	Pgm2	6E-04	0.2
Pdcd5	7E-05	0.1	Rock2	5E-09	0.2

Undefined

Control			*Notch2* $^{tm1.1Ecan}$		
Gene	*p*	FC	Gene	*p*	FC

Articular/Synovial Fibroblast

Control			*Notch2* $^{tm1.1Ecan}$		
Gene	*p*	FC	Gene	*p*	FC
Atp11a	4E-01	0.1	Fkrp	2E-01	0.1
Rnf227	3E-01	0.1	Sdha	4E-03	0.1
Ankfy1	5E-03	0.1	Aplp1	7E-01	0.1
Phactr2	1E-02	0.1	Creb3	2E-01	0.1
Egr1	1E-07	0.1	Ptpn12	5E-02	0.1
Rab3a	7E-01	0.1	Ppm1a	8E-03	0.1
Rnpc3	3E-01	0.1	Gramd1a	7E-01	0.1
Tmem160	2E-04	0.1	Zfp277	5E-02	0.1
Endov	4E-01	0.1	Pgm2	4E-01	0.1
Nckap5l	1E-01	0.1	Ube2q1	5E-02	0.1
Commd8	2E-03	0.1	Gatad2b	5E-03	0.1
Rsrp1	2E-05	0.1	Cherp	6E-01	0.1
Foxo3	6E-02	0.1	Gm4924	4E-01	0.1
Crbn	1E-01	0.1	Thap2	4E-01	0.1
Retsat	3E-01	0.1	Cntrl	4E-01	0.1
Calu	2E-06	0.1	Gga1	1E-01	0.1
Zfp654	1E-01	0.1	Zfp326	9E-02	0.1
Abhd2	7E-02	0.1	Napg	2E-01	0.1
Slc10a3	5E-01	0.1	Smad4	7E-02	0.1
Tor1b	2E-02	0.1	Smarcal1	4E-01	0.1
Tbx4	2E-02	0.1	Got2	1E-01	0.1
Cherp	4E-01	0.1	Ccdc66	3E-01	0.1
Zfp563	1E-01	0.1	Ryk	1E-02	0.1
Arhgdia	5E-05	0.1	Ypel5	2E-01	0.1
Strn3	3E-04	0.1	Styx	4E-01	0.1
Tet3	1E-02	0.1	Tmem106c	9E-02	0.1
Vti1b	7E-04	0.1	Arhgef11	3E-01	0.1
Sec24c	1E-01	0.1	Slc25a51	5E-02	0.1
Mrpl33	4E-06	0.1	Ints7	3E-01	0.1
Pip4k2a	1E-01	0.1	Iqsec2	3E-01	0.1
Spred2	2E-01	0.1	Mysm1	3E-03	0.1
Gng7	2E-01	0.1	Golga4	4E-04	0.1
Pacs1	4E-02	0.1	Ilk	9E-02	0.1
Mrps18a	1E-02	0.1	Adnp2	2E-01	0.1
Gfra4	4E-01	0.1	Gnpat	1E+00	0.1
Nynrin	6E-02	0.1	Pdha1	3E-05	0.1
Cntln	2E-01	0.1	Dhx35	7E-01	0.1
Gm4924	4E-01	0.1	Armcx5	7E-01	0.1
Ahcyl1	6E-05	0.1	Cap2	5E-01	0.1
Lyn	2E-02	0.1	Rab1a	2E-02	0.1
Wdr59	7E-01	0.1	4833420G17Rik	4E-01	0.1
Trappc13	2E-01	0.1	Irf2bpl	9E-02	0.1
Rom1	4E-01	0.1	Ppp2r3d	9E-01	0.1
Alkbh5	9E-03	0.1	Zrsr1	7E-01	0.1
Trmt10b	2E-01	0.1	Strn3	6E-03	0.1
Nipsnap2	1E-02	0.1	Alkbh5	3E-02	0.1
Gm2000	1E-02	0.1	Snapin	1E-02	0.1
Gfpt2	4E-01	0.1	Wdr37	3E-02	0.1
Tmem131	5E-02	0.1	Ftl1-ps1	1E-01	0.1
Zfp866	5E-01	0.1	Agl	6E-02	0.1
Ccdc6	2E-01	0.1	Sclt1	1E-01	0.1
Taok2	5E-01	0.1	Mpdu1	7E-01	0.1
Scrn3	4E-01	0.1	Mov10	4E-01	0.1
Mar2	1E-03	0.1	Vps26c	8E-01	0.1
Cog5	1E-02	0.1	Igfbp7	4E-04	0.1
Atp5e	5E-10	0.1	Cog3	3E-01	0.1
Sp2	3E-01	0.1	Tbc1d9b	2E-02	0.1
Ghr	7E-05	0.1	Btbd8	6E-01	0.1
Fam193b	7E-01	0.1	Drosha	4E-01	0.1
Zcchc10	4E-01	0.1	Zfp110	5E-01	0.1
Gtf2a1	7E-01	0.1	Zfp142	6E-01	0.1
Hdac1	4E-02	0.1	Trir	6E-03	0.1
Pkd2	1E-03	0.1	Emc9	1E-01	0.1
Prrg1	4E-02	0.1	Pogk	3E-01	0.1

Limb Mesenchyme						Chondrogenic					
Control			*Notch2*[tm1.1Ecan]			Control			*Notch2*[tm1.1Ecan]		
Gene	*p*	FC	Gene	*p*	FC	Gene	*p*	FC	Gene	*p*	FC

Fibroblast					
Control			*Notch2*[tm1.1Ecan]		
Gene	*p*	FC	Gene	*p*	FC
Snrnp200	2E-02	0.1	Pros1	3E-03	0.2
Mill2	3E-03	0.1	Tmem260	5E-04	0.2
Eml4	1E-02	0.1	Pqbp1	3E-01	0.2
Ywhaq	8E-05	0.1	Narf	2E-02	0.2
Sdhaf4	1E-02	0.1	Nampt	4E-06	0.2
Ddx6	7E-07	0.1	Ryk	4E-07	0.2
Cdip1	7E-03	0.1	Utp15	2E-02	0.2
Jdp2	8E-03	0.1	Cul3	2E-05	0.2
Rlf	1E-03	0.1	Spats2	8E-03	0.2
Dync2h1	3E-03	0.1	Ccar2	2E-01	0.2
Tmem45a	1E-02	0.1	Poldip2	4E-02	0.2
Klhdc2	4E-03	0.1	Dph3	2E-05	0.2
Scfd2	4E-02	0.1	Vps35l	7E-06	0.2
Mysm1	2E-02	0.1	Tesk2	9E-02	0.2
Pmpcb	1E-01	0.1	Rnf111	2E-04	0.2
Zfp521	9E-06	0.1	Golga1	2E-03	0.2
2310033P09Rik	3E-01	0.1	Ptpn21	2E-03	0.2
Zfp282	8E-02	0.1	Tcf3	2E-04	0.2
Snip1	1E-01	0.1	Yeats4	5E-05	0.2
Map2k1	3E-02	0.1	Brdt	9E-02	0.2
Mar2	8E-03	0.1	Cavin2	6E-02	0.2
6720427I07Rik	1E-01	0.1	Pi4ka	2E-02	0.2
Plekhm2	1E-02	0.1	Sox6	3E-08	0.2
Urm1	5E-02	0.1	Ap2b1	3E-05	0.2
Rsrc2	3E-04	0.1	Nmt2	6E-03	0.2
Hist3h2a	7E-01	0.1	Pnn	1E-05	0.2
Cuedc1	6E-02	0.1	Safb	4E-03	0.2
Tex261	5E-04	0.1	Rnf25	8E-02	0.2
Cox7a2	1E-08	0.1	Ube4a	1E-02	0.2
Dph5	2E-01	0.1	Lrwd1	2E-04	0.2
Tbk1	2E-02	0.1	Cox6c	9E-15	0.2
Slc38a9	3E-02	0.1	Lcn2	4E-03	0.2
4933407K13Rik	4E-01	0.1	Noc4l	2E-03	0.2
Gon4l	9E-04	0.1	Ccdc32	8E-02	0.2
Mtch2	5E-05	0.1	Dtnb	2E-02	0.2
Nol7	4E-05	0.1	Tmem53	5E-03	0.2
Commd10	3E-02	0.1	Pus10	3E-02	0.2
Tead1	5E-06	0.1	Safb2	2E-03	0.2
St7	2E-03	0.1	Spopl	6E-04	0.2
Nt5dc3	2E-02	0.1	Med25	1E-04	0.2
Chd4	8E-07	0.1	Snrpg	1E-13	0.2
Cdc5l	2E-03	0.1	Myl6	2E-14	0.2
Cep126	3E-01	0.1	Gabpa	1E-01	0.2
Meis3	5E-01	0.1	Exosc9	2E-02	0.2
Snapin	1E-02	0.1	Gtf2h2	4E-02	0.2
Crnkl1	7E-03	0.1	Psmd13	3E-05	0.2
Socs6	6E-02	0.1	B4gat1	4E-03	0.2
Rnf7	6E-05	0.1	Eif2ak4	7E-03	0.2
Atp6v1h	1E-02	0.1	Set	5E-08	0.2
Snupn	2E-01	0.1	Dpy30	8E-04	0.2
Prpf38b	1E-03	0.1	Lysmd3	4E-01	0.2
Zfp322a	1E-02	0.1	Tmem87a	1E-02	0.2
Gnl1	6E-02	0.1	1110046J04Rik	1E-02	0.2
Ube2m	3E-05	0.1	Gan	1E-02	0.2
Sdhaf3	3E-01	0.1	Trim33	2E-03	0.2
Dctn1	3E-04	0.1	Ciz1	1E-04	0.2
Cfap97	1E-01	0.1	Dnase2a	6E-04	0.2
Loxl2	2E-01	0.1	Ilrun	2E-03	0.2
Tcf20	7E-04	0.1	Kat5	5E-02	0.2
2700062C07Rik	2E-01	0.1	Top1mt	3E-01	0.2
Trappc5	3E-02	0.1	Schip1	3E-05	0.2
Tnip2	3E-01	0.1	Vps18	4E-02	0.2
Ppp4c	1E-03	0.1	Omd	1E-09	0.2
Il1r1	4E-09	0.1	Atf1	1E-03	0.2

Undefined					
Control			*Notch2*[tm1.1Ecan]		
Gene	*p*	FC	Gene	*p*	FC

Articular/Synovial Fibroblast					
Control			*Notch2*[tm1.1Ecan]		
Gene	*p*	FC	Gene	*p*	FC
Pou6f1	8E-02	0.1	Usp36	4E-01	0.1
Nme7	1E-01	0.1	B4galt6	2E-01	0.1
Txndc9	5E-03	0.1	Fbxl12	4E-01	0.1
Ep400	3E-02	0.1	Timm29	6E-01	0.1
Dusp7	5E-02	0.1	Pdcd6ip	2E-02	0.1
Sdha	4E-03	0.1	Fam174a	2E-01	0.1
Mcph1	2E-01	0.1	Pqbp1	8E-02	0.1
Atp2a2	3E-02	0.1	Slc35b4	5E-01	0.1
Pus1	2E-01	0.1	Cttn	3E-02	0.1
Pcnx3	4E-01	0.1	Nf1	8E-04	0.1
Ncdn	4E-01	0.1	4931406C07Rik	8E-02	0.1
Mau2	4E-01	0.1	Kdm2a	5E-04	0.1
Mxd1	3E-01	0.1	Tmem151a	8E-01	0.1
Sipa1	4E-01	0.1	5730455P16Rik	2E-01	0.1
Crebrf	3E-04	0.1	Gm4285	2E-01	0.1
Fancm	4E-01	0.1	Aebp2	3E-01	0.1
Ikzf5	3E-01	0.1	Ndufaf1	2E-01	0.1
D230025D16Rik	4E-01	0.1	Arid1a	3E-01	0.1
Sp3	3E-03	0.1	Edrf1	5E-01	0.1
Tmbim6	4E-04	0.1	Zfp120	3E-01	0.1
Capza2	2E-04	0.1	Gnptg	2E-01	0.1
Gm14325	3E-01	0.1	Gng12	8E-03	0.1
Gm4221	8E-01	0.1	Zfp518b	8E-01	0.1
Mysm1	1E-02	0.1	Cul4a	1E-01	0.1
Ercc4	1E-01	0.1	Pcif1	6E-01	0.1
Flywch1	3E-02	0.1	Topors	2E-01	0.1
Tax1bp3	9E-03	0.1	Cbll1	1E+00	0.1
Atp11b	1E-01	0.1	Mlh1	8E-01	0.1
Prkce	1E-01	0.1	Ireb2	7E-02	0.1
2610008E11Rik	5E-02	0.1	Fam160a2	8E-01	0.1
Sec14l1	2E-03	0.1	Tlcd1	1E-01	0.1
Clasp1	3E-02	0.1	Creb1	1E-01	0.1
Vps11	1E-01	0.1	Cox6b2	5E-01	0.1
mt-Nd2	7E-11	0.1	Sirt1	1E-01	0.1
Ralgapa1	8E-03	0.1	Ube2d1	6E-01	0.1
Atp13a3	6E-02	0.1	Kpna4	6E-03	0.1
Prickle2	1E-02	0.1	Rfx7	2E-02	0.1
Msrb1	5E-03	0.1	Mtg2	6E-01	0.1
Ap3d1	1E-02	0.1	Lrrc42	1E-02	0.1
Map2k4	1E-02	0.1	Tifa	1E-01	0.1
Fam102a	5E-01	0.1	Gnai2	2E-06	0.1
Arf3	1E-02	0.1	Lrba	1E-02	0.1
Celf1	3E-03	0.1	Dmac2	8E-01	0.1
Tmem135	1E-03	0.1	Tkt	2E-02	0.1
Snapc5	1E-02	0.1	Mmaa	2E-01	0.1
Ikzf2	9E-01	0.1	Habp4	2E-01	0.1
Cd47	7E-05	0.1	Utrn	3E-03	0.1
Rab8a	3E-01	0.1	Ubr1	1E-01	0.1
Ubac2	1E-02	0.1	R74862	4E-01	0.1
Eif4b	1E-03	0.1	Clic4	5E-03	0.1
Cept1	2E-01	0.1	Kras	2E-02	0.1
Acadvl	1E-01	0.1	Manea	5E-01	0.1
Greb1l	2E-01	0.1	Kri1	6E-01	0.1
Gzf1	9E-02	0.1	Tbccd1	7E-01	0.1
C87436	4E-01	0.1	Gfm2	1E+00	0.1
Blvra	3E-02	0.1	Churc1	1E-02	0.1
Usp7	6E-02	0.1	Axin1	2E-02	0.1
Kdm2a	3E-03	0.1	Mospd1	2E-01	0.1
Sfxn5	4E-01	0.1	Rab7	1E-03	0.1
Rc3h1	1E-02	0.1	Acp2	1E-01	0.1
Pyroxd1	9E-01	0.1	Txndc15	7E-03	0.1
Camk2g	1E-01	0.1	Slc35e2	9E-01	0.1
Nsd3	2E-03	0.1	Ccdc43	2E-01	0.1
Pik3ca	9E-03	0.1	Ube3b	4E-01	0.1

Limb Mesenchyme						Chondrogenic					
Control			Notch2 [tm1.1Ecan]			Control			Notch2 [tm1.1Ecan]		
Gene	*p*	FC	Gene	*p*	FC	Gene	*p*	FC	Gene	*p*	FC

Fibroblast					
Control			Notch2 [tm1.1Ecan]		
Gene	*p*	FC	Gene	*p*	FC
Vopp1	1E-02	0.1	Clmp	5E-03	0.2
Bcl2l13	4E-02	0.1	Snx9	2E-08	0.2
Prdm16	6E-03	0.1	Zbtb7b	9E-02	0.2
Rsf1	1E-02	0.1	Arfgef2	4E-02	0.2
Yif1b	2E-04	0.1	Ddx6	2E-07	0.2
Safb2	3E-02	0.1	Arhgef18	2E-02	0.2
Sox12	9E-01	0.1	Wipf1	8E-02	0.2
Ccdc136	1E-01	0.1	Acat1	1E-05	0.2
Trmt11	2E-01	0.1	Ak2	1E-04	0.2
Kat2a	2E-01	0.1	Ulk2	7E-05	0.2
Anapc10	1E-02	0.1	Ppcdc	1E-01	0.2
Sdcbp	2E-04	0.1	D5Ertd579e	6E-04	0.2
Rab11b	6E-04	0.1	Topors	6E-07	0.2
Cplane1	5E-03	0.1	Smarcb1	2E-04	0.2
Tmem246	9E-03	0.1	Pdcd5	1E-06	0.2
Trafd1	3E-03	0.1	Tgs1	4E-03	0.2
Zfp512	2E-01	0.1	Mtch1	1E-09	0.2
Rabif	5E-01	0.1	Cpeb3	5E-01	0.2
Amn1	5E-02	0.1	Mtus2	7E-01	0.2
Acad8	6E-02	0.1	Gpx7	2E-02	0.2
Ifi27	1E-05	0.1	Snx2	3E-04	0.2
Actr3	2E-04	0.1	Exoc3	7E-06	0.2
Kmt2a	1E-04	0.1	Vps8	1E-01	0.2
Cdkn2aipnl	8E-02	0.1	Derl2	1E-03	0.2
Mrpl48	4E-04	0.1	Arl2bp	9E-05	0.2
Ddb1	2E-04	0.1	Angel2	2E-03	0.2
Yeats4	1E-02	0.1	Elmsan1	1E-03	0.2
Adam19	2E-01	0.1	Zfp932	8E-01	0.2
Slc35a5	3E-01	0.1	Pin1	1E-04	0.2
Map4k4	4E-03	0.1	AI597479	3E-01	0.2
Tspan17	2E-01	0.1	Snrnp70	3E-06	0.2
Tpd52l2	6E-03	0.1	Apex2	7E-03	0.2
Caprin1	7E-06	0.1	Mettl8	1E-02	0.2
Szt2	1E-01	0.1	Zfp207	7E-08	0.2
Pphln1	5E-02	0.1	Hacd3	9E-02	0.2
Hivep1	8E-01	0.1	Ehmt2	2E-03	0.2
Lactb	1E-01	0.1	Mar2	2E-04	0.2
Pdzd8	1E-01	0.1	Tmod2	6E-03	0.2
Rnpepl1	3E-01	0.1	Prrg2	2E-01	0.2
Nipbl	2E-05	0.1	Dctn1	3E-05	0.2
Dot1l	4E-02	0.1	Zfp408	3E-02	0.2
Myl12b	2E-03	0.1	Tmco4	2E-03	0.2
Kctd18	4E-01	0.1	Ei24	2E-03	0.2
Gmpr	4E-01	0.1	Mfap1a	1E-02	0.2
Tcf3	2E-02	0.1	Sema3c	1E-04	0.2
Pithd1	3E-01	0.1	Trappc10	1E-03	0.2
Lrch4	7E-02	0.1	Mmadhc	2E-03	0.2
Pex19	4E-01	0.1	Apc	4E-03	0.2
Mettl9	1E-03	0.1	Crk	2E-02	0.2
Abi1	5E-04	0.1	Nr3c1	7E-05	0.2
Hdac9	9E-02	0.1	Snapc3	8E-03	0.2
Ndufa11	9E-04	0.1	Dennd4c	2E-02	0.2
Smarcb1	6E-03	0.1	Dhx29	8E-03	0.2
Sec23b	5E-02	0.1	Hprt	1E-03	0.2
Tmem259	1E-01	0.1	Fra10ac1	9E-03	0.2
Glod4	1E-02	0.1	Zfp871	2E-03	0.2
Smdt1	1E-05	0.1	Cemip	7E-01	0.2
Tdrd7	2E-01	0.1	Zfp143	2E-01	0.2
Tcf4	9E-08	0.1	Cebpb	2E-03	0.2
Tmem234	5E-08	0.1	Rp9	6E-05	0.2
Ndufa5	7E-05	0.1	Mier1	3E-04	0.2
Herc2	9E-04	0.1	Tbc1d24	3E-01	0.2
Adam10	1E-02	0.1	Fcho2	1E-04	0.2
Med10	2E-02	0.1	Fundc2	3E-10	0.2

Undefined					
Control			Notch2 [tm1.1Ecan]		
Gene	*p*	FC	Gene	*p*	FC

Articular/Synovial Fibroblast					
Control			Notch2 [tm1.1Ecan]		
Gene	*p*	FC	Gene	*p*	FC
Epha4	4E-01	0.1	Cpt1c	2E-01	0.1
Zmat2	4E-04	0.1	Pttg1ip	2E-03	0.1
Mdk	6E-01	0.1	Fnbp4	8E-02	0.1
Nol6	4E-01	0.1	Ptpn9	1E-02	0.1
Dstn	3E-06	0.1	Clasp1	7E-03	0.1
Wdr44	5E-02	0.1	Bag5	4E-02	0.1
Phkg2	5E-01	0.1	Zfp64	2E-01	0.1
Zfp87	4E-01	0.1	Vti1b	2E-03	0.1
Cox6b1	3E-09	0.1	Tesk2	1E+00	0.1
Smim12	4E-01	0.1	Tbcd	2E-01	0.1
Ndufv3	7E-07	0.1	Eif2ak1	3E-01	0.1
Cdk6	1E+00	0.1	Nck1	4E-03	0.1
Dhrs9	4E-01	0.1	Bin3	3E-01	0.1
Vps33b	5E-01	0.1	Parp4	2E-01	0.1
Elmod2	3E-01	0.1	Ptbp2	1E-01	0.1
Lyrm2	1E-02	0.1	Actr1b	8E-01	0.1
Dyrk2	7E-01	0.1	Klhl24	4E-02	0.1
Tbc1d20	2E-02	0.1	Taok1	3E-02	0.1
Socs4	4E-02	0.1	Arid1b	3E-03	0.1
Cox19	8E-03	0.1	F8	2E-01	0.1
Dennd5a	1E-03	0.1	Zfp398	3E-01	0.1
Lsm10	8E-02	0.1	2210016L21Rik	1E-02	0.1
Pbx2	5E-01	0.1	Agps	4E-02	0.1
Ints2	7E-01	0.1	Med13	5E-02	0.1
Pop4	1E-01	0.1	Mcrs1	5E-02	0.1
Plpp2	7E-01	0.1	Dcaf5	1E-01	0.1
Churc1	2E-03	0.1	Nfil3	1E-01	0.1
Dync2li1	3E-01	0.1	Ap3b1	1E-03	0.1
Slc7a6os	8E-01	0.1	Etnk1	6E-02	0.1
Cd151	7E-02	0.1	Rnf5	8E-01	0.1
Cstf2t	8E-01	0.1	Tead2	2E-01	0.1
Timm29	6E-01	0.1	Nfe2l2	2E-02	0.1
Cript	8E-02	0.1	Egr1	3E-04	0.1
Dhrs4	6E-02	0.1	0610040B10Rik	9E-01	0.1
Mdc1	1E+00	0.1	Tent4a	4E-01	0.1
Stat3	2E-04	0.1	Rragb	6E-01	0.1
Sufu	2E-01	0.1	Hoxc4	2E-01	0.1
Prrx1	2E-05	0.1	Slc4a4	4E-02	0.1
Fam193a	5E-03	0.1	Prdm4	3E-01	0.1
mt-Co2	4E-17	0.1	Ids	1E-01	0.1
Ythdf1	1E-01	0.1	Samd14	3E-01	0.1
Rpap1	4E-01	0.1	C1galt1c1	1E+00	0.1
Agl	3E-01	0.1	Pfdn5	5E-06	0.1
Pskh1	3E-01	0.1	Tent4b	3E-01	0.1
Tmem67	2E-01	0.1	Stx16	3E-01	0.1
Ctsd	3E-06	0.1	Zscan26	5E-01	0.1
Gigyf2	4E-03	0.1	Lta4h	2E-01	0.1
Gas2l1	2E-01	0.1	Oscp1	4E-01	0.1
Manea	3E-01	0.1	Ubr2	4E-03	0.1
Rab12	2E-02	0.1	Oaz2	1E-02	0.1
Itfg1	6E-03	0.1	Rcor1	7E-03	0.1
Chtop	6E-02	0.1	Rbm19	6E-01	0.1
Usp38	1E-01	0.1	Synj1	2E-01	0.1
Washc4	5E-03	0.1	Slc38a1	6E-02	0.1
Ten1	3E-01	0.1	Ppia	6E-08	0.1
Mllt1	4E-01	0.1	Ccnl2	8E-02	0.1
Vps37c	3E-01	0.1	Tnip2	6E-01	0.1
Slc38a9	6E-02	0.1	Ino80b	1E+00	0.1
Ankrd54	4E-01	0.1	Bnip3l	7E-03	0.1
Ilk	2E-02	0.1	Smad2	2E-02	0.1
Lrif1	4E-01	0.1	Nckap5	4E-02	0.1
Csnk1g3	2E-03	0.1	Oxa1l	3E-01	0.1
Naa20	3E-03	0.1	Tnfrsf11b	9E-03	0.1
Fars2	9E-03	0.1	Zw10	6E-01	0.1

Limb Mesenchyme						Chondrogenic						Fibroblast						Undefined						Articular/Synovial Fibroblast					
Control			*Notch2*$^{tm1.1Ecan}$			Control			*Notch2*$^{tm1.1Ecan}$			Control			*Notch2*$^{tm1.1Ecan}$			Control			*Notch2*$^{tm1.1Ecan}$			Control			*Notch2*$^{tm1.1Ecan}$		
Gene	*p*	FC	Gene	*p*	FC	Gene	*p*	FC	Gene	*p*	FC	Gene	*p*	FC	Gene	*p*	FC	Gene	*p*	FC	Gene	*p*	FC	Gene	*p*	FC	Gene	*p*	FC
												Acin1	2E-04	0.1	Creld1	6E-02	0.2							Smad4	6E-02	0.1	Zbtb5	9E-01	0.1
												Tyk2	6E-02	0.1	Rab12	2E-04	0.2							H1f0	2E-03	0.1	Tusc3	1E-02	0.1
												Syt1	6E-02	0.1	Tab3	5E-01	0.2							Cenpb	9E-04	0.1	Dnal1	7E-01	0.1
												Ptpn4	1E-01	0.1	Zer1	1E-01	0.2							Hps4	2E-01	0.1	Taf7	4E-01	0.1
												Rer1	8E-04	0.1	Arl6ip4	3E-07	0.2							Rit1	1E-01	0.1	Vhl	3E-01	0.1
												Rap2b	3E-02	0.1	Ccdc124	2E-04	0.2							2510009E07Rik	1E-01	0.1	Usp54	4E-01	0.1
												Eefsec	3E-02	0.1	Wrap53	9E-04	0.2							Ube3b	7E-01	0.1	Scaf1	3E-01	0.1
												Sept5	5E-02	0.1	Mrpl19	8E-03	0.2							Ptpn1	2E-03	0.1	Agtpbp1	1E+00	0.1
												Eef2kmt	6E-02	0.1	Ppp1cb	4E-09	0.2							Exoc8	1E-01	0.1	Utp6	7E-02	0.1
												Npas2	4E-01	0.1	Msl3l2	8E-02	0.2							Crtc1	3E-01	0.1	Slc4a3	1E+00	0.1
												Fam229b	3E-02	0.1	Rnh1	3E-08	0.2							Zfp358	9E-01	0.1	Fam53a	7E-01	0.1
												Nek4	1E-01	0.1	Sox5	1E-06	0.2							Lrpprc	2E-01	0.1	Pank1	8E-01	0.1
												Cnot4	3E-04	0.1	Taok3	5E-04	0.2							Rnaseh2c	6E-02	0.1	Fas	3E-01	0.1
												Crcp	4E-01	0.1	Tbccd1	1E-01	0.2							Lamtor4	1E-04	0.1	Acadm	8E-04	0.1
												Wdfy3	4E-04	0.1	Nfkb1	1E-02	0.2							Dnajc17	6E-01	0.1	Dhdds	1E-01	0.1
												Il17ra	2E-02	0.1	Tnks1bp1	4E-04	0.2							Hnrnpul1	8E-03	0.1	Pja1	3E-01	0.1
												Megf8	2E-01	0.1	Rbpms	2E-01	0.1							Enpp5	2E-01	0.1	Fgfr1op	6E-01	0.1
												Atp9b	3E-03	0.1	Tmem203	8E-01	0.1							Zfp410	6E-01	0.1	Chpt1	2E-02	0.1
												Mpp5	1E-02	0.1	Kctd3	4E-02	0.1							Map1lc3a	3E-05	0.1	Gpx1	2E-04	0.1
												Apex2	7E-03	0.1	Ino80dos	2E-02	0.1							Zfp983	6E-01	0.1	Phf8	3E-01	0.1
												Ass1	6E-02	0.1	Pdcd6ip	4E-06	0.1							Tomm5	5E-05	0.1	mt-Co2	5E-08	0.1
												Trap1	4E-01	0.1	Sf1	8E-05	0.1							Nbea	1E-03	0.1	Cars2	7E-01	0.1
												Asf1a	2E-02	0.1	Gm10561	3E-01	0.1							Cd276	3E-01	0.1	Mettl5	8E-01	0.1
												Edf1	6E-07	0.1	Parp3	3E-04	0.1							Map4k5	2E-01	0.1	Commd2	2E-01	0.1
												Ube2l3	2E-05	0.1	Pi4k2b	6E-02	0.1							Chd9	3E-03	0.1	Zfp938	4E-01	0.1
												Polr2b	3E-02	0.1	Rab3il1	4E-03	0.1							Tmem154	4E-01	0.1	Atpif1	5E-05	0.1
												Snrnp35	5E-01	0.1	Uxs1	2E-05	0.1							Dnm2	3E-03	0.1	Vkorc1l1	7E-02	0.1
												Txndc12	1E-02	0.1	Pltp	1E-04	0.1							Vps35	4E-04	0.1	Zfp160	1E-01	0.1
												Bdp1	1E-03	0.1	Mrpl11	5E-02	0.1							Fam160b1	2E-01	0.1	Cyp4f17	4E-01	0.1
												Fgf13	2E-03	0.1	Actn1	8E-06	0.1							Capns1	1E-06	0.1	Hyal2	5E-01	0.1
												Ginm1	7E-03	0.1	Ss18	3E-04	0.1							Gin1	8E-01	0.1	Pex16	3E-01	0.1
												Clstn1	1E-02	0.1	Ncoa2	3E-03	0.1							Tnpo1	3E-03	0.1	Rbfox1	6E-01	0.1
												Trpc1	2E-01	0.1	Hps5	2E-02	0.1							Ccdc71	6E-01	0.1	Dync1li1	1E-02	0.1
												Ppm1a	8E-04	0.1	Ccnt2	2E-02	0.1							Ptpn11	2E-03	0.1	Aagab	2E-01	0.1
												Ppard	2E-01	0.1	Zcchc14	1E-02	0.1							Mrpl28	7E-03	0.1	Usp15	7E-02	0.1
												Psmf1	4E-02	0.1	Btbd2	2E-02	0.1							Btbd8	7E-01	0.1	Ppp1r12a	4E-03	0.1
												Psmc3	2E-04	0.1	Il17ra	5E-02	0.1							Abraxas2	5E-02	0.1	Snx12	3E-01	0.1
												Grk6	3E-02	0.1	Gm13056	2E-01	0.1							Pxdn	6E-03	0.1	Txndc17	8E-03	0.1
												Snrpg	2E-06	0.1	Txndc15	2E-05	0.1							Cdk16	4E-02	0.1	Cyth2	6E-01	0.1
												Nog	1E+00	0.1	Prmt2	1E-02	0.1							Ap2b1	1E-02	0.1	Pcolce	2E-05	0.1
												Anapc4	2E-03	0.1	Dpagt1	6E-01	0.1							Cox6c	9E-13	0.1	Cerk	3E-01	0.1
												Eml2	2E-01	0.1	Atp13a3	5E-04	0.1							Atp6v1c1	1E-01	0.1	Naa25	8E-01	0.1
												Msh3	1E-01	0.1	Cep41	2E-03	0.1							Gpr173	3E-01	0.1	Prpf31	2E-01	0.1
												Gxylt1	2E-03	0.1	Vangl1	6E-02	0.1							Rnf169	3E-01	0.1	Rbm34	6E-01	0.1
												Ppp1r35	1E-01	0.1	Atrn	5E-01	0.1							Ythdf2	1E-02	0.1	Acox1	6E-02	0.1
												8030462N17Rik	2E-02	0.1	Abcb8	2E-01	0.1							Socs7	4E-01	0.1	Pgap2	9E-02	0.1
												Tmub2	5E-01	0.1	Commd4	4E-02	0.1							Arhgap1	2E-01	0.1	Ppa2	4E-02	0.1
												Mrps17	1E-03	0.1	Myl9	8E-01	0.1							Nsun3	8E-01	0.1	Lncpint	6E-02	0.1
												Tmem241	1E-01	0.1	Birc6	9E-07	0.1							Nupl2	8E-01	0.1	Prkaa1	9E-02	0.1
												Arhgap35	5E-03	0.1	Smyd5	1E-01	0.1							Hoxc5	3E-01	0.1	Lekr1	3E-01	0.1
												Abcb1b	2E-01	0.1	Dab2ip	5E-04	0.1							Mrps2	6E-01	0.1	Hscb	4E-02	0.1
												Thoc5	4E-01	0.1	Abi2	1E-02	0.1							Zfp384	9E-01	0.1	Rbmx	1E+00	0.1
												Mmp16	6E-02	0.1	Prmt5	1E-02	0.1							Phb	3E-01	0.1	Zkscan8	1E+00	0.1
												Ufm1	3E-04	0.1	Ccdc50	1E-04	0.1							Maml1	5E-01	0.1	Zhx2	2E-01	0.1
												R74862	3E-01	0.1	Fam53b	5E-02	0.1							Akap10	6E-02	0.1	Fis1	7E-03	0.1
												Fem1b	9E-02	0.1	Ppa2	5E-03	0.1							Lrrcc1	3E-01	0.1	4732471J01Rik	9E-01	0.1
												Mrpl40	1E-02	0.1	Meis2	5E-02	0.1							Aldh2	8E-06	0.1	mt-Co1	7E-06	0.1
												Fbxo17	7E-01	0.1	Ctsh	2E-06	0.1							Rhbdf1	5E-01	0.1	mt-Atp6	8E-08	0.1
												Hectd4	8E-02	0.1	Snx19	2E-01	0.1							Ttc17	2E-01	0.1	Setd1a	3E-01	0.1
												Pter	6E-02	0.1	Zfyve27	2E-02	0.1							Tor1aip2	5E-02	0.1	Nemf	2E-01	0.1
												9530068E07Rik	1E-03	0.1	Oxsr1	2E-01	0.1							Med9	1E-01	0.1	Cat	8E-02	0.1
												Gm15050	3E-01	0.1	Dnajc19	9E-06	0.1							Sdhc	2E-02	0.1	Lrfn4	3E-01	0.1
												Mob1b	1E-02	0.1	Gadd45g	5E-05	0.1							Rfx5	2E-01	0.1	Cenpb	2E-02	0.1
												Paf1	7E-02	0.1	Atpaf1	1E-02	0.1							Cttn	1E-03	0.1	Exosc7	4E-02	0.1

Limb Mesenchyme						Chondrogenic						Fibroblast						Undefined						Articular/Synovial Fibroblast					
Control			*Notch2* $^{tm1.1Ecan}$			Control			*Notch2* $^{tm1.1Ecan}$			Control			*Notch2* $^{tm1.1Ecan}$			Control			*Notch2* $^{tm1.1Ecan}$			Control			*Notch2* $^{tm1.1Ecan}$		
Gene	*p*	FC	Gene	*p*	FC	Gene	*p*	FC	Gene	*p*	FC	Gene	*p*	FC	Gene	*p*	FC	Gene	*p*	FC	Gene	*p*	FC	Gene	*p*	FC	Gene	*p*	FC
												Cops2	8E-02	0.1	Commd10	5E-04	0.1							Ccdc34	6E-04	0.1	Tia1	1E-02	0.1
												Ccni	3E-04	0.1	Tob2	5E-04	0.1							Pdcd7	4E-01	0.1	Commd7	4E-01	0.1
												Gatad2b	3E-03	0.1	Adar	3E-02	0.1							H2afj	7E-05	0.1	Fktn	2E-01	0.1
												Dynll1	4E-01	0.1	Ankrd13a	2E-04	0.1							Sap30l	4E-02	0.1	Crk	5E-02	0.1
												BC005537	4E-04	0.1	Dhx34	2E-01	0.1							Qrich1	6E-02	0.1	Pskh1	4E-01	0.1
												Snx16	1E-01	0.1	Kmt2e	2E-04	0.1							Kmt2c	1E-04	0.1	Kmt5b	7E-02	0.1
												Zswim4	1E-01	0.1	Toe1	3E-02	0.1							Nars2	9E-01	0.1	Scmh1	7E-02	0.1
												Aldh6a1	3E-01	0.1	Ino80e	5E-03	0.1							Lrrc8d	5E-01	0.1	Czib	8E-02	0.1
												Zfp52	1E-01	0.1	Tbc1d22b	1E-01	0.1							Emc3	3E-01	0.1	Epm2aip1	2E-01	0.1
												Dda1	1E-02	0.1	Rspry1	9E-03	0.1							Eif2s3x	1E-01	0.1	Mmgt2	2E-01	0.1
												Edc4	3E-01	0.1	Ctns	1E+00	0.1							2310057M21Rik	6E-01	0.1	Cpeb4	3E-02	0.1
												Dcaf7	5E-02	0.1	Zfp983	2E-01	0.1							Reep3	5E-03	0.1	Fam8a1	5E-01	0.1
												Tor1a	2E-02	0.1	Olfml3	1E-14	0.1							Mtm1	2E-01	0.1	Kptn	1E+00	0.1
												Map3k8	2E-01	0.1	Ppp1r18	8E-04	0.1							Snx13	1E-01	0.1	Pter	7E-01	0.1
												Ctif	9E-02	0.1	Fmnl2	6E-07	0.1							Bcl7a	8E-01	0.1	Lysmd4	7E-02	0.1
												Abi2	5E-04	0.1	Zfp532	5E-03	0.1							Gdi1	5E-02	0.1	Tmem9	5E-01	0.1
												Dido1	3E-02	0.1	Pcdh11x	2E-05	0.1							Etfa	5E-04	0.1	Ydjc	3E-01	0.1
												Natd1	3E-01	0.1	Mmgt2	1E-01	0.1							Atp6v1d	2E-02	0.1	Uqcc3	3E-03	0.1
												Hip1	3E-02	0.1	Ankhd1	5E-04	0.1							Zfp592	1E-01	0.1	Tmsb4x	2E-08	0.1
												Upf3a	3E-03	0.1	Osbpl6	2E-02	0.1							Mpst	1E-01	0.1	Sec16a	5E-01	0.1
												Bace2	6E-01	0.1	Marcks	3E-17	0.1							Trip4	3E-03	0.1	Fam129a	1E-01	0.1
												Fscn1	2E-02	0.1	Ccdc86	2E-04	0.1							Msto1	7E-01	0.1	Nrk	1E-04	0.1
												Abhd13	1E-01	0.1	Map3k1	2E-02	0.1							Slmap	1E-03	0.1	Gpx3	9E-01	0.1
												Setx	5E-03	0.1	Yy1	2E-03	0.1							Ago1	1E-01	0.1	Slc22a17	6E-01	0.1
												Cpsf3	5E-02	0.1	Polr2j	1E-05	0.1							Wsb1	2E-03	0.1	Galnt13	7E-02	0.1
												Cul4a	4E-02	0.1	Rere	9E-05	0.1							Cryl1	5E-02	0.1	0610030E20Rik	8E-02	0.1
												Swap70	7E-01	0.1	Cmtm4	3E-01	0.1							Dus2	8E-01	0.1	Mettl3	4E-01	0.1
												Inppl1	6E-02	0.1	Morc3	1E-02	0.1							Ccdc112	5E-01	0.1	Ostf1	2E-02	0.1
												Eny2	3E-04	0.1	Maml1	3E-02	0.1							Fbxl17	4E-03	0.1	Atp6v1f	6E-04	0.1
												Ndfip2	3E-03	0.1	Arhgef10	1E-02	0.1							Tmem63b	2E-01	0.1	Rmdn1	9E-03	0.1
												Piezo1	1E-02	0.1	Eny2	2E-07	0.1							Zmym1	4E-01	0.1	Bmper	5E-01	0.1
												Cops8	9E-03	0.1	Setd1a	4E-02	0.1							Lin7c	9E-02	0.1	Kdm7a	4E-01	0.1
												Xpo6	1E-02	0.1	Actr3	2E-04	0.1							Uimc1	8E-02	0.1	mt-Nd5	4E-03	0.1
												Cotl1	9E-01	0.1	Wdr60	2E-03	0.1							Zfp141	2E-01	0.1	Cmip	4E-02	0.1
												Gna13	2E-01	0.1	Kif3b	2E-01	0.1							Snhg15	5E-01	0.1	Pitpnm2	3E-01	0.1
												Brms1l	1E-01	0.1	Gtdc1	7E-03	0.1							Ube2q1	6E-04	0.1	Sike1	9E-02	0.1
												Ogfod2	1E-01	0.1	Nrd1	5E-05	0.1							Actr2	1E-04	0.1	1110008P14Rik	3E-03	0.1
												Usp10	1E-02	0.1	Rsf1	1E-02	0.1							Kdm4c	1E-02	0.1	Ccnc	4E-01	0.1
												Hps5	4E-02	0.1	Alas1	5E-03	0.1							Nme2	1E-06	0.1	Cdc34	2E-02	0.1
												Epn1	7E-01	0.1	Pum2	1E-03	0.1							1810024B03Rik	9E-01	0.1	Pip4k2b	6E-01	0.1
												Ndufb7	2E-06	0.1	Tent4b	3E-02	0.1							Khsrp	2E-01	0.1	Zc3h7b	1E-01	0.1
												Atl1	8E-01	0.1	Med15	8E-03	0.1							Capn7	3E-02	0.1	Aggf1	5E-02	0.1
												Pbx3	2E-03	0.1	Pik3r1	3E-04	0.1							Slc25a14	7E-01	0.1	Invs	4E-01	0.1
												Fbln5	2E-07	0.1	Tnxb	6E-07	0.1							Rabep2	2E-01	0.1	Hectd4	3E-01	0.1
												Crebl2	1E+00	0.1	Poldip3	2E-02	0.1							Naa40	4E-01	0.1	Hgs	2E-01	0.1
												Ccdc66	1E-02	0.1	Bptf	1E-03	0.1							Sh3bgrl	5E-07	0.1	Tmem143	2E-01	0.1
												Rsbn1l	9E-03	0.1	Pcbp2	3E-08	0.1							Atic	4E-01	0.1	Micos10	3E-04	0.1
												Sav1	1E-01	0.1	Gosr1	2E-03	0.1							Aarsd1	1E-01	0.1	Cpne3	2E-01	0.1
												Tyw5	1E-01	0.1	Tax1bp3	3E-05	0.1							Ptprs	3E-02	0.1	Bola3	3E-03	0.1
												Ext2	1E-03	0.1	Sec16b	2E-02	0.1							Akap7	6E-01	0.1	Myo9b	4E-01	0.1
												3110040N11Rik	5E-02	0.1	Chst2	8E-02	0.1							Ccdc191	2E-01	0.1	Tbc1d23	1E-02	0.1
												Mob2	1E-02	0.1	Acsl3	2E-02	0.1							Usp5	4E-01	0.1	Speg	7E-01	0.1
												Ptges3	4E-04	0.1	Ehd3	5E-01	0.1							Lrrc1	2E-01	0.1	Bmyc	4E-01	0.1
												Raf1	1E-02	0.1	Adipor2	3E-02	0.1							BC005624	1E-02	0.1	Fam185a	7E-01	0.1
												Vkorc1l1	3E-03	0.1	Atxn3	4E-01	0.1							Chchd4	8E-01	0.1	Exosc10	4E-02	0.1
												Osbpl11	6E-02	0.1	Taf11	1E-04	0.1							Sap30bp	2E-01	0.1	Gys1	3E-01	0.1
												Lamtor5	2E-03	0.1	Wdcp	3E-02	0.1							Hspbp1	1E-01	0.1	Slc35e4	4E-02	0.1
												Uba1	6E-04	0.1	Dnajc8	5E-05	0.1							Gtpbp1	8E-01	0.1	Mtpn	3E-03	0.1
												Foxd2os	2E-02	0.1	Ppm1b	5E-04	0.1							Phospho2	1E+00	0.1	Cript	5E-02	0.1
												Vps26b	5E-01	0.1	Acd	5E-03	0.1							Ppil2	3E-01	0.1	Camk2g	1E-01	0.1
												Kat6b	1E-02	0.1	Cox20	8E-06	0.1							Gm42418	1E-08	0.1	Ccser2	2E-02	0.1
												Mkln1	4E-03	0.1	Elf2	2E-03	0.1							Carm1	2E-01	0.1	Picalm	1E-02	0.1
												Osbpl9	7E-05	0.1	Nop56	5E-03	0.1							Sqstm1	4E-03	0.1	Slc39a3	8E-01	0.1
												Naa35	8E-02	0.1	Bbs7	2E-02	0.1							Fam210b	6E-01	0.1	Pak3	3E-02	0.1

Limb Mesenchyme					
Control			$Notch2^{tm1.1Ecan}$		
Gene	*p*	FC	Gene	*p*	FC

Chondrogenic					
Control			$Notch2^{tm1.1Ecan}$		
Gene	*p*	FC	Gene	*p*	FC

Fibroblast					
Control			$Notch2^{tm1.1Ecan}$		
Gene	*p*	FC	Gene	*p*	FC
Ttc13	9E-02	0.1	Dcun1d1	5E-02	0.1
AI837181	1E-01	0.1	Snapc1	1E-02	0.1
Elp1	2E-01	0.1	Ctnnbip1	2E-02	0.1
Fbxw7	6E-02	0.1	Kdm1a	9E-06	0.1
Pgrmc1	8E-03	0.1	Epc2	3E-02	0.1
Dhrs7b	2E-01	0.1	Tnks2	9E-04	0.1
Rfk	5E-03	0.1	Fndc3b	2E-06	0.1
Cald1	1E-05	0.1	Zfp668	2E-02	0.1
Ino80d	1E-01	0.1	Exosc7	5E-03	0.1
Ccdc62	8E-01	0.1	Zfp292	4E-03	0.1
Ano10	3E-01	0.1	AI837181	7E-05	0.1
Med16	1E-01	0.1	Spsb3	1E-02	0.1
Abhd2	3E-02	0.1	Dhx57	2E-03	0.1
U2surp	6E-03	0.1	Avl9	5E-02	0.1
Slc25a44	7E-02	0.1	Zfp866	3E-02	0.1
Ppm1b	2E-03	0.1	Triobp	9E-03	0.1
Gmcl1	5E-02	0.1	Slc35e2	4E-01	0.1
Lpcat2	4E-02	0.1	Man2a1	2E-03	0.1
Abhd8	7E-03	0.1	Hspg2	2E-05	0.1
Ttyh2	7E-02	0.1	Myg1	8E-03	0.1
Nop9	1E-01	0.1	Prpf18	2E-03	0.1
Tacc1	8E-03	0.1	Gtpbp3	2E-01	0.1
Otud6b	9E-03	0.1	Uhrf1bp1l	3E-02	0.1
Ubtd2	1E-02	0.1	Chmp6	4E-03	0.1
Gm20559	1E-01	0.1	Ranbp2	5E-05	0.1
Stx12	2E-04	0.1	Stk16	2E-04	0.1
Rabgef1	4E-03	0.1	Mapk8ip1	1E-02	0.1
Qrich1	9E-02	0.1	Phka1	8E-04	0.1
Srsf10	4E-06	0.1	Fam171a1	4E-02	0.1
Synpo	1E-03	0.1	Etnk1	4E-03	0.1
Aebp1	6E-02	0.1	Insig2	3E-04	0.1
Gabpb2	2E-01	0.1	Naglu	3E-04	0.1
Med15	1E-02	0.1	Ino80b	1E-01	0.1
H1f0	1E-01	0.1	Nhlrc2	3E-04	0.1
Pfdn6	2E-02	0.1	Angptl2	9E-03	0.1
Mpst	2E-01	0.1	Cdc16	4E-04	0.1
Gatad1	6E-05	0.1	Myl12b	6E-04	0.1
Vezf1	7E-04	0.1	Arl2	3E-02	0.1
1700084C06Rik	8E-02	0.1	Pet100	2E-04	0.1
Pde8a	6E-03	0.1	Tpd52l2	6E-06	0.1
Notch2	2E-04	0.1	Arrdc3	5E-02	0.1
Gm49336	9E-02	0.1	Lclat1	3E-02	0.1
Cep63	5E-02	0.1	Thoc2	1E-04	0.1
Rbm42	5E-02	0.1	Trak1	1E-02	0.1
Abat	1E-01	0.1	Cnot10	4E-04	0.1
Rnasek	5E-05	0.1	Bach1	7E-03	0.1
Mrps25	5E-02	0.1	Rnf216	2E-03	0.1
Rapgef2	2E-03	0.1	Hmgcl	7E-02	0.1
Cox11	6E-01	0.1	Osbpl1a	1E-03	0.1
Tmem120a	1E-02	0.1	Ten1	2E-02	0.1
Hmces	2E-01	0.1	Oaz2	1E-02	0.1
Galk2	4E-03	0.1	Timm22	3E-03	0.1
Gpatch11	3E-02	0.1	Sept2	3E-01	0.1
Hnrnpk	3E-06	0.1	Gm49359	4E-01	0.1
Zbtb45	2E-02	0.1	Dcp2	1E-02	0.1
Usp25	2E-01	0.1	Rab40c	1E-01	0.1
Med28	2E-03	0.1	Tbx5	3E-02	0.1
Pomp	3E-03	0.1	Ephb2	7E-01	0.1
Ldah	2E-02	0.1	Ylpm1	4E-03	0.1
C87436	3E-01	0.1	Arf5	8E-12	0.1
Snx7	8E-03	0.1	Pabpn1	5E-07	0.1
Fam118a	2E-01	0.1	Mar9	8E-01	0.1
Tacc2	4E-01	0.1	Ddx1	1E-05	0.1
Fbxl18	2E-01	0.1	Stau1	9E-03	0.1

Undefined					
Control			$Notch2^{tm1.1Ecan}$		
Gene	*p*	FC	Gene	*p*	FC

Articular/Synovial Fibroblast					
Control			$Notch2^{tm1.1Ecan}$		
Gene	*p*	FC	Gene	*p*	FC
Napg	1E-01	0.1	Zbtb43	5E-01	0.1
Usp13	9E-01	0.1	Fam50a	4E-03	0.1
Tmem98	7E-01	0.1	Clcc1	9E-02	0.1
Clip2	9E-01	0.1	Rab12	2E-02	0.1
Tusc1	6E-01	0.1	Ankhd1	2E-01	0.1
Rnf41	3E-01	0.1	Brpf1	9E-01	0.1
Cir1	1E-01	0.1	Cfl1	3E-04	0.1
Sacm1l	6E-02	0.1	Dctn6	2E-01	0.1
Ndufaf7	4E-01	0.1	Zmat3	1E-01	0.1
Zfp36	4E-03	0.1	Oga	4E-02	0.1
Pcif1	3E-01	0.1	Kdm4a	5E-01	0.1
Ccng1	7E-02	0.1	Aldoa	7E-04	0.1
Rala	1E-03	0.1	Spen	7E-01	0.1
Kansl1	9E-04	0.1	Ttc27	2E-01	0.1
Pttg1ip	2E-02	0.1	Cspp1	2E-02	0.1
mt-Co1	2E-13	0.1	Etfrf1	1E-01	0.1
Lap3	2E-02	0.1	Wdr45b	1E-02	0.1
Rab35	2E-01	0.1	Kctd10	2E-01	0.1
Irf1	5E-01	0.1	Bud13	7E-01	0.1
Osbpl8	2E-03	0.1	Tmlhe	2E-01	0.1
Slc39a9	4E-01	0.1	Pym1	6E-01	0.1
Cep44	9E-01	0.1	Mllt10	1E-02	0.1
Wdr11	5E-01	0.1	Nop53	6E-02	0.1
Ppp2r1a	3E-02	0.1	Capn10	8E-01	0.1
Inpp5f	2E-01	0.1	Slc25a26	4E-01	0.1
Pip4p2	3E-02	0.1	Wdr89	8E-01	0.1
Ubxn7	4E-01	0.1			
Lsm6	3E-02	0.1			
Pccb	9E-01	0.1			
Chmp1a	3E-01	0.1			
Ifnar2	6E-03	0.1			
Pbrm1	7E-03	0.1			
Mar7	7E-03	0.1			
Hes1	2E-01	0.1			
Ipo4	3E-01	0.1			
Ubl7	1E-01	0.1			
Ehd4	4E-03	0.1			
2810006K23Rik	8E-01	0.1			
Aqp1	1E+00	0.1			
Krt10	2E-01	0.1			
Iars2	3E-01	0.1			
Mien1	2E-02	0.1			
Plekhg5	5E-02	0.1			
Cetn2	1E-01	0.1			
Dpf2	2E-01	0.1			
Nab2	2E-01	0.1			
Glyr1	8E-02	0.1			
Zc3hc1	1E+00	0.1			
Rpgrip1l	2E-01	0.1			
Pus10	2E-01	0.1			
Parp4	3E-01	0.1			
Armc8	7E-01	0.1			
Zbtb1	3E-03	0.1			
8030462N17Rik	3E-01	0.1			
Sat1	5E-04	0.1			
Btrc	2E-01	0.1			
Mrpl27	1E-01	0.1			
Galnt7	3E-01	0.1			
Fbxl19	9E-01	0.1			
Brpf1	2E-01	0.1			
Smg1	3E-02	0.1			
Kdm6a	3E-02	0.1			
Kdm2b	1E-01	0.1			
Csrp1	5E-02	0.1			

Limb Mesenchyme					
Control			Notch2 $^{tm1.1Ecan}$		
Gene	*p*	FC	Gene	*p*	FC

Chondrogenic					
Control			Notch2 $^{tm1.1Ecan}$		
Gene	*p*	FC	Gene	*p*	FC

Fibroblast					
Control			Notch2 $^{tm1.1Ecan}$		
Gene	*p*	FC	Gene	*p*	FC
Kat14	4E-01	0.1	Tcn2	4E-03	0.1
Nedd8	3E-05	0.1	Tchp	5E-01	0.1
Fat4	2E-04	0.1	Serhl	1E-04	0.1
Nme1	2E-05	0.1	Col12a1	6E-09	0.1
Mettl16	2E-02	0.1	Immp1l	1E-04	0.1
Tulp4	3E-03	0.1	Tmem158	3E-03	0.1
Zmpste24	1E-02	0.1	Zdhhc20	7E-05	0.1
Cpq	7E-06	0.1	Pfkfb2	6E-01	0.1
Pbx1	2E-03	0.1	Sdc3	1E-03	0.1
Ubr4	2E-03	0.1	Hspa2	3E-03	0.1
Tfpt	2E-01	0.1	Crkl	4E-02	0.1
H2-T22	6E-02	0.1	Elovl6	5E-02	0.1
Exoc5	1E-02	0.1	Sec24b	2E-03	0.1
Ppp1r11	5E-03	0.1	Ankrd24	6E-02	0.1
Kpna1	4E-03	0.1	Zfp248	2E-02	0.1
Atr	3E-02	0.1	Mrpl33	4E-08	0.1
Taf11	2E-02	0.1	Gatc	7E-01	0.1
Agtrap	2E-02	0.1	Pitpnb	4E-05	0.1
Hsbp1	1E-03	0.1	Rnf185	7E-01	0.1
Gm45669	5E-01	0.1	Macrod2	1E-02	0.1
Zdhhc6	4E-03	0.1	Brms1l	2E-02	0.1
Ift122	4E-01	0.1	Ogdh	1E-03	0.1
Mrpl3	4E-02	0.1	Abhd8	4E-03	0.1
Sbf2	1E-02	0.1	Phlda3	7E-04	0.1
Fbrsl1	5E-01	0.1	Zfp346	1E-02	0.1
St6galnac6	2E-01	0.1	Tgif1	8E-01	0.1
Btbd7	5E-05	0.1	Rbm22	5E-03	0.1
Fn1	3E-07	0.1	Tnip1	3E-01	0.1
Mtf2	1E-02	0.1	Gatad2b	5E-04	0.1
Chchd4	1E-01	0.1	Stimate	7E-03	0.1
Wrnip1	7E-02	0.1	Swt1	7E-05	0.1
Sspn	3E-02	0.1	Rapgef2	2E-05	0.1
Strbp	2E-03	0.1	Zfp281	2E-02	0.1
Snx19	4E-01	0.1	Decr2	4E-01	0.1
Zfp207	4E-04	0.1	Acly	7E-06	0.1
Ids	5E-01	0.1	Slc11a2	1E-01	0.1
Stim1	1E-02	0.1	Bcorl1	3E-02	0.1
Dennd5a	4E-02	0.1	Ncbp3	1E-03	0.1
Fam216a	3E-02	0.1	Gclc	1E-03	0.1
Evl	3E-01	0.1	Entpd5	4E-02	0.1
Sf3b5	2E-04	0.1	Nipbl	2E-05	0.1
Rhoc	3E-05	0.1	Scamp1	2E-02	0.1
Slc25a17	4E-02	0.1	Pex1	5E-01	0.1
Ctdsp2	1E-02	0.1	Vipas39	4E-02	0.1
Acadm	2E-02	0.1	Tnfaip8	5E-05	0.1
Mrpl4	1E-02	0.1	Cd320	1E-02	0.1
Galc	2E-01	0.1	Atxn1	8E-07	0.1
Nelfb	8E-02	0.1	Cetn2	2E-03	0.1
Bag2	2E-01	0.1	Ybx3	3E-08	0.1
Tceanc2	1E-01	0.1	Trir	7E-06	0.1
Asah1	7E-04	0.1	Naa35	5E-03	0.1
Ints2	3E-02	0.1	Atp5g2	4E-09	0.1
Wdr26	1E-03	0.1	Cep44	3E-02	0.1
Exosc3	3E-01	0.1	Gmpr2	9E-02	0.1
Psma1	2E-02	0.1	Fer	1E-04	0.1
Tor2a	2E-01	0.1	Churc1	3E-03	0.1
Ap2b1	4E-03	0.1	Mertk	8E-02	0.1
Zdhhc1	2E-01	0.1	Cog4	1E-02	0.1
Polr3c	3E-01	0.1	Slc18b1	8E-01	0.1
Vps39	7E-02	0.1	Trip12	6E-06	0.1
Zfyve27	7E-01	0.1	Eif4enif1	2E-03	0.1
Crebzf	2E-01	0.1	Eif4ebp2	4E-02	0.1
Rbms3	4E-08	0.1	Calr	3E-08	0.1
Tchp	3E-02	0.1	Zcchc17	3E-03	0.1

Undefined					
Control			Notch2 $^{tm1.1Ecan}$		
Gene	*p*	FC	Gene	*p*	FC

Articular/Synovial Fibroblast					
Control			Notch2 $^{tm1.1Ecan}$		
Gene	*p*	FC	Gene	*p*	FC
Gtf3c6	5E-02	0.1			
Pgls	7E-04	0.1			
Ube2r2	4E-05	0.1			
Yipf1	3E-02	0.1			
Cops6	5E-02	0.1			
Tctex1d2	2E-01	0.1			
Gcc2	2E-02	0.1			
Cbfa2t2	4E-02	0.1			
Klf9	7E-03	0.1			
Acap2	8E-04	0.1			
Tcf20	1E-02	0.1			
Tep1	8E-01	0.1			
Cep120	1E-01	0.1			
R3hcc1l	1E-01	0.1			
Rfwd3	3E-01	0.1			
Nek4	3E-01	0.1			
Kifap3	7E-03	0.1			
Csnk1g1	6E-02	0.1			
Ccz1	8E-02	0.1			
Nemf	8E-03	0.1			
Ppp6r3	6E-02	0.1			
Hdac6	8E-01	0.1			
Zfp934	7E-01	0.1			
Glipr2	8E-02	0.1			
Rrn3	3E-01	0.1			
Socs6	3E-01	0.1			
Gucd1	3E-01	0.1			
Anks3	6E-01	0.1			
Cmc1	5E-04	0.1			
Scfd2	6E-01	0.1			
Eml1	1E-01	0.1			
Sema5a	9E-01	0.1			
Mmaa	4E-01	0.1			
Lman2	8E-03	0.1			
Gnai3	2E-03	0.1			
Arpc5	3E-03	0.1			
Rnf44	2E-01	0.1			
Gtf3c2	4E-01	0.1			
Steap2	8E-01	0.1			
Ostf1	5E-02	0.1			
Nudt1	8E-01	0.1			
mt-Atp6	2E-11	0.1			
Mink1	9E-01	0.1			
Gabarap	2E-07	0.1			
Dnajc12	3E-01	0.1			
Rab11fip2	3E-02	0.1			
Nphp3	9E-01	0.1			
Mis12	7E-01	0.1			
2410004B18Rik	7E-02	0.1			
Coa6	3E-01	0.1			
Smarcad1	2E-01	0.1			
Sh3bp5l	6E-01	0.1			
Polr3gl	6E-02	0.1			
Thumpd2	4E-01	0.1			
Snx27	5E-02	0.1			
Stk19	1E-01	0.1			
Frg1	2E-02	0.1			
Rbm18	5E-02	0.1			
Coa3	1E-02	0.1			
1700123M08Rik	3E-01	0.1			
mt-Nd1	2E-07	0.1			
Prune1	9E-01	0.1			
Slbp	1E-01	0.1			
Ccndbp1	1E-01	0.1			

Limb Mesenchyme						Chondrogenic						Fibroblast						Undefined						Articular/Synovial Fibroblast					
Control			$Notch2^{tm1.1Ecan}$			Control			$Notch2^{tm1.1Ecan}$			Control			$Notch2^{tm1.1Ecan}$			Control			$Notch2^{tm1.1Ecan}$			Control			$Notch2^{tm1.1Ecan}$		
Gene	*p*	FC	Gene	*p*	FC	Gene	*p*	FC	Gene	*p*	FC	Gene	*p*	FC	Gene	*p*	FC	Gene	*p*	FC	Gene	*p*	FC	Gene	*p*	FC	Gene	*p*	FC
												Nectin3	3E-02	0.1	Acer3	1E-02	0.1							Baiap2	1E-01	0.1			
												Tmed4	3E-02	0.1	Prrc2c	3E-06	0.1							Nmt2	9E-02	0.1			
												Nolc1	2E-01	0.1	Gps2	1E-02	0.1							Slc35b4	3E-01	0.1			
												Hps1	7E-01	0.1	Abi1	4E-04	0.1							Rps6kb2	3E-01	0.1			
												Zc3hav1	2E-02	0.1	Gspt1	1E-05	0.1							Fam210a	1E-01	0.1			
												Emc8	9E-03	0.1	Api5	1E-02	0.1							Dido1	2E-01	0.1			
												Vcpip1	7E-02	0.1	Gfm1	3E-01	0.1							Zdhhc17	2E-01	0.1			
												Tmx3	2E-03	0.1	Kdm4a	7E-02	0.1							Ints8	6E-01	0.1			
												Cdh13	4E-01	0.1	Mrtfa	3E-04	0.1							Eaf1	6E-01	0.1			
												Hoxa3	2E-02	0.1	Commd1	8E-08	0.1							Pkn2	1E-02	0.1			
												Myo19	2E-01	0.1	Mrpl12	1E-05	0.1							Pfdn5	1E-05	0.1			
												Dtnb	2E-02	0.1	Mfsd14b	3E-03	0.1							Atrip	8E-01	0.1			
												Ndufa9	1E-02	0.1	Serbp1	3E-10	0.1							Asxl2	5E-03	0.1			
												Pik3c2a	3E-02	0.1	Lman2	1E-04	0.1							Atxn3	3E-01	0.1			
												Ppa1	6E-02	0.1	Abcf2	4E-05	0.1							Vamp2	4E-02	0.1			
												Vps35l	9E-02	0.1	Sod1	9E-03	0.1							Slc16a3	5E-01	0.1			
												Naa50	2E-02	0.1	Cul4a	1E-01	0.1							Arl8a	2E-02	0.1			
												Lrp10	2E-02	0.1	Tmem234	1E-05	0.1							Phlpp2	3E-01	0.1			
												Synj1	1E-02	0.1	Zmiz1	8E-07	0.1							Zfp955b	3E-01	0.1			
												Sf1	2E-03	0.1	Mpg	1E-02	0.1							B230354K17Rik	6E-01	0.1			
												Pdha1	5E-03	0.1	Tfip11	2E-01	0.1							Atp5k	1E-04	0.1			
												Pias1	2E-02	0.1	Pxk	2E-03	0.1							Zfp933	5E-01	0.1			
												Rnf5	2E-01	0.1	Apoo	1E-02	0.1							Usp8	9E-02	0.1			
												Fam173a	9E-03	0.1	Rrp1	9E-03	0.1							Cplane1	9E-02	0.1			
												Sema3d	7E-02	0.1	Stard9	6E-01	0.1							Pgam5	8E-01	0.1			
												Pigv	3E-01	0.1	Actr8	1E-01	0.1							Cc2d1b	3E-01	0.1			
												Tmem176b	2E-03	0.1	Ddx52	4E-02	0.1							Prkd2	8E-01	0.1			
												Cux1	3E-03	0.1	Thoc1	2E-06	0.1							Stx7	7E-03	0.1			
												Rcan3	6E-01	0.1	Echdc2	9E-02	0.1							Pla2g4a	1E-01	0.1			
												Pdcd6ip	1E-03	0.1	Dnm2	3E-03	0.1							Sart3	6E-01	0.1			
												Cfap36	9E-04	0.1	Ubap2	7E-05	0.1							Kmt2e	2E-02	0.1			
												Sipa1l3	9E-03	0.1	Fbxl20	2E-01	0.1							Ndufa9	4E-01	0.1			
												Prdx4	2E-02	0.1	Armc1	6E-03	0.1							Zfp945	2E-01	0.1			
												Trappc8	3E-01	0.1	Ppip5k1	6E-02	0.1												
												Pus1	7E-01	0.1	Mlycd	5E-02	0.1												
												Sik2	2E-02	0.1	Lactb	2E-03	0.1												
												Flywch1	8E-03	0.1	Atad3a	1E-01	0.1												
												Atp7a	4E-01	0.1	Smo	3E-03	0.1												
												B3galt1	7E-06	0.1	Jpx	5E-01	0.1												
												Cstf2t	3E-01	0.1	Elk3	4E-02	0.1												
												Srcap	7E-02	0.1	Top2b	5E-04	0.1												
												Cd320	4E-02	0.1	Cacna2d1	3E-04	0.1												
												Rpap3	7E-02	0.1	Fbxo38	2E-03	0.1												
												Optn	1E-01	0.1	Mrps24	7E-04	0.1												
												Cpt1c	5E-01	0.1	Hipk3	1E-03	0.1												
												Setdb1	1E-01	0.1	Dpp8	5E-03	0.1												
												Riox2	1E-01	0.1	Dnajc13	2E-03	0.1												
												Zfp638	2E-04	0.1	Rc3h1	6E-03	0.1												
												Atf2	7E-02	0.1	Ubr5	1E-05	0.1												
												Ube2g2	1E-01	0.1	Hmbs	2E-01	0.1												
												Dmap1	4E-01	0.1	Ago1	3E-03	0.1												
												Pkn1	1E-01	0.1	Cbr4	2E-02	0.1												
												Osbp	3E-02	0.1	Bex3	2E-04	0.1												
												Dync2li1	1E-01	0.1	Zfp236	3E-01	0.1												
												Mrtfb	1E-02	0.1	Spag7	2E-04	0.1												
												Uchl3	2E-02	0.1	Usp4	2E-02	0.1												
												Prg4	1E-04	0.1	Ctdsp2	1E-05	0.1												
												Dicer1	4E-03	0.1	Gm20559	6E-01	0.1												
												Usp7	8E-03	0.1	Arhgap39	2E-02	0.1												
												Zfp790	5E-01	0.1	Chic1	7E-02	0.1												
												Zcchc17	5E-04	0.1	Slc29a1	2E-04	0.1												
												Gpm6b	2E-01	0.1	Scarb1	2E-02	0.1												
												Inf2	2E-01	0.1	Tra2b	4E-05	0.1												
												Ubxn2a	2E-01	0.1	Rela	2E-03	0.1												

Limb Mesenchyme						Chondrogenic						Fibroblast						Undefined						Articular/Synovial Fibroblast					
Control			*Notch2*$^{tm1.1Ecan}$			Control			*Notch2*$^{tm1.1Ecan}$			Control			*Notch2*$^{tm1.1Ecan}$			Control			*Notch2*$^{tm1.1Ecan}$			Control			*Notch2*$^{tm1.1Ecan}$		
Gene	*p*	FC	Gene	*p*	FC	Gene	*p*	FC	Gene	*p*	FC	Gene	*p*	FC	Gene	*p*	FC	Gene	*p*	FC	Gene	*p*	FC	Gene	*p*	FC	Gene	*p*	FC
												Ost4	1E-03	0.1	Rragc	2E-02	0.1												
												Zdhhc3	6E-03	0.1	Ints6	5E-02	0.1												
												4921524J17Rik	6E-02	0.1	Mtrex	4E-04	0.1												
												Safb	2E-02	0.1	Pex13	7E-02	0.1												
												Wasl	5E-04	0.1	Trpc1	5E-03	0.1												
												Cspp1	8E-02	0.1	Gm41724	1E-02	0.1												
												Rac1	5E-04	0.1	Rrn3	3E-01	0.1												
												Vps35	4E-03	0.1	Adat1	5E-01	0.1												
												Sec24c	5E-02	0.1	Ndufb8	4E-09	0.1												
												Ythdf2	3E-03	0.1	Hook2	4E-01	0.1												
												Mafl	9E-02	0.1	Ube2e3	4E-05	0.1												
												Bcr	5E-01	0.1	Smn1	1E-03	0.1												
												Spop	2E-03	0.1	Ube2i	2E-08	0.1												
												Tra2b	3E-03	0.1	Psma4	8E-06	0.1												
												Eipr1	2E-01	0.1	Txnl1	6E-06	0.1												
												Nfic	4E-05	0.1	Phf2	9E-03	0.1												
												Cdc34	1E-03	0.1	Irak2	3E-01	0.1												
												Tmem126b	7E-01	0.1	Galnt13	2E-01	0.1												
												Plaa	8E-02	0.1	Tra2a	5E-05	0.1												
												Supv3l1	8E-01	0.1	Ppard	5E-03	0.1												
												Ppm1f	9E-01	0.1	Arl5b	1E-01	0.1												
												Pip4p1	2E-02	0.1	Acot11	2E-01	0.1												
												Yod1	1E-01	0.1	Samd1	5E-02	0.1												
												Gnptab	2E-02	0.1	Tsga10	1E-03	0.1												
												Numa1	4E-02	0.1	Zfp90	1E-01	0.1												
												Lrig2	1E-01	0.1	Wdr59	8E-01	0.1												
												Mex3c	1E-01	0.1	Fbxw4	9E-02	0.1												
												Kat6a	3E-02	0.1	Zfp995	7E-02	0.1												
												Efemp1	1E-03	0.1	Dda1	4E-04	0.1												
												Aggf1	3E-02	0.1	Atg16l1	9E-02	0.1												
												F420014N23Rik	6E-01	0.1	Ccdc136	2E-02	0.1												
												Strn	2E-01	0.1	Tmem30a	1E-03	0.1												
												Tmem14a	6E-01	0.1	Pofut1	4E-02	0.1												
												Pcbp2	8E-06	0.1	Rnf20	3E-02	0.1												
												Ubl4a	1E-01	0.1	Slc25a44	1E-01	0.1												
												Coq6	5E-02	0.1	Kri1	2E-01	0.1												
												Nudt14	2E-01	0.1	Dnajc2	1E-03	0.1												
												Lrrc42	8E-03	0.1	Arf6	1E-03	0.1												
												Ccdc88a	1E-04	0.1	Polb	4E-03	0.1												
												Fam160b1	2E-01	0.1	Etfa	2E-06	0.1												
												Cpsf7	2E-02	0.1	Ppp1r37	5E-03	0.1												
												Tiprl	1E-01	0.1	Prr13	4E-05	0.1												
												Lsp1	6E-03	0.1	Gpx4	6E-12	0.1												
												Cisd1	7E-04	0.1	Ankrd52	2E-02	0.1												
												Dlg5	8E-02	0.1	Pcf11	2E-04	0.1												
												Pi4ka	2E-03	0.1	Tst	1E-02	0.1												
												Pfn1	3E-05	0.1	Slc20a2	1E-03	0.1												
												Otulin	1E-01	0.1	Osbpl5	4E-02	0.1												
												Arglu1	3E-05	0.1	Gon4l	2E-03	0.1												
												Tmc6	6E-01	0.1	Timp2	1E-18	0.1												
												Casd1	4E-01	0.1	Nbas	4E-01	0.1												
												Sfxn5	1E-01	0.1	H2-T22	5E-03	0.1												
												Tbcc	2E-01	0.1	Pds5a	9E-04	0.1												
												Sdccag8	3E-02	0.1	Ube2l3	3E-05	0.1												
												Gapdh	2E-03	0.1	Rad17	6E-03	0.1												
												Zfp326	6E-02	0.1	Msantd2	3E-02	0.1												
												Ifitm2	6E-04	0.1	Elp4	6E-04	0.1												
												Ncstn	2E-01	0.1	Ppp2r1a	4E-04	0.1												
												Myh9	4E-05	0.1	Tbl2	5E-02	0.1												
												Cetn4	1E-01	0.1	Trafd1	3E-02	0.1												
												Yju2	3E-01	0.1	Zfx	1E-03	0.1												
												Trps1	4E-07	0.1	Coq4	1E-01	0.1												
												Ubap2l	1E-04	0.1	Samhd1	1E-05	0.1												
												Cyp4v3	6E-02	0.1	Sufu	7E-02	0.1												

Limb Mesenchyme						Chondrogenic						Fibroblast						Undefined						Articular/Synovial Fibroblast					
Control			*Notch2* $^{tm1.1Ecan}$			Control			*Notch2* $^{tm1.1Ecan}$			Control			*Notch2* $^{tm1.1Ecan}$			Control			*Notch2* $^{tm1.1Ecan}$			Control			*Notch2* $^{tm1.1Ecan}$		
Gene	*p*	FC	Gene	*p*	FC	Gene	*p*	FC	Gene	*p*	FC	Gene	*p*	FC	Gene	*p*	FC	Gene	*p*	FC	Gene	*p*	FC	Gene	*p*	FC	Gene	*p*	FC
												Aldh16a1	4E-01	0.1	Klhdc3	3E-02	0.1												
												Rbbp7	7E-03	0.1	Gpaa1	2E-03	0.1												
												Bbs2	7E-01	0.1	Prr16	7E-01	0.1												
												Cdpf1	2E-01	0.1	Zfp810	6E-01	0.1												
												Zfp942	5E-01	0.1	Aco2	4E-05	0.1												
												Uqcrc1	2E-02	0.1	Csde1	2E-06	0.1												
												Rnf10	7E-04	0.1	Tmem134	5E-05	0.1												
												Dcaf1	8E-02	0.1	Znhit1	2E-05	0.1												
												Sec23ip	6E-02	0.1	BC005537	2E-03	0.1												
												Slc39a9	6E-01	0.1	Eps15l1	1E-02	0.1												
												Dedd	4E-02	0.1	Gtf3c3	2E-01	0.1												
												Strn4	1E-01	0.1	Atp6v0a4	4E-02	0.1												
												Pmpca	6E-02	0.1	Cnot4	1E-02	0.1												
												Retreg3	4E-02	0.1	Asna1	1E-02	0.1												
												Ube4a	2E-01	0.1	Tmc6	1E-01	0.1												
												Hdac3	5E-02	0.1	Gpr137	5E-03	0.1												
												Ap2a1	4E-02	0.1	Orc5	4E-02	0.1												
												Map4k3	1E-03	0.1	Slc25a40	8E-02	0.1												
												Tead3	2E-01	0.1	Cyth2	4E-02	0.1												
												Naf1	6E-02	0.1	Akr1e1	4E-02	0.1												
												Slc35b3	2E-01	0.1	Kmt2d	2E-02	0.1												
												Dazap2	4E-02	0.1	Sod3	3E-03	0.1												
												Slc30a9	3E-02	0.1	Ebp	6E-02	0.1												
												Gid4	2E-01	0.1	Micall1	1E-01	0.1												
												Hsph1	9E-03	0.1	Gtf3c2	6E-04	0.1												
												Sec61a2	7E-01	0.1	Mapk3	3E-04	0.1												
												Scaf4	2E-02	0.1	Strn	3E-02	0.1												
												Eif3l	2E-02	0.1	Cyb561d2	5E-02	0.1												
												Med26	3E-01	0.1	Tgfbr1	2E-03	0.1												
												Aatf	7E-02	0.1	Xiap	9E-03	0.1												
												Ralgapa1	6E-03	0.1	Lysmd4	2E-03	0.1												
												Slc16a4	7E-01	0.1	Pcgf6	5E-01	0.1												
												Scai	3E-02	0.1	Stat5b	8E-03	0.1												
												Zswim6	9E-03	0.1	Dnaja1	2E-09	0.1												
												Atg7	5E-03	0.1	Smad4	6E-04	0.1												
												Vamp7	3E-02	0.1	Zdhhc3	1E-02	0.1												
												Kiz	3E-01	0.1	Dnpep	4E-03	0.1												
												Plscr3	8E-03	0.1	Ubxn7	4E-01	0.1												
												Pi4kb	4E-02	0.1	Zc3h7b	1E-01	0.1												
												Arhgap17	7E-02	0.1	Gnptab	8E-03	0.1												
												Gm49359	9E-01	0.1	Gm4258	2E-01	0.1												
												Dnajb6	3E-05	0.1	Oga	9E-04	0.1												
												Cmtm7	9E-03	0.1	Micu2	3E-03	0.1												
												Ilrun	8E-03	0.1	Pnpla7	5E-01	0.1												
												Birc6	3E-05	0.1	Dcaf13	2E-01	0.1												
												Med21	1E-01	0.1	Papolg	8E-02	0.1												
												Map3k7	4E-02	0.1	Arid1b	5E-05	0.1												
												Slc25a20	2E-01	0.1	Psmg2	4E-04	0.1												
												Mpv17l	2E-01	0.1	Dusp22	4E-01	0.1												
												Xkr6	5E-01	0.1	Edem3	2E-01	0.1												
												Nudt2	9E-02	0.1	Stk11	3E-03	0.1												
												Zdhhc5	6E-02	0.1	Zfp182	7E-02	0.1												
												Irf1	3E-01	0.1	Herc4	3E-02	0.1												
												Mafb	4E-01	0.1	Ncor1	6E-05	0.1												
												Morc4	4E-01	0.1	Gtf3c1	3E-04	0.1												
												Ube2z	2E-02	0.1	Pik3cb	5E-03	0.1												
												Senp3	1E-01	0.1	Ap1m1	5E-03	0.1												
												Arhgap44	6E-02	0.1	Ctnna1	5E-05	0.1												
												Dvl3	4E-01	0.1	Lrrc42	3E-04	0.1												
												Zbtb24	1E-01	0.1	Galk2	1E-03	0.1												
												Nln	2E-02	0.1	Gsk3a	2E-02	0.1												
												Fubp1	2E-03	0.1	Tdrd7	3E-01	0.1												
												Slc41a2	6E-03	0.1	Ccn3	2E-06	0.1												
												Rras	2E-05	0.1	Stx6	2E-02	0.1												

Limb Mesenchyme						Chondrogenic						Fibroblast						Undefined						Articular/Synovial Fibroblast					
Control			*Notch2*$^{tm1.1Ecan}$			Control			*Notch2*$^{tm1.1Ecan}$			Control			*Notch2*$^{tm1.1Ecan}$			Control			*Notch2*$^{tm1.1Ecan}$			Control			*Notch2*$^{tm1.1Ecan}$		
Gene	*p*	FC	Gene	*p*	FC	Gene	*p*	FC	Gene	*p*	FC	Gene	*p*	FC	Gene	*p*	FC	Gene	*p*	FC	Gene	*p*	FC	Gene	*p*	FC	Gene	*p*	FC
												Pafah2	4E-01	0.1	Cpped1	6E-03	0.1												
												Adamts2	1E-03	0.1	Cdc42ep1	3E-01	0.1												
												Lemd3	1E-01	0.1	Eif6	4E-04	0.1												
												Rnf19a	3E-02	0.1	Dnajb6	2E-04	0.1												
												Tnks	5E-03	0.1	Trip6	2E-02	0.1												
												Cfap20	4E-02	0.1	Mlxip	2E-01	0.1												
												Wdr44	1E-01	0.1	Chd6	4E-03	0.1												
												9430038I01Rik	1E-01	0.1	Ints8	1E-02	0.1												
												Phf20	2E-02	0.1	Hsd17b4	2E-02	0.1												
												Ttc4	6E-01	0.1	Phf23	3E-01	0.1												
												Homer3	2E-02	0.1	Pak2	7E-05	0.1												
												Ensa	1E-01	0.1	Tbck	1E-03	0.1												
												Gclm	3E-02	0.1	Ppp4r1	7E-02	0.1												
												Rfx3	1E-01	0.1	Katna1	1E-02	0.1												
												Rpn1	3E-03	0.1	Zfp606	3E-02	0.1												
												Atrx	2E-04	0.1	Tcaim	8E-02	0.1												
												Klhdc4	1E-01	0.1	Eif2b4	4E-03	0.1												
												Wdtc1	6E-01	0.1	Gale	4E-02	0.1												
												Nucb2	1E-02	0.1	Dhx35	4E-01	0.1												
												Ccdc71	6E-02	0.1	Thra	8E-06	0.1												
												Seh1l	1E-02	0.1	Fyttd1	4E-02	0.1												
												Zfp143	2E-01	0.1	Osbpl11	6E-03	0.1												
												Qsox2	3E-01	0.1	Wbp4	3E-04	0.1												
												Eloc	1E-05	0.1	Abhd12	2E-07	0.1												
												Zfp994	2E-01	0.1	Fam107b	4E-04	0.1												
												Ctnnbl1	7E-02	0.1	Alg14	9E-04	0.1												
												Josd1	5E-02	0.1	Tox4	6E-02	0.1												
												Polr3e	2E-01	0.1	Sap30l	1E-01	0.1												
												Trpm7	7E-03	0.1	Mrps36	7E-04	0.1												
												Map7d1	9E-03	0.1	Dnm1l	3E-02	0.1												
												Rest	6E-03	0.1	Aff2	4E-03	0.1												
												Ctsl	1E-02	0.1	Tmem242	3E-01	0.1												
												Pltp	2E-02	0.1	Hmces	2E-03	0.1												
												Gsn	5E-14	0.1	Usp10	3E-03	0.1												
												Psma3	2E-05	0.1	Pgrmc1	8E-04	0.1												
												Phykpl	8E-01	0.1	Cbr1	1E-01	0.1												
												Adam17	2E-01	0.1	Ip6k2	1E-01	0.1												
												Gucd1	4E-01	0.1	Ttll7	1E-01	0.1												
												Pabpn1	2E-04	0.1	Cetn4	1E-01	0.1												
															Bpgm	8E-01	0.1												
															D8Ertd738e	4E-08	0.1												
															Xrn1	9E-04	0.1												
															Fam20b	7E-03	0.1												
															Ppp1r21	9E-02	0.1												
															Med28	8E-04	0.1												
															Cdk13	4E-05	0.1												
															Ppp6c	1E-04	0.1												
															Ccdc71	3E-02	0.1												
															Cwf19l2	9E-03	0.1												
															Daam1	7E-04	0.1												
															Ccdc126	6E-01	0.1												
															Snu13	3E-04	0.1												
															Spast	1E-02	0.1												
															Zc3h14	2E-02	0.1												
															Btbd7	7E-05	0.1												
															Dip2a	2E-01	0.1												
															Rusc2	3E-02	0.1												
															D6Wsu163e	1E-01	0.1												
															Xpo7	4E-03	0.1												
															Nemf	1E-03	0.1												
															Zdhhc18	1E-02	0.1												
															Mms19	7E-03	0.1												
															Gid8	1E-02	0.1												
															Pcnx4	1E-01	0.1												

Limb Mesenchyme						Chondrogenic						Fibroblast						Undefined						Articular/Synovial Fibroblast					
Control			*Notch2* $^{tm1.1Ecan}$			Control			*Notch2* $^{tm1.1Ecan}$			Control			*Notch2* $^{tm1.1Ecan}$			Control			*Notch2* $^{tm1.1Ecan}$			Control			*Notch2* $^{tm1.1Ecan}$		
**Gene**	***p***	**FC**	**Gene**	***p***	**FC**	**Gene**	***p***	**FC**	**Gene**	***p***	**FC**	**Gene**	***p***	**FC**	**Gene**	***p***	**FC**	**Gene**	***p***	**FC**	**Gene**	***p***	**FC**	**Gene**	***p***	**FC**	**Gene**	***p***	**FC**
															Strip1	4E-02	0.1												
															Wiz	7E-02	0.1												
															Ptpn12	1E-03	0.1												
															Uba1	1E-03	0.1												
															Abcf1	2E-05	0.1												
															Pdia4	2E-04	0.1												
															Bmp5	6E-03	0.1												
															Wrb	5E-01	0.1												
															Mphosph8	2E-02	0.1												
															Atad2b	7E-03	0.1												
															Chst12	8E-03	0.1												
															Sos2	3E-03	0.1												
															Rom1	2E-01	0.1												
															Atp7a	8E-02	0.1												
															Pdlim7	1E-03	0.1												
															Virma	3E-04	0.1												
															Rest	3E-02	0.1												
															Eif4g3	5E-03	0.1												
															Necap2	5E-01	0.1												
															Gfm2	2E-01	0.1												
															Fkbp15	5E-01	0.1												
															Ephx1	3E-04	0.1												
															Ttc9c	2E-01	0.1												
															Ppig	3E-04	0.1												
															Sdhd	2E-03	0.1												
															Abhd16a	3E-02	0.1												
															Ifitm2	4E-07	0.1												
															Cab39l	1E-02	0.1												
															Map4k4	2E-05	0.1												
															Sipa1l3	8E-02	0.1												
															Actr6	5E-02	0.1												
															Appl1	4E-03	0.1												
															Ccnt1	8E-03	0.1												
															Ajuba	7E-02	0.1												
															Frmd8	1E-01	0.1												
															Aagab	6E-02	0.1												
															Pinx1	3E-02	0.1												
															Ptdss1	6E-02	0.1												
															Scmh1	1E-03	0.1												
															Elp2	2E-01	0.1												
															Cdh13	9E-03	0.1												
															Med20	4E-01	0.1												
															Glt8d1	2E-03	0.1												
															Metrnl	6E-04	0.1												
															Ubxn2b	5E-02	0.1												
															Adamtsl5	4E-04	0.1												
															Shkbp1	2E-01	0.1												
															Dnajc25	3E-03	0.1												
															Cbr3	3E-01	0.1												
															Pcyt1a	3E-03	0.1												
															Skap2	3E-05	0.1												
															Npepps	1E-03	0.1												
															Neurl4	2E-01	0.1												
															Fam168a	2E-01	0.1												
															Arl6ip5	5E-05	0.1												
															Tmem37	1E-02	0.1												
															Kif3a	5E-03	0.1												
															Smim12	1E-04	0.1												
															Ube2j1	6E-02	0.1												
															Apex1	6E-01	0.1												
															Tysnd1	9E-01	0.1												
															Amotl1	2E-03	0.1												
															Anp32a	7E-06	0.1												
															Zfp142	2E-01	0.1												

Limb Mesenchyme						Chondrogenic						Fibroblast						Undefined						Articular/Synovial Fibroblast					
Control			*Notch2* $^{tm1.1Ecan}$			Control			*Notch2* $^{tm1.1Ecan}$			Control			*Notch2* $^{tm1.1Ecan}$			Control			*Notch2* $^{tm1.1Ecan}$			Control			*Notch2* $^{tm1.1Ecan}$		
Gene	*p*	FC	Gene	*p*	FC	Gene	*p*	FC	Gene	*p*	FC	Gene	*p*	FC	Gene	*p*	FC	Gene	*p*	FC	Gene	*p*	FC	Gene	*p*	FC	Gene	*p*	FC
															Hectd1	2E-03	0.1												
															Zfp820	4E-03	0.1												
															Bbip1	1E-03	0.1												
															Cic	8E-03	0.1												
															Prelid1	3E-06	0.1												
															Afap1l2	5E-02	0.1												
															Asb7	8E-03	0.1												
															Gtf2h5	3E-05	0.1												
															Tbk1	1E-02	0.1												
															Dlst	3E-03	0.1												
															Chtop	3E-04	0.1												
															Prkar1a	9E-04	0.1												
															Ube2d1	5E-04	0.1												
															Nek9	3E-03	0.1												
															Vps26a	2E-04	0.1												
															Nsmaf	5E-03	0.1												
															R3hdm1	2E-03	0.1												
															Rrp8	2E-01	0.1												
															Tmcc1	5E-05	0.1												
															Sesn3	2E-01	0.1												
															Ptrhd1	1E-02	0.1												
															Zfp580	8E-02	0.1												
															2810402E24Rik	3E-01	0.1												
															Fzd8	9E-04	0.1												
															Armc9	3E-01	0.1												
															Tmem184b	2E-02	0.1												
															Erf	2E-03	0.1												
															Zzef1	4E-03	0.1												
															Atp6v0a1	1E-02	0.1												
															Appl2	5E-03	0.1												
															Zfand6	2E-03	0.1												
															Carnmt1	3E-04	0.1												
															Elp6	4E-02	0.1												
															Taf6	2E-03	0.1												
															Gpat4	5E-03	0.1												
															Fam172a	2E-04	0.1												
															Chst14	6E-02	0.1												
															Mbtps2	3E-01	0.1												
															Tmem45a	2E-03	0.1												
															Rap1gds1	3E-03	0.1												
															Dnajc24	6E-04	0.1												
															Zfp422	3E-02	0.1												
															Rit1	1E-02	0.1												
															Adnp	8E-04	0.1												
															Zmpste24	9E-04	0.1												
															Ube4b	1E-02	0.1												
															Spaca6	2E-02	0.1												
															Nelfa	8E-02	0.1												
															C1ra	7E-03	0.1												
															Ppp6r2	4E-02	0.1												
															Golga4	2E-01	0.1												
															Borcs5	2E-01	0.1												
															Sp1	8E-02	0.1												
															Cops7b	7E-02	0.1												
															Rex1bd	6E-06	0.1												
															Mycbp	3E-03	0.1												
															Pdap1	3E-05	0.1												
															Tfcp2	2E-01	0.1												
															D10Wsu102e	7E-02	0.1												
															Rhod	4E-02	0.1												
															Pomp	4E-05	0.1												
															Evc	4E-02	0.1												
															1110059G10Rik	5E-02	0.1												
															Tbrg4	3E-01	0.1												

Limb Mesenchyme						Chondrogenic						Fibroblast						Undefined						Articular/Synovial Fibroblast					
Control			*Notch2*$^{tm1.1Ecan}$			Control			*Notch2*$^{tm1.1Ecan}$			Control			*Notch2*$^{tm1.1Ecan}$			Control			*Notch2*$^{tm1.1Ecan}$			Control			*Notch2*$^{tm1.1Ecan}$		
Gene	*p*	FC	Gene	*p*	FC	Gene	*p*	FC	Gene	*p*	FC	Gene	*p*	FC	Gene	*p*	FC	Gene	*p*	FC	Gene	*p*	FC	Gene	*p*	FC	Gene	*p*	FC
															Pttg1	1E-01	0.1												
															Nsd3	6E-03	0.1												
															Slc38a1	1E-04	0.1												
															Zfp62	7E-02	0.1												
															Ash1l	6E-05	0.1												
															Atrip	2E-02	0.1												
															Kpna6	6E-02	0.1												
															Bloc1s1	5E-01	0.1												
															Srp68	2E-02	0.1												
															Nmd3	2E-02	0.1												
															Dnmbp	6E-02	0.1												
															Slc38a9	1E-02	0.1												
															Sltm	1E-03	0.1												
															Tceal9	3E-04	0.1												
															Klhl15	2E-01	0.1												
															Glcci1	5E-02	0.1												
															Tmed4	9E-03	0.1												
															Hip1	9E-03	0.1												
															Gpx1	9E-07	0.1												
															Spag9	4E-05	0.1												
															Rb1cc1	1E-04	0.1												
															Anxa5	9E-10	0.1												
															Arih1	4E-05	0.1												
															Tle3	5E-01	0.1												
															Abcb10	1E-02	0.1												
															Dlg3	8E-02	0.1												
															Mbd5	2E-03	0.1												
															Dhx16	3E-01	0.1												
															Cspp1	8E-04	0.1												
															Ate1	4E-03	0.1												
															Brd1	1E-02	0.1												
															Trim44	1E-03	0.1												
															Psme3	4E-02	0.1												
															Ltbp1	1E-04	0.1												
															Tbc1d32	5E-02	0.1												
															P4htm	2E-01	0.1												
															Arglu1	2E-06	0.1												
															Xrn2	2E-03	0.1												
															2900089D17Rik	4E-01	0.1												
															Platr22	6E-03	0.1												
															Qrsl1	1E-01	0.1												
															Slc44a1	9E-04	0.1												
															Rbm14	4E-03	0.1												
															Nfrkb	2E-01	0.1												
															Tmem176a	5E-05	0.1												
															Zdhhc6	4E-03	0.1												
															Gdi2	3E-05	0.1												
															Cyb5d2	4E-02	0.1												
															Setbp1	1E-02	0.1												
															Hcfc1	2E-03	0.1												
															B3glct	5E-02	0.1												
															Pak3	7E-04	0.1												
															Prkrip1	5E-03	0.1												
															Ptpra	1E-04	0.1												
															Ccnc	1E-02	0.1												
															Zfp414	4E-02	0.1												
															Isca2	5E-01	0.1												
															Prdx2	5E-09	0.1												
															Nelfb	2E-03	0.1												
															Elovl1	2E-03	0.1												
															Kifap3	2E-03	0.1												
															Zbtb44	7E-01	0.1												
															Rab5c	9E-03	0.1												
															Sgce	3E-02	0.1												

Limb Mesenchyme						Chondrogenic						Fibroblast						Undefined						Articular/Synovial Fibroblast					
Control			*Notch2*$^{tm1.1Ecan}$			Control			*Notch2*$^{tm1.1Ecan}$			Control			*Notch2*$^{tm1.1Ecan}$			Control			*Notch2*$^{tm1.1Ecan}$			Control			*Notch2*$^{tm1.1Ecan}$		
Gene	*p*	FC	Gene	*p*	FC	Gene	*p*	FC	Gene	*p*	FC	Gene	*p*	FC	Gene	*p*	FC	Gene	*p*	FC	Gene	*p*	FC	Gene	*p*	FC	Gene	*p*	FC
															Sertad1	3E-03	0.1												
															9930021J03Rik	1E-03	0.1												
															Ifi27	1E-06	0.1												
															Ndufb7	5E-06	0.1												
															Hoxc9	2E-01	0.1												
															Bag6	5E-05	0.1												
															Rrp7a	2E-01	0.1												
															Nrtn	4E-01	0.1												
															Tbcel	2E-01	0.1												
															Chmp1b	1E-01	0.1												
															Mrgbp	8E-02	0.1												
															Akap11	3E-03	0.1												
															Cdkn2b	2E-03	0.1												
															Mgrn1	7E-03	0.1												
															Ppp3cb	2E-02	0.1												
															Cutc	1E-02	0.1												
															Akirin2	3E-02	0.1												
															Atp13a2	2E-01	0.1												
															Vcp	1E-05	0.1												
															Cdkal1	3E-04	0.1												
															Heca	3E-02	0.1												
															Grb2	2E-03	0.1												
															Hltf	9E-03	0.1												
															Dhx36	1E-03	0.1												
															Bcor	9E-02	0.1												
															Ppp1r11	1E-02	0.1												
															Clpx	7E-03	0.1												
															Avil	3E-01	0.1												
															Rhoc	7E-07	0.1												
															Qtrt1	6E-01	0.1												
															2510009E07Rik	5E-01	0.1												
															Impdh1	5E-02	0.1												
															Asb3	1E-02	0.1												
															Mt1	7E-05	0.1												
															Erbb2	7E-03	0.1												
															Pptc7	2E-01	0.1												
															Pfkfb4	2E-01	0.1												
															Gcc2	9E-02	0.1												
															Ubl4a	4E-04	0.1												
															Pigl	1E-01	0.1												
															Immt	9E-05	0.1												
															Wsb1	2E-04	0.1												
															Cpsf1	1E-01	0.1												
															Trub1	6E-01	0.1												
															Atp5b	4E-09	0.1												
															Tbcb	1E-04	0.1												
															Skiv2l	3E-02	0.1												
															Arhgef25	1E-01	0.1												
															Fndc3a	9E-03	0.1												
															Washc5	3E-02	0.1												
															Arl13b	9E-03	0.1												
															Atp5g3	2E-07	0.1												
															Ndel1	1E-01	0.1												
															Dgcr2	1E-02	0.1												
															Zcchc10	7E-02	0.1												
															Mlf2	1E-05	0.1												
															Pqlc3	3E-01	0.1												
															Pip4p2	6E-03	0.1												
															Rpp40	7E-01	0.1												
															Csnk2b	3E-02	0.1												
															Pabpc4l	3E-01	0.1												
															Med12	3E-02	0.1												
															Chd2	2E-03	0.1												
															Med7	3E-01	0.1												

Limb Mesenchyme						Chondrogenic						Fibroblast						Undefined						Articular/Synovial Fibroblast					
Control			*Notch2* $^{tm1.1Ecan}$			Control			*Notch2* $^{tm1.1Ecan}$			Control			*Notch2* $^{tm1.1Ecan}$			Control			*Notch2* $^{tm1.1Ecan}$			Control			*Notch2* $^{tm1.1Ecan}$		
Gene	*p*	FC	Gene	*p*	FC	Gene	*p*	FC	Gene	*p*	FC	Gene	*p*	FC	Gene	*p*	FC	Gene	*p*	FC	Gene	*p*	FC	Gene	*p*	FC	Gene	*p*	FC
															Chp1	2E-03	0.1												
															Epn2	3E-02	0.1												
															Zfp326	5E-04	0.1												
															Fbxl3	2E-02	0.1												
															Polr3f	2E-01	0.1												
															Sbf2	1E-02	0.1												
															Lmbr1l	2E-01	0.1												
															Tor1a	2E-02	0.1												
															Wdr53	9E-02	0.1												
															Relch	8E-03	0.1												
															Scfd2	2E-02	0.1												
															Txndc16	2E-01	0.1												
															Otud7b	2E-02	0.1												
															Zmat2	3E-06	0.1												
															Triap1	9E-03	0.1												
															Vps35	5E-04	0.1												
															Prr12	1E-01	0.1												
															Uxt	7E-02	0.1												
															Asb8	1E-01	0.1												
															Atp5k	2E-07	0.1												
															Klhl7	2E-03	0.1												
															Ttf1	6E-02	0.1												
															Dvl3	2E-02	0.1												
															Ahsa1	4E-03	0.1												
															Tut1	4E-01	0.1												
															Gorab	2E-01	0.1												
															Krit1	5E-02	0.1												
															Pdk2	4E-01	0.1												
															Sumo1	8E-06	0.1												
															Kmt2a	4E-03	0.1												
															Hoxc6	1E-02	0.1												
															Glod4	4E-02	0.1												
															Cdip1	4E-03	0.1												
															Rtl8b	1E-01	0.1												
															Mtrf1l	6E-02	0.1												
															Selenon	2E-03	0.1												
															Rgs7bp	4E-02	0.1												
															Zdhhc13	6E-01	0.1												
															Bmpr1b	2E-03	0.1												
															Desi2	2E-05	0.1												
															Bag1	1E-06	0.1												
															Cpne3	6E-03	0.1												
															Hoxc5	6E-03	0.1												
															Kdm2a	6E-03	0.1												
															Tnpo1	4E-03	0.1												
															Cerk	2E-01	0.1												
															Faf1	5E-04	0.1												
															Psma1	5E-05	0.1												
															Rras	1E-04	0.1												
															Ppil2	1E-02	0.1												
															Cln8	7E-01	0.1												
															Acad8	7E-02	0.1												
															Pip4k2c	4E-02	0.1												
															Zkscan8	1E-01	0.1												
															Atxn7	2E-02	0.1												
															Ywhae	6E-09	0.1												
															Fbxo22	5E-03	0.1												
															Stim1	1E-03	0.1												
															Tmem214	3E-01	0.1												
															Wasl	1E-03	0.1												
															Kansl1	5E-04	0.1												
															Atg7	1E-02	0.1												
															Idnk	9E-02	0.1												
															4931406C07Rik	2E-01	0.1												

Limb Mesenchyme						Chondrogenic						Fibroblast						Undefined						Articular/Synovial Fibroblast					
Control			*Notch2*$^{tm1.1Ecan}$			Control			*Notch2*$^{tm1.1Ecan}$			Control			*Notch2*$^{tm1.1Ecan}$			Control			*Notch2*$^{tm1.1Ecan}$			Control			*Notch2*$^{tm1.1Ecan}$		
Gene	*p*	FC	Gene	*p*	FC	Gene	*p*	FC	Gene	*p*	FC	Gene	*p*	FC	Gene	*p*	FC	Gene	*p*	FC	Gene	*p*	FC	Gene	*p*	FC	Gene	*p*	FC
															Fam131a	9E-02	0.1												
															Psmd1	4E-04	0.1												
															Senp7	3E-02	0.1												
															Rbms1	4E-09	0.1												
															Dhdds	5E-03	0.1												
															Utp25	2E-01	0.1												
															Cxcl16	1E-01	0.1												
															Ldha	2E-06	0.1												
															Htatsf1	2E-03	0.1												
															Dync1h1	6E-05	0.1												
															Mar8	5E-02	0.1												
															Mfsd10	1E-02	0.1												
															Rad23b	4E-04	0.1												
															Peak1	5E-08	0.1												
															Ttc39c	1E+00	0.1												
															Stag2	1E-03	0.1												
															Mrps21	3E-05	0.1												
															Sppl3	4E-02	0.1												
															Mafl	6E-03	0.1												
															Lamb2	8E-05	0.1												
															Brd4	2E-03	0.1												
															Zfp800	3E-02	0.1												
															Chchd6	1E-01	0.1												
															Mrpl48	2E-03	0.1												
															Cct4	2E-04	0.1												
															H2-T23	1E+00	0.1												
															Ireb2	4E-03	0.1												
															Scyl2	5E-01	0.1												
															Prdm2	2E-02	0.1												
															Aff1	8E-04	0.1												
															Gnas	2E-09	0.1												
															Tmem67	2E-01	0.1												
															Gpatch8	2E-03	0.1												
															Gm20342	4E-01	0.1												
															Micos10	2E-07	0.1												
															Fam222b	3E-02	0.1												
															Sirpa	6E-04	0.1												
															Fh1	6E-05	0.1												
															Rhobtb3	3E-04	0.1												
															Rcbtb2	7E-04	0.1												
															Snx13	2E-02	0.1												
															Wdr34	5E-01	0.1												
															Agbl3	6E-01	0.1												
															Mta3	4E-02	0.1												
															Sugp1	2E-01	0.1												
															9530068E07Rik	1E-04	0.1												
															Mrps33	2E-03	0.1												
															Scaf1	3E-02	0.1												
															Dmac1	1E-02	0.1												
															Gpank1	3E-01	0.1												
															Snrpa	4E-03	0.1												
															Plin3	2E-04	0.1												
															Ndst1	4E-03	0.1												
															Crlf2	3E-01	0.1												
															Mad2l2	2E-01	0.1												
															Cpne1	2E-01	0.1												
															Wsb2	5E-03	0.1												
															Ror2	2E-01	0.1												
															Lix1l	1E-03	0.1												
															AI987944	3E-01	0.1												
															Wdr36	2E-02	0.1												
															Apool	5E-03	0.1												
															Fxyd5	2E-04	0.1												
															Zfp523	2E-02	0.1												

Limb Mesenchyme						Chondrogenic						Fibroblast						Undefined						Articular/Synovial Fibroblast					
Control			*Notch2*$^{tm1.1Ecan}$			Control			*Notch2*$^{tm1.1Ecan}$			Control			*Notch2*$^{tm1.1Ecan}$			Control			*Notch2*$^{tm1.1Ecan}$			Control			*Notch2*$^{tm1.1Ecan}$		
Gene	*p*	FC	Gene	*p*	FC	Gene	*p*	FC	Gene	*p*	FC	Gene	*p*	FC	Gene	*p*	FC	Gene	*p*	FC	Gene	*p*	FC	Gene	*p*	FC	Gene	*p*	FC
															Usp11	2E-01	0.1												
															Crem	1E-01	0.1												
															Mllt3	2E-01	0.1												
															Dnajc5	1E-04	0.1												
															Blmh	2E-02	0.1												
															Mettl16	1E-02	0.1												
															Slc9a9	5E-02	0.1												
															Vti1a	1E-03	0.1												
															Gtf3a	3E-04	0.1												
															Bcas2	2E-05	0.1												
															Plcg2	5E-01	0.1												
															Gpbp1	2E-02	0.1												
															Bud23	7E-03	0.1												
															Cebpz	2E-03	0.1												
															Dusp7	2E-01	0.1												
															Adgrl1	7E-03	0.1												
															Rilpl2	1E-02	0.1												
															Mar2	1E-02	0.1												
															Caprin1	1E-04	0.1												
															Llgl1	6E-02	0.1												
															Rdx	8E-07	0.1												
															Zfp317	6E-02	0.1												
															Ap3s1	2E-04	0.1												
															Ube3b	5E-02	0.1												
															Cttnbp2	1E-01	0.1												
															Dctn5	2E-02	0.1												
															Vat1	2E-06	0.1												
															Chd8	3E-02	0.1												
															Tbp	2E-01	0.1												
															Cd99l2	4E-03	0.1												
															Tmem218	2E-01	0.1												
															Efcab14	3E-02	0.1												
															Slc35e1	1E-01	0.1												
															B3gat3	6E-02	0.1												
															Rsu1	3E-05	0.1												
															Zwint	2E-02	0.1												
															Srprb	6E-02	0.1												
															Ptprk	2E-02	0.1												
															Rps6kb1	2E-04	0.1												
															Mrpl32	5E-03	0.1												
															Wdr26	2E-02	0.1												
															Tial1	5E-02	0.1												
															Chrac1	7E-03	0.1												
															Zc3h15	6E-05	0.1												
															Pon2	2E-04	0.1												
															Rnf10	4E-04	0.1												
															Txndc12	5E-05	0.1												
															Ier5l	2E-01	0.1												
															Calcoco1	4E-02	0.1												
															Ip6k1	2E-01	0.1												
															Zswim8	1E-02	0.1												
															Flrt2	6E-02	0.1												
															Rnd3	2E-07	0.1												
															Rad51d	8E-01	0.1												
															Daam2	6E-02	0.1												
															Slc4a2	4E-02	0.1												
															Tmem219	4E-02	0.1												
															Spen	1E-02	0.1												
															Zfand1	3E-02	0.1												
															Tusc3	6E-04	0.1												

Table S2. Differentially expressed genes between *Notch2 $^{tm1.1Ecan}$* and control littermates in mesenchymal, limb mesenchyme, articular/synovial fibroblast, chondrogenic, adipogenic, osteogenic, macrophage and fibroblasts shown in Figure 7C and 7D. The number of cells expressing each gene and the intensity or level of expression is provided.

	**Control**	***Notch2 $^{tm1.1Ecan}$***	**Control**	***Notch2 $^{tm1.1Ecan}$***	***Notch2 $^{tm1.1Ecan}$* / Control**
**Gene**	**Percent Expressed**		**Level of Expression**		**Expression**
Foxc1	71.5	68.4	1.10	1.18	107.3%
Ebf3	34.5	32.1	0.43	0.44	103.2%
Prrx1	88.7	85.4	4.01	3.84	95.9%
Prrx2	69.3	62.0	1.30	1.17	89.8%
Trim2	41.4	33.1	0.40	0.33	83.0%
Gdf5	2.9	3.1	0.02	0.03	129.0%
Pdgfra	35.9	29.3	0.40	0.36	89.4%
Prg4	14.6	12.4	0.24	0.24	101.1%
Cd55	20.7	13.4	0.39	0.22	54.8%
Sox9	74.6	70.7	2.07	2.02	97.7%
Acan	38.0	45.1	1.34	2.31	172.5%
Col2a1	86.0	89.1	26.88	34.97	130.1%
Spp1	44.7	44.4	3.30	3.26	98.8%
Adipoq	1.5	0.6	0.13	0.06	48.4%
Pparg	17.5	17.3	0.19	0.23	120.9%
Grem1	17.3	17.8	0.44	0.66	151.3%
Sp7	0.9	0.9	0.01	0.01	136.6%
Runx2	49.8	43.9	1.01	0.95	94.3%
Lepr	1.5	1.0	0.01	0.01	95.4%
Cxcl12	55.9	50.5	2.65	2.09	79.0%
Bglap	0.5	0.5	0.00	0.00	112.9%
Clec11a	61.8	58.6	1.15	1.15	99.7%
Itga11	16.6	17.5	0.17	0.20	119.9%
Col1a1	92.9	90.3	29.67	28.28	95.3%
Lyz2	8.0	7.4	0.23	0.20	88.6%
Cx3cr1	2.3	2.0	0.02	0.01	93.1%
Calcr	0.0	0.0	0.00	0.00	0.0%
Acp5	5.7	4.9	0.04	0.04	104.8%
Bcl6	51.8	47.6	0.61	0.61	98.8%

	**Control**	***Notch2 $^{tm1.1Ecan}$***	**Control**	***Notch2 $^{tm1.1Ecan}$***	***Notch2 $^{tm1.1Ecan}$* / Control**
**Gene**	**Percent Expressed**		**Level of Expression**		**Expression**
Anxa8	24.3	22.7	0.34	0.31	91.9%
Slurp1	9.4	6.7	0.32	0.23	71.1%
Ociad2	11.5	8.7	0.09	0.07	82.5%
Dkk2	6.5	5.0	0.19	0.11	56.0%
Cxcl2	0.3	0.1	0.01	0.00	48.5%
Cxcl12	55.9	50.5	2.65	2.09	79.0%
Gas6	87.3	84.1	4.53	4.34	95.7%
Acvr2a	37.8	33.0	0.43	0.43	99.7%
Cxcl1	5.4	5.3	0.07	0.07	98.4%
Col15a1	3.7	5.7	0.04	0.07	166.8%
Pi16	1.8	1.5	0.02	0.02	85.3%
Cd55	20.7	13.4	0.39	0.22	54.8%
Cd248	46.4	43.0	1.06	1.06	100.2%
C7	0.2	0.0	0.00	0.00	4.0%
Fmo2	0.1	0.1	0.00	0.00	107.6%
Kitl	22.9	15.1	0.35	0.18	51.1%
Ccl11	0.0	0.0	0.00	0.00	118.3%
Smoc2	31.4	29.7	0.38	0.45	118.4%
Hsd11b1	2.6	2.5	0.02	0.02	116.4%
Prg4	14.6	12.4	0.24	0.24	101.1%
Clic5	0.2	0.2	0.00	0.00	93.0%
Itga6	25.3	24.1	0.25	0.28	113.2%
Gdf5	2.9	3.1	0.02	0.03	129.0%
Pdgfra	35.9	29.3	0.40	0.36	89.4%
Col1a1	92.9	90.3	29.67	28.28	95.3%
Col3a1	97.3	96.2	16.60	18.46	111.2%
Thy1	10.9	8.7	0.13	0.13	97.3%
Mcam	1.2	1.4	0.01	0.02	138.7%

Table S3. Differentially expressed genes in the articular/synovial fibroblast cluster between *Notch2* $^{tm1.1Ecan}$ and control littermate mice. The number of cells expressing each gene in the cluster and the intensity or level of expression is provided.

	Control	*Notch2* $^{tm1.1Ecan}$	Control	*Notch2* $^{tm1.1Ecan}$	*Notch2* $^{tm1.1Ecan}$ / Control
**Gene**	**Percent Expressed**		**Level of Expression**		**Expression**
Eln	46.4	62.0	1.00	1.91	190%
Pla1a	26.3	34.2	0.34	0.45	132%
Pla2g2e	11.3	11.9	0.09	0.12	131%
Mlana	23.0	26.6	0.31	0.41	130%
Dpt	43.4	52.5	0.65	0.84	129%
Htra4	13.4	14.5	0.22	0.26	116%
Olfml2a	27.7	24.9	0.25	0.29	113%
Col5a3	65.2	63.2	0.74	0.83	112%
Prg4	35.9	35.6	0.70	0.74	107%
Rarres2	56.0	53.3	0.60	0.64	106%
Kif1a	42.7	39.2	0.40	0.41	102%
Apcdd1	14.8	12.6	0.11	0.11	101%
Cd248	93.7	87.1	1.67	1.63	98%
Ntn1	41.6	34.4	0.40	0.39	96%
Galnt16	31.7	26.2	0.29	0.28	95%
Creb5	88.8	85.9	1.90	1.75	92%
Serpinb5	26.7	24.4	0.33	0.30	92%
Inhbb	13.4	10.2	0.09	0.08	91%
A330102I10Rik	16.2	11.6	0.14	0.13	90%
Tnxb	56.1	47.7	0.72	0.63	88%
Cd34	72.1	54.5	0.93	0.81	88%
Ly6a	93.7	80.4	2.82	2.46	87%
Gm48053	16.2	13.5	0.12	0.11	87%
Adamts5	92.6	82.5	2.24	1.92	86%
Plpp3	95.3	87.7	2.35	1.98	84%
Adm	63.0	49.7	0.95	0.80	84%
Tspan15	26.4	18.6	0.26	0.22	83%
Pamr1	55.0	44.1	0.76	0.63	83%
Thbd	63.3	49.6	0.94	0.78	82%
Nat8f3	12.0	8.3	0.11	0.09	80%
Myo16	13.7	9.9	0.15	0.12	79%
Emilin2	48.7	31.5	0.44	0.34	77%
Htr2a	15.9	12.1	0.15	0.11	76%
Ly6c1	81.8	59.8	1.57	1.20	76%
Fez1	38.5	30.3	0.36	0.27	76%
Clec3b	73.6	58.3	1.76	1.33	75%
Dpp4	24.9	18.2	0.29	0.21	72%
Agtr2	43.6	29.6	0.58	0.42	72%
Efhd1	22.3	14.0	0.22	0.16	69%
Pdzk1ip1	22.2	15.5	0.19	0.13	68%
Col6a6	12.7	7.3	0.15	0.10	66%
Gda	39.9	24.2	0.41	0.27	66%
Lrrn4cl	27.5	16.4	0.23	0.15	65%
Cd55	67.2	45.1	1.21	0.76	62%
Adgrd1	27.1	19.1	0.31	0.19	62%
Gm15675	27.1	15.0	0.26	0.16	61%
Gm16083	23.3	13.5	0.22	0.13	58%
Tek	18.4	8.9	0.22	0.11	51%
Nova1	15.5	6.8	0.14	0.06	44%
Xdh	17.2	8.3	0.17	0.07	44%

Table S4. Differentially expressed genes (log2FC) between the limb mesenchyme (cluster 3), pooled chondrogenic (clusters 6 and 12), pooled fibroblast (clusters 1, 2, 4 and 11), fibroblasts? (clusters 9 and 13), undefined (clusters 0, 5, 7 and 8) and articular/synovial fibroblast (cluster 10) and all other cell clusters and clusters from epiphyseal chondrocytes from *R26-NICD2* mice transfected with Ad-CMV-Cre (NOTCH2 activated) or Ad-CMV-GFP (control).

Limb Mesenchyme					
Control			NICD2		
Gene	p	FC	Gene	p	FC
Pdzrn4	3E-179	3.0	Tenm1	1E-154	3.0
Tenm1	2E-184	2.9	Pdzrn4	1E-96	2.6
Runx3	4E-79	2.6	Matn4	5E-142	2.5
Matn4	2E-105	2.5	Runx3	2E-59	2.4
Npnt	4E-72	2.3	Otor	1E-38	2.2
Gm10635	1E-55	2.2	Khdrbs2	1E-26	2.2
Eya1	8E-187	2.2	Eya1	3E-158	2.2
Pcp4	2E-18	2.1	Npnt	1E-74	2.2
Cilp2	3E-40	2.0	Gm12002	4E-40	2.1
Zim1	2E-75	1.9	Cobl	7E-144	1.7
Camk4	3E-178	1.9	Camk4	9E-91	1.7
Gm11802	5E-35	1.9	Adgrg6	6E-194	1.7
Adamts3	2E-52	1.9	Stk32b	2E-23	1.7
Otor	9E-52	1.8	Kcnk2	9E-134	1.6
Scube1	2E-31	1.8	Kirrel3	2E-50	1.6
Kirrel3	1E-61	1.8	Lef1	3E-47	1.6
Gm12002	4E-29	1.8	Slc26a7	4E-24	1.6
Stk32b	4E-52	1.8	Gm49708	7E-108	1.6
Faah	2E-29	1.7	Prr5l	2E-21	1.5
Slc17a9	2E-34	1.6	Adamts3	2E-21	1.5
Ptprq	4E-23	1.6	Slitrk5	7E-31	1.5
Fgf21	3E-25	1.6	A830012C17Rik	7E-29	1.5
Lef1	3E-42	1.6	Adam22	1E-45	1.5
Col12a1	6E-263	1.6	Lama1	2E-27	1.5
Kcnk2	1E-156	1.6	1700006J14Rik	2E-17	1.5
Thbs1	6E-168	1.6	Galnt3	1E-33	1.4
Pappa2	1E-54	1.6	Col12a1	2E-227	1.4
Hecw2	1E-128	1.6	Ptprd	7E-128	1.4
Ptchd4	2E-93	1.6	Fmod	4E-153	1.4
Hs3st3b1	2E-28	1.6	Shisa2	2E-48	1.4
Cobl	6E-147	1.5	Zim1	1E-45	1.4
Lmo7	1E-156	1.5	Thbs1	3E-141	1.4
Penk	3E-96	1.5	Tspan7	1E-18	1.4
Ntng1	2E-17	1.5	Penk	8E-70	1.4
Ptprd	5E-141	1.5	Tnfrsf11b	6E-53	1.4
Foxc2	9E-50	1.5	Peg3	4E-126	1.4
Hs3st3a1	4E-52	1.5	Hecw2	1E-71	1.4
Gm49708	5E-79	1.5	Gm16337	9E-23	1.4
Tox	8E-167	1.5	Sh3rf2	1E-42	1.3
Dmd	4E-144	1.4	Cybrd1	4E-21	1.3
Slc16a14	2E-27	1.4	Col11a1	1E-226	1.3
Col11a1	2E-271	1.4	Tox	4E-118	1.3
Erc2	2E-34	1.4	Galnt14	4E-17	1.3
A430027H14Rik	1E-21	1.4	Bmp5	2E-79	1.3
Epha3	5E-42	1.4	Rtl3	8E-90	1.3
Bmp5	2E-112	1.4	Dmd	5E-118	1.3
Aspn	4E-173	1.4	Ptprq	2E-20	1.3
Dock8	1E-56	1.4	Crybg1	6E-29	1.3
Tnfrsf11b	4E-75	1.4	Pappa2	2E-34	1.3
Adgrg6	2E-164	1.4	Fam180a	6E-37	1.3
Tspan7	6E-11	1.4	Mboat2	5E-48	1.3
Col8a2	7E-87	1.4	Ndnf	3E-71	1.3
4930447N08Rik	5E-22	1.4	Cnmd	2E-101	1.3
Galnt3	1E-45	1.4	Fibin	9E-122	1.3
Slitrk5	1E-17	1.4	Ptchd4	2E-43	1.3
Ndnf	4E-74	1.3	Prss23os	1E-09	1.2
Peg3	8E-138	1.3	4930447N08Rik	9E-17	1.2
Shisa2	6E-38	1.3	Kctd16	1E-29	1.2
Cyp26b1	3E-37	1.3	Erc2	1E-15	1.2
Epha2	2E-39	1.3	Lhx9	8E-32	1.2
Fmod	3E-152	1.3	Epha2	5E-28	1.2
Adam22	8E-48	1.3	Myom1	1E-18	1.2
Susd5	7E-73	1.3	Lmo7	1E-95	1.2
Usp29	2E-30	1.3	P3h2	3E-40	1.2
Lhx9	1E-35	1.3	Omd	3E-151	1.2
Nhs	3E-79	1.3	Ebf4	1E-12	1.2
Cpxm2	2E-62	1.3	Col8a2	1E-32	1.2
Gm13986	4E-55	1.3	Usp29	7E-20	1.2
Sh3rf2	9E-55	1.3	Fzd9	1E-38	1.2
Galnt14	7E-16	1.3	Susd5	3E-46	1.2

Chondrogenic					
Control			NICD2		
Gene	p	FC	Gene	p	FC
Ucma	0E+00	4.5	Ucma	0E+00	5.1
Col9a1	0E+00	4.5	Col9a1	0E+00	4.5
Col11a2	0E+00	4.3	Col11a2	2E-264	4.4
Wif1	0E+00	4.2	Wif1	0E+00	4.3
Meltf	7E-134	4.0	Msmp	9E-187	4.2
Acan	5E-261	3.9	Col2a1	0E+00	4.0
Cnmd	0E+00	3.9	Meltf	5E-100	4.0
Col2a1	0E+00	3.9	Cnmd	0E+00	4.0
Tmem213	4E-101	3.8	Acan	2E-188	3.9
Col9a3	0E+00	3.8	Col9a3	0E+00	3.9
Msmp	3E-170	3.7	Scrg1	0E+00	3.8
Cytl1	2E-84	3.6	Ppp1r1b	4E-115	3.7
Chil1	2E-230	3.6	Atp6v0a4	3E-282	3.6
Ppp1r1b	3E-186	3.4	Chil1	1E-213	3.5
Scrg1	0E+00	3.4	Lef1	3E-118	3.4
Fgfr3	5E-129	3.4	Svopl	4E-90	3.4
Lef1	9E-177	3.3	Acox2	3E-80	3.4
Ccn2	0E+00	3.3	Fzd9	1E-249	3.3
Rab11fip4	2E-170	3.3	Gm28153	8E-266	3.2
Atp6v0a4	0E+00	3.3	Fgfr3	1E-76	3.2
Gm28153	0E+00	3.2	Gdf10	1E-151	3.2
Gna14	7E-65	3.2	Ccn2	0E+00	3.1
Acox2	5E-92	3.1	Crispld1	0E+00	3.0
Svopl	3E-122	3.1	Susd5	4E-163	3.0
Susd5	1E-215	3.1	Rab11fip4	6E-128	3.0
Fzd9	1E-294	3.0	Nebl	1E-110	2.9
Cox4i2	2E-245	2.9	Stk26	0E+00	2.8
Crispld1	0E+00	2.9	Gm16070	2E-213	2.8
Omd	0E+00	2.9	Galnt3	6E-116	2.8
Nebl	5E-157	2.9	Mia	0E+00	2.8
Kcna6	5E-94	2.8	Stk32b	4E-81	2.8
St3gal6	0E+00	2.8	Omd	1E-302	2.7
Stk26	0E+00	2.8	St3gal6	6E-260	2.7
Mia	0E+00	2.8	Col9a2	3E-74	2.7
Galnt3	2E-151	2.7	Kcna6	6E-73	2.7
Kcnq5	0E+00	2.7	Kcnq5	1E-215	2.6
Fry	0E+00	2.6	Itga10	4E-164	2.6
Hrct1	3E-126	2.6	Sdk2	4E-193	2.6
Gdf10	2E-184	2.6	Spint2	7E-93	2.6
Sdk2	1E-280	2.5	Hapln1	6E-282	2.5
Hapln1	0E+00	2.5	Rorc	4E-57	2.5
Trpv4	4E-125	2.5	Usp29	2E-60	2.4
Gm16070	1E-233	2.5	Zim1	1E-49	2.4
Col9a2	4E-72	2.4	Fry	3E-258	2.4
Usp29	4E-105	2.4	Pdzrn4	3E-87	2.4
Stk32b	1E-102	2.4	Trpv4	4E-86	2.4
Rorc	3E-61	2.4	B4galnt2	4E-50	2.3
Itga10	1E-202	2.4	Cox4i2	5E-135	2.3
Adgrg6	0E+00	2.3	Tprg	1E-78	2.3
Bmp5	2E-236	2.3	Bmp5	6E-191	2.3
Papss2	3E-195	2.3	Slpi	5E-20	2.2
B4galnt2	7E-54	2.3	Syt1	7E-12	2.2
Fibin	2E-281	2.3	Col11a1	0E+00	2.2
Faah	4E-36	2.3	Il1rapl1	4E-230	2.2
Tspan13	1E-119	2.2	Sorbs2os	9E-66	2.2
Col4a6	1E-39	2.2	1700024B18Rik	1E-39	2.1
Il1rapl1	1E-275	2.2	Syn3	3E-147	2.1
Slc26a7	8E-37	2.2	Tspan13	1E-93	2.1
Spint2	5E-103	2.2	Fam180a	3E-52	2.1
Aldoc	2E-52	2.2	Hrct1	2E-69	2.1
Frzb	5E-84	2.1	Tnfrsf11b	2E-124	2.0
Cobl	3E-227	2.1	Peg3	5E-223	2.0
Camk4	1E-180	2.1	Etv5	1E-76	2.0
Col11a1	0E+00	2.1	Papss2	1E-102	2.0
Gm12002	2E-37	2.1	Slc8a3	8E-22	2.0
Zim1	8E-68	2.1	Camk4	3E-126	2.0
Peg3	1E-295	2.0	Gprc5c	3E-48	2.0
Dock8	2E-146	2.0	Aldoc	9E-27	1.9
Dmd	9E-287	2.0	Sh3rf2	1E-66	1.9
Gprc5c	2E-57	2.0	Eps8l2	8E-140	1.9

Fibroblast					
Control			NICD2		
Gene	p	FC	Gene	p	FC
Ncam2	2E-149	3.7	Sfrp1	0E+00	3.6
Sfrp1	0E+00	3.6	Scg2	2E-69	3.2
Scg2	3E-154	3.4	Ms4a4d	6E-85	3.2
Dpep1	0E+00	3.1	Dpep1	8E-251	3.1
Ms4a4d	7E-173	3.1	Dkk2	3E-226	2.8
Dkk2	0E+00	2.9	Thbs4	3E-161	2.8
Thbs4	0E+00	2.8	Mfap5	0E+00	2.6
Grm7	5E-138	2.7	Mfap4	0E+00	2.6
Mfap5	0E+00	2.6	En1	5E-220	2.5
Npy	2E-103	2.6	Gm16263	3E-76	2.5
Mfap4	0E+00	2.6	Npy	3E-51	2.4
Adh1	1E-171	2.5	Grm7	5E-59	2.4
Slc1a3	0E+00	2.5	Tnmd	5E-244	2.4
Tbxa2r	0E+00	2.4	Slc1a3	0E+00	2.4
En1	0E+00	2.4	Il33	2E-55	2.3
Pdgfd	0E+00	2.4	C1qtnf3	3E-71	2.3
C1qtnf3	3E-147	2.4	Pdgfd	1E-257	2.3
Tnmd	0E+00	2.4	Tbxa2r	2E-222	2.3
Tek	3E-131	2.3	Sult5a1	6E-74	2.2
Gm16263	1E-150	2.3	Fgf10	7E-180	2.2
Postn	0E+00	2.1	Slfn5	1E-144	2.2
Gm34907	1E-136	2.1	C1qtnf7	5E-100	2.1
Steap4	2E-282	2.1	Gm34907	5E-78	2.1
Sncaip	0E+00	2.0	Steap4	2E-121	2.1
Tfpi	6E-215	2.0	Tfpi	1E-104	2.0
Kcp	4E-124	2.0	Postn	0E+00	2.0
Brinp1	6E-122	2.0	Adh1	1E-62	2.0
Fgf10	0E+00	2.0	Kcp	2E-64	2.0
Has2	3E-256	2.0	Wnt16	5E-172	1.9
F3	0E+00	2.0	Ccbe1	2E-92	1.9
Gm15640	2E-107	2.0	Vegfd	0E+00	1.9
C1qtnf7	8E-209	2.0	Mndal	2E-156	1.9
Wnt16	0E+00	2.0	Serping1	0E+00	1.9
Slfn5	3E-262	2.0	Sncaip	4E-139	1.9
Serping1	0E+00	1.9	Ripor3	5E-61	1.9
Opcml	9E-98	1.9	F3	2E-164	1.9
Cygb	4E-190	1.9	Shroom3	2E-275	1.9
Mndal	0E+00	1.9	Has2	8E-138	1.9
Sult5a1	8E-132	1.9	Id4	2E-102	1.8
Ccbe1	2E-173	1.9	Acvr2a	0E+00	1.8
Vegfd	0E+00	1.9	Adgrd1	6E-110	1.8
Acvr2a	0E+00	1.9	Adam33	2E-293	1.8
Adgrd1	9E-200	1.9	Opcml	2E-44	1.8
Igfbp3	0E+00	1.9	Gm15640	3E-45	1.8
Ifi203	2E-226	1.8	Il1rl2	1E-81	1.8
Shroom3	0E+00	1.8	Brinp1	2E-59	1.8
Grem2	4E-176	1.8	Igfbp3	3E-161	1.8
Fbln2	0E+00	1.8	Cxcl14	2E-123	1.8
Prlr	3E-98	1.8	Gm4951	6E-115	1.8
Olfm2	1E-105	1.8	Agmo	3E-122	1.8
Slit2	0E+00	1.8	Ifi203	2E-108	1.8
Thy1	0E+00	1.8	Fbln2	0E+00	1.8
Akap12	0E+00	1.8	A730056A06Rik	1E-47	1.7
Gm4951	6E-246	1.8	Akap12	0E+00	1.7
Ramp2	0E+00	1.8	Ramp2	0E+00	1.7
Cxcl14	4E-245	1.8	Hand2os1	4E-72	1.7
Apod	0E+00	1.8	Pcsk5	1E-119	1.7
Adam33	0E+00	1.8	Olfm2	4E-61	1.7
Ripor3	1E-90	1.8	Ppef1	3E-44	1.7
Il1rl2	3E-165	1.8	Apod	5E-297	1.7
Samd5	0E+00	1.8	Aoc3	4E-102	1.7
Adam12	0E+00	1.7	Thy1	5E-241	1.7
Pcsk5	2E-268	1.7	Lbp	2E-261	1.7
Irx5	8E-107	1.7	Cygb	1E-84	1.7
Pcdh19	0E+00	1.7	Col28a1	4E-91	1.7
Agmo	7E-242	1.7	Pcdh19	3E-259	1.7
Col28a1	7E-174	1.7	Emx2	2E-53	1.7
F830016B08Rik	1E-138	1.7	Atp8b1	1E-175	1.7
Epha4	0E+00	1.7	Galnt17	8E-106	1.6
Sema3d	6E-110	1.7	Grem2	7E-87	1.6

Fibroblast?					
Control			NICD2		
Gene	p	FC	Gene	p	FC
Neil3	0E+00	8.1	Hist1h2af	0E+00	9.1
Hmmr	0E+00	8.0	Esco2	0E+00	8.2
Hist1h2af	0E+00	7.9	Neil3	0E+00	8.0
Cenpf	0E+00	7.7	Pbk	0E+00	7.8
Bub1	0E+00	7.7	Hmmr	0E+00	7.8
Esco2	0E+00	7.5	Ankle1	0E+00	7.8
Hist1h3b	0E+00	7.4	Aspm	0E+00	7.7
Mki67	0E+00	7.4	Cenpf	0E+00	7.6
Ska1	0E+00	7.4	Kif23	0E+00	7.5
Kif4	0E+00	7.3	Hist1h3b	0E+00	7.5
Hist1h3c	0E+00	7.3	Iqgap3	0E+00	7.5
Pbk	0E+00	7.2	Nuf2	0E+00	7.5
Nuf2	0E+00	7.2	Hist1h3c	0E+00	7.4
Plk1	0E+00	7.2	4930558J18Rik	0E+00	7.4
Kif14	0E+00	7.1	Plk1	0E+00	7.3
Aspm	0E+00	7.1	Kif14	0E+00	7.3
Kif18b	0E+00	7.1	Mki67	0E+00	7.2
Dlgap5	0E+00	7.0	Bub1	0E+00	7.2
Ckap2l	0E+00	7.0	Pclaf	0E+00	7.2
Sapcd2	0E+00	7.0	Ccna2	0E+00	7.1
Pclaf	0E+00	7.0	Ska1	0E+00	7.0
2010110K18Rik	2E-234	7.0	Nusap1	0E+00	7.0
Iqgap3	0E+00	7.0	Kif2c	0E+00	6.9
Kif2c	0E+00	6.9	Dlgap5	0E+00	6.9
Kif23	0E+00	6.8	Ckap2l	0E+00	6.8
4930558J18Rik	0E+00	6.8	Mxd3	0E+00	6.8
Nusap1	0E+00	6.8	Ube2c	0E+00	6.8
E2f8	0E+00	6.8	Depdc1b	0E+00	6.8
Ccna2	0E+00	6.7	Pif1	0E+00	6.8
Ankle1	0E+00	6.7	Kif4	0E+00	6.7
Ube2c	0E+00	6.7	Nek2	0E+00	6.7
Pif1	0E+00	6.7	Knl1	0E+00	6.7
Hist1h3g	6E-200	6.7	Troap	0E+00	6.6
Prc1	0E+00	6.7	Birc5	0E+00	6.6
Cep55	0E+00	6.6	Cep55	0E+00	6.6
Birc5	0E+00	6.6	Cenpe	0E+00	6.5
Troap	0E+00	6.6	Sapcd2	0E+00	6.5
Anln	0E+00	6.6	Cdca3	0E+00	6.5
Knl1	0E+00	6.6	Prc1	0E+00	6.5
Shcbp1	0E+00	6.6	Anln	0E+00	6.4
Cenpa	0E+00	6.5	Cenpa	0E+00	6.4
Cdca3	0E+00	6.5	Kif15	0E+00	6.3
Mxd3	0E+00	6.5	Kif11	0E+00	6.3
Nek2	0E+00	6.5	Hist1h2ag	1E-196	6.3
Melk	0E+00	6.4	Ccnb1	0E+00	6.3
Hist1h2ag	6E-240	6.4	Top2a	0E+00	6.3
Ccnb2	0E+00	6.4	Depdc1a	0E+00	6.3
Ccnb1	0E+00	6.4	Sgo1	0E+00	6.2
Pimreg	0E+00	6.3	Pimreg	0E+00	6.2
Cenpe	0E+00	6.3	Ttk	0E+00	6.2
Gm38037	0E+00	6.3	Shcbp1	0E+00	6.2
Depdc1b	0E+00	6.3	Melk	0E+00	6.2
Aunip	0E+00	6.3	Cdca2	0E+00	6.2
Top2a	0E+00	6.2	Kif18b	0E+00	6.2
Sgo2a	0E+00	6.2	Rad51ap1	0E+00	6.1
Ect2	0E+00	6.2	Sgo2a	0E+00	6.1
E2f7	0E+00	6.2	Ect2	0E+00	6.1
Kntc1	0E+00	6.2	Kntc1	0E+00	6.1
Aurkb	0E+00	6.2	Tk1	0E+00	6.1
Tk1	0E+00	6.1	Ccnb2	0E+00	6.1
Tpx2	0E+00	6.1	Ndc80	0E+00	6.1
Cdc25c	0E+00	6.1	E2f8	0E+00	6.0
Rad51ap1	0E+00	6.1	E2f7	0E+00	6.0
Kif15	0E+00	6.0	Exo1	0E+00	5.9
Cdca2	0E+00	6.0	Aunip	0E+00	5.9
Aurka	0E+00	6.0	Tpx2	0E+00	5.9
Ndc80	0E+00	5.9	Hist1h2ab	0E+00	5.9
Lrr1	0E+00	5.9	BC030867	0E+00	5.8
Sgo1	0E+00	5.9	Cdca5	0E+00	5.8
Ttk	0E+00	5.9	Aurka	0E+00	5.7

Undefined					
Control			NICD2		
Gene	p	FC	Gene	p	FC
Evi2a	7E-71	1.8	Myl4	1E-03	1.9
Fgf14	2E-17	1.4	Evi2a	1E-52	1.6
Myl4	5E-01	1.3	Fgf14	4E-22	1.5
St6galnac2	2E-17	1.2	Ccdc3	5E-22	1.2
Mmp3	1E-41	1.1	Mmp3	5E-47	1.2
Gm26917	3E-04	1.1	Pla1a	3E-17	1.1
Ccdc3	6E-12	1.1	Mmp13	2E-08	1.1
Pla1a	1E-19	1.0	Car9	6E-21	1.0
Car9	4E-21	1.0	Creb5	9E-51	1.0
Scn3a	9E-11	1.0	Bend5	5E-18	1.0
Creb5	6E-64	0.9	Cav3	3E-06	0.9
Ntrk3	7E-39	0.9	Frmd5	5E-09	0.9
Lin7a	4E-12	0.9	Ooep	2E-09	0.9
Bend5	2E-16	0.9	Gm28535	7E-16	0.9
Gm28535	3E-16	0.9	Enpep	4E-22	0.9
Tmod1	1E-19	0.8	Tmod1	4E-10	0.9
Rcan2	2E-38	0.8	Cdsn	4E-33	0.9
Cdsn	7E-37	0.8	Ano5	4E-09	0.8
Gm10848	1E-13	0.8	Itih2	6E-40	0.8
Cavin4	4E-06	0.8	Aqp1	2E-34	0.8
Kctd16	2E-02	0.8	Lin7a	8E-08	0.8
Bmp4	6E-53	0.8	Sox5	3E-91	0.8
Ppargc1a	2E-10	0.8	Slc40a1	2E-16	0.8
Klc3	1E-13	0.8	Stac	1E-05	0.7
Cited2	8E-66	0.8	Cited2	1E-40	0.7
Cacnb2	5E-71	0.8	Slc9a3r1	1E-37	0.7
Ano5	4E-08	0.8	Lhx9	1E-08	0.7
Slc9a3r1	8E-16	0.8	Spp1	3E-13	0.7
Hpgd	5E-05	0.8	Rcan2	2E-27	0.7
Gm13269	8E-15	0.8	Mafb	5E-47	0.7
Aldh1a1	2E-36	0.8	Ntrk3	3E-26	0.7
Retreg1	5E-36	0.8	Erg	2E-11	0.7
Enpep	4E-12	0.8	Barx1	1E-50	0.7
Aqp1	6E-43	0.7	Klc3	2E-07	0.7
Cap2	6E-08	0.7	Edil3	1E-94	0.7
Bcl11a	5E-08	0.7	Gm45338	7E-07	0.7
Dusp1	5E-23	0.7	Lpin3	1E-09	0.7
Sox5	8E-109	0.7	Sema5a	6E-19	0.7
Itih2	4E-40	0.7	Gm14636	1E-06	0.6
Gm49708	2E-20	0.7	Car6	2E-04	0.6
C730002L08Rik	2E-07	0.7	Adgrl3	2E-01	0.6
Sema5a	2E-31	0.7	Gm49708	8E-22	0.6
Slc40a1	2E-15	0.7	Itga8	8E-06	0.6
Cav3	3E-08	0.7	Cap2	2E-03	0.6
Thbd	3E-11	0.7	Ets2	2E-16	0.6
Adgrl3	4E-01	0.7	Ell2	4E-69	0.6
Gm15261	2E-13	0.7	Sned1	1E-80	0.6
Ndufa4l2	3E-19	0.7	Arhgap18	3E-31	0.6
Mafb	2E-26	0.7	Aldh1a1	6E-08	0.6
Gm45338	5E-14	0.6	Mme	6E-91	0.6
Cyp27a1	2E-05	0.6	Retreg1	4E-16	0.6
Gm13483	6E-11	0.6	Dkk3	3E-79	0.6
Lctl	7E-11	0.6	Dhrs9	2E-30	0.6
Arhgap18	9E-30	0.6	Cacnb2	5E-36	0.6
Lhx9	4E-07	0.6	Ank	6E-02	0.6
Hdac9	3E-09	0.6	Ndufa4l2	1E-15	0.6
Ntn4	7E-06	0.6	4933400C23Rik	2E-05	0.6
Barx1	5E-51	0.6	Hopx	2E-10	0.6
Clip4	1E-13	0.6	Masp1	4E-18	0.6
Efna1	2E-08	0.6	Mettl7a1	1E-16	0.6
Adra1b	8E-07	0.6	Ddit4l	1E-39	0.6
Car6	7E-06	0.6	Prg4	1E-11	0.6
Gm15222	3E-06	0.6	Plcg2	4E-05	0.6
Gm14636	2E-09	0.6	Rnd2	3E-02	0.6
Masp1	3E-21	0.6	Htra1	4E-56	0.6
Edil3	2E-86	0.6	Cx3cl1	3E-07	0.6
Rab3b	2E-05	0.6	Epas1	1E-05	0.6
Dkk3	7E-109	0.6	Saa3	3E-22	0.6
Ell2	2E-74	0.6	B230206L02Rik	7E-03	0.6
Gm12153	6E-04	0.6	Rassf2	3E-17	0.6

Articular/Synovial Fibroblast					
Control			NICD2		
Gene	p	FC	Gene	p	FC
Htra4	2E-141	3.9	Htra4	2E-116	4.0
F13a1	3E-43	3.7	Mlana	6E-62	3.4
Aldh1a2	7E-46	3.4	F13a1	2E-29	3.2
Crocc2	4E-82	3.0	Sfrp4	2E-40	3.1
Mlana	2E-51	3.0	Gm15675	8E-86	3.0
Dlgap2	1E-50	3.0	Crocc2	8E-38	2.9
Galnt9	5E-164	2.9	Aldh1a2	4E-34	2.8
Gm15675	9E-129	2.9	Tnxb	3E-177	2.8
Clec3b	1E-149	2.7	Avpr1a	3E-35	2.7
Sfrp4	6E-35	2.7	Ptchd1	6E-25	2.7
Tnxb	5E-213	2.7	Galnt9	7E-80	2.7
Gda	1E-186	2.6	Myo16	2E-31	2.7
Ppl	2E-177	2.6	Clec3b	6E-119	2.6
Myo16	2E-25	2.6	Gda	2E-86	2.6
Pla2g2e	7E-30	2.5	Cd55	3E-137	2.4
Tspan15	8E-43	2.4	Pla2g2e	3E-20	2.4
Pgr	7E-25	2.4	5830408C22Rik	7E-27	2.4
Gm16083	1E-82	2.4	Ppl	6E-103	2.4
Cd55	5E-175	2.4	Gm15987	4E-50	2.4
Cmtm8	3E-43	2.4	Rragd	1E-32	2.3
Gm15987	8E-49	2.4	Cmtm8	3E-30	2.3
Vav3	9E-68	2.3	Dlgap2	1E-20	2.3
Stac2	1E-45	2.3	Grem1	2E-24	2.2
Sparcl1	2E-122	2.3	Kif1a	4E-71	2.1
Lrrn4cl	9E-30	2.3	A330102I10Rik	3E-11	2.1
Kif1a	6E-110	2.3	Slfn5	1E-55	2.1
5830408C22Rik	1E-31	2.3	Tnik	7E-53	2.0
Lnx1	5E-33	2.2	Vav3	3E-42	2.0
Fam124a	1E-30	2.2	Rarres2	6E-68	2.0
Susd2	1E-43	2.2	Galnt16	8E-11	2.0
Slfn5	6E-94	2.2	Gm32014	5E-10	2.0
Nat8f3	8E-52	2.1	Xdh	3E-35	2.0
Tnik	6E-60	2.1	Gm16083	3E-28	2.0
Chst15	3E-45	2.1	Susd2	1E-29	1.9
Gm12796	3E-21	2.1	Creb5	1E-95	1.9
Olfml2a	2E-54	2.0	Ly6c1	2E-56	1.9
Gm47710	2E-32	2.0	Sparcl1	2E-72	1.9
Aif1l	2E-15	2.0	Fez1	5E-44	1.9
Serpina3g	3E-18	2.0	Nat8f3	7E-25	1.9
Sncg	5E-31	2.0	Pamr1	8E-31	1.8
Clec2g	8E-21	2.0	Chst15	3E-23	1.8
Xdh	4E-66	2.0	Stac2	4E-19	1.8
Creb5	9E-93	2.0	Fam124a	2E-15	1.8
Ly6c1	7E-73	1.9	Zbp1	5E-14	1.8
Ednrb	7E-17	1.9	Hp	3E-49	1.8
Rarres2	2E-101	1.9	Rad51b	1E-101	1.8
Galnt16	8E-14	1.9	Lrp4	3E-22	1.7
Pamr1	3E-31	1.9	Palm3	4E-21	1.7
Sned1	3E-205	1.9	Gm47710	3E-14	1.7
Plekha2	6E-73	1.9	Cdk15	1E-13	1.7
Rragd	3E-17	1.9	Phkg1	7E-12	1.7
A330102I10Rik	1E-19	1.9	Prg4	2E-12	1.7
Plin4	7E-53	1.9	Sncg	2E-06	1.7
Fez1	8E-60	1.8	Selenbp1	1E-49	1.7
Rad51b	2E-132	1.8	Serpinb1a	6E-42	1.7
Col6a6	2E-20	1.8	Ly6a	4E-92	1.7
Grem1	2E-18	1.8	H2-Q6	2E-07	1.7
Serpinb1a	2E-99	1.8	Plekha2	9E-44	1.7
Srgn	4E-36	1.8	Ggt5	1E-52	1.6
Zbp1	3E-14	1.8	Ackr3	2E-20	1.6
Ly6a	4E-143	1.8	Gm28535	2E-14	1.6
Palm3	2E-41	1.8	Procr	6E-27	1.6
Phkg1	4E-20	1.7	Rgs2	1E-28	1.6
Pla1a	4E-23	1.7	Sned1	3E-133	1.6
Procr	2E-56	1.7	Sfrp2	3E-74	1.6
Selenbp1	1E-83	1.7	Nbl1	4E-70	1.6
Ifi207	5E-22	1.7	Mustn1	8E-24	1.5
Map7d2	2E-19	1.7	Gm7160	5E-08	1.5
Sdk1	2E-71	1.7	Plin4	1E-21	1.5
Gm28710	2E-17	1.7	Timp4	1E-10	1.5

Limb Mesenchyme

Control Gene	p	FC	NICD2 Gene	p	FC
Lama3	6E-19	1.3	Gm15414	3E-13	1.1
Crybg1	2E-18	1.3	Itm2a	7E-146	1.1
Cybrd1	4E-29	1.3	Epha3	4E-24	1.1
Adamts6	1E-51	1.2	Hs3st3a1	3E-15	1.1
Plxnc1	4E-28	1.2	Gm14547	1E-12	1.1
Slc26a7	2E-21	1.2	Aqp1	3E-30	1.1
Ccdc24	7E-16	1.2	Mei4	6E-16	1.1
Fndc1	1E-67	1.2	Rpl39l	2E-20	1.1
A830012C17Rik	5E-32	1.2	Lama3	1E-35	1.1
Tmtc2	2E-36	1.2	Rtl1	8E-20	1.1
P3h2	2E-38	1.2	Slc12a2	1E-63	1.1
Gm14547	1E-20	1.2	Tprg	3E-30	1.1
Sh3tc2	3E-16	1.2	Slc7a3	9E-25	1.1
Extl1	1E-15	1.2	Prkg1	2E-134	1.1
Runx2	2E-78	1.2	Fndc1	8E-56	1.1
Trp63	6E-62	1.2	C230038L03Rik	3E-30	1.1
Slc7a3	2E-40	1.2	Cpxm2	1E-38	1.0
Omd	7E-176	1.2	Trp63	2E-41	1.0
Wscd2	1E-27	1.2	Ntng1	1E-06	1.0
Plxnb1	4E-22	1.2	Tfap2a	1E-14	1.0
Sorl1	2E-29	1.2	Gnao1	4E-23	1.0
Foxd2	2E-19	1.2	Mest	6E-117	1.0
Gm49267	5E-34	1.1	Pcdh11x	5E-73	1.0
Shank1	1E-35	1.1	Scx	4E-39	1.0
Fzd9	9E-56	1.1	Thbd	2E-44	1.0
Slc12a2	6E-80	1.1	Ablim1	3E-49	1.0
Mettl24	7E-21	1.1	Ccser1	2E-48	1.0
Sema3a	3E-58	1.1	Myo1b	2E-70	1.0
Itm2a	5E-122	1.1	Hsd17b1	6E-33	1.0
Chac1	2E-58	1.1	Chac1	3E-38	1.0
Hsd17b1	2E-50	1.1	Pakap	8E-80	1.0
Kctd16	1E-26	1.1	Dach2	6E-18	1.0
Rflnb	2E-25	1.1	Pag1	6E-46	1.0
Fzd6	4E-23	1.1	Capn6	1E-56	1.0
Clec11a	2E-96	1.1	Dlx5	8E-15	1.0
Rpl39l	6E-24	1.1	Clmn	2E-37	1.0
Zbtb18	3E-30	1.1	Nhs	5E-64	1.0
Pdgfc	7E-49	1.1	Mlip	2E-23	1.0
Rtl3	1E-49	1.1	Foxc2	2E-20	1.0
Capn6	2E-81	1.0	Dock8	1E-20	1.0
Cnmd	7E-77	1.0	Kcnt2	3E-35	1.0
Kctd15	2E-34	1.0	Adm2	3E-21	1.0
Tenm3	6E-103	1.0	Kcnma1	6E-17	1.0
Fibin	3E-102	1.0	Tasp1	3E-65	1.0
Ppfibp2	9E-30	1.0	Runx2	2E-38	1.0
Pde5a	2E-34	1.0	Aspn	9E-90	1.0
Pgm5	4E-17	1.0	Wscd2	3E-20	1.0
Klf5	1E-16	1.0	Tgfb2	3E-111	1.0
Aqp1	2E-58	1.0	Rab27b	6E-19	1.0
Gm15663	7E-16	1.0	Tenm3	4E-86	1.0
Mlip	6E-39	1.0	Ltbp1	6E-81	1.0
BC006965	1E-16	1.0	Itgbl1	2E-91	1.0
Prkg1	1E-125	1.0	Cxadr	2E-11	0.9
Mei4	6E-23	1.0	Calhm5	6E-44	0.9
Cdsn	8E-42	1.0	Slc6a9	2E-35	0.9
Mest	3E-116	1.0	Shank1	1E-18	0.9
Nog	5E-19	1.0	Gm49226	1E-18	0.9
Pcdh11x	2E-73	1.0	Apbb2	4E-116	0.9
Ooep	8E-37	1.0	Hs6st2	4E-41	0.9
Fzd5	6E-37	1.0	Cyyr1	5E-08	0.9
Spata13	3E-20	1.0	Sorbs2os	2E-23	0.9
Hs6st2	4E-73	1.0	Zfp462	7E-46	0.9
Mboat2	2E-40	1.0	Sorl1	8E-18	0.9
Tmem56	7E-12	1.0	Lrrn3	2E-17	0.9
Slpi	1E-15	1.0	Pde5a	4E-23	0.9
Fras1	9E-12	1.0	Flrt1	2E-23	0.9
Ltbp1	3E-84	1.0	Tenm4	4E-72	0.9
1700024B18Rik	9E-15	1.0	Clec11a	3E-68	0.9
Kcnma1	3E-20	1.0	Gm15655	4E-10	0.9
Lrrn3	1E-23	1.0	Fgd6	9E-21	0.9
Ogn	8E-178	1.0	Dpysl4	2E-16	0.9
Cnn2	3E-93	1.0	Ctnnd1	8E-67	0.9
Scx	3E-55	1.0	Nt5e	2E-19	0.9

Chondrogenic

Control Gene	p	FC	NICD2 Gene	p	FC
Gm13944	8E-39	2.0	D630045J12Rik	4E-131	1.9
Xylt1	2E-123	1.9	4933400C23Rik	3E-94	1.9
Pdzrn4	6E-109	1.9	Gm10848	1E-37	1.9
Tprg	4E-108	1.9	Plxnb1	5E-34	1.9
Wwp2	4E-138	1.9	Comp	5E-206	1.8
Gm12473	1E-30	1.9	Gm13919	6E-66	1.8
Rnf39	9E-38	1.9	Wwp2	1E-107	1.8
Slpi	3E-16	1.9	Dock8	9E-89	1.8
Fmod	4E-307	1.9	Cybrd1	9E-33	1.8
Rerg	3E-92	1.9	Plcg2	2E-37	1.8
Gm28054	1E-34	1.8	Rerg	3E-90	1.8
Syn3	2E-190	1.8	Frzb	2E-50	1.8
Kcnc3	2E-45	1.8	Slc40a1	4E-35	1.8
Nalcn	6E-23	1.8	Stxbp2	7E-43	1.8
Gm13919	5E-66	1.8	Dmd	6E-177	1.8
Syt1	5E-26	1.8	Nog	1E-22	1.8
4933400C23Rik	9E-124	1.8	Fmod	1E-220	1.8
Foxd1	7E-65	1.8	Reck	6E-169	1.8
Plcg2	4E-39	1.8	Kcnc3	6E-25	1.8
Npas3	3E-253	1.8	Zfp385b	2E-73	1.7
Eps8l2	2E-145	1.8	Trp63	2E-71	1.7
Shroom2	9E-33	1.7	Timp3	1E-146	1.7
D630045J12Rik	3E-153	1.7	Fibin	1E-169	1.7
Rbp4	1E-164	1.7	Foxd1	2E-44	1.7
Sh3rf2	8E-90	1.7	Erg	2E-77	1.7
P3h2	3E-117	1.7	Gm12002	2E-26	1.7
Comp	4E-202	1.7	P3h2	5E-80	1.7
Sorbs2os	4E-53	1.7	Klf5	3E-28	1.7
Tspan2	2E-51	1.7	Slc17a9	6E-27	1.7
Klf5	2E-34	1.7	Prkcb	4E-16	1.7
Sema3e	2E-94	1.7	BC006965	1E-20	1.7
Nog	4E-45	1.7	Platr22	7E-30	1.6
C430049B03Rik	2E-75	1.7	Xylt1	4E-76	1.6
Plxnb1	5E-34	1.6	Klc3	9E-42	1.6
Erg	7E-82	1.6	Sorbs2	2E-91	1.6
Fam180a	9E-39	1.6	Cp	6E-80	1.6
Ecm2	2E-100	1.6	Id1	9E-117	1.6
Thsd4	4E-17	1.6	Gm12473	2E-25	1.6
Slc8a3	7E-26	1.6	Cd24a	5E-45	1.6
Mgat4a	2E-41	1.6	Gm13269	3E-38	1.6
Cybrd1	1E-35	1.6	Cobl	1E-116	1.6
Ccdc68	1E-79	1.6	Chrnb1	3E-21	1.6
Smoc1	2E-103	1.6	Nt5e	6E-45	1.6
Edar	3E-28	1.6	Shisa2	9E-42	1.6
Tox	6E-186	1.6	Abtb2	2E-69	1.6
Prkcz	1E-31	1.6	C430049B03Rik	7E-61	1.6
Ldlrad3	2E-140	1.6	Fbln7	1E-124	1.6
Slc17a9	1E-24	1.6	Npr2	3E-100	1.6
Id1	2E-149	1.6	Kank1	9E-81	1.6
Reck	9E-172	1.6	Osgin2	2E-64	1.5
Nt5e	2E-49	1.5	Mlip	5E-46	1.5
Armh4	3E-156	1.5	Tox	5E-138	1.5
Pam	0E+00	1.5	Pcdh9	7E-121	1.5
Zfp385b	4E-94	1.5	Rbp4	9E-119	1.5
Abtb2	1E-86	1.5	Pdzk1ip1	4E-25	1.5
Pcdh9	6E-181	1.5	Smoc1	5E-109	1.5
Sgk3	1E-94	1.5	Isg20	3E-88	1.5
Etv5	2E-56	1.5	Mtus2	5E-24	1.5
Kank1	2E-119	1.5	Sox9	3E-148	1.5
Shisa2	8E-46	1.5	Tenm1	1E-20	1.5
Cd24a	6E-40	1.5	Ldlrad3	1E-117	1.5
Nrp2	5E-264	1.5	Edar	2E-24	1.5
Raph1	4E-122	1.5	Npas3	4E-145	1.5
Fbln7	5E-103	1.5	Map3k5	8E-37	1.5
Isg20	4E-110	1.5	C730002L08Rik	5E-16	1.5
Osgin2	1E-101	1.5	Ecm2	1E-59	1.5
Sntg2	2E-32	1.5	Gm45025	1E-32	1.5
Timp3	4E-173	1.4	Sap30	4E-100	1.5
Axin2	2E-24	1.4	Nrp2	2E-215	1.5
Tnfrsf11b	1E-173	1.4	S100b	2E-78	1.5
Gm45025	3E-38	1.4	Gm19705	6E-26	1.5
Brwd3	9E-28	1.4	Sema3e	2E-46	1.4
Sorbs2	1E-96	1.4	Spp1	1E-18	1.4

Fibroblast

Control Gene	p	FC	NICD2 Gene	p	FC
Lbp	0E+00	1.7	Slit2	1E-232	1.6
Ppef1	5E-91	1.7	Samd5	4E-194	1.6
Hs6st3	1E-101	1.7	Lgr5	8E-155	1.6
Emx2	1E-103	1.7	Gpr88	2E-62	1.6
Qpct	3E-161	1.6	Cpz	3E-69	1.6
Col14a1	0E+00	1.6	Hs6st3	1E-48	1.6
Galnt17	5E-205	1.6	Sema3d	2E-56	1.6
Cpz	2E-144	1.6	Epha4	8E-228	1.6
Cyp7b1	0E+00	1.6	Prlr	1E-39	1.6
Tagln	2E-254	1.6	Col14a1	0E+00	1.6
Id4	7E-134	1.6	Tfpi2	3E-31	1.6
A730056A06Rik	3E-101	1.6	Nav3	3E-61	1.5
Hand2os1	5E-142	1.6	Tshz2	0E+00	1.5
Tshz2	0E+00	1.6	Qpct	2E-72	1.5
S1pr3	3E-192	1.6	Nhsl2	6E-133	1.5
Slc27a6	2E-79	1.6	Kng2	2E-106	1.5
Aoc3	7E-198	1.6	Kcnip4	2E-230	1.5
Dlk1	3E-213	1.6	Cyp7b1	1E-190	1.5
Nav3	1E-141	1.6	Hgf	6E-241	1.5
Serpine1	1E-266	1.6	Gm13212	2E-49	1.5
Gpr39	5E-75	1.6	F830016B08Rik	8E-55	1.5
AW551984	1E-81	1.6	Ptn	3E-188	1.5
Nhsl2	1E-241	1.6	Thsd7a	3E-181	1.5
Gm13212	9E-100	1.5	Acvrl1	9E-97	1.5
Fbn1	0E+00	1.5	Igf1	6E-223	1.5
Atp8b1	3E-301	1.5	Adam12	2E-225	1.5
Gpr88	2E-144	1.5	Egfl6	1E-230	1.5
Acvrl1	6E-206	1.5	Slc27a6	1E-30	1.5
Plcb4	6E-248	1.5	Cyp26b1	7E-49	1.5
Crabp1	6E-98	1.5	Shc2	3E-49	1.5
Arhgap20	0E+00	1.5	Cacnb4	1E-24	1.5
Lgr5	1E-304	1.5	Emilin2	4E-223	1.5
9330154J02Rik	5E-70	1.5	Lrrtm3	3E-36	1.5
Ptn	0E+00	1.5	Parm1	2E-62	1.5
Tnfaip8l3	5E-99	1.5	Chl1	7E-27	1.5
Htr2a	6E-63	1.5	Igfbp6	4E-270	1.5
Thsd7a	0E+00	1.5	S1pr3	3E-101	1.5
Igf1	0E+00	1.5	Dlk1	4E-95	1.4
Ccl7	0E+00	1.5	Arhgap20	3E-246	1.4
Dact1	5E-127	1.5	Col8a1	2E-51	1.4
Shc2	3E-97	1.5	Ccl7	8E-212	1.4
Kcnip4	0E+00	1.5	Dysf	3E-55	1.4
Kng2	9E-202	1.5	Serpine1	3E-118	1.4
Hgf	0E+00	1.5	Tagln	5E-130	1.4
Chl1	9E-54	1.5	Irx5	8E-36	1.4
Egfl6	0E+00	1.5	Ctnna3	2E-26	1.4
Cacnb4	5E-65	1.4	Dock10	9E-132	1.4
Col8a1	9E-72	1.4	Dact1	2E-52	1.4
Tmcc3	2E-77	1.4	9330154J02Rik	2E-33	1.4
Mid1	0E+00	1.4	Clec3b	3E-267	1.4
Dysf	2E-123	1.4	Fbn1	0E+00	1.4
Igfbp6	0E+00	1.4	Cd34	2E-98	1.4
Pde8b	4E-59	1.4	Smoc2	2E-170	1.4
Apbb1ip	0E+00	1.4	Ly6a	0E+00	1.4
Dock10	4E-237	1.4	Plcb4	5E-100	1.4
Slc16a9	6E-58	1.4	Pde8b	1E-27	1.4
Gstm2	0E+00	1.4	Ebf2	1E-128	1.4
Ebf2	1E-223	1.3	Tnfaip8l3	8E-48	1.4
Emx2os	1E-91	1.3	Gstm2	0E+00	1.4
Crabp2	3E-254	1.3	Bmp6	2E-30	1.3
Cthrc1	0E+00	1.3	Ksr1	2E-42	1.3
Parm1	8E-100	1.3	Mid1	5E-265	1.3
Fndc5	1E-67	1.3	Lingo1	9E-35	1.3
Lama2	6E-197	1.3	Pth1r	6E-86	1.3
Ebf1	0E+00	1.3	Cmya5	2E-78	1.3
Cmya5	2E-171	1.3	Tmcc3	1E-33	1.3
Ksr1	8E-99	1.3	Emx2os	4E-42	1.3
Entpd2	4E-133	1.3	Trpm3	6E-37	1.3
Clec3b	0E+00	1.3	Cthrc1	6E-223	1.3
Pax1	1E-64	1.3	Lama2	4E-104	1.3
4833427G06Rik	3E-104	1.3	Slc16a9	5E-24	1.3
Cd34	5E-219	1.3	Apbb1ip	1E-239	1.2
Emilin2	0E+00	1.3	Ebf1	0E+00	1.2

Fibroblast?

Control Gene	p	FC	NICD2 Gene	p	FC
Kif11	0E+00	5.9	Knstrn	0E+00	5.7
BC030867	0E+00	5.9	Fbxo5	0E+00	5.7
Exo1	0E+00	5.9	Mis18bp1	0E+00	5.7
Knstrn	0E+00	5.9	Spc24	0E+00	5.6
Cdca5	0E+00	5.8	Gm38037	0E+00	5.6
Mis18bp1	0E+00	5.8	Cdc20	0E+00	5.6
Bub1b	0E+00	5.8	Asf1b	0E+00	5.6
Hist1h2bm	0E+00	5.7	Aurkb	0E+00	5.6
Cdc20	0E+00	5.7	Kif20b	0E+00	5.6
Depdc1a	0E+00	5.7	Lrr1	0E+00	5.5
Mcm10	0E+00	5.7	Eme1	0E+00	5.5
Fbxo5	0E+00	5.7	Cdc25c	0E+00	5.5
Ska3	0E+00	5.7	Cdca8	0E+00	5.5
Kif20b	0E+00	5.6	Gm2788	3E-303	5.5
Cdca8	0E+00	5.6	Espl1	0E+00	5.4
Spc24	0E+00	5.6	Cenph	0E+00	5.4
Clspn	0E+00	5.6	Kif20a	0E+00	5.4
Neurl1b	0E+00	5.6	Cenpm	0E+00	5.4
Hist1h1a	0E+00	5.5	Mcm10	0E+00	5.4
Cenpi	0E+00	5.5	Hist1h2an	0E+00	5.4
Asf1b	0E+00	5.5	Cdkn3	0E+00	5.4
Hist1h2ab	0E+00	5.5	Diaph3	0E+00	5.4
Prr11	0E+00	5.5	Polq	0E+00	5.3
Brip1	0E+00	5.4	Arhgef39	3E-287	5.3
Cdkn3	0E+00	5.4	Ercc6l	0E+00	5.3
Fanci	0E+00	5.4	Hist1h1a	0E+00	5.3
Cenph	0E+00	5.4	Ccnf	0E+00	5.3
Cenpm	0E+00	5.3	Clspn	0E+00	5.3
Arhgef39	2E-298	5.3	Hist1h2bm	0E+00	5.3
Ncaph	0E+00	5.3	Fanci	0E+00	5.3
Ercc6l	0E+00	5.3	Spc25	0E+00	5.3
Espl1	0E+00	5.3	Cit	0E+00	5.2
Kif20a	0E+00	5.3	Bub1b	0E+00	5.2
Eme1	0E+00	5.3	Rrm2	0E+00	5.2
Erfe	4E-277	5.3	Hist1h2ai	0E+00	5.2
Brca1	0E+00	5.2	Prr11	0E+00	5.2
Uhrf1	0E+00	5.2	Ncaph	0E+00	5.2
Rrm2	0E+00	5.2	Hist1h2ap	0E+00	5.2
Hist1h2ai	0E+00	5.2	Brca1	0E+00	5.2
Spdl1	0E+00	5.2	Uhrf1	0E+00	5.2
Bard1	0E+00	5.1	Spdl1	0E+00	5.1
Diaph3	0E+00	5.1	Cenpi	0E+00	5.1
Polq	0E+00	5.1	Tacc3	0E+00	5.1
Spc25	0E+00	5.1	Brip1	0E+00	5.1
Eldr	0E+00	5.1	Phf19	0E+00	5.1
Fam83d	0E+00	5.1	Ska3	0E+00	5.1
Fanca	0E+00	5.1	Nsl1	0E+00	5.1
Ccne2	0E+00	5.1	Ncapg	0E+00	5.1
Gen1	0E+00	5.1	Xkr5	7E-280	5.0
Ccnf	0E+00	5.1	Eldr	0E+00	5.0
Tacc3	0E+00	5.1	Gas2l3	0E+00	5.0
Ckap2	0E+00	5.1	Gen1	0E+00	5.0
Hist1h2ap	0E+00	5.0	Bard1	0E+00	5.0
Ccdc18	0E+00	5.0	Ccne2	0E+00	5.0
Racgap1	0E+00	4.9	Fam83d	0E+00	5.0
Cdc6	0E+00	4.9	Kif22	0E+00	5.0
Phf19	0E+00	4.9	Mybl2	0E+00	4.9
Nsl1	0E+00	4.9	Cenpu	0E+00	4.9
Cit	0E+00	4.9	Hist1h2bb	6E-300	4.9
Haspin	0E+00	4.9	Hist1h2ak	9E-277	4.9
Ncapg2	0E+00	4.8	Gm47207	7E-200	4.8
Ncapg	0E+00	4.8	Hist1h1b	0E+00	4.8
Recql4	1E-276	4.8	Ckap2	0E+00	4.8
Xkr5	0E+00	4.8	Haspin	0E+00	4.8
Efcab11	0E+00	4.7	Racgap1	0E+00	4.8
Gm2788	5E-268	4.7	Spag5	0E+00	4.8
Gm47207	3E-216	4.7	Kifc5b	0E+00	4.8
Hist1h2an	2E-281	4.7	Ccdc18	0E+00	4.8
Gas2l3	0E+00	4.7	Mns1	0E+00	4.8
Spag5	0E+00	4.7	Lockd	0E+00	4.7
Mns1	0E+00	4.7	Fanca	0E+00	4.7
Kifc5b	0E+00	4.6	Kifc1	0E+00	4.7
Oip5	0E+00	4.6	Hist1h2bj	0E+00	4.7

Undefined

Control Gene	p	FC	NICD2 Gene	p	FC
Spp1	2E-14	0.6	Qsox1	2E-53	0.6
Rassf2	2E-16	0.6	L3mbtl3	5E-17	0.6
Gm15564	7E-02	0.6	Cst6	3E-22	0.5
Mettl7a1	3E-28	0.6	Gm32618	6E-09	0.5
Ampd3	2E-13	0.6	Sdc2	2E-112	0.5
Gm28905	1E-07	0.6	Ampd3	3E-10	0.5
Osr2	2E-18	0.6	Ephb6	1E-07	0.5
Carns1	3E-07	0.6	Mt2	8E-26	0.5
Lpin3	9E-09	0.6	Tgfb2	8E-90	0.5
Qsox1	1E-66	0.6	Ogn	5E-143	0.5
Ehd3	6E-06	0.6	Rin1	1E-03	0.5
Sdc2	7E-108	0.6	Dusp1	2E-16	0.5
Prg4	8E-16	0.6	9530026P05Rik	3E-12	0.5
Zfp385b	4E-05	0.6	Cacnb1	2E-02	0.5
Htra1	5E-54	0.6	Rcor2	2E-01	0.5
Tgfb2	6E-83	0.6	Mkx	5E-09	0.5
Cst6	3E-21	0.5	Trp63	3E-03	0.5
Pik3r1	2E-42	0.5	Ccdc30	1E-03	0.5
Dhrs9	2E-30	0.5	Sphk1	1E-04	0.5
Rin1	4E-06	0.5	Rasl11a	2E-06	0.5
Ldhb	4E-19	0.5	Ppargc1a	9E-04	0.5
Ccser1	2E-27	0.5	C230038L03Rik	2E-05	0.5
Erg	8E-15	0.5	Cntnap2	4E-04	0.5
Gdpd1	1E-08	0.5	Zfp385b	3E-02	0.5
Ifit3b	7E-01	0.5	Sox6	5E-89	0.5
Nat14	5E-03	0.5	Pi15	5E-02	0.5
Stbd1	3E-07	0.5	Vit	5E-02	0.5
Ddit4l	5E-22	0.5	Efna1	4E-03	0.5
Cx3cl1	1E-04	0.5	Gm15261	1E-04	0.5
Prkg2	4E-02	0.5	Tspan13	2E-04	0.5
Rerg	8E-12	0.5	Adgrg2	6E-19	0.5
Enpp1	4E-12	0.5	Gpsm2	2E-27	0.5
Tnfrsf11b	2E-02	0.5	Ostn	4E-06	0.5
Nptx1	1E-04	0.5	Hmox1	2E-09	0.5
Dab2	1E-49	0.5	Gdpd1	7E-04	0.5
Ifit3	9E-04	0.5	Enpp1	2E-11	0.5
Pask	2E-01	0.5	Ccser1	6E-16	0.5
Gm32618	7E-07	0.5	Pdlim4	3E-24	0.5
Ets2	2E-11	0.5	Tenm4	6E-47	0.5
Sema3f	2E-06	0.5	Paqr3	2E-04	0.5
Jmjd7	2E-01	0.5	Serpine2	3E-68	0.5
Nme5	1E-04	0.5	Lcn2	2E-16	0.5
Ostn	1E-05	0.5	Pitx1	9E-44	0.5
Serpine2	2E-75	0.5	Ppp1r9a	5E-08	0.5
Sox4	6E-58	0.5	Robo1	5E-28	0.5
Klf8	4E-05	0.5	Sdc4	1E-49	0.5
Trp63	7E-09	0.5	Cpe	3E-84	0.5
Pde4b	4E-01	0.5	Pla2r1	2E-03	0.5
Flrt1	5E-05	0.5	Arhgap22	4E-02	0.5
Trpm6	6E-03	0.5	Sox4	7E-47	0.5
Adamts1	1E-05	0.5	Inpp4b	2E-05	0.4
Gm45025	1E-03	0.5	Itm2a	1E-75	0.4
Sh2d4a	9E-03	0.5	Gm26917	2E-06	0.4
4921531C22Rik	5E-01	0.5	Eya4	4E-41	0.4
Aqp5	2E-01	0.5	Plxnc1	5E-03	0.4
Sned1	2E-59	0.5	Tcf4	7E-116	0.4
Inpp4b	5E-10	0.5	Ccdc86	2E-08	0.4
B230206L02Rik	3E-04	0.5	Lefty1	6E-04	0.4
9530026P05Rik	2E-08	0.5	Spa17	2E-03	0.4
Fhl1	2E-07	0.5	Gchfr	1E-02	0.4
Mkx	2E-09	0.4	Cavin4	7E-02	0.4
Adm	1E-07	0.4	Sema3f	3E-04	0.4
Etv5	2E-08	0.4	Phactr1	2E-11	0.4
Itpr3	1E-05	0.4	Osr2	2E-13	0.4
Eya4	8E-60	0.4	Mtmr11	2E-02	0.4
Itga3	3E-03	0.4	Hectd2	6E-02	0.4
Gsap	2E-01	0.4	Pygl	6E-09	0.4
Arhgap22	9E-07	0.4	Pnmal2	1E-02	0.4
Cemip	3E-02	0.4	Gpc2	1E-02	0.4
Camk1d	2E-03	0.4	Fosl1	6E-01	0.4
Pdp1	3E-26	0.4	Dchs1	5E-03	0.4
Mmp16	5E-11	0.4	Bmp4	1E-16	0.4
Tcf4	6E-119	0.4	Meox1	3E-01	0.4

Articular/Synovial Fibroblast

Control Gene	p	FC	NICD2 Gene	p	FC
Ggt5	4E-57	1.7	Cd34	6E-22	1.5
Nat8f1	8E-41	1.7	BC028528	3E-28	1.5
Gm28535	2E-44	1.7	Ntn1	3E-12	1.5
Ptchd1	2E-24	1.6	Nat8f1	6E-25	1.5
Aox3	9E-11	1.6	Gm26771	6E-45	1.5
Adam23	3E-67	1.6	Nova1	3E-08	1.5
Adgrd1	2E-23	1.6	Snhg11	3E-23	1.5
Plac9b	1E-18	1.6	Adam23	3E-32	1.5
Nptx1	7E-13	1.6	Gsta4	1E-42	1.4
Gm36211	7E-10	1.6	Ednrb	9E-09	1.4
Plpp3	2E-157	1.6	Clec14a	7E-19	1.4
Nova1	2E-26	1.6	Sp100	7E-08	1.4
Sp110	1E-13	1.6	Olfml2a	2E-16	1.4
Plvap	3E-11	1.6	Plac9b	3E-08	1.4
Mustn1	5E-38	1.6	Inhbb	5E-08	1.4
Hgf	3E-74	1.6	Plvap	6E-12	1.4
Gm10638	2E-07	1.6	Plpp3	2E-90	1.4
Hp	3E-72	1.5	Phactr1	6E-42	1.4
4930594M22Rik	5E-05	1.5	Mturn	4E-15	1.4
C1qtnf7	7E-35	1.5	C1qtnf7	3E-24	1.3
Lrp4	4E-26	1.5	Ramp2	1E-39	1.3
Nbl1	2E-98	1.5	Gm16894	2E-07	1.3
Tmem158	5E-51	1.5	Cxcl12	7E-98	1.3
Snhg11	2E-38	1.5	H2-Q7	2E-08	1.3
BC028528	3E-32	1.5	Pla1a	8E-12	1.3
Adssl1	2E-15	1.5	Apoe	2E-70	1.3
Sash1	2E-130	1.5	Gngt2	3E-11	1.3
Cd34	4E-38	1.5	Calml4	4E-14	1.3
Saa3	4E-39	1.5	Gm10638	2E-02	1.3
Sfrp2	5E-84	1.5	Adssl1	3E-08	1.3
Htr2a	6E-13	1.4	Gpnmb	4E-81	1.3
Gm32014	4E-08	1.4	Lgals3	1E-90	1.3
Slc25a21	4E-10	1.4	Il17d	7E-16	1.3
Gm26771	7E-56	1.4	Lrrn4cl	8E-07	1.3
Cdk15	4E-10	1.4	Srgn	3E-17	1.3
Inhbb	1E-09	1.4	Phex	8E-13	1.3
Rspo2	4E-66	1.4	Osr2	2E-48	1.3
Gm7160	7E-11	1.4	Sash1	5E-87	1.3
Dpp4	6E-15	1.4	Gsn	7E-96	1.3
Ackr3	3E-25	1.4	Nsun7	1E-05	1.3
H2-Q7	1E-17	1.4	Sgip1	2E-04	1.3
Aldh3a1	3E-09	1.4	Ociad2	2E-17	1.3
A530013C23Rik	2E-11	1.4	Gla	1E-15	1.3
Sphk1	2E-11	1.4	Hgf	1E-34	1.2
Serpina3n	1E-52	1.4	Ifi204	4E-17	1.2
Ntn1	1E-18	1.4	Shc2	8E-07	1.2
Adamtsl5	3E-50	1.4	A530013C23Rik	3E-06	1.2
Phex	7E-20	1.4	Gata6	3E-06	1.2
Alms1	7E-37	1.4	Itpr3	2E-08	1.2
Cacna2d3	3E-15	1.3	Sbsn	1E-13	1.2
Rbpms	4E-26	1.3	Galm	7E-12	1.2
Apoe	2E-92	1.3	Lnx1	1E-04	1.2
Emilin2	1E-47	1.3	C4b	5E-55	1.2
Ociad2	9E-34	1.3	Gm17057	2E-06	1.2
Prg4	5E-09	1.3	Adamts15	2E-05	1.2
Ednra	2E-35	1.3	Gem	3E-25	1.2
Gm16894	1E-09	1.3	Cdr1os	4E-06	1.2
Slc16a10	3E-10	1.3	Fam49a	1E-07	1.2
Arhgap44	8E-20	1.3	Cela1	3E-07	1.2
Sp100	8E-12	1.3	Pax9	2E-04	1.2
Tmod1	7E-22	1.3	Arhgap44	6E-11	1.2
Bace2	1E-10	1.3	Tmem140	7E-13	1.2
B4galt5	1E-26	1.3	Rspo2	2E-40	1.2
Ptgfr	9E-15	1.3	Tmem158	3E-23	1.2
St3gal1	7E-59	1.3	Serpina3n	6E-30	1.2
Ifi204	9E-31	1.3	4930594M22Rik	8E-03	1.2
AA467197	2E-06	1.3	Cyp4v3	7E-21	1.2
Gla	9E-22	1.3	Mab21l1	8E-07	1.2
C4b	6E-75	1.3	Plscr4	4E-12	1.2
Cdr1os	6E-08	1.3	Bace2	1E-10	1.2
Add3	3E-72	1.3	Saa3	1E-29	1.2
Larp6	3E-09	1.3	H2-Q4	6E-08	1.2
Gsn	6E-125	1.2	Agtr2	5E-09	1.2

Limb Mesenchyme

Control Gene	p	FC	NICD2 Gene	p	FC
Fbxl7	1E-86	1.0	Gm43126	6E-14	0.9
Nkd1	3E-59	1.0	Sema3a	1E-34	0.9
Plb1	1E-15	1.0	Efna5	2E-60	0.9
Adamtsl3	4E-64	1.0	Edil3	3E-100	0.9
Rtl1	9E-24	1.0	Gpc4	4E-76	0.9
Gm15910	9E-17	1.0	Brwd3	5E-19	0.9
Gm15414	6E-17	1.0	Kctd15	2E-21	0.9
Clmn	3E-39	1.0	Mex3a	2E-29	0.9
Fgd6	5E-35	1.0	Flnb	8E-79	0.9
Dlx5	1E-21	0.9	Hbegf	9E-27	0.9
Ccser1	2E-63	0.9	Gm12526	2E-10	0.9
Fgfr2	2E-126	0.9	Cdkn1c	1E-51	0.9
Kcnt2	1E-35	0.9	Il1rapl1	7E-47	0.9
Bcat1	1E-45	0.9	Cth	2E-30	0.9
Calhm5	6E-37	0.9	Gm49890	1E-08	0.9
Ebf4	2E-11	0.9	Clcn5	7E-36	0.9
Abtb2	5E-37	0.9	Nrp2	7E-85	0.9
Aldh1l2	2E-84	0.9	Cplx2	8E-15	0.8
Rab27b	2E-25	0.9	Rbp1	2E-22	0.8
Apbb2	1E-121	0.9	Satb2	2E-14	0.8
Gm16152	6E-21	0.9	Sept6	2E-28	0.8
Slc7a5	1E-55	0.9	Gpr27	4E-20	0.8
Trib2	5E-31	0.9	Mmp13	3E-12	0.8
Adm2	6E-27	0.9	Pcdh9	3E-43	0.8
Sox9	3E-74	0.9	Stbd1	2E-18	0.8
P4ha3	3E-44	0.9	Gm13684	3E-10	0.8
Gm15655	6E-14	0.9	Slc7a5	2E-43	0.8
Herc3	8E-11	0.9	Asxl3	3E-07	0.8
Tenm4	1E-73	0.9	Cyp2d22	1E-05	0.8
C230038L03Rik	1E-24	0.9	App	4E-150	0.8
C430049B03Rik	1E-47	0.9	Ccdc68	7E-21	0.8
Slc6a9	2E-33	0.9	Stxbp2	2E-13	0.8
Cdkn1c	4E-65	0.9	Sox9	1E-51	0.8
Slc7a11	1E-30	0.9	C430049B03Rik	7E-30	0.8
Ostn	8E-29	0.9	Cdc42bpa	7E-88	0.8
Gnao1	4E-20	0.9	Ogn	2E-132	0.8
Cth	2E-23	0.9	Cntnap2	2E-13	0.8
4933400C23Rik	3E-44	0.9	Lin7a	4E-14	0.8
Pik3cb	1E-20	0.9	Etl4	7E-36	0.8
Cemip2	2E-34	0.9	Adamts6	1E-18	0.8
Ammecr1	5E-50	0.9	Tiam2	6E-86	0.8
Alcam	2E-28	0.9	Mab21l2	2E-46	0.8
Dpysl4	3E-13	0.9	Tchh	2E-11	0.8
Scn3a	2E-12	0.9	Sdk2	2E-47	0.8
Myo1b	3E-60	0.9	Larp1b	3E-25	0.8
Zbtb7c	2E-33	0.9	1700109K24Rik	7E-16	0.8
Gm49226	2E-15	0.9	Rnd1	3E-12	0.8
Larp1b	2E-32	0.9	Syne1	4E-83	0.8
Gm45025	1E-21	0.9	Kcnq5	3E-43	0.8
Gm48898	2E-16	0.9	Fbn2	8E-44	0.8
Cdc42bpa	9E-102	0.9	Gm26802	3E-15	0.8
Mex3a	9E-31	0.9	Spata13	1E-16	0.8
Gprc5c	7E-17	0.8	Pdgfc	1E-24	0.8
Itgbl1	7E-86	0.8	Plekhg1	1E-13	0.8
Zfp516	6E-67	0.8	Gm49267	3E-12	0.8
Mirg	2E-33	0.8	Cdkl5	8E-34	0.8
Fam180a	8E-13	0.8	Trib3	2E-39	0.8
C230034O21Rik	2E-09	0.8	D5Ertd579e	8E-49	0.8
Nrp2	2E-97	0.8	Fzd8	1E-55	0.8
Dach2	2E-18	0.8	Fam20a	4E-25	0.8
Ptch1	9E-28	0.8	Lrrc39	2E-05	0.8
Slc7a1	2E-59	0.8	Ehd3	7E-20	0.8
Syne1	2E-88	0.8	Cdsn	2E-24	0.8
Cyb5r2	1E-39	0.8	Nog	7E-10	0.8
Lyst	1E-42	0.8	Sorbs2	3E-29	0.8
Ercc6	8E-26	0.8	Igsf10	6E-55	0.8
Tsix	7E-21	0.8	Cemip	1E-19	0.8
Mthfd2	6E-85	0.8	Abtb2	2E-28	0.8
Jph2	6E-17	0.8	Gm13269	4E-09	0.8
Gpc2	3E-13	0.8	Gm13483	3E-16	0.8
Daam2	1E-36	0.8	Prkaa2	2E-13	0.8
Zfp462	3E-46	0.8	Vldlr	4E-29	0.8
Edil3	1E-88	0.8	Gm45025	3E-15	0.8

Chondrogenic

Control Gene	p	FC	NICD2 Gene	p	FC
Aff3	3E-242	1.4	Aff3	2E-185	1.4
S100b	3E-107	1.4	Sntg2	3E-25	1.4
Tent5c	1E-15	1.4	Gadd45a	1E-30	1.4
Platr22	3E-31	1.4	Lctl	1E-24	1.4
Col27a1	1E-46	1.4	Sox6	7E-213	1.4
Npr2	4E-91	1.4	Klf15	3E-23	1.4
Plb1	3E-16	1.4	Mmp13	1E-23	1.4
Fgd6	4E-51	1.4	Gm49708	2E-75	1.4
Map3k5	2E-37	1.4	Trpm6	8E-25	1.4
Sox6	3E-265	1.4	Adgrg6	6E-146	1.4
Ooep	1E-49	1.4	Saa3	4E-13	1.4
Rgs7bp	5E-16	1.4	Pam	0E+00	1.4
BC006965	1E-23	1.4	Etv1	1E-27	1.4
Msi2	1E-251	1.4	Msi2	2E-199	1.4
Rab27b	1E-34	1.4	Cacnb2	4E-80	1.4
Gm49226	4E-23	1.4	Brwd3	4E-27	1.4
C730002L08Rik	6E-26	1.4	Ninj1	4E-103	1.4
Plod2	1E-257	1.4	Arhgef3	1E-48	1.4
Epas1	4E-34	1.4	Ctsh	2E-182	1.4
Tead4	3E-18	1.4	Sox5	2E-177	1.4
Gm49708	5E-74	1.4	Raph1	6E-88	1.4
Fhl1	2E-113	1.4	Adamts9	3E-42	1.4
Gm38398	3E-20	1.3	Mmp3	2E-14	1.4
Barx1	9E-116	1.3	Gm28905	1E-21	1.4
Sox9	5E-178	1.3	Kcnk2	5E-86	1.4
Lcn2	4E-20	1.3	Slc26a7	6E-18	1.3
Ecrg4	5E-263	1.3	Fgd6	6E-25	1.3
Lin7a	2E-20	1.3	Lcn2	2E-25	1.3
1700024B18Rik	6E-18	1.3	Dync1i1	6E-17	1.3
Prkcb	1E-15	1.3	Gba2	1E-12	1.3
Tchh	5E-20	1.3	Ecrg4	3E-223	1.3
Ctdspl	1E-146	1.3	Epha2	2E-15	1.3
Stxbp6	2E-133	1.3	Armh4	5E-91	1.3
Kcnk2	2E-118	1.3	Cpne8	6E-162	1.3
Ctsh	3E-212	1.3	Atp6v0e2	2E-18	1.3
Atp6v0e2	3E-25	1.3	1700023F06Rik	4E-16	1.3
Rgp1	8E-34	1.3	Prkcz	8E-14	1.3
Crybg1	1E-16	1.3	Lmo7	6E-102	1.3
Loxl4	1E-14	1.3	Aff2	2E-74	1.3
Ninj1	2E-163	1.3	Gm13056	5E-36	1.3
Klc3	1E-37	1.3	Ccdc68	1E-29	1.3
Gm28905	1E-27	1.3	Prkaa2	1E-30	1.3
Smad9	4E-27	1.3	Cables1	2E-12	1.3
Atp10a	3E-56	1.3	Epas1	3E-29	1.3
Cpne8	5E-202	1.3	Gm49226	3E-19	1.3
Sap30	5E-137	1.3	Myo1d	3E-155	1.3
Lctl	4E-34	1.3	Tiparp	3E-100	1.3
Tenm1	3E-36	1.3	Boc	2E-96	1.2
Ell2	3E-176	1.3	Tpd52l1	1E-23	1.2
Nuak1	4E-64	1.3	Stxbp6	8E-105	1.2
Slc26a2	4E-27	1.3	Mgat4a	6E-17	1.2
Ogn	4E-238	1.3	Ptgis	1E-58	1.2
Aff2	7E-110	1.3	Ell2	2E-129	1.2
Slc40a1	2E-23	1.3	Axin2	2E-17	1.2
Grem1	2E-13	1.3	Fzd6	1E-18	1.2
Ptgis	2E-97	1.3	Dhrs9	1E-61	1.2
Chst11	4E-49	1.3	Itgbl1	8E-110	1.2
Larp1b	2E-57	1.3	Esr1	2E-33	1.2
Efcab1	9E-30	1.3	Ctdspl	6E-114	1.2
Esr1	4E-41	1.3	Gm49417	4E-16	1.2
Igf2	5E-82	1.3	Tspan2	2E-22	1.2
Klf15	1E-26	1.3	C230038L03Rik	1E-21	1.2
Myo1d	1E-182	1.3	Nuak2	4E-09	1.2
Gm2415	1E-16	1.3	Rnf144b	2E-11	1.2
Prkaa2	6E-27	1.3	Foxc2	1E-13	1.2
Stxbp2	8E-19	1.3	Ifitm10	1E-16	1.2
Foxc2	2E-16	1.2	Aopep	2E-283	1.2
Gadd45a	2E-19	1.2	Fgfr2	7E-171	1.2
Saa3	3E-13	1.2	Larp1b	8E-35	1.2
Cp	6E-43	1.2	Cemip	7E-16	1.2
Lmcd1	2E-54	1.2	Tchh	2E-10	1.2
Mlip	2E-38	1.2	Hoxaas3	1E-11	1.2
Aig1	7E-138	1.2	Cpe	4E-216	1.2

Fibroblast

Control Gene	p	FC	NICD2 Gene	p	FC
Lurap1l	0E+00	1.3	Pax1	8E-37	1.2
Myl9	7E-187	1.3	Gm48727	2E-38	1.2
Iigp1	6E-206	1.3	4933400A11Rik	5E-20	1.2
Iqgap2	6E-69	1.3	Vcam1	8E-287	1.2
Smoc2	2E-302	1.3	Mgst1	0E+00	1.2
Bmp6	3E-61	1.3	Cstad	3E-29	1.2
Pappa	0E+00	1.3	Kitl	1E-137	1.2
Kitl	0E+00	1.3	Iigp1	1E-94	1.2
Sp140	5E-87	1.3	Hand2	2E-58	1.2
Gm48727	2E-59	1.3	Agtr1a	7E-61	1.2
Cxcl12	0E+00	1.2	St3gal1	9E-233	1.2
Basp1	0E+00	1.2	Sp140	6E-42	1.2
Gas6	0E+00	1.2	Foxs1	9E-29	1.2
4933400A11Rik	2E-51	1.2	Ugcg	4E-186	1.2
Il1rn	0E+00	1.2	Crabp2	2E-100	1.2
Acta2	1E-63	1.2	Crabp1	6E-34	1.2
Kng1	2E-114	1.2	Slc8a1	6E-142	1.2
Vcam1	0E+00	1.2	Gas6	0E+00	1.2
Mgst1	0E+00	1.2	4833427G06Rik	1E-45	1.2
Frmd3	8E-45	1.2	Pappa	4E-285	1.2
Megf6	1E-165	1.2	Lurap1l	2E-221	1.2
Lingo1	2E-53	1.2	Clec14a	3E-79	1.2
Tex15	2E-52	1.2	Oasl2	3E-56	1.2
Slc8a1	8E-307	1.2	Gm12158	4E-72	1.2
Oasl2	4E-121	1.2	Kng1	5E-55	1.2
Gm12158	4E-155	1.2	Trim30a	1E-91	1.2
Tmeff2	3E-42	1.2	Selenop	1E-294	1.2
Eln	5E-170	1.2	Snhg11	3E-76	1.2
Pth1r	1E-138	1.2	Megf6	7E-92	1.2
Ly6a	0E+00	1.2	Fgf7	4E-292	1.2
Cdh2	0E+00	1.2	Gprin3	1E-57	1.2
Agtr1a	3E-128	1.2	Mn1	3E-91	1.2
St3gal1	0E+00	1.2	Rspo2	3E-142	1.2
Irx1	3E-73	1.2	Iqgap2	5E-21	1.2
Nat8f3	2E-67	1.2	Enc1	4E-88	1.2
Rspo2	0E+00	1.2	Tnfaip6	2E-52	1.2
Hand2	1E-111	1.2	Entpd2	4E-65	1.2
Ugcg	6E-273	1.2	Cdh2	9E-206	1.1
Igfbp4	0E+00	1.2	Olfml1	6E-118	1.1
Bckdhb	0E+00	1.2	Bckdhb	5E-179	1.1
Tnfaip6	4E-125	1.2	Lrp1b	4E-18	1.1
Tll1	2E-66	1.2	Matn2	1E-197	1.1
Col23a1	7E-37	1.2	Cxcl12	6E-231	1.1
Selenop	0E+00	1.2	Cd248	0E+00	1.1
Enc1	3E-186	1.2	Gng8	5E-59	1.1
Olfml1	1E-237	1.1	Myl9	8E-95	1.1
Trim30a	4E-190	1.1	Adamts15	2E-19	1.1
Ifi211	5E-222	1.1	Kazn	4E-71	1.1
Cd248	0E+00	1.1	Irx1	2E-33	1.1
Peli2	2E-248	1.1	Nat8f3	2E-28	1.1
Gng8	3E-128	1.1	Tll1	1E-36	1.1
Pappa2	5E-24	1.1	Igfbp4	0E+00	1.1
Rspo3	0E+00	1.1	Tex15	3E-19	1.1
Cnn1	9E-23	1.1	Tmeff2	2E-17	1.1
Cstad	7E-51	1.1	Peli2	5E-113	1.1
Mn1	4E-170	1.1	Frmd3	5E-18	1.1
Clec14a	2E-151	1.1	Cyp1b1	3E-182	1.1
Trpm3	7E-63	1.1	Pappa2	3E-07	1.1
Ptprn	7E-56	1.1	Ptprn	2E-29	1.1
Pygb	1E-214	1.1	Acta2	1E-30	1.1
Hoxc13	9E-74	1.1	Ifi211	3E-93	1.1
Snhg11	1E-131	1.1	Basp1	7E-262	1.1
Itga9	6E-155	1.1	Itga9	4E-82	1.1
Hic1	2E-111	1.1	Lmod1	4E-12	1.1
Fgf7	0E+00	1.1	Susd2	6E-37	1.1
Radil	7E-47	1.1	Gfra1	6E-43	1.1
Podn	1E-118	1.1	Hoxc11	4E-39	1.1
Gxylt2	0E+00	1.1	Adamtsl1	4E-84	1.1
Cyp1b1	0E+00	1.1	Nid1	1E-221	1.1
Kazn	1E-108	1.1	Col23a1	1E-19	1.1
Matn2	0E+00	1.1	Radil	8E-22	1.0
Dpyd	2E-77	1.1	Ly6c1	7E-148	1.0
Susd2	1E-72	1.0	Il1rn	2E-125	1.0

Fibroblast?

Control Gene	p	FC	NICD2 Gene	p	FC
Parpbp	0E+00	4.6	Hist1h2bh	4E-269	4.7
Lockd	0E+00	4.6	Rad54b	0E+00	4.7
Arhgap11a	0E+00	4.6	Parpbp	0E+00	4.7
Kifc1	0E+00	4.6	Ncapg2	0E+00	4.7
Tedc1	0E+00	4.6	Efcab11	0E+00	4.7
Hist1h1b	0E+00	4.5	Cdc6	0E+00	4.7
Cenpn	0E+00	4.5	Mtfr2	0E+00	4.6
Shisa8	5E-138	4.5	Tedc1	0E+00	4.6
Rad54b	0E+00	4.5	Oip5	0E+00	4.6
Cenpu	0E+00	4.5	Hist1h2ae	0E+00	4.6
Chtf18	0E+00	4.5	Gm14091	6E-184	4.6
Mtfr2	0E+00	4.5	Arhgap11a	0E+00	4.6
Hist1h2ae	0E+00	4.5	Erfe	5E-238	4.6
Fancd2	0E+00	4.5	Mastl	0E+00	4.5
Hist1h2bj	0E+00	4.5	Chtf18	0E+00	4.5
Mastl	0E+00	4.5	Ube2t	0E+00	4.4
Kif22	0E+00	4.5	Stil	0E+00	4.4
Rad51	0E+00	4.4	Gmnn	0E+00	4.4
Stil	0E+00	4.4	Incenp	0E+00	4.4
Ube2t	0E+00	4.4	Cip2a	0E+00	4.4
D030068K23Rik	2E-212	4.4	Rad51	0E+00	4.4
Mybl2	0E+00	4.4	Foxm1	0E+00	4.3
Fbxo48	5E-193	4.4	Atf7ip2	7E-144	4.3
Hist1h2ak	3E-249	4.3	Cenpn	0E+00	4.3
Cip2a	0E+00	4.3	Ticrr	0E+00	4.3
Ticrr	0E+00	4.3	Trip13	0E+00	4.3
Mcm5	0E+00	4.3	Recql4	3E-236	4.2
Hist1h2bb	0E+00	4.3	Smc2	0E+00	4.2
Dscc1	0E+00	4.3	Fbxo48	2E-199	4.2
Incenp	0E+00	4.3	Zwilch	0E+00	4.2
Chaf1b	0E+00	4.2	Cdc45	0E+00	4.2
Gmnn	0E+00	4.2	Tcf19	0E+00	4.2
Smc2	0E+00	4.2	Hist1h2bn	0E+00	4.1
Trip13	0E+00	4.2	Pkmyt1	0E+00	4.1
Tcf19	0E+00	4.2	Dscc1	0E+00	4.1
Zwilch	0E+00	4.2	Mcm5	0E+00	4.1
Fignl1	0E+00	4.2	Mlf1	0E+00	4.1
Hist1h2bn	3E-240	4.2	Cenpq	0E+00	4.1
Hist1h2bh	3E-248	4.2	Fancd2	0E+00	4.1
Foxm1	0E+00	4.2	Rmi2	0E+00	4.0
Cenpq	0E+00	4.1	Slfn9	0E+00	4.0
Gm14091	2E-177	4.1	Fignl1	0E+00	4.0
Gm41555	2E-279	4.1	4930452B06Rik	0E+00	4.0
Hist1h4k	1E-118	4.0	D030068K23Rik	3E-164	4.0
Rmi2	0E+00	4.0	Chaf1b	0E+00	4.0
2810408I11Rik	0E+00	4.0	Ncapd2	0E+00	4.0
Cdk1	0E+00	4.0	Cenpk	0E+00	4.0
Cdc45	0E+00	4.0	4930579G24Rik	0E+00	4.0
Cenpk	0E+00	3.9	Cdk1	0E+00	4.0
Dtl	0E+00	3.9	2810429I04Rik	7E-141	3.9
Pole	0E+00	3.9	Dtl	0E+00	3.9
Ncapd2	0E+00	3.9	Pole	0E+00	3.9
Mlf1	0E+00	3.9	Hist1h3i	0E+00	3.8
Pkmyt1	0E+00	3.9	Mad2l1	0E+00	3.8
Cks2	0E+00	3.9	Hist1h4a	6E-156	3.8
5430427M07Rik	1E-233	3.8	Gm41555	3E-283	3.8
Kif18a	0E+00	3.8	Rad54l	0E+00	3.8
Dbf4	0E+00	3.8	Fancb	0E+00	3.8
Hist2h2ac	0E+00	3.8	Stmn1	0E+00	3.8
Fancb	0E+00	3.8	Hist2h2ac	0E+00	3.8
Gtse1	0E+00	3.8	Cks2	0E+00	3.7
Fam81a	4E-177	3.8	Neurl1b	6E-273	3.7
Mad2l1	0E+00	3.8	Hist1h3f	3E-240	3.7
Hist1h3i	8E-308	3.8	Gtse1	0E+00	3.7
4930452B06Rik	0E+00	3.7	Dhfr	0E+00	3.7
Hist1h4a	2E-142	3.7	Lmnb1	0E+00	3.7
Stmn1	0E+00	3.7	Hmgb2	0E+00	3.7
Ulbp1	4E-151	3.7	Mybl1	0E+00	3.7
Hist1h3f	3E-234	3.7	Hist1h2bf	2E-169	3.7
Slfn9	0E+00	3.7	2810408I11Rik	0E+00	3.7
Rtkn2	2E-190	3.6	Fam111a	0E+00	3.7
4930579G24Rik	0E+00	3.6	Hells	0E+00	3.7
Hmgb2	0E+00	3.6	Fam81a	1E-218	3.6

Undefined

Control Gene	p	FC	NICD2 Gene	p	FC
Pdlim4	2E-19	0.4	Smoc1	5E-38	0.4
Aopep	3E-125	0.4	Podxl2	1E-13	0.4
Ahr	9E-28	0.4	Cib2	2E-13	0.4
Mlip	6E-07	0.4	Carns1	1E-02	0.4
Mef2a	2E-42	0.4	Btbd11	3E-14	0.4
Mme	2E-49	0.4	Cpq	2E-87	0.4
Arhgap6	3E-16	0.4	Dab2	1E-46	0.4
Angptl4	7E-23	0.4	Mmp16	3E-04	0.4
Gpsm2	6E-14	0.4	Nr3c2	4E-04	0.4
C230038L03Rik	6E-03	0.4	Stbd1	3E-02	0.4
Otud1	7E-03	0.4	Ypel1	2E-03	0.4
Mitf	6E-11	0.4	Nrp2	2E-62	0.4
Meox1	2E-01	0.4	Pcgf1	9E-01	0.4
Mab21l2	2E-25	0.4	Ldhb	4E-18	0.4
Pitx1	5E-47	0.4	Pon3	2E-19	0.4
Tenm4	2E-40	0.4	Thbd	8E-06	0.4
Robo1	9E-28	0.4	Adm	2E-03	0.4
E130114P18Rik	7E-03	0.4	Pawr	5E-16	0.4
Osr1	7E-09	0.4	Clip4	6E-03	0.4
Larp6	5E-03	0.4	Syt12	8E-03	0.4
Ogn	5E-120	0.4	Osr1	1E-04	0.4
5930430L01Rik	2E-08	0.4	Hoxa11os	7E-05	0.4
Sinhcaf	4E-01	0.4	Hdac9	4E-01	0.4
Fap	2E-04	0.4	E130114P18Rik	3E-03	0.4
Zeb1	6E-21	0.4	App	4E-108	0.4
Cbr2	1E-22	0.4	Mitf	1E-05	0.4
Slc38a1	2E-18	0.4	Lix1	3E-01	0.4
Trabd2b	6E-35	0.4	Tbx18	4E-08	0.4
Pon3	5E-20	0.4	Arhgap6	7E-09	0.4
Gm2115	2E-02	0.4	Tob1	2E-17	0.4
Gchfr	2E-05	0.4	Papolg	6E-03	0.4
9130008F23Rik	5E-03	0.4	Ahrr	6E-03	0.4
Adgrg2	4E-21	0.4	Mlip	1E-03	0.4
Ahrr	1E-03	0.4	Cbr2	1E-17	0.4
Hbegf	8E-07	0.4	Aopep	6E-93	0.4
Tob1	3E-15	0.4	Il18	1E-01	0.4
Ttc26	6E-03	0.4	Krba1	1E-01	0.4
Ank	5E-02	0.4	Gm32031	4E-01	0.4
Gramd4	3E-04	0.4	Gm16070	2E-08	0.4
Sntg2	8E-12	0.4	Fbln7	7E-12	0.4
Ppp1r9a	5E-05	0.4	Hoxa3	9E-04	0.4
Rasl11a	1E-03	0.4	Xrcc5	1E-11	0.4
Hmga2	8E-01	0.4	Agtr2	5E-04	0.4
Glrx	7E-09	0.4	Lmbr1l	3E-01	0.4
B930095G15Rik	4E-02	0.4	Mef2a	8E-25	0.4
Fgfbp3	1E-03	0.4	Acp5	3E-05	0.4
Adam22	1E-03	0.4	Rwdd2b	7E-01	0.4
Gm20631	1E-01	0.4	Slc23a2	9E-08	0.4
Ercc8	5E-02	0.4	1810021B22Rik	5E-01	0.4
Plod1	2E-23	0.4	Angptl4	1E-14	0.4
Saa3	2E-14	0.4	Pdp1	6E-16	0.4
Hspb6	9E-16	0.4	Pnrc1	6E-50	0.4
Rgs3	5E-05	0.4	8430429K09Rik	1E+00	0.4
Hes6	3E-03	0.4	5430405H02Rik	4E-05	0.4
Tbx5	8E-04	0.4	Serpinb1a	2E-01	0.4
Pygl	9E-08	0.4	Gm12655	7E-03	0.4
Nek8	1E+00	0.4	Gsta4	4E-01	0.4
Wdr45	9E-05	0.4	Hoxd9	3E-02	0.4
Lefty1	5E-05	0.4	Gm4876	1E-03	0.4
Npr3	2E-01	0.4	Cdr1os	8E-02	0.4
Tcte2	2E-01	0.4	Sh2d4a	3E-01	0.4
Mfge8	1E-54	0.4	Slc48a1	1E-17	0.4
Cpe	3E-73	0.4	Agbl5	4E-01	0.4
Epas1	2E-02	0.4	Smpdl3b	2E-01	0.4
Hoxd9	7E-04	0.4	Fosl2	2E-18	0.4
Pld1	2E-06	0.4	Ahr	3E-22	0.4
Tnfrsf23	9E-03	0.4	Otud1	1E+00	0.4
Angptl1	2E-07	0.4	Cplx2	1E-01	0.4
Slc48a1	9E-27	0.4	Klhl24	1E-09	0.4
Mecom	1E-25	0.4	Zfp335os	7E-01	0.4
Tmem131l	1E-01	0.4	Clu	1E-24	0.4
Gadd45a	5E-07	0.4	Angpt2	3E-02	0.4
Arsg	2E-02	0.4	Bcl3	7E-03	0.4

Articular/Synovial Fibroblast

Control Gene	p	FC	NICD2 Gene	p	FC
Col5a3	5E-57	1.2	Pygl	3E-17	1.2
Fgf18	3E-10	1.2	Add3	1E-51	1.2
Ebf1	8E-124	1.2	Cfb	2E-21	1.2
Cfb	3E-34	1.2	1700007K13Rik	3E-05	1.1
Calml4	5E-18	1.2	Zfp36l1	4E-57	1.1
Gm12158	2E-16	1.2	Emilin2	1E-27	1.1
Lama5	1E-06	1.2	Ptgfr	8E-06	1.1
Phactr1	2E-44	1.2	Lrmda	3E-23	1.1
Pygl	6E-25	1.2	Elmo1	2E-10	1.1
Spsb1	2E-15	1.2	Ahnak2	9E-54	1.1
Mturn	2E-23	1.2	Ebf1	2E-97	1.1
Sipa1l2	2E-06	1.2	B230206L02Rik	5E-06	1.1
Sbsn	6E-28	1.2	Man1c1	2E-24	1.1
Irak3	3E-28	1.2	Loxl1	2E-59	1.1
Agtr2	3E-18	1.2	B4galt5	2E-11	1.1
Steap3	3E-112	1.2	Adamtsl5	2E-15	1.1
Gm16364.1	8E-20	1.2	Alms1	5E-15	1.1
Shc2	5E-07	1.2	Cacna2d3	3E-06	1.1
Lgals3	2E-106	1.2	Fam13a	6E-08	1.1
Osr1	1E-38	1.2	Srgap1	2E-33	1.1
Gpnmb	2E-94	1.2	Bdh2	6E-10	1.1
Gngt2	3E-11	1.2	Sdk1	9E-17	1.1
Galm	2E-15	1.2	Slc25a42	1E-04	1.1
Errfi1	7E-19	1.2	Steap3	6E-62	1.1
Slfn2	1E-08	1.2	Nme5	5E-05	1.1
Mitf	2E-34	1.2	Nrn1	4E-16	1.1
Bmper	1E-05	1.2	Pde1a	8E-24	1.1
Gprc5b	2E-13	1.2	Tmod1	2E-06	1.1
Ramp2	7E-44	1.2	Cyp27a1	2E-03	1.1
Gem	2E-25	1.1	Osr1	1E-20	1.1
Maob	3E-09	1.1	Tpcn2	3E-06	1.0
Gm20125	2E-03	1.1	Aldh3a1	1E-08	1.0
Dpt	7E-41	1.1	Irak3	2E-14	1.0
Sema3b	2E-19	1.1	Ass1	2E-10	1.0
Ildr2	2E-19	1.1	Pltp	2E-40	1.0
Ralgds	1E-25	1.1	Sema6d	6E-05	1.0
Cyp4v3	6E-23	1.1	Sipa1l2	6E-08	1.0
Sema6d	8E-05	1.1	Apol9a	2E-06	1.0
Osr2	4E-43	1.1	Anpep	7E-11	1.0
Ass1	2E-17	1.1	Gstt1	7E-29	1.0
Lifr	6E-19	1.1	Stc1	6E-08	1.0
Man1c1	2E-29	1.1	Fgf14	8E-06	1.0
Psmb8	3E-43	1.1	Uap1l1	4E-30	1.0
Cyp1b1	9E-63	1.1	Phf11d	4E-14	1.0
S100a4	8E-64	1.1	Rbpms	2E-08	1.0
Il1r1	2E-78	1.1	Gm16364.1	4E-08	1.0
Gsta4	3E-53	1.1	Tbx18	9E-17	1.0
Pdpn	2E-57	1.1	Errfi1	5E-19	1.0
Rasl12	3E-06	1.1	Rasl12	2E-03	1.0
Arhgap6	3E-48	1.1	Stom	3E-06	1.0
Itpr3	9E-15	1.1	H2-T23	3E-20	1.0
Tmem26	4E-11	1.1	Svip	5E-03	1.0
H2-Q6	1E-05	1.1	Mt1	1E-74	1.0
Btbd11	1E-21	1.1	A4galt	2E-13	1.0
Nlrc5	1E-05	1.1	Negr1	1E-10	1.0
Rasl11a	2E-06	1.1	Kcnab1	2E-06	1.0
Bdh2	7E-17	1.1	Mitf	3E-21	1.0
AI413582	6E-49	1.1	Bcl3	2E-11	1.0
H2-Q4	4E-08	1.1	Insyn1	2E-11	1.0
Tbx18	1E-20	1.1	Rnf144a	7E-14	1.0
Cxcl12	5E-85	1.1	Slc16a2	6E-40	1.0
1500009L16Rik	5E-26	1.1	Usp35	3E-03	1.0
H2-M3	3E-15	1.1	Ralgds	2E-12	1.0
Gm17057	2E-04	1.1	Selenop	2E-43	1.0
Apol9a	2E-14	1.1	Ssh3	5E-03	1.0
Il13ra1	2E-15	1.1	Cyp1b1	1E-39	1.0
Sp140	1E-11	1.1	Sp140	4E-05	1.0
Slc6a17	2E-06	1.1	Eepd1	9E-05	1.0
Gm14488	1E-05	1.1	Pi15	2E-06	1.0
Bcl3	8E-13	1.0	AI413582	2E-26	1.0
Nlgn1	2E-03	1.0	Gldn	1E-11	1.0
Gstt1	5E-47	1.0	Maob	2E-06	1.0
Srgap1	1E-39	1.0	Rnf122	1E-04	0.9

Limb Mesenchyme					
Control			NICD2		
Gene	p	FC	Gene	p	FC
Ust	4E-35	0.8	Kctd14	4E-11	0.8
Tasp1	1E-55	0.8	Gprc5c	1E-13	0.8
4933400A11Rik	3E-10	0.8	Megf9	5E-27	0.8
Ar	3E-31	0.8	Plagl1	2E-18	0.8
Pakap	9E-49	0.8	Pakap.1	6E-54	0.8
Sdk2	1E-48	0.8	Isg20	4E-27	0.8
Cyp2c23	6E-11	0.8	Aldh1l2	5E-58	0.8
Gm43126	3E-12	0.8	Robo2	8E-37	0.8
Gm47271	5E-23	0.8	Lpar4	2E-46	0.8
Efna5	4E-62	0.8	Synpo	2E-29	0.8
Nuak1	4E-43	0.8	Plxnb1	1E-12	0.8
Pcdh9	7E-48	0.8	Gm13986	5E-25	0.8
Ak5	1E-41	0.8	Ccdc141	2E-07	0.8
Paqr3	2E-30	0.8	Paqr3	5E-21	0.8
Plscr2	1E-27	0.8	Ccnd2	5E-114	0.8
Igsf10	1E-62	0.8	Bcat1	8E-29	0.8
Kcnq5	6E-57	0.8	Gm15910	4E-07	0.8
Nsun4	3E-24	0.8	Zfp516	5E-42	0.8
Cdkl5	2E-43	0.8	Gm6994	9E-09	0.8
Brwd3	4E-17	0.8	Flrt3	2E-14	0.7
Flnb	3E-71	0.8	Ccnd1	9E-62	0.7
Cdk6	1E-14	0.8	Trib2	2E-24	0.7
Cpa6	9E-03	0.8	Igf2	3E-35	0.7
Sync	7E-09	0.8	Plxnc1	1E-09	0.7
Gm4876	9E-23	0.8	1700024B18Rik	5E-10	0.7
Tmtc1	5E-29	0.8	Fgfr2	4E-67	0.7
Tanc1	6E-37	0.8	Nsun4	6E-20	0.7
Agbl3	2E-15	0.8	Itga8	3E-15	0.7
Sox6	3E-89	0.8	Syt1	8E-13	0.7
Dusp6	6E-14	0.8	Gm10848	2E-09	0.7
Plag1	1E-20	0.8	Sox4	9E-73	0.7
Rbp1	2E-28	0.8	9630014M24Rik	5E-12	0.7
Fam20a	1E-24	0.8	Bmp4	1E-39	0.7
Tcfl5	5E-17	0.8	Shisa4	7E-40	0.7
Actn1	5E-81	0.7	Braf	3E-62	0.7
Fat1	3E-86	0.7	Mthfd2	3E-60	0.7
Rps6ka2	1E-12	0.7	Ppp4r4	9E-07	0.7
App	4E-144	0.7	Gm13919	2E-13	0.7
Synpo	7E-40	0.7	Elmo1	3E-23	0.7
4930533B01Rik	1E-17	0.7	Tead4	4E-08	0.7
Pag1	8E-20	0.7	Cul1	1E-56	0.7
Megf9	3E-26	0.7	Tns3	2E-64	0.7
Agap1	3E-53	0.7	Cpa6	2E-04	0.7
Shisa4	2E-54	0.7	Foxc1	2E-60	0.7
Rnf144b	6E-13	0.7	Sox6	2E-77	0.7
Foxp2	1E-58	0.7	Ammecr1	2E-36	0.7
Abi3bp	9E-122	0.7	Cnn2	9E-64	0.7
Igfbp2	4E-37	0.7	Jph2	3E-11	0.7
B830012L14Rik	5E-32	0.7	Pcdhga12	5E-03	0.7
Irs1	1E-37	0.7	Zbtb7c	2E-15	0.7
Adcy7	6E-06	0.7	Itgb8	1E-08	0.7
Rgs7bp	2E-07	0.7	Zfp827	2E-20	0.7
Epha5	3E-17	0.7	Zzef1	6E-37	0.7
Fzd8	1E-39	0.7	Nr4a2	4E-15	0.7
Sox4	3E-76	0.7	Rnf180	5E-07	0.7
Trib3	2E-45	0.7	Ldlrad3	2E-37	0.7
Col1a1	9E-146	0.7	Thrb	7E-11	0.7
Sinhcaf	5E-15	0.7	Tcfl5	1E-16	0.7
Hmgcll1	1E-20	0.7	Gm26827	5E-16	0.7
Vldlr	6E-32	0.7	Nipsnap1	2E-21	0.7
Prss23	1E-80	0.7	Plpp1	1E-91	0.7
Col1a2	7E-166	0.7	Rnf144b	2E-08	0.7
Cntnap2	1E-09	0.7	Gm50232	2E-06	0.7
Sorbs2os	2E-14	0.7	4930444A19Rik	8E-09	0.7
Creb3l1	3E-57	0.7	Rflnb	6E-13	0.7
Map2	2E-24	0.7	Agap1	1E-37	0.7
Itga11	3E-27	0.7	Ak5	1E-30	0.7
Kalrn	5E-21	0.7	Maf	1E-50	0.7
Stxbp2	5E-11	0.7	Gm45669	2E-05	0.7
Nebl	7E-15	0.7	Kalrn	2E-22	0.7
Foxd2os	5E-30	0.7	4933400C23Rik	8E-27	0.7
4930512B01Rik	2E-09	0.7	Gm10563	1E-05	0.7
Pycr1	2E-41	0.7	Ar	6E-16	0.7

Chondrogenic					
Control			NICD2		
Gene	p	FC	Gene	p	FC
Sox5	2E-210	1.2	Slc7a3	2E-18	1.2
Gm19705	5E-19	1.2	Pdgfc	7E-14	1.2
Mtus2	4E-26	1.2	Igf2	3E-65	1.2
Kctd14	3E-26	1.2	Ehd3	6E-24	1.2
Col12a1	1E-184	1.2	Sgk3	2E-42	1.2
Gm11146	7E-14	1.2	Smad9	1E-13	1.2
Cpe	3E-284	1.2	Shroom2	2E-13	1.2
App	0E+00	1.2	Barx1	1E-70	1.2
Gm10848	9E-28	1.2	Fchsd2	9E-84	1.2
Trpm6	1E-19	1.2	Pik3cb	1E-20	1.2
Adamts9	7E-36	1.2	Rgp1	2E-18	1.2
Flnb	6E-130	1.2	Itga3	8E-13	1.2
S100a1	1E-171	1.2	Jph1	3E-08	1.2
Prkg1	3E-196	1.2	Enpp2	4E-31	1.2
2610035D17Rik	1E-31	1.2	Gm15414	7E-10	1.2
Trp63	2E-43	1.2	Dcaf12l1	3E-18	1.2
Dhrs3	1E-102	1.2	Dhrs3	4E-75	1.2
Bcl6	2E-104	1.2	Cst6	2E-65	1.2
Fchsd2	7E-112	1.2	Samd4	1E-111	1.1
Samd4	5E-174	1.1	Sorl1	9E-21	1.1
Aldh1a1	2E-78	1.1	Foxc1	1E-137	1.1
Nupr1	6E-54	1.1	Cdkl5	3E-47	1.1
Mindy4	3E-40	1.1	9530026P05Rik	1E-32	1.1
Pcdh11x	1E-105	1.1	Tmem8b	3E-14	1.1
Gm42788	5E-15	1.1	Ism1	1E-47	1.1
Gm9968	3E-14	1.1	Ogn	3E-165	1.1
Kcnma1	9E-22	1.1	Flnb	3E-103	1.1
1700023F06Rik	8E-15	1.1	S100a1	6E-117	1.1
Arhgef3	2E-49	1.1	Aldh1a1	1E-38	1.1
Dcaf12l1	3E-23	1.1	Dab2	4E-141	1.1
Rnf144b	1E-19	1.1	Rapgef4	3E-09	1.1
Itgbl1	3E-125	1.1	2500002B13Rik	3E-11	1.1
9530026P05Rik	5E-41	1.1	Fhl1	2E-76	1.1
Lmo7	7E-123	1.1	Lmcd1	1E-49	1.1
Cables1	6E-11	1.1	Pde7b	2E-34	1.1
Baiap2l1	3E-59	1.1	Rab27b	3E-15	1.1
Boc	8E-113	1.1	Rnf32	1E-12	1.1
D630003M21Rik	2E-20	1.1	Plod2	2E-139	1.1
Gm43948	3E-20	1.1	Aig1	8E-91	1.1
Ctf1	6E-36	1.1	Daam2	2E-45	1.1
Gm12811	3E-24	1.1	Csmd1	2E-23	1.1
Map7d3	5E-23	1.1	Kcnma1	1E-06	1.1
Gm13269	4E-20	1.1	Trib2	7E-23	1.1
Pik3cb	2E-22	1.1	Pdgfrl	2E-48	1.1
Pdgfrl	5E-68	1.1	Ooep	4E-27	1.1
Adhfe1	5E-29	1.1	9630014M24Rik	1E-08	1.1
Pdzk1ip1	7E-12	1.1	Masp1	5E-21	1.1
Slc7a3	2E-17	1.1	Nupr1	3E-33	1.1
Tiparp	4E-104	1.1	2610035D17Rik	1E-20	1.1
Cacnb2	3E-91	1.1	Prkg1	4E-124	1.1
Cdc42bpa	5E-146	1.1	Zfp612	1E-15	1.1
Fzd6	4E-15	1.1	Tmtc4	5E-13	1.1
Ctnnal1	1E-47	1.1	Mtcl1	4E-07	1.1
Mdfi	9E-26	1.1	Gm9968	6E-10	1.1
Adm2	3E-19	1.1	Stc2	4E-11	1.1
Thbs1	7E-97	1.1	Adm2	8E-12	1.1
Mboat2	6E-29	1.1	Chst11	8E-27	1.0
Tor4a	1E-09	1.1	Fgf14	3E-16	1.0
Slc16a4	7E-32	1.1	Hmgcll1	3E-27	1.0
Nipal3	6E-14	1.1	Gm10974	5E-12	1.0
Foxc1	4E-165	1.1	Matn4	4E-15	1.0
Mtcl1	5E-19	1.1	Gm45338	3E-11	1.0
Fjx1	1E-16	1.1	Apbb2	6E-135	1.0
Mei4	2E-11	1.1	Tcf4	2E-228	1.0
Gm26911	6E-22	1.0	Mdfi	2E-15	1.0
Nos1ap	3E-15	1.0	Mindy4	9E-20	1.0
Daam2	1E-60	1.0	Tiam2	1E-98	1.0
Hecw2	7E-38	1.0	Gm16152	6E-10	1.0
Etv1	2E-15	1.0	Hivep2	2E-56	1.0
Spp1	4E-11	1.0	Gm4876	2E-29	1.0
Tiam2	3E-114	1.0	Bcl6	7E-51	1.0
Slc22a4	1E-12	1.0	Sema3a	3E-43	1.0
Gm4876	2E-30	1.0	Lin7a	3E-12	1.0

Fibroblast					
Control			NICD2		
Gene	p	FC	Gene	p	FC
Heg1	3E-89	1.0	Cnn1	2E-14	1.0
Ephb2	2E-142	1.0	Dpyd	4E-35	1.0
Lrp1b	5E-27	1.0	Gxylt2	8E-175	1.0
Lrrc4c	5E-50	1.0	Ifi204	6E-61	1.0
Pcdh18	5E-297	1.0	C1qtnf2	6E-34	1.0
Hoxc11	2E-80	1.0	Adamts12	2E-97	1.0
Gfra1	3E-80	1.0	Hoxc13	1E-34	1.0
Tpbg	8E-133	1.0	Rspo3	8E-222	1.0
Fzd4	8E-109	1.0	Tpbg	2E-73	1.0
Kif1a	3E-145	1.0	Trim30d	4E-69	1.0
Atp1b1	1E-92	1.0	Ptger4	5E-19	1.0
Ifi204	4E-126	1.0	Itga11	1E-86	1.0
Ptx3	0E+00	1.0	Gbp3	1E-30	1.0
Cacna1a	3E-225	1.0	Chst15	2E-22	1.0
Plscr2	8E-114	1.0	Fmo1	2E-34	1.0
Ptger4	4E-38	1.0	Cacna1a	3E-105	1.0
Fabp3	2E-114	1.0	Kif1a	3E-64	1.0
Nova1	1E-26	1.0	Cnrip1	1E-109	1.0
Cyp26b1	7E-58	1.0	Ccn3	9E-190	1.0
Cttnbp2	3E-124	1.0	Lrrc4c	4E-25	1.0
Nid1	0E+00	1.0	Gm16083	2E-27	1.0
Fmo1	8E-63	1.0	Plscr2	4E-55	1.0
Cacna1c	3E-226	1.0	Pygb	1E-90	1.0
Il1r1	0E+00	1.0	Prickle1	4E-134	1.0
Pdlim1	4E-133	1.0	Eya2	2E-10	1.0
Cnrip1	1E-213	1.0	Gucy1a1	2E-24	1.0
Gpx3	6E-94	1.0	Ephb2	3E-58	1.0
Ednra	5E-185	1.0	Sfrp2	0E+00	1.0
Gas2	0E+00	1.0	Mycbp2	1E-297	1.0
Sorcs2	1E-93	1.0	Hic1	2E-53	1.0
Tmem158	1E-115	1.0	Tmem158	2E-48	1.0
Ntn1	1E-58	1.0	Astn2	1E-27	1.0
Slc5a3	2E-128	1.0	Gpx3	2E-48	1.0
Prr5	8E-80	1.0	Ednra	5E-128	1.0
Itga11	2E-140	1.0	Mab21l1	2E-20	1.0
Pde1a	2E-165	0.9	Eln	3E-94	1.0
Aldh3a1	3E-22	0.9	Gm26771	2E-150	1.0
Tspan18	6E-92	0.9	Aldh3a1	2E-12	1.0
Gprin3	1E-71	0.9	Fgf18	3E-32	1.0
Gsdmd	4E-99	0.9	4933431K23Rik	2E-25	1.0
Frmd4a	2E-92	0.9	4833422C13Rik	3E-18	1.0
Plcl1	2E-213	0.9	Hoxd11	3E-22	1.0
4931406P16Rik	6E-144	0.9	Plxna4	5E-40	1.0
Tbc1d2b	1E-208	0.9	4931406P16Rik	2E-86	1.0
Adh7	4E-71	0.9	Fstl1	0E+00	1.0
Ebf3	4E-231	0.9	Igsf3	5E-43	1.0
Slc44a2	1E-172	0.9	Slc5a3	1E-50	1.0
C1qtnf2	6E-53	0.9	Gsdmd	2E-44	1.0
Prrg3	4E-93	0.9	Cpxm1	3E-157	0.9
Bvht	6E-49	0.9	Pde1a	1E-82	0.9
Plxna4	8E-77	0.9	Spry1	4E-25	0.9
Astn2	4E-51	0.9	Il1r1	6E-197	0.9
Sertad4	6E-95	0.9	Efemp1	2E-148	0.9
Fstl1	0E+00	0.9	Slc44a2	2E-92	0.9
Mcc	0E+00	0.9	Sytl2	1E-46	0.9
Oxr1	9E-108	0.9	Frmd4a	2E-55	0.9
Il1rap	1E-126	0.9	Gfpt2	6E-24	0.9
5930403N24Rik	5E-34	0.9	Gas2	3E-233	0.9
Sfrp2	0E+00	0.9	Ntn1	4E-26	0.9
Adamts12	2E-167	0.9	1500009L16Rik	5E-63	0.9
Ror1	5E-294	0.9	Gm12963	8E-19	0.9
Tbkbp1	4E-43	0.9	Sorcs2	7E-41	0.9
Chst15	2E-40	0.9	Prr5	7E-45	0.9
Lgals9	2E-140	0.9	Fabp3	9E-55	0.9
Prickle1	2E-231	0.9	Xkr4	5E-18	0.9
Mab21l1	2E-34	0.9	Inhba	3E-36	0.9
BC028528	4E-102	0.9	Il1rap	3E-52	0.9
Prss35	7E-15	0.9	Rab32	3E-72	0.9
Mycbp2	0E+00	0.9	4921539H07Rik	1E-13	0.9
Gm16083	3E-56	0.9	Podn	2E-42	0.9
1500009L16Rik	2E-105	0.9	Mcc	3E-147	0.9
Eya2	5E-15	0.9	Map1b	6E-114	0.9
Plekha4	3E-59	0.9	Negr1	5E-47	0.9

Fibroblast?					
Control			NICD2		
Gene	p	FC	Gene	p	FC
Rrm1	0E+00	3.6	Atad2	0E+00	3.6
Ccne1	0E+00	3.6	Hist1h3a	7E-142	3.6
Lmnb1	0E+00	3.6	Plk4	0E+00	3.6
Plk4	0E+00	3.5	Kif18a	0E+00	3.6
Mybl1	0E+00	3.5	Ulbp1	6E-190	3.6
Cep128	0E+00	3.5	Rrm1	0E+00	3.6
Hist1h2bf	1E-141	3.5	Fen1	0E+00	3.6
Hells	0E+00	3.5	Traip	0E+00	3.5
Kpna2	0E+00	3.5	Lig1	0E+00	3.5
Atad2	0E+00	3.5	Dbf4	0E+00	3.5
Rad18	0E+00	3.5	Cep128	0E+00	3.5
Fen1	0E+00	3.5	Arhgap19	2E-305	3.5
Fam111a	0E+00	3.5	Ccne1	0E+00	3.5
Dhfr	0E+00	3.5	Tube1	3E-306	3.5
Wdhd1	0E+00	3.4	Rad18	0E+00	3.5
2810429I04Rik	3E-92	3.4	Rtkn2	1E-184	3.4
Mcm8	0E+00	3.4	E2f2	1E-254	3.4
Nrm	0E+00	3.4	Dck	0E+00	3.4
Traip	0E+00	3.4	Mcm8	0E+00	3.4
Chaf1a	0E+00	3.4	Cenpl	0E+00	3.4
Hist1h2ad	5E-95	3.4	Ddias	0E+00	3.4
Rgs9	2E-90	3.3	Nrm	0E+00	3.3
Rad54l	0E+00	3.3	Prim1	0E+00	3.3
Lig1	0E+00	3.3	Wdhd1	0E+00	3.3
Blm	0E+00	3.3	Kif24	0E+00	3.3
Nek3	3E-216	3.3	Tnfaip8l1	1E-210	3.3
Tonsl	2E-278	3.3	Kpna2	0E+00	3.3
Kif24	0E+00	3.3	Cdc7	0E+00	3.3
Arhgap19	2E-263	3.3	Hist2h2bb	3E-240	3.3
Prim1	0E+00	3.3	Chaf1a	0E+00	3.2
Ccsap	7E-267	3.3	5430427M07Rik	9E-171	3.2
Tyms	0E+00	3.3	Psmc3ip	0E+00	3.2
Cenpl	0E+00	3.2	Blm	0E+00	3.2
Ddias	0E+00	3.2	Nek3	6E-245	3.2
E2f2	9E-220	3.2	Cdca7l	0E+00	3.2
Tube1	3E-241	3.2	Hist1h3e	0E+00	3.2
Mms22l	0E+00	3.1	Tyms	0E+00	3.2
Wdr90	1E-246	3.1	Zgrf1	0E+00	3.2
Mboat1	3E-258	3.1	Mboat1	0E+00	3.2
Hist1h3e	0E+00	3.1	Mms22l	0E+00	3.2
Dck	0E+00	3.1	Cenps	0E+00	3.2
Pkn3	2E-142	3.1	Suv39h2	0E+00	3.1
Cdt1	0E+00	3.1	Tonsl	0E+00	3.1
Hist1h3a	2E-111	3.1	Mis18a	0E+00	3.1
Rfc5	0E+00	3.1	Brca2	0E+00	3.1
Zgrf1	0E+00	3.1	Rfc5	0E+00	3.0
Brca2	0E+00	3.0	Dna2	5E-282	3.0
Hist2h2bb	6E-167	3.0	Ccsap	1E-274	3.0
Tnfaip8l1	4E-187	3.0	Pkn3	4E-128	3.0
Dna2	2E-201	3.0	Wdr62	6E-247	3.0
Mis18a	0E+00	3.0	Gins2	0E+00	3.0
Psmc3ip	0E+00	3.0	Mcm3	0E+00	3.0
Gm3550	1E-139	3.0	Bora	0E+00	3.0
Bora	3E-293	3.0	Ung	8E-132	3.0
Smc1b	9E-78	3.0	Gm3550	1E-148	3.0
Mcm3	0E+00	3.0	Rpa2	0E+00	3.0
Haus5	1E-267	3.0	Timeless	0E+00	3.0
Slc43a3	0E+00	3.0	Cenpp	0E+00	2.9
Cdc7	3E-262	3.0	Pidd1	7E-156	2.9
Cep72	3E-167	2.9	Cdt1	0E+00	2.9
Cenps	0E+00	2.9	Raet1d	0E+00	2.9
Cdc25b	0E+00	2.9	Atad5	0E+00	2.9
Atad5	0E+00	2.9	Pola1	0E+00	2.9
Timeless	3E-285	2.9	Slc43a3	0E+00	2.9
Wdr62	1E-203	2.9	Chek1	0E+00	2.9
Gins3	6E-302	2.9	Haus5	2E-296	2.9
Gins2	0E+00	2.9	Bok	0E+00	2.9
Lmnb2	3E-229	2.9	Zfp367	0E+00	2.9
H2afx	3E-299	2.9	Cdc25b	0E+00	2.9
Cenpp	0E+00	2.9	Usp51	6E-93	2.9
Suv39h2	1E-237	2.8	H2afx	0E+00	2.8
Cdca7l	5E-241	2.8	Hjurp	0E+00	2.8
Rpa2	0E+00	2.8	Wdr90	2E-238	2.8

Undefined					
Control			NICD2		
Gene	p	FC	Gene	p	FC
Mgp	5E-27	0.4	Antxr2	6E-10	0.4
Clba1	3E-04	0.4	Hpgd	6E-01	0.4
Smad3	2E-15	0.4	Mrgprf	4E-03	0.4
Gbp6	1E-01	0.4	Pex11a	2E-03	0.4
1700123M08Rik	3E-06	0.4	Lum	3E-37	0.4
Slc23a2	2E-05	0.4	Tcp11l2	8E-05	0.4
Mrgprf	1E-04	0.4	Runx1t1	3E-33	0.3
Tbx18	7E-11	0.4	Rora	2E-42	0.3
4933400C23Rik	4E-07	0.4	C430049B03Rik	2E-07	0.3
Spa17	5E-02	0.4	Kcnd2	8E-01	0.3
Gm15411	6E-03	0.4	Cst3	1E-95	0.3
Gm16070	8E-07	0.4	Morn4	6E-02	0.3
Sntb2	1E-38	0.4	Cxcl1	6E-01	0.3
Podxl2	2E-10	0.4	Trps1	7E-52	0.3
Sap30	9E-12	0.4	Calhm6	8E-01	0.3
Rap1gap2	2E-02	0.4	2810457G06Rik	1E+00	0.3
Lysmd4	4E-08	0.4	Hmga2	5E-01	0.3
Runx1t1	5E-41	0.4	Tmem69	5E-01	0.3
Gata2	3E-02	0.4	Zfp58	7E-01	0.3
Fbln7	3E-09	0.4	Mab21l2	1E-17	0.3
Tspan9	1E-09	0.4	Lysmd4	2E-04	0.3
Itm2a	1E-52	0.4	Mettl18	6E-01	0.3
Gm26887	5E-04	0.4	Gulp1	3E-27	0.3
Ooep	5E-06	0.4	Gm13056	4E-03	0.3
Tbpl1	2E-07	0.4	Grhpr	3E-01	0.3
Lcn2	3E-11	0.4	Fgf13	8E-01	0.3
Nt5dc2	8E-01	0.4	Zfp566	6E-02	0.3
Tbx4	2E-12	0.4	Gm8739	6E-04	0.3
Lmo1	2E-07	0.4	Eps8l2	2E-05	0.3
Cib2	3E-10	0.4	Rtl8b	8E-07	0.3
Syt12	8E-02	0.4	Mt1	9E-20	0.3
Mt2	7E-16	0.4	Nat14	1E-01	0.3
Gm4876	4E-06	0.4	Igsf10	9E-20	0.3
Mtcl1	9E-01	0.4	Sntb2	3E-38	0.3
Sox6	5E-66	0.4	Ralgds	2E-04	0.3
Gm10125	8E-03	0.4	Cmss1	2E-04	0.3
Brf2	6E-01	0.3	Aox1	3E-04	0.3
Vit	5E-01	0.3	Abcc4	2E-02	0.3
Cpq	1E-69	0.3	Cd80	2E-01	0.3
Lum	1E-29	0.3	Gm20631	2E-01	0.3
Tle1	2E-07	0.3	Shisa4	8E-13	0.3
Pnmal2	4E-03	0.3	Slc9a9	2E-16	0.3
Ccdc173	2E-02	0.3	Zfp827	2E-06	0.3
Xrcc5	2E-10	0.3	Hspb6	8E-07	0.3
Mertk	4E-04	0.3	Gypc	6E-02	0.3
Cdh13	2E-08	0.3	Lmo1	1E-06	0.3
Ccn5	3E-42	0.3	Sept6	2E-06	0.3
Igsf10	6E-27	0.3	Il1rl1	2E-01	0.3
Tctn2	2E-02	0.3	Dhrs3	1E-09	0.3
Colec12	9E-35	0.3	Cdkn1c	3E-20	0.3
Timp3	2E-24	0.3	Sft2d2	1E-06	0.3
Nipsnap1	9E-04	0.3	Vgll4	8E-17	0.3
Sdc4	5E-32	0.3	Enpp2	5E-10	0.3
Shisa4	3E-18	0.3	Mfge8	4E-36	0.3
Epha2	1E-01	0.3	Nipsnap1	7E-02	0.3
Cmss1	7E-08	0.3	Hmga1	6E-01	0.3
Nprl2	1E-01	0.3	Trmt5	6E-01	0.3
Hoxd10	5E-02	0.3	Ltbp1	2E-10	0.3
Slc6a17	2E-01	0.3	Cirbp	2E-06	0.3
Bdnf	2E-01	0.3	Rab11fip5	7E-04	0.3
Ubxn11	3E-01	0.3	Eps8	2E-10	0.3
Plcd3	1E-03	0.3	Atxn3	7E-03	0.3
Gabarapl1	6E-15	0.3	Gm26936	7E-01	0.3
Plat	2E-07	0.3	Rbpj	4E-22	0.3
Daam2	2E-10	0.3	Hoxa11	1E-06	0.3
Smox	8E-01	0.3	Tmem100	9E-01	0.3
Scx	5E-04	0.3	Ccdc68	1E-01	0.3
Gm15563	3E-02	0.3	Fam241b	8E-01	0.3
Tmem154	9E-03	0.3	Tbx4	1E-08	0.3
Stc1	7E-01	0.3	Rsph9	3E-04	0.3
Junos	4E-03	0.3	Gas1	2E-34	0.3
Aig1	8E-24	0.3	Srgap3	4E-26	0.3
Ccdc86	4E-04	0.3	Nrep	9E-01	0.3

Articular/Synovial Fibroblast					
Control			NICD2		
Gene	p	FC	Gene	p	FC
Fignl2	5E-04	1.0	Adgrd1	3E-05	0.9
Nsun7	2E-04	1.0	Fbxo32	2E-04	0.9
Gm7030	1E-06	1.0	S100a4	8E-36	0.9
Sms	3E-19	1.0	Efemp1	2E-34	0.9
Wnk4	2E-04	1.0	Slc27a1	3E-09	0.9
Lrmda	1E-30	1.0	Rin2	2E-37	0.9
Twf2	8E-25	1.0	Car11	6E-06	0.9
Adamts1	7E-35	1.0	Tgfbr3	4E-57	0.9
Rasa3	1E-28	1.0	Dtx4	1E-02	0.9
Medag	2E-42	1.0	Ppargc1a	5E-04	0.9
Clec14a	4E-10	1.0	Ctsk	3E-29	0.9
Stc1	3E-10	1.0	Acy3	2E-03	0.9
Cd248	4E-71	1.0	Angptl2	1E-10	0.9
Stom	6E-15	1.0	Mrgprf	1E-08	0.9
Pdzk1ip1	1E-05	1.0	Pik3ip1	7E-20	0.9
Ssc5d	4E-45	1.0	Trerf1	4E-22	0.9
Loxl1	1E-66	1.0	Ifit3	1E-13	0.9
Ahnak2	2E-60	1.0	Fignl2	6E-03	0.9
Enpep	8E-06	1.0	Mettl27	5E-05	0.9
Nrn1	2E-21	1.0	Stac	4E-01	0.9
Pcp4l1	4E-26	1.0	Fxyd1	1E-54	0.9
Ccl2	7E-07	1.0	Map2k6	2E-08	0.9
Unc13b	6E-06	1.0	Zfp385a	8E-04	0.9
Mab21l1	5E-05	1.0	Dapk1	2E-31	0.9
A4galt	7E-15	1.0	Twist2	8E-07	0.9
Rgs2	7E-16	1.0	P2rx4	3E-22	0.9
Fstl1	2E-105	1.0	Nr3c2	1E-04	0.9
Sirpa	2E-20	1.0	Sema3b	9E-09	0.9
Vill	2E-02	1.0	Dhx58os	6E-08	0.9
Rgs17	2E-24	1.0	Slc4a4	2E-03	0.9
Fnip2	1E-10	1.0	Abr	2E-05	0.9
Arhgap24	4E-57	1.0	Ralgps1	2E-02	0.9
Zfp874b	4E-06	1.0	Il1r1	1E-48	0.9
Pdgfra	2E-54	1.0	Enpep	6E-03	0.9
Nfkbiz	2E-21	1.0	Ksr1	1E-03	0.9
Sbno2	3E-20	1.0	Rab3il1	2E-08	0.9
Cela1	8E-08	1.0	Slc6a17	4E-02	0.9
Ksr1	6E-07	1.0	Fbln1	5E-28	0.9
Arsj	2E-19	1.0	Dpyd	3E-08	0.9
Zfp36l1	1E-52	1.0	Psmb8	3E-23	0.9
Nav1	2E-24	1.0	Gas1	2E-75	0.9
9530062K07Rik	9E-03	1.0	Fat3	5E-03	0.9
Adam32	3E-03	1.0	Fam241b	2E-05	0.9
Psmb9	3E-15	1.0	Colec12	1E-69	0.9
Pm20d1	4E-05	1.0	Adam33	3E-09	0.9
Ctsk	1E-30	1.0	Spink10	6E-02	0.9
2310001H17Rik	1E-09	1.0	Ddx60	5E-04	0.9
H2-T23	6E-29	0.9	Iigp1	4E-11	0.9
Pax9	2E-03	0.9	Sbno2	3E-14	0.9
Col18a1	8E-36	0.9	Tgfbi	9E-12	0.9
Efemp1	5E-53	0.9	Zfp874b	9E-03	0.9
Ugdh	1E-48	0.9	St3gal1	5E-23	0.9
Acy3	5E-05	0.9	Tuba4a	7E-04	0.9
Pltp	2E-47	0.9	Adamts1	4E-12	0.9
Pcbd1	7E-08	0.9	Cyp2u1	4E-02	0.9
Slc25a42	1E-03	0.9	Zfyve26	2E-04	0.9
Klf4	6E-37	0.9	Mafb	6E-20	0.9
Serpinb6a	2E-105	0.9	Acp5	3E-02	0.9
Slfn8	9E-09	0.9	E130102H24Rik	8E-08	0.9
Plau	7E-04	0.9	Tmem106a	1E-03	0.9
Gm15261	4E-14	0.9	Pcp4l1	3E-12	0.9
Plscr4	4E-12	0.9	A330076H08Rik	5E-08	0.9
Mt1	2E-80	0.9	Gm12963	6E-02	0.9
Scara3	6E-23	0.9	Pla2r1	3E-08	0.9
Phka2	1E-16	0.9	Gbp6	2E-04	0.9
Phf11d	1E-18	0.9	Rasa3	6E-17	0.9
Pde1a	7E-30	0.9	Gprc5b	3E-03	0.9
Abr	4E-09	0.9	Fgf18	2E-05	0.9
Phlda1	1E-02	0.9	Il16	6E-09	0.9
Uap1l1	1E-34	0.9	Fdxr	1E-05	0.9
Ampd3	3E-11	0.9	Inka2	2E-03	0.9
Eno2	3E-06	0.9	Slc25a21	7E-03	0.9
Lgals9	6E-22	0.9	Arhgap24	1E-40	0.9

Limb Mesenchyme					
Control			NICD2		
Gene	p	FC	Gene	p	FC
Plpp1	8E-106	0.7	Gm47271	3E-16	0.7
Snhg5	2E-28	0.7	Nid2	7E-30	0.7
Mab21l2	5E-54	0.7	Gm43728	3E-09	0.7
Cplx2	8E-11	0.7	Fat1	2E-73	0.7
Sobp	4E-41	0.7	Gm26981	2E-03	0.7
Cdh11	2E-65	0.7	Slc1a4	1E-26	0.7
Tprg	2E-20	0.7	Adamtsl3	3E-35	0.7
Prrx1	2E-78	0.7	Map4k3	2E-43	0.7
Il1rapl1	2E-42	0.7	Tmem8b	1E-12	0.7
1700109K24Rik	3E-15	0.7	Sntg2	2E-10	0.7
H2afy2	9E-40	0.7	Cnksr3	1E-31	0.7
Hbegf	3E-26	0.7	Nebl	8E-13	0.7
Cemip	2E-25	0.7	Lrch1	2E-34	0.7
Ackr4	2E-07	0.7	Arl4c	2E-14	0.7
Cald1	1E-125	0.7	Fscn1	2E-54	0.7
Slc26a2	9E-23	0.7	Slc7a1	1E-36	0.7
Gars	2E-87	0.7	Slit2	5E-05	0.7
Stbd1	1E-20	0.7	5430416N02Rik	3E-25	0.7
Tiam2	2E-66	0.7	Igfbp2	2E-27	0.7
Samd4	6E-64	0.7	Med12l	1E-08	0.7
Ltbp2	1E-42	0.7	Rab3b	6E-07	0.7
Cdc42ep3	2E-50	0.7	4930545L23Rik	8E-12	0.7
Plk2	5E-38	0.7	Gm16552	3E-04	0.7
Mkx	6E-21	0.7	Sobp	3E-42	0.7
Tab2	3E-52	0.7	Cyb5r2	3E-18	0.7
Pxdc1	3E-15	0.7	Tsix	9E-11	0.7
Iars	9E-60	0.7	Zbtb18	1E-12	0.7
Nedd9	9E-48	0.7	Nuak1	3E-30	0.7
Angptl6	1E-28	0.7	4930533B01Rik	5E-09	0.7
Gm13269	2E-13	0.7	Cyp26b1	5E-02	0.7
Wnt5a	2E-27	0.7	Samhd1	3E-39	0.7
Tns3	1E-55	0.7	Ism1	1E-23	0.7
Gpc4	4E-44	0.7	Wnk3	1E-07	0.7
Gm13483	9E-20	0.7	Pdlim5	3E-49	0.7
Ppp1r12b	4E-37	0.7	Stxbp6	8E-37	0.7
Dync1i1	6E-11	0.7	Id3	3E-54	0.7
Slc1a4	6E-28	0.7	Abi3bp	2E-86	0.7
Col24a1	4E-13	0.7	Pxdc1	5E-17	0.7
Gm43376	1E-06	0.7	Mthfr	2E-19	0.7
Homer2	3E-14	0.7	Sntb1	2E-17	0.7
Asxl3	3E-12	0.7	4921539H07Rik	4E-09	0.7
Slc2a12	2E-09	0.7	Fzd6	1E-11	0.7
Gm26802	4E-12	0.7	Gm49003	7E-06	0.7
Fat4	3E-56	0.7	Cdk6	2E-18	0.7
Slc40a1	7E-29	0.7	Wnt5a	3E-31	0.7
Prss35	3E-08	0.7	Gm45338	3E-12	0.7
5430416N02Rik	2E-26	0.7	Foxd1	1E-11	0.7
Fkbp10	1E-64	0.7	Tmtc2	4E-17	0.7
Gpr27	6E-20	0.7	Snhg5	2E-18	0.7
Lrch1	2E-34	0.7	Mirg	8E-19	0.7
Fnbp1l	3E-25	0.7	Fbxl7	2E-33	0.7
Snhg12	4E-45	0.7	9630028H03Rik	2E-17	0.7
Pdlim5	4E-58	0.7	Rps6ka2	2E-07	0.7
Cnn1	1E-11	0.7	Sinhcaf	2E-10	0.7
Aldh18a1	5E-33	0.7	Ppfibp2	2E-08	0.6
Met	3E-06	0.7	Pam	3E-97	0.6
Kank1	7E-30	0.7	Gpc2	5E-09	0.6
Hps3	5E-22	0.7	Cnnm4	3E-06	0.6
Sorbs2	4E-27	0.7	B830012L14Rik	1E-19	0.6
Braf	9E-56	0.7	Siah2	7E-19	0.6
Prr7	7E-27	0.7	Prrx1	9E-60	0.6
Ctnnd1	2E-39	0.7	Zfp873	4E-05	0.6
Gpr153	6E-20	0.7	Ptch1	2E-17	0.6
Ankrd44	1E-33	0.6	Gprc5a	2E-04	0.6
Fscn1	6E-53	0.6	Mthfd1l	1E-24	0.6
Fzd3	3E-12	0.6	Cyb5r1	5E-45	0.6
Myo10	5E-53	0.6	Ntn4	5E-20	0.6
Rhobtb3	1E-62	0.6	Sema3e	2E-14	0.6
Xpot	4E-49	0.6	Malrd1	2E-07	0.6
Msrb3	1E-46	0.6	Efcab1	5E-10	0.6
Hsph1	7E-53	0.6	Gm50163	1E-05	0.6
Cyb5r1	1E-58	0.6	Gm2115	1E-10	0.6
Chsy1	5E-40	0.6	Tanc1	6E-30	0.6

Chondrogenic					
Control			NICD2		
Gene	p	FC	Gene	p	FC
Gm13056	6E-40	1.0	Capn6	8E-42	1.0
Ism1	1E-68	1.0	Mboat2	4E-25	1.0
Galnt14	2E-07	1.0	Ippk	1E-14	1.0
Dixdc1	9E-46	1.0	Efcab1	2E-16	1.0
Epha2	2E-15	1.0	5031425E22Rik	1E-71	1.0
Fgfr2	7E-183	1.0	Cdc42bpa	2E-97	1.0
Rtl1	9E-09	1.0	Satb1	4E-10	1.0
C230038L03Rik	1E-21	1.0	Fign	1E-49	1.0
Pde7b	6E-38	1.0	Gm12811	6E-11	1.0
Gm49969	2E-27	1.0	B230216N24Rik	2E-09	1.0
Ptchd4	1E-15	1.0	Thbs1	3E-68	1.0
Crim1	2E-109	1.0	Col12a1	3E-97	1.0
Apbb2	5E-157	1.0	Bicd1	1E-08	1.0
Enpp2	1E-27	1.0	App	6E-209	1.0
Ifitm10	1E-13	1.0	Cystm1	1E-05	1.0
Cyp26b1	8E-17	1.0	Hbegf	2E-32	1.0
Lrrc75b	2E-14	1.0	Bcl2	2E-16	1.0
Sorl1	3E-16	1.0	Fancc	8E-36	1.0
Hmgcll1	4E-26	1.0	Cx3cl1	5E-15	1.0
Gm15270	1E-13	1.0	Dixdc1	4E-30	1.0
Ehd3	4E-23	1.0	Atg16l2	2E-27	1.0
Cdkl5	9E-58	1.0	9630028H03Rik	8E-19	1.0
B230216N24Rik	9E-09	1.0	Npnt	1E-06	1.0
Shroom4	1E-31	1.0	Adhfe1	2E-19	1.0
Aopep	2E-273	1.0	Galnt14	1E-09	1.0
Ntng2	7E-08	1.0	Rgl1	4E-41	1.0
Bcl2	1E-18	1.0	Gm26936	7E-14	1.0
Samd12	1E-37	1.0	Cmbl	1E-07	0.9
Sema3a	7E-52	1.0	Stxbp5	1E-42	0.9
9630014M24Rik	2E-12	1.0	Nrtn	9E-10	0.9
Fxyd5	8E-62	1.0	Pcdh11x	6E-63	0.9
Tcf4	2E-252	1.0	Cyp27a1	1E-07	0.9
Gm45338	1E-14	1.0	Pde10a	2E-11	0.9
Mest	4E-99	1.0	Plekha7	1E-06	0.9
Tmtc4	4E-08	1.0	Trabd2b	2E-67	0.9
Hoxa3	8E-25	1.0	Tbx5	5E-12	0.9
Pxdc1	1E-26	1.0	Gm43948	5E-08	0.9
Ppp1r3c	1E-15	1.0	Fam131a	1E-09	0.9
Cplx2	3E-08	1.0	Slc16a4	3E-16	0.9
Cxadr	2E-11	1.0	Gm49969	1E-17	0.9
Nme5	5E-12	1.0	Shroom4	2E-16	0.9
Nsun4	6E-19	1.0	Ptchd4	5E-09	0.9
Dab2	3E-129	0.9	4930545L23Rik	6E-10	0.9
Hoxa9	1E-107	0.9	Ostn	2E-05	0.9
Tspan6	6E-118	0.9	Bend5	2E-15	0.9
Dipk2a	2E-45	0.9	Tspan6	4E-73	0.9
Acot1	3E-10	0.9	Tenm4	6E-56	0.9
Nrtn	9E-19	0.9	B3galt1	3E-31	0.9
Fbxo2	2E-37	0.9	Nme5	5E-08	0.9
Phyh	3E-54	0.9	Mab21l2	6E-46	0.9
Med12l	5E-07	0.9	Gm26911	2E-08	0.9
Synpo	4E-45	0.9	Aox1	5E-24	0.9
Mmp3	6E-07	0.9	Mbnl3	1E-09	0.9
Ccdc80	2E-218	0.9	Ctnnal1	4E-20	0.9
Fign	1E-56	0.9	Pcgf5	8E-15	0.9
Tbx5	8E-13	0.9	Aph1c	9E-08	0.9
Dhrs9	1E-45	0.9	Dusp6	5E-11	0.9
Stxbp5	1E-54	0.9	Gm15270	7E-09	0.9
Etl4	1E-35	0.9	Sim2	7E-09	0.9
4930523C07Rik	5E-35	0.9	Sulf1	1E-41	0.9
Nat14	1E-11	0.9	A830012C17Rik	4E-09	0.9
Sox11	8E-05	0.9	Samd12	4E-21	0.9
Sulf1	3E-65	0.9	Hoxa3	2E-13	0.9
Pde4dip	2E-61	0.9	Tnfaip8	4E-43	0.9
Lrrc8c	2E-18	0.9	Ica1	3E-17	0.9
Nlgn1	6E-12	0.9	4930594M22Rik	2E-07	0.9
Cdkn1c	1E-48	0.9	Nfatc1	6E-14	0.9
Stac	6E-07	0.9	Tbx15	4E-99	0.9
Hmgn5	1E-22	0.9	Ctf1	5E-23	0.9
Gli2	2E-20	0.9	Grem1	3E-03	0.9
Bend5	3E-19	0.9	Ankrd28	8E-62	0.9
Tgfb2	2E-128	0.9	Cxadr	7E-10	0.9
9630028H03Rik	3E-22	0.9	Penk	7E-37	0.9

Fibroblast					
Control			NICD2		
Gene	p	FC	Gene	p	FC
Ccn3	0E+00	0.9	F730043M19Rik	1E-20	0.9
Gzme	8E-99	0.9	Tbkbp1	5E-20	0.9
Sytl2	1E-81	0.9	Fbln1	4E-103	0.9
Pkdcc	4E-94	0.9	Calcrl	2E-16	0.9
Ly6c1	2E-267	0.9	Cttnbp2	1E-47	0.9
Twist2	9E-106	0.9	Rftn1	1E-17	0.9
Emb	0E+00	0.9	Gzme	1E-55	0.9
Kctd1	7E-156	0.9	Tln2	2E-148	0.9
Gm26771	1E-277	0.9	Flnc	5E-111	0.9
Adamtsl1	6E-144	0.9	Tbc1d2b	1E-87	0.9
Trim30d	6E-137	0.9	Mfap2	7E-188	0.9
Crlf1	0E+00	0.9	Ebf3	2E-101	0.9
Mfap2	0E+00	0.9	Adamts6	4E-30	0.9
Fblim1	9E-41	0.9	Sertad4	9E-45	0.9
Rftn1	9E-33	0.9	Pkdcc	1E-49	0.9
Gbp3	7E-48	0.9	Fam227b	6E-49	0.9
Calcrl	1E-28	0.9	Slc25a21	2E-14	0.9
Ldb2	0E+00	0.9	Apba1	3E-43	0.9
Mar3	3E-213	0.9	Bvht	2E-21	0.9
Map1b	1E-219	0.9	Trim66	2E-16	0.9
Notch3	2E-48	0.9	Gstt1	4E-104	0.9
St8sia2	5E-60	0.9	Plekha4	4E-23	0.9
Lox	0E+00	0.9	Rnase4	9E-185	0.9
Nmnat2	9E-111	0.9	Notch3	2E-38	0.9
C130021I20Rik	3E-26	0.9	Slc6a6	5E-79	0.9
Nhsl1	8E-65	0.9	BC028528	1E-55	0.9
Cebpd	1E-162	0.9	Cacna2d3	1E-09	0.9
Inhba	6E-72	0.9	Emb	3E-203	0.9
Flnc	1E-206	0.9	C130021I20Rik	1E-13	0.9
Gatb	9E-115	0.9	Layn	2E-83	0.9
Dnm3	2E-135	0.9	Lgals9	5E-56	0.9
Apba1	5E-85	0.9	Gm15867	4E-39	0.9
Palmd	3E-83	0.9	Aplp1	1E-53	0.8
Cpxm1	3E-273	0.9	Prrg3	4E-38	0.8
Xkr4	3E-31	0.9	Prss35	5E-08	0.8
4933431K23Rik	1E-35	0.9	Ror1	1E-141	0.8
Nectin2	1E-73	0.9	Ptx3	2E-160	0.8
Dnm3os	6E-285	0.8	Dnm3	4E-61	0.8
Hoxd11	2E-45	0.8	H2-DMb1	1E-11	0.8
Kif26b	1E-67	0.8	Ephx2	8E-56	0.8
Rab32	6E-113	0.8	Ldb2	3E-168	0.8
Layn	2E-173	0.8	Apoe	5E-274	0.8
Rnase4	0E+00	0.8	Nmnat2	4E-55	0.8
Nnmt	0E+00	0.8	Chst1	4E-07	0.8
Adamts6	9E-65	0.8	Mdga1	6E-32	0.8
Gldn	1E-188	0.8	Phex	4E-10	0.8
Fam227b	7E-101	0.8	Plpp3	7E-191	0.8
Arxes1	6E-34	0.8	Pdlim1	2E-61	0.8
Eda	3E-84	0.8	Vmp1	0E+00	0.8
Man1a	2E-167	0.8	Man1a	4E-81	0.8
Negr1	1E-83	0.8	Gatb	2E-45	0.8
Adam19	7E-116	0.8	Dnm3os	1E-143	0.8
Gstt1	6E-205	0.8	Fzd5	2E-32	0.8
Gfpt2	4E-45	0.8	Vstm4	5E-33	0.8
Marcks	0E+00	0.8	Mar3	8E-92	0.8
Efemp1	1E-241	0.8	Arhgef9	3E-36	0.8
Celf2	0E+00	0.8	Mmp23	2E-182	0.8
Acvr1b	4E-37	0.8	Pcdh18	4E-108	0.8
Pyroxd2	5E-33	0.8	Mmp2	6E-182	0.8
Gm15867	5E-77	0.8	Heg1	8E-32	0.8
Vstm4	1E-62	0.8	Gucy1b1	1E-18	0.8
Psmb8	1E-202	0.8	Fblim1	5E-22	0.8
Nrg1	6E-31	0.8	Plcl1	1E-100	0.8
Mdga1	2E-54	0.8	Pknox2	5E-80	0.8
Aplp1	2E-96	0.8	Acvr1b	7E-20	0.8
Fhod1	3E-45	0.8	Wfdc17	2E-42	0.8
Lgi2	4E-49	0.8	Lox	0E+00	0.8
Nox4	3E-111	0.8	Kctd1	3E-63	0.8
Hccs	2E-68	0.8	Nectin2	2E-37	0.8
Olfml3	0E+00	0.8	Pyroxd2	1E-14	0.8
Gm12963	5E-30	0.8	Oxr1	4E-46	0.8
Itsn1	1E-205	0.8	Olfml3	3E-214	0.8
Plpp3	0E+00	0.8	Chd3	1E-143	0.8

Fibroblast?					
Control			NICD2		
Gene	p	FC	Gene	p	FC
Clec2l	1E-97	2.8	Gins3	0E+00	2.8
Pola1	0E+00	2.8	Ncapd3	0E+00	2.8
Tubb6	0E+00	2.8	BC055324	4E-235	2.7
Pmaip1	2E-80	2.8	Pold1	0E+00	2.7
Chek1	3E-302	2.8	Pcna	0E+00	2.7
BC055324	4E-217	2.8	Gins1	5E-258	2.7
Zfp367	3E-303	2.8	Cdkn2d	0E+00	2.7
Poc1a	5E-227	2.8	Zfand4	2E-211	2.7
Hjurp	0E+00	2.7	Rfc4	0E+00	2.7
Zfand4	7E-170	2.7	Lmnb2	3E-267	2.7
Ncapd3	0E+00	2.7	Tubb6	0E+00	2.7
Raet1d	5E-243	2.7	Hist1h4m	1E-93	2.7
Xrcc2	1E-222	2.7	Haus4	0E+00	2.6
Cgas	1E-252	2.7	Cenpw	0E+00	2.6
Hist1h2br	2E-133	2.7	Wdr76	0E+00	2.6
Bok	1E-269	2.7	Dnajc9	0E+00	2.6
Rangap1	0E+00	2.7	Fkbp5	4E-283	2.6
Dnph1	3E-295	2.7	Donson	1E-304	2.6
Pola2	3E-290	2.6	Hist1h2br	4E-122	2.6
Sass6	0E+00	2.6	Syt9	9E-81	2.6
Pcna	1E-278	2.6	Lhx2	2E-141	2.6
Rfc4	0E+00	2.6	Cgas	1E-295	2.6
Dnajc9	0E+00	2.6	Sass6	0E+00	2.6
Adrb2	2E-122	2.6	Rangap1	0E+00	2.6
Syce2	9E-296	2.6	Palb2	7E-162	2.6
Pidd1	6E-103	2.6	Poc1a	6E-268	2.6
Palb2	1E-123	2.6	Cep72	3E-146	2.6
Cenpw	0E+00	2.6	9630013D21Rik	1E-168	2.5
Pold1	2E-254	2.6	Hist1h4d	5E-270	2.5
Hirip3	9E-303	2.6	Mcm6	0E+00	2.5
Cdkn2d	4E-243	2.6	Mcm2	0E+00	2.5
Rbl1	5E-287	2.5	Rbl1	0E+00	2.5
Lhx2	3E-87	2.5	Dnph1	0E+00	2.5
Rad51c	4E-191	2.5	Syce2	0E+00	2.5
Dnmt1	0E+00	2.5	Prim2	0E+00	2.5
Kcnn4	3E-69	2.5	Rad51c	8E-209	2.5
Nsd2	4E-276	2.5	Xrcc2	3E-204	2.5
Haus4	3E-302	2.5	Pmf1	0E+00	2.5
Wdr76	3E-265	2.5	Nsd2	0E+00	2.5
Hist1h4d	3E-181	2.5	Mcm7	0E+00	2.5
Ezh2	0E+00	2.5	Dnmt1	0E+00	2.5
Usp51	8E-68	2.5	Hirip3	0E+00	2.5
Dock3	2E-63	2.5	H2afz	0E+00	2.5
Donson	8E-201	2.5	Cdh24	2E-112	2.5
Hat1	0E+00	2.4	Tinagl1	1E-75	2.5
G2e3	5E-234	2.4	Hat1	0E+00	2.5
H2afz	0E+00	2.4	Trim59	0E+00	2.5
Dnaaf2	1E-264	2.4	Selenoh	0E+00	2.4
Prim2	4E-251	2.4	Cep152	7E-270	2.4
Mcm6	3E-281	2.4	Lin9	0E+00	2.4
Trim59	7E-265	2.4	Tex30	0E+00	2.4
Mcm7	2E-285	2.4	Haus6	0E+00	2.4
Dsn1	1E-263	2.4	Pola2	4E-295	2.4
Hist1h4n	6E-100	2.4	Vrk1	0E+00	2.4
Ung	7E-56	2.4	G2e3	1E-283	2.4
Unc13c	1E-81	2.4	Tmpo	0E+00	2.3
Cep152	7E-159	2.4	Zranb3	4E-273	2.3
Lin9	1E-256	2.4	Topbp1	0E+00	2.3
Wee1	3E-232	2.3	Adrb2	2E-98	2.3
Pmf1	7E-287	2.3	Usp1	0E+00	2.3
Tex30	2E-219	2.3	Kcnab2	1E-93	2.3
Hist1h4m	4E-92	2.3	AU020206	2E-213	2.3
Tubb4b	5E-223	2.3	Wee1	0E+00	2.3
Klhl32	2E-64	2.3	Hyls1	1E-186	2.3
Zranb3	2E-231	2.3	Ndc1	0E+00	2.3
Mcm2	4E-247	2.3	Hist1h4c	2E-174	2.3
Usp1	0E+00	2.3	Tubb4b	2E-306	2.3
Syt9	2E-66	2.3	Lin54	4E-264	2.2
Ndc1	4E-246	2.3	Syne2	5E-285	2.2
Kcnab2	9E-82	2.3	Dnaaf2	1E-305	2.2
AU020206	1E-161	2.3	Ezh2	0E+00	2.2
Fkbp5	5E-205	2.3	Dock3	8E-69	2.2
Ddx11	1E-172	2.3	Pole2	6E-264	2.2

Undefined					
Control			NICD2		
Gene	p	FC	Gene	p	FC
Acsf3	9E-01	0.3	Mecom	6E-17	0.3
Gss	2E-04	0.3	Tmub1	5E-01	0.3
Eps8	2E-18	0.3	Brd3os	1E-02	0.3
Tspan13	7E-07	0.3	Tpk1	1E-05	0.3
Coq8a	9E-02	0.3	Selenbp1	5E-05	0.3
Gm12655	6E-02	0.3	Pacrg	3E-02	0.3
Rsph9	2E-05	0.3	Myo1d	2E-24	0.3
Gnai1	1E-01	0.3	Klc2	9E-01	0.3
Cdon	2E-19	0.3	Mterf4	3E-03	0.3
Hoxd3os1	6E-01	0.3	Fat4	3E-20	0.3
L3mbtl3	3E-09	0.3	Prkaa2	1E-01	0.3
Ankrd13b	1E-01	0.3	Pik3r1	3E-09	0.3
Nr3c2	3E-03	0.3	AW011738	1E-02	0.3
Fat4	3E-23	0.3	Prdm16	4E-07	0.3
Hilpda	3E-03	0.3	Rad52	1E-01	0.3
App	5E-117	0.3	Sntg2	5E-05	0.3
Cst3	4E-97	0.3	Ism1	3E-09	0.3
Hoxa13	6E-02	0.3	Slc25a38	2E-01	0.3
Hoxa11	1E-06	0.3	Ccn5	3E-50	0.3
Cox6a2	7E-01	0.3	Btg2	7E-07	0.3
Slc25a27	5E-03	0.3	Gramd1b	4E-02	0.3
Scyl3	3E-02	0.3	Ly96	2E-07	0.3
1700084C06Rik	1E-02	0.3	Mpped2	9E-04	0.3
Vim	6E-58	0.3	Gpm6b	3E-40	0.3
Cnksr3	5E-11	0.3	Irak4	3E-04	0.3
Serpinb1a	1E-01	0.3	Pdk2	8E-01	0.3
Hivep2	9E-09	0.3	Gm26724	1E-01	0.3
Rcan1	5E-01	0.3	Rbfox1	2E-01	0.3
Fosl2	6E-16	0.3	Zfp612	5E-01	0.3
Tcn2	1E-19	0.3	Zfand1	6E-04	0.3
Gm9929	4E-01	0.3	Daam2	3E-04	0.3
Phactr1	1E-07	0.3	Rdm1	1E-02	0.3
Dph5	4E-04	0.3	Fbxo44	9E-02	0.3
Gulp1	3E-40	0.3	Cul1	3E-13	0.3
Prkaa2	9E-03	0.3	9130008F23Rik	5E-01	0.3
Pram1	1E+00	0.3	Rnf135	9E-02	0.3
Mxra8	1E-43	0.3	Gm13483	6E-03	0.3
Nrp2	7E-66	0.3	Zeb1	3E-09	0.3
Lrrc32	9E-08	0.3	Vasn	3E-09	0.3
Klc4	2E-01	0.3	Sap30	5E-06	0.3
Adamts9	5E-01	0.3	Serpina3n	1E-01	0.3
Foxred2	1E-02	0.3	Tmem132a	1E-06	0.3
4931414P19Rik	4E-02	0.3	Prkg2	5E-01	0.3
Rdh10	6E-02	0.3	Arhgap28	5E-09	0.3
Zfp821	9E-03	0.3	Cd151	4E-14	0.3
B3galt4	1E-01	0.3	Jam2	1E-03	0.3
Fam241b	5E-02	0.3	Fhl1	2E-06	0.3
Il18	5E-02	0.3	Lrba	5E-13	0.3
Klf9	6E-08	0.3	Smox	5E-02	0.3
Ypel1	6E-02	0.3	Rab3b	1E-02	0.3
Hoxa3	2E-02	0.3	Fam102a	1E-01	0.3
Creld1	6E-05	0.3	Hoxa10	1E-18	0.3
Gm27017	2E-01	0.3	Plcd1	8E-03	0.3
Neat1	5E-23	0.3	Ccdc28a	9E-01	0.3
Ephb6	2E-02	0.3	Maf1	3E-04	0.3
2810002D19Rik	7E-02	0.3	Mdfi	4E-01	0.3
Cd151	7E-18	0.3	2200002D01Rik	1E+00	0.3
Ica1	1E-01	0.3	Fiz1	3E-01	0.3
Pla2r1	1E-02	0.3	Thtpa	3E-01	0.3
Dnaaf4	7E-02	0.3	Tle1	7E-04	0.3
Maf	3E-22	0.3	Egfl7	9E-02	0.3
Ism1	2E-09	0.3	Ica1	5E-03	0.3
Tmem98	3E-07	0.3	Pik3ip1	3E-05	0.3
Mamld1	4E-03	0.3	Ntn4	2E-01	0.3
C1rl	4E-03	0.3	Zfp2	8E-02	0.3
Pkia	8E-01	0.3	Aig1	2E-11	0.3
Peg10	9E-01	0.3	Gm12743	4E-03	0.3
Pnrc1	6E-35	0.3	Fam53b	4E-03	0.3
Pawr	3E-13	0.3	Ube4bos1	2E-01	0.3
Eps8l2	1E-08	0.3	Glrx	1E-04	0.3
Dus3l	8E-01	0.3	Trabd2b	2E-12	0.3
Pik3ip1	1E-07	0.3	Rerg	6E-03	0.3
Rora	1E-30	0.3	Scx	4E-01	0.3

Articular/Synovial Fibroblast					
Control			NICD2		
Gene	p	FC	Gene	p	FC
Pacrg	2E-03	0.9	Nod1	1E-04	0.9
Zeb2	2E-70	0.9	Gm26724	2E-05	0.8
Ttyh2	4E-08	0.9	Capg	3E-49	0.8
Pik3ip1	1E-26	0.9	Anks1b	9E-01	0.8
Plekho2	3E-15	0.9	Hsd17b11	8E-07	0.8
Capg	6E-75	0.9	Ildr2	2E-09	0.8
Sh3pxd2b	9E-19	0.9	Psmb9	6E-10	0.8
Igfbp5	2E-13	0.9	Ifit1	4E-08	0.8
Slco2a1	1E-05	0.9	H2-D1	4E-50	0.8
Thrb	2E-03	0.9	Lrrc27	2E-04	0.8
Abcd2	4E-08	0.9	Bahd1	3E-02	0.8
Cdc42ep2	3E-14	0.9	Abca1	3E-09	0.8
Cacna2d1	1E-22	0.9	Ssc5d	7E-15	0.8
Mettl27	2E-08	0.9	Hoxd3os1	3E-02	0.8
Tnfaip2	5E-35	0.9	Hsd3b7	8E-07	0.8
Elmo1	2E-05	0.9	Pdgfra	6E-21	0.8
Serpinb8	1E-09	0.9	Cacna2d1	2E-14	0.8
Mterf4	3E-11	0.9	Fstl1	2E-57	0.8
Slc16a2	1E-44	0.9	Ttyh2	5E-04	0.8
Rab7b	1E-07	0.9	1500009L16Rik	2E-20	0.8
Nod1	1E-03	0.9	Ampd3	2E-06	0.8
Trerf1	4E-26	0.9	Manba	2E-02	0.8
Clec2d	7E-37	0.9	Gm11944	5E-03	0.8
Peg10	8E-03	0.9	Scn2a	6E-04	0.8
Gm41609	3E-04	0.9	Sytl2	2E-04	0.8
B230206L02Rik	3E-04	0.9	Twf2	6E-07	0.8
Fbln1	3E-29	0.9	Lipa	5E-28	0.8
Atg7	2E-13	0.9	Lrrc8e	2E-02	0.8
Gstt2	3E-04	0.9	Fnip2	2E-03	0.8
Prkag2	2E-08	0.8	Fbxo31	5E-08	0.8
Anpep	4E-07	0.8	B2m	2E-82	0.8
Mill2	7E-16	0.8	A230057D06Rik	6E-12	0.8
Zfp641	5E-03	0.8	Tle6	1E-02	0.8
Trpm3	1E-03	0.8	Tmem38a	2E-01	0.8
Selenop	6E-31	0.8	Gstm1	2E-56	0.8
Pcolce2	1E-08	0.8	Medag	2E-18	0.8
Mt2	2E-45	0.8	Gm12158	7E-04	0.8
Rnf144a	1E-09	0.8	Trnp1	4E-10	0.8
Rab3d	8E-04	0.8	Slc25a23	2E-04	0.8
Il16	1E-09	0.8	Mill2	2E-07	0.8
Nsg1	2E-22	0.8	Col5a3	2E-26	0.8
Mrgbp	1E-08	0.8	Raet1e	7E-03	0.8
Svip	3E-03	0.8	H2-K1	1E-35	0.8
Gm10125	5E-04	0.8	Ctsb	5E-72	0.8
E130102H24Rik	4E-10	0.8	Snn	2E-03	0.8
Ephb6	3E-07	0.8	Mmp2	1E-34	0.8
Tmem140	3E-09	0.8	Btbd11	4E-10	0.8
Gm4951	1E-10	0.8	Clec2d	5E-26	0.8
Hopx	7E-08	0.8	1700096K18Rik	6E-02	0.8
Ugcg	9E-11	0.8	Cst3	7E-83	0.8
1700007K13Rik	5E-03	0.8	Sulf2	4E-22	0.8
Fmo5	2E-02	0.8	Igfbp5	3E-11	0.8
Fam78b	9E-06	0.8	Espn	9E-03	0.8
Iigp1	2E-16	0.8	Cdh13	7E-12	0.8
Insyn1	8E-04	0.8	Gm16201	1E-04	0.8
Pla2r1	3E-06	0.8	Pcolce2	1E-01	0.8
1700087I21Rik	6E-03	0.8	Cd248	2E-36	0.8
H2-K1	2E-59	0.8	Htra3	6E-15	0.8
Fat3	1E-05	0.8	Serpinb8	1E-05	0.8
Arrb1	3E-02	0.8	H2-M3	2E-05	0.8
Celf5	5E-02	0.8	Limch1	3E-04	0.8
C1qtnf1	5E-09	0.8	Sms	3E-06	0.8
Zfp385b	5E-04	0.8	Edem2	9E-05	0.8
Trim21	8E-03	0.8	Arhgef10l	5E-10	0.8
Zswim6	5E-15	0.8	Vegfb	7E-27	0.8
Raet1d	1E-03	0.8	Akr1b10	3E-04	0.8
Rcan2	2E-31	0.8	Dhx58	7E-03	0.8
Usp35	3E-02	0.8	Serpinb6a	8E-54	0.8
Kdsr	2E-08	0.8	Map3k8	8E-04	0.8
Raet1e	2E-05	0.8	Mt2	1E-38	0.8
Trib1	6E-06	0.8	Pip5k1b	4E-02	0.8
2900041M22Rik	2E-02	0.8	Ezh1	5E-05	0.8
Ank3	2E-32	0.8	Tpp1	6E-33	0.8

Limb Mesenchyme

Control			NICD2		
Gene	p	FC	Gene	p	FC
Entpd7	5E-19	0.6	Klhdc10	5E-30	0.6
Gm49890	3E-08	0.6	Epb41l4b	2E-07	0.6
Siah2	5E-20	0.6	P2rx6	9E-09	0.6
Foxd1	3E-12	0.6	Hmga2	8E-60	0.6
Hapln1	2E-58	0.6	Irs1	1E-22	0.6
Ccdc80	7E-85	0.6	Gm32856	9E-08	0.6
Ccnd2	2E-94	0.6	Egfros	2E-15	0.6
Phgdh	9E-61	0.6	Scarf2	2E-29	0.6
Hdac9	4E-04	0.6	Klf15	3E-09	0.6
Nt5e	1E-15	0.6	Gm16152	2E-06	0.6
Itga1	5E-30	0.6	Ltbp2	3E-31	0.6
Etl4	7E-25	0.6	B230216N24Rik	4E-08	0.6
Gm28379	1E-05	0.6	Gm49969	3E-19	0.6
Cdk17	1E-30	0.6	Rasal2	2E-27	0.6
Schip1	2E-17	0.6	Angptl6	4E-21	0.6
Ankmy1	8E-10	0.6	Rarres1	8E-09	0.6
Sept6	9E-22	0.6	Chsy1	3E-30	0.6
Zfp637	8E-55	0.6	Cacnb2	3E-25	0.6
Cnksr3	1E-34	0.6	Hrct1	6E-09	0.6
Elmo1	1E-23	0.6	Gm35188	2E-23	0.6
Aff2	5E-21	0.6	Col2a1	1E-127	0.6
Scarf2	1E-38	0.6	Slc38a1	5E-34	0.6
Me3	4E-25	0.6	Itga11	1E-26	0.6
2410018L13Rik	2E-14	0.6	Rec114	3E-07	0.6
1190005I06Rik	7E-23	0.6	Psph	2E-32	0.6
Igsf3	3E-15	0.6	Irx3	4E-08	0.6
Zfp827	2E-27	0.6	Pak3	4E-36	0.6
Gpt2	2E-19	0.6	Iars	2E-41	0.6
Fam107b	9E-24	0.6	Axin2	4E-09	0.6
Prkaa2	2E-18	0.6	Gm4876	4E-21	0.6
Gm16070	5E-28	0.6	Rian	2E-33	0.6
Ccdc68	2E-14	0.6	Gm39214	2E-06	0.6
Thrb	8E-13	0.6	Homer2	9E-09	0.6
Cep76	4E-11	0.6	Tmtc1	2E-23	0.6
Aqp5	9E-14	0.6	Gm26759	5E-09	0.6
Esf1	2E-35	0.6	Snhg12	5E-29	0.6
Acot2	1E-28	0.6	Pwwp3b	8E-11	0.6
Ldlrad3	6E-33	0.6	Lyst	4E-24	0.6
Rian	1E-36	0.6	Fmn1	5E-37	0.6
Ablim1	1E-23	0.6	Farp1	2E-57	0.6
Gm49969	1E-20	0.6	Samd4	5E-58	0.6
Eprs	3E-102	0.6	Aopep	2E-85	0.6
Erg	1E-17	0.6	Cald1	1E-94	0.6
Tchh	7E-10	0.6	9930012K11Rik	9E-06	0.6
Acta2	2E-29	0.6	Prdm15	4E-05	0.6
Robo2	4E-26	0.6	Platr22	3E-09	0.6
Alyref2	1E-07	0.6	Trpv4	2E-09	0.6
Fmn1	4E-46	0.6	Rhobtb3	5E-53	0.6
Samhd1	1E-37	0.6	Rai14	3E-28	0.6
Nexn	6E-19	0.6	H2afy2	6E-28	0.6
Gm15492	7E-08	0.6	Sap30	1E-23	0.6
Prss12	1E-05	0.6	Pycr1	4E-27	0.6
Psph	2E-35	0.6	Hoxa10	2E-40	0.6
Shtn1	1E-06	0.6	Ippk	2E-15	0.6
Adgra3	1E-25	0.6	Mkx	6E-14	0.6
Maf	4E-59	0.6	Gm10974	7E-10	0.6
Pam	6E-93	0.6	Lmo4	8E-45	0.6
Gm35188	8E-26	0.6	Fam107b	1E-25	0.6
Jade1	5E-21	0.6	Daam2	3E-16	0.6
Farp1	1E-51	0.6	Gm34455	8E-10	0.6
Lmo4	4E-60	0.6	Crim1	2E-35	0.6
Pwwp3b	8E-15	0.6	Nedd9	3E-31	0.6
Flrt3	2E-15	0.6	Gpt2	6E-16	0.6
Egfros	3E-13	0.6	Ooep	2E-17	0.6
Shox2	4E-30	0.6	Plcb1	6E-56	0.6
Gm10563	3E-07	0.6	P2ry10b	3E-12	0.6
Cdr2	6E-20	0.6	Aldh18a1	1E-27	0.6
Nr4a2	1E-14	0.6	Palmd	1E-21	0.6
Gchfr	2E-19	0.6	Ostn	4E-16	0.6
Zfp612	7E-12	0.6	Slc9a3r1	3E-20	0.6
B230216N24Rik	2E-10	0.6	Fjx1	2E-14	0.6
4930545L23Rik	5E-10	0.6	Gpr153	3E-11	0.6
Fbn2	1E-36	0.6	Foxp2	1E-33	0.6

Chondrogenic

Control			NICD2		
Gene	p	FC	Gene	p	FC
Per1	7E-17	0.9	Tmem154	5E-09	0.9
Matn4	4E-21	0.9	Hoxa9	5E-73	0.9
Gm10974	3E-08	0.9	B930095G15Rik	3E-08	0.9
Pdgfc	5E-23	0.9	Hoxa10	3E-103	0.9
Rgl1	5E-42	0.9	Cdkn1c	2E-36	0.9
Pde3a	1E-36	0.9	Hecw2	1E-08	0.9
Hoxa10	2E-135	0.9	Atp10a	3E-13	0.9
Plekha7	7E-07	0.9	B230118H07Rik	2E-44	0.9
Pdzrn3	2E-86	0.9	Ric3	2E-11	0.9
Gm49417	3E-10	0.9	Plagl1	3E-52	0.9
4930581F22Rik	7E-15	0.9	Gm47071	1E-13	0.9
Gm19522	3E-22	0.9	Ppm1h	7E-27	0.9
Fancc	4E-46	0.9	Per2	6E-12	0.9
Jph1	3E-06	0.9	Cplx2	2E-03	0.9
Slc12a2	4E-33	0.9	Setbp1	3E-112	0.9
Cst6	1E-75	0.9	Fxyd5	5E-32	0.9
Id3	1E-115	0.9	4933406I18Rik	1E-21	0.9
Phtf2	2E-18	0.9	Abcb9	5E-04	0.9
Hivep2	3E-68	0.9	Sh2d4a	1E-09	0.9
Gm26936	4E-10	0.9	Mef2c	6E-60	0.9
Cmtm8	1E-11	0.9	Nuak1	2E-30	0.9
Satb1	7E-10	0.9	Tfrc	2E-07	0.9
Masp1	2E-23	0.9	Dcaf11	4E-19	0.9
Fgf14	3E-14	0.9	Nedd9	7E-50	0.8
Fgfr1	2E-73	0.9	Gchfr	1E-13	0.8
Fzd8	2E-48	0.9	Phtf2	5E-14	0.8
D330050G23Rik	7E-06	0.9	Crybg1	1E-08	0.8
1700113A16Rik	2E-07	0.9	Stc1	7E-02	0.8
Nedd9	7E-58	0.9	Plcb1	2E-99	0.8
Cemip	9E-10	0.9	Gm32618	2E-13	0.8
Vamp5	3E-66	0.8	Arhgef28	6E-26	0.8
Abcb9	1E-10	0.8	Dusp14	5E-05	0.8
Gm15655	2E-07	0.8	Hoxa11	4E-21	0.8
B230118H07Rik	5E-83	0.8	Epb41l4b	9E-08	0.8
Gm16153	3E-07	0.8	Hotairm1	8E-14	0.8
Tmem8b	8E-10	0.8	Phlda1	8E-19	0.8
Robo1	3E-48	0.8	Per1	1E-08	0.8
Sobp	4E-49	0.8	Mettl7a1	8E-37	0.8
Itm2a	2E-96	0.8	Zbtb7c	7E-16	0.8
Cfap74	7E-11	0.8	Ypel1	5E-09	0.8
Dusp6	8E-14	0.8	D630003M21Rik	2E-09	0.8
Rnf32	2E-11	0.8	Sft2d2	2E-22	0.8
Stc2	7E-14	0.8	Dusp1	4E-25	0.8
Efnb2	3E-29	0.8	Tgfa	4E-08	0.8
Hoxa11	2E-37	0.8	Baiap2l1	2E-33	0.8
Gm15910	1E-05	0.8	Crim1	3E-64	0.8
Capn6	3E-45	0.8	Atp1b3	1E-60	0.8
Tnfaip8	9E-52	0.8	Hoxa5	5E-05	0.8
Zbtb7c	8E-21	0.8	Kirrel3	7E-07	0.8
Mab21l2	4E-61	0.8	Sobp	2E-31	0.8
Chrdl1	3E-20	0.8	Itm2a	1E-91	0.8
Cx3cl1	1E-08	0.8	Dipk2a	7E-26	0.8
Atg16l2	8E-20	0.8	Cdsn	2E-14	0.8
Pde5a	3E-20	0.8	Robo1	2E-55	0.8
Tasp1	2E-43	0.8	Gm47271	6E-09	0.8
Hoxa5	3E-09	0.8	Mtus1	2E-17	0.8
Nfatc1	5E-16	0.8	Fbxo2	5E-23	0.8
Gm31323	4E-06	0.8	Rcan2	8E-37	0.8
Per2	5E-11	0.8	Hmgn5	2E-08	0.8
5031425E22Rik	7E-54	0.8	Slc26a2	4E-06	0.8
Carmil1	2E-36	0.8	4930517O19Rik	3E-10	0.8
Hbegf	2E-28	0.8	Mei4	9E-09	0.8
Pcgf5	2E-19	0.8	Gata2	4E-07	0.8
G0s2	1E-22	0.8	Carmil1	9E-33	0.8
Cnksr3	1E-51	0.8	Gm43126	3E-04	0.8
Paqr3	5E-14	0.8	Slc6a9	2E-09	0.8
Slc16a14	1E-06	0.8	Cfap74	1E-07	0.8
B3galt1	2E-48	0.8	Slc22a14	9E-05	0.8
Pde10a	1E-16	0.8	Igfbp7	1E-19	0.8
Mylk	4E-14	0.8	Dock6	3E-10	0.8
Ippk	4E-08	0.8	Ldhb	3E-39	0.8
Zbed5	8E-07	0.8	Catspere2	1E-07	0.8
Gm47071	1E-20	0.8	Ppp1r3c	2E-09	0.8

Fibroblast

Control			NICD2		
Gene	p	FC	Gene	p	FC
Fgf18	1E-36	0.8	Crlf1	1E-258	0.8
Arhgef9	2E-67	0.8	Eda	2E-39	0.8
Mmp23	0E+00	0.8	Ang	8E-20	0.8
Slc1a5	3E-230	0.8	Cebpd	1E-71	0.8
Mmp2	0E+00	0.8	Lyn	1E-39	0.8
Fst	2E-127	0.8	Gldn	1E-100	0.8
Chd3	5E-258	0.8	Xdh	5E-28	0.8
F730043M19Rik	2E-26	0.8	Prdm6	1E-12	0.8
Fbln1	1E-165	0.8	Ube2l6	5E-12	0.8
Nek6	5E-144	0.8	Fzd4	5E-36	0.8
Lyn	1E-76	0.8	Rbms3	4E-181	0.8
Tln2	6E-220	0.8	Dnaaf3	5E-13	0.8
Mlkl	5E-71	0.8	Nnmt	6E-247	0.8
Tspan11	8E-22	0.8	Adam19	2E-63	0.8
Slc25a21	3E-24	0.8	Atp1b1	7E-29	0.8
Gpr176	5E-65	0.8	Bhlhe41	3E-19	0.8
Dpt	4E-187	0.8	Gpr176	6E-33	0.8
Cacna2d3	1E-20	0.8	Tle2	5E-49	0.8
Ccn4	1E-61	0.8	Fam78b	1E-23	0.8
Lama4	0E+00	0.8	Anpep	2E-35	0.8
Ang	6E-44	0.8	Palm3	3E-13	0.8
Arsi	5E-99	0.8	St5	1E-137	0.8
Dclk1	0E+00	0.8	Gm17501	2E-20	0.8
Vmp1	0E+00	0.8	Marcks	0E+00	0.8
Trim66	1E-26	0.8	Cacna1c	4E-85	0.7
St5	1E-244	0.8	Apobr	6E-18	0.7
Igsf3	2E-58	0.8	Twist2	3E-48	0.7
Spry1	2E-29	0.8	Stard9	1E-32	0.7
Gask1a	2E-21	0.8	Kif26b	7E-43	0.7
Ank3	6E-237	0.8	Palmd	4E-37	0.7
Pknox2	2E-139	0.8	Sik1	4E-16	0.7
Apoe	0E+00	0.8	Psmb8	1E-89	0.7
H2-DMb1	2E-22	0.7	Nav1	7E-49	0.7
Fam126a	9E-90	0.7	Irak3	8E-39	0.7
Chst2	6E-156	0.7	Fhod1	1E-18	0.7
Asap3	5E-104	0.7	Tlcd2	6E-26	0.7
Sik1	6E-30	0.7	Arxes1	9E-13	0.7
Tbc1d16	2E-24	0.7	Mlkl	3E-35	0.7
Scara5	6E-132	0.7	Itsn1	2E-91	0.7
Zfp36l1	1E-256	0.7	Nek6	4E-69	0.7
Anpep	1E-73	0.7	Gm17382	4E-16	0.7
Reps2	2E-72	0.7	Cercam	4E-69	0.7
Tle2	1E-84	0.7	Nrg1	2E-11	0.7
Parp14	1E-47	0.7	Celf2	1E-149	0.7
Slc6a6	5E-137	0.7	Ccn4	5E-34	0.7
Apobec3	4E-50	0.7	Ggt5	2E-49	0.7
Stard9	8E-63	0.7	Entpd6	7E-20	0.7
Ephx2	2E-87	0.7	Arsi	8E-54	0.7
Ube2l6	2E-19	0.7	Asap3	1E-47	0.7
Rbms3	2E-305	0.7	Slc1a5	3E-99	0.7
Mast4	1E-171	0.7	Ociad2	1E-20	0.7
Cox6b2	3E-92	0.7	Tgfbr3	5E-153	0.7
Nav1	4E-97	0.7	Nlgn2	8E-39	0.7
Jcad	2E-129	0.7	Rasa4	8E-26	0.7
Syde2	4E-22	0.7	2700069I18Rik	4E-25	0.7
Jarid2	2E-149	0.7	Lpl	0E+00	0.7
Dnaaf3	9E-21	0.7	Hivep3	3E-34	0.7
Col5a1	0E+00	0.7	Ndrg4	6E-19	0.7
Loxl2	3E-64	0.7	Nhsl1	2E-22	0.7
Prag1	6E-32	0.7	Jarid2	1E-88	0.7
Irak3	2E-85	0.7	Fst	3E-62	0.7
Gm17382	3E-28	0.7	Adh7	3E-27	0.7
3222401L13Rik	3E-44	0.7	Carmn	3E-31	0.7
Tesk2	2E-28	0.7	Garnl3	5E-22	0.7
Adgra2	5E-41	0.7	Ank3	1E-97	0.7
Bdh2	1E-71	0.7	Fam126a	7E-36	0.7
Kctd11	5E-43	0.7	Tmem119	1E-49	0.7
Xdh	2E-48	0.7	Tspan11	5E-08	0.7
Hivep3	6E-55	0.7	Lgals3bp	1E-136	0.7
Vcan	9E-219	0.7	Peg13	3E-12	0.7
Car13	4E-48	0.7	Meox1	7E-25	0.7
Garnl3	3E-41	0.7	Nox4	3E-44	0.7
Epha7	5E-59	0.7	Has2os	7E-32	0.7

Fibroblast?

Control			NICD2		
Gene	p	FC	Gene	p	FC
Selenoh	3E-252	2.3	Gm17092	1E-268	2.2
P2rx7	4E-07	2.3	Spdya	2E-83	2.2
Topbp1	0E+00	2.3	Nfatc2ip	5E-187	2.2
Haus6	1E-252	2.3	D430020J02Rik	1E-183	2.2
9630013D21Rik	5E-117	2.3	Dsn1	1E-245	2.2
Reep4	7E-184	2.3	Hist1h4n	3E-68	2.2
Lonrf3	2E-110	2.3	Eri1	0E+00	2.2
Tagln3	5E-49	2.3	C530008M17Rik	1E-152	2.2
Hyls1	2E-161	2.2	Mcm4	3E-307	2.2
Syk	7E-78	2.2	Skp2	3E-264	2.2
Spdya	6E-90	2.2	Ska2	8E-286	2.1
Suv39h1	1E-212	2.2	Reep4	2E-196	2.1
Skp2	5E-196	2.2	Polh	9E-234	2.1
Serpinb8	2E-94	2.2	Rfc3	0E+00	2.1
Ska2	1E-230	2.2	Zcwpw1	4E-168	2.1
Matn3	8E-50	2.2	Reep1	2E-121	2.1
Hist1h4h	1E-157	2.2	Gm12609	6E-88	2.1
Tmem144	5E-63	2.2	Smc4	0E+00	2.1
Depdc7	8E-96	2.2	Tubg1	3E-283	2.1
Gins1	8E-133	2.2	Tuba1b	0E+00	2.1
Scml2	9E-116	2.2	H1fx	3E-289	2.1
H1fx	3E-207	2.2	Scml2	3E-129	2.1
Vrk1	1E-232	2.2	Haus8	1E-282	2.1
Spon2	3E-42	2.2	Cdca7	4E-157	2.1
Zdhhc2	5E-118	2.1	Zdhhc2	4E-165	2.1
C530008M17Rik	2E-91	2.1	Rfc2	0E+00	2.1
Plxdc1	3E-74	2.1	Hist1h3d	1E-138	2.1
Rcc1	7E-148	2.1	Slbp	3E-302	2.1
Ndst3	1E-48	2.1	Rccd1	1E-189	2.1
Tinagl1	2E-78	2.1	Neto2	4E-158	2.1
Dut	4E-256	2.1	Serpinb8	2E-112	2.1
Dlk2	2E-40	2.1	Rcc1	5E-207	2.1
Mrnip	2E-163	2.1	Hist1h4h	2E-201	2.1
Gm12609	1E-71	2.1	Ddx11	6E-179	2.0
Slbp	6E-216	2.1	Depdc7	1E-88	2.0
Tuba1b	0E+00	2.1	Ccdc34	0E+00	2.0
Mcm4	8E-222	2.1	Ccdc14	2E-152	2.0
Polh	5E-173	2.1	Cnih2	6E-94	2.0
Neto2	5E-148	2.1	Pmaip1	4E-75	2.0
Pole2	2E-185	2.1	Hunk	2E-91	2.0
Eri1	5E-222	2.1	Gpbar1	4E-54	2.0
Gm17092	6E-179	2.1	Mdc1	2E-255	2.0
A330040F15Rik	8E-89	2.1	Cdk2	9E-185	2.0
Cdca7	1E-118	2.1	Dut	0E+00	2.0
Shmt1	4E-97	2.1	Ube2s	0E+00	2.0
Hist1h4c	9E-120	2.1	Pask	1E-208	2.0
Tmpo	2E-280	2.1	Fam122b	9E-117	2.0
Nfatc2ip	3E-141	2.1	Hmgb3	6E-228	2.0
Smc4	3E-297	2.1	Lonrf3	1E-115	2.0
Syne2	2E-201	2.1	Nasp	0E+00	2.0
Ccdc34	5E-263	2.0	Hmgn2	0E+00	2.0
Grip1	1E-31	2.0	Tagln3	7E-54	2.0
Rccd1	1E-137	2.0	Rad21	2E-300	2.0
Lin54	2E-202	2.0	Klhl32	4E-51	2.0
Rad21	1E-214	2.0	Lbr	2E-267	1.9
Fam122b	1E-122	2.0	Dpf1	5E-94	1.9
Cnih2	6E-92	2.0	Eri2	6E-201	1.9
Gm10282	3E-67	2.0	Siah1b	2E-124	1.9
BB557941	5E-49	2.0	Plekha7	4E-114	1.9
Haus8	3E-223	2.0	Suv39h1	6E-226	1.9
Gm26737	3E-86	2.0	Cep192	3E-264	1.9
Rfc3	9E-230	2.0	Gm10282	5E-53	1.9
Rfc2	1E-233	2.0	Grip1	2E-55	1.9
Eri2	6E-176	2.0	Cntrob	1E-174	1.9
D430020J02Rik	6E-122	2.0	Nxt1	3E-260	1.9
Lbr	6E-199	2.0	Hist1h1d	7E-197	1.9
Mab21l3	2E-39	2.0	Cks1b	0E+00	1.9
Ppih	6E-185	2.0	Lsm2	0E+00	1.9
Hmgn2	2E-268	2.0	A330040F15Rik	1E-105	1.9
Cks1b	1E-307	2.0	Relt	5E-47	1.9
Mdc1	6E-193	2.0	Rpa1	9E-247	1.9
Zcwpw1	2E-109	2.0	Gm29585	8E-28	1.9
Tubg1	7E-186	1.9	Khdrbs3	1E-239	1.9

Undefined

Control			NICD2		
Gene	p	FC	Gene	p	FC
Aste1	9E-01	0.3	Ggnbp1	2E-01	0.3
Plxnc1	4E-03	0.3	Clec2d	2E-09	0.3
Tprg	3E-04	0.3	Lrfn4	1E-01	0.3
Gm49179	1E+00	0.3	Dusp6	3E-01	0.3
Errfi1	1E-05	0.3	Decr2	1E-01	0.3
Itga6	1E-01	0.3	Gm41609	6E-02	0.3
Ddit3	2E-10	0.3	Apol9a	1E-01	0.3
Ly96	3E-07	0.3	Npr3	1E-01	0.3
Morn4	2E-01	0.3	Mllt1	1E-02	0.3
Snhg16	8E-02	0.3	Mon1a	6E-02	0.3
Sco2	9E-04	0.3	Il1rapl1	1E-17	0.3
Ptprf	2E-04	0.3	Alkbh6	5E-01	0.3
Egfr	3E-20	0.3	Ahnak2	3E-15	0.3
Paqr3	1E-03	0.3	Hoxa9	2E-10	0.3
Txlnb	1E-01	0.3	Lncppara	8E-07	0.3
Casp2	3E-01	0.3	Tspan9	6E-05	0.3
Eif4e3	5E-01	0.3	Plpp2	4E-03	0.3
1810059H22Rik	2E-01	0.3	Coasy	1E-01	0.3
Srgap3	2E-24	0.3	Hspb2	2E-01	0.3
Btbd11	3E-03	0.3	Cox6a2	4E-01	0.3
Adpgk	2E-01	0.3	Samd12	2E-01	0.3
D11Wsu47e	7E-01	0.3	Ttc26	2E-01	0.3
Selenbp1	3E-06	0.3	Plod1	1E-08	0.3
Pafah2	8E-03	0.3	Fign	2E-07	0.3
Hmgn3	3E-31	0.3	Plbd2	2E-11	0.3
Proser1	2E-01	0.3	Rnaset2b	5E-02	0.3
Zyx	1E-04	0.3	Stxbp5	9E-04	0.3
Rarg	6E-08	0.3	Dhx34	2E-01	0.3
Macrod1	6E-13	0.3	Cln3	3E-01	0.3
Zfp810	5E-01	0.3	Marcksl1	4E-07	0.3
Hmox1	1E-04	0.3	Bbs2	7E-01	0.3
Vps37d	1E-01	0.3	Abhd17c	2E-01	0.3
Gas1	5E-19	0.3	B4galt1	6E-06	0.3
Jam2	1E-04	0.3	Phyhd1	4E-09	0.3
Dock11	9E-02	0.3	Gabarapl1	2E-09	0.3
Gpd2	2E-03	0.3	1110059G10Rik	2E-06	0.3
Mboat2	2E-02	0.3	Zpr1	7E-01	0.3
C1ql3	2E-01	0.3	Zfp52	6E-01	0.3
Gm8739	4E-03	0.3	Fbxl12os	8E-01	0.3
Ltbp1	1E-14	0.3	Snx15	7E-06	0.3
Nmnat3	5E-04	0.3	P4ha1	3E-48	0.3
Sat1	1E-17	0.3	Mertk	9E-02	0.3
Dnah7b	1E-03	0.3	Cp	1E-04	0.3
Gm42418	6E-16	0.3	Brf2	5E-01	0.3
Crem	3E-02	0.3	Zfp68	4E-03	0.3
B4galt1	4E-09	0.3	Ifit3b	8E-01	0.3
2610306M01Rik	1E-02	0.3	Grem1	6E-02	0.3
Kif9	5E-01	0.3	Hmgn3	1E-27	0.3
Ccdc30	9E-02	0.3	1700123M08Rik	9E-02	0.3
Phip	2E-13	0.3	C1rl	1E-02	0.3
Agtr2	3E-02	0.3	Gstp1	3E-03	0.3
Nmrk1	9E-03	0.3	Smad3	3E-08	0.3
Hoxa11os	2E-03	0.3	Hyal1	2E-01	0.3
Il1rapl1	2E-20	0.3	Cyp2u1	2E-01	0.3
Hddc3	3E-01	0.3	Pld1	8E-04	0.3
Slc4a7	3E-06	0.3	Imp3	9E-16	0.3
Cdkn1c	1E-13	0.3	Eif2b2	2E-02	0.3
Stambpl1	7E-07	0.3	Hspa12b	6E-01	0.3
Gm10561	2E-03	0.3	6330562C20Rik	2E-01	0.3
P4ha1	7E-54	0.3	Neat1	1E-19	0.3
Tpd52l1	7E-02	0.3	Adpgk	1E+00	0.3
Mad2l2	5E-02	0.3	Mxra8	9E-33	0.3
Dhrs3	4E-05	0.3	Arl6ip5	6E-22	0.3
Fam13c	3E-10	0.3	Rragb	1E-01	0.3
Fam171a2	2E-02	0.3	Maf	3E-15	0.3
Plpp2	6E-03	0.3	Snx2	9E-12	0.3
Pdk2	9E-03	0.3	Grcc10	5E-43	0.3
Gm11266	1E-01	0.3	Pafah2	1E-01	0.3
Bach2	3E-01	0.3	Cgnl1	7E-06	0.3
AY036118	4E-04	0.3	Slc25a27	4E-02	0.3
Eral1	4E-01	0.3	Snx29	4E-02	0.3
Calhm6	6E-02	0.3	Mest	2E-14	0.3
Neu1	1E-04	0.3	Zfp386	1E-01	0.3

Articular/Synovial Fibroblast

Control			NICD2		
Gene	p	FC	Gene	p	FC
Apcdd1	3E-02	0.8	Nudt4	3E-46	0.8
Kcnab1	3E-01	0.8	Sirpa	7E-07	0.8
Jam2	9E-12	0.8	Klhl8	5E-02	0.8
Pcgf1	8E-04	0.8	Gstt2	3E-03	0.8
Dbi	6E-92	0.8	Nnmt	2E-35	0.8
B4galt1	1E-31	0.8	Sh3pxd2b	5E-09	0.8
Ntrk3	4E-15	0.8	Arrb1	9E-02	0.8
Fbxo32	8E-04	0.8	Ramp1	1E-04	0.8
Gm16201	3E-07	0.8	Col18a1	4E-25	0.8
Hsd3b7	4E-06	0.8	Atg7	1E-05	0.8
Gstm1	3E-67	0.8	2310001H17Rik	2E-03	0.7
Traf3ip2	6E-07	0.8	Dclre1c	3E-01	0.7
Colec12	6E-69	0.8	Hopx	3E-08	0.7
Rab29	1E-11	0.8	Larp6	2E-03	0.7
Hspb6	1E-21	0.8	F830016B08Rik	6E-02	0.7
Peli2	1E-21	0.8	Il17ra	4E-04	0.7
Aldh3b1	1E-09	0.8	Ppp1r9a	2E-03	0.7
Tgfbi	5E-13	0.8	Slfn8	6E-05	0.7
Kng1	3E-12	0.8	0610043K17Rik	6E-05	0.7
Lrrc27	2E-03	0.8	Gucy1b1	1E-01	0.7
Fbxo31	3E-08	0.8	Calcrl	3E-02	0.7
Rac3	1E-16	0.8	Arsg	1E-01	0.7
Coq8a	6E-03	0.8	Slc35d2	2E-02	0.7
Enpp2	1E-04	0.8	Gm10125	4E-03	0.7
Sord	2E-08	0.8	Prrg3	1E-03	0.7
Klhl8	1E-02	0.8	Sgsm2	4E-04	0.7
Dapk1	9E-30	0.8	Ttc38	4E-02	0.7
Rgcc	1E-01	0.7	Lgmn	2E-18	0.7
Fxyd1	3E-55	0.7	Atp6v1b2	2E-13	0.7
Ccl7	1E-06	0.7	Gbp2	2E-04	0.7
B2m	1E-99	0.7	Tmem26	6E-05	0.7
Limch1	3E-07	0.7	Dpp7	2E-12	0.7
Cdh13	2E-09	0.7	Dusp10	2E-02	0.7
Anks1b	4E-01	0.7	Mctp2	3E-03	0.7
Serac1	2E-02	0.7	Guca1a	7E-02	0.7
Jarid2	2E-24	0.7	Aebp1	4E-87	0.7
Tgfbr3	3E-52	0.7	Wdr91	1E-01	0.7
Ckb	1E-14	0.7	Eno2	1E-02	0.7
Mrgprf	4E-06	0.7	Hdac11	6E-03	0.7
Cyp2u1	1E-03	0.7	Klf4	3E-11	0.7
Gbp7	6E-07	0.7	Cog7	1E-04	0.7
Ctsb	5E-85	0.7	Kng2	1E-04	0.7
Il1rl2	7E-02	0.7	Abhd14b	1E-05	0.7
Gm12610	5E-03	0.7	Laptm4b	7E-05	0.7
Aldh1l1	5E-07	0.7	Gm12216	7E-03	0.7
Col4a1	9E-20	0.7	Nav1	4E-15	0.7
Pde8a	2E-16	0.7	Parp14	1E-02	0.7
Lbp	1E-13	0.7	Pir	1E-06	0.7
Nptxr	4E-02	0.7	Hdac5	3E-06	0.7
Tle2	2E-10	0.7	Thrb	2E-03	0.7
Arvcf	1E-02	0.7	Khk	5E-06	0.7
Pask	4E-02	0.7	Irgm2	3E-04	0.7
Car11	1E-04	0.7	Phka2	1E-03	0.7
Tgfa	5E-05	0.7	Gstt3	3E-06	0.7
Dgat2	7E-15	0.7	Unc13b	2E-02	0.7
Plcl2	4E-02	0.7	Adra1b	1E-03	0.7
Laptm4b	6E-07	0.7	Ednra	2E-14	0.7
Ndrg4	5E-05	0.7	Cpeb3	4E-06	0.7
Bin1	2E-30	0.7	Pdpn	3E-24	0.7
Mafb	3E-12	0.7	Cmya5	4E-04	0.7
Cxcl1	1E-02	0.7	Zfp395	1E-07	0.7
Rab3il1	3E-08	0.7	Cdc42ep2	3E-05	0.7
Pdxk	1E-04	0.7	Anks6	7E-02	0.7
Cyp27a1	8E-03	0.7	Ly96	7E-13	0.7
Adam33	2E-15	0.7	Ikbke	2E-03	0.7
Sema4b	6E-02	0.7	Ldb2	4E-16	0.7
Tpcn2	5E-03	0.7	Dbi	8E-62	0.7
H2-D1	5E-60	0.7	Fam117a	5E-02	0.7
Olfml3	5E-29	0.7	Fam171a1	2E-05	0.7
Zcchc24	4E-29	0.7	Pycard	3E-11	0.7
Pbx1	5E-44	0.7	Nfkbiz	1E-05	0.7
Aebp1	6E-108	0.7	Gm16638	6E-02	0.7
Adamts4	1E-01	0.7	Cdo1	6E-11	0.7

Limb Mesenchyme

Control Gene	p	FC	NICD2 Gene	p	FC
B230217O12Rik	6E-12	0.6	Col1a1	4E-86	0.6
Slc4a4	9E-10	0.6	Col9a3	8E-26	0.6
Cfap74	2E-14	0.6	Cdh11	3E-50	0.6
Npas3	4E-46	0.6	Frk	3E-12	0.6
Rasal2	3E-19	0.6	Gm10658	3E-06	0.6
Phf10	6E-46	0.6	Mpp6	1E-29	0.6
Fli1	3E-17	0.6	Aff3	6E-50	0.6
Hoxa10	3E-51	0.6	Slc4a7	2E-27	0.6
Tpm1	4E-69	0.6	Igdcc4	8E-19	0.6
Gm26827	5E-18	0.6	Rin1	8E-11	0.6
Ptprk	1E-19	0.6	Nupr1	6E-50	0.6
Nid2	6E-27	0.6	Mtss2	7E-27	0.6
Kctd14	3E-09	0.6	Xpot	1E-34	0.6
Stc2	3E-11	0.6	Col1a2	9E-101	0.6
Ippk	2E-17	0.6	Limch1	2E-25	0.6
Cacna1d	3E-14	0.6	2810457G06Rik	9E-09	0.6
Tead4	7E-07	0.6	Ajuba	1E-15	0.6
Pak1	1E-30	0.6	Gars	7E-56	0.6
Polr3e	8E-16	0.6	Pygo1	8E-23	0.6
Satb2	6E-12	0.6	Adra1b	1E-07	0.6
Slc4a7	4E-34	0.6	Dync1i1	3E-06	0.6
Klhdc10	3E-26	0.6	Slc25a33	1E-11	0.6
Ism1	2E-25	0.6	Fnbp1l	1E-28	0.6
Id3	9E-52	0.6	Rras2	5E-18	0.6
Gm11146	7E-11	0.6	Msi2	1E-48	0.6
Rcc2	2E-36	0.6	4931428F04Rik	2E-07	0.6
Col8a1	4E-29	0.6	Dchs1	4E-11	0.6
Tbx3	3E-08	0.6	Srf	6E-06	0.6
Trmt61b	4E-20	0.6	BC024978	8E-08	0.6
P2ry10b	3E-15	0.6	Pkd2l2	5E-08	0.6
Gm13919	3E-14	0.6	Eprs	2E-74	0.6
Ntn4	2E-13	0.6	Acot2	5E-23	0.6
Slc38a1	4E-29	0.6	Ivns1abp	1E-24	0.6
4930533K18Rik	3E-07	0.6	Npas3	6E-33	0.6
1700023F06Rik	1E-07	0.6	1190005I06Rik	3E-12	0.6
Mthfr	1E-15	0.6	Far1os	1E-09	0.6
Tgfb2	1E-60	0.6	Actn1	3E-50	0.6
Zfp579	1E-16	0.6	Esf1	2E-26	0.6
Palmd	5E-23	0.6	Plk2	6E-23	0.6
Lctl	4E-09	0.6	Lonp1	2E-19	0.5
Lrrc4	4E-26	0.6	Plscr2	3E-10	0.5
Pck2	1E-41	0.6	Inpp4b	1E-05	0.5
Cxadr	1E-07	0.6	Prep	2E-24	0.5
Mmp28	2E-12	0.6	Lrrc4	1E-17	0.5
Ppfibp1	2E-48	0.6	Myo10	1E-35	0.5
Sap30	3E-29	0.6	Hmgcll1	3E-11	0.5
Foxp4	5E-17	0.6	Ptprk	7E-19	0.5
Slit2	1E-04	0.6	Alcam	1E-15	0.5
Znrf3	1E-17	0.6	Map2	3E-13	0.5
Rin1	2E-10	0.6	Gm28153	4E-15	0.5
Gpr176	1E-13	0.6	Masp1	9E-16	0.5
Psat1	3E-34	0.6	Gm14858	1E-05	0.5
Gm3235	2E-06	0.6	Gm13778	4E-06	0.5
Alas2	4E-07	0.6	Cd109	9E-30	0.5
Foxc1	6E-46	0.6	Asns	7E-42	0.5
Asxl1	2E-36	0.6	Ptprf	1E-18	0.5
Pygo1	7E-24	0.6	Grb10	3E-51	0.5
Phyhd1	3E-37	0.6	Gsdme	2E-19	0.5
Pak3	1E-36	0.6	Mmp28	8E-10	0.5
Ankrd50	2E-15	0.6	Kank1	1E-17	0.5
Vezt	5E-25	0.6	Meg3	2E-42	0.5
Asns	2E-51	0.6	Alas2	1E-04	0.5
Aff3	2E-58	0.6	Fhl2	5E-14	0.5
5730522E02Rik	2E-10	0.5	Bop1	2E-11	0.5
Pde7b	7E-19	0.5	Osgin2	8E-15	0.5
Ivns1abp	1E-23	0.5	Mettl24	3E-06	0.5
Rcor2	3E-10	0.5	Myh9	3E-59	0.5
Fancc	3E-23	0.5	Fat4	3E-32	0.5
Wars2	1E-19	0.5	Gm28379	3E-06	0.5
Kif7	6E-08	0.5	Cdc42se2	2E-21	0.5
Ajuba	5E-17	0.5	Hapln1	2E-35	0.5
Efcab1	3E-09	0.5	Cdr2	7E-17	0.5
Gulp1	2E-62	0.5	Lars	8E-49	0.5

Chondrogenic

Control Gene	p	FC	NICD2 Gene	p	FC
Plagl1	5E-60	0.8	Chchd10	4E-12	0.8
Rtl3	5E-21	0.8	A930001C03Rik	2E-02	0.8
Wscd2	2E-05	0.8	Mest	8E-53	0.8
Lpar4	5E-45	0.8	Ncoa3	2E-30	0.8
Hotairm1	7E-17	0.8	Phyh	3E-30	0.8
Zbtb20	9E-171	0.8	Carns1	2E-05	0.8
Rassf3	9E-12	0.8	Srpx2	5E-84	0.8
Dync1i1	5E-16	0.8	Ak4	2E-07	0.8
4933406I18Rik	3E-23	0.8	Dlgap1	7E-05	0.8
Gm49959	1E-12	0.8	Tmem62	4E-06	0.8
Ostn	5E-04	0.8	Arid5a	3E-05	0.8
Adgra3	2E-33	0.8	Slc38a1	2E-35	0.8
Prelp	2E-40	0.8	Cep126	7E-06	0.8
Setbp1	1E-121	0.8	Tgfb2	1E-79	0.8
Fap	2E-17	0.8	Nsun4	6E-09	0.8
Mef2c	1E-70	0.8	Gm11146	4E-08	0.8
Tsc22d1	9E-104	0.8	Col27a1	1E-08	0.8
2500002B13Rik	8E-10	0.8	Synpo	3E-23	0.8
Slc6a9	4E-14	0.8	Rab3b	1E-08	0.8
Jade1	6E-21	0.8	Stbd1	8E-11	0.8
Aox1	3E-18	0.8	Zbtb46	9E-05	0.8
Skap2	7E-60	0.8	Adgra3	8E-29	0.8
Gba2	1E-05	0.8	Pdcd4	1E-94	0.8
4933431E20Rik	1E-09	0.8	Col8a2	1E-06	0.8
Tcte2	3E-08	0.8	0610043K17Rik	5E-11	0.8
St3gal4	2E-35	0.8	Cpq	5E-130	0.8
Adam22	4E-12	0.8	Vill	7E-09	0.8
Tmbim1	6E-43	0.8	Acot1	8E-06	0.7
Fam160a1	1E-05	0.8	Gm44686	4E-14	0.7
Sh2d4a	9E-13	0.7	Angptl6	4E-10	0.7
Gm16152	1E-08	0.7	Gprc5a	4E-05	0.7
Mkx	3E-27	0.7	a	4E-06	0.7
Tspyl4	2E-30	0.7	Mettl26	7E-29	0.7
Gm34455	2E-05	0.7	Pim3	8E-12	0.7
Pde3b	6E-23	0.7	Tspyl4	2E-22	0.7
Plcb1	7E-94	0.7	Rarg	2E-38	0.7
Gm26759	6E-07	0.7	4933431E20Rik	2E-06	0.7
Zfp612	9E-09	0.7	Apol7d	2E-05	0.7
Igfbp7	8E-27	0.7	Slc7a2	1E-31	0.7
A230072C01Rik	2E-04	0.7	Me3	2E-15	0.7
Arhgef28	3E-25	0.7	Malt1	5E-04	0.7
Ccpg1os	5E-14	0.7	Cnksr3	2E-27	0.7
Phlda1	5E-22	0.7	Ninl	3E-12	0.7
2700054A10Rik	4E-05	0.7	Kif13b	1E-37	0.7
Slc38a1	1E-37	0.7	Zbtb20	3E-117	0.7
4930444A19Rik	8E-06	0.7	Flrt1	3E-09	0.7
Penk	2E-21	0.7	Efnb2	5E-12	0.7
Pdcd4	3E-108	0.7	Jade1	6E-15	0.7
Miga1	7E-08	0.7	Id3	7E-66	0.7
Ppm1h	1E-26	0.7	Gm35188	2E-15	0.7
Cep126	2E-07	0.7	Mpped2	3E-07	0.7
Edil3	2E-92	0.7	Gm15563	6E-07	0.7
Mettl26	3E-37	0.7	Skap2	5E-40	0.7
Myo1b	4E-15	0.7	4930550C14Rik	5E-06	0.7
Usp6nl	5E-14	0.7	C1rl	6E-11	0.7
Tmem117	1E-06	0.7	Med12l	1E-03	0.7
Ica1	3E-18	0.7	Abca2	1E-03	0.7
Dach2	5E-05	0.7	Pdzrn3	8E-50	0.7
P4ha1	4E-161	0.7	Psd3	7E-35	0.7
Fam131a	5E-09	0.7	Fgfr1	2E-34	0.7
Clec11a	9E-50	0.7	Dcn	3E-80	0.7
Nuak2	2E-03	0.7	Gm26759	2E-03	0.7
Dusp1	7E-29	0.7	Hist1h2ac	5E-08	0.7
Tenm4	6E-47	0.7	Lrch1	2E-17	0.7
Angptl6	3E-12	0.7	Fxyd6	3E-07	0.7
Fgfrl1	5E-26	0.7	Lpar4	1E-33	0.7
Lrch1	6E-25	0.7	4930556M19Rik	7E-06	0.7
Nrk	3E-38	0.7	Miga1	1E-07	0.7
Srpx2	6E-93	0.7	Slc12a2	6E-23	0.7
Setd7	9E-42	0.7	Shisa4	9E-38	0.7
Pdlim4	7E-34	0.7	Pla2g12a	1E-20	0.7
Gm47350	8E-05	0.7	Pde5a	2E-13	0.7
Ankrd28	8E-48	0.7	Grb14	7E-18	0.7

Fibroblast

Control Gene	p	FC	NICD2 Gene	p	FC
Tlcd2	9E-44	0.7	Col5a1	7E-237	0.7
Entpd6	2E-38	0.7	Tbc1d16	3E-12	0.7
Ephb3	9E-53	0.7	Guca1a	3E-13	0.7
Cercam	3E-120	0.7	Plppr2	3E-20	0.7
Dennd4a	3E-115	0.7	Hmcn1	1E-85	0.7
Ggt5	5E-96	0.7	Adamtsl3	9E-79	0.7
Mmp11	2E-40	0.7	Dennd4a	1E-56	0.7
Mical2	5E-20	0.7	Parp14	3E-27	0.7
Col4a1	2E-174	0.7	Dclk1	1E-152	0.7
Phex	3E-17	0.7	Mgat3	4E-10	0.7
Carmn	3E-58	0.7	Col4a1	5E-93	0.7
6330403K07Rik	4E-66	0.7	Cachd1	6E-63	0.7
Has2os	5E-50	0.7	Mmp11	1E-22	0.7
Pmepa1	2E-276	0.7	Eef1akmt3	6E-17	0.7
Rasa3	6E-107	0.7	Col16a1	4E-74	0.7
Fam43a	1E-51	0.7	Mdk	5E-94	0.7
8430436N08Rik	5E-20	0.7	Ghr	2E-144	0.7
2700069I18Rik	1E-51	0.7	Gm43507	4E-11	0.7
Il17rd	2E-26	0.7	Jcad	7E-72	0.7
Jag1	2E-83	0.7	Syde2	7E-10	0.6
Nlgn2	1E-63	0.7	Bdh2	2E-31	0.6
Cdc42ep2	5E-90	0.7	Apobec3	1E-21	0.6
F2r	7E-52	0.7	Il4ra	2E-09	0.6
Smarca1	5E-79	0.7	Arap1	5E-15	0.6
Il34	3E-34	0.7	A230057D06Rik	7E-46	0.6
Rasa4	2E-48	0.6	Kctd11	6E-22	0.6
Klf7	5E-109	0.6	Dpt	2E-84	0.6
Tgfbr3	8E-277	0.6	Gm765	2E-04	0.6
Exoc3l4	4E-23	0.6	Bace2	6E-15	0.6
Lgals3bp	1E-238	0.6	Galnt13	2E-33	0.6
Nat8f1	3E-38	0.6	Arxes2	1E-25	0.6
Ifi27l2a	3E-87	0.6	Tspan18	3E-22	0.6
Cbx6	4E-103	0.6	Gm15345	1E-12	0.6
Ano3	1E-36	0.6	Il17rd	3E-09	0.6
Hmcn1	2E-177	0.6	Hccs	3E-20	0.6
Fam78b	1E-33	0.6	Lama4	1E-126	0.6
B3galnt1	7E-61	0.6	2010204K13Rik	6E-12	0.6
Apobr	6E-28	0.6	Reps2	4E-28	0.6
Gm17501	5E-30	0.6	Cacna1g	6E-21	0.6
Cachd1	2E-115	0.6	Cxcl16	6E-31	0.6
Prps2	7E-43	0.6	Il3ra	8E-42	0.6
Zfp992	7E-29	0.6	Casp4	4E-19	0.6
Arap1	3E-36	0.6	Ephb3	3E-24	0.6
Atxn1	0E+00	0.6	Purg	6E-33	0.6
Veph1	9E-23	0.6	Acss3	2E-17	0.6
Fzd5	7E-38	0.6	Fam43a	1E-18	0.6
Bhlhe41	6E-25	0.6	Chst2	8E-63	0.6
Gm43507	6E-18	0.6	Cox6b2	3E-44	0.6
Plppr2	5E-32	0.6	Mast4	1E-71	0.6
Gm15345	1E-26	0.6	Cdc42ep2	5E-46	0.6
Ndrg4	9E-31	0.6	3222401L13Rik	7E-22	0.6
Mdk	6E-177	0.6	Lrrc8e	7E-08	0.6
Cacna1b	9E-11	0.6	Socs5	2E-37	0.6
Efnb1	9E-53	0.6	Lgi2	1E-21	0.6
Cdc42ep5	3E-213	0.6	Zfp36l1	4E-103	0.6
Arhgap42	1E-138	0.6	Fignl2	6E-07	0.6
Pear1	8E-22	0.6	Cd46	5E-11	0.6
Slc1a6	2E-67	0.6	Nod1	2E-12	0.6
Dact3	9E-57	0.6	Hykk	4E-14	0.6
Purg	3E-66	0.6	Ptgr1	6E-27	0.6
Tnfaip2	8E-128	0.6	Prag1	2E-15	0.6
Eef1akmt3	6E-31	0.6	Shfl	3E-20	0.6
Rnf144a	3E-58	0.6	Trnp1	3E-35	0.6
Rap2a	2E-141	0.6	Rap2a	5E-62	0.6
2010204K13Rik	5E-25	0.6	6330403K07Rik	2E-32	0.6
Socs5	9E-66	0.6	Cpa6	2E-16	0.6
Col16a1	2E-133	0.6	Arsj	6E-49	0.6
Hebp2	4E-79	0.6	Adgra2	2E-17	0.6
Pros1	1E-176	0.6	Cxcl5	6E-88	0.6
Pced1b	1E-36	0.6	Thbs2	1E-141	0.6
Bace2	3E-27	0.6	Bmt2	1E-48	0.6
Chrna1os	3E-24	0.6	Dact3	4E-27	0.6
Dhcr24	4E-55	0.6	Prps2	2E-23	0.6

Fibroblast?

Control Gene	p	FC	NICD2 Gene	p	FC
Tedc2	6E-100	1.9	Mutyh	3E-61	1.9
Ccdc14	2E-97	1.9	Hist1h2bg	1E-74	1.9
Ube2s	2E-278	1.9	Dctpp1	0E+00	1.9
Cep295	2E-187	1.9	Mtbp	2E-195	1.9
Chek2	2E-158	1.9	Rims1	6E-42	1.9
Nasp	2E-215	1.9	Casp7	6E-148	1.9
Gm13415	2E-35	1.9	Jpt2	2E-230	1.9
Slc35g1	1E-55	1.9	Nup43	2E-219	1.9
Jpt1	8E-212	1.9	Anxa8	4E-107	1.9
Prr5l	2E-42	1.9	4933404O12Rik	1E-115	1.9
Cbx2	1E-42	1.9	Klhl23	2E-165	1.8
Cep192	7E-196	1.9	Pold3	3E-228	1.8
Hist1h2bg	1E-84	1.9	Tedc2	9E-105	1.8
Chst1	3E-76	1.9	Prr5l	2E-95	1.8
Lsm2	5E-289	1.9	Etaa1	1E-227	1.8
Styk1	8E-73	1.9	Mrnip	2E-150	1.8
Agbl2	1E-73	1.9	Shmt1	1E-120	1.8
Arl6ip1	6E-126	1.9	Pradc1	1E-215	1.8
Prrg4	8E-57	1.9	Cbx2	8E-54	1.8
Hmgb3	2E-138	1.9	Nanos1	7E-98	1.8
Haus3	1E-186	1.9	Tbc1d31	3E-201	1.8
Pold3	9E-184	1.9	Haus3	5E-223	1.8
Frmd4b	1E-61	1.8	Fxyd6	4E-201	1.8
4933404O12Rik	2E-82	1.8	Gemin6	1E-222	1.8
Morrbid	8E-140	1.8	Sdk1	8E-192	1.8
Etaa1	3E-163	1.8	Ccdc15	2E-160	1.8
Mtbp	1E-149	1.8	Hist1h1e	3E-164	1.8
Rpa1	9E-188	1.8	Rbmx2	1E-188	1.8
Cyp39a1	4E-110	1.8	Fancg	3E-73	1.8
Gm16576	2E-31	1.8	Jpt1	2E-269	1.8
Katnal2	5E-96	1.8	Nmral1	3E-122	1.8
Casp7	1E-110	1.8	Fam110a	6E-120	1.8
Tdrkh	2E-52	1.8	Ppih	5E-247	1.8
Hist1h3d	6E-100	1.8	Tnfsf9	2E-69	1.8
AC160336.1	1E-31	1.8	Cenpj	9E-157	1.8
Dctpp1	2E-205	1.8	Tinf2	2E-162	1.8
Enkd1	5E-119	1.8	Arl6ip1	2E-142	1.7
Kcnj15	1E-32	1.8	Hist1h2be	2E-123	1.7
Cntrob	4E-122	1.8	Dek	0E+00	1.7
Pbx4	2E-79	1.8	Ccp110	3E-153	1.7
Plekha7	5E-70	1.8	Cep295	5E-235	1.7
Khdrbs3	2E-148	1.8	Tjp2	5E-86	1.7
Gpr137c	2E-60	1.8	Orc6	1E-258	1.7
Cdk2	2E-102	1.8	Raet1e	4E-156	1.7
Terf1	7E-158	1.8	Nup107	2E-196	1.7
Rbmx2	4E-139	1.8	Cep57l1	9E-208	1.7
Reep1	5E-91	1.8	Nphp4	2E-53	1.7
Nxt1	9E-172	1.8	Terf1	3E-209	1.7
Nemp1	2E-153	1.8	Haus1	5E-218	1.7
Ccp110	1E-152	1.8	E2f1	4E-191	1.7
Tjp2	2E-62	1.7	Orc1	5E-105	1.7
Jpt2	2E-170	1.7	Hpf1	7E-296	1.7
Tinf2	2E-123	1.7	Chek2	1E-137	1.7
Ccdc15	8E-110	1.7	Gm12610	4E-103	1.7
Cdh24	5E-51	1.7	Tipin	2E-307	1.7
Nav2	3E-157	1.7	Styk1	2E-62	1.7
Hist1h1e	8E-110	1.7	Lrrc8b	1E-94	1.7
Gemin6	1E-145	1.7	Cyp39a1	7E-133	1.7
Rttn	9E-135	1.7	Rell1	4E-177	1.7
Fosl1	1E-92	1.7	Cmc2	6E-283	1.7
Rtel1	4E-103	1.7	Slf1	3E-221	1.7
Rell1	4E-131	1.7	Nup155	1E-198	1.7
Slf1	2E-147	1.7	Gpr137c	2E-100	1.7
Gprc5a	5E-57	1.7	Katnal2	5E-96	1.7
Perp	3E-35	1.7	Gm9949	7E-43	1.6
Miip	3E-71	1.7	Endod1	7E-144	1.6
Tnfsf9	5E-65	1.7	Rassf7	1E-70	1.6
Nup107	2E-128	1.7	Frmd4b	3E-49	1.6
Dek	1E-275	1.7	Rfwd3	4E-197	1.6
Tipin	1E-218	1.7	Zfp382	9E-102	1.6
Slc9a5	5E-49	1.7	Msh6	3E-143	1.6
Klhl23	2E-107	1.7	6720489N17Rik	3E-74	1.6
Tent5b	1E-34	1.7	Rnf157	7E-113	1.6

Undefined

Control Gene	p	FC	NICD2 Gene	p	FC
Rbpj	4E-19	0.3	Vim	3E-37	0.3
Antxr2	2E-05	0.3	Zfp945	1E-01	0.3
Wdr47	1E+00	0.3	Camk1d	5E-03	0.3
Ppm1h	6E-05	0.3	Gss	6E-03	0.3
Sgcd	7E-23	0.3	Tctn2	2E-01	0.3
Gm13056	5E-03	0.3	5930430L01Rik	2E-03	0.3
Aktip	2E-02	0.3	Mtss2	5E-09	0.3
Sft2d2	2E-03	0.3	Mex3a	3E-01	0.3
Samd12	1E-02	0.3	Traf6	4E-01	0.3
Plcd1	2E-03	0.3	Upf3b	5E-04	0.3
Gm26724	5E-04	0.3	Rufy1	7E-05	0.3
Pde10a	4E-05	0.3	Colec12	1E-16	0.3
Kcnrg	3E-02	0.3	Tmem26	5E-02	0.3
Tm2d2	1E-25	0.3	Tbpl1	2E-04	0.3
Eml2	2E-02	0.3	Myc	1E-01	0.3
Grcc10	2E-41	0.3	Polr1e	8E-02	0.3
Mbnl3	4E-02	0.3	Adamts1	2E-01	0.3
Fndc3a	7E-26	0.3	Tgif1	6E-03	0.3
Midn	1E-03	0.3	Ehd3	1E-01	0.3
Mterf4	1E-03	0.3	Kcnrg	6E-02	0.3
Smpdl3b	5E-01	0.3	Ino80dos	1E-01	0.3
Itih5	4E-05	0.3	Tm2d2	4E-20	0.3
Gpm6b	9E-30	0.3	Kazald1	1E-05	0.3
Apol9a	5E-02	0.3	Itpr3	1E-01	0.3
Igfbp2	3E-01	0.3	Nr6a1os	3E-01	0.3
Mest	1E-17	0.3	Zfp874b	5E-01	0.3
Tmem220	2E-01	0.3	Nfia	3E-21	0.3
Klhdc8a	8E-02	0.3	Ppm1h	5E-05	0.3
Myo1b	7E-03	0.3	Ccdc189	1E+00	0.3
Malat1	1E-84	0.3	Hoxa13	2E-01	0.3
Espn	5E-01	0.3	Hoxa7	1E-01	0.3
Twist1	4E-17	0.3	Inpp5k	3E-01	0.3
Trmt12	4E-01	0.3	Rpp38	3E-01	0.2
Ndufaf3	4E-04	0.3	Ccdc173	4E-02	0.2
Efna5	7E-14	0.3	Gm43713	9E-01	0.2
Cep131	1E-01	0.3	Anxa4	3E-13	0.2
Tpk1	2E-03	0.3	AW209491	5E-01	0.2
Abcc4	2E-02	0.3	Vcpkmt	6E-01	0.2
Pqlc1	7E-02	0.3	Ccdc39	4E-01	0.2
Efnb2	2E-03	0.3	Slc38a1	1E-06	0.2
Gsta4	2E-02	0.3	Mettl22	9E-01	0.2
Egfros	6E-03	0.3	Narf	8E-02	0.2
Sgk3	6E-03	0.3	Ikbke	9E-02	0.2
Dusp6	3E-02	0.3	Mir22hg	3E-07	0.2
Lrrn3	4E-02	0.3	Hist3h2a	9E-01	0.2
Lrrc49	6E-03	0.3	Zbtb5	3E-01	0.2
Mtmr11	2E-01	0.3	Acot11	1E-01	0.2
Fam151b	1E-01	0.3	Mef2b	1E+00	0.2
Pex11g	5E-02	0.3	9630014M24Rik	3E-01	0.2
Stxbp5	4E-07	0.3	Tbx5	1E-01	0.2
Zfp516	1E-07	0.2	Abi3bp	4E-34	0.2
Fbxo44	2E-01	0.2	Usp20	1E-01	0.2
Abi3bp	4E-32	0.2	Nfatc1	5E-01	0.2
Gfra4	3E-01	0.2	Coq3	3E-02	0.2
Plbd2	1E-13	0.2	Abhd6	1E+00	0.2
Idh2	8E-17	0.2	Micall1	9E-01	0.2
Kcne4	4E-02	0.2	Dus2	8E-01	0.2
Rpusd4	3E-01	0.2	Amdhd2	4E-02	0.2
Disp1	5E-03	0.2	Gas8	2E-01	0.2
Foxp1	5E-19	0.2	Slc22a5	4E-01	0.2
Klhl24	7E-07	0.2	Htra3	8E-07	0.2
Pcdhb17	3E-01	0.2	Wdr73	9E-01	0.2
Ermp1	3E-01	0.2	Dyrk1b	4E-01	0.2
Rnd3	5E-10	0.2	Tmem151a	6E-01	0.2
Tmem140	5E-02	0.2	Ppm1k	7E-01	0.2
N6amt1	2E-05	0.2	Pcid2	4E-01	0.2
Atxn1l	9E-01	0.2	Kdm4b	3E-01	0.2
Arl6ip5	1E-21	0.2	Egfr	3E-19	0.2
Nicn1	4E-02	0.2	Wbp1	5E-03	0.2
Mettl25	2E-01	0.2	Slc18b1	9E-02	0.2
Slc35c2	6E-03	0.2	Phip	4E-11	0.2
Arl4c	1E-01	0.2	Ggact	1E+00	0.2
Trps1	4E-34	0.2	Gm34455	5E-01	0.2

Articular/Synovial Fibroblast

Control Gene	p	FC	NICD2 Gene	p	FC
Kng2	2E-08	0.7	Ifit3b	1E-05	0.7
Dnm1	5E-25	0.7	Tcp11l2	2E-05	0.7
Gas1	2E-65	0.7	Fbxo44	1E-01	0.7
Dock9	3E-07	0.7	Adam32	3E-01	0.7
Syne2	1E-05	0.7	Arhgap6	2E-17	0.7
Fam171a1	1E-05	0.7	Rab36	7E-02	0.7
0610043K17Rik	1E-07	0.7	Smad3	1E-10	0.7
Deptor	4E-06	0.7	Zfp41	5E-01	0.7
Arhgap31	3E-11	0.7	Dpt	1E-21	0.7
Chl1	3E-02	0.7	Gramd1c	3E-01	0.7
Grtp1	5E-02	0.7	Rasl11a	7E-04	0.7
Vat1	2E-36	0.7	Vat1	6E-17	0.7
Zfp395	5E-08	0.7	C1ra	7E-26	0.7
Rap1gap2	5E-02	0.7	Enpp5	9E-06	0.7
Ror1	4E-18	0.7	Ctns	9E-04	0.7
Tbc1d2b	2E-14	0.7	Smyd3	5E-12	0.7
Fgf14	2E-03	0.7	Gm15787	2E-01	0.7
B3galnt1	3E-10	0.7	Mfsd12	2E-02	0.7
Mmp2	7E-30	0.7	Syne2	5E-05	0.7
Matn2	3E-10	0.7	Zfp976	3E-02	0.7
Glis3	3E-44	0.7	Abtb1	3E-01	0.7
Sh3bp5	9E-05	0.7	Cant1	1E-04	0.7
Plcd3	3E-03	0.7	Kng1	1E-05	0.7
Tppp3	5E-03	0.7	Arsj	4E-06	0.7
Gfpt2	3E-02	0.7	Spsb2	1E-10	0.7
Negr1	1E-07	0.7	Ror1	8E-14	0.7
Dhdh	4E-02	0.7	Dnah7b	2E-04	0.7
Sytl2	7E-05	0.7	Hspb6	1E-09	0.7
Gldn	3E-10	0.7	Gm41609	9E-02	0.7
Mmp14	7E-35	0.7	Scara3	1E-06	0.7
Vegfb	1E-32	0.7	Jak1	7E-49	0.7
Angptl2	1E-11	0.7	Dnase2a	2E-03	0.7
Vsir	3E-02	0.7	Il13ra1	4E-04	0.7
Scn1b	4E-28	0.7	Gm16759	1E-03	0.7
Gm16759	1E-05	0.7	Prdx4	2E-34	0.7
Epdr1	7E-18	0.7	Neu1	3E-05	0.7
Ifih1	3E-04	0.7	Npc2	1E-74	0.7
Snx29	7E-09	0.7	Itm2b	2E-68	0.7
Gstm7	4E-02	0.7	Dhtkd1	3E-01	0.7
Gm16263	1E-02	0.7	Pbx1	3E-28	0.7
Atp6v1b2	3E-15	0.7	Dcxr	7E-11	0.7
Plce1	9E-18	0.7	Jam2	7E-07	0.7
Ly96	3E-15	0.7	Magi2	7E-31	0.7
Tpp1	2E-43	0.7	Tprkb	7E-11	0.7
Dpyd	6E-04	0.7	Dffb	2E-01	0.7
Epb41l3	2E-24	0.7	Airn	5E-18	0.7
Tspan11	2E-01	0.7	Bin1	1E-15	0.7
Gm12963	5E-02	0.7	Tnfaip2	4E-16	0.7
Rin2	4E-27	0.7	2310022A10Rik	1E-01	0.7
Socs3	4E-04	0.7	Arl15	7E-21	0.7
Lrrc32	5E-12	0.7	2900093K20Rik	5E-02	0.7
Ptgr1	3E-08	0.7	Gm15261	3E-06	0.7
Lipa	8E-26	0.7	Vgll4	6E-22	0.7
Gng2	5E-05	0.7	Usp18	7E-02	0.7
Jak1	9E-56	0.7	Flcn	2E-02	0.6
Htra3	2E-13	0.7	Clu	1E-27	0.6
Ifit1	6E-08	0.7	Ugdh	2E-15	0.6
Gm49066	6E-02	0.7	Olfml3	4E-21	0.6
Fam102b	2E-16	0.7	Trpm3	1E-02	0.6
Mctp2	3E-03	0.7	Podxl2	2E-10	0.6
A230057D06Rik	1E-10	0.7	Tbc1d2b	2E-10	0.6
Clu	5E-40	0.7	Rnf150	1E-15	0.6
Retreg1	7E-06	0.7	Mr1	2E-02	0.6
Arhgef10l	6E-09	0.7	Tom1	8E-04	0.6
Gm10373	2E-02	0.7	Epb41l3	7E-13	0.6
Acaa2	2E-28	0.7	Slc26a11	2E-01	0.6
1110046J04Rik	2E-07	0.7	Mtmr4	8E-01	0.6
Lrrc17	8E-22	0.7	Zfp937	1E-01	0.6
Zfp385a	2E-05	0.7	Mettl7a1	3E-12	0.6
Vcan	2E-15	0.7	Scrn1	5E-05	0.6
Tshz3	1E-04	0.7	Gnpda1	1E-04	0.6
Parp14	7E-05	0.7	Sh3bp5	6E-04	0.6
1810034E14Rik	8E-02	0.7	Stat5a	4E-03	0.6

Limb Mesenchyme

Control Gene	p	FC	NICD2 Gene	p	FC
Myl9	5E-14	0.5	Cemip2	5E-13	0.5
Isg20	4E-14	0.5	Nop2	5E-11	0.5
Sipa1l1	5E-35	0.5	Col24a1	1E-06	0.5
Dpysl3	3E-21	0.5	Gm43948	1E-08	0.5
Rab39b	7E-12	0.5	Cd24a	1E-11	0.5
Kcne4	3E-14	0.5	Tspyl4	6E-17	0.5
Slc25a47	2E-07	0.5	Prdm11	5E-08	0.5
Mknk1	1E-15	0.5	Hyou1	4E-25	0.5
Sema3e	3E-18	0.5	Stk4	7E-16	0.5
Edar	1E-05	0.5	Shox2	3E-27	0.5
Atp10a	3E-18	0.5	Ccdc80	2E-68	0.5
Lin7a	2E-07	0.5	Fancc	5E-20	0.5
Msi2	2E-52	0.5	2900026A02Rik	7E-10	0.5
Gm48383	1E-10	0.5	A930005H10Rik	7E-12	0.5
Eml6	4E-13	0.5	Fkbp10	1E-34	0.5
Itgb8	5E-17	0.5	Sim2	9E-06	0.5
Stk17b	8E-13	0.5	Gab1	2E-26	0.5
Cog6	2E-20	0.5	Ell2	1E-38	0.5
Adra1b	7E-13	0.5	Yrdc	1E-22	0.5
Lrrc8c	5E-17	0.5	Plk3	3E-06	0.5
Shb	1E-06	0.5	Zfhx3	5E-41	0.5
Zfhx3	4E-40	0.5	Yod1	3E-07	0.5
Lonp1	2E-22	0.5	1700023F06Rik	5E-06	0.5
Klc2	1E-08	0.5	Veph1	2E-06	0.5
Lars	3E-58	0.5	Spryd3	3E-15	0.5
9930012K11Rik	1E-06	0.5	Fzd3	2E-10	0.5
Grb10	3E-61	0.5	Syn3	2E-35	0.5
Lpar4	7E-24	0.5	Dse	3E-39	0.5
Uhrf1bp1	2E-11	0.5	Msi1	8E-06	0.5
Lig4	3E-08	0.5	Cdc42ep3	3E-22	0.5
Zfp622	9E-32	0.5	Plekhh2	5E-34	0.5
2810457G06Rik	4E-08	0.5	Abhd6	3E-08	0.5
Id1	5E-30	0.5	Cacna1c	3E-15	0.5
Tspyl4	6E-21	0.5	Btbd19	3E-07	0.5
Fam227b	7E-07	0.5	Acot1	4E-05	0.5
C330011M18Rik	7E-06	0.5	A130010J15Rik	9E-09	0.5
2900076A07Rik	3E-15	0.5	Lrrc8c	2E-13	0.5
Zfp469	8E-14	0.5	Mxd1	7E-05	0.5
Eef1akmt3	3E-11	0.5	Rcc2	1E-26	0.5
Cnst	1E-11	0.5	Herpud1	4E-34	0.5
Nrbf2	4E-27	0.5	Mpped2	1E-14	0.5
C79798	1E-07	0.5	Ppfibp1	1E-34	0.5
Hmga2	1E-60	0.5	Mettl7a1	9E-20	0.5
Gfod2	6E-09	0.5	Pank1	2E-09	0.5
Eif3c	3E-93	0.5	Cavin4	2E-11	0.5
Tent5a	1E-30	0.5	Gm26672	3E-07	0.5
Abhd17c	6E-14	0.5	Gm17066	4E-06	0.5
Hoxaas3	2E-05	0.5	Gm16070	6E-13	0.5
B3gat2	8E-05	0.5	Me3	9E-17	0.5
Aopep	1E-74	0.5	Pard3bos1	7E-10	0.5
Garem1	4E-14	0.5	Aff2	2E-15	0.5
Cdc42se2	1E-21	0.5	Spata2	1E-07	0.5
Sec16b	9E-17	0.5	Arhgap5	5E-44	0.5
Akna	5E-05	0.5	Gm26749	7E-13	0.5
Rgl1	9E-25	0.5	Ptpn14	6E-26	0.5
Myh9	3E-60	0.5	Prelp	7E-21	0.5
Meg3	2E-48	0.5	Sdc2	1E-53	0.5
Yrdc	2E-19	0.5	Itga3	1E-06	0.5
Odc1	1E-30	0.5	2500004C02Rik	3E-05	0.5
Slc39a11	6E-18	0.5	Amotl2	4E-31	0.5
Hspa9	2E-61	0.5	Slc35d1	2E-09	0.5
Dse	2E-38	0.5	Lats2	2E-22	0.5
Fig4	4E-17	0.5	Znrf3	5E-17	0.5
Gnasas1	2E-13	0.5	Nrk	2E-15	0.5
Enox1	3E-16	0.5	Iffo2	2E-16	0.5
Plod2	2E-39	0.5	Ptprg	2E-24	0.5
Jazf1	8E-12	0.5	Phlpp1	9E-21	0.5
Atf4	3E-46	0.5	Pwp2	5E-06	0.5
Prep	7E-19	0.5	Erg	4E-12	0.5
Yod1	1E-09	0.5	Ehbp1	8E-32	0.5
Xist	1E-14	0.5	Tcf4	1E-72	0.5
Tmem62	5E-10	0.5	Amotl1	7E-24	0.5
Actg1	4E-41	0.5	Gm5089	5E-08	0.5

Chondrogenic

Control Gene	p	FC	NICD2 Gene	p	FC
Cdsn	9E-14	0.7	Pde4dip	1E-23	0.7
Ntng1	3E-05	0.7	Dgke	1E-04	0.7
Kirrel3	9E-19	0.7	Gm4258	8E-16	0.7
Atp1b3	3E-64	0.7	Zbtb39	4E-04	0.7
Tpd52l1	9E-11	0.7	Aldh6a1	6E-21	0.7
Osbpl6	1E-15	0.7	Pak3	1E-25	0.7
Otor	4E-03	0.7	Phlpp1	3E-26	0.7
Megf9	4E-10	0.7	Slc37a4	2E-06	0.7
Tmem154	7E-07	0.7	Vps37b	2E-05	0.7
A830012C17Rik	4E-09	0.7	Pitx1	4E-68	0.7
Morn2	2E-32	0.7	Hist1h1c	1E-35	0.7
Smo	3E-48	0.7	Clec11a	8E-32	0.7
Cdc42ep3	2E-57	0.7	Gm12655	7E-06	0.7
Rarg	6E-40	0.7	Dach2	2E-03	0.7
Acot11	4E-07	0.7	Epb41	5E-17	0.7
Zc3hav1l	1E-06	0.7	Gm42726	8E-05	0.7
Lrrk1	6E-48	0.7	Vamp5	1E-30	0.7
Frmd6	2E-43	0.7	Usp6nl	1E-09	0.7
Mbnl3	1E-08	0.7	Gm26827	1E-06	0.7
Mettl7a1	5E-37	0.7	Svip	2E-03	0.7
Acaa1b	2E-05	0.7	Setd7	3E-30	0.7
Grb14	6E-26	0.7	Smo	2E-40	0.7
Ninl	1E-13	0.7	Gng11	1E-11	0.7
Pla2g12a	5E-26	0.7	Myo1b	3E-14	0.7
Itga3	2E-08	0.7	Maf	6E-44	0.7
Sh3rf1	6E-19	0.7	Ccdc80	6E-113	0.7
Papss1	1E-23	0.7	Slc29a1	2E-26	0.7
Usp53	3E-11	0.7	Itih2	5E-18	0.7
Ccn1	5E-59	0.7	Rora	1E-69	0.7
Mtus1	2E-19	0.7	Fgfrl1	3E-14	0.7
B230369F24Rik	1E-03	0.7	Znrf3	1E-18	0.7
Abca2	1E-06	0.7	4930581F22Rik	7E-05	0.7
Tmem62	2E-06	0.7	Zswim4	3E-05	0.7
Gchfr	1E-17	0.7	Gsap	2E-02	0.7
Plekhh2	7E-41	0.7	Ank	7E-31	0.7
Stbd1	1E-12	0.7	Ckb	6E-11	0.7
Rnaset2b	6E-15	0.7	Nrk	5E-25	0.7
Arid5a	2E-05	0.7	Farp1	2E-41	0.7
Tbx15	2E-86	0.7	Syt12	4E-06	0.7
Cpq	1E-128	0.7	Ets2	7E-18	0.7
Gm12592	1E-08	0.7	Scx	4E-14	0.6
Stk38l	3E-25	0.7	Chrdl1	8E-10	0.6
Atoh8	6E-09	0.7	Enpp1	2E-05	0.6
Slc35d1	7E-11	0.7	Kantr	5E-11	0.6
Farp1	6E-48	0.7	Paqr3	2E-08	0.6
Hyal1	6E-05	0.7	Evi2a	2E-03	0.6
Tinagl1	8E-10	0.7	Ccpg1os	7E-08	0.6
Gm47271	2E-11	0.7	Tmod1	3E-03	0.6
Ikzf4	3E-09	0.7	Cbr2	2E-25	0.6
Sft2d2	6E-21	0.7	Fzd3	8E-06	0.6
Trps1	3E-117	0.6	Vit	2E-04	0.6
Shisa4	1E-43	0.6	Il18	1E-12	0.6
Slc45a4	5E-10	0.6	Adam22	2E-04	0.6
Dcaf11	4E-18	0.6	Retreg1	2E-12	0.6
Csmd1	2E-24	0.6	Stk38l	4E-17	0.6
Hs3st3a1	9E-07	0.6	Gm9929	3E-03	0.6
Bicd1	1E-06	0.6	Tasp1	1E-22	0.6
Tns3	9E-52	0.6	Dhx58os	1E-03	0.6
Dock6	1E-06	0.6	2700054A10Rik	2E-02	0.6
Lrba	1E-49	0.6	Gm19522	1E-07	0.6
Trib2	1E-14	0.6	Tom1l2	6E-29	0.6
Gm35188	1E-24	0.6	Tsc22d1	4E-50	0.6
Zfp462	2E-21	0.6	Nacc2	1E-16	0.6
Arl4a	3E-36	0.6	Rsph9	8E-11	0.6
Ptprd	1E-55	0.6	Lncppara	8E-38	0.6
Pld1	4E-17	0.6	Atp2b1	6E-49	0.6
Adamtsl3	2E-29	0.6	Afap1l2	4E-04	0.6
Il18	1E-12	0.6	Gm34455	9E-04	0.6
Epb41	5E-19	0.6	P4ha1	1E-103	0.6
Ets2	3E-18	0.6	Lrrk1	9E-35	0.6
Raver2	6E-04	0.6	Hoxa11os	5E-08	0.6
Abcc4	3E-09	0.6	Dancr	2E-05	0.6
Zbtb18	1E-05	0.6	Bcr	8E-11	0.6

Fibroblast

Control Gene	p	FC	NICD2 Gene	p	FC
Amot	2E-37	0.6	Hebp2	1E-36	0.6
Lpl	0E+00	0.6	Cdc42ep5	9E-105	0.6
Bmper	4E-09	0.6	Efemp2	1E-266	0.6
Cacna1g	9E-37	0.6	Ackr3	1E-23	0.6
Gm20033	2E-11	0.6	Bicc1	2E-180	0.6
Zfp982	2E-21	0.6	Tnfaip2	2E-64	0.6
Jak1	0E+00	0.6	Col4a2	6E-71	0.6
Cxcl5	2E-203	0.6	Serpinf1	3E-217	0.6
Porcn	6E-29	0.6	Ildr2	3E-22	0.6
Serpinf1	0E+00	0.6	Loxl2	3E-24	0.6
Ociad2	2E-36	0.6	Sgpp1	4E-40	0.6
Csf1	2E-245	0.6	Atxn1	8E-151	0.6
Mpp1	2E-88	0.6	Pear1	5E-12	0.6
Galnt18	4E-45	0.6	Rasa3	6E-48	0.6
Fam102b	4E-93	0.6	Pros1	2E-91	0.6
Trnp1	1E-72	0.6	Vegfc	4E-17	0.6
Cpa6	2E-30	0.6	Mmp19	1E-38	0.6
5033430I15Rik	2E-16	0.6	Ano3	2E-15	0.6
Vegfc	2E-38	0.6	Smim1	4E-88	0.6
Mgat3	1E-14	0.6	Csf1	2E-115	0.6
Hsd17b7	4E-46	0.6	Il34	7E-15	0.6
Nr2f2	9E-91	0.6	Fgf2	3E-20	0.6
Col4a2	2E-120	0.6	Gm4221	1E-12	0.6
Cxcl16	2E-54	0.6	Smarca1	2E-34	0.6
Gsc	3E-63	0.6	Efnb1	1E-24	0.6
4933424G05Rik	2E-12	0.6	Lrp4	3E-15	0.6
Casp4	5E-30	0.6	St3gal5	7E-25	0.6
Peak1	2E-167	0.6	Pmepa1	6E-131	0.6
Bicc1	0E+00	0.6	Btbd3	1E-38	0.6
Arsj	4E-83	0.6	Mical2	1E-08	0.6
Ackr3	4E-49	0.6	Capn1	5E-28	0.6
Arxes2	2E-47	0.6	Jak1	3E-207	0.6
Bmt2	7E-89	0.6	Cbx6	1E-46	0.6
Efemp2	0E+00	0.6	Pced1b	1E-17	0.6
Zfhx4	8E-194	0.6	Rab7b	1E-19	0.6
Tmem119	4E-69	0.6	Col27a1	4E-59	0.6
Guca1a	1E-19	0.6	Chrna1os	1E-10	0.6
St3gal5	2E-57	0.6	Il13ra1	7E-31	0.6
Ccl2	4E-70	0.6	Col8a2	2E-12	0.6
Nod1	8E-22	0.6	4933412E12Rik	3E-05	0.6
Il3ra	1E-83	0.6	B3galnt1	3E-23	0.6
Hspb1	8E-115	0.6	Cacnb3	2E-12	0.6
Ghr	2E-252	0.6	Bnc2	9E-62	0.6
Grebl1	3E-15	0.6	Gsc	3E-29	0.6
Gbp2	1E-41	0.6	Npas2	1E-09	0.6
Picalm	2E-160	0.6	Vcan	2E-80	0.6
Mfhas1	1E-56	0.6	Bgn	0E+00	0.6
C2	2E-18	0.6	Zfhx4	6E-90	0.6
Mgll	1E-71	0.6	Tesk2	1E-07	0.6
A230057D06Rik	3E-77	0.6	Adamts5	2E-44	0.6
Fhl2	2E-107	0.6	Fndc10	2E-08	0.6
Il13ra1	5E-61	0.6	B4galt5	2E-17	0.6
Hhat	7E-23	0.6	Dhcr24	6E-22	0.6
Psd4	7E-17	0.6	Timp1	4E-138	0.6
Ifitm3	0E+00	0.6	Kcne4	6E-13	0.6
B4galt5	2E-41	0.6	Ier5l	1E-48	0.6
Capn1	4E-63	0.6	Slc22a17	2E-15	0.6
Arrdc4	2E-48	0.6	Msx1	2E-09	0.6
Lgalsl	2E-47	0.6	Nr2f2	2E-48	0.5
Sgpp1	2E-73	0.6	Grasp	9E-16	0.5
Meox1	3E-45	0.6	Zfp984	4E-13	0.5
Abcg2	3E-62	0.6	Ifitm3	2E-223	0.5
Zfp984	2E-29	0.6	Peak1	1E-69	0.5
Wfdc17	7E-92	0.6	Exoc3l4	2E-08	0.5
Bgn	0E+00	0.6	Abhd14b	2E-24	0.5
Ier5l	2E-108	0.6	Klf7	1E-42	0.5
Gem	9E-74	0.6	Mfhas1	6E-25	0.5
Mmp19	3E-65	0.6	Fam102b	5E-42	0.5
Inka1	2E-48	0.6	Mark1	9E-20	0.5
Peg13	6E-17	0.6	Zfp982	2E-09	0.5
Sulf2	5E-117	0.6	Adam23	2E-33	0.5
Svil	7E-207	0.6	Mgst3	1E-73	0.5
Lamb1	7E-139	0.6	Abcg2	9E-31	0.5

Fibroblast?

Control Gene	p	FC	NICD2 Gene	p	FC
Dpf1	4E-64	1.7	Mis12	7E-199	1.6
Mutyh	6E-37	1.7	Rttn	9E-143	1.6
Nqo1	6E-134	1.7	Tiam1	2E-105	1.6
Gm11837	1E-48	1.7	Ttf2	2E-153	1.6
Fam221a	5E-60	1.7	Mamstr	4E-92	1.6
Nanos1	1E-59	1.7	Slc35g1	1E-38	1.6
Med18	1E-74	1.7	Nemp1	3E-161	1.6
Msh6	1E-120	1.7	Gm13889	1E-77	1.6
Hist1h2be	1E-117	1.7	Gm16576	1E-46	1.6
Ttf2	6E-123	1.7	Pfkfb1	4E-58	1.6
Fancm	1E-125	1.7	Slc9a5	2E-54	1.6
9430015G10Rik	1E-104	1.7	Med18	9E-95	1.6
Fhdc1	6E-46	1.7	Foxd2os	2E-147	1.6
Itprip	2E-32	1.7	Anapc15	1E-196	1.6
Nup43	4E-123	1.7	Tmem144	8E-42	1.6
Gm36569	2E-25	1.6	Gm12811	1E-91	1.6
Slurp1	5E-47	1.6	Lrrc3	2E-34	1.6
Fxyd6	1E-127	1.6	Rtel1	6E-134	1.6
Ddx39	1E-123	1.6	Zfp101	2E-128	1.6
Sdk1	3E-129	1.6	Umps	1E-168	1.6
Tbc1d31	1E-134	1.6	Dtymk	0E+00	1.6
Anxa8	1E-99	1.6	9430015G10Rik	2E-130	1.6
Cep57l1	2E-147	1.6	Rnd1	5E-71	1.6
Haus1	2E-154	1.6	Cdca4	2E-150	1.6
Nde1	1E-115	1.6	Nemp2	1E-63	1.6
Foxd2os	4E-119	1.6	Nup85	3E-170	1.6
Zfp382	8E-65	1.6	Tdrkh	2E-44	1.6
Hhip	3E-36	1.6	Tent5b	7E-42	1.6
Orc6	5E-175	1.6	Ncam1	2E-138	1.6
Anapc15	7E-161	1.6	Gm26737	3E-88	1.6
Siah1b	7E-80	1.6	Pold2	3E-129	1.6
Hmga1	3E-64	1.6	Hcn2	2E-57	1.6
Gm49980	2E-48	1.6	Gm10827	3E-46	1.5
Kcnk5	1E-39	1.6	Itpripl1	2E-62	1.5
Raet1e	1E-102	1.6	Tmem121	5E-138	1.5
Pask	3E-128	1.6	Aif1l	2E-34	1.5
Hpf1	3E-189	1.6	Sacs	1E-116	1.5
Nup155	2E-129	1.6	Cep76	1E-110	1.5
Ncam1	6E-117	1.6	Ppil1	6E-157	1.5
B020010K11Rik	4E-61	1.6	Agbl2	1E-53	1.5
Elovl7	5E-43	1.6	9530077C05Rik	7E-79	1.5
Cmc2	7E-197	1.6	Pbx4	4E-62	1.5
Fgfr1op	5E-77	1.6	Perp	1E-28	1.5
Hist1h1d	6E-122	1.6	Piga	2E-53	1.5
Lsm11	6E-78	1.6	Mdm1	1E-86	1.5
Nup85	5E-138	1.6	Nup160	1E-162	1.5
Tubb5	4E-303	1.6	Cep112	2E-152	1.5
Orc1	2E-95	1.6	Exosc8	2E-222	1.5
Mdm1	5E-61	1.6	Pms2	6E-145	1.5
Rprm	2E-36	1.6	Rasgrp3	5E-23	1.5
Pradc1	1E-117	1.6	Ywhah	2E-302	1.5
Nup160	2E-133	1.6	Atp10a	3E-137	1.5
Umps	2E-127	1.6	Cenpo	6E-98	1.5
Fzr1	4E-92	1.6	Fam221a	8E-54	1.5
E2f1	5E-121	1.6	Cdc25a	1E-129	1.5
6720489N17Rik	4E-67	1.6	Map2	2E-159	1.5
Ssx2ip	1E-110	1.6	Psip1	0E+00	1.5
Rnd1	8E-44	1.6	Fancm	8E-138	1.5
Nes	3E-145	1.6	Ssx2ip	2E-121	1.5
Aaas	1E-132	1.6	Enkd1	1E-118	1.5
Rfwd3	6E-132	1.5	Unc13c	3E-44	1.5
Exosc8	1E-178	1.5	Nqo1	1E-138	1.5
Spink2	3E-53	1.5	Ripk3	6E-138	1.5
Dtymk	8E-265	1.5	AC160336.1	1E-41	1.5
Endod1	1E-92	1.5	Tfdp1	5E-217	1.5
Nmral1	2E-78	1.5	Aaas	7E-150	1.5
Gm11290	3E-12	1.5	Nptxr	9E-105	1.5
Tiam1	1E-69	1.5	Aard	2E-34	1.5
Hcn2	1E-39	1.5	Cdk5rap2	2E-205	1.5
Gm9949	6E-40	1.5	Heyl	6E-41	1.5
Ripk3	1E-96	1.5	Tubb5	0E+00	1.5
Rad51b	2E-168	1.5	B020010K11Rik	1E-68	1.5
Rnf157	5E-68	1.5	Gjc1	2E-164	1.5

Undefined

Control Gene	p	FC	NICD2 Gene	p	FC
E130308A19Rik	1E-01	0.2	Fam214b	4E-01	0.2
Cep126	3E-01	0.2	Vps72	4E-03	0.2
Ahnak2	1E-15	0.2	D830024N08Rik	4E-01	0.2
Myo1d	4E-25	0.2	Crip2	5E-14	0.2
Phex	2E-01	0.2	Pitpnc1	3E-02	0.2
Cacnb1	4E-01	0.2	Gfra4	4E-01	0.2
Fam53b	1E-02	0.2	Slc22a18	9E-01	0.2
Fign	5E-07	0.2	Fbxo31	6E-01	0.2
Plekhb2	8E-02	0.2	Snhg17	6E-01	0.2
Vasn	8E-11	0.2	Nck2	2E-02	0.2
Ccdc9	7E-01	0.2	Morn2	8E-04	0.2
Foxc1	1E-18	0.2	Rgs3	5E-02	0.2
Tnfrsf12a	5E-04	0.2	Stoml1	9E-01	0.2
Tmtc1	3E-02	0.2	Clip2	1E-01	0.2
Pigb	1E-01	0.2	Itih5	1E-02	0.2
Irgq	4E-01	0.2	Gm26887	9E-02	0.2
Tubg2	2E-01	0.2	Dnal4	6E-02	0.2
Cyren	7E-01	0.2	Setbp1	6E-25	0.2
Tcp11l2	2E-02	0.2	Olfml2a	8E-05	0.2
Hspb2	6E-02	0.2	Malat1	2E-56	0.2
4930550C14Rik	3E-01	0.2	AY036118	3E-03	0.2
C030006K11Rik	7E-01	0.2	E130102H24Rik	5E-02	0.2
Slc11a2	3E-01	0.2	Aldh3b1	5E-02	0.2
Narf	8E-03	0.2	Slc4a7	1E-04	0.2
Zfp945	1E-02	0.2	Trmt10b	2E-01	0.2
Rnf113a1	5E-01	0.2	Spcs3	1E-05	0.2
Zbtb24	1E-01	0.2	Trp53cor1	7E-02	0.2
Fam214b	7E-03	0.2	Ecm2	3E-03	0.2
Ap5z1	5E-01	0.2	Rnmt	2E-03	0.2
Fbxw17	4E-07	0.2	Fhl3	3E-01	0.2
Vcpkmt	9E-02	0.2	C230035I16Rik	3E-01	0.2
Papolg	6E-02	0.2	Mia	9E-22	0.2
Aox1	8E-03	0.2	4930549G23Rik	1E+00	0.2
Arhgap28	2E-05	0.2	Itm2c	3E-15	0.2
Pltp	4E-11	0.2	Ddit3	3E-04	0.2
Klhl36	8E-01	0.2	Sirt4	3E-01	0.2
Cp	3E-03	0.2	Nat10	6E-01	0.2
Lrfn4	6E-01	0.2	Aldoa	2E-39	0.2
Lgmn	3E-12	0.2	Fbxw9	3E-01	0.2
Gm16759	8E-01	0.2	Plekhg5	9E-03	0.2
Gm10658	7E-01	0.2	Angptl1	2E-03	0.2
Slc37a4	8E-01	0.2	Pex6	3E-02	0.2
Pak3	5E-08	0.2	Thnsl2	1E+00	0.2
Gypc	8E-02	0.2	1110008P14Rik	3E-24	0.2
Med20	6E-01	0.2	Ctsb	1E-63	0.2
Hopx	2E-03	0.2	P3h3	2E-16	0.2
Gm11508	1E-01	0.2	Adamtsl4	2E-03	0.2
Inpp5k	1E-01	0.2	Elp6	4E-01	0.2
F420014N23Rik	9E-01	0.2	Cox20	5E-12	0.2
B3gnt9	2E-04	0.2	Pdk1	9E-02	0.2
Lrba	5E-11	0.2	Ino80b	2E-01	0.2
Ptpn1	5E-09	0.2	Gm11266	7E-01	0.2
3110009E18Rik	9E-01	0.2	Rap1gap2	1E-01	0.2
Fam229b	6E-02	0.2	Dnah7b	9E-02	0.2
Ankrd28	2E-09	0.2	Oplah	8E-01	0.2
Rtl8b	1E-03	0.2	Ccdc114	9E-01	0.2
Fbxo31	5E-02	0.2	Hyal2	3E-02	0.2
Shisa2	6E-01	0.2	Sgk3	3E-01	0.2
2200002D01Rik	9E-03	0.2	Tmem53	9E-02	0.2
Gm16845	3E-01	0.2	Lancl1	4E-04	0.2
Vgll4	9E-09	0.2	Cdon	4E-09	0.2
Wrap53	1E-01	0.2	Tmed4	1E-06	0.2
Tnfsf13	6E-01	0.2	Me3	3E-04	0.2
Syne1	8E-17	0.2	Ppcs	3E-01	0.2
Tmub1	6E-02	0.2	Hdac3	3E-02	0.2
Yipf2	7E-01	0.2	Tmub2	9E-03	0.2
Snx21	5E-03	0.2	Tmem98	3E-03	0.2
Dvl1	3E-01	0.2	Mboat2	2E-02	0.2
Tmeff1	1E-01	0.2	Ifi44	8E-04	0.2
Pim3	3E-03	0.2	Cpne8	3E-16	0.2
Vipas39	2E-02	0.2	Sgms2	1E-01	0.2
Zfp622	2E-06	0.2	Kdm5b	4E-14	0.2
Fmnl3	3E-01	0.2	Sema3b	6E-01	0.2

Articular/Synovial Fibroblast

Control Gene	p	FC	NICD2 Gene	p	FC
Gstt3	1E-06	0.7	Zfp820	1E-02	0.6
Nr3c2	5E-05	0.7	Gca	3E-01	0.6
Rtl8a	6E-15	0.7	Ifi30	2E-17	0.6
Cst3	3E-78	0.7	Traf3ip2	4E-03	0.6
Mcub	5E-09	0.7	C1s1	4E-13	0.6
Ezh1	2E-08	0.7	Hddc3	9E-02	0.6
Kif21a	4E-18	0.7	Col4a1	1E-19	0.6
Ccdc189	8E-02	0.6	Tap1	2E-02	0.6
Arsg	6E-02	0.6	Mxi1	4E-04	0.6
Casp4	7E-03	0.6	3110009E18Rik	9E-02	0.6
Man1a	4E-11	0.6	Irf7	6E-04	0.6
Dhx58os	3E-05	0.6	H2-T22	2E-07	0.6
Cmtm3	8E-20	0.6	Fbxl8	3E-01	0.6
Capn5	5E-02	0.6	Gdpd1	1E-01	0.6
Galnt2	3E-16	0.6	Plin3	6E-15	0.6
Map3k8	2E-02	0.6	Mterf2	1E-02	0.6
Gm12216	5E-02	0.6	Arhgap26	6E-03	0.6
Tmem106a	5E-04	0.6	Lhfpl2	3E-11	0.6
Rasa4	2E-05	0.6	Ddit4l	3E-20	0.6
Sec22c	4E-01	0.6	Epdr1	4E-11	0.6
Prdx4	2E-52	0.6	Rab29	1E-03	0.6
Ndrg1	3E-09	0.6	C230035I16Rik	4E-01	0.6
Ecm1	1E-38	0.6	Acox3	5E-02	0.6
Fgf10	2E-06	0.6	Klf9	2E-09	0.6
Neat1	5E-31	0.6	Mgst1	9E-41	0.6
Mettl7a1	3E-14	0.6	Inpp5e	1E-01	0.6
Lgmn	2E-19	0.6	Cdc14a	5E-02	0.6
Kat2b	2E-13	0.6	Efemp2	3E-56	0.6
Ppp1r9a	8E-04	0.6	Aldh1l1	1E-02	0.6
Lgr4	6E-05	0.6	B4galt1	9E-15	0.6
Acvrl1	6E-04	0.6	Cdc14b	3E-04	0.6
Map2k6	2E-04	0.6	Cygb	1E-02	0.6
Plin3	5E-23	0.6	Plce1	2E-05	0.6
Scrn1	4E-07	0.6	Naglu	1E-04	0.6
Il3ra	8E-10	0.6	Acad10	5E-02	0.6
Abhd4	1E-09	0.6	Tbx5	1E-01	0.6
Gm20559	2E-05	0.6	Zfp14	9E-02	0.6
Gm26724	1E-04	0.6	Mmp23	2E-22	0.6
Fsd1l	2E-02	0.6	C1qtnf1	5E-05	0.6
Adamts5	2E-14	0.6	Mmp14	7E-28	0.6
Prag1	1E-01	0.6	Hoxa7	4E-03	0.6
Bahd1	9E-02	0.6	Capn1	3E-04	0.6
Rab6b	2E-02	0.6	Dnm1	2E-09	0.6
P2rx4	3E-16	0.6	Osbpl10	6E-01	0.6
Slc27a1	3E-04	0.6	Dtx3l	5E-02	0.6
Aox1	2E-07	0.6	Hectd3	1E-01	0.6
Fam126a	3E-07	0.6	Plxna2	2E-08	0.6
Osbpl10	1E-01	0.6	Dgat2	9E-06	0.6
Khk	1E-07	0.6	Hs1bp3	1E-02	0.6
Sulf2	2E-21	0.6	Tap2	3E-05	0.6
Dusp10	1E-01	0.6	Scn1b	6E-14	0.6
Nnmt	2E-42	0.6	Rac3	5E-07	0.6
Dync1i1	6E-01	0.6	Neat1	4E-21	0.6
Zcchc2	7E-03	0.6	Stard10	8E-04	0.6
Rfx3	2E-02	0.6	Gm43507	5E-02	0.6
Noct	3E-01	0.6	Lgals9	1E-05	0.6
Mdga1	4E-02	0.6	Klhl13	4E-08	0.6
Cgnl1	1E-10	0.6	Mapre2	3E-08	0.6
Ifi211	1E-05	0.6	Pi4kb	4E-06	0.6
Olfml2b	1E-11	0.6	Igtp	4E-01	0.6
Rtl8b	3E-10	0.6	Zfp947	5E-01	0.6
Txlnb	2E-03	0.6	Ccdc189	2E-01	0.6
Slc41a3	1E-01	0.6	Retsat	1E-01	0.6
Fndc10	1E-01	0.6	Hoxd9	1E-01	0.6
Glul	2E-05	0.6	Trim30a	3E-04	0.6
Naglu	2E-06	0.6	Mapk13	1E-02	0.6
Trim47	2E-06	0.6	Dtx2	8E-02	0.6
Hspa12b	7E-02	0.6	Lefty1	7E-03	0.6
Tstd3	6E-04	0.6	Atp9a	2E-01	0.6
Gstm2	2E-23	0.6	Grasp	1E-01	0.6
Dnajc12	3E-03	0.6	Cgnl1	1E-11	0.6
Adam9	2E-15	0.6	Sh2d4a	5E-02	0.6
Mocs1	1E-02	0.6	Psap	1E-44	0.6

Limb Mesenchyme

Control Gene	p	FC	NICD2 Gene	p	FC
Nrip2	3E-09	0.5	Eif3c	2E-82	0.5
Cadm2	2E-05	0.5	Ak4	3E-06	0.5
Akirin2	2E-37	0.5	Brwd1	3E-24	0.5
4930444A19Rik	2E-11	0.5	Mrtfa	6E-21	0.5
Zxdb	3E-04	0.5	Rcc1l	2E-05	0.5
Spag4	2E-06	0.5	Psat1	2E-24	0.5
Fign	2E-21	0.5	Csrnp2	4E-05	0.5
Itpr1	1E-30	0.5	Zfp622	6E-21	0.5
Wasf1	8E-12	0.5	Rarg	1E-19	0.5
BC065397	9E-12	0.5	Jade2	1E-08	0.5
Nfxl1	2E-15	0.5	Cabp1	6E-04	0.5
Gm16279	9E-06	0.5	Setbp1	2E-47	0.5
Mef2c	2E-24	0.5	Agbl3	7E-09	0.5
Pakap.1	2E-22	0.5	Gm9929	2E-05	0.5
Osgin2	6E-17	0.5	Gm44686	3E-12	0.5
Gm49692	2E-07	0.5	Plcg2	8E-11	0.5
Serp1	5E-45	0.5	Slc20a2	6E-12	0.5
BC024978	5E-06	0.5	Lyrm7	3E-02	0.5
Rassf5	3E-10	0.5	Lrba	6E-24	0.5
Mtcl1	2E-09	0.5	Lmcd1	1E-18	0.5
Spata2	8E-09	0.5	Sema3f	1E-11	0.5
Rbm15b	1E-15	0.5	4933424G05Rik	9E-07	0.5
Impa2	3E-13	0.5	C79798	5E-04	0.5
Sybu	9E-11	0.5	Adamts9	4E-12	0.5
Klhl33	2E-05	0.5	4930557K07Rik	6E-06	0.5
Gm13056	2E-10	0.5	Tmeff1	2E-16	0.5
Zfp623	1E-09	0.5	Irgq	8E-08	0.5
Pecr	2E-12	0.5	Gchfr	4E-14	0.5
Itih2	3E-15	0.5	Gmds	2E-45	0.5
Tbl1x	1E-41	0.5	Xist	1E-08	0.5
Arhgap5	8E-44	0.5	Mdn1	1E-13	0.5
Gm2115	1E-10	0.5	Creb3l1	1E-32	0.5
Vps37b	3E-10	0.5	Baiap2l1	6E-11	0.5
Mpzl1	1E-17	0.5	Hspa9	4E-47	0.5
Bap1	1E-09	0.5	Lpar6	6E-10	0.5
Arl14ep	3E-24	0.5	Zfp637	9E-28	0.5
Cables1	9E-08	0.5	Tab2	4E-25	0.5
Slc25a33	3E-11	0.5	Cdk14	7E-43	0.5
Dipk2a	2E-17	0.5	Glul	1E-09	0.5
Papss2	2E-24	0.5	Prss23	2E-38	0.5
Med12l	1E-06	0.5	Chka	5E-12	0.5
Dchs1	5E-07	0.5	Chrdl1	4E-09	0.5
Gmds	2E-45	0.5	Gm43376	6E-06	0.5
Tinagl1	2E-07	0.5	Zswim1	1E-06	0.5
Igf2	9E-29	0.5	Rrp12	4E-06	0.5
Zfc3h1	1E-26	0.5	Rhbdd1	9E-16	0.5
Eif4ebp1	5E-44	0.5	Atf4	4E-39	0.5
4930455C13Rik	6E-04	0.5	Boc	3E-20	0.5
Plcb1	5E-43	0.5	Hsph1	1E-28	0.5
Selenoi	3E-13	0.5	Phgdh	1E-35	0.5
Gria3	6E-39	0.5	Cd82	2E-07	0.5
D10Wsu102e	9E-18	0.5	Tes	1E-10	0.5
D5Ertd579e	5E-24	0.5	Gnasas1	4E-12	0.5
Gnai1	4E-10	0.5	Gm15563	4E-10	0.5
Cacnb2	7E-30	0.5	Pak1	1E-18	0.5
Apobr	2E-08	0.5	Fzd7	2E-05	0.5
9630014M24Rik	5E-07	0.5	Gm19705	1E-07	0.5
Myh10	2E-10	0.5	Tpm1	7E-50	0.5
Nupr1	1E-54	0.5	Armc7	1E-04	0.5
Tars	2E-34	0.5	Rxfp1	4E-02	0.5
Igdcc4	3E-18	0.5	Dusp6	1E-08	0.5
Snx7	5E-26	0.5	Ercc6	7E-10	0.5
Bmp4	7E-33	0.5	B3gat2	4E-05	0.5
Arl4c	1E-11	0.5	Pomt2	8E-09	0.5
Cp	3E-17	0.5	Gm15050	4E-09	0.5
Herpud1	1E-39	0.5	Wfs1	1E-08	0.5
Ucma	2E-39	0.5	Smo	2E-23	0.5
Gm15563	2E-09	0.5	Dgke	1E-06	0.5
Enah	5E-22	0.5	Impa2	2E-12	0.5
Rec114	1E-05	0.5	Nrbf2	2E-19	0.5
Setbp1	1E-40	0.5	P4ha3	7E-17	0.5
Slc39a7	6E-39	0.5	Jade1	1E-11	0.5
Zfp11	2E-05	0.5	Pitx1	1E-37	0.5

Chondrogenic

Control Gene	p	FC	NICD2 Gene	p	FC
Cdc42se2	2E-22	0.6	Tctn2	2E-03	0.6
Slc41a2	1E-14	0.6	3110021N24Rik	1E-10	0.6
Gm26827	2E-08	0.6	Gulp1	6E-52	0.6
4930512B01Rik	1E-03	0.6	Hs3st3a1	2E-04	0.6
Zfp963	4E-05	0.6	Lrba	3E-31	0.6
Slc7a2	6E-28	0.6	Mtmr12	8E-07	0.6
Styxl1	9E-05	0.6	Kctd16	7E-05	0.6
Slc8b1	3E-02	0.6	Gm26944	2E-03	0.6
4930533B01Rik	5E-04	0.6	Ern1	2E-10	0.6
Epb41l4b	2E-05	0.6	Robo2	4E-09	0.6
Col8a2	2E-11	0.6	Lhx9	4E-03	0.6
Dcn	4E-86	0.6	Cmtm8	8E-06	0.6
Slc20a1	2E-07	0.6	Zfp169	1E-03	0.6
Lipo3	4E-11	0.6	Nudt6	2E-03	0.6
9530077C05Rik	3E-05	0.6	Net1	3E-09	0.6
Znrf3	1E-19	0.6	Mmp16	4E-09	0.6
Fxyd6	8E-11	0.6	Gm28375	1E-05	0.6
Igfbp5	3E-16	0.6	Abcc4	7E-06	0.6
Kif13b	8E-33	0.6	Gm15867	1E-05	0.6
Tom1l2	2E-35	0.6	Morn2	8E-19	0.6
Has2os	1E-06	0.6	Clba1	2E-05	0.6
6430590A07Rik	2E-10	0.6	Rtkn	1E-08	0.6
Mir22hg	8E-38	0.6	Gm16337	5E-02	0.6
Slc29a1	2E-31	0.6	Gm10658	1E-02	0.6
Phlpp1	9E-22	0.6	Pde3b	1E-10	0.6
Ak4	1E-04	0.6	Ptpn4	2E-16	0.6
Pnmal2	2E-05	0.6	D3Ertd751e	1E-06	0.6
5930430L01Rik	2E-13	0.6	Rtl1	1E-03	0.6
Pak3	2E-28	0.6	Mir22hg	2E-26	0.6
Magi3	6E-10	0.6	Tmem18	7E-05	0.6
Zfp827	2E-12	0.6	Klc4	2E-06	0.6
Soat1	6E-13	0.6	Ccn1	7E-38	0.6
Dlx5	4E-04	0.6	Chdl1	2E-02	0.6
Pitx1	9E-72	0.6	1700084C06Rik	1E-07	0.6
1700088E04Rik	1E-05	0.6	Gm16124	1E-06	0.6
Fzd3	8E-07	0.6	Car6	1E-02	0.6
Slc4a4	9E-03	0.6	Prelp	6E-30	0.6
Svip	2E-05	0.6	Itpr1	3E-34	0.6
Ogfod3	4E-15	0.6	G0s2	1E-15	0.6
Gpc6	2E-63	0.6	Gm16599	2E-05	0.6
8430436N08Rik	6E-03	0.6	Map7d3	4E-03	0.6
Bbs2	1E-08	0.6	Col4a5	2E-18	0.6
Rsph9	7E-12	0.6	Sh3bp4	6E-03	0.6
Ncoa3	2E-24	0.6	Edil3	4E-59	0.6
Tspan3	7E-136	0.6	Ipp	6E-03	0.6
Gm43126	1E-04	0.6	Cryzl2	1E-04	0.6
Ddb2	2E-04	0.6	Rbpj	5E-60	0.6
Avil	6E-03	0.6	Adamtsl3	2E-19	0.6
Slc25a23	1E-08	0.6	B230217C12Rik	3E-04	0.6
Vill	7E-04	0.6	Slc22a4	5E-03	0.6
Ric3	5E-07	0.6	Nyap1	8E-03	0.6
Catspere2	1E-07	0.6	Snhg4	7E-18	0.6
Cnnm4	5E-04	0.6	4930512B01Rik	9E-03	0.6
Lncppara	2E-40	0.6	Fjx1	6E-09	0.6
1500015A07Rik	6E-03	0.6	Tspan3	1E-97	0.6
Gulp1	1E-55	0.6	Nos1ap	7E-04	0.6
Runx2os1	1E-05	0.6	Otud1	3E-04	0.6
Pim3	6E-12	0.6	Jam2	5E-11	0.6
Ldhb	1E-37	0.6	Nr4a2	8E-04	0.6
Foxred2	3E-03	0.6	Usp53	4E-06	0.6
Trabd2b	9E-31	0.6	Ppip5k2	8E-11	0.6
Ficd	5E-06	0.6	Tmbim1	1E-16	0.6
Mir99ahg	2E-60	0.6	Etl4	2E-19	0.6
4930556M19Rik	9E-06	0.6	Foxp4	6E-08	0.6
C1rl	3E-13	0.6	Ttll7	5E-06	0.6
Enpp4	2E-04	0.6	Car9	3E-04	0.6
4930545L23Rik	2E-06	0.6	A130010J15Rik	3E-03	0.6
Fgfbp3	1E-04	0.6	P2ry10b	5E-05	0.6
Gm17435	9E-04	0.6	Plag1	2E-07	0.6
B930095G15Rik	9E-05	0.6	4930533B01Rik	3E-03	0.6
Csgalnact1	6E-49	0.6	Lrrn3	1E-05	0.6
Fndc3b	6E-81	0.6	Sdc2	3E-63	0.6
Vps37b	3E-07	0.6	Twnk	1E-03	0.6

Fibroblast

Control Gene	p	FC	NICD2 Gene	p	FC
Fgf2	4E-38	0.5	Jag1	3E-38	0.5
Il4ra	3E-13	0.5	Hhat	8E-11	0.5
Adam23	3E-70	0.5	St8sia2	1E-16	0.5
Deptor	1E-28	0.5	Fbln5	8E-87	0.5
Ncald	2E-08	0.5	Zfp992	7E-14	0.5
Rbms1	1E-295	0.5	4933424G05Rik	2E-04	0.5
Tmem198b	2E-18	0.5	Ankrd55	3E-10	0.5
Ildr2	1E-39	0.5	Meox2	1E-23	0.5
4930533K18Rik	6E-15	0.5	F2r	5E-20	0.5
Smim1	6E-149	0.5	Fras1	4E-05	0.5
Shfl	9E-36	0.5	A330076H08Rik	9E-14	0.5
Grasp	3E-28	0.5	Picalm	1E-71	0.5
Adamtsl3	4E-127	0.5	Slc38a4	3E-13	0.5
Abhd14b	4E-53	0.5	P4ha3	6E-34	0.5
Bnc2	3E-123	0.5	Plekhg2	2E-23	0.5
Tmem47	1E-50	0.5	Gbp2	1E-19	0.5
C3	1E-27	0.5	2610008E11Rik	3E-31	0.5
Pid1	5E-71	0.5	Lhfp	2E-101	0.5
Irx3	2E-19	0.5	Gm20631	3E-09	0.5
Rab7b	8E-35	0.5	Epha7	8E-23	0.5
Mark1	2E-34	0.5	Kif5c	7E-08	0.5
Alms1	3E-37	0.5	Sugct	2E-23	0.5
Timp1	4E-249	0.5	Porcn	1E-12	0.5
Ptges	2E-39	0.5	Fhl2	8E-46	0.5
Col18a1	4E-202	0.5	Hspb1	1E-54	0.5
Meox2	5E-47	0.5	Tcf7l2	2E-73	0.5
Palm3	1E-22	0.5	5033430I15Rik	7E-06	0.5
Fbln5	1E-165	0.5	4930533K18Rik	1E-06	0.5
Tent2	5E-128	0.5	Gm16201	5E-17	0.5
Dennd2a	2E-47	0.5	Hdx	2E-09	0.5
Cpeb2	1E-34	0.5	Scn2a	1E-07	0.5
Thbs2	3E-210	0.5	Veph1	2E-08	0.5
Arhgap29	2E-166	0.5	Gm9801	6E-11	0.5
Hykk	2E-20	0.5	Myo7a	1E-13	0.5
Scn2a	3E-18	0.5	Gem	1E-34	0.5
Lrp4	5E-27	0.5	Mapk7	1E-11	0.5
Ptgr1	4E-44	0.5	Rbms1	1E-130	0.5
Hdx	7E-20	0.5	Naprt	8E-09	0.5
Cd46	5E-18	0.5	Tmem150a	1E-27	0.5
Oat	4E-265	0.5	Gas7	1E-15	0.5
Slc38a6	8E-34	0.5	Mpp1	9E-36	0.5
Nrbp2	1E-17	0.5	Slc1a6	2E-29	0.5
Gpc3	6E-74	0.5	Mmp14	8E-93	0.5
Gucy1a2	3E-20	0.5	Srpx	6E-45	0.5
Ddx60	7E-26	0.5	Nrp1	1E-85	0.5
Pcdhgc3	6E-29	0.5	Renbp	5E-34	0.5
Fndc10	2E-14	0.5	Gpr173	7E-18	0.5
Wwc1	2E-09	0.5	Scara5	1E-38	0.5
Gm20707	6E-17	0.5	Amot	6E-13	0.5
Mapk7	6E-21	0.5	Lgalsl	3E-22	0.5
Ptk7	3E-58	0.5	Plce1	3E-59	0.5
Stx1a	1E-11	0.5	C2	1E-08	0.5
Col3a1	0E+00	0.5	Arhgap42	4E-54	0.5
Gngt2	8E-25	0.5	Naaa	3E-10	0.5
Lrrc8e	2E-12	0.5	Arrdc4	7E-21	0.5
Igtp	3E-14	0.5	Ece1	6E-27	0.5
Renbp	3E-66	0.5	Slc9a3r2	5E-26	0.5
Tacc1	8E-127	0.5	Dmpk	8E-26	0.5
Kif5c	8E-13	0.5	Lamb1	6E-68	0.5
Adamts5	7E-79	0.5	Ddx60	4E-14	0.5
Cacnb3	2E-23	0.5	Col3a1	8E-199	0.5
Ccdc102a	4E-88	0.5	C3	2E-10	0.5
Emc2	2E-189	0.5	Pabpc4l	1E-15	0.5
Tnfsf11	1E-07	0.5	Sulf2	2E-43	0.5
Ece1	6E-48	0.5	Il11ra1	2E-120	0.5
Mgst3	4E-135	0.5	Irgm2	3E-12	0.5
Galk2	1E-118	0.5	Gbp7	4E-14	0.5
Galnt13	1E-52	0.5	Pxylp1	4E-07	0.5
Bmp1	1E-105	0.5	Skil	3E-09	0.5
S1pr1	1E-21	0.5	Pdlim2	5E-74	0.5
Pcdh7	7E-138	0.5	Steap3	6E-82	0.5
Plce1	7E-135	0.5	Svil	2E-89	0.5
4930402H24Rik	1E-86	0.5	Pid1	6E-29	0.5

Fibroblast?

Control Gene	p	FC	NICD2 Gene	p	FC
Ttc9	2E-43	1.5	Nucks1	0E+00	1.5
Gm13889	1E-71	1.5	Lsm3	7E-238	1.5
Zfp101	5E-96	1.5	Mphosph9	1E-111	1.5
Zik1	5E-65	1.5	Lipg	5E-38	1.4
Fam110a	5E-86	1.5	Rif1	2E-159	1.4
Cdk5rap2	2E-143	1.5	Arpin	1E-105	1.4
Ngf	2E-33	1.5	Dhrs13	5E-103	1.4
Cdc25a	3E-103	1.5	Rin3	1E-67	1.4
Ppil1	4E-103	1.5	Zik1	3E-66	1.4
Stard8	9E-84	1.5	Hist2h2aa1	9E-51	1.4
Crip1	9E-120	1.5	Cenpt	5E-117	1.4
Hunk	1E-35	1.5	Gm13415	7E-43	1.4
Map2	2E-125	1.5	Anks1b	2E-61	1.4
Cenpj	8E-92	1.5	Morrbid	2E-177	1.4
Pdss1	5E-83	1.5	Pdss1	2E-122	1.4
Cdca4	3E-105	1.5	Pcnt	2E-153	1.4
Fancg	7E-44	1.5	Acot7	6E-124	1.4
2700049A03Rik	1E-98	1.5	Gm49980	9E-48	1.4
Psrc1	2E-107	1.5	Tmem117	5E-59	1.4
Cep112	1E-100	1.5	Poln	3E-60	1.4
Ccher1	2E-105	1.5	Prrg4	3E-34	1.4
Alg13	2E-99	1.4	Nde1	5E-134	1.4
Gjc1	1E-117	1.4	Ctc1	2E-121	1.4
Mis12	4E-133	1.4	Ppp1r18os	2E-42	1.4
Ckap5	1E-147	1.4	Plvap	1E-75	1.4
Piga	1E-55	1.4	Tuba1c	3E-129	1.4
Ywhah	9E-213	1.4	Lmod1	1E-53	1.4
Slco2a1	4E-63	1.4	C430002N11Rik	6E-30	1.4
Alg8	1E-94	1.4	Tagap1	4E-51	1.4
Zfp948	4E-114	1.4	4930503L19Rik	1E-126	1.4
Tcfl5	5E-53	1.4	Ccher1	4E-119	1.4
C2cd4a	1E-14	1.4	Lsm11	4E-98	1.4
Etv4	1E-27	1.4	Prkar2a	1E-133	1.4
Ccdc77	2E-94	1.4	Lsm8	3E-223	1.4
Il1rl1	3E-57	1.4	Kctd18	5E-100	1.4
Prkg2	3E-44	1.4	Gm5577	1E-68	1.4
Tfdp1	5E-140	1.4	Hspa4l	4E-82	1.4
Cep76	2E-70	1.4	Dcaf15	3E-44	1.4
Gch1	9E-37	1.4	Nav2	4E-147	1.4
Hspa4l	8E-81	1.4	Crip1	4E-145	1.4
Psip1	4E-199	1.4	Alg8	2E-118	1.4
Gfra2	3E-26	1.4	Zfp948	2E-145	1.4
Plvap	1E-43	1.4	Ddx39	4E-134	1.4
Gm5577	1E-57	1.4	Ahcy	1E-22	1.4
Bmp6	1E-35	1.4	Fgfr1op	7E-90	1.4
Pcnt	4E-104	1.4	Acaa1b	3E-68	1.4
Nphp4	3E-36	1.4	Afmid	2E-94	1.4
Pms2	1E-95	1.4	Cep70	1E-86	1.4
Pfkfb1	5E-36	1.4	Gm43305	2E-42	1.4
Rab15	6E-47	1.4	Casp8ap2	4E-160	1.4
Tmeff2	1E-44	1.4	Psrc1	8E-113	1.4
Arhgap33	4E-23	1.4	Rnaseh2b	2E-128	1.4
Med9os	1E-42	1.4	Mars2	2E-21	1.4
Nucks1	2E-265	1.4	Dusp8	5E-42	1.4
Plaur	1E-55	1.4	Tmeff2	2E-53	1.3
Kctd18	3E-86	1.4	Gm11837	6E-32	1.3
Tuba1c	2E-111	1.4	Slc25a10	1E-120	1.3
Tbc1d8	1E-25	1.4	Ran	0E+00	1.3
Bub3	6E-167	1.4	Ccdc163	4E-51	1.3
Cenpt	5E-96	1.4	Pgp	1E-238	1.3
Nemp2	4E-41	1.4	Gdf15	4E-69	1.3
Lrrc8b	3E-60	1.4	Kctd9	2E-103	1.3
Mamstr	7E-65	1.4	Stard8	8E-82	1.3
Aif1l	1E-41	1.4	Camk2n2	8E-116	1.3
C430002N11Rik	1E-29	1.4	Ckap5	4E-158	1.3
Sacs	1E-86	1.4	Rnaseh2a	5E-101	1.3
Gins4	5E-115	1.4	Ssrp1	1E-271	1.3
Vsig10	2E-31	1.4	Gprc5a	3E-55	1.3
Pim1	2E-61	1.4	Med9os	4E-46	1.3
Alyref	5E-186	1.4	1700025G04Rik	8E-124	1.3
Ptgs2	1E-31	1.4	Alyref	3E-251	1.3
Cenpo	2E-66	1.4	Bub3	1E-193	1.3
Anks1b	3E-46	1.4	Nup205	2E-126	1.3

Undefined

Control Gene	p	FC	NICD2 Gene	p	FC
Cys1	2E-01	0.2	Os9	2E-16	0.2
Pxdn	3E-06	0.2	Snx21	2E-01	0.2
Rnf25	1E-01	0.2	Gabbr1	9E-03	0.2
Arf6	6E-08	0.2	Ccdc28b	8E-01	0.2
Cirbp	2E-05	0.2	Cemip	4E-01	0.2
Ndufaf7	6E-03	0.2	Hivep2	4E-04	0.2
Syn3	1E-15	0.2	Ago1	2E-01	0.2
Ralgds	2E-02	0.2	Ptprf	1E-02	0.2
Tmem26	2E-01	0.2	B3gnt9	4E-04	0.2
Nfia	3E-21	0.2	Ccdc191	8E-03	0.2
Spata33	1E+00	0.2	Fgfbp3	7E-01	0.2
Gm41609	5E-01	0.2	6430590A07Rik	6E-02	0.2
Btg2	3E-04	0.2	Fbxw17	8E-05	0.2
Plekhg5	1E-04	0.2	Ctsh	1E-15	0.2
Ndfip2	5E-05	0.2	Gramd4	4E-01	0.2
Cgnl1	9E-05	0.2	Dnaaf4	6E-01	0.2
Sfxn3	3E-03	0.2	Hmcn1	6E-01	0.2
Gm20536	9E-01	0.2	Ppp3ca	1E-26	0.2
Imp3	6E-17	0.2	Ptprk	2E-03	0.2
Spsb3	2E-01	0.2	Ptpn1	1E-08	0.2
Tspan3	2E-31	0.2	Klf8	2E-01	0.2
Zc3h3	9E-01	0.2	Cebpb	6E-12	0.2
Tgif1	3E-02	0.2	Txndc15	1E-13	0.2
Adcy9	3E-02	0.2	Rbks	7E-01	0.2
Atp6ap2	3E-10	0.2	Zfand2a	1E-03	0.2
Foxo1	5E-03	0.2	Trmu	8E-01	0.2
Smg8	8E-01	0.2	Foxp2	5E-09	0.2
Rnaset2a	2E-02	0.2	Rhobtb2	3E-01	0.2
Nck2	7E-02	0.2	Cpeb4	2E-04	0.2
Bphl	2E-05	0.2	Pacsin3	5E-01	0.2
Gm15417	7E-06	0.2	Nckap5	1E-07	0.2
Cyb5rl	4E-01	0.2	Asb13	7E-01	0.2
Rufy1	4E-04	0.2	Thg1l	3E-01	0.2
Cyp4f13	3E-01	0.2	Hilpda	1E-01	0.2
Enpp2	2E-06	0.2	Pex11g	5E-01	0.2
Slc30a5	7E-03	0.2	Aspn	2E-05	0.2
Dchs1	6E-02	0.2	Nudt14	2E-01	0.2
Cmtm4	2E-01	0.2	Socs2	9E-01	0.2
0610043K17Rik	2E-01	0.2	E130308A19Rik	7E-01	0.2
Slc35e4	1E-02	0.2	Zfp955b	7E-02	0.2
Hoxa10	4E-13	0.2	Pigx	9E-06	0.2
Msantd3	3E-01	0.2	Ift122	3E-01	0.2
Slc18b1	7E-02	0.2	Nprl3	4E-01	0.2
Wdr73	4E-01	0.2	Sco2	4E-02	0.2
Dmac2	4E-01	0.2	S100a1	4E-15	0.2
Akap7	7E-02	0.2	Mad2l1bp	8E-01	0.2
Zfp827	5E-04	0.2	Dock4	4E-02	0.2
Mcat	9E-01	0.2	Mat2a	5E-05	0.2
Fam32a	1E-03	0.2	Eng	2E-01	0.2
Lncppara	3E-06	0.2	Fam107b	5E-01	0.2
2610301B20Rik	2E-02	0.2	Hnrnph3	2E-03	0.2
Ctsh	8E-18	0.2	Ndufaf1	8E-01	0.2
Eaf2	2E-01	0.2	Ada	5E-01	0.2
Smug1	3E-01	0.2	Tafla	1E+00	0.2
Crip2	3E-16	0.2	Zfp687	6E-01	0.2
Eef2kmt	2E-03	0.2	Tmed1	6E-05	0.2
Smoc1	2E-15	0.2	Per2	1E-01	0.2
D930016D06Rik	3E-01	0.2	Errfi1	4E-04	0.2
Mxi1	2E-01	0.2	Mturn	4E-01	0.2
Ptprk	8E-04	0.2	Amotl1	1E-06	0.2
Nfatc1	3E-01	0.2	Naaladl2	1E-14	0.2
Fahd2a	1E-01	0.2	Dock9	7E-01	0.2
Fat3	5E-02	0.2	Egfros	1E-01	0.2
Itm2c	6E-14	0.2	Zfp821	6E-01	0.2
Vegfa	3E-01	0.2	Ppard	6E-01	0.2
Rnaset2b	1E-01	0.2	Ccdc181	9E-01	0.2
Cd82	3E-01	0.2	Tefm	6E-01	0.2
Medag	9E-02	0.2	Gtf2h3	5E-01	0.2
Acot2	2E-03	0.2	Tpmt	1E+00	0.2
Shld1	1E-01	0.2	Hdhd3	1E+00	0.2
Oggl	7E-01	0.2	Aptx	1E-01	0.2
Tex14	2E-02	0.2	Lrrc32	1E-02	0.2
Mdk	6E-02	0.2	Yju2	5E-01	0.2

Articular/Synovial Fibroblast

Control Gene	p	FC	NICD2 Gene	p	FC
Dpp7	3E-10	0.6	Gm16536	3E-01	0.6
Inka2	2E-03	0.6	Ccdc39	1E-01	0.6
Dusp16	6E-05	0.6	Slc24a3	5E-16	0.6
Gm28905	1E-02	0.6	Mar9	2E-01	0.6
Gbp2	3E-04	0.6	Gm11266	8E-02	0.6
Tcp11l2	6E-07	0.6	Smpdl3a	5E-17	0.6
Olfml1	6E-05	0.6	Slc9a1	7E-03	0.6
Lox	1E-49	0.6	Klf10	1E-01	0.6
Plekhg3	6E-02	0.6	Man1a	1E-08	0.6
Dnase2a	1E-03	0.6	2410022M11Rik	6E-01	0.6
Npc2	2E-92	0.6	Gpsm2	5E-10	0.6
Smyd3	3E-09	0.6	Pot1b	2E-02	0.6
Enpp5	6E-08	0.6	Ugcg	7E-07	0.6
Mroh1	3E-06	0.6	Hspa12b	1E-02	0.6
Tnks	7E-19	0.6	Il3ra	1E-06	0.6
Nol3	1E-05	0.6	Acvrl1	3E-03	0.6
Tuba4a	3E-02	0.6	Lyrm9	3E-01	0.6
Gramd1c	1E-01	0.6	Mrgbp	4E-02	0.6
St6galnac2	4E-03	0.6	Tlr2	1E-01	0.6
Crip1	8E-40	0.6	Fbln7	3E-09	0.6
Dtx2	1E-04	0.6	Enpp4	4E-01	0.6
Zfp607b	4E-01	0.6	Glmp	1E-19	0.6
Ctso	2E-04	0.6	Tshz3	1E-03	0.6
Irgm2	9E-04	0.6	Kbtbd3	1E-01	0.6
Hsd17b11	7E-06	0.6	Hexa	2E-25	0.6
Baiap2	1E-09	0.6	Tctn1	5E-01	0.6
Slc4a4	3E-01	0.6	Fam102b	3E-11	0.6
Lsp1	3E-26	0.6	Arsb	1E-06	0.6
Lhpp	2E-01	0.6	Ptpdc1	2E-02	0.6
Trnp1	4E-07	0.6	Renbp	3E-04	0.6
Jun	4E-40	0.6	Spg21	1E-17	0.6
Gm14636	2E-01	0.6	Prtg	3E-01	0.6
Zc3h12a	3E-02	0.6	Ccdc149	1E-01	0.6
Prrg3	7E-04	0.6	Repin1	5E-01	0.6
Efemp2	1E-79	0.6	Pgpep1	7E-07	0.6
Camkmt	9E-06	0.6	Gm49959	2E-01	0.6
Il4ra	7E-02	0.6	Calhm2	1E-01	0.6
Tlr4	4E-05	0.6	Lbp	3E-05	0.6
Dkk3	6E-41	0.6	Man2b1	1E-09	0.6
Man2b1	2E-13	0.6	Pqlc3	2E-02	0.6
Prxl2b	7E-26	0.6	Tgfa	8E-03	0.6
C1s1	3E-14	0.6	Gba	4E-05	0.6
Bid	9E-02	0.6	Fcgrt	2E-33	0.6
2900005J15Rik	2E-01	0.6	Rab3d	2E-01	0.6
Prickle3	3E-01	0.6	Lox	2E-34	0.6
Fam102a	2E-02	0.6	Hyal1	3E-01	0.6
Ticam1	2E-01	0.6	Mmaa	2E-02	0.6
Helz2	1E-01	0.6	Ifi211	7E-03	0.6
Siae	6E-04	0.6	Tcn2	4E-13	0.6
Trim30a	5E-05	0.6	Arhgap21	5E-11	0.6
Arhgap22	9E-04	0.6	6330403K07Rik	3E-02	0.6
Pik3r2	4E-07	0.6	Ptgs1	3E-01	0.6
Plekhm2	1E-06	0.6	Acaa2	3E-14	0.6
Dhx58	2E-02	0.6	Oplah	5E-01	0.6
Gbp6	1E-01	0.6	Alas1	2E-06	0.6
Dtx3l	1E-03	0.6	Sncaip	7E-02	0.6
Gm765	5E-01	0.6	Sord	8E-10	0.6
Wnt16	4E-05	0.6	Ecm1	4E-24	0.6
Enah	1E-16	0.6	Zkscan6	2E-01	0.6
Prune2	7E-02	0.6	Agtrap	1E-02	0.6
Irgm1	1E-04	0.6	1110019D14Rik	3E-03	0.6
Gm12743	3E-04	0.6	Letm2	7E-04	0.6
Acad10	2E-02	0.6	Col24a1	4E-01	0.6
Ropn1l	2E-02	0.6	Nqo2	4E-02	0.6
Tlr2	1E-01	0.6	Peli2	3E-06	0.6
Mvp	2E-07	0.6	Sox5	5E-18	0.6
Adcy9	8E-05	0.6	Zeb2	3E-28	0.6
Zfp954	1E-01	0.6	Timp2	5E-66	0.6
Triobp	3E-06	0.6	Trp53inp1	8E-06	0.6
Stat5a	3E-02	0.6	Olfml1	5E-07	0.6
D6Wsu163e	1E-02	0.6	Cmtm3	4E-08	0.6
Mmp23	2E-21	0.6	Ccdc9b	4E-01	0.6
Ptgfrn	1E-04	0.6	Rtl8b	4E-04	0.6

Limb Mesenchyme					
Control			NICD2		
Gene	*p*	FC	Gene	*p*	FC
Cnn3	7E-60	0.5	Crispld1	5E-47	0.5
Clcn5	2E-15	0.5	Tgfb3	1E-23	0.5
9630028H03Rik	1E-09	0.5	4930511M06Rik	4E-04	0.5
Gramd1b	2E-10	0.5	Itpkc	5E-05	0.5
Gm26981	7E-05	0.5	Nacc2	3E-16	0.5
Col2a1	2E-118	0.5	Tspan9	4E-16	0.5
Palld	3E-41	0.5	Ide	1E-25	0.5
Nol10	2E-12	0.5	Tars	2E-29	0.5
P4ha1	3E-66	0.5	4930455C13Rik	4E-05	0.5
Sesn2	9E-18	0.5	Hps3	3E-08	0.5
P3h1	9E-24	0.5	Armh4	2E-16	0.5
Ankrd29	9E-14	0.5	Hk2	5E-13	0.5
Gdf10	1E-09	0.5	Ptgis	8E-17	0.5
Gm9968	8E-07	0.5	D1Ertd622e	5E-04	0.5
D030028A08Rik	2E-05	0.5	Dcaf12l1	3E-05	0.5
Gfpt1	4E-35	0.5	Cdc42ep1	8E-09	0.5
Epb41l4b	3E-05	0.5	Cdk17	1E-12	0.5
A930005H10Rik	1E-15	0.5	Satb1	1E-06	0.5
Plekhh2	6E-26	0.5	Sh3bp4	2E-06	0.5
Nomo1	1E-21	0.5	Stc2	3E-10	0.5
Kctd12	3E-14	0.5	Irs2	2E-10	0.5
Nars	2E-57	0.5	Tspan13	4E-13	0.5
Aars2	5E-04	0.5	Alg2	4E-11	0.5
Unc5c	9E-19	0.5	Rad23b	8E-31	0.5
Cd59a	1E-08	0.5	Slc26a2	2E-08	0.5
Gm49959	2E-09	0.5	Zfp579	2E-11	0.5
Glrb	3E-07	0.5	Zfp940	9E-05	0.5
Mettl14	4E-09	0.5	Gm17529	2E-06	0.5
Trp53rka	4E-09	0.4	P4ha1	3E-57	0.5
Gm15834	6E-09	0.4	Itga1	3E-18	0.5
Gm43948	2E-09	0.4	Prkg2	7E-08	0.5
B930095G15Rik	5E-08	0.4	Gm10130	4E-06	0.4
Gm20404	2E-06	0.4	Dipk2a	1E-14	0.4
Ptprf	4E-14	0.4	Epb41	1E-12	0.4
Lrrc59	1E-30	0.4	Galnt10	1E-11	0.4
Fjx1	2E-07	0.4	Gpr176	2E-11	0.4
Gm16599	7E-08	0.4	Fndc3b	2E-60	0.4
Nacc2	2E-19	0.4	Col11a2	2E-22	0.4
Uso1	5E-29	0.4	Adgra3	9E-14	0.4
Chrdl1	6E-11	0.4	Zbed4	3E-02	0.4
Rai14	4E-19	0.4	Gm20300	2E-05	0.4
Gm17501	1E-16	0.4	Ctps	7E-16	0.4
Sept7	2E-65	0.4	Zfp810	4E-13	0.4
Otulinl	2E-04	0.4	Ust	6E-13	0.4
Rarg	2E-20	0.4	Gm15614	8E-07	0.4
Neo1	2E-31	0.4	Trpm6	8E-06	0.4
Galnt10	1E-18	0.4	Prss35	7E-11	0.4
Skil	1E-11	0.4	Kdm6b	3E-13	0.4
Wwp2	2E-27	0.4	Myo1e	2E-35	0.4
Kdm4a	4E-17	0.4	Phf10	2E-29	0.4
Gm28153	4E-26	0.4	Zfp316	1E-05	0.4
Hoxa9	5E-25	0.4	Gm16599	7E-10	0.4
Zscan25	1E-06	0.4	Dhrs3	1E-16	0.4
Resf1	3E-13	0.4	Myo1d	2E-27	0.4
Dzip3	3E-20	0.4	Fmnl3	1E-12	0.4
Sema3f	6E-12	0.4	B930095G15Rik	2E-06	0.4
Pitx1	9E-44	0.4	Asxl1	8E-21	0.4
Cdk14	7E-47	0.4	Taf15	1E-35	0.4
Fst	8E-25	0.4	4930517O19Rik	6E-04	0.4
Kdelr3	1E-23	0.4	Tex14	3E-15	0.4
Gm10974	3E-06	0.4	Wars2	8E-13	0.4
Tert	2E-05	0.4	Prr7	4E-15	0.4
Amotl2	1E-29	0.4	Nckap5	4E-25	0.4
Nrk	4E-18	0.4	Gls	9E-31	0.4
Boc	1E-18	0.4	Stx11	2E-03	0.4
Ddit3	1E-33	0.4	Nol10	3E-12	0.4
Dhrs3	5E-18	0.4	Fbxl2	7E-12	0.4
Pim3	3E-11	0.4	Tpk1	4E-15	0.4
Itprid2	1E-10	0.4	Cdca7	1E-03	0.4
Rmnd5a	6E-17	0.4	Ctf1	2E-10	0.4
Abhd6	1E-06	0.4	Galnt4	2E-08	0.4
Tcea1	1E-51	0.4	Ppp1r12b	1E-12	0.4
Gm13402	6E-06	0.4	Ttl	1E-05	0.4

Chondrogenic					
Control			NICD2		
Gene	*p*	FC	Gene	*p*	FC
Cystm1	2E-02	0.6	Ifit2	2E-06	0.6
Bcar3	5E-15	0.6	Zdhhc12	1E-07	0.6
B230217C12Rik	3E-07	0.6	5930430L01Rik	9E-11	0.6
Snhg4	5E-23	0.6	Rragb	1E-05	0.6
a	6E-04	0.6	Lrrc75b	2E-04	0.6
Fut8	3E-16	0.6	Fli1	1E-04	0.6
Abhd6	7E-07	0.6	Tpcn1	6E-06	0.6
Zzef1	1E-28	0.6	Sdc4	6E-70	0.6
Kif6	1E-04	0.6	Gm5577	3E-02	0.6
Itih2	6E-18	0.6	Tex14	1E-17	0.6
Foxp4	3E-13	0.6	Lrrc49	2E-06	0.6
Znhit6	1E-20	0.6	Cd82	6E-07	0.6
Fli1	2E-06	0.6	Mir99ahg	1E-49	0.6
Hspb2	1E-12	0.6	Rgl3	2E-03	0.6
Rcan2	8E-31	0.6	Cpeb4	9E-14	0.6
Clcn3	8E-14	0.6	Mtss1	1E-05	0.6
Meg3	5E-70	0.6	Rassf3	7E-05	0.6
Cenpp	3E-37	0.6	Dusp18	5E-06	0.6
Zdhhc12	3E-13	0.6	Ltbp1	3E-28	0.6
Ciapin1	9E-13	0.6	Hdac9	2E-04	0.6
Ndufa4l2	2E-25	0.6	Zpbp	4E-02	0.6
Zswim4	4E-06	0.6	Akap7	7E-11	0.6
Dlgap1	4E-03	0.6	Bbs4	5E-03	0.6
Spata1	6E-07	0.6	Gm48678	2E-05	0.6
Col24a1	1E-03	0.6	Ptpn21	3E-11	0.6
Col4a5	2E-28	0.6	Itga6	5E-04	0.6
Gm16599	9E-04	0.6	6230400D17Rik	2E-02	0.5
Gm15050	1E-04	0.6	Osbpl9	1E-39	0.5
Gm19412	6E-06	0.6	Sil1	3E-28	0.5
6030443J06Rik	5E-10	0.6	Pak1	8E-14	0.5
Nacc2	1E-22	0.6	Gm12940	1E-03	0.5
2810457G06Rik	3E-07	0.6	Ift57	2E-17	0.5
Ern1	7E-10	0.6	Gm26887	5E-08	0.5
Hoxd9	8E-03	0.6	Pld1	1E-10	0.5
Gab1	2E-23	0.6	Scarf2	1E-15	0.5
Tmem246	3E-07	0.6	Cys1	1E-06	0.5
Tspan12	1E-05	0.6	Abhd6	7E-02	0.5
Cmbl	3E-03	0.6	Ids	9E-11	0.5
Tafa5	2E-16	0.6	2610306M01Rik	2E-03	0.5
Dstn	2E-76	0.6	Glcci1	8E-13	0.5
Gm44686	1E-08	0.6	Ggnbp1	5E-05	0.5
Rassf7	7E-04	0.6	Slc25a33	3E-04	0.5
Gm4258	3E-17	0.6	Akt3	1E-20	0.5
Mpp7	2E-15	0.6	Strbp	9E-39	0.5
Cbr2	4E-24	0.6	Prdm16	1E-11	0.5
Lhx9	3E-05	0.6	Tgoln1	1E-41	0.5
Cyp27a1	7E-06	0.6	Zfp827	2E-10	0.5
Itpr1	3E-35	0.5	Pxdc1	1E-05	0.5
Jam2	2E-11	0.5	Igfbp5	6E-03	0.5
C1ql3	3E-03	0.5	6530409C15Rik	3E-03	0.5
Kazald1	4E-18	0.5	Lysmd4	4E-09	0.5
Glcci1	1E-12	0.5	Cdkn2c	1E-21	0.5
Chadl	3E-04	0.5	Gm17106	6E-05	0.5
Map9	8E-05	0.5	Eml2	2E-06	0.5
Rsph3b	9E-05	0.5	1810059H22Rik	4E-03	0.5
Spa17	2E-05	0.5	Fam71e1	2E-04	0.5
Rgmb	6E-09	0.5	Sod3	5E-26	0.5
Ipp	1E-03	0.5	Gm50013	1E-02	0.5
Imp3	1E-58	0.5	Bcar3	2E-09	0.5
Ppip5k2	3E-14	0.5	Pde4d	1E-08	0.5
2810403D21Rik	1E-15	0.5	Osbpl6	2E-06	0.5
Gm32618	8E-09	0.5	1700029J07Rik	5E-03	0.5
Orai1	4E-15	0.5	Sorbs3	8E-11	0.5
Bend6	2E-04	0.5	Mgp	5E-02	0.5
Mknk1	4E-06	0.5	Tsix	2E-02	0.5
Fbxl2	2E-04	0.5	Gm11944	5E-03	0.5
Saraf	5E-38	0.5	Imp3	2E-40	0.5
Atp2b1	6E-52	0.5	Tcte2	6E-03	0.5
Crybg3	4E-12	0.5	Chadl	7E-03	0.5
Tmpo	8E-32	0.5	Aplp2	5E-59	0.5
Mamld1	2E-08	0.5	Bcl2l1	1E-13	0.5
Klhl21	8E-06	0.5	Hs6st2	1E-06	0.5
Psd3	5E-35	0.5	Plekhh2	9E-25	0.5

Fibroblast					
Control			NICD2		
Gene	*p*	FC	Gene	*p*	FC
Mss51	3E-10	0.5	Fat3	4E-12	0.5
Il11ra1	1E-243	0.5	Ier3	1E-29	0.5
2610008E11Rik	5E-43	0.5	4930402H24Rik	7E-46	0.5
Mmp14	3E-171	0.5	Stx1a	4E-05	0.5
Mrps6	6E-101	0.5	Cdkn2b	2E-46	0.5
Man1c1	1E-63	0.5	Oat	2E-138	0.5
Gm4221	2E-15	0.5	Car13	1E-11	0.5
Kcnab1	3E-28	0.5	Bmp1	1E-55	0.5
Irgm2	2E-22	0.5	Gm20559	4E-16	0.5
Abca9	1E-29	0.5	Cmtm3	7E-41	0.5
L3hypdh	5E-32	0.5	Rnf144a	1E-17	0.5
Plekhg2	3E-31	0.5	Tent2	7E-58	0.5
Zfp618	2E-31	0.5	S1pr1	1E-12	0.5
Hopx	1E-52	0.5	Tmem198b	5E-09	0.5
Srpx	5E-78	0.5	Hsd17b7	1E-16	0.5
Laptm4b	3E-33	0.5	Slit3	2E-22	0.5
Idi1	4E-65	0.5	Nat8f1	2E-13	0.5
Pdlim2	4E-138	0.5	Mgll	3E-24	0.5
Unc93b1	2E-61	0.5	Col18a1	7E-100	0.5
Gab3	3E-12	0.5	Tcf7l1	8E-50	0.5
Nrp1	3E-131	0.5	Rab40b	2E-20	0.5
Steap3	6E-154	0.5	Cpxm2	7E-09	0.5
Myo7a	2E-20	0.5	Abcb6	1E-04	0.5
Psmb9	5E-34	0.5	Gm13832	1E-09	0.5
Zfp423	6E-22	0.5	Junb	6E-67	0.5
Gas7	8E-36	0.5	Rnf150	4E-36	0.5
Slc9a3r2	6E-41	0.5	Inka1	2E-18	0.5
Stat2	6E-49	0.5	Galk2	6E-54	0.5
Acss3	4E-25	0.5	Dennd2a	4E-21	0.5
Gm13832	5E-19	0.5	Mov10	3E-12	0.5
Rab40b	8E-35	0.5	Zfp449	2E-12	0.5
Sqle	4E-55	0.5	Gm20707	3E-07	0.5
Pcyt1b	4E-14	0.5	Nfatc4	5E-31	0.5
Ttc39c	5E-22	0.5	Glis1	6E-20	0.5
Add3	6E-107	0.5	Gpc3	1E-32	0.5
Maged2	2E-118	0.5	Tap2	1E-26	0.5
Naaa	1E-16	0.5	Pcdhga10	6E-07	0.5
Ptgfr	2E-17	0.5	Tmem47	7E-20	0.5
Ndn	8E-111	0.5	Laptm4b	3E-17	0.5
Gsta4	2E-210	0.5	Flrt2	2E-41	0.5
Kctd12b	4E-10	0.5	Ccdc102a	1E-39	0.5
Vash2	9E-09	0.5	Gprasp2	1E-07	0.5
Gm20559	6E-29	0.5	Pamr1	1E-06	0.5
Slc38a4	1E-20	0.5	Arhgap29	3E-76	0.5
Lmx1b	3E-10	0.5	Sipa1l1	2E-18	0.5
Tubb2b	1E-64	0.5	Ptges	6E-15	0.5
Hdac11	1E-16	0.5	Lamb2	1E-35	0.5
Mvd	2E-21	0.5	Zfp991	1E-05	0.5
Spryd7	2E-38	0.5	Palld	5E-81	0.5
Tcf7l2	4E-108	0.5	Blcap	3E-15	0.5
Pamr1	2E-17	0.5	Gca	2E-07	0.5
Lsp1	7E-41	0.5	Lsp1	7E-22	0.5
Dram1	7E-41	0.5	Ap2a2	2E-70	0.5
Nfatc4	1E-55	0.4	Fndc4	5E-19	0.5
Msx1	2E-10	0.4	Unc93b1	1E-29	0.5
Hspa12b	2E-15	0.4	Cpeb2	1E-13	0.5
Sh3pxd2b	1E-67	0.4	Tnc	1E-16	0.5
Fndc4	4E-32	0.4	Vsir	3E-08	0.5
Pabpc4l	2E-23	0.4	Limk1	2E-06	0.5
Cyb5a	1E-262	0.4	Gpc6	5E-71	0.5
Gbp7	4E-19	0.4	Thbs3	2E-13	0.5
Gm9801	3E-15	0.4	Cebpa	5E-27	0.5
Cmtm3	4E-65	0.4	Stat2	3E-23	0.5
Srgap1	9E-67	0.4	Psd4	1E-07	0.5
Tap2	5E-47	0.4	Tacc1	1E-56	0.5
Ctso	4E-31	0.4	Spryd7	8E-20	0.5
Limk1	1E-11	0.4	Fxyd1	3E-84	0.5
Tmem150a	4E-48	0.4	Il6st	8E-101	0.4
Hmgcs1	2E-88	0.4	Gm30025	5E-10	0.4
Nxn	1E-41	0.4	Tlr3	5E-10	0.4
Ap2a2	1E-117	0.4	Maged2	3E-63	0.4
Ier3	1E-59	0.4	Gprc5b	1E-04	0.4
Hebp1	3E-74	0.4	Mid2	2E-08	0.4

Fibroblast?					
Control			NICD2		
Gene	*p*	FC	Gene	*p*	FC
Dhrs13	7E-76	1.4	Miip	9E-62	1.3
Tagap1	6E-44	1.4	Cspg4	2E-109	1.3
Klhdc8a	1E-66	1.4	Fxn	4E-120	1.3
Tmem121	4E-91	1.4	Csrp1	8E-130	1.3
Dclre1b	4E-40	1.4	Pfas	1E-79	1.3
Tubd1	1E-50	1.3	Hmgn5	5E-117	1.3
Gm12610	1E-44	1.3	2700049A03Rik	1E-104	1.3
Ran	2E-296	1.3	Gltp	6E-128	1.3
Gm14280	3E-18	1.3	Cdkn2aipnl	1E-96	1.3
Nup205	2E-101	1.3	Fzr1	1E-104	1.3
Rassf7	2E-46	1.3	Tcfl5	2E-70	1.3
Cep135	2E-67	1.3	Paxip1	1E-110	1.3
Nostrin	6E-30	1.3	Dars2	9E-80	1.3
Gm15246	2E-35	1.3	Mre11a	3E-120	1.3
Acot7	1E-85	1.3	Usp2	5E-86	1.3
Stk10	1E-45	1.3	Rab15	5E-49	1.3
Gdf15	2E-39	1.3	Nsmce4a	2E-190	1.3
Lrrc40	4E-78	1.3	Dynlt1a	3E-71	1.3
Ctnna2	2E-18	1.3	Ezr	1E-95	1.3
4930503L19Rik	8E-86	1.3	Gins4	9E-143	1.3
Rnaseh2a	4E-70	1.3	Ccdc82	6E-113	1.3
Mcph1	7E-96	1.3	Cntrl	9E-103	1.3
Snrpa1	2E-119	1.3	Prex2	5E-33	1.3
Prkar2a	5E-98	1.3	Prpf4	2E-106	1.3
Itpripl1	5E-42	1.3	Spink2	1E-44	1.3
Taf5	6E-74	1.3	Cse1l	2E-144	1.3
Ccnd1	3E-86	1.3	Rpa3	9E-129	1.3
Cep70	1E-66	1.3	Atm	2E-111	1.3
Atp10a	2E-80	1.3	Uchl5	1E-143	1.3
E230016K23Rik	6E-31	1.3	Mcph1	3E-134	1.3
Gm16091	1E-44	1.3	Homer2	7E-54	1.3
Atm	2E-90	1.3	Ncaph2	2E-143	1.3
Crabp1	1E-18	1.3	Spidr	2E-145	1.3
Pml	4E-89	1.3	Xpo1	3E-160	1.3
Gm10827	1E-22	1.3	Cep57	4E-136	1.2
Lsm3	3E-165	1.3	Lrrc40	8E-97	1.2
Cdkn2aipnl	5E-76	1.3	Orc2	3E-122	1.2
1700025G04Rik	2E-106	1.3	Acta2	3E-66	1.2
Rif1	4E-99	1.3	Tubd1	2E-81	1.2
Bend6	9E-52	1.3	Ccdc77	2E-98	1.2
Nsmce4a	2E-126	1.3	Arl6ip6	6E-114	1.2
Gm43305	2E-18	1.3	Anp32e	3E-184	1.2
Cntrl	5E-89	1.3	Pdxp	1E-34	1.2
Igsf9b	1E-32	1.3	Rbbp8	7E-173	1.2
Prune2	1E-41	1.3	Pcgf5	7E-75	1.2
Pold2	1E-90	1.3	Gm16091	8E-55	1.2
Lsm8	3E-141	1.3	Rnf168	1E-138	1.2
Casp8ap2	4E-118	1.3	Alg13	8E-103	1.2
9530077C05Rik	1E-50	1.3	Fam72a	2E-35	1.2
Ezr	1E-78	1.3	Nes	1E-163	1.2
Fxn	5E-75	1.3	Taf5	1E-77	1.2
Cd80	9E-70	1.3	Cd80	4E-64	1.2
3300005D01Rik	3E-23	1.3	Pim1	1E-56	1.2
Mre11a	4E-86	1.3	Nup133	2E-95	1.2
Ctc1	8E-87	1.3	Rad51b	2E-204	1.2
Cpa6	2E-33	1.3	Larp7	2E-150	1.2
Poln	7E-40	1.3	Ipp	2E-59	1.2
Ass1	3E-62	1.3	Snrpa1	6E-167	1.2
Tmem37	5E-59	1.3	Snrpd1	0E+00	1.2
Cep89	7E-44	1.3	Ngf	1E-53	1.2
Pgp	6E-165	1.3	Kank3	2E-32	1.2
Nup35	2E-105	1.3	Rnf227	2E-71	1.2
Dynlt1a	3E-62	1.3	Cklf	1E-105	1.2
Uchl5	1E-106	1.3	Ctnna2	1E-28	1.2
Xrcc1	1E-91	1.3	Fhdc1	1E-34	1.2
Slc25a40	5E-80	1.3	Trim37	1E-123	1.2
Pdxp	2E-29	1.3	Ranbp1	2E-300	1.2
Scrib	3E-63	1.3	Fbxo45	1E-59	1.2
Hmgn5	5E-63	1.3	Gm35330	4E-37	1.2
Ahcy	2E-34	1.3	Gemin2	3E-103	1.2
Nptxr	1E-66	1.3	Svop	3E-28	1.2
Ssrp1	2E-178	1.3	Dynlt1f	3E-102	1.2
Xpo1	1E-119	1.2	Xrcc1	2E-113	1.2

Undefined					
Control			NICD2		
Gene	*p*	FC	Gene	*p*	FC
Txndc15	2E-12	0.2	Macrod1	4E-07	0.2
Clu	2E-12	0.2	Ttll4	1E-01	0.2
Slc35a5	5E-01	0.2	Nmnat3	2E-01	0.2
Tmco4	3E-02	0.2	Ttc1	2E-02	0.2
Dusp18	6E-02	0.2	Ip6k2	2E-02	0.2
Garem1	7E-03	0.2	St6galnac4	3E-02	0.2
Ttc12	6E-01	0.2	Zfp408	4E-01	0.2
Ccdc28b	6E-02	0.2	Isoc2b	2E-01	0.2
Sgcb	4E-06	0.2	Kat8	2E-01	0.2
D630045J12Rik	1E-05	0.2	2700081O15Rik	2E-02	0.2
Ankrd9	8E-01	0.2	Tmem131l	6E-01	0.2
Hmcn1	2E-01	0.2	Nupr1	3E-16	0.2
Serpina3n	3E-02	0.2	Lrrc49	1E-01	0.2
Atg2a	3E-01	0.2	Pvt1	5E-09	0.2
Anxa4	2E-10	0.2	Slc46a1	6E-01	0.2
Plekha8	2E-01	0.2	Speg	1E-01	0.2
Ing4	8E-04	0.2	B4galt7	6E-01	0.2
Slc27a1	2E-02	0.2	Map9	8E-01	0.2
Angptl6	2E-04	0.2	Anks1	9E-01	0.2
Dipk1b	4E-04	0.2	Plat	3E-02	0.2
Hipk2	1E-07	0.2	Ccnd2	2E-22	0.2
Il15ra	3E-01	0.2	Ncoa3	2E-02	0.2
Mpped2	5E-03	0.2	Klc4	7E-01	0.2
Mynn	8E-01	0.2	Gm11084	2E-01	0.2
Atp6v1g1	4E-28	0.2	Gabarapl2	7E-10	0.2
Zfp30	9E-02	0.2	Wdr55	4E-01	0.2
Bloc1s3	8E-01	0.2	Eaf2	3E-01	0.2
Mt1	2E-12	0.2	Ttc23	8E-03	0.2
Tmc6	6E-01	0.2	Unc119	2E-01	0.2
Prdm16	5E-04	0.2	Dipk1b	2E-03	0.2
Slc25a37	8E-01	0.2	Efnb2	1E-01	0.2
Zfp775	5E-01	0.2	Lgals3	2E-16	0.2
Kremen1	3E-02	0.2	Armc9	6E-01	0.2
Fosl1	5E-01	0.2	Lrig2	5E-02	0.2
Pyroxd1	9E-03	0.2	Sgk1	3E-01	0.2
Ncoa3	4E-03	0.2	Tspyl1	3E-02	0.2
Mettl18	6E-01	0.2	Gm49959	5E-01	0.2
Emc1	5E-02	0.2	Ankrd28	2E-05	0.2
Psmd8	2E-13	0.2	Tut1	8E-01	0.2
Smtn	7E-03	0.2	Comp	4E-19	0.2
Mrm2	6E-01	0.2	A730081D07Rik	7E-01	0.2
Ero1l	2E-04	0.2	Bach2	6E-01	0.2
Trib3	2E-02	0.2	Gm19522	7E-01	0.2
Naprt	2E-01	0.2	Aqp5	3E-01	0.2
Prelid2	2E-02	0.2	Lemd2	8E-02	0.2
Pacsin3	1E+00	0.2	Cd82	4E-01	0.2
Zfp113	1E-01	0.2	Idnk	3E-03	0.2
Btbd19	8E-01	0.2	Hes6	9E-01	0.2
Rab36	9E-01	0.2	Mpg	3E-01	0.2
Cyld	2E-04	0.2	Gm15563	3E-01	0.2
Lca5l	6E-01	0.2	Kif3c	4E-01	0.2
Ccdc97	8E-01	0.2	Zfp580	7E-01	0.2
Rab39b	7E-01	0.2	Sparcl1	2E-01	0.2
Ifit2	8E-01	0.2	Ttc27	5E-01	0.2
Setd6	2E-01	0.2	Klhl8	1E+00	0.2
Emp2	1E-04	0.2	Pfkl	4E-03	0.2
Hdac8	5E-05	0.2	Mks1	9E-01	0.2
Dip2b	4E-07	0.2	Usf1	3E-01	0.2
Nrip2	3E-01	0.2	Cryzl2	1E-01	0.2
Socs2	4E-01	0.2	Eif4e3	8E-01	0.2
Sema3e	7E-03	0.2	Clba1	9E-02	0.2
Gdf15	4E-03	0.2	Proser1	3E-01	0.2
Jmjd8	3E-01	0.2	Zfp688	8E-01	0.2
Os9	6E-19	0.2	Tgfa	2E-01	0.2
Foxp2	4E-09	0.2	Cmtm4	1E-01	0.2
Mar9	7E-01	0.2	Ogfod2	2E-01	0.2
Ctsf	4E-06	0.2	Cops7b	4E-02	0.2
5430416N02Rik	3E-01	0.2	Trim24	1E-02	0.2
Rab6b	7E-01	0.2	Bphl	2E-03	0.2
C430049B03Rik	3E-04	0.2	Pyroxd1	8E-01	0.2
Tmem151a	4E-01	0.2	Snhg16	6E-01	0.2
Zswim1	8E-01	0.2	Srxn1	2E-02	0.2
Ccpg1os	6E-02	0.2	Zfp14	2E-01	0.2

Articular/Synovial Fibroblast					
Control			NICD2		
Gene	*p*	FC	Gene	*p*	FC
Arhgap39	3E-02	0.6	Dkk3	5E-27	0.6
G6pdx	3E-11	0.6	Ptgfrn	1E-03	0.6
C1ra	3E-27	0.6	Adcy9	1E-02	0.6
Snn	8E-02	0.6	Fahd2a	3E-01	0.6
Dcxr	1E-11	0.6	Arhgap39	1E-01	0.6
Uaca	2E-04	0.6	Polm	5E-01	0.6
Itm2b	3E-71	0.6	Echdc3	1E-01	0.6
Nudt4	4E-34	0.6	Tle2	2E-03	0.6
Slc24a3	1E-12	0.6	Aldh3b1	3E-03	0.6
A330076H08Rik	3E-02	0.6	Nrg1	7E-01	0.5
Igsf8	4E-10	0.6	Grn	3E-35	0.5
Rnf150	2E-12	0.6	Fam78b	2E-03	0.5
Fam13c	2E-17	0.6	Parp9	1E-03	0.5
Ifit3	5E-11	0.6	Gstk1	1E-02	0.5
Akap6	3E-03	0.6	Por	4E-06	0.5
Phldb1	6E-05	0.6	Lin37	5E-03	0.5
Gm20300	3E-01	0.6	Fpgt	3E-01	0.5
Cnrip1	8E-07	0.6	Dag1	6E-22	0.5
Filip1l	6E-09	0.6	Tcaf2	1E-01	0.5
Lefty1	3E-03	0.6	Sntb2	7E-25	0.5
Cited2	2E-28	0.6	Zbtb4	4E-04	0.5
Magi2	3E-32	0.6	Epas1	4E-03	0.5
Parp12	8E-03	0.6	Pnrc1	8E-28	0.5
Junos	2E-01	0.6	Reps2	4E-02	0.5
Epas1	6E-02	0.6	Lgr4	5E-05	0.5
Lpgat1	2E-18	0.6	Abcd2	1E-01	0.5
Agtrap	3E-07	0.6	Agpat3	2E-10	0.5
Zfyve26	1E-03	0.6	Rhobtb1	1E-03	0.5
Il17d	7E-03	0.6	Ypel3	1E-17	0.5
Zbtb4	4E-07	0.5	Kdm7a	3E-05	0.5
Twist2	3E-02	0.5	Prxl2b	4E-13	0.5
Lgals3bp	4E-20	0.5	Sphk1	1E-01	0.5
Pfkfb4	4E-02	0.5	Il1rl1	9E-02	0.5
Fhit	2E-06	0.5	Klhl24	2E-07	0.5
Rnh1	5E-34	0.5	Cited2	1E-11	0.5
Vwa5a	4E-11	0.5	Enah	4E-11	0.5
Sugct	2E-05	0.5	Arrdc3	6E-02	0.5
Tap2	4E-07	0.5	Bid	5E-01	0.5
Plekhn1	7E-02	0.5	Dennd2a	1E-02	0.5
Cpeb3	1E-05	0.5	Cryz	2E-03	0.5
Il1rl1	8E-06	0.5	Nlrc5	3E-01	0.5
Ppp1r14b	2E-28	0.5	Klhl36	4E-01	0.5
Capn1	2E-04	0.5	Mterf4	6E-02	0.5
Caprin2	9E-02	0.5	Gbp3	7E-02	0.5
Edem2	5E-04	0.5	Ifih1	3E-03	0.5
Dnah7b	3E-03	0.5	Vill	2E-01	0.5
1700088E04Rik	5E-02	0.5	Ndufa4l2	5E-02	0.5
Tprkb	5E-09	0.5	Glrx	3E-03	0.5
Fdxr	1E-02	0.5	Sqor	2E-03	0.5
Gdpd1	5E-01	0.5	Sfxn5	2E-02	0.5
Flcn	1E-01	0.5	Oasl2	2E-01	0.5
Tctn1	7E-01	0.5	Stat2	7E-04	0.5
Has2os	4E-02	0.5	Mfsd8	6E-01	0.5
Vegfd	2E-18	0.5	4930599N23Rik	1E-01	0.5
Lrrc73	4E-01	0.5	Gbp7	5E-03	0.5
Psap	5E-52	0.5	Maml3	6E-04	0.5
Clip4	3E-04	0.5	Ank3	2E-12	0.5
Arhgap21	2E-11	0.5	Psrc1	5E-02	0.5
Svil	2E-17	0.5	Gm9801	1E-01	0.5
Mamstr	6E-01	0.5	C330018D20Rik	3E-01	0.5
H2-T22	3E-07	0.5	Tstd3	2E-02	0.5
Nqo2	5E-02	0.5	Prr16	1E-06	0.5
Galnt18	2E-05	0.5	Ucp2	4E-03	0.5
Flnc	1E-09	0.5	Bst2	3E-22	0.5
Birc3	1E-01	0.5	Rtl8a	3E-06	0.5
Sqor	1E-04	0.5	Crip1	4E-22	0.5
Zmiz1	5E-19	0.5	Plekha5	2E-02	0.5
Pi4kb	4E-05	0.5	A330023F24Rik	3E-01	0.5
Scara5	3E-01	0.5	Pim1	2E-01	0.5
Mgst1	7E-54	0.5	Crebrf	5E-11	0.5
Agpat3	2E-14	0.5	Rab40b	1E-03	0.5
Efs	2E-05	0.5	Gabarapl1	5E-07	0.5
Pcdhga12	4E-01	0.5	Mblac2	1E-01	0.5

Limb Mesenchyme					
Control			NICD2		
Gene	p	FC	Gene	p	FC
Mtfr1	5E-15	0.4	Acp6	7E-09	0.4
Mars	3E-17	0.4	Tmtc4	5E-13	0.4
Gls	5E-34	0.4	Dab2	3E-42	0.4
Tll1	2E-05	0.4	Hist1h2ac	3E-03	0.4
Srm	1E-23	0.4	Id1	6E-19	0.4
Srpx	2E-15	0.4	Mfsd4a	2E-07	0.4
Dlg2	3E-07	0.4	Msrb3	1E-22	0.4
Ndufaf5	2E-10	0.4	Endov	2E-07	0.4
Mpp6	2E-29	0.4	Akirin2	1E-29	0.4
Vta1	1E-23	0.4	Gulp1	5E-34	0.4
Hax1	2E-27	0.4	Ddx21	6E-25	0.4
Smad9	2E-05	0.4	Ccdc134	6E-03	0.4
Myo1d	4E-29	0.4	Gm35330	1E-05	0.4
Gm48765	1E-07	0.4	Ttc39b	1E-15	0.4
Cenpp	2E-36	0.4	Gm48742	5E-10	0.4
Eif4g2	2E-85	0.4	Smc6	3E-32	0.4
Elovl4	7E-05	0.4	Slc7a11	1E-08	0.4
Ghitm	2E-49	0.4	Ciapin1	4E-13	0.4
Brd7	4E-35	0.4	Ice2	1E-09	0.4
Slc33a1	1E-14	0.4	Apobr	2E-05	0.4
Ehd3	6E-12	0.4	Odc1	1E-23	0.4
Rxfp1	2E-03	0.4	Macrod2	1E-06	0.4
Zfp647	8E-05	0.4	Phyhd1	3E-21	0.4
Tgfb3	1E-15	0.4	Hoxa9	8E-21	0.4
Mthfd1l	3E-13	0.4	Rundc1	5E-04	0.4
Lrba	5E-23	0.4	Msantd3	4E-11	0.4
4930556J24Rik	8E-05	0.4	Neo1	1E-22	0.4
Samd12	6E-07	0.4	Mtfr1	7E-15	0.4
Trim35	2E-32	0.4	Abca2	6E-06	0.4
Zzef1	5E-19	0.4	Gata2	7E-05	0.4
Hltf	2E-14	0.4	Senp3	3E-11	0.4
Sdr39u1	1E-12	0.4	Ccn4	4E-27	0.4
Mex3d	9E-13	0.4	Net1	5E-08	0.4
Taf15	1E-35	0.4	Myc	3E-05	0.4
Col5a2	4E-63	0.4	Eif4g2	1E-77	0.4
Zfp518b	7E-10	0.4	Aars	2E-24	0.4
Flna	5E-28	0.4	Hspa5	2E-59	0.4
Leprotl1	9E-33	0.4	Foxd2os	2E-14	0.4
Dnajc16	1E-08	0.4	Fhl1	7E-19	0.4
Gm44777	1E-05	0.4	Gfpt1	1E-24	0.4
Zfp131	2E-20	0.4	Ctdspl	3E-21	0.4
Tcf4	3E-60	0.4	Tfb1m	2E-05	0.4
Abhd17b	1E-16	0.4	Abhd13	1E-06	0.4
Chka	2E-11	0.4	Fyn	3E-14	0.4
Amotl1	1E-19	0.4	Piezo2	2E-23	0.4
Gm12153	4E-06	0.4	Frmd6	2E-19	0.4
Ctps	1E-20	0.4	Dpysl3	2E-13	0.4
Asap1	1E-33	0.4	Pcdhb22	3E-04	0.4
Gm15050	1E-07	0.4	Hoxc4	4E-10	0.4
Akap9	1E-29	0.4	Qtrt2	1E-07	0.4
E530011L22Rik	3E-09	0.4	Nf2	2E-10	0.4
Cdc42ep1	9E-08	0.4	Gm49463	2E-03	0.4
Gm39214	7E-04	0.4	Atp1a1	3E-24	0.4
Eya2	2E-05	0.4	Ddit3	3E-23	0.4
Herpud2	8E-16	0.4	Gm26930	1E-05	0.4
Sgk3	3E-13	0.4	Nfe2l1	4E-43	0.4
Hspbap1	3E-10	0.4	Atp10a	6E-11	0.4
Ide	8E-24	0.4	Ptpn21	9E-11	0.4
Rab3b	3E-06	0.4	Fam110b	2E-16	0.4
Gm26759	4E-07	0.4	Nkd1	6E-10	0.4
Dgke	5E-07	0.4	Gm32618	8E-08	0.4
Tpk1	5E-16	0.4	Wasf1	6E-09	0.4
Rhbdd1	6E-17	0.4	Galnt5	4E-03	0.4
Pinx1	7E-12	0.4	Gm26911	1E-04	0.4
Stk26	7E-20	0.4	4930533K18Rik	2E-04	0.4
Poli	1E-06	0.4	Mex3b	3E-05	0.4
Lims1	5E-38	0.4	Slc12a7	2E-05	0.4
Ern1	5E-13	0.4	Ptk7	8E-16	0.4
Sh3bp4	4E-07	0.4	Fign	9E-19	0.4
Rhobtb2	5E-07	0.4	Gm17227	8E-04	0.4
Cbx4	6E-11	0.4	Tgfbr2	7E-24	0.4
D16Ertd472e	2E-09	0.4	Ppp2r5a	1E-19	0.4
Ppp2r5b	2E-06	0.4	Zfp354c	2E-14	0.4

Chondrogenic					
Control			NICD2		
Gene	p	FC	Gene	p	FC
Tfrc	7E-05	0.5	Mtln	1E-29	0.5
Sim2	8E-05	0.5	Tubg2	3E-02	0.5
Tjp1	8E-29	0.5	Nomo1	5E-12	0.5
Hoxc4	8E-09	0.5	Spa17	1E-03	0.5
Mthfr	1E-07	0.5	Zzef1	2E-16	0.5
Aldh6a1	3E-15	0.5	Cyp26b1	6E-04	0.5
Dlc1	3E-60	0.5	Rnd2	9E-05	0.5
Slc25a33	3E-05	0.5	Nipsnap1	8E-07	0.5
Dusp14	3E-03	0.5	Lrrc8c	1E-03	0.5
3110021N24Rik	3E-11	0.5	Firre	5E-04	0.5
Rundc1	1E-06	0.5	Gmds	7E-52	0.5
4930594M22Rik	1E-04	0.5	Frmd6	5E-25	0.5
Gm42984	1E-03	0.5	N4bp2l1	3E-04	0.5
Pdrg1	7E-40	0.5	Ogfod3	2E-09	0.5
Tnrc6b	1E-68	0.5	Sesn3	9E-24	0.5
C030006K11Rik	5E-06	0.5	Prmt6	2E-01	0.5
Ptpn14	3E-28	0.5	Gm11266	1E-04	0.5
Ltbp1	5E-30	0.5	Plcd1	2E-09	0.5
Fbxo7	2E-13	0.5	Dlc1	4E-44	0.5
Angpt1	7E-18	0.5	Slc22a5	1E-03	0.5
Fam20b	1E-14	0.5	Syne1	7E-32	0.5
Bpgm	4E-06	0.5	2010001A14Rik	1E-03	0.5
Irs2	1E-06	0.5	Slc25a27	3E-03	0.5
Lonrf1	1E-07	0.5	Rin1	6E-03	0.5
Npnt	2E-04	0.5	Jade2	2E-05	0.5
Tex14	2E-17	0.5	Mafb	3E-09	0.5
Clba1	4E-07	0.5	Asrgl1	6E-07	0.5
Nipsnap1	2E-08	0.5	Wdyhv1	2E-03	0.5
Jade2	1E-04	0.5	Mamld1	7E-05	0.5
Gng11	4E-08	0.5	Tmem117	2E-02	0.5
Arl10	2E-07	0.5	Eps8	9E-14	0.5
Firre	5E-06	0.5	2900005J15Rik	3E-02	0.5
Zpbp	3E-03	0.5	Sdc3	1E-10	0.5
Sdc4	2E-64	0.5	3110056K07Rik	4E-05	0.5
Ttll7	2E-08	0.5	Hipk2	4E-22	0.5
Selenos	9E-103	0.5	Clcn3	5E-08	0.5
Gm20536	2E-04	0.5	Rdh10	3E-04	0.5
Aplp2	2E-83	0.5	Mkx	2E-10	0.5
Sdc2	2E-65	0.5	Tmem42	5E-10	0.5
Krba1	9E-05	0.5	Fap	7E-04	0.5
Hist1h1c	3E-27	0.5	Trps1	1E-57	0.5
Ide	2E-28	0.5	Yipf2	3E-05	0.5
Sgms2	6E-12	0.5	Klf12	3E-16	0.5
Vamp1	5E-03	0.5	Hoxd9	2E-02	0.5
Shank1	1E-02	0.5	6430590A07Rik	5E-04	0.5
Arv1	7E-05	0.5	Slc41a2	4E-08	0.5
Tm4sf1	3E-17	0.5	E130102H24Rik	2E-08	0.5
Ddr1	4E-07	0.5	Mthfr	1E-06	0.5
Tmem63a	1E-11	0.5	Sat1	2E-24	0.5
Sil1	3E-33	0.5	Rnaset2b	7E-06	0.5
Ifit3	5E-10	0.5	Ormdl2	2E-07	0.5
Plagl1	4E-05	0.5	Mettl25	6E-06	0.5
Crlf3	5E-05	0.5	Gm47167	2E-03	0.5
Ift57	3E-15	0.5	4930523C07Rik	3E-11	0.5
Gmds	4E-61	0.5	Slc20a1	1E-01	0.5
Abi2	2E-13	0.5	Pgrmc2	5E-39	0.5
Fam20c	3E-03	0.5	Pag1	7E-03	0.5
Nim1k	3E-07	0.5	2810457G06Rik	8E-06	0.5
Hist2h3c2	6E-04	0.5	Zfp239	5E-05	0.5
Spred1	2E-24	0.5	Ikzf4	9E-04	0.5
Caskin2	1E-03	0.5	Zhx2	3E-07	0.5
Nedd4l	1E-12	0.5	Gm15834	2E-03	0.5
Abat	4E-11	0.5	Mipep	4E-03	0.5
Ifit3b	2E-05	0.5	Abi2	4E-12	0.5
Gnasas1	2E-07	0.5	St3gal4	2E-11	0.5
Rdm1	8E-12	0.5	Kmt2e	2E-28	0.5
Zfp637	3E-32	0.5	Patj	2E-05	0.5
Tk2	2E-05	0.5	Rsph3a	1E-03	0.5
Nhs	7E-16	0.5	Gmpr	1E-03	0.5
Wdr27	3E-04	0.5	Pacrg	2E-02	0.5
3110056K07Rik	2E-09	0.5	Slc43a2	9E-03	0.5
Gm15563	2E-05	0.5	Zfp516	6E-16	0.5
Hyou1	9E-17	0.5	Morn4	9E-03	0.5

Fibroblast					
Control			NICD2		
Gene	p	FC	Gene	p	FC
Maob	2E-19	0.4	Pcdh7	2E-64	0.4
2900089D17Rik	3E-08	0.4	Agtr2	1E-09	0.4
Rnf150	1E-61	0.4	Zeb2	8E-98	0.4
Btbd3	9E-38	0.4	Chsy3	5E-13	0.4
Pxylp1	6E-11	0.4	Dram1	8E-19	0.4
B4galnt1	3E-09	0.4	Zfp937	2E-05	0.4
Pcp4l1	3E-55	0.4	Sh3pxd2b	4E-37	0.4
Rab9	1E-134	0.4	Hdac7	5E-25	0.4
Zfp937	9E-10	0.4	Slc38a6	2E-12	0.4
Glis1	6E-32	0.4	Myadm	6E-33	0.4
Chsy3	1E-17	0.4	Gucy1a2	5E-09	0.4
Fmnl2	3E-100	0.4	Ifi27l2a	1E-37	0.4
Fdps	1E-66	0.4	Fam149a	1E-16	0.4
Sh3rf3	4E-69	0.4	Eef2k	3E-20	0.4
Dmpk	2E-39	0.4	Magi1	2E-16	0.4
Lhfp	2E-159	0.4	Tnfsf11	1E-04	0.4
Tgfb1i1	5E-83	0.4	Rab9	6E-66	0.4
Fam149a	3E-27	0.4	Dnajb3	3E-06	0.4
Abcb6	2E-07	0.4	Gab3	8E-05	0.4
Gm30025	3E-21	0.4	Ssc5d	6E-29	0.4
Ydjc	2E-19	0.4	Gla	1E-11	0.4
Gprasp2	2E-14	0.4	Nrbp2	2E-07	0.4
Ankrd55	1E-12	0.4	Lima1	8E-77	0.4
Islr	2E-114	0.4	Enox1	3E-29	0.4
Enox1	9E-48	0.4	Nexn	3E-11	0.4
Fxyd1	1E-144	0.4	Ndn	2E-47	0.4
Ano6	8E-106	0.4	Cdh11	2E-121	0.4
Arhgap24	1E-124	0.4	Ccl2	3E-27	0.4
Hs6st1	4E-14	0.4	Srgap1	8E-30	0.4
Pir	3E-40	0.4	Mrps6	2E-35	0.4
Rassf8	1E-22	0.4	Ppl	1E-18	0.4
Prr16	2E-58	0.4	Npc2	4E-180	0.4
Zfp503	3E-111	0.4	Uprt	2E-05	0.4
Cpxm2	2E-14	0.4	Zfp618	9E-13	0.4
Pdgfrb	6E-85	0.4	Ptk7	2E-19	0.4
A330076H08Rik	4E-18	0.4	Pcdhgc3	6E-13	0.4
Zfp449	2E-19	0.4	Gadd45b	3E-19	0.4
Dyrk2	2E-23	0.4	Ydjc	1E-09	0.4
Ank2	2E-107	0.4	L3hypdh	7E-13	0.4
Acat2	2E-27	0.4	Ubtd2	1E-35	0.4
Gpr173	2E-27	0.4	Pik3c2a	4E-34	0.4
Erg28	3E-96	0.4	Mylk	3E-03	0.4
Rhoj	7E-77	0.4	Arhgap24	4E-70	0.4
Cyp51	3E-71	0.4	Adamts4	4E-05	0.4
Pdlim7	4E-70	0.4	Galnt18	2E-11	0.4
Dipk1a	1E-17	0.4	Igtp	4E-07	0.4
Zfp991	3E-08	0.4	Plod3	6E-66	0.4
Lima1	1E-149	0.4	Amer1	8E-14	0.4
Npc2	0E+00	0.4	Ramp1	7E-06	0.4
Dock1	4E-87	0.4	Slco2a1	1E-01	0.4
Slc22a17	1E-21	0.4	Filip1l	8E-24	0.4
Col27a1	7E-87	0.4	Lipa	2E-55	0.4
Pdk3	1E-60	0.4	Trerf1	8E-26	0.4
Gm28875	2E-17	0.4	Pcp4l1	9E-23	0.4
Klf2	7E-16	0.4	Ssbp2	3E-29	0.4
Hdac7	1E-42	0.4	Hebp1	1E-34	0.4
Rab3il1	2E-20	0.4	Islr	2E-53	0.4
Htra3	8E-46	0.4	Gpsm1	1E-08	0.4
Junb	1E-108	0.4	Cyb5a	5E-118	0.4
Cers6	1E-52	0.4	Pdpn	1E-44	0.4
Npas2	4E-09	0.4	Nrip1	2E-62	0.4
Msmo1	4E-55	0.4	Alms1	3E-11	0.4
Nsdhl	3E-32	0.4	Hs6st1	5E-08	0.4
Tmem168	3E-26	0.4	Spink10	1E-03	0.4
Gpc6	1E-115	0.4	Bst2	3E-77	0.4
Sigmar1	1E-66	0.4	Kcnab1	1E-11	0.4
Thbs3	1E-20	0.4	Camk2n1	1E-10	0.4
Ssbp2	1E-60	0.4	Gm4117	7E-08	0.4
Wwp1	1E-68	0.4	Ctso	1E-15	0.4
Sugct	3E-30	0.4	Ank2	4E-53	0.4
Prkcd	5E-23	0.4	Rassf8	2E-11	0.4
Txnrd3	3E-10	0.4	Smpdl3a	2E-48	0.4
Fras1	7E-06	0.4	Deptor	3E-08	0.4

Fibroblast?					
Control			NICD2		
Gene	p	FC	Gene	p	FC
Paxip1	6E-66	1.2	Ranbp17	9E-82	1.2
Rnf227	1E-58	1.2	Mbd4	2E-59	1.2
Slc25a10	1E-89	1.2	Brip1os	2E-133	1.2
Rbbp8	1E-121	1.2	Gstm6	1E-14	1.2
Inka2	6E-58	1.2	Cmklr1	1E-33	1.2
Anp32e	5E-139	1.2	Pml	1E-93	1.2
Pycard	2E-57	1.2	2700038G22Rik	3E-39	1.2
Gm35330	2E-32	1.2	Lyar	2E-137	1.2
Gltp	3E-84	1.2	Pkp4	1E-112	1.2
Chn1	3E-25	1.2	Mical2	6E-67	1.2
Zfp41	5E-45	1.2	Pcyox1l	5E-79	1.2
Proser3	3E-47	1.2	Recql	2E-98	1.2
Csel1	2E-92	1.2	Rflnb	1E-109	1.2
Hist2h2aa1	3E-42	1.2	Nup35	4E-130	1.2
Rnaseh2b	1E-84	1.2	Platr25	2E-95	1.2
Recql	2E-67	1.2	Rfc1	1E-188	1.2
Afmid	2E-54	1.2	Proser3	6E-58	1.2
Ncaph2	2E-102	1.2	Gm41609	7E-31	1.2
Rnf168	2E-99	1.2	Eif1ad	1E-137	1.2
Mphosph9	1E-74	1.2	Igsf9b	8E-33	1.2
Met	8E-45	1.2	Scrib	3E-80	1.2
Gm41609	9E-21	1.2	Ipo9	3E-108	1.2
Trim37	1E-96	1.2	Zfp41	2E-57	1.2
Panx1	4E-37	1.2	Tex15	5E-53	1.2
Tex15	2E-50	1.2	Samd1	1E-79	1.2
Gm12811	9E-53	1.2	Cdkn2c	2E-131	1.2
Cep57	9E-94	1.2	Lrrc8c	4E-100	1.2
Osbpl3	3E-59	1.2	Cep89	4E-55	1.2
Larp7	3E-100	1.2	Nudc	4E-293	1.2
Gm26787	6E-13	1.2	Slc6a17	2E-43	1.2
Pxmp2	2E-61	1.2	Sae1	9E-181	1.2
Nup62	1E-70	1.2	Samd12	5E-40	1.2
Ccdc163	2E-46	1.2	Ubr7	9E-121	1.2
Nup133	3E-77	1.2	Tubgcp2	3E-132	1.2
Ccdc138	1E-73	1.2	Cnn1	7E-28	1.2
Snrpd1	3E-225	1.2	Panx1	3E-51	1.2
Eda2r	2E-57	1.2	Fsd1l	1E-84	1.2
Efr3b	7E-15	1.2	Aplf	2E-49	1.2
Arpin	4E-75	1.2	Pycard	4E-68	1.2
Me2	9E-89	1.2	Prune2	8E-38	1.1
Spata7	6E-10	1.2	Srd5a1	2E-51	1.1
L3mbtl2	1E-53	1.2	Osbpl3	2E-49	1.1
Lmod1	1E-40	1.2	Galnt12	5E-26	1.1
Dcaf15	9E-44	1.2	Slc25a40	2E-77	1.1
Brip1os	7E-101	1.2	Senp1	7E-112	1.1
Fsd1l	3E-56	1.2	Coro1c	3E-96	1.1
Tmem129	9E-52	1.2	H2afv	2E-225	1.1
Sae1	1E-139	1.2	Cntln	2E-143	1.1
Thsd4	4E-31	1.2	Ttc9	2E-32	1.1
Lyar	9E-101	1.2	Mpp6	4E-121	1.1
Jade3	1E-14	1.2	Me2	2E-114	1.1
Cubn	4E-17	1.2	Mad1l1	5E-114	1.1
Cda	8E-48	1.2	Mpp2	3E-66	1.1
Pik3cb	3E-50	1.2	Zmynd19	4E-72	1.1
Ipo9	1E-83	1.2	Haus7	1E-96	1.1
Mbd4	2E-42	1.2	Tagln	3E-46	1.1
Pdlim1	2E-69	1.2	Hey1	7E-52	1.1
Hsp90aa1	4E-236	1.2	Tlnrd1	4E-78	1.1
Gask1b	2E-66	1.2	Dclre1b	1E-37	1.1
Nelfe	3E-95	1.2	Ccdc138	4E-80	1.1
Arl4c	4E-41	1.2	Helq	1E-56	1.1
Tubgcp6	7E-48	1.2	Stk10	2E-55	1.1
Cspg4	1E-76	1.2	Parp1	6E-111	1.1
Ranbp17	2E-64	1.2	Pms1	1E-72	1.1
Ccdc82	3E-75	1.2	Tmem100	4E-53	1.1
Apol9b	1E-22	1.2	9230114K14Rik	2E-102	1.1
Hspa14	2E-103	1.2	Spice1	2E-77	1.1
Rfx2	1E-20	1.2	Prkg2	3E-39	1.1
Spidr	5E-87	1.2	Cnot9	3E-102	1.1
Dusp8	2E-23	1.2	L3mbtl2	2E-60	1.1
Ipp	1E-34	1.2	Bmp6	5E-24	1.1
Nudt1	7E-58	1.2	Rad9a	6E-43	1.1
Lsm6	2E-160	1.1	Ass1	9E-48	1.1

Undefined					
Control			NICD2		
Gene	p	FC	Gene	p	FC
2700081O15Rik	1E-03	0.2	Jpx	5E-01	0.2
Timp2	4E-53	0.2	Kif3a	4E-03	0.2
Ppcs	4E-02	0.2	Zfp553	5E-01	0.2
Anxa8	9E-01	0.2	Flt3l	4E-01	0.2
Tlr2	1E-01	0.2	Baiap2l1	8E-01	0.2
Ccdc9b	1E+00	0.2	Tex14	4E-03	0.2
Efna4	3E-01	0.2	Gem	6E-03	0.2
Med31	4E-04	0.2	Fundc1	9E-03	0.2
Osbpl3	9E-01	0.2	Tceal1	4E-01	0.2
Pitpnc1	7E-04	0.2	Sqstm1	2E-05	0.2
Naaladl2	3E-20	0.2	Rpl36a-ps1	5E-01	0.2
Apbb2	3E-22	0.2	Zfp516	3E-05	0.2
Spin2c	9E-01	0.2	Vars2	4E-01	0.2
Gstm4	6E-01	0.2	Foxc1	8E-15	0.2
Marcksl1	3E-02	0.2	B9d1	3E-02	0.2
Lars2	3E-01	0.2	C4b	3E-06	0.2
Zbtb43	7E-02	0.2	Gtpbp4	5E-05	0.2
Mxd1	3E-01	0.2	Gmpr	7E-01	0.2
Robo2	9E-05	0.2	Gm42418	2E-14	0.2
Tbc1d7	6E-01	0.2	Rbak	5E-01	0.2
Fam107b	2E-01	0.2	Coil	8E-01	0.2
Rdh14	2E-02	0.2	Traf3ip1	8E-05	0.2
Exoc3	3E-13	0.2	Wdr75	9E-03	0.2
Pex11a	3E-02	0.2	Ero1l	1E-02	0.2
Hoxa9	2E-07	0.2	Tcn2	7E-08	0.2
Ctsl	8E-42	0.2	Borcs8	1E-02	0.2
Tfb2m	2E-03	0.2	Tacolos	3E-01	0.2
Zfp983	5E-01	0.2	Tmem223	1E-11	0.2
Antxr1	3E-07	0.2	Gnb5	9E-03	0.2
Snx6	5E-16	0.2	Capg	5E-13	0.2
Sqstm1	6E-08	0.2	Pam	3E-46	0.2
Mettl21a	2E-03	0.2	Dancr	3E-02	0.2
Tmem199	9E-02	0.2	Prdx2	2E-28	0.2
Mrpl38	2E-02	0.2	Slc11a2	2E-01	0.2
Ypel3	8E-12	0.2	Timp2	1E-46	0.2
Tpm2	3E-16	0.2	Col6a1	6E-20	0.2
Frmd6	3E-07	0.2	Etv5	1E-01	0.2
Tmem231	7E-01	0.2	Gm2a	7E-06	0.2
Hectd2	6E-01	0.2	2610021A01Rik	6E-01	0.2
Ogfod3	2E-01	0.2	Med31	5E-02	0.2
Fbxw9	9E-01	0.2	Rom1	9E-01	0.2
A930005H10Rik	1E-01	0.2	Ubxn6	3E-06	0.2
Gatc	1E-01	0.2	Pcolce	5E-33	0.2
Trim24	2E-03	0.2	Eya1	2E-02	0.2
1700088E04Rik	9E-01	0.2	Zyx	4E-01	0.2
Slc43a2	2E-01	0.2	Tmtc4	5E-01	0.2
Rab29	1E-01	0.2	Slc12a2	1E-02	0.2
Tpmt	5E-01	0.2	Arl3	4E-12	0.2
Gtpbp2	2E-01	0.2	Gm10125	8E-01	0.2
Dusp3	1E-02	0.2	Ccdc115	7E-01	0.2
Entpd7	2E-01	0.2	Sardh	2E-01	0.2
Phyhd1	2E-06	0.2	2610507I01Rik	6E-01	0.2
Zkscan17	9E-01	0.2	Hexb	7E-01	0.2
2310022B05Rik	2E-02	0.2	Calcoco1	8E-02	0.2
Zswim4	3E-01	0.2	Zfp141	6E-01	0.2
Gm11084	6E-01	0.2	S100a10	5E-13	0.2
Upf3b	4E-04	0.2	Ermp1	6E-01	0.2
Tmem185b	9E-01	0.2	Ap4s1	6E-03	0.2
Spcs3	3E-03	0.2	Prkx	8E-01	0.2
Hoxa7	1E-02	0.2	Naga	8E-03	0.2
B3gnt2	7E-03	0.2	Efna5	9E-08	0.2
Zfp955b	5E-01	0.2	Syne1	1E-06	0.2
Ifit1	7E-01	0.2	Zfp799	2E-01	0.2
Lemd2	4E-02	0.2	Sema3e	2E-03	0.2
Gm37768	1E-01	0.2	Isg15	6E-02	0.2
Ptpn3	7E-01	0.2	Coq8b	1E-01	0.2
Tmem265	3E-01	0.2	C030006K11Rik	3E-01	0.2
Tmed4	6E-04	0.2	Crebrf	4E-05	0.2
B9d1	2E-02	0.2	Sirt5	9E-01	0.2
Slc20a1	7E-01	0.2	Anxa2	7E-22	0.2
Aplp2	7E-26	0.2	Jade2	5E-01	0.2
Stxbp3	1E-02	0.2	Gpx1	2E-12	0.2
Ccndbp1	3E-03	0.2	Dnajc15	1E-08	0.2

Articular/Synovial Fibroblast					
Control			NICD2		
Gene	p	FC	Gene	p	FC
Atp13a2	5E-03	0.5	Gm11613	3E-01	0.5
Ano6	4E-16	0.5	Sat1	7E-06	0.5
Hk1	1E-11	0.5	Tifa	3E-01	0.5
Arhgap18	6E-08	0.5	Deptor	3E-05	0.5
Polm	2E-01	0.5	Pon3	1E-07	0.5
Akr1b8	3E-06	0.5	Zfp423	4E-01	0.5
Pik3r4	9E-04	0.5	Rnf31	2E-01	0.5
Ifi203	2E-01	0.5	Rab7b	1E-01	0.5
Pir	9E-07	0.5	Col4a2	3E-08	0.5
Ddit4l	3E-13	0.5	Ctsa	3E-19	0.5
Arl15	2E-17	0.5	Fam13c	4E-08	0.5
Mmp16	2E-06	0.5	Pik3r1	2E-12	0.5
Timp2	1E-80	0.5	Lgals3bp	4E-16	0.5
Slc9a1	5E-03	0.5	Pcmtd2	2E-04	0.5
Tatdn3	7E-02	0.5	Slc38a4	1E-01	0.5
Klf9	2E-12	0.5	Efs	8E-04	0.5
Ctps2	4E-06	0.5	Prkag2	2E-01	0.5
Mmp19	2E-05	0.5	Mmp11	4E-02	0.5
Gamt	2E-12	0.5	Cers4	4E-02	0.5
A330023F24Rik	3E-03	0.5	Gm46218	7E-02	0.5
Arhgap26	1E-01	0.5	Gm2a	4E-07	0.5
1700037C18Rik	6E-01	0.5	Hykk	2E-01	0.5
Ifi30	7E-17	0.5	Rxra	4E-03	0.5
Camk1d	7E-15	0.5	Inafm1	2E-01	0.5
Prxl2a	3E-06	0.5	Ndrg4	3E-01	0.5
Atp9a	3E-02	0.5	Ttc26	5E-01	0.5
Gm12905	6E-02	0.5	Snx2	9E-16	0.5
Nrp1	1E-12	0.5	Ntrk3	4E-03	0.5
Dennd2a	7E-03	0.5	Trib1	1E-02	0.5
Mmp11	2E-01	0.5	Abhd18	8E-03	0.5
Glt8d2	8E-03	0.5	Pfkfb2	3E-01	0.5
Etfbkmt	2E-01	0.5	Gng7	8E-02	0.5
Adm	4E-09	0.5	Orai3	2E-03	0.5
Pnpla7	3E-03	0.5	Pik3r2	4E-04	0.5
Hdac11	8E-02	0.5	Casp4	1E-01	0.5
Echdc3	1E-01	0.5	Slc38a7	2E-01	0.5
Itga2b	7E-02	0.5	Tnfsf12	4E-07	0.5
Sntb2	2E-26	0.5	Dglucy	4E-02	0.5
Tec	2E-02	0.5	Gm14410	9E-01	0.5
St5	5E-13	0.5	Mroh1	7E-03	0.5
Adam17	3E-07	0.5	Tle5	5E-36	0.5
Gpsm2	5E-11	0.5	Glt8d2	1E-01	0.5
Nudt7	7E-02	0.5	Urod	4E-04	0.5
Wdr81	2E-01	0.5	Slc41a3	3E-01	0.5
Cyp4f13	5E-03	0.5	Nmnat3	3E-02	0.5
Zfp518a	4E-03	0.5	Tle1	9E-03	0.5
C1qtnf2	1E-01	0.5	Gyg	7E-05	0.5
Prcp	8E-05	0.5	Itga2b	9E-02	0.5
Slc43a2	2E-01	0.5	Pde8a	2E-06	0.5
D130040H23Rik	4E-02	0.5	Olfml2b	9E-07	0.5
Cpt1a	5E-06	0.5	Irgm1	2E-01	0.5
Pnrc1	2E-31	0.5	Yipf2	4E-01	0.5
Lpin3	7E-02	0.5	Nptxr	5E-01	0.5
Tsku	7E-02	0.5	Tmem198b	4E-01	0.5
Sat1	2E-19	0.5	Ophn1	1E-05	0.5
Ddah2	1E-26	0.5	Gm45871	5E-01	0.5
Kctd12b	6E-01	0.5	Unc119b	1E-01	0.5
Pxn	8E-02	0.5	Ifitm3	2E-36	0.5
Fam151b	3E-01	0.5	Slc15a4	6E-01	0.5
Slc38a7	2E-02	0.5	Tyk2	2E-01	0.5
Vgll4	1E-10	0.5	Dhdh	2E-01	0.5
Pld2	7E-02	0.5	Spa17	7E-01	0.5
Dtnbp1	7E-30	0.5	Ctso	6E-02	0.5
Sesn1	2E-04	0.5	Zfp984	7E-02	0.5
Acvr1b	3E-01	0.5	Gm10373	2E-01	0.5
Mlkl	2E-02	0.5	Spsb1	2E-01	0.5
Fndc3a	3E-36	0.5	Fam126a	2E-01	0.5
Rab40b	2E-03	0.5	Ccdc171	3E-02	0.5
Stard9	4E-03	0.5	Ahrr	1E+00	0.5
Ndn	2E-14	0.5	Orai2	5E-01	0.5
Hoxd3os1	3E-01	0.5	Socs3	2E-01	0.5
Cd46	1E-02	0.5	Mknk2	9E-03	0.5
Paqr7	4E-01	0.5	Wdr35	8E-06	0.5

Limb Mesenchyme

Control Gene	p	FC	NICD2 Gene	p	FC
Slc16a4	2E-12	0.4	Arhgef18	5E-11	0.4
Fbxo42	4E-11	0.4	BC067074	3E-05	0.4
Brwd1	2E-21	0.4	Cars	1E-14	0.4
Ddx19a	6E-12	0.4	Prkab1	2E-09	0.4
Pemt	3E-05	0.4	Swap70	2E-11	0.4
Trpv4	6E-08	0.4	Pde10a	2E-14	0.4
Castor1	4E-09	0.4	Retreg1	1E-08	0.4
Gm35330	1E-07	0.4	P3h1	3E-19	0.4
Cpe	9E-45	0.4	Gas5	4E-49	0.4
Slc25a38	1E-11	0.4	Usp36	8E-11	0.4
N4bp2	4E-10	0.4	Insyn2a	3E-03	0.4
Dcaf12l1	9E-09	0.4	Tmem117	7E-03	0.4
Klf15	6E-08	0.4	Polr3e	2E-07	0.4
Nop58	3E-27	0.4	Shroom1	9E-06	0.4
Vars	2E-16	0.4	Sipa1l1	1E-20	0.4
Lhfp	1E-28	0.4	Lig3	1E-09	0.4
Fem1a	5E-07	0.4	Itpr1	8E-17	0.4
Coasy	1E-12	0.4	Met	2E-02	0.4
Msx1	2E-04	0.4	Ppip5k1	3E-04	0.4
Nol9	7E-13	0.4	Agbl5	2E-06	0.4
Prkci	5E-13	0.4	Cpe	5E-48	0.4
Wars	5E-11	0.4	Kif26b	2E-13	0.4
Cnnm4	8E-05	0.4	9130023H24Rik	8E-04	0.4
Klhl25	5E-04	0.4	Nmrk1	9E-09	0.4
Ctf1	2E-10	0.4	Pik3cb	1E-09	0.4
Sntb1	3E-21	0.4	Zfp212	2E-04	0.4
Myo1e	2E-32	0.4	Dhx37	4E-07	0.4
Ptpn21	8E-13	0.4	Akna	7E-03	0.4
Fbxl2	4E-13	0.4	Magi3	2E-12	0.4
Fndc3b	2E-51	0.4	Nomo1	2E-14	0.4
Gpsm1	7E-10	0.4	Arhgef2	2E-16	0.4
Nipsnap1	2E-10	0.4	Spint2	2E-05	0.4
Gab1	1E-16	0.4	Reck	6E-20	0.4
Dvl3	1E-12	0.4	Pck2	1E-23	0.4
Lcorl	5E-20	0.4	Maml1	5E-06	0.4
Gar1	2E-17	0.4	Aagab	1E-07	0.4
Fam131a	5E-07	0.4	Snd1	7E-27	0.4
Zfp354c	6E-17	0.4	Rsf1os1	2E-04	0.4
Cavin4	1E-07	0.4	Cbr2	3E-19	0.4
Bmi1	3E-11	0.4	Vars	6E-15	0.4
Rab30	2E-15	0.4	Pim3	2E-08	0.4
Tmem263	1E-36	0.4	Mapk6	7E-12	0.4
Pde10a	3E-14	0.4	Hspb2	1E-13	0.4
Snhg6	7E-27	0.4	Tmem132a	9E-13	0.4
Magi3	5E-12	0.4	Gm15738	3E-05	0.4
Zfp146	5E-13	0.4	Prune2	1E-05	0.4
Sntg2	5E-12	0.4	Ccdc157	5E-07	0.4
Gas5	9E-59	0.4	Rgl1	7E-14	0.4
Ikzf2	1E-08	0.4	Dctd	4E-08	0.4
Hspa5	8E-55	0.4	Tcea1	5E-42	0.4
Arhgef2	7E-20	0.4	Arl14ep	2E-14	0.4
Tle3	5E-07	0.4	2410018L13Rik	3E-07	0.4
Fhl2	8E-10	0.4	2700081O15Rik	2E-10	0.4
Ccnd1	2E-34	0.4	Unc5c	2E-15	0.4
Tbpl1	1E-16	0.4	Wwp2	6E-20	0.4
Galnt4	9E-06	0.4	Klc3	5E-07	0.4
Prtg	3E-08	0.4	Gm12153	2E-03	0.4
Akt3	1E-19	0.4	Dusp18	1E-06	0.4
Gm34455	5E-06	0.4	Hectd2	2E-06	0.4
Polr3d	4E-15	0.4	Slc7a2	6E-14	0.4
Cldn12	8E-07	0.4	Fst	2E-18	0.4
Bysl	9E-13	0.4	Plcd1	7E-13	0.4
Rnmt	1E-15	0.4	Ppp3ca	1E-34	0.4
Lins1	1E-06	0.4	Dlc1	1E-34	0.4
Nudcd1	3E-14	0.4	Slc40a1	8E-15	0.4
Mrtfa	9E-20	0.4	Nmnat3	2E-06	0.4
Trim66	1E-03	0.4	Cnn3	4E-31	0.4
Aars	3E-22	0.4	Gramd1b	2E-10	0.4
Cox6a2	4E-10	0.4	Marcksl1	3E-17	0.4
Mtbp	4E-06	0.4	Trp53bp2	6E-10	0.4
Fam160a1	2E-03	0.4	Rwdd1	1E-39	0.4
Mak16	3E-19	0.4	Lrrk1	3E-20	0.4
Farp2	1E-15	0.4	Ankrd44	3E-07	0.4

Chondrogenic

Control Gene	p	FC	NICD2 Gene	p	FC
Zfp36l2	4E-27	0.5	H2-Ke6	2E-10	0.5
Stard13	7E-28	0.5	Dse	3E-24	0.5
Cyb5r2	1E-07	0.5	Ddb2	2E-01	0.5
Piezo2	2E-40	0.5	Adcy9	2E-07	0.5
Aph1c	5E-04	0.5	4930484I04Rik	4E-02	0.5
Slc33a1	1E-16	0.5	Pdp2	2E-02	0.5
Gm36975	2E-05	0.5	Mageh1	2E-14	0.5
Akt3	4E-23	0.5	Rgmb	3E-05	0.5
Rragb	3E-06	0.5	Nat14	5E-03	0.5
Mme	2E-44	0.5	Tfeb	2E-03	0.5
Me3	5E-09	0.5	Rab36	3E-02	0.5
Aspn	4E-03	0.5	Gm42984	1E-02	0.5
Bcr	2E-08	0.5	Mar6	2E-12	0.5
Tgoln1	9E-50	0.5	Ogg1	1E-01	0.5
Ormdl2	8E-13	0.5	Cd151	4E-26	0.5
Gm28375	3E-05	0.5	Orai1	1E-06	0.5
Dse	9E-31	0.5	Taf4b	2E-02	0.5
Cd82	1E-06	0.5	Tjp1	3E-14	0.5
Zfp469	2E-04	0.5	1810026B05Rik	9E-18	0.5
4930481A15Rik	9E-04	0.5	Rfesd	2E-07	0.5
Herpud1	1E-33	0.5	Nap1l3	1E-02	0.5
Ssr3	1E-89	0.5	Krba1	1E-02	0.5
Pygo1	4E-13	0.5	Ifit3b	2E-04	0.5
Trib3	1E-03	0.5	Papss1	1E-12	0.5
Tmem42	4E-10	0.5	Bend6	1E-02	0.5
Rhobtb2	3E-03	0.5	Sdr39u1	8E-08	0.5
Golph3	2E-22	0.5	Gnai1	3E-03	0.5
Nr4a2	7E-05	0.5	Srgap3	8E-28	0.5
Zfp239	1E-05	0.5	Lrrc4	1E-12	0.5
Gm26887	2E-10	0.5	6330418K02Rik	7E-03	0.5
Morn1	4E-03	0.5	Spred1	3E-18	0.5
Rora	9E-48	0.5	2810001G20Rik	3E-04	0.5
Cdkn2c	6E-35	0.5	Mterf1a	2E-01	0.5
Fmn1	3E-29	0.5	Tob1	1E-10	0.5
Tfb1m	3E-03	0.5	Mertk	8E-04	0.5
Raly	3E-35	0.5	Gm19412	2E-03	0.5
Cdk17	2E-13	0.5	Zfp521	5E-42	0.5
Carns1	2E-04	0.5	4930444A19Rik	1E-01	0.5
Erap1	6E-06	0.5	Pdlim5	1E-29	0.5
Prdm16	6E-12	0.5	4930412C18Rik	1E-01	0.5
Morn4	1E-03	0.5	Nr4a1	3E-03	0.5
Nomo1	2E-13	0.5	Dnaaf4	3E-04	0.5
Ptpn4	7E-11	0.5	Cobll1	9E-11	0.5
Gmpr	3E-04	0.5	Cth	3E-03	0.5
Sdf2l1	1E-20	0.5	Spata1	6E-06	0.5
Ccnd2	3E-66	0.5	Atf6	1E-13	0.5
Nap1l3	2E-05	0.5	Kctd15	1E-02	0.5
Mgp	1E-03	0.5	Ptpn14	9E-17	0.5
Zfp335os	1E-02	0.5	Kazald1	5E-12	0.5
Sort1	1E-10	0.5	Meg3	8E-34	0.5
Tenm3	3E-14	0.5	4930455C13Rik	2E-02	0.5
Gm16845	5E-04	0.5	Fbxo7	4E-08	0.5
Prtg	1E-05	0.5	Trmt61b	4E-04	0.5
Dancr	1E-04	0.5	Zfp462	7E-10	0.5
Zhx2	4E-07	0.5	Neo1	2E-20	0.5
Pkia	2E-02	0.5	Nck2	5E-07	0.5
Samhd1	4E-13	0.5	Fuz	1E-05	0.5
Fmnl3	2E-08	0.5	Cetn4	5E-03	0.5
Pak1	7E-17	0.5	Zbtb38	3E-25	0.5
Gm9993	5E-04	0.5	Cdc42ep3	2E-21	0.5
Osbpl9	1E-48	0.5	Runx2os1	2E-02	0.5
Sod3	1E-24	0.5	5330438D12Rik	5E-14	0.5
Gm15614	4E-05	0.5	Il16	2E-08	0.5
Cryzl2	2E-07	0.5	Prxl2c	3E-13	0.5
Scarf2	2E-18	0.5	Pex11a	4E-05	0.5
Abca7	2E-02	0.5	Jpx	1E-04	0.5
Ntn4	1E-06	0.5	Mtss2	3E-12	0.5
Slc43a2	8E-03	0.5	Enpp5	3E-07	0.5
Car6	2E-01	0.5	Tns3	1E-23	0.5
Clcn5	4E-09	0.5	Sgk1	6E-07	0.5
Metrn	1E-06	0.5	Gm49959	6E-03	0.5
A730081D07Rik	1E-02	0.5	Wars2	2E-05	0.5
Gm50240	8E-06	0.5	Lins1	5E-03	0.5

Fibroblast

Control Gene	p	FC	NICD2 Gene	p	FC
Bst2	7E-159	0.4	Tent5a	1E-38	0.4
Blcap	2E-21	0.4	Kctd12b	2E-04	0.4
Tlr3	2E-14	0.4	Tgfb1i1	6E-42	0.4
Flrt2	5E-68	0.4	Maob	4E-10	0.4
Uba7	3E-09	0.4	B4galt1	2E-36	0.4
Glis3	7E-116	0.4	Fsd1	4E-03	0.4
Flywch2	4E-19	0.4	Zfp423	3E-09	0.4
Cebpa	2E-58	0.4	Tsc22d2	6E-37	0.4
Gm16201	3E-20	0.4	Jak2	3E-15	0.4
Pten	2E-161	0.4	Bmper	1E-01	0.4
Acsl3	9E-38	0.4	Ptgfr	2E-05	0.4
Zeb2	4E-168	0.4	Zfp105	1E-08	0.4
Vav2	5E-07	0.4	Wipi1	1E-59	0.4
Tmod2	9E-07	0.4	Psmb9	8E-15	0.4
Plod3	8E-119	0.4	Lrmda	1E-19	0.4
Fer	8E-74	0.4	Itm2b	1E-130	0.4
Tsc22d2	4E-73	0.4	2810433D01Rik	1E-05	0.4
Crppa	5E-24	0.4	Lmx1b	1E-04	0.4
Zfp105	6E-13	0.4	Slc16a2	6E-44	0.4
Blvrb	7E-149	0.4	Lasp1	4E-32	0.4
Terf1	3E-56	0.4	Itga1	1E-28	0.4
Lamb2	2E-55	0.4	Spsb1	5E-07	0.4
Amer1	1E-25	0.4	Dyrk2	4E-10	0.4
P4ha3	2E-39	0.4	Synj2	1E-04	0.4
Gse1	4E-19	0.4	Abca9	2E-10	0.4
Il17d	1E-17	0.4	Stard5	7E-14	0.4
Tram1l1	5E-11	0.4	Arsa	1E-16	0.4
AC103362.1	4E-07	0.4	Rhoq	2E-40	0.4
Mvb12b	7E-21	0.4	Flywch2	3E-10	0.4
Mov10	1E-15	0.4	Man1c1	6E-25	0.4
B4galt1	1E-70	0.4	Crppa	4E-12	0.4
Gsn	3E-237	0.4	Sema6d	9E-06	0.4
Nexn	2E-15	0.4	Il17d	1E-10	0.4
Cdh11	4E-206	0.4	Pten	7E-78	0.4
Magi1	1E-22	0.4	Prkar2b	9E-46	0.4
Cdkn2b	7E-68	0.4	Arhgef25	2E-50	0.4
Nat2	6E-10	0.4	Arvcf	3E-05	0.4
Sipa1l1	7E-27	0.4	Epb41l3	2E-46	0.4
Arsa	6E-28	0.4	Rmdn2	3E-10	0.4
Mid2	7E-15	0.4	Tram1l1	1E-05	0.4
Gm10941	6E-07	0.4	Bace1	1E-21	0.4
Eef2k	8E-32	0.4	Dock1	6E-41	0.4
Wipi1	4E-98	0.4	Ankrd27	6E-11	0.4
Slit3	5E-21	0.4	Rnf213	3E-18	0.4
Il6st	3E-145	0.4	Tpcn2	2E-04	0.4
Gm41724	1E-30	0.4	Gm41724	1E-15	0.4
Jak2	1E-26	0.4	Dlg2	7E-03	0.4
2810433D01Rik	8E-07	0.4	Sbsn	2E-25	0.4
Unc13b	6E-07	0.4	Acsl3	1E-18	0.4
Gtdc1	3E-27	0.4	Prr16	3E-22	0.4
Rhoq	9E-72	0.4	Nrn1	3E-25	0.4
Tshz3	2E-25	0.4	Parp3	3E-51	0.4
S100a4	5E-145	0.4	Zfr2	8E-10	0.4
Vcl	3E-34	0.4	Pus7l	3E-03	0.4
Tstd3	2E-21	0.4	Dcaf6	5E-28	0.4
Tcf7l1	8E-59	0.4	Ano6	9E-46	0.4
Kctd12	7E-73	0.4	Tshz3	2E-12	0.4
Glce	9E-25	0.4	Pdgfrb	1E-41	0.4
Ssc5d	2E-46	0.4	Mvd	1E-08	0.4
Gadd45b	6E-33	0.4	Ten1	9E-12	0.4
Ppl	2E-30	0.4	Pfn2	1E-41	0.4
Lrmda	9E-33	0.4	Nat2	2E-05	0.4
Micall2	1E-08	0.4	Sqle	1E-17	0.4
Nrip1	4E-101	0.4	Rab3il1	2E-08	0.4
Skil	7E-10	0.4	S100a4	1E-65	0.4
Rmdn2	9E-16	0.4	Erg28	9E-42	0.4
Fam13a	2E-09	0.4	Fer	2E-34	0.4
Ttyh3	3E-40	0.4	Emc2	2E-58	0.4
Aldh1l1	2E-20	0.4	Il17rc	9E-15	0.4
Myadm	5E-48	0.4	Zfp503	1E-48	0.4
Plcxd2	2E-11	0.4	Tap1	1E-06	0.4
Epb41l3	4E-89	0.4	Lrp6	8E-39	0.4
Lipa	7E-102	0.4	Setd4	3E-06	0.4

Fibroblast?

Control Gene	p	FC	NICD2 Gene	p	FC
Mpp6	7E-95	1.1	Itga6	3E-47	1.1
Rfc1	1E-139	1.1	Nt5dc2	8E-123	1.1
1600010M07Rik	3E-62	1.1	Lrrc75b	1E-22	1.1
Rad9a	5E-37	1.1	Nap1l1	0E+00	1.1
2700038G22Rik	6E-25	1.1	Hsp90aa1	0E+00	1.1
Dars2	1E-58	1.1	Gucy1a2	4E-80	1.1
Fam72a	3E-20	1.1	Cpa6	1E-39	1.1
2610020C07Rik	1E-78	1.1	Cep85	6E-71	1.1
Paqr4	4E-34	1.1	Timm50	1E-111	1.1
Arl6ip6	2E-78	1.1	Gk	8E-46	1.1
Gsr	9E-70	1.1	Pak4	1E-36	1.1
Prpf4	2E-80	1.1	Cav2	7E-89	1.1
Ranbp1	2E-203	1.1	Herc6	3E-82	1.1
Itga6	6E-39	1.1	Hspa14	1E-128	1.1
Herc3	6E-16	1.1	Exosc2	6E-66	1.1
Grin3a	1E-27	1.1	Zmym1	3E-87	1.1
Cbx5	1E-159	1.1	Cep135	2E-65	1.1
Abcb1b	1E-52	1.1	Pole3	4E-140	1.1
Rdh12	2E-23	1.1	Arl4c	7E-49	1.1
Ano1	2E-27	1.1	Zdhhc15	3E-69	1.1
A030001D20Rik	4E-42	1.1	Itgb3bp	3E-124	1.1
Carhsp1	4E-99	1.1	Card10	4E-29	1.1
Dynlt1f	1E-74	1.1	Fastkd3	3E-54	1.1
Pfas	9E-55	1.1	Cep290	2E-114	1.1
Chst7	5E-40	1.1	Ctsc	3E-61	1.1
Ppp1r18os	7E-24	1.1	Carhsp1	5E-138	1.1
Eif1ad	1E-95	1.1	Dis3	3E-71	1.1
Rin3	2E-32	1.1	Cenpc1	2E-94	1.1
Platr25	3E-77	1.1	Cbx5	3E-222	1.1
Cenpc1	4E-72	1.1	Specc1	1E-94	1.1
Tmem117	1E-27	1.1	Ppp2r5d	2E-82	1.1
Mad1l1	5E-95	1.1	Lrrc75a	1E-45	1.1
Cntln	2E-101	1.1	Plekhg3	6E-80	1.1
Ank1	2E-25	1.1	Lcorl	5E-94	1.1
Pcyox1l	5E-65	1.1	Chd1l	7E-55	1.1
1700048O20Rik	8E-23	1.1	Nudt1	6E-69	1.1
D030056L22Rik	8E-64	1.1	Tmem37	1E-65	1.1
Itgb3	1E-18	1.1	Tmem177	1E-20	1.1
Zmym1	1E-68	1.1	Pdlim1	4E-97	1.1
Tbc1d1	3E-46	1.1	Tdp2	9E-78	1.1
H2afv	1E-158	1.1	Pitpnm2	8E-58	1.1
Loxl4	1E-20	1.1	Rnaseh2c	1E-177	1.1
Cep85	9E-57	1.1	Apol9b	9E-25	1.1
Shmt2	4E-66	1.1	Cinp	1E-97	1.1
9230114K14Rik	5E-79	1.1	Sox11	8E-45	1.1
Ppm1g	3E-100	1.1	H2-Q4	4E-52	1.1
Tlnrd1	3E-66	1.1	Anp32b	0E+00	1.1
Slco3a1	2E-65	1.1	Suz12	2E-123	1.1
Mthfd1l	1E-77	1.1	Gm39090	7E-32	1.1
Rflnb	3E-63	1.1	Klf5	3E-34	1.1
Rpa3	2E-75	1.1	Msh2	1E-82	1.1
Mnd1	4E-41	1.1	Shmt2	7E-86	1.1
Cnot9	5E-68	1.1	Srsf7	7E-196	1.1
Cklf	4E-65	1.1	4930522L14Rik	2E-60	1.1
Helq	5E-50	1.1	Gask1b	6E-74	1.1
Camk2n2	4E-63	1.1	Rpp30	1E-86	1.1
Galnt12	3E-21	1.1	Rad50	6E-174	1.1
Ifrd2	1E-57	1.1	Rprm	3E-28	1.1
Dis3	2E-57	1.1	Nedd1	2E-82	1.1
Rnaseh2c	1E-130	1.1	Plaur	2E-50	1.1
Pald1	8E-21	1.1	Nelfe	3E-115	1.1
Exosc2	4E-47	1.1	Crybg1	5E-33	1.1
Zdhhc15	1E-48	1.1	Ank1	3E-35	1.1
Nudc	2E-185	1.1	Inka2	1E-61	1.0
Rxfp1	1E-23	1.1	Bend6	4E-49	1.0
Senp1	2E-68	1.1	Kpnb1	7E-146	1.0
Homer2	4E-36	1.1	Tsen15	8E-113	1.0
Pstpip1	7E-19	1.1	Naa40	1E-74	1.0
Pcgf5	9E-47	1.1	Eda2r	3E-71	1.0
Tm4sf1	3E-80	1.1	Pwwp3a	1E-87	1.0
Parp1	2E-63	1.1	Gucy1b1	6E-32	1.0
Timm50	5E-91	1.1	Siva1	2E-235	1.0
Srd5a1	5E-43	1.1	Nop56	2E-109	1.0

Undefined

Control Gene	p	FC	NICD2 Gene	p	FC
Serpinb9	4E-01	0.2	Cir1	2E-05	0.2
Brsk1	5E-01	0.2	Aplp2	6E-24	0.2
Ctu2	1E-01	0.2	Dph5	1E-01	0.2
Rdm1	5E-01	0.2	Ypel3	2E-12	0.2
Pebp1	3E-36	0.2	Cfap410	4E-01	0.2
Elmo1	5E-05	0.2	Ddit4	1E-01	0.2
Nr1h2	4E-03	0.2	Mynn	9E-01	0.2
Dlc1	3E-09	0.2	Ifit1	5E-01	0.2
Aldoa	8E-34	0.2	Pomk	1E+00	0.2
Gpi1	3E-10	0.2	Mrm3	9E-01	0.2
P3h3	2E-14	0.2	Epdr1	2E-03	0.2
Coq3	5E-03	0.2	Eef2kmt	4E-02	0.2
Csnk1e	2E-03	0.2	Pi4k2a	7E-02	0.2
Casp6	9E-02	0.2	Isy1	7E-01	0.2
Mapre3	5E-02	0.2	Mat2b	2E-05	0.2
Fam234a	2E-02	0.2	Ccpg1os	3E-01	0.2
Gm47071	2E-01	0.2	Akap7	2E-01	0.2
4921504A21Rik	1E-01	0.2	Nicn1	6E-01	0.2
Alkbh6	3E-02	0.2	Dmac1	5E-03	0.2
Rnd2	6E-01	0.2	Gtf3c6	8E-05	0.2
Sgms2	7E-03	0.2	Kti12	2E-01	0.2
Tvp23b	3E-08	0.2	Tmtc1	1E-01	0.2
Jun	2E-07	0.2	Thra	7E-13	0.2
Lmbr1l	6E-01	0.2	Tctn1	8E-01	0.2
Mettl2	1E+00	0.2	Plcl2	2E-01	0.2
Sap30bp	1E-01	0.2	Gm16845	3E-01	0.2
Gm49539	5E-01	0.2	Tmem67	4E-01	0.2
0610040B10Rik	6E-01	0.2	Tspyl4	6E-03	0.2
Catsper2	7E-01	0.2	Sirt3	4E-01	0.2
Ttc33	1E-01	0.2	Mgp	6E-14	0.2
C87436	6E-02	0.2	Tomm40l	2E-01	0.2
Trp53cor1	8E-02	0.2	Asna1	5E-01	0.2
Rel	9E-02	0.2	Mapre2	4E-01	0.2
Crlf2	1E-02	0.2	Crem	5E-01	0.2
Snhg17	7E-01	0.2	Sugp1	9E-01	0.2
Bbs2	7E-01	0.2	Pkm	2E-11	0.2
2810410L24Rik	9E-01	0.2	Pebp1	4E-29	0.2
Wdr27	5E-01	0.2	Ube2e3	2E-07	0.2
Reck	1E-11	0.2	Ppp3cc	7E-01	0.2
Eml5	9E-01	0.2	Thoc5	1E+00	0.2
Farp1	7E-09	0.2	Ppp2r3d	5E-01	0.2
Vps37c	4E-01	0.2	Thumpd1	8E-01	0.2
Def6	1E+00	0.2	Szt2	5E-01	0.2
Cd80	5E-01	0.2	Rundc1	8E-01	0.2
Gm9993	9E-01	0.2	Lrrn3	6E-01	0.2
Usf1	4E-01	0.2	Rabl2	8E-01	0.2
Lin37	3E-01	0.2	Ppm1f	5E-01	0.2
Ap3s2	7E-02	0.2	Robo2	1E-04	0.2
Malt1	7E-01	0.2	Gdf11	3E-01	0.2
Trim8	9E-01	0.2	Dmap1	5E-01	0.2
Dexi	2E-02	0.2	Fhit	3E-01	0.2
Igfbp5	2E-01	0.2	Gm14966	4E-01	0.2
Ppm1l	8E-02	0.2	Srfbp1	7E-02	0.2
Hs6st2	2E-01	0.2	Psmg3	9E-01	0.2
Gja1	1E-09	0.2	Zfp511	8E-01	0.2
Irf7	9E-04	0.2	Ccndbp1	1E-02	0.2
Plxna3	9E-01	0.2	Slc27a1	5E-01	0.2
Snhg18	2E-17	0.2	Dip2b	6E-06	0.2
Chac1	4E-02	0.2	Rgmb	8E-01	0.2
Rbfox1	8E-02	0.2	Ckb	2E-02	0.2
Tut1	1E+00	0.2	Prkar1b	4E-01	0.2
Phlpp1	3E-03	0.2	Zfpm1	4E-02	0.2
Acp5	1E-01	0.2	Ostf1	1E-09	0.2
Gpr27	6E-01	0.2	Dusp5	6E-01	0.2
Fbxl15	4E-01	0.2	Vps13c	5E-06	0.2
Wdr13	6E-01	0.2	Cnpy2	8E-38	0.2
Prdx2	5E-28	0.2	S100a16	3E-04	0.2
Mafg	3E-03	0.2	Nudt16	9E-01	0.2
Aspn	7E-12	0.2	Wdr83	9E-01	0.2
Zgpat	1E+00	0.2	Dbi	7E-30	0.2
Jund	2E-20	0.2	Nudt9	2E-03	0.2
Tspyl4	2E-03	0.2	Cyb5r2	1E-01	0.2
Pitpnm1	4E-01	0.2	Gpcpd1	2E-01	0.2

Articular/Synovial Fibroblast

Control Gene	p	FC	NICD2 Gene	p	FC
Ptpdc1	2E-02	0.5	Arhgef6	8E-03	0.5
Arsb	7E-07	0.5	Jarid2	7E-11	0.5
Il1rn	6E-09	0.5	AI480526	9E-01	0.5
Rbks	8E-02	0.5	Adamts2	1E-09	0.5
E030030I06Rik	6E-02	0.5	Igsf8	1E-04	0.5
Upp2	3E-08	0.5	Vcpkmt	2E-01	0.5
Eef2k	3E-06	0.5	Eya4	2E-18	0.5
Mef2a	2E-26	0.5	Nol3	1E-02	0.5
Acp5	1E-01	0.5	Casp9	2E-01	0.5
Gypc	2E-03	0.5	Zcchc24	9E-11	0.5
Lhfpl2	4E-08	0.5	Nr1h3	4E-02	0.5
Ddr2	4E-25	0.5	Plekho2	3E-02	0.5
Ripor1	2E-02	0.5	Rdh5	7E-02	0.5
Lcn2	1E-30	0.5	Klhdc1	2E-01	0.5
Tap1	2E-02	0.5	Dnajc12	1E-01	0.5
Rora	2E-31	0.5	Gm4951	9E-04	0.5
Snx2	3E-12	0.5	6330562C20Rik	5E-01	0.5
Dglucy	3E-03	0.5	Fnip1	6E-10	0.5
Lynx1	4E-04	0.5	Naaa	4E-01	0.5
Ccn5	2E-30	0.5	Il1rl2	4E-01	0.5
Zfp169	9E-01	0.5	Litaf	1E-04	0.5
Tubb2b	8E-04	0.5	Shld1	2E-02	0.5
Ucp2	1E-04	0.5	Brdt	3E-01	0.5
Tacc1	2E-19	0.5	2310010J17Rik	5E-03	0.5
Impact	3E-16	0.5	Cdkl2	3E-01	0.5
BC002059	2E-01	0.5	Per3	2E-04	0.5
Dusp3	6E-04	0.5	Arhgap31	1E-04	0.5
Peg13	3E-01	0.5	Lrsam1	2E-01	0.5
Zfp36	4E-05	0.5	Zbtb43	2E-01	0.5
Gsdmd	1E-02	0.5	Gm16008	6E-01	0.5
Ly6e	4E-25	0.5	Tnks	3E-08	0.5
Specc1	7E-02	0.5	Ppfibp2	1E-01	0.5
Htra1	2E-09	0.5	Entpd2	2E-01	0.5
Cfap69	2E-01	0.5	Prxl2a	3E-04	0.5
Pvt1	8E-16	0.5	Tob1	9E-05	0.5
Ypel3	5E-21	0.5	Rgcc	7E-01	0.5
Napepld	4E-01	0.5	A430073D23Rik	5E-01	0.5
Alas1	2E-08	0.5	Cfap300	7E-01	0.5
Vps13c	2E-14	0.5	Asah2	6E-02	0.5
Spink10	2E-02	0.5	B3galnt1	8E-02	0.5
Emc2	2E-24	0.5	Vmac	8E-01	0.5
Naprt	8E-02	0.5	Creld1	4E-02	0.5
Gm6712	5E-01	0.5	Stx16	2E-06	0.5
S100a13	4E-27	0.5	Dapp1	2E-01	0.5
Gpd1l	9E-02	0.5	Ap3m2	3E-01	0.5
Tle5	3E-51	0.5	Fbln5	1E-08	0.5
Pycard	4E-07	0.5	Plcxd2	3E-01	0.5
Rabgap1l	9E-11	0.5	2900052L18Rik	4E-01	0.5
4921531C22Rik	1E-01	0.5	Gm20559	4E-02	0.5
Mapk13	6E-03	0.5	Hook2	1E-01	0.5
Pros1	3E-13	0.5	Rhoq	1E-11	0.5
Airn	5E-16	0.5	Taf6l	2E-01	0.5
Fam13a	6E-02	0.5	Mkks	8E-01	0.5
Ell	6E-02	0.5	Ggta1	4E-01	0.5
Ramp1	2E-02	0.5	Zfand1	1E-02	0.5
Arsk	6E-04	0.5	Mndal	2E-02	0.5
Gm2a	4E-11	0.5	Gstm2	1E-11	0.5
Olfm1	2E-06	0.5	Gnptg	7E-04	0.5
Espn	3E-01	0.5	9130008F23Rik	2E-01	0.5
Hook2	1E-01	0.5	Igf2r	2E-10	0.5
Abcc10	5E-01	0.5	Htra1	2E-07	0.5
Adgrg2	4E-09	0.5	4921531C22Rik	6E-01	0.5
Prkn	3E-06	0.5	Vps33a	5E-02	0.5
Tle6	5E-02	0.5	Prkn	3E-08	0.5
Ptpn3	2E-02	0.5	Vegfd	3E-18	0.5
Jund	2E-33	0.5	Usp20	6E-01	0.5
Col3a1	1E-30	0.5	1110046J04Rik	3E-01	0.5
Mknk2	7E-05	0.5	Frmd8	2E-01	0.5
Igtp	3E-01	0.5	Abhd4	2E-02	0.5
Ldb2	4E-11	0.5	Phldb1	3E-02	0.5
Gcnt1	4E-01	0.5	Ttc39c	3E-01	0.5
Cdc42bpb	4E-05	0.5	Kdsr	7E-02	0.5
Enox2	1E-06	0.5	Xkr6	4E-01	0.5

Limb Mesenchyme						Chondrogenic					
Control			NICD2			Control			NICD2		
Gene	*p*	FC	Gene	*p*	FC	Gene	*p*	FC	Gene	*p*	FC
Cox10	2E-10	0.4	Nars	4E-42	0.4	A330023F24Rik	3E-04	0.5	Immp2l	1E-09	0.5
Ptpn14	6E-21	0.4	Hist2h4	6E-04	0.4	Gm5577	5E-02	0.5	C030006K11Rik	5E-03	0.5
Plcg2	3E-07	0.4	Zfp319	5E-08	0.4	Cd151	2E-29	0.5	Vezf1	3E-23	0.5
Mecom	8E-08	0.4	Enox1	2E-10	0.4	Rnd2	2E-04	0.5	BC024978	2E-01	0.5
Smn1	3E-16	0.4	Ecrg4	9E-34	0.4	Ccdc173	7E-07	0.5	Gm19710	6E-06	0.5
Gmfb	2E-15	0.4	Kifc3	2E-09	0.4	Cyp39a1	2E-04	0.5	Magi3	2E-07	0.5
Ctnnal1	5E-10	0.4	Eif2b2	2E-12	0.4	Bcl2l1	1E-14	0.5	Fam20c	1E-01	0.5
Rarres1	1E-04	0.4	Bend5	4E-14	0.4	Cald1	2E-73	0.5	Ripk2	2E-04	0.5
Nol8	2E-12	0.4	Adarb1	2E-05	0.4	Zbtb38	6E-27	0.5	Cep41	2E-05	0.5
Edem3	5E-14	0.4	Gdf10	3E-08	0.4	Trmt61b	8E-07	0.5	Smtn	2E-10	0.5
Sec24d	3E-26	0.4	Zfp790	3E-07	0.4	2010001A14Rik	4E-05	0.5	Mapre3	5E-11	0.5
Hyou1	1E-16	0.4	Mia	6E-65	0.4	Myo10	7E-31	0.5	Bbs2	7E-03	0.5
Gm48742	4E-10	0.4	B230217O12Rik	2E-06	0.4	Otud1	5E-04	0.5	Lrig3	6E-19	0.5
Gsdme	3E-12	0.4	Sgcd	1E-25	0.4	Agbl5	8E-04	0.5	Hmgn3	1E-50	0.5
Wdr74	1E-14	0.4	E130317F20Rik	4E-04	0.4	Pitpnc1	2E-17	0.5	Selenos	2E-62	0.5
Rrp12	2E-04	0.4	Zfp266	7E-18	0.4	Maf	4E-31	0.5	Arl4a	5E-11	0.5
Tsr1	1E-12	0.4	Fli1	3E-08	0.4	Gfpt1	5E-28	0.5	Ifit3	5E-05	0.5
Tbx15	1E-25	0.4	Cyb5rl	3E-01	0.4	Rbpj	6E-53	0.5	Mbnl1	5E-35	0.5
Wfs1	2E-10	0.4	Mtcl1	2E-05	0.4	0610043K17Rik	1E-05	0.5	Kcnt2	3E-02	0.5
Smyd5	6E-07	0.4	Aplp2	3E-41	0.4	A630072M18Rik	5E-04	0.5	Slc35d1	8E-03	0.5
Cop1	1E-26	0.4	Asap1	6E-27	0.4	D3Ertd751e	5E-09	0.5	Thbd	4E-06	0.5
Gm10561	4E-08	0.4	Pdp2	1E-04	0.4	Plcd1	2E-11	0.5	Tbc1d22a	1E-07	0.5
Zswim3	7E-05	0.4	Bcl2	4E-08	0.4	Ctnna1	2E-44	0.5	Dstn	2E-59	0.4
Fam98a	1E-16	0.4	Ern1	2E-08	0.4	Iqsec1	6E-14	0.5	Egfros	3E-05	0.4
Fam171b	3E-08	0.4	Uso1	2E-22	0.4	Hspa4l	1E-04	0.5	Hoxc5	4E-05	0.4
Zswim1	9E-08	0.4	Mex3d	3E-08	0.4	Gm10561	1E-03	0.5	Tcp11l2	1E-07	0.4
Pxdn	6E-19	0.4	Tsr1	6E-09	0.4	Fam107b	8E-08	0.5	Camk1d	1E-14	0.4
Tmeff1	5E-09	0.4	Amfr	5E-14	0.4	Pfkm	1E-05	0.5	Ide	2E-15	0.4
Cdk7	4E-22	0.4	4833418N02Rik	2E-04	0.4	Sesn3	4E-33	0.5	Cyth1	3E-04	0.4
Hk2	2E-11	0.4	Sphk2	4E-06	0.4	Btg2	2E-24	0.5	Soat1	3E-06	0.4
Uck2	7E-12	0.4	Pdp1	6E-16	0.4	Zfp94	3E-02	0.5	Ip6k2	4E-09	0.4
Rbmx	2E-11	0.4	Gm47350	6E-05	0.4	Gm48765	4E-02	0.5	Tmem246	2E-03	0.4
Endov	6E-08	0.4	Trim35	1E-23	0.4	Zfyve27	2E-02	0.5	Btg2	1E-19	0.4
Ubqln4	3E-10	0.4	4933400A11Rik	6E-04	0.4	Ccdc39	1E-03	0.5	Ap4s1	1E-12	0.4
Lpin2	2E-12	0.4	Cep126	5E-03	0.4	Ahi1	1E-21	0.4	Ppp1r13b	1E-04	0.4
Fgf13	2E-11	0.4	Abhd17b	7E-15	0.4	Glrb	6E-01	0.4	Ift122	3E-06	0.4
Gm5165	3E-04	0.4	Mnat1	5E-27	0.4	Mtln	5E-25	0.4	Zbtb18	1E-03	0.4
Galc	4E-08	0.4	Rasa2	7E-13	0.4	Crebrf	5E-26	0.4	Gm13986	7E-03	0.4
Pdcl	1E-11	0.4	Rab39b	7E-06	0.4	H2-Ke6	7E-13	0.4	Nt5dc1	2E-08	0.4
Inpp4b	1E-03	0.4	Gm49417	6E-05	0.4	Tgfa	1E-03	0.4	Klhl21	5E-03	0.4
Cdca7	2E-06	0.4	Dusp1	1E-11	0.4	Bckdk	3E-17	0.4	Pakap	3E-09	0.4
Itga8	2E-04	0.4	Ccn1	2E-21	0.4	Chac1	3E-03	0.4	Cgrrf1	2E-11	0.4
Prrc1	1E-21	0.4	Col8a1	6E-15	0.4	Grk4	1E-07	0.4	Hyou1	1E-09	0.4
Syn3	9E-25	0.4	Gm19522	2E-03	0.4	Zkscan5	1E-03	0.4	Stim2	6E-08	0.4
Mar5	7E-35	0.4	Rdh10	2E-06	0.4	Tex9	2E-04	0.4	Klhl24	3E-14	0.4
Tmem8b	1E-04	0.4	Noc3l	7E-12	0.4	Mmp16	1E-06	0.4	Apmap	2E-06	0.4
3110021N24Rik	5E-09	0.4	Rnf139	2E-10	0.4	Pgrmc2	1E-41	0.4	A930005H10Rik	1E-06	0.4
Ell2	5E-33	0.4	Col4a3bp	7E-11	0.4	Spata2	2E-03	0.4	Crebrf	4E-19	0.4
Rapgefl1	9E-05	0.4	Dync1li1	1E-14	0.4	Yod1	3E-05	0.4	Rab31	6E-11	0.4
Spata1	5E-12	0.4	Fam227b	4E-01	0.4	Yipf2	1E-05	0.4	Fndc3b	3E-40	0.4
A130010J15Rik	5E-06	0.4	Trit1	5E-07	0.4	Klc2	5E-04	0.4	Usf3	4E-04	0.4
Acot1	1E-04	0.4	Slc22a14	7E-05	0.4	Ldah	3E-20	0.4	Bhlhe40	2E-04	0.4
2900026A02Rik	9E-07	0.4	Sec16b	2E-11	0.4	Il15ra	2E-04	0.4	Dnaaf5	6E-02	0.4
Gm42439	1E-07	0.4	Gm15581	3E-04	0.4	Pdgfa	4E-18	0.4	Zfp113	2E-04	0.4
Frk	3E-08	0.4	Gria3	6E-21	0.4	4921511C10Rik	1E-02	0.4	Caskin2	2E-01	0.4
Kif5b	1E-48	0.4	Zbed5	2E-03	0.4	Cobll1	3E-14	0.4	Btg1	7E-19	0.4
Nop2	1E-11	0.4	Pinx1	5E-10	0.4	Zfp2	2E-03	0.4	Sertad4	2E-02	0.4
Ptprg	9E-20	0.4	Cp	8E-11	0.4	Rab3b	2E-03	0.4	Rwdd3	2E-02	0.4
8430436N08Rik	7E-03	0.4	Tbl1x	1E-19	0.4	Brd3os	6E-04	0.4	Ahi1	8E-11	0.4
Btg3	6E-23	0.4	Cry1	5E-08	0.4	Acp6	1E-03	0.4	Kcnd2	2E-07	0.4
Tmem117	7E-05	0.4	Firre	6E-05	0.4	Tmem243	2E-11	0.4	Crybg3	1E-05	0.4
Selenos	2E-55	0.4	Prkrip1	2E-11	0.4	Gm9856	9E-04	0.4	Zmat1	9E-02	0.4
Ereg	4E-03	0.4	Nubpl	2E-06	0.4	Clmn	6E-04	0.4	B4galt3	1E-01	0.4
Myc	4E-06	0.4	Lipo3	3E-06	0.4	Gm27003	1E-01	0.4	Castor1	4E-02	0.4
Ak4	3E-05	0.4	Fam98a	2E-16	0.4	Inca1	9E-03	0.4	Mmaa	3E-03	0.4
Dmac1	5E-19	0.4	Six4	1E-07	0.4	Stk39	1E-10	0.4	Eml5	1E-02	0.4
Bcl7a	1E-05	0.4	AC154200.1	1E-03	0.4	Pdp2	2E-02	0.4	Arhgef5	1E-06	0.4
Dnaja3	4E-12	0.4	Slmap	1E-16	0.4	Ddhd1	4E-05	0.4	Il15ra	1E-02	0.4
2610027K06Rik	6E-05	0.4	Tmem44	1E-03	0.4	Riiad1	1E-02	0.4	Zfp87	2E-02	0.4
Erbb2	5E-06	0.4	Rnf166	4E-08	0.4	Ank	1E-27	0.4	Ficd	1E-02	0.4
Arhgef3	7E-11	0.4	Usp10	3E-12	0.4	Slc35e3	3E-04	0.4	Pigv	3E-04	0.4
Nmnat3	7E-09	0.4	Per1	2E-07	0.4	Syne1	5E-30	0.4	Atp6v1a	2E-28	0.4
Osbpl6	1E-08	0.4	Rnf40	3E-04	0.4	Sp4	3E-05	0.4	Upf3b	2E-13	0.4

Fibroblast						Fibroblast?					
Control			NICD2			Control			NICD2		
Gene	*p*	FC	Gene	*p*	FC	Gene	*p*	FC	Gene	*p*	FC
9330179D12Rik	2E-05	0.4	Gli2	1E-06	0.4	Itgb3bp	5E-85	1.1	Lsm6	1E-205	1.0
Lrp6	3E-78	0.4	Vav2	3E-04	0.4	Jph2	2E-33	1.1	Trim47	8E-77	1.0
Tap1	2E-14	0.4	Kctd12	1E-29	0.4	Lrrc8c	6E-64	1.1	A030001D20Rik	1E-47	1.0
Hes1	3E-28	0.4	Dipk1a	9E-07	0.4	Mgme1	5E-55	1.1	Gm11290	4E-19	1.0
Tead3	8E-15	0.4	Cd44	1E-21	0.4	Cln6	8E-54	1.1	Myl9	5E-34	1.0
Tiam1	6E-04	0.4	Add3	2E-37	0.4	Lrwd1	5E-54	1.1	Hmgb1	0E+00	1.0
Trim16	9E-13	0.4	Vash2	2E-03	0.4	Lrp8	3E-37	1.1	Arf2	2E-82	1.0
Sh3bp5	3E-29	0.4	Dtx3l	6E-08	0.4	Hmgb1	2E-274	1.1	Hmga1	1E-32	1.0
Fkbp1b	3E-11	0.4	Fas	6E-11	0.4	Cd44	9E-65	1.1	Jade1	3E-69	1.0
Grin2d	1E-05	0.4	Dsel	3E-22	0.4	Rad50	6E-136	1.1	Jph2	2E-46	1.0
Pcdhga10	4E-08	0.4	Cblb	1E-21	0.4	AW551984	8E-24	1.1	Ptk2b	2E-37	1.0
Pik3c2a	2E-44	0.4	Nipal2	5E-04	0.4	Nap1l1	2E-222	1.1	Foxs1	2E-19	1.0
Eepd1	7E-08	0.4	Flot1	2E-44	0.4	Anp32b	2E-215	1.1	Tubgcp3	2E-78	1.0
Rnf213	4E-33	0.4	Glis3	6E-54	0.4	Zbed3	8E-50	1.1	Gm15246	2E-25	1.0
Ramp1	1E-08	0.4	C2cd2	3E-05	0.4	Frmd4a	4E-50	1.1	Ppm1g	7E-127	1.0
Dcaf6	7E-45	0.4	Ncald	2E-02	0.4	Kctd9	3E-59	1.1	Lrp8	4E-39	1.0
Clmp	3E-94	0.4	Cd302	3E-53	0.4	Arf2	4E-63	1.1	Srsf2	6E-184	1.0
Rps6ka5	4E-03	0.4	Sh3bp5	2E-13	0.4	Plp2	2E-76	1.0	Gch1	1E-27	1.0
Stard5	2E-22	0.4	Pcolce2	6E-23	0.4	Pomt1	1E-46	1.0	Gstcd	3E-90	1.0
Tent5a	2E-57	0.4	Mvb12b	4E-09	0.4	Gk	1E-46	1.0	Ddah1	6E-101	1.0
Lasp1	8E-45	0.4	Per3	1E-19	0.4	Tubgcp3	3E-62	1.0	E2f6	1E-80	1.0
Palld	1E-89	0.4	Nxn	4E-12	0.4	Ppm1d	4E-68	1.0	Nsg1	3E-135	1.0
Zfp217	9E-05	0.4	Epha3	3E-05	0.4	Rap1gap2	8E-28	1.0	Uevld	7E-61	1.0
Filip1l	3E-38	0.4	Adck2	5E-05	0.4	Thy1	3E-33	1.0	Cand2	4E-49	1.0
Ccdc12	3E-61	0.4	Slc35f5	4E-12	0.4	Srrt	2E-80	1.0	Lyrm9	4E-24	1.0
Zfp809	1E-16	0.4	Pcdhga5	5E-06	0.4	Nedd1	3E-63	1.0	Tfap4	5E-13	1.0
Cblb	7E-39	0.4	Idi1	4E-21	0.4	Cinp	7E-70	1.0	Nup62	5E-61	1.0
Frrs1	3E-21	0.4	Tnxb	1E-26	0.4	Ormdl1	6E-43	1.0	Frmd3	8E-34	1.0
Sema6d	9E-08	0.4	Adamts1	4E-25	0.4	Galnt18	9E-76	1.0	Paqr4	7E-32	1.0
Gprc5b	4E-07	0.4	H2-K1	2E-84	0.4	Mast2	3E-70	1.0	Dcbld1	2E-67	1.0
Abca8b	2E-04	0.4	Lamc1	3E-28	0.4	Samd1	2E-46	1.0	Rab23	5E-81	1.0
Rarres2	4E-78	0.4	Rarres2	1E-42	0.4	Acaa1b	2E-28	1.0	Abcb1b	4E-73	1.0
Fam171a1	1E-15	0.4	Mlxip	2E-08	0.4	Slc16a3	2E-39	1.0	Spata7	7E-14	1.0
Mdfic	1E-68	0.4	Tnks1bp1	1E-23	0.4	Lsm5	1E-117	1.0	Nbn	8E-87	1.0
Tulp4	8E-83	0.4	Pik3r3	3E-17	0.4	4930522L14Rik	5E-55	1.0	Arhgap33	4E-25	1.0
Parp3	4E-89	0.4	Prkcd	8E-09	0.4	Crip2	3E-64	1.0	Sac3d1	1E-96	1.0
Gla	2E-15	0.4	Fkbp1b	1E-05	0.4	Mical2	1E-31	1.0	Cda	1E-38	1.0
Mex3b	3E-06	0.4	Greb1l	3E-03	0.4	Usp2	6E-45	1.0	Tagln2	7E-217	1.0
Gsto1	8E-67	0.4	Fam228b	4E-04	0.4	Pole3	5E-88	1.0	Kras	7E-112	1.0
Gpsm1	6E-13	0.4	Tulp4	5E-44	0.4	B130055M24Rik	2E-52	1.0	Tssc4	3E-87	1.0
Il17rc	2E-25	0.3	Glce	3E-13	0.4	Cdan1	2E-33	1.0	Mgme1	2E-53	1.0
Afap1	2E-76	0.3	Wnt5a	6E-18	0.4	Rab3b	1E-26	1.0	Lsm5	2E-187	1.0
Tnc	2E-19	0.3	Pde3a	2E-20	0.4	Pitpnm2	5E-44	1.0	Mthfd1l	4E-80	1.0
Zc3h12a	9E-07	0.3	Slc36a1	7E-06	0.4	Syt1	2E-09	1.0	1810037I17Rik	2E-193	1.0
Dbp	3E-51	0.3	Ttyh3	3E-20	0.4	Cdkn2c	5E-79	1.0	Dennd2c	7E-37	1.0
Tnxb	1E-47	0.3	Gm12905	1E-05	0.4	Frmd3	5E-15	1.0	Mrpl18	1E-201	1.0
Golga7	1E-34	0.3	Sh3rf3	2E-28	0.4	Suz12	8E-85	1.0	Adh7	2E-35	1.0
Ubtd2	4E-48	0.3	Pir	4E-17	0.4	Tsen15	3E-76	1.0	Tpm4	7E-203	1.0
Sh3bp1	1E-10	0.3	2900089D17Rik	2E-03	0.4	Csrp1	6E-79	1.0	Larp1b	1E-57	1.0
Arhgef25	4E-75	0.3	Wwp1	3E-31	0.4	Msh2	6E-50	1.0	Gm36569	3E-21	1.0
Itm2b	3E-213	0.3	Gsta4	7E-89	0.4	Zfp808	7E-49	1.0	Impa2	4E-68	1.0
Naprt	3E-12	0.3	Klf16	2E-03	0.4	Uevld	7E-47	1.0	Pde3b	1E-76	1.0
Pfn2	1E-60	0.3	H2-M3	9E-07	0.3	Tagln	4E-31	1.0	2610020C07Rik	9E-73	1.0
Pik3r3	5E-33	0.3	Cpeb1	2E-16	0.3	Cnn1	1E-10	1.0	Ifrd2	1E-70	1.0
Slco2a1	8E-05	0.3	Tnks	4E-32	0.3	Lcorl	8E-61	1.0	Hras	1E-157	1.0
Gm765	6E-06	0.3	Msmo1	1E-21	0.3	Tpd52	6E-40	1.0	Sun2	3E-65	1.0
Ten1	1E-16	0.3	Washc3	3E-34	0.3	H60b	4E-19	1.0	Grin3a	1E-33	1.0
Tnks	6E-59	0.3	Vcl	2E-13	0.3	Pkp4	2E-59	1.0	Nhsl1	1E-51	1.0
Cdk19	4E-21	0.3	Klf2	8E-07	0.3	Tmem200a	3E-15	1.0	Usp37	3E-84	1.0
Dtx3l	2E-15	0.3	Ccdc12	8E-33	0.3	Ston2	7E-19	1.0	Rhebl1	3E-40	1.0
Slc16a2	2E-73	0.3	Gstt3	2E-10	0.3	Ubr7	2E-69	1.0	Cln6	1E-57	1.0
Uprt	2E-07	0.3	Pdlim7	3E-33	0.3	Cep290	4E-71	1.0	Arhgdig	4E-27	1.0
Morrbid	8E-14	0.3	Zc3h12a	7E-04	0.3	Schip1	7E-48	1.0	9030624G23Rik	1E-32	1.0
Fam228b	4E-06	0.3	Zfp469	4E-06	0.3	Lyrm9	4E-31	1.0	Sclt1	3E-72	1.0
Smpdl3a	6E-73	0.3	Rgma	8E-10	0.3	Psat1	4E-87	1.0	Zmat1	3E-54	1.0
Ppp1r3c	1E-05	0.3	Tmeff1	3E-11	0.3	Wdr35	1E-58	1.0	Slc35g2	3E-22	1.0
Tmem159	1E-31	0.3	Icam1	4E-18	0.3	Rnf219	1E-52	1.0	Sfmbt1	3E-74	1.0
H2-M3	3E-10	0.3	Tango2	2E-13	0.3	Gclc	2E-57	1.0	Smchd1	6E-149	1.0
Tmeff1	7E-21	0.3	Tmem123	2E-09	0.3	Gm10053	5E-16	1.0	Snrnp25	7E-75	1.0
Prrx2	9E-123	0.3	Rhoj	4E-33	0.3	Zw10	6E-48	1.0	Ankrd54	9E-48	1.0
H2-K1	7E-149	0.3	1110046J04Rik	4E-11	0.3	Cyp2r1	1E-25	1.0	Hnrnpd	7E-114	1.0
Nckipsd	2E-03	0.3	Cd55	6E-41	0.3	Orc2	7E-67	1.0	Dock11	6E-79	1.0
Gm20631	1E-13	0.3	Sigmar1	5E-24	0.3	Herc6	6E-61	1.0	Ptchd4	9E-47	1.0

Undefined						Articular/Synovial Fibroblast					
Control			NICD2			Control			NICD2		
Gene	*p*	FC	Gene	*p*	FC	Gene	*p*	FC	Gene	*p*	FC
Akt1s1	2E-05	0.2	Rcl1	6E-01	0.2	Adamts12	2E-01	0.5	Ap5z1	5E-01	0.5
Raet1e	2E-01	0.2	Gdf15	3E-01	0.2	Tmem198b	3E-01	0.5	Ctsf	7E-10	0.5
Atxn3	1E-01	0.2	Rab29	7E-01	0.2	Klhl13	5E-06	0.5	Tmem126b	2E-03	0.5
Slc27a3	1E-01	0.2	Atg14	6E-02	0.2	Sdc1	7E-03	0.5	Srgap3	7E-13	0.5
Scarf2	2E-03	0.2	Slc35c2	2E-01	0.2	Aldh4a1	8E-01	0.5	Map1lc3b	2E-35	0.5
Gm12258	1E+00	0.2	Arl5b	3E-01	0.2	Gnpda1	2E-03	0.5	Zfp467	1E-01	0.5
Nckap5	7E-05	0.2	Kdm4a	2E-01	0.2	Adamts2	2E-06	0.5	Dazap2	1E-05	0.5
Blzf1	2E-01	0.2	Lars2	5E-01	0.2	Pon3	2E-11	0.5	Vsir	7E-01	0.5
Id3	4E-14	0.2	Pltp	6E-06	0.2	Zeb1	6E-15	0.5	Cdc42bpg	2E-01	0.5
Kazald1	5E-05	0.2	Prkab1	5E-01	0.2	Klf10	2E-02	0.5	Rnh1	2E-18	0.5
Dgke	8E-01	0.2	0610043K17Rik	8E-01	0.2	Rev3l	1E-13	0.5	Zfp503	5E-10	0.5
Nt5dc3	7E-02	0.2	Rxrb	1E+00	0.2	Abhd14b	2E-04	0.5	Ei24	4E-08	0.5
Eya1	5E-04	0.2	Csad	9E-09	0.2	Carhsp1	2E-10	0.5	Car5b	2E-02	0.5
A930007I19Rik	9E-01	0.2	B3galt6	8E-01	0.2	Zeb2os	9E-01	0.5	Kdm5d	3E-02	0.5
Rgl2	2E-01	0.2	Xpr1	1E-01	0.2	Ikbkg	3E-03	0.5	Ropn1l	9E-01	0.5
Nkiras2	6E-01	0.2	Nmrk1	8E-01	0.2	Tbx5	2E-01	0.5	Rex1bd	7E-28	0.5
Parp8	7E-04	0.2	Pigk	1E-04	0.2	Ttc39c	5E-02	0.5	Oxld1	7E-02	0.5
Arl16	2E-01	0.2	Ppic	2E-17	0.2	Zfp563	6E-01	0.5	Pla2g15	7E-02	0.5
Adamts4	4E-01	0.2	Tyk2	6E-01	0.2	Spsb2	8E-09	0.5	Cercam	2E-03	0.5
Rbm43	6E-02	0.2	Rabif	7E-01	0.2	Wdsub1	7E-03	0.5	Col3a1	8E-24	0.5
Nupr1	1E-21	0.2	Nr1h2	4E-01	0.2	Vstm4	1E-01	0.5	Matn2	8E-05	0.5
Slc29a1	2E-03	0.2	Cox19	3E-03	0.2	Rnase4	2E-12	0.5	Azin2	1E-02	0.5
Cd109	4E-06	0.2	Farp1	1E-06	0.2	D830024N08Rik	5E-01	0.5	Kat2b	4E-03	0.5
Ring1	4E-02	0.2	Sept1	9E-01	0.2	Gmip	2E-01	0.5	Tbc1d32	7E-02	0.5
Usp20	6E-01	0.2	Naa25	5E-01	0.2	Gm32618	2E-02	0.5	Zfp524	2E-01	0.5
Rbbp8	6E-02	0.2	Psmd8	2E-07	0.2	AC149090.1	4E-15	0.5	Setdb2	8E-01	0.5
Ttc1	6E-03	0.2	Haghl	3E-01	0.2	Tspan5	3E-09	0.5	Usp21	9E-01	0.5
Hoxd8	3E-01	0.2	Fndc3a	2E-11	0.2	Serping1	4E-10	0.5	Rragb	7E-01	0.5
Ubxn6	1E-04	0.2	Polr2e	1E-05	0.2	Kif6	3E-01	0.5	Epb41l1	4E-02	0.5
Bloc1s6	1E-01	0.2	Gadd45a	9E-01	0.2	Myo7a	5E-01	0.5	Abcd1	2E-01	0.5
Mat2b	8E-06	0.2	Gstm1	1E-06	0.2	Taok3	3E-08	0.5	Bcl2l11	4E-02	0.5
Snrpn	1E+00	0.2	Mfsd11	9E-01	0.2	Tsga10	1E-01	0.5	Uaca	6E-02	0.5
Tefm	4E-01	0.2	A4galt	2E-01	0.2	Eya4	8E-22	0.5	Kif9	2E-01	0.5
Ap4b1	6E-01	0.2	Borcs7	3E-03	0.2	Gfra4	6E-03	0.5	Trafd1	3E-06	0.5
Ifih1	6E-01	0.2	Chmp1b	4E-01	0.2	Gabarapl1	8E-10	0.5	Hist4h4	8E-01	0.5
Wls	7E-22	0.2	Zfpl1	2E-01	0.2	Prelid2	1E-03	0.5	Sugct	1E-02	0.5
Dhodh	3E-01	0.2	Bckdk	3E-02	0.2	Arhgdib	3E-04	0.5	Gfra4	3E-01	0.5
Tmem223	2E-08	0.2	Arl15	3E-04	0.2	Dclre1c	8E-01	0.5	Map3k1	5E-01	0.5
Dock6	4E-01	0.2	Atp6v1g1	1E-15	0.2	Abhd18	2E-03	0.5	Tspan17	3E-06	0.4
Rad52	5E-01	0.2	B230118H07Rik	5E-05	0.2	Calcrl	3E-01	0.5	Ndrg1	2E-03	0.4
Olfm1	2E-03	0.2	Rex1bd	6E-21	0.2	Ostm1	3E-02	0.5	Btd	1E-02	0.4
Spred3	7E-01	0.2	Igfbp2	7E-01	0.2	Psd4	5E-01	0.5	Olfml1	4E-03	0.4
6430590A07Rik	3E-01	0.2	Sord	7E-01	0.2	Tifa	4E-01	0.5	Glul	4E-01	0.4
Morn2	4E-04	0.2	Crtc1	6E-01	0.2	Exosc7	3E-10	0.5	Rab6b	5E-01	0.4
Me3	9E-02	0.2	Ccser2	1E-07	0.2	Ccdc85b	5E-19	0.4	Oxa1l	1E-02	0.4
Dhx35	1E+00	0.2	Efna4	4E-01	0.2	Calhm2	1E-01	0.4	Gxylt2	4E-04	0.4
Commd9	7E-01	0.2	Llph	2E-05	0.2	Klhdc8b	2E-01	0.4	Mgmt	6E-10	0.4
Prpsap2	3E-01	0.2	Selenow	6E-18	0.2	Mapre2	1E-05	0.4	Camkmt	6E-02	0.4
Gm16541	9E-01	0.2	4921504A21Rik	1E+00	0.2	Fbn1	2E-29	0.4	Plaat3	3E-10	0.4
Ppic	2E-22	0.2	Nme4	7E-03	0.2	Thbd	9E-03	0.4	Cyp4f16	5E-01	0.4
Yju2	7E-01	0.2	Rnf113a1	7E-01	0.2	Rab42	6E-01	0.4	Gm17382	5E-01	0.4
1700052K11Rik	2E-01	0.2	4933407K13Rik	9E-01	0.2	Renbp	1E-06	0.4	Txnip	3E-09	0.4
2310022A10Rik	1E-01	0.2	Akt1s1	1E-03	0.2	Slc6a8	9E-04	0.4	Mef2a	4E-15	0.4
Ada	6E-01	0.2	Lhfpl2	3E-03	0.2	Epb41l1	6E-03	0.4	Stard9	2E-01	0.4
Cebpb	6E-07	0.2	Nifk	1E-02	0.2	Lekr1	4E-02	0.4	D130040H23Rik	6E-02	0.4
Ctsb	1E-59	0.2	Rnf130	8E-03	0.2	Cercam	2E-06	0.4	Ier5l	9E-05	0.4
Sardh	2E-02	0.2	Pask	2E-03	0.2	Syt12	7E-02	0.4	Gm15411	7E-01	0.4
3110021N24Rik	3E-01	0.2	Dhps	4E-01	0.2	Usp13	6E-01	0.4	Pcsk6	8E-02	0.4
Glrb	4E-01	0.2	Ciapin1	5E-01	0.2	Arhgef6	1E-02	0.4	Pomgnt2	4E-01	0.4
St6galnac4	2E-01	0.2	Ramac	1E-03	0.2	Acot13	1E-11	0.4	Ccdc28b	7E-03	0.4
Zfp763	2E-01	0.2	Rnf25	4E-02	0.2	Sox5	2E-11	0.4	Morn2	6E-03	0.4
Arl15	2E-02	0.2	Fry	1E-02	0.2	Hacl1	4E-03	0.4	Zfp808	2E-01	0.4
Tango6	9E-01	0.2	Gbe1	6E-01	0.2	Zfp503	2E-11	0.4	Dock9	3E-03	0.4
Sirt5	3E-01	0.2	Zcchc14	4E-02	0.2	Zbtb43	6E-02	0.4	Amdhd2	3E-02	0.4
Rabl2	9E-01	0.2	Dmac2l	9E-01	0.2	Dag1	2E-19	0.4	Nox4	4E-03	0.4
Zfand1	2E-01	0.2	Rogdi	9E-01	0.2	Ighmbp2	3E-01	0.4	Mcee	6E-08	0.4
Taco1os	9E-01	0.2	Rtl8a	1E-02	0.2	Ifitm3	1E-38	0.4	Adam9	8E-08	0.4
Eno3	6E-04	0.2	Ankrd16	9E-01	0.2	Car5b	2E-01	0.4	Plekhg5	3E-04	0.4
Smpd1	1E-04	0.2	Phf1	5E-01	0.2	Abcd1	4E-02	0.4	Zfp385b	6E-02	0.4
Cxcl1	4E-02	0.2	Nt5dc3	4E-01	0.2	Arhgef9	5E-02	0.4	Pros1	1E-08	0.4
Apbb1	2E-01	0.2	Patj	8E-01	0.2	Rab36	6E-01	0.4	Vstm4	3E-02	0.4
Tmem65	5E-05	0.2	Tiam2	6E-13	0.2	Hmga1	1E-01	0.4	Fbxl18	6E-01	0.4
Synrg	6E-03	0.2	Ecm1	3E-08	0.2	Cpeb1	1E-05	0.4	Rtp4	4E-01	0.4

Limb Mesenchyme

Control Gene	p	FC	NICD2 Gene	p	FC
Limch1	1E-21	0.4	Scyl3	1E-10	0.4
Uggt1	3E-17	0.4	Clba1	1E-05	0.4
Utp25	1E-07	0.4	Acot7	2E-10	0.4
Vgll3	5E-20	0.4	Leng9	9E-03	0.4
Fbxw8	1E-15	0.4	Ctnnal1	1E-08	0.4
Piezo2	1E-23	0.4	Lsg1	2E-10	0.4
Gm15559	1E-04	0.4	Slpi	2E-21	0.4
Tmem161b	7E-10	0.4	Gdpd1	4E-07	0.4
Fra10ac1	3E-13	0.4	Otulinl	1E-04	0.4
Zfp9	3E-14	0.4	Zfp276	2E-03	0.4
Ttc7b	1E-09	0.4	Rel	7E-09	0.4
Gtf2ird1	2E-13	0.4	1810013L24Rik	3E-12	0.4
Ssr3	6E-50	0.4	Mapkap1	1E-27	0.4
Sim2	6E-04	0.4	Entpd7	2E-09	0.4
Meis2	6E-05	0.4	Eml6	5E-07	0.4
Zfp169	5E-05	0.4	Gm50013	4E-06	0.4
Acot7	2E-11	0.4	Lig4	4E-05	0.4
Gm45669	1E-04	0.4	Slc12a4	9E-09	0.4
Tes	3E-09	0.4	Tbpl1	4E-12	0.4
Sept2	4E-11	0.4	Ahcyl1	3E-25	0.4
Lmx1b	3E-08	0.4	Gm26936	2E-09	0.4
Zfp521	3E-30	0.4	Slc1a6	6E-07	0.4
Tnk2	1E-04	0.4	Tjap1	5E-09	0.4
Atp8a1	9E-06	0.4	Tspan18	2E-08	0.4
Ddx31	7E-07	0.4	Mir100hg	2E-30	0.4
Thoc5	1E-09	0.4	Plxna1	2E-08	0.4
Mettl17	2E-06	0.4	Tmem100	1E-05	0.4
Snhg17	2E-07	0.4	Nepro	3E-04	0.4
Crim1	9E-21	0.4	3110009E18Rik	1E-05	0.4
Bmpr1b	2E-07	0.4	Asb7	9E-05	0.4
Brd3os	2E-07	0.4	Paxbp1	3E-11	0.4
Nbas	1E-16	0.4	Wdr1	1E-16	0.4
Lats1	2E-14	0.4	Trim8	2E-08	0.4
Fzd7	6E-09	0.4	Sh3rf1	3E-13	0.4
Tigar	1E-04	0.4	Papss2	1E-12	0.4
Sec31a	2E-44	0.4	Igip	6E-07	0.4
Hnrnpab	3E-40	0.4	Arhgef19	3E-07	0.4
Tmem132a	7E-12	0.4	Tbx15	1E-18	0.4
Thbd	2E-15	0.4	Lyrm1	8E-07	0.4
Pigg	4E-06	0.4	Dcaf12	2E-07	0.4
Top2b	1E-32	0.4	Atxn2l	9E-17	0.4
BC023719	4E-09	0.4	Hax1	3E-17	0.4
Fermt2	4E-18	0.4	Stt3b	2E-19	0.4
Hist2h4	1E-08	0.4	Sgk3	4E-13	0.4
Hemk1	3E-04	0.4	Serp1	2E-24	0.4
Exosc2	3E-05	0.4	Dzip1l	2E-08	0.4
Gm17066	2E-03	0.4	Ufsp1	1E-04	0.4
Mdm1	7E-03	0.4	Thnsl1	4E-05	0.4
Runx2os1	6E-07	0.4	D630045J12Rik	4E-12	0.4
Gm10130	2E-04	0.4	Rbm19	8E-06	0.4
Nckap5	6E-18	0.4	Pus7	7E-10	0.4
Gm13684	3E-05	0.4	4933431E20Rik	2E-05	0.4
Eif4e	4E-24	0.4	Snx7	2E-14	0.4
Snd1	3E-28	0.4	Itga10	3E-10	0.4
Fzd1	4E-11	0.4	Sprtn	8E-06	0.4
Gm16541	2E-03	0.4	Prkx	7E-05	0.4
Hs6st1	9E-09	0.4	Vezt	2E-09	0.4
Ncald	4E-06	0.4	Mpzl1	6E-09	0.4
Snhg14	7E-05	0.4	Dlg1	1E-16	0.4
C030006K11Rik	5E-08	0.4	Hes6	3E-10	0.4
Pex11g	2E-06	0.4	Sgms1	9E-12	0.4
0610009E02Rik	1E-09	0.4	Bcr	1E-07	0.4
Rbm4	8E-14	0.4	Fbxl20	6E-11	0.4
Ldah	5E-19	0.4	Acadsb	2E-18	0.4
Inpp5b	4E-10	0.4	Fxr2	3E-08	0.4
Ncdn	3E-07	0.4	Prps1l3	2E-05	0.4
Snhg16	1E-07	0.4	Vta1	4E-17	0.4
Zfr	3E-33	0.4	Kyat1	5E-03	0.4
Tprn	3E-04	0.4	Zzz3	2E-17	0.4
Pacc1	1E-04	0.4	Mef2c	2E-12	0.4
Fkrp	7E-10	0.4	Wdr36	4E-10	0.4
Arhgef19	3E-09	0.4	Ipo7	2E-18	0.4
A530017D24Rik	1E-03	0.4	Hivep1	4E-09	0.4

Chondrogenic

Control Gene	p	FC	NICD2 Gene	p	FC
Zswim1	2E-03	0.4	Malat1	3E-114	0.4
Rin1	1E-01	0.4	Wdr59	3E-02	0.4
Cpeb4	4E-08	0.4	Golph3	3E-13	0.4
Hipk2	5E-20	0.4	Tmsb15b1	5E-04	0.4
Sun2	1E-08	0.4	Antxr2	2E-11	0.4
Mtss1	4E-02	0.4	Mme	5E-31	0.4
Pakap	2E-10	0.4	Pawr	9E-18	0.4
6330418K02Rik	2E-03	0.4	Pdrg1	7E-23	0.4
Cyb5d2	2E-04	0.4	Synrg	7E-08	0.4
Phc2	4E-15	0.4	Spats2l	2E-05	0.4
Kmt2e	2E-26	0.4	Skil	1E-04	0.4
2900076A07Rik	1E-06	0.4	Acad11	2E-05	0.4
Gm17484	1E-02	0.4	Gm20404	8E-02	0.4
Atp6v1a	1E-33	0.4	Lum	8E-18	0.4
4930550C14Rik	3E-03	0.4	Lonrf1	4E-02	0.4
Hoxa11os	2E-03	0.4	Pkig	2E-17	0.4
Limk2	4E-07	0.4	Piezo2	2E-17	0.4
Rhbdd2	3E-04	0.4	Foxp2	1E-16	0.4
Gls	5E-28	0.4	Pigb	4E-04	0.4
Sipa1l1	2E-24	0.4	Csgalnact1	1E-21	0.4
Gm19710	3E-04	0.4	Grk4	2E-03	0.4
Itgb8	3E-04	0.4	Lemd3	2E-04	0.4
Sppl2a	3E-19	0.4	Ring1	1E-10	0.4
Sh3bp4	4E-02	0.4	Clip4	2E-05	0.4
Gm9725	1E-02	0.4	Fndc1	5E-03	0.4
Rsf1os1	4E-02	0.4	Fut8	1E-05	0.4
1810059H22Rik	2E-02	0.4	Clmn	3E-04	0.4
Galnt5	7E-03	0.4	Zfp141	2E-03	0.4
Pdcl	5E-10	0.4	Ndrg3	2E-08	0.4
Ubiad1	3E-02	0.4	Rundc1	2E-02	0.4
Cspg4	1E-05	0.4	Morn1	4E-02	0.4
Zfp521	1E-42	0.4	Cdc42se2	1E-11	0.4
Tbpl1	2E-11	0.4	Saraf	3E-15	0.4
Malat1	2E-125	0.4	Hspb2	7E-08	0.4
Klhl24	3E-15	0.4	Fmn1	2E-18	0.4
Ypel1	4E-04	0.4	Herpud1	2E-19	0.4
Rwdd3	4E-03	0.4	Sdf2l1	1E-11	0.4
Patj	2E-05	0.4	Mblac2	4E-03	0.4
Antxr1	1E-15	0.4	Pitpnc1	8E-15	0.4
Gm13986	3E-04	0.4	Raly	2E-25	0.4
Sorbs3	3E-08	0.4	Tmem238	7E-07	0.4
Gtpbp8	4E-10	0.4	Lipo3	6E-03	0.4
Hist1h2ac	2E-04	0.4	Ttc39b	6E-10	0.4
Mbnl2	8E-59	0.4	Gm26749	4E-04	0.4
0610009E02Rik	1E-03	0.4	Sp4	8E-03	0.4
Ripk2	3E-06	0.4	Nub1	2E-20	0.4
Tbc1d22a	4E-06	0.4	Dnmt3a	6E-07	0.4
Zfp516	4E-15	0.4	Plekhb2	1E-01	0.4
Upf3b	6E-14	0.4	Efcab7	2E-01	0.4
Tmem176a	2E-38	0.4	Neat1	6E-27	0.4
Gm26944	5E-02	0.4	Zfp637	1E-15	0.4
Rab4a	3E-06	0.4	Fgfbp3	2E-02	0.4
Dbndd2	3E-06	0.4	Snx18	9E-10	0.4
Rdh10	1E-02	0.4	Mxra8	2E-33	0.4
Gm16124	4E-05	0.4	Megf9	4E-03	0.4
Rab39b	8E-03	0.4	Adam32	1E-01	0.4
Ccdc62	1E-05	0.4	Sun2	1E-05	0.4
Sertad4	4E-03	0.4	Gm15564	2E-04	0.4
Os9	2E-37	0.4	Zfpm2	1E-17	0.4
B230217O12Rik	3E-03	0.4	Slc22a21	1E-01	0.4
Gm15867	9E-05	0.4	Gm15222	1E-02	0.4
Smpd1	2E-15	0.4	Klhl11	2E-03	0.4
Msl3l2	4E-04	0.4	Agbl3	2E-02	0.4
Ivns1abp	3E-11	0.4	4930481A15Rik	3E-02	0.4
Dhx58os	2E-02	0.4	1700088E04Rik	4E-02	0.4
Lmo4	2E-25	0.4	Gm20536	5E-02	0.4
Gm46367	8E-03	0.4	Slc25a53	9E-02	0.4
Arhgef19	3E-04	0.4	Ston1	4E-07	0.4
Gm15952	2E-04	0.4	Acyp1	4E-15	0.4
A430018G15Rik	9E-03	0.4	B3gnt2	1E-05	0.4
Spred3	2E-03	0.4	Ccdc189	4E-03	0.4
Foxd2os	1E-06	0.4	Proser1	2E-03	0.4
Lrp5	5E-04	0.4	Crtc2	1E-01	0.4

Fibroblast

Control Gene	p	FC	NICD2 Gene	p	FC
Atp2b4	4E-14	0.3	Manea	2E-11	0.3
Slc50a1	1E-110	0.3	Sat2	7E-09	0.3
Unc5b	2E-04	0.3	Fmnl2	6E-32	0.3
Utp14b	4E-04	0.3	Trim16	2E-05	0.3
Slc35f5	6E-21	0.3	Pdk3	1E-22	0.3
Mxra7	1E-93	0.3	Eepd1	2E-04	0.3
Prkar1b	5E-13	0.3	Gm20721	1E-06	0.3
Snx10	9E-21	0.3	Gm14295	1E-05	0.3
Tmem106a	6E-12	0.3	Clmp	4E-40	0.3
Lamc1	2E-47	0.3	Tubb2b	6E-23	0.3
Tpm1	3E-86	0.3	Akap13	3E-39	0.3
Cd302	4E-85	0.3	Dtx3	1E-20	0.3
Eml4	3E-49	0.3	H2-Q7	1E-04	0.3
Ankrd27	3E-17	0.3	Dbp	1E-26	0.3
Mpdz	7E-59	0.3	Alg11	4E-07	0.3
2510009E07Rik	1E-23	0.3	Mroh1	5E-11	0.3
Pdgfra	2E-73	0.3	Aldh1l1	2E-09	0.3
Fam117a	9E-11	0.3	Txnrd3	3E-04	0.3
Tmem86a	3E-11	0.3	Abcd2	6E-07	0.3
Cdc42ep4	3E-05	0.3	Fdxr	7E-04	0.3
Epha3	5E-08	0.3	Abcc5	1E-20	0.3
Zfp608	5E-44	0.3	Tmem168	3E-07	0.3
Acly	1E-50	0.3	Fam161a	2E-03	0.3
Klf16	1E-04	0.3	Mefd2	2E-16	0.3
Icam1	4E-32	0.3	Pcsk6	1E-08	0.3
Abcc5	5E-41	0.3	Afap1	3E-35	0.3
Gm4117	4E-11	0.3	Hmgcs1	2E-28	0.3
Prkar2b	4E-81	0.3	Ggcx	3E-15	0.3
Pip4k2a	3E-25	0.3	Sptlc2	7E-23	0.3
Tsku	2E-07	0.3	Gm49359	1E-08	0.3
Gm14296	9E-12	0.3	Gsn	1E-95	0.3
Col8a2	2E-11	0.3	Mpdz	9E-30	0.3
2410006H16Rik	3E-123	0.3	Osmr	1E-30	0.3
Rxfp1	6E-07	0.3	Atp13a3	9E-16	0.3
Zfp442	3E-08	0.3	Gm44148	9E-06	0.3
Adgrl2	1E-61	0.3	Tead1	9E-41	0.3
Wnt5a	1E-30	0.3	Fam171a1	2E-06	0.3
Camk2n1	1E-13	0.3	Otulinl	3E-05	0.3
Mios	2E-16	0.3	Trim12c	4E-12	0.3
Palm	3E-18	0.3	Gm14296	8E-06	0.3
Add1	2E-101	0.3	Fdps	4E-22	0.3
Cpt2	1E-20	0.3	Hopx	9E-18	0.3
Prdx4	3E-84	0.3	Fbn2	8E-30	0.3
Tnks1bp1	8E-36	0.3	Cers6	4E-20	0.3
Sh3bgrl3	4E-169	0.3	Wwc1	2E-02	0.3
Flot1	8E-64	0.3	Rcn1	7E-66	0.3
Cbr3	5E-49	0.3	Slc50a1	6E-54	0.3
Fbn2	5E-58	0.3	Tmem106a	5E-05	0.3
Tspan4	2E-93	0.3	Tns1	1E-15	0.3
Agtr2	3E-15	0.3	H2-DMa	9E-08	0.3
Synj2	2E-06	0.3	Cemip2	4E-13	0.3
Pigc	3E-19	0.3	Map1a	2E-27	0.3
Mlxip	1E-12	0.3	Zfp608	3E-22	0.3
Fancf	4E-12	0.3	Adcy2	2E-05	0.3
Tprkb	2E-28	0.3	Sh3bgrl3	2E-83	0.3
Ptgs1	4E-08	0.3	Fancf	9E-08	0.3
Sbsn	7E-49	0.3	Tmem86a	3E-06	0.3
Nrn1	2E-40	0.3	4732440D04Rik	3E-05	0.3
Fat3	2E-11	0.3	Snx9	9E-33	0.3
Gpx8	3E-124	0.3	Pigc	2E-11	0.3
Ccdc6	1E-27	0.3	Nbea	3E-28	0.3
Rnf145	2E-17	0.3	Gm15446	9E-03	0.3
Rara	1E-26	0.3	Cdc14a	4E-07	0.3
Tead1	4E-68	0.3	Gpx8	2E-63	0.3
Gca	4E-08	0.3	Ostm1	3E-07	0.3
Tusc1	4E-07	0.3	Col1a1	2E-96	0.3
Dsel	1E-31	0.3	Blvrb	1E-58	0.3
Wnt5b	8E-02	0.3	Ttc39c	4E-06	0.3
Nln	1E-26	0.3	Gaa	2E-45	0.3
Itga1	6E-32	0.3	Gm16364.1	3E-07	0.3
Nradd	2E-32	0.3	Frrs1	8E-08	0.3
Sptlc2	1E-38	0.3	Ccnyl1	2E-07	0.3
Ccdc93	1E-20	0.3	Rara	5E-13	0.3

Fibroblast?

Control Gene	p	FC	NICD2 Gene	p	FC
Tubgcp2	2E-71	1.0	Casp8	1E-87	1.0
Gstcd	3E-62	1.0	Rem1	9E-20	1.0
Ddah1	3E-67	1.0	Plp2	6E-91	1.0
1700006J14Rik	4E-20	1.0	Pald1	1E-31	1.0
Kpnb1	4E-95	1.0	Baz1a	1E-73	1.0
Sun2	4E-44	1.0	Afap1l2	6E-29	1.0
Aard	4E-22	1.0	Pomt1	3E-46	1.0
Plb1	3E-14	1.0	Aarsd1	2E-111	1.0
Tagln2	2E-132	1.0	Tmem107	7E-80	1.0
Pom121	8E-51	1.0	Zw10	8E-57	1.0
Sipa1	3E-47	1.0	Nup37	1E-77	1.0
Igf2bp1	8E-38	1.0	Gsr	3E-81	1.0
Siva1	1E-159	1.0	Myh10	9E-108	1.0
Ccdc61	3E-50	1.0	Rap1gap2	3E-47	1.0
Nop56	8E-68	1.0	Mcm9	1E-57	1.0
Pla2g2e	4E-10	1.0	Pik3cb	5E-50	1.0
Tekt2	4E-23	1.0	Cycs	8E-137	1.0
Gm21887	8E-16	1.0	Slc16a10	2E-32	1.0
Nectin1	4E-39	1.0	Psat1	7E-94	1.0
Srsf7	1E-131	1.0	Chst1	2E-39	0.9
Uhrf2	8E-76	1.0	Jazf1	7E-49	0.9
Nsg1	9E-90	1.0	Nans	5E-81	0.9
Fbxo45	1E-50	1.0	Met	2E-26	0.9
Lmf2	3E-55	1.0	Supt16	2E-165	0.9
Tpm4	4E-136	1.0	Lmf2	4E-69	0.9
Usp37	4E-63	1.0	1600010M07Rik	3E-69	0.9
Mrpl18	2E-145	1.0	Crip2	5E-74	0.9
Ift80	6E-46	1.0	Ppm1d	9E-66	0.9
Pxylp1	2E-28	1.0	Rarres1	2E-26	0.9
Iqgap2	7E-30	1.0	Grk6	3E-64	0.9
Adh7	5E-22	1.0	Hnrnpab	7E-210	0.9
Gemin2	8E-56	1.0	Ipo5	2E-96	0.9
Nans	5E-66	1.0	4930478M13Rik	9E-18	0.9
Arhgdig	3E-30	1.0	Uba2	7E-89	0.9
Klf5	1E-24	1.0	Trp53i11	5E-19	0.9
Synpo2	1E-18	1.0	Fosl1	6E-48	0.9
Chd1l	2E-25	1.0	Klhdc8a	1E-38	0.9
Coro1c	6E-49	1.0	Vrk2	2E-80	0.9
Usp49	1E-43	0.9	Slco3a1	7E-73	0.9
Sgms2	8E-28	0.9	3300005D01Rik	1E-18	0.9
Ccdc74a	1E-26	0.9	Ssna1	2E-134	0.9
Hras	9E-117	0.9	Ccnd1	2E-68	0.9
Samd12	1E-28	0.9	Prps1	2E-65	0.9
Hnrnpab	1E-140	0.9	Pkia	8E-41	0.9
Srsf2	4E-123	0.9	Cyyr1	1E-18	0.9
Galm	3E-34	0.9	Glrb	4E-38	0.9
Net1	9E-61	0.9	2900026A02Rik	4E-43	0.9
Tslp	4E-40	0.9	Tbc1d1	2E-66	0.9
Slc6a17	1E-29	0.9	Tslp	6E-16	0.9
1810037I17Rik	3E-138	0.9	Rbm15	2E-65	0.9
Elfn1	3E-14	0.9	Nup93	9E-76	0.9
Rexo5	1E-33	0.9	Msx2	2E-18	0.9
Psmb9	1E-27	0.9	Dzip1l	2E-66	0.9
Ssna1	1E-101	0.9	1700007K13Rik	5E-34	0.9
Rapgef4	4E-21	0.9	Srrt	3E-95	0.9
Nek10	2E-10	0.9	Peg12	6E-18	0.9
Naa40	2E-43	0.9	Zfp946	8E-46	0.9
Slx4	1E-32	0.9	Gnb1l	2E-50	0.9
Gm5141	1E-25	0.9	Crnde	4E-28	0.9
Cav2	2E-61	0.9	Dpy30	2E-104	0.9
4833427G06Rik	7E-19	0.9	Herc3	7E-30	0.9
Fxyd5	1E-45	0.9	Zfp748	4E-21	0.9
Tuft1	1E-17	0.9	Sept10	8E-82	0.9
Il15	7E-11	0.9	Tmem129	1E-48	0.9
Sept10	1E-60	0.9	Smc1a	3E-181	0.9
H2-Q4	6E-36	0.9	D030056L22Rik	1E-66	0.9
Aarsd1	9E-84	0.9	BC052040	5E-71	0.9
Tmem107	2E-61	0.9	Gm5141	3E-33	0.9
Smc1a	5E-130	0.9	Nsmce1	1E-99	0.9
Acsl5	1E-40	0.9	Casp3	9E-85	0.9
Debld1	6E-45	0.9	C730034F03Rik	3E-08	0.9
Smchd1	2E-101	0.9	Gm13470	2E-37	0.9
Nup37	5E-46	0.9	Gper1	4E-18	0.9

Undefined

Control Gene	p	FC	NICD2 Gene	p	FC
Ctsa	6E-13	0.2	Fdx2	2E-05	0.2
Terf2ip	9E-01	0.2	Nudt12	9E-01	0.2
Chmp1b	8E-01	0.2	Setd7	2E-02	0.2
Bckdha	5E-02	0.2	Ypel5	4E-03	0.2
Exoc7	4E-03	0.2	Dmac2	1E+00	0.2
Ggnbp1	4E-01	0.2	Ctsf	5E-05	0.2
Ddah2	3E-06	0.2	Elmo1	5E-03	0.2
Crebrf	2E-04	0.2	Zmat5	1E-03	0.2
Ecm2	4E-03	0.2	Mrps35	3E-01	0.2
Nt5e	5E-01	0.2	Sfxn3	9E-02	0.2
Pls3	7E-05	0.2	Gsdme	3E-01	0.2
Wdr83	4E-02	0.2	Slc9a8	3E-01	0.2
Pcid2	7E-01	0.2	Fam162a	1E-06	0.2
Nr4a2	9E-01	0.2	Lmbrd1	1E-02	0.2
Slc46a1	9E-01	0.2	Fam151b	7E-01	0.2
Gramd1b	7E-01	0.2	Rarg	2E-03	0.2
Eng	5E-02	0.2	Ndufaf7	5E-01	0.2
Lipo3	4E-01	0.2	Tmem11	3E-01	0.2
Fam131a	5E-01	0.2	Cfap74	2E-01	0.2
Lrrc57	7E-01	0.2	Msi2	1E-21	0.2
Patz1	4E-01	0.2	Slc35e2	1E+00	0.2
4933407K13Rik	7E-01	0.2	Bbc3	1E-01	0.2
Wipi2	8E-03	0.2	Atg13	2E-01	0.2
2310015A10Rik	7E-01	0.2	Ube2e1	3E-08	0.2
Thap3	4E-04	0.2	Medag	2E-01	0.2
Gstp1	3E-05	0.2	Idh2	4E-06	0.2
Lrrc8a	3E-01	0.2	B230217O12Rik	4E-01	0.2
Cox19	7E-04	0.2	Glmp	5E-12	0.2
Nrep	8E-01	0.2	Fbxo32	8E-01	0.2
Adck5	3E-01	0.2	Cnksr3	2E-01	0.2
Atat1	5E-01	0.2	Golgb1	5E-16	0.2
Synm	2E-01	0.2	3110040N11Rik	6E-02	0.2
Slc39a10	9E-01	0.2	2510002D24Rik	1E-01	0.2
Cgref1	9E-03	0.2	Ppm1l	1E-01	0.2
Ntmt1	2E-01	0.2	Lrrc8a	3E-01	0.2
Gtf2h3	7E-01	0.2	Zfp775	3E-01	0.2
Pcbd2	2E-06	0.2	Zbtb17	7E-01	0.2
Mmp28	9E-01	0.2	BC065397	5E-01	0.2
Nudt9	1E-02	0.2	Apbb2	2E-13	0.2
Ankrd55	7E-01	0.2	Jup	4E-02	0.2
Prmt5	1E+00	0.2	Midn	1E-02	0.2
Zfp52	6E-01	0.2	Entpd7	6E-01	0.2
Anxa1	3E-04	0.2	Cyb5r4	8E-01	0.2
Nol3	3E-02	0.2	Hspb8	2E-01	0.2
Rnf185	8E-01	0.2	Heatr1	8E-01	0.2
Fgd6	5E-01	0.2	Txn2	8E-06	0.2
Cir1	3E-05	0.2	Dlc1	4E-08	0.2
Vamp7	6E-03	0.2	Rasa1	9E-04	0.2
Urm1	2E-01	0.2	Ercc1	7E-01	0.2
Tmem42	3E-01	0.2	Nr2c2ap	4E-01	0.2
Zscan26	1E-01	0.2	Fkbp14	3E-02	0.2
Zfp51	8E-01	0.2	Btd	2E-01	0.2
Pbxip1	3E-06	0.2	Plxna2	4E-01	0.2
Hdac3	5E-02	0.2	Sdcbp	3E-06	0.2
Rac3	4E-02	0.2	Ifit3	3E-01	0.2
Rtl8a	9E-02	0.2	Fan1	9E-01	0.2
Cnppd1	8E-02	0.2	Ube2e2	1E-06	0.2
Sorl1	6E-01	0.2	Abcc1	2E-01	0.2
Ikzf4	7E-01	0.2	Chchd4	8E-01	0.2
Comp	3E-19	0.2	Parp6	2E-01	0.2
Sun2	3E-01	0.2	Fkbp10	4E-05	0.2
Pvt1	2E-05	0.2	Creld1	2E-01	0.2
Lrrc20	8E-01	0.2	Ggps1	8E-02	0.2
Carmil1	1E-03	0.2	Haus2	3E-01	0.2
Trp53bp2	2E-01	0.2	Plekha8	3E-01	0.2
Atg13	3E-01	0.2	BC029722	9E-01	0.2
Hfe	1E-01	0.2	Fmnl3	6E-01	0.2
Mir22hg	2E-05	0.2	Clpp	2E-02	0.2
Bsg	5E-41	0.2	4833420G17Rik	4E-03	0.2
Lancl1	2E-02	0.2	Gusb	2E-01	0.2
Atg4d	8E-01	0.2	Zfp622	2E-03	0.2
Krba1	8E-01	0.2	Adgre5	1E-01	0.2
Setbp1	7E-17	0.2	Abitram	4E-01	0.2

Articular/Synovial Fibroblast

Control Gene	p	FC	NICD2 Gene	p	FC
Evc	5E-01	0.4	Kank2	2E-04	0.4
Col6a3	5E-22	0.4	Cpeb1	2E-02	0.4
Psrc1	4E-04	0.4	Snrnp35	2E-01	0.4
Gm13470	2E-01	0.4	Atg2a	6E-01	0.4
Ddx60	2E-02	0.4	Jund	4E-18	0.4
Tnip2	4E-01	0.4	Gas2	5E-12	0.4
Gbp3	9E-02	0.4	Parp3	6E-12	0.4
Pcmtd2	6E-07	0.4	Fndc3a	2E-19	0.4
Zdhhc3	1E-07	0.4	Prune2	1E+00	0.4
Smpdl3a	3E-12	0.4	Smcr8	7E-01	0.4
Als2	2E-01	0.4	Tapbpl	4E-01	0.4
Stard10	5E-03	0.4	Arsk	1E-01	0.4
Vcpkmt	5E-02	0.4	Bbs2	2E-01	0.4
Bicd2	2E-03	0.4	Aste1	6E-01	0.4
Bst2	5E-20	0.4	Eda2r	2E-01	0.4
1700030K09Rik	5E-02	0.4	Cage1	5E-01	0.4
Maml3	2E-02	0.4	Galnt18	1E-02	0.4
Acap3	1E-01	0.4	Xaf1	1E-03	0.4
Wdr24	5E-01	0.4	Siae	2E-01	0.4
Man2b2	6E-03	0.4	Tbc1d14	3E-01	0.4
4930412C18Rik	9E-01	0.4	Gm15738	6E-01	0.4
2610002M06Rik	3E-02	0.4	Rfx3	3E-01	0.4
Cryz	2E-02	0.4	Plekha8	3E-01	0.4
Ikbke	8E-02	0.4	Snx29	2E-02	0.4
Cdo1	1E-13	0.4	Optn	3E-02	0.4
Mttp	5E-01	0.4	Sgtb	9E-01	0.4
Amdhd2	2E-02	0.4	Prkca	5E-08	0.4
Gm16008	5E-01	0.4	Xpa	1E-06	0.4
Meis1	1E-01	0.4	Efna2	4E-01	0.4
Slc25a23	2E-02	0.4	Cnppd1	6E-03	0.4
Adamtsl4	2E-03	0.4	Naprt	6E-01	0.4
Twsg1	6E-09	0.4	Gmip	5E-01	0.4
Btd	5E-03	0.4	Wrb	2E-01	0.4
Sh2d4a	5E-03	0.4	Dusp16	4E-02	0.4
Ppm1l	1E-04	0.4	Rsph9	2E-01	0.4
Frrs1	1E-03	0.4	Mtmr1	8E-03	0.4
Gm20631	5E-01	0.4	Usp54	7E-01	0.4
Cpne2	8E-05	0.4	Tbccd1	1E+00	0.4
Katnal2	4E-01	0.4	Arhgef9	5E-01	0.4
Dlgap4	7E-08	0.4	Samd9l	2E-05	0.4
Slc36a1	2E-01	0.4	Herc6	3E-01	0.4
Chrna1os	7E-01	0.4	Zfp991	7E-01	0.4
Slc22a18	4E-01	0.4	Cerk	9E-01	0.4
Sfxn5	1E-01	0.4	Zfp946	8E-01	0.4
Cyp39a1	8E-01	0.4	Rpap1	7E-01	0.4
Cdc14b	2E-03	0.4	Zswim6	2E-06	0.4
Maoa	9E-09	0.4	Dcaf4	6E-01	0.4
Zdhhc1	2E-02	0.4	Dtnbp1	6E-17	0.4
Il17ra	1E-01	0.4	Gm26944	5E-01	0.4
Col4a2	9E-06	0.4	AU041133	2E-01	0.4
Glmp	5E-17	0.4	Gypc	3E-01	0.4
Hebp2	5E-04	0.4	Arfgef2	4E-05	0.4
1110008P14Rik	1E-20	0.4	Sesn1	3E-01	0.4
Nmnat3	5E-01	0.4	Tmem231	7E-01	0.4
Slc16a7	9E-01	0.4	Appl2	1E-01	0.4
Cdkn1a	8E-12	0.4	Dleu2	3E-11	0.4
Tcf12	8E-16	0.4	Lrrc32	1E-03	0.4
Zfp820	9E-02	0.4	Hacl1	4E-02	0.4
Cog7	3E-02	0.4	Zfp995	6E-01	0.4
Thra	2E-25	0.4	Caprin2	9E-01	0.4
Grn	6E-31	0.4	Sema3f	5E-01	0.4
Ephx1	2E-13	0.4	Runx1t1	2E-15	0.4
Mfsd12	7E-02	0.4	Akr1e1	7E-03	0.4
Rpusd1	9E-01	0.4	Txndc16	7E-03	0.4
Plxnd1	2E-01	0.4	Serping1	6E-05	0.4
Gm15834	9E-01	0.4	Zdhhc1	6E-02	0.4
Lrp3	2E-01	0.4	Neurl2	7E-01	0.4
Cebpa	3E-04	0.4	Mmp16	2E-03	0.4
Acad12	5E-01	0.4	Cpt1c	2E-02	0.4
Hykk	2E-01	0.4	Coro1b	8E-01	0.4
Pmp22	2E-19	0.4	Gm12743	2E-01	0.4
Shisa5	9E-15	0.4	Kif21a	1E-05	0.4
Tcn2	6E-07	0.4	Slc43a2	7E-01	0.4

Limb Mesenchyme					
Control			NICD2		
Gene	p	FC	Gene	p	FC
Acrbp	6E-04	0.4	Ppip5k2	1E-06	0.4
Fam98b	5E-14	0.4	Arhgap12	5E-11	0.4
Fcsk	7E-04	0.4	Gm11906	4E-04	0.4
Ppic	4E-38	0.4	Gsk3a	3E-08	0.4
Gm17529	4E-06	0.4	Rpp40	3E-04	0.4
Zbed5	1E-05	0.4	Atxn1l	2E-08	0.4
Ptgis	8E-17	0.4	Fbxo42	5E-11	0.4
Cnot8	3E-14	0.4	Fcsk	7E-04	0.4
Mib1	3E-17	0.4	Polk	2E-09	0.4
Tmem8	5E-06	0.4	Zfp521	3E-27	0.4
Vps37a	7E-13	0.4	Sft2d2	3E-08	0.4
Epb41	1E-09	0.4	Dnaja3	8E-10	0.4
Ctdspl	3E-16	0.4	Abce1	1E-17	0.4
Jade2	2E-08	0.4	Myo18a	4E-08	0.3
Gm43728	4E-04	0.4	Kif7	2E-03	0.3
Tmem47	2E-07	0.4	Gcn1	3E-09	0.3
Dus2	4E-08	0.4	Ssr3	5E-41	0.3
Smco4	5E-17	0.4	Snhg16	2E-05	0.3
Sgms1	3E-17	0.4	Ube2e1	5E-20	0.3
Agpat4	2E-14	0.4	Mafg	7E-09	0.3
Ccnd3	3E-17	0.4	Rhou	2E-05	0.3
Strbp	6E-20	0.4	Myo19	7E-04	0.3
Mir100hg	5E-31	0.4	Zcwpw2	1E-04	0.3
Grb14	2E-10	0.4	Zfp983	1E-07	0.3
Usp6nl	1E-09	0.4	Riox1	1E-04	0.3
Gemin5	2E-08	0.3	Vezf1	2E-15	0.3
Usp11	1E-09	0.3	Cdipt	1E-11	0.3
Ifi44	4E-10	0.3	Sec61a1	6E-12	0.3
Hdac4	3E-09	0.3	Tmem94	9E-05	0.3
Nox1	1E-04	0.3	Ctnna1	6E-24	0.3
Tead1	2E-28	0.3	Patz1	3E-08	0.3
Serpinh1	5E-75	0.3	4930556J24Rik	1E-03	0.3
Acss3	2E-05	0.3	Zfp518b	2E-06	0.3
Tab1	1E-07	0.3	Gm15559	2E-03	0.3
Ica1	7E-09	0.3	Zbtb33	2E-09	0.3
Sec23a	1E-22	0.3	Lrrc47	5E-08	0.3
Heatr1	9E-06	0.3	Gga3	2E-04	0.3
Ostc	1E-35	0.3	Ric3	3E-06	0.3
Cmtm4	1E-10	0.3	Ddx31	3E-05	0.3
Spats2l	4E-08	0.3	Necap2	5E-12	0.3
Ppp3r1	1E-16	0.3	Farp2	1E-12	0.3
Ube2e1	2E-24	0.3	Rbpj	3E-26	0.3
Rnd3	4E-28	0.3	Wars	2E-09	0.3
Prps1l3	2E-06	0.3	Prdm16	3E-09	0.3
Lrrc75b	3E-05	0.3	Dmwd	2E-08	0.3
Ptpn13	3E-09	0.3	Gtf2ird1	7E-13	0.3
Pitpnc1	7E-15	0.3	Tjp1	3E-16	0.3
Cul1	3E-20	0.3	Tdg	3E-09	0.3
Cars	4E-14	0.3	Sdr39u1	7E-09	0.3
Rnf24	4E-13	0.3	Wdr43	2E-14	0.3
Lman1	4E-52	0.3	Mknk1	2E-07	0.3
Gpr180	8E-13	0.3	Luzp1	9E-26	0.3
Wasf3	3E-09	0.3	Gm17106	1E-05	0.3
Ikzf4	6E-04	0.3	Ndufaf5	6E-07	0.3
Zbtb33	6E-11	0.3	Parg	4E-12	0.3
Ipo7	2E-14	0.3	Eif3j2	2E-05	0.3
Fam13b	9E-18	0.3	Sept2	2E-09	0.3
Chchd7	2E-21	0.3	Rnd3	2E-21	0.3
Rel	2E-08	0.3	Nrde2	2E-08	0.3
Gm10848	1E-06	0.3	Selenos	4E-42	0.3
Ccdc157	3E-09	0.3	Rnf113a1	1E-03	0.3
Cbr2	1E-17	0.3	6530409C15Rik	6E-06	0.3
Ero1l	8E-19	0.3	Slc16a4	4E-07	0.3
Dusp18	1E-06	0.3	S100a1	6E-28	0.3
Loxl4	5E-05	0.3	Dkk3	1E-22	0.3
Ndufaf4	3E-13	0.3	Eef1e1	7E-13	0.3
Hspb2	1E-11	0.3	N4bp2	6E-10	0.3
Ddx21	5E-20	0.3	Pls3	8E-16	0.3
Cog5	2E-15	0.3	5730522E02Rik	4E-06	0.3
Ibtk	2E-10	0.3	Mecom	5E-06	0.3
Sdad1	4E-10	0.3	Cnot8	2E-08	0.3
Gm15222	8E-11	0.3	Yars	3E-12	0.3
Auts2	3E-23	0.3	Sept7	7E-38	0.3

Chondrogenic					
Control			NICD2		
Gene	p	FC	Gene	p	FC
Rnf139	8E-08	0.4	Tm2d3	5E-12	0.4
C1qtnf4	6E-04	0.4	Ogt	6E-19	0.4
Hmgb3	2E-06	0.4	Wdr6	4E-07	0.4
Tmem67	3E-03	0.4	Tmco4	4E-04	0.4
Gm20300	5E-03	0.4	Fam126b	1E-04	0.4
Cib2	2E-15	0.4	Slc35e3	1E-02	0.4
Fkbp10	6E-30	0.4	Amy1	3E-02	0.4
Cox20	4E-35	0.4	Hoxc10	4E-14	0.4
Ccdc115	8E-07	0.4	Rmnd5a	5E-10	0.4
Gm15411	2E-02	0.4	Phc2	1E-09	0.4
Airn	8E-34	0.4	Adss	6E-20	0.4
Mtmr12	5E-05	0.4	Zrsr1	1E-02	0.4
Jmy	7E-16	0.4	Fn1	6E-43	0.4
Ptch1	2E-04	0.4	Gm50240	1E-02	0.4
Gm27017	1E-01	0.4	Slc25a23	2E-02	0.4
Rom1	2E-05	0.4	Bpgm	5E-04	0.4
Nub1	1E-26	0.4	Ldlrap1	1E-05	0.4
Smim3	2E-05	0.4	Slc4a4	2E-01	0.4
Kantr	8E-08	0.4	Zbtb40	7E-03	0.4
Zc4h2	7E-02	0.4	Ltbp3	3E-23	0.4
Pard3bos1	2E-03	0.4	Rasa2	1E-07	0.4
A930005H10Rik	3E-06	0.4	Rrp12	2E-02	0.4
Acad10	1E-02	0.4	Hoxc4	2E-04	0.4
4930549G23Rik	2E-03	0.4	Zer1	1E-03	0.4
Wdr44	2E-08	0.4	Slc41a1	1E-05	0.4
Lrrc49	2E-06	0.4	Ddx19b	1E-02	0.4
Neo1	2E-25	0.4	Pdlim4	5E-10	0.4
Atf6	1E-13	0.4	Pank1	2E-02	0.4
Ndrg3	2E-12	0.4	4930557K07Rik	4E-01	0.4
2700081O15Rik	7E-09	0.4	Oma1	3E-03	0.4
BC067074	7E-02	0.4	Acaa1b	5E-02	0.4
2810013P06Rik	3E-07	0.4	Acp6	5E-03	0.4
Slc41a1	2E-05	0.4	Lncpint	2E-11	0.4
Lysmd4	1E-05	0.4	Fmnl3	5E-05	0.4
Mapkap1	2E-37	0.4	Zc3hav1l	2E-02	0.4
Tmub1	1E-03	0.4	Wdr5b	1E-01	0.4
Eno3	6E-03	0.4	Tmem63a	8E-03	0.4
Zfp952	2E-05	0.4	Nucb2	3E-22	0.4
Slc25a38	2E-03	0.4	Gatd3a	2E-08	0.4
Lrig3	2E-22	0.4	LTO1	9E-05	0.4
Fundc1	1E-13	0.4	Txndc11	2E-04	0.4
Mertk	2E-03	0.4	Nrip2	7E-02	0.4
Bbs4	2E-03	0.4	Ccdc28b	7E-05	0.4
Ccpg1	6E-20	0.4	Kif7	5E-02	0.4
Bmpr1a	6E-33	0.4	Rhebl1	3E-01	0.4
Zbtb11os1	8E-05	0.4	Mef2a	3E-18	0.4
Skil	1E-04	0.4	Brd3os	3E-02	0.4
Zfp566	3E-03	0.4	Bmpr1a	8E-26	0.4
Il16	1E-05	0.4	Necap2	2E-11	0.4
Ogg1	9E-04	0.4	Srebf1	2E-04	0.4
Abhd8	1E-12	0.4	Hoxa7	3E-06	0.4
Katnal2	7E-02	0.4	Gpc6	2E-24	0.4
Mettl14	5E-05	0.4	Mpp7	5E-06	0.4
Braf	1E-23	0.4	Tpk1	9E-07	0.4
Cd109	6E-22	0.4	Vezt	3E-08	0.4
Net1	3E-04	0.4	Ldah	4E-10	0.4
Pithd1	1E-05	0.4	mt-Nd6	4E-20	0.4
Tmco4	3E-04	0.4	Tafa5	5E-08	0.4
Slc37a4	1E-03	0.4	Adat1	2E-01	0.4
Mirg	3E-04	0.4	2410022M11Rik	2E-02	0.4
Pkig	1E-25	0.4	Polr2l	9E-12	0.4
Tanc1	5E-09	0.4	Ptprk	3E-05	0.4
Gpbp1l1	1E-08	0.4	Ccnd1	4E-19	0.4
Emilin1	6E-17	0.4	Plod1	4E-09	0.4
Nucb2	5E-28	0.4	Pigf	2E-04	0.4
Lyrm4	3E-03	0.4	Zfp560	2E-12	0.4
Fam174a	9E-26	0.4	Tnrc6b	6E-31	0.4
Ctnnd1	2E-14	0.4	Rab11fip3	5E-04	0.4
4930517O19Rik	1E-02	0.4	Fbxl12	2E-04	0.4
Flrt1	2E-04	0.4	Fos	5E-18	0.4
1700084C06Rik	5E-05	0.4	Ndufaf5	6E-03	0.4
Rian	2E-17	0.4	Tmem176a	2E-24	0.4
Crebzf	1E-08	0.4	Pex3	6E-06	0.4

Fibroblast					
Control			NICD2		
Gene	p	FC	Gene	p	FC
Mylk	8E-01	0.3	Aqp5	6E-04	0.3
Hif1a	1E-132	0.3	Nsdhl	5E-11	0.3
Igsf8	1E-32	0.3	Snx10	5E-10	0.3
Manea	2E-17	0.3	Zfp809	3E-07	0.3
Elovl1	2E-32	0.3	Rgcc	2E-11	0.3
Tmem123	1E-15	0.3	Gnas	3E-39	0.3
H2-DMa	2E-12	0.3	Cyp51	5E-20	0.3
Elac1	5E-06	0.3	5730409E04Rik	2E-10	0.3
Cdc14a	5E-14	0.3	Nudt16	3E-05	0.3
Eif3e	9E-183	0.3	Ccdc6	1E-15	0.3
Comtd1	5E-24	0.3	Igsf8	3E-16	0.3
Socs2	1E-16	0.3	Pdzd4	1E-06	0.3
St3gal2	1E-37	0.3	Olfml2b	4E-13	0.3
Fdft1	7E-34	0.3	Armcx6	2E-06	0.3
Pcdhga5	4E-08	0.3	Grin2d	5E-03	0.3
Ass1	2E-20	0.3	Bmpr2	7E-29	0.3
Washc3	9E-58	0.3	Ifi35	2E-13	0.3
Ralb	3E-35	0.3	Csrp2	1E-19	0.3
Otulinl	2E-06	0.3	Pgap1	3E-03	0.3
Tns1	1E-33	0.3	Adamtsl5	1E-13	0.3
Atp13a3	7E-31	0.3	Maged1	9E-66	0.3
Zfp677	7E-06	0.3	Azin2	2E-12	0.3
Rab42	2E-03	0.3	Dnhd1	3E-03	0.3
Sfxn5	6E-12	0.3	Cdc42ep4	4E-03	0.3
Bace1	4E-27	0.3	Emilin1	2E-20	0.3
Gm14295	3E-08	0.3	Gsto1	2E-26	0.3
Nlrx1	8E-04	0.3	Mfsd3	3E-04	0.3
Vegfb	6E-60	0.3	Asah2	1E-06	0.3
Tet1	3E-09	0.3	Medag	3E-28	0.3
Smarcd2	1E-23	0.3	2410006H16Rik	3E-58	0.3
Maged1	2E-119	0.3	Runx2os1	4E-03	0.3
Rcn1	5E-117	0.3	Ezh1	3E-11	0.3
Dpy19l1	6E-17	0.3	C1ra	7E-33	0.3
Vangl2	1E-05	0.3	Acat2	3E-09	0.3
Idh1	3E-152	0.3	Pbx1	6E-47	0.3
Abhd11	2E-17	0.3	Palm	2E-08	0.3
Sema6a	2E-09	0.3	Adm	7E-34	0.3
Ppp3cb	4E-60	0.3	C1s1	2E-15	0.3
Snx9	7E-59	0.3	Golga7	2E-16	0.3
Sc5d	1E-26	0.3	Micall2	7E-04	0.3
Dtx3	1E-31	0.3	Zfp46	6E-06	0.3
Medag	1E-51	0.3	Sri	2E-56	0.3
Rnf122	2E-03	0.3	Tspan4	1E-40	0.3
2810032G03Rik	9E-09	0.3	Gm10069	1E-01	0.3
Cd59b	1E-10	0.3	Add1	1E-45	0.3
Pbx1	3E-94	0.3	Lekr1	1E-03	0.3
Spsb1	1E-09	0.3	Tom1l1	4E-02	0.3
Wwc2	2E-54	0.3	C1qtnf4	7E-02	0.3
Amacr	1E-09	0.3	Stat3	5E-41	0.3
Hip1	6E-22	0.3	Zhx1	1E-18	0.3
2610001J05Rik	6E-41	0.3	Wwc2	5E-29	0.3
Pcsk6	3E-18	0.3	Acap3	8E-06	0.3
Rgma	5E-16	0.3	Dtd1	4E-23	0.3
Ehd1	1E-35	0.3	Ikzf2	1E-05	0.3
Dnajb3	3E-07	0.3	Tgfbi	1E-12	0.3
Evi5	4E-41	0.3	Mttp	2E-02	0.3
Rsu1	4E-81	0.3	Eml4	5E-22	0.3
Trim12a	2E-14	0.3	Zfp677	4E-03	0.3
Nlrc5	4E-03	0.3	Cdk19	3E-08	0.3
Lgr4	8E-21	0.3	Zfp874a	1E-02	0.3
Pcdhga7	3E-11	0.3	2510009E07Rik	8E-11	0.3
Zfp46	3E-10	0.3	Rsu1	2E-42	0.3
Abcd2	2E-13	0.3	Cd59b	1E-06	0.3
Asxl3	4E-04	0.3	3300002I08Rik	9E-07	0.3
Setd4	2E-09	0.3	Cacna1b	9E-02	0.3
Synm	6E-14	0.3	Tceal3	1E-07	0.3
Zfp469	1E-08	0.3	Pold4	4E-35	0.3
Wrn	1E-18	0.3	Adgrl2	4E-29	0.3
Dcun1d3	2E-13	0.3	Dnajc25	2E-26	0.3
Zcchc24	5E-55	0.3	Bmpr1b	6E-02	0.3
Zhx1	4E-29	0.3	Src	6E-05	0.3
Gm6710	1E-07	0.3	Tmem26	3E-03	0.3
Gaa	1E-71	0.3	Zfp951	4E-03	0.3

Fibroblast?					
Control			NICD2		
Gene	p	FC	Gene	p	FC
Zfp946	7E-36	0.9	Ivns1abp	4E-69	0.9
Gm50340	8E-22	0.9	Bcl2l12	7E-59	0.9
Dpy30	6E-86	0.9	Thsd4	4E-24	0.9
Klf10	1E-24	0.9	Timm8a1	3E-59	0.9
1700007K13Rik	4E-32	0.9	Rxfp1	2E-20	0.9
Hnrnpd	5E-60	0.9	Schip1	1E-63	0.9
AW554918	4E-60	0.9	Ehd4	2E-69	0.9
Ubald2	1E-41	0.9	Nin	2E-56	0.9
Gnb1l	5E-34	0.9	Smc3	4E-150	0.9
Prx	5E-19	0.9	Rhno1	1E-66	0.9
Supt16	8E-100	0.9	Ormdl1	6E-49	0.9
Rnf141	3E-22	0.9	Parp2	7E-91	0.9
Mdm2	8E-22	0.9	Crabp1	1E-20	0.9
Rb1	1E-54	0.9	E230016K23Rik	1E-13	0.9
Slc25a13	7E-47	0.9	Gkap1	4E-87	0.9
Stx11	1E-24	0.9	Adcy7	2E-22	0.9
E2f6	8E-48	0.9	Mmgt1	3E-68	0.9
Nsmce1	8E-75	0.9	Chst7	4E-33	0.9
Timm8a1	5E-43	0.9	Ctdspl2	3E-94	0.9
Tdp2	3E-52	0.9	Tubgcp6	1E-38	0.9
Kras	2E-61	0.9	Cpsf4	1E-47	0.9
Plekhg3	1E-45	0.9	Myom1	4E-18	0.9
Disc1	2E-13	0.9	Zfp808	4E-56	0.9
Ptk2b	3E-31	0.9	Ntn4	5E-43	0.9
Bcam	3E-15	0.9	B130055M24Rik	3E-56	0.9
Mcub	3E-46	0.9	Rmi1	2E-47	0.9
Casp3	2E-60	0.9	Pa2g4	2E-112	0.9
Emp1	8E-81	0.9	Rb1	2E-60	0.9
Ccl9	2E-26	0.9	Nup153	2E-73	0.9
Tarsl2	8E-37	0.9	Pla2g4a	3E-63	0.9
Fastkd3	1E-27	0.9	Sipa1	1E-49	0.9
Cby1	1E-48	0.9	Arhgef26	3E-51	0.9
Znhit3	8E-49	0.9	Mrpl49	1E-83	0.9
Snrnp25	5E-57	0.9	Npat	1E-67	0.9
Mapk12	3E-18	0.9	Ccdc141	6E-51	0.9
Ppp2r5d	1E-50	0.9	Mthfd1	2E-59	0.9
Pms1	8E-33	0.9	Zfp599	3E-24	0.9
BC052040	7E-46	0.9	Tamm41	9E-55	0.9
Naa50	5E-94	0.9	Ccdc57	1E-50	0.9
Ctdspl2	4E-69	0.9	Tm4sf1	2E-72	0.9
Gcfc2	2E-36	0.9	Pcbp3	1E-68	0.9
Txnrd1	5E-76	0.9	Wrap53	4E-45	0.9
Adcy7	1E-20	0.9	Nfyb	8E-68	0.9
Alcam	2E-41	0.9	Lmo1	6E-51	0.9
Smc6	6E-125	0.9	Tarsl2	7E-40	0.9
Pdik1l	3E-49	0.9	Nudt5	3E-90	0.9
Fbxl8	8E-35	0.9	Srpk1	3E-99	0.9
Trim7	3E-22	0.9	Wdr35	2E-69	0.9
Cacnb3	5E-20	0.9	Erlin1	5E-60	0.9
Wrap53	2E-40	0.9	Nectin1	1E-42	0.9
Gm31378	6E-13	0.9	Synpo2	8E-24	0.9
Elmo2	1E-43	0.9	Mnd1	3E-38	0.9
Gxylt1	2E-48	0.9	Pom121	2E-60	0.9
Cryzl2	5E-01	0.9	Zbed3	8E-46	0.9
Sh3bgrl2	4E-20	0.9	Cep44	2E-51	0.9
Tmem136	4E-07	0.9	Rgs4	1E-11	0.9
Nup153	3E-51	0.9	Dnase1l1	8E-55	0.9
Tpm3-rs7	8E-16	0.9	Mapk12	5E-18	0.9
Gm48228	1E-19	0.9	Meaf6	1E-72	0.9
Map3k5	4E-44	0.9	Usp31	1E-42	0.9
Rbpms2	2E-16	0.9	Calm2	4E-230	0.9
Pde3b	2E-53	0.9	Irx1	2E-17	0.9
Rbm15	3E-50	0.9	Stx11	1E-26	0.9
4930478M13Rik	5E-18	0.9	Naa50	2E-125	0.9
Hes1	5E-37	0.9	Shroom2	2E-23	0.9
Kirrel3	7E-25	0.9	Gpr19	7E-36	0.9
Gpn3	1E-45	0.9	Pxmp2	3E-40	0.9
Erlin1	2E-48	0.9	Tcof1	2E-88	0.9
Tyro3	1E-20	0.9	Rab27b	2E-32	0.9
Armh1	4E-18	0.9	Galnt18	3E-96	0.9
Amd1	4E-46	0.9	Igf2bp1	3E-44	0.8
Meaf6	1E-50	0.9	Fam177a	3E-33	0.8
Ildr2	7E-39	0.9	Ado	4E-58	0.8

Undefined					
Control			NICD2		
Gene	p	FC	Gene	p	FC
Zfp955a	5E-01	0.2	Ppie	3E-01	0.2
Rp2	5E-01	0.2	Slc41a2	8E-01	0.2
Fam129a	5E-04	0.2	Lrrc51	4E-01	0.2
Vps72	3E-01	0.2	Sfi1	5E-01	0.2
Ccdc28a	6E-01	0.2	Aldh2	2E-14	0.2
Prtg	5E-01	0.2	Angptl6	3E-01	0.2
Dnaja4	6E-02	0.2	Dtwd2	1E+00	0.2
Gm47710	4E-01	0.2	1190007I07Rik	9E-01	0.2
2410131K14Rik	5E-02	0.2	Luc7l	5E-01	0.2
Nubp2	9E-01	0.2	Gm15411	4E-01	0.2
Tmem222	2E-01	0.2	Ebna1bp2	7E-03	0.2
H13	1E-07	0.2	Cep126	8E-01	0.2
Tmem132a	3E-03	0.2	Pak3	6E-03	0.2
Magi2	5E-12	0.2	Gm15417	6E-03	0.2
Glmp	8E-09	0.2	Ift57	3E-02	0.2
1110008P14Rik	3E-17	0.2	Mtor	6E-01	0.2
Capg	3E-16	0.2	Cd320	1E-02	0.2
Vps26a	5E-01	0.2	Rnf41	7E-01	0.2
Jdp2	6E-04	0.2	Hars2	4E-01	0.2
Usp36	4E-01	0.2	Got1	7E-01	0.2
Ttc23	1E-03	0.2	Cebpzos	6E-04	0.2
Tusc2	2E-01	0.2	Itga2b	5E-01	0.2
Cnpy2	9E-30	0.2	Fap	2E-01	0.2
Ktn1	2E-08	0.2	Zfp248	4E-02	0.2
Prnp	4E-16	0.2	Esrra	4E-01	0.2
0610009E02Rik	6E-01	0.2	Abt1	1E-01	0.2
Cul1	2E-03	0.2	Gm9856	7E-01	0.2
Slc7a6os	3E-01	0.2	Pick1	9E-01	0.2
Sdr39u1	1E+00	0.2	Kif21a	2E-02	0.2
Abcc1	7E-02	0.2	Dync1i1	7E-01	0.2
Zfp703	2E-01	0.2	Igdcc4	5E-02	0.2
a	5E-01	0.2	Fam13c	2E-02	0.2
Slc9a8	2E-01	0.2	Tmc6	1E+00	0.2
Ndrg1	5E-02	0.2	Lrig1	5E-01	0.2
Stx6	1E-01	0.2	Tspan3	9E-18	0.2
Svep1	3E-06	0.2	Neurl2	8E-01	0.2
Fam89b	1E-01	0.2	Nfat5	3E-08	0.2
Mtg1	6E-01	0.2	Fbxo28	2E-01	0.2
Hexb	4E-03	0.2	Enpp5	7E-01	0.2
1810021B22Rik	7E-01	0.2	Pvr	6E-01	0.2
Zbtb17	6E-01	0.2	Rsl24d1	2E-01	0.2
Ccnq	2E-01	0.2	Ikzf4	8E-01	0.2
Gm34455	5E-02	0.2	Prmt3	6E-01	0.2
Zfp523	1E+00	0.2	Snrpa	2E-02	0.2
Spire1	4E-01	0.2	Scarb1	9E-01	0.2
Tor3a	1E-01	0.2	Stard10	8E-01	0.2
Irak4	6E-01	0.2	Pomgnt2	7E-01	0.2
Orai3	9E-01	0.2	Dexi	9E-02	0.2
Nudt16l1	8E-01	0.2	Jmjd8	7E-01	0.2
Kctd6	1E-01	0.2	Sobp	4E-03	0.2
Nfat5	2E-06	0.2	Nars2	9E-01	0.2
Bin3	9E-01	0.2	Mrpl16	9E-01	0.2
Acot8	4E-01	0.2	Enox2	1E-01	0.2
Chchd4	5E-01	0.2	Zfp263	4E-01	0.2
Zfp787	6E-01	0.2	Fam171a2	9E-01	0.2
Ethe1	1E-01	0.2	6330418K02Rik	1E-01	0.2
Pcolce	8E-23	0.2	Wdr4	3E-01	0.2
Vps13c	7E-06	0.2	Dixdc1	3E-01	0.2
Kat8	3E-01	0.2	2610306M01Rik	6E-01	0.2
Zfp2	9E-01	0.2	Adprm	8E-01	0.2
Dync2h1	1E-02	0.2	Sat1	7E-08	0.2
Rhot2	6E-01	0.2	Prelid2	9E-01	0.2
Ppt2	2E-01	0.2	Dnm1	1E+00	0.2
Mrps18a	1E-03	0.2	Dync2h1	9E-02	0.2
Pard6a	4E-01	0.2	Entr1	4E-01	0.2
Ppp1r35	2E-01	0.2	S100b	8E-01	0.2
Fam110b	8E-03	0.2	Gng11	2E-02	0.2
Gm16124	9E-01	0.2	Slc10a3	7E-01	0.2
Slc24a3	7E-06	0.2	Pfdn4	9E-01	0.2
Arhgef5	4E-02	0.2	Plpbp	8E-01	0.2
Kcnd2	1E+00	0.2	Tlr2	6E-01	0.2
Mccc1	5E-01	0.2	Yipf2	5E-01	0.2
Tns2	3E-01	0.2	Gm15564	5E-01	0.2

Articular/Synovial Fibroblast					
Control			NICD2		
Gene	p	FC	Gene	p	FC
Spg21	3E-12	0.4	Lpin1	1E-01	0.4
Dync2h1	2E-06	0.4	Plxnd1	8E-01	0.4
Igf2bp2	8E-06	0.4	Plekhm2	2E-03	0.4
Abca1	8E-04	0.4	Zdhhc14	8E-02	0.4
Fbxo25	1E-03	0.4	Sipa1	3E-01	0.4
Pla2g15	1E-01	0.4	Amer1	4E-02	0.4
3110009E18Rik	7E-02	0.4	Hmga1	2E-01	0.4
Cavin1	4E-20	0.4	Ppp2r3d	3E-01	0.4
Tyk2	3E-01	0.4	Cotl1	1E-01	0.4
Clstn1	6E-07	0.4	Cpped1	2E-02	0.4
Zbtb21	2E-01	0.4	Spata6	2E-05	0.4
Ang	9E-02	0.4	Mocs1	2E-01	0.4
Ehd2	4E-14	0.4	Slc2a8	9E-01	0.4
Ccdc114	9E-01	0.4	Rab11fip5	7E-02	0.4
Hscb	9E-03	0.4	Rora	1E-20	0.4
AU041133	4E-01	0.4	Nrp1	2E-12	0.4
Txndc16	4E-02	0.4	Gorasp1	7E-01	0.4
Plekha8	4E-01	0.4	Ubxn11	5E-01	0.4
Paox	4E-01	0.4	Srfbp1	1E-03	0.4
Zfp808	3E-01	0.4	Trim12c	1E-02	0.4
Asl	2E-01	0.4	Nudt13	6E-01	0.4
Tnfsf12	2E-06	0.4	Fahd1	1E-01	0.4
Dnajc13	4E-07	0.4	Has2os	3E-01	0.4
Zyx	1E-03	0.4	Gsdmd	2E-01	0.4
Cryl1	8E-02	0.4	Saal1	5E-01	0.4
Por	3E-05	0.4	Zfp687	8E-01	0.4
Slc7a6	1E-01	0.4	Barx1	1E-10	0.4
Icam1	1E-03	0.4	Hexb	2E-07	0.4
Marveld1	2E-03	0.4	Ctps2	1E-03	0.4
Gm28875	4E-01	0.4	Cbr3	4E-06	0.4
Trp53inp1	5E-04	0.4	Gga2	4E-05	0.4
Lrrc49	1E-01	0.4	Ftl1-ps1	7E-03	0.4
Gca	3E-01	0.4	Zfp874a	1E+00	0.4
Decr2	8E-02	0.4	Tbc1d9	1E-01	0.4
4930599N23Rik	1E-01	0.4	Fam234a	7E-03	0.4
Ulk1	4E-02	0.4	Sdc1	1E-02	0.4
Nectin1	3E-01	0.4	Commd4	2E-05	0.4
Col4a5	2E-07	0.4	Lcn2	7E-19	0.4
Cda	3E-01	0.4	Tnfrsf1a	3E-09	0.4
Akr1b10	3E-01	0.4	Mul1	3E-01	0.4
Lacc1	4E-01	0.4	Kcnrg	5E-01	0.4
Zbtb40	9E-01	0.4	Jun	2E-14	0.4
Sh3pxd2a	6E-10	0.4	Tmem9	6E-03	0.4
Pcsk6	2E-01	0.4	Sh3bp1	6E-01	0.4
Myo9a	2E-08	0.4	Flt3l	4E-01	0.4
Cc2d2a	5E-03	0.4	Fhit	4E-04	0.4
Pitpnm1	5E-01	0.4	Gm14305	7E-01	0.4
L3mbtl3	2E-02	0.4	Ddr2	3E-12	0.4
Gga3	7E-01	0.4	Ccdc167	2E-02	0.4
Srgap3	1E-12	0.4	Trim7	4E-01	0.4
Metrnl	2E-03	0.4	Serpine2	2E-17	0.4
Lmbr1	3E-03	0.4	Gamt	3E-03	0.4
Itpr2	5E-04	0.4	Pqlc2	3E-01	0.4
Rgs3	8E-05	0.4	Zeb2os	8E-01	0.4
Sik2	6E-07	0.4	Phc1	8E-01	0.4
Polrmt	9E-01	0.4	Glis3	1E-11	0.4
Ei24	4E-09	0.4	Cpt1a	8E-03	0.4
Akr1e1	1E-03	0.4	Gm6712	9E-01	0.4
Arsa	5E-03	0.4	1110012L19Rik	5E-03	0.4
Pfkfb2	5E-01	0.4	2610507I01Rik	3E-01	0.4
Selenon	1E-04	0.4	Tmem242	1E-03	0.4
Rpap1	3E-01	0.4	Plcd3	6E-01	0.4
Cnp	2E-02	0.4	Sumf1	2E-02	0.4
Itpk1	9E-01	0.4	Glb1l	6E-01	0.4
Slfn9	1E-13	0.4	Bmper	5E-01	0.4
Tle1	2E-03	0.4	Rnase4	2E-06	0.4
Lpcat2	9E-01	0.4	Nsg1	9E-08	0.4
Inf2	8E-03	0.4	Nek8	8E-01	0.4
Oplah	3E-01	0.4	Lamb2	1E-03	0.4
Sgpl1	1E-03	0.4	Gm12905	3E-01	0.4
Zfp12	7E-01	0.4	Fam229b	6E-02	0.4
Crem	1E-02	0.4	Hist3h2a	3E-01	0.4
Bbc3	6E-04	0.4	Adam19	2E-01	0.4

Limb Mesenchyme					
Control			NICD2		
Gene	*p*	FC	Gene	*p*	FC
Usp16	1E-19	0.3	Ets2	1E-05	0.3
Cry1	8E-08	0.3	Jmy	3E-09	0.3
Akap17b	3E-09	0.3	Fam234b	4E-03	0.3
Arel1	5E-09	0.3	Sec22c	8E-04	0.3
Spryd3	6E-10	0.3	Ston1	1E-10	0.3
Cd109	2E-20	0.3	Pgm3	3E-11	0.3
Rwdd1	2E-37	0.3	Galc	6E-05	0.3
Lipo3	5E-07	0.3	Pcx	3E-13	0.3
Gstm7	1E-03	0.3	Orai2	8E-05	0.3
Eif2s2	1E-46	0.3	Top2b	3E-25	0.3
Tbrg4	5E-06	0.3	Glce	2E-08	0.3
Mtss2	2E-17	0.3	Fam131a	6E-03	0.3
Efnb2	1E-11	0.3	Slc23a2	1E-05	0.3
Cox18	1E-10	0.3	E130308A19Rik	2E-05	0.3
Ccdc126	2E-09	0.3	Vgll3	2E-16	0.3
Plekha7	1E-05	0.3	Sntb2	1E-18	0.3
Aagab	2E-10	0.3	Rassf2	8E-10	0.3
Spty2d1	7E-14	0.3	Foxp4	2E-07	0.3
Ppip5k2	2E-11	0.3	Eif4ebp1	8E-24	0.3
Nfe2l1	6E-34	0.3	Clcn3	9E-08	0.3
2700069I18Rik	3E-06	0.3	Slco3a1	5E-09	0.3
Gm43713	1E-06	0.3	Fn1	2E-34	0.3
2500004C02Rik	6E-03	0.3	Pdcl	8E-10	0.3
Spint2	7E-05	0.3	Abcb8	3E-02	0.3
Cnot7	1E-11	0.3	Pex26	2E-03	0.3
Ppp2r5a	1E-17	0.3	Lcorl	1E-14	0.3
Heatr3	7E-13	0.3	Cop1	2E-19	0.3
Abcb9	9E-05	0.3	Mbnl2	6E-36	0.3
Pdp1	2E-14	0.3	Garem1	5E-08	0.3
Plekhh3	4E-08	0.3	Rmnd5a	7E-10	0.3
Cdipt	5E-13	0.3	Slc25a38	1E-07	0.3
Ttc39b	5E-12	0.3	Spin1	8E-13	0.3
Mia2	2E-22	0.3	Zfp942	1E-03	0.3
Pabpc4	1E-18	0.3	Pcdhgb6	3E-03	0.3
Bmpr1a	7E-27	0.3	Phldb2	8E-22	0.3
Serp2	5E-09	0.3	Akap9	4E-18	0.3
Zfp672	6E-10	0.3	Slc3a2	2E-20	0.3
Larp1	2E-17	0.3	Ninj1	4E-17	0.3
Parp8	6E-14	0.3	Senp5	1E-12	0.3
Sec16a	2E-10	0.3	Ikzf4	2E-05	0.3
Rnf139	2E-10	0.3	Cox4i2	8E-11	0.3
Csrnp2	7E-04	0.3	Elp3	1E-08	0.3
Hdlbp	2E-59	0.3	Bcar3	3E-12	0.3
Atxn2l	2E-15	0.3	Stk26	3E-17	0.3
Ccn4	1E-17	0.3	Calu	3E-35	0.3
Tent4b	2E-10	0.3	Ppm1k	1E-03	0.3
Dlg1	5E-21	0.3	Lrrc8b	6E-04	0.3
B630019K06Rik	2E-04	0.3	Sdc4	2E-26	0.3
Alg3	3E-07	0.3	Kdm6a	1E-10	0.3
Nphs1	4E-07	0.3	Ficd	2E-04	0.3
Mark3	7E-18	0.3	Ss18l2	4E-14	0.3
Rad23b	3E-22	0.3	Elp1	2E-10	0.3
Mmgt1	3E-09	0.3	Jrkl	6E-09	0.3
C1rl	2E-10	0.3	Pex5	9E-05	0.3
Rsf1os1	2E-05	0.3	Hnrnpab	4E-31	0.3
Rnf138	1E-12	0.3	Rbmx	5E-09	0.3
Ccn1	2E-22	0.3	Kansl2	2E-13	0.3
Ttc13	1E-10	0.3	Tspan3	3E-32	0.3
Azin1	4E-13	0.3	Prrc1	2E-18	0.3
Ttc3	9E-38	0.3	Tle3	2E-06	0.3
Rft1	5E-09	0.3	Sfxn3	5E-08	0.3
Elp1	1E-10	0.3	Irf2bp1	8E-12	0.3
Exog	1E-06	0.3	Ikzf2	6E-07	0.3
Hnrnph3	6E-20	0.3	Spindoc	7E-10	0.3
Gm15581	3E-03	0.3	Akt3	1E-13	0.3
Lpar6	1E-07	0.3	Cox10	4E-08	0.3
Pfkfb2	3E-09	0.3	Pecr	2E-05	0.3
Usb1	2E-04	0.3	Sesn3	7E-16	0.3
Tbc1d8b	1E-12	0.3	Pgrmc2	7E-22	0.3
Arv1	9E-06	0.3	Gar1	2E-13	0.3
Pcgf3	7E-11	0.3	Egfr	3E-20	0.3
Gcn1	6E-10	0.3	Prtg	2E-05	0.3
Cep83	5E-08	0.3	Spats2l	2E-08	0.3

Chondrogenic					
Control			NICD2		
Gene	*p*	FC	Gene	*p*	FC
Uxs1	2E-12	0.4	Fbxl2	2E-03	0.4
Ids	3E-07	0.4	Rsf1os1	7E-02	0.4
Ap4s1	7E-15	0.4	Parp8	7E-10	0.4
Snx18	1E-11	0.4	Me1	2E-20	0.4
Elmo1	3E-09	0.4	Slc18b1	6E-04	0.4
Rnf167	2E-09	0.4	Gabarapl1	2E-16	0.4
Gm15222	2E-02	0.4	Naaladl2	3E-24	0.4
Pon2	4E-14	0.4	Ttc26	3E-03	0.4
Fn1	1E-44	0.4	Ccdc134	9E-01	0.4
Cys1	5E-05	0.4	Taf1a	1E-02	0.4
Robo2	1E-03	0.4	Casc4	2E-25	0.4
Il17d	2E-02	0.4	Ptprg	5E-15	0.4
Ltbp3	1E-29	0.4	Usp50	7E-04	0.4
Ppp1r13b	1E-04	0.4	Ccnd2	3E-36	0.4
Phyhd1	3E-15	0.4	Ssr3	1E-46	0.4
Card19	1E-26	0.4	Antxr1	9E-13	0.4
Taf1a	1E-02	0.4	Rasl11a	3E-01	0.4
Mapre3	1E-10	0.4	BC002059	2E-02	0.4
Tns2	2E-08	0.4	Cox20	7E-24	0.4
Chrd	5E-03	0.4	Foxo1	3E-06	0.4
Ino80dos	4E-04	0.4	Stk39	6E-07	0.4
Fam71e1	1E-03	0.4	Abcb8	7E-01	0.4
Lysmd1	1E-03	0.4	Tm4sf1	5E-14	0.4
Cth	6E-04	0.4	Pdgfa	2E-10	0.4
Cep41	2E-05	0.4	Pygo1	7E-07	0.4
Gm48678	2E-04	0.4	Rsph3b	1E-01	0.4
1810024B03Rik	2E-02	0.4	Arpc5l	9E-20	0.4
Itga8	7E-03	0.4	Zfp287	2E-01	0.4
Mtmr10	6E-03	0.4	Smim3	3E-04	0.4
Tmem106c	5E-07	0.4	Mmachc	3E-01	0.4
5530601H04Rik	1E-04	0.4	Tbc1d10a	2E-02	0.4
Creb3l2	8E-21	0.4	Coq10a	6E-03	0.4
Snrpn	3E-02	0.4	Lrrc8d	1E-02	0.4
Oma1	5E-04	0.4	Gsdme	3E-03	0.4
Dand5	4E-04	0.4	BC005537	3E-11	0.4
Enpp1	3E-09	0.4	Crebzf	6E-06	0.4
Ube4bos1	3E-02	0.4	Gabarapl2	3E-37	0.4
Epm2a	9E-02	0.4	Lig4	3E-01	0.4
Ranbp17	3E-04	0.4	Sft2d3	2E-02	0.4
Clic4	3E-33	0.4	Pkd1	5E-07	0.4
Ahcyl2	2E-08	0.4	Ctnna1	1E-24	0.4
Gnl3l	3E-12	0.4	Rpusd4	3E-02	0.4
Gm15787	2E-01	0.4	Smpd1	2E-09	0.4
Ikbip	1E-40	0.4	Unc5c	1E-10	0.4
Fbxl12os	7E-02	0.4	Macrod1	4E-14	0.4
Mob2	2E-07	0.4	Ccdc126	4E-02	0.4
Casc4	2E-35	0.4	Zfp2	1E-01	0.4
Higd1a	6E-57	0.4	Ahcyl2	7E-07	0.4
Ube2cbp	4E-04	0.4	Gbe1	3E-04	0.4
Chic1	3E-06	0.4	Plxna2	7E-02	0.4
Tgfb3	5E-09	0.4	Hagh	4E-08	0.4
Kat6b	9E-21	0.4	Stac	3E-03	0.4
Napb	1E-03	0.4	Rnf215	1E-03	0.4
Sept1	4E-05	0.4	Pyroxd1	5E-03	0.4
Arhgap32	1E-05	0.4	Lrp8	2E-01	0.4
N4bp2l1	5E-02	0.4	Armcx5	6E-04	0.4
Zfp788	6E-04	0.4	Sox4	2E-19	0.4
Srebf1	5E-08	0.4	Cwc22	1E-07	0.4
Ugp2	5E-30	0.4	Higd1a	6E-39	0.4
Rhbdd1	4E-06	0.4	Zgpat	2E-05	0.4
1810021B22Rik	2E-02	0.4	Dzip3	1E-09	0.4
1810026B05Rik	5E-14	0.4	Kdm6a	5E-08	0.4
Wdr6	8E-10	0.4	A430033K04Rik	2E-02	0.4
Tcp11l2	5E-07	0.4	Sgms2	2E-04	0.4
Smtn	3E-13	0.4	Myo10	5E-16	0.4
Gde1	7E-18	0.4	2610507I01Rik	2E-02	0.4
Slc25a27	1E-06	0.4	D11Wsu47e	2E-01	0.4
Asxl1	6E-14	0.4	Atg13	2E-03	0.4
BC065397	9E-02	0.4	Snhg14	1E-01	0.4
Steap1	1E-01	0.4	Guk1	6E-16	0.4
Ccdc189	8E-03	0.4	Hyal2	1E-04	0.4
Smpdl3b	4E-03	0.4	Cyb5r2	1E-01	0.4
9430091E24Rik	3E-01	0.4	Zfp975	8E-03	0.4

Fibroblast					
Control			NICD2		
Gene	*p*	FC	Gene	*p*	FC
Nfib	3E-87	0.3	Acly	1E-20	0.3
Per3	4E-30	0.3	Mdfic	1E-29	0.3
Tsc22d3	1E-12	0.3	Calml4	7E-05	0.3
Spink10	2E-03	0.3	Trim12a	7E-08	0.3
Insyn1	3E-09	0.3	Rnf145	2E-07	0.3
Col5a3	6E-59	0.3	Lynx1	6E-08	0.3
4732440D04Rik	5E-09	0.3	Tstd3	4E-08	0.3
Gpr153	3E-06	0.3	Fbh1	5E-08	0.3
Slc36a1	5E-07	0.3	Tmem159	3E-14	0.3
Ccdc171	3E-11	0.3	Cxcl1	3E-08	0.3
Jade3	4E-04	0.3	Il17ra	2E-06	0.3
Maml3	5E-13	0.3	Man2b1	2E-15	0.3
Stat3	5E-67	0.3	H2-T23	8E-12	0.3
Lifr	3E-32	0.3	Fam117a	3E-05	0.3
Npepps	3E-63	0.3	Phf11d	3E-08	0.3
Ptprm	1E-52	0.3	Cfb	2E-07	0.3
Dip2a	1E-11	0.3	Mxra7	6E-41	0.3
Armcx6	2E-10	0.3	Mvp	9E-13	0.3
Osmr	1E-41	0.3	Numbl	2E-13	0.3
Mia3	4E-69	0.3	Agl	3E-12	0.3
Cfb	5E-09	0.3	Rps27	8E-233	0.3
Tango2	5E-17	0.3	Mia3	2E-39	0.3
Lrsam1	5E-11	0.3	Snx30	9E-07	0.3
Alg11	3E-11	0.3	Nlrx1	8E-02	0.3
Bmpr2	7E-45	0.3	Trpc1	2E-03	0.3
Galm	8E-08	0.3	Rnf19b	5E-03	0.3
Hpcal1	2E-12	0.3	Uba7	3E-03	0.3
Il1rl1	1E-15	0.3	Nkd1	6E-10	0.3
Gm16364.1	2E-10	0.3	Slc30a7	2E-09	0.3
Pgpep1	6E-18	0.3	Stam2	3E-16	0.3
Iqsec2	1E-14	0.3	Cpne2	1E-16	0.3
Ptgfrn	4E-11	0.3	Pdgfra	2E-32	0.3
Gcc1	3E-05	0.3	Rps6kb1	9E-20	0.3
Plekho2	2E-16	0.3	Jade3	2E-02	0.3
Arap2	5E-05	0.3	Daam1	2E-08	0.3
Frk	6E-14	0.3	Ifit1	4E-09	0.3
Il17ra	9E-08	0.3	Cyp4v3	1E-05	0.3
Nipal2	4E-05	0.3	Prkar1b	2E-05	0.3
Dhcr7	2E-05	0.3	Unc5b	6E-02	0.3
Slc4a2	4E-22	0.3	Paox	3E-03	0.3
Khk	5E-09	0.3	Elovl1	2E-17	0.3
Pde3a	8E-29	0.3	Irf2bpl	1E-11	0.3
Glipr2	2E-31	0.3	Tnfsf12	6E-18	0.3
Trim12c	1E-18	0.3	Rras	4E-34	0.3
Acap3	4E-08	0.3	Hipk3	7E-18	0.3
Tcirg1	3E-08	0.3	Orc4	9E-17	0.3
Pold4	6E-55	0.3	Frg2f1	1E-04	0.3
Nudt16	2E-07	0.3	Gm46430	3E-03	0.3
Cyb561a3	1E-04	0.3	Hip1	2E-11	0.3
Acvr1	3E-31	0.3	C1qtnf6	8E-10	0.3
Sri	8E-93	0.3	Adcy6	6E-08	0.3
Adm	2E-68	0.3	Ppp3cb	8E-30	0.3
Spats2l	3E-32	0.3	Vangl2	4E-03	0.3
Dapk1	1E-40	0.3	Svep1	5E-59	0.3
Ifi35	1E-20	0.3	Ifngr1	8E-17	0.3
Mcfd2	4E-23	0.3	Ahnak	2E-73	0.3
Tubb2a	1E-60	0.3	Shprh	2E-12	0.3
Adcy6	7E-12	0.3	Lpp	6E-50	0.3
Lhpp	2E-04	0.3	Htra3	7E-14	0.3
Acap2	8E-47	0.3	Slc41a3	1E-03	0.3
Tpcn2	2E-06	0.3	Prdx4	1E-35	0.3
Gm43672	1E-06	0.3	Loxl1	7E-39	0.3
Bcor	2E-08	0.3	Lrsam1	1E-05	0.3
Kcne4	8E-07	0.3	2610001J05Rik	4E-22	0.3
Orc4	2E-30	0.3	0610009L18Rik	5E-05	0.3
Med13l	4E-43	0.3	Trib1	3E-04	0.3
Cpeb1	5E-21	0.3	Srgap2	7E-22	0.3
Rps27	0E+00	0.3	Lgr4	2E-12	0.3
Col1a1	7E-151	0.3	Ralb	1E-17	0.3
Nfkbia	5E-27	0.3	Nln	3E-12	0.3
Mtm1	1E-07	0.3	Hddc3	8E-04	0.3
Elovl6	2E-23	0.3	Khk	9E-06	0.3
Gnpda2	4E-31	0.3	Mtm1	9E-06	0.3

Fibroblast?					
Control			NICD2		
Gene	*p*	FC	Gene	*p*	FC
Trp53i11	2E-12	0.9	C77080	5E-19	0.8
Ero1lb	7E-32	0.9	Pole4	1E-106	0.8
Hmga2	1E-32	0.9	Aimp2	7E-42	0.8
Pa2g4	1E-77	0.9	Frmd4a	8E-67	0.8
Prkcg	3E-16	0.9	Foxd1	1E-22	0.8
Cycs	2E-87	0.9	2610307P16Rik	4E-63	0.8
Tcof1	5E-58	0.9	Oser1	8E-54	0.8
A630089N07Rik	6E-47	0.9	Zbtb8a	3E-48	0.8
Inip	2E-38	0.9	1700048O20Rik	4E-23	0.8
Haus7	2E-48	0.9	Gpn3	3E-59	0.8
Smc3	2E-90	0.9	Srsf3	1E-160	0.8
Dzip1l	6E-42	0.9	Ints7	8E-65	0.8
Myef2	2E-57	0.9	Cstf2	2E-50	0.8
Dpy19l1	1E-46	0.9	Cdan1	3E-33	0.8
Ints7	2E-43	0.9	Ubald2	1E-45	0.8
Npat	1E-53	0.9	Vsig10	1E-27	0.8
Serpina3g	3E-13	0.9	Med30	2E-68	0.8
Sclt1	1E-46	0.9	Gm48727	7E-10	0.8
Txlnb	2E-07	0.9	Thop1	6E-43	0.8
Sdc1	2E-56	0.9	Cep83	2E-59	0.8
Mcmbp	9E-47	0.9	Znhit3	1E-67	0.8
Sephs1	6E-42	0.9	Slc9a3r1	1E-58	0.8
Tcirg1	1E-27	0.8	Tyro3	2E-30	0.8
Glrb	3E-23	0.8	Noc4l	1E-34	0.8
Gclm	3E-66	0.8	Smc5	2E-78	0.8
Tssc4	6E-55	0.8	Ak2	4E-93	0.8
Crybg1	6E-17	0.8	Faap24	2E-31	0.8
Rnf26	3E-44	0.8	Mbnl3	5E-49	0.8
Pak4	4E-30	0.8	Ckap4	5E-220	0.8
Nup188	3E-43	0.8	Nelfcd	3E-48	0.8
Ap5s1	1E-14	0.8	Nup50	7E-67	0.8
H2-Q6	3E-16	0.8	Zfp934	3E-62	0.8
Dennd2c	1E-29	0.8	Ankrd29	1E-49	0.8
Slc19a2	3E-25	0.8	Myo19	5E-42	0.8
Raph1	7E-49	0.8	Rbbp7	1E-142	0.8
Rras2	8E-24	0.8	Crybg3	2E-66	0.8
Anxa3	3E-48	0.8	Elmo2	3E-50	0.8
Ptprn	5E-15	0.8	Smc6	5E-164	0.8
Mrpl49	1E-50	0.8	Ccdc162	5E-20	0.8
Zmynd19	4E-32	0.8	Tpm2	8E-98	0.8
Baz1a	1E-43	0.8	U2af1	4E-145	0.8
Scx	2E-27	0.8	Nol4l	1E-18	0.8
Zdhhc16	1E-37	0.8	Pdik1l	4E-47	0.8
Mettl24	3E-19	0.8	2410018L13Rik	1E-40	0.8
Zmat1	2E-33	0.8	AW554918	2E-62	0.8
Vrk2	3E-44	0.8	Map3k5	2E-43	0.8
Pwwp3a	3E-47	0.8	4930414N06Rik	2E-17	0.8
Arhgap18	2E-47	0.8	Lzts3	2E-20	0.8
Rab23	3E-41	0.8	Oard1	2E-61	0.8
Rab7b	8E-26	0.8	Agfg2	4E-63	0.8
Eva1c	1E-18	0.8	Psmb9	3E-29	0.8
Tmem100	1E-26	0.8	Golm1	4E-60	0.8
Ipo5	7E-55	0.8	2810006K23Rik	4E-66	0.8
Cox6b2	4E-22	0.8	Chn1	5E-24	0.8
Sac3d1	2E-46	0.8	Fam118a	4E-25	0.8
Clic1	5E-151	0.8	Med4	9E-55	0.8
Mars2	8E-21	0.8	Nexn	1E-31	0.8
G3bp1	9E-90	0.8	Cep162	5E-59	0.8
Mcm9	2E-32	0.8	Uhrf2	2E-76	0.8
Ccdc57	7E-28	0.8	Them6	1E-16	0.8
Zfp54	6E-23	0.8	Cdkn1a	3E-71	0.8
Txn1	5E-176	0.8	Nup188	8E-59	0.8
Rgs12	1E-35	0.8	Thy1	2E-28	0.8
Med4	2E-47	0.8	Blvra	2E-55	0.8
Ptgr1	5E-40	0.8	Thyn1	6E-60	0.8
Ivns1abp	1E-42	0.8	Cables2	3E-31	0.8
Odf2l	2E-35	0.8	Slx4	4E-37	0.8
Apcdd1	9E-23	0.8	Gm15489	1E-17	0.8
2900026A02Rik	3E-29	0.8	Lrwd1	1E-40	0.8
Stn1	8E-45	0.8	Myg1	6E-54	0.8
Mmgt1	4E-42	0.8	Odf2l	8E-56	0.8
Fam177a	2E-34	0.8	Rpgrip1	1E-45	0.8
Mad2l1bp	6E-35	0.8	Rassf1	3E-55	0.8

Undefined					
Control			NICD2		
Gene	*p*	FC	Gene	*p*	FC
Gabbr1	9E-02	0.2	Scrn2	8E-01	0.2
Hps4	9E-01	0.2	Def8	4E-01	0.2
Tmem50b	5E-01	0.2	Zfp560	9E-04	0.2
D130017N08Rik	2E-02	0.2	Tspan31	4E-05	0.2
Tmem59	3E-26	0.2	Jrk	2E-01	0.2
Kif3a	1E-02	0.2	Chchd10	3E-01	0.2
Hmga1	8E-01	0.2	Gm12258	9E-01	0.2
Fuz	2E-01	0.2	1700084C06Rik	6E-01	0.2
Gyg	2E-01	0.2	Tmem42	5E-01	0.2
Calhm5	9E-02	0.2	Hipk2	8E-04	0.2
Cers2	2E-03	0.2	Cbr1	1E-02	0.2
Cd276	5E-01	0.2	Ccpg1	6E-02	0.2
Aga	1E-07	0.2	Tm2d3	7E-04	0.2
Rnpep	2E-01	0.2	2810402E24Rik	9E-01	0.2
Ninl	2E-01	0.2	C030034I22Rik	8E-01	0.2
Dancr	1E-01	0.2	Zkscan8	2E-01	0.2
Itgb5	4E-07	0.2	Poglut2	2E-01	0.2
Ppox	8E-01	0.2	Stxbp3	6E-02	0.2
Hacl1	7E-01	0.2	Gpi1	6E-06	0.2
Coa4	8E-01	0.2	Tmem199	5E-01	0.2
Cnnm3	1E-01	0.2	Gde1	1E-01	0.2
P4ha2	2E-06	0.2	Itfg2	8E-01	0.2
Lrch2	9E-01	0.2	Fbxl20	1E-02	0.2
Vps26b	1E-01	0.2	Mettl5	5E-01	0.2
Mapre2	1E-01	0.2	Ajuba	6E-01	0.2
Ggt7	8E-02	0.2	Ago4	8E-01	0.2
Srfbp1	2E-01	0.2	Scyl2	3E-01	0.2
Slc25a12	3E-02	0.2	Sdhaf1	6E-01	0.2
Fuca1	3E-11	0.2	Ndrg1	2E-02	0.2
Llph	3E-05	0.2	Rars2	3E-01	0.2
Stard10	2E-02	0.2	Ulk1	1E+00	0.2
Ppp2r3c	1E-01	0.2	Gm48678	9E-01	0.2
Gm9844	3E-02	0.2	Yrdc	6E-01	0.2
Wdr34	9E-01	0.2	Klhl12	7E-01	0.2
Grik5	5E-01	0.2	Prdx6	5E-03	0.2
Smim20	5E-03	0.2	Ppan	3E-01	0.2
Otulin	9E-01	0.2	Uqcc3	6E-07	0.2
Inf2	3E-01	0.2	Hivep1	6E-01	0.2
Snx15	1E-01	0.2	Kmt5c	9E-02	0.2
Ube2e2	5E-08	0.2	Rac3	8E-03	0.2
Adprm	6E-01	0.2	Tmem176b	3E-08	0.2
Sdf2	1E-08	0.2	Selenoo	4E-01	0.2
Wdr20	4E-01	0.2	Osbpl2	5E-01	0.2
Mks1	2E-01	0.2	Zfp521	2E-07	0.2
Ap4s1	4E-02	0.2	Slu7	4E-01	0.2
Ak4	7E-01	0.2	Coq4	8E-01	0.2
Tmem203	7E-01	0.2	Aktip	2E-01	0.2
Zscan12	8E-01	0.2	Jmy	3E-01	0.2
Slc25a26	9E-01	0.2	Pip4k2c	4E-01	0.2
Pot1b	3E-01	0.2	Dcp1b	7E-01	0.2
Hnrnph3	4E-05	0.2	Procr	5E-01	0.2
Zscan22	4E-01	0.2	Zfp383	7E-01	0.2
Ino80dos	7E-01	0.2	Rida	7E-01	0.2
Ube2e3	9E-07	0.2	Mamld1	4E-01	0.2
Oplah	1E+00	0.1	Bysl	2E-01	0.2
Trit1	1E+00	0.1	Junos	8E-01	0.2
Ap1m1	3E-01	0.1	Trappc4	1E-04	0.2
Trp53bp1	1E-01	0.1	Snx6	4E-09	0.2
Enpp5	3E-01	0.1	Mus81	4E-01	0.1
5031425E22Rik	8E-04	0.1	Chmp7	8E-01	0.1
Tmem62	9E-01	0.1	Lipo3	6E-01	0.1
Loxl1	6E-04	0.1	Cage1	4E-01	0.1
Fkbp10	3E-06	0.1	Iffo2	3E-01	0.1
Tax1bp3	7E-04	0.1	D330023K18Rik	7E-01	0.1
Gpx1	3E-13	0.1	Slc12a9	7E-01	0.1
Tmem230	3E-02	0.1	Fbxo36	8E-01	0.1
Nf2	3E-01	0.1	Tkfc	2E-01	0.1
Ift52	4E-03	0.1	4931414P19Rik	3E-01	0.1
Epdr1	5E-05	0.1	Fkbpl	9E-01	0.1
Cyb5r1	9E-04	0.1	Zfp146	7E-01	0.1
Zfp513	7E-01	0.1	Mapk13	9E-01	0.1
Wdr4	1E+00	0.1	Antxr1	9E-04	0.1
Rcor2	8E-01	0.1	Ccdc84	5E-02	0.1

Articular/Synovial Fibroblast					
Control			NICD2		
Gene	*p*	FC	Gene	*p*	FC
Grhpr	4E-03	0.4	Cstb	5E-21	0.4
Hdx	8E-01	0.4	Ubiad1	8E-01	0.4
Hspa12a	1E+00	0.4	Wdr81	9E-01	0.4
Agrp	5E-01	0.4	Vwa5a	2E-06	0.4
Mfsd8	7E-01	0.4	Wdr45	3E-01	0.4
Lncpint	6E-08	0.4	Dync2h1	5E-04	0.4
Cdkl2	4E-01	0.4	Ephb6	2E-01	0.4
Morn2	3E-04	0.4	B4gat1	2E-02	0.4
Ctns	1E-01	0.4	Acot13	3E-08	0.4
Gstk1	6E-02	0.4	Emc9	2E-03	0.4
Sumf1	3E-03	0.4	Nat2	7E-01	0.4
Slc38a4	3E-01	0.4	Vps35l	6E-03	0.4
Rbm43	4E-04	0.4	Ppp2r1b	5E-01	0.4
Irf1	4E-02	0.4	Crtc2	8E-01	0.4
Pafah2	1E-02	0.4	Tbkbp1	4E-01	0.4
Gpam	7E-01	0.4	Naa80	3E-01	0.4
Adam19	1E-01	0.4	Impact	1E-11	0.4
Zfp667	2E-01	0.4	1700030K09Rik	5E-01	0.4
Gm9801	3E-01	0.4	Coa7	6E-01	0.4
Zfp790	8E-01	0.4	Ccl2	5E-01	0.4
Kif9	5E-01	0.4	Gm14966	1E+00	0.4
Cd1d1	9E-04	0.4	Ift22	1E-03	0.4
Mef2d	2E-04	0.4	Man2b2	1E-01	0.4
Lrfn3	8E-01	0.4	Armc9	4E-02	0.4
Pim1	1E-01	0.4	Scpep1	3E-18	0.4
Xylt2	8E-01	0.4	3010001F23Rik	4E-01	0.4
Wbp1l	9E-04	0.4	Mllt6	8E-01	0.4
Man2a2	3E-02	0.4	4931414P19Rik	9E-01	0.4
Fer	1E-08	0.4	Rev3l	9E-08	0.4
P4htm	3E-01	0.4	Adck1	7E-01	0.4
Zfp874a	2E-01	0.4	Evi2a	4E-01	0.4
Plekha5	8E-01	0.4	Klf8	7E-01	0.4
Slc12a6	8E-02	0.4	Zmiz1	8E-07	0.4
Plekhb2	6E-01	0.4	Pde3a	2E-02	0.4
Ift22	4E-02	0.4	Tpra1	7E-01	0.4
Nsmce2	1E-12	0.4	Prkaa1	3E-01	0.4
Fam129b	8E-04	0.4	Thumpd2	5E-01	0.4
Vav2	7E-01	0.4	Zcchc2	8E-01	0.4
Pard3b	6E-09	0.4	Mthfs	2E-01	0.4
Brdt	8E-01	0.4	Chrna1os	8E-01	0.4
Ttll7	8E-03	0.4	Pced1b	8E-01	0.4
Gm47167	9E-01	0.4	Arrdc4	9E-02	0.4
Efna2	5E-01	0.4	Rab43	1E-01	0.4
Naaa	2E-01	0.4	Arhgdib	3E-02	0.4
Itpkb	2E-02	0.4	Shisa5	2E-09	0.4
Exoc4	1E-13	0.4	Mlkl	8E-01	0.4
Zfhx2	1E+00	0.4	Mvp	6E-03	0.4
Pgpep1	4E-04	0.4	Cul9	4E-02	0.4
Ermp1	9E-01	0.4	Fgd4	2E-01	0.4
Fnip1	6E-09	0.4	Adar	4E-01	0.4
Rras	7E-17	0.4	Tnfsf13	9E-01	0.4
Gng7	3E-01	0.4	Grik5	4E-01	0.4
Foxo3	2E-02	0.4	Cryl1	3E-01	0.4
Naaladl2	3E-13	0.4	Snx21	4E-02	0.4
Plekhg5	4E-03	0.4	Lpin3	6E-01	0.4
Lrrc75a	9E-01	0.4	Catspere2	3E-01	0.4
Smurf2	1E-01	0.4	9530026P05Rik	2E-01	0.4
Azin2	1E-02	0.4	Rfng	5E-01	0.4
Uhrf1bp1l	4E-04	0.4	Gm44148	2E-01	0.4
Gm20342	6E-01	0.4	Tsc1	7E-02	0.4
Fam189b	4E-02	0.4	Klhdc8b	3E-01	0.4
Irf7	2E-03	0.4	Dlgap4	2E-03	0.4
Trim68	9E-01	0.4	Rras	2E-11	0.4
Gemin8	1E-01	0.4	Tyw3	6E-01	0.4
Telo2	3E-01	0.4	Lhpp	7E-01	0.4
Ccdc181	5E-01	0.4	Rpain	2E-01	0.4
Hspb8	3E-05	0.4	Zfp975	3E-01	0.4
Podxl2	3E-05	0.4	Sh3d19	2E-09	0.4
Wwox	1E-08	0.4	Atp6v0d1	9E-06	0.4
Cbr2	7E-04	0.4	Rufy3	1E-02	0.4
Acly	1E-09	0.4	Retreg1	2E-01	0.4
Wdtc1	6E-02	0.4	Nphs1	1E+00	0.4
Fahd1	5E-02	0.4	Vim	2E-30	0.4

Limb Mesenchyme

Control Gene	p	FC	NICD2 Gene	p	FC
4833439L19Rik	3E-15	0.3	Vamp5	4E-08	0.3
Arhgef18	5E-11	0.3	Morn1	3E-05	0.3
Sesn3	2E-20	0.3	Ppic	1E-31	0.3
Gpc1	2E-09	0.3	Thada	2E-08	0.3
Tma16	7E-08	0.3	Tmem47	1E-04	0.3
Nsun5	5E-06	0.3	Gtf2i	2E-21	0.3
Iffo2	1E-08	0.3	Qsox1	1E-17	0.3
Egfr	3E-21	0.3	Zfp618	1E-07	0.3
Mpped2	2E-10	0.3	Spg20	4E-15	0.3
Zzz3	3E-15	0.3	Gm49336	2E-11	0.3
Per1	7E-09	0.3	Gpc1	3E-09	0.3
Magohb	3E-11	0.3	Kctd6	4E-05	0.3
Tspan3	7E-38	0.3	Parp8	2E-10	0.3
Prpf40b	2E-07	0.3	Six5	3E-04	0.3
Aplp2	1E-43	0.3	Nek1	4E-07	0.3
Lats2	1E-16	0.3	Zfp60	2E-09	0.3
Phf12	1E-12	0.3	Gm11508	5E-03	0.3
Alkbh1	8E-13	0.3	Gm16279	2E-04	0.3
Gm50013	4E-05	0.3	Lifr	9E-02	0.3
Gtf2ird2	3E-06	0.3	Dnajc17	1E-08	0.3
Macrod2	3E-11	0.3	Gtpbp8	2E-10	0.3
Slc12a4	1E-09	0.3	Oma1	3E-08	0.3
Bicc1	4E-23	0.3	Btaf1	6E-15	0.3
Zfp282	1E-07	0.3	Fgf2	8E-10	0.3
Grwd1	2E-05	0.3	Slc45a4	2E-08	0.3
Tra2b	3E-21	0.3	Metap1	2E-04	0.3
Gm4117	4E-07	0.3	Pheta1	2E-05	0.3
Ciapin1	2E-09	0.3	Abcc4	4E-06	0.3
Scfd1	2E-19	0.3	Tent4b	8E-09	0.3
Rwdd4a	1E-10	0.3	Klhl11	7E-07	0.3
Raver2	2E-03	0.3	Dzip3	3E-10	0.3
Ptk7	2E-10	0.3	Mark3	4E-14	0.3
Ehbp1	7E-20	0.3	Ghitm	1E-27	0.3
Ebag9	5E-12	0.3	Copg2	7E-11	0.3
Ecrg4	9E-34	0.3	Magohb	2E-09	0.3
Slc22a21	9E-08	0.3	Zfp971	5E-03	0.3
Urb1	1E-04	0.3	Sybu	6E-07	0.3
Mybbp1a	1E-14	0.3	Kctd3	3E-07	0.3
Slc45a4	3E-07	0.3	Whamm	5E-03	0.3
Gm32282	3E-04	0.3	Ormdl3	4E-12	0.3
Rtn4ip1	1E-02	0.3	Car6	1E-04	0.3
Ninj1	2E-17	0.3	Tpd52l1	2E-05	0.3
Ctnna1	5E-23	0.3	Uck2	4E-09	0.3
Atp1a1	4E-20	0.3	Samd12	3E-05	0.3
Chd1	8E-10	0.3	Poglut2	5E-12	0.3
Bach2	8E-05	0.3	Plxnb2	6E-14	0.3
Far1os	1E-07	0.3	Snhg14	5E-04	0.3
Kdelr2	1E-30	0.3	Samd14	7E-04	0.3
Xkr4	4E-01	0.3	Zxdb	2E-04	0.3
Necap2	3E-11	0.3	Tmbim1	7E-11	0.3
Retreg1	7E-14	0.3	Resf1	2E-06	0.3
Mlec	4E-34	0.3	Lrrc59	3E-17	0.3
Yars	4E-14	0.3	Casc4	3E-20	0.3
Zfp618	9E-10	0.3	Rbm4	3E-09	0.3
Lrrc41	8E-13	0.3	Tmem201	1E-04	0.3
Syvn1	2E-07	0.3	Eya2	2E-04	0.3
Gm49336	7E-10	0.3	Slc25a12	2E-08	0.3
Sgk1	5E-07	0.3	Ddx19b	2E-03	0.3
9930104L06Rik	4E-05	0.3	Cx3cl1	6E-03	0.3
Atrn	6E-13	0.3	Kif5b	9E-27	0.3
Megf6	5E-10	0.3	Eaf1	5E-06	0.3
Rbsn	8E-09	0.3	Slc35b2	8E-13	0.3
Ip6k2	6E-12	0.3	Mprip	1E-20	0.3
Poglut2	2E-10	0.3	Cebpg	5E-10	0.3
Maea	5E-14	0.3	Setd6	2E-04	0.3
Tex14	2E-14	0.3	Zfp870	2E-04	0.3
Uspl1	1E-06	0.3	Anks3	2E-08	0.3
Rnaset2a	4E-08	0.3	Mrc2	2E-12	0.3
Armh4	3E-12	0.3	Efna4	2E-06	0.3
Marcksl1	2E-13	0.3	4833439L19Rik	3E-10	0.3
Erc1	6E-14	0.3	Taf5l	3E-05	0.3
4930556M19Rik	7E-05	0.3	Bdnf	2E-05	0.3
Rrs1	2E-09	0.3	Sec31a	2E-34	0.3

Chondrogenic

Control Gene	p	FC	NICD2 Gene	p	FC
Slc39a14	2E-08	0.4	Lmo4	8E-18	0.4
Lpar6	3E-02	0.4	Wdpcp	3E-06	0.4
Fhit	4E-13	0.4	Swap70	4E-09	0.4
Slc25a12	1E-08	0.4	Frs2	2E-09	0.4
Gm11944	6E-03	0.4	Ints13	7E-04	0.4
Tex2	3E-02	0.4	Tef	3E-08	0.4
Thbd	2E-13	0.4	Cox15	6E-02	0.4
Necap2	7E-15	0.4	Smim27	6E-09	0.4
Tpcn1	6E-03	0.4	Cpeb2	7E-01	0.4
Arpc5l	8E-24	0.4	E130317F20Rik	2E-01	0.4
Gm14966	7E-03	0.4	Hhat	3E-02	0.4
Gm20342	7E-03	0.4	Mpp6	6E-11	0.4
Gm43672	2E-04	0.4	Gm38604	3E-01	0.4
Gm3448	5E-02	0.4	Gm36975	8E-02	0.4
Pex11a	1E-06	0.4	Top2b	7E-23	0.4
Dnajc3	3E-40	0.4	Gli2	4E-02	0.4
Rab31	5E-12	0.4	Fahd2a	1E-02	0.4
Mbnl1	5E-34	0.4	Kcnrg	4E-02	0.4
Gm15564	2E-03	0.4	Angpt1	1E-06	0.4
Dym	1E-09	0.4	Polr3g	1E-01	0.4
P2ry10b	2E-04	0.4	Rnf113a1	9E-02	0.4
Asrgl1	5E-05	0.4	Gfpt1	5E-15	0.4
Ring1	2E-09	0.4	Ing4	4E-10	0.4
Mterf1a	2E-02	0.4	Mxd1	2E-01	0.4
Pdlim5	5E-24	0.4	Cetn2	7E-10	0.4
Tpk1	3E-08	0.4	Gab1	2E-09	0.4
Hyal2	1E-07	0.4	Asxl1	5E-10	0.4
Arhgef5	9E-07	0.4	Cyp2u1	2E-01	0.4
Trafd1	8E-10	0.4	Pou2f1	3E-03	0.4
Ttll4	3E-02	0.4	Fat1	2E-16	0.4
Acyp1	1E-11	0.4	Smurf2	1E-12	0.4
Ppp3ca	5E-39	0.4	Nedd4l	7E-07	0.4
Golim4	9E-28	0.4	Zfp60	2E-03	0.4
Stat5a	2E-04	0.4	Bbx	6E-17	0.4
Strbp	6E-25	0.4	Aagab	3E-04	0.4
Ndufaf7	7E-06	0.4	Zfp12	2E-01	0.4
Lyst	2E-03	0.4	Gspt2	3E-03	0.4
Dgke	5E-02	0.4	Ptch1	8E-02	0.4
Gm44899	3E-02	0.4	Vps39	2E-03	0.4
Bbx	8E-22	0.4	Gm10561	7E-02	0.4
Evi2a	9E-02	0.4	Sppl2a	2E-13	0.4
Zfp112	1E-03	0.4	Coq8a	5E-03	0.4
Rab28	7E-21	0.4	Phactr2	2E-09	0.4
Kdm6a	2E-08	0.4	Mbnl2	6E-36	0.4
Phactr1	4E-05	0.4	Galnt11	2E-07	0.4
Glt8d2	3E-04	0.4	D5Ertd579e	2E-08	0.4
Trim41	8E-06	0.4	9430091E24Rik	9E-02	0.4
Smad7	1E-08	0.4	Phip	1E-12	0.4
BC005537	1E-12	0.4	Crip2	2E-21	0.4
Serpina3n	7E-03	0.4	1810024B03Rik	2E-02	0.4
Lin52	2E-05	0.4	Myo1e	5E-12	0.4
Ints4	3E-06	0.4	Metrn	1E-07	0.4
Otud3	1E-02	0.4	Shld1	1E-02	0.4
Tmem132a	1E-05	0.4	Vwa8	5E-06	0.4
Vldlr	6E-05	0.4	Mafk	3E-01	0.4
Uggt1	1E-12	0.4	Braf	2E-11	0.4
Ggact	9E-07	0.4	C3	1E-06	0.4
Atg3	3E-23	0.4	Gpbp1l1	1E-07	0.4
Ppp1r9a	1E-03	0.4	Rbbp9	1E-04	0.4
Loxl3	2E-06	0.4	Ift80	3E-04	0.4
Pigb	2E-03	0.4	Fam174a	2E-15	0.4
Ecpas	7E-23	0.4	Sorbs1	1E-07	0.4
Hoxa7	3E-04	0.4	Terf2ip	5E-04	0.4
Lum	1E-14	0.4	Usp46	5E-04	0.4
Wdr35	5E-11	0.4	Zc3h8	2E-01	0.4
Hoxc5	2E-03	0.4	Cyld	2E-11	0.4
Gm5165	1E-01	0.4	Vps51	4E-03	0.4
Pigv	2E-02	0.4	Emb	1E-20	0.4
Gm37768	7E-04	0.4	Kat6b	7E-12	0.4
Rps6ka1	8E-02	0.4	Slc39a11	3E-04	0.4
Macrod1	6E-18	0.4	Sp2	8E-02	0.4
Ap1g1	3E-15	0.4	Kansl1l	6E-08	0.4
Akap9	1E-25	0.4	Cdon	1E-09	0.4

Fibroblast

Control Gene	p	FC	NICD2 Gene	p	FC
Arhgef28	5E-08	0.3	Parp10	5E-03	0.3
Zbtb8a	3E-05	0.3	Gm28875	3E-03	0.3
Arhgef40	5E-25	0.3	Idh1	7E-78	0.3
Src	2E-07	0.3	Cdc42ep3	6E-11	0.3
Pdzd4	5E-09	0.3	Prkra	6E-21	0.3
Zfp709	3E-03	0.3	Arhgef40	1E-14	0.3
Arpc1b	4E-143	0.3	Wdr81	6E-03	0.3
Gm12905	5E-07	0.3	Comt	2E-34	0.3
Shisa5	3E-47	0.3	Ice1	3E-14	0.3
Vrk3	6E-12	0.3	Polg2	1E-02	0.3
A4galt	3E-12	0.3	Vrk3	8E-07	0.3
Gm44148	2E-07	0.3	Evi5	5E-20	0.3
Gstt3	6E-13	0.3	Tsku	1E-02	0.3
Mroh1	1E-11	0.3	Plekha2	5E-13	0.3
Pmm1	2E-48	0.3	Ccdc171	5E-07	0.3
Ice1	4E-22	0.3	Wrn	1E-09	0.3
Slc41a3	1E-06	0.3	Slc2a10	5E-04	0.3
Cemip2	3E-24	0.3	Nfib	3E-39	0.3
Sh3kbp1	3E-37	0.3	Tbx3	5E-02	0.3
Aprt	4E-131	0.3	Nckap5l	3E-05	0.3
Mfsd3	2E-06	0.3	Hdac11	1E-05	0.3
Mvp	2E-22	0.3	Abca1	7E-13	0.3
Ifngr1	1E-26	0.3	Tsc22d3	3E-05	0.3
Xaf1	2E-21	0.3	Armcx1	2E-12	0.3
Akap13	4E-49	0.3	Rps27rt	1E-13	0.3
Fdxr	2E-05	0.3	Abhd11	2E-07	0.3
Dtnbp1	3E-62	0.3	Gba	3E-06	0.3
Comt	6E-59	0.3	Sh3kbp1	2E-21	0.3
Slc39a1	3E-29	0.3	Bcor	2E-05	0.3
Gclm	1E-42	0.3	Ptprm	2E-24	0.3
Igf2bp1	2E-05	0.3	Slc39a1	3E-18	0.3
Pgap1	1E-04	0.3	Morrbid	4E-05	0.3
1110046J04Rik	4E-16	0.3	Irx3	5E-04	0.3
Fbh1	3E-13	0.3	Prickle2	4E-05	0.3
Tnfrsf1a	1E-47	0.3	Meis3	5E-07	0.3
Cldn12	3E-07	0.3	Prkab2	3E-03	0.3
Cd55	2E-82	0.3	Nynrin	3E-15	0.3
Srgap2	2E-37	0.3	Mios	1E-08	0.3
Cap1	8E-34	0.3	Plekho2	2E-07	0.3
Rps27rt	1E-22	0.3	Npepps	7E-32	0.3
Fkbp7	1E-85	0.3	Gse1	2E-05	0.3
Dtd1	1E-37	0.3	Sep8	1E-16	0.3
Numbl	5E-25	0.3	Sema6a	4E-06	0.3
Rps28	0E+00	0.3	Ccdc93	2E-09	0.3
Loxl1	3E-67	0.3	Fkbp7	5E-48	0.3
Irf2bp1	9E-24	0.3	Smarcd2	1E-09	0.3
3300002I08Rik	4E-09	0.3	Gmppa	2E-13	0.3
Hipk3	1E-27	0.3	Stim1	5E-13	0.3
Maml2	1E-61	0.3	Ap3m2	1E-02	0.3
Fsd1	2E-02	0.3	Rac3	1E-08	0.3
Cmtm6	3E-05	0.3	Csgalnact1	4E-26	0.3
Cmc4	2E-10	0.3	Serpinb9	7E-04	0.3
C1s1	9E-24	0.3	Vegfb	5E-25	0.3
Slc30a7	2E-15	0.3	Cul7	4E-09	0.3
Tm7sf2	3E-09	0.3	Ncor2	9E-17	0.3
Sat2	6E-12	0.3	Gm10941	1E-02	0.3
Tnfsf12	1E-30	0.3	Atoh8	2E-04	0.3
Phf11d	3E-10	0.3	Zxdc	4E-03	0.3
Pcolce2	8E-37	0.3	Vps8	9E-09	0.3
Traf5	1E-09	0.3	Runx1	2E-25	0.3
Rpl28	0E+00	0.3	Flcn	5E-04	0.3
Parp9	4E-16	0.3	Usp18	8E-04	0.3
Stard4	2E-06	0.3	Gm16008	6E-02	0.3
Agtrap	5E-17	0.3	Dapk1	1E-17	0.3
Pla2g15	1E-08	0.3	Rxfp1	4E-03	0.3
Arl5a	2E-22	0.3	Dip2a	8E-06	0.3
Map1a	2E-35	0.3	Dpy19l1	1E-06	0.3
Iah1	2E-39	0.3	Gpr153	7E-03	0.3
Tmem26	1E-06	0.3	Arl5a	2E-14	0.3
Rilpl1	8E-25	0.3	Ptgfrn	2E-05	0.3
Paqr7	3E-05	0.3	Ptgs1	9E-03	0.3
Flcn	1E-05	0.3	Zfp655	4E-08	0.3
Pusl1	2E-19	0.3	Arpc1b	7E-69	0.3

Fibroblast?

Control Gene	p	FC	NICD2 Gene	p	FC
9330159M07Rik	6E-33	0.8	Anxa3	8E-59	0.8
4930414N06Rik	4E-16	0.8	5730522E02Rik	5E-38	0.8
Pstpip2	9E-19	0.8	Ccdc74a	1E-24	0.8
Calm2	1E-160	0.8	Mast2	8E-66	0.8
Rbm3	2E-157	0.8	Dbr1	1E-35	0.8
1700066M21Rik	3E-21	0.8	Cby1	3E-48	0.8
Emd	7E-62	0.8	Cltb	8E-58	0.8
Nup50	4E-50	0.8	Slc24a5	5E-52	0.8
Saal1	2E-41	0.8	Serp2	2E-53	0.8
Kank3	2E-13	0.8	Gm11110	5E-26	0.8
Nrk	2E-33	0.8	A630089N07Rik	3E-55	0.8
Fam20c	2E-31	0.8	Ifit2	5E-47	0.8
Rpp30	2E-48	0.8	Pea15a	2E-69	0.8
Gm10501	1E-20	0.8	Slc25a13	5E-58	0.8
Dock11	4E-40	0.8	Pla1a	4E-41	0.8
Tspo	5E-84	0.8	Nxph4	3E-20	0.8
Nop9	7E-31	0.8	Loxl2	7E-46	0.8
Nbn	4E-40	0.8	Hmgxb4	3E-60	0.8
Aplf	1E-34	0.8	Map4k2	6E-27	0.8
U2af1	3E-105	0.8	Gm26590	1E-18	0.8
Hnrnpa3	1E-117	0.8	Dclre1a	4E-41	0.8
Tubb3	1E-14	0.8	Gm47283	5E-59	0.8
Srpk1	2E-61	0.8	B3galnt1	8E-48	0.8
Fut10	5E-36	0.8	Tead4	3E-13	0.8
Mzt1	1E-39	0.8	Acan	9E-30	0.8
Nin	3E-37	0.8	Anapc11	2E-125	0.8
LTO1	3E-32	0.8	C79798	2E-24	0.8
Cabp1	2E-15	0.8	Sephs1	8E-45	0.8
Lancl2	6E-29	0.8	Galm	3E-32	0.8
Svop	1E-12	0.8	Gm49692	2E-32	0.8
Bcl7a	9E-22	0.8	G3bp1	3E-113	0.8
Blvra	9E-38	0.8	Gm44649	6E-20	0.8
Arrb2	9E-20	0.8	Gm1673	2E-87	0.8
Ddx20	4E-33	0.8	Adgre5	2E-43	0.8
Fam216a	2E-44	0.8	C030037D09Rik	1E-20	0.8
Gm5914	2E-16	0.8	Phf6	1E-57	0.8
Ror2	2E-49	0.8	Phgdh	8E-71	0.8
Smyd4	1E-41	0.8	Rbbp4	3E-142	0.8
Mustn1	5E-22	0.8	Riox1	7E-22	0.8
Stk17b	4E-34	0.8	Mcmbp	2E-59	0.8
Axl	7E-46	0.8	Rnf219	8E-53	0.8
Eno3	1E-25	0.8	Ipo11	7E-53	0.8
Oscp1	3E-21	0.8	Zcchc8	2E-56	0.8
Smc5	1E-48	0.8	Pgm2l1	8E-26	0.8
Ereg	5E-28	0.8	Apln	4E-17	0.8
Tfdp2	2E-57	0.8	Slf2	2E-66	0.8
Rdh11	7E-33	0.8	AV099323	4E-18	0.8
Mlkl	6E-35	0.8	Cbx3	5E-176	0.8
Ruvbl2	9E-35	0.8	Snrnp40	6E-69	0.8
Jade1	6E-42	0.8	Usp49	7E-39	0.8
B3gnt2	8E-38	0.8	Gm42047	1E-20	0.8
Sfmbt1	5E-43	0.8	Mthfd2	2E-75	0.8
Cst6	3E-33	0.8	Iqgap1	1E-108	0.8
Zfp820	3E-17	0.8	Mrpl28	2E-80	0.8
Nptx1	8E-22	0.8	A430073D23Rik	7E-20	0.8
Ado	1E-47	0.8	Arhgap18	2E-44	0.8
Golm1	3E-39	0.8	Brd3	1E-67	0.8
Mapk13	1E-21	0.8	Ppp1r14a	1E-16	0.8
Gm41724	3E-30	0.8	Setdb2	7E-37	0.8
Nudt5	8E-64	0.8	Nop9	1E-45	0.8
Dgcr8	3E-44	0.8	Gm15581	3E-26	0.8
Gm42047	2E-12	0.8	Rbm3	3E-180	0.8
Zbtb8a	1E-33	0.8	Gm21887	2E-17	0.8
Pole4	9E-70	0.8	Toe1	8E-42	0.8
Mpp2	4E-24	0.8	Notch1	2E-62	0.8
Specc1l	3E-35	0.8	Rnf26	5E-45	0.8
Ckap4	3E-141	0.8	Mustn1	8E-22	0.8
Dbr1	9E-35	0.8	Rgs12	2E-63	0.8
Tnfrsf10b	4E-36	0.8	Prx	7E-21	0.8
Dot1l	9E-37	0.8	Tpd52	6E-49	0.8
Srrd	1E-15	0.8	Ift80	2E-33	0.8
D1Ertd622e	7E-18	0.8	Cnih4	1E-126	0.8
Sema3e	9E-29	0.8	Dcbld2	9E-53	0.8

Undefined

Control Gene	p	FC	NICD2 Gene	p	FC
B3gat3	4E-02	0.1	Mrpl24	2E-06	0.1
Trmt10b	5E-01	0.1	Aspscr1	8E-01	0.1
Spsb2	4E-01	0.1	Thap3	7E-02	0.1
Cib1	3E-01	0.1	Irgm1	4E-01	0.1
Mex3a	7E-01	0.1	Cnbp	2E-07	0.1
Tmem216	7E-01	0.1	Meis1	7E-01	0.1
Tmed1	1E-04	0.1	Aebp1	3E-30	0.1
Slc25a14	5E-01	0.1	Elmod3	9E-01	0.1
Sh3glb2	4E-02	0.1	Id3	8E-09	0.1
Wdr35	1E-02	0.1	Eno2	3E-01	0.1
Zfp629	8E-01	0.1	Gm47071	8E-01	0.1
Uqcc2	3E-18	0.1	Hoxc9	3E-01	0.1
Faap20	7E-02	0.1	Wls	1E-12	0.1
Rragb	5E-01	0.1	Disp1	6E-01	0.1
Klhdc2	1E-09	0.1	Scarf2	8E-03	0.1
Uck1	4E-01	0.1	Tmem62	6E-01	0.1
2310009B15Rik	4E-04	0.1	Hmgcl	7E-01	0.1
Thra	6E-11	0.1	Zfp1	7E-01	0.1
Spata6	1E-01	0.1	Zfp563	6E-01	0.1
Chmp6	2E-01	0.1	Wdr74	4E-01	0.1
Asb6	1E-01	0.1	Sgcd	2E-05	0.1
Kif13b	3E-02	0.1	2900093K20Rik	1E-01	0.1
Dleu2	2E-04	0.1	2610044O15Rik8	7E-01	0.1
Map6	1E-01	0.1	Mtrf1	1E-01	0.1
Snx29	6E-03	0.1	Shc1	7E-02	0.1
Alkbh5	4E-02	0.1	Dusp12	1E+00	0.1
Cpeb4	3E-02	0.1	Dusp18	7E-01	0.1
Usp3	9E-03	0.1	2310015A10Rik	7E-01	0.1
Raly	6E-06	0.1	Ankrd13c	6E-01	0.1
A930001C03Rik	8E-01	0.1	Bsg	1E-26	0.1
Arl3	9E-12	0.1	Gm16740	9E-01	0.1
Coq8b	7E-01	0.1	Gm37768	1E+00	0.1
Cyb5r2	5E-02	0.1	Clec11a	2E-04	0.1
Sgk1	4E-01	0.1	Slc25a33	8E-01	0.1
Gli3	8E-05	0.1	Cdk8	1E+00	0.1
Fbrsl1	4E-01	0.1	Ormdl2	9E-01	0.1
Pmp22	1E-04	0.1	Mgat4b	3E-01	0.1
Gtpbp6	6E-01	0.1	Pdcd4	3E-07	0.1
Pfdn1	9E-14	0.1	Ifit2	8E-01	0.1
Mmaa	6E-01	0.1	Cxcl5	4E-03	0.1
Dync2li1	3E-01	0.1	Cetn2	4E-01	0.1
Zfp39	6E-01	0.1	Rft1	2E-01	0.1
Kdm5d	5E-01	0.1	Gm48765	5E-01	0.1
Gatd1	3E-01	0.1	Arhgap24	6E-04	0.1
Impdh1	8E-01	0.1	Nbr1	1E-01	0.1
Egln2	6E-02	0.1	Slc24a3	2E-03	0.1
Tgoln1	1E-07	0.1	Mageh1	3E-01	0.1
Mrpl46	9E-01	0.1	Tsr3	8E-01	0.1
Tex9	8E-01	0.1	Kiz	3E-01	0.1
Chid1	1E-03	0.1	Lgmn	2E-04	0.1
Ccnd2	1E-14	0.1	Emp2	2E-02	0.1
Fstl3	1E+00	0.1	Bbs9	4E-01	0.1
Mtss2	4E-01	0.1	2810403D21Rik	9E-03	0.1
Fahd1	8E-01	0.1	Bid	3E-01	0.1
Pycrl	8E-01	0.1	Pde10a	2E-03	0.1
Rpap2	9E-01	0.1	Emc4	2E-03	0.1
Snrnp35	3E-01	0.1	Cyth3	2E-01	0.1
Clp1	7E-01	0.1	Cyb5d2	2E-01	0.1
Ppfibp2	2E-01	0.1	Tnip2	1E-02	0.1
Ranbp10	4E-01	0.1	Uhrf1bp1l	1E-02	0.1
Polr2e	1E-04	0.1	Adck1	2E-01	0.1
Rab10os	3E-01	0.1	Tmem220	6E-01	0.1
Ssc5d	3E-03	0.1	Rsbn1l	7E-01	0.1
Zdhhc7	9E-02	0.1	Cnppd1	9E-01	0.1
Naga	5E-01	0.1	Gpnmb	4E-15	0.1
Stxbp6	1E-05	0.1	Zfp142	8E-02	0.1
Myc	7E-01	0.1	Tmem29	7E-02	0.1
Ube4bos1	5E-01	0.1	Atp6ap2	1E-02	0.1
Gde1	2E-03	0.1	Fam3a	4E-01	0.1
Hist1h1c	5E-02	0.1	2310009B15Rik	5E-04	0.1
Aebp1	1E-26	0.1	Laptm4a	4E-37	0.1
Rftn2	1E-01	0.1	Uqcc2	2E-15	0.1
Mrpl24	3E-05	0.1	Zfp583	2E-01	0.1

Articular/Synovial Fibroblast

Control Gene	p	FC	NICD2 Gene	p	FC
Ubox5	9E-01	0.4	Cc2d2a	6E-04	0.4
Il18	4E-02	0.4	Tbc1d17	4E-01	0.4
Ggta1	5E-01	0.4	Rmdn1	1E-05	0.4
Parp11	7E-02	0.4	Atg14	2E-01	0.4
Tmem94	1E+00	0.4	Cyba	2E-16	0.4
Cotl1	8E-02	0.4	Ddah2	4E-11	0.4
Ccdc173	4E-01	0.4	Nfia	1E-15	0.4
Otud1	8E-01	0.4	Arl6ip5	4E-13	0.4
Cpd	6E-04	0.4	Trim30d	4E-01	0.4
Parp9	1E-02	0.4	Vps13c	4E-07	0.4
Rragb	3E-01	0.4	Tmem184b	5E-02	0.4
Hexa	1E-12	0.4	Gm20342	9E-01	0.4
Fam214a	2E-02	0.4	Triobp	6E-02	0.4
Snx33	2E-01	0.4	Ephx1	8E-07	0.4
Pik3c3	2E-01	0.4	Arhgap20	1E-04	0.4
Tnks1bp1	6E-04	0.4	Trim47	7E-02	0.4
Lysmd4	1E-02	0.4	Nhlrc3	1E+00	0.4
Chrd	9E-01	0.4	2010315B03Rik	5E-01	0.4
Cd80	1E-01	0.4	Zfp703	2E-02	0.4
Fcgrt	1E-23	0.4	Zfp825	6E-01	0.4
Asah2	1E-02	0.4	Fbxo6	2E-02	0.4
Prkar1b	6E-02	0.4	Foxp1	5E-16	0.4
Parp10	1E+00	0.4	Hexdc	6E-01	0.4
Nr6a1	1E-03	0.4	Tead3	7E-01	0.4
Sgsm2	9E-02	0.4	Exoc4	1E-09	0.4
Manba	3E-01	0.4	Itgb1bp1	6E-05	0.4
Usp24	2E-05	0.4	Zfp868	2E-01	0.4
Clip1	2E-06	0.4	Pcbd2	8E-09	0.4
Ccdc74a	6E-01	0.4	Chchd5	4E-01	0.4
Nudt12	9E-01	0.4	Cpne2	2E-02	0.4
Gm46218	6E-01	0.4	Wnt16	5E-02	0.4
Gigyf1	2E-01	0.4	Hfe	1E-02	0.4
Acsf3	4E-01	0.4	Gm14858	8E-01	0.4
Plxna2	4E-04	0.4	Ephx2	8E-04	0.4
Fbxo46	9E-01	0.4	Mettl21a	3E-01	0.4
Heca	8E-02	0.4	Hdhd3	9E-01	0.4
Dock11	4E-01	0.4	Rbm43	5E-01	0.4
Ephx2	1E-03	0.4	Galns	8E-01	0.4
4930447C04Rik	6E-01	0.4	Ech1	5E-12	0.4
Gm14403	9E-01	0.4	Zmat3	3E-03	0.4
Tmem37	4E-02	0.4	Agbl5	9E-01	0.4
Micall2	3E-01	0.4	Ica1	7E-01	0.4
Tlr3	3E-01	0.4	Ptgr1	7E-02	0.4
6530409C15Rik	6E-01	0.4	Parp10	4E-01	0.4
Snx21	3E-02	0.4	BC029722	1E-01	0.4
Nsmf	2E-01	0.4	Fundc2	1E-20	0.4
Itih5	6E-03	0.4	Phkg2	6E-01	0.4
Oasl2	9E-02	0.4	Pnpla6	6E-01	0.4
Rufy3	3E-02	0.4	Man2a2	2E-01	0.4
Fbln5	2E-05	0.4	Ilvbl	2E-01	0.4
Tbc1d32	6E-02	0.4	Emc2	5E-09	0.4
Igfbp6	2E-06	0.4	Dnhd1	6E-01	0.4
Gas2	2E-07	0.4	Birc3	1E+00	0.4
Smad3	3E-05	0.4	Gm3448	5E-01	0.4
Leng9	6E-01	0.4	Zc3h6	1E-01	0.4
Tbcel	7E-02	0.4	Lpcat2	8E-01	0.4
Cir1	3E-09	0.4	Cacnb2	4E-04	0.4
Zfp687	7E-01	0.4	Xpc	6E-01	0.4
Spa17	2E-01	0.4	Akr7a5	5E-05	0.4
Fam241b	2E-01	0.4	Bcas3	1E-05	0.4
Cxxc5	1E-05	0.4	Ano6	3E-08	0.4
Hs1bp3	1E-01	0.4	Gm16124	1E+00	0.4
Cnnm2	5E-01	0.4	Upp2	3E-04	0.4
Abca8b	8E-01	0.4	Napepld	8E-01	0.4
Wdr60	2E-03	0.4	Fam199x	6E-01	0.4
Ube4bos1	5E-01	0.4	Rdh13	6E-01	0.4
D3Ertd751e	2E-01	0.4	Pank4	5E-01	0.4
Gramd3	2E-01	0.4	Lrrc17	2E-11	0.4
Dsel	8E-04	0.4	Ypel2	6E-02	0.4
Map1lc3b	3E-29	0.4	Ckb	6E-05	0.4
Pnp	6E-10	0.4	Rcor2	7E-01	0.4
Lamb2	2E-04	0.4	Akap13	1E-09	0.4
Gm20732	5E-01	0.4	Cbr1	9E-06	0.4

Limb Mesenchyme

Control			NICD2		
Gene	*p*	FC	Gene	*p*	FC
Mief2	2E-04	0.3	Zfr	3E-21	0.3
Vamp5	1E-11	0.3	Zfp626	4E-03	0.3
Abhd13	8E-05	0.3	Dvl3	3E-07	0.3
Tmem214	5E-12	0.3	Dnaja4	8E-09	0.3
Gm12655	2E-05	0.3	Zfp94	8E-04	0.3
Kdm6b	6E-12	0.3	St3gal6	2E-10	0.3
Prkrip1	4E-10	0.3	Tbx3	2E-05	0.3
Mphosph6	1E-07	0.3	Arid5a	8E-05	0.3
Prpsap1	2E-12	0.3	Atg2b	3E-05	0.3
Ss18l2	1E-14	0.3	Ergic1	5E-16	0.3
Six5	3E-05	0.3	Agpat4	2E-07	0.3
Txlna	2E-14	0.3	Mmgt1	3E-05	0.3
Slc3a2	1E-22	0.3	Pld1	2E-10	0.3
Supt3	5E-11	0.3	Qk	9E-26	0.3
Alg12	5E-05	0.3	Pou6f1	1E-05	0.3
B230354K17Rik	4E-08	0.3	D10Wsu102e	2E-15	0.3
Med27	4E-10	0.3	Tmem167b	2E-09	0.3
Tdg	7E-10	0.3	Slc35e3	7E-07	0.3
Gpn2	9E-05	0.3	Arhgap28	6E-13	0.3
Khdc4	1E-11	0.3	Btg3	4E-13	0.3
Impg2	8E-04	0.3	Ktn1	4E-16	0.3
Slc2a10	2E-05	0.3	Impad1	9E-15	0.3
Fbxl12os	2E-04	0.3	Zfp335os	8E-03	0.3
Rabl6	7E-19	0.3	4930556M19Rik	5E-06	0.3
Rab5a	1E-21	0.3	Vps25	4E-03	0.3
Abitram	8E-09	0.3	Fstl3	3E-04	0.3
Akirin1	4E-12	0.3	Zc2hc1a	2E-11	0.3
Tubgcp5	2E-09	0.3	Csde1	2E-25	0.3
Mapkap1	9E-21	0.3	Pogz	7E-07	0.3
Smc6	4E-23	0.3	1500004A13Rik	3E-05	0.3
5330438D12Rik	1E-13	0.3	Tubgcp6	2E-04	0.3
Ankzf1	1E-06	0.3	Sertad2	7E-14	0.3
Plekhg1	6E-09	0.3	Usp30	2E-04	0.3
Cdk2ap1	6E-11	0.3	Dcun1d4	9E-07	0.3
Abce1	2E-16	0.3	Mettl16	5E-10	0.3
2410002F23Rik	3E-09	0.3	Stx6	4E-09	0.3
Zfp260	2E-18	0.3	Fra10ac1	3E-08	0.3
Armcx2	4E-10	0.3	Eif2d	1E-04	0.3
Armc7	6E-04	0.3	Abhd17c	1E-06	0.3
4921511C10Rik	2E-04	0.3	Def8	4E-06	0.3
Miga1	3E-05	0.3	Stambpl1	9E-10	0.3
Tigd5	3E-03	0.3	Smarcc1	6E-21	0.3
Phykpl	7E-09	0.3	Itgav	1E-10	0.3
Swap70	1E-09	0.3	Spcs3	2E-08	0.3
Galnt7	1E-07	0.3	Ppfia1	2E-09	0.3
Impad1	3E-15	0.3	Efnb2	6E-09	0.3
Lrp6	1E-23	0.3	Phc2	3E-12	0.3
Nf2	2E-11	0.3	D16Ertd472e	1E-05	0.3
Sbk1	8E-04	0.3	Cd2ap	5E-16	0.3
Phlpp1	1E-14	0.3	Cpq	3E-28	0.3
Gm16093	3E-05	0.3	Fgf13	2E-09	0.3
Paip2b	1E-06	0.3	B4galt3	2E-05	0.3
Golga3	1E-12	0.3	Gba2	3E-03	0.3
Paxbp1	1E-11	0.3	Ccnt1	8E-06	0.3
Golt1b	5E-12	0.3	Chst14	6E-07	0.3
Sertad2	4E-12	0.3	Cipc	1E-05	0.3
Gt(ROSA)26Sor	9E-06	0.3	Faf1	8E-19	0.3
Osbp	1E-09	0.3	Ica1	3E-07	0.3
Ripk2	3E-07	0.3	Thumpd3	1E-07	0.3
Tvp23b	4E-16	0.3	Nmt2	1E-06	0.3
Serpine2	4E-24	0.3	Rtkn	1E-07	0.3
Ptpn4	2E-10	0.3	Rassf5	7E-04	0.3
Sec23b	4E-08	0.3	Rrs1	1E-08	0.3
Itga6	2E-07	0.3	Alkbh1	2E-10	0.3
Spg20	4E-13	0.3	Pkd1	5E-10	0.3
Zyg11b	2E-12	0.3	Naaladl2	3E-20	0.3
Ddx54	1E-14	0.3	A230056P14Rik	3E-04	0.3
Crispld1	7E-50	0.3	Tlk2	4E-12	0.3
Slc35a2	2E-10	0.3	Bmpr1b	6E-06	0.3
Cdkn2aip	6E-08	0.3	Zfp275	3E-06	0.3
Eif3b	8E-14	0.3	Hdlbp	2E-44	0.3
Tpcn1	2E-07	0.3	Cct2	7E-34	0.3
Avil	1E-02	0.3	5330438D12Rik	4E-08	0.3

Chondrogenic

Control			NICD2		
Gene	*p*	FC	Gene	*p*	FC
Slc30a1	2E-03	0.4	Cd320	1E-10	0.4
Igf2r	4E-23	0.4	2510002D24Rik	9E-06	0.4
Ppp1r12b	6E-08	0.4	Tmem218	2E-05	0.4
Tm2d3	5E-16	0.4	Fbxo33	3E-01	0.4
Vta1	2E-15	0.4	Rhbdd1	9E-03	0.4
Tmed4	9E-14	0.4	Sec11c	2E-08	0.4
Eml6	1E-03	0.4	Atoh8	1E-14	0.4
Kcnt2	4E-03	0.4	Ccdc66	6E-04	0.4
Mfsd4a	2E-03	0.4	Twsg1	2E-07	0.3
Klhl11	7E-04	0.4	Snx25	3E-03	0.3
Mtss2	2E-12	0.4	Cers2	3E-08	0.3
Thnsl1	6E-02	0.4	Insr	2E-07	0.3
Dzip3	2E-10	0.4	Dand5	7E-02	0.3
Sorbs1	4E-08	0.4	Ecpas	4E-17	0.3
Txndc11	9E-05	0.4	Entpd7	1E-03	0.3
Traf3	1E-07	0.4	Aptx	4E-04	0.3
Serf1	3E-25	0.4	Hdhd5	2E-02	0.3
Cry1	2E-02	0.4	Rhobtb2	4E-01	0.3
Cav3	1E-01	0.4	Pithd1	3E-03	0.3
Usp50	2E-05	0.4	Slc4a3	1E-01	0.3
Fam126b	1E-06	0.4	Ranbp17	3E-03	0.3
Pop5	7E-15	0.4	Dnajc15	7E-17	0.3
Sgk1	3E-10	0.4	2210408F21Rik	1E-07	0.3
Pemt	4E-01	0.4	BC031181	6E-22	0.3
Vezt	4E-08	0.4	Dalrd3	1E-03	0.3
Rhobtb3	1E-22	0.4	Gm16740	1E-01	0.3
Tmem170b	6E-02	0.4	Map9	6E-03	0.3
Lnpep	5E-17	0.4	Mipol1	2E-06	0.3
Lrrc1	2E-02	0.4	Mef2b	9E-03	0.3
Ift122	4E-05	0.4	Dtnb	1E-04	0.3
Ifit2	2E-04	0.4	Rnaset2a	2E-04	0.3
Trdmt1	5E-02	0.3	Ndufa4l2	1E-06	0.3
Stt3b	5E-19	0.3	8430429K09Rik	3E-03	0.3
Ccdc28b	1E-06	0.3	Trim13	1E-01	0.3
1500004A13Rik	2E-04	0.3	Sort1	2E-03	0.3
Tfeb	1E-01	0.3	Trib3	1E-01	0.3
Ndufaf5	2E-02	0.3	Skp1a	2E-44	0.3
Snx15	1E-10	0.3	2610301B20Rik	3E-02	0.3
Hpgd	3E-01	0.3	Ttc5	3E-03	0.3
Prxl2c	1E-11	0.3	Ccdc191	2E-03	0.3
Zfyve9	2E-12	0.3	Tmem35b	4E-01	0.3
Gm26797	4E-03	0.3	Gm15614	6E-02	0.3
Gramd1c	7E-02	0.3	Fam120c	3E-05	0.3
Zfp866	1E-03	0.3	Tmx1	7E-12	0.3
Atp1a1	7E-20	0.3	Hgsnat	1E-06	0.3
Slc10a3	4E-01	0.3	Gm43672	7E-02	0.3
Tmed10	6E-76	0.3	Pacsin2	9E-08	0.3
Dnal1	1E-04	0.3	Thumpd3	1E-04	0.3
Nat10	1E-02	0.3	Ptprd	2E-16	0.3
E130317F20Rik	3E-02	0.3	Sppl2b	1E-01	0.3
Gtf2i	1E-24	0.3	Ubqln4	3E-02	0.3
Tgfbr1	1E-07	0.3	Gt(ROSA)26Sor	3E-02	0.3
Ptpn21	3E-05	0.3	Rnf167	1E-03	0.3
Gm43713	3E-04	0.3	Klf8	6E-01	0.3
Tmed9	1E-62	0.3	Rrs1	9E-05	0.3
Zc3h10	9E-03	0.3	Pigg	3E-01	0.3
Zfp975	2E-02	0.3	Bbs7	3E-02	0.3
Twsg1	4E-12	0.3	Ahcyl1	5E-18	0.3
Rfesd	2E-04	0.3	Txlng	6E-06	0.3
Cst3	3E-44	0.3	4930549G23Rik	3E-02	0.3
Slc1a4	8E-06	0.3	Ap1g1	3E-11	0.3
Wasf3	4E-04	0.3	1500015A07Rik	4E-02	0.3
St7l	1E-07	0.3	Ntn4	1E-01	0.3
Mctp2	4E-02	0.3	Zfhx2	1E-01	0.3
Spats2l	3E-05	0.3	Dvl1	2E-01	0.3
Gm11613	3E-03	0.3	Ppp2r3c	1E-03	0.3
Zfp719	2E-04	0.3	Micu3	7E-05	0.3
Ston1	2E-09	0.3	1110032A03Rik	5E-09	0.3
Slc22a5	3E-01	0.3	2700081O15Rik	9E-07	0.3
Fsd1	6E-02	0.3	Hint2	1E-13	0.3
Paip2b	8E-06	0.3	Pemt	2E-01	0.3
Kifc3	8E-03	0.3	Chsy1	1E-07	0.3
Gale	2E-02	0.3	Gm37240	2E-04	0.3

Fibroblast

Control			NICD2		
Gene	*p*	FC	Gene	*p*	FC
Rps6kb1	2E-30	0.3	Tusc1	4E-03	0.3
Zfr2	8E-09	0.3	Tead3	1E-04	0.3
Eva1b	5E-52	0.3	Lysmd3	4E-08	0.3
Cdr2l	5E-06	0.3	Acvr1	6E-14	0.3
Rras	6E-54	0.3	Atraid	3E-49	0.3
Pdpn	6E-51	0.3	Evc2	8E-10	0.3
Celf5	6E-05	0.3	Hif1a	1E-48	0.3
Bin1	3E-39	0.3	Dcun1d3	1E-05	0.3
Gm20721	5E-05	0.3	Comtd1	5E-09	0.3
Gm15446	2E-03	0.2	Atp2b4	2E-05	0.3
Dnajc25	4E-34	0.2	Sqor	4E-12	0.3
Srebf2	1E-25	0.2	Rps28	7E-200	0.3
Ggta1	3E-07	0.2	Lonrf1	2E-03	0.3
Ikzf2	3E-07	0.2	Zdhhc1	2E-06	0.3
Zfp655	1E-12	0.2	Med13l	3E-23	0.3
Prkra	1E-32	0.2	Ap3d1	3E-20	0.3
Snx30	5E-10	0.2	Cyp4f13	1E-03	0.3
C2cd2	1E-04	0.2	2810032G03Rik	1E-03	0.3
Ldlr	4E-24	0.2	Vamp2	4E-12	0.3
Cd44	6E-23	0.2	Cyb561a3	2E-02	0.3
Adamts4	1E-02	0.2	Lhpp	6E-02	0.3
Agl	3E-15	0.2	F420014N23Rik	5E-04	0.3
Hist2h4	7E-03	0.2	Shisa5	2E-25	0.3
Bex3	2E-69	0.2	Dok5	3E-03	0.3
Morc4	4E-15	0.2	Amacr	1E-04	0.3
Rbpms	5E-13	0.2	Cldn12	1E-03	0.3
Rps29	0E+00	0.2	Srebf2	2E-12	0.3
Egfl7	3E-12	0.2	Sh3bp1	4E-03	0.3
Srd5a3	6E-11	0.2	Il1rl1	1E-08	0.3
Ifngr2	9E-29	0.2	Stk40	2E-06	0.3
Snapc4	8E-03	0.2	Etfbkmt	2E-02	0.3
Lpp	9E-78	0.2	Samd9l	4E-12	0.3
Rnf149	3E-08	0.2	Col5a2	9E-60	0.3
Samd9l	2E-20	0.2	Prrx2	5E-37	0.3
Asah2	7E-10	0.2	B830012L14Rik	7E-06	0.3
Sept8	3E-24	0.2	Ebp	3E-14	0.3
Tceal3	7E-11	0.2	Dtnbp1	5E-31	0.3
Tmem173	4E-08	0.2	Bdnf	6E-02	0.3
Ahnak	4E-114	0.2	Traf5	3E-05	0.3
Fam161a	1E-04	0.2	Zbtb4	6E-08	0.3
Zbtb34	7E-04	0.2	Lamp2	1E-61	0.3
Ada	2E-08	0.2	Rassf5	5E-03	0.3
Armcx1	4E-15	0.2	Dpp7	7E-11	0.3
Dbn1	6E-27	0.2	Brdt	2E-02	0.3
Rps24	0E+00	0.2	Synm	2E-04	0.3
Runx1	2E-36	0.2	Tpst2	2E-07	0.3
Kctd10	6E-14	0.2	Rusc1	2E-02	0.3
Stam2	2E-24	0.2	2610002M06Rik	3E-06	0.3
Gopc	1E-19	0.2	Plekhj1	2E-22	0.3
Ezh1	3E-16	0.2	Nradd	1E-12	0.3
Magee1	4E-07	0.2	Errfi1	2E-01	0.3
Ap3d1	4E-29	0.2	H19	2E-33	0.2
5730409E04Rik	5E-12	0.2	Slc4a2	8E-10	0.2
Dok5	4E-05	0.2	Rrbp1	2E-88	0.2
Irak2	1E-06	0.2	Sept5	1E-01	0.2
Gnas	1E-44	0.2	Dcbld2	2E-06	0.2
Fbxo10	1E-06	0.2	Rad9b	2E-02	0.2
Lrrk2	2E-10	0.2	1110012L19Rik	3E-09	0.2
Dpp7	2E-19	0.2	Gcc1	8E-03	0.2
C1qtnf6	3E-16	0.2	Rpl28	7E-209	0.2
Adamts1	3E-48	0.2	Fut11	5E-10	0.2
Ccnyl1	1E-07	0.2	Hspa12b	2E-03	0.2
Usp25	7E-25	0.2	Washc1	3E-06	0.2
Efs	8E-14	0.2	Ppp1r15b	4E-06	0.2
Mospd3	7E-10	0.2	Tnfrsf1a	1E-25	0.2
Ptrh1	8E-08	0.2	Acap2	2E-20	0.2
Gm49359	3E-09	0.2	Actn1	1E-11	0.2
C1ra	8E-40	0.2	Cbr3	1E-17	0.2
Casp9	9E-06	0.2	Tcirg1	1E-03	0.2
Ephb4	5E-05	0.2	Eva1b	1E-25	0.2
Rab29	3E-12	0.2	Pip4k2a	6E-11	0.2
Castor1	2E-08	0.2	Aldh3a2	9E-05	0.2
Sod3	3E-34	0.2	Arhgef28	2E-02	0.2

Fibroblast?

Control			NICD2		
Gene	*p*	FC	Gene	*p*	FC
Odf2	2E-41	0.8	Tnfrsf10b	3E-39	0.8
Phgdh	1E-59	0.8	Mzt1	2E-52	0.8
2900041M22Rik	8E-16	0.8	Cav1	4E-76	0.8
Ugdh	2E-67	0.8	Ror2	1E-51	0.8
Zbtb16	1E-14	0.8	Hnrnpa3	3E-159	0.8
Triobp	1E-47	0.8	Notch3	5E-33	0.8
S100a4	6E-52	0.8	D1Ertd622e	4E-21	0.8
Cers6	4E-33	0.8	Pfdn6	1E-59	0.8
B930036N10Rik	1E-39	0.8	Gxylt1	9E-57	0.8
Ccl2	3E-19	0.8	Tmem237	6E-58	0.8
Gm42745	6E-11	0.8	Ccl2	3E-34	0.8
Ptgs1	4E-27	0.8	Hprt	6E-73	0.8
Tuba4a	2E-32	0.8	Begain	2E-15	0.8
Casp8	3E-42	0.8	Cggbp1	4E-111	0.7
Map3k6	5E-12	0.8	Nrk	2E-51	0.7
Pear1	1E-26	0.8	Idh3a	1E-66	0.7
Srsf3	7E-113	0.8	Csrnp2	2E-16	0.7
Cfap45	3E-13	0.8	Xndc1	4E-50	0.7
Ccdc162	3E-11	0.8	Smyd4	1E-36	0.7
Srxn1	3E-41	0.8	Smarca5	8E-158	0.7
Ilf2	1E-49	0.8	Gfod2	8E-16	0.7
Uba2	5E-57	0.8	Metrn	7E-56	0.7
Nt5dc2	9E-74	0.8	Ildr2	9E-41	0.7
Mettl15	1E-34	0.8	Lrrc20	6E-47	0.7
Dtx4	3E-06	0.8	Cyp2r1	2E-27	0.7
Arhgap22	2E-32	0.8	Dcp2	7E-52	0.7
Serp2	4E-38	0.8	Txnrd1	2E-81	0.7
Ptprj	3E-40	0.8	Ppat	5E-44	0.7
Lmo1	3E-36	0.8	Triobp	5E-48	0.7
Myo19	7E-30	0.8	Stk17b	4E-57	0.7
Apln	2E-24	0.8	Hmbs	6E-50	0.7
Anapc11	2E-93	0.8	Fgf13	1E-64	0.7
Cstf2	2E-33	0.8	Gm11627	3E-15	0.7
Cep250	2E-32	0.8	Thoc3	1E-62	0.7
Rbbp7	7E-104	0.8	Tex9	6E-33	0.7
Begain	1E-11	0.8	Rexo5	5E-39	0.7
Gas7	2E-45	0.8	Wdr54	2E-17	0.7
Nfyb	8E-45	0.8	Rgs10	2E-65	0.7
Pla2g4a	1E-48	0.8	Nubp1	2E-59	0.7
Tsku	2E-03	0.8	Apcdd1	1E-29	0.7
Gm12454	3E-11	0.8	Phf5a	4E-92	0.7
Rhebl1	3E-26	0.8	Hnrnpf	4E-159	0.7
Nup93	3E-43	0.8	Gtf2a2	4E-93	0.7
Olfm2	1E-15	0.8	Tmem47	5E-48	0.7
Snrnp40	2E-50	0.8	Rnf126	1E-43	0.7
Insl6	2E-17	0.8	C2cd3	4E-44	0.7
Gkap1	2E-46	0.8	Il15	5E-26	0.7
Trim47	7E-59	0.8	Zdhhc12	4E-37	0.7
Cand2	9E-28	0.8	Gclc	4E-50	0.7
AV099323	7E-12	0.8	Fbxl8	5E-34	0.7
Serpinb1a	6E-47	0.8	Itgb3	3E-19	0.7
Nelfcd	1E-36	0.8	Cox18	4E-39	0.7
Csrnp2	4E-18	0.8	Ppp1r2	7E-82	0.7
Slc35e1	2E-27	0.8	Fads2	6E-48	0.7
Sox11	8E-20	0.8	Dbndd2	8E-40	0.7
3110045C21Rik	2E-16	0.8	Col8a1	7E-57	0.7
Hmgxb4	5E-45	0.8	Chml	3E-12	0.7
Cep44	7E-35	0.8	Saal1	3E-33	0.7
Gm15706	5E-13	0.8	Ing5	7E-42	0.7
Kpna3	3E-45	0.8	Inip	5E-44	0.7
Ybx3	5E-82	0.8	Anapc5	7E-106	0.7
Gm11457	6E-12	0.8	Nup54	6E-48	0.7
Acta2	2E-16	0.8	Kmt5a	9E-77	0.7
Tamm41	1E-32	0.8	Ccdc61	3E-42	0.7
Mrs2	4E-37	0.8	Eln	3E-52	0.7
Rhno1	2E-38	0.8	Spout1	1E-26	0.7
Foxd1	3E-12	0.8	Steap1	9E-51	0.7
Katnb1	2E-29	0.8	Xrcc6	7E-45	0.7
Msh3	7E-28	0.8	Slc25a25	8E-38	0.7
Ppp1r8	3E-27	0.8	Cers6	5E-44	0.7
Myg1	2E-48	0.7	AI506816	2E-99	0.7
Banf1	4E-89	0.7	Fam216a	9E-57	0.7
Slc16a7	2E-25	0.7	Slc20a1	2E-46	0.7

Undefined

Control			NICD2		
Gene	*p*	FC	Gene	*p*	FC
Pycr2	2E-01	0.1	Cfap36	3E-04	0.1
1700029J07Rik	4E-01	0.1	Cep19	1E+00	0.1
Cebpzos	3E-04	0.1	Nfu1	3E-04	0.1
Cwc22	2E-01	0.1	Acbd4	6E-01	0.1
Zfp612	8E-01	0.1	Gm10561	7E-01	0.1
Ifi30	5E-03	0.1	Gclc	1E-01	0.1
Pygo1	5E-02	0.1	Saysd1	7E-04	0.1
Fam71e1	8E-01	0.1	Rabl3	1E-01	0.1
Btbd2	6E-01	0.1	Bcl6	3E-01	0.1
Mipep	5E-01	0.1	Gm49226	5E-01	0.1
Anxa2	5E-18	0.1	Tk2	2E-01	0.1
Slc35b1	1E-02	0.1	Zfp740	3E-01	0.1
Osbpl9	3E-04	0.1	Klhdc8b	7E-01	0.1
Nbl1	2E-02	0.1	Fam118b	1E+00	0.1
Rasa1	2E-04	0.1	Lig3	9E-01	0.1
Gnb5	4E-01	0.1	Urb1	5E-01	0.1
Gba	4E-01	0.1	Snhg18	6E-14	0.1
Fam20a	2E-01	0.1	Mrpl47	2E-01	0.1
Sept6	5E-01	0.1	Tmem230	1E-02	0.1
Dynlt3	1E-05	0.1	Zfp579	3E-01	0.1
Rsl24d1	4E-01	0.1	Asph	9E-04	0.1
Ostf1	2E-05	0.1	Cd109	3E-06	0.1
Sdf4	3E-14	0.1	Vipas39	8E-01	0.1
Supt4a	1E-05	0.1	Ctsa	7E-09	0.1
Ppp1r15a	1E-02	0.1	Aip	6E-01	0.1
BC003965	5E-01	0.1	Mettl17	6E-01	0.1
Brd3os	4E-01	0.1	Calml4	6E-01	0.1
Zfp672	6E-01	0.1	Gpbp1l1	5E-02	0.1
Sept1	8E-01	0.1	Hfe	6E-01	0.1
Adck1	3E-01	0.1	Nob1	1E-01	0.1
Tpi1	7E-08	0.1	Galt	3E-01	0.1
Gmpr	7E-01	0.1	Supt4a	1E-07	0.1
Gm10762	6E-01	0.1	Inhbb	6E-01	0.1
Pkm	1E-10	0.1	AI837181	3E-01	0.1
Gskip	1E-01	0.1	Calhm5	3E-01	0.1
Ppan	3E-01	0.1	Gm50240	3E-01	0.1
Zfp995	1E-01	0.1	Exoc3	5E-07	0.1
Slu7	2E-01	0.1	Acot8	2E-01	0.1
Ap4m1	6E-01	0.1	BC005624	1E-01	0.1
Ash2l	1E-01	0.1	Ndufs3	2E-04	0.1
2510002D24Rik	2E-02	0.1	Chkb	2E-01	0.1
Fkbp14	2E-02	0.1	Bzw2	2E-03	0.1
Gstm1	4E-04	0.1	Dock11	6E-01	0.1
Rce1	6E-01	0.1	Exosc4	1E-02	0.1
Nmnat2	5E-01	0.1	Ppox	6E-01	0.1
Pqlc2	9E-01	0.1	Garem1	6E-01	0.1
Gin1	6E-01	0.1	Slc39a10	9E-01	0.1
Rela	2E-01	0.1	Afg1l	8E-01	0.1
Polr3h	8E-01	0.1	1700030K09Rik	9E-01	0.1
Sft2d3	9E-01	0.1	Fes	2E-01	0.1
Nif3l1	8E-01	0.1	Rfesd	8E-01	0.1
Cryz	4E-01	0.1	Spred3	8E-01	0.1
Arid5b	8E-08	0.1	Kif13b	5E-02	0.1
Bsdc1	2E-01	0.1	Oaz2	5E-02	0.1
Aldh2	3E-08	0.1	Glrx2	3E-05	0.1
Mrpl2	8E-03	0.1	Aasdh	9E-01	0.1
4833420G17Rik	4E-02	0.1	Zfp646	5E-01	0.1
Lyplal1	6E-01	0.1	Zfp112	2E-01	0.1
4930557K07Rik	8E-01	0.1	Col4a5	9E-01	0.1
Pdcd6	1E-03	0.1	2010320M18Rik	5E-01	0.1
Smpd2	3E-01	0.1	Tax1bp3	7E-02	0.1
Ttyh2	3E-01	0.1	Ctsl	4E-18	0.1
Bckdk	1E-01	0.1	Park7	2E-14	0.1
Trappc6a	1E-02	0.1	Nabp2	5E-02	0.1
Zfp329	4E-01	0.1	Gpr108	1E+00	0.1
Lmf1	5E-01	0.1	Stxbp6	1E-03	0.1
Vps33b	5E-01	0.1	Hint2	3E-04	0.1
Ppie	2E-01	0.1	Plcd3	8E-01	0.1
Pgghg	9E-01	0.1	Gm15283	2E-03	0.1
2900060B14Rik	3E-01	0.1	Trit1	8E-01	0.1
Ttc13	7E-01	0.1	Magi2	2E-07	0.1
Ttll7	2E-01	0.1	Car11	6E-01	0.1
Tmem9	4E-02	0.1	Rbm4b	4E-01	0.1

Articular/Synovial Fibroblast

Control			NICD2		
Gene	*p*	FC	Gene	*p*	FC
Atxn3	4E-02	0.4	Galnt2	1E-03	0.4
5530601H04Rik	3E-01	0.4	Pard3b	9E-07	0.4
Arl6ip5	3E-17	0.4	Clip1	3E-07	0.4
Ccdc28a	8E-01	0.4	Mrtfb	9E-05	0.4
Hps6	8E-01	0.4	Frrs1	1E-01	0.4
Xpnpep1	4E-04	0.4	Adhfe1	3E-01	0.4
Guca1a	1E-01	0.4	Snx9	3E-10	0.4
Ifit3b	4E-03	0.4	Nt5dc3	3E-01	0.4
Gns	4E-10	0.4	Atg4c	5E-01	0.4
Micall1	5E-01	0.4	Ppm1h	1E-02	0.4
Pik3r1	7E-11	0.4	Sos2	6E-02	0.4
Scly	7E-01	0.4	Rab26os	1E+00	0.4
Zfp991	7E-01	0.4	Cd99l2	8E-03	0.4
Ap3m2	4E-01	0.4	1700086O06Rik	1E+00	0.4
Gm14858	1E+00	0.4	Ap5m1	4E-01	0.4
9530026P05Rik	3E-01	0.4	Ccdc85b	6E-08	0.4
Mpv17	4E-06	0.4	Ptcd1	9E-01	0.4
Anxa8	2E-01	0.4	Vps33b	6E-01	0.4
Man2a1	2E-07	0.4	4930484I04Rik	1E+00	0.4
Cetn4	8E-01	0.4	Adamts5	8E-03	0.4
Tmem220	1E+00	0.4	Zfp626	8E-01	0.4
Dalrd3	2E-01	0.4	Cdk19	4E-02	0.4
Fam161a	5E-01	0.4	S1pr2	3E-01	0.4
Zfp14	7E-01	0.4	Ciao1	2E-01	0.4
Gm14966	7E-01	0.4	Cdkl3	5E-01	0.4
Dcp1b	7E-01	0.4	G6pdx	2E-02	0.4
Zer1	2E-01	0.4	Elk1	2E-01	0.4
Samd9l	3E-05	0.4	Dnajc13	9E-05	0.4
Akap13	1E-09	0.4	Pfkfb4	9E-01	0.4
Abl1	3E-05	0.4	Selenon	3E-02	0.4
Mterf2	5E-01	0.4	Wrn	2E-01	0.4
Vim	5E-34	0.4	Gigyf1	2E-01	0.4
Tmem9	5E-04	0.4	Baiap2	2E-03	0.4
Palm	3E-02	0.4	Zbtb11os1	2E-01	0.4
Rhoq	4E-06	0.4	Slc6a8	1E-01	0.4
Anxa4	3E-13	0.4	4921504A21Rik	1E+00	0.4
Trim12a	5E-02	0.4	Diaph1	5E-03	0.4
Trim2	2E-04	0.4	Zfp961	1E+00	0.4
Runx1t1	6E-12	0.4	Dbp	7E-04	0.4
Pcdhga5	1E+00	0.4	Camk2n1	9E-01	0.4
Orc1	7E-01	0.4	Slco2a1	7E-01	0.4
Zfp105	9E-01	0.4	Ly6e	6E-12	0.4
Mfsd11	4E-01	0.4	Cdan1	8E-01	0.4
Fgd4	9E-02	0.4	Mmp19	8E-02	0.4
Tbc1d9	8E-01	0.4	Gpc3	5E-01	0.4
Zfp319	6E-01	0.4	Isoc2b	3E-02	0.4
Lmbr1l	7E-01	0.4	Gpd1l	8E-01	0.4
Mamld1	3E-01	0.4	Ano8	6E-01	0.4
Cpped1	2E-03	0.4	Wwox	5E-07	0.4
Pqlc3	5E-01	0.4	Gm13470	6E-01	0.4
Stat2	4E-03	0.4	Accs	1E+00	0.4
Gm49959	9E-01	0.4	Cd46	7E-01	0.4
Gga2	1E-05	0.3	H6pd	2E-01	0.4
Ptp4a3	7E-02	0.3	Zfp715	7E-01	0.4
Trabd2b	1E-04	0.3	Mertk	5E-02	0.4
Prr16	1E-01	0.3	Slc25a27	8E-01	0.4
Fam53b	2E-01	0.3	Zmym6	1E-01	0.3
Fyco1	3E-03	0.3	Aldh4a1	3E-01	0.3
Rnf13	3E-09	0.3	Islr	4E-07	0.3
Arntl2	8E-01	0.3	Thra	7E-15	0.3
Ctsa	1E-12	0.3	Casp6	9E-02	0.3
Megf8	7E-02	0.3	Ccdc181	9E-01	0.3
Cep19	1E-01	0.3	Fbxo46	8E-01	0.3
Zfp61	1E+00	0.3	1110008P14Rik	1E-13	0.3
Scn2a	4E-01	0.3	Gm50240	7E-01	0.3
Appl2	1E-02	0.3	Gm12258	8E-01	0.3
Gyg	2E-02	0.3	Tceanc	9E-01	0.3
Capzb	2E-15	0.3	Ccdc93	7E-02	0.3
Tob1	1E-06	0.3	Ggh	2E-09	0.3
Dhrs9	4E-05	0.3	Fam161a	7E-01	0.3
Ppp1r15b	1E-01	0.3	Ankrd54	9E-01	0.3
Hipk2	3E-06	0.3	Eef1akmt1	7E-05	0.3
Crebrf	3E-05	0.3	Tirap	9E-01	0.3

Limb Mesenchyme					
Control			NICD2		
Gene	*p*	FC	Gene	*p*	FC
Ublcp1	3E-13	0.3	Gm4258	2E-06	0.3
Slc25a16	3E-09	0.3	Ykt6	1E-11	0.3
Med22	6E-04	0.3	Pde4dip	2E-11	0.3
Slc25a32	3E-08	0.3	Proser1	7E-07	0.3
Bckdk	5E-10	0.3	Bmi1	2E-10	0.3
Tmem189	1E-09	0.3	Sike1	3E-08	0.3
Itga10	8E-12	0.3	B930036N10Rik	1E-07	0.3
Msi1	2E-04	0.3	Gm47283	7E-06	0.3
Cd82	9E-09	0.3	Uggt1	1E-12	0.3
Frmd6	4E-15	0.3	Ifi44	5E-07	0.3
Wdr75	3E-11	0.3	Zbtb26	2E-04	0.3
Ppme1	1E-12	0.3	Pcgf3	8E-10	0.3
Ppp4r3a	5E-11	0.3	Zfp141	3E-05	0.3
Fmnl3	3E-10	0.3	Tm9sf3	2E-29	0.3
Map4k3	2E-12	0.3	Cog6	4E-10	0.3
Ccdc47	8E-17	0.3	Ankfy1	8E-10	0.3
C1qtnf4	3E-07	0.3	Sec63	3E-18	0.3
Smo	6E-15	0.3	Usp50	8E-08	0.3
Tmem167b	8E-10	0.3	Pawr	4E-12	0.3
Jdp2	2E-19	0.3	Strbp	2E-13	0.3
Emp2	3E-12	0.3	Zswim3	3E-03	0.3
Rab11fip3	7E-08	0.3	Nme6	8E-05	0.3
Zscan2	1E-05	0.3	Atg9a	5E-05	0.3
Scmh1	2E-14	0.3	Trim7	1E-03	0.3
Thap2	2E-10	0.3	Adss	5E-13	0.3
Nfu1	1E-21	0.3	Gm15222	9E-05	0.3
Cbx1	2E-27	0.3	Zbtb46	2E-03	0.3
Ap1g1	1E-12	0.3	Pptc7	6E-07	0.3
Gm26936	2E-07	0.3	Akap1	4E-07	0.3
Stk4	2E-09	0.3	Oxsr1	3E-07	0.3
Sbf1	5E-08	0.3	Mybbp1a	9E-16	0.3
Stambpl1	1E-07	0.3	Brd1	2E-15	0.3
Mbnl2	9E-36	0.3	Eif2s2	2E-38	0.3
Efr3a	7E-13	0.3	Schip1	1E-08	0.3
Gnl3l	8E-12	0.3	Gm17501	3E-14	0.3
Itga3	3E-05	0.3	Khdrbs1	4E-21	0.3
Hbs1l	5E-12	0.3	Tmem245	4E-08	0.3
Wdr43	3E-13	0.3	Nop58	6E-17	0.3
Rras2	2E-12	0.3	Got1	3E-07	0.3
Prdm5	3E-12	0.3	Zfp2	3E-04	0.3
Slc35b1	5E-15	0.3	Mme	2E-19	0.3
Med14	2E-10	0.3	Espn	4E-03	0.3
Plcd1	2E-08	0.3	Edem3	6E-06	0.3
Pitpnm2	8E-04	0.3	Slc33a1	1E-07	0.3
Stxbp5	3E-11	0.3	Cep104	7E-07	0.3
Irgq	8E-07	0.3	Ndst1	3E-06	0.3
Macf1	8E-24	0.3	Lemd3	5E-08	0.3
Luzp1	3E-22	0.3	Hspa4l	2E-05	0.3
Mrps28	2E-18	0.3	Prkci	4E-09	0.3
Efhc1	3E-04	0.3	Gm12940	2E-03	0.3
Got1	5E-09	0.3	Fosb	2E-15	0.3
Fbxl19	7E-07	0.3	Zfp146	2E-07	0.3
Calu	3E-38	0.3	Eefsec	9E-08	0.3
Tfb1m	3E-05	0.3	Sec24d	1E-15	0.3
E130317F20Rik	6E-04	0.3	Wdr6	1E-07	0.3
2500002B13Rik	7E-06	0.3	Wdr48	1E-07	0.3
Mfsd4b4	8E-04	0.3	Tsc22d3	3E-08	0.3
Scyl3	5E-08	0.3	Lrp5	5E-06	0.3
Arfgap1	1E-11	0.3	Tmem98	2E-09	0.3
Dhx29	9E-10	0.3	Lix1	1E-03	0.3
2700054A10Rik	3E-04	0.3	Tspyl1	3E-10	0.3
Wwc1	1E-05	0.3	Zfp647	2E-03	0.3
Exoc2	1E-08	0.3	Mphosph10	6E-09	0.3
Zc3hav1l	7E-04	0.3	Plekha7	2E-04	0.3
Ecsit	7E-08	0.3	Camsap1	1E-04	0.3
Brms1	8E-10	0.3	Atad1	9E-12	0.3
Dnajc1	9E-25	0.3	Lats1	3E-07	0.3
Fam114a1	1E-29	0.3	Klc2	3E-05	0.3
Tjap1	2E-09	0.3	Hs6st1	1E-08	0.3
Gnaq	3E-19	0.3	Hmgb3	2E-07	0.3
Piga	2E-02	0.3	Wwc1	3E-04	0.3
Gm20721	1E-08	0.3	Cacna1d	1E-04	0.3
Rnf113a1	3E-06	0.3	Tmem63a	2E-06	0.3

Chondrogenic					
Control			NICD2		
Gene	*p*	FC	Gene	*p*	FC
Hnrnph3	9E-16	0.3	Cyp39a1	4E-01	0.3
Acadsb	2E-19	0.3	Ppp3cc	3E-03	0.3
Entpd7	3E-03	0.3	Slc45a4	3E-02	0.3
F420014N23Rik	5E-04	0.3	Esf1	1E-07	0.3
Tmem238	4E-10	0.3	Arnt2	2E-01	0.3
Cd2ap	2E-20	0.3	Cdk17	4E-06	0.3
Odc1	1E-14	0.3	Wdr44	1E-04	0.3
5430405H02Rik	7E-05	0.3	Mob2	2E-05	0.3
6230400D17Rik	6E-02	0.3	Gm10353	8E-01	0.3
Phf1	3E-05	0.3	Mccc2	5E-03	0.3
Gsap	1E-02	0.3	Fbxl12os	8E-02	0.3
Crip2	7E-28	0.3	Pik3r1	4E-10	0.3
Calhm6	2E-01	0.3	Tfb2m	2E-05	0.3
Gm9929	5E-02	0.3	Taz	4E-03	0.3
Siah2	8E-03	0.3	Tmem60	1E-11	0.3
Fos	6E-20	0.3	Btbd19	1E-01	0.3
Car9	8E-04	0.3	2900052L18Rik	8E-03	0.3
Ppp1r10	3E-16	0.3	Clk2	3E-04	0.3
Taf4	1E-01	0.3	Cux1	4E-11	0.3
Dcun1d4	5E-03	0.3	Card19	8E-09	0.3
Intu	6E-01	0.3	Gtpbp8	4E-06	0.3
Tmco3	1E-08	0.3	Pak1ip1	4E-18	0.3
Sypl	3E-16	0.3	Gprasp2	1E-02	0.3
Tmem60	3E-16	0.3	Ktn1	8E-23	0.3
Fbxl12	4E-05	0.3	5730522E02Rik	7E-01	0.3
Sec62	2E-63	0.3	Sgcd	2E-17	0.3
B3glct	1E-08	0.3	Wdr83	1E-04	0.3
Pank1	1E-03	0.3	Mb21d2	6E-01	0.3
Gm4890	5E-01	0.3	Arhgap32	9E-04	0.3
4933421O10Rik	1E-03	0.3	Med22	1E-01	0.3
Triqk	1E-07	0.3	Gm6712	6E-02	0.3
Prorsd1	2E-15	0.3	Rom1	3E-02	0.3
Gm4673	9E-02	0.3	Uggt1	7E-11	0.3
Agbl3	6E-04	0.3	Kbtbd4	1E-02	0.3
5330438D12Rik	2E-10	0.3	Zfp36l2	5E-14	0.3
Med22	2E-02	0.3	4921504A21Rik	7E-02	0.3
Acot8	6E-02	0.3	Fam20b	6E-04	0.3
Jpx	6E-04	0.3	Rnf139	3E-03	0.3
A930001C03Rik	7E-02	0.3	C1qtnf4	7E-02	0.3
Dnajb9	7E-12	0.3	Efna5	3E-12	0.3
Dusp12	4E-06	0.3	Tmem170b	4E-02	0.3
Btf3l4	8E-08	0.3	Gm15411	2E-01	0.3
Unc5c	3E-13	0.3	Fzd1	1E-02	0.3
Gt(ROSA)26Sor	3E-02	0.3	Slc23a2	9E-03	0.3
Jmjd8	1E-03	0.3	Sh3rf1	9E-07	0.3
Gabarapl2	2E-42	0.3	Copg2	3E-07	0.3
Enpp5	1E-06	0.3	A030001D20Rik	2E-01	0.3
Ccser2	1E-25	0.3	Triqk	3E-05	0.3
Thada	1E-09	0.3	Atg14	2E-03	0.3
Amotl2	6E-15	0.3	Os9	1E-18	0.3
Ets1	9E-06	0.3	Cd2ap	4E-14	0.3
Ankfy1	1E-10	0.3	Arhgap5	1E-19	0.3
Gm26749	1E-02	0.3	AY036118	6E-18	0.3
Ccnd1	5E-30	0.3	Lyrm4	6E-03	0.3
Dph7	3E-02	0.3	Vps53	2E-05	0.3
Lrrc4	1E-10	0.3	Pdk1	1E-03	0.3
Thtpa	5E-02	0.3	Gm15417	2E-08	0.3
Klhdc1	4E-03	0.3	Wdr27	3E-01	0.3
Phka1	4E-06	0.3	Iqsec1	2E-04	0.3
Nprl3	2E-04	0.3	Hoxc6	2E-04	0.3
Nudt6	2E-02	0.3	Gm27003	6E-01	0.3
Skp1a	4E-49	0.3	Ttc38	8E-02	0.3
Mfge8	7E-27	0.3	Zfp667	5E-02	0.3
2900093K20Rik	2E-01	0.3	Thada	5E-06	0.3
Kctd16	6E-04	0.3	Akap9	2E-13	0.3
Mtmr3	3E-16	0.3	Slc33a1	2E-05	0.3
Bcat1	7E-03	0.3	Acadsb	1E-12	0.3
Lrrc51	8E-05	0.3	Gnl3l	2E-09	0.3
Sec16b	2E-05	0.3	Gm14858	4E-01	0.3
Ajuba	6E-02	0.3	Ldlrad4	2E-10	0.3
Btbd19	2E-01	0.3	Phyhd1	5E-08	0.3
2610306M01Rik	6E-03	0.3	Cirbp	1E-06	0.3
Snhg18	2E-41	0.3	Gm44899	2E-01	0.3

Fibroblast					
Control			NICD2		
Gene	*p*	FC	Gene	*p*	FC
Plekhj1	3E-37	0.2	Tet1	9E-05	0.2
Pvr	2E-02	0.2	Ccl27a	4E-04	0.2
Litaf	7E-25	0.2	Snap29	1E-05	0.2
Trpc1	2E-04	0.2	Ccdc9b	9E-03	0.2
Rps19	0E+00	0.2	Tmco6	1E-01	0.2
Vps8	2E-13	0.2	Prickle3	2E-02	0.2
Rpl27a	0E+00	0.2	Eif3e	2E-55	0.2
Tceanc	3E-02	0.2	Arhgap44	1E-03	0.2
Fah	4E-06	0.2	Sc5d	6E-10	0.2
Rpl35	3E-259	0.2	Parp9	6E-12	0.2
H2-T22	1E-23	0.2	Tmem64	4E-03	0.2
Socs1	2E-08	0.2	Zfp267	2E-03	0.2
Plekha2	1E-22	0.2	Rps29	1E-224	0.2
Slc9a9	1E-30	0.2	Cap1	8E-15	0.2
Svep1	2E-87	0.2	Ephb4	4E-04	0.2
Zfas1	8E-36	0.2	Dlg4	4E-08	0.2
Tbck	4E-19	0.2	Rbms2	1E-23	0.2
Mfsd10	2E-14	0.2	Sipa1l2	6E-01	0.2
Rbms2	3E-41	0.2	Usb1	5E-04	0.2
Anxa6	6E-35	0.2	Ankrd39	3E-03	0.2
Evc2	9E-13	0.2	Snapc4	8E-02	0.2
Cpne2	4E-24	0.2	Slc2a13	1E-04	0.2
Nckap5l	7E-07	0.2	Rpl27a	5E-189	0.2
Rassf5	2E-04	0.2	St3gal2	9E-13	0.2
Tmem219	4E-18	0.2	Nol3	7E-06	0.2
Adgrl1	1E-13	0.2	Dhx33	4E-03	0.2
2310001H17Rik	2E-04	0.2	Cmc4	6E-05	0.2
Lamp2	2E-104	0.2	Serf2	4E-128	0.2
Ift27	7E-32	0.2	Tmem14a	1E-05	0.2
Cyp4f17	9E-07	0.2	Col5a3	2E-27	0.2
Sipa1l2	1E-01	0.2	Tpm1	7E-27	0.2
Aldh3a2	1E-09	0.2	P2rx4	7E-10	0.2
Adcy2	4E-03	0.2	Tmbim6	4E-34	0.2
Gm16835	5E-03	0.2	Fbxo10	3E-04	0.2
Cyp4v3	3E-08	0.2	Clint1	1E-40	0.2
Rpl36	4E-307	0.2	Cask	7E-29	0.2
Azin2	5E-15	0.2	Pxk	5E-08	0.2
Paox	2E-05	0.2	Tmem131	1E-11	0.2
Cxcl1	2E-17	0.2	Pgpep1	4E-07	0.2
Bhlhe40	4E-13	0.2	Angptl4	9E-14	0.2
Fut11	3E-16	0.2	Slc30a1	2E-02	0.2
Gmppa	3E-18	0.2	Carm1	1E-08	0.2
Zxdc	1E-04	0.2	Supt3	7E-14	0.2
Snhg6	1E-23	0.2	Auts2	9E-11	0.2
Tbl1xr1	2E-37	0.2	Iqsec2	5E-07	0.2
Wdr81	2E-04	0.2	Cdc16	9E-23	0.2
Tbl2	7E-09	0.2	Gramd3	8E-06	0.2
Stim1	4E-20	0.2	Adgrl1	1E-08	0.2
Sh3pxd2a	9E-41	0.2	Dusp23	8E-08	0.2
Rpl13	0E+00	0.2	Ptrh1	6E-04	0.2
Dlg4	1E-12	0.2	Gopc	4E-10	0.2
Prickle2	1E-09	0.2	Col1a2	6E-79	0.2
Lrrc42	3E-24	0.2	Pusl1	2E-09	0.2
Ppif	4E-03	0.2	Lmbr1	7E-04	0.2
Atoh8	5E-05	0.2	Lrfn3	2E-02	0.2
Acaa2	1E-37	0.2	Agtrap	2E-07	0.2
Scd2	9E-52	0.2	Hist2h4	2E-02	0.2
Ncor2	6E-22	0.2	Rps24	4E-162	0.2
Lbh	1E-25	0.2	Mirg	2E-03	0.2
Gm10516	1E-03	0.2	Parp12	3E-06	0.2
Csrp2	3E-24	0.2	Trim2	1E-10	0.2
Mamstr	2E-02	0.2	Glipr2	4E-12	0.2
Rps10	0E+00	0.2	Gpx7	9E-26	0.2
Gpx7	6E-46	0.2	Pofut2	2E-18	0.2
Fpgs	1E-06	0.2	Aprt	9E-50	0.2
Shprh	9E-15	0.2	Plcxd2	1E-03	0.2
Atf5	3E-46	0.2	C030014I23Rik	8E-05	0.2
Lrrc75a	2E-04	0.2	Rxra	4E-05	0.2
Ggcx	7E-14	0.2	Nenf	1E-55	0.2
Bloc1s1	2E-20	0.2	Ppp1r2	6E-16	0.2
Fbxo17	6E-12	0.2	B4galnt1	4E-02	0.2
Clint1	5E-71	0.2	Klhl29	4E-21	0.2
Serf2	6E-234	0.2	Gng7	9E-03	0.2

Fibroblast?					
Control			NICD2		
Gene	*p*	FC	Gene	*p*	FC
Pop4	5E-40	0.7	Raph1	3E-48	0.7
Bcl2l12	9E-28	0.7	Sgf29	7E-50	0.7
Cdc27	7E-41	0.7	Il20rb	5E-21	0.7
Frmd8	5E-35	0.7	Prkcg	7E-20	0.7
Chl1	3E-38	0.7	Clic1	1E-156	0.7
Cbfb	5E-44	0.7	Stn1	1E-42	0.7
Gucy1a2	3E-26	0.7	Cep250	3E-46	0.7
Ggta1	1E-29	0.7	Msh3	3E-42	0.7
Sec22c	3E-10	0.7	Acsl5	5E-40	0.7
Pdzd2	2E-18	0.7	Slc35e1	1E-40	0.7
2410018L13Rik	2E-19	0.7	Atp2b4	3E-47	0.7
Pcolce2	3E-41	0.7	Heg1	2E-32	0.7
Toe1	4E-35	0.7	Emd	4E-74	0.7
Ankrd54	7E-36	0.7	Alcam	5E-43	0.7
Gm47283	2E-30	0.7	B3gnt2	1E-52	0.7
Cwf19l1	2E-23	0.7	F2r	2E-39	0.7
Eftud2	6E-47	0.7	Rrp1b	3E-43	0.7
Irx1	4E-15	0.7	Armcx4	7E-35	0.7
Rdm1	3E-46	0.7	Ddx20	6E-31	0.7
Pea15a	1E-49	0.7	Trim7	5E-25	0.7
Odc1	1E-41	0.7	Cubn	1E-19	0.7
Xndc1	2E-37	0.7	Phf13	3E-45	0.7
Usp13	5E-24	0.7	Tfdp2	3E-67	0.7
Impa2	5E-36	0.7	Ier5	2E-45	0.7
Lin52	2E-33	0.7	Nudcd1	1E-45	0.7
Poc5	2E-27	0.7	Kpna3	1E-62	0.7
Cep83os	5E-32	0.7	H2-Q6	6E-16	0.7
Dnase1l1	1E-32	0.7	Srbd1	3E-52	0.7
Wdr54	1E-13	0.7	Pot1a	2E-37	0.7
Adgrd1	2E-16	0.7	Usp29	1E-16	0.7
Tnfrsf12a	3E-40	0.7	Mid1ip1	1E-30	0.7
Slc16a10	1E-25	0.7	Gm3604	1E-14	0.7
Tent4a	1E-35	0.7	Cisd1	6E-74	0.7
Mthfd1	2E-37	0.7	Wdr78	2E-20	0.7
Eed	1E-48	0.7	Prelp	3E-57	0.7
Slf2	3E-40	0.7	Efhd2	8E-50	0.7
Specc1	2E-45	0.7	Plb1	3E-14	0.7
Arpp19	2E-109	0.7	Ccdc25	7E-45	0.7
Phf6	3E-43	0.7	Txn1	6E-193	0.7
Zwint	2E-46	0.7	D130020L05Rik	1E-13	0.7
Ankrd55	3E-33	0.7	Pbx3	1E-76	0.7
Gstm6	1E-13	0.7	Mdm2	2E-33	0.7
Slc24a5	3E-36	0.7	Helb	8E-42	0.7
Spice1	8E-35	0.7	Ptpre	1E-27	0.7
Gm13470	6E-21	0.7	Nop58	2E-79	0.7
Tmem237	2E-45	0.7	Lnpk	3E-60	0.7
Zfp748	1E-07	0.7	Srsf4	1E-61	0.7
Pgm2l1	6E-25	0.7	Pih1d1	2E-54	0.7
AI506816	2E-66	0.7	Alad	6E-47	0.7
Ehd4	3E-41	0.7	Polk	6E-42	0.7
Parp2	1E-44	0.7	Cep78	8E-45	0.7
Rpgrip1	2E-34	0.7	Ero1lb	2E-26	0.7
Nhsl1	2E-29	0.7	Bicd2	2E-42	0.7
A430073D23Rik	8E-24	0.7	Pnn	4E-89	0.7
Stx2	2E-27	0.7	Trpm6	4E-21	0.7
Prps1	1E-34	0.7	Snrpf	4E-147	0.7
Ppat	2E-29	0.7	Prelid3b	3E-52	0.7
Pih1d1	1E-47	0.7	Ankrd26	3E-41	0.7
Steap1	4E-38	0.7	Tpm3	5E-135	0.7
Nubp1	2E-45	0.7	Fxyd5	3E-51	0.7
Gm1673	3E-60	0.7	Gm4890	1E-22	0.7
Tpm2	4E-70	0.7	Abraxas1	7E-39	0.7
Rbbp4	7E-92	0.7	E030030I06Rik	3E-23	0.7
Phf13	1E-35	0.7	Eed	2E-58	0.7
Hmbs	3E-31	0.7	Banf1	6E-110	0.7
Slc20a1	2E-28	0.7	Gm13986	3E-28	0.7
Msn	2E-70	0.7	Ppp2r2d	5E-51	0.7
Gtf2a2	5E-82	0.7	Gclm	4E-60	0.7
Rarres1	5E-10	0.7	Brms1l	1E-46	0.7
Spag4	5E-13	0.7	Dot1l	5E-43	0.7
Slc9a3r1	2E-26	0.7	Sumo2	4E-235	0.7
Cd34	3E-38	0.7	Zfp114	2E-17	0.7
Cnih4	2E-85	0.7	Pelp1	2E-48	0.7

Undefined					
Control			NICD2		
Gene	*p*	FC	Gene	*p*	FC
Tpd52	1E-01	0.1	Cryz	8E-01	0.1
Itga2b	5E-01	0.1	Myo1b	8E-03	0.1
Zfand6	7E-05	0.1	Surf2	3E-01	0.1
Naa80	8E-01	0.1	2900060B14Rik	1E-01	0.1
Bcl3	7E-01	0.1	Zfyve1	2E-01	0.1
Tspyl1	2E-01	0.1	4933434E20Rik	2E-02	0.1
Borcs7	7E-04	0.1	4930581F22Rik	6E-01	0.1
Cep19	8E-01	0.1	Churc1	6E-04	0.1
Cog8	3E-01	0.1	Oxld1	9E-01	0.1
Ap5m1	7E-01	0.1	Nfkbiz	1E-01	0.1
BC031181	2E-06	0.1	Qsox2	3E-02	0.1
Nabp2	8E-03	0.1	Lins1	8E-01	0.1
Med27	2E-01	0.1	Adss	1E-02	0.1
Zmat5	8E-03	0.1	Slc25a53	6E-01	0.1
Msi2	4E-20	0.1	Tada2b	1E-02	0.1
3110040N11Rik	2E-01	0.1	Gpatch4	9E-01	0.1
Rtp4	5E-01	0.1	Dpp7	6E-02	0.1
Shank1	4E-01	0.1	Pbxip1	2E-02	0.1
Sh3rf1	6E-01	0.1	Snhg4	2E-01	0.1
Sp4	5E-01	0.1	Sumf2	9E-02	0.1
Kat5	8E-01	0.1	Psmd10	4E-01	0.1
Wdr19	1E+00	0.1	Crbn	6E-01	0.1
Rpp40	9E-01	0.1	2810002D19Rik	2E-01	0.1
Thnsl2	8E-01	0.1	Ttll7	4E-01	0.1
Cipc	8E-01	0.1	Sfxn2	2E-01	0.1
Polr2c	2E-04	0.1	4930557K07Rik	7E-01	0.1
Abhd17c	6E-01	0.1	Emc1	4E-01	0.1
Zfp729a	7E-01	0.1	Urgcp	8E-01	0.1
Wdr74	2E-01	0.1	Ethe1	3E-01	0.1
Gng11	7E-02	0.1	Gale	7E-01	0.1
Nkapd1	8E-01	0.1	Mxd4	2E-09	0.1
Gtpbp4	3E-03	0.1	B3gat3	1E-01	0.1
Smn1	8E-02	0.1	Ift22	1E+00	0.1
Rbm15b	8E-01	0.1	A530072M11Rik	9E-01	0.1
Bivm	1E+00	0.1	Hmox2	1E-01	0.1
Thumpd1	2E-01	0.1	Rnf7	5E-06	0.1
Abtb1	8E-01	0.1	Ptch1	7E-01	0.1
Asph	4E-03	0.1	Slc29a1	8E-01	0.1
Wdpcp	2E-01	0.1	Snrnp27	5E-04	0.1
Abhd12	2E-03	0.1	Rgl2	1E+00	0.1
Cdkn2b	7E-05	0.1	Sppl2b	6E-01	0.1
Ubxn2b	7E-01	0.1	Rpgr	4E-01	0.1
E130102H24Rik	2E-01	0.1	Rnh1	7E-04	0.1
Polm	8E-01	0.1	Sertad1	8E-01	0.1
Ube2e1	2E-06	0.1	Smim3	8E-01	0.1
Ecm1	2E-08	0.1	Mapk3	2E-02	0.1
Harbi1	5E-01	0.1	A630072M18Rik	8E-01	0.1
Trim47	5E-02	0.1	Tmem35b	8E-01	0.1
Rnf217	2E-01	0.1	Pcdhb17	8E-01	0.1
Park7	3E-18	0.1	Gm17251	7E-01	0.1
B230118H07Rik	1E-03	0.1	Mmgt2	3E-01	0.1
Gatd3a	4E-01	0.1	Arntl2	1E-01	0.1
6530409C15Rik	4E-01	0.1	Mrpl43	3E-03	0.1
Pam	2E-44	0.1	Synm	9E-01	0.1
Hoxc10	5E-02	0.1	1810030O07Rik	2E-01	0.1
Ufc1	2E-09	0.1	Nt5dc1	7E-01	0.1
Zkscan14	9E-01	0.1	Gm9993	6E-01	0.1
Timm10	3E-01	0.1	Clcf1	3E-01	0.1
Lactb2	3E-01	0.1	Faim	4E-01	0.1
Ifi44	2E-01	0.1	Abtb1	9E-01	0.1
Ccdc84	5E-01	0.1	Extl2	1E+00	0.1
Ldah	1E-03	0.1	Eml5	6E-01	0.1
Trmt112	1E-06	0.1	Fjx1	3E-01	0.1
Cables1	5E-01	0.1	Qrsl1	3E-01	0.1
Pex11b	7E-01	0.1	Psmd5	2E-01	0.1
Dclre1c	1E+00	0.1	Prmt1	2E-04	0.1
Dhx34	9E-01	0.1	Arl10	7E-01	0.1
Kdm4a	4E-01	0.1	Eef1aknmt	5E-02	0.1
Grem1	2E-01	0.1	Mknk1	9E-01	0.1
Cfap74	8E-01	0.1	Plpp6	5E-01	0.1
Gm765	8E-01	0.1	Mettl27	3E-01	0.1
Tmem45a	8E-03	0.1	Arhgef19	6E-01	0.1
Qpctl	8E-01	0.1	Pnkd	3E-02	0.1

Articular/Synovial Fibroblast					
Control			NICD2		
Gene	*p*	FC	Gene	*p*	FC
4933434E20Rik	6E-05	0.3	Gm12353	5E-01	0.3
Mgll	1E-04	0.3	Lekr1	6E-01	0.3
Golph3l	4E-01	0.3	Dsel	3E-02	0.3
Rab9	8E-11	0.3	Tcf12	2E-09	0.3
Spata6	4E-05	0.3	Mme	1E-14	0.3
1110051M20Rik	1E-05	0.3	Gm26797	6E-01	0.3
3300002I08Rik	1E-01	0.3	Prcp	8E-02	0.3
Ccnyl1	7E-01	0.3	Rfx5	8E-01	0.3
Zfp729b	7E-01	0.3	Ino80dos	4E-01	0.3
Tspo	4E-14	0.3	Senp8	7E-01	0.3
Pepd	8E-03	0.3	Fam214a	5E-02	0.3
Cerk	8E-01	0.3	Cep85l	5E-01	0.3
Aff1	3E-05	0.3	Lcmt1	1E-02	0.3
Gm44148	7E-01	0.3	Vcan	6E-04	0.3
Comt	1E-11	0.3	Cfap69	1E+00	0.3
Mme	5E-20	0.3	Med20	8E-01	0.3
Trip6	6E-04	0.3	Zfp36	4E-02	0.3
Bicd1	8E-01	0.3	Crem	1E-01	0.3
Arhgef12	3E-08	0.3	Ighmbp2	9E-01	0.3
Trim16	2E-01	0.3	Il17rd	9E-01	0.3
Rreb1	3E-04	0.3	Ip6k1	9E-03	0.3
Gprasp2	1E+00	0.3	D6Wsu163e	6E-01	0.3
BC052040	4E-01	0.3	Gusb	2E-04	0.3
Prkce	7E-03	0.3	Ndn	2E-05	0.3
Mblac2	5E-01	0.3	Asl	6E-01	0.3
Tom1	3E-01	0.3	Fam53c	5E-01	0.3
Usp53	5E-01	0.3	Lsp1	9E-09	0.3
Abcd4	3E-02	0.3	Slc25a30	6E-01	0.3
Mfsd1	5E-05	0.3	Ncald	4E-01	0.3
Casp7	6E-01	0.3	4732440D04Rik	1E+00	0.3
Lama4	3E-08	0.3	Bcat2	2E-01	0.3
R74862	1E+00	0.3	C1galt1	2E-04	0.3
Cyp4f16	5E-01	0.3	H2-DMa	8E-01	0.3
Mn1	5E-01	0.3	Pnp	2E-05	0.3
Idh2	8E-09	0.3	Nme4	4E-04	0.3
Sos2	5E-01	0.3	Sort1	2E-01	0.3
Sirt2	4E-08	0.3	Homer3	2E-02	0.3
Herc4	8E-04	0.3	Clcn7	8E-01	0.3
Ccng1	1E-09	0.3	Eef2k	4E-02	0.3
Dnmbp	2E-01	0.3	Macrod1	8E-04	0.3
Eif3e	7E-25	0.3	Eng	6E-02	0.3
Plin2	2E-10	0.3	Gm28875	9E-01	0.3
Sgce	4E-06	0.3	Tet3	7E-02	0.3
Apln	6E-01	0.3	Tns1	1E-03	0.3
9330179D12Rik	8E-01	0.3	Lynx1	4E-01	0.3
6330403K07Rik	3E-02	0.3	Dalrd3	9E-01	0.3
Cdon	2E-10	0.3	Col4a5	1E-02	0.3
Hoxc5	4E-01	0.3	Scrn3	5E-01	0.3
Eda2r	1E-01	0.3	Ssbp4	5E-04	0.3
Rbl2	1E-02	0.3	Prkar1b	5E-01	0.3
Rnf217	1E-02	0.3	Prickle1	4E-06	0.3
Gcnt2	2E-01	0.3	Wdr34	4E-01	0.3
Zfp182	1E-02	0.3	Tk2	6E-01	0.3
Ino80b	3E-01	0.3	Catsper2	5E-01	0.3
Ocel1	5E-01	0.3	Palm	3E-01	0.3
9230114K14Rik	2E-01	0.3	Aamdc	6E-07	0.3
Mthfs	1E-01	0.3	Dera	1E-01	0.3
Pyroxd2	6E-01	0.3	Cnrip1	1E-01	0.3
H2-DMa	5E-02	0.3	Xpnpep1	8E-03	0.3
Srfbp1	4E-03	0.3	Tatdn3	9E-01	0.3
Wdr7	3E-02	0.3	Fkbpl	6E-01	0.3
Dnaaf4	1E+00	0.3	Cln3	4E-01	0.3
Rtp4	7E-02	0.3	Mcub	7E-02	0.3
Ppm1m	2E-03	0.3	Rnf13	1E-05	0.3
Rere	8E-12	0.3	Zfp286	6E-01	0.3
Tns1	1E-04	0.3	Arntl2	7E-01	0.3
Anxa1	7E-19	0.3	Pik3r4	5E-01	0.3
Arfgef2	5E-04	0.3	Rere	2E-09	0.3
Gpc3	1E-01	0.3	Irf1	2E-01	0.3
Ilvbl	2E-01	0.3	Uty	4E-03	0.3
Slc4a3	6E-01	0.3	Zfp932	9E-01	0.3
Patj	8E-01	0.3	D830024N08Rik	7E-01	0.3
Diaph1	2E-03	0.3	Zfp563	6E-01	0.3

Limb Mesenchyme					
Control			NICD2		
Gene	p	FC	Gene	p	FC
Smtn	3E-11	0.3	Idua	5E-05	0.3
Thbs2	4E-09	0.3	Ppp1r15a	4E-09	0.3
Klc3	1E-04	0.3	Nxn	1E-12	0.3
Esr1	2E-06	0.3	Mertk	3E-09	0.3
Uba5	2E-17	0.3	Surf6	2E-06	0.3
Magi1	2E-12	0.3	Mgat4a	3E-02	0.3
a	1E-05	0.3	Exoc2	2E-08	0.3
Nt5dc3	4E-06	0.3	Smarcad1	2E-11	0.3
Oma1	3E-07	0.3	Khdc4	1E-07	0.3
Xpo5	1E-10	0.3	Rhot1	2E-07	0.3
Golph3	2E-12	0.3	Trim24	8E-11	0.3
Gm26542	1E-05	0.3	Dpp8	4E-10	0.3
Gm15738	5E-06	0.3	Leprotl1	1E-16	0.3
Arhgef17	2E-10	0.3	Plod2	2E-20	0.3
Nxn	4E-13	0.3	6030443J06Rik	6E-05	0.3
Stk40	1E-08	0.3	Styx	1E-04	0.3
Ikbip	1E-22	0.3	Atg16l2	8E-06	0.3
Dclk2	5E-05	0.3	Gm47167	1E-04	0.3
Lrrk1	4E-13	0.3	Pomgnt1	1E-08	0.3
Mob1b	6E-09	0.3	Xrcc4	5E-08	0.3
Gmppb	4E-11	0.3	Btg2	4E-11	0.3
D1Ertd622e	5E-04	0.3	Trmt11	1E-04	0.3
Sec61b	1E-48	0.3	Dbn1	9E-11	0.3
Ttc26	1E-05	0.3	Pgs1	7E-06	0.3
Xrcc5	7E-14	0.3	Zfp469	5E-04	0.3
Cmpk1	4E-24	0.3	Harbi1	2E-05	0.3
Ston1	2E-11	0.3	Tmem189	2E-07	0.3
Ring1	2E-08	0.3	Dennd1b	1E-07	0.3
Gm19705	6E-03	0.3	Nol8	8E-06	0.3
Tmem218	4E-09	0.3	Aven	7E-08	0.3
Tapt1	2E-07	0.3	Map2k3os	3E-03	0.3
Adat1	2E-05	0.3	Sesn2	9E-10	0.3
Sphk2	9E-07	0.3	Nudt6	2E-03	0.3
Prkcz	9E-05	0.3	Trub1	2E-06	0.3
Intu	8E-03	0.3	Zfp568	1E-06	0.3
Reck	1E-19	0.3	Zscan2	1E-03	0.3
Cbwd1	7E-10	0.3	Mllt3	1E-06	0.3
Papolg	4E-07	0.3	Elac2	2E-05	0.3
Smarcc1	1E-21	0.3	Zkscan5	2E-02	0.3
1700113A16Rik	2E-05	0.3	Phip	5E-10	0.3
Chadl	5E-04	0.3	Fiz1	6E-07	0.3
Pgm3	3E-09	0.3	Clasp2	2E-06	0.3
Armc10	2E-05	0.3	Myd88	4E-05	0.3
Pno1	1E-11	0.3	Jdp2	1E-12	0.3
Ap3m1	2E-09	0.3	Grk4	1E-06	0.3
Cdc25a	9E-09	0.3	Ubxn7	1E-07	0.3
Gm30054	4E-07	0.3	Fam117b	1E-06	0.3
Shroom1	3E-03	0.3	Ptar1	1E-06	0.3
Alg2	3E-06	0.3	Zfp646	2E-09	0.3
Hacd3	7E-08	0.3	Atoh8	6E-04	0.3
Hilpda	2E-08	0.3	Igf1r	2E-15	0.3
Btf3l4	2E-08	0.3	Nckap5l	2E-03	0.3
Gask1b	4E-13	0.3	Sfxn1	2E-11	0.3
Dhx38	3E-07	0.3	Usp49	2E-04	0.3
Slc7a2	8E-13	0.3	Nub1	1E-13	0.3
Pogk	5E-07	0.3	Ulk1	9E-07	0.3
Znhit6	5E-11	0.3	Bnip2	4E-15	0.3
Copg2	2E-10	0.3	Irf2bp2	3E-10	0.3
Ormdl3	3E-13	0.3	2410002F23Rik	2E-08	0.3
Gm15345	6E-04	0.3	Mindy3	2E-06	0.3
Ptdss1	2E-07	0.3	Wasf3	2E-05	0.3
Ptar1	4E-08	0.3	Clip3	5E-07	0.3
Rnf41	1E-07	0.3	Nfxl1	5E-08	0.3
Traf3	2E-08	0.3	Atg16l1	2E-05	0.3
4933421O10Rik	7E-07	0.3	Zfp131	8E-09	0.3
Tnrc18	2E-20	0.3	Exo5	2E-05	0.3
Gpat4	2E-10	0.3	Set	1E-37	0.3
Nudt8	5E-09	0.3	Slc35b1	2E-10	0.3
Kctd5	6E-08	0.3	Snx11	4E-03	0.3
Cstf3	1E-10	0.3	Zfp566	5E-07	0.3
L3mbtl3	6E-08	0.3	Zfp760	3E-04	0.3
Eif2b2	5E-09	0.3	Otud4	1E-08	0.3
2310022B05Rik	2E-12	0.3	Tiparp	7E-11	0.3

Chondrogenic					
Control			NICD2		
Gene	p	FC	Gene	p	FC
2510002D24Rik	1E-04	0.3	Cenpp	3E-21	0.3
Znhit2	2E-05	0.3	Paip2b	2E-03	0.3
Vps53	4E-08	0.3	Fundc1	3E-07	0.3
Clasp2	2E-07	0.3	Dele1	8E-04	0.3
Btbd6	2E-01	0.3	Prss36	6E-02	0.3
Spire1	1E-06	0.3	Polr3e	2E-02	0.3
Zbtb14	2E-02	0.3	Clpb	1E-04	0.3
Bicral	3E-10	0.3	Prkab1	1E-02	0.3
Agpat4	5E-09	0.3	Zfp112	2E-01	0.3
Clec16a	3E-03	0.3	Ermp1	2E-02	0.3
1700029J07Rik	3E-02	0.3	Vgll4	1E-11	0.3
Mcu	7E-11	0.3	Dnajc3	1E-29	0.3
1600002K03Rik	1E-08	0.3	Med31	8E-08	0.3
Nr4a1	8E-02	0.3	Polb	2E-08	0.3
Ptar1	2E-03	0.3	Hps3	6E-02	0.3
4931414P19Rik	2E-02	0.3	Fzd7	7E-02	0.3
Endov	2E-02	0.3	Rec2	6E-06	0.3
Pigk	4E-16	0.3	Galnt7	3E-02	0.3
Srgap3	1E-19	0.3	Atmin	2E-01	0.3
Cfap97	3E-09	0.3	Shank1	4E-01	0.3
Btg3	3E-17	0.3	Tfb1m	5E-02	0.3
Exog	5E-02	0.3	Gm9856	1E-02	0.3
Lats2	2E-10	0.3	P4htm	3E-01	0.3
Synrg	2E-06	0.3	Fam229b	8E-03	0.3
Smpd2	4E-03	0.3	Tmed4	1E-06	0.3
Uggt2	8E-10	0.3	Jmy	5E-06	0.3
Eml2	1E-04	0.3	2810403D21Rik	7E-05	0.3
Slc25a53	3E-02	0.3	Stk4	5E-04	0.3
Clu	8E-07	0.3	Tspan12	5E-01	0.3
Fzd7	2E-02	0.3	Car11	4E-02	0.3
Osgepl1	8E-03	0.3	Ptpn2	1E-05	0.3
Dennd6a	8E-06	0.3	Zbtb9	5E-01	0.3
Sox4	6E-17	0.3	Ndrg1	1E-05	0.3
Pawr	3E-12	0.3	Ormdl3	3E-10	0.3
Slc22a21	2E-01	0.3	Tet2	5E-04	0.3
Cetn2	4E-14	0.3	Tnrc18	3E-15	0.3
Mlh3	1E-06	0.3	Lrrc1	1E-01	0.3
Zfp775	9E-02	0.3	Slc25a13	1E-01	0.3
Pacs2	2E-03	0.3	Cst3	3E-27	0.3
Cirbp	2E-11	0.3	Wdsub1	3E-02	0.3
Chchd10	1E-07	0.3	Zfp362	3E-02	0.3
Ccser1	8E-07	0.3	Zscan22	2E-01	0.3
Sparc	2E-55	0.3	Msrb2	4E-02	0.3
Mxd1	1E-02	0.3	Ppargc1a	2E-02	0.3
Hint2	7E-16	0.3	Itpr3	3E-03	0.3
Mageh1	2E-08	0.3	Tmed9	1E-40	0.3
Dzip1l	9E-03	0.3	Snx13	1E-05	0.3
Sh3d19	1E-13	0.3	Elmo1	4E-06	0.3
Ormdl3	2E-15	0.3	Phldb2	1E-12	0.3
Ganab	2E-10	0.3	Abhd8	1E-06	0.3
Arhgap5	1E-25	0.3	Gm45669	3E-02	0.3
Pdk2	7E-02	0.3	Mapkap1	2E-20	0.3
Dyrk1a	2E-09	0.3	Capn10	2E-02	0.3
Dnah7b	8E-03	0.3	Uxs1	2E-06	0.3
Tusc2	2E-05	0.3	Nol8	5E-04	0.3
Fkbp9	2E-26	0.3	Ints4	1E-02	0.3
Egfros	3E-02	0.3	Tyw3	4E-01	0.3
Crebl2	1E-02	0.3	Rhobtb3	1E-11	0.3
Tmx1	1E-11	0.3	Stk35	1E-01	0.3
2310010J17Rik	7E-06	0.3	Esco1	2E-03	0.3
Smn1	2E-08	0.3	Ctsf	5E-08	0.3
Naaladl2	9E-22	0.3	6030443J06Rik	5E-03	0.3
mt-Nd6	3E-14	0.3	Scara3	3E-05	0.3
Bsg	3E-48	0.3	Tmem39b	4E-02	0.3
Wdyhv1	9E-02	0.3	Clk1	1E-07	0.3
Bola1	1E-18	0.3	Atp1a1	9E-14	0.3
Slc39a13	1E-12	0.3	Tsacc	1E-02	0.3
Scx	2E-10	0.3	Nprl3	8E-03	0.3
Zfp369	2E-04	0.3	Aasdh	1E-01	0.3
Pigq	1E-02	0.3	Atg3	2E-13	0.3
Invs	1E-08	0.3	1190005I06Rik	2E-02	0.3
Ss18l2	7E-07	0.3	Slc44a1	4E-06	0.3
Tfdp2	3E-12	0.3	Bcas3	3E-09	0.3

Fibroblast					
Control			NICD2		
Gene	p	FC	Gene	p	FC
H2-Q7	2E-05	0.2	Socs2	2E-05	0.2
Aacs	1E-09	0.2	Nfkbia	5E-11	0.2
Rpl22	2E-272	0.2	Tmem19	2E-06	0.2
Cdc16	1E-34	0.2	Galnt10	2E-09	0.2
P2rx4	3E-17	0.2	Tsga10	3E-03	0.2
H19	2E-58	0.2	Magee1	3E-04	0.2
Gng10	3E-44	0.2	Magi2	6E-15	0.2
Nenf	1E-103	0.2	4930447C04Rik	1E-01	0.2
Mpv17	4E-23	0.2	2310001H17Rik	1E-01	0.2
Tmbim6	5E-59	0.2	Xaf1	4E-10	0.2
Meis3	3E-09	0.2	Angptl2	1E-05	0.2
Carm1	1E-14	0.2	Bex3	8E-34	0.2
Usp18	7E-05	0.2	Lrrk2	2E-04	0.2
Slc6a8	3E-14	0.2	Impact	4E-20	0.2
Angptl4	5E-23	0.2	Dbn1	1E-13	0.2
Ric8a	8E-12	0.2	Arsk	9E-05	0.2
Lrp3	1E-07	0.2	Rpl36	6E-144	0.2
Ccdc106	3E-01	0.2	Tbl1xr1	3E-18	0.2
Slc38a2	6E-30	0.2	Hoxd8	3E-05	0.2
Cdc42bpg	3E-04	0.2	Pdia5	1E-11	0.2
0610009L18Rik	3E-06	0.2	1700012D14Rik	1E-03	0.2
Actn1	1E-17	0.2	Hook2	9E-03	0.2
Acy1	1E-03	0.2	Cacna1d	1E-01	0.2
Abca1	4E-20	0.2	Tango6	3E-03	0.2
Klhl29	7E-39	0.2	Esyt1	1E-07	0.2
Rpl18a	1E-258	0.2	Elovl6	2E-08	0.2
Rps16	0E+00	0.2	Slc29a3	9E-04	0.2
Zfp267	3E-05	0.2	Bloc1s1	2E-09	0.2
Erbin	5E-35	0.2	Rps19	1E-161	0.2
Bcl3	5E-09	0.2	Ctsk	1E-33	0.2
Nbea	2E-25	0.2	Polr3d	4E-13	0.2
Sall2	7E-05	0.2	Inafm1	2E-05	0.2
Nynrin	6E-22	0.2	Samd8	3E-05	0.2
Hook2	4E-05	0.2	Tprkb	2E-07	0.2
Tmem64	9E-06	0.2	Lifr	1E-14	0.2
Rpl39	0E+00	0.2	Ttll5	5E-05	0.2
Snap29	4E-10	0.2	Prkd1	7E-08	0.2
Gxylt1	4E-06	0.2	Trim46	7E-05	0.2
Rad9b	3E-02	0.2	Man2b2	1E-03	0.2
Hdgfl3	8E-22	0.2	Epm2a	9E-02	0.2
Rrbp1	2E-131	0.2	B2m	1E-71	0.2
Myo9a	2E-30	0.2	AI413582	4E-10	0.2
Pdia5	3E-18	0.2	Htatip2	3E-15	0.2
Trim46	4E-07	0.2	Tubb2a	2E-20	0.2
Klhl36	1E-03	0.2	Tnfrsf23	4E-03	0.2
Aida	5E-29	0.2	Map3k14	5E-03	0.2
Lpar1	8E-29	0.2	Rilpl1	1E-11	0.2
Ebp	1E-21	0.2	Atf5	3E-22	0.2
Olfml2b	6E-17	0.2	Pld3	1E-22	0.2
Cela1	4E-06	0.2	Gga2	3E-12	0.2
Rpl31	4E-205	0.2	Slc38a10	5E-13	0.2
2610002M06Rik	2E-08	0.2	Gstm4	1E-02	0.2
Kank2	2E-20	0.2	Sod3	5E-18	0.2
Zfp951	6E-04	0.2	Zfp607b	5E-02	0.2
Polg2	2E-03	0.2	Ehd1	1E-13	0.2
Ccdc136	7E-14	0.2	1600012H06Rik	5E-07	0.2
Arhgap21	4E-21	0.2	Evi5l	4E-03	0.2
Lysmd2	3E-09	0.2	Tep1	5E-05	0.2
Trip6	3E-20	0.2	Rdh5	3E-02	0.2
Vsir	3E-06	0.2	Ric8a	7E-07	0.2
Pde8a	6E-28	0.2	Evc	7E-02	0.2
Cask	2E-49	0.2	Gpam	2E-03	0.2
Gng7	5E-04	0.2	Cpt2	3E-07	0.2
Map2k6	9E-05	0.2	Gxylt1	8E-03	0.2
Adamts2	2E-26	0.2	Maml3	2E-04	0.2
Emilin1	2E-22	0.2	Rab29	6E-06	0.2
Zdhhc1	6E-10	0.2	Fdft1	1E-11	0.2
Aqp5	9E-04	0.2	Ifngr2	1E-11	0.2
Uap1l1	8E-22	0.2	Zfas1	2E-17	0.2
H2-T23	8E-15	0.2	BC017158	2E-01	0.2
AI413582	3E-23	0.2	Rps10	1E-161	0.2
Cacng7	9E-07	0.2	Lysmd2	4E-05	0.2
Hmgcr	1E-13	0.2	Kctd10	1E-07	0.2

Fibroblast?					
Control			NICD2		
Gene	p	FC	Gene	p	FC
Uaca	5E-22	0.7	Pard6g	1E-39	0.7
Nop58	4E-60	0.7	Ablim1	4E-34	0.7
Cltb	7E-37	0.7	Ybx3	3E-102	0.7
Notch1	5E-36	0.7	Cd44	1E-50	0.7
Uap1	5E-33	0.7	Rras2	5E-28	0.7
Crybg3	2E-36	0.7	Ptma	2E-269	0.7
Ddb2	3E-18	0.7	Msn	3E-84	0.7
Cdkn1a	5E-47	0.7	Srsf1	3E-63	0.7
Zcchc8	4E-36	0.7	Plk3	1E-13	0.7
Tspan15	5E-23	0.7	Snapc2	4E-32	0.7
Tgfb1	1E-30	0.7	Magohb	1E-47	0.7
0610010F05Rik	2E-34	0.7	Atl2	4E-45	0.7
Iqgap1	4E-68	0.7	Arhgap10	1E-64	0.7
Ankrd26	4E-34	0.7	3110045C21Rik	5E-15	0.7
Tex9	2E-32	0.7	Cox6b2	4E-21	0.7
Rassf1	2E-36	0.7	Tent4a	3E-37	0.7
Smarca5	2E-97	0.7	Pop4	2E-46	0.7
Slc25a25	8E-30	0.7	Ing1	3E-58	0.7
Klf4	1E-39	0.7	Nova1	7E-23	0.7
Nolc1	6E-36	0.7	Gatad1	2E-99	0.7
Rmi1	4E-33	0.7	Pear1	1E-25	0.7
Fancl	3E-25	0.7	Cbfb	5E-61	0.7
Hnrnpf	3E-116	0.7	Mrs2	4E-38	0.7
Kdm8	1E-12	0.7	Arpp19	2E-128	0.7
Timp1	4E-66	0.7	Ak6	4E-50	0.7
Thyn1	5E-49	0.7	Psph	4E-44	0.7
4933408B17Rik	5E-19	0.7	Bckdk	1E-47	0.7
Ak2	3E-60	0.7	Ankrd9	9E-30	0.7
Zfp689	2E-13	0.7	Sdc1	7E-61	0.7
Pacsin2	2E-44	0.7	Gtf3c5	1E-23	0.7
Metrn	6E-42	0.7	Usp39	6E-45	0.7
Oard1	1E-39	0.7	Amd1	3E-38	0.7
Fbxo34	7E-33	0.7	Ptges3	6E-148	0.7
Grk2	8E-37	0.7	Cacna1c	9E-53	0.7
Larp1b	4E-34	0.7	Ereg	1E-27	0.7
Rbmxl1	2E-37	0.7	Med12l	2E-10	0.7
Idh3a	4E-47	0.7	Wbp11	5E-76	0.7
Tapbpl	2E-16	0.7	Dennd5b	3E-34	0.7
Mdga1	1E-28	0.7	Ubap2	2E-87	0.7
Pcdh19	1E-26	0.7	Tcirg1	3E-34	0.7
Orai2	4E-23	0.7	Fancl	1E-38	0.7
Stard4	5E-26	0.7	Asf1a	1E-53	0.7
Prps2	5E-36	0.7	Tmem109	6E-52	0.7
Cav1	4E-65	0.7	Slc16a7	3E-33	0.7
Cenpx	3E-69	0.7	Fut10	9E-38	0.7
Rassf3	5E-27	0.7	Dpy19l1	3E-44	0.7
Rftn1	3E-13	0.7	Zfp961	6E-15	0.7
Gm10069	2E-21	0.7	Soga1	2E-41	0.7
Baz1b	8E-62	0.7	Ppif	2E-21	0.7
Cisd2	2E-72	0.7	Fam20c	2E-35	0.7
Lnpk	2E-39	0.7	Kif2a	1E-74	0.7
Myzap	2E-22	0.7	Rnf144b	5E-20	0.7
Casp2	1E-27	0.7	Usp13	2E-28	0.7
Gm36033	9E-13	0.7	Cpox	4E-30	0.7
Dazap1	7E-53	0.7	Sh3rf2	5E-19	0.7
Glipr2	3E-33	0.7	Baz1b	2E-89	0.7
Ing5	4E-25	0.7	D330041H03Rik	3E-25	0.7
Crnde	2E-13	0.7	Slpi	3E-03	0.7
Anapc5	5E-72	0.7	Ccdc112	6E-35	0.7
Arap2	4E-21	0.7	Rab3ip	5E-26	0.7
Zfp934	1E-34	0.7	Ggta1	2E-33	0.7
Atp2b4	7E-17	0.7	Top3a	3E-32	0.7
Arnt2	2E-17	0.7	Lca5	1E-37	0.7
Cep162	1E-35	0.7	Gm10069	1E-24	0.7
Cep83	8E-30	0.7	Zdhhc14	1E-23	0.7
Sephs2	9E-28	0.7	Ybx1	6E-302	0.7
Tek	7E-09	0.7	Cenpx	2E-86	0.7
Tpm3	2E-101	0.7	Tead2	2E-50	0.7
9030624G23Rik	3E-18	0.7	Clcc1	1E-48	0.7
Mzf1	9E-11	0.7	Nhp2	5E-57	0.7
Ptprc	3E-37	0.7	Ppa1	3E-67	0.7
Tmem43	2E-39	0.7	Lingo1	5E-19	0.7
Fads2	2E-34	0.7	Sf3a3	1E-64	0.7

Undefined					
Control			NICD2		
Gene	p	FC	Gene	p	FC
Trim39	6E-01	0.1	Tmem218	2E-01	0.1
Pcmtd2	7E-02	0.1	Ndufs2	4E-08	0.1
Fam3a	2E-01	0.1	Raly	5E-02	0.1
Ahsa2	3E-01	0.1	Gadd45g	2E-01	0.1
Clec2d	2E-03	0.1	Ogfod3	7E-01	0.1
Fbxo28	9E-01	0.1	Fosb	4E-03	0.1
Atf6	2E-02	0.1	Zkscan1	2E-02	0.1
1700123O20Rik	4E-01	0.1	Eif3h	8E-13	0.1
Mterf2	6E-01	0.1	Arf6	2E-02	0.1
Ppp1cc	3E-04	0.1	Mllt11	8E-01	0.1
Zfp605	5E-01	0.1	Fam98c	5E-01	0.1
Mettl26	1E-01	0.1	Eml2	2E-01	0.1
Arrdc3	2E-01	0.1	Pqlc2	2E-01	0.1
Prkab1	1E-01	0.1	Slc30a5	8E-01	0.1
Churc1	2E-04	0.1	B9d2	1E+00	0.1
Brat1	9E-01	0.1	Zfp239	8E-01	0.1
Elmod3	4E-01	0.1	Slc27a3	1E+00	0.1
Tmed9	5E-19	0.1	P4ha2	2E-03	0.1
Snhg20	5E-01	0.1	Smpd2	7E-01	0.1
Laptm4a	2E-27	0.1	Sdhaf2	6E-02	0.1
Zfp954	6E-01	0.1	Ring1	6E-01	0.1
Gm2a	6E-03	0.1	Twnk	3E-01	0.1
Rabgef1	4E-02	0.1	Pou6f1	5E-01	0.1
Ccdc191	3E-01	0.1	Eid2b	1E-04	0.1
Ttc32	2E-01	0.1	Foxk1	3E-01	0.1
Anxa3	9E-01	0.1	Baiap2	2E-01	0.1
Nifk	3E-01	0.1	Tor1b	1E-01	0.1
Armc10	6E-01	0.1	Bcas3	2E-02	0.1
Mras	2E-02	0.1	Rsf1os1	8E-01	0.1
Rwdd3	8E-01	0.1	Wars	7E-01	0.1
Pard3bos1	7E-01	0.1	Spaca6	1E-01	0.1
Rabep2	2E-01	0.1	Slc19a1	6E-01	0.1
1810026B05Rik	3E-02	0.1	Taf2	7E-02	0.1
Arl5b	1E+00	0.1	Sars	1E-05	0.1
Spout1	3E-01	0.1	Jkamp	5E-03	0.1
Fam162a	3E-05	0.1	Mief1	2E-01	0.1
Aagab	4E-01	0.1	Pcp4l1	3E-01	0.1
Otud3	3E-01	0.1	Dgcr6	2E-02	0.1
Slc35a1	6E-02	0.1	Mthfs	7E-01	0.1
Gabarapl2	2E-05	0.1	Prnp	4E-10	0.1
Zfp53	9E-01	0.1	1110051M20Rik	1E-01	0.1
Lgals8	2E-02	0.1	Brms1	2E-01	0.1
Zfp169	8E-01	0.1	Kdm1a	7E-04	0.1
Lipt1	5E-01	0.1	Rnaset2a	1E-01	0.1
Oscp1	1E+00	0.1	Ube2i	2E-06	0.1
Rab30	7E-01	0.1	Rpap1	5E-01	0.1
Ctxn1	8E-01	0.1	Otub1	2E-01	0.1
Pard3b	5E-05	0.1	Ess2	2E-01	0.1
Irgm1	7E-01	0.1	Brox	9E-01	0.1
Uty	1E-01	0.1	Nucb1	2E-06	0.1
Ndufa12	2E-05	0.1	Hoxc10	1E-01	0.1
Smarcd1	1E-02	0.1	Mrpl41	1E-01	0.1
Cep44	5E-01	0.1	Jund	2E-08	0.1
A630072M18Rik	5E-01	0.1	Ciz1	6E-01	0.1
Ankrd10	5E-01	0.1	Arpc5l	4E-04	0.1
Smim3	8E-01	0.1	Cdc26	1E-05	0.1
Mettl27	6E-01	0.1	Parl	5E-01	0.1
Ypel2	9E-01	0.1	Col6a2	3E-07	0.1
Zfand5	9E-07	0.1	Dock5	3E-01	0.1
Enox2	7E-01	0.1	Pam16	4E-04	0.1
Tecpr1	5E-01	0.1	Il15ra	4E-01	0.1
Cxxc5	3E-02	0.1	Tpi1	3E-07	0.1
Itpr1	2E-03	0.1	Sh3glb2	2E-01	0.1
Tm2d3	2E-03	0.1	Mlst8	2E-01	0.1
Calml4	1E+00	0.1	Acp2	2E-01	0.1
Fam192a	7E-02	0.1	Alkbh2	6E-01	0.1
Ampd2	3E-02	0.1	Ints14	7E-01	0.1
Vac14	4E-02	0.1	Ldha	4E-06	0.1
H2-T23	1E+00	0.1	Paip1	2E-01	0.1
Ube2b	4E-13	0.1	Faap20	2E-01	0.1
Sdcbp	2E-03	0.1	Myl12b	2E-03	0.1
Coasy	4E-01	0.1	Zgpat	1E+00	0.1
Hyal2	7E-02	0.1	Bloc1s6	6E-01	0.1

Articular/Synovial Fibroblast					
Control			NICD2		
Gene	p	FC	Gene	p	FC
4930522L14Rik	9E-01	0.3	Tspo	4E-08	0.3
Rom1	4E-01	0.3	Zfp182	2E-01	0.3
Cdkn2a	2E-01	0.3	Nme7	1E-01	0.3
Pqlc1	3E-01	0.3	AC149090.1	1E-06	0.3
C920021L13Rik	8E-01	0.3	Ccdc114	4E-01	0.3
Tceanc	9E-01	0.3	Fbxo25	6E-01	0.3
Slc35d2	8E-01	0.3	Dvl2	8E-01	0.3
Irf2	4E-04	0.3	Aifm2	6E-01	0.3
Zfp984	5E-01	0.3	Pmp22	2E-08	0.3
Iffo1	7E-01	0.3	Nagpa	9E-01	0.3
Klhl12	8E-01	0.3	Fuca1	4E-12	0.3
Ethe1	1E-03	0.3	Firre	8E-01	0.3
Dapp1	8E-01	0.3	Mef2d	8E-02	0.3
Rfx5	8E-01	0.3	Ro60	2E-03	0.3
Dab2	2E-11	0.3	Mapk1ip1	5E-01	0.3
Zhx3	3E-02	0.3	Mpst	1E-01	0.3
Hspb1	3E-07	0.3	Bmp1	2E-03	0.3
Inpp5a	4E-02	0.3	Garnl3	6E-01	0.3
Ino80c	3E-01	0.3	Mdk	8E-02	0.3
Ncoa7	5E-02	0.3	Adamtsl4	1E-01	0.3
Gusb	8E-04	0.3	Ak3	1E-01	0.3
Zmat5	2E-07	0.3	Ikzf4	7E-01	0.3
Pdha1	8E-10	0.3	Ptp4a3	3E-01	0.3
Cstb	8E-24	0.3	Trim16	9E-01	0.3
Jmjd8	7E-01	0.3	Mpdu1	3E-01	0.3
Slc9a3r1	3E-01	0.3	Arnt	1E-04	0.3
Zfp13	9E-01	0.3	Slc12a6	9E-02	0.3
2900052L18Rik	6E-01	0.3	4930517O19Rik	8E-01	0.3
Abtb1	8E-01	0.3	F420014N23Rik	8E-01	0.3
Fam57a	4E-01	0.3	Ftl1	5E-34	0.3
Slc39a3	5E-01	0.3	Vhl	6E-01	0.3
Auh	4E-06	0.3	Clstn1	6E-03	0.3
Pard3bos1	1E+00	0.3	Sh3pxd2a	1E-04	0.3
Zfp943	1E-01	0.3	Cd63	3E-35	0.3
Ssbp2	2E-04	0.3	S100a13	8E-13	0.3
Mettl21a	5E-03	0.3	Pld3	2E-07	0.3
Pptc7	2E-01	0.3	Ang	7E-01	0.3
Zc3hav1	1E-05	0.3	Fam102a	4E-01	0.3
4930550C14Rik	1E+00	0.3	Nat9	3E-02	0.3
Ptcd1	6E-01	0.3	Grina	5E-08	0.3
Cant1	2E-01	0.3	Zfp235	5E-01	0.3
Wdpcp	2E-02	0.3	Hoxa13	7E-01	0.3
Neu1	2E-01	0.3	Zfp667	1E+00	0.3
1500015L24Rik	2E-02	0.3	Tti2	9E-01	0.3
Zfp738	2E-01	0.3	Taok3	3E-04	0.3
Eif5a2	1E+00	0.3	Gns	4E-06	0.3
Snhg14	7E-01	0.3	B3gnt9	7E-03	0.3
Pomk	6E-01	0.3	1110051M20Rik	2E-03	0.3
Khnyn	9E-01	0.3	Fbrsl1	4E-01	0.3
D430042O09Rik	2E-03	0.3	Plcl2	7E-01	0.3
Map2k5	3E-04	0.3	Xpr1	1E-01	0.3
Ggh	2E-11	0.3	Enox2	3E-03	0.3
Foxp1	5E-19	0.3	Rhobtb2	8E-01	0.3
Ubtd1	3E-02	0.3	Rgs17	3E-02	0.3
Iah1	1E-06	0.3	Irak4	4E-01	0.3
Pde4b	1E-01	0.3	Cpq	1E-15	0.3
Dusp1	1E-05	0.3	Orc5	6E-01	0.3
Lipo3	9E-01	0.3	Rilpl2	2E-01	0.3
Dhx34	4E-01	0.3	Nxpe4	3E-02	0.3
Bbs9	3E-01	0.3	Vipas39	6E-02	0.3
Fosl1	8E-01	0.3	Gfpt2	5E-01	0.3
Hsdl2	2E-01	0.3	Rabgap1l	2E-05	0.3
Mad2l2	2E-01	0.3	Tmem175	7E-02	0.3
Acot11	6E-01	0.3	Uhrf1bp1l	6E-02	0.3
Pxylp1	9E-01	0.3	Pafah2	8E-01	0.3
Eml5	1E+00	0.3	Dpy19l3	8E-01	0.3
Zscan12	7E-01	0.3	Gm5617	2E-02	0.3
S100a10	6E-14	0.3	Myo1e	3E-01	0.3
Layn	8E-03	0.3	Sirt5	7E-01	0.3
Prdx5	2E-13	0.3	Hdx	7E-01	0.3
Wdr91	9E-01	0.3	Mfsd11	6E-01	0.3
Cdk19	6E-02	0.3	Zfp790	5E-01	0.3
Arrdc3	6E-01	0.3	Mtmr10	9E-01	0.3

Limb Mesenchyme						Chondrogenic					
Control			NICD2			Control			NICD2		
Gene	*p*	FC	Gene	*p*	FC	Gene	*p*	FC	Gene	*p*	FC
Foxred2	2E-03	0.3	Tgif1	1E-04	0.3	Dbp	7E-06	0.3	Ube4b	4E-09	0.3
Syt1	2E-05	0.3	2900097C17Rik	1E-14	0.3	Acsf2	5E-01	0.3	Smpdl3b	2E-02	0.3
Gdf11	1E-05	0.3	Zc3hav1l	3E-04	0.3	Gli3	1E-18	0.3	Mir100hg	4E-17	0.3
Rnf4	8E-11	0.3	Cep41	2E-05	0.3	Tapbpl	1E-01	0.3	Bicral	3E-06	0.3
2900097C17Rik	7E-18	0.3	Simc1	2E-03	0.3	Mar6	2E-09	0.3	Nckap5	1E-09	0.3
Mertk	5E-09	0.3	Mdfic	1E-11	0.3	Zfp27	1E-01	0.3	Wdr34	7E-02	0.3
Chic1	1E-06	0.3	Nrip2	7E-03	0.3	Aasdh	4E-03	0.3	Gde1	2E-09	0.3
Tom1l2	4E-11	0.3	Chchd7	8E-14	0.3	Idua	3E-02	0.3	Gorasp1	3E-01	0.3
Txndc11	7E-09	0.3	Cables1	2E-04	0.3	4732471J01Rik	3E-03	0.3	3010001F23Rik	2E-01	0.3
Sparc	6E-43	0.3	Gm17435	4E-03	0.3	Cfap300	2E-01	0.3	Zfp949	1E-01	0.3
Tmem245	9E-08	0.3	Itch	3E-13	0.3	Gm26782	7E-08	0.3	Gm16536	7E-01	0.3
Stxbp4	5E-10	0.3	Mccc1	2E-06	0.3	Vezf1	4E-18	0.3	Znrd1as	1E-02	0.3
Ptk2	2E-13	0.3	Nfatc1	1E-04	0.3	Mxra8	6E-29	0.3	Zfp566	9E-02	0.3
Dbn1	3E-11	0.3	2210408I21Rik	2E-05	0.3	Smad6	1E-06	0.3	Lrch3	1E-07	0.3
AC103362.1	1E-05	0.3	Ndufaf4	6E-10	0.3	Gm12655	9E-03	0.3	Slc39a14	8E-05	0.3
Trim41	1E-07	0.3	Nars2	8E-06	0.3	Grsf1	3E-07	0.3	Nim1k	1E-02	0.3
Eml1	4E-07	0.3	Fbxw8	3E-09	0.3	Trim24	8E-09	0.3	Tenm3	3E-06	0.3
Snhg1	8E-18	0.3	Kcnq1ot1	9E-11	0.3	Usf3	2E-03	0.3	Wdr35	3E-05	0.3
Zbtb38	1E-12	0.3	Ttc13	1E-06	0.3	Zfp821	9E-04	0.3	Zfp24	2E-05	0.3
Atoh8	1E-04	0.3	Rybp	6E-08	0.3	Apmap	6E-04	0.3	Mccc1	3E-03	0.3
Rpap1	5E-06	0.3	Gm15834	5E-03	0.3	Dnal4	4E-05	0.3	Lin52	2E-02	0.3
Fgfr1	3E-19	0.3	Golga2	3E-09	0.3	Ccdc126	5E-04	0.3	Mks1	3E-01	0.3
Eaf1	8E-06	0.3	Atg4d	6E-04	0.3	2810001G20Rik	4E-02	0.3	4931428F04Rik	3E-01	0.3
Lysmd1	1E-04	0.3	Wsb1	5E-10	0.3	Cux1	2E-09	0.3	Ap3s2	3E-03	0.3
Rnf121	2E-07	0.3	Ldah	1E-10	0.3	Cd320	1E-12	0.3	Ttc33	4E-06	0.3
Dph6	1E-12	0.3	Cbwd1	7E-08	0.3	3110040N11Rik	2E-10	0.3	St7l	8E-04	0.3
Pomt2	4E-07	0.3	Arid1a	2E-09	0.3	Zmat1	1E-02	0.3	Acot11	2E-02	0.3
Mar6	2E-08	0.3	Fam114a1	2E-24	0.3	Kansl1l	2E-11	0.3	Zbed5	2E-01	0.3
Sh3rf1	5E-09	0.3	Amot	1E-08	0.3	Dcbld2	1E-03	0.3	Zkscan1	1E-08	0.3
Gtf2e1	2E-06	0.3	Eif4e	8E-15	0.3	Slc39a11	2E-04	0.3	Fam98b	3E-08	0.3
Vdac3	3E-28	0.3	Arf2	1E-07	0.3	Hoxc6	2E-06	0.3	Nop2	6E-02	0.3
Rsl24d1	8E-11	0.3	Ap1b1	1E-06	0.3	Dalrd3	6E-04	0.3	Avpi1	1E-10	0.3
Pstpip2	5E-04	0.3	Foxk2	5E-07	0.3	Cep63	3E-06	0.3	Klhdc8a	7E-02	0.3
Slc39a9	4E-07	0.3	Ndor1	2E-04	0.3	Rmnd5b	5E-04	0.3	Tle1	9E-04	0.3
Hist1h1c	5E-11	0.3	Aff4	2E-19	0.3	Ascc2	7E-06	0.3	Acsf2	7E-01	0.3
Senp7	4E-15	0.3	Cdc73	3E-09	0.3	Mzt1	3E-07	0.3	Sparc	5E-42	0.3
Nop56	2E-12	0.3	Hnrnpdl	2E-12	0.3	Usp11	1E-03	0.3	Dhx30	8E-06	0.3
Sec13	8E-16	0.3	Ebag9	9E-09	0.3	Ogt	5E-16	0.3	Wasf3	9E-03	0.3
Ralgapb	6E-09	0.3	BC065397	5E-04	0.3	Rasa2	1E-03	0.3	Clu	3E-08	0.3
Kyat1	2E-04	0.3	Wls	9E-30	0.3	Cetn4	2E-03	0.3	AW209491	7E-02	0.3
Rgp1	6E-09	0.3	Serpinh1	3E-51	0.3	Phactr4	4E-07	0.3	Dnajb9	2E-06	0.3
Casc4	1E-18	0.3	Hdac9	6E-02	0.3	Smim27	6E-08	0.3	Sh3glb2	1E-04	0.3
Usp36	1E-08	0.3	Igf2bp2	3E-11	0.3	Aktip	5E-08	0.3	Mplkip	9E-07	0.3
Nfatc1	2E-08	0.3	4930581F22Rik	3E-03	0.3	Tm9sf4	7E-25	0.3	Chd2	2E-10	0.3
Veph1	3E-06	0.3	Tfrc	2E-05	0.3	Usp20	2E-02	0.3	Ndor1	9E-02	0.3
Zfp821	2E-09	0.3	Zfp57	1E-03	0.3	Tctn2	4E-02	0.3	Lars2	2E-08	0.3
Car6	5E-04	0.3	Tsc22d1	6E-21	0.3	Yipf4	6E-24	0.3	Hdac6	2E-01	0.3
Mafg	3E-11	0.3	9430091E24Rik	7E-04	0.3	Gm14858	2E-01	0.3	Hipk1	6E-06	0.3
Slc37a4	6E-05	0.3	LTO1	9E-05	0.3	Syt11	1E-02	0.3	Bsg	4E-37	0.3
Elac2	6E-04	0.3	Alg1	2E-06	0.3	Phactr2	3E-11	0.3	Lrch2	8E-02	0.3
Qk	3E-22	0.3	Kdm5b	1E-14	0.3	Tubg2	2E-01	0.3	Sybu	2E-02	0.3
Gm39090	6E-03	0.3	Etv1	2E-03	0.3	Tatdn2	2E-01	0.3	Kctd6	1E-01	0.3
Ror2	2E-07	0.3	Atf6	1E-10	0.3	Zfp653	8E-01	0.3	Crlf3	4E-01	0.3
2410006H16Rik	3E-19	0.3	Ero1l	2E-12	0.3	Sdr39u1	1E-05	0.3	Fahd1	2E-02	0.3
Dcun1d4	2E-05	0.3	Nrd1	9E-15	0.3	Tsacc	1E-02	0.3	Bbs9	5E-03	0.3
Slc41a2	7E-09	0.3	Rab30	1E-09	0.3	Med24	5E-02	0.3	Naa16	7E-04	0.3
Nifk	6E-11	0.3	Kdm4b	3E-04	0.3	Xrcc4	4E-09	0.3	Gli3	4E-11	0.3
Igip	2E-06	0.3	Slc35c2	3E-08	0.3	Snrpb2	1E-08	0.3	Mmgt2	6E-04	0.3
N4bp1	2E-12	0.3	Emp2	1E-09	0.3	Fbf1	2E-02	0.3	Zfp821	3E-02	0.3
Galnt5	4E-04	0.3	Mak16	1E-10	0.3	Txndc15	7E-19	0.3	Ndufaf7	6E-02	0.3
Ppp1r12a	4E-16	0.3	Hspbap1	3E-02	0.3	Cdpf1	9E-05	0.3	Mtif2	4E-06	0.3
Pde1c	8E-03	0.3	Cd151	1E-12	0.3	Klf12	5E-11	0.3	Phf1	5E-02	0.3
Gufl	5E-07	0.3	Rab33b	1E-06	0.3	Copg2	3E-09	0.3	Wdr73	3E-02	0.3
Pelo	3E-10	0.3	Setd7	2E-09	0.3	2300009A05Rik	2E-06	0.3	Xrcc4	2E-05	0.3
Tmem44	1E-02	0.3	Zc3h7b	3E-07	0.3	Tob1	7E-11	0.3	Sypl	3E-09	0.3
Srpr	2E-21	0.3	Ttpal	2E-05	0.3	Lancl2	6E-01	0.3	Trappc6a	1E-09	0.3
Tsc22d2	3E-17	0.3	Ccnd3	8E-13	0.3	Mtg2	3E-04	0.3	Tti2	5E-01	0.3
Zfp266	2E-13	0.3	Taf1b	8E-06	0.3	Mmut	2E-05	0.3	Slc4a7	2E-05	0.3
Ddx55	3E-06	0.3	Esr1	8E-03	0.3	Desi2	5E-20	0.3	Sec62	2E-43	0.3
Chordc1	1E-15	0.3	Ncdn	2E-04	0.3	Cab39l	5E-09	0.3	Ints6l	7E-03	0.3
Saraf	2E-11	0.3	Gnl3l	6E-10	0.3	Ubxn2a	1E-05	0.3	Borcs6	1E-02	0.3
Efna4	7E-09	0.3	Szt2	3E-04	0.3	Lpin2	5E-03	0.3	Ube4bos1	2E-01	0.3
Fbxl18	2E-06	0.3	Ddx51	1E-02	0.3	Map2k3os	2E-01	0.3	Cage1	3E-01	0.3

Fibroblast						Fibroblast?					
Control			NICD2			Control			NICD2		
Gene	*p*	FC	Gene	*p*	FC	Gene	*p*	FC	Gene	*p*	FC
Efna2	6E-05	0.2	Myo9a	2E-14	0.2	Slc35g2	2E-12	0.7	Frmd8	2E-25	0.7
Rbfox1	1E-02	0.2	Cdr2l	2E-03	0.2	Usp31	1E-27	0.7	Trim27	4E-40	0.7
Cul7	2E-13	0.2	Mpv17	1E-11	0.2	Ier5	1E-36	0.7	Pde4a	8E-31	0.7
Dlg2	8E-02	0.2	Epn2	2E-07	0.2	Thop1	5E-34	0.7	Cdk6	1E-27	0.7
Msrb1	3E-26	0.2	Plxdc2	8E-19	0.2	Acd	4E-31	0.7	Hapln1	8E-24	0.7
Tmem126b	5E-10	0.2	Elf4	1E-08	0.2	Enho	5E-13	0.7	Bcdin3d	1E-16	0.7
Ifit1	5E-14	0.2	Zfp442	1E-02	0.2	Itpa	2E-29	0.7	Cdc27	1E-53	0.7
Golm1	2E-10	0.2	Calr	2E-36	0.2	Map6	2E-31	0.7	2610203C22Rik	1E-12	0.7
Prkd1	6E-11	0.2	A4galt	1E-05	0.2	Shtn1	2E-14	0.7	Ppwd1	9E-44	0.7
Col5a2	3E-87	0.2	Endog	8E-05	0.2	Pcbp3	5E-28	0.7	Ptprn	4E-13	0.7
Brdt	3E-03	0.2	Gm26672	5E-03	0.2	Afap1l2	5E-14	0.7	Spin4	2E-25	0.7
Elf4	2E-12	0.2	Ltbr	3E-08	0.2	Rrp1b	1E-25	0.7	Eftud2	3E-47	0.7
Gm14325	5E-21	0.2	Prkn	8E-10	0.2	Mthfd2	3E-44	0.7	Hnrnpa1	1E-98	0.7
Alpk1	6E-07	0.2	Cacng7	1E-04	0.2	Nol4l	9E-10	0.7	Mrpl51	5E-108	0.7
Ddah2	3E-49	0.2	Xkr6	1E-02	0.2	Pcbd1	2E-17	0.7	Slc20a2	2E-33	0.7
4930447C04Rik	2E-02	0.2	Ube2o	2E-05	0.2	Adgre5	7E-21	0.7	Nudt21	5E-87	0.7
D130040H23Rik	4E-05	0.2	Fbxo17	6E-08	0.2	Nav3	1E-11	0.7	Gmps	8E-72	0.7
Arvcf	2E-03	0.2	Adam15	3E-07	0.2	Chac2	9E-15	0.7	Tpm3-rs7	2E-16	0.7
Psmb10	7E-19	0.2	Ldlr	6E-10	0.2	Cbx3	2E-102	0.7	Tnfaip8	3E-57	0.7
Kctd17	5E-13	0.2	Snai1	2E-08	0.2	Gm11110	1E-12	0.7	Odf2	4E-44	0.7
Trappc2l	3E-50	0.2	Fam13a	1E-02	0.2	Ifi27l2a	1E-14	0.7	Dtx4	5E-06	0.7
Fam118a	4E-04	0.2	Srd5a3	1E-04	0.2	Pbdc1	2E-48	0.7	Myef2	4E-54	0.7
Gm10373	2E-03	0.2	Trappc2l	7E-29	0.2	AC103362.1	8E-10	0.7	A930001C03Rik	4E-28	0.7
Hoxd8	2E-07	0.2	Tmem219	3E-09	0.2	Ybx1	1E-211	0.7	Itpa	4E-43	0.7
Upf1	2E-13	0.2	Lmf1	7E-05	0.2	Xrcc6	9E-34	0.7	Aida	8E-59	0.7
Ltbr	2E-11	0.2	Rpl13	5E-155	0.2	Cyyr1	1E-09	0.7	Stub1	1E-146	0.7
Tmem14a	1E-08	0.2	Rps16	4E-158	0.2	Myh10	1E-50	0.7	Ciao2a	4E-51	0.7
Gm2000	1E-30	0.2	Cyp4f17	4E-06	0.2	Polk	3E-33	0.7	Slc16a3	2E-22	0.7
C1galt1	1E-12	0.2	Gtdc1	7E-08	0.2	Notch3	2E-17	0.7	Rab3b	5E-18	0.7
Daam1	2E-09	0.2	Eid1	8E-42	0.2	Dagla	5E-15	0.7	Odc1	1E-45	0.7
Lss	4E-06	0.2	Snhg6	8E-11	0.2	Ak6	5E-35	0.7	Slc22a4	2E-15	0.7
Esd	5E-101	0.2	Smyd5	3E-02	0.2	Fam217b	2E-16	0.7	Lrrc59	4E-65	0.7
Lynx1	6E-10	0.2	Sfxn5	9E-04	0.2	Grk6	4E-28	0.7	Prkch	4E-21	0.7
Trip10	2E-08	0.2	Uap1l1	5E-12	0.2	Osbpl6	1E-25	0.7	Spag4	2E-14	0.7
Tnrc6a	2E-33	0.2	Frk	3E-05	0.2	Erh	1E-74	0.7	Gpank1	6E-33	0.7
Eid1	6E-79	0.2	Ccdc136	2E-08	0.2	Angpt2	3E-23	0.7	Slc10a7	6E-48	0.7
Zfp251	2E-06	0.2	Vasp	8E-09	0.2	Klhdc4	5E-25	0.7	Npnt	1E-19	0.7
Fryl	1E-07	0.2	Prcp	6E-06	0.2	A430018G15Rik	9E-06	0.7	Ctps	9E-51	0.7
Ppp1r15b	1E-07	0.2	Ddah2	3E-25	0.2	Ccdc141	2E-13	0.7	Sinhcaf	7E-25	0.7
Frg2f1	2E-04	0.2	Stt3a	4E-13	0.2	Thoc3	1E-41	0.7	Rdm1	1E-53	0.6
Tbc1d24	3E-03	0.2	Rpl35	2E-119	0.2	Rab3ip	1E-20	0.7	Fam76b	5E-48	0.6
Gba	5E-09	0.2	Rnf149	2E-03	0.2	Dusp14	1E-20	0.7	Gm48228	3E-19	0.6
Med10	2E-23	0.2	Gab2	2E-04	0.2	Vps37b	2E-24	0.7	Sart3	3E-37	0.6
Dnpep	1E-22	0.2	Tnfrsf1b	1E-02	0.2	Fadd	7E-18	0.7	Arap2	3E-29	0.6
Evc	1E-04	0.2	Gnpda2	1E-09	0.2	Slc25a24	2E-39	0.7	Cdh13	4E-29	0.6
Klf10	1E-06	0.2	Zcchc24	3E-14	0.2	Tmem209	6E-32	0.7	Hdgfl2	1E-54	0.6
Tmem97	2E-12	0.2	Kdelr3	6E-11	0.2	D330041H03Rik	3E-22	0.7	Mgat4a	3E-19	0.6
Parp12	1E-08	0.2	Plk2	2E-04	0.2	Dnajb1	1E-44	0.7	Patl1	5E-21	0.6
Ctsk	4E-55	0.2	Sec23a	2E-15	0.2	C730034F03Rik	8E-06	0.7	Pbdc1	7E-61	0.6
1110012L19Rik	3E-14	0.2	Galm	1E-02	0.2	Zfp599	8E-12	0.7	Erh	3E-85	0.6
Rpl34	2E-249	0.2	Gm6710	2E-03	0.2	Dkc1	1E-31	0.7	Plau	4E-29	0.6
Tspo	3E-78	0.2	Meis2	1E-08	0.2	Hprt	2E-42	0.7	Stip1	1E-74	0.6
Rnf19b	2E-04	0.2	Mfsd10	6E-06	0.2	Cpsf4	2E-22	0.7	Set	2E-147	0.6
Adarb1	5E-05	0.2	Asah1	1E-32	0.2	Lrrcc1	9E-32	0.7	Aqp5	2E-07	0.6
Top1mt	2E-05	0.2	Gm10516	1E-01	0.2	Fli1	4E-18	0.7	Gab3	2E-18	0.6
Nxpe4	1E-16	0.2	Ddr2	4E-18	0.2	Depdc5	2E-34	0.7	Vps37b	2E-28	0.6
Atraid	8E-63	0.2	Sbno2	2E-06	0.2	Psmd13	3E-59	0.7	Pag1	6E-48	0.6
Tmem127	3E-09	0.2	Scamp5	1E-02	0.2	Trpv4	9E-19	0.7	Ilf2	4E-64	0.6
Gli2	6E-03	0.2	Gm14325	3E-11	0.2	Hdgf	8E-104	0.7	Cnnm4	3E-19	0.6
Rpl17	8E-279	0.2	Tbc1d24	6E-02	0.2	Slc25a28	1E-24	0.7	Dgcr8	2E-40	0.6
Tbx3	7E-03	0.2	Itprid2	2E-05	0.2	Vangl1	3E-26	0.7	Cwf19l1	2E-16	0.6
Galnt10	1E-12	0.2	Wnt5b	8E-01	0.2	Tubb2b	4E-26	0.7	Klhdc4	4E-36	0.6
Ccdc85b	2E-34	0.2	Mospd3	6E-05	0.2	Rnf144b	2E-15	0.7	Opa3	7E-38	0.6
Uba6	2E-11	0.2	Rpl22	1E-117	0.2	Srbd1	2E-38	0.7	Ppm1m	3E-47	0.6
Samd8	3E-08	0.2	Pigh	1E-02	0.2	Zfp595	2E-07	0.7	Gm5914	4E-16	0.6
Ssbp4	2E-25	0.2	Hpcal1	6E-06	0.2	Fam92a	7E-66	0.7	Def6	1E-26	0.6
Sgtb	6E-05	0.2	Tmem38b	1E-04	0.2	Trim62	3E-11	0.7	Pgap1	2E-28	0.6
Smyd5	9E-04	0.2	Homer1	5E-02	0.2	Ccng1	3E-28	0.7	Nolc1	2E-39	0.6
Epn2	5E-13	0.2	C4b	1E-09	0.2	Fez1	6E-28	0.7	Hint1	2E-231	0.6
Snrk	3E-11	0.2	Dpm3	1E-26	0.2	Gfod1	3E-10	0.7	Maz	3E-49	0.6
Gas1	1E-76	0.2	Coa4	3E-04	0.2	Kmt5a	1E-64	0.7	3110082I17Rik	4E-35	0.6
Dlg1	1E-28	0.2	Cd63	1E-57	0.2	Eefsec	4E-27	0.7	Spata24	2E-41	0.6
Rps20	7E-253	0.2	Pmm1	4E-17	0.2	Plau	2E-30	0.7	Specc1l	1E-28	0.6

Undefined						Articular/Synovial Fibroblast					
Control			NICD2			Control			NICD2		
Gene	*p*	FC	Gene	*p*	FC	Gene	*p*	FC	Gene	*p*	FC
Ccdc39	5E-01	0.1	Hif1an	4E-01	0.1	Spry2	6E-01	0.3	Vps13b	1E-04	0.3
Nphs1	6E-01	0.1	Ercc8	7E-01	0.1	Xkr6	5E-01	0.3	Rfxank	9E-01	0.3
Tmem11	2E-01	0.1	Lpar4	8E-01	0.1	Cic	2E-02	0.3	D930016D06Rik	4E-01	0.3
Rab11fip5	7E-01	0.1	Abat	9E-01	0.1	Iqcc	2E-01	0.3	Naaladl2	4E-09	0.3
Tmem115	3E-01	0.1	Phc2	6E-01	0.1	Map3k2	7E-04	0.3	Fam189b	2E-01	0.3
Slc25a30	7E-01	0.1	Mapkbp1	4E-01	0.1	Mms19	3E-01	0.3	Mapkapk3	7E-01	0.3
Pfkfb2	9E-01	0.1	Dleu2	4E-02	0.1	Tmem192	9E-04	0.3	Sec24c	5E-01	0.3
Creb3l1	3E-03	0.1	Zfp157	6E-01	0.1	Insig2	2E-05	0.3	Ehd2	3E-06	0.3
Pheta1	5E-01	0.1	Ppp4r1	8E-01	0.1	Tshz1	1E-02	0.3	Slc2a10	8E-01	0.3
Mtrf1	1E-01	0.1	Pard3b	2E-03	0.1	Prmt9	4E-01	0.3	Ttc7b	2E-01	0.3
Lrrfip1	1E-02	0.1	Adamts4	7E-01	0.1	Nr1h3	8E-02	0.3	Rpp25l	3E-04	0.3
Anxa7	3E-03	0.1	N6amt1	2E-01	0.1	Sdccag8	1E-05	0.3	D430042O09Rik	3E-02	0.3
Myo6	2E-01	0.1	Mogs	5E-01	0.1	Gba	9E-03	0.3	Fgf10	7E-02	0.3
Zfp40	1E+00	0.1	Tfpt	3E-01	0.1	Bmp1	3E-04	0.3	Slc35f5	2E-01	0.3
Spryd3	9E-01	0.1	S100a13	1E-03	0.1	Klhl24	2E-03	0.3	2510009E07Rik	6E-02	0.3
Ubxn7	2E-01	0.1	Vamp7	5E-01	0.1	Ints6	1E-02	0.3	Prelid2	8E-01	0.3
Mtx1	5E-01	0.1	Smn1	5E-01	0.1	Cdkn2b	2E-07	0.3	Iqsec2	7E-01	0.3
H3f3a	3E-29	0.1	Tmem63b	5E-01	0.1	Gtpbp3	6E-01	0.3	Cpeb4	8E-03	0.3
Slc25a33	5E-01	0.1	Cped1	3E-03	0.1	Dgka	7E-01	0.3	Ddx28	9E-01	0.3
Cdkn2a	1E-02	0.1	Adam22	1E+00	0.1	Rhobtb1	6E-02	0.3	D2hgdh	1E+00	0.3
Zfp521	1E-09	0.1	Zfp653	7E-01	0.1	Ttc5	2E-01	0.3	Trak1	2E-02	0.3
Fam204a	4E-03	0.1	Tgoln1	8E-05	0.1	Ro60	2E-02	0.3	Dusp3	2E-01	0.3
Ngdn	2E-01	0.1	Arrdc3	1E+00	0.1	Trmt2b	4E-01	0.3	Clcn6	8E-01	0.3
Gtf2ird1	2E-01	0.1	H2-T23	9E-01	0.1	Acer3	4E-02	0.3	Abl2	2E-02	0.3
Mettl1	3E-03	0.1	Zbtb24	8E-01	0.1	Anks1	2E-01	0.3	Cyp4f13	5E-01	0.3
Hif1an	2E-01	0.1	Sh3rf1	4E-01	0.1	Tnfrsf1a	4E-07	0.3	Acadm	2E-08	0.3
Fbxo36	1E-01	0.1	Fcgrt	2E-07	0.1	Cyba	3E-15	0.3	Trim34a	9E-01	0.3
Ndrg3	3E-01	0.1	Ttc5	4E-01	0.1	Chst2	1E-01	0.3	Uqcrh	7E-32	0.3
Hoxc6	5E-01	0.1	Pim3	7E-01	0.1	Retreg3	1E+00	0.3	Dnase1l1	6E-01	0.3
Elk3	3E-01	0.1	Tm4sf1	8E-01	0.1	Xpr1	5E-01	0.3	Lcmt2	7E-01	0.3
Osbpl6	7E-01	0.1	Dnajb6	1E-04	0.1	Flrt2	4E-03	0.3	Tm2d2	7E-07	0.3
Higd1a	4E-05	0.1	Sdccag8	7E-02	0.1	Reps2	4E-01	0.3	Mfn2	2E-01	0.3
Flrt2	9E-01	0.1	Mospd1	8E-02	0.1	Rapgef4	8E-01	0.3	Prr13	4E-06	0.3
Acadm	1E-08	0.1	Gtf2ird1	4E-01	0.1	Sik1	7E-01	0.3	Castor2	6E-01	0.3
6330562C20Rik	6E-01	0.1	Hist1h2bc	2E-05	0.1	Mtmr4	3E-01	0.3	Ubtd1	4E-01	0.3
Baz2a	4E-01	0.1	Sdf4	1E-11	0.1	A930037H05Rik	9E-01	0.3	Zfp704	1E-01	0.3
Dnttip1	3E-01	0.1	Slc25a3	1E-14	0.1	Rnf169	1E-04	0.3	Evc	7E-01	0.3
C1qtnf1	2E-01	0.1	Sp4	5E-01	0.1	Nudt16	8E-01	0.3	Snx32	5E-01	0.3
Taf1a	5E-01	0.1	Atat1	9E-01	0.1	Prkx	9E-01	0.3	Inpp5a	2E-01	0.3
Dpm1	2E-04	0.1	Gm28198	8E-01	0.1	Litaf	1E-02	0.3	Mat2a	1E-03	0.3
Ndufaf1	5E-01	0.1	Afg3l1	1E+00	0.1	Mdm2	2E-03	0.3	Abcd4	6E-01	0.3
Fkbp11	2E-02	0.1	Zfp113	1E+00	0.1	Hoxa7	2E-01	0.3	Cfap410	7E-01	0.3
Cobll1	3E-02	0.1	Mrps10	2E-01	0.1	Rex1bd	1E-16	0.3	Rnf169	3E-03	0.3
Zfp943	4E-01	0.1	Pus10	4E-01	0.1	Zfp2	1E+00	0.3	Bri3	7E-15	0.3
Mpnd	1E-01	0.1	Ftsj1	8E-02	0.1	9130008F23Rik	4E-01	0.3	Dram2	5E-01	0.3
Tcea3	1E-04	0.1	Irf7	3E-03	0.1	Trim34a	4E-01	0.3	Rab9	6E-09	0.3
Zfp157	9E-01	0.1	Mcub	5E-01	0.1	Tufm	1E-02	0.3	Polg2	9E-01	0.3
Elk1	6E-02	0.1	Clec16a	7E-01	0.1	Clmp	4E-07	0.3	Zbtb37	7E-01	0.3
Pcdh11x	3E-10	0.1	Neu1	5E-01	0.1	Efr3b	5E-01	0.3	Prune1	7E-01	0.3
Tdg	2E-01	0.1	Pdha1	4E-02	0.1	Dram2	8E-02	0.3	Vps11	5E-01	0.3
S100a6	5E-05	0.1	Flrt1	4E-01	0.1	Zfp775	8E-01	0.3	Mpv17	2E-02	0.3
Asl	6E-01	0.1	Psma3	6E-12	0.1	Mat2a	1E-07	0.3	Eci2	7E-06	0.3
Mul1	7E-01	0.1	Zfp36	3E-01	0.1	Prickle1	5E-04	0.3	Ankrd12	2E-07	0.3
Cd63	7E-30	0.1	Hps1	2E-01	0.1	D830050J10Rik	1E-01	0.3	Tex9	9E-01	0.3
Arl2	8E-03	0.1	Vps52	2E-01	0.1	Exoc3l4	9E-01	0.3	Zfp867	4E-01	0.3
Rfxank	5E-01	0.1	Gtf2b	1E+00	0.1	Zfp467	6E-01	0.3	Ostm1	5E-01	0.3
Ier5	7E-01	0.1	Msto1	9E-01	0.1	Nme4	5E-04	0.3	Elac1	7E-01	0.3
1500004A13Rik	4E-01	0.1	H3f3a	9E-25	0.1	Zfp346	4E-01	0.3	Rogdi	7E-01	0.3
Ltbp3	8E-05	0.1	Ecsit	9E-01	0.1	Slx1b	6E-01	0.3	Camk1d	2E-05	0.3
Wdr44	4E-01	0.1	Zfp64	9E-01	0.1	Lrp1	1E-21	0.3	Mgat4b	3E-01	0.3
Paqr7	2E-01	0.1	Psmb3	6E-05	0.1	Kctd2	5E-02	0.3	A430033K04Rik	8E-01	0.3
Mmgt2	9E-01	0.1	Pfdn1	3E-07	0.1	mt-Nd2	1E-27	0.3	mt-Co1	1E-48	0.3
Pnkd	9E-04	0.1	Akr7a5	9E-03	0.1	Cd164	5E-08	0.3	Apbb1	8E-01	0.3
Igdcc4	2E-01	0.1	Ttc4	8E-01	0.1	Slc27a3	1E+00	0.3	Eif4ebp2	8E-02	0.3
Mgat4b	1E-02	0.1	Tusc2	4E-01	0.1	Pla2g6	5E-01	0.3	Elovl6	2E-02	0.3
Pus3	6E-02	0.1	Abhd12	2E-01	0.1	Aida	5E-06	0.3	B3gntl1	1E+00	0.3
Gm16536	9E-01	0.1	Ptpn4	4E-01	0.1	Fam185a	9E-01	0.3	Ankrd55	8E-01	0.3
Ube2d1	7E-01	0.1	Kxd1	4E-02	0.1	Sirt5	7E-01	0.3	Zmiz2	6E-01	0.3
Esco1	5E-01	0.1	Ccdc22	2E-01	0.1	4732440D04Rik	7E-01	0.3	Spns1	4E-01	0.3
Aspscr1	7E-01	0.1	Ubc	9E-08	0.1	Ttc8	2E-01	0.3	Inf2	6E-01	0.3
Phpt1	3E-01	0.1	Zfp369	4E-01	0.1	Mcoln1	4E-01	0.3	Zfp992	8E-01	0.3
Gm5165	5E-01	0.1	Klhdc2	3E-05	0.1	Nhej1	7E-01	0.3	Magee1	1E+00	0.3
Ccpg1	5E-03	0.1	Slc16a1	1E-01	0.1	Macrod1	4E-06	0.3	Foxo3	1E-01	0.3

Limb Mesenchyme

Control Gene	p	FC	NICD2 Gene	p	FC
Speg	2E-04	0.3	B4galt7	5E-07	0.3
Vezf1	1E-13	0.3	Noc2l	4E-09	0.3
Plagl1	2E-12	0.3	Rrp8	8E-10	0.3
Nub1	2E-12	0.3	Utp25	2E-04	0.3
Dus4l	8E-04	0.3	Kctd5	2E-06	0.3
Zc2hc1a	1E-14	0.3	2810410L24Rik	2E-03	0.3
Glce	2E-08	0.3	Col9a2	2E-04	0.3
Tmbim1	2E-07	0.3	Ppp4r3a	2E-07	0.3
Taf2	3E-09	0.3	Tet2	5E-05	0.3
Tgfbr2	2E-11	0.3	Stk40	1E-06	0.3
Ppp1r15a	7E-15	0.3	Serpine2	4E-17	0.3
2700081O15Rik	5E-09	0.3	Akap11	1E-10	0.3
Kansl2	4E-14	0.3	Ddr1	6E-06	0.3
Rrp9	3E-07	0.3	Bmpr1a	6E-18	0.3
Shoc2	4E-14	0.3	Kif16b	2E-07	0.3
Mterf3	2E-11	0.3	Farsb	4E-06	0.3
Ankrd11	4E-23	0.3	Dop1b	1E-06	0.3
Adcy2	5E-11	0.3	Rab23	6E-07	0.3
Per2	2E-03	0.3	Gpr180	1E-10	0.3
Ice2	1E-05	0.3	Nr6a1os	8E-05	0.3
Acbd3	2E-20	0.3	Mib1	6E-12	0.3
Sars2	4E-03	0.3	Sbk1	1E-02	0.3
Etv1	6E-04	0.3	Zfp384	4E-05	0.3
Jpx	1E-07	0.3	Nfkb1	6E-09	0.3
Zc3h12c	6E-05	0.3	Narf	2E-08	0.3
Pggt1b	2E-10	0.3	Herpud2	2E-08	0.3
Tfg	2E-13	0.3	Uba5	6E-12	0.3
Anks3	4E-07	0.3	Mapk8	4E-07	0.3
Gmps	1E-14	0.3	Zyg11b	6E-09	0.3
Akt1	1E-12	0.3	Eno1	7E-18	0.3
Pdap1	7E-29	0.3	Zfx	1E-08	0.3
Dhx37	4E-06	0.3	Gm39469	5E-04	0.3
Anapc4	3E-13	0.3	Snx18	3E-08	0.3
Arf4	1E-41	0.3	Sars2	2E-02	0.3
Hivep1	1E-04	0.3	Slc30a9	1E-09	0.3
Gpn3	2E-06	0.3	Srp54a	3E-06	0.3
Sars	5E-27	0.3	BC005537	2E-09	0.3
Wdr1	3E-10	0.3	Znhit6	4E-09	0.3
Slc39a13	2E-12	0.3	Ap1g1	9E-10	0.3
Rcn3	7E-31	0.3	Ranbp6	3E-04	0.3
Rnf123	4E-06	0.3	Pdap1	3E-24	0.3
Ogt	6E-15	0.3	Tnfrsf12a	2E-13	0.3
Senp5	7E-11	0.3	Aptx	3E-07	0.3
Klhl22	7E-11	0.3	Ip6k2	2E-07	0.3
Sft2d2	2E-07	0.3	Atxn2	8E-14	0.3
Ubr5	6E-16	0.3	Ppme1	1E-06	0.3
Fam110b	5E-09	0.3	Nle1	6E-05	0.3
Ftsj1	1E-05	0.3	a	5E-05	0.3
Pdxdc1	3E-16	0.3	Adsl	2E-06	0.3
Ireb2	3E-09	0.3	Pdlim4	3E-11	0.3
Clasp2	3E-09	0.3	Gm5141	2E-02	0.3
AI506816	1E-14	0.3	Trim41	7E-09	0.3
Mlxip	9E-07	0.3	Axin1	7E-07	0.3
Col11a2	4E-26	0.3	Pdrg1	1E-12	0.3
Qsox2	1E-05	0.3	Zfp383	4E-03	0.3
Skap2	2E-11	0.3	Nab1	4E-06	0.3
Nat10	1E-06	0.3	Zfc3h1	4E-11	0.3
Chsy3	5E-11	0.3	Tln1	3E-22	0.3
Khdrbs1	1E-20	0.3	Peak1	3E-12	0.3
Slc35d1	4E-07	0.3	Pigv	6E-07	0.3
Wdr11	4E-07	0.3	Kat6b	1E-11	0.3
Thumpd3	1E-06	0.3	Nras	1E-08	0.3
Slc30a9	2E-08	0.3	Ogt	3E-10	0.3
Mettl1	4E-15	0.3	Gpsm1	6E-05	0.3
Mosmo	5E-10	0.3	Blmh	1E-09	0.3
Dcaf10	3E-09	0.3	Sirt6	1E-04	0.3
Pus7	1E-06	0.3	Gpatch4	2E-08	0.3
Timm8a1	3E-09	0.3	Ibtk	2E-09	0.3
Ptpn9	5E-12	0.3	Ostf1	5E-16	0.3
Bnip2	5E-21	0.3	Per2	3E-05	0.3
E2f3	4E-09	0.3	Exoc5	5E-09	0.3
Pds5a	5E-13	0.3	Rbm48	6E-06	0.3
2310014F06Rik	2E-06	0.3	Nradd	2E-07	0.3

Chondrogenic

Control Gene	p	FC	NICD2 Gene	p	FC
Sgcd	1E-17	0.3	Rpl39l	1E-01	0.3
BC031181	1E-29	0.3	Tle3	2E-01	0.3
2010320M18Rik	1E-05	0.3	Zfyve9	2E-07	0.3
Casd1	5E-07	0.3	Spin2c	3E-01	0.3
Clcc1	1E-06	0.3	Ppp1r37	3E-02	0.3
Taco1os	1E-01	0.3	Tet3	1E-02	0.3
Dele1	6E-03	0.3	Tmem220	6E-01	0.3
Alkbh6	1E-04	0.3	Ppt1	7E-10	0.3
Usp3	3E-13	0.3	Stx6	6E-03	0.3
Pdk1	5E-04	0.3	Herc1	3E-06	0.3
Spaca6	3E-08	0.3	E130114P18Rik	3E-01	0.3
Fam120c	1E-06	0.3	4921511C10Rik	4E-01	0.3
Oard1	1E-05	0.3	Arhgef18	4E-04	0.3
Ttc13	1E-04	0.3	Nfia	9E-25	0.3
Agrn	6E-06	0.3	Galc	3E-02	0.3
Ints6l	8E-02	0.3	Ddx5	4E-47	0.3
Guk1	7E-18	0.3	Btd	2E-03	0.3
Hoxc10	9E-14	0.3	1700037C18Rik	1E-01	0.3
Dok1	2E-01	0.3	Cmss1	6E-27	0.3
Akap7	4E-06	0.3	2200002D01Rik	1E-03	0.3
Gramd1b	5E-02	0.3	Ubxn11	4E-01	0.3
Rmnd5a	5E-06	0.3	Nr1d2	1E-06	0.3
Fzd1	3E-04	0.3	Gtf2ird2	3E-02	0.3
Slc35a2	5E-07	0.3	Yipf4	1E-18	0.3
Yrdc	2E-04	0.3	Gm26782	4E-06	0.3
Cyp2u1	2E-01	0.3	Snx11	8E-02	0.3
Tmtc2	6E-05	0.3	Hist1h2bc	2E-26	0.3
Sfi1	5E-03	0.3	Prkn	2E-08	0.3
Ankrd44	5E-05	0.3	4930556J24Rik	1E-01	0.3
0610009L18Rik	2E-01	0.3	3110040N11Rik	2E-05	0.3
Gm49539	1E-01	0.3	Prdm11	6E-02	0.3
Zfp58	4E-01	0.3	Ramac	1E-09	0.3
Bbs9	3E-05	0.3	Prpf40b	2E-01	0.3
Fnta	2E-18	0.3	Slc10a3	2E-01	0.3
Eps8	2E-06	0.3	Dcp1b	9E-02	0.3
Styx	2E-02	0.3	Kcnq1ot1	2E-10	0.3
Zfp976	5E-03	0.3	Slc35a5	8E-03	0.3
1810013L24Rik	9E-07	0.3	Cyren	7E-01	0.3
Pigp	1E-22	0.3	Pcmtd2	8E-06	0.3
Ddx31	3E-02	0.3	Gm15738	9E-02	0.3
Cbx1	1E-30	0.3	mt-Atp8	4E-11	0.3
Ubxn7	8E-08	0.3	Usp3	3E-08	0.3
Slc35e1	8E-02	0.3	A430018G15Rik	2E-01	0.3
Lman2l	8E-04	0.3	Sept1	9E-02	0.3
Itfg2	1E-02	0.3	Tmem106c	1E-04	0.3
Pex7	4E-08	0.3	Cfap300	4E-01	0.3
Ccdc57	2E-01	0.3	Btf3l4	6E-04	0.3
Tbc1d10a	4E-02	0.3	Ccser2	3E-16	0.3
Cwc22	7E-07	0.3	Cog6	1E-03	0.3
Gspt2	3E-02	0.3	Smim14	4E-17	0.3
Nme6	3E-02	0.3	Ccpg1	3E-09	0.3
Foxp2	1E-15	0.3	Med13	5E-10	0.3
Actr8	3E-04	0.3	Ltv1	4E-04	0.3
Dtnb	3E-04	0.3	4732471J01Rik	3E-02	0.3
Zfp316	1E-01	0.3	Clcn5	3E-04	0.3
Tmem176b	1E-26	0.3	Zfp788	8E-02	0.3
Nck2	1E-04	0.3	Ss18l2	8E-06	0.3
Mmachc	5E-02	0.3	Dgkd	2E-02	0.3
Dnhd1	3E-01	0.3	Nrep	1E-03	0.3
P3h3	7E-20	0.3	Ciapin1	1E-02	0.3
Arhgef18	2E-04	0.3	Gtf2i	8E-14	0.3
Ip6k2	1E-05	0.3	Fnta	4E-13	0.3
Rrp12	8E-02	0.3	Pde4b	8E-04	0.3
Bcas3	1E-09	0.3	Slc9a6	1E-01	0.3
Mbtps1	6E-12	0.3	1110002L01Rik	2E-01	0.3
Gin1	1E-02	0.3	Hist1h4i	1E-01	0.3
Zkscan1	2E-09	0.3	Traf6	4E-03	0.3
Ttc26	7E-02	0.3	Trmt11	7E-03	0.3
Tmtc1	6E-05	0.3	Ptpa	1E-09	0.3
Trappc10	3E-08	0.3	Loxl3	3E-02	0.3
Crtc3	5E-07	0.3	Ccdc47	2E-09	0.3
Itpr3	3E-03	0.3	Gm26917	5E-01	0.3
B830012L14Rik	3E-05	0.3	1700109K24Rik	3E-02	0.3

Fibroblast

Control Gene	p	FC	NICD2 Gene	p	FC
Mtx3	1E-03	0.2	Bcl2l2	8E-03	0.2
Usp22	5E-15	0.2	Sec23ip	2E-07	0.2
Ppp1r2	2E-22	0.2	Rpl18a	1E-109	0.2
Stk40	3E-09	0.2	Kctd17	1E-06	0.2
Dnajc13	3E-21	0.2	Zfp9	1E-07	0.2
Klhl26	8E-09	0.2	Atp9a	1E-02	0.2
Bmpr1b	9E-02	0.2	Fam219a	1E-04	0.2
Tceal8	1E-44	0.2	Rpl39	6E-142	0.2
Rpl37a	4E-283	0.2	Tsta3	3E-04	0.2
B2m	1E-116	0.2	Kank2	7E-11	0.2
Calr	7E-62	0.2	Map2k6	6E-02	0.2
Ttc21b	2E-10	0.2	Sft2d1	3E-05	0.2
Rpl12	9E-139	0.2	Gatd1	7E-06	0.2
Uba52	7E-82	0.2	Mob3a	9E-03	0.2
Sept11	6E-62	0.2	Spats2l	7E-13	0.2
Tmem151a	5E-06	0.2	Atf7	4E-11	0.2
Ccl27a	8E-06	0.2	Pip4k2b	6E-05	0.2
Ndufa3	3E-68	0.2	Klhl36	2E-01	0.2
Vasp	2E-13	0.2	Rpl31	9E-89	0.2
Tsta3	4E-08	0.2	Spag9	6E-31	0.2
Gm14305	1E-06	0.2	Gtpbp3	6E-02	0.2
Csgalnact1	2E-27	0.2	Fkbp11	3E-24	0.2
Dnhd1	1E-02	0.2	Coa5	5E-06	0.2
Esyt1	9E-11	0.2	Arsg	9E-02	0.2
Xkr6	2E-03	0.2	Stard4	5E-02	0.2
Gm8739	7E-01	0.2	Irak2	3E-03	0.2
Man2b1	3E-16	0.2	2310015A10Rik	2E-01	0.2
Lmbr1	7E-06	0.2	Bet1l	1E-20	0.2
Mif	8E-73	0.2	Gas1	3E-32	0.2
Fbxo32	2E-06	0.2	Sh3pxd2a	1E-19	0.2
Evi5l	2E-04	0.2	Ppp2cb	9E-08	0.2
Ostm1	4E-06	0.2	Bcl9l	3E-12	0.2
D730003I15Rik	2E-03	0.2	Arap2	2E-01	0.2
Rgs19	5E-09	0.2	Angptl1	6E-01	0.2
Mttp	5E-03	0.2	Mtx3	1E-01	0.2
Pigh	4E-04	0.2	Tmem205	1E-19	0.2
Tmem41a	4E-06	0.2	Zfp697	3E-03	0.2
Mvk	3E-10	0.2	Ift27	6E-15	0.2
Tcta	7E-12	0.2	Pcdhb17	9E-02	0.2
Rps15a	2E-249	0.2	Zswim6	2E-09	0.2
Dhx33	3E-04	0.2	Zfp57	9E-02	0.2
Fas	2E-05	0.2	Ctsz	1E-36	0.2
Adamts10	4E-10	0.2	Litaf	1E-11	0.2
Atf7	3E-20	0.2	Acaa2	9E-16	0.2
Notch1	2E-04	0.2	Oaf	2E-09	0.2
Snhg15	6E-10	0.2	Rpl37a	1E-139	0.2
Zfp652	3E-20	0.2	Cept1	6E-07	0.2
Bcl2l2	2E-04	0.2	Tsc22d4	6E-25	0.2
Vwa5a	2E-25	0.2	Hes1	1E-07	0.2
Klf13	4E-12	0.2	Fpgt	3E-02	0.2
Ripor1	2E-06	0.2	Arhgap21	2E-08	0.2
Tsga10	8E-04	0.2	Acad9	5E-03	0.2
Slc46a1	8E-04	0.2	Tmem126b	3E-05	0.2
Rxra	5E-09	0.2	Usp25	8E-11	0.2
Cldnd1	4E-26	0.2	Atg2b	5E-03	0.2
Gga2	8E-17	0.2	Tnks2	3E-19	0.2
Rpl37	4E-223	0.2	Zfp251	3E-03	0.2
Insyn2a	3E-03	0.2	Capn5	1E-01	0.2
Esyt2	7E-08	0.2	E430024I08Rik	2E-04	0.2
Tmem256	5E-64	0.2	Appl2	7E-07	0.2
Gm26802	4E-02	0.2	Ada	2E-04	0.2
Rpsa	1E-170	0.2	Nr1d1	3E-03	0.2
Fam8a1	5E-11	0.2	Erbin	4E-16	0.2
Cers4	6E-09	0.2	Ccng2	1E-03	0.2
Asah1	2E-50	0.2	Rpl12	5E-68	0.2
Syde1	1E-08	0.2	Scd2	1E-19	0.2
Nfil3	3E-10	0.2	Fgfrl1	6E-05	0.2
Rpl32	1E-241	0.2	Ggta1	5E-03	0.2
Cstb	2E-87	0.2	Gm10373	3E-01	0.2
Tsc22d4	5E-45	0.2	Rpl34	6E-114	0.2
Rpl9	6E-219	0.2	Morc4	2E-05	0.2
Impact	6E-28	0.2	Ndufa3	4E-31	0.2
Tgfbi	2E-19	0.2	Rraga	2E-19	0.2

Fibroblast?

Control Gene	p	FC	NICD2 Gene	p	FC
Abraxas1	3E-25	0.7	Ccng1	2E-25	0.6
Sf3a3	2E-45	0.7	4833427G06Rik	1E-14	0.6
Fam76b	2E-36	0.7	Wdr66	2E-17	0.6
Azin1	2E-37	0.7	Exog	2E-30	0.6
Ercc2	1E-04	0.7	Mdga1	4E-40	0.6
Gm49692	3E-13	0.7	Stard7	3E-35	0.6
Lca5	4E-23	0.7	Mettl9	1E-81	0.6
Ing1	1E-42	0.7	Mcub	5E-37	0.6
Ube2l6	1E-18	0.7	Tmem232	3E-12	0.6
Ucp2	2E-21	0.7	Ruvbl2	5E-46	0.6
Srsf4	1E-37	0.7	Tgfb1	3E-51	0.6
Ptchd4	9E-17	0.7	Cdc123	2E-70	0.6
Mtrr	3E-18	0.7	Gm20125	2E-17	0.6
Slc39a8	2E-12	0.7	Zdhhc16	2E-38	0.6
Radil	4E-14	0.7	Dcps	2E-37	0.6
Gm15581	7E-19	0.7	Susd5	9E-17	0.6
Slc10a7	2E-30	0.7	Lrrcc1	3E-43	0.6
Mfsd6	2E-13	0.7	Alg6	1E-38	0.6
Ubap2	1E-57	0.7	Tcerg1	3E-46	0.6
Lpcat1	5E-22	0.7	Cisd2	9E-90	0.6
2810006K23Rik	2E-31	0.7	Nt5c3b	5E-46	0.6
Taf1d	1E-52	0.7	1700037H04Rik	1E-39	0.6
Elf4	1E-37	0.7	Asap2	5E-45	0.6
Gm13986	4E-17	0.7	A430018G15Rik	1E-05	0.6
Med30	1E-41	0.7	Snrpe	4E-200	0.6
Slc20a2	3E-27	0.7	Tyw1	4E-40	0.6
Med9	5E-32	0.7	Slc27a3	2E-38	0.6
Ttl	9E-24	0.7	Oscp1	2E-20	0.6
Ncs1	2E-20	0.7	Eif2s1	4E-61	0.6
S100a6	2E-81	0.7	Snrpb	8E-128	0.6
Gm4890	4E-16	0.7	Tctn3	3E-18	0.6
1700017B05Rik	3E-24	0.6	Tmx1	8E-60	0.6
Bmpr1b	7E-28	0.6	Pxylp1	3E-27	0.6
Vars	5E-36	0.6	Primpol	2E-37	0.6
Ano3	9E-26	0.6	Gm13427	7E-14	0.6
Tmem109	2E-39	0.6	Map6	4E-31	0.6
Snapc2	3E-26	0.6	Gna11	5E-76	0.6
Gm49890	5E-07	0.6	Eefsec	3E-34	0.6
Ly75	1E-11	0.6	Ugdh	3E-57	0.6
Cacybp	1E-77	0.6	Glipr2	9E-46	0.6
Atr	1E-26	0.6	Actg1	2E-92	0.6
Asf1a	2E-40	0.6	Cd109	7E-50	0.6
Hdgfl2	2E-29	0.6	Pds5b	1E-51	0.6
Tcerg1	1E-37	0.6	Gfer	8E-46	0.6
Nxph4	1E-13	0.6	Mpi	2E-19	0.6
2610307P16Rik	2E-33	0.6	Mettl15	2E-33	0.6
Gtf3c5	2E-20	0.6	Ppp1r8	7E-36	0.6
Gmps	2E-47	0.6	Syne3	2E-46	0.6
S100a10	4E-78	0.6	Qrsl1	1E-21	0.6
Nt5c3b	4E-36	0.6	Sema6a	8E-06	0.6
Arhgap10	5E-40	0.6	Ppp5c	1E-33	0.6
Phf5a	2E-60	0.6	Hes1	1E-53	0.6
Inf2	1E-33	0.6	Sgms2	2E-31	0.6
Hint1	2E-179	0.6	Tarbp2	5E-25	0.6
Ccdc3	3E-10	0.6	Utp14b	6E-11	0.6
Tbl3	4E-24	0.6	Gm11457	1E-11	0.6
Dcbld2	2E-30	0.6	Nme1	1E-118	0.6
Helb	3E-29	0.6	Rbmxl1	9E-49	0.6
Cavin4	7E-16	0.6	Net1	9E-51	0.6
C2cd2	5E-20	0.6	Rdh11	8E-31	0.6
Maz	4E-37	0.6	Apol6	9E-16	0.6
Slc27a3	3E-25	0.6	9330159M07Rik	1E-26	0.6
1700037H04Rik	6E-30	0.6	Rtl3	1E-40	0.6
Btbd8	4E-21	0.6	Gm10138	2E-13	0.6
Arhgef26	8E-20	0.6	Dnajb1	2E-39	0.6
Gm28875	6E-25	0.6	Med9	9E-41	0.6
Alad	4E-30	0.6	S100a4	2E-40	0.6
Tyw1	8E-30	0.6	Ptgr1	5E-28	0.6
Ahctf1	8E-36	0.6	H2afy2	3E-42	0.6
Nxt2	3E-20	0.6	Rhbdl3	6E-20	0.6
Tfap4	4E-13	0.6	Sgk3	1E-42	0.6
Atl2	1E-32	0.6	Lin52	2E-39	0.6
Mrpl28	1E-57	0.6	Adamts14	4E-13	0.6

Undefined

Control Gene	p	FC	NICD2 Gene	p	FC
Kansl1l	2E-01	0.1	Zfp629	1E-02	0.1
Commd6	6E-03	0.1	Chmp2b	5E-01	0.1
Oaz2	1E-02	0.1	Rpap2	2E-01	0.1
Trnt1	1E-01	0.1	Zhx3	8E-01	0.1
Fundc1	7E-02	0.1	Ndufa7	2E-09	0.1
Fkrp	4E-01	0.1	E530011L22Rik	2E-01	0.1
Vldlr	2E-01	0.1	Stc1	1E+00	0.1
Ptcd1	7E-01	0.1	Pfkfb2	6E-01	0.1
Gm10353	1E+00	0.1	Svep1	2E-02	0.1
Tmem39b	4E-01	0.1	Chic1	4E-01	0.1
Rab23	1E+00	0.1	Anxa7	3E-02	0.1
Mink1	8E-01	0.1	Fgd1	1E-01	0.1
Uqcc1	3E-01	0.1	Ube2h	8E-05	0.1
Ppp1r11	3E-04	0.1	Smg8	6E-01	0.1
Ankrd46	7E-01	0.1	Atp6v1d	5E-02	0.1
Sympk	5E-01	0.1	Naxe	8E-03	0.1
Rufy2	1E+00	0.1	Bcl7a	6E-02	0.1
Xrcc4	7E-02	0.1	Gm17106	8E-01	0.1
Gpatch4	7E-01	0.1	BC031181	1E-06	0.1
Tsr1	7E-01	0.1	2310033P09Rik	7E-01	0.1
Isg20	4E-01	0.1	Avpi1	6E-01	0.1
Gm44686	4E-01	0.1	Cnpy3	3E-01	0.1
Adck2	1E+00	0.1	Tns2	1E-01	0.1
Marveld1	8E-01	0.1	Klhdc8a	5E-01	0.1
Faap100	7E-01	0.1	Kremen1	8E-01	0.1
Nfil3	8E-01	0.1	Rnf114	6E-01	0.1
Galnt4	8E-01	0.1	Tanc1	8E-01	0.1
Dlst	3E-01	0.1	Parp8	1E-02	0.1
1190005I06Rik	1E-01	0.1	Dedd	2E-01	0.1
Arhgap23	9E-01	0.1	Adrm1	3E-01	0.1
Atp6v1a	4E-05	0.1	Zfp647	6E-01	0.1
Mesd	6E-08	0.1	Dhx8	2E-01	0.1
Abhd14a	1E-02	0.1	Sgcb	4E-02	0.1
Eif2b2	2E-01	0.1	Ndst2	1E+00	0.1
Nip7	5E-02	0.1	Tmem59	1E-12	0.1
Zfp846	1E-01	0.1	Rbm19	3E-01	0.1
Mta2	9E-01	0.1	Zfp593	2E-01	0.1
Actr5	4E-01	0.1	Mettl21a	6E-01	0.1
Hint2	5E-04	0.1	Wdr33	5E-03	0.1
B9d2	9E-01	0.1	Ehmt1	2E-02	0.1
Haghl	2E-01	0.1	Phpt1	7E-01	0.1
Tnfaip8	1E-02	0.1	Anapc7	4E-01	0.1
Syf2	6E-02	0.1	Nfil3	3E-01	0.1
Mpp2	7E-01	0.1	Smtn	1E-01	0.1
Vps11	4E-01	0.1	Nol9	2E-01	0.1
Nbr1	1E-01	0.1	Med17	8E-01	0.1
Ptma	4E-32	0.1	Ebag9	2E-01	0.1
Uckl1	1E+00	0.1	Zfand5	1E-05	0.1
Zc3h10	6E-01	0.1	Nop16	7E-01	0.1
Zfp579	5E-01	0.1	Atf6b	4E-01	0.1
Dnal4	8E-01	0.1	Arhgef10l	8E-01	0.1
Jkamp	1E-02	0.1	Adgra3	6E-02	0.1
Zfp444	9E-01	0.1	Nr4a2	6E-01	0.1
Slc30a6	8E-01	0.1	Fam192a	4E-01	0.1
Nol11	1E-01	0.1	Gtpbp2	6E-01	0.1
Tmem17	6E-01	0.1	Cobll1	1E-01	0.1
Lmbrd1	1E-02	0.1	BC051226	3E-02	0.1
Selenow	2E-09	0.1	Tmem65	4E-02	0.1
Meis2	3E-01	0.1	Frg1	4E-02	0.1
Ythdf1	3E-01	0.1	Sdhd	1E+00	0.1
Desi2	4E-08	0.1	Sdr39u1	9E-01	0.1
Cox20	1E-05	0.1	Rnf122	9E-01	0.1
Zfyve27	5E-01	0.1	Sdf2	5E-06	0.1
Ramac	7E-03	0.1	Snrpb2	6E-02	0.1
Cep95	9E-01	0.1	Gm47863	1E-02	0.1
Gbe1	8E-01	0.1	Atg10	3E-02	0.1
Zkscan6	5E-01	0.1	Slc38a9	8E-01	0.1
Ube4b	5E-03	0.1	Ak4	7E-01	0.1
Slfn8	5E-01	0.1	Spryd3	3E-01	0.1
Kifc3	9E-01	0.1	Nfs1	7E-01	0.1
Dnajc4	6E-01	0.1	Uvssa	1E-01	0.1
Slc35e2	6E-01	0.1	Pgrmc1	8E-03	0.1
Rnh1	3E-08	0.1	Ppp2r3c	2E-01	0.1

Articular/Synovial Fibroblast

Control Gene	p	FC	NICD2 Gene	p	FC
Wbp2	4E-03	0.3	Bscl2	1E-06	0.3
A930001C03Rik	3E-01	0.3	Mfsd1	1E-02	0.3
Mocs3	9E-01	0.3	Eif3e	8E-15	0.3
Junb	3E-05	0.3	Kif3a	4E-02	0.3
Exoc7	3E-02	0.3	Cpsf4	1E-01	0.3
Exoc1	6E-01	0.3	Tmem53	3E-01	0.3
Scrn3	5E-01	0.3	Atraid	4E-13	0.3
Mfsd3	8E-01	0.3	Tacc1	3E-06	0.3
Cobll1	1E-02	0.3	Ccdc22	6E-01	0.3
Fars2	4E-04	0.3	Ttf1	5E-02	0.3
Zrsr1	5E-01	0.3	Mau2	2E-01	0.3
4930557K07Rik	6E-01	0.3	Zfp398	4E-01	0.3
Faxc	1E+00	0.3	Gk5	3E-01	0.3
Prkab2	8E-01	0.3	Il4ra	3E-01	0.3
Rnf114	2E-01	0.3	Syngap1	8E-01	0.3
Stx3	4E-01	0.3	Dmap1	7E-01	0.3
Wdfy1	4E-01	0.3	Als2	6E-01	0.3
Scarb1	4E-01	0.3	Cyp2j6	2E-02	0.3
Msrb2	4E-01	0.3	Uqcc3	2E-08	0.3
Eif4e3	8E-01	0.3	Src	7E-01	0.3
Shld1	4E-01	0.3	Fbxl15	1E+00	0.3
Zc3h6	5E-02	0.3	Gabarap	2E-30	0.3
St6galnac6	3E-02	0.3	Fam151b	7E-01	0.3
Nt5dc3	5E-01	0.3	Adi1	4E-01	0.3
Idua	9E-01	0.3	Cd1d1	4E-02	0.3
Mgat1	4E-02	0.3	Cd59b	6E-01	0.3
Ccdc122	5E-02	0.3	Ptprf	9E-02	0.3
Pigh	8E-01	0.3	Depdc5	9E-02	0.3
Notch1	8E-01	0.3	Ggnbp1	7E-01	0.3
Tecpr1	3E-01	0.3	Wnk3	4E-01	0.3
Prkca	3E-09	0.3	Junb	2E-04	0.3
Sec24c	2E-01	0.3	Exoc7	3E-02	0.3
Lrig2	1E-01	0.3	Gm37494	8E-02	0.3
Tti2	8E-01	0.3	Arhgef12	2E-05	0.3
Gm14410	6E-01	0.3	Sfxn4	1E+00	0.3
Galnt4	6E-01	0.3	Exosc7	1E-02	0.3
Tmem131l	5E-01	0.3	Dhx34	9E-01	0.3
Homer3	2E-02	0.3	Pptc7	3E-01	0.3
Nfrkb	7E-01	0.3	Pvt1	2E-05	0.3
4930517O19Rik	7E-01	0.3	Cxcl16	4E-01	0.3
D230025D16Rik	1E+00	0.3	Pet100	1E-05	0.3
Synrg	6E-03	0.3	Pik3c3	6E-01	0.3
Bmp2k	5E-01	0.3	Dyrk2	7E-01	0.3
Mgst3	4E-12	0.3	Ttc8	3E-01	0.3
Efhd2	2E-02	0.3	Mindy4	4E-01	0.3
Stat6	2E-02	0.3	L2hgdh	7E-01	0.3
Ube2e2	1E-08	0.3	Myo7a	9E-02	0.3
Tmem43	3E-01	0.3	Fbxo36	3E-01	0.3
Hadhb	1E-03	0.3	Mxd4	2E-11	0.3
S1pr1	5E-01	0.3	Hscb	4E-01	0.3
Daglb	5E-01	0.3	Plcg1	4E-01	0.3
Sugp1	4E-01	0.3	Sgta	1E-03	0.3
Ftl1	5E-38	0.3	Rps6kc1	5E-01	0.3
Vps4b	3E-05	0.3	2810032G03Rik	5E-01	0.3
Mtmr10	7E-01	0.3	Dyrk1b	6E-01	0.3
Tspyl2	2E-01	0.3	Armcx1	1E-01	0.3
Cd59b	8E-01	0.3	Sirt3	8E-01	0.3
Trak1	3E-01	0.3	Trim21	1E+00	0.3
Rundc1	8E-01	0.3	Pex1	1E+00	0.3
Antxr1	8E-09	0.3	Enpp2	6E-02	0.3
Isca1	4E-01	0.3	Pou6f1	8E-01	0.3
Pip4p1	6E-03	0.3	Crat	5E-01	0.3
Rbpj	3E-10	0.3	Wdr25	8E-01	0.3
Bcorl1	7E-01	0.3	Trim25	9E-01	0.3
Stx18	5E-04	0.3	mt-Atp8	1E-03	0.3
Nit1	1E-01	0.3	Gm14326	4E-01	0.3
Lrp11	5E-01	0.3	Tecpr1	8E-01	0.3
Zfat	9E-01	0.3	Anks1	7E-01	0.3
Stk24	2E-03	0.3	Washc1	6E-01	0.3
Fam117a	9E-01	0.3	Ift140	9E-01	0.3
Abca3	7E-01	0.3	9330159M07Rik	8E-01	0.3
Vegfa	7E-01	0.3	Aff1	2E-03	0.3
Osbpl7	5E-01	0.3	Gm47071	6E-01	0.3

Limb Mesenchyme

Control			NICD2		
Gene	*p*	FC	Gene	*p*	FC
Abcd3	8E-10	0.3	Sh3rf3	1E-09	0.3
Mrs2	2E-06	0.3	Pabpc4	2E-12	0.3
Mia	4E-55	0.3	Rnf19a	2E-04	0.3
Tulp3	8E-10	0.3	Sun2	2E-05	0.3
Dym	5E-10	0.3	Gm13402	3E-03	0.3
Sox11	2E-03	0.3	Flna	2E-15	0.3
Spata5	2E-13	0.3	Cttnbp2nl	3E-09	0.3
Eif2s3x	2E-09	0.3	Taok1	1E-13	0.3
Atp6v0a2	1E-07	0.3	Med19	2E-08	0.3
Lrig3	4E-15	0.3	Tmed9	4E-30	0.3
Lzic	2E-09	0.3	Zbtb39	2E-03	0.3
Paip1	3E-14	0.3	Lims1	5E-18	0.3
Shmt2	7E-08	0.3	4931414P19Rik	2E-03	0.3
Fiz1	5E-07	0.3	Rcan2	4E-04	0.3
Tyw3	7E-04	0.3	Letmd1	3E-04	0.3
Fam102a	4E-04	0.3	Vhl	3E-03	0.3
Mfge8	3E-17	0.3	Dlg2	8E-08	0.3
Polr2m	3E-17	0.3	Pak1ip1	4E-12	0.3
Morn1	3E-03	0.3	Ppp1r12c	1E-05	0.3
B230217C12Rik	4E-04	0.3	Grk5	1E-05	0.3
Nt5c2	4E-09	0.3	Ccdc181	2E-05	0.3
Gnb5	6E-08	0.3	Rab5a	2E-13	0.3
Axin2	3E-04	0.3	Psme3	4E-07	0.3
Bnc2	1E-09	0.3	Snhg18	6E-26	0.3
Tmem39a	6E-10	0.3	Taf4	3E-03	0.3
Adh7	2E-05	0.3	Phykpl	3E-07	0.3
Wdr6	3E-11	0.3	4930512B01Rik	4E-02	0.3
9130023H24Rik	6E-04	0.3	Gfm2	2E-06	0.3
Chst11	1E-08	0.3	Cbx1	3E-22	0.3
Mmadhc	3E-13	0.3	Tbc1d12	8E-09	0.3
Dnajc11	3E-08	0.3	Gga1	3E-03	0.3
Apln	1E-02	0.3	Bap1	8E-05	0.3
H13	7E-15	0.3	Chd1	4E-08	0.3
Parg	1E-10	0.3	Ptpn9	1E-10	0.3
Pdss2	9E-08	0.3	Dixdc1	7E-05	0.3
Luc7l	2E-10	0.3	Prmt3	2E-07	0.3
Nr6a1os	3E-06	0.3	Cnot7	5E-08	0.3
Fyttd1	1E-14	0.3	Klf12	3E-11	0.3
Gm45338	6E-06	0.3	Glrb	5E-02	0.3
Pde4dip	2E-13	0.3	Raf1	4E-07	0.3
Tmem248	7E-08	0.3	Mppe1	5E-06	0.3
Tmem161a	4E-06	0.3	Mtus2	3E-04	0.3
Tram1	3E-15	0.3	Ercc8	3E-04	0.3
Cd276	9E-08	0.3	Exosc1	1E-07	0.3
Zfp777	7E-05	0.3	Trim13	4E-04	0.3
Arcn1	2E-18	0.3	4921524J17Rik	4E-11	0.3
Rccl1	1E-05	0.3	Pigg	5E-04	0.3
Smarcad1	2E-11	0.3	Slc39a11	2E-06	0.3
Zfx	6E-10	0.3	E2f5	1E-06	0.3
Tm9sf2	6E-22	0.3	Wrap53	3E-01	0.3
6030443J06Rik	6E-08	0.3	Urb1	8E-03	0.3
Phc2	2E-07	0.3	Bckdk	4E-10	0.3
Vwa8	1E-09	0.3	Ccdc47	4E-10	0.3
Irx3	4E-06	0.3	Smad2	2E-09	0.3
Rbfox1	1E-05	0.3	Rhobtb2	1E-03	0.3
Slc30a1	4E-06	0.3	Wipf1	4E-06	0.3
Set	5E-36	0.3	Ints8	2E-04	0.3
Plscr1	3E-04	0.3	Vdac3	3E-23	0.3
Pdzrn3	1E-15	0.3	Rnf24	4E-08	0.3
Mmaa	9E-06	0.3	Rgs17	2E-04	0.3
Cct2	2E-29	0.3	Zc3hc1	1E-05	0.3
Tchp	7E-05	0.3	Zdhhc12	3E-07	0.3
Get4	3E-08	0.3	Shoc2	3E-11	0.3
Ep400	1E-07	0.3	Wdfy2	3E-06	0.3
Phka1	3E-08	0.3	Xbp1	6E-10	0.3
Zkscan17	6E-05	0.3	Slc39a7	3E-14	0.3
C1qtnf6	9E-08	0.3	Osbpl2	6E-07	0.3
Kat6b	2E-10	0.3	Slc4a1ap	2E-05	0.3
Noc3l	7E-08	0.3	Telo2	7E-05	0.3
Tlk2	4E-11	0.3	Ubfd1	1E-09	0.3
Lsm12	2E-20	0.3	R3hcc1	1E-06	0.3
Zfp292	2E-11	0.3	Cwc22	2E-07	0.3
Rubcn	2E-05	0.3	Mrps28	2E-10	0.3

Chondrogenic

Control			NICD2		
Gene	*p*	FC	Gene	*p*	FC
Aptx	1E-05	0.3	Abhd17c	1E-02	0.3
Galnt7	1E-03	0.3	Alg2	8E-03	0.3
Itga6	6E-04	0.3	Shc1	2E-09	0.3
Tmed1	4E-14	0.3	Dph7	7E-02	0.3
Sdc3	2E-04	0.3	Cep89	2E-02	0.3
AI597479	1E-02	0.3	Fam107b	7E-02	0.3
Otud6b	6E-06	0.3	Lman2l	2E-02	0.3
Zbtb26	2E-02	0.3	Cep131	1E-01	0.3
Avpi1	5E-11	0.3	Lrrc51	2E-02	0.3
Ttc3	9E-34	0.3	Phkb	2E-08	0.3
Swap70	9E-07	0.3	Ctu2	3E-02	0.3
Lncpint	1E-11	0.3	mt-Nd5	8E-36	0.3
Bbs7	9E-02	0.3	Fhit	5E-05	0.3
Mmaa	2E-03	0.3	Ier2	1E-08	0.3
Chsy3	4E-05	0.3	Nf2	4E-04	0.3
Pura	4E-19	0.3	Ehbp1	5E-09	0.3
Gm26802	3E-02	0.3	Itga8	4E-02	0.3
Pam16	3E-17	0.3	Erap1	5E-02	0.3
Ltbp2	6E-07	0.3	Man2c1os	8E-02	0.3
Gsdme	3E-04	0.3	Parp6	8E-02	0.3
2210408F21Rik	4E-09	0.3	Arl6	1E-02	0.3
Ube2e1	5E-16	0.3	Igip	1E-01	0.3
2610301B20Rik	7E-04	0.3	Zc2hc1a	4E-07	0.3
Ttc32	2E-04	0.3	Cutc	1E-01	0.3
Adal	8E-02	0.3	Pard3bos1	8E-03	0.3
BC051226	8E-02	0.3	Tdrd3	4E-06	0.3
Zfp560	3E-13	0.3	Tvp23b	7E-09	0.3
Trit1	3E-03	0.3	Pfkl	3E-06	0.3
Ybey	3E-01	0.3	Zfp27	2E-01	0.3
Chd1l	2E-01	0.3	Btg3	6E-10	0.3
Gpr27	2E-01	0.3	1700123M08Rik	3E-02	0.3
Zrsr1	8E-03	0.3	Slc37a3	9E-02	0.3
Vps51	9E-02	0.3	Cyb5d2	2E-02	0.3
Tet3	4E-05	0.3	Gls	3E-09	0.3
Sfmbt2	2E-01	0.3	Znhit6	4E-05	0.3
Nphp1	2E-04	0.3	Cyb5rl	1E-01	0.3
Btaf1	3E-12	0.3	Slc39a10	7E-04	0.3
AW146154	3E-02	0.3	Wdfy2	1E-05	0.3
Smim4	2E-09	0.3	St3gal3	1E-03	0.3
Rab36	9E-02	0.3	Spred3	2E-01	0.3
Tnrc18	2E-15	0.3	Crtc3	2E-09	0.3
Hipk1	1E-05	0.3	Bckdk	4E-06	0.3
Hace1	2E-02	0.3	Clic4	4E-15	0.3
Tmem186	2E-01	0.3	Pakap.1	3E-03	0.3
Slc49a4	2E-03	0.3	Ubxn7	6E-04	0.3
Cap2	4E-02	0.3	Gpr27	4E-01	0.3
Trp53rka	7E-02	0.3	Gm42418	3E-38	0.3
Lrrc8d	6E-02	0.3	Mlh3	2E-02	0.3
Spcs2	3E-29	0.3	Phtf1	6E-05	0.3
1190005I06Rik	1E-02	0.3	Zfp952	9E-02	0.3
Gm17529	3E-01	0.3	Stat5a	3E-01	0.3
Tmem167b	4E-04	0.3	G6pc3	7E-05	0.3
Tmem201	5E-02	0.3	Mfge8	7E-13	0.3
Krr1	7E-05	0.3	Mecp2	1E-04	0.3
2700046G09Rik	2E-02	0.3	Klhl7	6E-04	0.3
Ldlrad4	5E-07	0.3	1810062O18Rik	1E-01	0.3
Lrrc20	5E-03	0.3	Gm20300	3E-01	0.3
Syt12	5E-02	0.3	Ttc3	1E-20	0.3
Stard6	2E-01	0.3	Tmem185a	6E-02	0.3
Mpp6	1E-09	0.3	Gm48742	4E-02	0.3
Uros	7E-03	0.3	Dynlt3	1E-17	0.3
Vit	3E-03	0.3	Ppp1r9a	2E-03	0.3
Tmem18	6E-02	0.3	Gm16845	2E-01	0.3
Tbc1d22b	1E-01	0.3	Cmtm4	1E-03	0.3
Sppl2b	2E-01	0.3	Zfp976	4E-01	0.3
Irf2bp1	4E-06	0.3	Odc1	3E-08	0.3
Zfp24	5E-09	0.3	Kifc3	1E-01	0.3
Txnrd2	3E-02	0.3	Ccser1	3E-03	0.3
Cbx4	2E-03	0.3	Irf2bp1	4E-04	0.3
Top2b	8E-22	0.3	Gm14798	7E-01	0.3
Amotl1	3E-11	0.3	Arv1	5E-01	0.3
Cdk8	7E-08	0.3	Ccdc115	4E-04	0.3
Zfp974	3E-02	0.3	Cox6a2	5E-01	0.3

Fibroblast

Control			NICD2		
Gene	*p*	FC	Gene	*p*	FC
Rps7	6E-236	0.2	Mta3	8E-09	0.2
Tnks2	2E-32	0.2	Serpinb6a	4E-42	0.2
Smurf1	4E-09	0.2	Gm43672	9E-03	0.2
Ankrd24	3E-08	0.2	Elac1	2E-01	0.2
Htatip2	5E-23	0.2	Alg9	3E-03	0.2
Dpm3	1E-42	0.2	Esyt2	4E-05	0.2
Rab26os	4E-04	0.2	Ier2	2E-06	0.2
Thnsl2	3E-05	0.2	Sco2	2E-05	0.2
C1qtnf4	2E-02	0.2	Armcx3	7E-18	0.2
Rps3	3E-196	0.2	Tgfbrap1	2E-02	0.2
Rps18	1E-156	0.2	Iah1	2E-12	0.2
Washc1	1E-07	0.2	Aebp1	8E-22	0.2
Gm11808	9E-25	0.2	Gm16835	8E-02	0.2
B4galt2	6E-11	0.2	Pld2	9E-02	0.2
Emp1	2E-16	0.2	Cers4	5E-06	0.2
Rps11	2E-243	0.2	Ptpn13	3E-08	0.2
Pmvk	4E-12	0.2	Pip5k1c	6E-04	0.2
Gm16008	6E-02	0.2	Efs	5E-05	0.2
Pld3	6E-31	0.2	Cmtm6	9E-02	0.2
Lekr1	3E-04	0.2	Slc38a2	1E-11	0.2
Dcn	1E-03	0.2	Casp9	3E-03	0.2
Trim32	1E-04	0.2	Psmb10	1E-10	0.2
Parva	4E-28	0.2	Adamts2	6E-13	0.2
Rhobtb1	4E-06	0.2	Gng10	2E-17	0.2
Rbp1	7E-10	0.2	Zfp651	4E-07	0.2
Kmt2c	1E-20	0.2	Izumo4	1E-05	0.2
Gask1b	6E-11	0.2	Prdm5	9E-10	0.2
Adamtsl5	1E-13	0.2	Lgals8	2E-09	0.2
Pofut2	1E-23	0.2	Irs1	7E-10	0.2
Vamp2	4E-14	0.2	Pcdhga7	5E-05	0.2
Zbtb4	3E-09	0.2	Fpgs	9E-03	0.2
Rpl38	4E-216	0.2	Nxpe4	6E-11	0.2
Tmem19	7E-10	0.2	Ap3b1	4E-15	0.2
Parp10	2E-02	0.2	Tm7sf2	2E-05	0.2
Tpst2	7E-08	0.2	Pisd	4E-02	0.2
Cotl1	2E-12	0.2	Trim25	2E-04	0.2
Adck2	2E-03	0.2	Bmyc	5E-13	0.2
Hacd1	5E-17	0.2	Usp22	2E-07	0.2
Grk5	3E-12	0.2	Thnsl2	9E-03	0.2
Endog	5E-07	0.2	Fryl	9E-04	0.2
Dubr	9E-07	0.2	Pafah1b3	1E-08	0.2
Zswim6	3E-19	0.2	Tbc1d23	2E-06	0.2
Zfp800	3E-03	0.2	Lrrc32	2E-08	0.2
Pxk	5E-10	0.2	Sybu	2E-01	0.2
Gng12	3E-33	0.2	Nfil3	4E-04	0.2
Prdm5	3E-14	0.2	Rpl17	5E-119	0.2
Armcx3	3E-29	0.2	Fah	7E-03	0.2
Sec23a	3E-23	0.2	Vangl1	4E-04	0.2
Pafah1b3	1E-14	0.2	Slc6a8	2E-05	0.2
Nadk2	2E-05	0.2	Celf5	6E-02	0.2
Snn	2E-05	0.2	Coro1b	3E-03	0.2
Rac3	6E-09	0.2	Ggh	8E-18	0.2
Klf6	7E-21	0.2	Nanp	1E-01	0.2
Rps13	4E-213	0.2	Cfap69	2E-02	0.2
Six4	9E-02	0.2	Zdhhc17	5E-06	0.2
Cd63	2E-86	0.2	Tcta	6E-07	0.2
Plat	3E-06	0.2	Tmem127	7E-05	0.2
Phldb2	1E-63	0.2	Hacd1	9E-10	0.2
Calm3	9E-50	0.2	A930007I19Rik	9E-02	0.2
0610010K14Rik	6E-24	0.2	Als2	3E-02	0.2
Zbtb37	9E-06	0.2	Ssbp4	1E-16	0.2
Plxdc2	7E-31	0.2	Gm30054	7E-01	0.2
Zdhhc15	1E-01	0.2	Tmem106b	2E-16	0.2
Rgs17	2E-12	0.2	Ccdc106	5E-01	0.2
Atp6v0d1	2E-28	0.2	Sept9	9E-08	0.2
Dnm2	1E-18	0.2	Cyba	1E-28	0.2
Tmem131	8E-12	0.2	Acad10	1E-01	0.2
Snx4	6E-25	0.2	Tceal8	3E-24	0.2
Stt3a	1E-19	0.2	Calm3	6E-28	0.2
Rps5	6E-154	0.2	Rspry1	2E-08	0.2
Eogt	4E-02	0.2	Dnajc13	4E-12	0.2
Tmem205	2E-25	0.2	Syde1	8E-04	0.2
Spag9	3E-45	0.2	Tnrc6a	6E-15	0.2

Fibroblast?

Control			NICD2		
Gene	*p*	FC	Gene	*p*	FC
Lmln	2E-11	0.6	C2cd2	2E-30	0.6
Nme1	6E-84	0.6	Sars2	4E-16	0.6
Cggbp1	7E-67	0.6	Acp1	3E-101	0.6
Fam57a	9E-19	0.6	Agtpbp1	8E-39	0.6
Hus1	1E-17	0.6	Rad1	3E-31	0.6
Gm2115	2E-26	0.6	Arrb2	1E-17	0.6
Ppp2r2d	6E-51	0.6	Zc3hav1l	1E-20	0.6
Actl6a	1E-32	0.6	Ahctf1	2E-43	0.6
Snx25	6E-27	0.6	Lrrc56	6E-15	0.6
Hmgcll1	1E-18	0.6	E2f3	1E-37	0.6
Ppwd1	3E-28	0.6	Paip2b	3E-35	0.6
Clcc1	3E-36	0.6	Flrt1	1E-21	0.6
Nmt2	1E-29	0.6	Dxo	3E-27	0.6
Snrpf	5E-91	0.6	1700086O06Rik	4E-23	0.6
Acad8	2E-07	0.6	Dazap1	2E-58	0.6
Sgf29	2E-28	0.6	Egln1	4E-55	0.6
Ppip5k1	1E-17	0.6	Elf4	5E-43	0.6
Oser1	2E-29	0.6	Tspo	2E-71	0.6
Pgk1	3E-71	0.6	Cabp1	3E-12	0.6
Bckdk	3E-36	0.6	Fancf	2E-26	0.6
Ptma	6E-171	0.6	Galnt1	3E-53	0.6
H2afy	1E-49	0.6	Topors	1E-43	0.6
Dgkz	5E-26	0.6	Dmd	6E-43	0.6
B3galnt1	6E-34	0.6	Adgrg6	2E-41	0.6
Srsf1	1E-42	0.6	Zbtb14	2E-26	0.6
Gfer	2E-40	0.6	Zbtb45	5E-24	0.6
Brd3	8E-43	0.6	Mad2l1bp	1E-39	0.6
Ppp1r13l	7E-12	0.6	Pcolce2	1E-45	0.6
Hnrnpa1	3E-75	0.6	Scx	5E-16	0.6
Wbp11	8E-59	0.6	Mlkl	7E-30	0.6
C2cd3	7E-28	0.6	2310057M21Rik	3E-22	0.6
Lrrc20	2E-21	0.6	Sap18	1E-58	0.6
Gramd1c	3E-14	0.6	Jade3	8E-15	0.6
1700086O06Rik	6E-20	0.6	Yeats4	8E-43	0.6
Gimap9	1E-10	0.6	Usp14	3E-67	0.6
Set	2E-91	0.6	Hacd4	1E-39	0.6
Lrrc75a	5E-26	0.6	Cep41	9E-32	0.6
Pm20d1	4E-13	0.6	Nfic	6E-90	0.6
Nsun5	2E-14	0.6	H2afy	5E-65	0.6
Ampd3	6E-27	0.6	Ptp4a3	2E-16	0.6
Nuak2	4E-20	0.6	Hdgf	3E-124	0.6
Susd5	3E-11	0.6	Fam92a	1E-79	0.6
Cavin2	2E-19	0.6	Fkbp2	4E-101	0.6
Agfg2	2E-33	0.6	Btbd8	1E-29	0.6
Myof	9E-57	0.6	Aldh16a1	1E-24	0.6
Egfl7	2E-14	0.6	Ino80c	5E-18	0.6
3110082I17Rik	5E-25	0.6	Vbp1	1E-29	0.6
Lpcat4	2E-10	0.6	Psmg2	9E-48	0.6
Asrgl1	6E-24	0.6	Ptprj	1E-37	0.6
Loxl2	9E-25	0.6	Sf1	7E-47	0.6
Elovl5	2E-24	0.6	Bcl7c	1E-84	0.6
Cacna1c	2E-16	0.6	Tmem97	5E-48	0.6
Sppl	3E-19	0.6	Wrnip1	3E-30	0.6
Otud6b	5E-26	0.6	Ets1	8E-31	0.6
Hacd4	1E-29	0.6	Inf2	3E-36	0.6
Faap24	1E-24	0.6	Cep83os	4E-29	0.6
Gm13684	7E-12	0.6	Coq7	2E-33	0.6
Atp1b1	7E-16	0.6	Gm10501	1E-16	0.6
Gpr19	3E-17	0.6	Cpsf2	9E-44	0.6
Stip1	6E-57	0.6	Exosc7	4E-38	0.6
Spata24	2E-27	0.6	Cacybp	1E-94	0.6
Gm16062	4E-13	0.6	Dusp14	7E-14	0.6
Cnot6	9E-38	0.6	Tnfrsf12a	3E-36	0.6
Rbm14	3E-28	0.6	Katnb1	2E-23	0.6
Opa3	6E-22	0.6	Rps6ka5	1E-52	0.6
Dennd5b	3E-24	0.6	Emc8	2E-48	0.6
Gatad1	3E-68	0.6	Gfra4	1E-27	0.6
Pard6g	8E-28	0.6	Uck2	3E-31	0.6
Tmed5	1E-41	0.6	Cnn2	2E-40	0.6
Pds5b	6E-33	0.6	Pcbd1	1E-13	0.6
Gatad2a	4E-39	0.6	Elovl5	6E-33	0.6
2610203C22Rik	7E-11	0.6	Prps2	1E-33	0.6
Gtpbp10	6E-24	0.6	Fstl3	5E-23	0.6

Undefined

Control			NICD2		
Gene	*p*	FC	Gene	*p*	FC
Rnf44	1E+00	0.1	Eif3g	4E-01	0.1
Mrps34	9E-03	0.1	Dcaf13	3E-01	0.1
Ncstn	2E-02	0.1	Foxp1	8E-03	0.1
Kif6	8E-01	0.1	Zfp788	8E-01	0.1
Ruvbl1	9E-04	0.1	Mrpl44	6E-01	0.1
Rnf123	6E-01	0.1	Aff1	5E-02	0.1
Tmtc4	8E-01	0.1	Snap23	3E-02	0.1
Crcp	9E-01	0.1	Slc16a3	1E+00	0.1
Fastkd2	5E-01	0.1	Rrp1	3E-03	0.1
Gm42439	5E-01	0.1	Wdr47	7E-01	0.1
Glt8d1	5E-05	0.1	Ntmt1	6E-01	0.1
Dnajc17	1E+00	0.1	Nrbp1	8E-02	0.1
Nub1	6E-02	0.1	1810034E14Rik	4E-01	0.1
Zfp68	1E-01	0.1	Shank1	4E-01	0.1
Nomo1	7E-02	0.1	Ndufaf3	6E-01	0.1
Stk19	3E-01	0.1	Zmym2	1E-01	0.1
Agap1	3E-03	0.1	Ppp6r2	7E-01	0.1
Acads	3E-02	0.1	H2-D1	3E-09	0.1
Mapk8ip1	8E-01	0.1	Dhx35	4E-01	0.1
Tgfbr2	2E-03	0.1	Fam32a	9E-03	0.1
Psen2	8E-01	0.1	Ssc5d	1E-02	0.1
Slc12a9	5E-01	0.1	Strbp	7E-03	0.1
Chrd	5E-01	0.1	Wdr45	9E-01	0.1
D830024N08Rik	5E-01	0.1	Khk	7E-01	0.1
Xpa	2E-01	0.1	Timm9	4E-01	0.1
Ccdc32	3E-01	0.1	Fopnl	4E-01	0.1
Clip1	1E-02	0.1	Dnajb2	5E-01	0.1
Alg9	8E-01	0.1	Scyl3	9E-02	0.1
Tpcn2	2E-01	0.1	Olfml1	3E-01	0.1
Hspbp1	5E-01	0.1	Zfp606	3E-01	0.1
Micos10	3E-08	0.1	Zfp955a	6E-01	0.1
Calcoco1	5E-01	0.1	Casp6	4E-01	0.1
Fbxo38	9E-01	0.1	Dap	3E-02	0.1
Spns1	6E-01	0.1	Smim8	5E-01	0.1
Wdr6	6E-01	0.1	Lcmt2	9E-01	0.1
Sema3b	4E-01	0.1	Ifi30	7E-01	0.1
Cdk8	6E-01	0.1	Flcn	2E-01	0.1
Wbp1	4E-01	0.1	Shf	5E-01	0.1
Tmem94	7E-01	0.1	Psenen	2E-06	0.1
Sphk2	9E-01	0.1	Gli3	2E-02	0.1
Dpy19l3	5E-01	0.1	Wdsub1	8E-01	0.1
Slc4a3	7E-01	0.1	Gna12	3E-01	0.1
Zfp560	4E-02	0.1	Prpf19	4E-01	0.1
Thtpa	8E-01	0.1	Acox3	4E-01	0.1
Baiap2l1	3E-01	0.1	Clip3	2E-01	0.1
Rbm41	1E-01	0.1	Tmem241	7E-01	0.1
Clpp	5E-02	0.1	Chid1	9E-01	0.1
Amotl1	3E-03	0.1	Pkig	2E-01	0.1
Lekr1	7E-02	0.1	Ergic3	6E-05	0.1
Anxa5	6E-12	0.1	6030443J06Rik	9E-01	0.1
Nphp1	6E-01	0.1	Guk1	2E-03	0.1
Zfp397	5E-01	0.1	Sap30bp	4E-01	0.1
Scyl2	9E-01	0.1	Elk3	4E-01	0.1
Fam174a	9E-05	0.1	Gnptg	6E-01	0.1
Grhpr	6E-01	0.1	Kdm6b	2E-01	0.1
Abcd4	9E-01	0.1	Tmem140	6E-01	0.1
Grwd1	9E-01	0.1	Hspbp1	4E-01	0.1
Rnf135	4E-01	0.1	Stk25	9E-01	0.1
Hexa	7E-07	0.1	Fbrs	7E-01	0.1
Mpp6	7E-01	0.1	Cd24a	6E-01	0.1
Cyth2	3E-01	0.1	Tpd52l1	4E-01	0.1
Bzw2	3E-02	0.1	Tet3	7E-01	0.1
Hist1h2bc	6E-06	0.1	D630045J12Rik	3E-01	0.1
Rrp36	7E-01	0.1	Rhbdd2	2E-01	0.1
Ppp3cc	3E-01	0.1	Prmt9	3E-01	0.1
Slc9a9	2E-02	0.1	Cfap97	9E-01	0.1
Zfp146	1E+00	0.1	Ddah2	8E-03	0.1
Ptprd	1E-04	0.1	Kdm3a	6E-01	0.1
Col6a1	1E-08	0.1	C1qtnf1	1E+00	0.1
Polr2k	3E-03	0.1	Ddx6	3E-03	0.1
Fam207a	2E-01	0.1	Pxdn	5E-03	0.1
Clcn3	2E-01	0.1	Cluap1	7E-01	0.1
Bicra	9E-01	0.1	Bckdha	4E-01	0.1

Articular/Synovial Fibroblast

Control			NICD2		
Gene	*p*	FC	Gene	*p*	FC
Mpp2	8E-01	0.3	Retreg2	9E-02	0.3
Sipa1	8E-01	0.3	Ptrh1	1E+00	0.3
Lemd2	6E-02	0.3	Dnajc27	8E-01	0.3
Fam120b	2E-04	0.3	Enpp1	6E-03	0.3
E130308A19Rik	9E-01	0.3	Phlda1	9E-01	0.3
mt-Atp6	7E-49	0.3	Mapk8ip1	1E+00	0.3
Tmem53	5E-02	0.3	Zc3hav1	2E-02	0.3
Clcn6	8E-01	0.3	Ppif	4E-01	0.3
Gm11944	7E-01	0.3	Coq5	9E-01	0.3
Cfap300	9E-01	0.3	Emp3	1E-19	0.3
Ano5	3E-01	0.3	Tmem41a	6E-01	0.3
Zfp53	6E-01	0.3	Dnmbp	8E-01	0.3
Hoxa13	8E-01	0.3	Sat2	5E-01	0.3
mt-Nd5	1E-19	0.3	Itpr2	1E-02	0.3
Plcxd2	1E+00	0.3	Zfp760	1E+00	0.3
Cyb5a	3E-19	0.3	Cyb5a	1E-10	0.3
Gm14305	9E-01	0.3	Pced1a	7E-01	0.3
C030034I22Rik	4E-01	0.3	Mknk1	8E-01	0.3
Tmem14a	4E-01	0.3	Cdkn2a	3E-01	0.3
Eml3	3E-01	0.3	Wdr24	6E-01	0.3
Gnb5	2E-01	0.3	Daglb	1E+00	0.3
Gm47071	7E-01	0.3	Atg10	3E-02	0.3
Vps13b	2E-04	0.3	Klhl12	9E-01	0.3
Acadl	3E-16	0.3	Slx1b	8E-01	0.3
Ldlr	4E-02	0.3	Tmem87b	3E-01	0.3
Nicn1	1E-01	0.3	Cdkn1a	4E-06	0.3
Wrn	5E-02	0.3	Galk2	3E-05	0.3
Cds2	4E-01	0.3	Tef	4E-02	0.3
Rps6kc1	4E-01	0.3	Igf2bp2	1E-01	0.3
Rad52	8E-01	0.3	Aldh6a1	2E-01	0.3
Eef1akmt1	5E-03	0.3	Samd14	9E-01	0.3
Mxd4	1E-11	0.3	Layn	8E-02	0.3
1700096K18Rik	6E-01	0.3	Tmem241	5E-01	0.3
Fbxo44	6E-01	0.3	Atl1	8E-01	0.3
6330562C20Rik	9E-01	0.3	Adcy6	8E-01	0.3
Sh2b3	8E-01	0.3	Myl12b	7E-08	0.3
Dyrk1b	5E-01	0.3	Galc	8E-01	0.3
Slc2a8	5E-01	0.3	Purg	8E-02	0.3
Gpatch1	4E-01	0.3	Retreg3	9E-01	0.3
Snap29	8E-01	0.3	Zfp994	9E-01	0.3
Slc16a1	5E-01	0.3	Gucd1	9E-01	0.3
Cage1	8E-01	0.3	1810058I24Rik	5E-13	0.3
Tmem35b	9E-01	0.3	Iffo1	9E-01	0.3
Gm50013	6E-01	0.3	Fsd1l	1E+00	0.3
Lamc1	8E-04	0.3	Bicral	2E-01	0.3
Cdk5	8E-01	0.3	L3mbtl3	6E-01	0.3
Cpq	6E-13	0.3	mt-Atp6	2E-44	0.3
Enpp4	8E-01	0.3	Fuom	3E-02	0.3
4930481A15Rik	7E-01	0.3	Tmem192	3E-02	0.3
Ndst2	3E-01	0.3	Pxylp1	7E-01	0.3
Fth1	3E-40	0.3	Aqp1	9E-01	0.3
Sh3bgrl3	7E-22	0.3	Ppil2	3E-02	0.3
Naga	1E-01	0.3	Bbc3	4E-01	0.3
Ap5z1	1E+00	0.3	Pigq	7E-01	0.3
Lrrc28	6E-01	0.3	Tsku	6E-01	0.3
Atp6v1e1	3E-11	0.3	Lamp2	9E-18	0.3
Fbxl20	2E-03	0.3	Maoa	3E-02	0.3
Snx12	4E-05	0.3	Rgl2	8E-01	0.3
Pdlim2	1E-08	0.3	Tesk2	3E-01	0.3
Rpain	2E-01	0.3	Crppa	8E-01	0.3
Gas7	2E-04	0.3	Fbxo28	6E-01	0.3
Cluap1	3E-02	0.3	Lrrc28	4E-01	0.3
Apobec3	9E-01	0.3	Cys1	8E-01	0.3
Tns2	4E-01	0.3	Asah1	4E-10	0.3
Prr13	1E-04	0.3	Il17rc	3E-01	0.3
Slk	8E-03	0.3	Det1	6E-01	0.3
Sri	1E-11	0.3	Inpp5k	8E-01	0.3
Zbtb9	9E-01	0.3	Zfp213	5E-01	0.3
Mks1	9E-01	0.3	Pgap1	9E-01	0.3
Taf5l	1E+00	0.3	Ap4m1	9E-01	0.3
Hnrnpll	5E-02	0.3	Slc36a1	7E-01	0.3
Zdhhc7	4E-01	0.3	Tmem65	2E-03	0.3
Cst6	8E-04	0.3	2900060B14Rik	9E-01	0.3

Limb Mesenchyme

Control Gene	p	FC	NICD2 Gene	p	FC
Inafm2	7E-05	0.3	Xrcc5	4E-09	0.3
Dcaf7	6E-09	0.3	Sec23a	1E-10	0.3
Slc22a14	8E-04	0.3	Ttc3	6E-22	0.3
Edrf1	4E-07	0.3	Ankrd50	1E-06	0.3
Thada	7E-10	0.3	Snai2	2E-10	0.3
Zfp668	2E-04	0.3	Desi2	8E-15	0.3
Gm11457	7E-04	0.3	Ppargc1a	6E-04	0.3
Rai1	3E-07	0.3	Armcx5	4E-06	0.3
Fhl3	2E-04	0.3	Tmf1	4E-13	0.3
Qsox1	1E-12	0.3	Spred2	1E-05	0.3
Zw10	5E-05	0.3	Arf3	7E-10	0.3
Tmem67	8E-05	0.3	Ptpn13	7E-06	0.3
Apaf1	2E-07	0.3	Tmem263	8E-15	0.3
Gm14858	1E-04	0.3	Pum3	4E-09	0.3
Raly	3E-15	0.3	Jagn1	2E-10	0.3
Snhg18	1E-28	0.3	Ywhaz	8E-28	0.3
Ckap4	8E-24	0.3	Copa	6E-18	0.3
Kat2a	1E-04	0.3	1700084C06Rik	6E-05	0.3
Gm14798	8E-04	0.3	Get4	1E-06	0.3
Slc10a7	1E-09	0.3	Ptk2	8E-09	0.3
Nip7	2E-09	0.3	Ctsh	8E-19	0.3
Rabepk	4E-08	0.3	Ranbp3	3E-07	0.3
Zcwpw2	3E-04	0.3	Eps8l2	2E-06	0.3
Zfp24	5E-10	0.3	Ythdf3	5E-11	0.3
Rprd1a	3E-09	0.3	Tsg101	1E-10	0.3
Map3k4	4E-08	0.3	Slc25a47	4E-03	0.3
Traf2	5E-04	0.3	2610035D17Rik	5E-06	0.3
Msantd3	1E-06	0.3	Rai1	6E-07	0.3
Hoxc4	1E-07	0.3	Lman1	1E-28	0.3
B930036N10Rik	4E-07	0.3	Med24	1E-05	0.3
Capn10	2E-05	0.3	Hmg20a	2E-09	0.3
1810013L24Rik	3E-09	0.3	Zfp9	5E-05	0.3
Srp68	2E-10	0.3	Mia2	4E-11	0.3
Ddb1	7E-16	0.3	Col5a2	2E-28	0.3
Purb	4E-15	0.3	Serf1	4E-14	0.3
Eif4g1	1E-16	0.3	Zbtb6	3E-07	0.3
Pard3bos1	6E-06	0.3	Zfp715	3E-04	0.3
Cpsf2	2E-08	0.3	Actg1	1E-15	0.3
Rc3h2	7E-11	0.3	Tmub2	4E-05	0.3
Dcaf11	1E-06	0.3	Zfp397	7E-08	0.3
Atf6	2E-09	0.3	Mtpap	4E-06	0.3
Rars2	1E-08	0.3	Cttn	1E-10	0.3
Otub2	2E-03	0.3	Mars	3E-08	0.3
Arl5b	5E-06	0.3	Med13	2E-10	0.3
Apba3	2E-06	0.3	Med1	6E-11	0.3
Pomgnt1	2E-07	0.3	Brix1	3E-09	0.3
Smndc1	5E-10	0.3	Ldlrap1	2E-05	0.3
Osbpl2	1E-08	0.3	Glis2	1E-04	0.3
Ids	8E-06	0.3	Phactr4	5E-08	0.3
Aven	2E-09	0.3	Wac	4E-15	0.3
Igf1r	4E-15	0.3	Nt5c2	3E-06	0.3
Tmpo	5E-16	0.3	Wdr75	2E-08	0.3
Gps1	5E-11	0.3	Cavin2	6E-01	0.3
Tmsb4x	5E-19	0.3	Cerk	2E-03	0.3
Cog2	1E-07	0.3	Usp11	2E-07	0.3
Zbtb17	1E-05	0.3	Magi1	4E-11	0.3
Sec63	4E-15	0.3	Ypel1	1E-05	0.3
Gm47167	4E-06	0.3	Dhx57	3E-07	0.3
AW209491	8E-06	0.3	Larp1	1E-11	0.3
Rabl3	6E-05	0.3	Osbpl6	4E-05	0.3
Gm32618	8E-06	0.3	Acbd3	4E-16	0.3
Bud23	2E-09	0.3	Gadd45g	1E-09	0.3
Smim3	3E-05	0.3	Golph3	2E-09	0.3
Lrch2	3E-03	0.3	Gm19710	3E-05	0.3
Tfcp2	4E-07	0.3	Zfp281	3E-06	0.3
Spin1	6E-09	0.3	Gm48765	5E-03	0.3
Rbm34	1E-06	0.3	Adh7	8E-03	0.3
Eef1akmt4	2E-07	0.3	B230217C12Rik	9E-06	0.3
Qrsl1	6E-05	0.3	Pycr2	1E-07	0.3
Arfip1	1E-07	0.3	Pdzrn3	1E-09	0.3
Gtf2i	2E-17	0.3	Senp2	4E-08	0.3
Lrrc20	3E-04	0.3	Yes1	4E-06	0.3
Ndor1	3E-06	0.3	Zfp64	1E-06	0.3

Chondrogenic

Control Gene	p	FC	NICD2 Gene	p	FC
Pitpnm2	4E-02	0.3	Mknk1	2E-02	0.3
Rabac1	3E-35	0.3	Dmac2	3E-01	0.3
Zfp146	1E-03	0.3	2300009A05Rik	9E-04	0.3
Stim2	1E-04	0.3	Cald1	2E-25	0.3
Zmym6	3E-04	0.3	Aldh2	1E-20	0.3
Proser1	5E-03	0.3	Zfp946	8E-01	0.3
Dap	6E-27	0.3	Gm20732	3E-01	0.3
Hoxa13	5E-01	0.3	Tbpl1	4E-04	0.3
Zfp971	3E-01	0.3	Zfp869	5E-03	0.3
Pag1	2E-02	0.3	Spsb3	1E-01	0.3
BC002059	6E-02	0.3	Stard13	1E-09	0.3
Specc1l	1E-11	0.3	Txndc15	1E-11	0.3
Cep85l	8E-02	0.3	Rufy1	2E-03	0.3
1110032A03Rik	4E-09	0.3	Iqcb1	3E-03	0.3
Pink1	2E-06	0.3	Arhgap39	2E-01	0.3
C230035I16Rik	1E-01	0.3	Tusc2	1E-01	0.3
Lman1	5E-41	0.3	Fam214b	5E-03	0.3
B3gnt2	7E-04	0.3	Snrpn	3E-01	0.3
Ktn1	1E-19	0.3	Gm15050	6E-02	0.3
Ddrgk1	5E-18	0.3	Arid1b	3E-09	0.3
Cers2	4E-08	0.3	Narf	5E-02	0.3
1700012D14Rik	4E-03	0.3	Abat	7E-03	0.3
Slc44a1	2E-08	0.3	Mtmr10	2E-02	0.3
Erlec1	9E-27	0.3	Traf3	2E-03	0.3
Ivd	1E-08	0.3	Slc25a38	8E-02	0.3
Bcl7b	6E-08	0.3	Nfat5	4E-16	0.3
Rnf130	2E-12	0.3	Abhd17b	2E-04	0.3
Zfp949	6E-02	0.3	Exoc3	2E-10	0.3
Wdr73	1E-01	0.3	Nek9	6E-06	0.3
2610020C07Rik	8E-03	0.3	Smim4	6E-07	0.3
Rpgr	5E-02	0.3	Tpra1	6E-02	0.3
Gm37240	8E-06	0.3	Acot8	4E-01	0.3
Ahcyl1	4E-17	0.3	Rhbdd2	1E-01	0.3
Auts2	4E-20	0.3	Szt2	5E-01	0.3
Gm12353	2E-02	0.3	Cped1	8E-10	0.3
Mylip	6E-06	0.3	Lpar6	5E-01	0.3
Rab26os	1E-01	0.3	Creb3l2	1E-07	0.3
Tmx4	9E-07	0.3	Abca3	2E-01	0.3
Nrip2	1E-01	0.3	Usp24	1E-05	0.3
Mipol1	1E-05	0.3	Tbc1d22b	3E-01	0.3
Polr2l	2E-10	0.3	Dennd1b	5E-04	0.3
Bmi1	3E-04	0.3	Pde3a	2E-03	0.3
Lrig2	2E-04	0.3	Lats2	9E-08	0.3
Dstyk	6E-04	0.3	Letm2	6E-02	0.3
Usp54	3E-02	0.3	Cnot7	3E-05	0.3
Cnpy3	3E-05	0.3	Rian	8E-06	0.3
Cgrrf1	8E-09	0.3	Bola1	2E-09	0.3
Exosc3	9E-05	0.3	Hinfp	5E-01	0.3
Vgll3	7E-08	0.3	Pon3	1E-05	0.3
Ccdc157	2E-02	0.3	Ctbp2	3E-02	0.3
Unc50	3E-17	0.3	Tmem167b	8E-03	0.3
Zfp35	7E-02	0.3	Fut10	2E-01	0.3
Tbc1d13	2E-01	0.3	Ccdc157	6E-02	0.3
Tmem218	6E-06	0.3	Tab1	1E-01	0.3
Micu3	2E-04	0.3	Fzd8	5E-05	0.3
Nfe2l1	2E-23	0.3	Tvp23a	4E-01	0.3
Cebpzos	4E-11	0.3	Tbc1d19	3E-03	0.3
Rtkn	2E-02	0.3	Hps5	2E-02	0.3
Adck1	8E-02	0.3	Lrrc20	9E-02	0.3
Pacsin2	6E-10	0.3	P3h3	5E-12	0.3
Letmd1	1E-01	0.3	Ggact	6E-03	0.3
Myo9b	1E-07	0.3	Aifm2	2E-01	0.3
Cped1	1E-12	0.3	Lrrc8a	5E-04	0.3
Atg14	7E-04	0.3	Tmed10	9E-35	0.3
Gm11266	1E-02	0.3	B3glct	2E-04	0.3
E130102H24Rik	1E-02	0.3	Btbd6	6E-01	0.3
Ndufaf3	2E-08	0.3	Fkbp10	8E-11	0.3
E530011L22Rik	1E-01	0.3	A330023F24Rik	2E-02	0.3
Aven	3E-07	0.3	Adat2	1E-01	0.3
Tapt1	1E-02	0.3	Bloc1s6	1E-03	0.3
Crtc1	2E-03	0.3	A630072M18Rik	3E-01	0.3
Abhd14a	2E-11	0.3	Sec14l1	6E-05	0.3
Med31	3E-07	0.3	Inpp5k	1E-02	0.3

Fibroblast

Control Gene	p	FC	NICD2 Gene	p	FC
Ubqln2	3E-07	0.2	Sept11	6E-31	0.2
C920021L13Rik	6E-02	0.2	Xbp1	7E-14	0.2
Trib1	1E-02	0.2	Fam8a1	3E-06	0.2
Bri3bp	3E-16	0.2	Gm2000	2E-15	0.2
Trmt61a	1E-02	0.2	Gclm	4E-15	0.2
Trim25	9E-06	0.2	Osbpl7	8E-02	0.2
Bcl9l	9E-19	0.2	H2-T22	3E-09	0.2
Plekhf1	1E-09	0.2	Pex1	2E-02	0.2
Ltbp4	6E-05	0.2	Psme1	3E-17	0.2
Ggt7	5E-05	0.2	Msi1	3E-01	0.2
Slc7a6	1E-05	0.2	Fez1	1E-06	0.2
Gatd1	5E-08	0.2	Gns	5E-12	0.2
Sh3yl1	1E-04	0.2	Uba52	3E-38	0.2
Aldh16a1	2E-03	0.2	Fam91a1	4E-06	0.2
Stox2	6E-13	0.2	Dnpep	3E-12	0.2
Amdhd2	2E-07	0.2	Col24a1	2E-02	0.2
E430024I08Rik	8E-06	0.2	Rps20	6E-109	0.2
Snai1	5E-13	0.2	Svbp	5E-23	0.2
Kidins220	1E-20	0.2	Dctn6	7E-11	0.2
Gm5617	3E-13	0.2	Apeh	4E-05	0.2
Thoc6	5E-08	0.2	Leng8	2E-03	0.2
Supt3	2E-15	0.2	Zfp652	5E-11	0.2
Ctsz	2E-57	0.2	Tnpo2	1E-03	0.2
Col1a2	3E-108	0.2	Efl1	7E-05	0.2
Slc31a2	2E-03	0.2	Procr	2E-04	0.2
Phka2	2E-03	0.2	Tbck	3E-07	0.2
Gprasp1	3E-12	0.2	Vav3	2E-05	0.2
Zfp874a	1E-02	0.2	Ass1	4E-08	0.2
Lysmd3	4E-08	0.2	Gm11808	4E-15	0.2
Ube2w	7E-23	0.2	Etv6	2E-06	0.2
Ano8	5E-02	0.2	Arfgef2	2E-05	0.2
Fau	2E-203	0.2	Tmsb15b2	5E-03	0.2
Rock2	1E-20	0.2	Snx33	2E-03	0.2
Pi4ka	1E-10	0.2	Slc25a32	1E-03	0.2
Sox12	5E-04	0.2	Rpl38	4E-105	0.2
Map7d1	1E-13	0.2	Socs1	4E-04	0.2
Plk2	1E-10	0.2	Rpl9	5E-97	0.2
Rpl11	7E-200	0.2	Trip6	4E-08	0.2
Prkd3	6E-08	0.2	Zfp286	2E-02	0.2
Sept9	4E-13	0.2	Dlg1	1E-13	0.2
Gab2	1E-04	0.2	Cog4	2E-07	0.2
Mgmt	7E-21	0.2	Tor2a	9E-05	0.2
Fpgt	3E-03	0.2	Kdm4c	7E-08	0.2
Smim11	6E-25	0.2	Zfp110	1E-05	0.2
Zfp947	3E-01	0.2	D130040H23Rik	8E-03	0.2
Cybc1	7E-04	0.2	Zfc3h1	7E-10	0.2
Stk3	2E-15	0.2	Atxn7l1	4E-06	0.2
Hddc3	3E-03	0.2	Snrk	4E-06	0.2
Casp12	2E-19	0.2	2700046G09Rik	5E-02	0.2
Zfp607b	4E-02	0.2	Ccdc85b	2E-13	0.2
Wipf1	4E-14	0.2	Anxa6	2E-13	0.2
Ppp2cb	7E-13	0.2	Gnptab	3E-02	0.2
Kdelr3	6E-16	0.2	Bcl3	5E-04	0.2
Rpl8	3E-178	0.2	Gm5617	2E-08	0.2
Agps	2E-12	0.2	Dgka	2E-03	0.2
Pnp	1E-22	0.2	Lrrc42	6E-08	0.2
Lrrc17	3E-44	0.2	Fuca2	9E-05	0.2
Gabpa	3E-07	0.2	Fbxo25	3E-04	0.2
Tfe3	5E-08	0.2	Creg1	2E-18	0.2
Pwwp3b	2E-02	0.2	Gria3	6E-16	0.2
Ube2o	8E-08	0.2	Relch	5E-06	0.2
Nkd1	2E-07	0.2	1700096K18Rik	2E-02	0.2
Rraga	1E-28	0.2	Fam171b	8E-04	0.2
Plxnd1	5E-08	0.2	St6galnac6	6E-04	0.2
Angptl1	9E-02	0.2	Snapc3	9E-06	0.2
Efl1	1E-07	0.2	Cstb	7E-41	0.2
Rpl6	3E-166	0.2	Rpl37	3E-95	0.2
Ripk3	6E-08	0.2	Rps15a	2E-103	0.2
F8	2E-02	0.2	Rps18	6E-70	0.2
Homer1	2E-03	0.2	Ap5z1	3E-01	0.2
Gnptab	1E-04	0.2	Nfix	5E-35	0.2
Hook3	4E-29	0.2	Cda	2E-01	0.2
Eif3f	6E-70	0.2	F8	2E-01	0.2

Fibroblast?

Control Gene	p	FC	NICD2 Gene	p	FC
Cisd1	2E-47	0.6	Cnot6	3E-39	0.6
Sumo2	6E-154	0.6	Abcf2	2E-36	0.6
Kif2a	4E-50	0.6	Hace1	6E-33	0.6
Cfap43	2E-09	0.6	Fadd	1E-22	0.6
Qrsl1	3E-18	0.6	Arrb1	6E-17	0.6
4933412E12Rik	7E-14	0.6	Azin1	5E-49	0.6
AA467197	3E-09	0.6	Tmem43	2E-37	0.6
Mbnl3	3E-30	0.6	Tmem64	4E-29	0.6
Stub1	1E-103	0.6	Adk	2E-65	0.6
Cep97	2E-17	0.6	1110059E24Rik	1E-28	0.6
L2hgdh	6E-26	0.6	Tspan12	1E-33	0.6
Myo1c	9E-37	0.6	Vars	3E-39	0.6
Ciao2a	1E-41	0.6	Adgra2	1E-16	0.6
Tmx1	9E-39	0.6	Tbl2	1E-32	0.6
Acp1	6E-72	0.6	Cep97	4E-25	0.6
Nudt21	1E-45	0.6	Tmem186	3E-22	0.6
Ptges3	1E-90	0.6	B4galnt1	1E-09	0.6
Snrpb	1E-83	0.6	Ppp1r7	3E-40	0.6
Snrpe	2E-137	0.6	Tnfaip1	3E-40	0.6
Tubgcp4	2E-27	0.6	Tusc1	2E-23	0.6
Tead2	5E-32	0.6	Clmn	5E-21	0.6
Cpox	3E-26	0.6	Zwint	5E-47	0.6
Cad	2E-27	0.6	Axl	1E-39	0.6
Nhp2	9E-33	0.6	Zfp689	3E-13	0.6
Magohb	2E-35	0.6	Bcat1	4E-31	0.6
Cox18	1E-30	0.6	0610010F05Rik	3E-37	0.6
Lclat1	5E-26	0.6	Eea1	4E-53	0.6
Ift140	3E-24	0.6	Slc29a1	1E-41	0.6
Hbegf	5E-11	0.6	Gm19605	4E-08	0.6
Usp39	2E-27	0.6	Pithd1	6E-32	0.6
Cotl1	1E-38	0.6	Lsm4	6E-82	0.6
Lsp1	4E-21	0.6	Hnrnpa2b1	3E-134	0.6
Ifit2	5E-33	0.6	Tfam	1E-36	0.6
Dach2	5E-14	0.6	Fnbp1l	3E-33	0.6
Syne3	6E-27	0.6	Sema3e	1E-33	0.6
5730522E02Rik	2E-17	0.6	Emp1	1E-47	0.6
Phlda3	3E-43	0.6	Actn1	2E-51	0.6
Ppp1r2	4E-46	0.6	Zscan25	2E-03	0.6
Pfdn6	3E-48	0.6	Dpysl3	3E-28	0.6
Cplane2	3E-11	0.6	Psmc4	3E-45	0.6
Gm36211	4E-11	0.6	Phlda3	1E-60	0.6
Jazf1	1E-23	0.6	Smpd4	3E-28	0.6
Hdac10	1E-13	0.6	Stx2	2E-24	0.6
Limch1	2E-24	0.6	Pspc1	5E-41	0.6
Entpd5	4E-14	0.6	Limd2	9E-29	0.6
Tctn3	1E-12	0.6	Tti1	9E-22	0.6
Setdb2	2E-19	0.6	Ccn4	2E-31	0.6
Primpol	3E-21	0.6	Rpp21	1E-33	0.6
Pelp1	9E-28	0.6	Gps1	6E-47	0.6
Ptger4	6E-11	0.6	Rheb	5E-89	0.6
Flot2	4E-27	0.6	Map3k20	2E-46	0.6
Zbtb46	6E-12	0.6	Gm28875	2E-29	0.6
Gm15987	2E-24	0.6	Scoc	3E-63	0.6
Fkbp2	2E-71	0.6	C1ql3	9E-22	0.6
Ubash3b	8E-23	0.6	Trim28	8E-46	0.6
Rab13	8E-24	0.6	Sephs2	4E-34	0.6
Patl1	8E-23	0.6	Cbx1	2E-87	0.6
Ccdc25	3E-33	0.6	Gart	8E-30	0.6
Tmem177	1E-09	0.6	Hars	2E-34	0.6
Pot1a	2E-24	0.6	Mfsd12	2E-18	0.6
Kbtbd2	1E-28	0.6	Slc25a5	1E-62	0.6
Tnk2	7E-15	0.6	Tubgcp4	9E-32	0.6
Ipo11	2E-31	0.6	Taf6	2E-30	0.6
Zfp111	7E-21	0.6	Btbd19	5E-17	0.6
Ttc9c	3E-31	0.6	Gm16337	4E-17	0.6
Gpatch3	1E-05	0.6	Zfp365	6E-23	0.6
Ncoa7	2E-30	0.6	Bcl7a	4E-31	0.6
Cenpb	3E-43	0.6	Zdhhc6	7E-36	0.6
Arsj	1E-31	0.6	Dnajc10	5E-51	0.6
Gm10138	7E-10	0.6	Gtf3a	9E-30	0.6
Deps	2E-25	0.6	Prkd2	1E-24	0.6
Vopp1	1E-22	0.6	Caprin2	3E-24	0.6
Napb	2E-01	0.6	Litaf	2E-35	0.6

Undefined

Control Gene	p	FC	NICD2 Gene	p	FC
Heatr1	9E-01	0.1	Galnt4	7E-01	0.1
1110051M20Rik	5E-01	0.1	Cnpy4	2E-01	0.1
Mettl5	4E-01	0.1	Plekhn1	2E-01	0.1
Tpgs1	4E-01	0.1	Gskip	9E-01	0.1
Mthfsd	1E+00	0.1	Atp6v1a	3E-02	0.1
Hagh	8E-01	0.1	Zcchc9	8E-01	0.1
Brms1	9E-01	0.1	Desi2	1E-02	0.1
Glt8d2	5E-01	0.1	Tmem185b	3E-02	0.1
Ift81	7E-01	0.1	Stn1	4E-01	0.1
S100a1	2E-08	0.1	Rnf2	3E-01	0.1
Zc3h6	9E-01	0.1	Polr2k	3E-04	0.1
Acad10	6E-01	0.1	mt-Nd5	2E-13	0.1
Eif3g	1E-02	0.1	Gtf2h2	1E-01	0.1
Gstt2	8E-01	0.1	Cbx8	5E-01	0.1
Egr1	7E-02	0.1	Bmp2k	3E-01	0.1
Arv1	2E-01	0.1	Idua	9E-01	0.1
Gk5	8E-01	0.1	Sertad2	5E-01	0.1
R74862	5E-02	0.1	Zfp322a	5E-01	0.1
Secisbp2	4E-01	0.1	Bag5	3E-01	0.1
Actr8	7E-01	0.1	Zfp609	6E-01	0.1
Mmadhc	1E-01	0.1	Malsu1	2E-01	0.1
Leng9	3E-01	0.1	Pcdh9	3E-05	0.1
Usp21	2E-01	0.1	Zcchc10	3E-02	0.1
Sirt2	4E-03	0.1	Slc30a6	8E-01	0.1
Ccser2	8E-05	0.1	Jam3	7E-01	0.1
Abhd17b	2E-01	0.1	Psmc2	2E-02	0.1
Tmub2	3E-01	0.1	Brd7	3E-03	0.1
Tsr3	6E-02	0.1	Saal1	7E-01	0.1
Cwc25	1E+00	0.1	Crispld1	5E-46	0.1
Banp	1E+00	0.1	Bivm	1E-01	0.1
Crtc1	1E+00	0.1	Pomp	3E-05	0.1
Acsf2	9E-01	0.1	Ube2b	6E-09	0.1
4930581F22Rik	3E-01	0.1	Rab36	6E-01	0.1
Gm36198	7E-01	0.1	Oma1	9E-01	0.1
Zfp28	8E-01	0.1	Timmdc1	9E-01	0.1
Rit1	2E-01	0.1	Rce1	4E-01	0.1
Nemf	1E-01	0.1	Cgref1	2E-01	0.1
Zfp398	9E-01	0.1	Zfp983	8E-01	0.1
Ube2f	2E-02	0.1	Ephx1	8E-03	0.1
Dhrs11	7E-01	0.1	Bud23	4E-01	0.1
Bbs5	3E-01	0.1	Ttc13	8E-01	0.1
Fan1	2E-01	0.1	Commd6	1E-01	0.1
Unc119	3E-01	0.1	Tmed9	3E-12	0.1
Isoc2b	1E+00	0.1	Mtr	7E-01	0.1
Rpl10-ps3	5E-01	0.1	Tbc1d17	8E-01	0.1
Rnf11	3E-03	0.1	Zfyve27	4E-01	0.1
Taz	8E-01	0.1	Glt8d1	1E-01	0.1
Pdcd4	2E-06	0.1	Brsk1	4E-01	0.1
Ndufs2	9E-07	0.1	Emc10	3E-04	0.1
Ptcd3	1E+00	0.1	Utp14a	9E-02	0.1
Ttc5	6E-01	0.1	Srsf5	3E-04	0.1
Ift57	1E-01	0.1	Pla2g15	3E-01	0.1
Ergic3	1E-07	0.1	Jun	1E-02	0.1
5730455P16Rik	8E-01	0.1	5031425E22Rik	6E-04	0.1
Cage1	6E-01	0.1	Rsrc2	8E-02	0.1
Arhgef10l	3E-01	0.1	Stradb	5E-01	0.1
6330418K02Rik	6E-01	0.1	Ktn1	3E-04	0.1
Kansl2	2E-01	0.1	Cyth2	5E-01	0.1
Fem1c	9E-01	0.1	Mfsd8	3E-01	0.1
Ebna1bp2	3E-01	0.1	Emg1	5E-02	0.1
Zfp830	9E-01	0.1	Rab2b	8E-01	0.1
Ephx1	2E-02	0.1	Mkrn1	2E-01	0.1
Tsg101	6E-03	0.1	mt-Nd6	6E-01	0.1
Mcrs1	4E-01	0.1	Vps11	5E-01	0.1
Acp2	7E-01	0.1	Ppt2	1E+00	0.1
Tpd52l2	2E-01	0.1	Pygo1	5E-01	0.1
Vezf1	5E-03	0.1	Hps3	6E-01	0.1
Zfp799	9E-01	0.1	Qtrt2	3E-01	0.1
Tex264	4E-02	0.1	Synrg	3E-01	0.1
2900052L18Rik	8E-01	0.1	Cdk2ap2	7E-02	0.1
Rab8b	4E-01	0.1	Ptpn23	2E-02	0.1
Nsfl1c	5E-01	0.1	Phlpp1	4E-02	0.1
Polr2g	6E-03	0.1	Dlst	1E-01	0.1

Articular/Synovial Fibroblast

Control Gene	p	FC	NICD2 Gene	p	FC
Igf2r	3E-06	0.3	Cd81	9E-14	0.3
Ppp3cc	3E-01	0.3	C2cd5	1E-01	0.3
Atp2b1	1E-05	0.3	1810062O18Rik	8E-01	0.3
Lyrm9	3E-01	0.3	Clip4	7E-01	0.3
Dok5	6E-01	0.3	Eif5a2	5E-01	0.3
Atmin	4E-01	0.3	Vps37c	9E-01	0.3
Nbea	8E-03	0.3	Lmbr1	2E-01	0.3
Tceal3	3E-02	0.3	Zfp58	6E-01	0.3
Il34	9E-01	0.3	Slc25a44	9E-01	0.3
Aph1b	5E-01	0.3	Ganc	7E-01	0.3
Cbr3	4E-03	0.3	Elmod2	6E-01	0.3
Ssbp4	1E-04	0.3	Akt2	2E-02	0.3
Depdc5	4E-01	0.3	Tceal3	3E-01	0.3
2810402E24Rik	8E-01	0.3	D830050J10Rik	6E-01	0.3
Ece2	5E-01	0.3	Sirt2	2E-03	0.3
Gm26532	7E-01	0.3	Rsph3b	7E-01	0.3
Taf8	6E-01	0.3	Rasa4	7E-01	0.3
Cavin3	1E-10	0.3	4930522L14Rik	8E-01	0.3
Nudt3	2E-02	0.3	Gm30054	5E-01	0.3
Sh3d19	3E-08	0.3	Hoxd8	8E-01	0.3
Kank2	2E-04	0.3	Parp11	6E-01	0.3
Notch2	5E-03	0.3	Cobll1	2E-01	0.3
Fbln7	2E-03	0.3	Slc7a6	6E-01	0.3
Dffa	4E-01	0.3	Mgst3	5E-08	0.3
Zcchc4	1E+00	0.3	mt-Nd2	3E-22	0.3
Ciz1	3E-01	0.3	Vps26c	6E-01	0.3
Mxi1	4E-01	0.3	Ppt1	6E-05	0.3
Nfia	3E-12	0.3	Cln6	6E-01	0.3
Rabl2	8E-01	0.3	Adam17	2E-01	0.3
Zfp317	8E-01	0.3	Spg11	3E-01	0.3
Tmem123	8E-01	0.3	Gaa	6E-07	0.3
Slc35f6	8E-01	0.3	Kiz	4E-02	0.3
Apba1	5E-01	0.3	Polr3gl	1E-01	0.3
Atp6v0a1	3E-03	0.3	Tgs1	1E-02	0.3
Oxsm	8E-01	0.3	Map2k5	3E-02	0.3
Klf12	7E-02	0.3	Ccdc74a	7E-01	0.3
S1pr2	9E-01	0.3	Bbs9	4E-01	0.3
Hoxd9	5E-01	0.3	Acads	1E-01	0.3
Atp7a	4E-01	0.3	Chmp7	9E-01	0.3
Rufy2	8E-03	0.3	Ppp1r14b	5E-05	0.3
Akap1	8E-01	0.3	Wdr7	3E-01	0.3
Creld1	1E-01	0.3	Dnajc4	4E-02	0.3
Prdm15	1E+00	0.3	Zfp61	8E-01	0.3
Emp3	1E-23	0.3	Micall1	1E+00	0.3
Idh1	1E-12	0.3	Babam2	1E-04	0.3
Cacnb1	1E+00	0.3	Sdccag8	3E-03	0.3
Dhrs11	6E-01	0.3	Ccdc173	8E-01	0.3
Dock4	1E-02	0.3	Rgs3	2E-01	0.3
Nudt1	8E-01	0.3	Vps45	6E-01	0.3
Cdk5rap1	9E-01	0.3	Vps72	2E-03	0.3
Gm26944	3E-01	0.3	2610002M06Rik	2E-01	0.3
Scpep1	3E-14	0.3	Crtc1	8E-01	0.3
Sdf2l1	5E-03	0.3	Gm26881	8E-01	0.3
Zfp677	4E-01	0.3	Zfp595	6E-01	0.3
Sdcbp	4E-08	0.3	Znfx1	3E-01	0.3
Sidt2	1E-01	0.3	Gatb	5E-01	0.3
Optn	2E-01	0.3	Trabd2b	3E-03	0.3
Myo1e	2E-01	0.3	Pacs1	1E-01	0.3
Tspan17	8E-03	0.3	Aox1	1E-01	0.3
Inafm1	5E-01	0.3	Nim1k	6E-01	0.3
Nfkbia	3E-02	0.3	Wdtc1	9E-01	0.3
Tbc1d24	5E-01	0.3	Irf9	3E-01	0.3
Cuedc2	4E-08	0.3	Fbn1	1E-12	0.3
Commd4	8E-03	0.3	Ctsz	2E-09	0.3
3010001F23Rik	7E-01	0.3	Rnf114	6E-01	0.3
Map1a	2E-03	0.3	Actr8	7E-01	0.3
Oat	5E-11	0.3	Parp12	7E-01	0.3
Kdm3a	1E+00	0.3	Dclk2	8E-01	0.3
Mib2	7E-01	0.3	Bcl2l2	7E-01	0.3
Hdac5	4E-01	0.3	Rffl	7E-01	0.3
Sfxn4	9E-01	0.3	Ccdc130	9E-01	0.3
Ahnak	7E-16	0.3	Lpgat1	6E-05	0.3
Tapbp	8E-05	0.3	Ifnar2	8E-07	0.3

Limb Mesenchyme

Control			NICD2		
Gene	*p*	FC	Gene	*p*	FC
Clcn3	3E-05	0.3	Zfp260	9E-13	0.3
Stt3b	6E-14	0.3	Dusp14	9E-04	0.3
Faf1	4E-14	0.3	Exog	8E-04	0.3
Cab39	2E-09	0.3	Eipr1	4E-05	0.3
Rnf8	8E-08	0.3	Hmgcr	1E-05	0.3
Lemd3	1E-07	0.3	Me1	1E-14	0.3
Nprl3	2E-06	0.3	Brd7	2E-12	0.3
Hif1an	6E-07	0.3	Ppcs	1E-05	0.3
Pgrmc2	8E-16	0.3	Dhx38	1E-05	0.3
Acp6	1E-05	0.3	Gdf11	3E-07	0.3
Ppan	1E-06	0.3	Saraf	4E-10	0.3
Cgref1	2E-09	0.3	Ostc	4E-20	0.3
Mapk8	4E-06	0.3	Apmap	1E-03	0.3
Chtf8	1E-04	0.3	Ccser2	4E-12	0.3
Tpm2	2E-16	0.3	Pds5a	5E-08	0.3
Cct6a	1E-19	0.3	B3glct	1E-07	0.3
Fgf7	1E-04	0.3	Mcat	3E-04	0.3
Irf2bp2	1E-09	0.3	Apbb3	7E-03	0.3
Kcnq1ot1	6E-10	0.3	Rnf185	3E-06	0.3
Dcun1d2	3E-06	0.3	Gnaq	2E-14	0.3
Isoc1	6E-10	0.3	H13	9E-14	0.3
Gm19710	6E-06	0.3	Zfp324	4E-04	0.3
Slmap	4E-11	0.3	Osbpl9	1E-12	0.3
Eif2b3	9E-09	0.3	Mar6	7E-07	0.3
Ypel1	1E-05	0.3	Rasa1	6E-10	0.3
Slc35a4	6E-08	0.3	Itgb5	7E-10	0.3
Dpp8	9E-09	0.3	Ivd	2E-07	0.3
Car9	6E-06	0.3	Mob2	1E-07	0.3
Alg5	4E-13	0.3	Rae1	1E-08	0.3
Gart	8E-10	0.3	Gm11146	4E-03	0.3
Ofd1	1E-06	0.3	Ssh2	1E-07	0.3
Hcfc2	2E-07	0.3	Cep63	5E-06	0.3
Tmf1	6E-13	0.3	Plagl1	7E-11	0.3
Btaf1	4E-11	0.3	Zfand3	6E-14	0.3
Pkp4	3E-07	0.3	Prss36	3E-05	0.3
Tesk1	8E-07	0.3	Zfp707	1E-04	0.3
Gsk3b	4E-19	0.3	Cct6a	2E-19	0.3
Gnl2	1E-06	0.3	Clic4	8E-15	0.3
Erich1	8E-09	0.3	Caprin1	4E-21	0.3
Trp53cor1	2E-07	0.3	Calcoco1	5E-08	0.3
Zfp983	3E-06	0.3	Chuk	4E-07	0.3
Mettl18	2E-04	0.3	Gmfb	4E-07	0.3
Zbtb11	2E-05	0.3	Ttll11	2E-03	0.3
Csde1	2E-25	0.3	Mark4	5E-05	0.3
Morf4l2	3E-33	0.3	6720427I07Rik	9E-08	0.3
Gm26749	1E-04	0.3	Fzd5	2E-03	0.3
Rab18	8E-25	0.3	Sars	1E-18	0.3
Dpagt1	4E-05	0.3	Ankrd11	4E-19	0.3
Fam49b	3E-08	0.3	Dusp5	3E-04	0.3
4930550C14Rik	3E-03	0.3	Gemin5	2E-06	0.3
Phyh	3E-10	0.3	Ebna1bp2	1E-11	0.3
Cdv3	2E-19	0.3	Nifk	2E-09	0.3
Mbtps2	2E-07	0.3	Sirt1	3E-04	0.3
Cycs	1E-20	0.3	Cmpk1	4E-17	0.3
Mocs3	2E-05	0.3	Tapt1	4E-06	0.3
Smpd4	8E-05	0.3	Pelo	3E-08	0.3
Ankrd52	2E-06	0.3	Cog2	1E-07	0.3
Zfp180	5E-04	0.3	Gsted	2E-03	0.3
Tjp1	1E-11	0.3	Cog1	4E-04	0.3
Usp4	6E-08	0.3	Kpna6	2E-08	0.3
Rap1gap2	5E-03	0.3	Fam20c	4E-04	0.3
Rmnd5b	5E-09	0.3	Aig1	1E-10	0.3
Slc25a22	2E-04	0.3	Bnc2	9E-07	0.2
Uggt2	6E-07	0.3	Evl	9E-08	0.2
Gstm6	1E-02	0.3	Trim44	1E-10	0.2
Ppp3ca	8E-24	0.3	Mar5	4E-17	0.2
Dhx57	4E-07	0.3	Nolc1	3E-10	0.2
Eya4	3E-17	0.3	Mettl14	3E-05	0.2
Ercc6l2	8E-08	0.3	Srsf10	5E-15	0.2
Scly	2E-03	0.3	Strn3	1E-15	0.2
Noc2l	6E-08	0.3	Heatr1	8E-05	0.2
Rell1	2E-06	0.3	Smarca4	1E-10	0.2
E230016M11Rik	3E-05	0.3	Selenoi	2E-05	0.2

Chondrogenic

Control			NICD2		
Gene	*p*	FC	Gene	*p*	FC
Sat1	6E-16	0.3	Vamp7	2E-07	0.3
Adprm	8E-05	0.3	Gnaq	6E-08	0.3
Ppm1b	1E-06	0.3	Araf	1E-07	0.3
Pi15	3E-02	0.3	Zfp13	5E-01	0.3
Clip4	2E-04	0.3	Fra10ac1	7E-05	0.3
Hs6st2	8E-04	0.3	Hnrnph3	3E-10	0.3
Lemd3	8E-03	0.3	Nr6a1	9E-05	0.3
Sept6	2E-02	0.3	Tmem176b	3E-17	0.3
Dynlt3	3E-21	0.3	Calcoco1	1E-02	0.3
1810062O18Rik	1E-02	0.3	Dync2h1	2E-04	0.3
Zfp654	1E-03	0.3	Vta1	1E-07	0.3
Wdr36	4E-08	0.3	Trp53bp1	5E-04	0.3
Oxnad1	1E-01	0.3	Akap11	1E-04	0.3
Serp2	4E-02	0.3	Amotl1	9E-09	0.3
Gm26542	3E-02	0.3	Cbx1	1E-20	0.3
Lcor	6E-09	0.3	Ankrd44	4E-03	0.3
Dph6	3E-08	0.3	Gid4	4E-06	0.3
Dync2h1	7E-08	0.3	Gm5165	3E-01	0.3
Aph1b	2E-02	0.3	C1ql3	9E-01	0.3
Bckdha	2E-04	0.3	Bckdha	1E-03	0.3
Qsox1	2E-10	0.3	Rnf130	6E-08	0.3
Apoo	3E-03	0.3	Bap1	5E-01	0.3
Gm17106	2E-02	0.3	Eml6	4E-03	0.3
Wars2	3E-03	0.3	Zfp867	2E-01	0.3
Nol8	4E-03	0.3	Tmub1	3E-02	0.3
Rfx5	3E-02	0.3	Dbp	6E-03	0.3
Iqcb1	1E-04	0.3	Mppe1	2E-01	0.3
Rcc2	9E-08	0.3	Creb1	2E-04	0.3
Prdm11	3E-01	0.3	Utp14a	3E-03	0.3
Akap11	7E-10	0.3	Pex7	8E-03	0.3
Slc7a1	4E-05	0.3	Rab39b	2E-01	0.3
Pkd1	2E-07	0.3	Dop1b	1E-02	0.3
Hgsnat	1E-04	0.3	Idua	1E-01	0.3
Dnajc15	7E-13	0.3	1600002K03Rik	1E-04	0.3
Kctd15	3E-01	0.3	Tmed1	6E-06	0.3
Mex3a	3E-02	0.3	Gnasas1	3E-01	0.3
Inpp4b	2E-02	0.3	Dnah7b	4E-02	0.3
Zfr	5E-18	0.3	B9d2	9E-02	0.3
Fiz1	2E-03	0.3	Acbd5	2E-07	0.3
Mapre2	3E-07	0.3	Zfp692	3E-01	0.3
Dnaja4	7E-03	0.3	Ly96	9E-04	0.3
Ccdc47	2E-11	0.3	Rcor2	1E-01	0.3
Zfpm1	4E-01	0.3	Nol7	2E-13	0.3
Dmac2	1E-02	0.3	Hoxa13	9E-01	0.3
2810402E24Rik	2E-01	0.3	Zc3h12b	6E-02	0.3
Nfat5	1E-22	0.3	Abhd18	5E-03	0.3
Thbs3	7E-03	0.3	Irak1bp1	2E-06	0.3
1700037C18Rik	2E-01	0.3	Sdf2	4E-16	0.3
Exoc3	9E-20	0.3	Pon2	7E-05	0.3
Psph	5E-04	0.3	Lancl2	8E-01	0.3
Btg1	4E-09	0.3	Smad6	1E-03	0.3
Mccc1	3E-03	0.3	Angel2	4E-05	0.3
Galnt11	1E-05	0.3	Prpsap1	9E-04	0.3
4930557K07Rik	7E-02	0.3	Gbf1	6E-10	0.3
Ube2g2	3E-04	0.3	Glul	7E-02	0.3
Hspbap1	4E-02	0.3	Snhg18	4E-18	0.3
Ctsf	2E-09	0.3	Steap1	7E-01	0.3
Mtif2	5E-09	0.3	Mtmr3	2E-06	0.3
Mks1	1E-01	0.3	Tmpo	4E-14	0.3
Alg2	3E-02	0.3	Abhd14a	2E-08	0.3
Prkab1	1E-02	0.3	Zdhhc20	3E-04	0.3
Hmgn3	2E-29	0.3	Tmem243	2E-02	0.3
Arl2	6E-18	0.3	Nfu1	9E-10	0.3
Ranbp6	5E-02	0.3	Pdp1	4E-03	0.3
Efna5	4E-12	0.3	Lnpep	4E-07	0.3
Zfp248	2E-01	0.3	Dyrk1b	1E-01	0.3
Gm45669	7E-02	0.3	Nmnat1	9E-01	0.3
Adss	2E-12	0.3	Rassf2	5E-02	0.3
Hagh	1E-09	0.3	Zfr	2E-15	0.3
Creld2	8E-08	0.3	Id2	3E-04	0.3
Vamp7	3E-06	0.3	Dstyk	6E-03	0.3
Trp53bp1	8E-05	0.3	Fam135a	8E-03	0.3
Tef	2E-06	0.3	Klc2	2E-01	0.3

Fibroblast

Control			NICD2		
Gene	*p*	FC	Gene	*p*	FC
Irf7	7E-11	0.2	Mga	7E-10	0.2
Tmem38b	2E-05	0.2	Tmem173	3E-04	0.2
Sertad3	8E-10	0.2	Ncln	1E-02	0.2
Mypop	1E-05	0.2	Stox2	3E-09	0.2
Nudt18	9E-06	0.2	Sh3yl1	6E-02	0.2
Rps14	4E-210	0.2	Gprasp1	6E-07	0.2
Ncln	1E-05	0.2	Gm17276	1E-01	0.2
Prkn	3E-13	0.2	Ric1	3E-05	0.2
Mboat7	4E-06	0.2	Efr3a	1E-10	0.2
Serpinb6a	2E-68	0.2	Sptbn1	4E-28	0.2
Foxn3	2E-29	0.2	Lrp3	1E-03	0.2
Kirrel	2E-20	0.2	Ube2w	2E-12	0.2
Acss2	1E-17	0.2	Rhob	9E-08	0.2
Rpl21	3E-173	0.2	Crtap	4E-18	0.2
Map3k14	5E-03	0.2	Igfbp7	8E-90	0.2
Sbf2	2E-21	0.2	Rpl32	4E-100	0.2
Tmem106b	1E-23	0.2	Tfpt	4E-03	0.2
Mb21d2	4E-02	0.2	Zfp729b	1E-02	0.2
Lonrf1	2E-02	0.2	Efna2	2E-02	0.2
Trim21	5E-02	0.2	Rab13	5E-09	0.2
Slc3a2	3E-28	0.2	Pik3r2	1E-04	0.2
Ddr2	9E-24	0.2	Atp8a1	3E-02	0.2
Magi2	9E-17	0.2	Trpm4	1E-01	0.2
Lmf1	2E-07	0.2	Klhl26	3E-03	0.2
Dgka	7E-04	0.2	Lpar1	8E-11	0.2
Inafm1	2E-06	0.2	Zfp709	2E-01	0.2
Tom1l1	3E-02	0.2	Cldnd1	8E-13	0.2
Arfgef2	1E-07	0.2	Gm8739	4E-01	0.2
Syne3	7E-08	0.2	Mustn1	4E-06	0.2
Triobp	4E-06	0.2	Cdc42bpg	1E-01	0.2
Tom1	3E-04	0.2	Acss2	7E-09	0.2
Fam219a	1E-04	0.2	Ltbp2	2E-01	0.2
Vangl1	2E-06	0.2	Rps11	5E-104	0.2
Pdxk	1E-06	0.2	Rps7	6E-97	0.2
1110038B12Rik	2E-49	0.2	Tcea3	4E-13	0.2
Dynlt1f	9E-07	0.2	Ascc3	2E-11	0.2
Rps4x	2E-153	0.2	Maml2	5E-18	0.2
Sept5	7E-02	0.2	Foxn3	5E-15	0.2
Cyba	5E-48	0.2	Rgs19	1E-03	0.2
Nr4a1	3E-02	0.2	Ilvbl	2E-04	0.2
Capn5	9E-02	0.2	Golga3	9E-06	0.2
Zswim7	2E-04	0.2	Parva	5E-13	0.2
Polr3d	4E-13	0.2	Rnf122	4E-01	0.2
Tmtc3	8E-07	0.2	B4galt2	7E-05	0.2
Lrfn3	7E-02	0.2	Polm	2E-01	0.2
Slc38a10	3E-14	0.2	Igf2bp1	2E-02	0.2
Pex1	2E-03	0.2	Acsl4	8E-04	0.2
Tbc1d9b	4E-11	0.2	Gng12	1E-15	0.2
Ier2	1E-11	0.2	Eogt	3E-01	0.2
Rpl9-ps6	1E-12	0.2	Atp6v0d1	3E-16	0.2
Aldh9a1	2E-08	0.2	Gm14326	6E-03	0.2
Efr3a	1E-18	0.2	Aldh9a1	2E-04	0.2
Tmsb15b2	1E-04	0.2	Insyn1	2E-02	0.2
Rpl29	2E-144	0.2	Col6a3	2E-13	0.2
Bod1l	1E-20	0.2	Aga	1E-15	0.2
Akr1b8	2E-10	0.2	Golm1	7E-04	0.2
Galnt9	5E-09	0.2	Aldh4a1	9E-02	0.2
Izumo4	4E-07	0.2	Rps14	4E-104	0.2
Ggh	4E-27	0.2	Bod1l	7E-12	0.2
Psme1	4E-27	0.2	Ttc21b	3E-04	0.2
Zfp9	1E-08	0.2	Psap	4E-40	0.2
Mark4	4E-03	0.2	Ehbp1l1	3E-03	0.2
Retsat	3E-02	0.2	Cad	1E-02	0.2
Rpl3	4E-161	0.2	Mfsd1	3E-06	0.2
Tmco6	3E-01	0.2	Eif3f	5E-34	0.2
Adam15	1E-08	0.2	Mmab	1E-02	0.2
Mmab	2E-06	0.2	Rab26os	2E-02	0.2
Lrrc32	2E-10	0.2	Tbl2	2E-03	0.2
Relch	3E-08	0.2	Pwwp3b	3E-01	0.2
Pcdhga12	2E-03	0.2	Rock2	7E-10	0.2
Rps2	1E-106	0.2	Cdkl3	4E-06	0.2
Bag3	2E-09	0.2	2310014F06Rik	3E-02	0.2
Zfp36	3E-07	0.2	Vwa5a	3E-13	0.2

Fibroblast?

Control			NICD2		
Gene	*p*	FC	Gene	*p*	FC
Nup54	5E-30	0.6	Dhx15	4E-51	0.6
Nfic	2E-64	0.6	Lclat1	2E-28	0.6
Mrps11	6E-28	0.6	Zbtb41	2E-29	0.6
Ubl4a	6E-30	0.6	Cacnb3	6E-20	0.6
Gipc1	6E-25	0.6	Dhrs7b	3E-39	0.6
Tnfaip1	6E-26	0.6	Ubl4a	9E-40	0.6
Apol9a	4E-18	0.6	Psmd13	2E-58	0.6
Cdk6	4E-20	0.6	Vdac1	1E-98	0.6
Nlrc5	2E-14	0.6	Rbm14	4E-27	0.6
Rbm43	3E-21	0.6	Ddb2	6E-18	0.6
Tead4	3E-09	0.6	Arid4a	2E-38	0.6
Ppa1	2E-40	0.6	Paics	4E-60	0.6
Aen	4E-26	0.6	Fam219b	2E-13	0.6
Sema3c	2E-31	0.6	Taf12	2E-43	0.6
Dchs2	2E-10	0.6	Uap1	1E-33	0.6
Nqo2	1E-29	0.6	Slx4ip	4E-29	0.6
Prps1l3	2E-16	0.6	Tap1	4E-25	0.6
Naif1	2E-08	0.6	Hist1h4i	5E-27	0.6
Armcx4	3E-21	0.6	Ess2	3E-20	0.6
Psph	1E-29	0.6	Enpp4	4E-19	0.6
Prrg1	9E-23	0.6	Cavin4	2E-13	0.6
Nfya	2E-24	0.6	Rassf3	1E-26	0.6
Agtpbp1	6E-29	0.6	Ttc9c	9E-35	0.6
Ttll4	1E-25	0.6	Gatad2a	3E-43	0.6
Gna11	1E-49	0.6	Sfpq	1E-55	0.6
Ankrd29	1E-26	0.6	Zbtb18	5E-21	0.6
Tcaf2	6E-14	0.6	4833422C13Rik	1E-03	0.6
Kin	2E-12	0.6	Gm15910	1E-05	0.6
Dnajc10	2E-44	0.6	Zfp180	2E-27	0.6
Map4k2	4E-17	0.6	Actb	4E-110	0.6
Katnbl1	4E-24	0.6	Pitpnm1	5E-18	0.6
Gpank1	4E-22	0.6	Vopp1	8E-30	0.6
Gcnt1	9E-19	0.6	Zfp820	4E-26	0.6
Dcp2	2E-24	0.6	Gpr137b	6E-30	0.6
Actg1	3E-62	0.6	Tmx2	6E-34	0.6
Mapre1	2E-56	0.6	Ppia	4E-253	0.6
Abca8b	9E-14	0.6	Tsen34	9E-71	0.6
Gm2415	3E-07	0.6	Snrpc	4E-44	0.6
Dpysl3	5E-28	0.6	Mrps22	6E-24	0.6
Zfp385a	3E-19	0.6	Khsrp	3E-38	0.6
Bicd2	2E-26	0.6	Emp2	2E-46	0.6
Pgam1	4E-66	0.6	Adarb1	1E-13	0.6
Sipa1l2	1E-20	0.6	Angpt2	3E-30	0.6
Rnf126	1E-24	0.6	Poc5	3E-28	0.6
Pkia	1E-25	0.6	Dkc1	3E-39	0.6
Zdhhc12	2E-21	0.6	Fuom	2E-36	0.6
Gps1	2E-33	0.6	Gm17655	3E-11	0.6
Tmod3	6E-32	0.6	Kdm2b	3E-32	0.6
Bcl7c	2E-50	0.6	Gcnt2	5E-32	0.6
Obsl1	1E-26	0.6	Evl	4E-42	0.6
Mrpl51	9E-69	0.6	Plcl1	2E-42	0.6
Cep78	3E-22	0.6	Cenpb	5E-52	0.6
Lrch4	4E-18	0.6	Grk2	5E-26	0.6
Paip2b	6E-28	0.6	Esyt2	4E-40	0.6
Cep170b	1E-24	0.6	Gm17494	9E-14	0.6
Ncbp1	2E-27	0.6	BC049715	8E-13	0.6
Rprd1b	7E-30	0.6	Gm2115	2E-24	0.6
Adk	2E-48	0.6	Casp2	9E-20	0.6
Wnt5b	2E-22	0.6	Acd	1E-34	0.5
Prkch	3E-11	0.6	Aen	2E-32	0.5
Top3a	2E-25	0.6	L2hgdh	3E-19	0.5
Plcl2	4E-17	0.6	Fam57a	1E-16	0.5
Tesk2	5E-23	0.6	Jag1	5E-29	0.5
Tsen34	5E-53	0.6	Zbtb40	1E-03	0.5
Wdr12	4E-22	0.6	Tmem131l	3E-27	0.5
Cables2	9E-16	0.6	Riox2	7E-22	0.5
Poc1b	1E-19	0.6	Ano3	4E-22	0.5
Cbx1	6E-59	0.6	Pank1	1E-26	0.5
Sap18b	2E-14	0.6	Abhd2	1E-24	0.5
3830408C21Rik	2E-07	0.6	Myo1c	3E-39	0.5
Tnfaip6	7E-30	0.6	Zfp213	3E-18	0.5
Armt1	6E-21	0.6	Ptbp3	1E-45	0.5
Nav1	2E-31	0.6	Tdp1	2E-27	0.5

Undefined

Control			NICD2		
Gene	*p*	FC	Gene	*p*	FC
Mrpl45	2E-01	0.1	Cd276	8E-01	0.1
Nmnat1	9E-01	0.1	Sil1	1E-03	0.1
1810030O07Rik	5E-01	0.1	Slc25a51	9E-01	0.1
Rnf130	2E-02	0.1	Bud13	5E-01	0.1
Ajuba	7E-01	0.1	Rel	7E-01	0.1
Map2	2E-01	0.1	Tec	3E-01	0.1
Fads3	1E-01	0.1	Mif	4E-04	0.1
Cmc1	2E-02	0.1	Hps4	5E-01	0.1
Immp2l	6E-01	0.1	Smim20	9E-02	0.1
Maf1	4E-01	0.1	Fundc2	3E-04	0.1
Rbm12b2	8E-01	0.1	Dus1l	5E-01	0.1
Isg15	1E-05	0.1	Alg12	6E-01	0.1
Taf6l	3E-01	0.1	Taok2	2E-01	0.1
Slc25a3	9E-15	0.1	Aff3	2E-03	0.1
Fopnl	1E-01	0.1	Hif1a	1E-06	0.1
Rbm18	2E-01	0.1	Fbf1	4E-01	0.1
Zcchc10	9E-01	0.1	Gm26797	8E-01	0.1
Gsdme	5E-01	0.1	Aga	5E-05	0.1
Samd14	2E-01	0.1	Pcmtd2	2E-01	0.1
C1galt1c1	6E-01	0.1	Wdr44	8E-01	0.1
Pdcd2	1E-01	0.1	Tasp1	1E-02	0.1
Tmem234	6E-06	0.1	Mapre3	5E-01	0.1
Rmdn1	4E-01	0.1	Bax	1E-05	0.1
Mrpl9	3E-01	0.1	Snta1	1E+00	0.1
Ythdf2	1E+00	0.1	Tmem251	4E-02	0.1
Strn4	8E-01	0.1	Klf9	4E-02	0.1
2900005J15Rik	9E-01	0.1	Acsf2	6E-01	0.1
Rbm4b	7E-01	0.1	Nid2	4E-02	0.1
Zbtb7c	2E-01	0.1	Exoc7	9E-01	0.1
Nprl3	6E-01	0.1	Rhod	9E-01	0.1
Ndufaf5	7E-01	0.1	Mesd	9E-05	0.1
Ak5	3E-02	0.1	Mpv17	5E-01	0.1
Gm12811	7E-01	0.1	Polr2c	7E-02	0.1
Fbxo6	7E-01	0.1	Zfp397	7E-01	0.1
Snhg9	8E-02	0.1	Ntpcr	8E-01	0.1
Tpra1	8E-01	0.1	Mcrip1	6E-02	0.1
Pcbp1	4E-04	0.1	Usp3	4E-01	0.1
Dab2ip	8E-02	0.1	Aasdhppt	8E-01	0.1
Snw1	9E-04	0.1	Phkg2	9E-02	0.1
Ap2s1	2E-03	0.1	Sco1	7E-02	0.1
Riox1	1E+00	0.1	Fbxl15	8E-01	0.1
Mageh1	9E-02	0.1	Fxyd5	4E-03	0.1
Uhrf1bp1l	3E-01	0.1	Zfand6	2E-02	0.1
Bfar	9E-02	0.1	Smo	2E-03	0.1
Hyou1	5E-01	0.1	Kmt2d	1E-01	0.1
Afg3l1	1E-01	0.1	Grb14	2E-02	0.1
Slc6a9	4E-01	0.1	Trmt112	1E-04	0.1
Hprt	4E-01	0.1	Dab2ip	1E+00	0.1
Edrf1	1E+00	0.1	Shkbp1	6E-01	0.1
Brd7	5E-03	0.1	Fads3	1E-01	0.1
AC149090.1	4E-02	0.1	Fcf1	6E-01	0.1
Trabd	6E-01	0.1	H3f3b	7E-22	0.1
Ppid	6E-02	0.1	Gtf2i	6E-03	0.1
Smim14	1E-03	0.1	Nr6a1	4E-01	0.1
Gm44148	9E-01	0.1	Rnf187	3E-01	0.1
Sipa1	4E-01	0.1	Mtln	2E-01	0.1
Zrsr2	4E-01	0.1	Impdh1	6E-01	0.1
Nmi	3E-01	0.1	Skp1a	1E-06	0.1
Tab1	3E-01	0.1	Acyp1	3E-01	0.1
Polr3a	6E-01	0.1	Noc3l	6E-01	0.1
Pcnx4	4E-01	0.1	Hacd3	9E-01	0.1
Glod4	6E-02	0.1	Brix1	5E-01	0.1
Cdip1	7E-01	0.1	Urm1	1E+00	0.1
Ublcp1	7E-01	0.1	Wdr27	4E-01	0.1
Usp30	1E-01	0.1	Zscan26	4E-01	0.1
Zpr1	9E-01	0.1	Rab3a	4E-01	0.1
Ppp3ca	4E-09	0.1	Hsd17b10	1E-03	0.1
Qars	5E-01	0.1	Ndufa6	2E-07	0.1
Arl10	8E-01	0.1	Sdhaf3	2E-01	0.1
Pptc7	8E-01	0.1	Gfm2	5E-01	0.1
Cdk9	7E-01	0.1	Trib3	4E-02	0.1
Cd164	4E-04	0.1	Spsb2	2E-01	0.1
Siae	9E-01	0.1	Dhx40	2E-01	0.1

Articular/Synovial Fibroblast

Control			NICD2		
Gene	*p*	FC	Gene	*p*	FC
Stard5	4E-01	0.3	Plekhb2	8E-01	0.3
mt-Co1	2E-46	0.3	Mlx	4E-01	0.3
Tmem65	5E-03	0.3	Psme1	5E-07	0.3
Cramp1l	7E-01	0.3	Zfp963	8E-01	0.3
Tmem106b	2E-08	0.3	Rfx7	3E-02	0.3
Git2	2E-01	0.3	Gmpr	6E-01	0.3
Gpd2	1E-01	0.3	Akr1b8	2E-01	0.3
4632427E13Rik	3E-03	0.3	Mettl23	7E-03	0.3
Gm5089	8E-01	0.3	Thap3	7E-03	0.3
Dand5	7E-01	0.3	Cttnbp2	2E-01	0.3
Orai3	4E-01	0.3	Jmjd8	6E-01	0.3
Dnajc18	2E-02	0.3	Angptl4	3E-02	0.3
Nars2	1E+00	0.3	Lrp1	4E-11	0.3
Plaat3	6E-07	0.3	Ifngr2	3E-02	0.3
Tm2d2	2E-07	0.3	Rcan1	9E-01	0.3
Cep126	5E-01	0.3	Lpl	9E-12	0.3
Ap4e1	9E-01	0.3	0610040B10Rik	6E-01	0.3
Car13	9E-01	0.3	Ethe1	4E-01	0.3
Psen1	4E-01	0.3	Surf1	2E-01	0.3
Zfp975	1E+00	0.3	Galnt7	9E-01	0.3
mt-Nd3	7E-16	0.3	Zfp951	8E-01	0.3
Anxa11	5E-01	0.3	Unk	1E+00	0.3
Lcmt1	4E-02	0.3	Zfp933	6E-01	0.3
Polr3g	4E-01	0.3	Tex264	8E-03	0.3
Bud13	6E-01	0.3	Lrig1	8E-01	0.3
Irak4	1E+00	0.3	Ints3	6E-01	0.3
mt-Co3	9E-42	0.3	Phf20	1E-02	0.3
Cabin1	2E-01	0.3	Lrp11	3E-01	0.3
Plec	8E-09	0.3	Taco1	8E-01	0.3
Zfp423	4E-01	0.3	Tapbp	3E-03	0.3
A630072M18Rik	1E+00	0.3	Baz2a	4E-01	0.3
Sat2	7E-01	0.3	Gm36975	4E-01	0.3
Lima1	1E-10	0.3	Pan2	7E-01	0.3
Rhobtb2	8E-01	0.3	AW549877	3E-01	0.3
Creb1	2E-02	0.3	Rnf213	4E-02	0.3
C2cd2	7E-01	0.3	Cabin1	1E-01	0.3
Tbrg4	8E-01	0.3	1700084C06Rik	6E-01	0.3
Mnd1	2E-01	0.3	Aldh2	3E-11	0.3
Mmp3	1E-05	0.3	Plcb3	3E-01	0.3
Smyd4	8E-01	0.3	Ncoa2	9E-03	0.3
Stat5b	2E-01	0.3	Atp6v1a	7E-07	0.3
Crppa	4E-01	0.3	Ccng1	6E-07	0.3
Rsf1os1	5E-01	0.3	Trim2	2E-02	0.3
Mllt6	8E-01	0.3	Nid2	1E-01	0.3
Metap1d	3E-03	0.3	Capzb	2E-07	0.3
E130307A14Rik	2E-01	0.3	Endog	4E-01	0.3
Trim12c	1E-01	0.3	Ago4	9E-01	0.3
Sfxn3	2E-01	0.3	Gab2	9E-02	0.3
Saal1	6E-01	0.3	Zfp280b	9E-01	0.3
Dop1b	7E-01	0.3	Ddx41	2E-01	0.3
Slc11a2	4E-01	0.3	Cpd	5E-02	0.3
B3gnt9	2E-01	0.3	Irf2bpl	7E-02	0.3
Lrrc1	5E-01	0.3	Zmynd8	2E-03	0.3
Tmem62	5E-01	0.3	Lysmd4	7E-02	0.3
Pnmal2	8E-01	0.3	Cdon	1E-07	0.3
Slc15a4	9E-01	0.3	CAAA01147332.1	1E+00	0.3
Zfand1	1E-01	0.3	Notch2	1E-02	0.3
Repin1	7E-01	0.3	Zfp869	6E-01	0.3
Parp4	7E-02	0.3	Ino80b	2E-01	0.3
Vps13a	4E-02	0.3	Dusp23	6E-01	0.3
Gm16124	9E-01	0.3	Mtif3	7E-01	0.3
Nxpe4	7E-03	0.3	Ankrd24	3E-01	0.3
Brpf3	9E-01	0.3	Lrrc75a	9E-01	0.3
Ap3d1	1E-02	0.3	Pias4	8E-01	0.3
Aqp1	3E-01	0.3	mt-Co3	7E-30	0.3
Tmem184b	2E-02	0.3	Pink1	6E-02	0.3
Pml	7E-01	0.3	Acap3	5E-01	0.3
Ccdc191	5E-01	0.3	Hdac8	1E-03	0.3
1810062O18Rik	6E-01	0.3	Cisd3	4E-01	0.3
Xpa	2E-03	0.3	Nupl2	5E-01	0.3
Psme1	6E-08	0.3	Sidt2	3E-01	0.3
Vps35l	2E-02	0.3	Vars2	5E-01	0.3
Akt2	1E-03	0.3	Trim68	6E-01	0.3

Limb Mesenchyme

Control			NICD2		
Gene	p	FC	Gene	p	FC
Cebpg	9E-09	0.3	Tmem230	9E-09	0.2
Gm4924	1E-06	0.3	Fhl3	2E-03	0.2
Strap	9E-14	0.3	Hnrnph1	4E-09	0.2
Ifrd1	3E-15	0.3	Trmt61a	8E-03	0.2
Gm47863	8E-05	0.3	Zfp451	6E-08	0.2
Pdrg1	2E-14	0.3	Arih1	7E-12	0.2
Gm17106	6E-07	0.3	Mtmr2	2E-06	0.2
Fam177a	2E-05	0.3	Rbm22	5E-08	0.2
Tgfbr1	4E-07	0.3	Tead1	2E-15	0.2
Amfr	1E-08	0.3	Lrp6	2E-16	0.2
Gm50232	4E-05	0.3	Nim1k	2E-03	0.2
Map4	4E-19	0.3	Timp3	3E-17	0.2
Ddx27	1E-08	0.3	Vmac	8E-03	0.2
Atxn2	1E-10	0.3	Slc39a13	4E-09	0.2
Aff4	5E-17	0.3	Arfip1	4E-09	0.2
Cdk13	2E-09	0.3	Polr2m	3E-11	0.2
Rnaseh1	3E-05	0.3	Pum2	8E-11	0.2
Nrd1	2E-13	0.3	Tasor2	5E-07	0.2
Pdp2	1E-03	0.3	Sec16a	3E-08	0.2
Nucb2	8E-13	0.2	Mcu	1E-08	0.2
Nab2	5E-07	0.2	2810001G20Rik	1E-05	0.2
3110040N11Rik	4E-09	0.2	Kpna1	9E-08	0.2
Supv3l1	4E-05	0.2	Faap24	3E-01	0.2
Stx6	3E-08	0.2	Mir22hg	2E-07	0.2
Pkia	5E-04	0.2	Arid1b	8E-08	0.2
Vamp7	4E-08	0.2	Jkamp	7E-10	0.2
Bms1	8E-08	0.2	Maea	3E-07	0.2
Rcbtb1	3E-07	0.2	Carmil1	9E-08	0.2
Slit3	2E-14	0.2	Dnajc11	2E-06	0.2
Cops7b	7E-06	0.2	Trim33	3E-09	0.2
Yipf5	1E-14	0.2	Necap1	3E-06	0.2
Gm44686	3E-07	0.2	Carns1	4E-03	0.2
Ddhd1	7E-06	0.2	Cpsf2	8E-07	0.2
Wdr24	1E-03	0.2	Srm	1E-10	0.2
Srsf10	2E-15	0.2	Mta1	1E-08	0.2
6330418K02Rik	3E-05	0.2	Tarbp2	3E-05	0.2
Tsc22d3	3E-06	0.2	Smarcd3	1E-07	0.2
Mppe1	2E-05	0.2	Dancr	1E-05	0.2
Lsm14b	2E-05	0.2	Otub2	4E-02	0.2
Dcaf15	7E-03	0.2	Zfp951	5E-03	0.2
Camk2d	1E-12	0.2	Jazf1	8E-05	0.2
Cptp	2E-03	0.2	Ino80d	8E-08	0.2
Prmt1	5E-16	0.2	Mfn1	5E-06	0.2
Slc30a6	1E-06	0.2	Lsm14a	2E-09	0.2
Dhx8	1E-09	0.2	Plxna3	2E-03	0.2
Kif3c	5E-05	0.2	Shf	2E-04	0.2
4933408B17Rik	3E-04	0.2	Smarcd1	2E-09	0.2
Grsf1	3E-10	0.2	Ncbp3	5E-07	0.2
Sfxn2	5E-05	0.2	Slc39a6	2E-05	0.2
Api5	8E-11	0.2	Lrrc58	4E-14	0.2
Foxn3	3E-13	0.2	B4galnt1	2E-04	0.2
6720427I07Rik	8E-09	0.2	Zfp628	6E-03	0.2
Rybp	8E-08	0.2	Zfas1	5E-12	0.2
Ppfia1	1E-10	0.2	Pxdn	2E-09	0.2
Tomm40	4E-09	0.2	Fam98b	6E-10	0.2
Slc35e3	3E-06	0.2	Anapc4	3E-09	0.2
Elp3	5E-07	0.2	Npr2	5E-09	0.2
Mamld1	8E-05	0.2	E230016M11Rik	1E-04	0.2
Arfgef1	2E-10	0.2	Bambi	4E-05	0.2
Trappc11	4E-06	0.2	Ubqln4	3E-04	0.2
Slc35c2	1E-08	0.2	Kcmf1	5E-13	0.2
LTO1	5E-05	0.2	Cdk7	6E-09	0.2
Lgr5	5E-03	0.2	Lrrc20	9E-05	0.2
Snrpb2	4E-10	0.2	Tom1l2	1E-08	0.2
Ascc2	4E-06	0.2	Socs7	8E-04	0.2
Utrn	1E-18	0.2	Gm47071	2E-03	0.2
Ift122	8E-06	0.2	Nexn	3E-04	0.2
Ranbp2	2E-10	0.2	Rpf2	4E-05	0.2
Mllt3	7E-07	0.2	Nat14	1E-04	0.2
Klhl11	2E-05	0.2	Lcor	2E-09	0.2
Uri1	6E-12	0.2	Glt8d2	2E-05	0.2
Gabbr1	6E-09	0.2	Hbs1l	1E-08	0.2
Eif2ak4	1E-07	0.2	Hoxc10	2E-10	0.2

Chondrogenic

Control			NICD2		
Gene	p	FC	Gene	p	FC
Slc25a14	1E-01	0.3	Slc35a1	8E-04	0.3
Rcbtb1	1E-06	0.3	Nfe2l1	3E-20	0.3
Nubpl	1E-02	0.3	Ccdc39	3E-01	0.3
Zfp141	7E-02	0.3	Tgfb3	1E-07	0.3
D130017N08Rik	2E-02	0.3	Snx15	1E-03	0.3
Ergic1	4E-11	0.3	Qsox2	6E-02	0.3
Yju2	1E-01	0.3	Zfp930	5E-01	0.3
Clip1	1E-09	0.3	Zfp513	3E-01	0.3
Mpped2	1E-01	0.3	Parp4	3E-04	0.3
Arl3	8E-24	0.3	Atat1	6E-02	0.3
Vps72	6E-09	0.3	Btaf1	2E-05	0.3
Xpot	1E-07	0.3	Arl3	2E-21	0.3
Slc25a13	1E-01	0.3	Lpcat1	7E-01	0.3
Ice2	1E-02	0.3	Ppp1r10	2E-08	0.3
Rufy1	5E-03	0.3	Gm9993	2E-01	0.3
Pnrc2	7E-11	0.3	Vps37d	9E-01	0.3
Rbmx	9E-03	0.3	Ssbp1	2E-11	0.3
Kcnq1ot1	3E-10	0.3	Asb3	8E-04	0.3
Zfr2	6E-03	0.3	Lrp12	3E-01	0.3
Slc7a5	2E-03	0.3	Rabac1	3E-23	0.3
Arhgap31	2E-03	0.3	Gm12353	6E-02	0.3
Traf3ip1	2E-02	0.3	Zfp280d	8E-08	0.3
Rnf114	1E-02	0.3	Tma16	2E-01	0.3
Hdhd5	2E-01	0.3	Naa60	3E-03	0.3
P3h1	4E-06	0.3	Prorsd1	1E-06	0.3
Mnat1	5E-14	0.3	Rabl3	2E-01	0.3
Naa60	5E-04	0.3	Zfp51	5E-01	0.3
Ttc39b	9E-05	0.3	Ulk1	1E-01	0.3
Tspyl1	1E-07	0.3	Cog5	1E-04	0.3
Avl9	3E-03	0.3	L3mbtl2	5E-01	0.3
Spata6	5E-03	0.3	Map3k8	6E-01	0.3
2410022M11Rik	4E-02	0.3	Desi2	1E-12	0.3
Sec14l1	2E-08	0.3	Rbmx	3E-03	0.3
Taf8	1E-01	0.3	Fam185a	5E-01	0.3
Traf3ip2	8E-03	0.3	Zfp707	7E-01	0.3
Klhdc8b	7E-02	0.3	Sdf4	2E-17	0.3
Clk1	3E-08	0.3	D830050J10Rik	1E-02	0.3
Eefsec	4E-02	0.3	Cib2	3E-05	0.3
Pecr	5E-03	0.3	Selenoo	2E-01	0.3
Fra10ac1	3E-05	0.3	Tut1	1E-01	0.3
Ano5	9E-02	0.3	Dyrk1a	2E-05	0.3
Gm15738	7E-01	0.3	Pcnx	8E-04	0.3
Eif4ebp3	1E+00	0.3	Pef1	1E-02	0.3
Vps39	4E-02	0.3	Jmjd8	6E-02	0.3
Phf7	3E-02	0.3	Rnf220	3E-02	0.3
Gna12	1E-03	0.3	Ccdc173	2E-01	0.3
Ext2	1E-14	0.3	D230025D16Rik	7E-01	0.3
Sec11c	4E-06	0.3	Mettl14	1E-01	0.3
Parp8	1E-05	0.3	Afg3l2	1E-02	0.3
Xpc	2E-02	0.3	Serf1	2E-10	0.3
Mlh1	2E-01	0.3	Atg2a	2E-01	0.3
Pi4k2a	1E-06	0.3	Zfp281	3E-02	0.2
Pkp4	4E-05	0.3	Gphn	6E-10	0.2
Wbp1	6E-05	0.3	Serp2	1E-01	0.2
Ift52	1E-05	0.3	Nol11	8E-05	0.2
Hspa5	3E-29	0.3	Asf1a	8E-04	0.2
Zfp317	5E-03	0.3	Ric8b	2E-01	0.2
Sdf4	1E-28	0.3	Fam98c	1E-01	0.2
Dad1	4E-32	0.3	Aar2	7E-02	0.2
Zfpm2	1E-12	0.3	Gm47863	3E-01	0.2
Mrpl44	1E-04	0.3	Gabbr1	2E-03	0.2
Gtpbp1	4E-01	0.3	Mar8	9E-04	0.2
Pacrg	4E-01	0.3	Pfkm	9E-02	0.2
Mplkip	3E-06	0.3	Mfsd4a	8E-02	0.2
1700030K09Rik	3E-02	0.3	2310010J17Rik	2E-04	0.2
Ptprk	8E-04	0.3	Pias2	1E-04	0.2
Pex3	2E-03	0.3	Slx1b	1E-01	0.2
Ric8b	4E-02	0.3	Fam53b	2E-02	0.2
Ppt1	3E-11	0.3	Gtpbp2	2E-01	0.2
Tctex1d2	1E-05	0.3	Tmem161b	3E-01	0.2
Twnk	1E-01	0.3	Sdcbp	2E-11	0.2
Zfp40	3E-01	0.3	Iffo2	4E-01	0.2
Lrrc29	6E-02	0.3	Satb2	4E-01	0.2

Fibroblast

Control			NICD2		
Gene	p	FC	Gene	p	FC
Tnfrsf1b	3E-03	0.2	Abca5	1E-02	0.2
Prcp	1E-07	0.2	Hmgcr	4E-06	0.2
C4b	4E-15	0.2	C920021L13Rik	3E-01	0.2
Sbno2	3E-09	0.2	Scpep1	4E-18	0.2
Igfbp7	9E-153	0.2	Sertad3	6E-06	0.2
Als2	2E-02	0.2	Plxna3	7E-01	0.2
C030014I23Rik	5E-05	0.2	Fzd2	2E-11	0.2
Cept1	2E-08	0.2	Snhg15	9E-05	0.2
Gramd3	5E-06	0.2	Tom1	4E-03	0.2
Gm10069	5E-01	0.2	Fbxo32	5E-03	0.2
Dusp23	7E-07	0.2	Igf2bp3	4E-07	0.2
Mex3c	5E-06	0.2	Arhgef10	2E-04	0.2
Rhob	4E-13	0.2	D2hgdh	2E-01	0.2
Epb41l4aos	4E-10	0.2	Tbc1d17	2E-04	0.2
Abcb10	3E-03	0.2	Ankrd24	5E-03	0.2
Crtap	5E-30	0.2	Slc7a6	4E-02	0.2
1700096K18Rik	2E-03	0.2	Dnm1	2E-09	0.2
Bmyc	1E-19	0.2	Tmem41a	9E-03	0.2
Ctnnb1	2E-27	0.2	Rpl11	3E-88	0.2
Sft2d1	3E-05	0.2	Gstm1	1E-23	0.2
Slc2a13	8E-05	0.2	Cltc	3E-26	0.2
Svbp	2E-35	0.2	Rps6ka5	1E-01	0.2
Arsb	7E-06	0.2	Abhd18	6E-04	0.2
Dgkh	2E-02	0.2	Tmem256	6E-27	0.2
Rpl23	1E-207	0.2	Trip10	1E-02	0.2
Tgds	1E-05	0.2	Adamts10	5E-06	0.2
Numb	5E-10	0.2	Klf6	7E-10	0.2
Acsl4	5E-08	0.2	Gm14305	1E-03	0.2
Tmem192	2E-06	0.2	Large1	2E-12	0.2
Plxna1	1E-04	0.2	Sfxn4	2E-01	0.2
Stat6	3E-07	0.2	Gabarap	3E-59	0.2
Hectd3	2E-02	0.2	Fbxl19	7E-02	0.2
Sptbn1	4E-50	0.2	Hook3	1E-15	0.2
Cast	2E-33	0.2	Lss	5E-03	0.2
Calml4	2E-05	0.2	Adarb1	4E-03	0.2
Rpl30	6E-170	0.2	Stat5b	3E-04	0.2
Acad9	2E-03	0.2	Ano8	2E-01	0.2
Ech1	1E-33	0.2	Kdm1b	1E-01	0.2
Vps9d1	3E-02	0.2	Man2a1	4E-07	0.2
Nfkb1	2E-09	0.2	Rpsa	2E-65	0.2
Smyd3	7E-10	0.2	Angpt1	6E-07	0.2
Cavin1	6E-29	0.2	Stat6	2E-05	0.2
Tspyl2	4E-07	0.2	Fam160b1	1E-05	0.2
Dlg5	1E-06	0.2	Fau	1E-87	0.2
Slfn8	1E-03	0.2	Slc31a2	4E-02	0.2
BC034090	2E-01	0.2	Ripor1	3E-03	0.2
Runx2os1	4E-01	0.2	Kdelr1	2E-24	0.2
Zfp318	1E-08	0.2	Zfp36	1E-03	0.2
Bet1l	1E-24	0.2	Slfn8	1E-01	0.2
Fam91a1	2E-08	0.2	Plxna1	1E-02	0.2
Sec23ip	3E-08	0.2	Surf4	3E-18	0.2
Wdr89	4E-12	0.2	Vti1b	3E-13	0.2
Hdac4	9E-05	0.2	Zfp994	7E-03	0.2
Fam160b1	9E-07	0.2	Pfkfb3	8E-05	0.2
Epb41l2	3E-19	0.2	Vps41	2E-12	0.2
St6galnac6	3E-05	0.2	Mill2	7E-05	0.2
Ttll5	2E-04	0.2	Rps13	7E-88	0.2
Dctn6	4E-14	0.2	Ttc7b	5E-04	0.2
Tuba4a	6E-04	0.2	Nus1	3E-10	0.2
Appl1	3E-15	0.2	Selenom	7E-22	0.2
Dnmbp	2E-04	0.2	Cd47	8E-16	0.2
Leng8	3E-05	0.2	Vapb	3E-16	0.2
Zfp994	1E-04	0.2	Smyd3	3E-07	0.2
BC017158	3E-02	0.2	Tspan14	1E-01	0.2
Rpl24	5E-171	0.2	Snx4	2E-13	0.2
Nfix	2E-53	0.2	Gm14410	1E-01	0.2
Etv6	9E-10	0.2	Srpx2	3E-23	0.2
Clec2d	2E-13	0.2	Map7d1	3E-07	0.2
Rgs2	3E-04	0.2	Surf1	4E-06	0.2
Nudt7	3E-05	0.2	Med10	1E-09	0.2
Ap3b1	7E-18	0.2	Neu1	6E-04	0.2
Mzt2	7E-09	0.2	Cpne3	7E-11	0.2
Abcb1b	9E-03	0.2	Btbd2	8E-03	0.2

Fibroblast?

Control			NICD2		
Gene	p	FC	Gene	p	FC
Prelid3b	7E-32	0.6	S100a6	1E-95	0.5
Pakap.1	4E-37	0.6	Cdk2ap1	5E-37	0.5
Wdr78	2E-20	0.6	Arid5a	8E-19	0.5
Slc2a9	2E-13	0.6	Ints10	1E-29	0.5
Dclk2	9E-10	0.6	Wnt5b	4E-40	0.5
Pla1a	8E-09	0.6	Atr	3E-33	0.5
Mettl9	1E-47	0.6	Hnrnpu	1E-104	0.5
Trim16	2E-19	0.6	Mettl24	1E-15	0.5
Phldb1	1E-19	0.6	Prps1l3	3E-16	0.5
Rad1	6E-24	0.6	Ctcf	7E-61	0.5
Itih5	6E-15	0.6	Slc1a6	4E-23	0.5
Gpn2	3E-13	0.6	Cptp	2E-16	0.5
Usp14	4E-53	0.6	Prpf31	7E-38	0.5
Brms1l	6E-24	0.6	Rpusd1	1E-22	0.5
Gng3	1E-08	0.6	Fst	2E-30	0.5
Rin1	3E-15	0.6	Mapre1	4E-69	0.5
Bcat1	8E-25	0.6	Taf1d	3E-41	0.5
Rcan3	5E-24	0.6	Dubr	9E-22	0.5
Lmna	4E-78	0.6	Apip	2E-43	0.5
Mpi	7E-15	0.6	Zdhhc3	2E-38	0.5
Asap2	6E-25	0.5	Slc35a3	4E-32	0.5
Psmd5	3E-25	0.5	Mettl14	4E-19	0.5
Rgs10	9E-39	0.5	Spcs3	1E-32	0.5
Ctcf	1E-38	0.5	Actn4	3E-62	0.5
Sf1	6E-34	0.5	U2af2	3E-44	0.5
Galnt1	2E-30	0.5	Osbpl6	7E-34	0.5
Dbndd2	1E-12	0.5	Actl6a	1E-38	0.5
Ndufaf2	5E-38	0.5	Pgam1	2E-73	0.5
Trim27	1E-29	0.5	Platr22	7E-13	0.5
Aida	7E-38	0.5	Nxt2	3E-26	0.5
Tpgs2	8E-22	0.5	Slc22a15	7E-19	0.5
Apaf1	2E-21	0.5	1700066M21Rik	2E-15	0.5
Zfand2a	5E-16	0.5	Nmt2	9E-31	0.5
S1pr3	6E-11	0.5	Pm20d1	1E-08	0.5
Fnbp1l	1E-23	0.5	Abracl	7E-52	0.5
Otud4	5E-19	0.5	Msantd3	8E-33	0.5
E2f3	2E-21	0.5	Ubac1	3E-30	0.5
Basp1	9E-26	0.5	Ythdc2	8E-27	0.5
Wrn	6E-27	0.5	Tbc1d4	7E-30	0.5
Caprin2	4E-14	0.5	Gcfc2	6E-30	0.5
Rab27b	2E-17	0.5	Col15a1	1E-22	0.5
Setd6	2E-17	0.5	Phlda1	5E-28	0.5
Ccdc117	1E-19	0.5	Ccdc167	3E-27	0.5
Ets1	6E-33	0.5	Lpin2	2E-29	0.5
Actn4	1E-54	0.5	Tmem183a	3E-33	0.5
Prdx1	2E-56	0.5	Brpf1	1E-24	0.5
Sox12	2E-14	0.5	Pacc1	3E-18	0.5
Gal3st4	8E-09	0.5	Fam149a	4E-16	0.5
Heg1	3E-20	0.5	Hspd1	3E-71	0.5
Mrps25	3E-37	0.5	Ccdc117	5E-27	0.5
Bcl2	1E-13	0.5	Mrpl12	3E-52	0.5
Aimp2	3E-29	0.5	Zbed4	4E-20	0.5
Psd3	3E-37	0.5	Pcyt1b	3E-14	0.5
Trpt1	6E-06	0.5	Btbd10	6E-26	0.5
Lsm4	1E-59	0.5	Rbm10	1E-30	0.5
Impdh2	1E-45	0.5	4933408B17Rik	8E-17	0.5
Hnrnpul1	2E-29	0.5	Adgrd1	2E-13	0.5
Exog	5E-19	0.5	Mpv17l2	2E-39	0.5
Taf6	5E-27	0.5	Cotl1	4E-30	0.5
Dpp4	3E-09	0.5	Mrpl14	1E-68	0.5
Stk35	2E-21	0.5	Lama5	6E-12	0.5
Eea1	3E-33	0.5	Tmem209	4E-25	0.5
Apip	2E-31	0.5	Tirap	1E-08	0.5
Egln1	1E-27	0.5	2310022A10Rik	5E-27	0.5
Taf12	4E-33	0.5	Tmod2	9E-16	0.5
Ppm1m	3E-24	0.5	Slco2a1	1E-19	0.5
Eif2s1	1E-37	0.5	Fhl1	4E-18	0.5
Nutf2	1E-08	0.5	Adsl	5E-24	0.5
Uck2	2E-23	0.5	6030442K20Rik	4E-18	0.5
Mllt1	2E-19	0.5	Senp2	7E-29	0.5
Slc25a5	1E-47	0.5	Stard4	3E-36	0.5
Ppia	3E-181	0.5	Tcp1	3E-78	0.5
Chpf	2E-27	0.5	Elof1	1E-46	0.5

Undefined

Control			NICD2		
Gene	p	FC	Gene	p	FC
1700109H08Rik	4E-01	0.1	Nbl1	3E-01	0.1
Akr7a5	7E-02	0.1	Wdr53	9E-01	0.1
Polrmt	8E-01	0.1	Mettl1	9E-01	0.1
Rex1bd	2E-09	0.1	Lypla2	2E-01	0.1
Got1	9E-01	0.1	Tsg101	4E-02	0.1
Dnttip2	2E-01	0.1	Mob2	6E-01	0.1
Nt5dc1	2E-01	0.1	Trappc6a	3E-01	0.1
Sirt3	6E-01	0.1	C1galt1	6E-01	0.1
Bbc3	9E-01	0.1	Acadm	1E-07	0.1
Tia1	5E-02	0.1	Rbbp9	8E-01	0.1
4930481A15Rik	9E-01	0.1	Arid5b	2E-04	0.1
Clec11a	3E-03	0.1	Tbc1d7	3E-01	0.1
Cacul1	6E-01	0.1	Mcl1	2E-01	0.1
Apmap	9E-01	0.1	Fahd2a	5E-01	0.1
Zfp647	5E-01	0.1	Nkiras2	2E-01	0.1
Prpsap1	6E-01	0.1	Fahd1	9E-01	0.1
Thrb	6E-01	0.1	0610009E02Rik	5E-01	0.1
Meis1	1E+00	0.1	Fam131a	9E-01	0.1
Med13	3E-01	0.1	Zfp444	7E-01	0.1
4930517O19Rik	7E-01	0.1	Nsun5	3E-01	0.1
Ddhd1	8E-01	0.1	Dhx36	3E-02	0.1
Eno2	6E-01	0.1	Anxa5	1E-06	0.1
Atp6ap1	2E-03	0.1	Ptcd3	9E-01	0.1
Lamtor5	6E-06	0.1	Dync2li1	7E-01	0.1
Sfxn4	1E+00	0.1	Smg9	5E-02	0.1
Zfyve1	4E-01	0.1	Tfcp2	7E-01	0.1
Prpf19	1E+00	0.1	Ist1	5E-01	0.1
Cnpy4	1E-01	0.1	Dhx37	6E-01	0.1
Ogt	4E-03	0.1	Anxa1	3E-02	0.1
Scnm1	5E-01	0.1	Ip6k1	6E-01	0.1
Rrp1	2E-05	0.1	Hagh	7E-01	0.1
Dcaf12	6E-01	0.1	Gm19710	6E-01	0.1
Vta1	6E-02	0.1	Prkci	5E-01	0.1
Cd2bp2	9E-01	0.1	Echdc3	9E-01	0.1
Gpatch2l	1E+00	0.1	Tpcn1	4E-01	0.1
Mthfsl	8E-01	0.1	Gar1	5E-01	0.1
Mrps31	8E-01	0.1	Pmpcb	5E-03	0.1
Otud4	6E-01	0.1	Sumo1	7E-04	0.1
Gm14418	1E-01	0.1	Wbp11	8E-01	0.1
Chst14	9E-01	0.1	Pdcd6	8E-04	0.1
Top3a	2E-03	0.1	Vwa8	1E-01	0.1
Pced1a	4E-01	0.1	Wdr41	8E-01	0.1
Nucb1	3E-05	0.1	Kbtbd4	1E-01	0.1
Mtr	4E-01	0.1	Gatd1	6E-01	0.1
Zscan29	1E+00	0.1	mt-Co1	6E-35	0.1
Phax	1E-02	0.1	Mrps23	7E-01	0.1
Slc1a4	2E-01	0.1	A430005L14Rik	3E-01	0.1
Phtf1	5E-01	0.1	Drg2	1E-01	0.1
Ikbkb	7E-01	0.1	Trabd	7E-01	0.1
Cdipt	6E-01	0.1	Chchd2	7E-23	0.1
Gm15283	1E-01	0.1	Dbt	3E-01	0.1
Grb2	1E-01	0.1	Cyld	8E-01	0.1
Ttc4	7E-01	0.1	Coa6	8E-01	0.1
Ptpn21	4E-01	0.1	Acad10	9E-01	0.1
Rpap3	9E-02	0.1	Tgfbr2	2E-01	0.1
Nr2c1	5E-01	0.1	Azi2	5E-01	0.1
Trappc2	4E-01	0.1	Zfyve26	1E+00	0.1
Rere	3E-04	0.1	Txnip	2E-01	0.1
Tmem29	2E-01	0.1	Med20	5E-01	0.1
Guk1	5E-02	0.1	Ecrg4	6E-15	0.1
Atf1	4E-01	0.1	AW146154	1E-01	0.1
Tm7sf3	3E-01	0.1	Ola1	8E-03	0.1
Aldh1l2	6E-01	0.1	Plekhh2	6E-03	0.1
Vopp1	1E+00	0.1	Txnrd2	9E-01	0.1
C2cd3	7E-01	0.1	Sall2	5E-03	0.1
Exosc1	4E-01	0.1	Cebpz	8E-02	0.1
Trafd1	9E-02	0.1	Dpm1	1E-03	0.1
Cuedc2	3E-02	0.1	Slc15a4	7E-02	0.1
Sdccag8	8E-02	0.1	Lztfl1	6E-02	0.1
Chrdl1	9E-02	0.1	Hltf	3E-01	0.1
Atg12	3E-01	0.1	Abhd14a	9E-02	0.1
Prmt2	7E-01	0.1	Mbtd1	3E-02	0.1
Ist1	4E-01	0.1	Pigq	3E-01	0.1

Articular/Synovial Fibroblast

Control			NICD2		
Gene	p	FC	Gene	p	FC
Vit	2E-01	0.3	Lncpint	5E-02	0.3
BC017158	6E-01	0.3	Sertad3	4E-01	0.3
Oxct1	3E-07	0.3	Mrps18a	8E-04	0.3
Mef2b	8E-01	0.3	Eif3f	7E-14	0.3
Zfp710	5E-01	0.3	E430024I08Rik	6E-01	0.3
Tmem98	3E-01	0.3	Morc4	7E-01	0.3
Gm12353	9E-01	0.3	Ano5	4E-01	0.3
Rbms1	4E-12	0.3	Nudt9	1E-01	0.3
mt-Co2	1E-53	0.3	Afg1l	7E-01	0.3
Fam76a	5E-05	0.3	Patj	9E-01	0.3
Sdc2	2E-07	0.3	Snx12	6E-03	0.3
Cln6	8E-01	0.3	Anxa1	3E-12	0.3
Gas6	7E-13	0.3	Klhl15	7E-01	0.3
Nphp3	5E-01	0.3	Lrfn3	5E-01	0.3
Plaur	1E+00	0.3	Klc4	7E-01	0.3
Atxn7l1	2E-01	0.3	Cep19	5E-01	0.3
Mgmt	2E-04	0.3	Synj2	8E-01	0.3
Lmtk2	8E-01	0.3	Tmem106b	8E-05	0.3
Rnf25	3E-02	0.3	Ptpn3	5E-01	0.3
Lrrk2	8E-01	0.3	Cmc1	4E-05	0.3
Axl	2E-01	0.2	Zc3h12b	6E-02	0.3
Zfp229	9E-01	0.2	Hhat	8E-01	0.3
Adprm	9E-01	0.2	Vps28	4E-03	0.3
Adnp2	6E-01	0.2	Tspyl2	3E-01	0.3
Egfl7	6E-01	0.2	Sgsm3	8E-01	0.3
D330041H03Rik	1E+00	0.2	Fam214b	8E-01	0.3
Parp6	7E-01	0.2	Mpi	5E-01	0.3
Igf2bp3	4E-02	0.2	Atp2c1	3E-01	0.3
Zfp626	5E-01	0.2	Gm20721	9E-01	0.3
Zbtb7a	4E-05	0.2	Vps37d	1E+00	0.3
Pacs1	2E-01	0.2	mt-Nd5	8E-17	0.3
Poli	4E-01	0.2	Svil	2E-05	0.3
Usp28	3E-01	0.2	Mnd1	7E-02	0.3
Klf3	1E-04	0.2	Ccdc32	4E-01	0.3
Kat2a	8E-01	0.2	Prcc	1E+00	0.3
Thap4	1E+00	0.2	Pip4p1	1E-01	0.3
Ago1	7E-01	0.2	Abcc5	3E-03	0.3
Cnbd2	6E-01	0.2	Ifit2	5E-01	0.3
Zfp994	5E-01	0.2	Coq10a	9E-01	0.3
Nox4	2E-01	0.2	Prkce	2E-02	0.3
Zhx1	2E-02	0.2	Fth1	4E-23	0.3
Ahdc1	6E-02	0.2	Idh3b	3E-03	0.3
Clec16a	3E-01	0.2	Zscan26	2E-01	0.3
Lamb1	6E-05	0.2	Bach1	7E-01	0.3
Zfyve1	4E-01	0.2	Pgap2	8E-02	0.3
Arhgap10	3E-02	0.2	Ppp1r16a	4E-01	0.3
Tex264	5E-07	0.2	Nfat5	2E-06	0.3
Tbc1d17	9E-02	0.2	Npepps	1E-04	0.3
Nipsnap2	8E-03	0.2	Zfp36l2	9E-06	0.3
2310010J17Rik	1E-01	0.2	Narf	7E-01	0.3
Rab3gap2	1E-02	0.2	Kremen1	1E+00	0.3
Mapk1ip1	8E-01	0.2	Agrp	3E-01	0.3
Foxo1	7E-01	0.2	Rabep2	9E-01	0.3
Elf4	1E-01	0.2	Gm14296	8E-01	0.3
Sdsl	6E-01	0.2	Gpatch1	6E-01	0.3
Cand2	6E-01	0.2	Wdr19	1E+00	0.3
Rdh5	9E-01	0.2	Gprasp1	8E-02	0.3
Aldh9a1	2E-01	0.2	Yju2	6E-01	0.3
Mtrf1l	8E-01	0.2	Ssbp2	3E-02	0.3
Flii	8E-03	0.2	Nemf	1E-03	0.3
Prune1	5E-01	0.2	Wbp11	7E-02	0.3
Tbkbp1	6E-01	0.2	Mrrf	2E-01	0.3
Abca2	3E-01	0.2	Anxa11	5E-01	0.2
Nhlrc2	2E-01	0.2	Dgka	6E-01	0.2
Psmb10	1E-01	0.2	2700097O09Rik	8E-01	0.2
BC029722	5E-01	0.2	Apcdd1	3E-01	0.2
Cep89	7E-01	0.2	Eif2b4	3E-01	0.2
Rab40c	8E-01	0.2	Hdac7	4E-01	0.2
Serhl	3E-01	0.2	Ddt	4E-07	0.2
2310039H08Rik	1E-02	0.2	Pex12	4E-01	0.2
Anks6	5E-01	0.2	Mov10	1E+00	0.2
mt-Nd4	6E-30	0.2	Atp13a2	9E-01	0.2
Pet100	1E-06	0.2	Spred3	9E-01	0.2

Limb Mesenchyme

Control			NICD2		
Gene	p	FC	Gene	p	FC
Mbnl1	3E-16	0.2	Lrrc41	6E-08	0.2
Rab2a	5E-43	0.2	Gnl3	4E-08	0.2
Tmem154	7E-04	0.2	Rab6a	3E-13	0.2
Nek9	5E-11	0.2	E530011L22Rik	2E-05	0.2
Fam135a	5E-08	0.2	Rbm6	4E-09	0.2
Zfp280c	3E-07	0.2	4933407K13Rik	2E-03	0.2
Nbea	5E-12	0.2	Paip1	1E-11	0.2
Ppp4r1	5E-06	0.2	Trim39	6E-05	0.2
Gpr137c	3E-03	0.2	Morc2a	3E-07	0.2
Plekhf2	1E-04	0.2	Raly	1E-12	0.2
Vasp	4E-06	0.2	Bcar1	2E-04	0.2
Cacfd1	6E-07	0.2	Itih2	7E-08	0.2
Zfp451	3E-08	0.2	Bbs4	7E-03	0.2
Plekha3	2E-10	0.2	Eral1	9E-02	0.2
Col9a3	2E-21	0.2	Srpr	1E-14	0.2
Plxna3	9E-05	0.2	Gm36198	5E-03	0.2
Hpgds	8E-08	0.2	Mapk1ip1l	5E-08	0.2
Ssr2	1E-11	0.2	Mb21d2	7E-04	0.2
Rnf19a	1E-08	0.2	Pdk1	3E-05	0.2
Gpatch2	4E-07	0.2	Abi2	1E-06	0.2
Kcmf1	2E-13	0.2	Rbm15b	6E-05	0.2
Zfp334	4E-04	0.2	Plaa	1E-07	0.2
Plcg1	2E-06	0.2	Rab2a	9E-37	0.2
Ramac	8E-13	0.2	Tmem223	4E-12	0.2
Exoc6b	2E-11	0.2	Cmtr1	2E-07	0.2
Gorasp2	1E-12	0.2	Sgms2	4E-07	0.2
Stk39	3E-06	0.2	Tceanc2	5E-08	0.2
Plekhm3	1E-06	0.2	Slc4a3	9E-04	0.2
Stk16	2E-09	0.2	Eaf2	4E-04	0.2
Cnpy3	3E-08	0.2	Tspan6	4E-08	0.2
Gm38604	9E-05	0.2	Tia1	2E-10	0.2
Gtf2f2	2E-09	0.2	Phf12	1E-05	0.2
Utp18	3E-08	0.2	Kmt5b	1E-08	0.2
Cops9	2E-19	0.2	Wipi2	6E-06	0.2
Map2k3os	1E-02	0.2	Klf2	1E-07	0.2
Arpc5l	3E-18	0.2	Fbh1	1E-06	0.2
Ttc27	1E-06	0.2	Gsk3b	2E-16	0.2
Dync1li1	2E-08	0.2	Actr1a	8E-08	0.2
Rin3	6E-02	0.2	Snhg1	2E-09	0.2
Dnaaf4	4E-03	0.2	Pogk	3E-06	0.2
Napb	8E-05	0.2	E2f3	2E-07	0.2
Slc35e1	3E-07	0.2	Rnf111	1E-07	0.2
Fkbp9	2E-16	0.2	Recql5	2E-04	0.2
Nepro	6E-03	0.2	C330013E15Rik	2E-02	0.2
Zbtb41	6E-07	0.2	Pwp1	2E-07	0.2
Rab6a	2E-13	0.2	Micu3	1E-06	0.2
Surf4	8E-17	0.2	Prpf40b	1E-04	0.2
Vopp1	6E-04	0.2	Nab2	1E-07	0.2
Mtap	2E-10	0.2	Ccdc91	3E-05	0.2
B4galt7	2E-05	0.2	Tm9sf2	1E-17	0.2
Nol6	1E-06	0.2	Bloc1s3	6E-03	0.2
Mob3a	7E-05	0.2	Sema4c	5E-04	0.2
Ccdc8	3E-05	0.2	Trappc4	4E-17	0.2
Ubox5	2E-05	0.2	Maml2	7E-10	0.2
Ppat	9E-06	0.2	Mbnl1	1E-14	0.2
Habp4	2E-07	0.2	Myo1c	2E-07	0.2
8030451A03Rik	1E-12	0.2	Ankzf1	1E-05	0.2
Slc25a46	4E-08	0.2	Mrs2	6E-06	0.2
Oxnad1	2E-06	0.2	Golgb1	2E-19	0.2
Sntb2	1E-12	0.2	D230025D16Rik	5E-04	0.2
Ebna1bp2	4E-10	0.2	Zdhhc9	1E-05	0.2
Ddx49	5E-07	0.2	Pafah1b1	2E-25	0.2
Fn1	1E-23	0.2	Rnf121	4E-05	0.2
Rnf20	2E-08	0.2	Arfgap1	4E-08	0.2
Mdn1	2E-06	0.2	Mtrf1l	5E-05	0.2
Jmy	3E-07	0.2	Fzd2	2E-14	0.2
Shkbp1	2E-04	0.2	Mcl1	3E-12	0.2
Snai2	8E-13	0.2	Fam210a	3E-06	0.2
Elavl1	6E-12	0.2	Dgcr6	1E-10	0.2
Snx18	5E-07	0.2	AW209491	1E-04	0.2
Zbtb26	7E-05	0.2	Ankrd16	4E-06	0.2
Tent4a	1E-04	0.2	Rhot2	6E-04	0.2
Bet1	2E-12	0.2	Akt1s1	4E-08	0.2

Chondrogenic

Control			NICD2		
Gene	p	FC	Gene	p	FC
Strada	2E-02	0.3	Trdmt1	1E-01	0.2
Nfu1	5E-10	0.3	Alg12	3E-01	0.2
Tram2	3E-06	0.3	Dnajc4	1E-01	0.2
Izumo4	6E-04	0.3	Spire1	4E-03	0.2
Ramac	3E-08	0.3	Coq9	2E-03	0.2
Kmt5b	1E-06	0.3	Adprm	5E-02	0.2
Abhd17c	9E-03	0.3	Hmgb3	4E-02	0.2
Zmiz2	6E-02	0.3	Hist4h4	7E-01	0.2
Cmss1	2E-22	0.3	Trim24	4E-04	0.2
Gstm5	7E-10	0.3	Med24	7E-02	0.2
Gm16740	1E-02	0.3	Gtpbp4	2E-10	0.2
Fzd2	2E-11	0.3	Nmnat3	1E-01	0.2
Nlk	1E-04	0.3	Tns2	1E-02	0.2
Phpt1	3E-03	0.3	Golim4	9E-12	0.2
Msantd4	2E-06	0.3	Ctdsp2	7E-08	0.2
Zfp235	1E-01	0.3	Khdc4	7E-04	0.2
Antxr2	2E-03	0.3	Scyl3	3E-01	0.2
Lmbrd1	1E-07	0.3	Slc35e2	9E-02	0.2
P4ha2	4E-09	0.3	Zkscan17	2E-01	0.2
Cgref1	3E-04	0.3	Vamp4	2E-05	0.2
Efcab7	2E-01	0.3	Ino80dos	1E-01	0.2
Kansl2	5E-08	0.3	Zfp40	5E-01	0.2
Gm5089	6E-01	0.3	Vps13c	6E-07	0.2
Cyld	6E-09	0.3	Snhg9	8E-01	0.2
Utp25	2E-02	0.3	P3h4	3E-08	0.2
4930430F08Rik	5E-03	0.3	Lpin3	7E-01	0.2
Ddx5	6E-47	0.3	Apoo	1E-01	0.2
Recql5	5E-02	0.3	Slmap	2E-05	0.2
Rbbp9	8E-04	0.3	Ears2	6E-01	0.2
Klc4	3E-03	0.3	Spcs2	9E-17	0.2
Ccdc51	2E-01	0.3	Sfi1	2E-02	0.2
Jkamp	2E-08	0.3	E4f1	4E-01	0.2
Abca5	1E-01	0.3	Eogt	1E+00	0.2
Pex2	9E-08	0.3	Cnnm2	1E-02	0.2
Ing4	4E-08	0.3	Tchp	1E-01	0.2
Slc37a3	4E-04	0.2	Rita1	9E-02	0.2
Nt5dc1	9E-04	0.2	Slc1a4	2E-01	0.2
Phf6	3E-03	0.2	F8	5E-01	0.2
Ubn2	2E-13	0.2	Rhot2	7E-01	0.2
Eml5	3E-01	0.2	Slc9a8	9E-02	0.2
Nbr1	2E-06	0.2	Fam222b	2E-02	0.2
Cox14	3E-25	0.2	Pafah2	1E+00	0.2
Exd2	2E-01	0.2	D130017N08Rik	3E-01	0.2
Lins1	1E-01	0.2	Ascc2	1E-01	0.2
Gm14798	5E-01	0.2	Ambra1	5E-08	0.2
Irak1bp1	1E-05	0.2	Gm11613	4E-01	0.2
Trim39	2E-01	0.2	Naa35	1E-05	0.2
2310014F06Rik	1E-02	0.2	Fgf2	5E-02	0.2
Slc22a15	7E-01	0.2	Kcne4	8E-01	0.2
Zmym2	3E-08	0.2	Ccni	3E-10	0.2
Lrrc8b	8E-01	0.2	1110059G10Rik	1E-03	0.2
Fam135a	2E-04	0.2	Dexi	7E-04	0.2
Smim14	5E-20	0.2	Borcs7	1E-05	0.2
5730522E02Rik	2E-01	0.2	Fmc1	2E-05	0.2
Bmp4	1E-02	0.2	Rnf103	7E-02	0.2
Mrps28	5E-12	0.2	Ip6k1	4E-05	0.2
Zfp983	3E-01	0.2	Gm26542	4E-01	0.2
Pcnx	6E-06	0.2	Ammecr1	2E-02	0.2
Nucb1	2E-23	0.2	Plekhg5	2E-02	0.2
Zfp512	1E-03	0.2	Pink1	8E-05	0.2
Lpcat1	4E-01	0.2	Gm42439	6E-01	0.2
Ubxn11	1E-01	0.2	Alkbh6	6E-03	0.2
Senp7	4E-08	0.2	Mink1	2E-01	0.2
Paip1	3E-10	0.2	Larp4b	3E-07	0.2
Apbb3	1E-01	0.2	Cdk13	2E-07	0.2
Traf6	2E-02	0.2	Trappc9	1E-03	0.2
Nol7	2E-15	0.2	Zfp622	2E-03	0.2
Gm36198	4E-01	0.2	Zfp703	4E-02	0.2
Gm10785	2E-01	0.2	2810433D01Rik	2E-01	0.2
Kiz	4E-06	0.2	Calhm6	2E-01	0.2
Ssbp1	3E-15	0.2	Casc3	1E-02	0.2
Cox15	2E-01	0.2	Lrp5	2E-01	0.2
Gm20721	9E-04	0.2	Ercc6	2E-01	0.2

Fibroblast

Control			NICD2		
Gene	p	FC	Gene	p	FC
Fuca2	1E-06	0.2	Ctsl	6E-39	0.2
Hsd17b11	9E-08	0.2	Tmem164	2E-05	0.2
Rab3d	9E-04	0.2	Bin1	2E-11	0.2
Prkab2	9E-03	0.2	Tmem151a	4E-03	0.2
Slc29a3	7E-04	0.2	D6Wsu163e	9E-03	0.2
Zfp286	7E-04	0.2	Poglut3	2E-06	0.2
Appl2	1E-07	0.2	Tmem87b	2E-04	0.2
Scpep1	3E-27	0.2	Anks6	1E-01	0.2
Pfkfb3	4E-07	0.2	Rufy2	9E-06	0.2
Rpl13a	2E-123	0.2	Rps3	2E-72	0.2
Bcorl1	4E-03	0.2	Mnd1	8E-01	0.2
Llgl1	1E-01	0.2	Nol6	2E-02	0.2
Dock7	3E-16	0.2	Rpl6	6E-73	0.2
Fam171b	3E-07	0.2	Osbpl1a	3E-07	0.2
Slc2a10	1E-02	0.2	Slc46a1	2E-02	0.2
Gm14410	4E-02	0.2	Gm39469	1E-03	0.2
Vat1	1E-21	0.2	Hectd3	5E-02	0.2
Rpl10a	1E-120	0.2	Brsk1	2E-02	0.2
Ppa2	2E-15	0.1	Nbas	9E-04	0.2
Fez1	1E-16	0.1	Numb	4E-06	0.2
Dcbld2	7E-05	0.1	Stat1	5E-11	0.2
Lactb	2E-08	0.1	Zfp800	3E-01	0.2
Rpl26	7E-178	0.1	Chfr	7E-05	0.2
Zfp189	2E-01	0.1	Aida	1E-10	0.2
Gm45669	7E-02	0.1	Uba6	5E-05	0.2
Pip5k1c	7E-05	0.1	Gls	5E-11	0.2
Kdelr1	2E-37	0.1	Gask1b	2E-04	0.2
Fbxo25	3E-06	0.1	Rab6b	3E-03	0.2
Gstm4	1E-02	0.1	Pi4ka	5E-05	0.2
Nr1d1	5E-04	0.1	Tmem50a	2E-21	0.2
Coa5	6E-07	0.1	Rpl8	4E-76	0.2
Tspan14	9E-02	0.1	Ggt7	2E-03	0.2
Tspan17	9E-15	0.1	Gdap2	7E-02	0.2
Fkbp11	4E-29	0.1	Stk3	3E-08	0.2
B830012L14Rik	1E-04	0.1	Kidins220	1E-09	0.2
Pabpc1	3E-59	0.1	Hs1bp3	4E-04	0.2
Rps15	9E-103	0.1	Msrb1	4E-12	0.2
Iqce	7E-04	0.1	Elovl5	9E-03	0.2
D2hgdh	1E-01	0.1	B230307C23Rik	1E-03	0.2
Ankrd39	2E-04	0.1	0610010K14Rik	1E-12	0.2
Zfp651	2E-07	0.1	Gm14164	2E-02	0.2
Gria3	2E-22	0.1	Tmem192	2E-03	0.2
Elovl5	2E-03	0.1	Tspyl2	3E-05	0.2
Anks6	2E-02	0.1	Apbb3	7E-01	0.2
Tbc1d17	6E-05	0.1	Upf1	9E-05	0.2
Rusc1	2E-02	0.1	Harbi1	1E-01	0.2
Rpl27	7E-141	0.1	Hcfc1r1	5E-20	0.2
Hspa12a	1E-02	0.1	Smim11	5E-12	0.2
Gns	8E-17	0.1	Tmem97	1E-03	0.2
Armc6	4E-02	0.1	Irf2	6E-08	0.2
Etfbkmt	2E-02	0.1	Tm7sf3	4E-05	0.2
Pip4k2b	2E-04	0.1	Sbf2	1E-11	0.2
Aebp1	1E-25	0.1	Dhcr7	1E-01	0.2
Lmna	6E-47	0.1	Msrb3	5E-06	0.2
Gm14403	3E-02	0.1	Ripk1	1E-07	0.2
Gstm1	1E-41	0.1	Zfp318	7E-04	0.2
Rspry1	4E-10	0.1	Ankrd12	2E-13	0.2
Abca5	5E-03	0.1	Ccdc186	6E-09	0.2
Plcb3	1E-06	0.1	Slc3a2	1E-13	0.2
1700012D14Rik	1E-03	0.1	Selenon	1E-05	0.2
Mga	1E-13	0.1	Smad6	2E-04	0.2
Nus1	8E-16	0.1	Dffa	2E-01	0.2
Ilvbl	2E-06	0.1	Zfp623	8E-02	0.2
Grina	4E-20	0.1	Utp14b	4E-01	0.2
Fam189b	7E-05	0.1	Hspa2	2E-01	0.2
Rpl7	8E-135	0.1	Eif2s3y	2E-05	0.2
Lgals8	4E-10	0.1	Arfgap1	7E-07	0.2
Inpp5e	4E-01	0.1	Naga	2E-03	0.2
Cdh13	4E-05	0.1	Bag3	9E-05	0.2
Ptpn12	7E-07	0.1	Sgtb	4E-02	0.2
Coro1b	8E-03	0.1	Plcb3	1E-04	0.2
Cad	2E-03	0.1	Fam114a1	9E-21	0.2
Col6a3	3E-21	0.1	Chd9	5E-08	0.2

Fibroblast?

Control			NICD2		
Gene	p	FC	Gene	p	FC
Zfp365	2E-18	0.5	Nos1ap	7E-17	0.5
Dhrs7b	4E-29	0.5	D130017N08Rik	3E-18	0.5
Terf2	3E-23	0.5	Stxbp6	2E-38	0.5
Noct	1E-15	0.5	Zfp930	7E-15	0.5
Sap18	1E-38	0.5	Eny2	8E-91	0.5
Pank2	5E-25	0.5	Srxn1	1E-33	0.5
Stard6	5E-14	0.5	Smtn	2E-34	0.5
Usp29	1E-10	0.5	Fbxo34	5E-31	0.5
Zfp217	1E-13	0.5	A330015K06Rik	4E-12	0.5
Stard7	4E-23	0.5	Gm12153	2E-16	0.5
Zdhhc6	2E-22	0.5	Pgf	5E-13	0.5
Limk1	1E-13	0.5	Gm16833	5E-14	0.5
Lrch2	5E-23	0.5	Ubash3b	1E-26	0.5
Vbp1	2E-24	0.5	Gpn2	2E-15	0.5
Mgat2	3E-39	0.5	Bahcc1	7E-27	0.5
Hapln1	4E-07	0.5	Hmgn1	2E-156	0.5
U2af2	3E-28	0.5	1810055G02Rik	3E-30	0.5
Pwwp2b	3E-15	0.5	Cth	2E-19	0.5
Nol7	7E-31	0.5	Manf	3E-88	0.5
1110059E24Rik	8E-24	0.5	Cdk4	7E-89	0.5
Ints2	1E-24	0.5	Myof	2E-60	0.5
Dclre1a	8E-24	0.5	Dhx9	1E-45	0.5
Slc35e4	8E-20	0.5	Snip1	4E-28	0.5
Taf1	6E-26	0.5	Hccs	5E-27	0.5
Twist2	4E-15	0.5	Lsp1	2E-29	0.5
Nap1l3	7E-07	0.5	Akap6	1E-17	0.5
Hspd1	1E-38	0.5	Msl3	7E-38	0.5
Tbl2	9E-20	0.5	Slc38a1	3E-29	0.5
Fgd1	6E-28	0.5	Ncs1	4E-27	0.5
Tmem186	1E-15	0.5	Ints11	1E-28	0.5
Hp1bp3	8E-40	0.5	Sin3a	5E-32	0.5
Aqp5	2E-09	0.5	Ermp1	5E-25	0.5
Tfam	5E-21	0.5	Tpr	7E-115	0.5
Isoc1	6E-24	0.5	Sorbs3	3E-30	0.5
Tbc1d10b	2E-17	0.5	Slc39a8	2E-13	0.5
Zfp52	8E-19	0.5	Pmpca	3E-19	0.5
Pknox1	1E-21	0.5	Mpp7	7E-29	0.5
Cherp	5E-17	0.5	Efcab7	4E-21	0.5
Pds5a	4E-29	0.5	Setd6	2E-19	0.5
Afdn	1E-26	0.5	Ncbp1	5E-29	0.5
Pcyt1a	2E-22	0.5	Kcnt2	1E-26	0.5
Fancf	6E-24	0.5	Tardbp	6E-45	0.5
Tnfaip8	7E-30	0.5	Ndufaf2	2E-43	0.5
Ctps	7E-29	0.5	Eif3j2	4E-13	0.5
Ptbp1	2E-25	0.5	Gm50340	5E-12	0.5
H19	2E-32	0.5	Mgat2	2E-55	0.5
Trpm6	3E-15	0.5	Prss35	4E-15	0.5
Hnrnpu	2E-72	0.5	Tshz1	1E-31	0.5
Def6	7E-25	0.5	Zfp91	2E-81	0.5
Akap6	3E-15	0.5	Zscan22	4E-13	0.5
Lasp1	3E-25	0.5	Flot2	4E-33	0.5
Kcnt2	6E-20	0.5	Stard3nl	3E-34	0.5
Otud1	4E-07	0.5	Stk32c	2E-12	0.5
Vash2	1E-08	0.5	S100a16	6E-35	0.5
Tada1	2E-15	0.5	Vkorc1l1	4E-20	0.5
Brd9	3E-32	0.5	Tra2b	1E-48	0.5
Zfp626	1E-12	0.5	Mrps25	2E-37	0.5
Pld2	4E-20	0.5	Afdn	3E-31	0.5
Gm49336	1E-18	0.5	Col5a3	3E-36	0.5
Cdc123	1E-44	0.5	Fntb	5E-30	0.5
Fgf13	7E-35	0.5	1700017B05Rik	8E-24	0.5
Gm19426	4E-10	0.5	Srprb	2E-33	0.5
Zdhhc21	3E-21	0.5	Pde5a	2E-34	0.5
Coq7	1E-29	0.5	Snhg15	8E-27	0.5
Ccdc22	1E-20	0.5	Mmachc	2E-08	0.5
5430416N02Rik	9E-25	0.5	Kif5c	1E-08	0.5
Zfp114	5E-08	0.5	Rrp9	8E-12	0.5
Acan	4E-15	0.5	Fndc4	5E-21	0.5
Dhx38	1E-22	0.5	Lasp1	6E-24	0.5
Actn1	4E-29	0.5	Eif6	1E-40	0.5
Ipmk	7E-22	0.5	Snx7	3E-40	0.5
Bex3	8E-38	0.5	Taf1	3E-37	0.5
Tial1	2E-26	0.5	Hp1bp3	2E-51	0.5

Undefined

Control			NICD2		
Gene	p	FC	Gene	p	FC
Lman1	2E-12	0.1	Tma16	8E-01	0.1
Scamp2	3E-02	0.1	Taf11	1E-01	0.1
Pmm2	1E-01	0.1	Dusp3	8E-01	0.1
Cbll1	3E-01	0.1	Med24	5E-02	0.1
Rrm2b	9E-01	0.1	Slc25a23	4E-01	0.1
Rad51d	9E-01	0.1	Spcs2	1E-05	0.1
Chchd10	1E-01	0.1	Snai2	1E-01	0.1
Cyp2u1	5E-01	0.1	Capn10	4E-01	0.1
Saraf	3E-02	0.1	Btbd6	2E-01	0.1
Eif3d	1E-02	0.1	Sgta	2E-01	0.1
Gt(ROSA)26Sor	8E-01	0.1	Znrd2	6E-01	0.1
Pgrmc1	1E-02	0.1	Zfp266	2E-01	0.1
Mrps27	5E-01	0.1	Pou2f1	6E-01	0.1
Pfdn4	3E-01	0.1	Ino80c	5E-01	0.1
Cdk5	7E-01	0.1	Dcaf8	5E-03	0.1
Hltf	7E-01	0.1	Rbm12b2	6E-02	0.1
Ptdss2	3E-01	0.1	Nip7	8E-01	0.1
Zfp426	4E-01	0.1	Ccdc57	8E-03	0.1
Utp14a	2E-01	0.1	Btf3l4	7E-01	0.1
AI480526	3E-01	0.1	Ints6l	4E-01	0.1
Dtwd2	4E-01	0.1	Naa20	6E-02	0.1
Cbx8	8E-01	0.1	Cdk4	5E-04	0.1
1110002L01Rik	4E-01	0.1	Timm10	5E-01	0.1
Gtf2a1	8E-01	0.1	Mysm1	4E-01	0.1
Polb	8E-02	0.1	Trp53bp1	9E-01	0.1
Prpf38a	7E-01	0.1	Rsbn1	9E-01	0.1
Rsph3a	2E-01	0.1	Ints11	1E-01	0.1
Ddx47	7E-01	0.1	Atad3a	2E-02	0.1
AI837181	5E-01	0.1	Acot2	8E-01	0.1
Snrnp27	8E-03	0.1	Psmb6	6E-03	0.1
Metap1d	6E-02	0.1	Maoa	1E+00	0.1
Igf2bp2	4E-01	0.1	Ndfip1	1E-10	0.1
Cetn2	2E-01	0.1	C87436	2E-01	0.1
Lta4h	8E-02	0.1	Cuedc2	3E-02	0.1
Cebpz	3E-03	0.1	Cep95	2E-02	0.1
Aco2	2E-03	0.1	Ocrl	7E-01	0.1
Ccdc124	1E-01	0.1	Ldah	9E-02	0.1
Gm19522	2E-01	0.1	Sdad1	9E-01	0.1
Wdr75	2E-01	0.1			
Ikbke	1E-01	0.1			
Foxp4	7E-01	0.1			
Hbp1	8E-01	0.1			
Gdap2	4E-02	0.1			
Klhl21	2E-01	0.1			
Atp6v0c	4E-10	0.1			
Pibf1	5E-01	0.1			
Stam	3E-01	0.1			

Articular/Synovial Fibroblast

Control			NICD2		
Gene	p	FC	Gene	p	FC
Nsdhl	9E-02	0.2	Col6a3	4E-07	0.2
Slc4a2	2E-01	0.2	Eaf2	1E+00	0.2
Rgmb	6E-01	0.2	Hbp1	6E-03	0.2
Natd1	5E-01	0.2	Adck2	8E-01	0.2
6030458C11Rik	7E-01	0.2	Acadl	1E-06	0.2
Snx32	8E-01	0.2	Gm37768	5E-01	0.2
Dennd2c	8E-01	0.2	Zfp768	5E-01	0.2
Pld3	2E-06	0.2	Faap100	2E-01	0.2
Kremen1	1E+00	0.2	Sergef	5E-01	0.2
Zfp800	8E-02	0.2	Zfp788	5E-01	0.2
Cep250	6E-01	0.2	Gstm4	5E-01	0.2
Saysd1	2E-03	0.2	Megf8	1E+00	0.2
mt-Nd1	9E-23	0.2	Zfat	7E-01	0.2
Zfp703	1E-01	0.2	Ifi203	4E-01	0.2
Gcat	8E-01	0.2	Mpdz	2E-03	0.2
Zfp507	6E-01	0.2	Prkd2	9E-01	0.2
Gigyf2	5E-05	0.2	Pi4ka	2E-02	0.2
Xylt1	1E-04	0.2	Abca5	8E-01	0.2
Dtd1	2E-03	0.2	Aga	3E-06	0.2
Pdzd4	5E-01	0.2	Cnnm2	1E+00	0.2
Cplane1	2E-02	0.2	Tlr4	7E-01	0.2
Fundc2	4E-15	0.2	Naga	9E-01	0.2
Sergef	1E-01	0.2	Sik1	9E-01	0.2
Gm26672	8E-01	0.2	Aldh3a2	8E-01	0.2
Cnot9	1E+00	0.2	Il1rn	2E-01	0.2
Slc17a5	4E-01	0.2	St5	8E-04	0.2
Pcbp3	6E-01	0.2	Slc25a28	6E-01	0.2
Lamp2	1E-17	0.2	Sap30bp	1E-01	0.2
Rusc1	6E-01	0.2	Ndufv3	4E-08	0.2
Rab13	4E-03	0.2	Pbxip1	4E-05	0.2
Cln3	3E-01	0.2	Wdr13	7E-01	0.2
Eif4ebp2	3E-02	0.2	Ints6l	5E-01	0.2
Bach1	4E-01	0.2	Zfp729b	8E-01	0.2
Tbx4	8E-02	0.2	Snapc5	1E-02	0.2
2900060B14Rik	3E-01	0.2	Mgat5	4E-01	0.2
Pink1	3E-02	0.2	Slc22a18	9E-01	0.2
Xaf1	3E-01	0.2	mt-Co2	1E-40	0.2
Phf20	3E-03	0.2	Usp28	6E-01	0.2
Tmem251	6E-01	0.2	Ap4b1	4E-01	0.2
Herc6	8E-01	0.2	Mib2	4E-01	0.2
Platr25	7E-01	0.2	Ocel1	8E-01	0.2
Cdc14a	9E-01	0.2	Vps13a	2E-01	0.2
Zfp235	9E-01	0.2	Prickle3	7E-01	0.2
Wrb	7E-01	0.2	Mospd2	3E-01	0.2
Cdadc1	2E-01	0.2	Smim14	5E-05	0.2
Mast3	8E-01	0.2	Hmox1	2E-02	0.2
Zfp617	1E+00	0.2	Nsmce2	2E-05	0.2
Rapgef2	4E-03	0.2	Dync2li1	1E-01	0.2
Mtmr11	5E-01	0.2	Aldh9a1	5E-01	0.2
Casp12	2E-02	0.2	Plpbp	2E-01	0.2
Ankrd12	5E-06	0.2	Iah1	2E-03	0.2
Hebp1	8E-03	0.2	Phc3	4E-02	0.2
Pot1a	9E-01	0.2	Ece2	9E-01	0.2
Dmpk	2E-02	0.2	Trip6	8E-02	0.2
Tctn2	5E-01	0.2	Marveld1	3E-01	0.2
Retreg2	2E-01	0.2	Cpxm1	6E-03	0.2
Cttnbp2	7E-03	0.2	Bbs5	6E-01	0.2
Mtmr14	8E-01	0.2	Fut11	5E-01	0.2
2010204K13Rik	6E-01	0.2	Prdm15	6E-01	0.2
Calr	2E-14	0.2	Ift172	9E-01	0.2
Ptrh1	1E+00	0.2	Ube3b	8E-01	0.2
6430590A07Rik	9E-01	0.2	Miga2	3E-01	0.2
Tmem265	8E-01	0.2	Mrpl23	3E-06	0.2
Ptprs	4E-05	0.2	5930430L01Rik	6E-01	0.2
Cd63	2E-25	0.2	Insig2	2E-03	0.2
Capn7	5E-01	0.2	Fyco1	1E-01	0.2
2610507I01Rik	9E-01	0.2	Vangl1	6E-01	0.2
Zxdc	8E-01	0.2	Stard5	9E-01	0.2
2610044O15Rik8	9E-01	0.2	Cep95	7E-01	0.2
Pip5k1b	3E-01	0.2	Idua	4E-01	0.2
Mndal	4E-01	0.2	Tut7	2E-03	0.2
Atrnl1	6E-02	0.2	Fndc4	7E-01	0.2
Uqcc1	8E-01	0.2	Zfp866	6E-01	0.2

Limb Mesenchyme

Control Gene	*p*	FC	NICD2 Gene	*p*	FC
Hus1	9E-04	0.2	Uri1	1E-08	0.2
Trp53rkb	6E-03	0.2	Fbf1	4E-05	0.2
Arl1	8E-23	0.2	Ift20	9E-23	0.2
Slc52a2	7E-04	0.2	Mbtps1	1E-10	0.2
Edc4	1E-05	0.2	Heatr3	9E-06	0.2
Tmed9	4E-28	0.2	Lrrc14	6E-05	0.2
Dab2ip	2E-08	0.2	Mbtps2	8E-05	0.2
Hotairm1	2E-08	0.2	L3mbtl3	1E-04	0.2
Msh3	2E-05	0.2	Ggact	1E-06	0.2
Tsg101	4E-11	0.2	Tyw5	4E-03	0.2
Dnaaf5	1E-04	0.2	Rab10	2E-18	0.2
Spindoc	7E-05	0.2	Gorasp2	4E-12	0.2
Pkd2l2	4E-02	0.2	Rffl	4E-04	0.2
Vbp1	4E-08	0.2	Bsdc1	8E-07	0.2
Gpr107	1E-06	0.2	Trmt61b	2E-06	0.2
Agbl5	5E-04	0.2	Adpgk	8E-06	0.2
Cdk5rap1	7E-04	0.2	Farsa	1E-06	0.2
Senp6	5E-17	0.2	Gm13056	9E-07	0.2
Ulk1	9E-07	0.2	Tram2	5E-07	0.2
Tdrd3	9E-10	0.2	Cgrefl	2E-06	0.2
Fbxw4	8E-07	0.2	Grwd1	5E-04	0.2
Fam219b	4E-05	0.2	Bysl	4E-08	0.2
Tbc1d31	5E-08	0.2	Gm13832	4E-03	0.2
Rrbp1	4E-32	0.2	Cuedc1	1E-07	0.2
Wdr41	2E-06	0.2	Ubr5	2E-10	0.2
1110002L01Rik	8E-04	0.2	Fam13b	3E-07	0.2
Tpd52l1	2E-04	0.2	Ddx1	1E-11	0.2
Gm26911	5E-05	0.2	Dnajc1	2E-13	0.2
Vcl	1E-05	0.2	Dph6	1E-07	0.2
Arxes2	1E-06	0.2	Cct3	2E-14	0.2
Rhbdd2	3E-04	0.2	Rps6ka5	3E-05	0.2
Med30	4E-08	0.2	Grik5	8E-06	0.2
Camsap1	9E-09	0.2	Gja1	8E-11	0.2
Ttpal	1E-05	0.2	Gmps	2E-10	0.2
Dock7	1E-09	0.2	Tra2b	2E-09	0.2
Gli2	6E-08	0.2	Wdr46	2E-04	0.2
Def6	4E-05	0.2	Ireb2	4E-07	0.2
Tmem64	3E-05	0.2	Fry	5E-13	0.2
Gtpbp8	1E-05	0.2	Rbsn	6E-06	0.2
Rbm22	3E-09	0.2	BC034090	1E-04	0.2
Vhl	4E-05	0.2	Zfp719	3E-05	0.2
Glg1	6E-18	0.2	Camk2g	1E-06	0.2
Tnrc6b	6E-15	0.2	Hacd3	1E-06	0.2
Orc4	2E-09	0.2	Dhx16	2E-05	0.2
Ssh1	8E-06	0.2	Pus10	1E-06	0.2
Adpgk	1E-08	0.2	Brd3os	1E-04	0.2
Kpna1	7E-07	0.2	Nkiras1	4E-05	0.2
Zfp84	9E-07	0.2	Akt1	5E-10	0.2
Zdhhc13	1E-03	0.2	1810034E14Rik	1E-03	0.2
Mex3b	2E-03	0.2	Trp53inp2	3E-06	0.2
Rdh10	2E-06	0.2	Gm4924	5E-04	0.2
Kcnip4	2E-08	0.2	Polr3d	7E-08	0.2
Zfp160	5E-06	0.2	Ints1	3E-04	0.2
Sp4	5E-04	0.2	Fam49b	6E-07	0.2
Sil1	4E-10	0.2	Skap2	1E-08	0.2
Tmem115	9E-07	0.2	Papola	6E-15	0.2
Ufl1	6E-09	0.2	Wdr82	1E-06	0.2
Tubgcp3	4E-05	0.2	Slc10a7	2E-05	0.2
Asb7	2E-07	0.2	Atf6b	1E-06	0.2
Irf2bp1	3E-08	0.2	Stk17b	7E-04	0.2
Slc19a2	4E-04	0.2	Gpr107	3E-06	0.2
Ngrn	5E-07	0.2	Tmem115	6E-03	0.2
Ccdc141	6E-02	0.2	Fbxo11	8E-11	0.2
A730063M14Rik	1E-03	0.2	Oxnad1	1E-03	0.2
Phrf1	8E-06	0.2	Tars2	1E-04	0.2
Atad1	7E-09	0.2	Gm38604	2E-03	0.2
Kctd3	2E-06	0.2	Gm28375	1E-04	0.2
Commd9	3E-08	0.2	Cul3	1E-11	0.2
Zmym2	2E-08	0.2	Raph1	4E-09	0.2
Arfip2	2E-06	0.2	Pprc1	3E-05	0.2
A030001D20Rik	8E-03	0.2	Ndfip2	7E-07	0.2
Gramd1a	5E-06	0.2	Zfp239	8E-04	0.2
Ddx20	8E-04	0.2	Rbm28	2E-09	0.2

Chondrogenic

Control Gene	*p*	FC	NICD2 Gene	*p*	FC
Zfp869	9E-03	0.2	Col24a1	4E-01	0.2
Luzp1	1E-14	0.2	Tgif1	5E-03	0.2
Birc2	5E-03	0.2	Fbxw17	4E-03	0.2
Dhtkd1	1E-01	0.2	Rangrf	1E-02	0.2
9130008F23Rik	2E-01	0.2	Gstm5	4E-06	0.2
Ndufa1	1E-24	0.2	Tceal1	4E-02	0.2
Mtfr1	3E-04	0.2	Vps26c	9E-02	0.2
Def8	3E-02	0.2	Zyg11b	1E-03	0.2
Letm2	1E-02	0.2	Unc119b	4E-01	0.2
Dock5	2E-01	0.2	Ints12	5E-01	0.2
Cep57	4E-06	0.2	Taf5l	2E-01	0.2
Dop1b	8E-03	0.2	Cstf2t	3E-02	0.2
Far1os	2E-01	0.2	Traf2	8E-01	0.2
Plod1	6E-08	0.2	Dnal1	2E-02	0.2
Esf1	8E-08	0.2	Krt10	1E-02	0.2
Smarcd3	1E-04	0.2	Tm9sf4	6E-09	0.2
Ppp1r37	8E-03	0.2	Tanc1	2E-02	0.2
Zer1	1E-01	0.2	Endov	6E-01	0.2
Pcif1	2E-05	0.2	Lcor	5E-04	0.2
Cwf19l2	4E-07	0.2	Cry2	8E-02	0.2
Map3k1	9E-02	0.2	Usf1	2E-01	0.2
BC024978	4E-01	0.2	Zfp37	1E-01	0.2
Syvn1	1E-01	0.2	Pigk	2E-05	0.2
Cnot7	3E-04	0.2	Gm20342	3E-01	0.2
Tmem128	1E-05	0.2	Rusc2	1E-01	0.2
Pdss2	4E-04	0.2	Tob2	3E-02	0.2
Nckap5	4E-10	0.2	Creld2	2E-07	0.2
Gfm2	1E-03	0.2	Alkbh7	4E-01	0.2
Churc1	1E-11	0.2	Zfp397	5E-03	0.2
Dusp18	6E-02	0.2	Arid4b	3E-10	0.2
Fam229b	3E-03	0.2	Pcgf6	4E-01	0.2
Nek9	1E-03	0.2	Hspbap1	1E-01	0.2
Mppe1	2E-02	0.2	Secisbp2	6E-02	0.2
Sgms1	5E-07	0.2	Nucb1	9E-18	0.2
2900005J15Rik	4E-01	0.2	Sdhaf3	4E-02	0.2
Bpnt1	2E-01	0.2	Bloc1s5	1E-01	0.2
Fmc1	2E-09	0.2	Dbt	7E-02	0.2
Klhl13	5E-04	0.2	Irs2	8E-01	0.2
3010001F23Rik	2E-01	0.2	Gin1	3E-01	0.2
Pon3	7E-07	0.2	Zfp248	6E-02	0.2
Neat1	1E-16	0.2	Airn	3E-13	0.2
Mettl18	2E-01	0.2	Camsap2	3E-03	0.2
Msrb3	4E-14	0.2	Gm16541	7E-01	0.2
Rfx1	3E-01	0.2	Ccdc28a	8E-03	0.2
Sdf2	1E-15	0.2	Bcat2	2E-02	0.2
mt-Nd5	1E-31	0.2	Park7	1E-25	0.2
Gm6712	4E-01	0.2	Arl10	3E-01	0.2
Malt1	2E-01	0.2	Aktip	2E-02	0.2
Phf10	9E-07	0.2	Prtg	5E-01	0.2
Rnf215	3E-02	0.2	Faf1	2E-09	0.2
Nepro	7E-01	0.2	Cacng7	2E-01	0.2
Cfap36	4E-15	0.2	Mill2	2E-01	0.2
Scrn2	4E-03	0.2	Mcl1	2E-10	0.2
Pyroxd1	3E-02	0.2	E230016M11Rik	4E-01	0.2
Tmem230	1E-04	0.2	Sgcb	3E-04	0.2
Wdsub1	1E-02	0.2	Tdg	2E-02	0.2
Fat1	5E-16	0.2	Cebpzos	2E-08	0.2
Prnp	9E-21	0.2	Hypk	7E-09	0.2
Thap2	3E-06	0.2	Mob3c	5E-01	0.2
Ccdc30	3E-02	0.2	Mesd	3E-15	0.2
Ccdc112	8E-01	0.2	Bnip1	2E-01	0.2
Acbd4	4E-02	0.2	Hmgxb3	3E-02	0.2
Gm28198	4E-02	0.2	Epm2aip1	3E-03	0.2
Mta1	4E-07	0.2	Gm15952	2E-01	0.2
Fcho2	2E-08	0.2	Lrrc8b	3E-01	0.2
2310009A05Rik	2E-07	0.2	Nubpl	3E-02	0.2
Trappc9	2E-05	0.2	Tmem230	3E-04	0.2
Tmem216	2E-03	0.2	Shox2	1E-02	0.2
Mpp5	1E-03	0.2	Stambp	8E-05	0.2
Pcyox1	3E-06	0.2	Tmem30a	5E-12	0.2
Pja2	5E-12	0.2	Gm43713	1E-01	0.2
Ttc7b	1E-03	0.2	Dap	9E-14	0.2
Lars2	2E-07	0.2	Fbxo9	1E-03	0.2

Fibroblast

Control Gene	*p*	FC	NICD2 Gene	*p*	FC
Armcx2	1E-06	0.1	C1galt1	3E-06	0.2
Pomgnt2	2E-02	0.1	Dpy19l4	8E-04	0.2
Rps3a1	4E-142	0.1	Klf13	8E-04	0.2
Ythdc2	2E-03	0.1	Rpl3	6E-71	0.2
Hist4h4	6E-01	0.1	Zbtb44	1E-03	0.2
Trim2	1E-09	0.1	Hsd17b11	7E-04	0.2
Mnd1	4E-01	0.1	Mospd2	4E-05	0.2
Eef1aknmt	5E-03	0.1	Ap5m1	9E-02	0.2
Csk	1E-06	0.1	Eef2	9E-37	0.2
Itga2b	7E-03	0.1	Zfp931	2E-03	0.2
Gabarap	2E-89	0.1	Ttc37	1E-05	0.2
Eef1g	8E-69	0.1	Crl1	2E-05	0.2
Tmem164	5E-06	0.1	Appl1	9E-09	0.2
Itprid2	1E-06	0.1	Prkd3	9E-04	0.2
Socs3	6E-04	0.1	Fbxl7	1E-05	0.2
Gm26672	4E-02	0.1	Ifitm2	2E-35	0.2
Vps29	1E-27	0.1	B3gntl1	4E-01	0.2
Ptpn13	8E-10	0.1	Pard3	4E-09	0.2
Stat1	4E-20	0.1	Esd	1E-34	0.2
Thap12	5E-12	0.1	Pi4kb	4E-04	0.2
Iqcc	7E-04	0.1	Dhtkd1	2E-01	0.2
Ankrd12	3E-22	0.1	Rps15	3E-52	0.2
Borcs5	4E-03	0.1	Upp2	9E-04	0.2
Tirap	7E-04	0.1	Cavin2	1E-16	0.2
Vapb	2E-21	0.1	Rpl29	3E-62	0.2
Pprc1	3E-04	0.1	Mboat7	3E-03	0.1
Auh	3E-12	0.1	Foxp1	1E-23	0.1
Gtf2e1	5E-03	0.1	Hdgfl3	9E-08	0.1
Trpm4	5E-02	0.1	Rpl26	1E-85	0.1
Xbp1	1E-16	0.1	Gda	2E-05	0.1
Cd47	4E-26	0.1	Tceanc	3E-01	0.1
Cltc	3E-35	0.1	Atp8b2	7E-04	0.1
1500015L24Rik	7E-07	0.1	Kmt2c	3E-08	0.1
Fam114a1	2E-33	0.1	Cast	2E-16	0.1
Idnk	2E-09	0.1	Bach1	2E-04	0.1
Gm14326	2E-04	0.1	BC029722	1E-05	0.1
Pisd	4E-03	0.1	Arl1	4E-22	0.1
Gm46430	4E-02	0.1	Rhobtb1	4E-02	0.1
Atp8b2	5E-06	0.1	Dcn	2E-02	0.1
Tmsb15b1	3E-02	0.1	Derl1	1E-14	0.1
Bach1	4E-06	0.1	Apobec1	1E-07	0.1
Rps27a	2E-131	0.1	Tasor	2E-05	0.1
Ccdc186	3E-13	0.1	Itga2b	2E-01	0.1
Chd9	7E-12	0.1	Mypop	1E-01	0.1
Ifitm2	1E-63	0.1	Rps5	3E-56	0.1
BC029722	5E-09	0.1	D730003I15Rik	1E-01	0.1
Coq8b	1E-04	0.1	Puf60	9E-06	0.1
Arsk	9E-05	0.1	Gstm5	2E-13	0.1
Cpne3	2E-13	0.1	Egfl7	6E-04	0.1
Man2a1	8E-10	0.1	Prepl	1E-02	0.1
Zfp512b	4E-05	0.1	Twf2	8E-04	0.1
Wdr60	2E-09	0.1	Klhl18	7E-02	0.1
Gm39469	2E-05	0.1	Klhl13	5E-03	0.1
Itgav	3E-11	0.1	Ptdss2	1E-02	0.1
Cyb5r3	4E-26	0.1	Ifnar2	7E-11	0.1
Zfp110	4E-06	0.1	Rbpms	5E-05	0.1
Creg1	1E-27	0.1	Mbd5	4E-08	0.1
Znfx1	2E-05	0.1	Plat	1E-02	0.1
Diaph2	3E-12	0.1	Lrrc17	8E-18	0.1
Selenom	8E-31	0.1	Copa	3E-16	0.1
Sys1	4E-20	0.1	Grina	9E-12	0.1
Vps41	7E-17	0.1	Copz2	2E-16	0.1
Ascc3	1E-13	0.1	Scyl1	3E-04	0.1
Gpam	5E-03	0.1	Top1mt	1E-02	0.1
Auts2	1E-05	0.1	Pdzd8	2E-04	0.1
Slc25a32	5E-03	0.1	Rpl24	5E-78	0.1
Rab13	6E-13	0.1	Snhg16	2E-01	0.1
Surf4	4E-24	0.1	Zfp26	2E-04	0.1
Fam168a	3E-11	0.1	Tmtc3	2E-04	0.1
Ssh1	4E-05	0.1	Klhl42	3E-01	0.1
Foxk1	1E-04	0.1	Arhgap10	2E-05	0.1
Dram2	1E-06	0.1	Eef1akmt4	9E-02	0.1
Ncoa1	3E-08	0.1	Smurf1	4E-04	0.1

Fibroblast?

Control Gene	*p*	FC	NICD2 Gene	*p*	FC
Pde4a	2E-13	0.5	Tchp	7E-17	0.5
Sh3bp4	7E-16	0.5	Ints5	4E-23	0.5
Galnt14	3E-09	0.5	Rnf141	4E-28	0.5
Pnn	3E-40	0.5	Snhg5	3E-25	0.5
Myadm	3E-25	0.5	Zdhhc18	2E-23	0.5
6030442K20Rik	7E-13	0.5	Hnrnpul1	7E-40	0.5
Abracl	5E-44	0.5	Chtf8	3E-19	0.5
Mpp7	1E-21	0.5	Gps2	2E-29	0.5
Med19	1E-19	0.5	Nacc1	5E-30	0.5
Dele1	2E-22	0.5	Slc7a5	1E-29	0.5
Vdac1	7E-63	0.5	Rbpms2	6E-11	0.5
Yeats4	4E-29	0.5	Rdx	5E-81	0.5
Cnot6l	3E-25	0.5	Mrgbp	2E-22	0.5
Tmem138	1E-15	0.5	Crocc	7E-16	0.5
Mpv17l2	3E-20	0.5	Gtpbp10	7E-22	0.5
A830018L16Rik	1E-06	0.5	Hus1	4E-20	0.5
Ap2a1	1E-27	0.5	Sf3b5	1E-81	0.5
Spin4	3E-11	0.5	Slc25a24	6E-27	0.5
Bcdin3d	5E-17	0.5	Cfap43	2E-12	0.5
Nsmce2	1E-39	0.5	Magoh	3E-42	0.5
Scoc	7E-34	0.5	Shb	4E-05	0.5
Setd1a	2E-22	0.5	Pcyt1a	3E-27	0.5
Carf	6E-03	0.5	Rrn3	2E-18	0.5
Tbc1d25	3E-03	0.5	Noct	3E-25	0.5
Sync	6E-09	0.5	Mrpl40	5E-29	0.5
Paics	2E-38	0.5	Rtn4ip1	8E-10	0.5
Slc29a1	7E-24	0.5	Mrpl46	1E-16	0.5
Pde8b	1E-05	0.5	Tsr2	5E-15	0.5
Poll	1E-12	0.5	Mrpl13	8E-41	0.5
Hnrnpa2b1	1E-70	0.5	4933406I18Rik	4E-23	0.5
Col8a1	1E-36	0.5	Pigf	3E-26	0.5
Mrpl40	3E-20	0.5	Acap3	6E-25	0.5
Gpaa1	3E-21	0.5	Mtch1	3E-57	0.5
Tra2b	1E-37	0.5	Tbc1d10b	1E-19	0.5
Alg14	2E-17	0.5	Ncl	2E-82	0.5
Ctdp1	3E-16	0.5	Vps36	3E-42	0.5
Gnb4	2E-26	0.5	Gm30938	9E-09	0.5
Emc8	9E-33	0.5	Pin1	2E-42	0.5
Mtf2	2E-26	0.5	Nap1l4	4E-42	0.5
Eny2	2E-53	0.5	Klf4	1E-23	0.5
Tpr	1E-74	0.5	Cnot6l	1E-29	0.5
Ttc7	4E-19	0.5	S100a10	6E-76	0.5
Rock2	6E-49	0.5	Ube2l6	7E-17	0.5
Bak1	2E-20	0.5	Morc4	2E-28	0.5
Setx	2E-21	0.5	Nhsl2	5E-20	0.5
Smarcc1	1E-37	0.5	Ehbp1l1	7E-26	0.5
Arhgdib	2E-22	0.5	Ints2	2E-28	0.5
Gm20125	6E-11	0.5	Ncoa7	2E-31	0.5
Ppp1r7	2E-33	0.5	Rbm48	2E-15	0.5
Emp2	4E-32	0.5	Calhm2	9E-25	0.5
Stom	1E-17	0.5	Fam217b	8E-13	0.5
Ipo7	1E-22	0.5	Mfsd14b	2E-26	0.5
Elof1	1E-39	0.5	Epb41	7E-27	0.5
Sfpq	1E-36	0.5	Scly	3E-14	0.5
Cgnl1	2E-20	0.5	Brd9	2E-32	0.5
Vav2	1E-14	0.5	Ctdspl	4E-39	0.5
C1qtnf3	5E-06	0.5	Mlh1	2E-22	0.5
Sart3	3E-21	0.5	Pycr2	4E-22	0.5
Npm3	4E-20	0.5	Btbd6	9E-17	0.5
Map3k20	2E-30	0.5	Chsy3	5E-35	0.5
Arrdc1	9E-15	0.5	Apol9a	2E-17	0.5
P2ry10b	4E-16	0.5	Mtcp1	3E-13	0.5
Pgap1	4E-15	0.5	Limch1	2E-21	0.5
Fuom	4E-23	0.5	Trp53	4E-33	0.5
Mrpl47	2E-08	0.5	Mtcl1	2E-16	0.5
Pspc1	1E-22	0.5	Ttll4	1E-28	0.5
Ctnnal1	1E-17	0.5	Pgk1	3E-70	0.5
Gtf3a	7E-21	0.5	Rab13	5E-31	0.5
Zfp213	2E-15	0.5	Uvrag	6E-31	0.5
Tspan2	8E-19	0.5	Rsph1	3E-07	0.5
Ei24	1E-22	0.5	Sept8	9E-32	0.5
Enpp1	4E-17	0.5	Wrn	7E-35	0.5
Leo1	1E-20	0.5	Dagla	7E-14	0.5

Undefined

Control Gene	*p*	FC	NICD2 Gene	*p*	FC

Articular/Synovial Fibroblast

Control Gene	*p*	FC	NICD2 Gene	*p*	FC
Tmem175	7E-02	0.2	Antxr1	8E-05	0.2
Kat14	1E+00	0.2	Prrg2	7E-01	0.2
Asah1	2E-09	0.2	Ccdc61	5E-01	0.2
Gm26881	9E-01	0.2	Trim12a	3E-01	0.2
Pgap2	1E-02	0.2	Nbea	5E-04	0.2
Rmc1	7E-01	0.2	Zfp365	3E-01	0.2
Crebl2	9E-01	0.2	Rmc1	4E-01	0.2
Ano3	5E-01	0.2	Crot	2E-01	0.2
Afmid	4E-01	0.2	Tmed8	7E-01	0.2
Nin	8E-01	0.2	Nudt12	5E-01	0.2
Zbtb11os1	8E-01	0.2	2010204K13Rik	4E-01	0.2
Zfp947	8E-01	0.2	Zfp280d	3E-02	0.2
Tmem241	2E-01	0.2	Sowahc	5E-01	0.2
Acbd4	6E-01	0.2	Ece1	7E-01	0.2
Csf1	6E-07	0.2	Vps26a	2E-02	0.2
Uty	1E-01	0.2	Map3k2	2E-01	0.2
Itga5	1E-01	0.2	Zfp688	6E-01	0.2
Fbxo30	9E-01	0.2	mt-Nd4	2E-26	0.2
Elovl6	3E-02	0.2	Idh2	3E-04	0.2
Atp9b	4E-02	0.2	Zbtb9	3E-01	0.2
Cyb561a3	8E-01	0.2	D330023K18Rik	8E-01	0.2
Hexim1	2E-01	0.2	Map2k3	2E-01	0.2
Tbc1d9b	4E-02	0.2	Sgk1	6E-03	0.2
Tulp2	1E-01	0.2	Gm10130	4E-01	0.2
Dhtkd1	4E-01	0.2	Icam1	9E-02	0.2
Polr2a	5E-04	0.2	Zfp931	9E-01	0.2
Mrtfb	8E-02	0.2	Supt4a	1E-04	0.2
Zfp760	1E+00	0.2	E030030I06Rik	7E-01	0.2
Arhgef10	2E-01	0.2	Gcfc2	1E+00	0.2
Mppe1	1E+00	0.2	Amy1	2E-01	0.2
Fuca1	2E-10	0.2	Zmat5	6E-03	0.2
D330023K18Rik	8E-01	0.2	Washc2	4E-03	0.2
Ifi35	4E-02	0.2	Dhx40	2E-02	0.2
Gnao1	7E-01	0.2	Lrrc49	7E-01	0.2
Sema3c	6E-01	0.2	Nthl1	7E-01	0.2
Tex15	7E-01	0.2	Cir1	9E-02	0.2
Tnfsf13	7E-01	0.2	C030014I23Rik	1E+00	0.2
Eya3	2E-01	0.2	Arpc1a	1E-03	0.2
Pcyt2	7E-02	0.2	Slc2a13	2E-01	0.2
Enpp1	4E-02	0.2	Zfp444	8E-01	0.2
Capn2	4E-05	0.2	Slc35e2	6E-01	0.2
Apool	3E-01	0.2	Atp6v0b	3E-08	0.2
Kcnrg	3E-01	0.2	Clmp	4E-03	0.2
Zfp955a	9E-01	0.2	Hk1	1E-01	0.2
Numb	1E-01	0.2	Lima1	1E-07	0.2
Psen2	3E-01	0.2	Nfil3	5E-01	0.2
Abcc5	2E-02	0.2	Efna1	4E-01	0.2
Spr	2E-02	0.2	Rbpj	8E-06	0.2
Dnajc4	2E-01	0.2	Ergic3	6E-07	0.2
Ece1	2E-01	0.2	Ggact	3E-01	0.2
Cd99l2	2E-01	0.2	Pdk2	7E-01	0.2
Gabarap	6E-29	0.2	Sh3bp5l	7E-01	0.2
Ctdsp2	1E-04	0.2	Zfp438	6E-01	0.2
Acox3	4E-01	0.2	Chic1	1E+00	0.2
Gm37494	4E-01	0.2	Tsga10	8E-01	0.2
Styxl1	9E-01	0.2	Gin1	8E-01	0.2
Thumpd2	7E-01	0.2	Ifi35	3E-01	0.2
Sncaip	3E-01	0.2	Erap1	6E-01	0.2
Gm7072	9E-01	0.2	Pip5k1c	9E-01	0.2
Hexdc	7E-01	0.2	AI837181	1E-01	0.2
Cdc42ep4	9E-01	0.2	Tcea3	1E-05	0.2
Nagpa	5E-01	0.2	Rabl2	3E-01	0.2
Dcaf4	6E-01	0.2	Pick1	7E-01	0.2
Zfp839	5E-01	0.2	Psen1	9E-01	0.2
Rnf103	3E-01	0.2	Pkn1	4E-01	0.2
Zfp141	4E-01	0.2	Mysm1	1E-01	0.2
Dcakd	4E-01	0.2	AW146154	8E-01	0.2
C1galt1	9E-03	0.2	Snap29	6E-01	0.2
Rnf40	7E-01	0.2	Atp7a	5E-01	0.2
Hsdl1	3E-01	0.2	Slc16a7	3E-01	0.2
Letm2	1E+00	0.2	Phf1	8E-01	0.2
Irf2bpl	4E-01	0.2	Slc38a6	8E-01	0.2
Itgb1bp1	7E-03	0.2	Fbxw11	9E-02	0.2

Limb Mesenchyme					
Control			NICD2		
Gene	*p*	FC	Gene	*p*	FC
Fzd2	5E-15	0.2	Celf4	1E-04	0.2
Zfp81	4E-05	0.2	Atp6v0a2	6E-06	0.2
Dbt	2E-04	0.2	Tanc2	1E-06	0.2
Fbxo21	3E-06	0.2	Ifrd1	6E-12	0.2
Tctn3	3E-02	0.2	Cers2	1E-08	0.2
Npr2	4E-11	0.2	Cpeb4	1E-06	0.2
Aplp1	6E-04	0.2	Zfp623	1E-03	0.2
S100pbp	2E-06	0.2	Sparc	7E-26	0.2
Cep78	2E-05	0.2	Lins1	1E-03	0.2
Zfp113	2E-05	0.2	Tmem164	2E-04	0.2
Zfp963	6E-03	0.2	Usp3	9E-10	0.2
Polr1a	5E-05	0.2	Ing4	4E-08	0.2
Cnot6	3E-08	0.2	Morf4l2	5E-27	0.2
Dars	3E-09	0.2	Golim4	1E-11	0.2
Adss	2E-09	0.2	Kdm1a	4E-12	0.2
Ints6l	2E-04	0.2	Hdac8	5E-07	0.2
Maml2	6E-12	0.2	Cyld	6E-09	0.2
Tyw5	5E-05	0.2	Camsap2	5E-04	0.2
Mapkapk2	6E-07	0.2	Nol6	1E-04	0.2
Arhgap28	1E-12	0.2	Eif2ak3	9E-04	0.2
Pitpnb	2E-09	0.2	Trrap	5E-07	0.2
Usp39	6E-08	0.2	Ccdc8	3E-05	0.2
2610021A01Rik	4E-04	0.2	Zfp143	5E-04	0.2
Ahcyl1	2E-10	0.2	Rnmt	1E-07	0.2
Zmynd11	3E-11	0.2	Unc119	1E-06	0.2
Stxbp6	4E-07	0.2	Ears2	8E-05	0.2
Bcar1	5E-05	0.2	Rubcn	1E-05	0.2
Fam129a	1E-12	0.2	Pcdhga7	2E-02	0.2
Ube2f	2E-07	0.2	Eif2b3	1E-11	0.2
Aptx	2E-07	0.2	Paip2b	7E-06	0.2
Ttc19	3E-06	0.2	Pyroxd1	2E-07	0.2
Zfp810	8E-07	0.2	Pdxdc1	4E-10	0.2
Dlc1	8E-17	0.2	Prdm2	4E-08	0.2
Bcar3	1E-08	0.2	Tchp	3E-03	0.2
Dr1	5E-07	0.2	Qsox2	1E-05	0.2
Cers2	5E-08	0.2	Tead2	8E-07	0.2
Riok3	4E-09	0.2	Eif3a	8E-19	0.2
Copb2	8E-20	0.2	Gm37768	1E-04	0.2
Crtc2	2E-03	0.2	Stard6	2E-02	0.2
Zfp763	3E-03	0.2	1810026B05Rik	1E-07	0.2
Taf4b	2E-03	0.2	Sec22a	6E-07	0.2
Zfp628	4E-04	0.2	Txndc11	4E-05	0.2
Med20	2E-05	0.2	Strn4	6E-06	0.2
Zfp422	8E-06	0.2	Eif2s3x	6E-08	0.2
Chd2	4E-10	0.2	Dynlt3	1E-13	0.2
Gm9929	5E-03	0.2	AU041133	5E-04	0.2
Chchd4	5E-05	0.2	Zfp821	8E-06	0.2
Ubp1	8E-08	0.2	Nrbp1	2E-08	0.2
Ubxn7	2E-08	0.2	Snhg6	3E-13	0.2
Zdhhc5	4E-08	0.2	Baiap2	9E-05	0.2
Glcci1	5E-07	0.2	Asb6	2E-03	0.2
Cpq	2E-18	0.2	Fam241b	3E-04	0.2
Kdm5b	1E-13	0.2	Ankrd13b	4E-03	0.2
Pawr	4E-09	0.2	Smndc1	2E-07	0.2
Gid4	9E-08	0.2	Gpat4	3E-07	0.2
Atg16l1	4E-05	0.2	Slc11a2	4E-04	0.2
Mtfmt	7E-04	0.2	Hes1	6E-04	0.2
Igf2bp2	2E-08	0.2	Arhgef5	3E-05	0.2
Zfp568	4E-05	0.2	Hnrnpm	4E-14	0.2
Six4	5E-04	0.2	Purb	8E-10	0.2
Sos1	2E-07	0.2	Avpi1	3E-08	0.2
Nradd	2E-07	0.2	A630072M18Rik	2E-03	0.2
Taco1	2E-04	0.2	Pum1	2E-11	0.2
B4galt3	1E-05	0.2	Grb14	6E-08	0.2
2810433D01Rik	2E-05	0.2	Osbp	5E-08	0.2
Spout1	8E-05	0.2	Tmem39b	9E-04	0.2
Szrd1	1E-07	0.2	Dclk2	3E-01	0.2
Sehl1	1E-07	0.2	Pdss2	4E-05	0.2
Lrrc8a	4E-07	0.2	Hspa4	2E-16	0.2
Gtpbp10	6E-05	0.2	Fam135a	4E-07	0.2
Ykt6	4E-06	0.2	Rnf38	4E-05	0.2
Exoc5	5E-08	0.2	Acad11	3E-05	0.2
Tfb2m	2E-06	0.2	C1qtnf4	4E-06	0.2

Chondrogenic					
Control			NICD2		
Gene	*p*	FC	Gene	*p*	FC
Lrpap1	7E-15	0.2	Nbr1	7E-06	0.2
Ptpa	1E-07	0.2	Otud6b	2E-03	0.2
Lztfl1	2E-11	0.2	Zfp326	1E-05	0.2
Tmem30a	1E-13	0.2	Pfkfb3	3E-01	0.2
Zc2hc1a	1E-05	0.2	Zfp654	3E-03	0.2
Whamm	4E-02	0.2	Kdm3a	5E-01	0.2
Ift46	8E-04	0.2	Ccdc84	2E-01	0.2
Stambp	7E-07	0.2	Akap17b	2E-01	0.2
Alg12	6E-01	0.2	Vgll3	2E-03	0.2
Vgll4	1E-14	0.2	Stx3	2E-01	0.2
Fuz	4E-03	0.2	Vps13d	2E-03	0.2
Zkscan17	3E-02	0.2	Dpy19l3	4E-01	0.2
Zkscan8	3E-02	0.2	Slc25a12	3E-02	0.2
Marveld1	7E-05	0.2	Clk4	3E-03	0.2
Ppip5k1	3E-02	0.2	Atf6b	5E-02	0.2
Polb	3E-08	0.2	Stt3b	4E-07	0.2
4833439L19Rik	1E-06	0.2	Cap2	2E-01	0.2
Glg1	4E-17	0.2	Rab26os	7E-01	0.2
D10Wsu102e	7E-04	0.2	Tmem199	2E-01	0.2
Akap10	4E-03	0.2	Plekha8	1E-02	0.2
Abcb7	1E-02	0.2	Ganab	1E-05	0.2
D5Ertd579e	1E-05	0.2	Pcif1	7E-04	0.2
Acad11	6E-02	0.2	Leng9	1E-01	0.2
Zfp113	6E-02	0.2	Homer1	1E-01	0.2
Pomt2	1E-01	0.2	Pan2	6E-01	0.2
Ift88	9E-03	0.2	Gm1976	1E-02	0.2
Rnf170	7E-04	0.2	Ell	2E-01	0.2
Ccdc88a	2E-12	0.2	Pigp	1E-08	0.2
Gne	1E-02	0.2	Atrip	1E-01	0.2
Ift20	4E-24	0.2	Hfe	1E-02	0.2
Nr1d2	1E-04	0.2	Fan1	9E-02	0.2
Spg20	1E-08	0.2	Yrdc	1E-02	0.2
Bcl9	9E-05	0.2	Spata2	4E-01	0.2
Prkx	8E-01	0.2	1810013L24Rik	2E-03	0.2
Rnf14	7E-08	0.2	Ugp2	7E-12	0.2
Trim13	3E-01	0.2	Mbtd1	1E-04	0.2
Mob3c	2E-01	0.2	Lrp10	7E-04	0.2
Prrx1	1E-07	0.2	Ubr4	5E-06	0.2
Txlng	3E-06	0.2	4833439L19Rik	3E-04	0.2
Rnf141	8E-02	0.2	Sepsecs	2E-01	0.2
Zyg11b	2E-06	0.2	Etfrf1	6E-02	0.2
Kmt2d	1E-02	0.2	Msrb3	4E-08	0.2
Qk	8E-16	0.2	Gripap1	8E-05	0.2
Ttll11	2E-01	0.2	Pde4a	3E-01	0.2
Abcc1	3E-04	0.2	Fndc3a	1E-05	0.2
Dnmt3a	3E-04	0.2	Pomk	1E-01	0.2
Papolg	2E-01	0.2	Hbs1l	4E-03	0.2
Rbks	8E-02	0.2	Glg1	6E-16	0.2
Pak1ip1	7E-13	0.2	Med14	5E-02	0.2
Pomgnt1	1E-02	0.2	Tst	4E-02	0.2
Mir100hg	6E-15	0.2	B4galt4	4E-01	0.2
Ppm1k	4E-01	0.2	Polrmt	3E-01	0.2
Ggnbp1	8E-02	0.2	Sar1b	4E-09	0.2
Tmco6	5E-01	0.2	Arl2bp	1E-05	0.2
Gpt2	2E-01	0.2	Rhou	1E-01	0.2
Fam98b	4E-06	0.2	D2hgdh	7E-01	0.2
Plbd2	2E-09	0.2	Rbks	9E-01	0.2
Sec23b	2E-03	0.2	Chic1	5E-02	0.2
Ubr1	1E-06	0.2	Uggt2	2E-03	0.2
Camta2	6E-02	0.2	Akap10	3E-02	0.2
Abitram	2E-02	0.2	Luzp1	2E-07	0.2
Pomgnt2	3E-01	0.2	4930430F08Rik	3E-01	0.2
Borcs6	1E-02	0.2	Fubp1	3E-11	0.2
4921504A21Rik	8E-02	0.2	Gfod2	1E-01	0.2
Pex10	4E-01	0.2	Cox14	4E-18	0.2
Plpbp	2E-03	0.2	Ppp2r5a	1E-06	0.2
Ecsit	4E-02	0.2	Aff4	1E-11	0.2
Srpr	5E-18	0.2	Dcaf8	1E-07	0.2
Bahcc1	5E-02	0.2	Cfap298	3E-02	0.2
Sec22a	1E-04	0.2	Pls3	5E-11	0.2
Tvp23a	1E-01	0.2	Gpr89	2E-03	0.2
B9d1	5E-04	0.2	Ing3	2E-02	0.2
Pigf	3E-02	0.2	Zfp398	4E-02	0.2

Fibroblast					
Control			NICD2		
Gene	*p*	FC	Gene	*p*	FC
Trim34a	1E-02	0.1	Hdac4	2E-02	0.1
Rps8	5E-128	0.1	Rnf41	5E-05	0.1
Bag2	3E-03	0.1	Cyb5r3	3E-14	0.1
Tnip1	2E-05	0.1	Agps	1E-05	0.1
Zfp780b	3E-03	0.1	Ccdc71l	2E-01	0.1
Dpy19l4	7E-06	0.1	Plekhf1	4E-04	0.1
Irf1	6E-04	0.1	Wdr25	8E-01	0.1
Pex19	2E-05	0.1	Rbfox1	2E-01	0.1
Tmem50a	2E-29	0.1	Dlg5	3E-03	0.1
Nol3	2E-05	0.1	Tpp1	9E-12	0.1
Pcdhb17	1E-01	0.1	Uhrf1bp1	1E-01	0.1
Klhl28	8E-03	0.1	Mgmt	2E-10	0.1
Shf	2E-02	0.1	Cep350	5E-06	0.1
Eef2	6E-56	0.1	Crebl2	1E-01	0.1
Flad1	3E-04	0.1	Rpl30	3E-72	0.1
Gamt	7E-11	0.1	Zbtb8a	1E-01	0.1
Zdhhc17	4E-06	0.1	Fbxo46	4E-01	0.1
Angptl2	2E-04	0.1	Aldh16a1	1E-01	0.1
Sin3b	8E-23	0.1	Wdr89	1E-05	0.1
Pkd2	7E-12	0.1	Zeb2os	9E-03	0.1
Vti1b	2E-17	0.1	Mb21d2	3E-01	0.1
Nagk	7E-13	0.1	Rps4x	5E-61	0.1
Mapkapk3	2E-02	0.1	Zfat	3E-01	0.1
Enoph1	7E-02	0.1	Ssr4	7E-33	0.1
Rfng	9E-02	0.1	Iqce	7E-03	0.1
Osbpl7	3E-02	0.1	Gamt	6E-08	0.1
Ttbk2	3E-02	0.1	Insyn2a	9E-02	0.1
Sqor	1E-08	0.1	Naglu	4E-02	0.1
Zbtb44	8E-05	0.1	1500015L24Rik	4E-03	0.1
Zfp365	2E-02	0.1	Pycr1	7E-06	0.1
Rpia	4E-01	0.1	Katnal1	1E-02	0.1
Focad	6E-05	0.1	Vps26b	7E-03	0.1
Copz2	8E-26	0.1	Usp46	5E-06	0.1
Rpl14	8E-91	0.1	Rpl21	3E-65	0.1
Atxn7l1	8E-06	0.1	Washc4	1E-06	0.1
Msrb3	2E-06	0.1	Prelid2	2E-03	0.1
Iars2	4E-08	0.1	Clk3	2E-03	0.1
Psap	5E-60	0.1	Srgn	2E-01	0.1
Gm15050	8E-03	0.1	Rian	4E-06	0.1
Kdm4c	5E-09	0.1	Rpl13a	3E-55	0.1
Sco2	2E-05	0.1	Usp21	2E-01	0.1
Cog4	4E-08	0.1	Pofut1	1E-02	0.1
Eif2s3y	7E-08	0.1	Mex3b	3E-01	0.1
Cdc42ep3	2E-06	0.1	Csf2ra	7E-04	0.1
Tasor	3E-06	0.1	Rpl23	7E-84	0.1
AU022252	7E-04	0.1	Mapk1ip1	6E-02	0.1
Fam199x	7E-02	0.1	Mpv17l	4E-03	0.1
Plpp5	1E-06	0.1	Cog3	3E-02	0.1
1600012H06Rik	1E-05	0.1	Armcx2	2E-03	0.1
Slc26a11	1E-01	0.1	Zfp61	1E-01	0.1
Icmt	1E-01	0.1	Nphp3	1E-01	0.1
Pcyt2	3E-06	0.1	Cavin1	5E-14	0.1
Csf2ra	1E-04	0.1	Lmna	2E-23	0.1
Arhgap10	2E-06	0.1	Nmi	5E-02	0.1
Selenon	2E-07	0.1	Sipa1l3	2E-02	0.1
Hs1bp3	4E-05	0.1	Mark4	1E-01	0.1
Arl13b	3E-07	0.1	Erlec1	4E-15	0.1
Stom	1E-03	0.1	Sparcl1	2E-06	0.1
Irs1	1E-09	0.1	Tcn2	2E-06	0.1
Large1	2E-16	0.1	Cc2d1a	4E-02	0.1
Mrps36	5E-21	0.1	Trmt13	6E-02	0.1
Acsl5	5E-04	0.1	Mylip	3E-04	0.1
Rplp2	2E-124	0.1	Strn3	9E-14	0.1
Prdm11	8E-01	0.1	AU022252	2E-02	0.1
Pparg	1E-47	0.1	Coq8b	8E-03	0.1
Nit2	3E-05	0.1	Rps2	6E-39	0.1
Zfp870	4E-02	0.1	Armc6	2E-01	0.1
Derl1	4E-20	0.1	Sesn2	1E-02	0.1
Rpl18	7E-99	0.1	Gpnmb	7E-25	0.1
Cep350	8E-08	0.1	2310039H08Rik	5E-04	0.1
Eif4e2	3E-21	0.1	Nr4a1	1E+00	0.1
Atxn7l3	8E-06	0.1	Twist1	9E-09	0.1
Hefc1r1	1E-26	0.1	Ercc4	1E-01	0.1

Fibroblast?					
Control			NICD2		
Gene	*p*	FC	Gene	*p*	FC
Calm1	4E-92	0.5	Snu13	2E-49	0.5
Polr3g	2E-10	0.5	Mphosph8	1E-39	0.5
Sars2	3E-11	0.5	5430416N02Rik	8E-31	0.5
Med11	3E-10	0.5	1810009A15Rik	6E-13	0.5
Arpc5l	5E-38	0.5	Snta1	8E-25	0.5
Tmem47	1E-25	0.5	Ppp4r3a	9E-26	0.5
Tmem131l	7E-21	0.5	Shisa2	5E-14	0.5
Rheb	3E-51	0.5	Slc35c1	4E-09	0.5
Tmx2	1E-22	0.5	Mrto4	1E-30	0.5
Coq2	2E-27	0.5	Phactr4	7E-25	0.5
Mrto4	3E-16	0.5	Idh2	6E-53	0.5
Trmt6	7E-18	0.5	Mtrr	2E-17	0.5
Zc3h7a	1E-20	0.5	Tarbp1	5E-09	0.5
Vps16	3E-22	0.5	Trpv4	5E-14	0.5
Pxk	6E-19	0.5	Tmed5	7E-37	0.5
Gm15489	2E-13	0.5	Nfya	4E-20	0.5
Car13	2E-21	0.5	Rock2	9E-66	0.5
Gpx7	2E-21	0.5	Bmpr1b	9E-34	0.5
Dubr	3E-19	0.5	Zc3h18	4E-31	0.5
Layn	2E-19	0.5	Trir	6E-67	0.5
Plagl2	2E-18	0.5	Otud6b	8E-26	0.5
Soga1	2E-16	0.5	Ptbp1	5E-30	0.5
Ermp1	1E-20	0.5	Trmt2b	2E-20	0.5
Ints11	8E-24	0.5	Srsf10	1E-47	0.5
Evc	5E-20	0.5	Pacsin2	2E-33	0.5
Smpd4	7E-19	0.5	Oxnad1	8E-23	0.5
Car5b	4E-22	0.5	Raly	1E-50	0.5
Abhd2	2E-23	0.5	Arpc5l	8E-49	0.5
Stxbp1	4E-22	0.5	Pcmt1	4E-56	0.5
Efcab7	1E-18	0.5	Plagl2	4E-23	0.5
Cbx6	3E-25	0.5	Psd3	2E-36	0.5
Cd109	4E-26	0.5	Ddx1	1E-52	0.5
Celf4	4E-19	0.5	Bex3	8E-42	0.5
Arsi	3E-17	0.5	Dok1	3E-17	0.5
Sh3bp1	1E-13	0.5	Dhrs4	6E-37	0.5
Ess2	2E-15	0.5	Cpd	2E-32	0.5
Lrrc57	6E-19	0.5	Pank2	3E-37	0.5
Pde12	4E-16	0.5	Ifi27l2a	8E-12	0.5
Nos1ap	6E-14	0.5	Dynll2	2E-55	0.5
Alg6	1E-23	0.5	Tmem106c	4E-33	0.5
Platr22	1E-09	0.5	Fus	4E-63	0.5
Slx4ip	7E-21	0.5	Klf13	9E-32	0.5
Map3k3	2E-19	0.5	Rbm43	3E-30	0.5
Cpd	3E-24	0.5	Trmt6	2E-25	0.5
Mrpl14	3E-42	0.5	Myadm	2E-30	0.5
Ltbp1	2E-21	0.5	Plekhf2	1E-16	0.5
Sin3a	5E-23	0.5	Srrm1	1E-67	0.5
Acap3	4E-18	0.5	Sf3b4	5E-31	0.5
Mier2	3E-13	0.5	Fkbp4	3E-32	0.5
Litaf	7E-23	0.5	Depdc5	2E-23	0.5
Mfsd14b	2E-20	0.5	Cerk	5E-25	0.5
Mrpl12	9E-36	0.5	2900041M22Rik	4E-14	0.5
Smtn	3E-19	0.5	2500004C02Rik	4E-13	0.5
Camkk1	2E-10	0.5	Wdr12	5E-18	0.5
Tardbp	9E-28	0.5	Rcan3	2E-23	0.5
Esd	2E-26	0.5	Ywhaq	3E-66	0.5
Zc3h8	2E-15	0.5	Ino80e	7E-25	0.5
Zbed4	3E-11	0.5	Enho	9E-19	0.5
Tor1a	4E-18	0.5	Otud4	7E-23	0.5
Ncl	6E-50	0.5	Mtap	4E-21	0.5
Prpf3	5E-22	0.5	Ttc7	3E-25	0.5
Ccdc134	1E-14	0.5	Pex	6E-39	0.5
Syncrip	1E-37	0.5	Efr3b	2E-23	0.5
Gab2	3E-27	0.5	Gpaa1	1E-30	0.5
Gsdme	5E-23	0.5	Aebp2	9E-30	0.5
Bag2	4E-14	0.5	Mrps11	6E-27	0.5
Cdk2ap1	9E-24	0.5	Pfn1	5E-110	0.5
Ccdc167	2E-24	0.5	Polr3k	1E-29	0.5
Immt	3E-47	0.5	Zc3h7a	1E-28	0.5
Ablim1	1E-22	0.5	Htra2	3E-29	0.5
Pigf	4E-21	0.5	Ctdp1	9E-23	0.5
Lrrc59	7E-31	0.5	B230219D22Rik	8E-70	0.5
Klhl5	8E-24	0.5	Ece2	7E-23	0.5

Undefined					
Control			NICD2		
Gene	*p*	FC	Gene	*p*	FC

Articular/Synovial Fibroblast					
Control			NICD2		
Gene	*p*	FC	Gene	*p*	FC
Tesk2	5E-01	0.2	Srd5a1	3E-01	0.2
Prps2	9E-01	0.2	Fam193b	6E-01	0.2
B4gat1	4E-01	0.2	Abcb8	7E-01	0.2
Hmgcl	7E-01	0.2	Prps2	7E-01	0.2
Eid1	9E-14	0.2	Rbl2	8E-01	0.2
Tbck	3E-03	0.2	Wdr59	6E-01	0.2
Eci2	2E-06	0.2	Acox1	5E-02	0.2
1110019D14Rik	9E-01	0.2	Rsrp1	6E-07	0.2
Zfp976	1E+00	0.2	Brpf3	5E-01	0.2
Cpeb4	2E-01	0.2	Atox1	8E-11	0.2
Mta3	4E-02	0.2	Zfp623	6E-01	0.2
Pdpr	9E-01	0.2	Acp2	7E-01	0.2
Use1	1E-08	0.2	Dusp11	7E-03	0.2
Ess2	8E-01	0.2	Eid1	1E-08	0.2
Islr	2E-05	0.2	1300002E11Rik	2E-01	0.2
Pigl	4E-01	0.2	Oxsm	6E-01	0.2
Slc2a13	5E-01	0.2	Igfbp6	7E-04	0.2
Pcbd2	5E-06	0.2	Cc2d1a	1E+00	0.2
Galnt1	4E-03	0.2	Zbtb26	2E-01	0.2
Dazap2	1E-01	0.2	Ubac2	4E-01	0.2
Ifngr1	1E-01	0.2	Zhx3	2E-01	0.2
Osgepl1	5E-01	0.2	Tax1bp3	4E-02	0.2
Atp11c	6E-01	0.2	Thtpa	2E-01	0.2
Trp53cor1	5E-01	0.2	Slc12a9	6E-01	0.2
Casp9	4E-01	0.2	Tbc1d20	8E-03	0.2
Nhlrc3	6E-01	0.2	Rnf170	9E-01	0.2
Fbxl3	3E-02	0.2	Flnc	7E-02	0.2
Zfp36l2	1E-03	0.2	Plod1	2E-02	0.2
Ccdc93	6E-01	0.2	Parp4	4E-01	0.2
Ypel2	6E-01	0.2	Myo6	4E-01	0.2
Vipas39	9E-01	0.2	Socs4	6E-01	0.2
Mapkapk3	9E-01	0.2	Zfp949	7E-01	0.2
Elf1	4E-03	0.2	Gng2	2E-01	0.2
Fam172a	6E-06	0.2	Oat	2E-07	0.2
Casp6	5E-01	0.2	Arsa	9E-01	0.2
Cc2d1a	9E-01	0.2	A930037H05Rik	7E-01	0.2
Sun1	8E-01	0.2	Ncoa7	2E-01	0.2
Svep1	2E-08	0.2	Plekhn1	6E-01	0.2
Zkscan6	7E-01	0.2	Zfp317	8E-01	0.2
Pcdhgc3	9E-01	0.2	Glis1	1E+00	0.2
Sfmbt1	5E-01	0.2	Mbtps2	5E-01	0.2
Barx1	4E-08	0.2	Lrrc29	6E-01	0.2
Ints9	8E-01	0.2	Dnajc14	7E-01	0.2
Lta4h	2E-01	0.2	Pdia5	7E-03	0.2
Disp1	6E-02	0.2	Rnf123	9E-01	0.2
Ube3b	8E-01	0.2	Mif	4E-06	0.2
Myl12b	1E-06	0.2	A530072M11Rik	3E-01	0.2
Ttll11	9E-01	0.2	Arhgef10	4E-01	0.2
Retsat	7E-01	0.2	Krt10	5E-01	0.2
Cpne3	2E-02	0.2	Fam126b	7E-01	0.2
Dip2a	6E-01	0.2	Zmym3	4E-01	0.2
Tcf7l1	1E-02	0.2	Casp12	1E-02	0.2
Ncoa2	1E-02	0.2	Auh	2E-02	0.2
Polr3gl	2E-01	0.2	Lpar1	5E-02	0.2
Tmem127	5E-01	0.2	Dnajc24	8E-02	0.2
Adam10	3E-04	0.2	Trim11	2E-01	0.2
Ggact	1E+00	0.2	Ak1	2E-02	0.2
Nme7	6E-02	0.2	Cd164	3E-03	0.2
Igbp1	5E-03	0.2	Selenow	3E-06	0.2
C1rl	6E-01	0.2	Idnk	6E-01	0.2
Emc9	1E-02	0.2	Tollip	8E-01	0.2
Mapkbp1	5E-01	0.2	Relb	7E-01	0.2
Ier5l	1E-01	0.2	Zfand2a	7E-01	0.2
Cep85	9E-01	0.2	Nestn	7E-02	0.2
2810403D21Rik	2E-01	0.2	Lrwd1	4E-01	0.2
Dennd4c	6E-02	0.2	Plpp2	1E+00	0.2
Gatd1	1E+00	0.2	Tbcel	8E-01	0.2
Zfp65	1E+00	0.2	Cryzl2	7E-01	0.2
Dleu2	3E-04	0.2	Stim1	4E-02	0.2
Pcdhga7	6E-01	0.2	Ttll7	8E-01	0.2
Apbb3	4E-01	0.2	Mcat	3E-01	0.2
Pip4k2b	5E-01	0.2	Plin2	2E-04	0.2
Sestd1	3E-01	0.2	Mical1	4E-01	0.2

Limb Mesenchyme					
Control			NICD2		
Gene	p	FC	Gene	p	FC
Senp2	6E-05	0.2	Zbtb7b	8E-05	0.2
Ift20	5E-21	0.2	Utp20	2E-04	0.2
Pak1ip1	3E-13	0.2	Rnf19b	2E-04	0.2
Cd2ap	7E-13	0.2	Csnk1a1	4E-23	0.2
Pwp1	8E-07	0.2	Hmbox1	2E-06	0.2
Fam160b1	3E-06	0.2	Lrrc8a	1E-04	0.2
Pum2	5E-10	0.2	Csnk1g2	1E-07	0.2
Alg1	1E-05	0.2	Scmh1	5E-09	0.2
Pls3	2E-12	0.2	Pik3ca	4E-06	0.2
Pcdhb14	1E-01	0.2	Ddx23	1E-04	0.2
Gm26887	7E-06	0.2	Rtca	2E-04	0.2
Dstyk	1E-06	0.2	AI506816	9E-11	0.2
Lin52	3E-04	0.2	Ganab	1E-06	0.2
BC031181	2E-17	0.2	Malt1	2E-02	0.2
Apbb3	5E-04	0.2	Zdhhc20	5E-08	0.2
Abraxas1	5E-03	0.2	Ncstn	1E-06	0.2
Nmt2	4E-06	0.2	Zkscan17	6E-03	0.2
Ythdf3	2E-09	0.2	Stxbp5	3E-06	0.2
Zfp330	2E-06	0.2	Hira	6E-05	0.2
1700123O20Rik	2E-08	0.2	Cbx4	2E-05	0.2
Gale	1E-05	0.2	Usp39	2E-05	0.2
Ttc37	3E-08	0.2	Vps37a	3E-06	0.2
Cep41	6E-06	0.2	Afdn	3E-06	0.2
Metap1	2E-05	0.2	Erich1	1E-07	0.2
Vps39	5E-05	0.2	Trmt10c	1E-05	0.2
Lin7c	3E-09	0.2	Rfx1	1E-01	0.2
Gbf1	3E-13	0.2	Lonrf1	8E-04	0.2
4921524J17Rik	3E-08	0.2	Casc3	5E-06	0.2
Sfxn1	1E-09	0.2	Mon2	1E-06	0.2
Hnrnpdl	4E-11	0.2	Eif1a	4E-13	0.2
Sdc2	9E-22	0.2	Golga5	4E-08	0.2
Orai2	3E-04	0.2	2310022B05Rik	6E-06	0.2
Herc1	3E-06	0.2	Zfp282	2E-03	0.2
Cdkn2aipnl	2E-05	0.2	Ppp1r12a	3E-08	0.2
Spaca6	2E-07	0.2	Mras	2E-07	0.2
Pheta1	5E-04	0.2	Alkbh8	6E-06	0.2
Ggcx	2E-07	0.2	Prdm4	8E-03	0.2
Xbp1	3E-09	0.2	Chmp6	2E-04	0.2
Krr1	3E-05	0.2	Pram1	1E-01	0.2
Top3b	4E-07	0.2	Plscr1	1E-03	0.2
Dpy19l3	2E-04	0.2	Zfp408	2E-05	0.2
Suco	5E-07	0.2	Eif3b	6E-10	0.2
Eif1a	2E-13	0.2	Ccdc30	5E-05	0.2
Csnk1a1	9E-27	0.2	Cstf3	7E-07	0.2
Arhgap12	4E-06	0.2	Mindy4	2E-05	0.2
Lsm14a	9E-09	0.2	Usp16	5E-08	0.2
Zbtb2	2E-02	0.2	2410006H16Rik	3E-09	0.2
Mid1	2E-17	0.2	Ngrn	1E-06	0.2
Ftx	4E-07	0.2	Ap3m1	5E-05	0.2
Pogz	5E-04	0.2	Zfyve16	7E-06	0.2
Trappc10	3E-07	0.2	Kdm5c	1E-07	0.2
Prkab2	2E-03	0.2	Trappc2	8E-05	0.2
Elk4	1E-05	0.2	Wdyhv1	3E-04	0.2
Chtop	8E-09	0.2	Spen	2E-06	0.2
Prkg2	4E-02	0.2	Plcg1	2E-05	0.2
Rbpj	9E-16	0.2	Srp68	1E-06	0.2
S100a1	1E-19	0.2	Fem1c	6E-06	0.2
Rhou	1E-06	0.2	Stxbp4	8E-07	0.2
Supt5	3E-10	0.2	Chd6	7E-09	0.2
Entr1	7E-07	0.2	Rnf167	5E-05	0.2
Caprin1	1E-18	0.2	Cdk12	3E-09	0.2
Taf8	7E-03	0.2	Rwdd4a	5E-06	0.2
Peli1	1E-06	0.2	Gm4285	8E-04	0.2
2610035D17Rik	1E-05	0.2	Rc3h2	3E-06	0.2
Casc3	3E-05	0.2	Dus3l	2E-04	0.2
Ptbp1	1E-08	0.2	Arhgdig	2E-03	0.2
Creb1	9E-09	0.2	Ranbp17	5E-05	0.2
Rgs10	1E-05	0.2	Slc30a5	3E-08	0.2
Foxj2	4E-05	0.2	Prrc2a	3E-08	0.2
Golim4	1E-14	0.2	Gm10762	4E-04	0.2
Tbc1d22b	1E-04	0.2	Adamtsl4	3E-07	0.2
Lsg1	2E-08	0.2	Hace1	3E-03	0.2
2810021J22Rik	1E-03	0.2	Kctd12	5E-05	0.2

Chondrogenic					
Control			NICD2		
Gene	p	FC	Gene	p	FC
Krt10	1E-03	0.2	Gm10762	3E-01	0.2
Fbxo9	6E-05	0.2	Pus10	6E-02	0.2
Srr	2E-03	0.2	Plekha3	1E-02	0.2
Mttp	1E-01	0.2	Ubxn6	9E-09	0.2
Dyrk1b	2E-01	0.2	Ppp1r15a	3E-03	0.2
Bivm	4E-02	0.2	Fbxl5	1E-02	0.2
Znrd1as	7E-02	0.2	Gm16552	2E-01	0.2
Setd6	6E-01	0.2	Exd2	1E-01	0.2
Vkorc1	2E-24	0.2	Zkscan8	3E-01	0.2
Lancl1	1E-04	0.2	Sphk2	7E-01	0.2
Med14	2E-04	0.2	Pgghg	1E-01	0.2
Zfp219	8E-02	0.2	Tfdp2	6E-06	0.2
Rnf138	5E-04	0.2	Phykpl	2E-03	0.2
Kdm3b	7E-06	0.2	2610020C07Rik	6E-02	0.2
Ndnf	6E-04	0.2	Mmut	2E-03	0.2
Mia2	3E-09	0.2	Gtf2a1	2E-03	0.2
Cfap69	2E-01	0.2	St6galnac4	5E-01	0.2
Araf	1E-07	0.2	Pnrc1	1E-10	0.2
Rnpepl1	2E-01	0.2	Colec12	1E-01	0.2
Impa2	3E-02	0.2	Specc1l	7E-06	0.2
Fbxl18	6E-01	0.2	Mapre2	3E-04	0.2
Arid1b	2E-09	0.2	Erlec1	2E-14	0.2
Gm32031	2E-01	0.2	Abitram	6E-02	0.2
Fkbp14	3E-07	0.2	D16Ertd472e	5E-02	0.2
Dgkd	2E-02	0.2	Tmtc2	3E-01	0.2
Mar9	7E-02	0.2	Ubl3	5E-05	0.2
Osbp	1E-06	0.2	Crebl2	5E-01	0.2
Pttg1	3E-05	0.2	Dzip1l	2E-01	0.2
Chd2	2E-08	0.2	Trappc10	5E-03	0.2
Bccip	3E-07	0.2	Zfp523	5E-01	0.2
Selenok	2E-25	0.2	Nat10	6E-01	0.2
Riox1	4E-01	0.2	Ppt2	1E-01	0.2
Narf	4E-03	0.2	Etfa	5E-10	0.2
Tmx2	1E-01	0.2	Resf1	1E-01	0.2
Paip2	7E-25	0.2	Rnf24	3E-02	0.2
Zfp322a	1E-04	0.2	Mosmo	2E-03	0.2
Patz1	4E-02	0.2	Fcho2	9E-07	0.2
Trp53bp2	1E-01	0.2	Sap18b	7E-01	0.2
Jrk	9E-01	0.2	Agbl5	1E-01	0.2
Phtf1	2E-06	0.2	Mylk	2E-01	0.2
Tm9sf2	3E-13	0.2	Rbm4b	4E-03	0.2
Atg13	8E-04	0.2	Vps11	6E-02	0.2
Rab3gap1	4E-06	0.2	Smad7	1E-02	0.2
Ift80	4E-03	0.2	Map3k3	2E-03	0.2
Arnt2	6E-01	0.2	Mtmr1	9E-02	0.2
Herc1	8E-04	0.2	Smn1	5E-04	0.2
Mbd2	5E-11	0.2	Ruvbl1	2E-08	0.2
Parg	4E-05	0.2	1110051M20Rik	3E-04	0.2
Ube4b	3E-07	0.2	Tex2	1E-01	0.2
Spryd3	3E-03	0.2	Ubr1	2E-05	0.2
Fan1	2E-01	0.2	Armh3	3E-03	0.2
Mcts2	2E-02	0.2	Kmt2c	2E-07	0.2
Grb10	2E-12	0.2	Ubap1	3E-02	0.2
Tle6	3E-01	0.2	0610009L18Rik	9E-01	0.2
1700109K24Rik	1E-01	0.2	2900076A07Rik	4E-02	0.2
2900097C17Rik	3E-10	0.2	Nhs	1E-06	0.2
Mblac2	2E-01	0.2	Junos	3E-01	0.2
Purb	2E-08	0.2	Ift172	8E-02	0.2
Phospho2	2E-02	0.2	Cpeb3	9E-03	0.2
Ubxn8	2E-02	0.2	Prr3	8E-02	0.2
Tra2b	1E-09	0.2	Hk1	2E-05	0.2
Rai1	7E-03	0.2	Cpsf6	5E-06	0.2
Slc30a9	8E-05	0.2	Ttc13	2E-01	0.2
Cbx5	6E-15	0.2	Hist2h2aa1	6E-01	0.2
Afdn	2E-06	0.2	Ormdl1	7E-01	0.2
Ttc38	4E-01	0.2	Dym	2E-02	0.2
Parp4	7E-04	0.2	Zfp609	3E-03	0.2
Prss36	2E-01	0.2	Myo18a	1E-02	0.2
Arl6	1E-01	0.2	Echdc3	5E-01	0.2
Snx25	1E-01	0.2	Pam16	1E-08	0.2
Gid4	9E-06	0.2	Scnm1	2E-02	0.2
Oaz2	3E-06	0.2	Wdr46	6E-02	0.2
H2afv	1E-34	0.2	Nme6	1E-01	0.2

Fibroblast					
Control			NICD2		
Gene	p	FC	Gene	p	FC
Dnm1	1E-13	0.1	Zc3h10	8E-01	0.1
B4galt4	1E-02	0.1	Bahd1	3E-01	0.1
Dynll1	1E-45	0.1	Ptcd1	3E-01	0.1
Cpne1	7E-08	0.1	Tmed3	6E-19	0.1
Cep170	4E-11	0.1	Hexa	8E-10	0.1
Zfp280b	1E-01	0.1	Rpl7	2E-63	0.1
Dync1h1	1E-16	0.1	Srp54a	1E-02	0.1
Inpp1	1E-03	0.1	Zfp938	3E-02	0.1
Tnrc6c	2E-16	0.1	Zfp354c	1E-05	0.1
Ipo13	1E-01	0.1	Ssh1	2E-03	0.1
Rps23	2E-123	0.1	Vps35l	3E-05	0.1
Ctps	3E-07	0.1	Pstk	1E-04	0.1
Suco	7E-05	0.1	Txnip	4E-10	0.1
Kdm1b	1E-01	0.1	Ttc38	3E-01	0.1
Ssr4	4E-51	0.1	Snhg17	2E-02	0.1
Gys1	2E-05	0.1	Uspl1	4E-01	0.1
Cyp4f13	6E-03	0.1	Rpl27	5E-63	0.1
Extl2	3E-04	0.1	Cav1	2E-12	0.1
Wrb	4E-03	0.1	Klhdc8b	1E-02	0.1
Ick	2E-08	0.1	Glb1	2E-04	0.1
Ddah1	1E-01	0.1	Ech1	7E-15	0.1
Cc2d1a	1E-02	0.1	Parn	2E-03	0.1
Mta3	5E-08	0.1	Erbb2	3E-01	0.1
Ints6	3E-05	0.1	Zdhhc9	6E-03	0.1
B230307C23Rik	5E-04	0.1	Yif1a	7E-09	0.1
Poglut3	3E-07	0.1	Pkd2	8E-07	0.1
Rps26	1E-85	0.1	Llgl1	4E-01	0.1
Gm15283	5E-11	0.1	Nudt7	2E-03	0.1
Snhg17	1E-02	0.1	Pabpc1	1E-24	0.1
Gm4285	1E-01	0.1	Runx2	7E-21	0.1
Il10rb	3E-04	0.1	Gcc2	4E-05	0.1
Syngap1	2E-01	0.1	Rbp1	2E-02	0.1
Map1s	2E-03	0.1	Zfp947	9E-01	0.1
Katnal1	4E-04	0.1	Thoc6	1E-03	0.1
Klhl18	3E-02	0.1	Atp13a1	1E-02	0.1
Mpzl1	2E-03	0.1	Pja1	1E-04	0.1
Rpl35a	8E-108	0.1	Kirrel	3E-08	0.1
Chfr	1E-05	0.1	Dhx57	2E-03	0.1
Six1	1E-02	0.1	Dnm2	2E-06	0.1
Gdi1	6E-08	0.1	Pskh1	3E-01	0.1
Yipf3	5E-15	0.1	Syngap1	3E-01	0.1
Tfpt	1E-03	0.1	Mex3c	1E-02	0.1
Pelo	3E-05	0.1	Alpk1	1E-02	0.1
Mustn1	9E-05	0.1	Tradd	1E-03	0.1
Rchy1	9E-09	0.1	Mif	1E-19	0.1
Atp13a1	6E-04	0.1	Stom	1E-02	0.1
Usp9x	2E-15	0.1	Mar9	3E-01	0.1
Serpinb9	5E-03	0.1	Dock7	2E-07	0.1
Bad	2E-14	0.1	Usp9x	2E-09	0.1
Sec24a	8E-07	0.1	Akr1b8	9E-05	0.1
Tceal1	3E-04	0.1	Ick	3E-05	0.1
Usp46	2E-10	0.1	Nadk2	2E-02	0.1
Bzw2	2E-09	0.1	Pmp22	1E-13	0.1
Pdha1	7E-16	0.1	Stard10	5E-04	0.1
Ncoa7	3E-05	0.1	Sox12	4E-01	0.1
Dbnl	4E-12	0.1	Pcnx3	9E-02	0.1
Atg2b	6E-03	0.1	Gm45669	2E-01	0.1
Umad1	1E-03	0.1	Nudt18	6E-02	0.1
Pdzd8	4E-06	0.1	Rpl10a	4E-48	0.1
Tbc1d25	6E-02	0.1	Fam189b	3E-03	0.1
Rpl23a	9E-61	0.1	1110038B12Rik	6E-17	0.1
Csnk1g1	5E-11	0.1	Il10rb	1E-02	0.1
Zfc3h1	4E-11	0.1	Pelo	1E-04	0.1
Plxna3	3E-01	0.1	Pcyt2	5E-03	0.1
Zfp324	3E-01	0.1	Bcorl1	3E-02	0.1
Nectin1	2E-01	0.1	Tbc1d20	5E-06	0.1
Zfp518a	2E-02	0.1	Icmt	5E-01	0.1
Htra2	6E-07	0.1	Wasf3	1E-02	0.1
Mbd5	3E-10	0.1	Creb3l2	3E-05	0.1
Ddit4	2E-05	0.1	Grk5	2E-04	0.1
Arl1	2E-28	0.1	Hsp90b1	2E-15	0.1
C1qtnf12	1E-07	0.1	Cdk5	2E-02	0.1
Fam207a	7E-05	0.1	Sys1	3E-11	0.1

Fibroblast?					
Control			NICD2		
Gene	p	FC	Gene	p	FC
Ints1	5E-18	0.5	Plekhh3	3E-19	0.5
Zbtb41	1E-22	0.5	Ube2n	7E-41	0.5
Msl3	8E-34	0.5	Abcg2	6E-23	0.5
Dhx9	5E-31	0.5	Fh1	4E-28	0.5
Utp20	7E-22	0.5	Prrg1	7E-32	0.5
Snx16	2E-15	0.5	Lage3	1E-36	0.5
Btbd19	5E-14	0.5	Agpat5	1E-27	0.5
Lrrc75b	1E-14	0.5	Tmem201	1E-16	0.5
Nmnat1	6E-03	0.5	Nqo2	2E-28	0.5
Nacc1	3E-20	0.5	Gucy1a1	7E-14	0.5
Polr3k	2E-25	0.5	Desi1	7E-23	0.5
Grwd1	2E-10	0.5	Tpgs2	2E-29	0.5
Pdpn	9E-18	0.5	Zfp275	2E-22	0.5
Trappc13	2E-23	0.5	Zfp568	2E-22	0.5
Tmem154	4E-13	0.5	Uaca	4E-18	0.5
Ap1s1	6E-30	0.5	Nav1	3E-28	0.5
Cdr2	1E-14	0.5	Crabp2	8E-15	0.5
Khsrp	2E-22	0.5	Pakap.1	6E-41	0.5
Wars	6E-15	0.5	Gal3st4	1E-09	0.5
Tmod2	8E-13	0.5	Coq2	7E-31	0.5
Nova1	3E-15	0.5	Filip1l	2E-18	0.5
Dtnb	2E-21	0.5	Stom	2E-16	0.5
Celf5	2E-11	0.5	Med19	2E-27	0.5
Ptbp3	7E-23	0.5	Ccdc88a	2E-42	0.5
Gsdmd	7E-13	0.5	Snx25	3E-24	0.5
Ntn4	2E-13	0.5	Zfp111	7E-22	0.5
Efhd2	4E-23	0.5	Arsj	1E-34	0.5
Tnc	3E-17	0.5	Pcgf6	1E-19	0.5
Farp2	1E-20	0.5	Apaf1	2E-27	0.5
Ehbp1l1	1E-17	0.5	Gipc1	6E-26	0.5
Lrp1b	3E-06	0.5	Pwwp2a	8E-26	0.5
Gab3	2E-12	0.5	Ppp4r4	4E-11	0.5
Gm43728	6E-10	0.5	Eva1c	6E-09	0.5
Manf	5E-57	0.5	Map3k3	5E-26	0.5
Pbx3	5E-35	0.5	Rasal2	9E-38	0.5
Aldh16a1	3E-16	0.5	Zfp280b	1E-21	0.5
Hmgn1	7E-114	0.5	Clip4	3E-20	0.5
Brpf1	2E-16	0.5	Pdzd11	4E-24	0.5
Tent5c	1E-11	0.5	Vangl1	2E-17	0.5
Gart	8E-18	0.5	Eogt	4E-21	0.5
Gfra4	6E-16	0.5	Gpx7	3E-22	0.5
Rab35	4E-22	0.5	Rprd1b	1E-27	0.5
Scrn2	3E-03	0.5	Ndufa5	6E-84	0.5
Trim28	3E-24	0.5	Polr2f	2E-40	0.5
Ankrd13a	1E-22	0.5	Lpcat4	1E-10	0.5
Snu13	2E-34	0.5	Cep170b	3E-25	0.5
Lzts3	3E-12	0.5	Arhgap32	6E-20	0.5
Trpm4	1E-12	0.5	Eif4e	1E-35	0.5
Pcsk6	3E-21	0.5	Klhl2	1E-19	0.5
Prdm4	5E-13	0.5	Sh3bp4	1E-11	0.5
Nsmce3	7E-16	0.5	Pmm1	8E-28	0.5
Tcp1	5E-54	0.5	Fabp5	3E-75	0.5
Gm9887	1E-09	0.5	Fibin	3E-27	0.5
Eif6	4E-30	0.5	Tmem18	2E-17	0.5
Myl9	1E-10	0.5	Pctp	6E-11	0.5
Hars	3E-24	0.5	Asrgl1	5E-29	0.5
C79798	2E-12	0.5	Utp20	2E-27	0.5
Txndc12	2E-25	0.5	Smarcc1	6E-48	0.5
Adss	2E-23	0.5	Tspyl5	3E-11	0.5
Tigar	6E-10	0.5	Trpm4	2E-14	0.5
Evl	5E-22	0.5	Cdc42ep4	1E-10	0.5
Snhg15	4E-17	0.5	Mtmr14	2E-14	0.5
Mrps26	2E-26	0.5	Tcaf2	1E-15	0.5
Utp3	2E-42	0.5	Cand1	3E-22	0.5
Gbp2	8E-14	0.5	Eif4a3	7E-26	0.5
Zbtb48	4E-09	0.5	Tubb2b	4E-30	0.5
Actb	5E-68	0.5	Polr3g	5E-13	0.5
Spaca1	1E-06	0.5	Trmo	1E-13	0.5
Zfp958	3E-12	0.5	Rab35	9E-26	0.5
Lrrfip1	1E-27	0.5	Gar1	1E-23	0.5
Gar1	7E-18	0.5	Sf3a1	4E-25	0.5
Zfp180	2E-17	0.5	U2af1l4	2E-14	0.5
Il4ra	5E-13	0.5	Elf2	1E-33	0.5

Undefined					
Control			NICD2		
Gene	p	FC	Gene	p	FC

Articular/Synovial Fibroblast					
Control			NICD2		
Gene	p	FC	Gene	p	FC
Gm30025	8E-01	0.2	Mon1a	9E-01	0.2
Bri3	2E-11	0.2	Zeb1	8E-03	0.2
Gm15411	4E-01	0.2	Wipf2	8E-01	0.2
Taf1a	8E-01	0.2	Oxnad1	4E-01	0.2
Gucd1	5E-01	0.2	Atp6v1e1	2E-04	0.2
Mtmr1	1E+00	0.2	Tceal1	9E-01	0.2
Ddi2	9E-02	0.2	Slc27a3	2E-01	0.2
Zfp81	7E-01	0.2	Lrrk2	7E-01	0.2
Vps37d	8E-01	0.2	Cuedc2	2E-05	0.2
Rgl2	4E-01	0.2	Rab3a	8E-01	0.2
Ccdc167	2E-01	0.2	Zc3h3	9E-01	0.2
Insig1	4E-03	0.2	Arl5b	9E-01	0.2
Afg1l	1E+00	0.2	Sgpp1	3E-01	0.2
A430073D23Rik	8E-01	0.2	Prdx5	3E-06	0.2
2210016L21Rik	1E-03	0.2	Abl1	2E-01	0.2
Bphl	7E-03	0.2	Dcp1a	1E+00	0.2
Slc25a30	2E-01	0.2	Ufc1	7E-06	0.2
Snx9	9E-05	0.2	Rad51d	7E-01	0.2
Anxa7	9E-03	0.2	Nmi	5E-01	0.2
Zfp867	6E-01	0.2	Trp53cor1	8E-01	0.2
Fam126b	8E-01	0.2	Gtf2ird2	7E-01	0.2
Gm20275	8E-01	0.2	9230114K14Rik	3E-01	0.2
0610010F05Rik	8E-01	0.2	Arhgap28	2E-02	0.2
Rad51d	6E-01	0.2	Tmem176b	3E-06	0.2
Elmsan1	4E-01	0.2	Ptprs	1E-03	0.2
Trafd1	9E-02	0.2	Gm14164	5E-01	0.2
Adat2	3E-01	0.2	Fuz	5E-01	0.2
Usp45	5E-01	0.2	Gm16552	6E-01	0.2
Plcb3	4E-01	0.2	Scaper	9E-03	0.2
AU022252	1E+00	0.2	Cyp7b1	5E-01	0.2
Atp8b2	7E-01	0.2	Nrde2	5E-01	0.2
Kif1b	3E-04	0.2	Mettl3	5E-01	0.2
Cd302	3E-05	0.2	Noct	2E-01	0.2
Sphk2	1E+00	0.2	4632427E13Rik	4E-02	0.2
Acads	4E-02	0.2	Rap2b	9E-01	0.2
Zfp87	6E-01	0.2	Map9	8E-02	0.2
Rab43	8E-01	0.2	Ino80c	8E-01	0.2
Synj1	8E-01	0.2	Zfp873	2E-01	0.2
Aamdc	3E-03	0.2	mt-Cytb	8E-26	0.2
Elmod3	9E-01	0.2	2610037D02Rik	7E-01	0.2
mt-Cytb	2E-32	0.2	Rnf145	9E-01	0.2
Rab5b	6E-02	0.2	Laptm4a	2E-13	0.2
Wdr35	6E-02	0.2	Gm10941	9E-01	0.2
Accs	9E-01	0.2	Fuca2	1E-01	0.2
Spred2	5E-01	0.2	Rcan2	1E-09	0.2
1300002E11Rik	4E-01	0.2	Ddx59	8E-01	0.2
Cd81	6E-14	0.2	Gpd2	1E+00	0.2
Grik5	7E-01	0.2	Ifitm2	2E-14	0.2
Gpr39	4E-02	0.2	Zcchc14	6E-01	0.2
Gm11084	8E-01	0.2	Zfp35	5E-01	0.2
Rprd2	6E-03	0.2	Rbms1	2E-07	0.2
Ubc	1E-13	0.2	Pdpr	6E-01	0.2
Vps13d	2E-01	0.2	Lsm10	3E-01	0.2
Ano8	7E-01	0.2	Atp6ap1	9E-03	0.2
Mindy2	1E-01	0.2	Tradd	1E+00	0.2
AW146154	7E-01	0.2	Thap7	4E-01	0.2
Fhod1	5E-01	0.2	Zfp958	4E-01	0.2
Dtwd2	8E-01	0.2	Blcap	5E-01	0.2
2700097O09Rik	5E-01	0.2	Itga5	8E-01	0.2
Clip3	1E-01	0.2	Ube2e2	5E-05	0.2
Mageh1	2E-01	0.2	3300002I08Rik	9E-01	0.2
Ptpre	6E-01	0.2	Pcsk5	3E-01	0.2
Tgs1	1E-01	0.2	6230400D17Rik	9E-01	0.2
Cars2	9E-01	0.2	Tbc1d22a	7E-01	0.2
Adgre5	6E-01	0.2	Kxd1	5E-03	0.2
Mbip	3E-01	0.2	Tmem123	6E-01	0.2
Leng8	9E-01	0.2	Rab10os	9E-01	0.2
Tada2a	5E-01	0.2	Cavin3	4E-07	0.2
Dclk1	4E-05	0.2	Alpk1	1E+00	0.2
Slc26a11	4E-01	0.2	Wdr27	3E-01	0.2
Gm17484	5E-01	0.2	Klhl18	3E-01	0.2
AW549877	1E-01	0.2	Ubxn2b	9E-01	0.2
Asap3	9E-01	0.2	Tmem185b	3E-01	0.2

Limb Mesenchyme					
Control			NICD2		
Gene	p	FC	Gene	p	FC
4931428F04Rik	7E-04	0.2	Smurf2	9E-09	0.2
Rwdd2b	9E-03	0.2	Arnt2	2E-04	0.2
Plxdc2	1E-12	0.2	Itprid2	9E-05	0.2
Stt3a	6E-10	0.2	Ddb2	2E-02	0.2
Cdc37l1	2E-09	0.2	Safb2	7E-06	0.2
Lztfl1	2E-10	0.2	Tubg2	2E-04	0.2
Apoo	7E-06	0.2	Kat2a	3E-05	0.2
P3h3	2E-11	0.2	Sap18b	2E-04	0.2
Zbtb5	7E-04	0.2	Ciz1	3E-05	0.2
Carm1	3E-05	0.2	Ppp1r9a	3E-05	0.2
Hoxc9	2E-06	0.2	Srp72	6E-12	0.2
Glis2	7E-07	0.2	Large1	5E-04	0.2
Ppargc1a	3E-07	0.2	Cacna1b	6E-02	0.2
Taok1	3E-11	0.2	Araf	1E-06	0.2
Prickle3	1E-03	0.2	Usp34	4E-13	0.2
Fam192a	3E-07	0.2	Psmd12	7E-11	0.2
Arfgap3	4E-11	0.2	Cbx8	7E-04	0.2
Cdk11b	1E-11	0.2	Aak1	7E-08	0.2
Prmt7	5E-08	0.2	Casd1	2E-06	0.2
Serf1	1E-14	0.2	Hnrnpu	4E-21	0.2
Nme6	1E-05	0.2	Parva	3E-09	0.2
Wdr78	4E-03	0.2	Morn4	4E-04	0.2
Hmgb3	4E-05	0.2	Map2k4	6E-07	0.2
Inhba	3E-07	0.2	Eif5	3E-16	0.2
Sec22b	1E-13	0.2	Pard6g	4E-05	0.2
Mprip	7E-13	0.2	Bicd1	3E-03	0.2
Nup98	2E-07	0.2	Usp21	2E-03	0.2
Mphosph10	3E-07	0.2	Kri1	3E-04	0.2
Fbxl12	3E-07	0.2	Fbxl12os	4E-03	0.2
2700062C07Rik	1E-05	0.2	Zfyve27	1E-03	0.2
Armc9	1E-06	0.2	Sec61b	4E-26	0.2
Thtpa	4E-03	0.2	Asb13	2E-03	0.2
Tm9sf3	3E-18	0.2	Shb	2E-05	0.2
Mpi	7E-03	0.2	Pafah1b2	1E-10	0.2
Lysmd3	8E-07	0.2	Arf4	5E-28	0.2
Trim39	7E-04	0.2	Ubr4	2E-07	0.2
Senp3	8E-07	0.2	Timm8a1	4E-06	0.2
Surf6	1E-07	0.2	Ctif	2E-05	0.2
Actb	4E-16	0.2	B130055M24Rik	8E-03	0.2
Sar1a	3E-15	0.2	Aftph	4E-05	0.2
Cul3	1E-14	0.2	Gtf3c4	7E-05	0.2
Maml1	6E-07	0.2	Camk2d	6E-14	0.2
6030458C11Rik	3E-06	0.2	Stim2	2E-04	0.2
Timm23	9E-16	0.2	Ank2	9E-08	0.2
Ywhaz	1E-21	0.2	Mlec	3E-13	0.2
Rpn1	1E-14	0.2	Gng11	3E-08	0.2
Nek1	1E-07	0.2	Ddx54	3E-07	0.2
Tatdn1	2E-05	0.2	Eif4g1	3E-13	0.2
AU040320	9E-07	0.2	Mfap1b	9E-04	0.2
Noa1	2E-05	0.2	Ahi1	1E-06	0.2
Pdzd8	7E-06	0.2	Ddx5	2E-23	0.2
Fubp1	6E-13	0.2	Zfand6	6E-10	0.2
Pgrmc1	2E-12	0.2	Fkbp9	2E-08	0.2
Zfp746	3E-05	0.2	Rnf4	4E-05	0.2
Mras	2E-09	0.2	Mto1	3E-05	0.2
Dstn	3E-15	0.2	Crls1	1E-05	0.2
Dnajc7	1E-08	0.2	Zfp583	5E-04	0.2
Tia1	2E-07	0.2	Robo1	2E-15	0.2
Pdgfa	5E-13	0.2	Copb2	6E-18	0.2
Mb21d2	4E-03	0.2	Gtf2a1	1E-06	0.2
Wls	2E-22	0.2	Kdelr3	1E-08	0.2
Card19	7E-10	0.2	Cdk5rap1	2E-04	0.2
Rnf38	1E-05	0.2	Togaram1	2E-05	0.2
Hoxc8	3E-08	0.2	Ago1	2E-06	0.2
Igf2r	8E-10	0.2	Tfb2m	2E-05	0.2
Mrpl20	2E-19	0.2	Tbc1d25	9E-02	0.2
Bbx	6E-11	0.2	Myh10	9E-06	0.2
Cyp2d22	4E-03	0.2	Mob1b	6E-06	0.2
Selenoo	6E-04	0.2	Dstn	1E-12	0.2
Ankrd13a	6E-07	0.2	Gtf2e1	2E-04	0.2
Kmt2d	3E-06	0.2	Utrn	8E-12	0.2
Gm31323	3E-02	0.2	Glrx5	2E-12	0.2
Rassf2	3E-04	0.2	Mtr	3E-06	0.2

Chondrogenic					
Control			NICD2		
Gene	p	FC	Gene	p	FC
A530072M11Rik	8E-01	0.2	Xrcc5	9E-06	0.2
Ltv1	2E-02	0.2	AW146154	4E-02	0.2
Lypla1	2E-03	0.2	Zbtb12	3E-01	0.2
Cpeb3	9E-03	0.2	Pibf1	2E-03	0.2
Pcdhga10	7E-01	0.2	Clpx	1E-03	0.2
Pde4a	4E-01	0.2	Tmco3	2E-03	0.2
Ggps1	3E-05	0.2	Ndufa1	1E-13	0.2
A030001D20Rik	8E-01	0.2	Ralgapa1	3E-07	0.2
Shc1	2E-05	0.2	Habp4	2E-02	0.2
Ngrn	4E-04	0.2	Tifa	4E-01	0.2
Gabarapl1	2E-07	0.2	Xpo5	6E-05	0.2
Slc18b1	9E-03	0.2	Utp25	3E-01	0.2
Zfp933	1E-01	0.2	Dolk	6E-01	0.2
Fahd2a	3E-02	0.2	Tjap1	3E-02	0.2
Dhx30	2E-04	0.2	Ehd4	1E-01	0.2
Usf2	4E-05	0.2	Tcaim	1E-01	0.2
Zc3h4	2E-02	0.2	Paip2	1E-20	0.2
2310033P09Rik	2E-03	0.2	Snx14	5E-03	0.2
Slc35e2	5E-02	0.2	Tmem216	6E-02	0.2
R3hdm1	6E-09	0.2	Ttll4	2E-01	0.2
Zfp68	2E-03	0.2	Fam117b	6E-02	0.2
Vwa8	5E-04	0.2	Jkamp	6E-06	0.2
Mindy3	1E-02	0.2	Ttll1	7E-01	0.2
Man2b2	3E-02	0.2	Sh3d19	1E-05	0.2
Cln3	2E-01	0.2	2810013P06Rik	1E-01	0.2
Cmtr1	2E-02	0.2	Rfxank	9E-01	0.2
Ascc1	1E-03	0.2	Churc1	4E-08	0.2
Sar1b	7E-13	0.2	Tab3	6E-01	0.2
Zfp398	8E-02	0.2	Nrde2	4E-01	0.2
Mdn1	1E-02	0.2	Cfap36	4E-09	0.2
St7	1E-03	0.2	Runx1	5E-05	0.2
Usp46	5E-04	0.2	Nob1	1E-01	0.2
Zfp280d	1E-06	0.2	Crlf2	6E-03	0.2
Esco1	1E-03	0.2	4833420G17Rik	6E-04	0.2
Shb	3E-01	0.2	Cdadc1	4E-02	0.2
Polr2d	7E-05	0.2	Zfp740	2E-02	0.2
Mrpl41	1E-06	0.2	Thumpd1	4E-03	0.2
AW209491	1E-01	0.2	Fastkd1	9E-01	0.2
Lrp10	1E-04	0.2	Ubn2	2E-08	0.2
Gnaq	5E-09	0.2	Cab39l	2E-05	0.2
Rubcn	1E-02	0.2	Rtl3	3E-05	0.2
Cptp	6E-01	0.2	Rpap2	1E-02	0.2
Fam98a	1E-04	0.2	Nkiras1	1E-01	0.2
Ccdc66	1E-02	0.2	Tfg	3E-07	0.2
Wdfy2	1E-02	0.2	Erich1	4E-02	0.2
Pnpla6	1E-01	0.2	Lmbrd1	3E-04	0.2
Polg2	2E-01	0.2	Unc45a	2E-01	0.2
Abi3bp	2E-15	0.2	2010320M18Rik	1E-02	0.2
Rab2b	4E-03	0.2	Trp53rka	3E-01	0.2
Taz	3E-03	0.2	Hexdc	5E-01	0.2
Ttc33	1E-07	0.2	Dock4	2E-01	0.2
Gorasp1	3E-01	0.2	Clasp2	1E-04	0.2
Tmem161b	8E-02	0.2	Bcl9	2E-03	0.2
Mettl17	2E-01	0.2	0610009E02Rik	3E-01	0.2
Smurf2	5E-09	0.2	Gpat4	6E-02	0.2
Smim10l1	3E-14	0.2	Pkp4	3E-01	0.2
Mrc2	5E-06	0.2	Pip4p2	8E-05	0.2
Nmnat3	3E-01	0.2	Dnajc7	4E-07	0.2
Faf1	5E-09	0.2	Rnf114	1E-01	0.2
Cmpk1	4E-13	0.2	Sgms1	5E-04	0.2
Abcd3	6E-06	0.2	Cep19	4E-01	0.2
Usp39	1E-02	0.2	Eci1	2E-05	0.2
Lmbr1l	3E-01	0.2	Tyk2	3E-01	0.2
Gm47167	2E-01	0.2	2310033P09Rik	2E-01	0.2
Pdia6	1E-28	0.2	Ddr1	5E-02	0.2
G6pc3	6E-03	0.2	Plscr1	5E-01	0.2
Ppie	1E-03	0.2	Jmjd1c	9E-06	0.2
R3hdm2	2E-11	0.2	Kctd3	8E-01	0.2
Myo18a	3E-02	0.2	Cldn34c1	9E-01	0.2
Sdhaf1	1E-03	0.2	Dgcr6	2E-06	0.2
Sirt3	4E-02	0.2	Thbs3	2E-01	0.2
Arntl	3E-01	0.2	Dhdh	9E-01	0.2
Zfp362	3E-01	0.2	Pik3ip1	1E-03	0.2

Fibroblast					
Control			NICD2		
Gene	p	FC	Gene	p	FC
Zmat3	5E-06	0.1	Irf7	1E-04	0.1
Map2k1	8E-08	0.1	Tnrc6c	5E-10	0.1
Btbd2	4E-04	0.1	Rpl9-ps6	2E-05	0.1
Sh3gl1	4E-10	0.1	Bfar	7E-05	0.1
Isca1	6E-04	0.1	Cul5	3E-09	0.1
Cyb561d2	9E-07	0.1	Tbc1d9b	5E-04	0.1
Trappc1	8E-11	0.1	Sin3b	1E-12	0.1
Meis2	4E-11	0.1	Zfp281	1E-01	0.1
As3mt	1E-01	0.1	Arpc1a	5E-12	0.1
Copa	2E-20	0.1	Zdhhc4	7E-03	0.1
Usp40	3E-06	0.1	Piezo2	5E-05	0.1
Rpl19	4E-115	0.1	Slc39a13	5E-08	0.1
Gm12743	9E-03	0.1	Vps9d1	2E-01	0.1
1810055G02Rik	2E-05	0.1	Ppa2	9E-08	0.1
Pard3	1E-10	0.1	Pla2g15	9E-03	0.1
Igf2bp3	3E-09	0.1	Ifih1	8E-03	0.1
Dcp2	5E-04	0.1	Pmvk	1E-03	0.1
Btbd10	7E-05	0.1	Castor1	1E-02	0.1
Prepl	3E-02	0.1	Idnk	1E-05	0.1
Upp2	1E-04	0.1	Lactb	1E-03	0.1
Ric1	5E-05	0.1	AW549877	1E-04	0.1
Zfat	1E-01	0.1	Dnajc1	9E-10	0.1
Plin3	1E-11	0.1	Ubqln2	9E-03	0.1
Nsa2	9E-40	0.1	Bag2	7E-02	0.1
Plscr3	3E-09	0.1	Secisbp2l	4E-05	0.1
Pofut1	2E-04	0.1	Lrig3	6E-06	0.1
Tbc1d5	6E-10	0.1	Tuba4a	5E-02	0.1
Atg4c	6E-03	0.1	Rcn3	1E-19	0.1
Mapk9	3E-05	0.1	Arpc3	2E-18	0.1
Pja1	2E-06	0.1	Xpa	2E-06	0.1
Abracl	7E-17	0.1	Tmem9	2E-03	0.1
Krtcap2	3E-42	0.1	Dynll1	9E-26	0.1
Dffa	2E-01	0.1	Plekhn1	8E-02	0.1
Vps26b	4E-04	0.1	Plpp5	7E-04	0.1
Gls	1E-13	0.1	Mvk	3E-03	0.1
Birc3	9E-03	0.1	Hsd3b7	5E-02	0.1
Pycard	2E-13	0.1	Igfbp5	4E-07	0.1
Rpl7a	2E-68	0.1	Tbc1d5	7E-06	0.1
Scyl1	6E-06	0.1	Fam129a	4E-09	0.1
Zfp281	4E-02	0.1	Dlat	5E-08	0.1
Galk1	9E-12	0.1	Blzf1	5E-03	0.1
Zfp595	2E-01	0.1	Snx19	6E-03	0.1
Glb1l	8E-04	0.1	Rchy1	2E-04	0.1
Hacd2	1E-04	0.1	Napb	9E-01	0.1
Rnf41	1E-05	0.1	Obsl1	7E-02	0.1
Fkbp1a	1E-30	0.1	Trim34a	3E-02	0.1
Mfsd1	5E-06	0.1	Atxn7l3	3E-03	0.1
Rps9	9E-94	0.1	Zfp512b	7E-02	0.1
F420014N23Rik	6E-03	0.1	Npr3	3E-05	0.1
Hsp90b1	3E-24	0.1	Aacs	9E-04	0.1
Rplp1	1E-100	0.1	Asb1	5E-01	0.1
Itpkb	3E-04	0.1	Yipf3	4E-08	0.1
Ttc37	1E-06	0.1	Fech	9E-05	0.1
Isg15	3E-22	0.1	Rps3a1	1E-55	0.1
Mapk14	1E-05	0.1	Galk1	7E-08	0.1
Clk3	1E-03	0.1	Atg9a	6E-01	0.1
Zfp467	4E-04	0.1	Nrip2	2E-01	0.1
Mt1	7E-38	0.1	Mtfmt	1E-01	0.1
Arhgap44	2E-02	0.1	Rps27a	3E-54	0.1
Cavin2	1E-31	0.1	D630003M21Rik	8E-02	0.1
Smarca2	3E-14	0.1	Hoxc8	7E-01	0.1
Zfp597	5E-02	0.1	Phf21a	1E-06	0.1
Apobec1	3E-14	0.1	Vat1	3E-10	0.1
Hoxc8	5E-01	0.1	Klhl15	4E-01	0.1
Fnbp1	4E-07	0.1	Tfe3	2E-03	0.1
Fam122a	5E-07	0.1	Epb41l4aos	3E-04	0.1
Ifnar2	1E-10	0.1	E2f4	1E-02	0.1
Ctsl	1E-47	0.1	Socs3	4E-02	0.1
Eif3k	1E-37	0.1	Sec24d	3E-07	0.1
Tnpo2	1E-02	0.1	Lztr1	8E-03	0.1
Tor2a	5E-04	0.1	Rplp2	1E-55	0.1
1700017B05Rik	3E-01	0.1	Casp12	6E-08	0.1
Ankzf1	1E-01	0.1	Kctd13	2E-01	0.1

Fibroblast?					
Control			NICD2		
Gene	p	FC	Gene	p	FC
Gan	3E-15	0.5	Twist2	1E-24	0.5
Dmd	2E-16	0.5	B930036N10Rik	2E-31	0.5
Gm44649	2E-10	0.5	Hrct1	6E-15	0.5
Myo5a	4E-22	0.5	Lmna	2E-74	0.5
Cpne2	2E-14	0.5	Timp1	1E-41	0.4
Exosc7	3E-21	0.5	Mllt1	2E-24	0.4
Zdhhc3	2E-23	0.5	Il1rl1	2E-29	0.4
D130020L05Rik	2E-13	0.5	Sumo3	5E-34	0.4
Snupn	7E-06	0.5	Uck1	4E-18	0.4
Srrm1	7E-54	0.5	Osgepl1	2E-15	0.4
C330007P06Rik	9E-17	0.5	Eif5a	2E-54	0.4
Ppp1r14a	1E-04	0.5	Nox1	2E-11	0.4
Zscan18	5E-02	0.5	Sf3a2	4E-18	0.4
Ints5	2E-14	0.5	Cenpv	4E-15	0.4
Prpf31	7E-22	0.5	Lin7a	5E-17	0.4
Ints10	6E-18	0.5	Greb1l	2E-17	0.4
Trir	4E-48	0.5	2500002B13Rik	4E-09	0.4
Ccdc112	1E-19	0.5	Sept7	2E-72	0.4
Cnot1	7E-18	0.5	Wdr5	2E-19	0.4
Noc4l	1E-16	0.5	Rapgef4	2E-16	0.4
Eif2b3	4E-04	0.5	Slc7a1	5E-28	0.4
Flrt1	6E-15	0.5	Ptrh2	6E-25	0.4
Psmg2	8E-23	0.5	Radil	1E-14	0.4
Cdk19	3E-20	0.5	Fam107b	1E-21	0.4
Fndc4	7E-15	0.5	Calm1	6E-109	0.4
Pin1	2E-28	0.5	Scaf11	2E-51	0.4
Lrrc8d	8E-14	0.5	Zfp385a	6E-17	0.4
Ube2n	2E-31	0.5	Ranbp3	3E-18	0.4
Gm41764	1E-07	0.5	Syncrip	5E-43	0.4
Dhx32	4E-18	0.5	Eloc	2E-54	0.4
Gfm1	6E-13	0.5	Lims1	8E-50	0.4
Arhgef2	6E-18	0.5	Ubiad1	1E-09	0.4
Nono	8E-32	0.4	Gle1	5E-29	0.4
Stard3nl	2E-29	0.4	Clasp2	9E-25	0.4
Nosip	3E-12	0.4	Utp3	2E-40	0.4
Resf1	7E-12	0.4	Tfpi2	3E-11	0.4
Mfsd12	5E-14	0.4	Thoc7	3E-65	0.4
Anapc7	8E-18	0.4	Dnajc2	2E-26	0.4
Pfkp	3E-17	0.4	Basp1	9E-24	0.4
Dhrs4	9E-24	0.4	Sp3	2E-30	0.4
Ctdspl	3E-28	0.4	Limk2	2E-20	0.4
Pde6d	9E-23	0.4	Cdr2	2E-14	0.4
Trak2	2E-14	0.4	Nup88	2E-20	0.4
Ptms	6E-45	0.4	D10Wsu102e	6E-15	0.4
Pfn1	2E-72	0.4	Cdkn2a	2E-42	0.4
Haus2	1E-18	0.4	Fgd1	6E-27	0.4
Acp6	4E-08	0.4	Mtpn	8E-51	0.4
Plcb4	2E-22	0.4	Fam20b	4E-24	0.4
Sorbs3	2E-11	0.4	Gm19705	3E-09	0.4
Ooep	2E-07	0.4	Nab1	1E-25	0.4
Lgals1	3E-90	0.4	Tm9sf1	6E-23	0.4
Ccn4	1E-06	0.4	Ctnnbip1	1E-31	0.4
Polg	1E-16	0.4	Rbmx	4E-29	0.4
Tti1	1E-18	0.4	Ncald	2E-15	0.4
Tfb1m	3E-14	0.4	Mepce	4E-22	0.4
Kdm2b	8E-18	0.4	Polr2d	2E-19	0.4
Atpaf2	1E-16	0.4	Galnt17	1E-10	0.4
Fam118a	4E-16	0.4	Kdm8	6E-13	0.4
Rbm10	3E-19	0.4	Slk	2E-25	0.4
Ubac1	1E-21	0.4	Serpinb1a	5E-37	0.4
Sh2d4a	6E-14	0.4	Twsg1	1E-25	0.4
Mif4gd	4E-12	0.4	Pds5a	7E-30	0.4
Grem2	2E-07	0.4	Cdc42ep1	1E-21	0.4
Creld2	2E-20	0.4	Ergic1	5E-24	0.4
Osbpl10	2E-14	0.4	Phtf2	3E-20	0.4
Dynll2	7E-34	0.4	Spata13	9E-15	0.4
Brd8	6E-22	0.4	Slc35a4	6E-22	0.4
Slk	4E-19	0.4	Mfsd6	1E-10	0.4
Crabp2	1E-22	0.4	Ada	3E-26	0.4
Ftsj3	6E-15	0.4	Wdr1	2E-24	0.4
Mrpl13	6E-31	0.4	Chpf	4E-25	0.4
E030030I06Rik	5E-16	0.4	Ipo7	4E-21	0.4
			Pdzd2	1E-22	0.4

Undefined					
Control			NICD2		
Gene	p	FC	Gene	p	FC

Articular/Synovial Fibroblast					
Control			NICD2		
Gene	p	FC	Gene	p	FC
Entpd5	3E-01	0.2	Tmsb15b2	3E-01	0.2
Zfp524	7E-01	0.2	1700021F05Rik	5E-01	0.2
Lrsam1	9E-01	0.2	Coq8b	1E+00	0.2
Gm4924	8E-01	0.2	Cnpy2	3E-14	0.2
Cers4	4E-01	0.2	Mtmr14	4E-01	0.2
Gxylt2	2E-02	0.2	Snw1	3E-04	0.2
Etnk1	2E-03	0.2	Marf1	8E-02	0.2
Dnase1l1	7E-01	0.2	Pdha1	8E-03	0.2
Fam111a	2E-06	0.2	1190007I07Rik	9E-01	0.2
Rb1cc1	1E-03	0.2	Gm48678	9E-01	0.2
Zfp157	6E-01	0.2	Pqlc1	6E-01	0.2
Prorp	4E-01	0.2	Pitpnc1	9E-01	0.2
Nim1k	5E-01	0.2	Fbxl3	3E-01	0.2
Arnt	4E-02	0.2	Lyplal1	3E-01	0.2
Klhl7	9E-01	0.2	Smim1	3E-04	0.2
Gm36975	3E-01	0.2	Uba52	1E-09	0.2
Ankrd42	4E-01	0.2	Cpne3	4E-02	0.2
Cox15	1E-01	0.2	Mthfsd	8E-01	0.2
Ube4a	8E-02	0.2	Prdm16	9E-01	0.2
D730003I15Rik	5E-01	0.2	Atp6v0c	2E-07	0.2
Trim25	9E-01	0.2	Nop53	1E-01	0.2
Fam214b	6E-01	0.2	Eml3	8E-01	0.2
Mllt3	3E-01	0.2	Anxa8	8E-01	0.2
Rbbp9	4E-01	0.2	Supv3l1	5E-01	0.2
Cpxm1	5E-02	0.2	Ago3	8E-02	0.2
Zadh2	3E-01	0.2	Axl	2E-01	0.2
Nudt14	7E-01	0.2	Lamp1	1E-10	0.2
Cox6b2	1E-01	0.2	Ctsl	3E-11	0.2
Tmem201	8E-01	0.2	Csf2ra	4E-01	0.2
5031439G07Rik	3E-01	0.2	Tlnrd1	4E-01	0.2
Rpgrip1l	9E-01	0.2	Snrk	5E-01	0.2
Ip6k1	5E-02	0.2	Pdcd2l	8E-01	0.2
Aar2	7E-01	0.2	Taf4	3E-01	0.2
Cutc	8E-01	0.2	Vps39	4E-01	0.2
Fuz	8E-01	0.2	Six5	2E-01	0.2
Acyp2	1E-03	0.2	Tnip2	5E-01	0.2
Parp3	1E-04	0.2	Stx8	2E-02	0.2
Ppp1cc	4E-04	0.2	Parn	6E-01	0.2
Kdm7a	6E-01	0.2	Cxcl1	7E-01	0.2
Tmem41b	2E-01	0.2	Pcolce	8E-07	0.2
Uckl1	9E-01	0.2	Atxn1l	2E-01	0.2
Fbxo36	3E-01	0.2	Akap8l	1E-01	0.2
Klf8	6E-01	0.2	Pdlim2	4E-04	0.2
Atox1	2E-11	0.2	Tesk1	5E-01	0.2
Rfxank	3E-01	0.2	Sun1	9E-01	0.2
Ccng2	5E-01	0.2	BC002059	4E-01	0.2
Poglut3	3E-02	0.2	Hspb8	5E-01	0.2
Gm38604	4E-01	0.2	Cln5	2E-01	0.2
Cast	9E-07	0.2	Atp6v0a1	2E-02	0.2
Yes1	8E-01	0.2	Iqcc	8E-01	0.2
Alpk1	4E-01	0.2	Rnf40	7E-01	0.2
Crtc3	3E-02	0.2	Fam117b	5E-01	0.2
Brf2	9E-01	0.2	Nr1d1	6E-01	0.2
Wdr20	8E-01	0.2	Pxmp4	9E-01	0.2
Noc4l	3E-01	0.2	Ammecr1l	5E-01	0.2
Cradd	9E-01	0.2	4833420G17Rik	3E-01	0.2
Syne3	4E-01	0.2	Zfp655	8E-01	0.2
Rnf213	9E-02	0.2	Stx3	1E+00	0.2
Slc35e2	4E-01	0.2	9930104L06Rik	3E-01	0.2
Slain2	1E-02	0.2	Dip2a	9E-01	0.2
Zfp51	6E-01	0.2	Setd4	4E-01	0.2
Slc23a2	3E-01	0.2	Abhd2	9E-01	0.2
Nmral1	7E-01	0.2	Zfand5	5E-03	0.2
Csad	2E-03	0.2	Add1	3E-04	0.2
Gm17251	8E-01	0.2	Tmem141	4E-01	0.2
Zfp286	9E-01	0.2	Gm49179	8E-01	0.2
Ccdc68	8E-01	0.2	Rom1	8E-01	0.2
Cab39l	1E-03	0.2	Tmem222	1E+00	0.2
Arhgap20	7E-06	0.2	Evc2	7E-01	0.2
Ppp6r2	5E-01	0.2	Maged2	1E-01	0.2
Tmem63b	3E-01	0.2	Clybl	3E-01	0.2
Arpc1a	1E-05	0.2	Napa	2E-02	0.2
Dusp18	8E-01	0.2	Zfp2	3E-01	0.2

Limb Mesenchyme					
Control			NICD2		
Gene	*p*	FC	Gene	*p*	FC
Plagl2	9E-04	0.2	Ctbp2	2E-04	0.2
3010001F23Rik	8E-05	0.2	Smim13	8E-06	0.2
E130308A19Rik	2E-04	0.2	Tbcd	3E-05	0.2
Zfp362	3E-04	0.2	Zfp113	2E-05	0.2
Smarcd1	2E-06	0.2	Snx13	6E-05	0.2
Ccdc186	9E-10	0.2	Ercc4	8E-04	0.2
Zkscan7	1E-02	0.2	Cog5	2E-07	0.2
Atg16l2	8E-06	0.2	Pdgfa	5E-09	0.2
Focad	4E-05	0.2	Ints2	1E-03	0.2
Strn3	6E-18	0.2	Gm26887	4E-05	0.2
Alkbh6	1E-05	0.2	Arhgap23	2E-06	0.2
Rsph3b	1E-04	0.2	Rap2c	5E-06	0.2
Fbxo30	2E-05	0.2	Cbx3	2E-17	0.2
Nyap1	3E-02	0.2	Usp6nl	8E-05	0.2
St3gal6	6E-14	0.2	Csnk1d	2E-05	0.2
Fkbp14	6E-11	0.2	Vipas39	2E-05	0.2
Gm26782	6E-07	0.2	3110021N24Rik	2E-04	0.2
Pum3	1E-08	0.2	Ube2h	2E-11	0.2
Pafah1b2	1E-10	0.2	Ppp3r1	8E-07	0.2
Baiap2l1	2E-08	0.2	Clk2	5E-04	0.2
Pbx3	1E-09	0.2	Tmco3	6E-06	0.2
Hspa4	2E-15	0.2	Nthl1	1E-05	0.2
Usp50	1E-07	0.2	Mfge8	8E-11	0.2
Prdm2	7E-09	0.2	Oga	1E-04	0.2
Tmtc4	7E-09	0.2	Pan2	7E-03	0.2
Naa15	1E-08	0.2	Zfp148	1E-09	0.2
Cul2	2E-07	0.2	Klf3	9E-10	0.2
Gng11	2E-05	0.2	Hnrnpa0	3E-19	0.2
Kmt5b	8E-07	0.2	1810055G02Rik	2E-05	0.2
Qser1	1E-06	0.2	Lrch3	3E-07	0.2
Gspt1	4E-18	0.2	2310011J03Rik	6E-05	0.2
Rnaset2b	1E-06	0.2	Lsm14b	4E-05	0.2
Tspan2	6E-06	0.2	Ogfod2	3E-05	0.2
Tle4	1E-03	0.2	Prrx2	1E-11	0.2
Zfp865	3E-05	0.2	Tsen54	3E-03	0.2
Arf3	9E-07	0.2	Dip2b	3E-06	0.2
Gtf3c3	7E-06	0.2	Arv1	7E-03	0.2
St7l	2E-06	0.2	Rnf146	2E-07	0.2
Zc3h18	3E-09	0.2	Brd4	7E-09	0.2
Atp6v0e2	8E-03	0.2	Cpsf6	2E-07	0.2
Trappc2	9E-06	0.2	Frs2	4E-07	0.2
Ufsp1	8E-04	0.2	Usp9x	2E-11	0.2
Kdm5c	9E-06	0.2	Sema5a	2E-07	0.2
Farsb	1E-07	0.2	1110002L01Rik	2E-05	0.2
Brms1l	3E-06	0.2	Ublcp1	2E-06	0.2
4933424G05Rik	5E-02	0.2	Ubox5	2E-03	0.2
Ganab	1E-07	0.2	Dnhd1	7E-03	0.2
Cep104	5E-05	0.2	Foxred1	1E-03	0.2
Rap2c	3E-06	0.2	Smn1	1E-05	0.2
Eif4enif1	3E-06	0.2	Smad4	1E-07	0.2
Copb1	5E-17	0.2	Atp11b	6E-05	0.2
Lancl1	8E-07	0.2	Dars	2E-09	0.2
Gpbp1	8E-12	0.2	Cfap74	1E-03	0.2
Cct3	1E-15	0.2	Tstd2	4E-05	0.2
A730017L22Rik	2E-02	0.2	Ppp1r13b	1E-05	0.2
Ddx50	1E-16	0.2	1600002K03Rik	6E-06	0.2
Kif16b	3E-07	0.2	Ubiad1	2E-03	0.2
Rtkn	2E-06	0.2	Sfmbt2	2E-02	0.2
Metap2	9E-21	0.2	Magt1	4E-07	0.2
Hnrnpa0	1E-20	0.2	Serpinb9	2E-03	0.2
Aak1	5E-09	0.2	Atxn7l3	4E-06	0.2
Asph	4E-09	0.2	Dcaf11	2E-04	0.2
Naa30	9E-05	0.2	Gm41556	4E-03	0.2
Slc35a3	6E-05	0.2	Atrn	4E-06	0.2
Golga2	2E-07	0.2	Tm9sf4	2E-10	0.2
2610037D02Rik	7E-05	0.2	C330007P06Rik	1E-06	0.2
Rcc1	3E-04	0.2	Erc1	1E-07	0.2
Akap11	1E-08	0.2	Manf	1E-14	0.2
Dhodh	7E-03	0.2	Rtcb	9E-08	0.2
Gm26672	2E-06	0.2	Gm4117	7E-07	0.2
Ap1ar	2E-05	0.2	Rsph3a	6E-04	0.2
Traf3ip1	2E-06	0.2	Nup98	3E-06	0.2
Orc2	4E-07	0.2	Dnm1l	1E-06	0.2

Chondrogenic					
Control			NICD2		
Gene	*p*	FC	Gene	*p*	FC
Khdc4	5E-04	0.2	Pop5	6E-04	0.2
Cyth1	4E-02	0.2	Naxd	6E-06	0.2
Mccc2	3E-03	0.2	Rab3gap1	4E-05	0.2
Txndc12	2E-07	0.2	Rab4a	6E-01	0.2
Slc12a6	1E-02	0.2	Agfg1	8E-04	0.2
Ptpn1	7E-07	0.2	1810009A15Rik	9E-02	0.2
Hdhd3	4E-01	0.2	Nphp1	4E-02	0.2
Fbxl20	2E-03	0.2	Hlcs	1E-01	0.2
4931428F04Rik	3E-01	0.2	Ercc8	6E-01	0.2
Gm49267	4E-01	0.2	Dkc1	3E-03	0.2
Ubr3	3E-08	0.2	Adpgk	2E-01	0.2
0610040B10Rik	7E-02	0.2	Fbxo44	5E-01	0.2
Abhd13	4E-01	0.2	Clasp1	6E-04	0.2
Tomm7	6E-23	0.2	Rhod	1E-02	0.2
Nek1	2E-02	0.2	Zfp235	2E-01	0.2
Zfp579	4E-02	0.2	Vipas39	8E-02	0.2
Macf1	7E-12	0.2	Nop10	6E-14	0.2
Gbf1	2E-09	0.2	Gpank1	4E-01	0.2
Pdia3	1E-36	0.2	Tbcel	3E-02	0.2
8430429K09Rik	2E-01	0.2	Rcbtb1	4E-03	0.2
Castor1	1E+00	0.2	Rab11b	2E-08	0.2
Frs2	2E-04	0.2	Arhgef11	3E-02	0.2
1500011B03Rik	2E-04	0.2	Trim8	3E-01	0.2
Irs1	3E-04	0.2	Nphs1	4E-01	0.2
Pomk	7E-02	0.2	Wbp1	5E-03	0.2
Ercc6	1E-01	0.2	1500004A13Rik	3E-02	0.2
Phykpl	9E-03	0.2	Rbbp8	3E-02	0.2
1190007I07Rik	1E-01	0.2	Ppp3ca	2E-12	0.2
Snw1	6E-13	0.2	Mettl1	3E-05	0.2
Tsix	1E-01	0.2	Rdm1	2E-01	0.2
Pfkfb2	4E-01	0.2	Carf	4E-01	0.2
Mar8	4E-04	0.2	Tbc1d8b	1E-03	0.2
Fgf2	2E-03	0.2	Arl6ip5	2E-09	0.2
Ptpn2	2E-03	0.2	0610009B22Rik	2E-04	0.2
Sugp1	5E-02	0.2	Ak5	2E-01	0.2
Atl3	3E-09	0.2	Gale	9E-01	0.2
Armc9	3E-02	0.2	Sumf1	4E-01	0.2
Dis3l	3E-01	0.2	Syvn1	6E-01	0.2
Mphosph9	7E-01	0.2	Vldlr	2E-01	0.2
Plpp6	3E-01	0.2	Tent4b	3E-01	0.2
Adat2	6E-01	0.2	E130307A14Rik	8E-02	0.2
F8	2E-01	0.2	Saysd1	4E-05	0.2
Msrb2	2E-01	0.2	Zfp646	2E-02	0.2
Mettl5	1E-02	0.2	Emc1	2E-01	0.2
Tmem237	2E-04	0.2	Phf8	5E-01	0.2
Stxbp4	2E-02	0.2	Rbm15b	2E-01	0.2
Me1	7E-18	0.2	Zfp84	1E-01	0.2
Lmbrd2	1E-01	0.2	Fyco1	2E-02	0.2
Trub2	6E-02	0.2	Fkbp14	1E-05	0.2
Hp1bp3	7E-17	0.2	Ubxn2a	6E-03	0.2
Tmem223	2E-12	0.2	Fam160a2	1E-01	0.2
Sfxn2	6E-01	0.2	Polr3h	5E-02	0.2
A430033K04Rik	6E-01	0.2	Ifi44	9E-01	0.2
Rrp8	9E-04	0.2	Cog8	1E-02	0.2
Zfp266	9E-07	0.2	Zeb1	3E-05	0.2
Srsf10	4E-12	0.2	Trit1	2E-01	0.2
Dmac2l	1E-01	0.2	Klhl13	2E-02	0.2
Aifm1	8E-05	0.2	Tmem87b	3E-01	0.2
Noa1	1E-01	0.2	Crbn	6E-02	0.2
Dcaf8	1E-09	0.2	Srcap	1E-04	0.2
Gm30054	3E-01	0.2	Hps6	7E-01	0.2
Crbn	4E-03	0.2	Cox16	2E-12	0.2
Oxld1	2E-01	0.2	Cep44	1E-02	0.2
Mmgt1	5E-02	0.2	Gpd2	4E-01	0.2
Chid1	5E-04	0.2	Wdr70	8E-04	0.2
Ddx19b	2E-01	0.2	Irak2	5E-01	0.2
Tbc1d23	1E-04	0.2	Grcc10	7E-17	0.2
Fggy	9E-03	0.2	Samhd1	1E-02	0.2
Poglut2	4E-05	0.2	Atp23	1E-01	0.2
Id2	4E-04	0.2	Pdia3	6E-31	0.2
Tra2a	1E-07	0.2	Pcyox1	4E-03	0.2
Igsf10	8E-03	0.2	Ulk4	5E-01	0.2
Arid4b	4E-10	0.2	Ppm1l	2E-03	0.2

Fibroblast					
Control			NICD2		
Gene	*p*	FC	Gene	*p*	FC
Pramef8	1E-01	0.1	Extl3	2E-03	0.1
Castor2	3E-02	0.1	Fnbp1	3E-04	0.1
S100a11	2E-45	0.1	Htra2	7E-04	0.1
Snapc3	3E-05	0.1	Kdm5c	3E-04	0.1
Cdkl3	1E-05	0.1	Cybc1	1E-01	0.1
Aga	7E-17	0.1	Rab30	6E-03	0.1
Ncs1	2E-03	0.1	Rpl10-ps3	2E-03	0.1
Lsm10	2E-09	0.1	Lix1l	1E-07	0.1
Tradd	7E-05	0.1	Gstk1	1E-02	0.1
Nanp	2E-01	0.1	Pomgnt2	1E-01	0.1
Ecm1	4E-20	0.1	Ddx54	2E-03	0.1
Zfp758	2E-01	0.1	Zdhhc24	2E-01	0.1
Ankrd42	7E-02	0.1	Gabpa	1E-03	0.1
Ncdn	8E-02	0.1	H6pd	1E-01	0.1
Ophn1	3E-03	0.1	Rnft1	1E-04	0.1
H6pd	1E-02	0.1	Nktr	7E-08	0.1
Trim56	4E-02	0.1	Scn1b	4E-07	0.1
Usp45	3E-04	0.1	Acy1	1E-01	0.1
Zfp61	8E-02	0.1	Pdxk	7E-03	0.1
Pskh1	1E-01	0.1	Klf10	4E-02	0.1
Setd1b	4E-04	0.1	Serinc1	1E-10	0.1
Abhd16a	2E-05	0.1	Cpne1	7E-04	0.1
Pld2	5E-01	0.1	Atl1	2E-01	0.1
Cav1	2E-21	0.1	Notch1	2E-01	0.1
Pced1a	9E-03	0.1	Sptssa	7E-05	0.1
Wasf3	1E-03	0.1	Sec24a	2E-03	0.1
Gcc2	4E-07	0.1	Ints6	3E-02	0.1
Gm10130	3E-01	0.1	BC034090	8E-01	0.1
Metrnl	2E-02	0.1	Abhd2	7E-02	0.1
Gstm5	1E-19	0.1	Zfp870	2E-01	0.1
Zfp931	2E-03	0.1	Agrp	6E-01	0.1
Nisch	5E-21	0.1	Dmtf1	3E-04	0.1
Pou6f1	5E-02	0.1	Znfx1	6E-03	0.1
Zfp12	2E-01	0.1	Wdr60	2E-04	0.1
Ctns	3E-02	0.1	Trmt61a	2E-01	0.1
Aplf	2E-01	0.1	Epb41l2	1E-07	0.1
Kras	2E-12	0.1	Map2k1	2E-04	0.1
Zfp354c	3E-07	0.1	Ttl	6E-01	0.1
Cr1l	3E-07	0.1	Nsmf	3E-02	0.1
Washc2	2E-10	0.1	Fam199x	4E-01	0.1
Twf2	7E-05	0.1	Bri3bp	4E-04	0.1
Plekhn1	6E-02	0.1	Slc26a11	5E-01	0.1
Fam117b	3E-04	0.1	Fam120a	1E-06	0.1
Pi4kb	3E-05	0.1	Cdh13	8E-03	0.1
Gosr2	1E-05	0.1	Rps8	2E-50	0.1
Vmac	5E-04	0.1	Praf2	1E-08	0.1
Stxbp1	2E-02	0.1	Isg15	1E-10	0.1
Gnai3	1E-13	0.1	Snx12	1E-06	0.1
Scand1	3E-27	0.1	Tcf20	1E-04	0.1
Gpm6b	1E-14	0.1	Usp16	1E-06	0.1
Rai14	9E-05	0.1	Slc25a36	2E-02	0.1
Pus7	9E-04	0.1	Rai14	4E-03	0.1
Obsl1	8E-02	0.1	Atxn7l3b	2E-17	0.1
Dera	7E-03	0.1	Slc4a3	2E-01	0.1
Ccdc71l	1E-02	0.1	Tbc1d22b	3E-01	0.1
Ddb1	4E-14	0.1	Vps37d	3E-01	0.1
Rfx3	2E-02	0.1	Castor2	8E-02	0.1
Mtmr6	2E-05	0.1	Zfp758	5E-01	0.1
Nfkbiz	1E-02	0.1	Gpr107	8E-03	0.1
Gm45871	2E-01	0.1	Arfip2	6E-02	0.1
Mfsd5	2E-04	0.1	Atp11a	9E-03	0.1
9430038I01Rik	5E-03	0.1	Fubp3	3E-03	0.1
Tep1	3E-03	0.1	Nisch	4E-12	0.1
Extl3	1E-04	0.1	Amdhd2	2E-03	0.1
Apeh	2E-03	0.1	Ttc12	1E-01	0.1
Dhx57	8E-04	0.1	Abhd16a	1E-03	0.1
Tceal9	2E-26	0.1	Lsm10	9E-07	0.1
Nme4	8E-08	0.1	Gpatch2l	6E-02	0.1
Prelid2	2E-04	0.1	Smarca2	1E-08	0.1
Eif1a	7E-15	0.1	Fto	6E-05	0.1
Fes	8E-02	0.1	Tmsb15b1	9E-02	0.1
Sfxn4	5E-01	0.1	Gm15050	9E-02	0.1
Clcn6	2E-01	0.1	Npc1	5E-03	0.1

Fibroblast?					
Control			NICD2		
Gene	*p*	FC	Gene	*p*	FC
Dnajc2	5E-20	0.4	Arl6ip4	1E-34	0.4
Sgk3	1E-19	0.4	Glis2	3E-24	0.4
Htra2	1E-21	0.4	Carnmt1	1E-29	0.4
5430403G16Rik	1E-11	0.4	Zfp664	2E-24	0.4
Zc3hav1	5E-21	0.4	Cystm1	2E-04	0.4
Lhx9	1E-07	0.4	Obsl1	5E-25	0.4
Tm9sf1	1E-14	0.4	Chac2	4E-13	0.4
Cxcl14	1E-09	0.4	4930447N08Rik	1E-12	0.4
Ino80c	5E-15	0.4	Gm15886	3E-12	0.4
Psmc4	4E-21	0.4	Pcsk6	4E-28	0.4
Pank4	6E-10	0.4	Blvrb	8E-25	0.4
Lrrc45	2E-19	0.4	Slc25a28	6E-27	0.4
Bbof1	6E-08	0.4	Trim21	1E-05	0.4
Raly	1E-31	0.4	Evc	2E-22	0.4
Abcg2	2E-16	0.4	Lin37	2E-12	0.4
Wdr66	2E-10	0.4	Pakap	5E-34	0.4
Lrrc56	4E-06	0.4	Lpcat1	1E-24	0.4
Simc1	3E-16	0.4	C330007P06Rik	3E-24	0.4
Tubb2a	2E-23	0.4	5031439G07Rik	5E-11	0.4
Dhx15	2E-26	0.4	Psmd5	4E-19	0.4
Ldha	2E-44	0.4	Taf15	6E-47	0.4
Foxd2	2E-06	0.4	Anks6	5E-11	0.4
2310057M21Rik	8E-15	0.4	Nphp1	5E-20	0.4
Calhm2	9E-17	0.4	Akip1	3E-23	0.4
F2r	6E-14	0.4	Immt	1E-44	0.4
Ago2	5E-23	0.4	Clic4	2E-44	0.4
Pithd1	1E-17	0.4	Nsmce2	3E-40	0.4
Babam1	3E-09	0.4	Katnbl1	5E-26	0.4
Cdk4	1E-57	0.4	Dcaf1	1E-23	0.4
Nudcd1	5E-21	0.4	Cbr2	2E-19	0.4
Cars	2E-19	0.4	Zbtb12	8E-11	0.4
Ppp1ca	3E-53	0.4	Myo5a	1E-30	0.4
Cobll1	3E-28	0.4	Scarb1	7E-23	0.4
Zc3hav1l	4E-13	0.4	Gnl1	1E-26	0.4
Asb1	5E-05	0.4	Kbtbd2	1E-21	0.4
Prss35	8E-09	0.4	Bloc1s2	3E-18	0.4
Zbtb45	5E-15	0.4	Tspan7	9E-11	0.4
Mrpl42	9E-33	0.4	Tbc1d10a	8E-22	0.4
Gm44777	2E-07	0.4	Iqgap2	6E-23	0.4
Ccdc88a	4E-29	0.4	Car13	2E-22	0.4
Kat7	3E-19	0.4	Higd1a	5E-48	0.4
Gm39090	1E-10	0.4	Wdr53	3E-14	0.4
Nap1l4	3E-27	0.4	Akt1	6E-26	0.4
Pdzd8	2E-20	0.4	Mical3	3E-22	0.4
Tmem106c	1E-19	0.4	Nsun6	3E-24	0.4
S100a16	2E-15	0.4	Ccnd3	2E-27	0.4
Psmd14	1E-31	0.4	Acat2	2E-18	0.4
Hccs	5E-18	0.4	Gm49336	7E-22	0.4
Tsr2	7E-19	0.4	Foxk2	2E-22	0.4
Bahcc1	3E-16	0.4	Gab2	2E-32	0.4
H2afy2	8E-21	0.4	Mprip	4E-45	0.4
Srl	1E-12	0.4	Slc7a11	2E-16	0.4
Mtch1	3E-40	0.4	Ppp1ca	2E-68	0.4
Sgcd	6E-29	0.4	Mbd3	5E-26	0.4
Spg7	3E-20	0.4	Pot1b	5E-20	0.4
Ndufa5	2E-61	0.4	Nlrc5	8E-15	0.4
Tspan11	2E-08	0.4	Mei4	1E-14	0.4
Mtcl1	1E-11	0.4	Sptssa	3E-33	0.4
Aebp2	2E-20	0.4	Ints1	7E-17	0.4
Arid4a	5E-19	0.4	Slc25a30	2E-08	0.4
Cep295nl	4E-08	0.4	Lancl2	2E-20	0.4
Calm3	3E-29	0.4	Nono	3E-46	0.4
Carnmt1	4E-23	0.4	Klf2	3E-23	0.4
Twsg1	6E-21	0.4	Zfp54	5E-12	0.4
Spout1	2E-15	0.4	Isoc1	2E-23	0.4
Hspa12a	2E-09	0.4	Plcb4	5E-21	0.4
Tchp	5E-15	0.4	Gfod1	6E-17	0.4
Plk3	6E-10	0.4	Pcdh19	4E-15	0.4
Tepsin	2E-08	0.4	Sox12	1E-14	0.4
U2af1l4	4E-16	0.4	Zfp212	2E-17	0.4
Gcat	7E-13	0.4	Ppp1r3e	8E-10	0.4
Srsf10	3E-34	0.4	Rerg	2E-13	0.4
Slc38a1	1E-19	0.4	Setd1a	7E-25	0.4

Undefined					
Control			NICD2		
Gene	*p*	FC	Gene	*p*	FC

Articular/Synovial Fibroblast					
Control			NICD2		
Gene	*p*	FC	Gene	*p*	FC
Ccar2	3E-01	0.2	Ndst2	4E-01	0.2
Npepps	1E-04	0.2	Fam20c	2E-01	0.2
Aldh7a1	1E-02	0.2	Zbtb10	6E-01	0.2
Sema5a	9E-01	0.2	Trappc1	3E-02	0.2
Inpp5f	6E-01	0.2	Ccn5	1E-07	0.2
Gspt2	8E-01	0.2	F8	7E-01	0.2
Whamm	8E-01	0.2	Mpp2	4E-01	0.2
Cdc42bpg	3E-01	0.2	Camk1	2E-02	0.2
Cyp2j6	9E-01	0.2	Igf2bp3	2E-01	0.2
Ier3	3E-01	0.2	Dnttip1	4E-02	0.2
Pdk2	8E-01	0.2	Rbms2	5E-03	0.2
Gk5	7E-01	0.2	Ppm1l	9E-01	0.2
Vps28	5E-04	0.2	Herc4	4E-01	0.2
Atp6v0d1	3E-04	0.2	Hmgcl	8E-01	0.2
Mesd	1E-07	0.2	Psmb10	4E-01	0.2
Lrig1	7E-01	0.2	Sh3bgrl3	2E-08	0.2
Il17rc	7E-01	0.2	Sdcbp	2E-02	0.2
Nsun6	9E-01	0.2	Ntpcr	4E-01	0.2
Fbxl8	9E-01	0.2	Gsc	1E+00	0.2
Zkscan7	5E-01	0.2	mt-Nd3	1E-07	0.2
Tlnrd1	1E+00	0.2	4933434E20Rik	2E-01	0.2
S100a6	1E-12	0.2	Ift46	4E-01	0.2
Pabpc4l	4E-01	0.2	Prr12	7E-01	0.2
Myzap	6E-01	0.2	Nelfb	7E-01	0.2
Bbs5	7E-01	0.2	Mcm9	6E-01	0.2
Trp53bp2	1E+00	0.2	Mettl4	7E-01	0.2
Tram1l1	5E-01	0.2	Heca	1E+00	0.2
Zfp398	7E-01	0.2	Zfp236	5E-01	0.2
Castor2	6E-01	0.2	Atf2	1E-01	0.2
Slc25a44	5E-01	0.2	Wdpcp	7E-01	0.2
9330159M07Rik	9E-01	0.2	Junos	9E-01	0.2
Six1	4E-01	0.2	Immp2l	6E-01	0.2
Blvrb	2E-07	0.2	St6galnac6	8E-01	0.2
Cyld	1E-01	0.2	Ccpg1	9E-02	0.2
Gm15283	7E-02	0.2	Glb1	6E-01	0.2
Vps26c	6E-01	0.2	Gm11084	8E-01	0.2
Gm48678	5E-01	0.2	Cherp	7E-01	0.2
Fbxw11	2E-01	0.2	Ubr2	6E-02	0.2
Urod	3E-01	0.2	Coa6	9E-02	0.2
Cbx6	1E-01	0.2	Zfp62	8E-01	0.2
Nmi	4E-01	0.2	1700012D14Rik	8E-01	0.2
Nfat5	2E-05	0.2	Zcchc4	6E-01	0.2
Dnal1	9E-01	0.2	B9d1	7E-01	0.2
Aldh3a2	9E-01	0.2	Dzip1	1E-01	0.2
Inpp4a	9E-01	0.2	Pfdn5	3E-11	0.2
Tle4	9E-01	0.2	Cdc42bpb	9E-01	0.2
Adcy6	5E-01	0.2	Fam53b	7E-01	0.2
1810058I24Rik	2E-09	0.2	Lix1	9E-01	0.2
Tkt	5E-05	0.2	Arhgap17	7E-02	0.2
Nsmaf	4E-01	0.2	Pnisr	3E-03	0.2
Vrk2	8E-01	0.2	2310015A10Rik	7E-01	0.2
Mtg1	6E-01	0.2	Commd8	1E-01	0.2
Fbxl5	6E-01	0.2	Nudt18	4E-01	0.2
Ftsj1	6E-01	0.2	Sco2	7E-01	0.2
Fbxo34	7E-01	0.2	Ccdc51	3E-01	0.2
Gm47863	9E-01	0.2	Zfp219	5E-01	0.2
Zbtb17	6E-01	0.2	Aldh16a1	7E-01	0.2
Hddc3	9E-01	0.2	Stradb	4E-01	0.2
Stoml1	6E-01	0.2	Pten	1E-05	0.2
Gpr19	6E-01	0.2	Coil	8E-01	0.2
Wdr73	9E-01	0.2	Abcb7	9E-01	0.2
Crbn	6E-01	0.2	Yipf3	2E-03	0.2
Adam15	2E-01	0.2	Apobec3	9E-01	0.2
Hps5	8E-01	0.2	Cand2	4E-01	0.2
Ech1	5E-06	0.2	Nt5dc2	6E-01	0.2
Ppt1	2E-04	0.2	Fbxw4	1E+00	0.2
Frmd8	7E-01	0.2	Platr25	7E-01	0.2
Nsun3	3E-01	0.2	Zbtb17	5E-01	0.2
Map3k12	6E-01	0.2	Zfp113	3E-01	0.2
Zmym6	1E+00	0.2	Dennd4c	4E-01	0.2
AI987944	1E+00	0.2	Ubap1	4E-01	0.2
Rsrp1	3E-07	0.2	Dtwd2	3E-01	0.2
Myd88	1E+00	0.2	Hipk2	3E-02	0.2

Limb Mesenchyme						Chondrogenic					
Control			NICD2			Control			NICD2		
Gene	*p*	FC	Gene	*p*	FC	Gene	*p*	FC	Gene	*p*	FC
Map3k11	4E-02	0.2	Snhg4	3E-07	0.2	Kpna1	1E-02	0.2	Clec16a	2E-02	0.2
Plaa	2E-05	0.2	Usp19	1E-05	0.2	Taf4b	1E+00	0.2	Ufl1	1E-03	0.2
C730002L08Rik	2E-03	0.2	Nme1	6E-15	0.2	Park7	5E-25	0.2	Ngrn	1E-03	0.2
Adamts9	1E-08	0.2	Gpkow	5E-05	0.2	2310015A10Rik	9E-01	0.2	Arfrp1	2E-01	0.2
Bud31	9E-09	0.2	Tbc1d19	1E-05	0.2	Btbd1	2E-06	0.2	Marveld1	2E-02	0.2
Slc35a1	3E-07	0.2	Bach2	1E-04	0.2	Tmem87a	1E-03	0.2	Pnisr	1E-07	0.2
Jrkl	7E-06	0.2	Nelfa	5E-05	0.2	Thumpd3	1E-02	0.2	Invs	6E-03	0.2
Tspan6	2E-08	0.2	Prmt6	7E-04	0.2	Borcs8	3E-07	0.2	Lancl1	4E-03	0.2
Rab33b	8E-05	0.2	Utp18	4E-05	0.2	Nudt9	6E-06	0.2	Slc30a9	3E-04	0.2
Zfas1	1E-09	0.2	Riok3	6E-11	0.2	Gm15417	4E-05	0.2	Chac2	2E-01	0.2
Zswim8	3E-08	0.2	Fgfr1	5E-08	0.2	Zscan22	7E-01	0.2	Mbd6	3E-01	0.2
Eif5a2	8E-04	0.2	Amd1	3E-05	0.2	Tmem189	1E-02	0.2	Acad8	7E-01	0.2
Noc4l	1E-03	0.2	Sp2	2E-04	0.2	Arfrp1	8E-04	0.2	Styx	8E-02	0.2
Arid5a	3E-03	0.2	Pde7b	2E-07	0.2	Prkci	5E-03	0.2	Sh3bgrl	5E-09	0.2
Kri1	2E-05	0.2	Arhgef17	2E-06	0.2	Ccnt2	2E-04	0.2	Scap	3E-01	0.2
Bicra	5E-07	0.2	Dhx9	8E-08	0.2	Fam173a	4E-07	0.2	Pura	3E-10	0.2
Trrap	5E-08	0.2	Zfp111	7E-02	0.2	Cul9	2E-02	0.2	Tbc1d13	4E-01	0.2
Pdlim4	4E-09	0.2	Trio	7E-09	0.2	Kat6a	1E-02	0.2	Setd6	7E-01	0.2
Drg2	4E-04	0.2	Zfp68	3E-07	0.2	Zmym5	3E-05	0.2	Lrig2	1E-01	0.2
Xpo7	1E-07	0.2	Ube2g2	8E-04	0.2	Tle1	1E-03	0.2	Rbm48	2E-01	0.2
Abcc10	3E-03	0.2	Dcun1d1	1E-05	0.2	Usp24	8E-05	0.2	Gm14966	1E-01	0.2
Aasdhppt	5E-07	0.2	Zfp945	5E-05	0.2	Aasdhppt	8E-05	0.2	Mdn1	7E-03	0.2
Zfp39	6E-04	0.2	Adat1	2E-03	0.2	Scyl3	1E-01	0.2	Trmu	2E-01	0.2
Twist1	3E-13	0.2	Zbtb10	1E-02	0.2	Eif3c	6E-13	0.2	Ddx21	9E-07	0.2
Idua	6E-05	0.2	Celf1	4E-06	0.2	Usp9x	4E-10	0.2	Ankrd10	1E-01	0.2
Dynlrb1	4E-19	0.2	Kdm3b	4E-05	0.2	Map3k4	8E-03	0.2	B230354K17Rik	7E-02	0.2
Ubqln1	1E-06	0.2	Msantd2	8E-06	0.2	Mpzl1	1E-02	0.2	D830024N08Rik	2E-01	0.2
Itgb1	4E-25	0.2	Slc30a4	1E-03	0.2	Casc3	9E-04	0.2	Ivns1abp	5E-03	0.2
Gstcd	1E-06	0.2	N4bp1	7E-09	0.2	Fktn	4E-02	0.2	Mars	2E-01	0.2
Zc4h2	4E-03	0.2	Usp42	7E-04	0.2	Hlcs	7E-02	0.2	Trim34a	9E-01	0.2
Tmem167	1E-27	0.2	Slc49a4	1E-04	0.2	Pdp1	3E-05	0.2	Wdtc1	2E-01	0.2
Kti12	1E-04	0.2	Slit3	1E-13	0.2	Ep300	4E-05	0.2	Dync2li1	2E-02	0.2
Gpn1	5E-05	0.2	Mier2	7E-02	0.2	Zc3h14	4E-07	0.2	Cspg4	3E-02	0.2
Ano10	1E-05	0.2	Bfar	4E-05	0.2	Lpin3	3E-01	0.2	Lztfl1	1E-05	0.2
Hrct1	3E-04	0.2	Fubp1	3E-09	0.2	Spindoc	1E-01	0.2	Qk	2E-08	0.2
Sco1	2E-04	0.2	Gm15417	2E-05	0.2	Pogz	1E-02	0.2	Fam241b	3E-01	0.2
Slc30a5	2E-09	0.2	Utp14a	7E-06	0.2	Paf1	2E-03	0.2	Dhx16	1E-01	0.2
Dhtkd1	5E-04	0.2	Gm48678	1E-03	0.2	Wdr48	3E-04	0.2	Irf9	4E-01	0.2
Pbrm1	2E-13	0.2	Smpdl3b	4E-03	0.2	Zfp790	1E-01	0.2	Yju2	4E-01	0.2
Jkamp	6E-10	0.2	Smarca5	3E-15	0.2	Ints13	4E-02	0.2	Hnrnph1	8E-06	0.2
Dgcr6	5E-09	0.2	Rabl6	2E-07	0.2	Zfhx2	6E-01	0.2	R3hdm2	3E-07	0.2
Pdcl3	1E-09	0.2	Pbxip1	8E-09	0.2	Gm16835	2E-01	0.2	Kdm3b	7E-04	0.2
2210408I21Rik	8E-08	0.2	Anxa2	9E-24	0.2	Ak5	2E-02	0.2	Mrpl38	1E-01	0.2
Htt	2E-05	0.2	Dcun1d5	3E-12	0.2	Spice1	6E-01	0.2	Rab11fip2	3E-03	0.2
Rbfox2	8E-15	0.2	Klhl22	2E-05	0.2	D130020L05Rik	8E-01	0.2	Hnrnpr	7E-10	0.2
Riox2	9E-05	0.2	Car9	4E-03	0.2	N4bp2	5E-02	0.2	Med19	1E-03	0.2
Mylk	4E-10	0.2	4921511C10Rik	1E-03	0.2	Mesd	5E-13	0.2	Fkbp9	9E-09	0.2
Fam204a	3E-08	0.2	Orc4	3E-05	0.2	Gtf3c5	7E-01	0.2	Nr6a1os	5E-01	0.2
Zfp90	1E-04	0.2	Nop16	1E-06	0.2	Zscan29	4E-01	0.2	Zfp346	2E-01	0.2
Tpm4	3E-10	0.2	Cyth1	7E-03	0.2	Zmynd11	1E-06	0.2	Ppcs	1E-01	0.2
Mcu	6E-07	0.2	Ankib1	2E-07	0.2	Kctd20	3E-02	0.2	Cbx4	7E-01	0.2
Fosb	1E-09	0.2	Aifm1	4E-06	0.2	Kif7	4E-01	0.2	Mthfsl	4E-01	0.2
Smarce1	2E-11	0.2	Bnip1	1E-05	0.2	Gm26549	7E-01	0.2	E530011L22Rik	3E-01	0.2
Zfp148	2E-10	0.2	Arl6ip4	9E-09	0.2	Rfxap	6E-03	0.2	Pick1	4E-01	0.2
Ptx3	4E-11	0.2	Exosc9	9E-05	0.2	Hnrnpr	8E-12	0.2	Selenok	3E-16	0.2
Tln1	9E-16	0.2	Mtap	1E-08	0.2	Zc3h12c	4E-01	0.2	Tcea3	3E-08	0.2
Nono	7E-11	0.2	Dmac1	4E-08	0.2	Zfp263	1E-01	0.2	Mfsd14a	3E-02	0.2
Ddx10	2E-06	0.2	Eya4	4E-11	0.2	Alg3	2E-01	0.2	Dhtkd1	4E-01	0.2
Ankra2	9E-07	0.2	Prorp	5E-04	0.2	Rilpl2	3E-02	0.2	Yod1	6E-01	0.2
Gtf2a1	3E-06	0.2	Smco4	2E-08	0.2	Lig4	6E-01	0.2	Nr3c2	5E-01	0.2
Sike1	4E-07	0.2	Nt5dc1	2E-04	0.2	Trp53i13	5E-02	0.2	Sf3b6	7E-15	0.2
Trub2	5E-06	0.2	Uggt2	2E-06	0.2	Uso1	1E-05	0.2	Fbxl4	4E-01	0.2
Atf6b	3E-07	0.2	Txlna	4E-08	0.2	Arl6ip5	4E-11	0.2	Vps37a	7E-03	0.2
Nars2	3E-05	0.2	Dhx29	1E-05	0.2	Hist1h4i	2E-02	0.2	Bahcc1	9E-02	0.2
Gadd45b	2E-08	0.2	Nono	6E-10	0.2	AY036118	1E-15	0.2	Snapin	4E-04	0.2
Stard6	2E-02	0.2	Macf1	3E-12	0.2	Med19	4E-05	0.2	Mettl5	3E-01	0.2
Fam214b	3E-06	0.2	mt-Nd6	6E-07	0.2	B4galt4	3E-01	0.2	Ppp2r2a	2E-06	0.2
Ubac1	1E-07	0.2	Gm28198	9E-02	0.2	Naa80	3E-01	0.2	Polr2d	1E-03	0.2
Ncoa4	2E-06	0.2	Arfgef1	2E-07	0.2	Phkg2	9E-02	0.2	Ap4e1	4E-01	0.2
Fads3	1E-06	0.2	Rsrc1	9E-12	0.2	Mkrn1	1E-05	0.2	Afdn	8E-04	0.2
Itgb5	1E-07	0.2	Pfas	3E-02	0.2	Gpbp1	2E-07	0.2	Rfxap	2E-01	0.2
Zfp346	4E-05	0.2	Ssbp3	3E-08	0.2	Grcc10	6E-19	0.2	Lin7c	2E-02	0.2
Cog3	7E-06	0.2	Rest	5E-08	0.2	Trmu	2E-01	0.2	Phka1	7E-02	0.2

Fibroblast						Fibroblast?					
Control			NICD2			Control			NICD2		
Gene	*p*	FC	Gene	*p*	FC	Gene	*p*	FC	Gene	*p*	FC
Oaf	8E-07	0.1	Copb1	8E-13	0.1	Gm3604	2E-07	0.4	Nectin3	6E-25	0.4
S1pr2	1E-03	0.1	Gdi1	9E-05	0.1	Dimt1	3E-12	0.4	Nsun5	8E-12	0.4
Ccdc9b	2E-02	0.1	Rcor1	3E-04	0.1	Cep164	2E-18	0.4	Prpf38a	2E-21	0.4
2310015A10Rik	6E-01	0.1	Gm14798	7E-01	0.1	Sema3d	2E-12	0.4	Hdgfl3	2E-20	0.4
Osbpl11	2E-03	0.1	Ticam1	4E-01	0.1	Uros	3E-03	0.4	Zc3hav1	1E-28	0.4
A930007I19Rik	7E-02	0.1	Rps23	1E-52	0.1	Abhd10	9E-16	0.4	Cactin	1E-24	0.4
Wdfy1	7E-02	0.1	2410131K14Rik	2E-01	0.1	Smpdl3b	4E-19	0.4	Frat2	6E-10	0.4
Tbc1d23	9E-06	0.1	Trim23	2E-02	0.1	Junos	1E-16	0.4	Lrrc57	4E-19	0.4
Lix1l	3E-09	0.1	Chst12	3E-02	0.1	Gca	7E-08	0.4	Syt12	1E-19	0.4
Gdap2	6E-03	0.1	Parp11	2E-01	0.1	Tmem97	3E-19	0.4	Socs4	9E-18	0.4
Ankrd17	1E-12	0.1	Diaph2	7E-05	0.1	8030462N17Rik	9E-12	0.4	Cdc34	2E-23	0.4
Rhou	9E-03	0.1	Zfp605	2E-01	0.1	Zfp512	2E-09	0.4	Creld2	2E-26	0.4
Fzd2	7E-12	0.1	Washc2	4E-07	0.1	Zdhhc18	2E-12	0.4	Mrpl42	2E-34	0.4
Uhrf2	7E-05	0.1	Eef1g	1E-24	0.1	Clic4	1E-32	0.4	Cryl1	2E-20	0.4
Stx4a	6E-09	0.1	Dhrs7	9E-07	0.1	Stxbp6	7E-19	0.4	Map7d3	9E-20	0.4
Rpl36a-ps1	7E-03	0.1	Pqlc3	1E-01	0.1	Nsun6	2E-18	0.4	Rp2	4E-22	0.4
Stag1	4E-13	0.1	Tmed8	2E-01	0.1	Prkd2	2E-13	0.4	Hdac9	2E-22	0.4
Tmem87b	2E-04	0.1	Dync1h1	3E-08	0.1	Mepce	2E-16	0.4	Klf10	2E-16	0.4
Nf1	3E-08	0.1	Dgkh	5E-01	0.1	Ywhaq	1E-47	0.4	Mrps26	9E-28	0.4
Brsk1	1E-02	0.1	C1qtnf12	3E-05	0.1	Mtpn	2E-40	0.4	Zfp422	1E-19	0.4
Mospd2	7E-05	0.1	Mdp1	1E-04	0.1	Gcnt2	4E-16	0.4	Palmd	5E-22	0.4
Fbxw7	2E-01	0.1	C030034I22Rik	3E-01	0.1	Alkbh4	2E-07	0.4	Ptms	9E-53	0.4
Klhl9	1E-07	0.1	Tmem181a	1E-01	0.1	Klhl42	3E-10	0.4	Lmbrd2	4E-21	0.4
Gm10076	2E-28	0.1	Tbrg1	6E-14	0.1	Agpat5	1E-16	0.4	H2-Q7	2E-17	0.4
Glb1	5E-05	0.1	Arsb	3E-02	0.1	Cab39l	1E-20	0.4	Slitrk5	1E-08	0.4
Dlat	5E-11	0.1	Zdhhc15	5E-01	0.1	Naa16	7E-18	0.4	Eif4enif1	2E-21	0.4
Ccdc8	4E-03	0.1	Rbsn	1E-03	0.1	Fam98b	3E-19	0.4	Prpf3	3E-21	0.4
Kctd13	2E-01	0.1	Mier3	3E-02	0.1	B230219D22Rik	1E-38	0.4	Cad	2E-22	0.4
Arpc1a	2E-16	0.1	Setd1b	4E-03	0.1	Topors	2E-21	0.4	Lacc1	7E-23	0.4
Ipmk	5E-02	0.1	Snx7	8E-10	0.1	Cpsf2	9E-18	0.4	Cyth2	3E-12	0.4
Rpl36a	7E-57	0.1	Abl2	8E-03	0.1	Clasp2	8E-12	0.4	Fli1	6E-24	0.4
Oxa1l	3E-03	0.1	Mfsd5	8E-03	0.1	Fmnl3	3E-22	0.4	Cox7a2	2E-96	0.4
Naca	1E-64	0.1	Slc9a9	9E-06	0.1	Ccm2	1E-21	0.4	Cops3	1E-23	0.4
Hscb	3E-04	0.1	Trim32	6E-02	0.1	Zc3h18	5E-21	0.4	Mrpl16	1E-17	0.4
Prmt7	3E-02	0.1	Rpl35a	8E-46	0.1	Mbd1	2E-16	0.4	Brcc3	5E-22	0.4
4930599N23Rik	4E-02	0.1	Git1	5E-01	0.1	Ewsr1	3E-19	0.4	Man2a1	1E-29	0.4
Arhgef10	5E-05	0.1	Birc3	1E-01	0.1	Slc7a11	8E-13	0.4	Txnl1	1E-59	0.4
Snx7	6E-16	0.1	Gm12743	6E-02	0.1	Ppp1cc	3E-23	0.4	Pphln1	2E-21	0.4
Scaper	5E-06	0.1	Mapk14	3E-03	0.1	Jph1	9E-09	0.4	Cars	5E-22	0.4
Fech	1E-05	0.1	Thap12	3E-05	0.1	Fkbp4	8E-23	0.4	Zfp597	2E-16	0.4
Cul5	3E-11	0.1	Focad	2E-03	0.1	Zfp597	5E-14	0.4	Sgcd	1E-32	0.4
Taf7	3E-02	0.1	Pex19	5E-03	0.1	Arl6ip4	5E-29	0.4	Lrrfip1	4E-34	0.4
Rack1	8E-51	0.1	Zcwpw2	4E-01	0.1	Igsf8	2E-15	0.4	Trmt2a	8E-19	0.4
Ttc12	2E-02	0.1	Nfkb2	2E-01	0.1	Zfp91	3E-42	0.4	Eif2b3	5E-08	0.4
Fez2	2E-05	0.1	Grn	8E-14	0.1	Akip1	5E-16	0.4	Ttll5	9E-23	0.4
Neu1	8E-04	0.1	Auh	2E-04	0.1	Pcmt1	1E-29	0.4	Tmem245	1E-21	0.4
Tbc1d12	4E-05	0.1	Tspan17	1E-06	0.1	Tmcc2	4E-09	0.4	Alg14	1E-24	0.4
Irf2	3E-07	0.1	Hspb8	2E-04	0.1	Cdc23	5E-15	0.4	Dglucy	4E-21	0.4
Sgsh	5E-01	0.1	Rab22a	4E-07	0.1	Ehmt2	2E-20	0.4	Gspt1	1E-45	0.4
Cd9	6E-05	0.1	Mzt2	1E-03	0.1	Pank1	6E-18	0.4	Igsf8	5E-26	0.4
Far1	5E-05	0.1	Nfkb1	3E-04	0.1	Ptpn3	4E-16	0.4	Fdxacb1	1E-08	0.4
Rpl15	2E-80	0.1	Slc17a5	2E-01	0.1	Rbmx	3E-21	0.4	Etl4	2E-25	0.4
Snhg3	9E-07	0.1	Rpl14	6E-37	0.1	Qser1	4E-11	0.4	Fmnl3	7E-23	0.4
Errfi1	2E-01	0.1	Cdkn2a	9E-01	0.1	Tarbp2	4E-14	0.4	Tmx4	7E-27	0.4
Xpa	1E-07	0.1	Fam122a	1E-04	0.1	Snip1	4E-14	0.4	Tsku	8E-10	0.4
Zfpm2	1E-02	0.1	Fam160b2	5E-02	0.1	Snrpc	4E-26	0.4	Arhgap22	5E-20	0.4
Nbas	2E-03	0.1	Rpl23a	3E-26	0.1	Emx2	1E-08	0.4	Rin1	7E-17	0.4
Eno2	1E-02	0.1	Eif4e2	6E-09	0.1	Hs6st2	5E-26	0.4	Ccdc134	2E-16	0.4
Flna	3E-03	0.1	Dpp3	1E-02	0.1	Tmem183a	2E-19	0.4	Ppip5k1	1E-20	0.4
Srpx2	3E-31	0.1	Armh3	1E-01	0.1	Fam124a	3E-11	0.4	Zfand2a	4E-23	0.4
Sipa1l3	6E-04	0.1	Ano10	4E-03	0.1	Rrn3	7E-18	0.4	Tor1a	8E-25	0.4
Ap5m1	4E-02	0.1	Haghl	3E-06	0.1	Rapgef6	1E-24	0.4	Anapc7	6E-25	0.4
Fcgrt	1E-16	0.1	Scand1	3E-14	0.1	Prelp	7E-16	0.4	Ctnnal1	2E-12	0.4
Dhx58	9E-02	0.1	Bad	2E-07	0.1	Sh3yl1	7E-05	0.4	Parp16	7E-09	0.4
Ap3m2	4E-01	0.1	Entpd5	4E-01	0.1	Pkm	6E-53	0.4	Pkn2	8E-40	0.4
Chic2	7E-10	0.1	Map1s	4E-02	0.1	Lpin2	5E-17	0.4	Chrd	7E-11	0.4
Arpc3	1E-24	0.1	Syne3	2E-02	0.1	4930549G23Rik	4E-12	0.4	Ccdc68	9E-12	0.4
Surf1	2E-04	0.1	Krtcap2	1E-20	0.1	Eif5a	1E-39	0.4	Srm	5E-13	0.4
Fgfrl1	4E-04	0.1	Nsun6	1E-01	0.1	Zfp212	2E-13	0.4	Utp15	5E-15	0.4
Yif1a	2E-11	0.1	Amotl2	6E-07	0.1	Ccdc51	5E-16	0.4	Npepl1	2E-14	0.4
Map4k4	2E-10	0.1	Eif3k	9E-19	0.1	Zfp280b	3E-11	0.4	Arhgef2	5E-25	0.4
Czib	9E-11	0.1	Glmp	2E-05	0.1	Sf3b4	5E-15	0.4	Mtfp1	3E-10	0.4
Taf4b	2E-01	0.1	Strn	2E-02	0.1	Slc4a7	2E-16	0.4	Rbbp5	4E-14	0.4

Undefined						Articular/Synovial Fibroblast					
Control			NICD2			Control			NICD2		
Gene	*p*	FC	Gene	*p*	FC	Gene	*p*	FC	Gene	*p*	FC
						Calcoco1	4E-01	0.2	Hyal2	1E+00	0.2
						Gm34455	7E-01	0.2	Zfp110	2E-01	0.2
						Scfd2	4E-01	0.2	Mapk7	4E-01	0.2
						Rnf157	2E-01	0.2	Peg13	6E-01	0.2
						Rcan1	6E-01	0.2	Ssbp3	7E-03	0.2
						Mvd	3E-01	0.2	Rara	8E-01	0.2
						Snapc5	2E-02	0.2	Dock4	4E-01	0.2
						Stx16	1E-01	0.2	Anapc10	1E-01	0.2
						Mif	8E-06	0.2	Mtmr11	7E-01	0.2
						Gm19522	6E-01	0.2	Nudt14	8E-01	0.2
						Wrap73	5E-01	0.2	Pcnx4	9E-01	0.2
						Smap2	7E-02	0.2	Atp11a	6E-01	0.2
						Zfp512b	6E-01	0.2	Cluap1	1E+00	0.2
						Fam120c	6E-01	0.2	Cds2	6E-01	0.2
						Bcas3	2E-03	0.2	Slc27a4	6E-01	0.2
						Rmdn1	5E-02	0.2	Acyp2	3E-02	0.2
						Mkrn1	3E-01	0.2	Sgce	3E-02	0.2
						Ints5	5E-01	0.2	Dennd1a	2E-02	0.2
						Rap2b	6E-01	0.2	Cep152	2E-01	0.2
						Gpm6b	4E-06	0.2	Apba3	8E-01	0.2
						Fam149a	8E-01	0.2	Bsdc1	5E-01	0.2
						Dpy19l3	9E-01	0.2	Tmtc2	2E-01	0.2
						Cpne8	3E-06	0.2	Atp8b2	4E-01	0.2
						Arf6	2E-02	0.2	Il1rap	9E-01	0.2
						Zdhhc14	6E-01	0.2	Cd151	1E-02	0.2
						Atg10	9E-02	0.2	Bin3	7E-01	0.2
						Kansl1	4E-03	0.2	Chp1	1E-02	0.2
						Tmem242	5E-01	0.2	Usp40	3E-01	0.2
						Aldh2	2E-07	0.2	Acer3	3E-01	0.2
						Dclre1a	5E-01	0.2	Pou2f1	1E+00	0.2
						Ifit2	8E-01	0.2	mt-Nd1	1E-12	0.2
						Serpine2	3E-09	0.2	2210016L21Rik	1E-02	0.2
						Yipf2	9E-01	0.2	Lars2	2E-02	0.2
						C1qtnf12	3E-02	0.2	Tmem181a	6E-01	0.2
						Acadm	9E-07	0.2	Sdsl	4E-01	0.2
						Mysm1	2E-01	0.2	Snx6	3E-05	0.2
						Zscan26	2E-01	0.2	Dpm3	2E-06	0.2
						Cat	2E-06	0.2	Mgme1	5E-01	0.2
						Nemf	2E-02	0.2	Tmub1	6E-01	0.2
						Fuca2	4E-01	0.2	Ndst1	5E-01	0.2
						Tradd	8E-01	0.2	Tbc1d4	1E+00	0.2
						Pdia4	1E-04	0.2	Nln	3E-01	0.2
						Smcr8	3E-01	0.2	Lix1l	2E-02	0.2
						Slc25a24	4E-01	0.2	Serf2	1E-19	0.2
						Clybl	6E-02	0.2	Cenpv	6E-01	0.2
						Magee1	9E-01	0.2	Clk1	6E-02	0.2
						Sumf2	1E-01	0.2	Wdr11	9E-01	0.2
						Zfp239	6E-01	0.2	A430018G15Rik	5E-01	0.2
						Pip4k2c	9E-01	0.2	Mavs	4E-01	0.2
						Pde4a	1E+00	0.2	Dok5	4E-01	0.2
						Zc3h10	6E-01	0.2	Cib2	4E-01	0.2
						Rogdi	2E-01	0.2	Fbxl20	6E-01	0.2
						Mindy1	7E-01	0.2	Plgrkt	6E-02	0.2
						Hectd4	8E-01	0.2	Twsg1	1E-01	0.2
						Rbms2	1E-02	0.2	Trp53bp1	6E-01	0.2
						Nfil3	3E-01	0.2	Khnyn	4E-01	0.2
						Zfp934	9E-01	0.2	Ube4a	9E-02	0.2
						Zfp963	8E-01	0.2	Wdr60	3E-01	0.2
						Copz1	1E-02	0.2	Zc3h10	4E-01	0.2
						Usp18	7E-01	0.2	Ormdl1	3E-01	0.2
						Stx8	4E-04	0.2	Stard3	3E-01	0.2
						Zmynd8	5E-02	0.2	Cry2	8E-01	0.2
						Ago4	7E-01	0.2	Stat1	2E-01	0.2
						Pkn1	7E-01	0.2	Slc16a1	3E-01	0.2
						Uap1	1E-01	0.2	Tspan4	2E-04	0.2
						CAAA01147332.1	9E-01	0.2	Telo2	6E-01	0.2
						Mpst	4E-01	0.2	Lacc1	5E-01	0.2
						Cbr1	5E-04	0.2	Carhsp1	4E-02	0.2
						Gab2	8E-01	0.2	1810034E14Rik	5E-01	0.2
						Nat2	3E-01	0.2	Shc1	4E-02	0.2
						Cln5	5E-01	0.2	Rab3gap2	7E-01	0.2
						Stam2	8E-02	0.2	Fars2	8E-02	0.2
						Camk2n1	1E+00	0.2	Ciao3	6E-01	0.2

Limb Mesenchyme					
Control			NICD2		
Gene	p	FC	Gene	p	FC
Dip2c	1E-10	0.2	Zcchc7	5E-10	0.2
Dcun1d1	3E-06	0.2	Sap18	7E-07	0.2
Haus2	2E-05	0.2	Psmd5	1E-06	0.2
4930484I04Rik	1E-02	0.2	Azin1	1E-07	0.2
Ctbp2	1E-04	0.2	Nlk	4E-06	0.2
Sf3a1	8E-06	0.2	Pkia	8E-03	0.2
Sart3	2E-05	0.2	Tomm70a	2E-06	0.2
Rab31	6E-08	0.2	Ccdc163	5E-03	0.2
Atmin	4E-03	0.2	Nr4a1	2E-05	0.2
Cacna1c	2E-07	0.2	Vcl	8E-07	0.2
Pigu	1E-05	0.2	Mcm3ap	1E-05	0.2
Cbl	6E-05	0.2	Cmtm6	2E-03	0.2
Mindy3	1E-05	0.2	Trim66	2E-02	0.2
Strip1	3E-06	0.2	Pdzd8	2E-04	0.2
Nrf1	3E-07	0.2	Hmg20b	2E-07	0.2
Zfp583	1E-03	0.2	Rcbtb1	2E-05	0.2
Uros	1E-06	0.2	Crk	1E-06	0.2
4930511M06Rik	9E-04	0.2	Dimt1	9E-05	0.2
Snhg8	2E-12	0.2	Med23	3E-05	0.2
Desi1	1E-06	0.2	Rab4b	4E-08	0.2
Brd1	2E-09	0.2	Fahd2a	2E-05	0.2
Yars2	1E-05	0.2	Sil1	7E-07	0.2
Reps1	6E-04	0.2	Plekhf2	1E-02	0.2
Carmn	2E-05	0.2	Hoxc6	8E-04	0.2
Fam114a2	6E-08	0.2	Zkscan1	4E-06	0.2
Lrrc58	5E-11	0.2	Zfp563	3E-02	0.2
Ahi1	6E-07	0.2	Api5	5E-08	0.2
Stx5a	4E-09	0.2	Clstn1	3E-06	0.2
Lsm8	8E-10	0.2	Snrnp200	7E-07	0.2
Edc3	1E-05	0.2	Birc2	3E-04	0.2
Itch	4E-08	0.2	Cep76	2E-03	0.2
Ap1b1	4E-06	0.2	Steap2	8E-05	0.2
Ipp	5E-03	0.2	Otud1	5E-02	0.2
Ddx18	3E-07	0.2	Tardbp	2E-06	0.2
Epc1	9E-07	0.2	Casp2	6E-02	0.2
Ext1	2E-14	0.2	Yme1l1	3E-11	0.2
Mtmr12	4E-04	0.2	Zfp292	5E-08	0.2
Wsb1	4E-07	0.2	Utp11	4E-07	0.2
Crcp	2E-06	0.2	Ncs1	6E-04	0.2
Ccdc66	2E-07	0.2	Gm20404	2E-02	0.2
Psmg3	6E-07	0.2	Mosmo	5E-05	0.2
Sltm	4E-09	0.2	Abat	1E-06	0.2
Eri3	3E-10	0.2	Gm20342	5E-04	0.2
Fbxw5	3E-05	0.2	Mycbp	2E-05	0.2
Ndel1	6E-08	0.2	2810004N23Rik	3E-07	0.2
Snx11	2E-04	0.2	Antxr1	5E-08	0.2
Slc1a6	1E-05	0.2	Epb41l2	6E-08	0.2
Hnrnph1	3E-07	0.2	Arxes2	9E-05	0.2
Ankrd28	1E-07	0.2	Rnf214	2E-05	0.2
Syde1	1E-06	0.2	Dnajc7	5E-08	0.2
Rab10	9E-14	0.2	Lpin2	1E-04	0.2
Carnmt1	9E-07	0.2	Ptpn2	3E-06	0.2
Pdhb	4E-06	0.2	Ralgapb	5E-07	0.2
Lyar	2E-10	0.2	Tmem238	4E-06	0.2
Armh3	1E-04	0.2	Riiad1	5E-03	0.2
Btbd19	1E-02	0.2	Nip7	1E-07	0.2
Golga5	7E-08	0.2	Vbp1	6E-07	0.2
Mtpap	2E-06	0.2	Gnpnat1	3E-06	0.2
Zkscan1	3E-06	0.2	Syvn1	3E-03	0.2
Timmdc1	6E-06	0.2	Nol11	1E-05	0.2
Fbxo10	1E-05	0.2	Timm9	1E-06	0.2
Riox1	5E-02	0.2	Edrf1	6E-04	0.2
Ttl	9E-04	0.2	Cd59a	2E-05	0.2
Zfp598	2E-04	0.2	Ddx10	6E-07	0.2
Shroom4	1E-05	0.2	Ucma	3E-46	0.2
Zbtb9	8E-05	0.2	Nrf1	7E-06	0.2
Tbrg1	1E-16	0.2	Slc35e4	3E-03	0.2
Gm12258	2E-02	0.2	Rsl1d1	6E-11	0.2
Naa25	9E-05	0.2	Otud6b	1E-05	0.2
Srp72	1E-11	0.2	Nbas	3E-05	0.2
Ttbk2	1E-04	0.2	Mbtd1	3E-06	0.2
Tmem164	7E-07	0.2	Phka1	4E-06	0.2
Dmwd	5E-06	0.2	Ptpn4	6E-06	0.2

Chondrogenic					
Control			NICD2		
Gene	p	FC	Gene	p	FC
Arel1	4E-01	0.2	Fam234a	4E-02	0.2
Daglb	3E-01	0.2	Gna12	1E-02	0.2
Arhgef6	7E-02	0.2	Parg	1E-02	0.2
Cbll1	4E-03	0.2	Usp12	3E-01	0.2
Gtf2f2	3E-01	0.2	Cdk8	8E-05	0.2
Steap2	3E-02	0.2	Tmem223	3E-08	0.2
Arhgef17	7E-04	0.2	4933434E20Rik	9E-03	0.2
Veph1	9E-02	0.2	Slc2a8	2E-01	0.2
Ddx50	1E-14	0.2	Zfp664	1E-03	0.2
Itpr2	6E-05	0.2	Ctr9	1E-02	0.2
Huwe1	6E-10	0.2	Polr2m	8E-10	0.2
Pip4p2	1E-04	0.2	Gm46367	1E+00	0.2
Tchp	2E-01	0.2	5530601H04Rik	2E-01	0.2
Sybu	2E-02	0.2	Serpina3n	4E-01	0.2
Vmac	2E-01	0.2	Camsap1	1E-02	0.2
Polr2m	1E-10	0.2	Pogz	5E-02	0.2
Saysd1	3E-07	0.2	Ankrd52	2E-01	0.2
Nrep	1E-01	0.2	Gm4285	1E+00	0.2
Wdpcp	8E-04	0.2	Slc25a26	4E-01	0.2
Ttc8	1E-02	0.2	Yap1	6E-03	0.2
Phf21a	5E-08	0.2	Zc3h12c	7E-01	0.2
Gatd3a	1E-03	0.2	Mpp5	2E-02	0.2
Cep83os	1E+00	0.2	Tmem189	2E-01	0.2
Gtf2b	7E-06	0.2	Cmtm6	1E-01	0.2
Creb1	6E-04	0.2	Selenoi	5E-01	0.2
Pram1	8E-02	0.2	B930036N10Rik	2E-01	0.2
Ostc	7E-12	0.2	Sod1	9E-07	0.2
Ryk	2E-11	0.2	Phactr4	1E-02	0.2
Fuca2	3E-04	0.2	Ivd	3E-03	0.2
Adipor1	2E-09	0.2	Uqcc3	8E-07	0.2
Aldh2	7E-16	0.2	Nudcd3	5E-07	0.2
Tmem245	1E-03	0.2	Wdr4	7E-01	0.2
Dars	5E-08	0.2	Dcun1d4	6E-02	0.2
Bbs12	7E-01	0.2	Mafg	1E-02	0.2
Ammecr1	2E-02	0.2	Cdc37l1	1E-05	0.2
Vps13d	2E-02	0.2	B230217O12Rik	5E-01	0.2
Tfb2m	3E-02	0.2	Cpsf3	8E-03	0.2
Fam53b	2E-02	0.2	Lrp1	1E-13	0.2
Abhd18	2E-01	0.2	Ppp6r2	1E-01	0.2
Cep97	5E-01	0.2	Mtif3	1E-01	0.2
Phip	3E-06	0.2	Ccdc73	5E-01	0.2
Mib2	2E-01	0.2	Bcar1	6E-01	0.2
Srprb	3E-03	0.2	Vmac	2E-01	0.2
Gpr107	1E-03	0.2	Aste1	8E-01	0.2
Slc9a6	2E-01	0.2	Brsk1	6E-01	0.2
Tvp23b	2E-08	0.2	Ift88	1E-02	0.2
Zc3h7b	1E-02	0.2	Ndufaf3	5E-04	0.2
E4f1	3E-01	0.2	Fbxw4	3E-02	0.2
Snx13	1E-03	0.2	Tpd52	7E-02	0.2
Srsf4	5E-04	0.2	5430405H02Rik	1E-01	0.2
Smim13	3E-03	0.2	Nsun3	8E-01	0.2
Rflnb	8E-01	0.2	Znrf2	6E-02	0.2
Nrf1	1E-03	0.2	Uri1	4E-05	0.2
Atp11b	3E-03	0.2	Ddx49	2E-01	0.2
Gm47863	5E-01	0.2	Map3k4	7E-03	0.2
B930036N10Rik	1E-01	0.2	Stard3	5E-01	0.2
Zbed4	1E+00	0.2	B3galt6	5E-01	0.2
Itfg1	6E-05	0.2	Gpt2	6E-01	0.2
Kifap3	1E-09	0.2	Fbxo21	2E-01	0.2
Msto1	2E-01	0.2	Mettl18	6E-01	0.2
Tmem107	1E-01	0.2	Timm29	4E-01	0.2
Ubap2	1E-06	0.2	Whamm	4E-01	0.2
Fam98c	5E-01	0.2	Tra2b	4E-06	0.2
Slc39a7	5E-09	0.2	Zfp579	4E-01	0.2
Zfp354c	4E-02	0.2	Gas5	5E-08	0.2
Cttnbp2nl	3E-03	0.2	Tmem161a	3E-01	0.2
Wbp2	2E-03	0.2	Grsf1	5E-02	0.2
Smox	2E-01	0.2	Itfg2	1E-01	0.2
Eif1	1E-22	0.2	Kat5	4E-01	0.2
Bcdin3d	4E-01	0.2	Mbtps1	2E-04	0.2
Bloc1s5	1E-01	0.2	Ctnnd1	3E-03	0.2
Slc11a2	3E-02	0.2	B9d1	3E-02	0.2
Tsr1	1E-01	0.2	Smim8	2E-04	0.2

Fibroblast					
Control			NICD2		
Gene	p	FC	Gene	p	FC
Tmed3	9E-24	0.1	Pck2	3E-04	0.1
Gm14164	1E-02	0.1	Asl	4E-01	0.1
Vps35	6E-12	0.1	Dera	4E-02	0.1
Rab34	9E-16	0.1	Zswim7	2E-01	0.1
Cbr4	3E-03	0.1	Zfp365	1E-01	0.1
			Kmt5b	7E-04	0.1
			Stx18	1E-02	0.1
			Atxn10	3E-11	0.1
			Camta2	2E-01	0.1
			Tcaf1	6E-07	0.1
			Zbtb11os1	7E-03	0.1
			Ptpn12	1E-03	0.1
			P3h1	1E-04	0.1
			Txndc5	7E-08	0.1
			Rap1b	4E-11	0.1
			Lbh	2E-05	0.1
			Dnajb14	1E-03	0.1
			Ddb1	2E-08	0.1
			Flna	8E-02	0.1
			Trip12	6E-08	0.1
			Atg4a	3E-02	0.1
			Pramef8	3E-01	0.1
			Mccc1	4E-03	0.1
			Dbnl	3E-06	0.1
			Btrc	2E-03	0.1
			Abcb1b	3E-01	0.1
			Mrps36	8E-10	0.1
			Zfpm2	1E-01	0.1
			Sirt7	6E-02	0.1
			Ppp2r5b	8E-02	0.1
			Lrrc75a	3E-01	0.1
			Nedd4	4E-22	0.1
			Dnmbp	5E-02	0.1
			Ak1	6E-09	0.1
			Zfp358	9E-02	0.1
			Gtf2e1	3E-02	0.1
			Pdha1	5E-09	0.1
			Abcb10	2E-01	0.1
			Plscr3	3E-05	0.1
			Pde8a	8E-08	0.1
			Pprc1	5E-02	0.1
			Letm2	5E-02	0.1
			Rab33b	2E-01	0.1
			Dlg3	6E-02	0.1
			Sel1l	2E-06	0.1
			Acox3	3E-01	0.1
			Isca1	4E-02	0.1
			Adal	7E-01	0.1
			Ppp2r3a	1E-03	0.1
			Rpl18	1E-34	0.1
			Leprot	1E-04	0.1
			Snhg20	1E-01	0.1
			Rabep2	1E-01	0.1
			Gm46218	8E-02	0.1
			Elf1	1E-03	0.1
			Gm26797	7E-01	0.1
			Fam117b	3E-02	0.1
			Plpp1	3E-07	0.1
			Proca1	2E-01	0.1
			Klhl9	4E-05	0.1
			Csk	3E-02	0.1
			Rps12	9E-32	0.1
			Tspo	2E-26	0.1
			Dolpp1	5E-01	0.1
			Macrod2	2E-01	0.1
			Atp7a	1E-01	0.1
			Fkbp15	7E-04	0.1
			Pou6f1	9E-02	0.1
			Galns	2E-01	0.1
			Galnt2	5E-05	0.1
			Cd81	1E-12	0.1
			4932438A13Rik	1E-03	0.1
			Ctns	2E-01	0.1

Fibroblast?					
Control			NICD2		
Gene	p	FC	Gene	p	FC
Wasf1	2E-18	0.4	Ddx19a	8E-19	0.4
Usp28	4E-12	0.4	Irf1	2E-25	0.4
Snhg5	1E-14	0.4	Slfn8	2E-19	0.4
Sipa1l3	1E-11	0.4	Rac1	1E-48	0.4
Plpp2	8E-17	0.4	Ankrd49	2E-20	0.4
Bclaf3	2E-14	0.4	Snrpg	7E-105	0.4
Cep41	7E-17	0.4	Polg	6E-14	0.4
Spata5	4E-11	0.4	Pde12	3E-21	0.4
Glis2	1E-16	0.4	Celf5	5E-13	0.4
Uvrag	7E-15	0.4	Pck2	2E-28	0.4
Gm50163	1E-06	0.4	Lama3	6E-17	0.4
Rdx	6E-50	0.4	Dpysl2	5E-29	0.4
Tusc1	8E-13	0.4	Tnc	2E-18	0.4
Fam118b	1E-06	0.4	Hspe1-rs1	4E-14	0.4
Abcf2	6E-15	0.4	Pkd2l2	1E-06	0.4
Ptrh2	3E-16	0.4	Ewsr1	1E-31	0.4
Sh3rf2	4E-09	0.4	Rgs17	4E-15	0.4
Gm34455	2E-03	0.4	Terf2	7E-23	0.4
Arpc1b	2E-26	0.4	Nudcd2	7E-38	0.4
Ddx27	4E-16	0.4	Zfp219	2E-11	0.4
Rmdn3	9E-16	0.4	Zbtb25	2E-18	0.4
Upf3b	3E-18	0.4	Cstf1	1E-18	0.4
Polr2c	3E-14	0.4	Mcu	3E-24	0.4
Rsph1	1E-07	0.4	Esyt1	2E-20	0.4
Dynll1	1E-28	0.4	Zcrb1	9E-54	0.4
Bmp2k	4E-15	0.4	Ppp1cc	2E-39	0.4
Mtmr2	1E-13	0.4	Gsdme	2E-18	0.4
Apbb1ip	3E-12	0.4	Nol7	1E-30	0.4
Med14	1E-17	0.4	Zfp959	5E-06	0.4
Lims1	1E-30	0.4	Ahsa1	4E-16	0.4
Sgsm2	5E-16	0.4	Cdyl	1E-18	0.4
Blvrb	1E-19	0.4	Rbm17	2E-34	0.4
Cth	4E-09	0.4	Hnrnpul2	4E-21	0.4
Taf15	4E-24	0.4	Rybp	6E-15	0.4
Agps	6E-16	0.4	Cdc23	4E-18	0.4
Dxo	6E-18	0.4	Klhl5	1E-20	0.4
Mrps22	7E-19	0.4	Gtf2f2	2E-17	0.4
Trp53	7E-23	0.4	Etaa1os	7E-13	0.4
Ppp4r3a	8E-18	0.4	Nkap	7E-20	0.4
Mei4	3E-07	0.4	Calm3	2E-35	0.4
Zfp449	1E-15	0.4	Zdhhc21	1E-19	0.4
Psme3	2E-17	0.4	Polr2c	7E-21	0.4
Gm26590	1E-07	0.4	Ap1s1	9E-34	0.4
Ppif	5E-13	0.4	Utp6	1E-21	0.4
C1ql3	2E-07	0.4	Pdap1	3E-53	0.4
Ctnnbip1	4E-20	0.4	Prelid1	6E-48	0.4
4930453N24Rik	4E-20	0.4	Sav1	9E-17	0.4
Gle1	2E-20	0.4	Raver2	2E-12	0.4
Cdc42ep2	1E-08	0.4	Tmcc2	4E-11	0.4
Tap1	4E-12	0.4	Gm4876	2E-17	0.4
Gapdh	3E-72	0.4	Ddx27	6E-24	0.4
Ap1g2	3E-06	0.4	Ptpn11	4E-22	0.4
Rpp21	3E-20	0.4	Mad2l2	3E-22	0.4
Brpf3	6E-17	0.4	Prkce	3E-27	0.4
Armc1	3E-19	0.4	Eef1akmt2	7E-11	0.4
Ddx1	7E-32	0.4	Actr3	6E-34	0.4
Cfl1	6E-50	0.4	Itga5	6E-22	0.4
Elf2	1E-22	0.4	Thap12	2E-23	0.4
Slc8b1	1E-05	0.4	Phlpp1	4E-21	0.4
Fus	5E-32	0.4	Fermt2	1E-23	0.4
Frat2	1E-05	0.4	Tial1	5E-24	0.4
Fh1	3E-19	0.4	Cbx6	4E-20	0.4
Ints13	1E-06	0.4	Hdac1	4E-26	0.4
Anks6	3E-07	0.4	Tcp11l1	5E-15	0.4
Coa7	2E-13	0.4	Pabpn1	2E-38	0.4
Slc25a37	1E-17	0.4	Kif5b	5E-48	0.4
Gm17494	2E-06	0.4	Serbp1	9E-98	0.4
Ergic2	4E-30	0.4	Ei24	1E-21	0.4
Map4k4	4E-17	0.4	Trmt44	3E-09	0.4
Tmem232	2E-07	0.4	Sgsm2	1E-17	0.4
Lin37	2E-16	0.4	Agps	9E-20	0.4
Gucd1	5E-14	0.4	Men1	4E-17	0.4
Sumo3	8E-25	0.4	Trim16	4E-11	0.4

Undefined					
Control			NICD2		
Gene	p	FC	Gene	p	FC

Articular/Synovial Fibroblast					
Control			NICD2		
Gene	p	FC	Gene	p	FC
Zfp407	5E-01	0.2	Rpl29	3E-18	0.2
Brat1	4E-01	0.2	Pcdhb22	1E-01	0.2
Ski	4E-03	0.2	Sdf4	1E-05	0.2
Scap	3E-01	0.2	Dhrs9	5E-01	0.2
Uhrf2	1E+00	0.2	Adam15	6E-01	0.2
Mfap1a	1E+00	0.2	Enho	2E-01	0.2
Pskh1	8E-01	0.2	Kif16b	8E-01	0.2
Mospd2	3E-01	0.2	2810403D21Rik	3E-01	0.2
Pex6	7E-01	0.2	Mbd5	2E-03	0.2
Pcnx	1E+00	0.2	Coq9	9E-01	0.2
Zfp758	4E-01	0.2	Zbtb20	3E-06	0.2
Snx14	7E-01	0.2	Cog1	8E-01	0.2
Zfp397	4E-01	0.2	Usp30	3E-01	0.2
Ulk4	8E-01	0.2	Ptdss2	7E-01	0.2
Smg8	4E-01	0.2	A630072M18Rik	2E-01	0.2
Rpl36a-ps1	6E-01	0.2	Cox7a2l	2E-07	0.2
Rictor	3E-01	0.2	Vav2	2E-01	0.2
Vopp1	7E-01	0.2	Cox4i1	1E-15	0.2
Slc31a2	6E-01	0.2	Tcf7l1	9E-04	0.2
Ganc	1E+00	0.2	Mtfmt	5E-01	0.2
Relb	1E+00	0.2	Dab2	3E-05	0.2
Rsph9	5E-01	0.2	Plat	9E-01	0.2
Sec61a2	7E-01	0.2	Gm16835	4E-01	0.2
Acox1	7E-02	0.2	B230307C23Rik	2E-01	0.2
Wnk1	3E-04	0.2	Hebp2	4E-01	0.2
Prdm16	3E-01	0.2	D230025D16Rik	6E-01	0.2
Gnptg	4E-01	0.2	Pdcd4	7E-05	0.2
Arid5b	2E-05	0.2	Stk24	1E-02	0.2
Inpp1	8E-01	0.2	Pepd	7E-01	0.2
Zfp58	3E-01	0.2	Cebpb	2E-02	0.2
Bcl2l11	5E-01	0.2	Pef1	4E-01	0.2
Ctsf	1E-01	0.2	H2afj	1E-03	0.2
E4f1	8E-01	0.2	Ahcyl2	9E-01	0.2
Zcchc14	6E-01	0.2	Ppp1r37	6E-01	0.2
Ifnar2	1E-04	0.2	C1qtnf12	2E-01	0.2
Ankrd27	4E-01	0.2	Ccpg1os	1E+00	0.2
Ttc26	5E-01	0.2	Atg12	8E-01	0.2
Hexb	9E-02	0.2	Atxn7l1	1E-01	0.2
Rnf115	2E-01	0.2	Ift80	4E-01	0.2
Zfp119a	3E-01	0.2	Zfp319	4E-01	0.2
Mri1	8E-01	0.2	Sufu	9E-01	0.2
Atpaf1	7E-01	0.2	Rnf217	4E-01	0.2
Cyb5r4	9E-01	0.2	Dchs1	7E-01	0.2
Dmap1	9E-01	0.2	Ccdc9	9E-01	0.2
Extl2	5E-01	0.2	Rab22a	2E-02	0.2
Vps26a	3E-02	0.2	Zbtb24	4E-01	0.2
4931428F04Rik	3E-01	0.2	Arnt2	3E-01	0.2
Zfp335os	5E-01	0.2	Gm4924	6E-01	0.2
Otud7b	3E-01	0.2	Gm20732	5E-01	0.2
Atraid	6E-08	0.2	Scand1	2E-06	0.2
Ftl1-ps1	7E-02	0.2	Chpf2	1E+00	0.2
Mbd5	1E-03	0.2	Zfp267	4E-01	0.2
Pxmp4	3E-01	0.2	Shroom3	1E-01	0.2
AY036118	8E-08	0.2	Fbrs	7E-01	0.2
Tvp23a	3E-01	0.2	Rnf130	2E-01	0.2
Glis1	7E-01	0.2	B9d2	9E-01	0.2
Abhd11	1E+00	0.2	Bloc1s5	4E-01	0.2
Rpusd4	2E-01	0.2	Mdga1	8E-01	0.2
Ap1s2	6E-01	0.2	Hoxc4	4E-01	0.2
Tecpr2	1E+00	0.2	Plekha4	8E-01	0.2
Garnl3	9E-01	0.2	Psenen	2E-04	0.2
Btrc	9E-02	0.2	Aldh7a1	9E-02	0.2
Dhx40	2E-01	0.2	Sema4c	3E-01	0.2
Plekhm1	5E-01	0.2	Ipmk	2E-01	0.2
Ppp2r1b	4E-01	0.2	Rps27	1E-18	0.2
Mmut	4E-02	0.2	Cavin1	6E-04	0.2
Lars2	4E-02	0.2	Mus81	1E-01	0.2
Tmem135	2E-01	0.2	Eral1	3E-01	0.2
Tln2	8E-03	0.2	Med9	5E-01	0.2
Zfp868	5E-01	0.2	Egfl7	5E-01	0.2
Lpar1	1E-02	0.2	Cbr2	9E-01	0.2
2810002D19Rik	5E-01	0.2	Rnf227	6E-01	0.2
Cep170	3E-02	0.2	Sri	2E-04	0.2

Limb Mesenchyme

Control			NICD2		
Gene	*p*	FC	Gene	*p*	FC
Gm11613	9E-04	0.2	1810009A15Rik	3E-06	0.2
Mapk8ip3	4E-04	0.2	Slc39a14	4E-06	0.2
Birc6	1E-10	0.2	Zdhhc5	5E-05	0.2
Icmt	4E-04	0.2	Rnf34	1E-04	0.2
Tm9sf4	2E-10	0.2	Elp2	3E-05	0.2
Tmed2	1E-17	0.2	Chordc1	2E-08	0.2
Pigk	9E-07	0.2	Scfd1	2E-09	0.2
Ssr1	2E-11	0.2	Fxr1	8E-11	0.2
Dimt1	2E-07	0.2	Ammecr1l	7E-06	0.2
Mta1	2E-07	0.2	3110056K07Rik	1E-02	0.2
Crkl	3E-06	0.2	Bicdl1	2E-02	0.2
Rptor	9E-06	0.2	Clptm1l	1E-06	0.2
Akt1s1	3E-09	0.2	Exoc3	8E-10	0.2
Nudcd2	9E-12	0.2	Wdr18	1E-05	0.2
Cox15	3E-03	0.2	Bicc1	7E-12	0.2
Dlg3	5E-05	0.2	Mynn	2E-04	0.2
3110056K07Rik	6E-05	0.2	Castor1	3E-03	0.2
Morc2a	8E-06	0.2	Clasp1	1E-05	0.2
Serinc1	2E-12	0.2	Siah1a	5E-06	0.2
Pcnp	2E-11	0.2	Fam222b	5E-06	0.2
Nedd4	2E-36	0.2	Ramac	8E-08	0.2
Srsf9	5E-14	0.2	Safb	3E-04	0.2
Cmtr1	4E-05	0.2	Pqlc1	5E-03	0.2
Hdac2	4E-08	0.2	Rrp15	2E-04	0.2
Pam16	1E-10	0.2	Gpatch2	6E-05	0.2
Lig3	2E-06	0.2	Ube2j1	4E-06	0.2
Map2k4	1E-06	0.2	Mbd2	2E-08	0.2
Wdr92	2E-04	0.2	Slc39a3	4E-02	0.2
Tmem18	5E-05	0.2	Tbx4	5E-06	0.2
A930001C03Rik	2E-02	0.2	Fyttd1	1E-08	0.2
2810004N23Rik	3E-08	0.2	Pbx3	1E-07	0.2
Ttc28	2E-13	0.2	Hotairm1	1E-05	0.2
Polr3k	1E-08	0.2	Tmem214	8E-06	0.2
Cep63	8E-07	0.2	Luc7l	2E-06	0.2
Cul4b	6E-06	0.2	Dock6	7E-04	0.2
Srpk1	4E-07	0.2	Zfp286	5E-03	0.2
Fhl1	6E-09	0.2	Efcab7	6E-01	0.2
Slc35b2	1E-07	0.2	Atrnl1	8E-06	0.2
Acadsb	4E-10	0.2	Arid2	5E-04	0.2
Abt1	9E-05	0.2	Setd5	4E-09	0.2
Snhg4	5E-07	0.2	Kdm4a	6E-06	0.2
Hnrnpul2	5E-10	0.2	Prdm5	3E-06	0.2
Zfp513	4E-04	0.2	Slc39a9	1E-05	0.2
Pank1	5E-05	0.2	Mettl1	4E-10	0.2
Svbp	5E-11	0.2	Ppm1a	1E-07	0.2
Zfp12	1E-04	0.2	Uckl1	1E-04	0.2
Trps1	1E-12	0.2	Syncrip	2E-11	0.2
Eif5	6E-19	0.2	Csnk2a1	1E-13	0.2
Eid2	7E-06	0.2	Pbrm1	2E-12	0.2
Phospho2	4E-04	0.2	Armcx4	3E-03	0.2
Rnf215	4E-04	0.2	Prpf18	3E-05	0.2
Asb13	1E-03	0.2	Ftsj3	1E-05	0.2
Mex3c	1E-05	0.2	Rbm17	9E-08	0.2
Armcx4	5E-05	0.2	Sept11	2E-13	0.2
Tfrc	9E-07	0.2	Zc3h14	3E-06	0.2
Ergic1	4E-08	0.2	Cpne8	3E-10	0.2
Rsl1d1	5E-11	0.2	Zfp420	5E-02	0.2
Pafah1b1	7E-23	0.2	Efr3a	8E-06	0.2
Naa16	2E-06	0.2	Fam120c	1E-05	0.2
Trim45	5E-03	0.2	Prpsap1	3E-06	0.2
Arhgef5	1E-05	0.2	Clip4	3E-05	0.2
Wdr36	1E-06	0.2	Arl8b	4E-04	0.2
Ddx39b	2E-11	0.2	Tor3a	6E-04	0.2
Cmas	6E-08	0.2	5930430L01Rik	3E-05	0.2
Ccdc97	5E-05	0.2	Acbd5	7E-08	0.2
Det1	1E-04	0.2	Dbt	1E-05	0.2
Csnk1g3	8E-09	0.2	Def6	1E-03	0.2
Tnpo1	6E-07	0.2	B3gntl1	1E-03	0.2
Nmrk1	3E-04	0.2	Glg1	4E-13	0.2
Fxr2	1E-06	0.2	Usp5	6E-07	0.2
Rbm45	4E-05	0.2	Serp2	1E-04	0.2
Smim13	6E-06	0.2	Ywhae	1E-22	0.2
Zfp61	1E-03	0.2	Cdc37	2E-14	0.2

Chondrogenic

Control			NICD2		
Gene	*p*	FC	Gene	*p*	FC
Zfp119a	3E-01	0.2	1810034E14Rik	2E-01	0.2
Rab11fip2	1E-03	0.2	Bnip3	4E-07	0.2
Sdcbp	1E-10	0.2	Fbxl20	2E-03	0.2
Tdp2	2E-01	0.2	Hyal1	4E-01	0.2
Pls3	5E-08	0.2	Ecd	7E-01	0.2
Gtf2ird2	3E-01	0.2	Lypla1	1E-01	0.2
Rfc3	5E-02	0.2	C87436	2E-01	0.2
Stk17b	2E-01	0.2	Dgcr2	2E-02	0.2
Tars2	4E-01	0.2	Chid1	2E-03	0.2
Epc1	3E-04	0.2	Zc3h14	1E-05	0.2
Snx24	8E-05	0.2	Ggps1	8E-03	0.2
Rabl6	2E-06	0.2	Cdc14b	2E-01	0.2
Zfp825	5E-02	0.2	Pknox1	2E-01	0.2
C030034I22Rik	8E-01	0.2	Cpsf7	2E-01	0.2
Pex11g	3E-01	0.2	Slc7a1	6E-02	0.2
Suz12	1E-05	0.2	Ptpn3	1E+00	0.2
Pigt	3E-02	0.2	Ngly1	3E-02	0.2
Plscr1	7E-01	0.2	Kat6a	3E-04	0.2
Spin2c	3E-01	0.2	Ik	4E-06	0.2
Gm50013	5E-01	0.2	Emilin1	9E-04	0.2
Trim37	2E-03	0.2	Nbeal1	1E-04	0.2
Tbc1d8b	1E-02	0.2	Myo6	6E-02	0.2
Ndor1	2E-01	0.2	Arhgap31	3E-01	0.2
Kdm5b	3E-07	0.2	Gm26672	4E-01	0.2
Rasl11a	3E-01	0.2	Ogdh	1E-04	0.2
Hexdc	5E-01	0.2	Ikbip	4E-07	0.2
Pnkd	5E-10	0.2	Sh2b1	2E-01	0.2
Acox1	6E-06	0.2	Gss	4E-02	0.2
Shox2	5E-03	0.2	Hoxc9	2E-01	0.2
Klhl22	7E-04	0.2	Eif1	3E-18	0.2
D430042O09Rik	1E-02	0.2	Ikbke	9E-01	0.2
Rnf24	4E-03	0.2	2310039H08Rik	4E-03	0.2
AU040320	3E-04	0.2	D130040H23Rik	5E-01	0.2
Cisd2	3E-08	0.2	Spindoc	6E-02	0.2
Fbxo36	1E-02	0.2	Fam110a	9E-01	0.2
Ppt2	1E-01	0.2	Gm8739	5E-02	0.2
Ccdc191	1E-02	0.2	Zfp219	1E+00	0.2
Rab33b	2E-01	0.2	Birc2	6E-02	0.2
Slc35b1	2E-06	0.2	AI597479	8E-01	0.2
Hsd17b1	8E-01	0.2	Lipt2	7E-01	0.2
Trim33	1E-02	0.2	Zfp874b	3E-01	0.2
Cnnm2	1E-02	0.2	Huwe1	5E-09	0.2
Atmin	6E-01	0.2	Rab28	3E-05	0.2
Fem1c	5E-04	0.2	Rtp4	9E-01	0.2
Tmem260	3E-03	0.2	Spryd4	5E-01	0.2
Cox11	1E-01	0.2	AU040320	3E-02	0.2
Pex13	5E-08	0.2	Rnf14	1E-04	0.2
Ccni	2E-07	0.2	Cebpz	4E-05	0.2
Fam110a	8E-01	0.2	Fbxo46	5E-01	0.2
Nrd1	9E-10	0.2	Cwf19l2	4E-03	0.2
Vamp8	1E-08	0.2	Qsox1	7E-04	0.2
Zfp799	3E-02	0.2	Xylt2	9E-01	0.2
Smdt1	4E-23	0.2	Hspa4	4E-12	0.2
Dpp8	1E-04	0.2	Itm2c	4E-07	0.2
Elp2	2E-01	0.2	Gfm2	2E-01	0.2
Ccdc28a	1E-01	0.2	Ube2e1	3E-06	0.2
Slc12a9	1E+00	0.2	Eri3	2E-05	0.2
Banp	7E-03	0.2	Brcc3	2E-02	0.2
Optn	6E-02	0.2	Elmod3	9E-01	0.2
Trip4	1E-03	0.2	Wls	2E-11	0.2
Tmem248	5E-04	0.2	Exosc5	1E-02	0.2
P3h4	9E-09	0.2	Noa1	6E-01	0.2
Cbwd1	5E-02	0.2	Rpap3	5E-02	0.2
S100pbp	7E-02	0.2	Alkbh5	2E-03	0.2
Dgcr6	1E-07	0.2	Kif1bp	1E-01	0.2
Kif16b	4E-02	0.2	Socs6	3E-02	0.2
Zcchc14	5E-02	0.2	Fyttd1	4E-08	0.2
Tjap1	1E-02	0.2	Mex3a	1E-01	0.2
Zfp646	6E-02	0.2	Cdk7	1E-04	0.2
Rnaset2a	2E-02	0.2	Ash2l	5E-03	0.2
Tia1	8E-05	0.2	Tomm7	5E-15	0.2
Zbtb39	4E-01	0.2	Mtf1	1E-01	0.2
Nabp2	3E-05	0.2	R3hdm1	2E-04	0.2

Fibroblast

Control			NICD2		
Gene	*p*	FC	Gene	*p*	FC
			Rabif	2E-03	0.1
			Zcchc7	6E-05	0.1
			Ndst2	2E-01	0.1
			Lrp5	3E-01	0.1
			Rpl19	4E-44	0.1
			Ankrd17	1E-07	0.1
			Cdkl2	6E-01	0.1
			Gk5	8E-01	0.1
			Tle5	3E-14	0.1
			Ppif	6E-01	0.1
			Fbxl6	3E-02	0.1
			Gja1	9E-02	0.1
			Snapc1	5E-03	0.1
			Spata5	2E-01	0.1
			Nagk	9E-06	0.1
			Umad1	1E-01	0.1
			Sall2	3E-02	0.1
			Itpkb	2E-02	0.1
			Chpf2	3E-02	0.1
			Rps9	6E-39	0.1
			Fam120b	4E-03	0.1
			Ptpdc1	2E-01	0.1
			Glb1l	6E-02	0.1
			Fam168a	8E-05	0.1
			Ccdc107	2E-04	0.1
			Trp53rkb	6E-01	0.1
			Ift81	8E-02	0.1
			Mapkapk3	1E-01	0.1
			Eno2	5E-02	0.1
			Zfp810	3E-02	0.1
			Clptm1l	1E-05	0.1
			Tdrd7	2E-02	0.1
			Gorasp1	6E-01	0.1
			Rplp1	5E-38	0.1
			Gm10076	8E-18	0.1
			Tmem179b	3E-02	0.1
			Ganc	2E-02	0.1
			Tmem39a	4E-03	0.1
			Rffl	5E-01	0.1
			C1galt1c1	1E-01	0.1
			Scaper	3E-03	0.1
			Wipf1	4E-04	0.1
			Tle6	1E-01	0.1

Fibroblast?

Control			NICD2		
Gene	*p*	FC	Gene	*p*	FC
Piezo1	5E-22	0.4	Ap2a1	6E-27	0.4
Agfg1	3E-09	0.4	Unc5b	7E-11	0.4
B4galt6	3E-17	0.4	Itih5	5E-20	0.4
Tbc1d4	2E-15	0.4	Sec23b	4E-13	0.4
Hnrnpl	2E-26	0.4	Pkig	4E-24	0.4
Phactr4	1E-19	0.4	Dnajc3	2E-48	0.4
Msantd2	2E-14	0.4	Nhs	2E-22	0.4
Eogt	9E-13	0.4	Tnk2	3E-16	0.4
Pwwp2a	8E-14	0.4	Ptger4	3E-11	0.4
Fyn	9E-15	0.4	Ccm2	2E-21	0.4
Gm19605	2E-09	0.4	Zfp760	4E-16	0.4
Sf3b5	5E-47	0.4	Suco	3E-21	0.4
Esyt2	1E-20	0.4	Bcl2	2E-17	0.4
Pphln1	5E-14	0.4	Gramd1c	3E-10	0.4
Pdzd11	8E-15	0.4	Mrpl55	3E-23	0.4
Cenpv	5E-17	0.4	Gfm1	2E-17	0.4
Acot9	1E-12	0.4	Abhd10	3E-18	0.4
St3gal4	9E-17	0.4	Cavin3	2E-50	0.4
Gm12153	2E-07	0.4	Prkab1	2E-17	0.4
Rpl39l	2E-05	0.4	Prep	2E-15	0.4
Diaph1	2E-18	0.4	Poc1b	5E-20	0.4
Mphosph8	2E-22	0.4	Msantd2	1E-19	0.4
Snta1	2E-20	0.4	Timm22	1E-19	0.4
Adsl	2E-14	0.4	Pde6d	3E-26	0.4
Tmem26	6E-16	0.4	H19	1E-32	0.4
C030037D09Rik	1E-07	0.4	Tubb2a	2E-28	0.4
Usp54	3E-13	0.4	Tmem189	4E-18	0.4
Tulp4	2E-20	0.4	Crlf3	2E-21	0.4
Mid1ip1	3E-18	0.4	Cramp1l	3E-20	0.4
Eif1ax	2E-36	0.4	Cars2	3E-20	0.4
Cdc42ep4	1E-10	0.4	Bcar1	6E-16	0.4
Ttc39a	1E-08	0.4	Gemin8	1E-16	0.4
H2-Q7	5E-15	0.4	Rmdn3	2E-15	0.4
Tmem18	7E-17	0.4	Map4	5E-44	0.4
Gm19705	5E-09	0.4	Bola3	5E-43	0.4
Rpain	1E-16	0.4	Bak1	2E-20	0.4
Gm27241	5E-07	0.4	Prmt7	7E-16	0.4
Gm37982	3E-06	0.4	Gadd45g	1E-22	0.4
Vps36	2E-26	0.4	Mlycd	3E-10	0.4
Slc36a4	1E-12	0.4	Slc36a4	4E-16	0.4
Mtmr12	1E-15	0.4	Hnrnpdl	6E-31	0.4
Lrp4	3E-16	0.4	Smco4	2E-23	0.4
Smyd2	2E-14	0.4	Cst6	4E-10	0.4
Wdyhv1	2E-14	0.4	Cnot10	1E-20	0.4
Cpt1c	2E-14	0.4	Als2	8E-22	0.4
Trappc1	9E-14	0.4	P4ha3	1E-23	0.4
Nhsl2	5E-09	0.4	Myo1b	4E-23	0.4
Dpagt1	9E-15	0.4	Papss1	8E-23	0.4
Lyrm7	3E-07	0.4	Upf3b	1E-20	0.4
Fntb	5E-17	0.4	Cstf3	4E-22	0.4
Esyt1	1E-17	0.4	Eno3	2E-15	0.4
Smarca4	1E-20	0.4	Gpatch3	1E-05	0.4
Traf3ip1	7E-16	0.4	Ethe1	2E-14	0.4
Atf7ip	3E-13	0.4	Psmb8	2E-13	0.4
Pycr2	3E-16	0.4	Shisa4	2E-22	0.4
Ppp5c	3E-16	0.4	Med14	6E-20	0.4
Traf4	1E-12	0.4	Adss	4E-26	0.4
Mrpl3	4E-12	0.4	Ptpn14	5E-15	0.4
Stk11	3E-13	0.4	Ergic2	1E-32	0.4
Capg	1E-21	0.4	Epas1	4E-13	0.4
Iqck	2E-08	0.4	Mavs	3E-11	0.4
Dhrs9	9E-08	0.4	Sms	6E-25	0.4
Ino80e	1E-14	0.4	Smarca4	9E-28	0.4
Dmwd	3E-17	0.4	Phf10	3E-25	0.4
Rcbtb2	3E-10	0.4	Trmt10a	6E-19	0.4
Zfp568	2E-15	0.4	Ccdc9b	8E-06	0.4
Pqlc3	1E-13	0.4	Osbpl10	7E-15	0.4
Atp11c	1E-17	0.4	Dach2	3E-11	0.4
Fam126a	4E-18	0.4	Flrt3	2E-12	0.4
Plscr4	7E-18	0.4	Intu	2E-11	0.4
Trnau1ap	5E-15	0.4	Atp11c	6E-21	0.4
Lzic	4E-16	0.4	Pde8a	7E-24	0.4
Rpusd1	5E-12	0.4	Adgrl1	3E-17	0.4

Undefined

Control			NICD2		
Gene	*p*	FC	Gene	*p*	FC

Articular/Synovial Fibroblast

Control			NICD2		
Gene	*p*	FC	Gene	*p*	FC
Map2k3	5E-02	0.2	Naa10	9E-02	0.2
Mccc2	7E-01	0.2	Adat2	3E-01	0.2
Cnppd1	6E-01	0.2	Nacc2	6E-01	0.2
Gm1976	4E-01	0.2	Tmem176a	3E-03	0.2
Slc8a1	2E-03	0.2	Abhd11	7E-01	0.2
Tbk1	4E-01	0.2	Man2a1	2E-01	0.2
Babam2	6E-03	0.2	Gprasp2	6E-01	0.2
Nit2	5E-01	0.2	Atat1	1E+00	0.2
Slc35f5	3E-01	0.2	Comt	9E-04	0.2
Atg14	4E-01	0.2	Ist1	7E-01	0.2
Zfp949	7E-01	0.2	Map3k14	5E-01	0.2
Nudt2	3E-02	0.2	Gm10561	7E-01	0.2
Ccdc82	9E-01	0.2	Mcoln1	3E-01	0.2
Stxbp1	5E-01	0.2	Elf1	4E-01	0.2
Cul9	8E-01	0.2	Dhrs7b	7E-01	0.2
Proca1	9E-01	0.2	Cdk10	7E-01	0.2
Gm1673	3E-04	0.2	Sppl3	1E-01	0.2
Mrps18a	3E-03	0.2	Zfp277	8E-01	0.2
Slc44a1	1E-01	0.2	AI987944	3E-01	0.2
Rnf130	6E-02	0.2	Nmnat1	3E-01	0.2
Add1	2E-03	0.2	Foxo1	6E-01	0.2
Snap23	6E-02	0.2	Vcam1	9E-02	0.2
H6pd	5E-01	0.2	Bphl	2E-01	0.2
Man2c1os	5E-01	0.2	Pdxk	3E-01	0.2
Atl1	3E-01	0.2	Poglut3	2E-01	0.2
Ggnbp1	2E-01	0.2	Tbc1d9b	3E-01	0.2
5430405H02Rik	1E+00	0.2	Dennd2c	3E-01	0.2
Flt3l	9E-01	0.2	Hmox2	4E-02	0.2
Txnip	1E-02	0.2	Pigt	7E-01	0.2
Dnajb2	3E-01	0.2	Mfap2	3E-02	0.2
Smarcd2	4E-01	0.2	Soga1	9E-01	0.2
Nampt	1E-01	0.2	Zranb1	3E-01	0.2
Ap5m1	3E-01	0.2	Avl9	9E-01	0.2
Git1	9E-01	0.2	Esrra	4E-01	0.2
C3	4E-01	0.2	Coprs	4E-01	0.2
Lamp1	2E-12	0.2	Rpl37a	1E-22	0.2
Rpp21	3E-01	0.2	Filip1l	1E-02	0.2
Lasp1	2E-01	0.2	4930556M19Rik	1E-01	0.2
Phlda3	5E-04	0.2	Snx30	9E-01	0.2
Sgta	1E-02	0.2	Epm2a	6E-01	0.2
Zfp959	5E-01	0.2	Irf2	2E-01	0.2
Pex19	5E-01	0.2	Gpcpd1	5E-01	0.2
Fbxo38	4E-01	0.2	Fbxo30	2E-01	0.2
Lman2	8E-04	0.2	Taf7	5E-01	0.2
Zdhhc24	3E-01	0.2	Plau	4E-01	0.2
Immp2l	5E-01	0.2	Sdf2l1	7E-02	0.2
Rft1	5E-01	0.2	Pip4k2b	9E-01	0.2
Mov10	6E-01	0.2	Ghr	3E-05	0.2
Faap100	3E-01	0.2	Camta2	2E-01	0.2
Tusc2	9E-01	0.2	Vrk3	5E-01	0.2
Glud1	1E-04	0.2	Slc19a2	9E-01	0.2
Fndc4	9E-01	0.2	Rundc1	4E-01	0.2
Igf2bp1	4E-01	0.2	Aar2	7E-01	0.2
Sugp2	6E-01	0.2	Pip5k1a	3E-01	0.2
Washc1	7E-01	0.2	Fam172a	4E-03	0.2
Bmyc	3E-03	0.2	Pdia3	6E-07	0.2
Nedd1	1E+00	0.2	Gpm6b	2E-05	0.2
Ppt2	6E-01	0.2	Plagl2	1E-01	0.2
Cep97	2E-01	0.2	Poglut1	3E-01	0.2
Ppp1r18	3E-01	0.2	Ppp1r15b	1E+00	0.2
Fan1	1E+00	0.2	Arhgap18	8E-02	0.2
Tmem106c	9E-01	0.2	Dip2b	4E-02	0.2
Pou2f1	9E-01	0.2	Mvb12a	2E-01	0.2
Cdkl3	6E-01	0.2	Tbck	2E-01	0.2
Pfkm	4E-01	0.2	Togaram1	6E-01	0.2
Cenpv	5E-01	0.2	Rhog	5E-01	0.2
Uqcrh	1E-15	0.2	Ntan1	1E-02	0.2
Pogz	7E-01	0.2	Thap4	5E-01	0.2
Gm10941	7E-01	0.2	Calr	6E-06	0.2
Cpsf4	2E-01	0.2	Gnas	1E-03	0.2
Eid2b	4E-01	0.2	Tmem220	5E-01	0.2
Ccdc39	6E-01	0.2	Smarcal1	9E-01	0.2
Rps6ka3	2E-01	0.2	Brat1	6E-01	0.2

Limb Mesenchyme						Chondrogenic						Fibroblast						Fibroblast?						Undefined						Articular/Synovial Fibroblast					
Control			NICD2			Control			NICD2			Control			NICD2			Control			NICD2			Control			NICD2			Control			NICD2		
Gene	p	FC	Gene	p	FC	Gene	p	FC	Gene	p	FC	Gene	p	FC	Gene	p	FC	Gene	p	FC	Gene	p	FC	Gene	p	FC	Gene	p	FC	Gene	p	FC	Gene	p	FC
Styx	9E-05	0.2	Pcsk7	9E-03	0.2	Pigg	3E-01	0.2	Fat4	2E-02	0.2							Pcgf6	1E-14	0.4	Mtmr4	8E-15	0.4							Dph7	6E-01	0.2	A730081D07Rik	5E-01	0.2
Rnf6	3E-08	0.2	Kctd10	2E-04	0.2	Pou2f1	2E-02	0.2	Wdr19	3E-01	0.2							Senp2	5E-17	0.4	Actr1a	2E-16	0.4							Aebp2	2E-01	0.2	Ccdc30	7E-01	0.2
Jpt2	3E-05	0.2	Sun1	5E-05	0.2	Nob1	6E-02	0.2	Zfp974	7E-01	0.2							Steap2	4E-17	0.4	Ube2m	3E-46	0.4							Sspn	4E-01	0.2	Fkbp1b	4E-01	0.2
Arf1	3E-17	0.2	Itgb1	2E-22	0.2	Hist1h2bc	3E-14	0.2	Spg20	3E-04	0.2							Actr3	5E-31	0.4	Tuba1a	4E-47	0.4							Prpf39	4E-01	0.2	Srr	9E-01	0.2
BC005537	2E-07	0.2	Prpf31	5E-06	0.2	Aff4	5E-09	0.2	2810410L24Rik	4E-01	0.2							Nek7	7E-16	0.4	Xpo4	6E-21	0.4							Grasp	6E-01	0.2	Slk	2E-01	0.2
Bnip1	8E-05	0.2	Ikbip	2E-11	0.2	Erbb2	4E-01	0.2	Mnt	3E-01	0.2							Prelid1	2E-39	0.4	Ptges3l	3E-11	0.4							Grina	9E-03	0.2	Fam120b	3E-01	0.2
Grk4	5E-06	0.2	Ppp2r5e	3E-06	0.2	A930007I19Rik	6E-01	0.2	Slc11a2	6E-01	0.2							Igfbp2	2E-12	0.4	Cfl1	2E-58	0.4							Sowahc	5E-01	0.2	Elmod3	4E-01	0.2
Ipo11	7E-07	0.2	Surf2	7E-04	0.2	Ttll12	4E-01	0.2	Polr3gl	6E-02	0.2							Ptgis	7E-17	0.4	Bvht	4E-13	0.4							Stx4a	1E-02	0.2	Cda	5E-01	0.2
Nrbp1	3E-09	0.2	Arglu1	4E-12	0.2	Zfp14	1E-01	0.2	Nbdy	1E-09	0.2							Mtr	2E-14	0.4	Exosc1	2E-18	0.4							Ccdc171	9E-01	0.2	Pde4a	2E-01	0.2
Atg5	3E-07	0.2	Gpbp1l1	1E-05	0.2	Sclt1	2E-02	0.2	A930037H05Rik	5E-01	0.2							Stk32c	3E-07	0.4	Tmed8	2E-11	0.4							Mar8	7E-01	0.2	Crtc3	5E-01	0.2
Kpna6	2E-04	0.2	Senp6	5E-12	0.2	Ambra1	4E-05	0.2	Arel1	5E-01	0.2							Galnt2	1E-18	0.4	Simc1	5E-19	0.4							Fpgt	6E-01	0.2	Maf1	5E-01	0.2
BC034090	1E-03	0.2	Map1s	3E-04	0.2	Nop10	3E-13	0.2	Ankrd13c	2E-01	0.2							Agap3	5E-09	0.4	Ago2	6E-20	0.4							Suox	7E-01	0.2	Gm2000	2E-03	0.2
Tcp11l1	6E-04	0.2	Rbak	6E-04	0.2	Apex2	8E-03	0.2	Ccl25	3E-01	0.2							Clip4	7E-16	0.4	Gpn1	1E-12	0.4							Nid2	1E-01	0.2	Fbxl12os	7E-01	0.2
Trappc4	2E-11	0.2	Rusc2	3E-05	0.2	Cdkn1b	2E-05	0.2	Paip1	8E-05	0.2							Pak1	3E-13	0.4	Ccdc51	1E-18	0.4							Pram1	7E-01	0.2	Sept1	3E-01	0.2
Slc19a1	7E-04	0.2	Ptdss1	8E-05	0.2	Abcc10	6E-01	0.2	2700097O09Rik	7E-01	0.2							Ankrd9	1E-18	0.4	Anapc1	6E-21	0.4							Mthfsl	6E-01	0.2	Tpgs1	9E-02	0.2
Bdp1	1E-07	0.2	Palld	4E-08	0.2	D330023K18Rik	1E-01	0.2	Cdkn2aip	8E-01	0.2							Rnps1	3E-21	0.4	Snrpd2	4E-54	0.4							Nudt18	7E-01	0.2	Chkb	6E-01	0.2
Utp20	1E-06	0.2	Arl8a	8E-08	0.2	Ccny	2E-07	0.2	AW011738	6E-01	0.2							Adgrl1	3E-16	0.4	Slc35d1	4E-20	0.4							Atp6v1h	3E-01	0.2	Calm3	2E-03	0.2
Tspyl1	2E-08	0.2	Dicer1	3E-05	0.2	Mettl1	5E-05	0.2	Ddx46	2E-04	0.2							Kat14	2E-14	0.4	Tomm40	2E-19	0.4							Ptges3l	4E-01	0.2	Gm9993	4E-01	0.2
Sec24a	2E-04	0.2	Zfp867	4E-03	0.2	Etfa	7E-10	0.2	Slc35b1	4E-03	0.2							Pakap	2E-19	0.4	Abcb7	2E-22	0.4							Polg2	2E-01	0.2	Cep162	5E-01	0.2
Eno3	2E-04	0.2	Spata1	1E-04	0.2	Ubxn6	1E-08	0.2	Pecr	4E-01	0.2							Tmem45a	2E-19	0.4	Limk1	5E-15	0.4							Tigd2	9E-01	0.2	Ccdc73	4E-01	0.2
Polr1e	5E-04	0.2	Fam53b	7E-05	0.2	Rbm48	1E-01	0.2	Pebp1	2E-17	0.2							Snrpg	3E-70	0.4	Brd8	4E-24	0.4							Fam50a	7E-03	0.2	Prxl2c	5E-02	0.2
Fggy	3E-06	0.2	Psmd1	3E-13	0.2	Pde4d	1E-02	0.2	Ccdc9	2E-01	0.2							Nectin3	9E-18	0.4	Timm23	5E-39	0.4							Nt5dc1	9E-01	0.2	Ctnnbl1	9E-01	0.2
Smarca5	4E-15	0.2	Lrrc57	7E-03	0.2	Slc9a8	4E-01	0.2	Hp1bp3	2E-12	0.2							Acer3	7E-13	0.4	Plscr1	6E-18	0.4							Mgat5	9E-01	0.2	Pnpla7	4E-01	0.2
Aasdh	7E-04	0.2	Hoxc8	3E-05	0.2	Gm38604	9E-01	0.2	Klhdc8b	7E-01	0.2							Akt1	4E-17	0.4	Edem1	2E-16	0.4							Prrg2	7E-01	0.2	Abhd14a	4E-01	0.2
Mkrn2	6E-05	0.2	Arpc5l	5E-13	0.2	Snd1	2E-07	0.2	Rbm27	5E-03	0.2							Atxn2l	6E-12	0.4	Erc1	1E-26	0.4							Uqcc3	5E-04	0.2	Zfp930	4E-01	0.2
Mtmr2	7E-06	0.2	Vps37b	7E-04	0.2	Tbce	9E-03	0.2	Med26	6E-01	0.2							Clpb	8E-16	0.4	Zfp472	1E-12	0.4							F8a	7E-01	0.2	Gm34455	8E-02	0.2
Aatf	1E-06	0.2	1700029J07Rik	9E-03	0.2	Dnajc1	9E-10	0.2	Zfp787	5E-01	0.2							Ythdc2	3E-13	0.4	Ap2m1	6E-41	0.4							Cpt1c	2E-01	0.2	2810013P06Rik	7E-01	0.2
mt-Nd6	3E-06	0.2	Stk38l	1E-07	0.2	Alkbh2	1E-01	0.2	Lyst	1E-01	0.2							Rac1	9E-33	0.4	Dvl2	1E-16	0.4							Ptprf	7E-01	0.2	Iqcb1	1E+00	0.2
Gnl3	3E-08	0.2	Spred3	1E-02	0.2	Dop1a	2E-02	0.2	Cep63	7E-02	0.2							Bola3	2E-33	0.4	Pias1	5E-13	0.4							Zfp365	5E-01	0.2	Uhmk1	1E-01	0.2
Papola	1E-14	0.2	Isoc1	3E-04	0.2	Harbi1	2E-01	0.2	Glrx2	1E-06	0.2							Dok1	7E-13	0.4	Bzw1	1E-46	0.4							Zfr2	6E-01	0.2	Wbp2	9E-01	0.2
Nt5dc1	3E-05	0.2	Glcci1	4E-04	0.2	Thap3	5E-04	0.2	Mbd2	4E-05	0.2							Vkorc1l1	2E-17	0.4	Tmod3	6E-27	0.4							Tdrd7	8E-01	0.2	Eif3k	1E-05	0.2
Ppp6r2	1E-05	0.2	Sltm	2E-07	0.2	Psenen	9E-14	0.2	Stk19	3E-02	0.2							Tdp1	5E-13	0.4	Sema3c	1E-17	0.4							Dennd1a	3E-03	0.2	Rps28	1E-18	0.2
Pfn2	3E-06	0.2	Hoxc9	3E-04	0.2	Inpp4a	3E-01	0.2	Cuedc1	1E-02	0.2							Pnkp	2E-15	0.4	Cox10	2E-19	0.4							Mnt	4E-01	0.2	Dmac2	4E-01	0.2
Crls1	9E-07	0.2	Kansl1l	1E-07	0.2	4833420G17Rik	1E-04	0.2	Gm20275	2E-01	0.2							Slc38a4	6E-13	0.4	Pop1	2E-11	0.4							Sco2	5E-01	0.2	Rit1	9E-01	0.2
Adamtsl1	5E-13	0.2	Tom1l1	2E-02	0.2	Hdlbp	6E-24	0.2	Prorp	5E-01	0.2							Mrpl33	1E-52	0.4	Serhl	5E-18	0.4							Focad	7E-01	0.2	Snupn	5E-01	0.2
B4galt6	9E-05	0.2	Prkab2	1E-03	0.2	Hk2	8E-02	0.2	Atxn3	1E-01	0.2							Grik1	7E-05	0.4	Stxbp1	3E-23	0.4							Fgd1	3E-01	0.2	Ttll1	3E-01	0.2
Gmcl1	4E-05	0.2	Caskin2	9E-03	0.2	Rnf113a2	2E-02	0.2	Phkg2	5E-01	0.2							Zfp770	1E-05	0.4	Gm9887	8E-10	0.4							4921504A21Rik	3E-01	0.2	Saysd1	6E-02	0.2
Tsc22d1	2E-13	0.2	Pcnx3	2E-05	0.2	Pnrc1	4E-10	0.2	Tm9sf2	8E-09	0.2							Sec14l1	6E-16	0.4	Dynll1	2E-27	0.4							Trim32	7E-01	0.2	Klhl2	5E-01	0.2
Rbm28	9E-10	0.2	Ptpn1	4E-08	0.2	Arl2bp	5E-06	0.2	Fbxw5	6E-01	0.2							Suco	2E-16	0.4	Ilf3	2E-21	0.4							Flot1	3E-02	0.2	Sf3b2	2E-04	0.2
Smad5	1E-05	0.2	Hoxa11	1E-02	0.2	Pts	2E-04	0.2	Mterf3	6E-02	0.2							Il17rc	8E-16	0.4	Pxk	3E-19	0.4							2310022B05Rik	3E-01	0.2	Med25	6E-01	0.2
Hoxa3	3E-04	0.2	Det1	6E-03	0.2	Mbd6	6E-02	0.2	Wdr37	2E-01	0.2							Epha7	8E-09	0.4	Pdlim7	2E-12	0.4							Bad	3E-05	0.2	Zfp740	6E-01	0.2
Atxn7l3	3E-06	0.2	Bivm	4E-03	0.2	Larp4b	2E-05	0.2	Wsb1	1E-01	0.2							Serbp1	1E-60	0.4	Txnl4a	1E-26	0.4							Nlgn2	5E-01	0.2	Itpkb	6E-01	0.2
Axin1	1E-05	0.2	Nek9	3E-05	0.2	Gas5	5E-07	0.2	Usp36	6E-01	0.2							Thoc7	8E-37	0.4	Gne	4E-11	0.4							Inpp5k	8E-01	0.2	Ankle2	9E-01	0.2
Map2k2	6E-12	0.2	Wdr74	2E-06	0.2	Stk19	6E-03	0.2	Ttc4	6E-03	0.2							Wsb2	2E-14	0.4	Gm49959	7E-16	0.4							Wdr45	9E-01	0.2	Ypel1	4E-01	0.2
Prorsd1	3E-08	0.2	Hmgn3	2E-11	0.2	Prkag1	2E-02	0.2	Rnf13	2E-04	0.2							Isy1	5E-18	0.4	Tubb3	1E-20	0.4							Dtd2	6E-01	0.2	Stambp	7E-01	0.2
Timp3	1E-13	0.2	Kif3c	2E-04	0.2	Foxj3	6E-03	0.2	Ankfy1	5E-03	0.2							Lacc1	3E-17	0.4	Cct6a	2E-32	0.4							Gmeb1	3E-01	0.2	Acot11	7E-01	0.2
Arhgef1	1E-05	0.2	Lman2l	2E-04	0.2	Brf1	3E-01	0.2	Rnf138	2E-02	0.2							Tsen2	2E-09	0.4	Slc35b2	3E-11	0.4							Dzip1	1E-01	0.2	Lamc1	8E-02	0.2
Zcchc7	2E-08	0.2	Slc18b1	3E-05	0.2	Fxr1	2E-10	0.2	Kif13a	2E-02	0.2							Bola2	2E-27	0.4	Nanp	8E-21	0.4							Qrich1	2E-01	0.2	Phf13	9E-01	0.2
Dnajc30	2E-05	0.2	Unk	1E-02	0.2	2200002D01Rik	6E-02	0.2	Chchd7	2E-03	0.2							Mical3	3E-18	0.4	Prdx1	3E-51	0.4							Scand1	5E-08	0.2	Rps6ka3	2E-01	0.2
Tomm70a	1E-07	0.2	Inpp4a	1E-03	0.2	Mmgt2	1E-02	0.2	Utp4	1E-01	0.2							Mavs	2E-12	0.4	Rnps1	3E-26	0.4							Limd1	2E-01	0.2	Ccndbp1	9E-01	0.2
Lmcd1	7E-06	0.2	Chpf2	2E-04	0.2	Fam219b	2E-01	0.2	Dcaf17	4E-01	0.2							Coq6	6E-16	0.4	LTO1	9E-12	0.4							Ndufa4l2	2E-01	0.2	Etv5	4E-01	0.2
Pja2	2E-09	0.2	2900076A07Rik	3E-03	0.2	Prpf6	3E-06	0.2	Leprotl1	1E-04	0.2							Spin2c	2E-02	0.4	Rtn3	4E-38	0.4							Spata33	8E-01	0.2	Phpt1	5E-01	0.2
Rxrb	4E-06	0.2	Gt(ROSA)26Sor	6E-05	0.2	Kbtbd4	2E-01	0.2	Poglut2	4E-03	0.2							Retreg2	8E-14	0.4	Pld2	1E-19	0.4							Zfp120	5E-01	0.2	Isg15	6E-01	0.2
Nus1	6E-06	0.2	Ccdc186	2E-06	0.2	Zfp740	1E-01	0.2	Efcab14	5E-03	0.2							Ncln	6E-09	0.4	Stard6	2E-13	0.4							Pex1	9E-01	0.2	Fam171a2	9E-01	0.2
Prkcsh	2E-09	0.2	Uimc1	7E-06	0.2	Edem2	2E-01	0.2	Gmfb	3E-02	0.2							Gm20528	4E-08	0.4	Plekhg2	1E-20	0.4							Kmt5c	5E-01	0.2	Churc1	5E-03	0.2
Ppp6r1	2E-05	0.2	Dr1	2E-05	0.2	Gsk3b	2E-08	0.2	Nxt2	5E-01	0.2							Plekho2	7E-11	0.4	Yif1b	2E-27	0.4							2700046G09Rik	5E-01	0.2	Dguok	4E-01	0.2
Gnb1	1E-15	0.2	Appbp2	3E-06	0.2	Foxk2	6E-02	0.2	Ndufaf4	7E-03	0.2							Slfn2	1E-03	0.4	Prkaca	3E-20	0.4							Ddt	7E-04	0.2	Tnrc6c	5E-03	0.2
Ccdc107	5E-11	0.2	Med27	3E-05	0.2	Atr	8E-02	0.2	Nfkbib	6E-01	0.2							Scaf11	5E-31	0.4	Grem2	8E-10	0.4							Zkscan3	1E-01	0.2	Zfp90	4E-01	0.2
Hcfc1	3E-05	0.2	Poldip2	3E-05	0.2	Fyttd1	1E-08	0.2	Chmp1b	3E-02	0.2							Akr1b3	3E-17	0.4	Klf11	2E-12	0.4							Ankrd13b	8E-01	0.2	Cd82	8E-01	0.2
Ankfy1	9E-07	0.2	Bbx	3E-08	0.2	Tmem141	4E-03	0.2	Mnat1	4E-06	0.2							Prkab1	6E-13	0.4	Col4a1	4E-20	0.4							Emc10	8E-05	0.2	Clk4	9E-01	0.2
Tiparp	1E-06	0.2	Eloa	5E-06	0.2	1810034E14Rik	5E-01	0.2	Zcchc14	2E-01	0.2							Ar	2E-13	0.4	Cnot1	1E-20	0.4							Synj2	7E-01	0.2	Hs2st1	3E-01	0.2
Kif13b	8E-06	0.2	Tmsb4x	2E-14	0.2	Coq10b	7E-03	0.2	Ss18	1E-01	0.2							Cnst	8E-09	0.4	Psmd14	9E-32	0.4							Eif4b	1E-02	0.2	Cdkn2b	4E-02	0.2
Ubiad1	9E-03	0.2	Orc2	6E-07	0.2	Lipt1	2E-01	0.2	Smim7	9E-05	0.2							Crtc2	2E-10	0.4	Gm14167	2E-09	0.4							mt-Atp8	2E-01	0.2	Nicn1	7E-01	0.2
Tmem126a	2E-12	0.2	Smg6	3E-06	0.2	St3gal3	1E-02	0.2	Pnpla6	3E-01	0.2							Phf10	2E-17	0.4	Fmr1	5E-41	0.4							Dhx32	6E-01	0.2	Scly	3E-01	0.2
Akap1	2E-04	0.2	Zfp27	3E-02	0.2	Fndc3a	3E-06	0.2	Pigq	3E-01	0.2							Ogfrl1	4E-19	0.4	Bmp2k	1E-20	0.4							Gm11266	9E-01	0.2	Gm14325	3E-01	0.2
Prdm16	3E-05	0.2	Rrbp1	1E-21	0.2	Rbm27	1E-04	0.2	Sipa1l1	5E-08	0.2							Tgfbr1	3E-14	0.4	Kat7	8E-18	0.4							Pigm	4E-01	0.2	Angpt2	5E-01	0.2
Crebzf	1E-04	0.2	Gon4l	6E-04	0.2	Ubr4	3E-03	0.2	Ogfod2	3E-01	0.2							Etl4	1E-19	0.4	Txndc12	5E-26	0.4							Pias1	9E-02	0.2	Dolk	3E-01	0.2
Krtcap2	2E-20	0.2	Metap2	2E-17	0.2	4933434E20Rik	6E-04	0.2	Clp1	6E-01	0.2							Pde1c	4E-06	0.4	Ssh2	3E-26	0.4							Ppp6r3	8E-01	0.2	Ermard	3E-01	0.2
Arnt2	8E-04	0.2	Ppp2r2a	1E-06	0.2	Tbp	1E-01	0.2	Foxd2os	2E-01	0.2							Rnft2	1E-07	0.4	Vdac3	1E-46	0.4							Frg2f1	3E-01	0.2	Nhej1	8E-02	0.2
Slc20a2	2E-05	0.2	Zfp81	1E-03	0.2	Bsdc1	1E-01	0.2	9130008F23Rik	6E-01	0.2							Csgalnact2	6E-15	0.4	Fkbp3	3E-42	0.4							Ypel1	8E-01	0.2	Bud13	3E-01	0.2
Rnf166	1E-07	0.2	Xpo5	9E-06	0.2	Gabbr1	3E-02	0.2	Aifm1	3E-03	0.2							BC049715	1E-06	0.4	Rnf8	4E-12	0.4							Flot2	5E-01	0.2	Zfp955b	6E-01	0.2
Agrn	2E-06	0.2	Ranbp2	8E-06	0.2	Pan2	8E-01	0.2	Trip4	5E-03	0.2							Get4	2E-16	0.4	Hcfc2	3E-15	0.4							Tor1b	2E-02	0.2	Nenf	3E-06	0.2
Clba1	2E-04	0.2	Arhgef3	3E-05	0.2	Nfia	4E-13	0.2	Scarb1	5E-01	0.2							Sertad1	7E-09	0.4	Zfp13	3E-10	0.4							Ppp2r1a	1E-01	0.2	Fau	5E-18	0.2

Limb Mesenchyme					
Control			NICD2		
Gene	*p*	FC	Gene	*p*	FC
Tmem209	3E-03	0.2	Zmynd11	7E-09	0.2
Dctd	9E-05	0.2	Psme4	1E-10	0.2
Rusc2	4E-05	0.2	Gm16541	7E-04	0.2
Rspry1	3E-06	0.2	Ccdc43	1E-04	0.2
Tmem258	2E-18	0.2	Fermt2	2E-07	0.2
Chst7	1E-01	0.2	Dhx35	1E-03	0.2
Mdfi	3E-04	0.2	Ddx50	2E-13	0.2
Mnat1	1E-09	0.2	Cnst	6E-02	0.2
Ecpas	1E-08	0.2	Zfp335	7E-03	0.2
Coro1c	2E-06	0.2	Tent5a	4E-05	0.2
Frs2	5E-07	0.2	Hltf	2E-05	0.2
Kif2a	8E-09	0.2	Hmgxb3	7E-05	0.2
Myo1c	5E-05	0.2	Bcl2l1	4E-06	0.2
Stx16	9E-08	0.2	Vps4a	7E-06	0.2
Gm32856	8E-03	0.2	Tbcel	2E-05	0.2
Tomm20	1E-16	0.2	Gss	1E-04	0.2
Jcad	1E-05	0.2	Mrpl39	4E-04	0.2
Zfp26	2E-05	0.2	Spcs2	2E-12	0.2
Fbxo44	2E-03	0.2	Fam114a2	1E-06	0.2
Sec61a1	2E-07	0.2	St7l	2E-06	0.2
Hoxc10	3E-09	0.2	Sec13	1E-10	0.2
Rcl1	4E-05	0.2	Atad3a	3E-03	0.2
1600010M07Rik	2E-06	0.2	Eif1	5E-33	0.2
Galnt1	1E-06	0.2	Dynlt1a	9E-01	0.2
Ube3a	1E-11	0.2	Pafah1b3	7E-07	0.2
Tmub2	2E-05	0.2	Usp32	5E-06	0.2
Zc3h12b	9E-07	0.2	Dnajc16	8E-03	0.2
Unc50	8E-09	0.2	Limd1	4E-06	0.2
Fubp3	5E-07	0.2	Zc3h18	2E-07	0.2
Heatr5a	3E-06	0.2	Tiprl	4E-08	0.2
Eif3a	3E-16	0.2	Kctd13	2E-03	0.2
Foxk2	2E-05	0.2	Zfp668	6E-02	0.2
Ints13	9E-06	0.2	Bpnt1	2E-02	0.2
Mir22hg	3E-07	0.2	Srsf9	2E-11	0.2
Dlst	4E-07	0.2	Kmt2d	8E-06	0.2
Zfp384	8E-06	0.2	Prkd1	4E-06	0.2
Usp10	1E-06	0.2	Cd276	2E-04	0.2
Gpcpd1	1E-06	0.2	Dhx30	7E-06	0.2
Fastk	8E-07	0.2	Heatr5a	2E-04	0.2
Amn1	1E-05	0.2	Lrp12	8E-05	0.2
Prmt5	6E-05	0.2	Trnt1	1E-05	0.2
Ranbp10	6E-06	0.2	Canx	2E-15	0.2
Phldb2	2E-06	0.2	Lsm12	3E-11	0.2
Osbpl9	3E-10	0.2	Usp47	2E-05	0.2
Pgs1	2E-05	0.2	Stoml1	1E-04	0.2
Wac	5E-11	0.2	Snhg8	1E-08	0.2
Lcor	4E-06	0.2	Pcnp	2E-09	0.2
Rbak	4E-04	0.2	Rexo4	5E-07	0.2
Zfp60	2E-04	0.2	Dym	1E-04	0.2
Zfp112	2E-04	0.2	Sar1b	7E-08	0.2
Nfkb1	2E-06	0.2	Galnt1	2E-06	0.2
Ets2	3E-07	0.2	Ddx27	2E-06	0.2
Ehmt1	2E-08	0.2	Mrpl21	3E-04	0.2
Ubr3	8E-08	0.2	Faxc	9E-03	0.2
Vapa	5E-15	0.2	Hnrnpr	6E-12	0.2
Cnot2	1E-06	0.2	Mapre3	3E-05	0.2
E2f1	2E-04	0.2	AI597479	4E-05	0.2
Cbx3	5E-15	0.2	Ripk2	8E-05	0.2
Atl3	3E-09	0.2	Vangl2	4E-04	0.2
Clasp1	1E-05	0.2	Plekha1	1E-04	0.2
Ncoa6	2E-05	0.2	Ubap2	9E-07	0.2
Trmt10a	1E-04	0.2	Cnot2	3E-05	0.2
Wdr26	3E-08	0.2	Txnrd1	9E-09	0.2
Ccdc149	4E-03	0.2	Fam160b1	1E-05	0.2
Cryzl2	9E-11	0.2	Tmsb10	2E-14	0.2
St3gal4	7E-09	0.2	Nufip2	5E-08	0.2
Wdr46	2E-04	0.2	Sppl2b	2E-02	0.2
Dcun1d5	4E-11	0.2	Nhlrc3	3E-03	0.2
1110004F10Rik	2E-16	0.2	Zfp84	8E-04	0.2
Usp34	5E-13	0.2	Rpn1	9E-10	0.2
Hmg20a	4E-07	0.2	Tmem161b	1E-04	0.2
Anapc1	2E-06	0.2	Strap	9E-08	0.2
Clptm1l	3E-08	0.2	Copg1	5E-06	0.2

Chondrogenic					
Control			NICD2		
Gene	*p*	FC	Gene	*p*	FC
Ep400	1E-03	0.2	Dus4l	5E-01	0.2
Amn1	7E-03	0.2	Arl5b	3E-01	0.2
Cep19	2E-01	0.2	Zmynd11	2E-06	0.2
Rab5a	8E-08	0.2	Akap8l	1E-04	0.2
Pygo2	7E-01	0.2	Cdkn1b	6E-04	0.2
Armc5	3E-01	0.2	Ate1	8E-04	0.2
Nr2c2	1E-02	0.2	Flt3l	8E-01	0.2
Id4	3E-03	0.2	Alkbh1	4E-02	0.2
Mical1	3E-01	0.2	Pex2	9E-03	0.2
Asb8	3E-02	0.2	Rnpc3	3E-02	0.2
Ago1	3E-02	0.2	Pnkp	5E-01	0.2
B230354K17Rik	1E-01	0.2	Xist	4E-01	0.2
B4galt7	3E-01	0.2	Eif2ak3	2E-01	0.2
Brat1	2E-01	0.2	Mettl4	9E-01	0.2
Nrde2	9E-02	0.2	Edc3	4E-01	0.2
Ccdc84	8E-01	0.2	Mcee	1E-04	0.2
Pus3	4E-01	0.2	Sipa1l3	4E-01	0.2
Golgb1	7E-17	0.2	Txnrd2	9E-01	0.2
Mlst8	5E-01	0.2	Bloc1s3	8E-01	0.2
Fxr2	5E-02	0.2	Rap2c	1E-01	0.2
Slmap	2E-04	0.2	Polr2g	2E-04	0.2
Bcl7a	5E-01	0.2	Slc35c2	9E-02	0.2
Tmem38b	9E-02	0.2	2610021A01Rik	7E-01	0.2
Cryab	4E-22	0.2	Senp7	1E-04	0.2
Vbp1	2E-04	0.2	Lekr1	9E-01	0.2
Hdac9	1E-02	0.2	Dcaf5	1E-01	0.2
Dhx16	8E-02	0.2	Foxk2	3E-01	0.2
Anapc4	2E-04	0.2	Zmym2	4E-03	0.2
Lcorl	2E-05	0.2	2900097C17Rik	3E-04	0.2
Dcps	3E-02	0.2	Aftph	4E-02	0.2
Rad23a	1E-04	0.2	Ddx24	3E-09	0.2
Gm10516	3E-01	0.2	Ptges3l	9E-01	0.2
Rbm15b	1E-01	0.2	Ndfip2	2E-02	0.2
Coro7	2E-01	0.2	Wapl	2E-05	0.2
Mr1	6E-01	0.2	Exoc6b	5E-05	0.2
Vps13c	5E-07	0.2	Slc25a14	9E-01	0.2
Hs2st1	4E-04	0.2	Gtf3c4	3E-01	0.2
Gle1	3E-02	0.2	Ergic1	7E-07	0.2
Ebna1bp2	2E-07	0.2	Usp32	8E-03	0.2
Trmt10b	2E-01	0.2	Lamtor5	1E-07	0.2
Sgcb	1E-04	0.2	Zfp280c	9E-02	0.2
Ralgapb	3E-02	0.2	Impa2	5E-01	0.2
Kdm1b	7E-01	0.2	Zdhhc16	7E-01	0.2
Zfp438	4E-01	0.2	Ccdc114	4E-01	0.2
Cby1	4E-05	0.2	Rpl7l1	9E-05	0.2
Tm2d2	8E-12	0.2	Kansl2	2E-02	0.2
Emb	2E-09	0.2	Dhx40	7E-04	0.2
Cyb5r1	1E-02	0.2	Dhx36	3E-08	0.2
Coq4	5E-01	0.2	Otud3	8E-01	0.2
Tab1	1E+00	0.2	Abi3bp	2E-09	0.2
Tdg	1E-02	0.2	Sbno1	3E-04	0.2
Mapk8ip1	5E-01	0.2	Camta2	4E-01	0.2
Clpb	4E-02	0.2	Ddhd1	2E-01	0.2
Mrpl24	1E-08	0.2	Plagl2	1E+00	0.2
Ralgapa1	9E-06	0.2	Epc1	6E-04	0.2
Ufl1	1E-03	0.2	Kptn	4E-01	0.2
Gm37494	3E-03	0.2	H13	2E-05	0.2
Spout1	4E-01	0.2	Hpgd	2E-01	0.2
Ptprg	1E-04	0.2	Ube2g1	2E-03	0.2
Trappc2	3E-02	0.2	Chchd4	7E-01	0.2
Cdk2ap2	8E-06	0.2	Mpzl1	5E-01	0.2
Nbeal1	2E-06	0.2	Fem1c	1E-02	0.2
Pxdn	1E-02	0.2	Atp11b	6E-02	0.2
Foxo1	6E-03	0.2	N4bp2l2	1E-06	0.2
Akap8l	2E-04	0.2	Dad1	3E-15	0.2
Kcnrg	2E-01	0.2	Ccdc107	9E-06	0.2
Fosb	6E-04	0.2	Ascc1	8E-02	0.2
Sepsecs	7E-02	0.2	Tapbpl	4E-01	0.2
Irak1	7E-03	0.2	Wdr48	9E-02	0.2
Brsk1	4E-01	0.2	Zc3h3	9E-01	0.2
B130055M24Rik	6E-02	0.2	Ier5	5E-01	0.2
Tcaim	7E-02	0.2	Rnf214	3E-03	0.2
Prpf8	6E-06	0.2	Ajuba	9E-01	0.2

Fibroblast					
Control			NICD2		
Gene	*p*	FC	Gene	*p*	FC

Fibroblast?					
Control			NICD2		
Gene	*p*	FC	Gene	*p*	FC
Thap1	3E-13	0.4	Ephb2	8E-16	0.4
Ccdc122	1E-15	0.4	Usp28	3E-14	0.4
Sh3rf1	2E-19	0.4	Snrnp70	2E-36	0.4
Ercc6l2	4E-14	0.4	Gtpbp1	1E-16	0.4
Heca	1E-16	0.4	Gorab	3E-17	0.4
Nxn	7E-14	0.4	Ptpa	4E-14	0.4
F3	8E-11	0.4	Csgalnact2	1E-15	0.4
Mosmo	1E-16	0.4	Traf7	3E-14	0.4
Ldlrad4	5E-21	0.4	Ctbp1	6E-21	0.4
Cox7a2	2E-63	0.4	Hnrnpl	8E-25	0.4
Adamtsl5	4E-14	0.4	Zbtb22	4E-08	0.4
Cers5	2E-13	0.4	Gli2	4E-16	0.4
Ttll12	2E-11	0.4	Poll	2E-11	0.4
Cers4	1E-15	0.4	Kri1	3E-09	0.4
Gm13056	2E-14	0.4	Hdac2	1E-21	0.4
Cdc42ep1	1E-09	0.4	4921524J17Rik	1E-25	0.4
Rtn3	1E-28	0.4	Taf3	3E-19	0.4
Slc8a1	3E-22	0.4	Kpna4	7E-27	0.4
Ilf3	3E-13	0.4	Enoph1	1E-12	0.4
Bloc1s2	1E-15	0.4	Gm41724	2E-13	0.4
Rbm48	3E-12	0.4	Zc3h4	9E-21	0.4
Nudt22	6E-17	0.4	Arnt2	6E-17	0.4
Mtf1	8E-06	0.4	Ppp6c	1E-15	0.4
Rbm17	2E-20	0.4	Rftn1	9E-10	0.4
Traf5	7E-10	0.4	Homer3	6E-18	0.4
Eif4e	7E-21	0.4	Lmo7	2E-25	0.4
Raver2	3E-11	0.4	Zbtb46	1E-08	0.4
Klhl25	2E-02	0.4	Uqcr10	2E-72	0.4
Pcx	3E-27	0.4	Tpm1	2E-29	0.4
Utp23	4E-17	0.4	Eif1ax	1E-44	0.4
Cadm2	7E-09	0.4	Wdr91	5E-07	0.4
Gabpb1	6E-16	0.4	Gsk3a	1E-16	0.4
Ahsa1	5E-16	0.4	Steap2	3E-21	0.4
Cast	8E-25	0.4	Dgkz	4E-17	0.4
Hrct1	2E-11	0.4	Cab39l	2E-23	0.4
Calcrl	8E-05	0.4	Csnk1e	2E-15	0.4
Mlst8	2E-04	0.4	Rer1	1E-49	0.4
4930412C18Rik	4E-04	0.4	Tspan18	6E-15	0.4
Dcaf7	1E-11	0.4	Lgals1	2E-77	0.4
1500011B03Rik	1E-13	0.4	Exosc10	1E-18	0.4
Plekhg2	2E-14	0.4	Get4	2E-21	0.4
Sdc3	3E-16	0.4	Lrrc45	3E-18	0.4
Zfp422	8E-09	0.4	P2ry10b	5E-16	0.4
Kcnip3	9E-07	0.4	Farp2	5E-17	0.4
Cep131	3E-12	0.4	Hira	8E-21	0.4
Alas2	1E-04	0.4	Wnt4	8E-12	0.4
Gpsm2	1E-18	0.4	Selenoi	3E-20	0.4
Tnpo2	8E-13	0.4	Npm3	4E-18	0.4
Col28a1	1E-07	0.4	Tceal9	4E-58	0.4
Pi4k2b	9E-16	0.4	Ap1g2	3E-08	0.4
Bzw1	3E-30	0.4	Pak1	2E-15	0.4
Ptpn11	4E-19	0.4	Ankrd52	4E-16	0.4
Prkaca	2E-14	0.4	Uchl3	2E-19	0.4
Sema4b	1E-09	0.4	Bclaf3	3E-21	0.4
Pdap1	3E-35	0.4	Tspan2	9E-19	0.4
Cdc34	2E-19	0.4	Tec	2E-14	0.4
Gtf2ird1	4E-13	0.4	Rhoc	7E-42	0.4
Cdyl	1E-13	0.4	Ppp1r12c	5E-14	0.4
Olfm1	8E-19	0.4	Aldh5a1	1E-08	0.4
Fam20b	1E-15	0.4	Gm15675	1E-08	0.4
Chd1	2E-15	0.4	Cers5	3E-21	0.4
Dpp9	3E-12	0.4	Setx	5E-18	0.4
Ttll5	7E-13	0.4	Ankrd44	1E-24	0.4
Vamp3	5E-14	0.4	Tmem14c	8E-43	0.4
Bag1	1E-41	0.4	Gapdh	7E-74	0.4
Erf	5E-14	0.4	Ttl	1E-18	0.4
Plekhh3	8E-13	0.4	Lipt2	3E-11	0.4
Cnep1r1	3E-17	0.4	Tnfaip6	2E-29	0.4
Sp3	3E-17	0.4	Vill	4E-15	0.4
Dusp10	4E-09	0.4	Prrc2a	9E-20	0.4
Zcrb1	7E-32	0.4	Elp6	2E-16	0.4
Maff	7E-07	0.4	Taf6l	1E-13	0.4
Gm42067	9E-08	0.4	Gm13056	2E-13	0.4

Undefined					
Control			NICD2		
Gene	*p*	FC	Gene	*p*	FC

Articular/Synovial Fibroblast					
Control			NICD2		
Gene	*p*	FC	Gene	*p*	FC
Dus1l	7E-01	0.2	Rpl11	2E-19	0.2
Rusc2	7E-01	0.2	Plekhf1	6E-01	0.2
Ice1	7E-01	0.2	Sh3yl1	2E-01	0.2
Arsi	7E-01	0.2	Atf7	4E-03	0.2
Morc2a	7E-01	0.2	Cradd	8E-01	0.2
Rufy1	5E-01	0.2	Mypop	5E-01	0.2
Slx4	1E-01	0.2	Etnk1	5E-01	0.2
Gss	7E-01	0.2	Ccdc57	6E-01	0.2
Armt1	5E-01	0.2	Mrps33	2E-02	0.2
Pparg	1E-02	0.2	Dtd1	7E-01	0.2
Fam199x	6E-01	0.2	mt-Nd4l	8E-06	0.2
Gm28198	6E-01	0.2	Eif2s3y	4E-01	0.2
Cygb	3E-01	0.2	Ppp1cc	2E-01	0.2
Tet2	8E-01	0.2	Dhrs11	3E-01	0.2
Poglut1	4E-02	0.2	Slc22a17	3E-01	0.2
Arl10	7E-01	0.2	Pikfyve	7E-01	0.2
Dlc1	2E-05	0.2	Zfp871	6E-01	0.2
Pi4ka	6E-01	0.2	Oscp1	7E-01	0.2
Vps41	1E-02	0.2	Rb1cc1	4E-02	0.2
Vti1a	7E-02	0.2	Unc93b1	5E-01	0.2
Eaf2	9E-01	0.2	Ror2	3E-01	0.2
Dip2c	2E-02	0.2	Comtd1	4E-01	0.2
Nek6	4E-01	0.2	Mast3	6E-01	0.2
Mgat4b	3E-01	0.2	Tmem69	3E-01	0.2
Gm46430	6E-01	0.2	Vkorc1	1E-04	0.2
Slc39a10	7E-01	0.2	Fbxw17	1E+00	0.2
Tcaf1	5E-02	0.2	Rela	4E-01	0.2
Ftx	7E-01	0.2	Zfhx2	7E-02	0.2
Gramd4	3E-01	0.2	Micos10	4E-05	0.2
Xpo4	6E-01	0.2	Ddx52	4E-01	0.2
Dubr	2E-01	0.2	Asb13	6E-01	0.2
Ccdc84	5E-01	0.2	Tbx4	9E-02	0.2
Fbrsl1	5E-01	0.2	Cuedc1	9E-01	0.2
Kdm5d	8E-01	0.2	Map1a	3E-02	0.2
Neurl2	6E-01	0.2	Zfp729a	3E-01	0.2
Osbpl3	6E-01	0.2	Slc25a51	6E-01	0.2
Knop1	2E-01	0.2	Tmem104	4E-01	0.2
Cyb5r3	4E-04	0.2	Dop1b	8E-01	0.2
Mpdu1	9E-01	0.2	Rps9	1E-18	0.2
Tmem143	3E-01	0.2	Ankrd28	2E-01	0.2
Gorasp1	3E-01	0.2	Tchp	3E-01	0.2
Tmtc3	6E-01	0.2	Ankmy2	9E-01	0.2
Hmbox1	5E-01	0.2	Cnnm3	5E-01	0.2
Setd4	7E-01	0.2	Slc39a3	3E-01	0.2
Rpl37a	5E-24	0.2	Zfp53	1E+00	0.2
Armcx1	3E-01	0.2	Pofut1	7E-01	0.2
Abi1	1E-01	0.2	Rpl27a	8E-18	0.2
Oscp1	8E-01	0.2	Tmem251	9E-01	0.2
Sgtb	5E-01	0.2	Osbpl7	2E-01	0.2
Lpp	5E-06	0.2	Rab4a	4E-01	0.2
Tmem203	4E-01	0.2	Daxx	9E-02	0.2
Sptlc2	1E-01	0.2	Tmem127	7E-01	0.2
Sardh	4E-01	0.2	Blvrb	1E-04	0.2
Zfp212	3E-01	0.2	Rsf1os1	2E-01	0.2
Pbx2	9E-01	0.2	Mrps21	2E-04	0.2
Ctdp1	4E-01	0.2	Zfp322a	5E-01	0.2
Snx1	7E-02	0.2	Mrps35	7E-01	0.2
Nbr1	1E-01	0.2	Abhd12	5E-01	0.2
Zfp512	9E-01	0.2	Rlf	5E-01	0.2
Dchs1	2E-01	0.2	Trmt112	2E-03	0.2
Kiz	3E-01	0.2	Rrp7a	5E-01	0.2
Chmp2b	1E-02	0.2	1110032A03Rik	2E-01	0.2
Unk	1E-01	0.2	Mkrn1	4E-01	0.2
Yap1	8E-01	0.2	Ints6	8E-01	0.2
Phkg2	3E-01	0.2	Tcta	8E-01	0.2
Trim11	5E-01	0.2	Rrm2b	6E-01	0.2
Cxcl5	5E-02	0.2	Prorp	2E-01	0.2
Tollip	9E-01	0.2	Tusc2	6E-01	0.2
Adar	7E-01	0.2	Stxbp1	9E-01	0.2
Gon4l	3E-01	0.2	Zfp938	8E-01	0.2
Ccdc32	1E+00	0.2	Zfp12	2E-01	0.2
Aga	1E-03	0.2	Bet1l	2E-02	0.2
Aup1	6E-01	0.2	Cyld	3E-01	0.2

Limb Mesenchyme					
Control			NICD2		
Gene	*p*	FC	Gene	*p*	FC
Ppa1	6E-12	0.2	Gm31323	6E-03	0.2
Zfp369	8E-06	0.2	Mpp2	2E-04	0.2
1700084C06Rik	6E-06	0.2	Ak6	5E-06	0.2
Hspa13	1E-06	0.2	Nvl	3E-04	0.2
Mrps23	2E-06	0.2	Tefm	2E-03	0.2
Mon2	2E-06	0.2	Acyp1	2E-05	0.2
Sec23ip	1E-05	0.2	Cep131	2E-02	0.2
Pum1	2E-08	0.2	Zbtb38	7E-06	0.2
Tcea3	4E-09	0.2	Tmem209	2E-04	0.2
1700029J07Rik	5E-03	0.2	Gtf2f2	3E-05	0.2
Mccc1	5E-05	0.2	Ankrd13a	8E-05	0.2
Fam20b	7E-06	0.2	Zdhhc21	1E-03	0.2
Tnfrsf12a	6E-09	0.2	Sfxn2	4E-04	0.2
Decr2	3E-06	0.2	Zfhx2	1E-02	0.2
Puf60	6E-06	0.2	Gtf3c5	2E-01	0.2
Nthl1	9E-05	0.2	Atg3	1E-07	0.2
Zdhhc12	2E-04	0.2	Ralgapa1	8E-06	0.2
Tmem181a	2E-05	0.2	Plekhm3	2E-05	0.2
Zcchc14	1E-05	0.2	Cep83	1E-04	0.2
Tmsb10	9E-15	0.2	Arfgap3	2E-06	0.2
Aph1a	8E-07	0.2	Bcl9	4E-06	0.2
Gm16552	1E-03	0.2	Hnrnph3	1E-09	0.2
Kifap3	7E-08	0.2	Exoc6b	2E-06	0.2
Med1	1E-06	0.2	Mtf2	2E-06	0.2
Rae1	9E-07	0.2	Dync1li2	1E-05	0.2
Dennd4a	3E-07	0.2	Nedd4l	9E-04	0.2
Psmg1	1E-04	0.2	Sel1l	3E-06	0.2
Actr2	3E-09	0.2	Brox	5E-04	0.2
Fbxo11	1E-07	0.2	Nfx1	2E-05	0.2
Phactr4	5E-05	0.2	Rab18	1E-13	0.2
Bdnf	7E-03	0.2	Kti12	3E-04	0.2
Trp53	3E-06	0.2	Morf4l1	1E-27	0.2
Acy1	2E-03	0.2	Shmt2	8E-06	0.2
Evl	4E-06	0.2	Nap1l4	3E-08	0.2
Rbm4b	2E-07	0.2	Mast2	4E-06	0.2
AI597479	3E-04	0.2	Actr3	4E-09	0.2
Gmeb1	1E-06	0.2	Eif4enif1	4E-05	0.2
Chid1	3E-05	0.2	Fam168b	1E-03	0.2
Mindy4	4E-05	0.2	Gtf3c3	6E-05	0.2
Med26	6E-06	0.2	Zbtb17	9E-04	0.2
Arl6ip4	2E-09	0.2	Ube2f	1E-07	0.2
Ift57	4E-06	0.2	Ccdc107	2E-07	0.2
Capn7	3E-04	0.2	Mapk1	6E-05	0.2
Eif1	2E-36	0.2	Aatf	5E-06	0.2
Ints4	1E-05	0.2	Pdzd11	2E-07	0.2
Eipr1	1E-05	0.2	Ctnnbip1	1E-05	0.2
Dnajc17	2E-04	0.2	Adgrg2	2E-07	0.2
Cep85	4E-05	0.2	Rassf3	4E-04	0.2
Afg1l	8E-03	0.2	Ints4	2E-04	0.2
Trp53i13	2E-04	0.2	Pigm	4E-03	0.2
Nploc4	2E-04	0.2	Eif2b5	2E-06	0.2
Slc10a3	1E-04	0.2	Tsc22d2	5E-06	0.2
Rpf2	7E-06	0.2	Hsf1	2E-04	0.2
Npc1	3E-04	0.2	Rita1	5E-05	0.2
Klhl9	2E-08	0.2	Tmem246	3E-03	0.2
Pcca	2E-06	0.2	Sbno1	2E-06	0.2
Kdm1a	2E-09	0.2	Phrf1	8E-06	0.2
Tmem138	3E-05	0.2	Gm49692	4E-02	0.2
Alg6	3E-05	0.2	Ylpm1	7E-06	0.2
Surf2	7E-06	0.2	Ndel1	7E-07	0.2
Wipf2	5E-05	0.2	Akap7	3E-06	0.2
1810034E14Rik	3E-04	0.2	Golga3	2E-06	0.2
Catsper2	1E-02	0.2	Fzd1	3E-05	0.2
Pigq	1E-05	0.2	Trps1	8E-12	0.2
Snrnp200	2E-06	0.2	Cep170	5E-07	0.2
Sf3b3	4E-09	0.2	Smarce1	1E-09	0.2
Larp4	4E-07	0.2	Ybx3	1E-11	0.2
Morf4l1	9E-31	0.2	Gm9856	3E-04	0.2
Gna12	1E-05	0.2	Mar9	7E-05	0.2
Zfp213	7E-04	0.2	Bdp1	3E-06	0.2
Cyp4f16	2E-03	0.2	Mgrn1	1E-02	0.2
Ddx3x	2E-08	0.2	Rnf14	6E-05	0.2
Tmem69	4E-03	0.2	Ier5	2E-05	0.2

Chondrogenic					
Control			NICD2		
Gene	*p*	FC	Gene	*p*	FC
Zfp260	1E-07	0.2	Eif4g2	1E-15	0.2
Zfp523	3E-01	0.2	Pnpla8	5E-06	0.2
Igip	5E-01	0.2	Gatad2b	1E-03	0.2
Zfp445	2E-03	0.2	B830012L14Rik	1E-02	0.2
Zfp182	2E-02	0.2	Strada	2E-01	0.2
Lefty1	5E-01	0.2	Ddx10	4E-02	0.2
Rpp38	3E-01	0.2	Gm37494	1E-01	0.2
Zfp553	9E-02	0.2	Nadk2	6E-01	0.2
Dnaaf4	7E-01	0.2	Son	2E-12	0.2
B3galnt2	3E-01	0.2	Mia2	2E-05	0.2
Nom1	1E-02	0.2	Banp	4E-01	0.2
Rbbp6	4E-06	0.2	Rnf38	2E-01	0.2
Dusp7	5E-02	0.2	Smug1	9E-01	0.2
Tmbim4	8E-08	0.2	Zscan26	1E-02	0.2
Tln1	2E-11	0.2	Zfp758	7E-01	0.2
Fbxo21	1E-01	0.2	1300002E11Rik	2E-01	0.2
Scfd2	8E-02	0.2	Wbp2	2E-02	0.2
Tcf25	1E-12	0.2	Kpna1	1E-03	0.2
LTO1	3E-01	0.2	Wdr78	7E-01	0.2
Fbxw17	3E-02	0.2	Rabgef1	8E-04	0.2
Mllt10	2E-05	0.2	Mapkapk2	7E-02	0.2
Mau2	6E-02	0.2	Timmdc1	6E-02	0.2
Spsb3	7E-02	0.2	Tmem98	8E-02	0.2
Tfg	2E-06	0.2	Anks3	4E-01	0.2
Chd6	5E-06	0.2	Arhgap18	3E-02	0.2
Ift81	2E-02	0.2	Zfp146	1E-01	0.2
Pxmp2	2E-01	0.2	H2afv	3E-24	0.2
Srp14	1E-21	0.2	Cnot6l	5E-03	0.2
Rsrc2	6E-08	0.2	Fam133b	1E-02	0.2
Wdr13	3E-01	0.2	Abtb1	6E-01	0.2
Tdrd3	2E-03	0.2	Sec22a	2E-03	0.2
Rbak	5E-01	0.2	Plxdc2	2E-06	0.2
Rbm4b	1E-03	0.2	Zfp629	5E-01	0.2
Stc1	7E-09	0.2	Ccnl1	2E-02	0.2
Sec31a	5E-14	0.2	Agpat5	1E-01	0.2
Rrs1	7E-03	0.2	E2f5	5E-01	0.2
Wdr53	3E-02	0.2	Scrn2	6E-01	0.2
Sf3b6	5E-14	0.2	Ext2	1E-05	0.2
Coq9	8E-02	0.2	Psmg2	2E-02	0.2
Impa1	6E-03	0.2	Pdss2	2E-01	0.2
Zkscan14	5E-01	0.2	Plpbp	2E-02	0.2
Micall1	6E-02	0.2	Fastkd2	3E-01	0.2
Phkb	9E-06	0.2	Phf10	3E-02	0.2
2810032G03Rik	7E-01	0.2	Vps26a	2E-03	0.2
Tmod1	9E-01	0.2	Eif4a2	4E-06	0.2
Zfp87	6E-01	0.2	Zfyve1	4E-01	0.2
Rnf121	2E-02	0.2	Tspyl1	8E-03	0.2
Usp36	3E-02	0.2	Zranb1	8E-03	0.2
0610009B22Rik	5E-06	0.2	Usp11	1E-02	0.2
Wdr78	5E-01	0.2	Pts	8E-04	0.2
2210408I21Rik	4E-02	0.2	Chrac1	3E-03	0.2
Sod1	3E-07	0.2	Glud1	2E-05	0.2
Agfg1	2E-04	0.2	Zfp672	2E-01	0.2
Tasor2	6E-03	0.2	Cnpy3	1E-01	0.2
Mettl15	6E-01	0.2	Ppp1r35	2E-01	0.2
Poglut1	8E-05	0.2	Agpat4	1E-01	0.2
Acbd5	3E-04	0.2	Ptpn1	1E-02	0.2
Ccdc90b	5E-08	0.2	Wnk3	4E-01	0.2
Gphn	5E-08	0.2	Gpbp1	4E-05	0.2
Gm49797	7E-02	0.2	Tm2d2	5E-10	0.2
Nfs1	1E-02	0.2	Eps15l1	2E-01	0.2
Dcun1d1	1E-02	0.2	Ccdc88a	7E-04	0.2
Wwox	9E-05	0.2	Hspa5	3E-10	0.2
Ndufaf4	4E-02	0.2	Foxo3	1E-01	0.2
Eif4g2	1E-18	0.2	Mirg	4E-01	0.2
Alkbh5	3E-05	0.2	Nab2	4E-01	0.2
Ctdsp2	4E-07	0.2	Zfp768	5E-01	0.2
Cnot2	2E-04	0.2	Msantd4	3E-03	0.2
Cstf3	9E-03	0.2	Orai2	6E-01	0.2
mt-Co1	2E-42	0.2	Gm48765	6E-01	0.2
Tet2	2E-01	0.2	Tmem87a	2E-01	0.2
Prpsap2	8E-02	0.2	Ubxn8	6E-01	0.2
Chsy1	1E-04	0.2	Rprd1b	9E-02	0.2

Fibroblast					
Control			NICD2		
Gene	*p*	FC	Gene	*p*	FC

Fibroblast?					
Control			NICD2		
Gene	*p*	FC	Gene	*p*	FC
Cct7	2E-21	0.4	Ipmk	7E-18	0.4
Thap12	3E-18	0.4	Col6a2	6E-32	0.4
Cerk	3E-12	0.4	Dnase2a	5E-16	0.4
Ikzf5	5E-11	0.4	1190005I06Rik	2E-13	0.4
Khdrbs1	5E-27	0.4	Npas2	4E-18	0.4
Smim12	3E-11	0.4	Fah	4E-10	0.4
Sh2b3	2E-11	0.4	Exosc5	4E-17	0.4
Stard9	7E-08	0.4	Pde3a	3E-21	0.4
Man2a1	6E-14	0.4	Bdnf	5E-12	0.4
Gm43462	3E-06	0.4	Vps16	1E-20	0.4
4933439C10Rik	4E-06	0.4	Man1b1	2E-12	0.4
Retreg1	2E-04	0.4	Wrap73	3E-15	0.4
Tmem201	1E-12	0.4	Smad2	4E-20	0.4
Exosc5	7E-15	0.4	Prkd3	4E-16	0.4
Uchl3	4E-14	0.4	Uxs1	2E-18	0.4
Sowahc	2E-13	0.4	Gars	3E-22	0.3
Gsap	3E-13	0.4	Zfp575	3E-07	0.3
Spcs3	1E-12	0.4	Sema3d	2E-07	0.3
Ap2m1	8E-29	0.4	Impdh2	2E-28	0.3
Galnt3	2E-14	0.4	Glt8d2	7E-19	0.3
Hace1	5E-15	0.4	Nfkbil1	3E-15	0.3
Lrp11	7E-11	0.4	Ccar2	7E-03	0.3
Dync1i1	3E-16	0.4	Armc1	3E-20	0.3
Epb41l2	5E-17	0.4	Glce	3E-17	0.3
Crocc	1E-10	0.4	Asnsd1	7E-21	0.3
Zfp760	7E-12	0.4	Mrpl33	3E-57	0.3
Arl2bp	3E-11	0.4	Slc19a2	6E-16	0.3
Gtpbp1	2E-11	0.4	Phf7	5E-08	0.3
Dgkq	4E-04	0.4	Lxn	8E-10	0.3
Arhgdia	1E-25	0.4	Gm16867	1E-08	0.3
Myo18a	7E-13	0.4	Tcf3	4E-18	0.3
Fkbp3	7E-28	0.4	Plscr4	8E-19	0.3
Pcyt1b	7E-10	0.4	Ube2z	8E-19	0.3
Scn3a	3E-10	0.4	Traf4	9E-23	0.3
Psmb8	7E-13	0.4	Zfp825	2E-09	0.3
C77080	4E-27	0.4	Mtmr2	2E-21	0.3
Smoc2	1E-14	0.4	Dnajc19	5E-33	0.3
Icmt	1E-11	0.4	Mrps7	3E-21	0.3
Eif2b5	3E-12	0.4	Cdc5l	3E-16	0.3
Cnot11	3E-08	0.4	Nudt19	7E-16	0.3
Taf4	1E-11	0.4	Ifrd1	3E-17	0.3
Oxnad1	2E-09	0.4	Ddx39b	2E-20	0.3
Cavin3	2E-29	0.4	Ythdf2	3E-16	0.3
Pkn2	1E-22	0.4	Tbc1d8b	5E-15	0.3
Npnt	9E-05	0.4	Epha2	2E-07	0.3
Mgll	1E-18	0.4	Gm13402	2E-07	0.3
Snrnp70	3E-27	0.4	Nipa2	6E-19	0.3
Prep	7E-07	0.4	Vac14	8E-16	0.3
Itga5	4E-15	0.4	Cyth3	1E-26	0.3
Ccnyl1	2E-11	0.4	Lhx9	4E-08	0.3
Ankrd39	2E-05	0.4	Rpgrip1l	2E-16	0.3
Zfp346	1E-13	0.4	Bag1	4E-47	0.3
Cfap100	4E-06	0.4	Pop5	2E-20	0.3
Hdac1	3E-21	0.4	Plrg1	4E-07	0.3
Dglucy	1E-13	0.4	Lonp1	2E-14	0.3
Eif4a3	1E-15	0.3	M6pr	1E-17	0.3
Pop5	2E-17	0.3	Cnep1r1	2E-19	0.3
Snrpd2	2E-37	0.3	Fblim1	1E-04	0.3
4930511M06Rik	8E-06	0.3	Sdc3	4E-22	0.3
Plekhf2	2E-11	0.3	Prdm4	8E-08	0.3
Galns	1E-11	0.3	Kars	3E-15	0.3
Taf6l	1E-13	0.3	Nat2	4E-10	0.3
Ssh2	5E-18	0.3	Abhd13	2E-15	0.3
Polr2l	1E-15	0.3	Prkrip1	7E-18	0.3
Ubtf	9E-16	0.3	Wapl	1E-19	0.3
Fopnl	4E-15	0.3	Emsy	2E-16	0.3
Msantd3	2E-15	0.3	Strap	3E-21	0.3
Triap1	5E-10	0.3	Magt1	1E-26	0.3
Mettl14	1E-11	0.3	Fubp1	4E-21	0.3
Cand1	8E-14	0.3	Abce1	2E-19	0.3
Bud13	9E-13	0.3	Ltn1	3E-17	0.3
Trerf1	6E-17	0.3	Efs	4E-17	0.3
Gmcl1	9E-12	0.3	Ppp4c	5E-18	0.3

Undefined					
Control			NICD2		
Gene	*p*	FC	Gene	*p*	FC

Articular/Synovial Fibroblast					
Control			NICD2		
Gene	*p*	FC	Gene	*p*	FC
Ctsz	5E-06	0.2	Ier3	1E+00	0.2
Zfp715	6E-01	0.2	Cnp	9E-01	0.2
Nrg1	5E-03	0.2	Otud1	4E-01	0.2
1700037H04Rik	4E-01	0.2	Lzic	2E-01	0.2
Entpd6	2E-01	0.2	Casp3	1E+00	0.2
Pard3	3E-02	0.2	Asph	1E-02	0.2
Wipf2	8E-01	0.2	Prnp	1E-03	0.2
Cox7a2l	5E-08	0.2	Gsap	3E-01	0.2
Adal	1E-01	0.2	Pnkd	3E-02	0.2
2310022A10Rik	1E+00	0.2	Vps41	7E-02	0.2
Tbc1d5	1E-01	0.2	Otud3	5E-01	0.2
Tanc2	2E-01	0.2	Elf4	2E-01	0.2
Ncstn	2E-01	0.2	Suox	3E-01	0.2
Zfp931	6E-01	0.2	Cmc4	8E-01	0.2
Gm14296	1E+00	0.2	Adprh	5E-01	0.2
Tmem231	5E-01	0.2	Bcl9l	9E-02	0.2
Hectd3	3E-01	0.2	Tut4	1E-02	0.2
Gm4117	4E-01	0.2	Nlk	7E-01	0.2
Cpsf3	6E-01	0.2	Csad	1E-01	0.2
Rpgr	5E-01	0.2	Tmem80	3E-01	0.2
Endog	9E-01	0.2	Hspb2	6E-01	0.2
Mr1	6E-01	0.2	Tmem218	6E-01	0.2
Sft2d3	1E+00	0.2	Dubr	9E-01	0.2
Arhgef40	2E-01	0.2	Cebpa	1E-01	0.2
Psmd8	3E-03	0.2	Myd88	5E-01	0.2
Gm49539	2E-01	0.2	Smpd4	1E-01	0.2
Fam234a	1E-01	0.2	3830406C13Rik	1E-01	0.2
Ubl7	8E-01	0.2	Cd109	2E-01	0.2
Gab3	7E-01	0.2	Slc35e3	2E-01	0.2
Syap1	2E-02	0.2	Zswim7	5E-01	0.2
Clk2	5E-01	0.2	Lancl1	8E-01	0.2
Mboat7	1E+00	0.2	Zfp944	2E-01	0.2
Rps27rt	5E-01	0.2	Elp6	2E-01	0.2
Atp6ap1	2E-02	0.2	Meis2	9E-01	0.2
Bclaf3	6E-01	0.2	Ebpl	9E-03	0.2
Atg2a	6E-01	0.2	4930402H24Rik	1E-01	0.2
Zfp39	4E-01	0.2	Vps13d	3E-01	0.2
C1galt1c1	8E-01	0.2	Zbtb7a	1E-01	0.2
Zswim4	2E-01	0.2	Ap3s2	5E-01	0.2
Atp8b1	6E-01	0.2	Tsc22d4	8E-03	0.2
Gabpb2	6E-01	0.2	Mfsd3	5E-01	0.2
Dhx35	5E-01	0.2	Fbxo4	4E-01	0.2
Pex11a	8E-01	0.2	Wbp1	9E-01	0.2
Sgpp1	6E-01	0.2	Glt8d1	1E-02	0.2
Kdm2b	6E-01	0.2	Pip4k2c	4E-01	0.2
Nudt9	9E-02	0.2	Vps9d1	5E-01	0.2
Chm	7E-01	0.1	Fkbp8	6E-02	0.2
Stn1	4E-01	0.1	Kdm3a	9E-01	0.2
Cmc1	3E-02	0.1	Usp53	9E-01	0.2
Ralgapa2	6E-01	0.1	Apba1	2E-01	0.2
4933431E20Rik	4E-01	0.1	Trmt2b	3E-01	0.2
Arhgap42	2E-01	0.1	Dmpk	9E-01	0.2
9930104L06Rik	4E-01	0.1	Ntmt1	9E-01	0.2
Cflar	3E-01	0.1	Rpl9	2E-14	0.2
Zfp638	9E-03	0.1	Malat1	3E-09	0.2
Prkaca	3E-01	0.1	Alkbh6	5E-01	0.2
Foxred2	7E-01	0.1	Armc6	9E-02	0.2
Mzt2	4E-01	0.1	Eif3h	1E-04	0.2
Ube2h	9E-03	0.1	Nosip	1E-01	0.2
Pbxip1	1E-02	0.1	Cab39l	4E-02	0.2
Tep1	7E-01	0.1	Slc31a2	7E-01	0.2
Chchd5	8E-01	0.1	Thap11	6E-01	0.2
Zfp944	2E-01	0.1	Pts	4E-01	0.2
Bin3	8E-01	0.1	Zadh2	9E-01	0.2
Timm29	4E-01	0.1	Zhx1	4E-01	0.2
Gli3	3E-03	0.1	Naa20	2E-01	0.2
Zbtb10	7E-01	0.1	Cpne8	7E-04	0.2
Trim24	4E-01	0.1	Gm9856	2E-01	0.2
Dedd	2E-01	0.1	Tsc2	4E-01	0.2
Thap11	9E-01	0.1	Rps12	2E-08	0.2
Coprs	6E-01	0.1	Oxct1	1E-02	0.2
Marf1	3E-01	0.1	Lrba	5E-02	0.2
Spag9	3E-04	0.1	Gpbp1l1	9E-01	0.2

Limb Mesenchyme					
Control			NICD2		
Gene	*p*	FC	Gene	*p*	FC
Gjc1	3E-07	0.2	Runx1t1	3E-10	0.2
Cthrc1	1E-06	0.2	Btbd6	5E-04	0.2
Zfp2	3E-03	0.2	Atl3	1E-07	0.2
Zfp768	7E-05	0.2	Nfu1	3E-09	0.2
Fastkd2	3E-04	0.2	Zbtb20	4E-12	0.2
2810001G20Rik	1E-04	0.2	Mmadhc	4E-07	0.2
Rbm12b2	4E-03	0.2	Rnps1	5E-07	0.2
Pnpt1	3E-04	0.2	Gm10353	1E-02	0.2
Sgcd	6E-11	0.2	0610009E02Rik	2E-04	0.2
Zpr1	5E-05	0.2	Sptlc1	9E-04	0.2
Brix1	1E-05	0.2	Nop14	2E-05	0.2
Banp	3E-06	0.2	Hspg2	3E-10	0.2
Rbbp4	6E-12	0.2	Ankrd29	2E-09	0.2
Tmem184b	1E-04	0.2	Arcn1	1E-11	0.2
Rmdn3	2E-04	0.2	Fbxo21	7E-05	0.2
Ints8	9E-06	0.2	Tnrc18	8E-09	0.2
Fem1c	7E-07	0.2	Dab2ip	1E-05	0.2
B4galnt1	6E-03	0.2	Apex2	8E-06	0.2
Pdcd2l	7E-05	0.2	Crbn	4E-03	0.2
Azi2	6E-05	0.2	Mfsd8	5E-04	0.2
Mbtd1	3E-07	0.2	Tmed7	1E-06	0.2
Trim24	8E-07	0.2	Itpripl2	9E-07	0.2
Thg1l	1E-02	0.2	Suco	2E-06	0.2
Gm37768	3E-05	0.2	Ubxn6	7E-06	0.2
Klhl21	2E-03	0.2	Mxd4	8E-10	0.2
Lonrf1	3E-04	0.2	Apex1	2E-07	0.2
S1pr1	7E-03	0.2	Ube4a	2E-04	0.2
Mbd3	1E-10	0.2	Gabbr1	1E-04	0.2
Ubn2	4E-09	0.2	Kremen1	2E-03	0.2
Ddx23	5E-06	0.2	Elmsan1	4E-04	0.2
Gtf2h2	1E-04	0.2	Plagl2	6E-03	0.2
Banf1	5E-13	0.2	Them4	2E-02	0.2
Ddi2	3E-04	0.2	Rbfox2	3E-09	0.2
Pyroxd1	3E-05	0.2	Rcl1	1E-04	0.2
Nsmce3	1E-07	0.2	Nudt8	1E-04	0.2
Slc25a12	8E-06	0.2	Rhbdd3	9E-04	0.2
Cdk12	2E-07	0.2	Uspl1	9E-05	0.2
Zfp410	2E-04	0.2	Kif13b	5E-06	0.2
Dhx33	1E-02	0.2	Polr3f	8E-04	0.2
Ddx42	2E-08	0.2	Pmm2	1E-05	0.2
Snf8	4E-10	0.2	Tmem184c	3E-05	0.2
Ptprm	5E-10	0.2	Cdk2ap1	1E-06	0.2
Chuk	3E-06	0.2	Xylt2	1E-02	0.2
2610020C07Rik	3E-05	0.2	Rnf138	8E-06	0.2
Psmd1	6E-11	0.2	Nbeal1	6E-08	0.2
Adipor1	1E-08	0.2	BC031181	8E-10	0.2
Map9	2E-02	0.2	Ttc4	2E-06	0.2
Mink1	4E-04	0.2	Crlf3	3E-05	0.2
Ext2	3E-08	0.2	Edem1	3E-05	0.2
Ppp3cc	1E-04	0.2	Gask1b	4E-06	0.2
Arhgef25	1E-07	0.2	9430038I01Rik	6E-05	0.2
Morn4	4E-04	0.2	C1qtnf1	4E-04	0.2
Sf3a2	7E-05	0.2	Ncl	9E-18	0.2
Gm31763	3E-02	0.2	Eri3	2E-06	0.2
Rangrf	7E-04	0.2	Tnfsf11	6E-05	0.2
Dusp11	8E-11	0.2	Zbtb41	1E-04	0.2
Wwtr1	2E-09	0.2	Spast	1E-04	0.2
Tank	8E-08	0.2	Rnf170	3E-03	0.2
Zdhhc18	1E-04	0.2	Tmlhe	3E-04	0.2
Gm15952	3E-04	0.2	Agpat1	3E-04	0.2
Dnm1l	6E-07	0.2	Tmem43	1E-03	0.2
Arih1	4E-09	0.2	Gm20536	3E-03	0.2
Triqk	2E-05	0.2	Cltc	2E-12	0.2
Zfp692	1E-04	0.2	Trappc11	5E-04	0.2
Mbd2	7E-09	0.2	Nr2c2ap	1E-02	0.2
Wiz	1E-04	0.2	Accs	7E-03	0.2
Eif4h	3E-08	0.2	Hnrnpa1	8E-09	0.2
Nolc1	5E-08	0.2	Xpo6	5E-05	0.2
Ptrh2	3E-05	0.2	Birc6	1E-08	0.2
Dhx9	7E-09	0.2	Sfr1	6E-15	0.2
Pigv	4E-04	0.2	Fam177a	4E-05	0.2
Nbdy	1E-12	0.2	Pes1	2E-05	0.2
Lncppara	1E-05	0.2	Ecpas	1E-08	0.2

Chondrogenic					
Control			NICD2		
Gene	*p*	FC	Gene	*p*	FC
Tcea3	1E-09	0.2	Sarnp	5E-08	0.2
Fam151b	2E-01	0.2	Smg6	2E-03	0.2
Nutf2	6E-01	0.2	Rptor	1E-02	0.2
Leprotl1	2E-05	0.2	Wfs1	8E-01	0.2
Ppp3cc	2E-01	0.2	S100pbp	1E-01	0.2
Snupn	3E-01	0.2	Kif16b	1E-01	0.2
Gpr89	3E-02	0.2	Rab5a	3E-04	0.2
Akap17b	5E-01	0.2	Ppp1r16a	9E-01	0.2
Odf2l	2E-01	0.2	Mrpl41	1E-04	0.2
Ubr5	2E-04	0.2	Lman1	5E-12	0.2
Cry2	3E-01	0.2	Slc39a9	2E-01	0.2
Secisbp2	3E-02	0.2	Cbll1	7E-02	0.2
Rusc2	9E-02	0.2	Kdm5b	2E-04	0.2
Ubald2	1E-03	0.2	Dph6	2E-02	0.2
Pcm1	2E-09	0.2	Tk2	3E-01	0.2
Bap1	4E-01	0.2	Fam13b	9E-03	0.2
Tmem161a	4E-03	0.2	Pfdn1	9E-11	0.2
6720427I07Rik	2E-02	0.2	Zc3h4	9E-01	0.2
Pigl	7E-01	0.2	Tbc1d1	8E-01	0.2
Prkn	2E-04	0.2	Katna1	9E-02	0.2
Miga2	8E-01	0.2	Nampt	9E-03	0.2
Fam53a	1E-02	0.2	Cerk	3E-01	0.2
Ddx24	3E-11	0.2	Atad2b	2E-02	0.2
Uhmk1	2E-01	0.2	Mul1	9E-01	0.2
Smim19	1E-06	0.2	Stxbp4	4E-02	0.2
Gm42439	6E-01	0.2	C2cd5	3E-01	0.2
Zfp846	9E-02	0.2	Usp20	8E-01	0.2
Zbtb17	6E-01	0.2	Clcc1	9E-03	0.2
Ddx42	3E-06	0.2	Spryd3	1E-01	0.2
Timmdc1	1E-02	0.2	Rbm33	2E-02	0.2
Coq8a	6E-01	0.2	Ddx20	9E-01	0.2
Fam185a	4E-01	0.2	St7	5E-02	0.2
Ppcs	4E-01	0.2	Dnajc18	1E-01	0.2
Nae1	4E-03	0.2	Asl	2E-01	0.2
Trmt13	5E-02	0.2	Cdipt	2E-03	0.2
Utp14a	1E-02	0.2	Adck5	4E-01	0.2
Mat2b	6E-07	0.2	Ddrgk1	3E-06	0.2
4932438A13Rik	5E-04	0.2	Mterf2	4E-01	0.2
Slc35c2	5E-03	0.2	Srpr	3E-09	0.2
Tmub2	2E-01	0.2	Fam118b	1E+00	0.2
Slc35a1	9E-04	0.2	Limk2	8E-01	0.2
Kctd3	2E-01	0.2	Dnaja3	5E-02	0.2
Serpine2	9E-08	0.2	Ccdc51	7E-01	0.2
Mettl23	1E-05	0.2	Pdk2	3E-01	0.2
Kansl3	1E-01	0.2	Mmgt1	6E-01	0.2
Det1	4E-01	0.2	Gart	6E-01	0.2
Zfp1	5E-02	0.2	Hectd2	5E-01	0.2
Abhd17b	6E-04	0.2	Ttc12	1E+00	0.2
Ccdc114	6E-01	0.2	Dis3l2	2E-03	0.2
Rdh13	6E-01	0.2	Dnajb11	1E-05	0.2
Amy1	7E-01	0.2	Ensa	1E-02	0.2
Uchl3	3E-04	0.2	Cep97	7E-01	0.2
Lrp1	2E-13	0.2	Armc10	6E-01	0.2
Gmps	1E-06	0.2	Smim10l1	4E-07	0.2
Ccdc91	1E-01	0.2	Rrp7a	3E-01	0.2
Pcmtd2	2E-03	0.2	Ccdc62	5E-01	0.2
Abca3	9E-01	0.2	Ep300	9E-03	0.2
Ppp2r5a	1E-05	0.2	Cyb5r1	6E-01	0.2
Mecp2	7E-04	0.2	Eif2ak1	6E-02	0.2
Jazf1	5E-01	0.2	Spry2	1E-01	0.2
Tecpr2	2E-01	0.2	Col4a3bp	2E-01	0.2
Foxj2	2E-01	0.2	Heatr1	8E-01	0.2
Kctd6	5E-01	0.2	Ankhd1	1E-02	0.2
Med25	5E-03	0.2	Tmem222	2E-01	0.2
Ccz1	8E-05	0.2	Cdip1	2E-02	0.2
Snx19	2E-01	0.2	Zfp444	4E-01	0.2
Jtb	2E-07	0.2	Msra	5E-01	0.2
Uri1	4E-07	0.2	Ccdc181	9E-01	0.2
Slc2a10	7E-01	0.2	Smarcd1	3E-03	0.2
D830050J10Rik	7E-02	0.2	Tmem201	7E-01	0.2
Lrrc8a	9E-02	0.2	Nutf2	9E-01	0.2
Zfp704	6E-03	0.2	Blmh	7E-03	0.2
Zfp287	8E-01	0.2	Grk5	2E-01	0.2

Fibroblast					
Control			NICD2		
Gene	*p*	FC	Gene	*p*	FC

Fibroblast?					
Control			NICD2		
Gene	*p*	FC	Gene	*p*	FC
Kars	6E-15	0.3	Mtf2	6E-18	0.3
Gnl1	9E-16	0.3	Optn	2E-18	0.3
Clk3	1E-11	0.3	Zcchc3	2E-17	0.3
Gli2	2E-14	0.3	Zbtb48	2E-05	0.3
Aifm1	1E-17	0.3	Pank4	6E-12	0.3
Ephb2	2E-12	0.3	Pde7a	3E-16	0.3
Dapp1	1E-11	0.3	Ptgfrn	4E-18	0.3
Slc35a3	2E-13	0.3	Trdmt1	3E-13	0.3
Sf3a1	9E-16	0.3	Pdcd7	7E-16	0.3
Ethe1	1E-14	0.3	Rpl39l	6E-05	0.3
Wrnip1	1E-13	0.3	Traf3ip1	6E-22	0.3
Sptssa	2E-21	0.3	Gm12526	9E-09	0.3
Emsy	2E-15	0.3	Ptprk	1E-15	0.3
Tpi1	5E-32	0.3	Luc7l3	9E-24	0.3
Limk2	9E-15	0.3	Ahi1	8E-22	0.3
Tbc1d9	3E-06	0.3	Fam129b	3E-18	0.3
Cnih1	3E-22	0.3	Nav3	2E-09	0.3
Pitrm1	5E-11	0.3	Mrps17	3E-19	0.3
Ap1s2	9E-15	0.3	Impa1	2E-12	0.3
Rbm45	3E-07	0.3	Nfx1	4E-16	0.3
Prkce	7E-15	0.3	Cpsf1	2E-10	0.3
Zbtb18	1E-11	0.3	Ngdn	4E-13	0.3
Utp15	1E-14	0.3	Macrod2	7E-27	0.3
Eloc	6E-29	0.3	Cldn34c1	2E-09	0.3
Arf6	2E-19	0.3	Cep164	2E-21	0.3
Bbs1	4E-07	0.3	Tsfm	3E-12	0.3
Hist1h4i	7E-15	0.3	Adcy3	2E-14	0.3
Ptpn14	1E-15	0.3	Nxpe3	9E-11	0.3
Napepld	3E-09	0.3	Cdk16	3E-14	0.3
Sord	8E-14	0.3	1700108F19Rik	2E-07	0.3
Limd2	5E-13	0.3	Gm20707	7E-04	0.3
Rtca	2E-13	0.3	Ing2	6E-12	0.3
Ppp1r14b	8E-24	0.3	Mrm2	1E-11	0.3
Cwc15	3E-26	0.3	Khdrbs1	3E-29	0.3
Mtus1	2E-10	0.3	Atl3	2E-22	0.3
Ccnk	3E-11	0.3	Srsf6	2E-23	0.3
Hnrnpdl	3E-16	0.3	Sh3rf1	1E-20	0.3
Mfap5	3E-12	0.3	Oxct1	2E-35	0.3
Mpp1	2E-10	0.3	4930549G23Rik	9E-11	0.3
Prrc2a	2E-16	0.3	Wdr18	2E-12	0.3
Arap1	2E-06	0.3	Ilkap	1E-11	0.3
Ltn1	1E-14	0.3	Rbm8a	3E-19	0.3
Txnl1	5E-41	0.3	Srrd	6E-12	0.3
Pitpnm1	5E-11	0.3	Ankrd55	6E-23	0.3
Gcsh	5E-16	0.3	Pmm2	6E-13	0.3
Zfp825	5E-11	0.3	Caprin1	3E-38	0.3
Stx1a	7E-06	0.3	Gnb4	1E-20	0.3
Polr2f	1E-31	0.3	Jph1	4E-09	0.3
Vegfb	3E-18	0.3	Tln2	3E-15	0.3
Zfp131	2E-13	0.3	Arhgap6	4E-23	0.3
Fkbp1b	3E-11	0.3	Mtrex	2E-14	0.3
Ldlrap1	3E-12	0.3	Plxnb2	3E-20	0.3
Magoh	9E-20	0.3	Miga2	4E-15	0.3
Cfl2	2E-20	0.3	Snx16	5E-19	0.3
Shroom4	2E-13	0.3	D6Wsu163e	2E-09	0.3
Zbtb2	1E-09	0.3	Qser1	3E-18	0.3
Cstf3	2E-11	0.3	Urb2	1E-09	0.3
Lrrc8e	4E-10	0.3	Btaf1	2E-17	0.3
Map4	4E-22	0.3	Atic	5E-12	0.3
Pcdh7	2E-17	0.3	Tulp4	8E-22	0.3
Caprin1	4E-31	0.3	Wdyhv1	1E-13	0.3
Med8	4E-16	0.3	Prdm10	2E-15	0.3
Lingo1	1E-09	0.3	Grsf1	8E-14	0.3
Tomm40	2E-10	0.3	Klhl42	9E-16	0.3
Cep126	5E-08	0.3	Papss2	2E-22	0.3
Exoc3l4	2E-14	0.3	Rps19bp1	2E-15	0.3
Atrip	8E-16	0.3	Hnrnpm	8E-30	0.3
Dok4	2E-07	0.3	Spg7	1E-17	0.3
Capn2	9E-16	0.3	Gm16278	6E-09	0.3
Eif4enif1	3E-10	0.3	Zfp131	1E-17	0.3
Rer1	1E-31	0.3	Hspa8	6E-66	0.3
Ccdc62	5E-10	0.3	Exd1	2E-09	0.3
Zfp472	1E-08	0.3	Gnai1	1E-15	0.3

Undefined					
Control			NICD2		
Gene	*p*	FC	Gene	*p*	FC

Articular/Synovial Fibroblast					
Control			NICD2		
Gene	*p*	FC	Gene	*p*	FC
Zmat3	5E-01	0.1	Sqstm1	2E-04	0.2
0610030E20Rik	3E-01	0.1	Lamtor4	3E-03	0.2
4933407K13Rik	5E-01	0.1	Mindy2	1E+00	0.2
Pcdh19	7E-01	0.1	Unkl	3E-01	0.2
Ccdc73	7E-01	0.1	Atp8b1	6E-01	0.2
Tsc1	8E-01	0.1	Tpd52l1	2E-01	0.1
Jmjd1c	1E-01	0.1	Copg2	9E-01	0.1
Fto	5E-03	0.1	Mbtd1	7E-01	0.1
Slc39a1	3E-01	0.1	Rps6kb2	2E-01	0.1
Pced1b	9E-01	0.1	Deaf1	7E-01	0.1
Uqcc2	1E-05	0.1	Sec61a2	8E-01	0.1
Sp1	6E-01	0.1	Stxbp3	3E-01	0.1
Srd5a1	9E-02	0.1	Champ1	7E-01	0.1
Mcee	1E-02	0.1	Szt2	3E-01	0.1
Pdia3	6E-07	0.1	Ranbp10	8E-01	0.1
Brip1os	3E-01	0.1	Eef2kmt	6E-01	0.1
Helb	2E-01	0.1	Extl3	8E-01	0.1
Dvl2	7E-01	0.1	Cnbd2	4E-01	0.1
Sh2b1	5E-01	0.1	Cyth1	7E-01	0.1
Rab11fip5	9E-01	0.1	D330041H03Rik	3E-01	0.1
Styx	6E-01	0.1	Gtf2h3	5E-01	0.1
Smim8	9E-02	0.1	Sgpl1	1E+00	0.1
Prtg	4E-01	0.1	C030034I22Rik	2E-01	0.1
Ammecr1l	9E-01	0.1	Setd7	3E-01	0.1
2510009E07Rik	3E-01	0.1	Sp3	4E-02	0.1
Fam193b	1E+00	0.1	Ncoa5	8E-01	0.1
Fkbpl	4E-01	0.1	Lmbrd1	1E-01	0.1
Bloc1s1	7E-01	0.1	Zfp189	2E-01	0.1
Nectin2	9E-01	0.1	Bmp2k	9E-01	0.1
Ccdc174	6E-01	0.1	Meox2	3E-01	0.1
Tmem38a	3E-01	0.1	Stat5b	9E-01	0.1
Arpc1b	2E-06	0.1	Nagk	1E-01	0.1
Fbxl15	6E-01	0.1	Traf5	3E-01	0.1
Arl16	8E-01	0.1	Zdhhc7	4E-01	0.1
Mkrn2	8E-01	0.1	Zfp942	6E-01	0.1
Patz1	3E-01	0.1	Dus3l	3E-01	0.1
Zfp513	4E-01	0.1	Tsen2	3E-01	0.1
Tmlhe	9E-01	0.1	Mtmr3	3E-01	0.1
Ubap1	9E-01	0.1	Ust	9E-01	0.1
Gpalpp1	9E-01	0.1	Nek4	3E-01	0.1
Slc29a3	7E-01	0.1	Setd1b	9E-01	0.1
Timm44	3E-01	0.1	Hspb1	3E-02	0.1
Gm14325	1E-01	0.1	Ttc5	7E-01	0.1
Gm4890	2E-01	0.1	Trp53bp2	1E+00	0.1
0610012G03Rik	6E-04	0.1	Arhgef1	1E+00	0.1
Clic1	1E-07	0.1	Akap17b	2E-01	0.1
4931414P19Rik	4E-01	0.1	Tnrc6b	2E-03	0.1
Washc5	7E-01	0.1	Gpi1	4E-03	0.1
Snx6	1E-03	0.1	Maml1	3E-01	0.1
Lclat1	3E-01	0.1	Ddi2	8E-01	0.1
Gucy1a2	2E-01	0.1	Snx24	8E-01	0.1
Ltbp1	4E-01	0.1	4933424G05Rik	1E-01	0.1
Sap30bp	6E-01	0.1	Casp2	2E-01	0.1
Slf2	9E-01	0.1	Zfp518a	9E-01	0.1
Slc35a5	9E-01	0.1	Jmjd7	2E-01	0.1
Tfip11	9E-01	0.1	Gemin8	7E-01	0.1
Ipo4	9E-01	0.1	Rnasek	3E-03	0.1
Ift27	2E-02	0.1	Decr2	1E+00	0.1
Gm48742	6E-01	0.1	Tspan14	4E-01	0.1
Ankrd54	1E-01	0.1	Tsen54	2E-01	0.1
Tor2a	7E-01	0.1	Zfp952	4E-02	0.1
Ralbp1	1E-02	0.1	Bri3bp	3E-01	0.1
Zfp955b	9E-01	0.1	Rnf185	3E-01	0.1
Rps27	4E-16	0.1	Apeh	9E-01	0.1
Nt5dc2	3E-01	0.1	Pcyt2	3E-01	0.1
Deaf1	4E-01	0.1	Hdac3	4E-01	0.1
Supt4a	6E-03	0.1	Dact1	1E+00	0.1
Sertad3	9E-01	0.1	Mfn1	8E-01	0.1
Cenpx	2E-04	0.1	Uqcrc1	9E-02	0.1
Kctd20	8E-01	0.1	Calcoco1	4E-01	0.1
Ccpg1	5E-02	0.1	Rbm18	1E+00	0.1
Rbm48	1E-01	0.1	1700123M08Rik	5E-01	0.1
Slc38a9	8E-01	0.1	Mrpl30	1E-02	0.1

Limb Mesenchyme

Control			NICD2		
Gene	*p*	FC	Gene	*p*	FC
Slc25a53	1E-03	0.2	Bud31	2E-06	0.2
Vcp	6E-15	0.2	Gmppb	3E-06	0.2
Tpst1	1E-07	0.2	Kdm5a	3E-06	0.2
Cstf2	6E-04	0.2	Zfp830	1E-04	0.2
Atp13a3	4E-07	0.2	Ube3a	1E-07	0.2
Smarcd3	5E-05	0.2	Acp2	3E-05	0.2
Setd5	9E-11	0.2	Mau2	4E-05	0.2
Amot	5E-07	0.2	Arel1	2E-04	0.2
Mir99ahg	1E-12	0.2	Wdr44	4E-04	0.2
Hmg20b	4E-09	0.2	Nipa2	2E-04	0.2
Uqcrq	1E-17	0.2	Ppil4	1E-03	0.2
Ddx51	2E-03	0.2	Gnl2	6E-05	0.2
Prrx2	3E-09	0.2	Map4k5	8E-06	0.2
Ambra1	1E-06	0.2	Sardh	1E-03	0.2
Tug1	1E-06	0.2	Syt11	8E-05	0.2
Hspg2	3E-09	0.2	Tpm2	1E-13	0.2
Copg1	9E-08	0.2	Nae1	2E-04	0.2
Dgkq	4E-03	0.2	Zfp410	6E-04	0.2
Ccn2	5E-40	0.2	Supt5	5E-08	0.2
Dnajb14	5E-06	0.2	Sec23b	4E-04	0.2
Coq10b	2E-05	0.2	Hoxc5	7E-05	0.2
Plpbp	3E-06	0.2	Vapa	5E-14	0.2
Sgms2	3E-05	0.2	Wbp4	9E-08	0.2
D130017N08Rik	1E-02	0.2	Spout1	2E-03	0.2
Gm13832	1E-04	0.2	Ift74	6E-07	0.2
Arf2	2E-04	0.2	A230072C01Rik	6E-03	0.2
Ank2	8E-07	0.2	Ncoa4	2E-05	0.2
Atxn7l3b	3E-13	0.2	Trdmt1	2E-02	0.2
Dnajb11	3E-08	0.2	Ikzf5	5E-03	0.2
Prkdc	3E-05	0.2	Mat2b	1E-07	0.2
Zscan21	1E-05	0.2	Xrcc6	2E-04	0.2
BC067074	2E-03	0.2	Mrm2	1E-02	0.2
Flrt1	2E-04	0.2	Ash2l	2E-04	0.2
Rbm17	2E-09	0.2	Scaf8	6E-07	0.2
Aig1	1E-09	0.2	Pcm1	4E-08	0.2
Nufip1	4E-05	0.2	Stx5a	6E-07	0.2
Mpp5	7E-06	0.2	Slc37a4	2E-02	0.2
Fbxl20	1E-05	0.2	Tmem29	4E-05	0.2
Dynlt3	4E-10	0.2	Prkd3	3E-04	0.2
Mtrex	8E-06	0.2	Dip2c	2E-08	0.2
Snupn	2E-06	0.2	Prmt1	5E-07	0.2
Primpol	3E-04	0.2	Timm23	2E-09	0.2
Kdm6a	7E-06	0.2	Rnf20	3E-04	0.2
Npat	3E-05	0.2	Lnx2	1E-01	0.2
Rasa1	6E-09	0.2	Nus1	1E-05	0.2
Hectd2	2E-05	0.2	Pdhb	2E-05	0.2
Podxl2	3E-04	0.2	Crcp	8E-04	0.2
Chmp6	5E-05	0.2	Atl1	5E-04	0.2
Ric3	5E-05	0.2	Ccny	6E-08	0.2
Csnk1d	1E-06	0.2	Coro1c	4E-06	0.2
Thyn1	1E-07	0.2	Hnrnpd	4E-09	0.2
B230118H07Rik	9E-08	0.2	Cox18	3E-04	0.2
Rabggtb	2E-05	0.2	Myo6	5E-05	0.2
Tbc1d19	7E-05	0.2	Ywhaq	8E-10	0.2
Actr5	2E-02	0.2	Ptp4a2	1E-11	0.2
Dapk3	2E-06	0.2	Chd2	8E-06	0.2
Zfp974	2E-03	0.2	Socs6	8E-04	0.2
Fam117b	2E-04	0.2	Rcor2	9E-04	0.2
Ttc4	3E-05	0.2	Trip11	1E-06	0.2
Ears2	3E-03	0.2	Ext1	1E-08	0.2
Nfyb	2E-05	0.2	Efl1	2E-06	0.2
Usp49	1E-02	0.2	Six1	4E-07	0.2
E2f5	3E-05	0.2	Cdk11b	2E-06	0.2
Zfp523	1E-04	0.2	Mapkapk2	2E-04	0.2
Hoxc6	6E-06	0.2	Ptbp1	7E-06	0.2
Mcrip1	3E-08	0.2	Abitram	3E-04	0.2
Nfic	7E-11	0.2	Ift43	1E-05	0.2
Kmt2b	8E-04	0.2	Csk	2E-04	0.2
Ago1	8E-06	0.2	Eif2ak4	4E-06	0.2
Gm48678	1E-03	0.2	Gspt1	2E-08	0.2
Mfap1a	8E-05	0.2	Lhfp	2E-08	0.2
Phf20l1	3E-07	0.2	Mettl25	5E-05	0.2
Ccnt2	1E-04	0.2	Dcaf7	2E-04	0.2

Chondrogenic

Control			NICD2		
Gene	*p*	FC	Gene	*p*	FC
Msl2	2E-01	0.2	Brms1	3E-01	0.2
Myh9	7E-06	0.2	Relb	6E-01	0.2
Mief1	5E-01	0.2	Scly	8E-01	0.2
2610021A01Rik	6E-01	0.2	Nipal2	5E-01	0.2
Itpkb	1E-01	0.2	Pex11g	9E-01	0.2
Morf4l1	5E-31	0.2	Trim41	3E-01	0.2
Bcar1	3E-01	0.2	Dennd6a	4E-02	0.2
Trim35	1E-05	0.2	Sirt3	3E-01	0.2
Adnp2	3E-01	0.2	Utp6	2E-02	0.2
Dcun1d2	3E-01	0.2	Tapt1	6E-01	0.2
Edem3	2E-01	0.2	Cds2	5E-01	0.2
Appbp2	1E-02	0.2	Ubr3	5E-03	0.2
Smarcad1	3E-03	0.2	Thrap3	6E-08	0.2
Cab39	6E-03	0.2	Mycbp	3E-02	0.2
Naa16	7E-02	0.2	Arhgef17	1E-01	0.2
H13	2E-06	0.2	Max	1E-02	0.2
Zcchc7	2E-07	0.2	Rmdn1	2E-02	0.2
Cacul1	4E-02	0.2	Nek1	3E-01	0.2
Insr	2E-02	0.2	Amd1	3E-01	0.2
Slc25a37	5E-02	0.2	Brwd1	1E-03	0.2
Cdk7	4E-05	0.2	Snai1	5E-01	0.2
Ggct	3E-01	0.2	Polr2b	5E-03	0.2
Mef2b	3E-01	0.2	Msto1	2E-01	0.2
N4bp2l2	9E-06	0.2	Nfs1	2E-02	0.2
Hnrnph1	4E-05	0.2	Egr1	2E-05	0.2
Zfp622	9E-05	0.2	Pnkd	1E-05	0.2
D830024N08Rik	2E-01	0.2	Cd80	6E-01	0.2
Mtmr1	9E-02	0.2	Sec23b	2E-01	0.2
Dus2	3E-01	0.2	Per3	1E-01	0.2
Hltf	1E-03	0.2	Mrps28	8E-04	0.2
Sfxn1	9E-05	0.2	2700046G09Rik	8E-01	0.2
Zhx3	7E-02	0.2	Zc3h6	7E-02	0.2
Dicer1	2E-02	0.2	Pwp2	6E-01	0.2
Dpy19l3	4E-01	0.2	Adipor1	1E-06	0.2
Smim26	1E-03	0.2	Trabd	4E-01	0.2
Polr3e	3E-01	0.2	Ift52	9E-03	0.2
Tceal1	2E-01	0.2	Ubald2	1E-02	0.2
2310039H08Rik	6E-04	0.2	Cep85l	2E-01	0.2
Nsmce3	3E-03	0.2	Wdr41	2E-01	0.2
Rpap1	4E-01	0.2	Gm26797	6E-01	0.2
Sik3	2E-05	0.2	6720427I07Rik	6E-02	0.2
Exoc2	5E-03	0.2	Rybp	9E-03	0.2
Clip3	1E-02	0.2	Plpp6	4E-01	0.2
Sertad2	4E-03	0.2	Cd109	5E-04	0.2
Osmr	9E-03	0.2	Rab3gap2	2E-01	0.2
Gm48383	5E-01	0.2	Ikbkg	4E-01	0.2
Leng9	3E-01	0.2	Dhx32	8E-01	0.2
Flvcr1	6E-01	0.2	1700086O06Rik	4E-01	0.2
Ccdc122	1E-01	0.2	Dpp8	6E-02	0.2
Slc35b2	1E-03	0.2	Mphosph8	4E-04	0.2
Map4k5	2E-02	0.2	AI987944	9E-02	0.2
Sec61b	2E-16	0.2	Golgb1	3E-09	0.2
Btbd9	5E-04	0.2	Pdcl	5E-02	0.2
Dnajc11	1E-01	0.2	Rsf1	4E-04	0.2
Gm15559	5E-01	0.2	Nrd1	2E-04	0.2
Usp42	4E-01	0.2	Ebag9	1E-01	0.2
Mfap1b	3E-02	0.2	Faxc	9E-01	0.2
Plekhg5	2E-02	0.2	Papolg	1E-01	0.2
Nr6a1os	1E+00	0.2	Metap1d	3E-02	0.2
Cisd1	1E-08	0.2	Mtg1	5E-01	0.2
Amfr	7E-03	0.2	Nt5c3	4E-01	0.2
Pinx1	5E-02	0.2	Brat1	3E-01	0.2
Fubp1	7E-09	0.2	Pxmp2	8E-01	0.2
Unc45a	3E-01	0.2	Zbtb11	3E-02	0.2
Ranbp2	8E-05	0.2	Lnx2	8E-01	0.2
Obsl1	4E-01	0.2	Cdk2ap2	5E-05	0.2
Rab11fip3	9E-02	0.2	Lmx1b	8E-01	0.2
Pou6f1	2E-01	0.2	Zhx3	4E-02	0.2
AI480526	4E-01	0.2	Tmem203	2E-01	0.2
1700025G04Rik	3E-02	0.2	Cir1	1E-04	0.2
Ssb	8E-12	0.2	Impa1	2E-01	0.2
Nkiras1	4E-02	0.2	Purb	3E-05	0.2
AW549877	6E-04	0.2	Zmym6	2E-01	0.2

Fibroblast

Control			NICD2		
Gene	*p*	FC	Gene	*p*	FC

Fibroblast?

Control			NICD2		
Gene	*p*	FC	Gene	*p*	FC
Taf9b	4E-08	0.3	Psma1	2E-19	0.3
Enoph1	7E-10	0.3	Fryl	2E-18	0.3
Wdr5b	2E-09	0.3	Slc8b1	1E-04	0.3
Fubp1	2E-19	0.3	Sbsn	3E-03	0.3
E130309D02Rik	3E-10	0.3	Yae1d1	9E-11	0.3
Ccnd3	2E-11	0.3	Pomgnt1	1E-13	0.3
P4ha3	5E-17	0.3	Sap18b	1E-16	0.3
Asnsd1	5E-15	0.3	Bola2	4E-30	0.3
Gnai1	4E-12	0.3	Setd3	3E-14	0.3
Cyth3	1E-12	0.3	Stk35	5E-17	0.3
Ppp4c	2E-13	0.3	Mars	4E-19	0.3
Gpr137b	1E-20	0.3	Hnrnpk	2E-45	0.3
Phlda1	2E-16	0.3	Fam98a	6E-16	0.3
Smco4	7E-14	0.3	Zfp81	6E-21	0.3
Spata13	2E-09	0.3	Ctbp2	2E-17	0.3
Fip1l1	3E-17	0.3	Kat14	3E-15	0.3
Lpin3	2E-03	0.3	Ptpn3	1E-20	0.3
2010204K13Rik	2E-03	0.3	Pfkp	4E-21	0.3
Irf1	7E-17	0.3	Dnajc21	3E-16	0.3
Pde7a	5E-13	0.3	Ccdc43	3E-09	0.3
Gnb2	2E-24	0.3	Map3k1	9E-17	0.3
Oxsr1	8E-09	0.3	Piezo1	3E-20	0.3
4933406I18Rik	4E-15	0.3	A230072C01Rik	3E-03	0.3
Itpr3	1E-11	0.3	Hmg20b	1E-19	0.3
Apoo	1E-15	0.3	Bag2	8E-15	0.3
Fam122a	7E-12	0.3	Cct7	2E-22	0.3
Ahi1	3E-15	0.3	Ehmt2	4E-13	0.3
Ube2m	3E-31	0.3	Phb2	1E-18	0.3
Memo1	6E-09	0.3	Dnajc8	1E-33	0.3
Cmtm4	9E-13	0.3	Ap5s1	3E-11	0.3
Arrb1	6E-09	0.3	Polr3h	1E-13	0.3
Gm15886	1E-06	0.3	Bcl10	2E-14	0.3
Cdkn2a	2E-25	0.3	Nudt22	1E-16	0.3
Rps6ka5	9E-24	0.3	Med8	6E-18	0.3
Gm31645	9E-07	0.3	Faap100	4E-15	0.3
Phlpp2	3E-13	0.3	Retreg2	7E-16	0.3
Bicd1	7E-09	0.3	Napepld	3E-13	0.3
Pum2	2E-13	0.3	Acer3	1E-14	0.3
Mrpl55	1E-11	0.3	Samhd1	1E-22	0.3
Gpd2	4E-14	0.3	Cdk19	6E-18	0.3
Stox2	6E-05	0.3	Klf7	6E-15	0.3
Flna	2E-16	0.3	St7	1E-17	0.3
B4galnt1	8E-07	0.3	Vav2	7E-11	0.3
Qdpr	9E-07	0.3	Ptp4a2	8E-43	0.3
Fam162a	2E-19	0.3	Apbb1ip	2E-11	0.3
Arntl	7E-10	0.3	Fbxl14	4E-12	0.3
Samd9l	3E-08	0.3	Zc3h14	7E-17	0.3
Syt12	1E-14	0.3	Lrrc17	1E-07	0.3
Nufip1	4E-14	0.3	Trappc5	2E-14	0.3
4930447N08Rik	2E-07	0.3	Naa16	4E-17	0.3
Ptp4a2	9E-33	0.3	Pycrl	2E-15	0.3
Erc1	6E-14	0.3	Thbd	4E-17	0.3
Nudcd2	1E-20	0.3	1500011B03Rik	1E-17	0.3
Cops7b	4E-10	0.3	Sh2b3	5E-12	0.3
Ppp1r12c	3E-12	0.3	Pdcd5	1E-41	0.3
Bdnf	4E-14	0.3	Nsmce3	4E-17	0.3
Wdr1	7E-15	0.3	Igfbp2	2E-13	0.3
Plaat1	1E-06	0.3	Dctd	1E-12	0.3
Zfp35	3E-10	0.3	Pwwp3b	2E-16	0.3
Tmem19	1E-11	0.3	Dhx32	1E-13	0.3
Peg10	3E-10	0.3	Ankrd13a	1E-18	0.3
Tcp11l1	5E-08	0.3	Acy3	1E-08	0.3
Ogfr	5E-10	0.3	Gpd2	9E-20	0.3
Nup214	3E-12	0.3	Ofd1	3E-14	0.3
Pop1	7E-11	0.3	Ilk	4E-14	0.3
Pde8a	1E-15	0.3	Plxnb1	5E-12	0.3
Lama5	3E-05	0.3	Fjx1	3E-12	0.3
Acap2	2E-15	0.3	Galns	3E-14	0.3
Sh3kbp1	6E-13	0.3	Setdb1	1E-12	0.3
Mex3b	3E-08	0.3	Rnf4	7E-14	0.3
Mrps7	6E-14	0.3	Pdzd8	7E-17	0.3
Anapc1	6E-14	0.3	Rbm22	6E-14	0.3
Eif3b	2E-13	0.3	Bud23	6E-13	0.3

Undefined

Control			NICD2		
Gene	*p*	FC	Gene	*p*	FC

Articular/Synovial Fibroblast

Control			NICD2		
Gene	*p*	FC	Gene	*p*	FC
Ncoa3	5E-01	0.1	Dcps	1E+00	0.1
S100a11	4E-08	0.1	R74862	3E-01	0.1
2610021A01Rik	2E-01	0.1	Tbcc	6E-01	0.1
Ankrd16	2E-01	0.1	Kif3b	7E-01	0.1
2810032G03Rik	6E-01	0.1	Prr3	2E-01	0.1
Zfp688	5E-01	0.1	Ddx58	1E-01	0.1
Ninl	9E-01	0.1	E130308A19Rik	4E-01	0.1
Rp2	7E-01	0.1	Tusc1	4E-02	0.1
Coa3	6E-04	0.1	Hsd17b4	3E-01	0.1
Zfp709	4E-01	0.1	Rpl28	3E-13	0.1
Elf2	7E-02	0.1	Trap1	9E-01	0.1
Bcl2l13	5E-01	0.1	Erlin2	4E-01	0.1
Casp2	2E-01	0.1	D130017N08Rik	3E-01	0.1
Itgb3bp	1E-01	0.1	Tm7sf3	2E-01	0.1
Zfp414	5E-01	0.1	Dand5	4E-01	0.1
Lrrcc1	8E-01	0.1	Ptgr2	8E-01	0.1
Spcs3	8E-01	0.1	Faxc	1E-01	0.1
Dock1	3E-03	0.1	Crtap	5E-03	0.1
Elovl1	9E-01	0.1	Hebp1	3E-01	0.1
Hlcs	4E-01	0.1	Tmem150a	1E+00	0.1
Ehmt2	8E-01	0.1	Psen2	1E+00	0.1
Dmac2l	3E-01	0.1	Bckdha	5E-01	0.1
Ubn1	1E-03	0.1	Scamp3	5E-01	0.1
Zfand2b	5E-01	0.1	Mrps36	3E-02	0.1
Mrpl45	5E-01	0.1	As3mt	5E-01	0.1
Gm4258	3E-01	0.1	Amacr	4E-01	0.1
Glb1	2E-01	0.1	Pdia4	2E-03	0.1
Trappc6a	1E-01	0.1	Stag2	8E-02	0.1
Lpin1	7E-01	0.1	Scarb1	6E-01	0.1
Ak1	1E-01	0.1	Bivm	1E-01	0.1
Atxn1	3E-02	0.1	Dtd2	9E-01	0.1
Bvht	8E-02	0.1	Dnlz	5E-02	0.1
Cd44	9E-01	0.1	Vopp1	1E-01	0.1
Cd109	1E-01	0.1	Elk3	7E-01	0.1
Anapc13	2E-03	0.1	Rnf38	4E-01	0.1
Rhoj	1E-01	0.1	Fam129a	1E-01	0.1
Pdpk1	5E-01	0.1	Acyp1	1E-01	0.1
Ppp1r16a	2E-01	0.1	Hsdl2	1E+00	0.1
Hmgxb3	4E-01	0.1	Scamp2	1E+00	0.1
Hgs	5E-01	0.1	Mageh1	4E-01	0.1
Camk1	3E-02	0.1	2700049A03Rik	4E-01	0.1
Chkb	2E-01	0.1	Eif4b	1E-03	0.1
Smug1	3E-01	0.1	Bnip3	1E-01	0.1
Ocrl	4E-01	0.1	Rhod	7E-01	0.1
Gprasp1	8E-01	0.1	Hps6	4E-01	0.1
Mfn1	8E-01	0.1	Proser3	2E-01	0.1
2700049A03Rik	3E-01	0.1	Map4k4	9E-02	0.1
Borcs8	8E-02	0.1	Golph3l	8E-01	0.1
Cdyl2	8E-01	0.1	Ccnk	7E-01	0.1
Rnf145	8E-01	0.1	Kmt2e	4E-03	0.1
Ist1	3E-01	0.1	Ap4e1	3E-01	0.1
Cep162	6E-01	0.1	Coro7	5E-01	0.1
Lgals8	2E-01	0.1	Tcirg1	2E-01	0.1
Zfp961	4E-01	0.1	Ripor1	6E-01	0.1
Gm9993	5E-01	0.1	Polrmt	3E-01	0.1
Morc4	8E-01	0.1	Bnip3l	2E-02	0.1
Ipmk	2E-01	0.1	Rufy2	7E-01	0.1
Osmr	7E-01	0.1	Eif2ak2	4E-02	0.1
Heatr6	5E-01	0.1	Zfp943	6E-01	0.1
Oga	6E-01	0.1	Gpr19	1E-01	0.1
Tmx3	5E-02	0.1	Acaa1b	7E-01	0.1
Fig4	9E-01	0.1	Zfp160	8E-01	0.1
Gm16845	3E-01	0.1	Sik2	4E-01	0.1
Chst12	6E-01	0.1	Kdm5a	3E-01	0.1
Ccdc57	3E-01	0.1	Rab13	2E-01	0.1
1110002L01Rik	8E-01	0.1	Prpsap2	3E-01	0.1
Ermard	4E-01	0.1	Tcaf1	2E-01	0.1
Gm4221	7E-01	0.1	Lmtk2	3E-01	0.1
Serf2	2E-14	0.1	Flii	2E-01	0.1
Slc38a10	2E-01	0.1	Ttc21b	9E-01	0.1
Zfp865	9E-02	0.1	2210408F21Rik	1E+00	0.1
Rpl28	2E-16	0.1	Nck2	8E-01	0.1
Cdc42ep5	7E-03	0.1	Tbc1d12	8E-01	0.1

Limb Mesenchyme					
Control			NICD2		
Gene	p	FC	Gene	p	FC
Hdhd2	4E-06	0.2	St7	1E-05	0.2
Armc6	4E-03	0.2	Tgoln1	2E-06	0.2
Nt5m	2E-05	0.2	G6pc3	5E-04	0.2
Mlh3	5E-04	0.2	Traf2	4E-03	0.2
Wdfy3	1E-07	0.2	Stip1	2E-06	0.2
Nom1	1E-04	0.2	Dennd6a	5E-05	0.2
Ing4	3E-08	0.2	Hectd1	6E-08	0.2
Oxsr1	6E-05	0.2	Bms1	5E-05	0.2
Ccdc91	8E-06	0.2	Mphosph6	6E-05	0.2
Rgs12	8E-06	0.2	Mettl2	4E-04	0.2
Srprb	8E-06	0.2	Prkdc	1E-04	0.2
Dnajc2	5E-07	0.2	Kmt2c	9E-05	0.2
Rsrc1	2E-11	0.2	Foxj2	3E-04	0.2
Actl6a	2E-05	0.2	Col9a1	1E-21	0.2
Rbm6	1E-09	0.2	B3galnt2	2E-03	0.2
Plod1	1E-08	0.2	Slc37a3	6E-05	0.2
4930557K07Rik	3E-03	0.2	Eif5b	3E-10	0.2
Nipbl	8E-10	0.2	Ube3c	2E-06	0.2
Msl2	3E-05	0.2	Fads3	2E-04	0.2
Phf6	5E-06	0.2	Phf21a	2E-07	0.2
Slc4a1ap	4E-06	0.2	Dmtf1	2E-04	0.2
Dyrk1a	2E-05	0.2	Uba3	3E-04	0.2
Raph1	7E-09	0.2	Med26	2E-03	0.2
Armcx6	1E-05	0.2	Tmem17	2E-04	0.2
Zfp316	2E-04	0.2	Ttc27	2E-05	0.2
Pou6f1	2E-05	0.2	1110004F10Rik	1E-12	0.2
Exoc3	4E-08	0.2	Klhl42	4E-02	0.2
Ttc23	1E-05	0.2	Urb2	3E-02	0.2
Dedd	2E-04	0.2	Bin3	2E-04	0.2
Osbpl5	3E-06	0.2	D17H6S53E	2E-02	0.2
Bivm	6E-04	0.2	Pno1	2E-04	0.2
Etf1	2E-08	0.2	Naa16	1E-02	0.2
Rnf146	4E-06	0.2	Ube4b	9E-07	0.2
Mical3	5E-06	0.2	Snrpb2	4E-05	0.2
Cdc37	5E-12	0.2	Copb1	1E-10	0.2
Armc5	4E-04	0.2	Xpc	5E-04	0.2
Atg3	1E-07	0.2	Ahctf1	7E-04	0.2
Zup1	8E-06	0.2	Snf8	1E-07	0.2
Cttn	3E-09	0.2	Ngly1	6E-04	0.2
Mettl7a1	9E-09	0.2	5730455P16Rik	4E-05	0.2
Efna1	4E-02	0.2	Zdhhc16	6E-04	0.2
Gng5	6E-27	0.2	Rab4a	2E-03	0.2
Prpf4b	5E-09	0.2	Tyw3	7E-02	0.2
Socs4	3E-04	0.2	Creld2	8E-05	0.2
Ift52	2E-05	0.2	Supt20	2E-05	0.2
Cttnbp2nl	5E-05	0.2	Fastkd1	4E-03	0.2
Rfesd	1E-05	0.2	Zfp334	2E-04	0.2
Cyth1	3E-03	0.2	Rbm18	6E-05	0.2
2510039O18Rik	4E-06	0.2	Uqcc1	2E-03	0.2
Pprc1	3E-05	0.2	Atp2a2	2E-07	0.2
Tspan9	1E-06	0.2	Cops3	3E-06	0.2
Snrpd1	1E-15	0.2	Zscan29	5E-03	0.2
Riok2	2E-06	0.2	Pja2	1E-06	0.2
Fbrs	2E-04	0.2	Drg2	4E-03	0.2
Pi4k2b	4E-04	0.2	Cdk13	8E-06	0.2
Fgf2	1E-04	0.2	Zfp317	2E-05	0.2
Ubr1	3E-06	0.2	U2af2	6E-06	0.2
Psmc6	3E-11	0.2	Ankrd28	2E-06	0.2
Rbm26	5E-08	0.2	Far1	4E-04	0.2
Nufip2	1E-07	0.2	Zfp639	2E-05	0.2
Rnps1	2E-08	0.2	Ruvbl1	1E-05	0.2
Ube2d2a	2E-09	0.2	Brf1	3E-03	0.2
Rab4b	1E-07	0.2	Ypel2	8E-05	0.2
Rfxap	2E-04	0.2	Btbd1	3E-07	0.2
Ltv1	7E-05	0.2	Npc1	5E-05	0.2
Stard13	3E-08	0.2	Arhgap1	1E-04	0.2
Apex2	2E-06	0.2	Nacc1	8E-04	0.2
D2hgdh	1E-03	0.2	Kdm2b	1E-04	0.2
Myl12a	2E-09	0.2	Mllt11	2E-03	0.2
Ptcd3	2E-04	0.2	Parp6	1E-02	0.2
Mtfr1l	2E-06	0.2	Huwe1	3E-08	0.2
Nap1l4	2E-08	0.2	Micu1	2E-05	0.2
Ufsp2	1E-07	0.2	Pitpnc1	1E-06	0.2

Chondrogenic					
Control			NICD2		
Gene	p	FC	Gene	p	FC
Aagab	3E-01	0.2	Itpkb	3E-01	0.2
Slc39a9	2E-01	0.2	Gnb1l	9E-01	0.2
Cog8	4E-02	0.2	Wdr13	4E-01	0.2
Clptm1l	5E-06	0.2	Krr1	3E-01	0.2
Dcaf5	1E-02	0.2	Pinx1	6E-02	0.2
Wfs1	3E-01	0.2	Snw1	6E-07	0.2
Prorp	1E-01	0.2	Rrnad1	7E-01	0.2
Sh3glb2	2E-02	0.2	Slc38a2	3E-06	0.2
Rbbp4	4E-10	0.2	Pja2	1E-05	0.2
Rnps1	3E-05	0.2	Rnpep	1E-01	0.2
Mbd5	6E-07	0.2	Mtx3	8E-01	0.2
Wls	7E-08	0.2	Uck2	2E-01	0.2
Golga2	1E-02	0.2	Unc50	2E-04	0.2
Mosmo	5E-03	0.2	Gar1	4E-02	0.2
Hmbox1	2E-02	0.2	Pnmal2	5E-01	0.2
Trpm7	1E-05	0.2	Tbl1x	1E-03	0.2
Itgb5	3E-07	0.2	Rpn2	1E-06	0.2
Abtb1	8E-01	0.2	Tyw5	4E-01	0.2
Gm26672	3E-01	0.2	2210408I21Rik	4E-01	0.2
Map2	7E-04	0.2	Ppp1r8	4E-01	0.2
Ate1	3E-02	0.2	Mterf4	4E-01	0.2
AC154200.1	2E-01	0.2	Crot	5E-02	0.2
Tbc1d19	2E-02	0.2	Rbm26	1E-04	0.2
Ears2	6E-01	0.2	Zbtb11os1	4E-01	0.2
Erich1	7E-02	0.2	Zmiz2	6E-01	0.2
Rab11b	3E-07	0.2	Ublcp1	1E-02	0.2
Hus1	1E+00	0.2	Cmpk1	1E-07	0.2
Arf2	1E-02	0.2	Wdr83os	8E-08	0.2
1110002L01Rik	7E-02	0.2	Ptdss1	5E-01	0.2
Son	1E-15	0.2	Cgref1	5E-02	0.2
Sgf29	2E-01	0.2	Pdia6	1E-12	0.2
Itm2c	1E-06	0.2	Irak4	8E-01	0.2
Hfe	8E-02	0.2	Anxa2	1E-14	0.2
Ago3	2E-02	0.2	Nipbl	3E-06	0.2
Yipf6	3E-02	0.2	Nmt2	2E-01	0.2
Hoxc8	4E-01	0.2	Pnrc2	6E-05	0.2
Mterf3	1E-02	0.2	Zbtb7b	7E-01	0.2
Mettl25	2E-01	0.2	Ppie	4E-02	0.2
Tcp11l1	9E-02	0.2	Dok1	5E-01	0.2
Pik3r1	6E-04	0.2	Fam13c	2E-02	0.2
Cuedc1	4E-03	0.2	Srp14	2E-15	0.2
Cdc37l1	3E-04	0.2	Bcl7b	2E-02	0.2
Mink1	2E-01	0.2	Celf1	8E-05	0.2
Polr2b	8E-04	0.2	Pi4k2a	2E-01	0.2
Dnajb2	7E-02	0.2	Cyp4f13	8E-01	0.2
Rbm22	9E-04	0.2	Tprgl	5E-02	0.2
Ppfibp1	8E-03	0.2	Steap2	1E-01	0.2
Clpx	3E-03	0.2	Sco1	6E-01	0.2
Xpo7	3E-04	0.2	Pccb	1E-01	0.2
Mast3	3E-01	0.2	Arhgef6	2E-01	0.2
Alg14	2E-03	0.2	Uchl3	7E-04	0.2
Arhgef11	2E-03	0.2	Srr	3E-01	0.2
Pebp1	2E-18	0.2	Zfp369	5E-01	0.2
Rsrc1	3E-10	0.2	Ssb	7E-12	0.2
Cnbd2	6E-02	0.2	Glo1	8E-07	0.2
Skiv2l	1E-01	0.2	Gemin8	1E+00	0.2
Dync2li1	4E-03	0.2	Akr7a5	6E-02	0.2
Kdm4b	4E-01	0.2	Swt1	2E-01	0.2
Pot1b	1E-01	0.2	Spout1	7E-01	0.2
Trim68	6E-01	0.2	Ptcd2	3E-01	0.2
Tada2a	6E-02	0.2	Agk	6E-01	0.2
Sphk2	5E-01	0.2	4933421O10Rik	9E-01	0.2
Lin7c	2E-03	0.2	Plbd2	4E-04	0.2
Cox16	5E-11	0.2	Mllt6	7E-01	0.2
Snap47	3E-05	0.2	Pik3c3	9E-01	0.2
Rsf1	8E-05	0.2	Ttc1	3E-03	0.2
Tbc1d32	2E-01	0.2	Zdhhc5	3E-02	0.2
mt-Nd4	4E-25	0.2	Rel	9E-01	0.2
Slc12a4	2E-02	0.2	Noc3l	8E-02	0.2
Ptpn3	5E-01	0.2	Rsbn1	2E-01	0.2
Usp32	4E-02	0.2	Tsnax	2E-03	0.2
Ift172	3E-01	0.2	Vps72	8E-03	0.2
Fam118b	3E-01	0.2	Tmem260	1E-01	0.2

Fibroblast					
Control			NICD2		
Gene	p	FC	Gene	p	FC

Fibroblast?					
Control			NICD2		
Gene	p	FC	Gene	p	FC
Hmces	2E-13	0.3	Ntn1	1E-13	0.3
Sdhd	2E-10	0.3	Haus2	1E-17	0.3
Slc35d1	8E-11	0.3	Exosc9	7E-20	0.3
Cops6	4E-22	0.3	Rhot2	7E-13	0.3
Ddx19a	4E-14	0.3	Ppig	5E-35	0.3
Ankrd49	1E-13	0.3	Plekha3	8E-14	0.3
Zc3h11a	9E-05	0.3	Edc4	1E-16	0.3
Polr2d	4E-13	0.3	Il17rd	1E-07	0.3
Zfp961	1E-11	0.3	Stx12	4E-18	0.3
Brcc3	2E-15	0.3	Slc2a9	1E-07	0.3
Cyb5r3	7E-23	0.3	Fam220a.1	5E-05	0.3
Mprip	1E-18	0.3	Rps6kb2	2E-16	0.3
Ercc5	5E-06	0.3	Purb	6E-19	0.3
Zfp275	8E-13	0.3	Ciapin1	3E-15	0.3
Gars	1E-15	0.3	Actr1b	6E-11	0.3
Higd1a	4E-26	0.3	Tbp	6E-17	0.3
Ppig	2E-22	0.3	Dmwd	9E-14	0.3
Mtrex	6E-11	0.3	Capn2	4E-17	0.3
Zscan22	9E-10	0.3	Pi4k2b	6E-15	0.3
Cd2bp2	3E-11	0.3	Aifm1	4E-19	0.3
Nab1	1E-08	0.3	Fbl	6E-12	0.3
Npas2	7E-09	0.3	Tesk2	6E-15	0.3
Ints9	4E-12	0.3	Mark1	7E-14	0.3
Glrx5	8E-21	0.3	Ifi35	1E-10	0.3
Prpf38a	1E-11	0.3	Itpr3	4E-12	0.3
Psma1	1E-12	0.3	Gid8	1E-10	0.3
Pgm2	3E-10	0.3	4930473A02Rik	2E-07	0.3
Crkl	7E-14	0.3	Arhgdia	7E-32	0.3
Rogdi	5E-11	0.3	Tnpo3	3E-13	0.3
Syn1	3E-06	0.3	Cacna1b	3E-10	0.3
Hsph1	2E-11	0.3	Gabpb1	1E-13	0.3
Cystm1	4E-06	0.3	Dele1	2E-15	0.3
Als2	7E-09	0.3	Suds3	2E-12	0.3
Edem1	4E-13	0.3	Chd1	3E-16	0.3
Zbtb25	2E-08	0.3	Zfp346	8E-16	0.3
Ptpdc1	4E-10	0.3	Apoo	1E-14	0.3
Nexn	1E-12	0.3	Ddx59	9E-12	0.3
Gspt1	2E-19	0.3	Atp1b3	5E-31	0.3
Dpysl2	2E-15	0.3	Rprd1a	1E-11	0.3
Gcdh	2E-08	0.3	2310014F06Rik	5E-17	0.3
Fmr1	6E-28	0.3	B4galt6	1E-17	0.3
2500002B13Rik	7E-08	0.3	Trappc13	4E-17	0.3
Riox2	5E-15	0.3	Micall2	2E-10	0.3
Adgrg6	2E-09	0.3	Zfp217	4E-10	0.3
Rhbdd2	1E-13	0.3	Gm50163	1E-07	0.3
Slc22a4	7E-08	0.3	Snrpd3	9E-25	0.3
Slc39a6	1E-13	0.3	Mak16	5E-09	0.3
Sybu	2E-10	0.3	Lrch4	6E-14	0.3
1810055G02Rik	1E-13	0.3	Bud31	1E-12	0.3
Ubqln4	9E-11	0.3	Ccdc122	1E-18	0.3
Hnrnpul2	4E-11	0.3	Rtca	5E-18	0.3
Stk16	6E-11	0.3	Dipk1a	7E-17	0.3
Riox1	2E-07	0.3	Nipsnap3b	4E-15	0.3
Stx12	1E-14	0.3	Coq10b	6E-15	0.3
Nol9	6E-07	0.3	Ttc13	2E-12	0.3
Rnf217	1E-09	0.3	Tgfbr1	2E-18	0.3
Oip5os1	3E-14	0.3	Pno1	3E-17	0.3
Rngtt	1E-12	0.3	Dpagt1	2E-16	0.3
Ece2	3E-12	0.3	Tmem231	2E-05	0.3
Vill	3E-10	0.3	Ubtf	2E-17	0.3
Ppp4r1	3E-11	0.3	Spire1	5E-15	0.3
Cnot10	3E-12	0.3	Epb41l2	2E-20	0.3
Chchd1	6E-18	0.3	Commd1	5E-26	0.3
Grb14	4E-04	0.3	Zfp958	4E-15	0.3
Zbtb1	3E-06	0.3	Gsdmd	6E-06	0.3
Traf2	7E-10	0.3	Mms19	7E-17	0.3
Ppp1r16a	2E-07	0.3	Pdhx	2E-17	0.3
Cptp	2E-09	0.3	Mrpl19	4E-11	0.3
Spry1	5E-04	0.3	Dimt1	5E-15	0.3
Sestd1	5E-15	0.3	Cnot11	9E-12	0.3
Dag1	1E-02	0.3	Dnajb3	1E-08	0.3
Tbp	3E-12	0.3	Wbp4	9E-20	0.3
Mgat4a	8E-08	0.3	Phldb1	3E-14	0.3

Undefined					
Control			NICD2		
Gene	p	FC	Gene	p	FC

Articular/Synovial Fibroblast					
Control			NICD2		
Gene	p	FC	Gene	p	FC
Ghr	4E-05	0.1	Cspp1	3E-01	0.1
Ercc4	2E-01	0.1	Zfp647	4E-01	0.1
Slc35b3	7E-01	0.1	Epm2aip1	8E-01	0.1
Hdac8	2E-01	0.1	Os9	3E-02	0.1
Dcaf17	1E+00	0.1	Mafk	3E-01	0.1
Tmem117	4E-01	0.1	Rapgef1	9E-01	0.1
Atf2	7E-01	0.1	Scarb2	5E-01	0.1
Lonp2	2E-02	0.1	Lrrcc1	9E-01	0.1
Tmem243	1E+00	0.1	Dcaf17	6E-01	0.1
Rlf	3E-01	0.1	5430405H02Rik	6E-01	0.1
Gm17382	3E-01	0.1	2010001A14Rik	2E-01	0.1
5031425E22Rik	9E-03	0.1	Efna4	7E-01	0.1
Vps18	3E-01	0.1	Tctex1d2	6E-01	0.1
Anxa3	1E+00	0.1	Zfp955a	1E-01	0.1
Cacul1	8E-01	0.1	Pms1	3E-01	0.1
Rab22a	3E-02	0.1	Stx18	6E-01	0.1
Rfx7	2E-01	0.1	Sap130	4E-01	0.1
Ube2v1	7E-02	0.1	Nprl2	3E-01	0.1
Tmem87b	4E-01	0.1	Zfp384	3E-01	0.1
Mtrf1	2E-01	0.1	Slc25a26	3E-01	0.1
Zfp444	3E-01	0.1	Kansl1l	4E-01	0.1
Sfi1	4E-01	0.1	Pan3	2E-01	0.1
Slc12a9	2E-01	0.1	Smarca2	3E-02	0.1
Sh3kbp1	9E-02	0.1	Atp6v1f	8E-04	0.1
Hoxc9	5E-01	0.1	Wdsub1	7E-01	0.1
Taco1os	5E-01	0.1	2810002D19Rik	6E-01	0.1
Kif16b	8E-01	0.1	Atp6v1d	1E-01	0.1
Slc25a27	4E-01	0.1	Scyl2	6E-01	0.1
Ndufa3	5E-05	0.1	Elac2	2E-01	0.1
Map6	8E-01	0.1	Proca1	4E-01	0.1
Ascc3	2E-01	0.1	Snx1	3E-01	0.1
mt-Nd4l	4E-06	0.1	Stn1	7E-01	0.1
Wasl	3E-01	0.1	Gm1673	6E-02	0.1
Adgrl2	3E-01	0.1	Ubald2	6E-01	0.1
Stk11	3E-01	0.1	Ghdc	5E-01	0.1
Phkb	3E-01	0.1	Kdm2a	3E-01	0.1
E430024I08Rik	6E-01	0.1	Zfp157	3E-01	0.1
Psenen	6E-04	0.1	Hps1	6E-01	0.1
Abcg2	1E+00	0.1	Mllt3	4E-01	0.1
Zfp697	5E-01	0.1	Rrnad1	2E-01	0.1
Meis2	1E+00	0.1	Ivd	8E-01	0.1
Zfp938	4E-01	0.1	Dipk1b	8E-01	0.1
Plp2	4E-01	0.1	Adprm	6E-01	0.1
H2afj	9E-05	0.1	Cetn4	3E-01	0.1
Cstf1	1E-01	0.1	Pknox1	3E-01	0.1
Rnf185	7E-01	0.1	Slc49a4	6E-01	0.1
Rpp38	5E-01	0.1	Znhit3	9E-01	0.1
Kri1	2E-01	0.1	Pml	1E+00	0.1
Dnajb4	4E-01	0.1	Aspscr1	4E-01	0.1
Tmc6	4E-01	0.1	Cep250	9E-01	0.1
Trip10	1E+00	0.1	Cep170b	9E-01	0.1
Usp21	2E-01	0.1	Gm42418	3E-12	0.1
Hsd17b7	5E-01	0.1	Zyx	7E-01	0.1
Rbm41	9E-01	0.1	Chm	6E-01	0.1
Dnal4	2E-01	0.1	Uevld	9E-01	0.1
Ppard	1E+00	0.1	Stam	8E-01	0.1
Rbm5	2E-02	0.1	Tmem60	5E-01	0.1
Ttc33	4E-01	0.1	Zup1	8E-01	0.1
Zfp950	3E-01	0.1	Ifngr1	8E-01	0.1
Srsf5	2E-04	0.1	Mpnd	2E-01	0.1
Tgfbr2	3E-04	0.1	Ercc5	9E-01	0.1
Arid1b	2E-01	0.1	Ppm1m	4E-01	0.1
AI480526	1E-01	0.1	Lama4	2E-02	0.1
Rps28	9E-16	0.1	Rreb1	1E-01	0.1
1110059G10Rik	1E-01	0.1	Mar7	4E-01	0.1
Hoxc6	6E-01	0.1	Ccdc68	6E-01	0.1
Ndufs7	1E-03	0.1	Rnf103	7E-01	0.1
Ccnt2	4E-01	0.1	Dlc1	2E-04	0.1
Mfap1b	6E-01	0.1	Gm19412	5E-01	0.1
Zmym1	3E-01	0.1	Proser1	2E-01	0.1
Tmco6	3E-01	0.1	Tuba1c	1E-01	0.1
Efcab7	3E-01	0.1	Rwdd3	2E-01	0.1
Trim56	4E-01	0.1	B230217C12Rik	6E-01	0.1

Limb Mesenchyme					
Control			NICD2		
Gene	p	FC	Gene	p	FC
Zfp106	2E-09	0.2	Gm14798	1E-02	0.2
Zcchc4	3E-04	0.2	Col6a1	2E-07	0.2
2410131K14Rik	2E-05	0.2	Dnaja2	2E-11	0.2
Tasor2	1E-04	0.2	Fbxl17	3E-06	0.2
Twistnb	2E-05	0.2	Riox2	1E-03	0.2
Afdn	8E-07	0.2	Srprb	2E-04	0.2
Zbtb21	7E-03	0.2	Pygo2	5E-04	0.2
Gnpnat1	3E-06	0.2	Mbd1	8E-04	0.2
Atpaf1	1E-04	0.2	Zmpste24	4E-05	0.2
Rpl7l1	1E-06	0.2	Ythdf1	1E-04	0.2
Nck1	2E-06	0.2	Vamp7	6E-05	0.2
Rps19bp1	4E-06	0.2	Dennd4a	2E-07	0.2
Ric1	2E-05	0.2	Ccn2	2E-20	0.2
Arhgdig	1E-02	0.2	Pcgf2	6E-05	0.2
Dancr	3E-04	0.2	Rab11fip3	1E-03	0.2
Ankrd17	2E-08	0.2	Tomm20	3E-14	0.2
Zbtb1	1E-05	0.2	Zfp365	5E-04	0.2
Fyn	4E-06	0.2	Msl1	1E-03	0.2
Acyp1	2E-05	0.2	Ankrd52	7E-04	0.2
Polb	7E-06	0.2	Dot1l	3E-04	0.2
Naaladl2	8E-11	0.2	Srbd1	1E-05	0.2
Anapc5	5E-11	0.2	R3hdm2	3E-07	0.2
Rragb	8E-04	0.2	Plekha3	3E-04	0.2
Zmynd19	2E-04	0.2	Rars	4E-06	0.2
Tmed7	4E-07	0.2	Nr2c2	3E-04	0.2
Pi4k2a	8E-06	0.2	Jcad	2E-05	0.2
Os9	7E-09	0.2	Ube2v2	2E-07	0.2
Raf1	1E-05	0.2	Elk4	3E-04	0.2
Zfp950	8E-06	0.2	Top3b	1E-05	0.2
Nphp1	3E-04	0.2	Ubn2	5E-07	0.2
Cep170	1E-06	0.2	Hnrnpa3	4E-11	0.2
Brat1	5E-03	0.2	Os9	4E-08	0.2
Prpf4	2E-04	0.2	Gde1	1E-05	0.2
Psme4	4E-08	0.2	Herc1	1E-03	0.2
Ldlrap1	8E-05	0.2	Tnfaip1	6E-05	0.2
Fam53a	6E-05	0.2	Tatdn1	4E-05	0.2
Atp11b	7E-05	0.2	Atxn7l3b	8E-12	0.2
Sirt6	2E-03	0.2	Tti2	1E-03	0.2
Actr3	6E-09	0.2	Thg1l	2E-04	0.2
Mdfic	2E-06	0.2	Pknox1	1E-02	0.2
Rnd2	2E-02	0.2	Rnf6	4E-05	0.2
Maff	2E-02	0.2	Agfg1	1E-05	0.2
Xrn2	4E-08	0.2	Tnrc6b	8E-08	0.2
Usp7	2E-05	0.2	Gm47863	4E-03	0.2
Slc16a7	8E-03	0.2	Uqcrq	1E-15	0.2
Zfp707	1E-02	0.2	Eif2s1	4E-07	0.2
Zfp317	1E-03	0.2	Tfcp2	5E-05	0.2
Cbfb	2E-06	0.2	Tbc1d20	8E-06	0.2
Aen	3E-06	0.2	Prpf40a	1E-08	0.2
Tdp1	1E-03	0.2	Tmem42	2E-03	0.2
Hint3	3E-04	0.2	Dnal1	7E-04	0.2
Nlk	8E-06	0.2	Uvrag	8E-05	0.2
Wipi2	6E-04	0.2	Sfxn4	2E-02	0.2
Cdh2	4E-15	0.2	Ddx46	2E-06	0.2
Gpatch3	1E-02	0.2	Chtf8	2E-02	0.2
Kif5c	1E-02	0.2	Pard6a	2E-05	0.2
Kpna4	2E-08	0.2	Rmdn3	4E-03	0.2
Dhx15	2E-06	0.2	Asb1	2E-02	0.2
Zdhhc16	8E-04	0.2	B230219D22Rik	2E-08	0.2
Rrn3	2E-05	0.2	Lancl2	2E-02	0.2
Slc25a30	2E-03	0.2	Srsf3	5E-10	0.2
Trp53bp1	5E-05	0.2	Tmem199	1E-04	0.2
Atf2	7E-06	0.2	Dcaf10	4E-04	0.2
Zfp281	3E-05	0.2	Nedd4	2E-21	0.2
Btbd1	5E-06	0.2	Srsf1	2E-06	0.2
Nkiras1	5E-06	0.2	Tcf25	3E-11	0.2
Gopc	5E-05	0.2	Lnpep	1E-04	0.2
Usp32	5E-05	0.2	Utp4	2E-03	0.2
Ybx3	6E-09	0.2	Rcn3	9E-11	0.2
St7	5E-06	0.2	Gatad2b	2E-05	0.2
Slc30a4	3E-04	0.2	Hist1h1c	5E-06	0.2
Tardbp	3E-06	0.2	Cbfa2t2	2E-04	0.2
Slc25a40	9E-05	0.2	Bckdha	1E-05	0.2

Chondrogenic					
Control			NICD2		
Gene	p	FC	Gene	p	FC
Mef2a	1E-04	0.2	Dnal4	3E-01	0.2
Selenof	1E-19	0.2	mt-Nd4	4E-28	0.2
Kdm5a	2E-04	0.2	Sec61a2	4E-01	0.2
Aldh18a1	3E-02	0.2	Plcd3	1E+00	0.2
Etnk1	8E-03	0.2	Mrpl24	9E-05	0.2
Ppp6r3	5E-04	0.2	Bsdc1	3E-01	0.2
Hist2h4	8E-01	0.2	Tatdn2	8E-01	0.2
Xrcc6	8E-02	0.2	5730480H06Rik	2E-01	0.2
Atp23	4E-01	0.2	Spaca6	2E-02	0.2
Glt8d1	1E-04	0.2	Dusp12	8E-02	0.2
Naxd	1E-05	0.2	Rnf185	5E-01	0.2
Naa35	4E-03	0.2	Sod2	5E-05	0.2
Tsc22d4	4E-08	0.2	Wipf2	5E-01	0.2
Mpv17l	1E-01	0.2	Trpm7	5E-04	0.2
A930037H05Rik	5E-01	0.2	4933407K13Rik	7E-01	0.2
Dnajb11	2E-07	0.2	Camk2n1	3E-01	0.2
Dhx35	6E-01	0.2	Hmgcl	6E-01	0.2
Paxx	1E-02	0.2	Uty	6E-02	0.2
Bscl2	5E-05	0.2	Sfxn2	9E-01	0.2
Car11	4E-01	0.2	Vps37c	2E-01	0.2
Dnajc16	2E-01	0.2	Prkdc	2E-02	0.2
Ganc	1E-01	0.2	Zfp963	8E-01	0.2
AI987944	6E-01	0.2	Atp6v1g1	6E-06	0.2
Camk2n1	8E-01	0.2	Canx	1E-09	0.2
Jmjd1c	1E-06	0.2	Zfp182	8E-01	0.2
Zdhhc20	4E-02	0.2	Ifitm2	4E-07	0.2
Capn15	6E-02	0.2	Trappc11	5E-01	0.2
Adcy2	1E-02	0.2	Ar	5E-02	0.2
C1d	2E-06	0.2	Gmps	7E-06	0.2
Prr3	6E-02	0.2	Ahctf1	1E-01	0.2
Xpo5	1E-02	0.2	Taf3	5E-01	0.2
Lats1	9E-03	0.2	Gnl2	5E-02	0.2
Usp49	9E-01	0.2	Hoxc8	1E-01	0.2
Safb	3E-03	0.2	Mcu	4E-03	0.1
Cmtm4	4E-02	0.2	Pdik1l	3E-01	0.1
Hoxc9	2E-02	0.2	Rev3l	1E-03	0.1
Trappc12	3E-01	0.2	Itfg1	2E-04	0.1
Isyna1	8E-01	0.2	Csad	2E-05	0.1
Ankrd13c	1E-02	0.2	Slc25a37	6E-01	0.1
Trappc11	2E-01	0.2	Hibch	1E-01	0.1
Coq2	5E-03	0.2	Mat2b	3E-04	0.1
Ptpn13	2E-01	0.2	Rcl1	8E-01	0.1
Sdhaf2	5E-03	0.2	2310009A05Rik	5E-04	0.1
Zbtb7b	3E-01	0.2	Tex9	7E-01	0.1
Nr2c2ap	2E-01	0.2	Ube3c	4E-02	0.1
Eif4a2	6E-08	0.2	Fzd2	3E-04	0.1
Arfip1	9E-03	0.2	Tctex1d2	7E-03	0.1
Fem1a	3E-01	0.2	Ankrd42	6E-01	0.1
Zfp871	5E-03	0.2	Rfx7	2E-02	0.1
Trappc6a	2E-04	0.1	Fggy	3E-01	0.1
Tulp3	3E-02	0.1	Ostc	3E-06	0.1
Atrip	2E-01	0.1	Pik3ca	4E-02	0.1
Zbtb1	2E-02	0.1	Gramd4	3E-01	0.1
Mipep	2E-01	0.1	Kpna6	8E-02	0.1
BC005624	2E-06	0.1	Snd1	2E-05	0.1
Pnisr	5E-06	0.1	Slc48a1	9E-04	0.1
Med30	3E-02	0.1	Tfcp2	1E-01	0.1
Rab8b	2E-03	0.1	P3h1	6E-03	0.1
Nf2	3E-02	0.1	Zcwpw2	8E-01	0.1
Fance	2E-01	0.1	Pmpca	8E-03	0.1
Echdc1	4E-05	0.1	Cmas	3E-02	0.1
Lrrc17	3E-06	0.1	Tmem132a	5E-01	0.1
Iqgap1	3E-08	0.1	Mapk8	2E-01	0.1
E130114P18Rik	5E-01	0.1	Pds5b	2E-03	0.1
Pcdhb22	4E-01	0.1	Hs2st1	3E-02	0.1
Mettl4	3E-01	0.1	Mllt10	1E-02	0.1
Fam222b	2E-01	0.1	Spty2d1	7E-01	0.1
Utrn	4E-06	0.1	Camkmt	1E+00	0.1
Tmem241	6E-01	0.1	Bag3	6E-03	0.1
Vps33b	3E-01	0.1	Trp53inp1	3E-02	0.1
Trmt61a	8E-01	0.1	Kcmf1	1E-04	0.1
Srsf9	9E-09	0.1	Pomt2	8E-01	0.1
Znrf2	3E-01	0.1	Ube2e2	9E-04	0.1

Fibroblast					
Control			NICD2		
Gene	p	FC	Gene	p	FC

Fibroblast?					
Control			NICD2		
Gene	p	FC	Gene	p	FC
Smg9	2E-10	0.3	Mitd1	4E-17	0.3
Fam107b	9E-11	0.3	Zfp871	4E-16	0.3
Eif4ebp3	3E-10	0.3	Ddhd1	1E-15	0.3
Ntn1	1E-12	0.3	Rapgef6	2E-20	0.3
Klf13	1E-10	0.3	Cast	1E-26	0.3
Ybey	7E-06	0.3	Cfap20	2E-14	0.3
Shoc2	9E-14	0.3	Gadd45b	8E-17	0.3
Mndal	5E-03	0.3	Gm44777	5E-08	0.3
Mrpl46	6E-16	0.3	Arsi	9E-17	0.3
Gpc1	6E-15	0.3	Grk5	2E-15	0.3
Sav1	8E-13	0.3	Tdg	5E-12	0.3
Timm23	3E-21	0.3	Trim11	6E-16	0.3
Cmip	4E-14	0.3	Cobll1	7E-25	0.3
Hdgfl3	1E-12	0.3	Acat1	4E-21	0.3
Micall2	2E-09	0.3	Fndc10	1E-04	0.3
Il20rb	3E-06	0.3	Fads1	4E-16	0.3
Hdac6	4E-12	0.3	Gpd1l	2E-05	0.3
Pdhx	9E-15	0.3	Msrb1	5E-05	0.3
Dlgap1	1E-09	0.3	Dda1	7E-14	0.3
Mrpl15	1E-09	0.3	Yaf2	2E-16	0.3
Gadd45g	6E-11	0.3	Rnft2	8E-10	0.3
Gm14798	7E-07	0.3	Lnx2	1E-08	0.3
Pabpn1	3E-22	0.3	Leo1	1E-19	0.3
Ano6	4E-14	0.3	Eif3j1	8E-14	0.3
Tmem238	5E-12	0.3	Rnpep	2E-12	0.3
Cdc5l	1E-15	0.3	Jup	1E-09	0.3
Eif2ak2	9E-14	0.3	S100a11	3E-46	0.3
Ing2	2E-08	0.3	Tlcd1	5E-22	0.3
Bvht	1E-08	0.3	Commd10	9E-12	0.3
Cfap20	2E-15	0.3	Cwc15	5E-22	0.3
Sbsn	5E-10	0.3	Fam104a	7E-33	0.3
Ctbp1	1E-12	0.3	Zc3h8	4E-11	0.3
Adamts5	7E-11	0.3	Bpnt1	1E-13	0.3
Tmem123	8E-10	0.3	Smcr8	8E-09	0.3
Nphp1	1E-12	0.3	Lta4h	1E-14	0.3
Tln2	9E-14	0.3	Dync1i1	1E-12	0.3
Mbd3	6E-12	0.3	Xpo5	5E-14	0.3
Mcu	2E-12	0.3	Ikzf5	2E-17	0.3
Taf3	6E-14	0.3	Jcad	3E-17	0.3
Fam129b	4E-15	0.3	Prss23	2E-13	0.3
Papss1	3E-14	0.3	Prr14	5E-14	0.3
Tcf3	7E-15	0.3	Cetn4	6E-17	0.3
Bbs12	9E-07	0.3	Acy1	1E-04	0.3
Bpnt1	1E-09	0.3	Nek7	2E-14	0.3
Mlh1	2E-12	0.3	Ttc32	3E-10	0.3
Pdcd5	3E-26	0.3	Rpap3	5E-14	0.3
Tspan12	1E-09	0.3	Erg28	3E-20	0.3
Chchd6	4E-05	0.3	5033430I15Rik	2E-10	0.3
Ube2e3	1E-12	0.3	Api5	1E-15	0.3
Utp6	3E-14	0.3	Rbpms	5E-16	0.3
Myl4	1E-31	0.3	Zfp706	1E-29	0.3
Map3k7	4E-12	0.3	Kctd5	2E-15	0.3
Zdhhc8	2E-13	0.3	Shoc2	7E-18	0.3
Rrs1	9E-09	0.3	Tbc1d15	4E-20	0.3
Tnfrsf1b	1E-09	0.3	Fbxo42	2E-17	0.3
Txlna	7E-13	0.3	Adprhl2	6E-16	0.3
Mfn2	4E-10	0.3	Cdr2l	5E-14	0.3
Rcc2	3E-14	0.3	Tmem17	5E-13	0.3
Sorcs2	2E-07	0.3	Tmem200a	6E-06	0.3
Phtf2	7E-14	0.3	Tuba4a	5E-11	0.3
Wdr91	2E-09	0.3	Prtg	4E-04	0.3
Dnajb3	9E-07	0.3	Phrf1	3E-12	0.3
Trmt10a	2E-12	0.3	Sec14l1	4E-15	0.3
Tmem64	1E-12	0.3	Med22	6E-12	0.3
Akap8	2E-11	0.3	Arglu1	2E-27	0.3
Cic	7E-10	0.3	Rngtt	1E-17	0.3
Snrnp35	3E-13	0.3	Cd3eap	9E-13	0.3
Lonp1	3E-12	0.3	Acap2	3E-19	0.3
St3gal5	2E-15	0.3	Slc41a3	3E-14	0.3
Usp10	1E-07	0.3	Zfp956	2E-08	0.3
Klk8	1E-08	0.3	Eif4h	4E-19	0.3
Plec	1E-17	0.3	Lgalsl	1E-15	0.3
Trmo	1E-04	0.3	Arrdc1	2E-12	0.3

Undefined					
Control			NICD2		
Gene	p	FC	Gene	p	FC

Articular/Synovial Fibroblast					
Control			NICD2		
Gene	p	FC	Gene	p	FC
Epm2aip1	7E-01	0.1	Rcbtb1	7E-01	0.1
Cacna1d	3E-01	0.1	Mien1	3E-02	0.1
Zup1	7E-01	0.1	Aup1	5E-01	0.1
Rsu1	1E-04	0.1	Nphp1	9E-01	0.1
Baz2a	5E-01	0.1	Krba1	7E-01	0.1
Rps12	5E-08	0.1	Rp2	3E-01	0.1
Cep95	5E-01	0.1	Rpl38	8E-11	0.1
Cdc25b	7E-01	0.1	Use1	6E-03	0.1
Golm1	5E-01	0.1	Lnpep	6E-01	0.1
Tspan4	3E-04	0.1	Iqce	2E-01	0.1
Gm46367	2E-01	0.1	Ppp3cc	5E-01	0.1
Commd10	5E-01	0.1	Tkt	2E-02	0.1
Mga	2E-01	0.1	Snx33	9E-01	0.1
Taz	1E+00	0.1	Klf11	7E-02	0.1
Dyrk2	7E-01	0.1	Mar8	8E-01	0.1
Mkks	5E-01	0.1	Rpl3	2E-11	0.1
Akr7a5	4E-02	0.1	Hoxc9	6E-01	0.1
Mxd1	6E-01	0.1	Fan1	2E-01	0.1
Eif2a	3E-01	0.1	Syt12	1E-01	0.1
Rnf135	4E-01	0.1	Wdr47	5E-01	0.1
Dynlt1f	3E-01	0.1	Mettl18	3E-01	0.1
Wdr55	4E-01	0.1	Cwc25	2E-01	0.1
Eif2s3y	5E-01	0.1	Osbpl1a	4E-01	0.1
Tut7	1E-01	0.1	Dcaf6	5E-01	0.1
Rhog	7E-01	0.1	Gramd3	7E-01	0.1
Snai2	3E-01	0.1	Ttyh3	5E-01	0.1
Alg11	2E-01	0.1	Dpf2	1E+00	0.1
Prpf6	1E-01	0.1	Dtnb	5E-01	0.1
Arpc3	5E-04	0.1	Mrpl45	8E-01	0.1
Vps37c	3E-01	0.1	Cxxc5	4E-01	0.1
Fkbp14	2E-01	0.1	Nphp3	3E-01	0.1
Kif13a	1E+00	0.1	Btbd9	9E-01	0.1
Vars2	6E-02	0.1	Syvn1	3E-01	0.1
Ppip5k1	6E-01	0.1	Ahdc1	1E+00	0.1
Atg4a	6E-01	0.1	Hlcs	9E-01	0.1
Med26	6E-01	0.1	Rps24	7E-10	0.1
Ift46	1E+00	0.1	Vps29	6E-02	0.1
Mgrn1	9E-01	0.1	Zdhhc4	9E-01	0.1
Rhou	8E-01	0.1	Gm42047	5E-01	0.1
Rps19	9E-17	0.1	Nutf2	3E-01	0.1
Ctif	2E-01	0.1	Sardh	1E-01	0.1
Zfp992	2E-01	0.1	Zfp959	3E-01	0.1
Glyr1	8E-02	0.1	Trappc12	4E-01	0.1
Ppm1h	2E-01	0.1	Pigb	5E-01	0.1
Tkfc	1E-01	0.1	Gm32618	3E-01	0.1
Gm11613	2E-01	0.1	Hexim1	9E-01	0.1
Nenf	2E-05	0.1	Ubn1	4E-01	0.1
Stk38	1E-02	0.1	Ecsit	2E-01	0.1
Mtmr3	4E-01	0.1	Tbl1xr1	2E-02	0.1
Sptssa	8E-02	0.1	Rps19	1E-12	0.1
D130017N08Rik	4E-01	0.1	Natd1	7E-01	0.1
Eif2d	2E-01	0.1	Gatd3a	9E-01	0.1
Slc30a4	4E-01	0.1	Cdadc1	1E+00	0.1
Smurf1	6E-01	0.1	Tspan5	4E-01	0.1
Ints7	4E-01	0.1	Mapkbp1	4E-01	0.1
Sept1	6E-01	0.1	Arid5b	3E-03	0.1
Parn	8E-01	0.1	Rpgr	3E-01	0.1
Plekho1	5E-02	0.1	Eml1	1E+00	0.1
Zfyve9	2E-01	0.1	Sh3kbp1	2E-01	0.1
Scamp5	3E-01	0.1	Uhrf2	1E+00	0.1
Socs7	5E-01	0.1	Slc35b3	4E-01	0.1
Usp20	2E-01	0.1	St3gal3	7E-01	0.1
Klhl36	7E-01	0.1	Lactb2	8E-01	0.1
Rnf19b	6E-01	0.1	Synm	4E-01	0.1
Lztr1	7E-01	0.1	Dhx57	8E-01	0.1
Dera	8E-01	0.1	Fam50a	4E-01	0.1
Mapk3	8E-02	0.1	P4htm	4E-01	0.1
Mrps21	6E-04	0.1	Ankrd52	2E-01	0.1
Gcdh	3E-01	0.1	Aggf1	8E-01	0.1
Eml4	9E-01	0.1	Ap3b1	7E-02	0.1
Umad1	6E-01	0.1	Rgmb	8E-02	0.1
Uba52	6E-05	0.1	Capns1	2E-03	0.1
Rragc	3E-01	0.1	Tgfbr2	7E-02	0.1

Limb Mesenchyme						Chondrogenic						Fibroblast						Fibroblast?						Undefined						Articular/Synovial Fibroblast					
Control			NICD2			Control			NICD2			Control			NICD2			Control			NICD2			Control			NICD2			Control			NICD2		
Gene	*p*	FC	Gene	*p*	FC	Gene	*p*	FC	Gene	*p*	FC	Gene	*p*	FC	Gene	*p*	FC	Gene	*p*	FC	Gene	*p*	FC	Gene	*p*	FC	Gene	*p*	FC	Gene	*p*	FC	Gene	*p*	FC
Rsbn1	2E-05	0.2	Tada3	3E-04	0.2	Elp4	1E-02	0.1	Psmf1	7E-01	0.1							Snx7	6E-16	0.3	Gmip	3E-14	0.3							Ptpn23	9E-01	0.1	Ptrhd1	7E-01	0.1
Socs6	1E-06	0.2	Fig4	4E-04	0.2	Tiprl	2E-03	0.1	Zfp710	4E-01	0.1							Gngt2	1E-07	0.3	Agbl3	5E-11	0.3							Rtn3	3E-03	0.1	Elp2	7E-01	0.1
Parva	9E-08	0.2	Epg5	4E-03	0.2	Pias2	4E-02	0.1	Zfp74	3E-01	0.1							Hoxa5	4E-07	0.3	Ash2l	4E-14	0.3							Mbtps2	4E-01	0.1	Lgals8	6E-01	0.1
Klhl42	7E-04	0.2	Pikfyve	1E-04	0.2	Ap1b1	3E-01	0.1	Zfp58	6E-01	0.1							Cnnm4	4E-07	0.3	Cdc42se2	1E-15	0.3							Rfk	5E-01	0.1	Chchd6	8E-01	0.1
Tacc2	7E-05	0.2	Uchl3	3E-06	0.2	Ttc5	2E-02	0.1	Rdh11	9E-01	0.1							Snrpd3	4E-15	0.3	Mrpl10	1E-14	0.3							Zfp60	7E-01	0.1	Zfp748	4E-01	0.1
Zfp740	2E-04	0.2	Mettl17	9E-04	0.2	Hspb6	2E-03	0.1	Zmat5	3E-03	0.1							Psmd9	5E-15	0.3	Tbxa2r	2E-10	0.3							Arhgap23	8E-01	0.1	Klhl7	7E-01	0.1
Letmd1	1E-03	0.2	Eps15l1	9E-04	0.2	Polr2g	7E-04	0.1	Nudt22	6E-01	0.1							Tmem14c	3E-24	0.3	Mtmr12	1E-15	0.3							Gm10516	2E-01	0.1	Zfp239	3E-01	0.1
2810410L24Rik	7E-03	0.2	Nsun2	1E-05	0.2	Grpel2	2E-01	0.1	Frk	4E-01	0.1							Nrf1	2E-08	0.3	Arf6	2E-14	0.3							Galk1	5E-02	0.1	Elp4	3E-01	0.1
Fbxo22	6E-05	0.2	Ccdc137	2E-05	0.2	Fbxw5	4E-01	0.1	Ago4	8E-01	0.1							Itpk1	4E-10	0.3	Txlna	3E-13	0.3							Rpl27a	2E-16	0.1	Zfp414	1E-01	0.1
Tbc1d12	3E-05	0.2	Afg3l2	2E-04	0.2	Ppp1r35	3E-02	0.1	Oard1	3E-01	0.1							Purb	2E-14	0.3	Mapk14	5E-13	0.3							Fbxo17	7E-01	0.1	Pard3bos1	8E-01	0.1
Ankrd16	5E-04	0.2	Zswim8	6E-04	0.2	Zfyve1	5E-02	0.1	Higd2a	1E-05	0.1							Mmp3	3E-03	0.3	Myh9	2E-25	0.3							Zkscan1	6E-01	0.1	Arap1	3E-01	0.1
Ssb	1E-12	0.2	Cmtm4	2E-03	0.2	Tanc2	1E-02	0.1	Actl6a	3E-02	0.1							Abcb6	3E-06	0.3	Mkrn2	6E-12	0.3							Zcchc8	5E-01	0.1	Dnajb4	4E-01	0.1
Plscr3	6E-07	0.2	Zbtb9	4E-03	0.2	Calcoco1	3E-02	0.1	Rsrc1	7E-07	0.1							Pde5a	6E-20	0.3	Zswim3	7E-09	0.3							Gm14164	5E-02	0.1	Thbd	3E-01	0.1
Nfya	3E-03	0.2	Ddx19a	2E-04	0.2	Dpysl2	1E-01	0.1	Spred2	8E-01	0.1							Psmd2	2E-16	0.3	Vash2	4E-07	0.3							Zfp142	2E-01	0.1	Rp9	7E-02	0.1
Setd7	9E-05	0.2	Zcchc4	8E-04	0.2	Angel2	9E-03	0.1	Txnip	7E-03	0.1							Nup88	7E-13	0.3	Lrch2	3E-14	0.3							Clip2	3E-01	0.1	Acly	1E-01	0.1
Trappc3	5E-04	0.2	Cops8	6E-05	0.2	Sap18b	8E-01	0.1	Mib1	8E-03	0.1							Arid5a	4E-09	0.3	Myl12a	3E-26	0.3							Znrd1as	1E+00	0.1	Ddit4	8E-01	0.1
Cul5	3E-06	0.2	Ep400	2E-03	0.2	Mepce	2E-01	0.1	Nr2c2ap	8E-01	0.1							Mbnl1	2E-17	0.3	Ndufv1	2E-18	0.3							Cyb5rl	2E-01	0.1	Dcaf8	3E-01	0.1
Utp14a	1E-05	0.2	Cyb5d2	3E-03	0.2	Cnot4	1E-03	0.1	Mrpl47	3E-01	0.1							Tmx4	6E-14	0.3	Bbs12	9E-11	0.3							Elmo2	1E-01	0.1	Zfp30	9E-01	0.1
Lancl2	4E-04	0.2	Lrp11	9E-03	0.2	Snx33	2E-01	0.1	Asb13	5E-01	0.1							Hspa1b	5E-08	0.3	Tfb1m	9E-14	0.3							Casd1	9E-01	0.1	F8a	1E-01	0.1
Ylpm1	3E-06	0.2	Tspan2	7E-04	0.2	Fbxl14	9E-01	0.1	Pcf11	3E-01	0.1							Hspa8	2E-42	0.3	Camta1	6E-13	0.3							Zmym5	9E-01	0.1	Cybc1	1E-01	0.1
Letm1	8E-05	0.2	Hnrnpul2	2E-05	0.2	Nol6	2E-01	0.1	Fbxo3	4E-02	0.1							Galnt17	2E-06	0.3	Bnip2	7E-25	0.3							Klhl20	9E-01	0.1	Slc17a5	2E-01	0.1
Rest	4E-06	0.2	Lrrc42	1E-04	0.2	Fkbp3	4E-07	0.1	Lefty1	8E-01	0.1							Supv3l1	3E-12	0.3	Tigar	8E-11	0.3							Tug1	1E+00	0.1	Tmtc1	6E-01	0.1
Dcp1a	6E-03	0.2	Ddhd1	9E-05	0.2	Ogfod2	6E-01	0.1	Arl2	5E-05	0.1							Slc22a15	4E-10	0.3	Golt1b	2E-17	0.3							As3mt	3E-01	0.1	Arhgef5	5E-01	0.1
1110038B12Rik	4E-10	0.2	Hsf2	4E-04	0.2	Mrpl57	1E-06	0.1	Fbxl17	2E-05	0.1							Glce	3E-10	0.3	Rwdd1	3E-29	0.3							Scaf1	3E-01	0.1	Nrip1	2E-01	0.1
B3glct	7E-05	0.2	Pcyox1	3E-03	0.2	Fbxo8	2E-01	0.1	Igf2r	3E-06	0.1							Zfp664	3E-09	0.3	Pard6a	5E-10	0.3							Tmem150a	6E-01	0.1	Tab3	3E-01	0.1
Fbxl4	3E-04	0.2	Zfp280c	1E-04	0.2	Rmdn1	2E-04	0.1	Fkrp	7E-01	0.1							Nipa2	2E-12	0.3	Ubqln4	3E-14	0.3							Elp4	8E-01	0.1	Txndc15	2E-02	0.1
Zfp868	2E-02	0.2	Alkbh6	1E-04	0.2	Hspa4	3E-09	0.1	Spata6	6E-02	0.1							Prr14	1E-10	0.3	Npas3	2E-17	0.3							Isoc2b	9E-01	0.1	Rabgap1	8E-02	0.1
Setd2	3E-06	0.2	Golga4	6E-05	0.2	Gss	2E-01	0.1	Prr14	3E-01	0.1							Hnrnpa0	4E-27	0.3	Flna	9E-18	0.3							Traf6	4E-01	0.1	1700037H04Rik	3E-01	0.1
Ubr4	1E-05	0.2	Tut1	3E-04	0.2	Ddx23	5E-03	0.1	Mepce	5E-01	0.1							Trmt2b	5E-09	0.3	Tmco1	9E-25	0.3							Lcmt2	2E-01	0.1	Anxa4	3E-02	0.1
Clns1a	8E-05	0.2	Asph	3E-05	0.2	B3galt4	4E-01	0.1	Pggt1b	1E-01	0.1							Ift74	9E-10	0.3	Ube2e3	1E-16	0.3							Zbtb44	4E-01	0.1	Wdr78	5E-01	0.1
E2f4	7E-05	0.2	Agrn	3E-05	0.2	Tsr2	7E-01	0.1	Gm14636	3E-01	0.1							Commd1	3E-18	0.3	Fip1l1	5E-19	0.3							Sap18b	2E-01	0.1	Cbx8	2E-01	0.1
Fam241b	5E-04	0.2	Syde1	2E-04	0.2	Serinc5	4E-02	0.1	Ift20	5E-08	0.1							Cyth2	4E-08	0.3	Zbtb9	1E-08	0.3							Unc93b1	3E-01	0.1	Mipep	3E-01	0.1
Trpm6	7E-03	0.2	5530601H04Rik	3E-04	0.2	Gabpb2	5E-02	0.1	Hint3	3E-01	0.1							Utp14b	2E-08	0.3	Prpf40a	1E-25	0.3							C2	3E-01	0.1	9430015G10Rik	2E-01	0.1
Clic4	7E-09	0.2	Zmat3	2E-03	0.2	Camk2d	7E-06	0.1	Cacul1	1E-01	0.1							Cldn34c1	9E-11	0.3	Cd2bp2	5E-15	0.3							Plcg1	4E-01	0.1	Mrpl24	1E-01	0.1
Ppp1r8	6E-05	0.2	Galnt11	1E-04	0.2	Sh3bgrl	2E-07	0.1	H3f3a	9E-20	0.1							Garnl3	8E-13	0.3	Me1	2E-20	0.3							Idh3g	2E-01	0.1	S1pr1	4E-01	0.1
Tsen54	1E-03	0.2	Cox6a2	9E-05	0.2	Zfp995	8E-02	0.1	Clip1	2E-02	0.1							Unc5b	8E-07	0.3	Smyd2	1E-16	0.3							Fam193a	5E-02	0.1	Lemd2	2E-01	0.1
Map6	7E-04	0.2	Vps18	6E-01	0.2	Phf12	8E-03	0.1	Wipi2	1E-01	0.1							Sf3a2	6E-13	0.3	Adamts5	2E-16	0.3							A730081D07Rik	5E-01	0.1	Vegfa	7E-01	0.1
Micu3	2E-04	0.2	Terf2	5E-05	0.2	Ogfod1	1E-01	0.1	Ift46	8E-02	0.1							Shisa4	4E-12	0.3	Pgls	5E-31	0.3							Tmem104	3E-01	0.1	Mmut	8E-01	0.1
Bpnt1	3E-03	0.2	Zfyve9	2E-05	0.2	Cdc73	1E-03	0.1	Uimc1	5E-02	0.1							Slc25a30	5E-07	0.3	Arhgap42	1E-11	0.3							Zfp946	2E-01	0.1	Gltp	6E-01	0.1
Crlf2	4E-05	0.2	Rgs12	4E-05	0.2	Ap3s2	3E-02	0.1	Tsc22d4	5E-05	0.1							Ddx59	1E-07	0.3	Ift140	7E-13	0.3							Hspbp1	1E-01	0.1	Phactr2	3E-01	0.1
Zcrb1	9E-12	0.2	Zfp507	5E-04	0.2	Tm9sf3	9E-10	0.1	Apbb1	8E-01	0.1							Arl6	5E-12	0.3	Rtf1	6E-17	0.3							9530068E07Rik	2E-02	0.1	Gm42984	3E-01	0.1
Man1a2	2E-07	0.2	Pip4p2	2E-04	0.2	Mapk1ip1	6E-02	0.1	Znhit2	4E-01	0.1							Tex10	6E-15	0.3	Mpp1	2E-09	0.3							Mark2	5E-01	0.1	Nckap5l	3E-01	0.1
Zfyve9	3E-05	0.2	Alg3	8E-04	0.2	Lix1l	7E-04	0.1	Ccdc8	5E-01	0.1							Ppp1r11	3E-11	0.3	Gm31763	2E-09	0.3							Ndufv3	8E-05	0.1	Msra	1E-01	0.1
Tra2a	4E-06	0.2	Pdcd4	3E-10	0.2	Ndufaf6	3E-01	0.1	Bysl	2E-01	0.1							Tmed8	7E-11	0.3	Cetn3	4E-16	0.3							Thg1l	3E-01	0.1	Idh1	7E-05	0.1
Fam118a	8E-03	0.2	2510039O18Rik	2E-03	0.2	Pigh	6E-01	0.1	Usp9x	5E-04	0.1							Luc7l3	6E-16	0.3	Pnkp	1E-16	0.3							Selenow	1E-03	0.1	Slc30a1	4E-01	0.1
Gemin7	7E-08	0.2	Fam129a	8E-08	0.2	Rc3h2	4E-03	0.1	Kifap3	2E-03	0.1							Cavin1	1E-18	0.3	Irx5	2E-09	0.3							Siah1a	8E-01	0.1	Abi1	1E-01	0.1
Ankib1	4E-06	0.2	Ptcd2	2E-04	0.2	Cstf2t	4E-01	0.1	Akirin2	9E-04	0.1							Irf2bp1	2E-10	0.3	Prkag1	7E-11	0.3							U2af1l4	9E-01	0.1	Rab11fip2	5E-01	0.1
Mea1	2E-07	0.2	Nadk	3E-05	0.2	Gtpbp2	1E-02	0.1	Utrn	1E-05	0.1							Med6	2E-12	0.3	Ndufb8	4E-30	0.3							Ubl3	3E-01	0.1	Josd2	2E-01	0.1
Gm10762	4E-04	0.2	Drosha	6E-03	0.2	Gar1	1E-02	0.1	Poglut1	7E-02	0.1							Pmml	3E-07	0.3	Tgfb3	2E-08	0.3							Osbpl5	3E-01	0.1	Dpagt1	1E-01	0.1
Wdr4	3E-05	0.2	Clns1a	1E-04	0.2	Zkscan7	6E-01	0.1	1700030K09Rik	7E-01	0.1							Spata48	2E-05	0.3	Apobec1	2E-15	0.3							Ttbk2	6E-02	0.1	Rpl17	4E-10	0.1
Cped1	8E-09	0.2	Dync1i2	1E-09	0.2	Enho	4E-01	0.1	Osbpl5	5E-01	0.1							Exosc10	4E-12	0.3	Nufip1	2E-14	0.3							Rapgef6	5E-01	0.1	Tmem159	7E-01	0.1
Zdhhc8	2E-04	0.2	Rbfox1	4E-03	0.2	Immp2l	5E-04	0.1	Gtf3c2	3E-01	0.1							Fhl3	8E-07	0.3	Ptprf	5E-13	0.3							Rap2a	4E-01	0.1	Rpl36	1E-08	0.1
Tet3	5E-04	0.2	Pnrc2	1E-04	0.2	Zfp513	9E-01	0.1	Hltf	9E-02	0.1							Gzf1	1E-08	0.3	Ccnyl1	3E-16	0.3							Arid4a	4E-01	0.1	Ric1	6E-01	0.1
Ptpn2	1E-05	0.2	Nprl3	4E-05	0.2	Tle3	2E-01	0.1	Ccdc130	5E-01	0.1							Wnk1	1E-16	0.3	Tex10	1E-16	0.3							Cdan1	5E-02	0.1	Trps1	4E-04	0.1
Cops2	2E-07	0.2	Spty2d1	9E-06	0.2	Cluap1	1E-02	0.1	Pum1	6E-05	0.1							Mad2l2	2E-11	0.3	Tll1	8E-07	0.3							Rpl29	4E-11	0.1	Zfp638	3E-01	0.1
Pafah1b3	5E-07	0.2	Dhx36	5E-08	0.2	Ubac2	4E-02	0.1	Rufy2	6E-02	0.1							Kif5b	1E-19	0.3	Uqcrq	2E-39	0.3							Smim7	2E-01	0.1	Ube4b	4E-01	0.1
Scamp2	2E-04	0.2	Yeats2	2E-03	0.2	Bloc1s3	3E-01	0.1	Stard10	9E-02	0.1							Trip6	4E-08	0.3	Dvl1	4E-11	0.3							Zfp46	6E-01	0.1	Ndufa13	2E-05	0.1
Pdcd10	2E-09	0.2	Aco2	5E-05	0.2	Ndfip2	3E-03	0.1	Gpr108	2E-01	0.1							Trim2	3E-13	0.3	Arl4a	8E-22	0.3							Rexo1	5E-01	0.1	Taf13	9E-01	0.1
Afg3l2	1E-05	0.2	Il15ra	1E-02	0.2	Chd1	1E-03	0.1	Kif3b	4E-01	0.1							Cd3eap	9E-11	0.3	Sgsm3	1E-05	0.3							Slc25a20	9E-02	0.1	Zfp738	3E-01	0.1
Arglu1	4E-09	0.2	Dynlrb1	5E-11	0.2	Zswim9	7E-01	0.1	Snhg12	8E-01	0.1							Ifi204	2E-10	0.3	Rbm45	3E-12	0.3							Gm17018	8E-01	0.1	Nek6	6E-01	0.1
Ywhae	1E-18	0.2	Zfp963	4E-02	0.2	Orai2	7E-01	0.1	Phc3	2E-02	0.1							Polr2h	2E-05	0.3	Psme3	2E-17	0.3							Ppp2r3d	1E-01	0.1	Lamtor2	2E-02	0.1
Psme3	4E-05	0.2	Max	3E-06	0.2	Gtf3c2	1E-01	0.1	Rogdi	1E+00	0.1							Myh9	5E-20	0.3	Fhl2	1E-22	0.3							Mau2	1E+00	0.1	Galt	3E-01	0.1
Trnt1	5E-06	0.2	Enah	1E-02	0.2	Srd5a1	6E-01	0.1	Mff	3E-04	0.1							Ppp1r18	5E-11	0.3	Hnrnph1	3E-13	0.3							Tk2	4E-01	0.1	Chmp2b	1E-01	0.1
Sap30l	3E-07	0.2	Prpf4b	8E-11	0.2	Rpn2	6E-07	0.1	Morf4l1	3E-18	0.1							Cdh13	2E-11	0.3	Prpf19	5E-15	0.3							Cep112	1E+00	0.1	Zdhhc3	3E-01	0.1
Brap	4E-04	0.2	Sbf1	1E-04	0.2	Tma16	2E-01	0.1	Pcm1	3E-05	0.1							Mgat4b	3E-04	0.3	Ss18	2E-15	0.3							Uqcr11	5E-07	0.1	Zfp445	4E-01	0.1
Zrsr1	4E-04	0.2	Pfdn2	5E-06	0.2	Vhl	4E-01	0.1	Tm9sf1	3E-02	0.1							Dvl1	3E-11	0.3	Loxl3	1E-14	0.3							Znfx1	4E-01	0.1	Dlg5	7E-01	0.1
Ube2j2	2E-06	0.2	Trmu	1E-03	0.2	Cryl1	2E-01	0.1	Napb	9E-01	0.1							Morc4	7E-12	0.3	Resf1	2E-11	0.3							Myadm	3E-01	0.1	Dnpep	7E-01	0.1
Siah1a	5E-05	0.2	Gtf3c1	1E-03	0.2	Sod2	3E-11	0.1	Babam2	4E-04	0.1							Zc3h14	3E-12	0.3	St6galnac2	2E-10	0.3							Mrpl39	4E-01	0.1	Rpl21	5E-10	0.1
1810009A15Rik	1E-04	0.2	Uhrf1bp1	9E-04	0.2	Ddx58	2E-02	0.1	2810402E24Rik	7E-01	0.1							Gtf3c4	2E-06	0.3	Atrip	1E-16	0.3							Sdhd	9E-01	0.1	Flvcr1	1E-01	0.1
Pcdhga5	2E-02	0.2	Ambra1	6E-06	0.2	Rnf19a	2E-01	0.1	Tra2a	4E-03	0.1							Hira	2E-09	0.3	Rcc2	6E-14	0.3							Esrra	5E-01	0.1	Cast	8E-02	0.1
Gpatch4	3E-05	0.2	Dkc1	1E-05	0.2	Atrn	3E-02	0.1	Mien1	2E-07	0.1							Col5a3	3E-12	0.3	Atpaf2	7E-17	0.3							Klf6	3E-01	0.1	Cep350	9E-01	0.1
Rbms3	3E-13	0.2	Faf2	2E-05	0.2	Xist	3E-01	0.1	Alg1	7E-01	0.1							Mfsd10	2E-13	0.3	Sestd1	6E-15	0.3							Ankrd10	3E-01	0.1	Arl8b	3E-01	0.1
Tead2	4E-05	0.2	Cbl	3E-05	0.2	AW554918	2E-02	0.1	Map3k12	2E-01	0.1							A930001C03Rik	1E-11	0.3	Tmem123	1E-15	0.3							Plat	9E-01	0.1	Relch	4E-01	0.1

Limb Mesenchyme

Control			NICD2		
Gene	*p*	FC	Gene	*p*	FC
Apopt1	4E-06	0.2	Mex3c	5E-04	0.2
Zfp646	3E-05	0.2	Tbc1d10a	5E-05	0.2
Ruvbl1	2E-05	0.2	Aen	2E-06	0.2
Gm26944	3E-04	0.2	Supt6	8E-06	0.2
Klhl5	3E-04	0.2	Tmem185b	1E-03	0.2
Gpr137	1E-04	0.2	Traf3ip1	2E-05	0.2
Zfp46	2E-03	0.2	Sec22b	6E-07	0.2
Eif3j1	1E-07	0.2	Gm14410	1E-02	0.2
Cd24a	5E-10	0.2	Terf2ip	6E-04	0.2
Pnpla8	2E-08	0.2	Banf1	5E-08	0.2
Fkbp11	1E-06	0.2	Smg1	9E-06	0.2
Fxyd5	1E-11	0.2	Arntl	1E-02	0.2
Ube2i	7E-12	0.2	Exosc4	1E-05	0.2
Nr2c1	9E-05	0.2	Gmcl1	1E-04	0.2
Ctnnbl1	3E-04	0.2	E130309D02Rik	4E-04	0.2
Hspd1	6E-14	0.2	Ddx18	4E-05	0.2
Nop14	5E-06	0.2	Xrn2	2E-08	0.2
Cops4	4E-06	0.2	Vac14	1E-03	0.2
Tbc1d22a	4E-05	0.2	Ercc3	9E-04	0.2
Prkd1	5E-06	0.2	Tug1	3E-03	0.2
Ccdc115	3E-04	0.2	Mbd3	4E-06	0.2
Dpp3	7E-05	0.2	Pemt	3E-03	0.2
Eef1e1	9E-05	0.2	Sar1a	6E-09	0.2
Lemd2	8E-05	0.2	Wiz	2E-05	0.2
Med15	1E-04	0.2	Insr	3E-04	0.2
Srsf1	2E-06	0.2	Gid4	1E-04	0.2
Npepl1	2E-04	0.2	Mtrr	7E-03	0.2
Tmem101	3E-04	0.2	Khnyn	3E-03	0.2
Tiprl	2E-05	0.2	Cap2	4E-04	0.2
Sipa1l3	2E-03	0.2	Ift52	1E-04	0.2
Pigs	2E-04	0.2	Tulp3	5E-06	0.2
Blzf1	3E-05	0.2	Csnk1g3	8E-06	0.2
2200002D01Rik	2E-02	0.2	Cog3	7E-04	0.2
Ercc3	4E-04	0.2	Kif2a	1E-05	0.2
Mrpl37	4E-04	0.2	Tmem243	3E-02	0.2
Copz2	2E-12	0.2	Imp3	1E-08	0.2
Rab1a	2E-07	0.2	Hilpda	3E-06	0.2
Sypl	4E-05	0.2	Vamp1	6E-02	0.2
Ube2o	1E-04	0.2	Prkcsh	1E-07	0.2
Sra1	1E-10	0.2	Xxylt1	8E-05	0.2
Appbp2	6E-05	0.2	Mfn2	7E-04	0.2
Tmem223	1E-07	0.2	Tsnax	6E-05	0.2
Mpv17l	7E-05	0.2	Gm36975	4E-02	0.2
Zfp652	1E-06	0.2	Rusc1	1E-02	0.2
Ppp1r21	2E-04	0.2	Serinc1	1E-08	0.2
Dcaf1	1E-05	0.2	Snapin	3E-06	0.2
Itfg2	5E-03	0.2	Zbtb21	1E-02	0.2
Ncbp1	4E-04	0.2	Pacs2	3E-04	0.2
Usp5	7E-06	0.2	Trak2	5E-04	0.2
Ndufs1	3E-07	0.2	Cyth2	2E-05	0.2
Nphp3	2E-04	0.2	Pkig	8E-06	0.2
Coq4	1E-03	0.2	Ddx42	1E-06	0.2
Fam110a	8E-04	0.2	Dhx8	7E-05	0.2
Slc25a25	4E-03	0.2	Rnf217	6E-04	0.2
Prrc2a	4E-05	0.2	Anxa5	1E-14	0.2
U2af2	2E-05	0.2	Rnf2	4E-05	0.2
Manf	1E-10	0.2	Picalm	2E-08	0.2
Sf3b6	9E-13	0.2	Ttc19	2E-04	0.2
Cops3	6E-06	0.2	Neurl4	2E-02	0.2
Frg2f1	4E-02	0.2	Cbll1	5E-04	0.2
Zfp329	1E-04	0.2	Nutf2	2E-02	0.2
Herc2	6E-06	0.2	Dusp11	7E-09	0.2
Polk	1E-04	0.2	Lzic	4E-06	0.2
Smcr8	9E-02	0.2	Lrp8	5E-01	0.2
Fam234a	1E-04	0.2	Trim68	9E-03	0.2
Golgb1	5E-11	0.2	Map9	2E-04	0.2
Mfn1	6E-04	0.2	3010001F23Rik	3E-02	0.2
Hnrnpd	6E-10	0.2	Tk2	2E-03	0.2
Csnk2a1	3E-11	0.2	Brms1	3E-03	0.2
Hp1bp3	9E-12	0.2	Tesk1	7E-05	0.2
Gfm1	2E-04	0.2	Klhdc2	1E-07	0.2
Atad3a	8E-04	0.2	Slc41a2	4E-05	0.2
Bcl2l11	1E-02	0.2	Tpp2	5E-05	0.2

Chondrogenic

Control			NICD2		
Gene	*p*	FC	Gene	*p*	FC
Rbm8a	2E-06	0.1	Adar	8E-01	0.1
Dhrs7	1E-03	0.1	Cluap1	2E-01	0.1
Zfp13	3E-01	0.1	Eng	2E-01	0.1
Runx1	1E-07	0.1	Erlin1	9E-02	0.1
Dgcr2	6E-02	0.1	Msl2	3E-01	0.1
Hsph1	2E-03	0.1	0610040B10Rik	8E-01	0.1
Epm2aip1	1E-02	0.1	Accs	1E+00	0.1
Gm42418	7E-38	0.1	Atp6ap1	2E-04	0.1
Ttf1	8E-02	0.1	Rsrp1	1E-06	0.1
Hint3	3E-02	0.1	Dicer1	8E-02	0.1
Mbtd1	6E-03	0.1	Fam3a	8E-01	0.1
Dis3l2	8E-03	0.1	Pacs2	9E-01	0.1
Hmgxb4	8E-02	0.1	Mindy2	7E-01	0.1
Dnaja3	9E-03	0.1	Lyrm9	1E+00	0.1
Pigyl	5E-04	0.1	Zfp943	2E-01	0.1
Atg9a	4E-01	0.1	Ccnc	6E-01	0.1
Poli	8E-01	0.1	Foxk1	3E-01	0.1
Egfr	1E-03	0.1	Casd1	4E-02	0.1
Gripap1	2E-02	0.1	Diablo	1E-01	0.1
Ccdc181	4E-01	0.1	Ppp6r3	6E-02	0.1
Mid2	2E-01	0.1	Irf3	2E-01	0.1
Pibf1	2E-02	0.1	Phf21a	6E-06	0.1
Gmeb2	2E-01	0.1	Fosb	4E-02	0.1
Cebpz	5E-03	0.1	Rnf25	2E-01	0.1
Rnf166	7E-02	0.1	Nubp2	8E-01	0.1
2310009B15Rik	4E-03	0.1	Bphl	4E-02	0.1
Rdh5	6E-01	0.1	Sfxn1	1E-03	0.1
Gm4285	8E-01	0.1	D10Wsu102e	3E-02	0.1
Zfp37	6E-01	0.1	Zmym5	3E-02	0.1
B3gat3	1E-02	0.1	Zfp148	3E-04	0.1
Exosc2	6E-01	0.1	Gtf2b	4E-02	0.1
Vps37c	3E-01	0.1	Cdc73	3E-02	0.1
Erp44	3E-05	0.1	Btrc	3E-02	0.1
Aak1	2E-03	0.1	Yae1d1	3E-02	0.1
Txnip	1E-02	0.1	Cfap410	6E-01	0.1
Ankhd1	6E-04	0.1	Retreg2	2E-01	0.1
Cd59a	1E-01	0.1	Gm13483	6E-01	0.1
Tst	1E-01	0.1	Hmga2	2E-06	0.1
Rsbn1l	1E-01	0.1	Tmem126a	1E-05	0.1
Tcfl5	2E-01	0.1	Champ1	3E-01	0.1
Tmf1	3E-04	0.1	Islr	7E-03	0.1
Ngly1	1E-03	0.1	Arf3	4E-02	0.1
Wac	2E-06	0.1	Scamp1	3E-01	0.1
Cutc	8E-01	0.1	C2cd3	7E-01	0.1
Rictor	9E-02	0.1	Las1l	4E-01	0.1
Ifitm2	1E-07	0.1	Arih1	2E-05	0.1
Ppib	3E-16	0.1	Sclt1	1E-01	0.1
Eaf2	6E-01	0.1	Setx	6E-02	0.1
Sumf2	5E-03	0.1	Slc35e1	1E+00	0.1
Ankmy2	5E-02	0.1	Nifk	5E-02	0.1
Slc23a2	3E-01	0.1	Chmp6	3E-01	0.1
Usp37	3E-02	0.1	Taf1b	4E-01	0.1
Wdr19	2E-01	0.1	Amfr	1E-02	0.1
Slc2a1	2E-01	0.1	Trub2	4E-01	0.1
Zfp830	6E-01	0.1	Brap	4E-01	0.1
Angptl1	4E-04	0.1	Cipc	1E+00	0.1
Gorab	3E-01	0.1	Atg4c	9E-01	0.1
Erp29	7E-08	0.1	Smdt1	1E-08	0.1
Pspc1	6E-03	0.1	Pcnp	5E-05	0.1
Ubxn4	8E-10	0.1	Mcts1	4E-04	0.1
C3	4E-06	0.1	Mindy3	5E-01	0.1
Ogdh	1E-03	0.1	Ebna1bp2	3E-03	0.1
Slc25a3	4E-14	0.1	Stip1	1E-04	0.1
Slc39a10	2E-02	0.1	Ylpm1	7E-02	0.1
1810009A15Rik	4E-01	0.1	Acaca	7E-03	0.1
Efcab14	4E-02	0.1	Chd6	3E-03	0.1
Srp9	2E-10	0.1	Dmac1	7E-04	0.1
Iffo2	5E-01	0.1	Bcl2l13	1E-01	0.1
Zbtb8os	8E-04	0.1	BC005561	1E-01	0.1
Matr3	2E-05	0.1	Fam43a	8E-01	0.1
Rwdd2b	8E-01	0.1	Csde1	5E-08	0.1
Lrch3	6E-03	0.1	Ralgapb	3E-01	0.1
Zfp609	8E-04	0.1	Ercc6l2	3E-02	0.1

Fibroblast

Control			NICD2		
Gene	*p*	FC	Gene	*p*	FC

Fibroblast?

Control			NICD2		
Gene	*p*	FC	Gene	*p*	FC
S100pbp	6E-09	0.3	Hnrnpa0	7E-38	0.3
Gps2	8E-12	0.3	Pkm	6E-39	0.3
Xrn2	9E-20	0.3	Mbnl1	6E-19	0.3
Acsl4	2E-11	0.3	Tpbg	9E-19	0.3
Phrf1	6E-11	0.3	Arpc2	1E-39	0.3
Hnrnpm	8E-22	0.3	Chchd3	2E-19	0.3
Ap4m1	2E-09	0.3	2310033P09Rik	8E-14	0.3
Mars	2E-09	0.3	Peg10	2E-10	0.3
Impa1	5E-12	0.3	Megf9	1E-12	0.3
Akr1b10	3E-05	0.3	Paf1	5E-09	0.3
Zfp706	9E-20	0.3	Cs	3E-11	0.3
Gsk3a	1E-12	0.3	Ift74	8E-11	0.3
Plcl1	3E-19	0.3	Cnih1	3E-17	0.3
Yae1d1	9E-09	0.3	Airn	5E-13	0.3
Cactin	4E-14	0.3	Tmem134	1E-17	0.3
Thnsl1	4E-07	0.3	Ssb	6E-35	0.3
Strap	9E-12	0.3	Cbwd1	1E-14	0.3
Asxl3	3E-03	0.3	Arsb	3E-17	0.3
St7	2E-11	0.3	Cnot3	1E-16	0.3
Dexr	4E-02	0.3	Cnbp	2E-18	0.3
Gm28501	2E-07	0.3	Nr3c1	2E-20	0.3
Map7d3	1E-13	0.3	Nvl	5E-15	0.3
Dph3	4E-14	0.3	Elavl1	1E-15	0.3
Src	4E-09	0.3	Cherp	7E-14	0.3
Rfx5	5E-09	0.3	Clcf1	1E-04	0.3
Dnajc8	7E-16	0.3	Trmt61b	6E-15	0.3
Csk	6E-07	0.3	Psmb7	1E-14	0.3
Ctnna1	1E-16	0.3	Nme2	2E-59	0.3
Sec23b	2E-12	0.3	Arhgap31	9E-19	0.3
Pno1	1E-05	0.3	Heca	4E-15	0.3
Gng12	5E-14	0.3	Gm15559	1E-09	0.3
Mark1	1E-09	0.3	Pstpip2	1E-11	0.3
Taco1	7E-11	0.3	Ldha	6E-29	0.3
Rilp	2E-01	0.3	Zbtb2	2E-09	0.3
Fam104a	2E-21	0.3	Bud13	3E-14	0.3
Sept5	1E-11	0.3	Bcor	2E-14	0.3
Epha2	9E-07	0.3	Epha3	2E-10	0.3
Vat1l	2E-09	0.3	Rap1gds1	2E-13	0.3
Wdr25	2E-03	0.3	Aqr	1E-16	0.3
Pvr	3E-09	0.3	Cox11	1E-12	0.3
Ahcyl1	1E-11	0.3	Lrrc42	6E-13	0.3
Unk	1E-10	0.3	Tmem45a	2E-15	0.3
Itpripl2	3E-13	0.3	Chchd1	1E-26	0.3
Lefty1	3E-13	0.3	Rab7b	6E-14	0.3
E2f4	4E-12	0.3	Smg5	3E-16	0.3
Cdr2l	1E-10	0.3	Irf2bp1	9E-13	0.3
Dnajc19	2E-21	0.3	Htatsf1	1E-14	0.3
Nme6	1E-11	0.3	Nap1l3	3E-09	0.3
Mapk1	6E-13	0.3	Agap3	5E-12	0.3
Dipk1a	1E-15	0.3	Usp54	2E-11	0.3
Zc3h12c	2E-13	0.3	Gtf3c1	5E-13	0.3
Cyc1	5E-17	0.3	Eif3b	4E-12	0.3
Tor2a	3E-08	0.3	Gmcl1	3E-13	0.3
Tbc1d10a	9E-14	0.3	Rpia	4E-11	0.3
Plcb3	5E-12	0.3	1700109H08Rik	1E-05	0.3
Tceal9	9E-30	0.3	Msra	2E-14	0.3
Ptges3l	5E-09	0.3	Hacd2	1E-21	0.3
Actr1a	3E-10	0.3	Ogfr	4E-14	0.3
Selenoi	4E-12	0.3	Pitrm1	1E-14	0.3
Sept8	4E-11	0.3	Spata5	5E-14	0.3
Pigo	2E-07	0.3	Memo1	1E-15	0.3
Stag1	8E-14	0.3	Zfp764	2E-05	0.3
Med7	8E-11	0.3	Gbp2	2E-06	0.3
Rrp8	2E-08	0.3	Far1	3E-16	0.3
Pxdc1	2E-12	0.3	Bmper	7E-29	0.3
Gm16867	1E-06	0.3	Pitpnb	2E-15	0.3
Tut4	2E-11	0.3	Slc39a6	1E-13	0.3
Pkd2l2	1E-05	0.3	Cops5	8E-12	0.3
Lmbrd2	4E-10	0.3	Psmd9	9E-16	0.3
Pgls	5E-28	0.3	Nup214	4E-12	0.3
Urb1	4E-07	0.3	Gcsh	1E-15	0.3
Tpbg	1E-14	0.3	Arhgdib	3E-14	0.3
Timm22	3E-13	0.3	Cmip	1E-13	0.3

Undefined

Control			NICD2		
Gene	*p*	FC	Gene	*p*	FC

Articular/Synovial Fibroblast

Control			NICD2		
Gene	*p*	FC	Gene	*p*	FC
Ddit4	7E-01	0.1	Polr3a	2E-01	0.1
Arhgap28	3E-01	0.1	Zfyve27	4E-01	0.1
Gaa	1E-02	0.1	Ankrd39	6E-01	0.1
Adck2	4E-01	0.1	Haghl	5E-01	0.1
Fastkd3	1E-02	0.1	Carf	3E-01	0.1
Fkbp1b	2E-01	0.1	Coq4	1E-01	0.1
Shc1	2E-02	0.1	Tmlhe	8E-01	0.1
Hdac6	1E-01	0.1	Svep1	1E-02	0.1
Pqbp1	2E-01	0.1	Tecpr2	3E-01	0.1
Mbnl3	1E-01	0.1	Plpp6	9E-02	0.1
Mrps31	8E-01	0.1	Lpp	2E-03	0.1
4930402H24Rik	3E-01	0.1	Trim56	2E-01	0.1
Tax1bp3	2E-01	0.1	Pcgf1	3E-01	0.1
Sdha	1E-02	0.1	Atp5j2	2E-04	0.1
Siva1	1E-03	0.1	Osbpl11	8E-02	0.1
Coa6	1E-01	0.1	Polr2a	8E-02	0.1
Sppl3	3E-01	0.1	Zfp974	4E-01	0.1
Gm5617	3E-01	0.1	Nit2	7E-01	0.1
Spopl	7E-01	0.1	Eif2b1	1E-01	0.1
Tomm7	1E-04	0.1	Psme2	7E-02	0.1
Tmem256	4E-03	0.1	Fbxo11	6E-01	0.1
Dpysl2	8E-02	0.1	E130307A14Rik	3E-01	0.1
Lrrc8b	1E-01	0.1	Sdhaf4	4E-02	0.1
Ppcdc	7E-01	0.1	S100a6	2E-04	0.1
Pik3ca	5E-01	0.1	Tmem131l	1E-01	0.1
BC005624	4E-01	0.1	Ikbkg	3E-01	0.1
Zfp780b	1E-01	0.1	Git2	8E-01	0.1
Nxt2	2E-01	0.1	Ap5s1	2E-01	0.1
Rmdn2	4E-01	0.1	Pias1	4E-01	0.1
Clasrp	2E-01	0.1	Tomm7	5E-03	0.1
Nucb1	1E-02	0.1	Fam171b	3E-01	0.1
Bbs2	4E-01	0.1	Inpp4b	2E-01	0.1
Wdr47	2E-01	0.1	Tbp	8E-02	0.1
Tasor	6E-01	0.1	Leng8	2E-01	0.1
Dctn2	6E-03	0.1	Cd302	2E-02	0.1
Socs1	6E-01	0.1	Ap1m1	5E-01	0.1
Dolk	7E-01	0.1	Uqcr11	5E-06	0.1
Nynrin	7E-01	0.1	Ccdc126	2E-01	0.1
Atp6v0c	1E-04	0.1	Fbxo38	1E+00	0.1
Kdm2a	3E-02	0.1	Nsa2	3E-04	0.1
Pcolce	9E-05	0.1	Vwa8	9E-01	0.1
Tubg2	3E-01	0.1	Stx4a	5E-01	0.1
Stag2	1E-01	0.1	Dnajb2	8E-01	0.1
Cln8	2E-01	0.1	Lamtor1	3E-01	0.1
Etv3	3E-01	0.1	Ulk1	3E-01	0.1
Psme2	1E-02	0.1	Trim23	2E-01	0.1
Sp4	3E-01	0.1	1700017B05Rik	3E-02	0.1
Asna1	5E-01	0.1	C1qtnf2	6E-01	0.1
Mtmr9	4E-01	0.1	Eif2a	3E-01	0.1
Snx30	4E-01	0.1	Gon4l	8E-01	0.1
Jpx	4E-01	0.1	Btrc	4E-01	0.1
Babam1	2E-01	0.1	Flot1	3E-01	0.1
Kazn	5E-01	0.1	Edrf1	2E-01	0.1
Nr6a1os	7E-01	0.1	Ppp4r3b	4E-01	0.1
Rfx1	8E-02	0.1	Sephs1	4E-01	0.1
Pgls	3E-04	0.1	Terf2	4E-01	0.1
Mapk9	9E-01	0.1	Hps4	4E-01	0.1
Zfp655	6E-01	0.1	Ap3d1	7E-01	0.1
Pcyt1a	8E-01	0.1	Stag1	3E-02	0.1
Igf1r	3E-02	0.1	Gas6	5E-05	0.1
Src	6E-01	0.1	Ttc12	8E-02	0.1
Qdpr	6E-01	0.1	Rnd2	6E-01	0.1
Tet3	8E-01	0.1	Fam222b	3E-01	0.1
Rmi1	4E-01	0.1	Man2c1os	2E-01	0.1
Laptm4a	1E-06	0.1	Cyhr1	1E-01	0.1
Tfpt	7E-01	0.1	Tubb2b	9E-01	0.1
Atf1	6E-01	0.1	Helq	2E-01	0.1
Ap3b1	1E-01	0.1	Egln2	3E-01	0.1
Sema3f	6E-01	0.1	Hoxc6	8E-01	0.1
Slc38a6	7E-01	0.1	Fam32a	5E-01	0.1
Maged2	6E-02	0.1	Gspt2	7E-01	0.1
Eif2ak1	9E-01	0.1	Ccng2	5E-01	0.1
Bnip3l	3E-03	0.1	Gigyf2	4E-02	0.1

Limb Mesenchyme

Control Gene	p	FC	NICD2 Gene	p	FC
Slc23a2	3E-05	0.2	Sgpl1	1E-04	0.2
Zfand6	1E-06	0.2	Zfyve1	6E-05	0.2
Prdm11	6E-03	0.2	Wipf2	3E-03	0.2
Smarcb1	1E-07	0.2	Ubap2l	3E-11	0.2
Setd1a	5E-05	0.2	Map3k12	4E-04	0.2
Xrcc4	8E-05	0.2	Ikbkb	1E-03	0.2
Wdr45b	3E-05	0.2	Atic	2E-04	0.2
Eny2	2E-11	0.2	Vasp	8E-06	0.2
Orc5	1E-04	0.2	Pced1a	3E-04	0.2
Stim2	2E-05	0.2	Inhba	8E-07	0.2
Syncrip	6E-08	0.2	Elavl1	1E-05	0.2
Fchsd2	6E-06	0.2	B3gnt9	1E-03	0.2
Htatsf1	1E-05	0.2	Atr	7E-03	0.2
Nle1	5E-04	0.2	Akap10	4E-04	0.2
Tatdn2	2E-03	0.2	Loxl2	1E-02	0.2
Large1	9E-02	0.2	Adam10	4E-04	0.2
Copa	2E-09	0.2	Mea1	1E-06	0.2
Mat2b	4E-07	0.2	Zfp106	8E-08	0.2
Ostf1	2E-06	0.2	Mbd6	2E-03	0.2
Rrp15	2E-05	0.2	Tsc1	5E-03	0.2
Tcf20	1E-04	0.2	Cacfd1	9E-04	0.2
Gm1976	1E-04	0.2	Hdac2	2E-05	0.2
Bend6	5E-03	0.2	Wdr83	2E-06	0.2
Ppp2r3c	5E-04	0.2	Rabgap1	5E-05	0.2
Asap2	2E-05	0.2	Ppp4r3b	1E-05	0.2
Bcl10	5E-05	0.2	B230354K17Rik	2E-03	0.2
Klhdc3	9E-06	0.2	Speccl1	3E-06	0.2
Ccny	7E-06	0.2	Inpp5b	4E-03	0.2
Atrx	1E-08	0.2	Rbm34	2E-04	0.2
Prkar2a	7E-05	0.2	Dhx15	4E-06	0.2
Mapre3	1E-04	0.2	C030006K11Rik	2E-02	0.2
Fance	9E-05	0.2	Ccdc115	3E-05	0.2
Traf4	4E-04	0.2	Chmp5	2E-08	0.2
Serpinb9	2E-05	0.2	Polb	2E-05	0.2
Rbm19	1E-04	0.2	Carnmt1	7E-06	0.2
Atg13	7E-05	0.2	Tmem67	4E-03	0.2
Zfp37	1E-03	0.2	Pigs	1E-04	0.2
Col9a2	3E-05	0.2	Plscr3	2E-06	0.2
Vipas39	1E-04	0.2	Mamld1	4E-03	0.2
Slc18b1	8E-05	0.2	Gtf3c6	1E-05	0.2
Toe1	4E-03	0.2	Zdhhc17	7E-04	0.2
Srsf6	2E-07	0.2	Gskip	5E-03	0.2
Klhl12	3E-04	0.2	Ranbp9	2E-03	0.2
Tgds	1E-03	0.2	Wdr24	2E-02	0.2
Csnk1e	1E-04	0.2	Ints5	3E-03	0.2
Stimate	5E-05	0.2	Znrf2	6E-05	0.2
Fam149b	7E-04	0.2	Elovl6	8E-05	0.2
Gm5577	1E-02	0.2	Ubxn4	7E-08	0.2
Mbtps1	6E-06	0.2	Usp15	1E-04	0.2
Usp3	5E-06	0.2	Jam3	2E-04	0.2
Itpripl2	1E-05	0.2	Tmx1	1E-06	0.2
Bcl9	3E-05	0.2	Sp4	2E-03	0.2
4933431E20Rik	3E-03	0.2	Mtdh	2E-08	0.2
Rab5if	9E-09	0.2	Mindy2	3E-04	0.2
Sbno1	6E-07	0.2	Chm	4E-04	0.2
Afg3l1	1E-05	0.2	Zbtb5	3E-03	0.2
Dicer1	1E-04	0.2	Btbd7	3E-04	0.2
Usp9x	7E-09	0.2	Rabggtb	9E-04	0.2
Pip4p2	6E-06	0.2	Usp8	4E-05	0.2
Mllt11	3E-04	0.2	Sos1	2E-03	0.2
Paip2	9E-15	0.2	D830024N08Rik	1E-02	0.2
Zfp639	4E-05	0.2	Armc10	4E-03	0.2
Ppp2r5e	1E-04	0.2	Poc1b	8E-03	0.2
Map3k12	1E-03	0.2	Gm16286	3E-07	0.2
Dixdc1	4E-05	0.2	Tmem161a	9E-04	0.2
Elp5	1E-04	0.2	Pcca	6E-06	0.2
Rnf170	1E-04	0.2	Emc6	1E-06	0.2
Zfp942	2E-03	0.2	Dcun1d2	1E-03	0.2
Iqcb1	3E-04	0.2	Pigu	6E-04	0.2
Pdzd11	3E-06	0.2	Msl2	8E-04	0.2
Inppl1	2E-04	0.2	Rab12	3E-05	0.2
Fbxo46	1E-03	0.2	Miga1	7E-03	0.2
Hars2	2E-03	0.2	Nt5c	4E-06	0.2

Chondrogenic

Control Gene	p	FC	NICD2 Gene	p	FC
Maco1	2E-02	0.1	Clptm1	1E-01	0.1
Ik	1E-04	0.1	Lamtor4	3E-07	0.1
Armcx5	2E-01	0.1	Zfp598	6E-01	0.1
Pex5	2E-01	0.1	Sec16b	3E-01	0.1
Chchd7	7E-05	0.1	L2hgdh	9E-01	0.1
Rybp	2E-01	0.1	Slc25a20	5E-01	0.1
Rnf181	5E-01	0.1	Zc3h7b	7E-01	0.1
Helz	8E-03	0.1	Wwox	2E-03	0.1
Gm10658	8E-01	0.1	Mical1	8E-01	0.1
Zfp706	6E-09	0.1	Zfp983	4E-01	0.1
Akirin2	5E-04	0.1	Kiz	6E-02	0.1
Fbxo42	6E-02	0.1	Izumo4	4E-01	0.1
Ago4	4E-01	0.1	Dhx34	8E-01	0.1
Asf1a	2E-02	0.1	Slc39a13	4E-02	0.1
Wdr83os	4E-08	0.1	Prnp	3E-08	0.1
Chd4	3E-06	0.1	Pex13	7E-03	0.1
Katna1	5E-02	0.1	mt-Co1	1E-27	0.1
Dvl3	1E-01	0.1	Slc25a47	7E-01	0.1
Med1	7E-03	0.1	Mar9	4E-01	0.1
Tcea1	3E-06	0.1	Ddx3y	2E-02	0.1
Trim44	3E-04	0.1	Sept6	1E-01	0.1
Zfp26	3E-01	0.1	Rbbp6	1E-04	0.1
Med12	2E-01	0.1	Mindy1	6E-01	0.1
Dnajb6	9E-09	0.1	Vamp8	1E-04	0.1
Mill2	1E-01	0.1	Vhl	4E-01	0.1
Wdr83	6E-02	0.1	Ermard	9E-01	0.1
Tmem259	3E-02	0.1	Tom1	7E-01	0.1
Nbdy	8E-07	0.1	Rabep1	3E-03	0.1
Fam193b	6E-01	0.1	Lama3	7E-01	0.1
Gspt1	6E-07	0.1	Kri1	1E+00	0.1
Prr14	4E-02	0.1	Smim26	1E-01	0.1
Usp34	8E-08	0.1	Zfp719	4E-01	0.1
Nop2	4E-01	0.1	Cbwd1	2E-01	0.1
Ccdc138	8E-01	0.1	Ccdc112	7E-01	0.1
Ankra2	2E-03	0.1	Abca5	1E+00	0.1
2810006K23Rik	5E-01	0.1	Mtfr1	3E-01	0.1
Tmem126a	1E-06	0.1	Srsf4	1E-02	0.1
Cc2d1b	2E-01	0.1	BC005624	8E-05	0.1
Serp1	3E-04	0.1	Sik3	7E-03	0.1
Sgsm3	5E-01	0.1	Zranb2	9E-06	0.1
Cdadc1	2E-02	0.1	Tmem80	4E-01	0.1
Prpf39	2E-02	0.1	Fbxo8	3E-01	0.1
Tsr3	3E-02	0.1	Anapc4	2E-03	0.1
Arhgef1	2E-02	0.1	Mark3	5E-03	0.1
Rbm6	6E-06	0.1	Rab11fip5	8E-01	0.1
Vps13a	4E-02	0.1	Creld1	3E-01	0.1
2900060B14Rik	3E-01	0.1	Ddx23	7E-02	0.1
Slc38a9	3E-02	0.1	Emc6	3E-05	0.1
Mphosph10	6E-03	0.1	Slc4a1ap	1E-01	0.1
Prkca	2E-01	0.1	Arfgef1	8E-02	0.1
Cntrl	2E-01	0.1	Lyrm2	2E-03	0.1
Rpf1	2E-01	0.1	Rnf123	8E-01	0.1
Mgrn1	2E-01	0.1	Ephx1	1E-02	0.1
Resf1	1E-01	0.1	Zbtb6	5E-01	0.1
Hk1	4E-03	0.1	Sertad2	2E-02	0.1
Mien1	2E-06	0.1	Klhdc2	1E-04	0.1
1300002E11Rik	7E-02	0.1	Zbtb33	5E-01	0.1
Mars	7E-02	0.1	Mettl15	8E-01	0.1
Prickle3	4E-01	0.1	9930104L06Rik	7E-01	0.1
Zfand6	7E-05	0.1	Atf2	1E-02	0.1
Pgrmc1	7E-06	0.1	Med27	5E-01	0.1
Tmem68	6E-01	0.1	Phf7	7E-01	0.1
Hbs1l	1E-02	0.1	Eaf2	7E-01	0.1
Phax	4E-04	0.1	Ftx	4E-01	0.1
Ddx46	2E-04	0.1	Ppm1b	7E-02	0.1
Tob2	2E-01	0.1	Zfp704	2E-01	0.1
Tmem94	3E-01	0.1	Zfyve27	8E-01	0.1
Ccdc107	2E-04	0.1	Slc9a3r1	8E-02	0.1
C87436	2E-01	0.1	Atpsckmt	3E-01	0.1
Trip11	2E-04	0.1	Klhdc1	5E-01	0.1
Gfod2	9E-01	0.1	Fgfr1op2	8E-04	0.1
Ncoa4	6E-02	0.1	Bdp1	2E-01	0.1
Iars	1E-02	0.1	Grwd1	8E-01	0.1

Fibroblast

Control Gene	p	FC	NICD2 Gene	p	FC

Fibroblast?

Control Gene	p	FC	NICD2 Gene	p	FC
Ccdc43	2E-09	0.3	Slc12a7	7E-09	0.3
Txnl4a	8E-16	0.3	Tmem98	1E-14	0.3
Yif1b	1E-13	0.3	Gnb2	2E-29	0.3
Spsb1	2E-10	0.3	Dusp7	6E-15	0.3
2700054A10Rik	9E-08	0.3	Foxred1	4E-08	0.3
Rhoc	1E-22	0.3	Gab1	2E-12	0.3
Cops3	7E-15	0.3	Mfsd10	2E-11	0.3
Dnase2a	3E-10	0.3	Tmem19	2E-12	0.3
Tnpo3	4E-10	0.3	Acot9	2E-14	0.3
Phb2	4E-13	0.3	Xrn2	3E-19	0.3
Dock5	3E-14	0.3	Dclk2	3E-10	0.3
Lage3	7E-14	0.3	A430005L14Rik	5E-12	0.3
Gorab	1E-08	0.3	Hsph1	1E-16	0.3
Slc7a5	2E-11	0.3	E2f4	4E-11	0.3
Tshz1	4E-10	0.3	N4bp1	2E-14	0.3
Ttc13	4E-12	0.3	Asns	4E-13	0.3
Abhd6	2E-06	0.3	Sbk1	5E-07	0.3
Gfod2	8E-08	0.3	Tysnd1	1E-12	0.3
Lars	5E-15	0.3	Repin1	4E-08	0.3
Cryl1	5E-12	0.3	Armt1	5E-15	0.3
Jcad	8E-07	0.3	Twistnb	1E-11	0.3
5031439G07Rik	4E-07	0.3	St3gal4	2E-17	0.3
Serhl	3E-10	0.3	Arl2bp	4E-12	0.3
Rab31	7E-12	0.3	Tead1	1E-20	0.3
Uxs1	1E-12	0.3	Gtf2f1	2E-10	0.3
Plpbp	5E-07	0.3	Zbp1	2E-06	0.3
Pias1	9E-09	0.3	Pprc1	3E-07	0.3
Il6ra	2E-06	0.3	Rhog	1E-15	0.3
Enpp4	2E-10	0.3	Tpi1	1E-30	0.3
Timm29	1E-10	0.3	Vegfb	3E-18	0.3
Nab2	2E-10	0.3	Tcf20	7E-13	0.3
Phlpp1	4E-12	0.3	Pxdc1	4E-16	0.3
Sec11c	4E-14	0.3	Pde4b	4E-11	0.3
Bod1	4E-11	0.3	Trappc1	4E-09	0.3
D130017N08Rik	1E-11	0.3	Cebpg	9E-13	0.3
Gnai2	3E-36	0.3	Dpp9	4E-16	0.3
Gm15675	4E-07	0.3	Stim1	9E-14	0.3
Arsb	1E-11	0.3	Itpk1	1E-08	0.3
Got2	1E-10	0.3	Pnpt1	2E-14	0.3
Rps19bp1	2E-10	0.3	Fem1c	5E-14	0.3
Pot1b	2E-13	0.3	Cops7b	1E-12	0.3
Lrrc42	7E-10	0.3	Tmbim1	1E-10	0.3
Crlf3	6E-13	0.3	Pwp1	3E-14	0.3
Efs	2E-10	0.3	Cryab	5E-12	0.3
Ptpa	7E-12	0.3	4930453N24Rik	2E-15	0.3
Hpcal1	1E-10	0.3	Ehd2	4E-20	0.3
Dtwd1	4E-05	0.3	Dyrk1a	7E-15	0.3
Rtf1	4E-14	0.3	Ogg1	3E-10	0.3
Vdac3	4E-21	0.3	Tmtc4	3E-22	0.3
Pck2	9E-12	0.3	Brpf3	1E-14	0.3
Lta4h	4E-12	0.3	Cfl2	6E-18	0.3
Aldh4a1	9E-09	0.3	Nploc4	8E-14	0.3
Srprb	1E-11	0.3	Ift22	2E-13	0.3
Nipsnap3b	9E-08	0.3	Myzap	3E-12	0.3
Eya2	1E-06	0.3	Erf	8E-15	0.3
Ranbp2	5E-10	0.3	Tpst2	8E-15	0.3
Wapl	1E-12	0.3	Ltbp1	2E-14	0.3
Zfp763	4E-10	0.3	Utp11	6E-12	0.3
Mlf2	6E-12	0.3	Rap1a	8E-14	0.3
Sdc4	5E-12	0.3	Wsb2	2E-09	0.3
Wbp4	6E-15	0.3	Mtr	1E-14	0.3
Gm47710	3E-09	0.3	Polr2l	3E-12	0.3
Mboat7	4E-13	0.3	Lars	2E-19	0.3
Tle5	3E-22	0.3	Ap2s1	2E-16	0.3
Wdr36	7E-11	0.3	Nmi	3E-12	0.3
Dnajb11	5E-17	0.3	Cyc1	7E-19	0.3
Utp18	2E-09	0.3	8030462N17Rik	3E-14	0.3
Magt1	5E-13	0.3	Tmem63a	5E-18	0.3
Sinhcaf	3E-08	0.3	Plcd3	2E-15	0.3
Setdb1	6E-07	0.3	Gtf2e2	2E-17	0.3
Cnn2	2E-13	0.3	Ddx46	7E-14	0.3
Slc27a6	8E-05	0.3	Ddx21	2E-07	0.3
Dnajc3	1E-21	0.3	Ppp1r35	9E-15	0.3

Undefined

Control Gene	p	FC	NICD2 Gene	p	FC

Articular/Synovial Fibroblast

Control Gene	p	FC	NICD2 Gene	p	FC
Scaper	5E-01	0.1	Alg6	2E-01	0.1
Fkrp	1E-01	0.1	Rsf1	2E-01	0.1
Mtfmt	3E-01	0.1	Mcrs1	7E-01	0.1
Zfp316	4E-01	0.1	Csrp2	3E-01	0.1
Casp3	4E-01	0.1	Cd9	1E-01	0.1
Gm10785	1E-01	0.1	Sugp1	9E-01	0.1
Mrpl23	2E-03	0.1	Btg1	1E-01	0.1
Lin37	4E-01	0.1	Rps10	1E-09	0.1
Gpi1	3E-02	0.1	Glyr1	2E-01	0.1
Mrps33	8E-03	0.1	Dnajc18	1E+00	0.1
Slc25a26	1E-01	0.1	Rps3	8E-09	0.1
Nagk	2E-01	0.1	Prkaca	9E-01	0.1
Malat1	3E-06	0.1	Hadh	2E-01	0.1
Txnrd3	1E-01	0.1	Glud1	2E-01	0.1
Gtpbp10	2E-01	0.1	Cuta	2E-01	0.1
Zfp111	3E-02	0.1	Dpm1	1E-01	0.1
Vasn	6E-01	0.1	Nmrk1	4E-01	0.1
Mavs	2E-01	0.1	Rab40c	3E-01	0.1
Ubxn2b	2E-01	0.1	Hist1h2be	1E-01	0.1
Nek4	6E-01	0.1	Paxx	8E-01	0.1
Rptor	8E-01	0.1	Cnot4	2E-01	0.1
Ptgr2	8E-01	0.1	Dock1	2E-01	0.1
Ccdc61	8E-01	0.1	Mettl17	7E-02	0.1
Ick	5E-01	0.1	Plbd2	8E-02	0.1
Xrn1	7E-02	0.1	Slc41a1	4E-01	0.1
Fibp	7E-02	0.1	Car13	7E-01	0.1
Cc2d1b	4E-01	0.1	Rpl22	2E-08	0.1
Gna13	9E-01	0.1	Scap	2E-01	0.1
Asxl2	6E-01	0.1	Hist1h2bc	2E-03	0.1
Dis3l2	7E-01	0.1	Hagh	5E-01	0.1
Sirt4	4E-01	0.1	Pcdhb17	6E-01	0.1
Sept10	9E-01	0.1	Nkiras2	3E-01	0.1
Antxr2	8E-01	0.1	Rufy1	4E-01	0.1
Mmd	7E-01	0.1	Ttc28	2E-01	0.1
Asb13	2E-01	0.1	Abhd16a	8E-01	0.1
Gdf15	8E-01	0.1	Mettl26	2E-01	0.1
Got2	2E-01	0.1	Zhx2	8E-02	0.1
Ep300	5E-01	0.1	Tmem256	2E-02	0.1
Spice1	5E-01	0.1	Oaz2	1E+00	0.1
Phactr2	9E-01	0.1	Zfp65	2E-01	0.1
Lmo1	7E-01	0.1	Vps16	4E-01	0.1
Tuba1c	7E-02	0.1	Gcc1	2E-01	0.1
Rela	9E-01	0.1	Jmjd1c	7E-01	0.1
Anapc10	1E+00	0.1	Kctd2	4E-01	0.1
Abl2	5E-01	0.1	Igbp1	4E-01	0.1
Fads1	8E-01	0.1	Sp4	4E-01	0.1
Vps45	9E-02	0.1	Sdhaf3	2E-01	0.1
Tmem19	9E-01	0.1	Taf6	5E-01	0.1
Osbpl9	8E-02	0.1	Kmt5c	2E-01	0.1
Gdap2	4E-01	0.1	Fam120c	4E-01	0.1
Ppfibp2	1E-01	0.1	Amz2	8E-01	0.1
Sharpin	3E-01	0.1	Rpl35	1E-06	0.1
Usp54	4E-01	0.1	Araf	1E+00	0.1
Tmem176a	3E-02	0.1	Mxd1	5E-01	0.1
Pigv	1E-01	0.1	Bcas2	8E-02	0.1
Dhdds	6E-01	0.1	Clec16a	6E-01	0.1
Kctd11	4E-01	0.1	Numb	5E-01	0.1
Psd3	4E-01	0.1	Sugp2	5E-01	0.1
Mettl3	3E-01	0.1	Dbnl	4E-01	0.1
Ap2a2	3E-01	0.1	Mn1	2E-01	0.1
Zfp799	4E-01	0.1	0610030E20Rik	5E-01	0.1
Rcbtb2	6E-01	0.1	Ankrd40	3E-01	0.1
Aasdh	3E-01	0.1	Nr1h2	9E-01	0.1
Gm9856	4E-01	0.1	Gm26887	2E-01	0.1
Hoxc4	9E-01	0.1	Rad54l2	6E-01	0.1
B3galt4	3E-01	0.1	Mcm3ap	8E-01	0.1
Tmem141	1E+00	0.1	1110059G10Rik	1E+00	0.1
Mcl1	7E-02	0.1	D130020L05Rik	5E-01	0.1
Prkra	3E-01	0.1	Simc1	2E-01	0.1
Zfp445	5E-01	0.1	Fam160a2	6E-01	0.1
Pofut1	7E-01	0.1	Rps4x	4E-06	0.1
Pten	1E-02	0.1	2810433D01Rik	3E-01	0.1
Pcid2	1E-01	0.1	Sipa1l3	5E-01	0.1

Limb Mesenchyme

Control			NICD2		
**Gene**	***p***	**FC**	**Gene**	***p***	**FC**
Ppm1a	7E-06	0.2	Ppp2r3a	4E-06	0.2
Chmp7	2E-04	0.2	Ctxn1	4E-05	0.2
Rlim	3E-06	0.2	Cnpy3	5E-04	0.2
Polr2d	2E-07	0.2	Ncoa1	3E-03	0.2
Jam3	1E-05	0.2	Mterf3	1E-03	0.2
Sft2d1	7E-04	0.2	Apbb1	4E-04	0.2
Opa1	6E-06	0.2	Arpc2	3E-14	0.2
Gtpbp4	5E-07	0.2	Dnajb11	1E-05	0.2
Golga1	5E-04	0.2	Rnf123	3E-03	0.2
Rassf3	8E-05	0.2	Midn	2E-04	0.2
Ficd	3E-04	0.2	Map4	1E-06	0.2
Srp9	4E-13	0.2	Gapvd1	1E-04	0.2
Pex3	6E-04	0.2	Pfkfb2	2E-02	0.2
Drosha	7E-03	0.2	Maip1	5E-05	0.2
Gm36198	1E-02	0.2	Tor1aip2	4E-05	0.2
Eif2b5	2E-06	0.2	Auts2	4E-06	0.2
Numa1	1E-06	0.2	Sypl	9E-06	0.2
Zfp142	5E-03	0.2	Skil	1E-04	0.2
Wdr83	1E-04	0.2	Akap8	3E-03	0.2
Sf3b4	2E-05	0.2	Dgcr8	3E-03	0.2
B230219D22Rik	3E-08	0.2	Ercc6l2	3E-04	0.2
Jrk	5E-02	0.2	Hmgn1	4E-18	0.2
Prxl2c	2E-04	0.2	Snrpd3	5E-09	0.2
Wdr82	2E-04	0.2	Map3k11	4E-02	0.2
Fto	1E-06	0.2	Smim14	5E-07	0.2
Uba3	1E-03	0.2	Rbbp9	7E-05	0.2
Zfp867	2E-03	0.2	Zmym4	3E-05	0.2
Kctd6	8E-05	0.2	Urm1	4E-04	0.2
Jagn1	2E-05	0.2	Fip1l1	8E-06	0.2
Zdhhc9	2E-04	0.2	Actb	2E-08	0.2
Insr	3E-05	0.2	Poldip3	5E-03	0.2
Hnrnpa2b1	1E-13	0.2	2700069I18Rik	3E-04	0.2
Cox6a1	3E-13	0.2	Yipf5	1E-07	0.2
Tbl3	1E-03	0.2	Tcaim	2E-03	0.2
Sprtn	2E-03	0.2	Antxr2	6E-06	0.2
Gm9844	2E-03	0.2	Kdelr2	8E-10	0.2
Zfyve16	2E-04	0.2	Ctr9	8E-05	0.2
Rnf40	2E-04	0.2	Guk1	4E-06	0.2
Nktr	2E-07	0.2	Cmas	7E-05	0.2
Eif3d	2E-05	0.2	Thoc1	6E-03	0.2
Sav1	6E-04	0.2	Ddx3x	5E-05	0.1
Zfp979	1E-02	0.2	Zfp979	2E-02	0.1
Trub1	7E-05	0.2	Emilin1	5E-04	0.1
9430060I03Rik	1E-02	0.2	Supv3l1	5E-03	0.1
Cdkal1	5E-05	0.2	Prpf6	2E-05	0.1
Pym1	5E-04	0.2	Qser1	1E-04	0.1
Cdk4	6E-09	0.2	Alyref2	5E-03	0.1
Sun1	1E-04	0.2	Larp4	6E-04	0.1
Nif3l1	2E-03	0.2	Hp1bp3	8E-10	0.1
Zswim4	6E-03	0.2	Thumpd1	1E-05	0.1
Npm1	2E-19	0.2	Phb	4E-02	0.1
Ccz1	1E-04	0.2	Adipor1	4E-06	0.1
Cdc73	1E-04	0.2	Snx27	4E-05	0.1
Npm3	2E-06	0.2	Phc3	5E-05	0.1
Zc3h7b	3E-04	0.2	Chchd4	1E-03	0.1
Zfp1	2E-05	0.2	Minpp1	4E-03	0.1
Ube2v2	2E-05	0.2	Mob1a	3E-04	0.1
Snx4	2E-05	0.2	Dis3l	3E-03	0.1
Obsl1	3E-04	0.2	Pfkl	1E-04	0.1
Smg5	1E-03	0.2	Dvl1	2E-03	0.1
Scyl2	6E-04	0.2	Plxdc2	3E-05	0.1
Galnt11	4E-06	0.2	Pard3	2E-06	0.1
Cdc5l	2E-05	0.2	Son	5E-11	0.1
Phf2	9E-05	0.2	Cyp51	9E-02	0.1
Chd6	3E-06	0.2	Emsy	2E-04	0.1
Rbm48	8E-04	0.2	Slx1b	4E-03	0.1
Spry1	6E-03	0.2	Rev1	4E-03	0.1
Ncl	2E-15	0.2	Cry2	5E-03	0.1
Dusp10	2E-03	0.2	Myo9b	3E-04	0.1
Rbl2	3E-06	0.2	Tmed2	4E-12	0.1
Limd1	4E-05	0.2	Hspd1	5E-11	0.1
Bahcc1	1E-04	0.2	Arf1	3E-10	0.1
Fam199x	3E-04	0.2	Uros	3E-04	0.1

Chondrogenic

Control			NICD2		
**Gene**	***p***	**FC**	**Gene**	***p***	**FC**
Cdk11b	7E-05	0.1	Zkscan3	2E-02	0.1
Nupl2	7E-01	0.1	Ube2b	2E-07	0.1
Gm48742	2E-01	0.1	Cry1	8E-01	0.1
Mocs3	5E-01	0.1	Daglb	6E-01	0.1
Rpp14	3E-01	0.1	Tcp11l1	4E-01	0.1
Islr	1E-01	0.1	Sec31a	4E-08	0.1
Cog5	5E-03	0.1	Zfp1	3E-01	0.1
Clptm1	3E-02	0.1	Gas2l1	9E-01	0.1
Cpsf6	6E-03	0.1	Ccz1	2E-02	0.1
Senp6	3E-06	0.1	Skiv2l	9E-01	0.1
Stx16	7E-05	0.1	Scarb2	2E-02	0.1
Gart	1E-01	0.1	Ccdc141	5E-01	0.1
Rpp30	2E-01	0.1	Scai	2E-01	0.1
1110051M20Rik	2E-02	0.1	Zfp189	9E-01	0.1
1110065P20Rik	8E-06	0.1	Zmiz1	4E-03	0.1
B3gntl1	7E-01	0.1	Ndufa12	2E-05	0.1
B3galt6	9E-01	0.1	Arfip1	9E-02	0.1
Ormdl1	1E+00	0.1	Naa15	1E-02	0.1
Cdon	8E-02	0.1	Mrpl2	4E-02	0.1
5430416N02Rik	6E-01	0.1	Slc25a36	3E-02	0.1
Nck1	2E-03	0.1	Cdpf1	5E-01	0.1
Fkbpl	6E-01	0.1	Golph3l	6E-01	0.1
Tceanc2	1E-01	0.1	Nabp2	4E-03	0.1
Rnmt	8E-02	0.1	Timm22	7E-02	0.1
Zfp692	5E-01	0.1	Fam114a2	1E-04	0.1
Nt5dc2	2E-01	0.1	Mylip	2E-01	0.1
Mprip	1E-05	0.1	Wdr36	5E-03	0.1
Ube2b	1E-08	0.1	Rabl6	9E-03	0.1
Gm16541	7E-01	0.1	Insig1	6E-01	0.1
Slc25a47	5E-01	0.1	Actr8	4E-01	0.1
Orc3	1E-01	0.1	Smpd2	8E-01	0.1
1700123M08Rik	6E-01	0.1	Brox	1E-01	0.1
Zfp959	7E-01	0.1	Zswim1	1E+00	0.1
Fam133b	2E-02	0.1	Serinc3	9E-03	0.1
Pik3ca	1E-02	0.1	Plekhm1	8E-01	0.1
Yaf2	3E-02	0.1	Ccdc30	9E-01	0.1
Fam204a	2E-03	0.1	Nlk	2E-01	0.1
Gpr108	1E-01	0.1	Fbxo38	2E-01	0.1
Slc30a5	5E-03	0.1	Lrpap1	4E-06	0.1
Canx	2E-09	0.1	Fbxo11	7E-04	0.1
Speg	9E-01	0.1	Ddx50	9E-06	0.1
Snhg12	9E-01	0.1	Oaz2	6E-03	0.1
Yipf1	7E-02	0.1	Zfp512	8E-01	0.1
Zfp664	3E-02	0.1	Hadha	6E-07	0.1
Prepl	4E-01	0.1	Cep290	4E-01	0.1
Naa25	2E-01	0.1	1110019D14Rik	1E+00	0.1
Srp72	8E-07	0.1	Sdhaf1	3E-02	0.1
Gm35330	3E-01	0.1	Tmem138	3E-01	0.1
Hax1	2E-03	0.1	Zfp617	5E-01	0.1
Plekha3	2E-02	0.1	Ahdc1	3E-01	0.1
Rhbdd3	2E-01	0.1	Hprt	4E-02	0.1
Ergic3	2E-08	0.1	Alg5	3E-02	0.1
Map2k7	3E-01	0.1	2810004N23Rik	6E-03	0.1
Minpp1	7E-02	0.1	Gm2a	4E-02	0.1
Pccb	3E-01	0.1	Zbtb1	6E-01	0.1
Abcf1	2E-06	0.1	Tnfrsf10b	6E-01	0.1
Clstn1	4E-03	0.1	Ranbp2	1E-02	0.1
Tgfbrap1	1E-01	0.1	Rsl1d1	6E-05	0.1
Tsen54	9E-01	0.1	Nudt8	1E-01	0.1
Nfkbib	3E-01	0.1	Acad10	7E-01	0.1
Cryzl1	4E-03	0.1	Cog3	2E-01	0.1
Lsm1	9E-05	0.1	Zscan21	7E-01	0.1
Camsap1	1E-01	0.1	Scrn3	6E-01	0.1
Bcat2	1E-01	0.1	Dhrs11	4E-01	0.1
Gm10762	2E-01	0.1	Serinc5	1E-01	0.1
Tnpo1	9E-03	0.1	Tmem17	8E-01	0.1
Atxn7l1	3E-01	0.1	Rhot1	3E-01	0.1
A430005L14Rik	2E-02	0.1	Zfand6	3E-04	0.1
Hist3h2a	1E-01	0.1	Polr2k	2E-06	0.1
Pdpr	4E-01	0.1	Baz2b	4E-03	0.1
Sun1	2E-01	0.1	Fbf1	6E-01	0.1
Prrc1	1E-04	0.1	Bcl2l2	9E-01	0.1
Bptf	9E-04	0.1	Bptf	3E-03	0.1

Fibroblast

Control			NICD2		
**Gene**	***p***	**FC**	**Gene**	***p***	**FC**

Fibroblast?

Control			NICD2		
**Gene**	***p***	**FC**	**Gene**	***p***	**FC**
Abce1	2E-11	0.3	Acad8	2E-08	0.3
Tars2	8E-13	0.3	Kctd6	2E-11	0.3
Brms1	1E-07	0.3	Ube2g2	3E-09	0.3
Fstl3	2E-07	0.3	Nek8	7E-13	0.3
Hacd2	2E-17	0.3	Garem1	3E-14	0.3
Tmtc3	5E-12	0.3	Esd	6E-10	0.3
Ripor1	2E-11	0.3	Utp23	3E-13	0.3
Plekha4	1E-09	0.3	Uros	2E-05	0.3
Ssb	3E-24	0.3	Zswim9	4E-11	0.3
Bax	8E-17	0.3	Rad23b	1E-12	0.3
Prickle2	6E-06	0.3	Zc3h15	7E-24	0.3
Nsf	1E-08	0.3	Zfp334	2E-07	0.3
Mrgbp	2E-13	0.3	Iws1	5E-12	0.3
Snx3	2E-23	0.3	Cd34	3E-13	0.3
Fat1	3E-12	0.3	Lsm12	2E-20	0.3
Gas8	2E-07	0.3	Cox5a	6E-30	0.3
Zfp976	2E-04	0.3	Bod1	9E-15	0.3
Btg3	2E-04	0.3	Cyb5r3	1E-25	0.3
Fam120a	2E-12	0.3	Htt	4E-14	0.3
Mettl6	2E-09	0.3	Chchd6	1E-11	0.3
Mpg	3E-07	0.3	Ube2l3	7E-20	0.3
Fhl2	1E-15	0.3	Hbegf	2E-15	0.3
Mtmr4	1E-13	0.3	Snrpn	7E-06	0.3
Fbxo30	2E-09	0.3	Map3k11	5E-11	0.3
Srsf6	2E-13	0.3	Prmt1	1E-11	0.3
Pag1	5E-12	0.3	Cdkl2	2E-09	0.3
Plxnb1	3E-05	0.3	Cd82	3E-13	0.3
Macf1	4E-12	0.3	Cavin2	1E-14	0.3
Npepl1	3E-12	0.3	9930012K11Rik	3E-07	0.3
Wdr5	1E-10	0.3	Adar	2E-09	0.3
Letm1	4E-11	0.3	Thap1	3E-08	0.3
Actr1b	2E-12	0.3	Hcfc1	2E-13	0.3
Hnrnph1	5E-10	0.3	Wipf1	6E-16	0.3
2310022A10Rik	2E-13	0.3	Inpp5b	1E-13	0.3
Htatsf1	9E-10	0.3	Ptgis	4E-13	0.3
Cct6a	2E-15	0.3	Bax	2E-24	0.3
Optn	2E-13	0.3	Fopnl	8E-10	0.3
Umad1	5E-09	0.3	S100pbp	9E-15	0.3
Arglu1	4E-17	0.3	Slc12a6	5E-13	0.3
Plcd3	4E-09	0.3	Kirrel3	5E-11	0.3
Eno1b	2E-05	0.3	Arhgef19	7E-12	0.3
Mink1	2E-10	0.3	Ep400	2E-15	0.3
Smad2	2E-07	0.3	Taf10	4E-24	0.3
Chchd3	1E-11	0.3	Eef1e1	3E-10	0.3
Tgs1	2E-11	0.3	Sh3bp1	9E-12	0.3
Emilin2	6E-09	0.3	Strn4	4E-12	0.3
Rtn4ip1	6E-07	0.3	Zfp410	1E-10	0.3
Zfp653	1E-05	0.3	Dlgap1	5E-09	0.3
Ddx21	2E-05	0.3	Alkbh2	1E-09	0.3
Ube2a	1E-07	0.3	Cpt1c	2E-12	0.3
Pmm2	9E-10	0.3	Mlst8	2E-08	0.3
Rab32	2E-10	0.3	Psmc3	9E-22	0.3
Rec114	9E-04	0.3	Cmtm7	5E-12	0.3
Exosc9	4E-10	0.3	Mrm3	6E-11	0.3
Sh3bgrl3	3E-21	0.3	Ctxn1	1E-11	0.3
Hpgd	2E-08	0.3	Ucp2	5E-22	0.3
Exosc1	7E-10	0.3	Babam1	4E-10	0.3
Homer3	3E-11	0.3	Stk11	4E-09	0.3
Cstf1	2E-11	0.3	Atn1	5E-10	0.3
Dcaf1	1E-10	0.3	Mrps18c	4E-17	0.3
Galnt10	1E-07	0.3	Mlf2	1E-17	0.3
Fbxo42	7E-09	0.3	Zfp53	2E-09	0.3
Cars2	6E-12	0.3	Ddx56	2E-10	0.3
Oxct1	4E-19	0.3	Ing3	1E-09	0.3
Scly	1E-05	0.3	Ythdc1	3E-13	0.3
Fbl	7E-08	0.3	Mrpl3	3E-12	0.3
Mlycd	1E-05	0.3	Akap8	2E-16	0.3
Cryab	8E-10	0.3	Gm49179	2E-08	0.3
Pacs1	1E-10	0.3	Fam98b	5E-14	0.3
Btaf1	2E-10	0.3	Zdhhc13	3E-14	0.3
Il1rl2	4E-07	0.3	Gnai2	2E-43	0.3
Mrfap1	2E-30	0.3	Aven	1E-10	0.3
Pir	6E-12	0.3	Pafah1b2	3E-11	0.3

Undefined

Control			NICD2		
**Gene**	***p***	**FC**	**Gene**	***p***	**FC**

Articular/Synovial Fibroblast

Control			NICD2		
**Gene**	***p***	**FC**	**Gene**	***p***	**FC**
Dctn1	5E-01	0.1	Cln8	1E-01	0.1
Acsl5	5E-01	0.1	Lrpap1	4E-02	0.1
Atp6v1a	2E-02	0.1	Tigd2	4E-01	0.1
Efna1	2E-01	0.1	Nfkbia	8E-01	0.1
Suclg2	5E-01	0.1	Ubl3	7E-01	0.1
Smarca2	1E-02	0.1	Fig4	3E-01	0.1
Ddx51	2E-01	0.1	Gnb5	2E-01	0.1
Pcnx4	1E+00	0.1	Hgs	5E-01	0.1
Def6	3E-01	0.1	Zfp442	2E-01	0.1
3830406C13Rik	3E-01	0.1	Adgrg2	4E-01	0.1
Wiz	8E-01	0.1	Rprd2	9E-01	0.1
Orc3	9E-01	0.1	Lamtor3	3E-01	0.1
Capns1	8E-04	0.1	Cr1l	3E-01	0.1
Ints3	3E-01	0.1	Ski	2E-01	0.1
Plpp6	2E-01	0.1	Prkab2	4E-01	0.1
Lzts2	6E-01	0.1	Socs2	8E-01	0.1
Stag1	5E-02	0.1	2210016F16Rik	9E-01	0.1
1500004A13Rik	9E-01	0.1	Ppp2r3a	9E-01	0.1
Eng	7E-01	0.1	Paox	2E-01	0.1
Pef1	2E-01	0.1	Ccdc66	6E-01	0.1
Atp6v1f	2E-04	0.1	Ccdc71l	5E-02	0.1
Pnpla6	5E-01	0.1	Pld2	5E-01	0.1
Mien1	4E-02	0.1	Med23	4E-01	0.1
Hbp1	2E-01	0.1	Meis1	5E-01	0.1
Wdr25	3E-01	0.1	AY036118	2E-02	0.1
Klc1	6E-01	0.1	4933407K13Rik	2E-01	0.1
Soga1	5E-01	0.1	Gm30025	4E-01	0.1
Prnp	1E-03	0.1	Crlf1	9E-01	0.1
Usp2	4E-01	0.1	Nek9	6E-01	0.1
Mrpl51	1E-02	0.1	Creg1	8E-03	0.1
Cox7b	8E-06	0.1	Fbxo2	5E-01	0.1
Zfp72	4E-01	0.1	Rpl13	1E-07	0.1
Lypla2	4E-01	0.1	Gtpbp3	4E-01	0.1
Nxf1	5E-01	0.1	Acbd4	7E-01	0.1
Reep3	3E-01	0.1	Snrpn	3E-01	0.1
Uvssa	5E-01	0.1	Ilrun	6E-01	0.1
Tor3a	7E-01	0.1	Wdr55	1E-01	0.1
Nckap5l	4E-01	0.1	E130114P18Rik	3E-01	0.1
Glrx	8E-01	0.1	Slc7a11	3E-02	0.1
St6galnac4	3E-01	0.1	Ubl5	2E-02	0.1
Zbtb14	1E-01	0.1	Aida	9E-01	0.1
Ndufa13	4E-05	0.1	Rpl10a	1E-06	0.1
Zfp324	2E-01	0.1	Tmx3	8E-01	0.1
Snx16	9E-01	0.1	Armh3	2E-01	0.1
Slc18b1	3E-01	0.1	Lifr	3E-02	0.1
Ggcx	1E+00	0.1	H2-Ke6	4E-01	0.1
Galnt11	9E-01	0.1	Tsacc	5E-01	0.1
Erlin2	6E-01	0.1	Lancl2	1E-01	0.1
Zfand5	6E-01	0.1	Zfand2b	9E-01	0.1
Pttg1ip	3E-01	0.1	Ndufa3	2E-02	0.1
Irf9	3E-01	0.1	Rpl6	1E-07	0.1
Ccdc22	2E-01	0.1			
Rfng	2E-01	0.1			
Ube4b	3E-01	0.1			
Smim1	2E-02	0.1			
Mycbp	8E-01	0.1			
Zfp873	3E-01	0.1			
Smg9	1E-01	0.1			
Dipk1b	4E-01	0.1			
Msmo1	1E-01	0.1			
Dut	7E-04	0.1			
Mcm3ap	3E-01	0.1			
Gtf2h2	1E+00	0.1			

Limb Mesenchyme						Chondrogenic						Fibroblast						Fibroblast?						Undefined						Articular/Synovial Fibroblast					
Control			NICD2			Control			NICD2			Control			NICD2			Control			NICD2			Control			NICD2			Control			NICD2		
Gene	*p*	FC	Gene	*p*	FC	Gene	*p*	FC	Gene	*p*	FC	Gene	*p*	FC	Gene	*p*	FC	Gene	*p*	FC	Gene	*p*	FC	Gene	*p*	FC	Gene	*p*	FC	Gene	*p*	FC	Gene	*p*	FC
Mical1	2E-02	0.2	Hif1an	2E-04	0.1	Fndc1	5E-01	0.1	Ptp4a2	5E-07	0.1							Cdk16	7E-11	0.3	Ube2k	2E-23	0.3												
Lrrfip2	2E-05	0.2	Herc2	1E-04	0.1	Eef2kmt	9E-02	0.1	0610030E20Rik	9E-01	0.1							Psmb7	9E-15	0.3	Coa7	2E-08	0.3												
Inip	5E-04	0.2	Rnf11	4E-06	0.1	Mbd3	7E-06	0.1	Tox4	6E-02	0.1							Dyrk1a	4E-11	0.3	Phlpp2	3E-16	0.3												
Zfp68	1E-05	0.2	Trp53cor1	2E-04	0.1	Mon2	1E-02	0.1	Gtf2e2	8E-02	0.1							Mycbp	9E-04	0.3	Abt1	1E-14	0.3												
Exosc4	6E-06	0.2	Atg5	6E-05	0.1	Borcs7	4E-03	0.1	Gm12258	9E-01	0.1							Slc35b4	6E-11	0.3	Ranbp2	7E-14	0.3												
Ube4b	4E-06	0.2	Nipbl	1E-06	0.1	Ldlrap1	6E-01	0.1	Tmem173	6E-01	0.1							Mgat5	3E-12	0.3	Eif4g1	3E-16	0.3												
Gja1	8E-07	0.2	Ctbp1	2E-05	0.1	Gtpbp4	1E-04	0.1	Vasn	5E-02	0.1							Dvl2	3E-11	0.3	Glrx5	4E-17	0.3												
Cdk10	7E-04	0.2	Zbtb1	2E-04	0.1	Trim7	5E-01	0.1	Rubcn	6E-01	0.1							Wipf1	8E-12	0.3	Cops6	1E-18	0.3												
Creb3	2E-06	0.2	Grpel2	2E-03	0.1	Rap2c	8E-02	0.1	Dkk3	1E+00	0.1							Ptk7	1E-06	0.3	Phtf1	2E-14	0.3												
Robo1	5E-12	0.2	Alkbh5	1E-04	0.1	Cdk2ap1	8E-02	0.1	Borcs8	6E-04	0.1							Idh2	2E-13	0.3	Tsn	1E-27	0.3												
Gosr1	2E-04	0.2	Tbp	6E-03	0.1	Tcta	2E-02	0.1	Ino80c	8E-01	0.1							Nr3c1	7E-12	0.3	Gm47710	3E-07	0.3												
G6pc3	2E-04	0.2	Cox16	6E-08	0.1	Rnf20	4E-04	0.1	Clip3	3E-01	0.1							Tbc1d15	5E-13	0.3	Cavin1	3E-16	0.3												
Nol12	4E-06	0.2	Tmem248	5E-04	0.1	Gsk3a	2E-01	0.1	Dmtf1	2E-01	0.1							2200002D01Rik	2E-08	0.3	Sec11c	1E-17	0.3												
Myo19	2E-02	0.2	Trappc10	3E-04	0.1	Frg2f1	6E-01	0.1	Cpsf1	9E-01	0.1							Cpne1	6E-05	0.3	Nckap1	2E-13	0.3												
Grpel1	7E-08	0.2	Ube3b	3E-03	0.1	Pdxdc1	2E-04	0.1	Ift81	2E-01	0.1							Urb2	9E-08	0.3	Taf4	6E-12	0.3												
Ccar2	2E-02	0.2	Cul2	6E-04	0.1	Fam13b	3E-03	0.1	Dop1a	2E-01	0.1							Mms19	8E-11	0.3	Utp18	4E-11	0.3												
Atg9a	2E-04	0.2	Prrc2c	7E-10	0.1	Alg5	3E-03	0.1	Fuca2	8E-01	0.1							Cox10	6E-10	0.3	Inhba	5E-11	0.3												
Rhod	1E-04	0.2	Gtpbp4	9E-06	0.1	Ikbke	5E-01	0.1	Zfp322a	2E-01	0.1							Igfbp6	1E-06	0.3	Prr5	1E-08	0.3												
Sart1	6E-04	0.2	Mkln1	1E-03	0.1	Wdr5	9E-01	0.1	Preb	4E-01	0.1							Plrg1	2E-10	0.3	Sdf2l1	2E-14	0.3												
Rhobtb1	2E-02	0.2	Zfp865	7E-04	0.1	4632427E13Rik	2E-02	0.1	Trak1	3E-01	0.1							Rhoa	6E-24	0.3	Mier2	1E-06	0.3												
Spred1	4E-07	0.2	Znhit2	8E-04	0.1	Xndc1	6E-01	0.1	4931406C07Rik	1E-02	0.1							Mtap	2E-07	0.3	Ppp1r14b	3E-23	0.3												
Zc3h7a	7E-06	0.2	Puf60	3E-05	0.1	Dennd1b	8E-02	0.1	Efna4	9E-01	0.1							Trmt2a	3E-09	0.3	Fam234b	2E-07	0.3												
Nfx1	2E-06	0.1	Tcf3	2E-04	0.1	Snapc5	1E-03	0.1	Scfd2	6E-01	0.1							Ndufb8	4E-21	0.3	Slc25a22	4E-07	0.3												
Lrrc40	1E-03	0.1	Rexo1	7E-03	0.1	Snx14	4E-01	0.1	Tbl1xr1	2E-04	0.1							Slc41a3	5E-09	0.3	Lrfn4	2E-06	0.3												
Tango6	9E-04	0.1	Snhg17	6E-03	0.1	Polrmt	7E-01	0.1	2310015A10Rik	9E-01	0.1							Acat1	1E-12	0.3	Mmadhc	3E-11	0.3												
Emilin1	5E-05	0.1	Leo1	9E-05	0.1	Upp2	3E-02	0.1	Nudt13	8E-01	0.1							Mb21d2	2E-08	0.3	Tmsb10	7E-31	0.3												
Hnrnpr	1E-09	0.1	Tram1	3E-06	0.1	Ubxn2b	4E-01	0.1	Eef2kmt	8E-03	0.1							Bclaf1	4E-15	0.3	Fam162a	5E-20	0.2												
Cdc23	3E-04	0.1	8430429K09Rik	7E-03	0.1	Msh2	2E-01	0.1	Tln1	7E-06	0.1							Cyld	1E-05	0.3	Mrpl15	6E-08	0.2												
Taz	1E-04	0.1	Bmpr2	3E-06	0.1	Snrnp200	1E-01	0.1	Jmjd7	3E-01	0.1							Ppp1r35	2E-07	0.3	Letm1	2E-14	0.2												
Cpsf6	3E-05	0.1	Pdcl3	9E-06	0.1	Hsf2	4E-01	0.1	Psenen	1E-06	0.1							Zfp219	1E-10	0.3	Nox4	9E-17	0.2												
Ctdp1	2E-02	0.1	Zfp950	1E-04	0.1	Sft2d3	2E-01	0.1	Ndfip1	2E-09	0.1							Thbd	9E-12	0.3	Smad1	7E-15	0.2												
Knop1	3E-05	0.1	Stx18	2E-05	0.1	Txn2	4E-05	0.1	Mak16	1E-02	0.1							Far1	3E-09	0.3	Ercc6l2	8E-09	0.2												
Selenof	1E-19	0.1	AI480526	5E-03	0.1	Gm20404	1E+00	0.1	Ak3	1E-01	0.1							Ngdn	2E-06	0.3	Itpripl2	4E-12	0.2												
Fbxl14	3E-03	0.1	Txn2	4E-07	0.1	Zfp383	4E-01	0.1	Zfp746	2E-01	0.1							Ten1	2E-12	0.3	Fscn1	8E-15	0.2												
Gm4221	3E-03	0.1	Fbxw17	3E-04	0.1	Atl1	6E-01	0.1	Efna1	1E+00	0.1							Zc3h4	3E-11	0.3	Pip5k1b	1E-15	0.2												
Avpi1	5E-06	0.1	Exosc10	2E-02	0.1	Glb1l	3E-01	0.1	Mpv17l	8E-01	0.1							Tpcn1	1E-09	0.3	Mid2	2E-16	0.2												
Mthfsd	1E-04	0.1	Poli	1E-01	0.1	Atpsckmt	2E-01	0.1	Abcc1	5E-01	0.1							Grk5	8E-10	0.3	Hikeshi	5E-17	0.2												
Ubald1	1E-04	0.1	Cops2	2E-06	0.1	Smndc1	1E-02	0.1	Maml1	5E-01	0.1							Etaa1os	2E-04	0.3	Crim1	4E-08	0.2												
Mfsd4a	3E-04	0.1	Cdc42bpb	3E-04	0.1	Dcaf1	8E-03	0.1	Fgfr1op	2E-01	0.1							Rasa3	6E-21	0.3	Psmg1	2E-05	0.2												
Ubfd1	9E-05	0.1	Ilkap	2E-06	0.1	Inpp5k	2E-01	0.1	Hsf1	2E-01	0.1							Faap100	6E-08	0.3	Mphosph6	3E-09	0.2												
1600020E01Rik	2E-04	0.1	Cc2d1b	1E-02	0.1	Stx17	2E-02	0.1	Tmem128	8E-02	0.1							4921524J17Rik	2E-07	0.3	Pus1	1E-12	0.2												
Rdh13	9E-03	0.1	Fez2	9E-04	0.1	Rnf44	4E-01	0.1	Ptpn13	5E-02	0.1							Matr3	3E-14	0.3	Lrrc8d	2E-11	0.2												
Phip	5E-06	0.1	Nr3c1	1E-04	0.1	Zcchc17	3E-04	0.1	Abcf1	4E-05	0.1							Stim1	6E-09	0.3	Ppp1r13l	1E-07	0.2												
Ddx5	2E-15	0.1	Gramd1a	2E-03	0.1	Tpm2	5E-13	0.1	1810058I24Rik	6E-08	0.1							Pdk4	3E-07	0.3	Rps27l	2E-31	0.2												
Pigm	4E-03	0.1	Kmt2b	6E-02	0.1	Mark3	4E-03	0.1	Tsr3	3E-02	0.1							Fst	3E-14	0.3	Atf7ip	8E-12	0.2												
Kansl3	4E-04	0.1	Ltv1	1E-04	0.1	Eif2b5	1E-02	0.1	Dolpp1	7E-01	0.1							Ebp	1E-09	0.3	Plcl2	2E-13	0.2												
Cep131	1E-02	0.1	Zrsr2	8E-06	0.1	Mrpl1	5E-02	0.1	Atr	8E-01	0.1							Socs4	2E-09	0.3	Supv3l1	4E-11	0.2												
0610009L18Rik	2E-02	0.1	Ppid	5E-06	0.1	Fbxl5	4E-02	0.1	Wdr45	7E-01	0.1							Desi1	3E-10	0.3	Gpat4	2E-13	0.2												
Thrap3	1E-09	0.1	Ap4s1	1E-04	0.1	Tmem98	1E-01	0.1	Rabggtb	8E-02	0.1							Galk1	2E-09	0.3	Agfg1	4E-10	0.2												
Clk3	6E-04	0.1	Eif4a2	5E-08	0.1	Papola	1E-05	0.1	Zmym1	7E-01	0.1							Rap1gds1	6E-11	0.3	Tars2	1E-15	0.2												
Pwp2	2E-02	0.1	Slain2	1E-03	0.1	Fam120b	1E-01	0.1	Trub1	6E-01	0.1							Cetn3	3E-11	0.3	Ints9	4E-14	0.2												
Wrnip1	5E-03	0.1	Lrig3	3E-07	0.1	Armcx4	7E-01	0.1	Tbx4	9E-01	0.1							Pygo2	4E-07	0.3	Dlst	1E-15	0.2												
Rbbp9	8E-04	0.1	Carmn	2E-02	0.1	Chd8	4E-02	0.1	Sec16a	8E-02	0.1							Nme2	5E-40	0.3	Klf16	8E-10	0.2												
Rictor	3E-03	0.1	Trpm4	9E-02	0.1	Map3k3	1E-01	0.1	Trim68	7E-01	0.1							Pign	6E-11	0.3	Zfp39	5E-07	0.2												
Eif3g	2E-06	0.1	Rexo2	2E-12	0.1	Camsap2	3E-02	0.1	Atl3	2E-03	0.1							Epb41	4E-12	0.3	Tut4	1E-11	0.2												
Ccnc	1E-03	0.1	Ppat	9E-04	0.1	Taf6	8E-01	0.1	Med25	3E-01	0.1							B3glct	6E-07	0.3	Dph3	7E-15	0.2												
Adal	4E-04	0.1	Dcaf1	2E-03	0.1	Irf9	9E-01	0.1	Armc8	1E+00	0.1							Eno1	2E-10	0.3	Wdr48	5E-12	0.2												
Tcf3	6E-05	0.1	Dffb	7E-03	0.1	Tmem185a	3E-01	0.1	Srsf9	3E-05	0.1							Plekha3	2E-08	0.3	Erich1	9E-05	0.2												
Dhx36	1E-08	0.1	Nde1	1E-04	0.1	A130010J15Rik	1E+00	0.1	Eps15	3E-01	0.1							Ormdl2	1E-09	0.3	Ldlrap1	7E-14	0.2												
Cox16	2E-09	0.1	Crlf2	2E-05	0.1	Hypk	4E-04	0.1	Paxx	1E-01	0.1							Adcy3	8E-11	0.3	Ehd1	5E-10	0.2												
Cops8	2E-04	0.1	Trp53bp1	2E-04	0.1	Tnfrsf1b	7E-01	0.1	Ppard	4E-01	0.1							Commd2	3E-07	0.3	Pdpn	3E-12	0.2												
Sfr1	3E-14	0.1	Skp1a	2E-10	0.1	Klhl9	2E-03	0.1	Tgfbr2	1E-02	0.1							Myo1b	6E-13	0.3	Smim15	1E-05	0.2												
Uckl1	2E-03	0.1	Arid4b	5E-06	0.1	Pakap.1	5E-02	0.1	Ttc7b	5E-01	0.1							Tmem134	1E-13	0.3	Slc25a11	1E-11	0.2												
Mus81	2E-03	0.1	Wdr4	4E-03	0.1	Trmt2a	9E-01	0.1	Cln5	2E-01	0.1							2310033P09Rik	6E-10	0.3	Dhx38	5E-12	0.2												
Zfp609	1E-04	0.1	Ino80	4E-03	0.1	Taf1b	4E-01	0.1	Nemf	2E-01	0.1							Armcx2	2E-11	0.3	Snx3	6E-24	0.2												
Eif5b	6E-09	0.1	Eml5	2E-03	0.1	Zbtb11	2E-02	0.1	Zmym4	2E-03	0.1							Trappc5	1E-09	0.3	Dtnb	3E-16	0.2												
Terf2ip	9E-06	0.1	Med14	1E-04	0.1	Ubr2	5E-03	0.1	Fth1	3E-08	0.1							Samhd1	5E-13	0.3	Creb3l1	6E-10	0.2												
Tmx1	3E-04	0.1	Tmem39a	1E-04	0.1	Sarnp	1E-06	0.1	Kat7	1E-01	0.1							Glud1	2E-16	0.3	Akr1b3	1E-11	0.2												
Rtca	2E-05	0.1	Cand1	1E-03	0.1	Eipr1	7E-01	0.1	Erlin2	8E-01	0.1							Ehd2	2E-13	0.3	Kcnq5	1E-18	0.2												
Masp1	2E-07	0.1	Gm26542	5E-03	0.1	Gpx4	3E-13	0.1	Amz2	1E-01	0.1							2500004C02Rik	1E-05	0.3	Znhit2	7E-08	0.2												
Denr	2E-08	0.1	Pa2g4	8E-06	0.1	Tfam	3E-01	0.1	Tlr2	1E+00	0.1							Gpat4	8E-12	0.3	Grwd1	3E-12	0.2												
Gpbp1l1	1E-04	0.1	Ro60	6E-04	0.1	Gorasp2	3E-05	0.1	Zfp445	2E-01	0.1							Wdr48	3E-10	0.3	Mosmo	2E-12	0.2												
Btg2	1E-09	0.1	Srsf11	3E-07	0.1	Gemin8	8E-01	0.1	Lsm1	1E-02	0.1							Tmem245	1E-09	0.3	Diaph1	5E-13	0.2												

Limb Mesenchyme						Chondrogenic					
Control			NICD2			Control			NICD2		
Gene	*p*	FC	Gene	*p*	FC	Gene	*p*	FC	Gene	*p*	FC
Eif2s1	1E-05	0.1	Zfp280d	2E-04	0.1	Zfp961	8E-01	0.1	Srsf10	2E-05	0.1
Utp11	2E-06	0.1	Ssr2	2E-06	0.1	Ddx3y	1E-01	0.1	Senp6	7E-05	0.1
Gatad2a	2E-05	0.1	Srpk1	7E-05	0.1	Fat4	2E-02	0.1	Tes	5E-02	0.1
C1qtnf1	2E-04	0.1	Frzb	4E-03	0.1	Hdac6	8E-01	0.1	Trim44	7E-04	0.1
Abcb8	7E-03	0.1	Pex7	6E-04	0.1	Zfp688	5E-01	0.1	Rpap1	1E+00	0.1
Actr1a	1E-04	0.1	Bclaf1	3E-05	0.1	Taldo1	2E-13	0.1	Eif3c	4E-04	0.1
Eps15l1	4E-04	0.1	Sec11a	9E-08	0.1	Hprt	4E-03	0.1	Fam120b	5E-01	0.1
Fam234b	2E-02	0.1	S100pbp	9E-04	0.1	Rnf216	2E-03	0.1	Ephb6	6E-01	0.1
Ppid	1E-05	0.1	Tmem87b	9E-05	0.1	Kif3b	2E-01	0.1	Zfp316	8E-01	0.1
Trmt13	2E-05	0.1	Fnta	1E-05	0.1	Sec16a	6E-03	0.1	Nme1	2E-07	0.1
Atad2b	6E-05	0.1	Banp	3E-04	0.1	Polr3h	4E-02	0.1	Oxsm	9E-01	0.1
Rpgr	2E-02	0.1	Reps1	6E-04	0.1	Pycr1	2E-01	0.1	Dnaja4	5E-01	0.1
Ube4bos1	1E-02	0.1	Cep68	2E-03	0.1	Vps37a	1E-02	0.1	Ocrl	2E-01	0.1
Gm28375	2E-04	0.1	C1galt1c1	4E-02	0.1	Haghl	6E-01	0.1	Srp9	3E-07	0.1
Ankle2	4E-05	0.1	Tmem11	3E-05	0.1	Fbxl17	7E-04	0.1	Tcf25	1E-07	0.1
Zfhx2	1E-03	0.1	Cped1	5E-06	0.1	Btbd3	8E-03	0.1	Kdm5d	5E-01	0.1
Ppp4r3b	2E-05	0.1	Thrap3	1E-07	0.1	Cdkl3	3E-01	0.1	Ccnl2	3E-02	0.1
Ciao3	3E-05	0.1	Slc10a3	2E-03	0.1	Flt3l	7E-01	0.1	Fam53a	2E-01	0.1
Rab21	5E-07	0.1	Atrx	5E-09	0.1	Tfcp2	5E-01	0.1	Ddx28	8E-01	0.1
Zfp651	8E-05	0.1	Riok1	2E-03	0.1	Kpna6	8E-02	0.1	Klhl15	7E-01	0.1
Setd6	1E-01	0.1	Synj2bp	2E-05	0.1	Prr14l	7E-02	0.1	Ago1	3E-01	0.1
Tgfbrap1	2E-03	0.1	Zfp952	5E-04	0.1	Cdk13	5E-04	0.1	Tspan31	9E-04	0.1
Fgfrl1	1E-04	0.1	Dusp12	3E-04	0.1	Zfp943	6E-01	0.1	Optn	3E-01	0.1
Ppie	2E-04	0.1	Tmed1	1E-05	0.1	Zkscan6	7E-01	0.1	1110004F10Rik	1E-04	0.1
Sacs	2E-04	0.1	Tbc1d22a	8E-04	0.1	Rasa1	6E-04	0.1	Ttc32	4E-01	0.1
Derl2	9E-05	0.1	Itpa	9E-04	0.1	Sec22b	4E-03	0.1	Pdcd2l	8E-01	0.1
Yae1d1	1E-05	0.1	Foxn3	6E-05	0.1	Telo2	5E-01	0.1	Scrib	5E-01	0.1
Usp19	2E-04	0.1	Dock7	3E-05	0.1	Tmem39a	9E-03	0.1	Krit1	9E-03	0.1
Rnf220	1E-04	0.1	Blzf1	2E-04	0.1	Crkl	3E-01	0.1	mt-Nd2	1E-15	0.1
Spcs2	3E-10	0.1	Akap17b	2E-02	0.1	Crlf2	2E-02	0.1	Pttg1	1E-01	0.1
Lrrc73	2E-02	0.1	Utp6	3E-03	0.1	Riok1	2E-02	0.1	Prpsap2	7E-01	0.1
Rftn2	8E-07	0.1	Zfp287	9E-02	0.1	Setdb1	7E-02	0.1	Zfp30	6E-01	0.1
Ppp4r2	2E-07	0.1	Ttc37	5E-03	0.1	Fam168b	4E-02	0.1	Agap1	3E-02	0.1
Cox11	9E-05	0.1	Nkapd1	2E-03	0.1	Zfp189	8E-01	0.1	Spast	1E-01	0.1
Nfkbil1	3E-05	0.1	Scoc	4E-08	0.1	Rpl39l	2E-01	0.1	Zfp426	4E-01	0.1
Ccdc130	2E-03	0.1	Cfap300	4E-02	0.1	Qpctl	8E-01	0.1	Prdm10	7E-01	0.1
Clip1	1E-07	0.1	Hist1h3d	3E-01	0.1	Stard8	8E-01	0.1	Crcp	3E-01	0.1
Gm47283	2E-05	0.1	Zc3h15	5E-08	0.1	Zzz3	1E-04	0.1	Stk25	1E-01	0.1
Cwc27	2E-04	0.1	Tbc1d15	3E-03	0.1	Mphosph8	1E-03	0.1	Trim46	3E-01	0.1
P2rx6	4E-02	0.1	Ccdc39	8E-03	0.1	Rel	6E-01	0.1	Msh2	8E-02	0.1
Otud6b	4E-05	0.1	Gm20275	5E-02	0.1	Ddx21	2E-06	0.1	Rasa1	1E-03	0.1
Spast	4E-05	0.1	Rps19bp1	2E-03	0.1	Ppp6r2	5E-01	0.1	Ppfibp1	1E-01	0.1
Ddx1	4E-07	0.1	Gadd45a	2E-13	0.1	Fuca1	5E-06	0.1	Syf2	4E-03	0.1
Phf14	2E-06	0.1	Cpsf7	2E-03	0.1	Nap1l4	2E-05	0.1	Fgd4	4E-01	0.1
Mtif2	3E-05	0.1	Ncoa3	4E-04	0.1	Ttc1	2E-03	0.1	Bmi1	6E-02	0.1
Atf7	3E-06	0.1	Rgp1	4E-04	0.1	Kif1bp	4E-01	0.1	Slc27a4	1E+00	0.1
Pdcd7	2E-04	0.1	Gli3	7E-07	0.1	Zscan26	2E-02	0.1	Tmem129	5E-01	0.1
Slc29a3	4E-04	0.1	Dact3	5E-03	0.1	Gm10130	7E-01	0.1	Rps6kc1	2E-01	0.1
Tanc2	7E-06	0.1	Cacul1	7E-04	0.1	Tom1l1	5E-01	0.1	Tram2	6E-02	0.1
Med23	3E-04	0.1	Dock11	2E-01	0.1	Grk5	5E-01	0.1	Coq2	6E-02	0.1
Ep300	6E-04	0.1	Gas2l1	8E-04	0.1	Qser1	3E-02	0.1	Ginm1	8E-05	0.1
Map3k7	5E-04	0.1	Dennd5a	4E-05	0.1	Cplane1	4E-02	0.1	Lats1	9E-02	0.1
Fam120a	4E-05	0.1	Ppp1cb	2E-06	0.1	Slc20a2	2E-01	0.1	Zfp846	5E-01	0.1
Ivd	2E-07	0.1	Hdac4	8E-05	0.1	Exoc6b	2E-03	0.1	Tgfbr1	2E-01	0.1
Stip1	1E-06	0.1	Gan	3E-04	0.1	Thrap3	2E-05	0.1	Mrpl57	7E-04	0.1
Rpap2	3E-05	0.1	Gpbp1	9E-06	0.1	Cwf19l1	8E-01	0.1	Snupn	6E-01	0.1
Ftsj3	4E-05	0.1	Tmem69	1E-02	0.1	Yae1d1	3E-03	0.1	Eif5b	3E-05	0.1
Sccpdh	5E-04	0.1	Slc25a51	6E-04	0.1	Tyw3	8E-01	0.1	Cops7a	2E-02	0.1
Zdhhc20	7E-05	0.1	Usp7	3E-03	0.1	Xpnpep3	3E-01	0.1	Hbp1	3E-03	0.1
Asb1	2E-03	0.1	Dyrk1b	1E-03	0.1	Arfgef1	9E-04	0.1	Fbrs	4E-01	0.1
Itgav	4E-05	0.1	Llph	5E-06	0.1	Adgrl1	3E-01	0.1	Mrpl1	8E-02	0.1
Mapk1ip1l	4E-05	0.1	Nckap1	1E-05	0.1	Taf13	2E-01	0.1	Chchd6	3E-01	0.1
Pdia4	3E-10	0.1	Itga6	1E-03	0.1	Med16	9E-01	0.1	Htatsf1	2E-02	0.1
Ddx47	1E-03	0.1	Cds2	5E-02	0.1	Polr3d	6E-02	0.1	Mrps14	2E-06	0.1
Mme	1E-08	0.1	Rbbp4	8E-08	0.1	Cep290	5E-02	0.1	Cep170b	9E-01	0.1
Mcts1	1E-06	0.1	Atad2b	4E-04	0.1	Tmem129	7E-01	0.1	Serpine2	1E-04	0.1
Trabd	2E-03	0.1	Vcpip1	4E-04	0.1	Polr3gl	3E-01	0.1	Edrf1	3E-01	0.1
Celf1	7E-06	0.1	Rbm12b2	3E-03	0.1	Pcnp	1E-04	0.1	Pcdhb22	3E-01	0.1
Ube4a	9E-04	0.1	Usf2	3E-03	0.1	mt-Atp8	6E-03	0.1	Abcd3	7E-02	0.1
Ccdc181	3E-04	0.1	Rsf1	7E-06	0.1	Ubl3	3E-04	0.1	Usp34	3E-04	0.1
Acbd6	1E-05	0.1	Gbf1	2E-05	0.1	Ncoa2	6E-02	0.1	Sun1	8E-01	0.1
Bicd2	2E-05	0.1	Fbxl14	1E-02	0.1	Tor1a	1E-02	0.1	Atp6v1f	4E-08	0.1
Glrx5	5E-07	0.1	Wwtr1	1E-05	0.1	Zscan21	4E-01	0.1	Akt2	2E-01	0.1

Fibroblast					
Control			NICD2		
Gene	*p*	FC	Gene	*p*	FC

Fibroblast?					
Control			NICD2		
Gene	*p*	FC	Gene	*p*	FC
Tsfm	3E-10	0.3	Ppp2ca	2E-16	0.2
Xpo4	1E-10	0.3	Hdac4	2E-12	0.2
Tkfc	6E-08	0.3	Vma21	3E-12	0.2
Map2k3	1E-10	0.3	Ap1ar	4E-11	0.2
Nomo1	3E-08	0.3	Dolpp1	5E-08	0.2
Tmsb10	2E-22	0.3	Rpf1	5E-11	0.2
Dhx16	7E-09	0.3	Psmd2	3E-18	0.2
Ube2i	8E-19	0.3	Pip4k2a	2E-12	0.2
Rpgrip1l	6E-13	0.3	Mrpl20	1E-20	0.2
Hcfc1	1E-08	0.3	Shroom4	5E-16	0.2
Sf3b1	5E-15	0.3	Nrbp2	8E-06	0.2
Aars	5E-10	0.3	Nepro	2E-09	0.2
B4galt5	7E-07	0.3	Hsf2	9E-11	0.2
Slc7a1	2E-09	0.3	Brms1	3E-12	0.2
Zcchc3	1E-10	0.3	Tnmd	1E-05	0.2
Mitd1	3E-12	0.3	Adpgk	9E-14	0.2
Gss	3E-09	0.3	Clpb	5E-15	0.2
Adat1	8E-09	0.3	Usp25	4E-07	0.2
Dctn1	2E-07	0.3	Mar5	8E-18	0.2
Rprd1a	3E-08	0.3	Apex2	2E-12	0.2
1810009A15Rik	3E-12	0.3	Arl6	7E-11	0.2
9330136K24Rik	2E-05	0.3	Tmem41a	3E-13	0.2
Alg10b	9E-11	0.3	E4f1	1E-05	0.2
D330050I16Rik	1E-06	0.2	Dstn	6E-13	0.2
Eif4g1	9E-15	0.2	Exoc3l4	9E-08	0.2
Zbtb14	5E-07	0.2	Lzic	3E-11	0.2
Usp12	5E-11	0.2	Cdc42bpg	2E-11	0.2
Inhba	2E-06	0.2	Maob	3E-04	0.2
Cyb5b	4E-08	0.2	Rbm15b	4E-12	0.2
Nt5c3	8E-10	0.2	Myo18a	5E-15	0.2
Hic1	7E-07	0.2	Ccng2	5E-04	0.2
Akr1b8	3E-13	0.2	Atpif1	2E-33	0.2
Nudt13	3E-10	0.2	Phf20	3E-10	0.2
Atg4d	8E-06	0.2	Aars	6E-13	0.2
Nyap1	3E-06	0.2	Atp5f1	3E-29	0.2
Sirpa	3E-16	0.2	Gale	7E-11	0.2
Nfx1	1E-06	0.2	Gpc1	1E-15	0.2
Tsta3	2E-10	0.2	Caap1	2E-11	0.2
Atp2b1	1E-09	0.2	Smim12	2E-07	0.2
Vac14	5E-15	0.2	Abca8b	4E-07	0.2
Zfp612	1E-09	0.2	Cpsf6	4E-12	0.2
Golt1b	2E-09	0.2	Grtp1	3E-03	0.2
Slc16a1	4E-11	0.2	Wdr34	4E-13	0.2
Chtf8	4E-09	0.2	Qpctl	3E-13	0.2
Smg5	7E-09	0.2	Degs1	1E-13	0.2
Rbm28	7E-14	0.2	Ddx23	3E-11	0.2
Tcf7l2	1E-09	0.2	Rcbtb2	2E-13	0.2
Atp1b3	8E-20	0.2	Plxnd1	8E-14	0.2
Ttc38	8E-01	0.2	Smn1	4E-06	0.2
Ddit4l	1E-15	0.2	Camkk2	3E-14	0.2
Plekhm1	2E-02	0.2	Irs2	2E-11	0.2
Tyw5	2E-10	0.2	Ctdnep1	4E-10	0.2
Masp1	2E-12	0.2	Enpp1	4E-11	0.2
Pamr1	4E-09	0.2	Pgs1	4E-12	0.2
Fndc1	1E-08	0.2	Spsb3	2E-08	0.2
Tmem63a	1E-09	0.2	Dnajb11	3E-16	0.2
Zcchc10	4E-08	0.2	4933412E12Rik	6E-09	0.2
Zfp207	4E-09	0.2	Atxn2l	2E-11	0.2
Irak1bp1	2E-09	0.2	Atp5g1	1E-22	0.2
Rps27l	3E-27	0.2	Zfp763	1E-12	0.2
Abca7	4E-06	0.2	Tle4	5E-13	0.2
Cebpg	2E-10	0.2	Safb	3E-11	0.2
Spata6	2E-10	0.2	Shq1	7E-11	0.2
Gm5535	5E-06	0.2	Slc25a39	3E-12	0.2
Arhgap32	2E-10	0.2	Trp53i13	6E-11	0.2
U2surp	5E-14	0.2	Tmem138	5E-13	0.2
Synj2	1E-11	0.2	Slc35d2	3E-10	0.2
Dhdh	1E-06	0.2	Sypl	4E-10	0.2
Zfp13	7E-03	0.2	Timm17a	8E-12	0.2
Gm17066	1E-04	0.2	Wdr47	2E-12	0.2
Timm13	7E-20	0.2	Ppp1r18	6E-14	0.2
Mrps17	6E-16	0.2	Rbm28	1E-13	0.2
Ddx39b	1E-10	0.2	Wasf2	3E-19	0.2

Undefined						Articular/Synovial Fibroblast					
Control			NICD2			Control			NICD2		
Gene	*p*	FC	Gene	*p*	FC	Gene	*p*	FC	Gene	*p*	FC

Limb Mesenchyme					
Control			NICD2		
Gene	*p*	FC	Gene	*p*	FC
Terf2	2E-03	0.1	Fads2	9E-03	0.1
Rps6ka3	2E-06	0.1	Abcf1	2E-07	0.1
H2afy	6E-06	0.1	Gemin7	2E-06	0.1
Timm17a	3E-06	0.1	Arhgap35	7E-05	0.1
Mospd1	4E-03	0.1	P3h4	3E-06	0.1
Mbd6	4E-04	0.1	Rbm26	1E-05	0.1
P3h4	1E-05	0.1	Cstf2	3E-03	0.1
Ddx46	4E-05	0.1	Gps1	3E-04	0.1
Enoph1	7E-03	0.1	Qpctl	5E-03	0.1
Lnpk	1E-04	0.1	Med22	4E-03	0.1
Ptpn1	5E-05	0.1	Bcl10	2E-04	0.1
Hectd1	8E-06	0.1	Adat2	4E-01	0.1
Esco1	3E-04	0.1	Rnf8	9E-04	0.1
Adsl	9E-03	0.1	Dele1	7E-03	0.1
Slc12a7	1E-02	0.1	Afap1	2E-05	0.1
Faim	1E-04	0.1	Zc3h4	3E-04	0.1
Nelfa	1E-03	0.1	Gm27003	1E-01	0.1
Tmem87b	1E-03	0.1	Zmynd19	7E-03	0.1
Cfl2	8E-08	0.1	Abcb10	5E-03	0.1
Tstd2	4E-04	0.1	Npm1	5E-19	0.1
Lrch3	2E-05	0.1	Slc35b4	2E-02	0.1
Gosr2	9E-04	0.1	Tfg	2E-05	0.1
Wdr70	7E-04	0.1	Pigb	1E-03	0.1
Zfp956	4E-02	0.1	Tbc1d8b	5E-05	0.1
Epb41l2	5E-05	0.1	Scly	4E-02	0.1
Stambp	1E-03	0.1	Aasdhppt	1E-04	0.1
Ugp2	1E-10	0.1	Invs	2E-03	0.1
Pgghg	7E-05	0.1	Ring1	2E-05	0.1
Tubgcp6	1E-03	0.1	Ythdf2	2E-05	0.1
B3gnt2	2E-04	0.1	Smg7	8E-05	0.1
Nipal2	5E-02	0.1	Mogs	9E-03	0.1
Ing3	1E-04	0.1	Rell1	6E-03	0.1
Tpmt	8E-04	0.1	Zswim4	4E-03	0.1
Ppp2ca	4E-08	0.1	Zscan21	3E-03	0.1
Trip12	3E-06	0.1	Zpr1	9E-05	0.1
Apmap	1E-04	0.1	Dpysl2	1E-03	0.1
Invs	4E-05	0.1	Slc22a21	8E-03	0.1
Fam104a	5E-10	0.1	Ddx39b	2E-05	0.1
Zfp428	6E-04	0.1	Pym1	1E-02	0.1
Steap2	9E-05	0.1	Tmem167	4E-12	0.1
Nt5c	2E-07	0.1	Sap130	2E-04	0.1
Zfp383	1E-02	0.1	Pitpnb	4E-05	0.1
Mogs	8E-04	0.1	Kidins220	2E-04	0.1
Atp2a2	8E-07	0.1	Gm27241	2E-03	0.1
Arf6	6E-05	0.1	Rps6kb2	2E-02	0.1
Brd3	4E-05	0.1	Pld2	4E-01	0.1
Gm12353	9E-05	0.1	Ckap4	1E-09	0.1
Dis3	1E-04	0.1	Abl1	3E-04	0.1
Ranbp6	1E-03	0.1	Klf6	4E-04	0.1
Picalm	2E-07	0.1	Xpo7	3E-04	0.1
Zfp426	8E-05	0.1	Mvb12b	2E-05	0.1
Rnf168	8E-06	0.1	Igf2r	1E-03	0.1
Abat	7E-05	0.1	Aplp1	2E-03	0.1
Cacna1g	4E-04	0.1	Srpx	5E-02	0.1
Mylip	2E-04	0.1	Wbp11	2E-04	0.1
Efcab14	8E-04	0.1	Alkbh3	3E-05	0.1
Snx8	9E-05	0.1	Tmc6	5E-03	0.1
Sox5	5E-17	0.1	Actr2	1E-03	0.1
Magt1	3E-06	0.1	Leng8	6E-03	0.1
Arid1a	2E-04	0.1	Taco1os	2E-02	0.1
Atf5	3E-10	0.1	Selenot	3E-04	0.1
Dph5	2E-03	0.1	Cryzl2	4E-03	0.1
Wapl	1E-06	0.1	Pdpk1	2E-04	0.1
Srsf3	1E-08	0.1	Rabgef1	4E-03	0.1
Nudcd3	1E-05	0.1	Phf2	1E-03	0.1
Proser1	2E-05	0.1	Klhl12	1E-03	0.1
Manea	2E-04	0.1	Wwc2	2E-05	0.1
Gm15764	1E-01	0.1	Smcr8	1E-01	0.1
5530601H04Rik	8E-04	0.1	1600010M07Rik	1E-02	0.1
Mtg2	2E-03	0.1	Arhgap39	3E-03	0.1
Zfp143	5E-04	0.1	Megf8	3E-03	0.1
Vps54	3E-04	0.1	Ddb1	2E-06	0.1
Nop16	8E-04	0.1	Hdhd2	2E-04	0.1

Chondrogenic					
Control			NICD2		
Gene	*p*	FC	Gene	*p*	FC
Zcrb1	2E-07	0.1	Map3k20	3E-03	0.1
Babam2	3E-03	0.1	Dag1	5E-05	0.1
Sin3a	2E-01	0.1	Cox17	4E-05	0.1
Ublcp1	4E-04	0.1	Pbrm1	4E-04	0.1
Ubap1	2E-01	0.1	Hs6st1	4E-01	0.1
mt-Nd4l	2E-09	0.1	Bnip3l	9E-05	0.1
Diablo	5E-01	0.1	Ndufaf6	1E+00	0.1
Pds5b	8E-04	0.1	Nsmaf	4E-01	0.1
Rras2	6E-03	0.1	Tmem33	1E-02	0.1
mt-Cytb	2E-25	0.1	Slc49a4	4E-01	0.1
Cds2	4E-01	0.1	Ndufa6	1E-07	0.1
C2cd5	3E-02	0.1	Dcun1d1	6E-02	0.1
Sirt1	1E+00	0.1	Tsg101	8E-04	0.1
Isoc2b	3E-01	0.1	Tor1aip1	2E-03	0.1
Nudt19	1E-01	0.1	Rsrc2	2E-03	0.1
Pfkl	2E-02	0.1	Mrc2	4E-02	0.1
Smarcd1	1E-02	0.1	Lsg1	2E-01	0.1
Snx11	9E-01	0.1	Mrps31	7E-02	0.1
Rab10os	3E-01	0.1	Jup	6E-01	0.1
Pbxip1	3E-03	0.1	Mgrn1	9E-01	0.1
Glo1	3E-05	0.1	Qtrt2	7E-01	0.1
Hibch	8E-02	0.1	Lonp2	3E-02	0.1
Zgpat	4E-01	0.1	Kdm1b	8E-01	0.1
Pcsk7	8E-02	0.1	Fancl	8E-01	0.1
Sltm	7E-03	0.1	Rnf11	5E-04	0.1
Ash1l	3E-05	0.1	Zfp729a	7E-01	0.1
Cand1	2E-02	0.1	Slc31a1	4E-01	0.1
Htatsf1	6E-03	0.1	Rnf146	2E-01	0.1
Timm22	2E-03	0.1	Tapbp	1E-01	0.1
Ppil4	4E-02	0.1	Zfp799	8E-01	0.1
Dhx36	3E-05	0.1	Taf6	2E-01	0.1
Tifa	6E-01	0.1	Exosc3	4E-01	0.1
Atp6v1d	5E-04	0.1	Zfp329	1E-01	0.1
Cdc26	3E-05	0.1	Mrtfb	9E-02	0.1
Rab3a	5E-01	0.1	Dennd5a	2E-01	0.1
Zdhhc17	1E-01	0.1	Zbtb17	9E-01	0.1
Ccdc8	7E-01	0.1	Selenof	1E-07	0.1
Spcs3	6E-02	0.1	Eno1	5E-04	0.1
Ensa	3E-03	0.1	Abcf3	4E-01	0.1
Dcp1a	2E-01	0.1	Nr1d1	9E-01	0.1
Dnajc10	9E-03	0.1	Pitpna	1E-03	0.1
Prpf40b	2E-01	0.1	C9orf72	6E-01	0.1
Rbm45	3E-01	0.1	Elk3	2E-01	0.1
Rcn2	1E-04	0.1	Epg5	9E-01	0.1
Nab1	2E-01	0.1	Pkia	8E-01	0.1
Zdhhc18	9E-01	0.1	Acox1	6E-03	0.1
Snhg20	7E-01	0.1	Zfp260	6E-03	0.1
Fam3a	3E-01	0.1	Cbx8	1E+00	0.1
Tmem59	3E-09	0.1	N6amt1	8E-02	0.1
Trmt11	5E-01	0.1	Mfap3	6E-01	0.1
Eif5b	5E-07	0.1	Snrnp27	2E-04	0.1
Pcolce	6E-09	0.1	Tmem251	5E-01	0.1
Slc52a2	8E-01	0.1	Gmeb2	4E-01	0.1
Cops4	3E-04	0.1	Cisd1	9E-06	0.1
Dctd	5E-01	0.1	Btbd1	3E-03	0.1
Rmdn3	6E-01	0.1	Hspb6	3E-02	0.1
Sos2	2E-02	0.1	Zbtb45	8E-01	0.1
Cdk5rap3	2E-03	0.1	Eefsec	9E-01	0.1
Cfap410	5E-01	0.1	Mtfr1l	2E-01	0.1
Fnbp1l	8E-02	0.1	Ubap2	1E-03	0.1
Nol10	8E-01	0.1	Nelfb	4E-01	0.1
Qrsl1	9E-01	0.1	Ubac2	2E-01	0.1
Camk2g	7E-02	0.1	Rnf217	4E-01	0.1
Wdr70	1E-03	0.1	Dennd2c	7E-01	0.1
Zmat2	1E-05	0.1	Sacm1l	1E-02	0.1
Mfap1a	1E-01	0.1	Zfp14	7E-01	0.1
Ubqln4	1E+00	0.1	Dnajc1	6E-05	0.1
Tor1aip1	2E-03	0.1	Cisd2	2E-03	0.1
Ankrd54	9E-01	0.1	Far1os	6E-01	0.1
Pnpla8	3E-04	0.1	Chd1	1E-01	0.1
Ptp4a2	2E-06	0.1	Vkorc1	2E-05	0.1
Cfap298	2E-01	0.1	Pdcd6	9E-04	0.1
Rab2a	7E-11	0.1	Gm14410	7E-01	0.1

Fibroblast					
Control			NICD2		
Gene	*p*	FC	Gene	*p*	FC

Fibroblast?					
Control			NICD2		
Gene	*p*	FC	Gene	*p*	FC
Prmt1	1E-06	0.2	D3Ertd751e	2E-14	0.2
Prkrip1	2E-10	0.2	Slc4a7	8E-15	0.2
Itpr1	3E-08	0.2	Eif4ebp1	2E-10	0.2
Asns	2E-09	0.2	Trnau1ap	9E-13	0.2
Hspa13	9E-12	0.2	Sirpa	3E-21	0.2
Uchl1	5E-33	0.2	Pir	6E-09	0.2
Pmpca	4E-10	0.2	Igf2r	3E-09	0.2
Uqcr10	2E-39	0.2	8030451A03Rik	6E-04	0.2
Fam219b	9E-10	0.2	Uxt	7E-11	0.2
Cbr3	2E-07	0.2	Cldnd1	1E-10	0.2
Rbbp5	2E-10	0.2	Atg4d	2E-08	0.2
Rerg	1E-07	0.2	Slc35b4	9E-15	0.2
Rev1	3E-07	0.2	Rpain	4E-16	0.2
Pym1	2E-08	0.2	Phc1	1E-12	0.2
Acat2	5E-15	0.2	Rasa3	2E-15	0.2
Ada	5E-13	0.2	Ncbp3	7E-08	0.2
Fbxl14	2E-11	0.2	Gan	2E-13	0.2
Pkig	1E-08	0.2	Celf4	2E-09	0.2
4930444A19Rik	3E-08	0.2	Arpc4	6E-12	0.2
Usp5	1E-04	0.2	Mpg	3E-11	0.2
Mmd	6E-08	0.2	BC005537	1E-12	0.2
Phtf1	2E-07	0.2	Smpdl3b	1E-13	0.2
Hdac2	1E-11	0.2	Fat1	7E-15	0.2
Sgpp1	2E-07	0.2	Dcaf7	1E-10	0.2
Gchfr	2E-11	0.2	Tent2	3E-09	0.2
Mob2	4E-08	0.2	Parp9	2E-09	0.2
Prkd3	2E-11	0.2	Fem1b	5E-11	0.2
Dnajc21	2E-06	0.2	Hs6st2	1E-25	0.2
Rpia	2E-08	0.2	Kat2a	3E-06	0.2
Btbd10	3E-09	0.2	Agrp	7E-14	0.2
Ndufv1	3E-12	0.2	Thnsl1	2E-06	0.2
Gtf2f1	3E-08	0.2	Alg10b	1E-10	0.2
Plk2	4E-07	0.2	Pygo2	1E-07	0.2
Snap29	3E-05	0.2	Srsf9	2E-11	0.2
Tspan7	3E-05	0.2	Kazn	3E-11	0.2
BC023719	6E-05	0.2	Matr3	1E-14	0.2
Atl3	1E-07	0.2	Psmf1	5E-12	0.2
Ywhaz	6E-14	0.2	Ccdc22	9E-10	0.2
Chchd5	2E-08	0.2	Capza2	7E-19	0.2
Setd3	9E-10	0.2	Tmtc3	1E-11	0.2
Kctd5	1E-07	0.2	Ptpdc1	2E-11	0.2
Slc25a14	1E-11	0.2	Chchd5	3E-11	0.2
Zranb2	4E-12	0.2	Ldlrad4	4E-08	0.2
Lrig1	7E-10	0.2	Hspa13	4E-11	0.2
Miga2	9E-10	0.2	Ptar1	9E-14	0.2
Mar5	4E-12	0.2	Hpgds	5E-16	0.2
Pnpt1	9E-12	0.2	Sbf1	1E-10	0.2
B9d2	4E-09	0.2	Ampd2	3E-08	0.2
Sesn2	8E-05	0.2	Pak2	5E-14	0.2
Tbc1d8b	4E-10	0.2	Git2	4E-10	0.2
Tmem165	6E-13	0.2	Adam12	2E-24	0.2
Actr6	9E-11	0.2	Gde1	5E-13	0.2
Msl3l2	6E-09	0.2	Epb41l4b	3E-06	0.2
Cdc42bpg	1E-07	0.2	Rrp8	2E-08	0.2
Bnip2	1E-14	0.2	Ano6	1E-12	0.2
Cfap298	1E-10	0.2	Triap1	3E-11	0.2
Prpf18	8E-09	0.2	Icmt	1E-11	0.2
Xiap	1E-05	0.2	Bag4	1E-14	0.2
Ss18	9E-09	0.2	Nkapd1	3E-10	0.2
Rbpms	4E-16	0.2	Polr2h	8E-08	0.2
Slc25a42	1E-07	0.2	Stoml2	4E-12	0.2
9130401M01Rik	8E-06	0.2	Sord	2E-14	0.2
Zfp764	4E-05	0.2	Polrmt	1E-05	0.2
Loxl3	3E-10	0.2	Prkar2b	7E-24	0.2
Slc1a5	2E-11	0.2	Stag1	4E-14	0.2
Nr4a2	9E-13	0.2	Ap2a2	2E-10	0.2
Supt20	2E-05	0.2	Tbl3	3E-12	0.2
Hsf4	5E-06	0.2	Zfp326	3E-10	0.2
Rock1	2E-14	0.2	Dzip3	4E-11	0.2
Fam133b	7E-09	0.2	Ube2i	1E-22	0.2
Dhrs1	6E-05	0.2	Ywhae	1E-30	0.2
Drg1	1E-10	0.2	Traf2	2E-09	0.2
4930484I04Rik	1E-07	0.2	Slc7a3	3E-07	0.2

Undefined					
Control			NICD2		
Gene	*p*	FC	Gene	*p*	FC

Articular/Synovial Fibroblast					
Control			NICD2		
Gene	*p*	FC	Gene	*p*	FC

Limb Mesenchyme					
Control			NICD2		
Gene	*p*	FC	Gene	*p*	FC
2310011J03Rik	1E-04	0.1	Ankrd17	3E-04	0.1
Ttc17	3E-03	0.1	Pla2g12a	5E-03	0.1
Hexdc	2E-03	0.1	Hnrnpf	2E-09	0.1
Zfand3	3E-06	0.1	Fgfr1op2	4E-06	0.1
Csnk1g2	2E-05	0.1	Rlim	4E-04	0.1
Pkn2	2E-06	0.1	Fbxl4	3E-03	0.1
Txn2	1E-07	0.1	Zfp740	3E-05	0.1
Minpp1	8E-04	0.1	Agpat5	7E-04	0.1
Ash2l	2E-05	0.1	Adprhl2	6E-04	0.1
Gm15417	3E-05	0.1	Rnf220	5E-04	0.1
Tnpo2	4E-04	0.1	Card19	1E-04	0.1
Pfdn1	1E-11	0.1	Wdr26	4E-05	0.1
Zfp397	3E-04	0.1	Stat5a	4E-03	0.1
Ctif	4E-04	0.1	Serinc3	3E-04	0.1
Csnk2a2	3E-05	0.1	Spopl	3E-04	0.1
Gle1	2E-04	0.1	Polr3k	7E-05	0.1
BC017158	5E-03	0.1	Vps72	2E-04	0.1
Rab3gap1	1E-04	0.1	Naa25	4E-04	0.1
Atp7a	1E-02	0.1	Rbm41	8E-04	0.1
Paxx	2E-05	0.1	Nol9	2E-04	0.1
Cstf2t	9E-04	0.1	Hras	9E-07	0.1
Tbcd	4E-04	0.1	Gng5	8E-17	0.1
Anks6	7E-03	0.1	Tmem63b	1E-03	0.1
Gm16286	7E-06	0.1	Dusp19	2E-03	0.1
Ptp4a2	2E-09	0.1	Dctn4	1E-04	0.1
Rbm27	9E-04	0.1	Itfg1	3E-04	0.1
Camk2g	3E-05	0.1	Zfp426	3E-03	0.1
Ggact	4E-03	0.1	Zfp263	1E-02	0.1
Susd6	8E-05	0.1	Zcchc8	2E-03	0.1
Rnf187	1E-05	0.1	Zswim9	3E-02	0.1
Cmtm7	7E-06	0.1	Grk2	3E-03	0.1
Plpp5	3E-05	0.1	Uba1	1E-04	0.1
Itm2c	6E-07	0.1	Ubr3	2E-04	0.1
Psmd12	1E-08	0.1	Fam89b	4E-05	0.1
Syt11	9E-05	0.1	Psmd6	2E-06	0.1
D630045J12Rik	3E-05	0.1	Nrbp2	2E-02	0.1
Prpf39	3E-04	0.1	Setdb1	8E-05	0.1
Rassf7	3E-02	0.1	Mllt1	6E-05	0.1
Hnrnpa3	7E-11	0.1	Ergic2	3E-05	0.1
Dop1b	1E-05	0.1	Usp4	2E-03	0.1
Ktn1	3E-06	0.1	Zfp868	2E-02	0.1
Ltn1	2E-03	0.1	Abraxas1	2E-02	0.1
Dusp12	3E-05	0.1	Amn1	3E-03	0.1
Pfkm	2E-04	0.1	Zfp442	4E-02	0.1
Tep1	2E-04	0.1	Tspan31	2E-05	0.1
Cltc	3E-08	0.1	Mob3a	8E-04	0.1
9130008F23Rik	2E-02	0.1	Eps8	1E-02	0.1
Bcl2l1	5E-05	0.1	Etf1	2E-06	0.1
Caap1	4E-04	0.1	Dnajc15	2E-06	0.1
Plekha1	2E-03	0.1	Ccnt2	8E-04	0.1
Usf1	2E-03	0.1	Ocrl	5E-04	0.1
Rnf111	6E-05	0.1	Crot	1E-03	0.1
Arpc2	5E-12	0.1	Jrk	4E-03	0.1
Armcx5	2E-03	0.1	Fbxl19	5E-02	0.1
Canx	6E-11	0.1	Rfx5	3E-03	0.1
Rcor3	4E-04	0.1	Slc38a7	2E-02	0.1
Trim44	8E-05	0.1	Map3k3	9E-04	0.1
Tafa5	1E-13	0.1	G3bp1	7E-06	0.1
Tmem42	4E-06	0.1	AW146154	3E-02	0.1
Frzb	3E-03	0.1	Mtfmt	7E-03	0.1
Nme1	1E-10	0.1	Ube2z	1E-03	0.1
Gtf2e2	2E-05	0.1	Zfp598	5E-03	0.1
Coil	9E-04	0.1	Mthfd2l	3E-03	0.1
A230072C01Rik	2E-02	0.1	9930021J03Rik	2E-03	0.1
Mrpl10	5E-04	0.1	Rbm4b	2E-04	0.1
Alkbh2	2E-03	0.1	Yipf4	1E-04	0.1
Tmco3	3E-05	0.1	Chchd1	2E-06	0.1
Ankrd49	3E-04	0.1	Zc3h7a	1E-03	0.1
Mff	4E-06	0.1	Slc35a1	1E-03	0.1
Nkap	9E-05	0.1	Eif4ebp3	7E-02	0.1
Zfp62	3E-04	0.1	Foxn2	3E-02	0.1
Pus3	3E-03	0.1	Ep300	5E-04	0.1
Gatad2b	2E-05	0.1	Ccdc126	2E-03	0.1

Chondrogenic					
Control			NICD2		
Gene	*p*	FC	Gene	*p*	FC
Gbe1	5E-01	0.1	Trmt13	7E-01	0.1
Pus7	8E-01	0.1	Chd4	4E-04	0.1
Smg6	4E-02	0.1	Phpt1	2E-01	0.1
Stard3	4E-01	0.1	Etnk1	2E-03	0.1
Tbl1x	1E-03	0.1	Ndufaf8	3E-03	0.1
AI506816	1E-04	0.1	Zkscan5	9E-01	0.1
Tmem138	7E-01	0.1	Haghl	4E-01	0.1
Gnptg	1E-02	0.1	Tmem175	2E-01	0.1
Sos1	1E-01	0.1	Sos2	5E-01	0.1
Tox4	2E-01	0.1	Ddit3	9E-01	0.1
Rnf123	5E-01	0.1	Nono	6E-05	0.1
Supt3	2E-01	0.1	Phf6	3E-01	0.1
Setd5	5E-05	0.1	Pdpr	9E-01	0.1
Ost4	2E-09	0.1	Rdh14	2E-01	0.1
Sav1	4E-01	0.1	Osbp	2E-01	0.1
Dkk3	1E+00	0.1	Rc3h2	1E-02	0.1
Dkc1	7E-02	0.1	Tmem248	4E-02	0.1
Pip4k2c	2E-01	0.1	Map3k10	3E-01	0.1
Uqcc3	6E-05	0.1	Men1	7E-01	0.1
Fcf1	6E-03	0.1	Fam204a	4E-02	0.1
Mterf2	4E-01	0.1	Rbm22	2E-02	0.1
H3f3a	4E-15	0.1	Usp33	2E-01	0.1
Scamp1	1E-01	0.1	Nt5c2	2E-01	0.1
Slc19a1	6E-01	0.1	Sub1	4E-06	0.1
Yme1l1	4E-04	0.1	Cdkal1	9E-03	0.1
Ing3	9E-01	0.1	Flrt3	5E-01	0.1
Lars	8E-03	0.1	4632427E13Rik	4E-02	0.1
Trp53cor1	2E-01	0.1	Prmt5	9E-02	0.1
Ppp2r3c	5E-01	0.1	Tmx4	1E-01	0.1
Carf	1E+00	0.1	Zfp68	2E-01	0.1
Zfp111	5E-01	0.1	Gm17018	6E-01	0.1
Clk4	3E-02	0.1	Mapk8ip3	3E-01	0.1
Zfp950	4E-03	0.1	CAAA01118383.1	1E-01	0.1
Ppme1	7E-02	0.1	Dus3l	9E-01	0.1
Brox	3E-01	0.1	Usf2	8E-02	0.1
Ttbk2	9E-01	0.1	Zbtb8os	6E-02	0.1
Mark2	4E-02	0.1	Xpc	9E-01	0.1
Ubl5	4E-10	0.1	Gmeb1	7E-01	0.1
Irf2bp2	3E-02	0.1	Dido1	4E-01	0.1
Sacm1l	6E-03	0.1	Rpp38	7E-01	0.1
Luc7l2	1E-07	0.1	Lss	1E+00	0.1
Simc1	2E-01	0.1	Lrrfip2	3E-02	0.1
Magoh	2E-03	0.1	Golga1	2E-01	0.1
Smim8	2E-02	0.1	Jdp2	4E-01	0.1
Vps26c	1E-01	0.1	Afg1l	8E-01	0.1
Wapl	1E-03	0.1	Tm9sf3	4E-06	0.1
2810433D01Rik	2E-01	0.1	Helz	1E-01	0.1
Rftn2	9E-02	0.1	Ahr	4E-01	0.1
Qtrt2	6E-01	0.1	Macf1	4E-03	0.1
Alg6	6E-01	0.1	Scaf8	3E-02	0.1
Ndufs5	3E-10	0.1	Pum2	2E-01	0.1
Mier3	7E-01	0.1	Fxr2	3E-01	0.1
Prkdc	1E-01	0.1	Hnrnph2	6E-02	0.1
Mrps31	2E-01	0.1	Slc30a5	3E-01	0.1
Srcap	5E-02	0.1	Fuca1	3E-04	0.1
Cstf2	9E-01	0.1	Def8	9E-01	0.1
Sat2	1E-01	0.1	Med16	7E-01	0.1
Sdsl	1E+00	0.1	Gpatch2	3E-01	0.1
Actl6a	4E-02	0.1	Brd7	1E-02	0.1
Rhebl1	6E-01	0.1	Tm2d1	5E-03	0.1
Ptrhd1	5E-02	0.1	Snrpb2	7E-02	0.1
Parn	7E-02	0.1	Polr3f	8E-01	0.1
Pus10	5E-01	0.1	Dpy30	2E-02	0.1
Copb2	2E-07	0.1	Ryk	3E-04	0.1
Rbl2	9E-01	0.1	Qtrt1	9E-01	0.1
Tspyl2	2E-01	0.1	Cul9	4E-01	0.1
Xpr1	2E-01	0.1	Snhg20	9E-01	0.1
Dvl1	6E-01	0.1	Fbxl15	7E-01	0.1
1110004F10Rik	1E-05	0.1	Mau2	7E-01	0.1
Otud5	3E-01	0.1	Babam1	2E-01	0.1
Tatdn1	8E-02	0.1	Igf2bp2	2E-02	0.1
Max	1E-02	0.1	Glmn	2E-01	0.1
Tmem203	4E-01	0.1	Snap47	3E-03	0.1

Fibroblast					
Control			NICD2		
Gene	*p*	FC	Gene	*p*	FC

Fibroblast?					
Control			NICD2		
Gene	*p*	FC	Gene	*p*	FC
Med22	4E-09	0.2	Adprh	7E-08	0.2
Rnf5	1E-08	0.2	Tmco4	1E-11	0.2
Ppp6c	5E-06	0.2	Pak1ip1	6E-09	0.2
Clns1a	1E-09	0.2	Hs6st3	1E-07	0.2
Uba6	1E-12	0.2	Zfp248	1E-02	0.2
Rbm22	7E-06	0.2	Nae1	6E-10	0.2
Tysnd1	4E-11	0.2	Polr2a	2E-11	0.2
Htr2a	1E-05	0.2	Polr2b	5E-09	0.2
Filip1l	5E-08	0.2	Pelo	9E-07	0.2
Gde1	3E-11	0.2	Cep131	7E-07	0.2
Prmt7	2E-10	0.2	Nsf	1E-10	0.2
Uqcrq	1E-25	0.2	Deaf1	5E-09	0.2
Psmc3	3E-14	0.2	Drg1	8E-13	0.2
Sept7	3E-21	0.2	Snx5	7E-11	0.2
Abca3	5E-07	0.2	Cmtm4	4E-13	0.2
Qpct	6E-07	0.2	Plk2	8E-11	0.2
Strn	1E-10	0.2	Sdhd	2E-12	0.2
Apmap	2E-10	0.2	Reep5	2E-23	0.2
Mfsd11	2E-11	0.2	H3f3a	5E-33	0.2
Tmco4	6E-08	0.2	Gna13	3E-13	0.2
Foxred1	5E-09	0.2	Stag2	3E-11	0.2
Lsm12	5E-12	0.2	Gpc4	2E-12	0.2
Entpd6	5E-09	0.2	Numa1	1E-11	0.2
Ube2z	2E-11	0.2	Oxsr1	2E-13	0.2
Klhl18	7E-03	0.2	Cobl	2E-15	0.2
Slc35d2	2E-08	0.2	Mgat5	8E-11	0.2
Kctd10	3E-10	0.2	Dab2ip	3E-10	0.2
Rhod	4E-08	0.2	U2surp	1E-15	0.2
Mapk9	4E-07	0.2	Slc35e4	1E-12	0.2
Elavl1	1E-09	0.2	Anp32a	2E-10	0.2
Abcb7	1E-14	0.2	Rrs1	4E-09	0.2
Klhl2	1E-07	0.2	Ccdc127	1E-11	0.2
Clmn	1E-05	0.2	B230307C23Rik	5E-10	0.2
Men1	4E-09	0.2	Unk	8E-14	0.2
Cwf19l2	4E-06	0.2	Cisd3	9E-11	0.2
Wdr18	2E-08	0.2	Pdlim5	8E-11	0.2
Pias3	2E-09	0.2	Csf2ra	2E-03	0.2
Chka	4E-09	0.2	Fyn	3E-12	0.2
Kctd12b	5E-06	0.2	2900052L18Rik	9E-09	0.2
Axin2	4E-05	0.2	Mical1	2E-12	0.2
Zswim7	4E-09	0.2	Hpcal1	8E-14	0.2
Bcor	2E-11	0.2	St3gal5	1E-16	0.2
Erc2	1E-08	0.2	Kcnab1	9E-13	0.2
Ppp1r37	8E-06	0.2	Siah2	7E-11	0.2
Knop1	5E-06	0.2	Phf20l1	3E-11	0.2
Ddx46	6E-11	0.2	Mif4gd	2E-13	0.2
Rwdd4a	7E-10	0.2	Trak2	8E-14	0.2
Zfp954	2E-06	0.2	Ncln	2E-15	0.2
Daglb	1E-10	0.2	Tmem165	6E-15	0.2
Taf10	8E-19	0.2	Mrfap1	4E-36	0.2
Ilk	3E-08	0.2	Pacs1	4E-11	0.2
Utp11	2E-09	0.2	Isy1	4E-14	0.2
Sgms1	3E-07	0.2	Ccdc12	2E-06	0.2
Diablo	1E-10	0.2	Pop7	1E-10	0.2
Zcchc17	6E-08	0.2	Oaf	2E-03	0.2
Zfp938	9E-06	0.2	Nrf1	3E-11	0.2
Srgap2	1E-09	0.2	Pym1	3E-12	0.2
Tuba1a	2E-17	0.2	Imp4	7E-12	0.2
Hnrnpk	2E-20	0.2	Irf7	1E-02	0.2
Nup98	8E-08	0.2	Bicd1	2E-07	0.2
Emc1	4E-08	0.2	Tns2	6E-13	0.2
Sh2d3c	8E-05	0.2	Commd2	1E-08	0.2
Hmg20b	6E-13	0.2	Phip	1E-13	0.2
Rbm8a	1E-08	0.2	Cmtr1	1E-12	0.2
Psmd3	3E-08	0.2	Igf1r	5E-12	0.2
Aqr	8E-10	0.2	S1pr3	5E-09	0.2
Ssr1	9E-15	0.2	Arfgap2	1E-06	0.2
Cldnd1	3E-09	0.2	Junos	2E-14	0.2
2410004B18Rik	3E-11	0.2	Fam204a	1E-10	0.2
Elp1	4E-08	0.2	Spry1	2E-08	0.2
Msrb1	8E-02	0.2	Exoc8	4E-11	0.2
Arl13b	9E-09	0.2	Gpsm2	3E-11	0.2
Arpc2	3E-23	0.2	Six4	1E-08	0.2

Undefined					
Control			NICD2		
Gene	*p*	FC	Gene	*p*	FC

Articular/Synovial Fibroblast					
Control			NICD2		
Gene	*p*	FC	Gene	*p*	FC

Limb Mesenchyme

Control			NICD2		
Gene	*p*	FC	Gene	*p*	FC
Tbc1d20	2E-04	0.1	Ttc17	2E-04	0.1
Pcgf2	2E-03	0.1	Dclre1c	1E-02	0.1
Rab3a	8E-05	0.1	Grk6	4E-03	0.1
Dnal4	4E-05	0.1	Ttc28	5E-07	0.1
Rita1	1E-04	0.1	Lrch4	2E-03	0.1
Aco2	4E-07	0.1	Commd9	2E-03	0.1
Zc3h15	1E-09	0.1	Scd2	1E-03	0.1
Carmil1	6E-07	0.1	Ubl3	4E-05	0.1
Arhgap1	2E-04	0.1	Unc50	2E-05	0.1
Col4a5	2E-03	0.1	B3galt6	3E-03	0.1
Pigf	1E-05	0.1	Trip12	7E-05	0.1
Znhit2	2E-03	0.1	Ccdc71	1E-05	0.1
Acbd5	5E-04	0.1	Cdkn2aip	5E-04	0.1
Pan2	4E-02	0.1	Trim26	4E-04	0.1
Mef2b	2E-03	0.1	Krr1	1E-04	0.1
Mecr	6E-04	0.1	Clock	1E-03	0.1
Med19	2E-04	0.1	Srgap3	2E-07	0.1
Dmtf1	2E-04	0.1	Tmem218	2E-04	0.1
B3gnt9	2E-04	0.1	Med30	6E-04	0.1
Sf1	2E-05	0.1	Kansl1	5E-06	0.1
Lyrm4	7E-04	0.1	Ptx3	7E-07	0.1
Gpr19	5E-02	0.1	Mrpl44	2E-02	0.1
Nsd1	6E-09	0.1	Mmaa	9E-04	0.1
Epn1	8E-04	0.1	Taf2	6E-04	0.1
Dop1a	5E-05	0.1	Ddx59	4E-02	0.1
Eef2kmt	5E-04	0.1	Triap1	2E-04	0.1
Ago2	7E-04	0.1	Ppp4r2	6E-06	0.1
Tmem259	5E-04	0.1	Srsf6	2E-06	0.1
Cdr2l	4E-03	0.1	Cept1	2E-02	0.1
Hnrnpa1	1E-08	0.1	Lactb2	2E-03	0.1
Asphd2	4E-03	0.1	Traf3	7E-04	0.1
Wipf1	6E-04	0.1	Smg9	8E-03	0.1
5730480H06Rik	1E-03	0.1	Creb1	3E-03	0.1
Maged1	3E-08	0.1	Slc25a11	1E-04	0.1
Faf2	5E-05	0.1	Avl9	7E-03	0.1
Rnf11	2E-06	0.1	Ddx6	1E-06	0.1
1810026B05Rik	8E-05	0.1	Tacc2	4E-04	0.1
Cdpf1	1E-04	0.1	Sf3a3	3E-04	0.1
Tfam	3E-04	0.1	Pdcd7	6E-04	0.1
Mtif3	1E-03	0.1	Kif3b	3E-04	0.1
Bcl2	1E-02	0.1	Ppp2r3c	2E-04	0.1
Golga4	2E-04	0.1	Csnk1e	3E-04	0.1
Ccni	4E-06	0.1	Adcy3	6E-05	0.1
Cactin	5E-03	0.1	Cdc26	1E-06	0.1
Klhdc2	4E-06	0.1	Tmem154	1E-01	0.1
Snapc3	5E-05	0.1	Prpf19	2E-04	0.1
Mob4	1E-04	0.1	Ankrd10	8E-04	0.1
Mxd4	2E-08	0.1	Zfp24	4E-03	0.1
Zfp729b	2E-03	0.1	Cops9	1E-07	0.1
Tbp	5E-04	0.1	Slc25a16	6E-03	0.1
Slc11a2	7E-04	0.1	Smg8	7E-02	0.1
Pdk1	2E-04	0.1	Dpp3	4E-04	0.1
Ing1	2E-04	0.1	Ube2q2	3E-05	0.1
Pigb	6E-03	0.1	Klhl20	1E-03	0.1
C130021I20Rik	5E-03	0.1	Slc39a10	2E-03	0.1
2610044O15Rik8	8E-04	0.1	Ggnbp1	1E-02	0.1
Ggnbp1	4E-02	0.1	Pibf1	1E-04	0.1
Cdkl3	8E-04	0.1	Mr1	2E-01	0.1
Nup188	2E-05	0.1	Timm22	6E-04	0.1
Pdhx	1E-03	0.1	Mpp5	2E-03	0.1
Thoc1	2E-04	0.1	Zfp35	4E-02	0.1
Sept11	8E-09	0.1	Cd99l2	3E-02	0.1
Urb2	3E-03	0.1	Pdia6	7E-10	0.1
Epb41l4aos	2E-04	0.1	Slc52a2	8E-03	0.1
Riok1	1E-04	0.1	Cdyl	6E-05	0.1
Brox	6E-05	0.1	Zbtb12	9E-04	0.1
Maip1	1E-03	0.1	Gm15411	5E-02	0.1
Mettl16	5E-04	0.1	Tomm34	2E-03	0.1
Smim15	7E-05	0.1	Jmjd1c	2E-04	0.1
Rbm10	1E-04	0.1	Cfl2	1E-07	0.1
Stx18	9E-05	0.1	Atg13	7E-03	0.1
Trmt61a	4E-03	0.1	Mef2b	3E-03	0.1
Brsk1	2E-02	0.1	Tra2a	1E-04	0.1

Chondrogenic

Control			NICD2		
Gene	*p*	FC	Gene	*p*	FC
Gid8	6E-02	0.1	Disp1	7E-01	0.1
Opa3	3E-01	0.1	Ttc14	2E-01	0.1
Mmp28	9E-01	0.1	Stag2	8E-02	0.1
Senp2	1E-01	0.1	Cops4	6E-03	0.1
Aqr	4E-02	0.1	Anxa7	9E-03	0.1
Epg5	6E-01	0.1	Tanc2	8E-03	0.1
Cse1l	1E-01	0.1	Slc25a3	1E-07	0.1
Rcl1	3E-01	0.1	Cbx5	2E-05	0.1
Slc25a36	1E-02	0.1	Matr3	1E-04	0.1
Usp38	7E-01	0.1	Zcchc7	2E-03	0.1
Golga4	9E-03	0.1	Sec61b	3E-06	0.1
Tlk2	9E-03	0.1	Zup1	6E-01	0.1
Atat1	1E-01	0.1	Gpsm2	3E-02	0.1
Acp2	4E-01	0.1	Zkscan7	5E-01	0.1
Uhrf1bp1	6E-01	0.1	Dnajc10	7E-04	0.1
Cox6a2	5E-01	0.1	Kbtbd3	6E-01	0.1
Smim7	9E-03	0.1	Mier1	9E-03	0.1
Asb13	1E+00	0.1	Clstn1	3E-03	0.1
Coq10a	9E-01	0.1	Xrn1	6E-02	0.1
Mtor	3E-01	0.1	Btbd9	9E-02	0.1
Lrrc47	6E-01	0.1	Cabin1	1E-01	0.1
Taf15	3E-03	0.1	Ndufb3	2E-04	0.1
Nceh1	2E-01	0.1	Hmbox1	4E-01	0.1
Cerk	4E-01	0.1	Smim20	3E-01	0.1
Tmcc1	1E-01	0.1	Ttc19	9E-02	0.1
Foxred1	9E-01	0.1	Rab40c	6E-01	0.1
Szrd1	1E-01	0.1	Wiz	6E-01	0.1
Ndufaf2	7E-02	0.1	Zfp606	6E-01	0.1
Smarcc1	7E-05	0.1	Nceh1	5E-01	0.1
P4hb	3E-08	0.1	Ccdc117	8E-01	0.1
Cdipt	2E-01	0.1	Top3b	6E-01	0.1
Ndufb3	9E-08	0.1	Spin1	6E-01	0.1
Hars2	6E-01	0.1	Wdr20	4E-01	0.1
Ddah1	6E-03	0.1	Rsbn1l	3E-01	0.1
Rc3h1	9E-02	0.1	Rere	2E-03	0.1
Bcl2l2	9E-01	0.1	Wdr33	5E-03	0.1
Surf1	6E-02	0.1	Lix1l	2E-01	0.1
Actr6	4E-01	0.1	Amn1	4E-01	0.1
Peli1	2E-01	0.1	Prpf39	3E-01	0.1
Eif2ak1	8E-02	0.1	Ppp4r3b	3E-02	0.1
Frg1	5E-03	0.1	Nupl2	8E-01	0.1
Tmsb15b1	6E-01	0.1	Dnajc27	8E-01	0.1
Fahd1	1E-01	0.1	Uhrf1bp1	9E-01	0.1
Dus4l	4E-01	0.1	Ubl5	2E-07	0.1
Atp5j	1E-14	0.1	Cby1	1E-01	0.1
Nipa1	7E-01	0.1	Sp1	6E-01	0.1
Praf2	2E-03	0.1	Ube2f	1E-01	0.1
Thoc5	3E-01	0.1	Mettl23	8E-02	0.1
Tsen15	2E-02	0.1	Hnrnpdl	2E-03	0.1
Glrx2	6E-04	0.1	Zfp763	5E-01	0.1
Fbxl4	4E-01	0.1	Osgepl1	7E-01	0.1
Adar	6E-01	0.1	Nek8	4E-01	0.1
Pik3ip1	8E-02	0.1	Tug1	4E-01	0.1
Cdk12	8E-04	0.1	Commd9	4E-01	0.1
Odr4	4E-04	0.1	Trp53inp2	6E-01	0.1
Nrbp1	3E-03	0.1	Crebbp	4E-03	0.1
Habp4	3E-01	0.1	Smim13	8E-01	0.1
Clock	5E-02	0.1	Pigs	4E-01	0.1
Zfp667	9E-01	0.1	Phf14	3E-02	0.1
Aftph	2E-01	0.1	Erbb2	9E-01	0.1
Pnkp	9E-02	0.1	Slc2a1	1E+00	0.1
Ppcdc	7E-01	0.1	Wdr24	6E-01	0.1
Scoc	3E-04	0.1	Ice2	9E-01	0.1
Cog6	1E-01	0.1	Fam98a	1E-01	0.1
Tbk1	6E-02	0.1	Kmt5b	8E-02	0.1
Rptor	3E-01	0.1	Pds5a	6E-02	0.1
R3hcc1l	5E-01	0.1	Ube2e3	4E-04	0.1
Fbxl7	1E-01	0.1	Elp1	5E-01	0.1
Zfp760	2E-01	0.1	Klhl42	2E-01	0.1
Rnpc3	2E-01	0.1	Crkl	8E-01	0.1
Afg1l	5E-01	0.1	Bag4	8E-01	0.1
Mrpl38	3E-02	0.1	Fam192a	2E-01	0.1
Mynn	2E-01	0.1	Spg7	7E-02	0.1

Fibroblast

Control			NICD2		
Gene	*p*	FC	Gene	*p*	FC

Fibroblast?

Control			NICD2		
Gene	*p*	FC	Gene	*p*	FC
Eif4ebp2	4E-09	0.2	Tecpr2	7E-13	0.2
Fbln2	1E-08	0.2	D8Ertd738e	6E-22	0.2
Caskin2	1E-05	0.2	Cables1	1E-07	0.2
Gtf2f2	2E-09	0.2	2310009B15Rik	3E-07	0.2
S100a13	4E-08	0.2	Hs6st1	5E-08	0.2
Mdm4	3E-07	0.2	Gtf2e1	1E-05	0.2
Cfap47	2E-05	0.2	Mblac1	1E-06	0.2
Uck1	8E-09	0.2	Noc2l	2E-06	0.2
Orc5	2E-10	0.2	Psmb2	2E-13	0.2
Abhd13	2E-09	0.2	Cct3	1E-12	0.2
Slc35a4	2E-09	0.2	Eif2ak2	1E-10	0.2
Pip4k2a	3E-11	0.2	Axin2	3E-05	0.2
Mrps23	4E-08	0.2	Tgfb1i1	2E-04	0.2
Bbs9	5E-10	0.2	Snn	1E-09	0.2
Cspp1	6E-08	0.2	Tsta3	2E-10	0.2
Lmtk2	4E-08	0.2	Ptgs1	2E-09	0.2
Osbpl1a	4E-05	0.2	Oip5os1	1E-13	0.2
Etv1	8E-05	0.2	Mrps16	6E-11	0.2
Ergic1	5E-11	0.2	Mast3	6E-12	0.2
Timm17b	9E-11	0.2	Ssr1	7E-18	0.2
Ddx23	2E-07	0.2	Atad3a	4E-13	0.2
Api5	7E-08	0.2	Dhx29	1E-08	0.2
Snx11	1E-06	0.2	Eif2s2	3E-12	0.2
Hnrnph2	3E-09	0.2	Adamts15	9E-07	0.2
Lrrc49	9E-12	0.2	Psmd3	1E-10	0.2
Eif4h	1E-09	0.2	Pknox1	3E-11	0.2
Plxnb2	3E-10	0.2	Klhdc3	7E-10	0.2
Kif1c	5E-08	0.2	1110019D14Rik	3E-08	0.2
Lrp5	3E-06	0.2	Zfp612	1E-07	0.2
Bag4	4E-10	0.2	Ndufab1	3E-18	0.2
Gpn1	1E-08	0.2	Fnbp4	5E-09	0.2
Dgkh	4E-11	0.2	Atp5g3	5E-17	0.2
Stimate	2E-08	0.2	Chordc1	3E-04	0.2
Plaat3	3E-09	0.2	Galk1	5E-07	0.2
Gemin7	7E-07	0.2	Polr2m	1E-12	0.2
2610507B11Rik	3E-07	0.2	Pqlc3	2E-10	0.2
Trdmt1	1E-07	0.2	2700054A10Rik	3E-07	0.2
Smdt1	1E-20	0.2	Cx3cl1	4E-11	0.2
Rbms1	5E-16	0.2	Cep68	6E-10	0.2
Ctbp2	2E-09	0.2	Uba6	5E-10	0.2
Slc25a39	2E-11	0.2	Macf1	2E-14	0.2
Ikbkg	3E-07	0.2	Klf6	7E-10	0.2
Bcas2	1E-08	0.2	Apmap	1E-10	0.2
Mterf3	3E-01	0.2	Sprtn	2E-12	0.2
Atf3	9E-01	0.2	Atp5o	5E-23	0.2
Greb1l	3E-09	0.2	Mdh2	2E-08	0.2
Elp4	1E-08	0.2	Flnb	1E-16	0.2
Rap2b	4E-09	0.2	Fnip2	2E-13	0.2
Mapk14	6E-08	0.2	Dus1l	4E-12	0.2
Slc12a6	2E-06	0.2	Gm7030	2E-08	0.2
Phf20	9E-08	0.2	Tmem265	1E-07	0.2
Llgl1	9E-09	0.2	Metrnl	2E-09	0.2
Zfp57	2E-06	0.2	Gid4	3E-09	0.2
Tfip11	2E-08	0.2	Otud1	6E-09	0.2
Insyn2a	8E-06	0.2	Cers4	2E-11	0.2
Gm9844	3E-06	0.2	Mier1	4E-13	0.2
Gtf3c2	2E-09	0.2	Zfp512	6E-10	0.2
Rasal2	6E-11	0.2	Kansl3	2E-10	0.2
Tead1	2E-08	0.2	Josd1	3E-09	0.2
Ptar1	9E-10	0.2	Sap30bp	1E-05	0.2
Pgam5	6E-09	0.2	Smarcad1	2E-10	0.2
Eif2s3x	2E-09	0.2	Mrps15	9E-09	0.2
D10Wsu102e	8E-09	0.2	Slc27a4	9E-10	0.2
Apoa2	2E-04	0.2	Gm9844	2E-09	0.2
Flnb	3E-13	0.2	B3glct	5E-08	0.2
Hlx	2E-05	0.2	Runx2os1	2E-07	0.2
Kansl3	2E-08	0.2	Elovl4	1E-06	0.2
2900052L18Rik	2E-08	0.2	Cct8	2E-10	0.2
Cramp1l	1E-09	0.2	Ubp1	2E-07	0.2
Kpna4	4E-11	0.2	Lipo3	6E-09	0.2
Slc9a1	9E-11	0.2	Gm12655	2E-10	0.2
Adra1b	3E-10	0.2	Esr1	1E-09	0.2
Nox1	2E-05	0.2	Ubqln2	5E-12	0.2

Undefined

Control			NICD2		
Gene	*p*	FC	Gene	*p*	FC

Articular/Synovial Fibroblast

Control			NICD2		
Gene	*p*	FC	Gene	*p*	FC

Limb Mesenchyme						Chondrogenic						Fibroblast						Fibroblast?						Undefined						Articular/Synovial Fibroblast					
Control			NICD2			Control			NICD2			Control			NICD2			Control			NICD2			Control			NICD2			Control			NICD2		
Gene	p	FC	Gene	p	FC	Gene	p	FC	Gene	p	FC	Gene	p	FC	Gene	p	FC	Gene	p	FC	Gene	p	FC	Gene	p	FC	Gene	p	FC	Gene	p	FC	Gene	p	FC
Fam222b	4E-03	0.1	Seh1l	2E-05	0.1	Dbt	1E-01	0.1	Prdx3	4E-02	0.1							Med24	5E-08	0.2	Ttbk2	2E-10	0.2												
Ift43	2E-05	0.1	Rdm1	4E-03	0.1	Socs7	9E-01	0.1	Ddx55	8E-01	0.1							Rad23b	3E-08	0.2	Adat1	3E-10	0.2												
Wdr59	3E-04	0.1	Atp9b	1E-03	0.1	Eid2	6E-03	0.1	Ebpl	1E-02	0.1							Palmd	4E-04	0.2	Fam133b	6E-07	0.2												
Cry2	6E-05	0.1	Tnpo1	7E-04	0.1	Atp6v0e	8E-06	0.1	Gpr107	2E-01	0.1							4930556N13Rik	2E-04	0.2	Il17rc	8E-12	0.2												
Lactb	9E-04	0.1	Ppp2ca	3E-06	0.1	Atg4a	7E-01	0.1	Usp10	7E-02	0.1							Inka1	9E-08	0.2	Ncor2	9E-11	0.2												
Mier3	7E-04	0.1	Lrfn4	8E-04	0.1	Icam1	3E-01	0.1	Capn7	8E-01	0.1							Atp5o	4E-16	0.2	Crkl	6E-09	0.2												
Rexo5	1E-01	0.1	Hs2st1	1E-04	0.1	Rnf157	8E-01	0.1	Wac	1E-03	0.1							Mrpl16	5E-10	0.2	Bclaf1	1E-13	0.2												
Lrpprc	2E-04	0.1	Ipo13	4E-03	0.1	Gatc	1E+00	0.1	Polr3c	5E-01	0.1							Rfxap	2E-07	0.2	Zfp251	2E-08	0.2												
Hinfp	1E-02	0.1	Ddx20	1E-02	0.1	Paics	3E-03	0.1	Kif3a	1E-01	0.1							Coq10b	2E-10	0.2	Tsg101	5E-10	0.2												
Slain2	1E-03	0.1	Strip1	8E-04	0.1	Zcwpw2	4E-01	0.1	Stau2	5E-01	0.1							Epas1	2E-06	0.2	Tomm20	6E-14	0.2												
Emc4	2E-05	0.1	Sorbs3	5E-04	0.1	Stip1	1E-02	0.1	Xpo7	4E-02	0.1							Trim3	6E-08	0.2	Ywhag	2E-08	0.2												
Tns2	2E-03	0.1	Polr1b	2E-02	0.1	Zfp74	4E-01	0.1	Rfx3	8E-01	0.1							Acot11	1E-06	0.2	Gsap	7E-10	0.2												
Ldb1	5E-05	0.1	Ascc2	2E-03	0.1	Cetn3	5E-03	0.1	Cramp1l	6E-01	0.1							Gpc3	4E-07	0.2	Fkbp1a	4E-16	0.2												
Bet1l	8E-06	0.1	Abcf3	4E-03	0.1	Tlr2	8E-01	0.1	Tnfsf13	2E-01	0.1							Reep5	2E-20	0.2	Cald1	5E-15	0.2												
Rpp40	2E-03	0.1	Cnot1	7E-05	0.1	Ebpl	8E-05	0.1	Maea	7E-02	0.1							Adprhl2	5E-11	0.2	Dicer1	1E-07	0.2												
Znrf1	7E-04	0.1	Atat1	1E-03	0.1	Scarb2	2E-02	0.1	Mthfd1l	5E-01	0.1							Iws1	2E-08	0.2	Ralgps1	2E-08	0.2												
Srp54a	1E-03	0.1	Pus7l	3E-02	0.1	CAAA01118383.1	1E-01	0.1	Rcn2	1E-03	0.1							Ndufb7	2E-12	0.2	Mrps27	1E-05	0.2												
Ubxn4	4E-08	0.1	Soga1	1E-02	0.1	Cnot9	4E-01	0.1	Irf2bp2	6E-02	0.1							Btbd6	2E-07	0.2	Cyb5b	1E-12	0.2												
1810055G02Rik	1E-03	0.1	Taf1d	3E-04	0.1	6030458C11Rik	2E-01	0.1	Fam151b	9E-01	0.1							Ywhae	5E-23	0.2	Cfap298	2E-11	0.2												
Ift172	1E-06	0.1	Zfp39	4E-02	0.1	Brd7	8E-04	0.1	Ddx31	1E+00	0.1							Ppp1r3f	1E-04	0.2	Eif3c	2E-17	0.2												
Zc3h14	1E-04	0.1	Bend6	9E-03	0.1	Nme1	5E-06	0.1	Scoc	2E-03	0.1							Ptgfrn	2E-10	0.2	2610507B11Rik	4E-11	0.2												
Acaca	4E-08	0.1	Tma16	4E-04	0.1	Mrps14	2E-05	0.1	Mettl17	8E-01	0.1							Pcbp2	6E-22	0.2	Tbx3	3E-09	0.2												
Rab28	1E-03	0.1	Tufm	7E-03	0.1	Trmt44	8E-01	0.1	Patz1	4E-01	0.1							Cmtr1	5E-10	0.2	Scrn3	2E-10	0.2												
Uba1	2E-05	0.1	Prpf38a	1E-04	0.1	Hacd3	6E-02	0.1	Gtf3c3	2E-01	0.1							Plscr1	8E-11	0.2	Exosc3	5E-10	0.2												
Hmgn1	2E-13	0.1	Smim19	9E-05	0.1	Acaca	4E-03	0.1	Mark2	2E-01	0.1							D8Ertd738e	3E-16	0.2	Gpr27	5E-07	0.2												
Serinc3	3E-06	0.1	Ninl	2E-04	0.1	Dmtf1	2E-02	0.1	Wdr53	1E+00	0.1							Slc25a11	3E-05	0.2	Lrrfip2	3E-06	0.2												
2300009A05Rik	1E-04	0.1	Mecr	2E-03	0.1	Prr12	6E-01	0.1	Upf2	1E-01	0.1							Taf2	7E-08	0.2	2410004B18Rik	4E-12	0.2												
Bop1	7E-03	0.1	Txndc5	1E-03	0.1	Lamtor2	1E-06	0.1	Mta1	2E-01	0.1							Lurap1l	4E-09	0.2	Spag1	8E-07	0.2												
Chchd3	8E-06	0.1	Prpf3	3E-03	0.1	Prpf3	3E-01	0.1	Tmbim4	1E-03	0.1							Suds3	9E-09	0.2	Timm9	5E-08	0.2												
Scfd2	1E-05	0.1	2700054A10Rik	1E-01	0.1	6530409C15Rik	8E-01	0.1	Zfp706	3E-05	0.1							Ncald	3E-06	0.2	Insyn1	7E-07	0.2												
Asf1a	2E-04	0.1	Dnajb14	3E-03	0.1	Exosc10	3E-01	0.1	Igsf10	8E-01	0.1							Uba3	9E-08	0.2	Hmgn3	1E-10	0.2												
Gzf1	1E-04	0.1	Map2k5	2E-02	0.1	Fbxw4	9E-02	0.1	Itsn2	5E-01	0.1							Wwp1	5E-09	0.2	Upf3a	3E-10	0.2												
Pak2	1E-06	0.1	Ldb1	7E-04	0.1	Ssu72	6E-06	0.1	Vps33b	7E-01	0.1							Trappc10	1E-08	0.2	Vamp5	5E-08	0.2												
Cdk9	3E-04	0.1	Iws1	9E-04	0.1	Med13	3E-03	0.1	1700052K11Rik	8E-01	0.1							N4bp1	6E-07	0.2	Fut8	3E-11	0.2												
Zmym5	2E-04	0.1	Acaca	2E-07	0.1	Nfyc	5E-01	0.1	Malsu1	2E-02	0.1							Mapk3	5E-08	0.2	Ndufb7	8E-16	0.2												
Usp15	6E-04	0.1	Hcfc2	8E-04	0.1	Pum2	2E-02	0.1	Enox2	5E-01	0.1							Rtl6	2E-04	0.2	Ostf1	1E-10	0.2												
Tefm	2E-03	0.1	Virma	6E-04	0.1	Ints14	2E-01	0.1	Usp38	9E-01	0.1							Erg28	5E-14	0.2	Ampd3	2E-09	0.2												
Arhgef6	4E-03	0.1	Rap1gds1	1E-04	0.1	Micu2	3E-02	0.1	Ypel2	6E-01	0.1							Mrps16	3E-08	0.2	Rhbdd2	3E-13	0.2												
Hagh	1E-06	0.1	Sec23ip	1E-04	0.1	Slain2	1E-01	0.1	Ube2q2	4E-02	0.1							Ccnc	1E-08	0.2	Rabggtb	7E-12	0.2												
Cpox	2E-04	0.1	Smtn	2E-03	0.1	Ppp6r1	4E-01	0.1	Usp37	2E-01	0.1							Nhs	1E-07	0.2	Tbcb	1E-08	0.2												
Ppcs	5E-04	0.1	Parn	4E-03	0.1	Phc3	2E-02	0.1	Usp54	8E-01	0.1							Ing3	1E-08	0.2	Pum2	4E-09	0.2												
Tbc1d15	2E-05	0.1	Adck5	3E-04	0.1	Ppat	2E-01	0.1	Pcca	6E-02	0.1							Traf3	3E-06	0.2	Zcchc17	4E-08	0.2												
Clpx	1E-04	0.1	Gm10561	2E-02	0.1	Gng5	7E-08	0.1	Togaram1	1E-01	0.1							Tead3	2E-07	0.2	Cct5	4E-10	0.2												
Zmpste24	1E-03	0.1	Ecd	2E-03	0.1	Mrtfb	8E-02	0.1	Insig2	4E-02	0.1							Sirt1	7E-06	0.2	Dapp1	2E-09	0.2												
Uqcc1	2E-05	0.1	Coq4	8E-03	0.1	C9orf72	2E-01	0.1	Rrp8	2E-01	0.1							Adgra2	2E-09	0.2	Gm16093	2E-05	0.2												
Sec11a	3E-06	0.1	Mtfr1l	6E-05	0.1	Rnf168	1E-01	0.1	Pex1	1E+00	0.1							Col6a2	5E-09	0.2	Mrpl57	8E-11	0.2												
Thoc3	7E-04	0.1	Phyh	3E-02	0.1	BC005561	6E-01	0.1	Atp5j	3E-10	0.1							Luzp1	1E-09	0.2	Nr2f6	2E-07	0.2												
Usp38	1E-03	0.1	Mrrf	1E-04	0.1	C1s1	2E-01	0.1	Dock5	9E-01	0.1							Ube2l3	6E-12	0.2	Dohh	2E-07	0.2												
Med25	2E-04	0.1	Polr2d	1E-04	0.1	B9d2	4E-01	0.1	Ptcd1	3E-01	0.1							2310061I04Rik	6E-12	0.2	Timm13	6E-17	0.2												
Pccb	9E-05	0.1	Pacsin2	4E-04	0.1	Sumo1	1E-06	0.1	Zbed4	6E-01	0.1							Rbp1	2E-10	0.2	Ctnna1	8E-14	0.2												
Dusp7	2E-03	0.1	Ccpg1	4E-04	0.1	Snapc1	7E-02	0.1	Asb6	3E-01	0.1							Cox5a	2E-20	0.2	Pitpna	2E-11	0.2												
Gabarapl2	8E-09	0.1	Atg14	4E-04	0.1	Rmc1	1E+00	0.1	Faap20	8E-01	0.1							Ube2k	7E-12	0.2	Hnrnpll	1E-11	0.2												
Rer1	8E-08	0.1	Ntmt1	8E-05	0.1	Armc8	7E-01	0.1	Dnajc17	7E-01	0.1							Eef1e1	3E-09	0.2	Gchfr	4E-10	0.2												
Ube2w	2E-04	0.1	Ifnar1	9E-04	0.1	Crnkl1	8E-02	0.1	H3f3b	3E-09	0.1							Gm49959	8E-08	0.2	Foxn2	2E-09	0.2												
Rnf135	8E-03	0.1	Hps5	7E-04	0.1	Stk16	8E-03	0.1	Sf3b1	3E-03	0.1							Gne	1E-08	0.2	Mycbp	5E-09	0.2												
Ralgapa1	5E-04	0.1	Zfp180	4E-03	0.1	Cpsf7	5E-02	0.1	Snx33	7E-01	0.1							Traf7	3E-09	0.2	Tmem128	6E-11	0.2												
Ecm2	1E-04	0.1	Pcmtd1	5E-04	0.1	Raf1	9E-03	0.1	U2af1l4	6E-01	0.1							Kbtbd4	2E-09	0.2	Tmem238	8E-09	0.2												
Sel1l	4E-06	0.1	Tob2	4E-03	0.1	Rdh14	2E-01	0.1	Kmt2a	1E-02	0.1							Naa20	2E-07	0.2	Chchd7	3E-05	0.2												
Ccser2	2E-06	0.1	Ints13	5E-02	0.1	Arf4	2E-09	0.1	Mon2	5E-02	0.1							Ythdc1	4E-10	0.2	Ppp4r1	7E-10	0.2												
Map3k3	2E-04	0.1	Zfp629	8E-02	0.1	Ehd4	6E-01	0.1	Mrpl55	3E-02	0.1							Ensa	5E-08	0.2	Zfp626	1E-07	0.2												
Bbip1	3E-05	0.1	Gm43713	4E-04	0.1	Zfp65	8E-01	0.1	Gm10785	5E-01	0.1							Bop1	3E-09	0.2	Mfap5	2E-04	0.2												
Trio	1E-05	0.1	Arl1	8E-09	0.1	Yars2	4E-01	0.1	Smad5	8E-01	0.1							Uspl1	1E-07	0.2	Kin	4E-08	0.2												
Rnf34	8E-05	0.1	Fam214b	5E-03	0.1	Snai2	3E-01	0.1	Fxr1	6E-04	0.1							M6pr	3E-07	0.2	P4htm	2E-07	0.2												
Mta2	5E-04	0.1	Slc9a8	1E-04	0.1	Prdm5	6E-03	0.1	Ppme1	2E-01	0.1							Rwdd1	3E-11	0.2	Sytl4	1E-02	0.2												
Zfp326	1E-05	0.1	Qrich1	2E-04	0.1	D330041H03Rik	7E-01	0.1	Hsph1	8E-02	0.1							Srf	3E-09	0.2	Usp10	4E-09	0.2												
Polr3b	5E-03	0.1	Hmgcs1	7E-01	0.1	2900052L18Rik	1E+00	0.1	Chtf8	7E-01	0.1							Rnf8	1E-07	0.2	Pdrg1	1E-07	0.2												
Ift74	1E-05	0.1	Rraga	1E-05	0.1	Katnbl1	3E-01	0.1	Slc35a4	8E-01	0.1							Nae1	2E-06	0.2	Mob1a	1E-05	0.2												
St3gal3	3E-05	0.1	Npat	5E-04	0.1	Agap1	1E-02	0.1	Gpr153	3E-01	0.1							Olfml2b	3E-04	0.2	Gabpb2	3E-09	0.2												
Hmbox1	4E-03	0.1	Hspa13	3E-04	0.1	Gnpnat1	3E-01	0.1	Acbd4	8E-01	0.1							Anxa2	9E-19	0.2	Gucd1	1E-06	0.2												
2610507B11Rik	7E-05	0.1	B4galt4	3E-03	0.1	mt-Co3	1E-15	0.1	Ctbs	7E-01	0.1							Nvl	3E-08	0.2	Smdt1	4E-21	0.2												
Gm11508	4E-02	0.1	Sox5	2E-14	0.1	Mvb12a	1E-02	0.1	D430042O09Rik	6E-01	0.1							Klf11	5E-06	0.2	Stox2	5E-05	0.2												
Ammecr1l	1E-02	0.1	Ube2cbp	9E-02	0.1	Tmem35b	8E-01	0.1	Rnf141	6E-01	0.1							Pde4b	6E-11	0.2	Rock1	5E-15	0.2												
Eef1akmt2	1E-03	0.1	Mterf1a	2E-02	0.1	Igf1r	3E-02	0.1	Nsd1	1E-03	0.1							Sap30	2E-09	0.2	Cyld	5E-10	0.2												

Limb Mesenchyme					
Control			NICD2		
Gene	*p*	FC	Gene	*p*	FC
Brpf1	8E-04	0.1	Mlh1	9E-03	0.1
Zmym4	3E-05	0.1	Pus3	1E-02	0.1
Tmem199	3E-03	0.1	Actr1b	1E-02	0.1
Cdc123	3E-05	0.1	Tmem101	4E-04	0.1
Crebl2	4E-04	0.1	Bzw1	2E-07	0.1
Mrc2	3E-05	0.1	Ube2i	7E-08	0.1
Cdk5rap3	1E-04	0.1	Vasn	1E-03	0.1
Katna1	2E-04	0.1	Tvp23b	6E-05	0.1
Mmgt2	1E-04	0.1	Uhmk1	3E-03	0.1
Rars	5E-04	0.1	Sart1	4E-03	0.1
Trim8	1E-03	0.1	Gm16867	3E-01	0.1
Dhx40	8E-05	0.1	Tbrg1	1E-07	0.1
Tsnax	4E-04	0.1	Setd2	1E-03	0.1
Harbi1	3E-03	0.1	Smpd1	9E-05	0.1
Rnf216	2E-03	0.1	Tbc1d14	1E-03	0.1
Stard3nl	6E-05	0.1	Chmp1b	2E-03	0.1
Zfp871	5E-04	0.1	Pcbp4	2E-04	0.1
Phpt1	3E-04	0.1	Vps13d	2E-04	0.1
Yeats4	1E-04	0.1	Gm26549	8E-01	0.1
Fam136a	5E-06	0.1	Dph5	1E-03	0.1
Ppm1g	6E-06	0.1	Mid1ip1	6E-03	0.1
Eif2ak1	9E-04	0.1	Acad9	3E-03	0.1
Nol11	3E-04	0.1	Zfp330	1E-02	0.1
Rhebl1	6E-02	0.1	Ubp1	4E-04	0.1
Map2k1	2E-03	0.1	Pam16	3E-05	0.1
Smg8	1E-03	0.1	Gm46367	6E-03	0.1
Med17	3E-03	0.1	Psmg3	1E-03	0.1
Gm16536	1E-03	0.1	Ogdh	1E-03	0.1
Rad54l2	3E-04	0.1	Mrpl20	8E-09	0.1
Prkab1	7E-04	0.1	Gorab	1E-03	0.1
Zfp236	1E-04	0.1	Wdr70	4E-05	0.1
Pla2g6	3E-02	0.1	C9orf72	2E-04	0.1
Fbxo8	2E-05	0.1	Ehmt1	8E-05	0.1
Nae1	2E-03	0.1	Rrnad1	6E-03	0.1
Rab23	2E-03	0.1	Zfp560	5E-04	0.1
Vamp8	6E-07	0.1	Zfp318	1E-02	0.1
Dele1	1E-02	0.1	Mrps9	1E-03	0.1
Fnbp4	4E-03	0.1	Impdh1	2E-03	0.1
Fbxl17	4E-06	0.1	Lpar1	8E-04	0.1
Rab3ip	5E-02	0.1	Rbl2	6E-03	0.1
Trim27	2E-04	0.1	Zfp160	5E-04	0.1
Tgif1	3E-04	0.1	Mrpl37	6E-03	0.1
Edem1	2E-03	0.1	1810030O07Rik	4E-05	0.1
Spred3	4E-03	0.1	Dtnb	1E-01	0.1
Eva1b	2E-03	0.1	Eif4g3	7E-04	0.1
Myo9b	9E-04	0.1	4930447C04Rik	3E-02	0.1
Ndrg3	8E-04	0.1	Fam192a	3E-03	0.1
Prmt3	4E-04	0.1	Gm20721	2E-05	0.1
Rce1	8E-05	0.1	A530072M11Rik	5E-02	0.1
Mrm3	6E-03	0.1	Pigt	9E-04	0.1
Kdm1b	2E-02	0.1	Slc35e1	3E-02	0.1
Klhl13	4E-03	0.1	Speg	5E-03	0.1
Zfp335	2E-02	0.1	Tada1	1E-03	0.1
Man2c1os	4E-03	0.1	Surf4	1E-05	0.1
Mob1a	3E-04	0.1	Nabp1	4E-05	0.1
Hnrnpk	3E-10	0.1	Cacng7	4E-03	0.1
Hnrnpu	2E-10	0.1	Rnpc3	1E-03	0.1
Stag2	3E-05	0.1	Fkrp	2E-04	0.1
Exosc9	1E-04	0.1	Ccnl1	9E-04	0.1
Mettl2	2E-04	0.1	Rbm7	2E-04	0.1
Togaram1	9E-04	0.1	Fam20b	3E-03	0.1
Bpgm	2E-03	0.1	Apaf1	2E-03	0.1
Slc35c1	2E-02	0.1	Fancm	7E-02	0.1
Strn4	6E-03	0.1	Prrc2b	5E-04	0.1
Sppl2a	1E-04	0.1	Armc5	2E-02	0.1
Ubap2l	2E-08	0.1	Rad52	1E-03	0.1
Pacsin2	7E-05	0.1	Bptf	3E-04	0.1
Wdfy2	2E-05	0.1	H2afy	3E-05	0.1
Sf3a3	7E-05	0.1	Apc	2E-03	0.1
Ube2r2	3E-08	0.1	Ahsa2	3E-04	0.1
Dhdds	1E-02	0.1	Scyl1	2E-03	0.1
Tmem104	5E-03	0.1	Map11	2E-02	0.1
Mar8	2E-04	0.1	Wdr78	9E-03	0.1

Chondrogenic					
Control			NICD2		
Gene	*p*	FC	Gene	*p*	FC
Dleu2	2E-04	0.1	Snx29	9E-02	0.1
Polr3g	7E-01	0.1	Isyna1	8E-01	0.1
Csde1	2E-05	0.1	Ftsj3	6E-01	0.1
Arih1	7E-03	0.1	Ptk2	1E-01	0.1
Tbc1d4	2E-01	0.1	Maf1	7E-01	0.1
Trmt2b	4E-01	0.1	Zfp266	1E-02	0.1
Ube2g1	4E-02	0.1	Fktn	7E-01	0.1
Zmym1	9E-01	0.1	Plpp2	7E-01	0.1
Alkbh1	2E-02	0.1	Aven	5E-01	0.1
Emc4	1E-01	0.1	Chchd2	3E-14	0.1
Hadh	4E-04	0.1	Gpx1	1E-04	0.1
Ahdc1	1E-01	0.1	Osbpl2	3E-01	0.1
Blzf1	6E-01	0.1	Plxna3	1E+00	0.1
Alg1	4E-01	0.1	Ppp1r11	1E-02	0.1
4930455C13Rik	4E-01	0.1	Hax1	4E-02	0.1
Cnot8	3E-01	0.1	Bhlhb9	2E-01	0.1
Sdhaf4	5E-03	0.1	Pxn	5E-01	0.1
Celf1	4E-02	0.1	Letm1	9E-01	0.1
Pomp	8E-06	0.1	Gtf2h2	4E-01	0.1
Relb	5E-01	0.1	Zfp790	8E-01	0.1
Hadha	2E-04	0.1	Ddx58	1E+00	0.1
Tspan31	1E-03	0.1	Gm16759	8E-01	0.1
Afg3l2	2E-01	0.1	Cnbd2	8E-01	0.1
Ercc6l2	3E-01	0.1			
Ankle2	2E-01	0.1			
Faap20	3E-02	0.1			
Med11	2E-01	0.1			
Ttc17	2E-01	0.1			
Fbxo44	5E-01	0.1			
Nufip2	2E-03	0.1			
Pfdn1	2E-06	0.1			
Cbx3	1E-05	0.1			
Numa1	3E-02	0.1			
Ube2v2	8E-03	0.1			
Rexo2	8E-07	0.1			
Anxa2	1E-08	0.1			
Nt5m	1E-01	0.1			
9930021J03Rik	5E-02	0.1			
Snai1	8E-01	0.1			
Utp3	8E-03	0.1			
Echdc3	8E-01	0.1			

Fibroblast					
Control			NICD2		
Gene	*p*	FC	Gene	*p*	FC

Fibroblast?					
Control			NICD2		
Gene	*p*	FC	Gene	*p*	FC
Msra	7E-10	0.2	0610010K14Rik	1E-03	0.2
Tsc2	4E-10	0.2	Zfp641	8E-07	0.2
Zfp930	9E-09	0.2	Map3k7	6E-10	0.2
Efcab2	1E-05	0.2	Colgalt1	3E-13	0.2
Crk	8E-08	0.2	Atp2a2	1E-12	0.2
Slc25a15	5E-07	0.2	Map4k4	3E-09	0.2
Ccdc124	1E-06	0.2	Tmem184b	3E-06	0.2
Pusl1	2E-04	0.2	Ryk	3E-15	0.2
Efemp1	1E-09	0.2	Ctf1	2E-09	0.2
Pitpnb	8E-09	0.2	Dlg5	6E-09	0.2
Sypl	6E-07	0.2	Nme6	2E-11	0.2
Card19	2E-06	0.2	Luzp1	2E-12	0.2
Rwdd2b	6E-07	0.2	Ppid	4E-06	0.2
Metrnl	4E-04	0.2	Nubp2	9E-11	0.2
Med12l	5E-04	0.2	Zfp692	1E-10	0.2
Wdr53	6E-10	0.2	1500009L16Rik	8E-11	0.2
Wfs1	1E-07	0.2	Rhoa	1E-19	0.2
Zhx3	5E-11	0.2	Traf5	4E-13	0.2
Pafah1b2	1E-08	0.2	Gm43462	2E-06	0.2
Cbwd1	2E-08	0.2	Zfpm1	2E-09	0.2
Atn1	9E-09	0.2	Tnpo2	1E-10	0.2
Ccdc28b	1E-06	0.2	Gnb1	5E-17	0.2
Ryk	9E-11	0.2	Fance	8E-09	0.2
Xpo5	5E-07	0.2	Dnlz	7E-08	0.2
Bmi1	2E-07	0.2	Morc3	3E-12	0.2
Psmg3	4E-09	0.2	Dhodh	2E-08	0.2
Rexo2	1E-14	0.2	Psmc1	6E-06	0.2
Tmco1	5E-11	0.2	Cnst	3E-10	0.2
Vma21	3E-07	0.2	Ppfibp1	4E-09	0.2
Edc4	3E-09	0.2	Rbck1	1E-07	0.2
Ppl	3E-12	0.2	Ebp	2E-10	0.2
Cnbp	7E-12	0.2	Pomp	1E-17	0.2
Ptprf	5E-10	0.2	Emg1	1E-03	0.2
Map3k12	1E-08	0.2	Zbtb38	7E-08	0.2
Dda1	9E-07	0.2	Zfp512b	7E-10	0.2
Lsm1	1E-08	0.2	Zfp35	5E-09	0.2
Ubn1	3E-07	0.2	Zfp428	1E-12	0.2
6430548M08Rik	7E-05	0.2	Mrpl32	4E-07	0.2
Rhog	3E-09	0.2	Entpd5	1E-09	0.2
Spint2	2E-06	0.2	Spr	9E-07	0.2
B230307C23Rik	3E-09	0.2	Pcbp2	2E-23	0.2
Trim11	6E-09	0.2	Nxf1	2E-11	0.2
Mrpl10	5E-11	0.2	Ubn1	3E-09	0.2
Fhl1	8E-09	0.2	Spred2	2E-12	0.2
Nat2	4E-08	0.2	B9d2	2E-12	0.2
Gm10135	2E-03	0.2	Slc25a17	9E-08	0.2
Yaf2	4E-09	0.2	Tbrg4	4E-09	0.2
Macrod2	9E-12	0.2	Ndufaf5	3E-11	0.2
Uimc1	2E-04	0.2	Phykpl	6E-08	0.2
Eif5a2	3E-01	0.2	Nab2	9E-13	0.2
Zfp593	1E-07	0.2	Lurap1l	9E-10	0.2
BC005537	1E-09	0.2	Tmsb4x	2E-25	0.2
Ddx51	1E-05	0.2	Gtf2ird1	7E-10	0.2
Ankrd44	3E-07	0.2	Mlec	3E-16	0.2
Pitpna	2E-09	0.2	Csnk2a2	5E-06	0.2
Cct3	4E-08	0.2	Sorcs2	1E-08	0.2
G6pdx	8E-08	0.2	Atg101	6E-08	0.2
Nkain2	2E-06	0.2	Efnb2	6E-14	0.2
Dus1l	5E-09	0.2	Naa38	6E-06	0.2
Arrdc3	1E-07	0.2	Cab39	1E-06	0.2
Natd1	8E-05	0.2	Fzd6	2E-06	0.2
Rnpep	5E-09	0.2	Spata6	3E-11	0.2
Cnot3	5E-07	0.2	Dhdds	3E-06	0.2
Nudt19	3E-06	0.2	Nhlrc2	4E-11	0.2
Pomgnt1	6E-08	0.2	Zhx3	3E-12	0.2
Ctps2	7E-09	0.2	Lsm1	2E-11	0.2
Imp4	2E-07	0.2	Eif2s3x	4E-06	0.2
Serpinb6a	2E-14	0.2	Trip6	8E-08	0.2
Abhd17b	7E-07	0.2	Sh3bgrl3	1E-15	0.2
Ppp1r13b	3E-04	0.2	Kctd10	3E-13	0.2
Adam19	1E-08	0.2	Rnaseh1	2E-09	0.2
Tirap	5E-07	0.2	9130023H24Rik	7E-07	0.2
Cxadr	1E-06	0.2	Natd1	4E-06	0.2

Undefined					
Control			NICD2		
Gene	*p*	FC	Gene	*p*	FC

Articular/Synovial Fibroblast					
Control			NICD2		
Gene	*p*	FC	Gene	*p*	FC

Limb Mesenchyme					
Control			NICD2		
Gene	*p*	FC	Gene	*p*	FC
Abca2	7E-03	0.1	Gpr137	3E-04	0.1
Tm7sf3	1E-04	0.1	Hus1	6E-02	0.1
Acin1	4E-06	0.1	Sav1	4E-04	0.1
Ahctf1	1E-04	0.1	Rrn3	2E-04	0.1
Bloc1s4	4E-04	0.1	Ltbp3	2E-04	0.1
Zfp14	4E-03	0.1	Prorsd1	2E-04	0.1
Limk2	3E-04	0.1	Rbm39	2E-09	0.1
Elp4	1E-03	0.1	Arhgef7	1E-03	0.1
Hacd1	2E-02	0.1	Mzt1	5E-05	0.1
Rhot1	2E-04	0.1	Nom1	4E-02	0.1
Snx19	7E-04	0.1	Golt1b	2E-03	0.1
Dhx30	3E-03	0.1	Nif3l1	2E-03	0.1
Nup93	6E-04	0.1	Zmiz2	8E-04	0.1
Tcte2	2E-04	0.1	Nfatc3	3E-04	0.1
Hist1h2ac	3E-05	0.1	Mtmr14	9E-03	0.1
Zfp58	1E-02	0.1	Vcp	4E-07	0.1
Txnrd3	2E-02	0.1	Mff	3E-06	0.1
Stox2	6E-04	0.1	Cnnm2	4E-02	0.1
Tmem222	2E-03	0.1	Rbm5	5E-04	0.1
Ccdc58	3E-04	0.1	Zbtb43	2E-03	0.1
Armc8	2E-04	0.1	Sepsecs	2E-02	0.1
Utp3	3E-05	0.1	Rnaseh1	4E-03	0.1
Sacm1l	1E-03	0.1	Dlg5	1E-03	0.1
Srsf11	2E-06	0.1	Itfg2	6E-03	0.1
Cisd1	8E-08	0.1	Zdhhc24	3E-03	0.1
Mlf2	8E-06	0.1	Sfi1	3E-03	0.1
Dync1i2	9E-07	0.1	Ppp6r2	5E-03	0.1
B3galt6	4E-03	0.1	Chmp2b	4E-04	0.1
Fgfr1op2	3E-05	0.1	Eny2	9E-07	0.1
Mtdh	3E-08	0.1	Mga	5E-04	0.1
Safb	8E-05	0.1	Nsd1	3E-05	0.1
Smarca4	4E-04	0.1	Stam	3E-05	0.1
Skp1a	2E-09	0.1	Cep97	2E-02	0.1
Pbxip1	9E-06	0.1	Mink1	3E-03	0.1
Safb2	2E-04	0.1	Mapre1	3E-06	0.1
Thbs3	5E-02	0.1	Cep164	7E-03	0.1
Tmem68	2E-03	0.1	Armc8	6E-04	0.1
Hnrnpf	2E-08	0.1	Ubqln2	2E-03	0.1
Phkb	1E-04	0.1	Zfp706	1E-08	0.1
Tbl2	8E-03	0.1	Ccni	2E-05	0.1
Nras	4E-05	0.1	Ipo8	2E-03	0.1
Psmd7	7E-07	0.1	Zfp664	3E-04	0.1
Myo6	5E-04	0.1	Ankrd46	2E-03	0.1
Fcho2	1E-04	0.1	Rpp14	1E-02	0.1
Cyp27a1	2E-02	0.1	Usf3	1E-03	0.1
Cwf19l1	3E-02	0.1	Ing1	1E-04	0.1
Pygo2	2E-04	0.1	Iqsec1	4E-04	0.1
Piezo1	2E-05	0.1	Agap3	2E-03	0.1
Ino80	3E-03	0.1	Coro7	4E-04	0.1
E130309D02Rik	7E-03	0.1	Pafah2	1E-03	0.1
Wbp4	4E-05	0.1	Cdc23	6E-04	0.1
Lrif1	2E-04	0.1	Ppp1r11	1E-05	0.1
Cox4i2	8E-12	0.1	Dnmt3a	4E-04	0.1
Atrip	9E-04	0.1	Nudcd1	2E-05	0.1
Slc38a10	9E-04	0.1	Nipa1	2E-04	0.1
Sar1b	4E-05	0.1	Zfp532	9E-04	0.1
Cryab	1E-12	0.1	Hdgf	1E-08	0.1
Ncoa1	2E-04	0.1	Apoo	6E-04	0.1
Cherp	2E-04	0.1	Fam234a	6E-04	0.1
Pnrc2	3E-04	0.1	Rngtt	1E-03	0.1
1700052K11Rik	1E-03	0.1	Brpf3	1E-02	0.1
Abcf2	2E-04	0.1	Phf20l1	2E-03	0.1
Phf21a	3E-06	0.1	Gm11613	8E-03	0.1
Dpf2	6E-04	0.1	Mical3	1E-02	0.1
Gtpbp6	2E-03	0.1	Gm49797	1E-03	0.1
Col4a3bp	7E-04	0.1	Med25	4E-03	0.1
Pafah2	2E-05	0.1	Rnpepl1	2E-03	0.1
Faxc	7E-03	0.1	Cdc40	6E-04	0.1
Fitm2	8E-03	0.1	Sec24b	4E-04	0.1
Fam118b	2E-04	0.1	Fam174a	3E-05	0.1
Slc49a4	1E-04	0.1	Secisbp2	4E-04	0.1
Psma3	1E-09	0.1	Kpnb1	5E-04	0.1
Ccar1	3E-04	0.1	Slc25a26	4E-04	0.1

Chondrogenic					
Control			NICD2		
Gene	*p*	FC	Gene	*p*	FC

Fibroblast					
Control			NICD2		
Gene	*p*	FC	Gene	*p*	FC

Fibroblast?					
Control			NICD2		
Gene	*p*	FC	Gene	*p*	FC
Cetn4	8E-09	0.2	Tubgcp5	5E-09	0.2
Klf16	4E-05	0.2	Dgkh	4E-11	0.2
Rnf44	2E-03	0.2	Tfip11	5E-10	0.2
Ppm1b	3E-08	0.2	Med24	5E-10	0.2
Mmadhc	1E-04	0.2	Cfap100	3E-07	0.2
Sfxn1	3E-05	0.2	Wfs1	2E-10	0.2
Ap2s1	4E-13	0.2	Rfxap	2E-11	0.2
Psmd6	2E-08	0.2	Eif4a1	2E-09	0.2
Wasf2	2E-11	0.2	Msto1	4E-06	0.2
Prkaa2	2E-07	0.2	Cthrc1	5E-11	0.2
Wrap73	3E-08	0.2	Taf11	2E-05	0.2
Rad51d	4E-06	0.2	Jmjd6	6E-09	0.2
Rfx1	3E-06	0.2	Sh2d4a	3E-08	0.2
Man2c1	5E-09	0.2	Polr1a	2E-02	0.2
Vcpkmt	9E-10	0.2	Abi2	5E-09	0.2
Dip2a	4E-09	0.2	Sgms1	5E-07	0.2
Zfp81	1E-07	0.2	Rel	3E-11	0.2
F730043M19Rik	2E-05	0.2	Trpc1	7E-11	0.2
Tpm1	9E-12	0.2	Rad23a	3E-08	0.2
Dnajc27	2E-06	0.2	Ddx19b	3E-06	0.2
Camk1d	2E-04	0.2	Dnajb4	6E-06	0.2
Noc2l	5E-04	0.2	Ube2a	7E-11	0.2
H1f0	8E-03	0.2	Zranb2	9E-11	0.2
Iffo1	1E-09	0.2	Arl2	3E-09	0.2
Upf3a	4E-08	0.2	Ttll12	2E-07	0.2
Tnfrsf11b	5E-05	0.2	Tmem50b	7E-12	0.2
Anxa1	3E-13	0.2	Psmg4	2E-04	0.2
Prkag1	1E-07	0.2	Tex261	6E-10	0.2
Gm42726	9E-04	0.2	Bambi	2E-13	0.2
Farsb	4E-04	0.2	Zfp414	1E-10	0.2
Tada2a	2E-06	0.2	Slc25a37	2E-12	0.2
Sf3b3	1E-05	0.2	Rsl1d1	1E-04	0.2
Zfp142	2E-08	0.2	Pon2	1E-08	0.2
Dzip1	7E-06	0.2	Dnm1l	1E-08	0.2
Fzd7	9E-08	0.2	Wdr46	3E-10	0.2
Bhlhb9	9E-06	0.2	Col28a1	2E-09	0.2
Atp5g3	3E-16	0.2	Clcn6	5E-05	0.2
Mecom	3E-04	0.2	Gm17501	4E-14	0.2
C130036L24Rik	4E-05	0.2	Snx11	4E-08	0.2
Pard6a	1E-05	0.2	Ywhaz	4E-16	0.2
Nfatc1	2E-08	0.2	Elp4	5E-10	0.2
Ccdc59	3E-06	0.2	Gm49226	6E-09	0.2
Cnpy4	8E-08	0.2	Zfp593	7E-07	0.2
Med21	3E-04	0.2	Rce1	3E-09	0.2
Mrpl21	2E-08	0.2	Rev1	2E-07	0.2
Naa10	6E-08	0.2	Psmd1	8E-11	0.2
Gm48855	5E-06	0.2	Eno1	8E-10	0.2
Abt1	2E-08	0.2	Coa4	2E-09	0.2
Pacc1	1E-05	0.2	Lrig1	8E-13	0.2
Fnbp1	7E-08	0.2	Peli1	4E-09	0.2
Tpst2	9E-08	0.2	Zbtb7c	2E-10	0.2
Wdr46	5E-05	0.2	Il4ra	2E-11	0.2
Dock9	3E-07	0.2	Ppp1r16a	1E-07	0.2
Prkci	5E-06	0.2	Krr1	3E-09	0.2
Ctdsp1	6E-06	0.2	Zbtb26	2E-10	0.2
Nfe2l2	7E-08	0.2	Spint2	1E-05	0.2
Nhlrc2	5E-09	0.2	Blmh	2E-04	0.2
Tnrc6a	2E-07	0.2	Mogs	1E-08	0.2
Clcf1	1E-07	0.2	Timm17b	9E-09	0.2
Atp5f1	7E-18	0.2	Cdh2	1E-28	0.2
Mipol1	2E-06	0.2	Slc16a1	7E-11	0.2
Fjx1	8E-05	0.2	Tex2	9E-12	0.2
Galc	7E-09	0.2	Bmi1	5E-09	0.2
Tma7	4E-19	0.2	Rnpc3	3E-11	0.2
Gnb1	1E-13	0.2	Ttpal	6E-09	0.2
Eif2b1	5E-08	0.2	Cgnl1	1E-10	0.2
Shq1	2E-07	0.2	Vps13a	3E-08	0.2
Nxf1	2E-08	0.2	Nt5m	1E-07	0.2
Ccnt1	4E-04	0.2	Sptlc2	9E-07	0.2
Zbtb38	3E-05	0.2	Mbd2	5E-09	0.2
Rp2	5E-13	0.2	Eif3a	3E-14	0.2
Prr5	3E-05	0.2	D5Ertd579e	1E-05	0.2
Erich1	2E-05	0.2	Dtwd1	1E-09	0.2

Undefined					
Control			NICD2		
Gene	*p*	FC	Gene	*p*	FC

Articular/Synovial Fibroblast					
Control			NICD2		
Gene	*p*	FC	Gene	*p*	FC

Limb Mesenchyme						Chondrogenic						Fibroblast						Fibroblast?						Undefined						Articular/Synovial Fibroblast					
Control			NICD2			Control			NICD2			Control			NICD2			Control			NICD2			Control			NICD2			Control			NICD2		
Gene	*p*	FC	Gene	*p*	FC	Gene	*p*	FC	Gene	*p*	FC	Gene	*p*	FC	Gene	*p*	FC	Gene	*p*	FC	Gene	*p*	FC	Gene	*p*	FC	Gene	*p*	FC	Gene	*p*	FC	Gene	*p*	FC
Nsrp1	2E-05	0.1	Tada2a	2E-04	0.1													Faf1	9E-08	0.2	Nutf2	2E-08	0.2												
Gripap1	1E-04	0.1	Habp4	2E-04	0.1													Lactb	4E-03	0.2	Metap2	6E-15	0.2												
Smad3	1E-05	0.1	Zrsr1	3E-05	0.1													Gid4	5E-07	0.2	Syde1	2E-10	0.2												
Atat1	1E-04	0.1	Ptprm	7E-06	0.1													Thrap3	1E-09	0.2	Tead3	2E-07	0.2												
Uchl3	1E-03	0.1	Mrps2	7E-03	0.1													Spop	4E-08	0.2	Epb41l3	4E-09	0.2												
Vps33a	2E-03	0.1	Kazald1	9E-03	0.1													Zfp326	1E-06	0.2	Rmnd5b	1E-07	0.2												
Midn	7E-06	0.1	Bcdin3d	3E-02	0.1													Nanp	6E-09	0.2	Nt5c3	2E-11	0.2												
Cwc22	2E-03	0.1	Nfat5	9E-06	0.1													Parva	2E-07	0.2	B3galt1	1E-15	0.2												
Ilkap	7E-04	0.1	Rab2b	2E-02	0.1													Zfp410	3E-09	0.2	Zkscan17	3E-10	0.2												
Dcaf12	6E-04	0.1	Dpy19l4	8E-04	0.1													Parp11	2E-06	0.2	Fbxw8	5E-05	0.2												
Samd4b	2E-03	0.1	Epc1	9E-04	0.1													Twf1	8E-08	0.2	Tmem164	5E-10	0.2												
Snrnp70	5E-06	0.1	Ccdc28a	1E-03	0.1													Ehd1	5E-08	0.2	Uggt1	6E-09	0.2												
Ino80d	1E-04	0.1	Sf3b6	5E-07	0.1													Klf7	6E-06	0.2	Wdr70	3E-07	0.2												
Rraga	7E-06	0.1	Arhgef1	7E-04	0.1													Ltbp2	9E-07	0.2	Tubg2	9E-04	0.2												
Scaf11	1E-06	0.1	Fxyd5	6E-07	0.1													Aven	2E-07	0.2	Osbpl1a	1E-10	0.2												
Pex5	7E-04	0.1	AU040320	1E-04	0.1													Zfp511	8E-04	0.2	Arpc1b	3E-09	0.2												
Phf5a	2E-05	0.1	Ptpn11	1E-03	0.1													Cisd3	2E-08	0.2	Kbtbd4	2E-10	0.2												
Ppil3	1E-04	0.1	Acvr1	5E-04	0.1													Sart1	7E-08	0.2	Phf2	1E-06	0.2												
Mageh1	7E-03	0.1	Chd8	3E-03	0.1													Pbrm1	2E-11	0.2	Hmga2	2E-06	0.2												
Hoxc5	1E-04	0.1	Sf3b3	2E-03	0.1													Adprh	5E-05	0.2	Ahsa2	7E-07	0.2												
Calm2	8E-16	0.1	Gfod2	4E-03	0.1													Ywhag	1E-09	0.2	Ddx51	7E-08	0.2												
Aftph	3E-04	0.1	Pla2g6	8E-02	0.1													Plcxd2	6E-05	0.2	Atg3	1E-08	0.2												
Foxj3	5E-04	0.1	Ankra2	3E-04	0.1													Exoc8	9E-08	0.2	Stk26	7E-12	0.2												
Map2k7	2E-04	0.1	Esrra	2E-03	0.1													Rnf4	4E-07	0.2	Pgam5	2E-10	0.2												
Cdc26	8E-06	0.1	Dnaaf5	4E-02	0.1													Ptp4a3	1E-06	0.2	Adra1b	1E-09	0.2												
Sub1	6E-06	0.1	Dus2	2E-03	0.1													Clcn7	2E-08	0.2	Prcc	2E-06	0.2												
Scaf1	3E-04	0.1	Psmc6	2E-07	0.1													Pfdn1	6E-07	0.2	Tmem248	6E-07	0.2												
Fam168b	1E-03	0.1	Spg7	3E-02	0.1													Itga10	4E-07	0.2	Dtnbp1	2E-07	0.2												
Twnk	9E-03	0.1	Rab8b	2E-05	0.1													Ep400	4E-10	0.2	Commd8	2E-07	0.2												
Chpf	2E-03	0.1	Parl	1E-03	0.1													BC005561	5E-06	0.2	Tma7	5E-24	0.2												
Micu2	8E-06	0.1	Fam204a	5E-04	0.1													Psmc1	1E-07	0.2	Cdr1os	6E-08	0.2												
Eefsec	1E-04	0.1	Actn4	1E-03	0.1													Slc27a4	2E-07	0.2	Olfm2	4E-06	0.2												
Msto1	2E-03	0.1	Rnf5	1E-04	0.1													Josd1	6E-08	0.2	Ogdh	2E-08	0.2												
Fbxo3	2E-04	0.1	Tpm4	4E-07	0.1													Zdhhc5	9E-06	0.2	Zfp207	8E-07	0.2												
Pex10	1E-02	0.1	Gnai2	4E-09	0.1													Mier1	1E-10	0.2	Thoc1	8E-09	0.2												
Psmb6	1E-07	0.1	Hoxa3	7E-03	0.1													Cgref1	1E-07	0.2	Ufd1	2E-06	0.2												
Ppig	2E-06	0.1	Gm12258	1E-01	0.1													Ptprg	4E-06	0.2	Fam120a	4E-08	0.2												
Tmem63b	4E-03	0.1	Eif3j1	2E-05	0.1													Pwp1	6E-08	0.2	Uqcc2	2E-16	0.2												
Pick1	4E-03	0.1	Cstf2t	3E-02	0.1													Rpn1	9E-09	0.2	Uimc1	6E-07	0.2												
Rpl4	2E-13	0.1	Srrt	1E-03	0.1													Phf20l1	3E-08	0.2	Thrap3	7E-09	0.2												
Adprm	4E-03	0.1	Phf14	8E-06	0.1													1700007L15Rik	5E-06	0.2	Gnpnat1	5E-07	0.2												
Map11	3E-02	0.1	Mnt	5E-04	0.1													R3hcc1	3E-04	0.2	Eif4g2	1E-19	0.2												
Zcchc3	4E-03	0.1	Utp14b	3E-02	0.1													Avpi1	4E-07	0.2	Clk3	9E-08	0.2												
Ssbp1	1E-05	0.1	Psmc5	2E-05	0.1													Ftsj1	6E-09	0.2	Wnk1	4E-10	0.2												
Mrpl21	1E-05	0.1	Lrrc8d	2E-03	0.1													Fnbp4	2E-07	0.2	Sorbs1	3E-14	0.2												
A930015D03Rik	2E-03	0.1	Pithd1	8E-04	0.1													Slc7a3	3E-06	0.2	Psmc6	2E-10	0.2												
Gtf3c6	3E-03	0.1	Chmp4b	9E-06	0.1													Srsf9	1E-08	0.2	Sh3gl1	1E-06	0.2												
Cops7a	2E-04	0.1	Usp37	4E-04	0.1													Mak16	3E-06	0.2	Psma4	7E-18	0.2												
Trappc9	1E-03	0.1	Erlin2	1E-02	0.1													Ccar1	8E-08	0.2	Rab42	2E-07	0.2												
Fam92a	1E-05	0.1	Ranbp10	6E-03	0.1													Psmd1	2E-10	0.2	Usp48	3E-10	0.2												
Parn	5E-04	0.1	Tnpo3	9E-05	0.1													Gtf2e2	1E-07	0.2	C330018D20Rik	2E-10	0.2												
Arid2	4E-04	0.1	Hnrnpa2b1	2E-09	0.1													Man1b1	9E-09	0.2	Zfp469	9E-08	0.2												
Scoc	3E-06	0.1	Ssb	7E-09	0.1													Eif3j1	6E-07	0.2	Galnt3	1E-05	0.2												
Slc46a1	1E-03	0.1	Zfp90	4E-03	0.1													Impad1	2E-07	0.2	Gcat	1E-09	0.2												
Atg10	5E-04	0.1	Zcrb1	2E-06	0.1													Bdp1	1E-04	0.2	Osbp	4E-07	0.2												
Yif1a	1E-04	0.1	Paxx	9E-04	0.1													Ubqln1	6E-05	0.2	Atp5g2	7E-21	0.2												
Plxna1	6E-05	0.1	Aldh6a1	8E-05	0.1													Arhgef40	8E-08	0.2	Lgals9	5E-11	0.2												
Rnf214	1E-03	0.1	Ankrd49	7E-05	0.1													Nupl2	7E-08	0.2	Hsd17b12	7E-11	0.2												
Rnf103	4E-04	0.1	Fam219b	4E-03	0.1													C2cd5	1E-07	0.2	Gm15987	1E-11	0.2												
Ndfip2	2E-04	0.1	Cct5	1E-05	0.1													Kctd17	2E-07	0.2	Chtop	7E-08	0.2												
Hypk	2E-05	0.1	H6pd	4E-03	0.1													Mrps15	3E-08	0.2	Cfdp1	1E-11	0.2												
Pdcd6ip	2E-04	0.1	Numa1	3E-03	0.1													Ranbp3	9E-09	0.2	Mapk1	1E-09	0.2												
Snrnp27	3E-06	0.1	Pigk	1E-04	0.1													Zfp871	1E-06	0.2	Vdac2	1E-09	0.2												
Pdcd2	5E-04	0.1	Dync1h1	5E-05	0.1													Scrn3	7E-09	0.2	Nuak2	3E-14	0.2												
Nedd4l	2E-03	0.1	Lin7c	6E-03	0.1													Tollip	1E-03	0.2	Plekhb2	2E-09	0.2												
4930581F22Rik	5E-03	0.1	Lxn	5E-06	0.1													Pop7	2E-05	0.2	Adam10	4E-11	0.2												
Tmem120a	2E-08	0.1	Acan	6E-10	0.1													Tor1b	8E-08	0.2	Camsap1	4E-09	0.2												
Cc2d1b	5E-03	0.1	Ddx17	1E-05	0.1													Entr1	7E-09	0.2	Ppp1r11	1E-09	0.2												
Trip4	1E-03	0.1	Haus2	1E-02	0.1													Spred2	4E-08	0.2	R3hdm1	4E-09	0.2												
Fbxw17	7E-04	0.1	Mrps5	4E-04	0.1													Rnf6	4E-04	0.2	Anxa1	3E-14	0.2												
Celf4	1E-03	0.1	Afg1l	2E-03	0.1													Isyna1	1E-08	0.2	Nomo1	2E-10	0.2												
Mydgf	5E-06	0.1	Lias	2E-02	0.1													Mta2	5E-08	0.2	Snrnp35	9E-11	0.2												
Yeats2	2E-03	0.1	Shq1	1E-01	0.1													Prpf40a	1E-09	0.2	Pbrm1	5E-12	0.2												

Limb Mesenchyme

Control			NICD2		
Gene	*p*	FC	Gene	*p*	FC
Lrfn3	2E-03	0.1	Ppp1r37	3E-03	0.1
Pkd1	2E-04	0.1	Selenof	1E-12	0.1
Eno1	1E-07	0.1	Kat6a	6E-04	0.1
Nanos1	2E-02	0.1	Yae1d1	2E-03	0.1
Ing5	3E-03	0.1	Zfp512	2E-02	0.1
Nvl	3E-03	0.1	Itsn2	1E-02	0.1
Ipo4	6E-04	0.1	Fbxl12	4E-03	0.1
Ppp2r3a	2E-03	0.1	Usp20	1E-02	0.1
Col27a1	4E-08	0.1	Podn	8E-03	0.1
Usf3	1E-03	0.1	Atg2a	2E-03	0.1
Rabgap1	2E-06	0.1	Fam104a	1E-06	0.1
Zfp74	4E-04	0.1	Gatd3a	1E-04	0.1
Miga2	7E-03	0.1	Knop1	6E-05	0.1
Zfp251	1E-02	0.1	Dmxl1	9E-04	0.1
Trmu	2E-03	0.1	Ube2r2	1E-06	0.1
Sestd1	1E-03	0.1	Pdcd11	4E-03	0.1
Mrpl32	3E-04	0.1	Orc3	3E-05	0.1
Sin3a	2E-04	0.1	Katnbl1	3E-03	0.1
Psmc3	8E-05	0.1	Atf1	6E-03	0.1
Pced1a	2E-03	0.1	Atp6v1a	1E-04	0.1
Erh	7E-06	0.1	Spred1	6E-06	0.1
Poldip3	5E-03	0.1	Nudcd2	6E-05	0.1
Cnih1	2E-04	0.1	Hinfp	5E-02	0.1
Lrrc14	5E-03	0.1	Nisch	2E-07	0.1
Samd14	9E-03	0.1	Kifap3	7E-04	0.1
Chmp1b	3E-05	0.1	Enho	1E-04	0.1
Mfsd5	8E-04	0.1	Naa15	1E-04	0.1
Smad2	1E-04	0.1	B3gnt2	2E-03	0.1
Bod1	2E-04	0.1	Scfd2	4E-03	0.1
Gorab	1E-03	0.1	Lancl1	4E-03	0.1
Pfn1	3E-08	0.1	Sik1	8E-03	0.1
Gm4258	7E-03	0.1	Sart3	4E-03	0.1
Pdik1l	3E-03	0.1	Zc3h12b	6E-04	0.1
Sh3bgrl	6E-05	0.1	Hspa1b	9E-03	0.1
Tmem183a	2E-03	0.1	Thoc5	5E-04	0.1
Chchd1	6E-07	0.1	Rnf10	7E-06	0.1
Ngly1	2E-03	0.1	Ankmy2	2E-03	0.1
Dhx16	3E-03	0.1	Ik	2E-04	0.1
Cct7	2E-06	0.1	Actl6a	6E-03	0.1
Cnot11	5E-03	0.1	Pus1	2E-03	0.1
Ergic2	3E-04	0.1	Wdr37	5E-03	0.1
Pofut2	6E-05	0.1	R3hdm1	2E-04	0.1
Rcn2	3E-06	0.1	Rbm27	3E-03	0.1
Coro7	7E-03	0.1	Tex10	4E-03	0.1
Mark2	2E-04	0.1	Pdia4	9E-07	0.1
Scai	2E-04	0.1	Nbea	2E-05	0.1
Smim10l1	3E-06	0.1	Dpm2	2E-04	0.1
Tfdp2	8E-04	0.1	Cldn12	4E-04	0.1
Ubap2	2E-04	0.1	Nudt3	3E-04	0.1
Amz2	3E-03	0.1	Mir99ahg	2E-05	0.1
Kars	2E-03	0.1	Arl6ip6	3E-04	0.1
Vac14	4E-03	0.1	Sbf2	7E-04	0.1
Zfp790	3E-03	0.1	Snip1	3E-03	0.1
Srp19	2E-07	0.1	Pcdhb17	1E-02	0.1
Epg5	3E-03	0.1	Clip1	9E-05	0.1
Dnajb9	2E-04	0.1	Lsm8	3E-06	0.1
Acot8	3E-03	0.1	Las1l	2E-03	0.1
Rala	1E-05	0.1	Sh3bgrl	1E-05	0.1
Smad1	8E-05	0.1	Rab21	7E-06	0.1
Crnkl1	3E-04	0.1	Rab31	1E-03	0.1
Ccdc124	2E-04	0.1	Yars2	3E-02	0.1
Huwe1	5E-06	0.1	Crkl	1E-02	0.1
Nabp1	3E-06	0.1	Brat1	5E-03	0.1
Gpaa1	4E-05	0.1	Stk11	2E-02	0.1
Cul4a	6E-03	0.1	Ofd1	5E-03	0.1
Bag5	1E-04	0.1	Jmjd6	5E-04	0.1
Dclre1a	8E-03	0.1	Chd4	5E-06	0.1
Kpnb1	1E-03	0.1	Mtrex	3E-04	0.1
Nbeal1	6E-05	0.1	Zfp369	4E-03	0.1
Tmem216	4E-04	0.1	Timm44	2E-03	0.1
Zcchc10	7E-04	0.1	Tmem258	9E-06	0.1
Ube2a	2E-04	0.1	Hspe1-rs1	1E-02	0.1
Zfp830	1E-03	0.1	Rcan3	6E-03	0.1

Chondrogenic

Control			NICD2		
Gene	*p*	FC	Gene	*p*	FC

Fibroblast

Control			NICD2		
Gene	*p*	FC	Gene	*p*	FC

Fibroblast?

Control			NICD2		
Gene	*p*	FC	Gene	*p*	FC
Atic	5E-06	0.2	Rala	3E-07	0.2
Scaf4	1E-06	0.2	Ap1s2	8E-13	0.2
Hmgn3	1E-07	0.2	Rwdd4a	5E-09	0.2
Unc5c	5E-05	0.2	Ttc39b	3E-09	0.2
Nln	5E-09	0.2	Mbd1	6E-10	0.2
Rgmb	2E-09	0.2	Zfp451	2E-07	0.2
Prpf19	2E-08	0.2	Gtf2h4	3E-09	0.2
Rtl3	3E-09	0.2	Ccdc59	2E-06	0.2
Snx5	5E-09	0.2	Uqcrb	2E-12	0.2
Gm16599	8E-07	0.2	Tgs1	7E-11	0.2
Gtf2h4	2E-07	0.2	Mettl6	2E-10	0.2
Rap1a	1E-09	0.2	Gng10	2E-07	0.2
Mblac1	4E-05	0.2	Smg9	3E-09	0.2
Gon4l	8E-04	0.2	Gm11508	2E-07	0.2
Dennd5a	3E-05	0.2	Ensa	4E-07	0.2
Isg20l2	3E-09	0.2	Prkcd	1E-07	0.2
Ly6c1	3E-14	0.2	Zfp777	1E-09	0.2
Ilkap	7E-07	0.2	Cxcl14	3E-10	0.2
Mogs	6E-10	0.2	Ppm1b	1E-09	0.2
Utrn	5E-06	0.2	Ppie	2E-09	0.2
Uqcrfs1	4E-04	0.2	Med16	1E-10	0.2
Uba1	6E-08	0.2	Tufm	1E-05	0.2
Hip1	7E-10	0.2	Zfp787	1E-09	0.2
Nxpe3	1E-05	0.2	Chl1	3E-09	0.2
Bloc1s4	3E-08	0.2	Srgap2	5E-10	0.2
Alms1	8E-15	0.2	Wdcp	1E-08	0.2
Nckap1	6E-09	0.2	Rictor	3E-06	0.2
D330050G23Rik	1E-03	0.2	Itpr1	2E-09	0.2
Stoml2	2E-08	0.2	Metap1	1E-08	0.2
Morc3	1E-06	0.2	Faf1	8E-07	0.2
Acsf2	6E-07	0.2	Rnf113a2	1E-06	0.2
Foxk2	2E-07	0.2	Kdelr2	2E-13	0.2
Atpif1	8E-16	0.2	Arl13b	2E-09	0.2
Capza2	3E-10	0.2	Dctn1	2E-07	0.2
Ndufb6	6E-07	0.2	Fem1a	5E-07	0.2
Adam10	6E-07	0.2	Cmpk1	5E-11	0.2
Atg101	6E-07	0.2	Synpo	1E-11	0.2
Itgav	8E-06	0.2	Atoh8	7E-07	0.2
Gna13	2E-08	0.2	Cpne2	9E-11	0.2
Tsn	5E-13	0.2	Capn15	1E-07	0.2
Shisa2	2E-04	0.2	BC005561	1E-05	0.2
Ciapin1	1E-06	0.2	Fads3	3E-09	0.2
Prdm10	6E-08	0.2	Sp1	2E-08	0.2
Cab39	1E-06	0.2	Bcl2l1	2E-08	0.2
Slc35b2	2E-06	0.2	Trerf1	3E-09	0.2
Atf5	4E-04	0.2	Plpp2	4E-09	0.2
Fem1b	2E-07	0.2	Fbxo21	1E-07	0.2
Cdc42se2	1E-08	0.2	Card19	4E-06	0.2
Grtp1	4E-05	0.2	Rdh13	1E-08	0.2
Emp3	3E-15	0.2	Ctu2	4E-09	0.2
Snrnp200	2E-05	0.2	Sap30l	8E-08	0.2
Ubp1	2E-06	0.2	Isca2	4E-03	0.2
Arhgap21	2E-10	0.2	Psmd12	2E-07	0.2
Aspscr1	1E-06	0.2	Pcdh18	9E-07	0.2
Seh1l	6E-09	0.2	Ate1	1E-06	0.2
Epb41l3	1E-07	0.2	Gng12	6E-10	0.2
Camkk2	3E-09	0.2	Larp1	4E-08	0.2
Rint1	1E-07	0.2	Gpkow	3E-10	0.2
Ssu72	3E-04	0.2	Heatr5a	2E-06	0.2
Kif5c	9E-05	0.2	Yeats2	1E-11	0.2
Wdr70	8E-06	0.2	Acp6	1E-08	0.2
Fam171a1	3E-09	0.2	Ywhab	7E-13	0.2
Dolpp1	4E-08	0.2	Ints3	3E-12	0.2
Mgp	2E-05	0.2	Anapc2	3E-07	0.2
Sdf2l1	1E-07	0.2	Heatr6	7E-08	0.2
Tex261	3E-07	0.2	Gcnt1	1E-09	0.2
Gm14418	4E-03	0.2	Znrd1	3E-03	0.2
Zc3h15	2E-11	0.2	Myl6	3E-15	0.2
Zfp932	3E-08	0.2	Agap1	3E-10	0.2
Ap1ar	4E-07	0.2	Zfp707	2E-07	0.2
Akap12	6E-07	0.2	Bhlhb9	4E-08	0.2
Timm9	2E-06	0.2	Nxn	7E-14	0.2
Apex2	2E-08	0.2	Nrg1	2E-16	0.2

Undefined

Control			NICD2		
Gene	*p*	FC	Gene	*p*	FC

Articular/Synovial Fibroblast

Control			NICD2		
Gene	*p*	FC	Gene	*p*	FC

Limb Mesenchyme					
Control			NICD2		
Gene	*p*	FC	Gene	*p*	FC
Csgalnact2	2E-04	0.1	Fem1b	2E-04	0.1
Zfp592	1E-03	0.1	Katnb1	4E-02	0.1
Fip1l1	6E-05	0.1	Tmem30a	2E-04	0.1
Pcnx3	1E-03	0.1	Fam149b	5E-04	0.1
Nim1k	2E-02	0.1	Tmem104	6E-03	0.1
Fgd1	6E-04	0.1	Gart	1E-03	0.1
Timm9	2E-03	0.1	Abhd14a	1E-03	0.1
Chpf2	2E-03	0.1	Actr8	2E-03	0.1
Nup214	7E-04	0.1	Adcy9	1E-02	0.1
Hddc2	4E-05	0.1	Tmx2	1E-03	0.1
Tars2	6E-04	0.1	S100a10	9E-07	0.1
Gsk3a	3E-03	0.1	Ptcd3	2E-03	0.1
Osbpl10	4E-02	0.1	Dnajc27	7E-02	0.1
Polr2k	5E-05	0.1	Rabep1	4E-04	0.1
Ube2h	4E-06	0.1	Clpx	2E-03	0.1
Btbd9	3E-04	0.1	Slc7a6	1E-01	0.1
Zfp358	2E-04	0.1	Tbce	2E-03	0.1
Nfyc	5E-04	0.1	Ap1ar	4E-04	0.1
Trim33	3E-04	0.1	Pfdn1	3E-07	0.1
Rasa2	6E-04	0.1	Tmcc1	1E-02	0.1
Ddx19b	5E-03	0.1	Ipo4	2E-04	0.1
Ppp2r2a	1E-04	0.1	Ano10	4E-03	0.1
Sdhb	2E-07	0.1	Tpm3	5E-08	0.1
Htra1	2E-04	0.1	Hsp90b1	2E-13	0.1
Wdr44	4E-02	0.1	Arl2	1E-04	0.1
Tmem230	3E-05	0.1	Nynrin	5E-03	0.1
Gnpat	9E-05	0.1	Stk16	2E-03	0.1
Runx1t1	1E-05	0.1	Gm39090	5E-02	0.1
Pex12	7E-03	0.1	Cab39	4E-03	0.1
Pcgf6	2E-03	0.1	Crebbp	1E-04	0.1
Cggbp1	3E-05	0.1	Grsf1	2E-03	0.1
Phf8	6E-03	0.1	Smad7	3E-04	0.1
Fxr1	9E-06	0.1	C1qtnf6	1E-04	0.1
Mettl26	4E-05	0.1	Klf8	5E-03	0.1
Ccdc112	3E-04	0.1	Trp53	2E-04	0.1
Akr1a1	7E-11	0.1	Atf5	1E-07	0.1
Gcnt1	3E-03	0.1	Dcaf8	4E-04	0.1
Usp46	1E-04	0.1	Gfm1	1E-03	0.1
Smg6	4E-04	0.1	Pgrmc1	2E-04	0.1
Agtpbp1	4E-03	0.1	Kpna4	4E-04	0.1
Mrps5	7E-03	0.1	Epn1	6E-04	0.1
Txnl1	3E-06	0.1	Mrpl38	1E-02	0.1
Gnai2	2E-09	0.1	Ufl1	1E-03	0.1
Rexo2	8E-08	0.1	Baz2b	2E-04	0.1
Rusc1	3E-02	0.1	2610507B11Rik	9E-04	0.1
Ascc1	2E-03	0.1	Mcrip1	4E-05	0.1
Msantd2	2E-04	0.1	Trmt2a	1E-02	0.1
Smim14	2E-06	0.1	Tma7	2E-10	0.1
Shprh	4E-04	0.1	Mipol1	5E-04	0.1
Dnajc21	2E-03	0.1	Ftsj1	3E-02	0.1
Hnrnpm	8E-05	0.1	Mllt10	4E-03	0.1
Kdm2a	5E-04	0.1	Ryk	4E-05	0.1
Atl1	9E-03	0.1	Uba6	6E-03	0.1
Tor1aip2	6E-04	0.1	Itm2c	2E-05	0.1
Zfp566	4E-03	0.1	Rnf31	4E-02	0.1
Dhrs13	2E-02	0.1	Tmem126a	1E-04	0.1
Zfp287	1E-02	0.1	Ddi2	1E-02	0.1
Arih2	3E-03	0.1	Ldlrad4	7E-03	0.1
Magoh	6E-06	0.1	Vps26c	4E-03	0.1
AI837181	4E-04	0.1	Tor1a	4E-03	0.1
Sh3bp5l	5E-03	0.1	Acin1	1E-05	0.1
Tmem208	1E-04	0.1	Mtmr3	1E-04	0.1
Osgepl1	2E-02	0.1	Pex2	5E-04	0.1
Zbtb20	8E-08	0.1	Nf1	4E-04	0.1
A930007I19Rik	7E-04	0.1	Tada2b	6E-03	0.1
Bcr	2E-03	0.1	Trappc8	4E-03	0.1
Clstn1	4E-04	0.1	Xiap	7E-04	0.1
Ncs1	2E-03	0.1	Msantd4	3E-03	0.1
R3hdm2	4E-05	0.1	Gm20045	3E-02	0.1
Tbce	1E-03	0.1	Ric1	4E-03	0.1
Sucla2	1E-05	0.1	Prmt2	2E-03	0.1
Pnkp	2E-04	0.1	Tcf20	3E-02	0.1
Cnppd1	8E-04	0.1	Ubac1	2E-04	0.1

Chondrogenic						Fibroblast					
Control			NICD2			Control			NICD2		
Gene	*p*	FC	Gene	*p*	FC	Gene	*p*	FC	Gene	*p*	FC

Fibroblast?					
Control			NICD2		
Gene	*p*	FC	Gene	*p*	FC
Pcbp4	6E-09	0.2	Arhgef7	8E-09	0.2
Zmpste24	2E-07	0.2	Fez1	3E-09	0.2
Maoa	3E-06	0.2	Vamp8	3E-08	0.2
Mast3	6E-10	0.2	Stk16	1E-07	0.2
Xxylt1	6E-09	0.2	9130401M01Rik	2E-06	0.2
Pdlim7	8E-04	0.2	a	2E-06	0.2
Afap1	7E-07	0.2	Gmds	1E-06	0.2
Anxa5	4E-14	0.2	Pdgfc	4E-14	0.2
Psma4	3E-11	0.2	Mapk13	4E-06	0.2
Rai14	8E-06	0.2	Sipa1l2	1E-11	0.2
Commd10	2E-07	0.2	C2cd5	2E-12	0.2
1700023H06Rik	7E-04	0.2	Trpt1	1E-06	0.2
Cnp	6E-12	0.2	Mtx2	3E-05	0.2
Arhgap6	4E-12	0.2	Ahcyl1	1E-07	0.2
Scara3	3E-10	0.2	Eif5	5E-13	0.2
Phf11d	2E-12	0.2	Ublcp1	8E-05	0.2
Qsox2	1E-08	0.2	Vgll3	2E-08	0.2
Prrc2c	2E-10	0.2	Pmp22	8E-12	0.2
Sqor	4E-05	0.2	Fap	4E-12	0.2
Ppfibp1	1E-07	0.2	Zfp518b	6E-08	0.2
Ralgps1	9E-06	0.2	Tmem267	6E-07	0.2
Ttc32	7E-10	0.2	Tram2	1E-07	0.2
Atp1a1	7E-07	0.2	Ufsp1	5E-05	0.2
Spag1	2E-04	0.2	Yars2	1E-08	0.2
St3gal2	3E-07	0.2	4930523C07Rik	2E-08	0.2
Inpp5b	8E-09	0.2	S100a13	2E-06	0.2
Sap30l	1E-05	0.2	Olfm1	5E-11	0.2
Metap2	6E-10	0.2	Trim24	2E-07	0.2
Ube2o	8E-04	0.2	Msl3l2	2E-09	0.2
Wdr37	8E-07	0.2	Abca7	1E-06	0.2
Mtx1	6E-05	0.2	Srf	2E-08	0.2
Spink10	5E-05	0.2	Slc25a15	1E-07	0.2
Rnf169	6E-05	0.2	Ptcd3	3E-09	0.2
Pip5k1c	1E-06	0.2	Mrpl21	2E-07	0.2
Ufsp1	4E-04	0.2	Stambpl1	8E-07	0.2
Arhgap31	1E-08	0.2	Ocel1	3E-05	0.2
Wsb1	6E-07	0.2	Msx1	1E-05	0.2
Tiprl	9E-04	0.2	Zfp105	2E-04	0.2
Zswim3	1E-03	0.2	Nars	2E-05	0.2
Grpel1	1E-09	0.2	Crk	6E-07	0.2
Pon2	2E-05	0.2	Utrn	1E-10	0.2
Eif4g2	8E-15	0.2	Tfrc	5E-14	0.2
Tmem234	2E-12	0.2	Clasrp	4E-09	0.2
Numa1	2E-06	0.2	Umad1	9E-09	0.2
Vim	9E-11	0.2	Cdkn2aip	1E-06	0.2
Atad3a	5E-06	0.2	Usp42	7E-08	0.2
4930430F08Rik	3E-09	0.2	Garnl3	6E-10	0.2
Samd5	1E-06	0.2	Mta1	2E-08	0.2
Gid8	6E-09	0.2	Mpdz	3E-08	0.2
Polr2e	6E-08	0.2	Minpp1	1E-08	0.2
A430005L14Rik	2E-05	0.2	Pigu	6E-07	0.2
Gemin5	9E-06	0.2	Pias3	4E-08	0.2
H3f3a	8E-13	0.2	Baiap2	7E-08	0.2
Cbr2	3E-06	0.2	Sec22c	3E-06	0.2
Mbd2	3E-08	0.2	Akap12	9E-05	0.2
Slc25a19	8E-06	0.2	Trappc12	1E-09	0.2
Smarce1	9E-07	0.2	Rela	1E-10	0.2
Unc119b	1E-06	0.2	Ammecr1	5E-08	0.2
Yy1	2E-04	0.2	Efnb1	7E-07	0.2
Tcf20	1E-06	0.2	Qpct	3E-06	0.2
Cdc16	6E-06	0.2	Bap1	9E-10	0.2
Fam160a2	3E-06	0.2	Naa20	7E-06	0.2
Igf1r	2E-06	0.2	Smoc2	3E-09	0.2
Mif	2E-07	0.2	Wdr5b	9E-09	0.2
Nnat	2E-02	0.2	Dennd6a	8E-04	0.2
Gm15446	9E-04	0.2	Mcat	2E-05	0.2
Ankrd52	2E-08	0.2	Spats2	4E-05	0.2
Spire1	3E-09	0.2	Zdhhc8	9E-10	0.2
Dlk1	1E-07	0.2	Mrps31	2E-04	0.2
Atp5g1	2E-09	0.2	Tpp2	6E-05	0.2
Commd8	2E-06	0.2	Pcm1	2E-07	0.2
Dnajc25	2E-06	0.2	Hspg2	1E-08	0.2
6030458C11Rik	4E-07	0.2	Kctd2	6E-05	0.2

Undefined						Articular/Synovial Fibroblast					
Control			NICD2			Control			NICD2		
Gene	*p*	FC	Gene	*p*	FC	Gene	*p*	FC	Gene	*p*	FC

Limb Mesenchyme					
Control			NICD2		
Gene	*p*	FC	Gene	*p*	FC
Pole4	6E-04	0.1	Stk39	3E-03	0.1
Mrps2	9E-03	0.1	Ubap1	3E-03	0.1
Tlk1	7E-05	0.1	Ano8	1E-02	0.1
Slc39a6	1E-03	0.1	Dnajc3	5E-05	0.1
Greb1l	2E-02	0.1	Sfpq	1E-04	0.1
Fam3a	1E-03	0.1	Ltn1	2E-04	0.1
Nsun2	1E-04	0.1	Phax	3E-03	0.1
Abl1	2E-04	0.1	Shprh	3E-02	0.1
Slc30a7	3E-04	0.1	Poc5	2E-02	0.1
Vasn	1E-03	0.1	Borcs6	1E-03	0.1
Napg	8E-04	0.1	Ccdc66	1E-03	0.1
Mapk6	1E-05	0.1	Fbxo38	3E-03	0.1
Cct5	8E-05	0.1	Glo1	6E-05	0.1
Slc35e2	1E-03	0.1	Il18	3E-04	0.1
Prelid3b	4E-03	0.1	Bmp2k	7E-03	0.1
Sec61g	7E-10	0.1	Pgam1	4E-07	0.1
Prrc2c	3E-06	0.1	Usp48	2E-03	0.1
Tulp2	7E-03	0.1	Sugp2	6E-02	0.1
Ift88	2E-03	0.1	2200002D01Rik	1E-03	0.1
Zfp869	1E-02	0.1	Akirin1	7E-03	0.1
Aifm2	1E-02	0.1	A930037H05Rik	5E-02	0.1
Ash1l	1E-04	0.1	Lmbrd2	6E-03	0.1
Ube2e3	5E-06	0.1	Lactb	1E-02	0.1
Camta2	1E-02	0.1	Nop56	6E-05	0.1
Thoc7	1E-05	0.1	Ighmbp2	5E-02	0.1
Yme1l1	1E-04	0.1	Atp6v1g1	1E-08	0.1
Iqgap1	6E-05	0.1	Tbrg4	1E-02	0.1
Ndufb9	8E-14	0.1	Erf	1E-02	0.1
Rxylt1	7E-05	0.1	Szrd1	2E-03	0.1
Helz	1E-04	0.1	Pign	2E-03	0.1
Mtf2	9E-04	0.1	Spata5	7E-03	0.1
Preb	1E-04	0.1	Ndufs4	6E-05	0.1
Nr1d2	2E-03	0.1	Focad	1E-03	0.1
Mafk	5E-02	0.1	Med15	6E-03	0.1
4632427E13Rik	7E-05	0.1	Tomm40	4E-04	0.1
Aifm1	2E-03	0.1	Rbm14	1E-02	0.1
Zfp706	2E-06	0.1	Rab5if	2E-07	0.1
Arl2	1E-04	0.1	Map2k2	2E-05	0.1
Bzw1	4E-07	0.1	Gm5165	3E-02	0.1
Lnpep	8E-04	0.1	Pnpt1	2E-02	0.1
Vars2	2E-02	0.1	Rpgrip1	1E-01	0.1
Katnal1	8E-03	0.1	Gnai3	2E-03	0.1
Cgrrf1	8E-03	0.1	Leng1	2E-03	0.1
Aph1b	2E-03	0.1	Zfp169	6E-02	0.1
Tsn	8E-06	0.1	Ptges3	1E-07	0.1
Cyth2	2E-03	0.1	Sf3a1	7E-04	0.1
Mfap3	3E-03	0.1	Gm15270	1E-02	0.1
Timm22	7E-04	0.1	Rnf41	1E-03	0.1
Rpusd3	1E-01	0.1	Atp11a	4E-03	0.1
Cnpy2	2E-10	0.1	Sucla2	3E-04	0.1
Zc3hc1	5E-03	0.1	Tmem170b	2E-01	0.1
Slc25a11	3E-04	0.1	Ntn3	6E-03	0.1
Psmd14	4E-04	0.1	Mtpn	4E-05	0.1
Arid4b	8E-05	0.1	Pcdhga5	2E-02	0.1
Tcof1	2E-04	0.1	Smim10l1	3E-06	0.1
Ruvbl2	9E-05	0.1	Btbd9	5E-03	0.1
Rexo1	3E-03	0.1	Scap	9E-03	0.1
Alg9	1E-02	0.1	Ankrd13c	1E-04	0.1
Tox4	1E-03	0.1	Fbxo10	1E-02	0.1
Prmt6	1E-02	0.1	Igsf3	2E-02	0.1
Mapk1	2E-04	0.1	Laptm4a	5E-10	0.1
Zfand2a	2E-02	0.1	Ccdc174	3E-04	0.1
Gm49539	1E-02	0.1	E2f4	2E-02	0.1
Rbm39	8E-10	0.1	Nsrp1	3E-04	0.1
Phf23	1E-03	0.1	Phkb	7E-03	0.1
Pycr2	1E-03	0.1	Gm11944	2E-02	0.1
Ddr1	9E-05	0.1	Dhodh	7E-02	0.1
Ints9	2E-03	0.1	Cops7b	2E-04	0.1
Tbc1d9	3E-03	0.1	Trmt6	5E-03	0.1
Alkbh8	3E-03	0.1	Nuak2	3E-01	0.1
Calcoco1	1E-04	0.1	Senp7	4E-04	0.1
Arid1b	3E-04	0.1	Pex11g	4E-03	0.1
Gm49179	4E-03	0.1	H1f0	4E-04	0.1

Chondrogenic					
Control			NICD2		
Gene	*p*	FC	Gene	*p*	FC

Fibroblast					
Control			NICD2		
Gene	*p*	FC	Gene	*p*	FC

Fibroblast?					
Control			NICD2		
Gene	*p*	FC	Gene	*p*	FC
Pomp	2E-11	0.2	Pign	1E-06	0.2
Zfp143	4E-07	0.2	Med6	9E-09	0.2
Phb	5E-03	0.2	Gemin7	2E-06	0.2
Nfyc	1E-07	0.2	Sf3b3	8E-08	0.2
Caap1	2E-06	0.2	Tor1b	2E-07	0.2
Emc3	2E-07	0.2	Adnp2	8E-12	0.2
Ubtd1	3E-06	0.2	Shkbp1	6E-03	0.2
Eif3a	6E-09	0.2	Mrps14	4E-06	0.2
Add3	5E-10	0.2	Ninl	1E-09	0.2
Zmat3	7E-07	0.2	Tcea1	4E-09	0.2
Gstp1	6E-02	0.2	Clns1a	4E-06	0.2
Rnpc3	2E-06	0.2	Snupn	1E-08	0.2
Mrps18c	5E-09	0.2	Glod4	2E-08	0.2
Necap2	1E-04	0.2	Shld2	4E-08	0.2
Mpdz	4E-07	0.2	Adh5	2E-04	0.2
Ank	4E-03	0.2	Mus81	4E-12	0.2
Arid1a	3E-04	0.2	Pdcd6ip	2E-05	0.2
Ublcp1	2E-06	0.2	Mrps2	2E-08	0.2
Eif3c	2E-09	0.2	Sik1	4E-09	0.2
Arpc4	3E-04	0.2	Mfsd11	3E-11	0.2
Fxr2	3E-06	0.2	Zfp982	7E-07	0.2
Abhd8	2E-04	0.2	AC103362.1	5E-04	0.2
Eef1d	5E-10	0.2	Atp23	2E-05	0.2
Tmsb4x	7E-16	0.2	Gm12166	6E-06	0.2
Ifrd1	3E-06	0.2	Osgep	2E-04	0.2
Herc2	1E-06	0.2	Chchd4	7E-09	0.2
Wnk3	3E-04	0.2	Hnrnpr	4E-08	0.2
Tmem256	3E-10	0.2	Ankrd11	1E-09	0.2
Tlcd1	2E-09	0.2	Stimate	3E-09	0.2
Thoc1	1E-04	0.2	Col6a3	3E-05	0.2
Rpsa	2E-18	0.2	Abr	3E-08	0.2
Rassf8	4E-09	0.2	Mapk3	2E-07	0.2
Sgsh	6E-06	0.2	Zc3h12c	4E-09	0.2
Gmeb2	6E-07	0.2	Tle5	4E-10	0.2
Ncor2	4E-04	0.2	Tmem234	2E-12	0.2
Eml3	3E-10	0.2	Vamp3	1E-05	0.2
Ndufab1	5E-10	0.2	4930444A19Rik	2E-08	0.2
Fryl	4E-07	0.2	Syngr2	2E-07	0.2
Gm10658	1E-05	0.2	Tada2a	3E-08	0.2
Cs	8E-08	0.2	Msrb2	4E-04	0.2
Id1	3E-15	0.2	Naa10	6E-08	0.2
Ppcdc	6E-09	0.2	Tsen2	6E-08	0.2
Timm17a	2E-05	0.2	Tor2a	4E-08	0.2
Ppp2cb	4E-04	0.2	Slc7a6os	5E-04	0.2
Rgs17	1E-05	0.2	Ccdc124	7E-03	0.2
Sco1	3E-05	0.2	Rexo2	8E-14	0.2
Apba1	6E-07	0.2	Med11	3E-09	0.2
Minpp1	3E-06	0.2	Smg8	1E-08	0.2
Myl12a	7E-10	0.2	Col4a2	1E-04	0.2
Ndufaf6	4E-09	0.2	Smarcd2	2E-07	0.2
Mkrn2	3E-08	0.2	Gng5	1E-14	0.2
Eif3d	3E-05	0.2	Polr2j	1E-05	0.2
Bambi	2E-05	0.2	Nom1	1E-05	0.2
Cc2d2a	3E-04	0.2	Rsph3b	7E-08	0.2
Borcs5	3E-04	0.2	Hinfp	5E-07	0.1
Pias2	2E-04	0.2	Inpp1	3E-05	0.1
Safb	7E-06	0.2	Samd14	2E-10	0.1
Atp5j	4E-16	0.2	Exoc5	2E-05	0.1
Chd4	5E-09	0.2	Trappc10	7E-07	0.1
Git1	3E-08	0.2	Celf1	9E-07	0.1
Tbrg4	4E-06	0.2	Naa15	4E-05	0.1
Rnaseh1	3E-07	0.2	Dock5	2E-14	0.1
Ccdc12	5E-05	0.2	G3bp2	2E-06	0.1
Kat2a	4E-07	0.2	Traf3	8E-10	0.1
Sh3glb1	8E-12	0.2	Amfr	2E-07	0.1
Mrm3	8E-08	0.2	Mgat4b	2E-08	0.1
Lgals9	2E-07	0.2	Ar	1E-08	0.1
Impdh1	3E-05	0.2	Arhgef10	4E-10	0.1
Fbxw2	3E-04	0.2	Ankrd42	2E-06	0.1
Rps6kb2	2E-07	0.2	Derl2	2E-08	0.1
Rnf113a2	6E-05	0.2	Eif2b1	3E-09	0.1
Oxr1	1E-05	0.2	Wasf1	4E-07	0.1
A730056A06Rik	2E-02	0.2	Atp5b	2E-20	0.1

Undefined					
Control			NICD2		
Gene	*p*	FC	Gene	*p*	FC

Articular/Synovial Fibroblast					
Control			NICD2		
Gene	*p*	FC	Gene	*p*	FC

Limb Mesenchyme						Chondrogenic						Fibroblast						Fibroblast?						Undefined						Articular/Synovial Fibroblast					
Control			NICD2			Control			NICD2			Control			NICD2			Control			NICD2			Control			NICD2			Control			NICD2		
Gene	*p*	FC	Gene	*p*	FC	Gene	*p*	FC	Gene	*p*	FC	Gene	*p*	FC	Gene	*p*	FC	Gene	*p*	FC	Gene	*p*	FC	Gene	*p*	FC	Gene	*p*	FC	Gene	*p*	FC	Gene	*p*	FC
Hist1h3d	5E-02	0.1	N4bp2l2	5E-04	0.1													Smarcad1	1E-06	0.2	Adcy6	6E-07	0.1												
Zfp91	2E-05	0.1	Denr	6E-06	0.1													Prox2	2E-03	0.2	Pinx1	4E-05	0.1												
8430429K09Rik	1E-03	0.1	Tlk1	3E-03	0.1													Gm49179	8E-07	0.2	Eif1b	3E-06	0.1												
Tmem184c	1E-02	0.1	Mrps31	1E-04	0.1													Clasrp	2E-09	0.2	Prpf4b	3E-07	0.1												
Tmem147	2E-05	0.1	Katna1	1E-03	0.1													Pak1ip1	5E-06	0.2	Edf1	2E-14	0.1												
Rpe	7E-03	0.1	Stag2	2E-03	0.1													Pcnp	2E-08	0.2	Ddb1	1E-06	0.1												
Rnf44	2E-04	0.1	Txnl1	2E-08	0.1													Ash2l	3E-07	0.2	Cux1	2E-05	0.1												
Slc25a26	8E-03	0.1	Timm29	4E-03	0.1													Cfdp1	1E-06	0.2	Galnt2	3E-07	0.1												
Mpp7	1E-03	0.1	A930015D03Rik	5E-04	0.1													Rest	8E-07	0.2	Bop1	5E-10	0.1												
2810032G03Rik	8E-03	0.1	Zfp326	2E-03	0.1													Dnm1l	8E-06	0.2	Veph1	5E-10	0.1												
Pik3ca	1E-03	0.1	Atf7	5E-03	0.1													S100a11	9E-12	0.2	Rlim	3E-07	0.1												
Tbc1d10b	6E-03	0.1	Eml1	8E-03	0.1													Atp5g2	7E-15	0.2	Sf3b1	1E-09	0.1												
Timm10b	1E-06	0.1	Tmem241	2E-02	0.1													B4galt7	9E-04	0.2	Snf8	7E-07	0.1												
Eif4e3	4E-05	0.1	Fto	4E-04	0.1													Smad1	5E-08	0.2	Hnrnpc	2E-02	0.1												
Crebbp	4E-05	0.1	Fam91a1	4E-03	0.1													Klf6	6E-06	0.2	Elob	5E-19	0.1												
Trim28	6E-03	0.1	Cyp4f17	2E-01	0.1													Smim15	2E-07	0.2	Sept2	3E-06	0.1												
Dennd1b	7E-04	0.1	Zup1	2E-03	0.1													Asphd2	4E-05	0.2	Pdcl3	9E-04	0.1												
Ap2b1	3E-04	0.1	Gpr108	2E-03	0.1													Fah	2E-08	0.2	Tpmt	2E-12	0.1												
Phc1	2E-02	0.1	Nfic	4E-05	0.1													Fblim1	8E-04	0.2	Vps50	6E-05	0.1												
Unc119	5E-03	0.1	Ccnk	7E-03	0.1													Abi2	4E-07	0.2	Rexo1	6E-09	0.1												
Mrpl44	1E-03	0.1	Sec24a	9E-03	0.1													Dctd	2E-06	0.2	Mapk9	6E-07	0.1												
Ddb2	2E-01	0.1	Eif3g	2E-04	0.1													Serpine1	6E-10	0.2	1110004F10Rik	1E-06	0.1												
Pptc7	3E-03	0.1	Kin	2E-04	0.1													Noml	5E-04	0.2	Rap2b	3E-07	0.1												
Suclg2	8E-03	0.1	Zbtb24	4E-03	0.1													Cd9	1E-08	0.2	Ndufaf6	9E-10	0.1												
Hras	1E-05	0.1	Ap5m1	2E-02	0.1													Capn5	6E-05	0.2	Ift27	4E-06	0.1												
Them4	1E-03	0.1	Zfp407	7E-04	0.1													Znrd2	3E-03	0.2	Barx1	9E-06	0.1												
Psmc1	5E-04	0.1	Ppp4r1	3E-03	0.1													Lpar1	8E-05	0.2	Dennd4c	3E-06	0.1												
Prkacb	3E-03	0.1	Map3k4	1E-03	0.1													Atp2a2	4E-07	0.2	Hk2	8E-04	0.1												
Pphln1	6E-04	0.1	Nup93	2E-02	0.1													Zbtb5	2E-08	0.2	Tmem38a	4E-10	0.1												
Trim13	6E-03	0.1	Jmjd8	1E-02	0.1													Plac9b	7E-07	0.2	Ccnc	3E-06	0.1												
Hsf1	1E-02	0.1	Gpd2	2E-02	0.1													Car1	8E-05	0.2	Arhgap21	3E-11	0.1												
Ddx6	2E-04	0.1	Slc30a6	2E-04	0.1													Ilrun	1E-04	0.2	Prkci	5E-09	0.1												
Tceanc2	2E-04	0.1	Ncoa6	1E-03	0.1													Airn	3E-04	0.2	Fam20a	2E-09	0.1												
Lrch4	2E-02	0.1	Mta2	6E-03	0.1													Sbk1	3E-05	0.2	Eipr1	9E-10	0.1												
Pfdn4	1E-03	0.1	Rpl7l1	2E-04	0.1													Hikeshi	1E-05	0.2	9330154J02Rik	1E-05	0.1												
Srrt	3E-04	0.1	Zfp267	1E-03	0.1													Irf2bpl	8E-05	0.2	Fam122a	8E-06	0.1												
Sh2b3	8E-02	0.1	Tank	2E-04	0.1													Yeats2	2E-06	0.2	Slc12a4	1E-10	0.1												
Ubxn8	9E-04	0.1	Csnk1g1	2E-03	0.1													Fam98a	6E-06	0.2	Pigo	1E-05	0.1												
Msl1	6E-04	0.1	Klhl21	2E-02	0.1													Six1	1E-07	0.2	Gm45338	2E-07	0.1												
Hint2	2E-05	0.1	Rdh11	2E-01	0.1													Phc2	7E-06	0.2	Gm49463	7E-05	0.1												
Mapre1	7E-05	0.1	Rprd1a	1E-02	0.1													Tdg	1E-04	0.2	Mrpl50	8E-06	0.1												
Vps13d	2E-03	0.1	Arl5b	2E-03	0.1													Nsun2	2E-05	0.2	Wars	2E-07	0.1												
Marveld1	5E-04	0.1	Abt1	1E-03	0.1													Nrg1	4E-12	0.2	Anxa5	4E-12	0.1												
Adnp	2E-03	0.1	Armt1	7E-01	0.1													Tex14	9E-12	0.2	Igtp	2E-06	0.1												
Plxnb2	4E-04	0.1	Psmd4	3E-07	0.1													Cpsf1	7E-08	0.2	Eif3d	3E-03	0.1												
Ssbp3	4E-04	0.1	Zfp574	1E-01	0.1													Edf1	2E-12	0.2	Hnrnph2	4E-06	0.1												
Yaf2	2E-03	0.1	Zfp398	7E-04	0.1													Tmem189	2E-07	0.2	2700062C07Rik	3E-05	0.1												
Brd2	8E-05	0.1	Gatad1	4E-04	0.1													Gdpgp1	6E-05	0.2	Zbtb10	6E-11	0.1												
Slc25a28	2E-02	0.1	Paip2	1E-07	0.1													Eif2b2	4E-07	0.2	Fam149b	3E-06	0.1												
Tceal8	2E-05	0.1	Bcl6	5E-04	0.1													Psmb2	8E-10	0.2	Slc25a32	7E-09	0.1												
Srsf4	2E-03	0.1	Tent4a	1E-02	0.1													Repin1	7E-05	0.2	Myo9b	4E-07	0.1												
Evi5l	1E-02	0.1	Fgfbp3	3E-02	0.1													Trp53i13	9E-07	0.2	Mrps23	2E-07	0.1												
Zrsr2	2E-04	0.1	Abcd3	2E-03	0.1													Tnpo1	1E-06	0.2	Pcnp	4E-08	0.1												
Krcc1	2E-04	0.1	Tsr2	1E-02	0.1													Slc35e3	1E-07	0.2	Psma7	6E-09	0.1												
Bcas3	6E-04	0.1	Dnajb12	4E-03	0.1													Ppfia1	5E-05	0.2	Eif4ebp3	7E-08	0.1												
Nckap5l	2E-02	0.1	Ube2m	9E-06	0.1													Chchd4	2E-07	0.2	Cops8	1E-06	0.1												
Ube2z	6E-03	0.1	Mtif3	4E-03	0.1													Rab42	8E-08	0.2	Rap2a	4E-10	0.1												
Rab40c	9E-05	0.1	Wdr33	4E-03	0.1													Syngr2	2E-07	0.2	Bbs7	7E-10	0.1												
Zfhx4	1E-04	0.1	Bcl7b	1E-03	0.1													Dnajc4	2E-04	0.2	Znrd1as	2E-06	0.1												
Tmem128	1E-03	0.1	Rab10os	1E-03	0.1													Shld2	3E-09	0.2	Scrn2	2E-07	0.1												
Dync1h1	6E-05	0.1	Ext2	8E-04	0.1													Senp5	2E-05	0.2	Rassf8	8E-09	0.1												
Dnajc3	8E-07	0.1	Tmem134	4E-06	0.1													Gm7030	1E-08	0.2	Me3	2E-08	0.1												
Erlec1	7E-06	0.1	Secisbp2l	3E-03	0.1													Tmem267	2E-05	0.2	Yars	5E-08	0.1												
Vapb	4E-05	0.1	Mtmr12	1E-02	0.1													Eif4ebp1	7E-06	0.2	Mrpl17	1E-05	0.1												
Rab8b	3E-04	0.1	Dnajc30	8E-05	0.1													Cct5	2E-06	0.1	Glud1	6E-10	0.1												
Fus	1E-05	0.1	Trip4	8E-04	0.1													Crot	5E-01	0.1	Actr6	3E-05	0.1												
Pcx	2E-09	0.1	Thap2	6E-03	0.1													Gnpnat1	3E-04	0.1	Arl14ep	2E-03	0.1												
Ube2q1	1E-03	0.1	Mtif2	6E-04	0.1													Tle4	9E-06	0.1	Atxn1l	2E-09	0.1												
Zfp532	2E-03	0.1	Rbm25	8E-05	0.1													Clock	3E-05	0.1	Ppcdc	3E-05	0.1												
Utp15	3E-03	0.1	Fam199x	7E-02	0.1													Tmem128	1E-05	0.1	Senp5	2E-04	0.1												
Mgat5	6E-03	0.1	Esco1	5E-03	0.1													Stag2	1E-05	0.1	Knop1	3E-06	0.1												
Rpl5	3E-11	0.1	Scaf4	3E-03	0.1													Mindy3	8E-08	0.1	Ap3m1	1E-06	0.1												
Enpp4	3E-02	0.1	Dnm2	4E-04	0.1													Il1rapl1	3E-06	0.1	Col6a1	6E-08	0.1												

Limb Mesenchyme					
Control			NICD2		
Gene	p	FC	Gene	p	FC
Igbp1	3E-03	0.1	Ppp1r8	1E-03	0.1
Mplkip	1E-04	0.1	Drg1	2E-03	0.1
Cep68	2E-03	0.1	Pdcd6ip	4E-04	0.1
Tmed1	1E-03	0.1	Nek7	2E-03	0.1
Mrps18b	2E-03	0.1	Smurf1	4E-03	0.1
Nipa2	5E-03	0.1	Tm7sf3	4E-03	0.1
Anapc7	1E-04	0.1	Erbin	2E-04	0.1
Arl6ip6	2E-03	0.1	Ubr1	2E-03	0.1
Zfyve21	8E-04	0.1	Phf23	7E-04	0.1
Pcyt1b	6E-02	0.1	Pan3	3E-02	0.1
Angel2	1E-03	0.1	Rrp9	1E-02	0.1
Vps4a	1E-02	0.1	Btf3l4	6E-04	0.1
C1ql3	4E-02	0.1	Snrnp70	2E-04	0.1
Ctnnbip1	4E-03	0.1	Nucb2	1E-03	0.1
Ube3c	2E-03	0.1	Ncbp1	5E-04	0.1
Afap1	7E-05	0.1	Gpalpp1	8E-04	0.1
Mipol1	2E-03	0.1	Nmd3	3E-04	0.1
Ythdf1	8E-04	0.1	Trim28	2E-02	0.1
C2cd3	2E-03	0.1	Dpp9	4E-03	0.1
Mllt10	1E-04	0.1	Cul5	7E-04	0.1
Klhdc4	3E-03	0.1	Rufy1	2E-02	0.1
Rpl12	4E-10	0.1	Ptges2	2E-04	0.1
Ick	3E-03	0.1	Dis3	2E-02	0.1
4930453N24Rik	2E-03	0.1	Ssbp1	2E-04	0.1
Clip2	8E-03	0.1	Cfap36	8E-05	0.1
Txndc5	1E-05	0.1	H19	2E-01	0.1
Klf2	2E-03	0.1	Ppig	2E-04	0.1
Ninl	3E-04	0.1	Bet1	1E-03	0.1
Nat14	5E-04	0.1	Msh3	9E-04	0.1
Fry	1E-07	0.1	Gtf2h2	2E-04	0.1
Lrfn4	2E-04	0.1	Riok2	7E-03	0.1
Ap4e1	8E-03	0.1	Rfx7	2E-04	0.1
Lclat1	8E-03	0.1	Vps52	6E-03	0.1
Son	8E-08	0.1	Cwc27	2E-03	0.1
Stk35	9E-03	0.1	Mxra8	7E-05	0.1
Gdi2	3E-06	0.1	Mtf1	1E-02	0.1
Rnf114	3E-03	0.1	Nme4	6E-04	0.1
Pkn1	1E-02	0.1	Ubxn8	1E-03	0.1
Qtrt2	4E-03	0.1	Tcf12	3E-05	0.1
Nos1ap	7E-02	0.1	Inka2	3E-02	0.1
Gskip	8E-03	0.1	Ube2g1	9E-04	0.1
Rngtt	2E-03	0.1	Lysmd4	2E-03	0.1
Ankrd13c	4E-04	0.1	Cnot6	4E-03	0.1
Ranbp9	5E-03	0.1	Mgat1	7E-03	0.1
Dnhd1	1E-02	0.1	Polr1a	1E-03	0.1
Amy1	3E-03	0.1	Pin1	1E-04	0.1
Hivep2	5E-07	0.1	Zfp513	3E-03	0.1
Nob1	8E-04	0.1	Kras	1E-04	0.1
Gramd3	7E-04	0.1	Rc3h1	5E-04	0.1
Ddx17	6E-06	0.1	Dus4l	3E-03	0.1
Mfsd3	1E-02	0.1	Myo5a	5E-03	0.1
Dnajb12	1E-03	0.1	Triqk	3E-05	0.1
Nubpl	7E-04	0.1	Pi4k2a	8E-03	0.1
E2f6	1E-03	0.1	Nol12	3E-03	0.1
Sec24b	8E-04	0.1	Phospho2	5E-03	0.1
Inpp5f	6E-04	0.1	Ruvbl2	6E-05	0.1
Lzts2	5E-03	0.1	Pggt1b	2E-04	0.1
Fam8a1	6E-03	0.1	Pttg1ip	2E-03	0.1
Tada2b	2E-02	0.1	Camkmt	1E-01	0.1
Trim23	2E-03	0.1	Slc25a27	5E-02	0.1
Stk25	2E-03	0.1	Rabl3	7E-03	0.1
Dkc1	1E-03	0.1	Otub1	4E-04	0.1
1110065P20Rik	4E-05	0.1	Cycs	2E-08	0.1
Hmgn3	1E-06	0.1	Ncald	2E-01	0.1
Zfp560	7E-04	0.1	Dnajb4	1E-02	0.1
Rad9b	3E-02	0.1	Prkar1a	4E-05	0.1
Smad7	2E-03	0.1	Stard3	7E-03	0.1
Ik	3E-04	0.1	Thap1	6E-02	0.1
Anp32b	1E-10	0.1	Setd3	6E-03	0.1
Xpo1	5E-04	0.1	Slc25a36	1E-02	0.1
Pabpn1	3E-04	0.1	Ccdc9	1E-02	0.1
Trp53bp2	5E-03	0.1	Faim	1E-03	0.1
Pes1	1E-02	0.1	Ufsp2	2E-03	0.1

Chondrogenic					
Control			NICD2		
Gene	p	FC	Gene	p	FC

Fibroblast					
Control			NICD2		
Gene	p	FC	Gene	p	FC

Fibroblast?					
Control			NICD2		
Gene	p	FC	Gene	p	FC
Nfkbil1	2E-06	0.1	Map3k12	3E-06	0.1
Efnb1	2E-04	0.1	Nfyc	6E-08	0.1
Man1a2	5E-06	0.1	Cic	5E-07	0.1
Slc25a46	3E-03	0.1	Rpp25l	2E-05	0.1
Tmem69	3E-05	0.1	Nmd3	1E-07	0.1
Gm32856	2E-02	0.1	Micos10	5E-13	0.1
Rab5if	2E-08	0.1	Abhd17c	7E-08	0.1
Trp53inp1	6E-02	0.1	Polr2i	5E-06	0.1
Ccdc130	8E-08	0.1	Mpzl1	6E-10	0.1
Fkbp1a	2E-05	0.1	B4galt5	3E-14	0.1
Gm49463	1E-03	0.1	Uchl1	5E-20	0.1
Ecd	4E-04	0.1	Mdm4	2E-05	0.1
Ier3ip1	2E-05	0.1	Xiap	1E-06	0.1
Dhx29	2E-06	0.1	Stac	5E-12	0.1
Hspg2	3E-06	0.1	Rrp7a	2E-04	0.1
Flrt2	2E-06	0.1	Maoa	1E-06	0.1
Rad23a	2E-05	0.1	Scaf4	1E-08	0.1
Cops7a	5E-02	0.1	Tiprl	1E-04	0.1
Tmem248	1E-05	0.1	Aste1	7E-07	0.1
Elp5	5E-07	0.1	Rars	9E-05	0.1
Vdac2	6E-11	0.1	Tbc1d7	7E-09	0.1
Pkdcc	2E-04	0.1	Nt5c2	8E-06	0.1
Kat2b	1E-08	0.1	Zhx2	4E-08	0.1
Nkap	1E-05	0.1	Nsd3	5E-08	0.1
Mlec	1E-09	0.1	Adcy2	5E-13	0.1
Nadk	6E-04	0.1	Map1b	2E-07	0.1
Foxn2	2E-06	0.1	Efcab2	6E-05	0.1
Ciao2b	2E-06	0.1	Taok2	3E-09	0.1
G3bp2	4E-06	0.1	Rbm41	3E-05	0.1
Siah2	3E-08	0.1	Ccnt1	4E-06	0.1
Hspbp1	1E-04	0.1	Cspp1	1E-07	0.1
Mfap1a	2E-03	0.1	Cdk17	1E-08	0.1
Mta1	2E-03	0.1	Ndufaf4	2E-06	0.1
Chsy3	1E-07	0.1	Nop14	8E-06	0.1
Ifi35	4E-06	0.1	Prrc2c	1E-08	0.1
Polr2m	4E-08	0.1	Spink10	9E-07	0.1
Casc3	7E-06	0.1	Nosip	2E-08	0.1
Mgst3	5E-05	0.1	Tnfsf11	2E-10	0.1
Ddr2	4E-04	0.1	Grin2d	1E-04	0.1
Samd4b	1E-05	0.1	Zswim7	7E-09	0.1
Srp9	1E-10	0.1	Apba1	2E-08	0.1
Mtcp1	3E-04	0.1	Med7	1E-07	0.1
Cd55	2E-11	0.1	Plec	1E-08	0.1
Ralb	5E-08	0.1	Srp9	3E-12	0.1
Snrnp48	2E-06	0.1	Nup98	3E-07	0.1
Adcy6	3E-06	0.1	Ccar1	4E-06	0.1
Taf5l	8E-08	0.1	Armcx2	8E-09	0.1
Bcl2l1	2E-07	0.1	Ebna1bp2	1E-04	0.1
Slc4a2	7E-05	0.1	Med26	1E-06	0.1
Pctp	4E-04	0.1	Emc1	3E-07	0.1
Cmpk1	9E-07	0.1	Nubpl	7E-07	0.1
Degs1	7E-06	0.1	Dnm2	3E-03	0.1
Polr2a	2E-07	0.1	Tnpo1	3E-07	0.1
Dmap1	1E-08	0.1	2410002F23Rik	2E-05	0.1
Ppid	6E-03	0.1	Grpel1	5E-09	0.1
Trio	5E-04	0.1	Appl1	1E-09	0.1
Lgalsl	3E-06	0.1	Map2k3	1E-08	0.1
Nfkb2	2E-07	0.1	Snrnp48	2E-05	0.1
Zfpm1	1E-05	0.1	Cdc42se1	5E-06	0.1
Sp1	1E-04	0.1	Layn	5E-08	0.1
Adcy2	2E-07	0.1	Zrsr2	1E-05	0.1
Gm15261	1E-05	0.1	Vps35	3E-08	0.1
Zfp982	9E-05	0.1	Bckdhb	9E-03	0.1
Ndufaf5	1E-09	0.1	Fam3c	3E-06	0.1
Psmc6	4E-06	0.1	Ndufb6	2E-08	0.1
Ift22	1E-06	0.1	Rnf217	3E-08	0.1
Stk25	2E-03	0.1	Gngt2	8E-10	0.1
Akap1	3E-05	0.1	Phf11d	5E-09	0.1
Pan2	1E-04	0.1	Ralb	5E-07	0.1
Cdk9	2E-07	0.1	Tnfrsf21	7E-07	0.1
Arhgef19	3E-06	0.1	Cul4b	5E-05	0.1
Trmt61b	3E-08	0.1	Ddit4l	1E-12	0.1
Ralbp1	1E-06	0.1	Gas8	6E-07	0.1

Undefined					
Control			NICD2		
Gene	p	FC	Gene	p	FC

Articular/Synovial Fibroblast					
Control			NICD2		
Gene	p	FC	Gene	p	FC

Limb Mesenchyme					
Control			NICD2		
Gene	*p*	FC	Gene	*p*	FC
Parl	8E-03	0.1	Agl	3E-03	0.1
Pigc	1E-03	0.1	Dgkd	7E-04	0.1
Znrd1as	1E-03	0.1	Cdc37l1	6E-04	0.1
Ybx1	1E-10	0.1	Sppl2a	2E-03	0.1
Apex1	2E-04	0.1	Supt3	2E-02	0.1
Wdr33	9E-05	0.1	Gm26532	2E-01	0.1
Serbp1	6E-11	0.1	Rapgef6	2E-03	0.1
Rpp14	3E-03	0.1	Nemf	3E-03	0.1
Slc26a11	2E-02	0.1	Adk	2E-04	0.1
Eif1ax	3E-07	0.1	Rmnd1	1E-03	0.1
2010001A14Rik	6E-03	0.1	Wdr13	8E-03	0.1
Wbp1	3E-04	0.1	Fam71e1	6E-02	0.1
Recql5	1E-02	0.1	Smim3	8E-03	0.1
Rnf14	9E-03	0.1	Emc4	4E-03	0.1
Ufd1	2E-03	0.1	Gpn3	2E-03	0.1
Ints3	2E-03	0.1	Mid1	2E-10	0.1
Pin1	7E-05	0.1	Mtor	4E-03	0.1
Ptpn11	8E-04	0.1	Timm21	5E-03	0.1
Osbpl11	3E-03	0.1	Zfp157	5E-03	0.1
Laptm4a	2E-09	0.1	Strn	1E-01	0.1
Fam120c	1E-03	0.1	Cwf19l2	6E-03	0.1
Akap7	2E-05	0.1	Hnrnpk	8E-07	0.1
Zfp386	4E-04	0.1	1110038B12Rik	3E-04	0.1
			Fam118b	1E-03	0.1
			Nfs1	9E-03	0.1
			Osbpl5	7E-03	0.1
			Mrps27	8E-03	0.1
			Zfp91	3E-05	0.1
			Eif4h	1E-03	0.1
			Oip5os1	5E-05	0.1
			Susd6	3E-04	0.1
			Igf2bp3	2E-03	0.1
			Ube2w	2E-03	0.1
			2310014F06Rik	1E-02	0.1
			Zfp553	5E-04	0.1
			Lztfl1	4E-03	0.1
			Bod1	2E-03	0.1
			Zmym5	4E-04	0.1
			Pgm2	7E-02	0.1
			Tcerg1	2E-03	0.1
			Asnsd1	2E-03	0.1
			Chchd3	1E-03	0.1
			Fcho2	9E-04	0.1
			Lin52	3E-02	0.1
			Slc35a5	5E-02	0.1
			Gabarapl2	1E-05	0.1
			Rdh13	4E-01	0.1
			Dlg3	6E-03	0.1
			Rpp38	2E-03	0.1
			Fktn	8E-03	0.1
			Exd2	3E-02	0.1
			Elp4	3E-02	0.1
			Grb2	2E-03	0.1
			Rab1a	3E-04	0.1
			Sppl3	1E-03	0.1
			Maco1	2E-03	0.1
			Mrps18b	3E-03	0.1
			Hypk	1E-03	0.1
			Matr3	2E-03	0.1
			Gnai1	1E-03	0.1
			Desi1	2E-03	0.1
			Dda1	4E-03	0.1
			Kat14	2E-02	0.1
			Dph3	1E-04	0.1
			Mtmr10	2E-03	0.1

Chondrogenic					
Control			NICD2		
Gene	*p*	FC	Gene	*p*	FC

Fibroblast					
Control			NICD2		
Gene	*p*	FC	Gene	*p*	FC

Fibroblast?					
Control			NICD2		
Gene	*p*	FC	Gene	*p*	FC
Stim2	5E-07	0.1	Psmd7	8E-05	0.1
Spr	4E-04	0.1	Tapt1	1E-08	0.1
Baiap2	3E-07	0.1	Man1a2	2E-08	0.1
Ephb4	2E-07	0.1	Nedd8	9E-14	0.1
Sptbn1	1E-08	0.1	Tspan9	5E-06	0.1
Ythdf2	9E-07	0.1	Cnnm3	4E-08	0.1
Cfap410	4E-05	0.1	Ube2d2a	9E-07	0.1
Cnbd2	1E-04	0.1	Dcxr	3E-01	0.1
Nr2f6	8E-03	0.1	Gm36033	3E-06	0.1
Mrpl20	2E-09	0.1	Spp1	1E-09	0.1
Smg7	2E-05	0.1	Ndfip2	5E-07	0.1
Psma2	1E-07	0.1	Gmpr2	5E-07	0.1
Dtnbp1	1E-05	0.1	Rbm19	2E-08	0.1
Tada3	6E-05	0.1	Polr3f	6E-10	0.1
Hinfp	7E-06	0.1	Sept5	2E-09	0.1
Fbf1	3E-06	0.1	Rab5c	2E-03	0.1
Tent2	2E-05	0.1	Bcas2	5E-06	0.1
Mtx2	8E-04	0.1	Tollip	7E-05	0.1
Ruvbl1	3E-03	0.1	Fry	4E-11	0.1
Zswim1	3E-04	0.1	Zfp995	9E-09	0.1
Pcm1	8E-06	0.1	Axin1	3E-05	0.1
Znrd1	4E-04	0.1	Efna2	4E-07	0.1
Tram1	3E-04	0.1	Rpn1	3E-08	0.1
Ap4e1	2E-03	0.1	Lama2	5E-06	0.1
Zfp605	1E-07	0.1	Tex14	2E-10	0.1
Tstd2	5E-04	0.1	Rhod	5E-06	0.1
Osr1	1E-05	0.1	Tyw5	6E-11	0.1
Tyw3	5E-05	0.1	Cxxc1	2E-11	0.1
Papss2	6E-12	0.1	Bhlhe41	2E-01	0.1
Rexo4	7E-07	0.1	Mllt10	2E-07	0.1
Dlst	1E-06	0.1	Atp5j	2E-18	0.1
Copg1	3E-04	0.1	Sdc4	2E-04	0.1
Mov10	4E-06	0.1	Fam135a	6E-07	0.1
Rap2a	7E-09	0.1	Acsf2	1E-07	0.1
Cyp4f16	7E-06	0.1	Pomt2	2E-08	0.1
Zrsr2	8E-06	0.1	Galnt10	1E-07	0.1
Zdhhc14	1E-05	0.1	Cdc37	1E-05	0.1
Hspb8	5E-03	0.1	Fam49b	2E-05	0.1
Tmbim4	8E-02	0.1	Ugp2	2E-06	0.1
Tubgcp5	3E-07	0.1	Fam118b	1E-06	0.1
Tmem98	4E-09	0.1	Rabgef1	5E-05	0.1
Ofd1	2E-08	0.1	Nop16	1E-05	0.1
Csnk1g1	5E-05	0.1	Cul2	2E-05	0.1
Rsl1d1	5E-03	0.1	Kcmf1	3E-05	0.1
Gfpt2	1E-08	0.1	Fdps	1E-09	0.1
Gm6133	1E-04	0.1	Anxa2	5E-14	0.1
Psmd12	2E-03	0.1	Gpbp1	5E-05	0.1
Gpkow	3E-07	0.1	Pfdn4	4E-05	0.1
Klf2	4E-10	0.1	Hspe1	2E-06	0.1
Chrd	5E-07	0.1	Pgm2	2E-13	0.1
2310011J03Rik	4E-04	0.1	Tram1	1E-04	0.1
Exosc3	7E-05	0.1	Arhgap17	4E-05	0.1
Rad17	8E-05	0.1	Eif2d	1E-07	0.1
Fam149a	3E-07	0.1	Oaz1	8E-16	0.1
B3galt1	1E-10	0.1	Eya3	8E-08	0.1
Cdk2ap2	3E-03	0.1	Bloc1s4	3E-07	0.1
Psmb4	4E-07	0.1	Ddx42	3E-06	0.1
Vgll3	4E-06	0.1	Tra2a	4E-06	0.1
Cul4b	1E-03	0.1	Sh3glb1	1E-11	0.1
Mrps14	3E-07	0.1	Rnf5	1E-07	0.1
Cdkl2	2E-04	0.1	Psmb4	1E-05	0.1
Mrpl57	4E-05	0.1	Ndufa10	9E-06	0.1
Rubcn	5E-05	0.1	Psmd6	9E-09	0.1
Glod4	2E-07	0.1	Abhd17b	8E-07	0.1
Prr13	3E-04	0.1	Wdr36	3E-05	0.1
Mettl16	3E-04	0.1	Ap4m1	7E-08	0.1
Ctu1	4E-05	0.1	Cnot7	1E-04	0.1
Rbck1	2E-06	0.1	St3gal3	1E-07	0.1
Csnk2b	1E-02	0.1	Gm43728	1E-06	0.1
Map7d1	3E-05	0.1	Tmem250-ps	1E-05	0.1
Flii	3E-04	0.1	Zfp143	5E-10	0.1
Derl2	2E-05	0.1	Lysmd2	2E-07	0.1
Trap1	4E-04	0.1	Ppp1cb	3E-05	0.1

Undefined					
Control			NICD2		
Gene	*p*	FC	Gene	*p*	FC

Articular/Synovial Fibroblast					
Control			NICD2		
Gene	*p*	FC	Gene	*p*	FC

Limb Mesenchyme						Chondrogenic						Fibroblast						Fibroblast?						Undefined						Articular/Synovial Fibroblast					
Control			NICD2			Control			NICD2			Control			NICD2			Control			NICD2			Control			NICD2			Control			NICD2		
Gene	*p*	FC	Gene	*p*	FC	Gene	*p*	FC	Gene	*p*	FC	Gene	*p*	FC	Gene	*p*	FC	Gene	*p*	FC	Gene	*p*	FC	Gene	*p*	FC	Gene	*p*	FC	Gene	*p*	FC	Gene	*p*	FC
																		Snapc1	5E-02	0.1	Phb	2E-06	0.1												
																		Rtn4	2E-05	0.1	Wac	6E-06	0.1												
																		Exoc5	1E-04	0.1	Rab31	4E-09	0.1												
																		Fam20a	6E-05	0.1	Polr2e	9E-06	0.1												
																		Xpnpep1	2E-04	0.1	Capza1	2E-05	0.1												
																		Jpx	2E-05	0.1	Tatdn2	7E-05	0.1												
																		Magee1	9E-06	0.1	Hmgcll1	1E-07	0.1												
																		Mrpl32	3E-04	0.1	Nmt1	2E-05	0.1												
																		Elob	3E-14	0.1	Smarcb1	1E-07	0.1												
																		Smcr8	3E-05	0.1	Rai1	2E-07	0.1												
																		Sun1	2E-06	0.1	Pigq	6E-09	0.1												
																		Rhbdl3	2E-04	0.1	Gm15834	7E-06	0.1												
																		Adam12	2E-23	0.1	Bcl7b	2E-07	0.1												
																		Hsd17b12	8E-06	0.1	Zfp523	8E-09	0.1												
																		Fermt2	3E-04	0.1	Gtf2a1	5E-07	0.1												
																		Tex2	3E-08	0.1	Chd4	4E-08	0.1												
																		Nol11	4E-03	0.1	Irak2	4E-06	0.1												
																		Lyrm4	3E-05	0.1	Zranb1	2E-05	0.1												
																		Ppip5k2	1E-02	0.1	Fam151b	2E-02	0.1												
																		Oaf	7E-04	0.1	Gm36198	1E-07	0.1												
																		1810044D09Rik	4E-04	0.1	Dock1	2E-05	0.1												
																		Lbh	3E-10	0.1	Isg20l2	3E-08	0.1												
																		Cux1	4E-06	0.1	Alas2	2E-04	0.1												
																		Ncbp2	3E-06	0.1	Sympk	5E-05	0.1												
																		Chic1	2E-02	0.1	Pla2g6	2E-03	0.1												
																		Uggt1	4E-05	0.1	Gm15564	5E-01	0.1												
																		Mkks	5E-04	0.1	Fnbp1	1E-06	0.1												
																		Slc44a3	2E-04	0.1	Zfp52	4E-09	0.1												
																		Nrbp2	3E-06	0.1	Dixdc1	6E-11	0.1												
																		Actr2	9E-03	0.1	Coprs	1E-05	0.1												
																		Nubpl	1E-06	0.1	Id1	6E-19	0.1												
																		Acy1	4E-04	0.1	Zmat3	5E-06	0.1												
																		Gm46430	1E-02	0.1	Impad1	2E-04	0.1												
																		Dixdc1	2E-07	0.1	Ralbp1	2E-08	0.1												
																		Tmem50b	1E-08	0.1	Herc2	2E-05	0.1												
																		Adnp2	2E-07	0.1	Klhl22	2E-03	0.1												
																		Fem1c	1E-05	0.1	Adnp	3E-04	0.1												
																		Csnk1e	6E-06	0.1	Slc4a4	1E-17	0.1												
																		Ssbp4	2E-04	0.1	Arhgap5	5E-06	0.1												
																		Eif3l	5E-06	0.1	Spop	2E-05	0.1												
																		Prpf38b	7E-05	0.1	Rpusd3	8E-07	0.1												
																		Hspa1a	2E-04	0.1	Mrps30	7E-07	0.1												
																		Eif5	4E-05	0.1	Susd6	1E-06	0.1												
																		Nploc4	1E-07	0.1	Xaf1	2E-05	0.1												
																		Ahsa2	1E-05	0.1	Zfp639	2E-05	0.1												
																		Afg3l1	7E-04	0.1	Psmc2	3E-05	0.1												
																		Uqcrc1	1E-04	0.1	Med28	9E-07	0.1												
																		Col4a1	2E-04	0.1	Ndufa12	2E-04	0.1												
																		Gng5	9E-11	0.1	Znhit1	6E-02	0.1												
																		Oaz1	2E-14	0.1	Smarce1	9E-05	0.1												
																		Ap1m1	2E-08	0.1	Safb2	4E-08	0.1												
																		Zfp956	1E-04	0.1	Actr2	4E-05	0.1												
																		Hnrnpc	4E-05	0.1	1700052K11Rik	2E-08	0.1												
																		Tmcc3	1E-03	0.1	Med21	2E-04	0.1												
																		Cpsf6	4E-05	0.1	Coq6	1E-08	0.1												
																		Anxa4	2E-03	0.1	Vldlr	5E-08	0.1												
																		Asb3	4E-05	0.1	Mrpl4	6E-04	0.1												
																		Ppp2ca	1E-04	0.1	Ptprs	9E-06	0.1												
																		Gemin8	3E-07	0.1	Elmsan1	2E-10	0.1												
																		Colgalt1	2E-05	0.1	Cpped1	3E-05	0.1												
																		Adrm1	2E-05	0.1	Ier3ip1	3E-09	0.1												
																		Hnrnpll	9E-04	0.1	Birc6	1E-06	0.1												
																		Lipo3	2E-07	0.1	Vps4a	6E-03	0.1												
																		Psma7	3E-06	0.1	Fbxl5	4E-06	0.1												
																		Trnt1	7E-03	0.1	Tsen54	7E-09	0.1												
																		Kdelr2	5E-07	0.1	Serpine1	2E-16	0.1												
																		Psmf1	1E-07	0.1	Atp2b1	4E-07	0.1												
																		Nop16	3E-04	0.1	Zswim1	3E-05	0.1												
																		Ctdnep1	5E-05	0.1	Fhod1	2E-06	0.1												
																		Car6	4E-03	0.1	Tstd2	6E-06	0.1												
																		Dnlz	1E-04	0.1	0610009B22Rik	8E-04	0.1												
																		Fam234b	1E-04	0.1	Nudt2	2E-03	0.1												
																		Isca2	3E-02	0.1	Parva	7E-07	0.1												

Limb Mesenchyme						Chondrogenic						Fibroblast						Fibroblast?						Undefined						Articular/Synovial Fibroblast					
Control			NICD2			Control			NICD2			Control			NICD2			Control			NICD2			Control			NICD2			Control			NICD2		
Gene	*p*	FC	Gene	*p*	FC	Gene	*p*	FC	Gene	*p*	FC	Gene	*p*	FC	Gene	*p*	FC	Gene	*p*	FC	Gene	*p*	FC	Gene	*p*	FC	Gene	*p*	FC	Gene	*p*	FC	Gene	*p*	FC
																		Twistnb	1E-05	0.1	Cox15	4E-09	0.1												
																		Tmem161b	2E-06	0.1	Eef1d	1E-07	0.1												
																		Elmsan1	2E-07	0.1	Ptprd	2E-08	0.1												
																		Elac2	2E-06	0.1	Sema3a	2E-09	0.1												
																		Mpzl1	7E-06	0.1	D930016D06Rik	2E-08	0.1												
																		Ppp2r5c	2E-05	0.1	Xab2	2E-04	0.1												
																		Gng10	2E-05	0.1	Gtf2h5	2E-08	0.1												
																		Tpp2	9E-06	0.1	B130034C11Rik	2E-04	0.1												
																		1110004F10Rik	2E-06	0.1	Bmp4	1E-07	0.1												
																		Lypla1	9E-04	0.1	Pid1	7E-11	0.1												
																		Atp13a3	9E-06	0.1	Cops4	3E-03	0.1												
																		Pdcd7	6E-07	0.1	Gnl3l	3E-03	0.1												
																		Sap130	2E-04	0.1	Srek1	4E-06	0.1												
																		Rpap3	4E-08	0.1	Slc25a42	2E-07	0.1												
																		Prdx4	3E-13	0.1	Hspbp1	1E-02	0.1												
																		Acadl	1E-06	0.1	Tapbpl	3E-07	0.1												
																		Gm10638	3E-04	0.1	Gm34455	1E-05	0.1												
																		Lxn	2E-02	0.1	Ppp2cb	4E-04	0.1												
																		Hectd2	2E-04	0.1	Rest	5E-05	0.1												
																		Zfp882	7E-04	0.1	Sp2	1E-10	0.1												
																		Fut8	2E-06	0.1	Gm11266	8E-08	0.1												
																		Parp9	2E-07	0.1	Klc1	4E-06	0.1												
																		Atp5b	5E-13	0.1	Znrf1	1E-03	0.1												
																		Camta1	9E-06	0.1	Cc2d2a	2E-04	0.1												
																		Pigq	7E-09	0.1	Atp6v0a2	1E-04	0.1												
																		Gtf3c1	6E-05	0.1	Nsun2	2E-05	0.1												
																		Rabggtb	2E-04	0.1	Ccdc97	5E-08	0.1												
																		Srm	3E-02	0.1	Zbtb8os	3E-03	0.1												
																		Dst	3E-05	0.1	Slc8a1	4E-16	0.1												
																		Nr2c2	2E-04	0.1	Capg	1E-04	0.1												
																		Psmc2	1E-03	0.1	D17H6S53E	2E-07	0.1												
																		Gpr89	1E-05	0.1	Atf5	7E-05	0.1												
																		Dcaf4	1E-04	0.1	Cpxm2	2E-10	0.1												
																		Slirp	5E-05	0.1	Zmym3	2E-07	0.1												
																		Ppp2r2a	1E-02	0.1	Gtf3c2	1E-09	0.1												
																		Meox2	8E-06	0.1	Car6	3E-03	0.1												
																		Cdc37	3E-04	0.1	Csnk2b	9E-03	0.1												
																		Fam49b	2E-05	0.1	Rnf187	1E-06	0.1												
																		D5Ertd579e	1E-04	0.1	Dhx58	4E-04	0.1												
																		Apex1	9E-02	0.1	Egfl7	9E-12	0.1												
																		Nt5m	7E-03	0.1	Usp6nl	2E-07	0.1												
																		Polr2b	5E-05	0.1	5530601H04Rik	3E-05	0.1												
																		Tspan14	3E-06	0.1	Pdk4	3E-09	0.1												
																		Cdh2	6E-15	0.1	Dhrs9	6E-05	0.1												
																		Hspa4	2E-04	0.1	Itgb1	3E-12	0.1												
																		Nop2	8E-06	0.1	Llgl1	9E-10	0.1												
																		Atp11b	7E-05	0.1	Paxbp1	3E-07	0.1												
																		Zfp574	2E-05	0.1	Dnmbp	4E-07	0.1												
																		Map9	1E-05	0.1	Zfp653	1E-01	0.1												
																		Mmachc	1E-03	0.1	Ufsp2	5E-02	0.1												
																		Atpsckmt	5E-06	0.1	Map7d1	7E-06	0.1												
																		Bap1	9E-06	0.1	Cwf19l2	8E-04	0.1												
																		0610010K14Rik	2E-03	0.1	Pdia5	3E-06	0.1												
																		Tomm20	3E-06	0.1	Ephx1	2E-04	0.1												
																		2610001J05Rik	3E-03	0.1	Retreg1	8E-08	0.1												
																		Gm13402	2E-04	0.1	Swi5	2E-05	0.1												
																		Mier3	3E-03	0.1	Rnf220	3E-05	0.1												
																		Kpna1	1E-04	0.1	Gtpbp2	5E-06	0.1												
																		Luc7l	7E-04	0.1	Gm16599	3E-05	0.1												
																		Castor2	3E-06	0.1	Daglb	3E-08	0.1												
																		Zfp287	6E-06	0.1	Olfml2a	2E-06	0.1												
																		Git2	7E-08	0.1	Lypla1	2E-06	0.1												
																		Ppp1r3e	3E-03	0.1	Necap1	5E-08	0.1												
																		Tomm34	2E-06	0.1	Pheta2	3E-04	0.1												
																		Nsd3	8E-05	0.1	Cep126	9E-07	0.1												
																		Abcf1	1E-04	0.1	Fxr2	7E-08	0.1												
																		Trim32	9E-06	0.1	Ccnk	4E-06	0.1												
																		Irx5	2E-03	0.1	Rcn2	6E-06	0.1												
																		Abhd17c	3E-08	0.1	Atp11b	3E-05	0.1												
																		Grpel2	1E-05	0.1	Aggf1	3E-05	0.1												
																		Pabpc1	2E-08	0.1	Afg3l1	1E-05	0.1												
																		Usp7	2E-03	0.1	Tyw3	4E-04	0.1												
																		Stbd1	3E-05	0.1	Xylt1	2E-18	0.1												

Limb Mesenchyme						Chondrogenic						Fibroblast					
Control			NICD2			Control			NICD2			Control			NICD2		
Gene	*p*	FC	Gene	*p*	FC	Gene	*p*	FC	Gene	*p*	FC	Gene	*p*	FC	Gene	*p*	FC

Fibroblast?					
Control			NICD2		
Gene	*p*	FC	Gene	*p*	FC
Pak2	1E-05	0.1	Cdk11b	7E-09	0.1
Gcn1	2E-04	0.1	Pacs2	8E-05	0.1
Hspe1	7E-04	0.1	Lyrm4	2E-08	0.1
Ndufaf4	3E-04	0.1	Twist1	3E-04	0.1
Mllt10	6E-05	0.1	Stim2	3E-07	0.1
Capza1	3E-07	0.1	Ruvbl1	4E-01	0.1
Gramd4	5E-04	0.1	Ripor1	3E-09	0.1
Ephx1	2E-04	0.1	Ciao2b	4E-06	0.1
Polr1b	7E-05	0.1	Mex3b	1E-06	0.1
Heatr6	3E-05	0.1	Ptges2	7E-06	0.1
Csf1	8E-04	0.1	Masp1	3E-08	0.1
Ppp4r2	2E-03	0.1	Alms1	7E-16	0.1
Sf3b2	4E-07	0.1	Psma2	4E-06	0.1
Zfp668	8E-04	0.1	Atpsckmt	5E-08	0.1
Rbm33	2E-03	0.1	Slc4a2	2E-05	0.1
Cox11	6E-08	0.1	Noa1	2E-04	0.1
Pid1	1E-07	0.1	Pcdh7	2E-09	0.1
Cpped1	6E-05	0.1	Dnttip2	9E-03	0.1
Tram2	1E-04	0.1	Ncbp2	3E-04	0.1
Wdr82	5E-05	0.1	Synj2bp	1E-03	0.1
Ift27	3E-03	0.1	Adamtsl1	4E-12	0.1
Nampt	5E-01	0.1	Pls3	4E-04	0.1
Ric3	1E-04	0.1	Mob2	2E-07	0.1
Vat1	2E-03	0.1	Parp11	6E-07	0.1
Bud31	4E-06	0.1	Lamb2	2E-05	0.1
Reps1	4E-05	0.1	Rexo4	1E-07	0.1
Synj1	2E-06	0.1	Tgfb2	5E-02	0.1
Sugp2	2E-08	0.1	Mrpl1	7E-04	0.1
Cops8	3E-04	0.1	Cnp	1E-09	0.1
0610009B22Rik	3E-03	0.1	Ahdc1	3E-04	0.1
Stard5	6E-08	0.1	Trappc6b	6E-05	0.1
Sh3gl1	3E-03	0.1	Mrps24	2E-07	0.1
Cdk11b	6E-05	0.1	Dennd5a	1E-04	0.1
Zfp598	4E-07	0.1	Arhgef40	2E-05	0.1
Ppp2r1b	5E-06	0.1	Fer	5E-05	0.1
Rpe	3E-07	0.1	Twf1	8E-04	0.1
Nmi	4E-07	0.1	Hexdc	2E-05	0.1
Pip5k1b	1E-08	0.1	Dars	1E-02	0.1
Rpl41	1E-18	0.1	Brd4	2E-06	0.1
Emc9	1E-03	0.1	Prr14l	1E-06	0.1
Xpnpep3	7E-06	0.1	Hspb6	1E-06	0.1
Etfdh	1E-06	0.1	Rad51d	3E-08	0.1
Tnfrsf23	1E-06	0.1	Xxylt1	2E-06	0.1
Vps50	6E-06	0.1	Etv6	2E-04	0.1
Usp15	2E-04	0.1	Cdc42ep2	3E-08	0.1
Frk	4E-04	0.1	Flywch1	3E-06	0.1
Ndufa11	7E-05	0.1	E130309D02Rik	1E-07	0.1
Zranb1	9E-06	0.1	Gm8098	2E-05	0.1
Aagab	3E-03	0.1	Fam49a	2E-06	0.1
Eif4a1	3E-03	0.1	Rnf6	9E-05	0.1
Rars	1E-04	0.1	Dnaja2	9E-04	0.1
Adam15	1E-04	0.1	Grpel2	5E-06	0.1
Zfp592	1E-05	0.1	Rcan1	6E-07	0.1
Plekhb2	2E-07	0.1	Gm16286	6E-05	0.1
Dstn	2E-06	0.1	Dnajc27	9E-07	0.1
2310009B15Rik	3E-05	0.1	St3gal2	3E-05	0.1
Ccng2	2E-06	0.1	Hspa4	7E-05	0.1
Pdcd6ip	1E-04	0.1	Eml3	8E-09	0.1
Rexo1	7E-05	0.1	Abcf1	5E-05	0.1
Pigu	2E-05	0.1	Gemin5	4E-05	0.1
C1qtnf2	4E-06	0.1	Nck1	2E-04	0.1
Trabd	2E-06	0.1	Ppil3	5E-01	0.1
Gm31763	9E-03	0.1	Snhg6	5E-04	0.1
Pdgfd	4E-06	0.1	Entpd7	3E-04	0.1
Capn15	1E-04	0.1	Ano8	4E-05	0.1
Cul4a	1E-04	0.1	Casc3	1E-06	0.1
Zmat2	1E-01	0.1	Prdx4	7E-13	0.1
Snf8	1E-05	0.1	Gstp1	1E-02	0.1
Amfr	6E-05	0.1	Klhdc2	1E-03	0.1
Phip	4E-08	0.1	Smad7	2E-05	0.1
Micall1	1E-03	0.1	Prmt3	4E-07	0.1
Cnot7	2E-05	0.1	Lrrc47	4E-04	0.1
Mrps24	4E-06	0.1	Gm12940	3E-03	0.1

Undefined						Articular/Synovial Fibroblast					
Control			NICD2			Control			NICD2		
Gene	*p*	FC	Gene	*p*	FC	Gene	*p*	FC	Gene	*p*	FC

Limb Mesenchyme						Chondrogenic						Fibroblast					
Control			NICD2			Control			NICD2			Control			NICD2		
Gene	*p*	FC	Gene	*p*	FC	Gene	*p*	FC	Gene	*p*	FC	Gene	*p*	FC	Gene	*p*	FC

Fibroblast?					
Control			NICD2		
Gene	*p*	FC	Gene	*p*	FC
Smn1	1E-05	0.1	Gm15261	9E-12	0.1
1500009L16Rik	3E-07	0.1	Smarcd1	4E-05	0.1
Irak1	1E-04	0.1	Gtf3c4	5E-09	0.1
Nudt9	3E-04	0.1	Fam110b	1E-07	0.1
Prr14l	9E-04	0.1	Ogfrl1	2E-09	0.1
Uqcc2	6E-07	0.1	Epn2	4E-06	0.1
Cxxc1	1E-07	0.1	Fam126a	2E-10	0.1
1190005I06Rik	7E-06	0.1	Mrpl47	4E-05	0.1
Ndufa4	1E-10	0.1	Tln1	3E-05	0.1
Snx27	2E-05	0.1	Igfbp6	6E-02	0.1
Trim24	1E-03	0.1	Kdelr3	9E-04	0.1
Fance	1E-05	0.1	Wwp1	1E-05	0.1
Mrpl50	1E-03	0.1	Scaf1	8E-05	0.1
Adamts10	1E-05	0.1	Sf3b6	7E-06	0.1
Dzip3	3E-05	0.1	Larp4b	2E-07	0.1
Micos10	1E-08	0.1	Rab32	8E-04	0.1
Col6a3	4E-05	0.1	Cox6a2	1E-05	0.1
Larp4b	6E-06	0.1	Tango2	1E-07	0.1
Oxsm	1E-05	0.1	Lrch1	2E-08	0.1
Xab2	1E-06	0.1	Trim30a	7E-07	0.1
Tomm22	7E-04	0.1	Ddx41	3E-03	0.1
Twnk	3E-06	0.1	Ssu72	1E-04	0.1
Nck2	6E-07	0.1	D830050J10Rik	2E-02	0.1
Tmem170	2E-05	0.1	Rpe	1E-07	0.1
Ctf1	4E-07	0.1	Plekha1	2E-07	0.1
Ap2a2	3E-04	0.1	Maip1	4E-04	0.1
Exoc1	2E-06	0.1	Phc2	2E-06	0.1
Elp6	4E-06	0.1	Mllt11	1E-05	0.1
Rapgef2	5E-04	0.1	Mex3d	1E-07	0.1
Taldo1	2E-06	0.1	Rbm34	3E-05	0.1
Rps6kb1	2E-04	0.1	Smap2	1E-04	0.1
Fam149b	2E-05	0.1	Insyn2a	6E-08	0.1
Wdr55	6E-06	0.1	Cep170	1E-03	0.1
Heatr5a	3E-05	0.1	Abhd8	5E-08	0.1
Gtf2h5	4E-05	0.1	Hipk1	1E-06	0.1
Necap1	7E-06	0.1	Eif2b2	5E-06	0.1
Ywhab	9E-07	0.1	Naa30	2E-04	0.1
Zim1	1E-11	0.1	Zfp595	2E-07	0.1
Fdxacb1	2E-04	0.1	Arhgef1	1E-06	0.1
Ifi30	3E-07	0.1	Pcbp1	2E-06	0.1
Scap	3E-06	0.1	Btg3	5E-05	0.1
Prg4	1E-10	0.1	Rad17	7E-06	0.1
Me1	3E-07	0.1	Dtx3l	2E-05	0.1
Ppp2r5e	2E-04	0.1	Usp12	1E-07	0.1
Rpusd3	6E-04	0.1	Gpr89	9E-07	0.1
Mex3d	7E-03	0.1	Virma	2E-03	0.1
Fer	7E-05	0.1	Cxxc5	5E-05	0.1
Slc35c1	5E-05	0.1	Zfp362	6E-09	0.1
Tra2a	1E-04	0.1	Smarcc2	2E-03	0.1
Mgat1	2E-04	0.1	Csk	2E-06	0.1
Zfp617	3E-06	0.1	Trim68	5E-06	0.1
Hddc2	6E-02	0.1	Rnf181	1E-07	0.1
Nr4a1	3E-03	0.1	Unc119b	1E-07	0.1
Nelfa	1E-06	0.1	Emc3	2E-05	0.1
Mphosph10	6E-07	0.1	Xpo7	7E-05	0.1
Eif4e2	3E-05	0.1	Ddx52	3E-03	0.1
Cndp2	4E-06	0.1	Ecm2	4E-09	0.1
Ostf1	1E-03	0.1	Vps26b	9E-03	0.1
Trim30a	2E-05	0.1	Gm48226	2E-06	0.1
Rgl2	8E-04	0.1	Elovl1	1E-04	0.1
Pdia5	2E-05	0.1	Rapgef2	1E-06	0.1
Arhgap35	2E-04	0.1	BC034090	2E-06	0.1
Erlin2	3E-05	0.1	Mob4	7E-04	0.1
Exo5	1E-06	0.1	Cc2d1b	3E-10	0.1
Tshz3	2E-05	0.1			
Zfp518b	2E-06	0.1			
Usp48	3E-05	0.1			
Vamp5	4E-03	0.1			
Cul3	3E-03	0.1			
Plaa	2E-04	0.1			
Anapc2	1E-04	0.1			
Ndufa10	3E-03	0.1			
Tsen54	3E-05	0.1			

Undefined						Articular/Synovial Fibroblast					
Control			NICD2			Control			NICD2		
Gene	*p*	FC	Gene	*p*	FC	Gene	*p*	FC	Gene	*p*	FC

Limb Mesenchyme						Chondrogenic						Fibroblast						Fibroblast?						Undefined						Articular/Synovial Fibroblast					
Control			NICD2			Control			NICD2			Control			NICD2			Control			NICD2			Control			NICD2			Control			NICD2		
Gene	*p*	FC	Gene	*p*	FC	Gene	*p*	FC	Gene	*p*	FC	Gene	*p*	FC	Gene	*p*	FC	Gene	*p*	FC	Gene	*p*	FC	Gene	*p*	FC	Gene	*p*	FC	Gene	*p*	FC	Gene	*p*	FC
																		Bcar3	6E-05	0.1															
																		Ints3	3E-06	0.1															
																		Cald1	2E-04	0.1															
																		Dido1	3E-04	0.1															
																		Hsf2	2E-07	0.1															
																		Inppl1	1E-06	0.1															
																		Vps13a	8E-05	0.1															
																		Tent4b	9E-07	0.1															
																		Gm17501	1E-07	0.1															
																		Phax	3E-02	0.1															
																		Rbm34	5E-06	0.1															
																		Coprs	5E-05	0.1															
																		Commd4	3E-02	0.1															
																		Epha3	4E-04	0.1															
																		Cyb5r4	1E-01	0.1															
																		Dnm2	5E-05	0.1															
																		Nedd4l	5E-06	0.1															
																		Med16	2E-06	0.1															
																		Psmg4	2E-04	0.1															
																		Zbtb8os	1E-04	0.1															
																		Ctbs	3E-06	0.1															
																		Fam13b	5E-04	0.1															
																		Sympk	6E-06	0.1															
																		Tor1aip2	3E-04	0.1															
																		Znhit2	6E-05	0.1															
																		Znrd1as	2E-07	0.1															
																		Mob3a	2E-08	0.1															
																		Ell	6E-07	0.1															
																		Gm45338	1E-05	0.1															
																		Slc30a4	1E-05	0.1															
																		Suox	7E-05	0.1															
																		C230035I16Rik	8E-04	0.1															
																		Rgs4	1E-04	0.1															
																		Itgb1	6E-08	0.1															
																		Cd82	1E-06	0.1															
																		Ogg1	2E-07	0.1															
																		Hnrnpr	1E-03	0.1															
																		Pfdn4	2E-02	0.1															
																		Nol10	1E-06	0.1															
																		Rnf187	1E-03	0.1															
																		Synpo	5E-05	0.1															
																		Ston1	5E-04	0.1															
																		Ccdc127	1E-02	0.1															
																		Adgrl2	3E-04	0.1															
																		Rbm19	4E-06	0.1															
																		Mfsd4b4	4E-04	0.1															
																		Ptrh1	1E-06	0.1															
																		Pmp22	2E-06	0.1															
																		Zbtb7c	2E-06	0.1															
																		Samd14	3E-06	0.1															
																		Phc1	1E-04	0.1															
																		Kank2	6E-06	0.1															
																		Srek1	2E-03	0.1															
																		B3gat3	4E-05	0.1															
																		Gm28403	6E-04	0.1															

Table S5. Differentially expressed genes between NICD2-expressing and control cells associated with mesenchymal, limb mesenchyme, articular/synovial fibroblast, chondrogenic, adipogenic, osteogenic, macrophages and fibroblast cells shown in Figure 9B and 9C. The number of cells expressing each gene and the intensity or level of expression is provided.

	Control	NICD2	Control	NICD2	NICD2/Control
Gene	Percent Expressed		Level of Expression		Expression
Foxc1	71.5	68.6	0.83	0.80	96.0%
Ebf3	49.3	46.6	0.48	0.46	96.4%
Prrx1	95.1	95.0	4.11	4.88	118.7%
Prrx2	90.8	88.6	2.05	1.81	88.4%
Trim2	59.4	53.5	0.49	0.42	85.5%
Gdf5	2.4	1.0	0.01	0.01	40.1%
Pdgfra	69.0	68.6	0.98	0.99	100.8%
Prg4	41.4	36.7	1.17	0.97	82.6%
Cd55	40.7	35.0	0.56	0.41	73.7%
Sox9	67.4	53.8	1.10	0.74	67.3%
Acan	7.7	7.0	0.09	0.07	77.9%
Col2a1	50.8	42.3	3.58	2.85	79.7%
Spp1	83.8	68.8	12.42	9.29	74.8%
Adipoq	5.3	3.5	0.30	0.27	88.9%
Pparg	27.7	38.7	0.30	0.51	169.0%
Grem1	15.3	9.5	0.29	0.14	47.0%
Sp7	0.8	0.4	0.00	0.00	54.1%
Runx2	59.7	58.1	0.76	0.73	96.1%
Lepr	1.8	1.7	0.01	0.01	100.5%
Cxcl12	90.0	90.2	6.25	6.90	110.5%
Bglap	0.7	0.6	0.00	0.01	156.9%
Clec11a	58.9	57.8	0.78	0.79	100.8%
Itga11	32.8	38.6	0.26	0.35	133.7%
Col1a1	99.1	98.4	32.35	33.55	103.7%
Lyz2	18.1	14.1	0.88	0.67	76.7%
Cx3cr1	4.1	4.0	0.02	0.02	99.1%
Calcr	0.0	0.0	0.00	0.00	116.0%
Acp5	14.1	16.4	0.08	0.10	138.5%
Bcl6	51.2	45.7	0.43	0.36	82.9%

	Control	NICD2	Control	NICD2	NICD2/Control
Gene	Percent Expressed		Level of Expression		Expression
Anxa8	24.11	20.03	0.25	0.21	82.7%
Slurp1	3.30	1.28	0.04	0.01	21.6%
Ociad2	24.12	17.12	0.15	0.11	69.4%
Dkk2	23.84	19.57	0.74	0.50	67.3%
Cxcl2	1.72	1.44	0.02	0.01	61.9%
Cxcl12	90.02	90.24	6.25	6.90	110.5%
Gas6	97.29	97.71	8.76	10.64	121.5%
Acvr2a	54.72	57.05	0.61	0.69	112.3%
Cxcl1	14.97	15.85	0.21	0.21	101.6%
Col15a1	6.07	11.61	0.06	0.11	186.0%
Pi16	2.39	1.80	0.02	0.01	69.2%
Cd55	40.73	35.05	0.56	0.41	73.7%
Cd248	77.88	78.79	1.59	1.71	107.9%
C7	0.08	0.08	0.00	0.00	116.1%
Fmo2	0.19	0.27	0.00	0.00	93.0%
Kitl	45.61	37.71	0.55	0.41	74.9%
Ccl11	0.26	0.26	0.00	0.00	75.6%
Smoc2	40.61	37.90	0.36	0.34	95.5%
Hsd11b1	3.93	4.57	0.02	0.02	112.7%
Prg4	41.42	36.74	1.17	0.97	82.6%
Clic5	0.39	0.24	0.00	0.00	87.3%
Itga6	15.55	13.27	0.09	0.08	83.0%
Gdf5	2.36	1.00	0.01	0.01	40.1%
Pdgfra	69.00	68.62	0.98	0.99	100.8%
Col1a1	99.07	98.45	32.35	33.55	103.7%
Col3a1	99.33	99.12	22.26	25.07	112.6%
Thy1	24.68	28.52	0.20	0.27	133.9%
Mcam	1.55	1.79	0.01	0.01	117.9%

Table S6. Differentially expressed genes in the undefined cluster between NICD2-expressing and control cells. The number of cells expressing each gene and the intensity or level of expression is provided.

	Control	NICD2	Control	NICD2	NICD2/Control
**Gene**	**Percent Expressed**		**Level of Expression**		**Expression**
Tnfsf11	5.5	25.3	0.03	0.17	527%
Itga8	13.5	30.3	0.07	0.26	355%
Rtl3	43.9	62.8	0.30	0.55	187%
4921539H07Rik	9.9	15.0	0.04	0.07	183%
Ccdc3	17.5	23.7	0.11	0.20	183%
Frmd5	13.8	19.8	0.12	0.22	179%
Rarres1	15.1	19.3	0.07	0.12	167%
Slitrk5	8.8	13.9	0.04	0.07	166%
Adh7	21.0	24.4	0.11	0.18	155%
Itga11	54.5	62.3	0.35	0.42	120%
Ndnf	64.5	67.0	0.88	1.02	116%
Tll1	16.3	16.5	0.15	0.17	115%
Eya1	36.6	36.5	0.29	0.33	114%
Mei4	17.8	16.0	0.08	0.09	113%
Cpxm2	52.0	54.0	0.39	0.43	111%
Calhm5	45.5	43.1	0.24	0.27	111%
Car9	23.6	22.4	0.13	0.14	108%
Aspn	96.3	95.8	1.77	1.86	105%
Aqp5	20.9	22.1	0.15	0.15	102%
Aplp1	39.6	37.4	0.21	0.21	100%
Perp	11.1	10.0	0.06	0.06	99%
Kcnt2	41.9	39.0	0.27	0.27	98%
Aldh1l2	86.9	82.3	0.83	0.79	95%
Cth	33.7	29.9	0.21	0.20	94%
Igfbp2	60.8	55.8	1.15	1.08	94%
Ptn	86.1	75.6	2.29	2.14	93%
Egfl6	69.7	64.2	1.51	1.40	93%
Kcnip4	86.5	76.2	2.37	2.17	92%
Otor	12.1	11.1	0.19	0.18	91%
Hsd17b1	40.5	35.6	0.21	0.19	90%
Trim66	14.1	12.6	0.06	0.06	89%
Ostn	40.3	39.0	0.43	0.38	89%
Igf1	78.7	73.6	1.39	1.22	88%
Hs3st3a1	21.7	14.3	0.11	0.09	87%
Slc7a3	24.0	17.5	0.10	0.09	84%
Trib3	62.4	54.1	0.41	0.35	84%
Pappa2	22.2	19.5	0.21	0.17	80%
Mkx	52.7	44.3	0.48	0.38	79%
Plxnc1	26.6	19.8	0.14	0.11	78%
Chac1	49.7	38.4	0.28	0.22	77%
Prss12	12.2	8.9	0.06	0.04	74%
Kctd16	19.5	12.4	0.18	0.13	73%
Sgcz	11.8	8.2	0.17	0.12	71%
Scn3a	14.1	9.8	0.07	0.05	70%
Meox1	44.9	32.9	0.50	0.33	66%
Aqp1	61.8	43.6	0.55	0.34	61%
Adamts3	24.4	14.9	0.20	0.11	58%
Gm2115	38.5	26.6	0.29	0.17	58%
Scx	48.6	27.1	0.32	0.19	57%
Epha5	18.9	10.8	0.21	0.11	53%

Table S7. Primers used for genotyping.

Allele	Strand	Sequence 5'-3'	Amplicon Size (bp)
Notch2 $^{tm1.1Ecan}$	Fwd	5'-CCCTTCTCTCTGTGCGGTAG-3'	WT = 308
	Rev	5'-CTCAGAGCCAAAGCCTCACTG-3'	*Notch2 $^{tm1.1Ecan}$* = 403
Notch2 ^{WT}	Fwd	5'-GCTCAGACCATTGTGCCAACCTAT-3'	100
	Rev	5'-CAGCAGCATTTGAGGAGGCGTAA-3'	
R26-NICD2	Fwd	5'-AAGGGACTGGCTGCTATTGG-3'	WT = 224<br>Rosa = 420
	Rev	5'-ATATCACGGGTAGCCAACGC-3'	
	WT Forward	5'-CTCTCCCAAAGTCGCTCTG-3'	
	WT Reverse	5'-TACTCCGAGGCGGATCACAAGC-3'	

Figure S1. Differential gene expression by the *Notch2*$^{tm1.1Ecan}$ mutation in epiphyseal chondrocytes determined by bulk RNA-Seq. RNA-Seq analysis of 50 most differentially expressed genes by the *Notch2*$^{tm1.1Ecan}$ mutation shown as means ± SD and individual values of the number of fragment mapping to the gene of interest; n = 3 - 4 in control (white bars) and *Notch2*$^{tm1.1Ecan}$ (grey bars) biological replicates; $p < 0.05$ for all values by unpaired t tests.

Figure S1

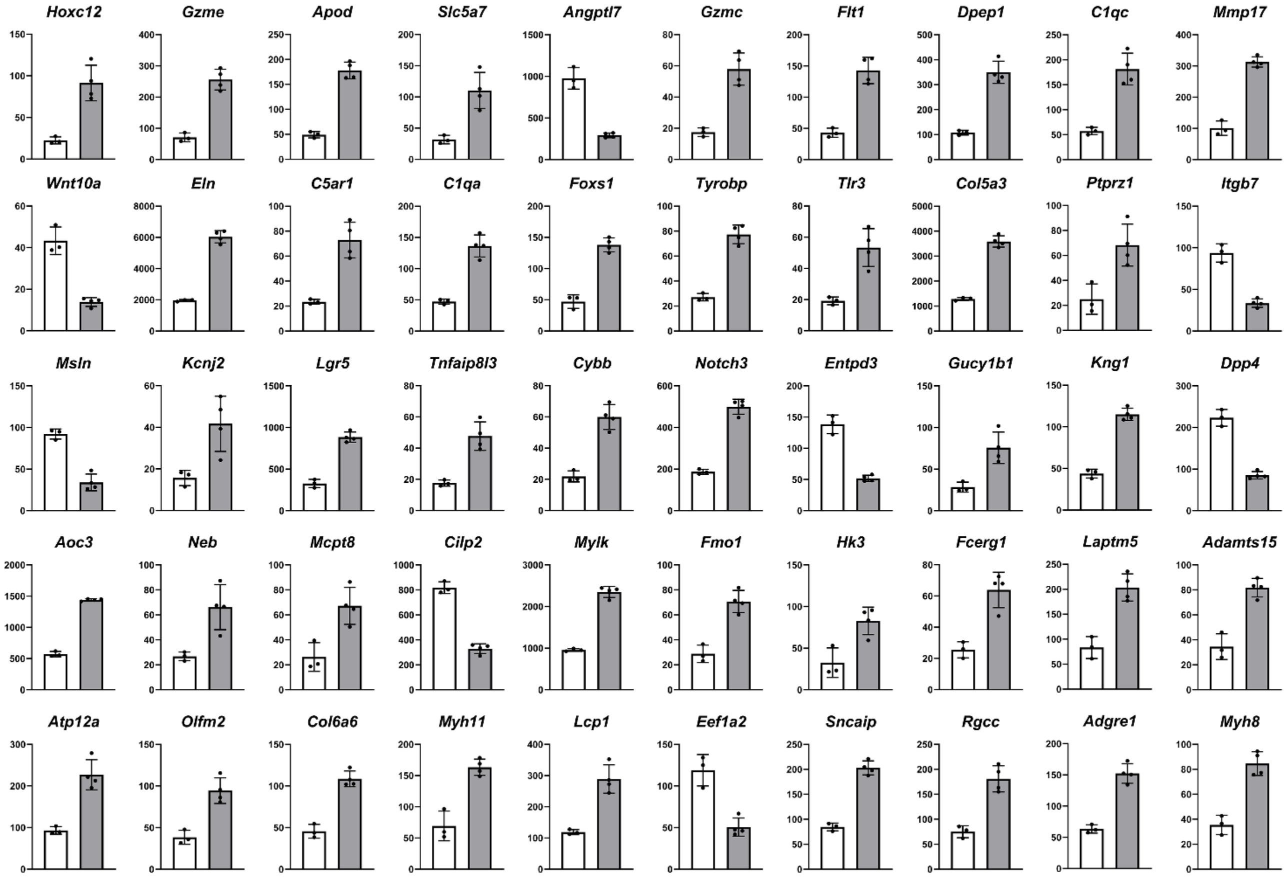

Figure S2. Differential gene expression by NICD2 activation in epiphyseal chondrocytes determined by bulk RNA-Seq. RNA-Seq analysis of 50 most differentially expressed genes by NICD2 shown as means ± SD and individual values of the number of fragments mapping to the gene of interest; n = 4 in control (white bars) and NICD2-expression (grey bars) biological replicates; $p < 0.05$ for all values by unpaired t tests.

Figure S2

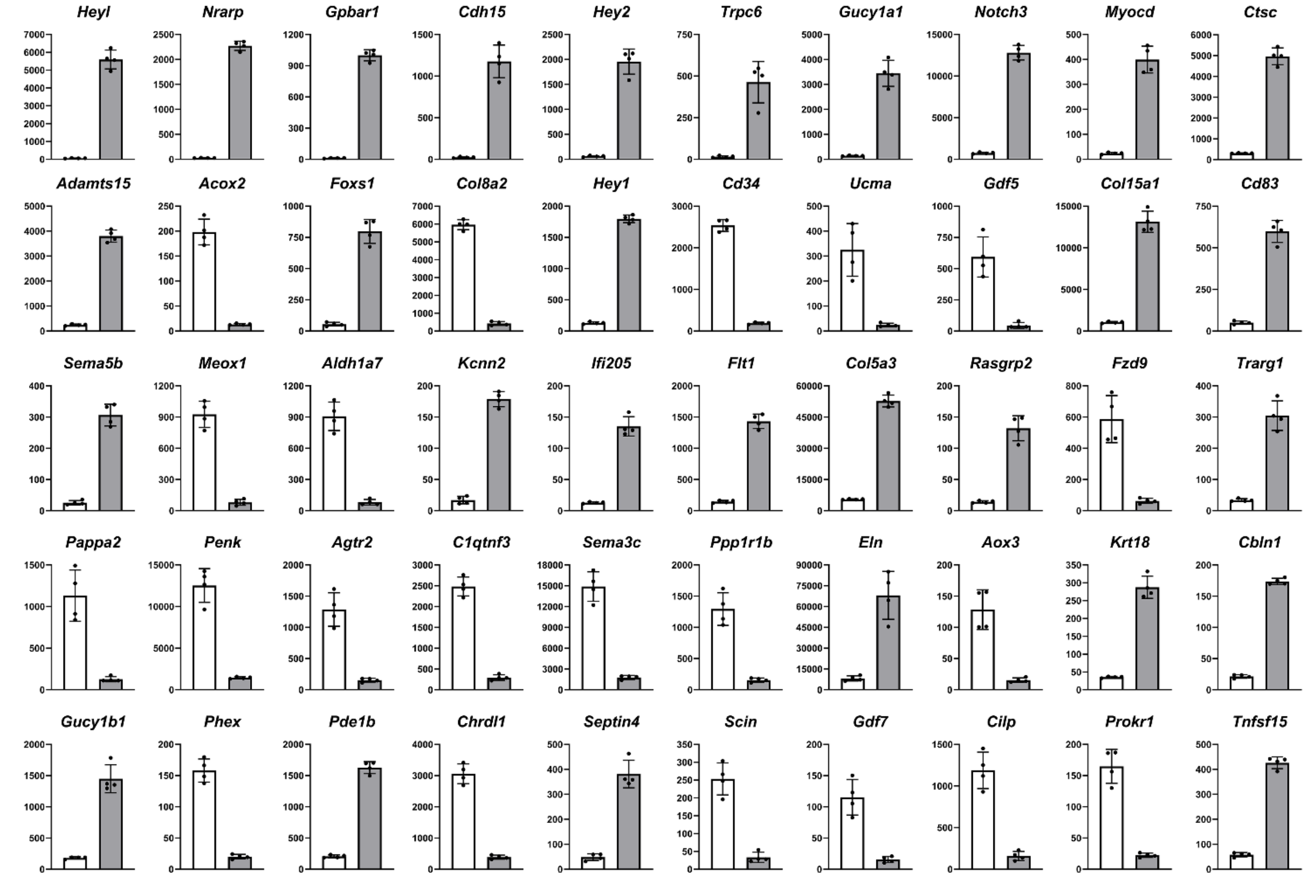

Figure S3. Dot plot displaying the expression of *Notch1*, *2*, *3* and *4*, *Hes1*, *Hey1*, *2* and *l* and *Jagged1*. In A. in clusters from control and *$Notch2^{tm1.1Ecan}$* cells or B. in clusters from control and *NICD2*-epxressing cells. Clusters were identified by uniform manifold approximation from experimental and control epiphyseal chondrocytes. In C. differential gene expression of *Hes1*, *Hey1*, *Hey2* and *Heyl* in control and *NICD2*-expressing epiphyseal chondrocytes. Red denotes higher and blue denotes lower than average expression, and the size of the circle represents the percentage of cells expressing each gene.

Figure S3

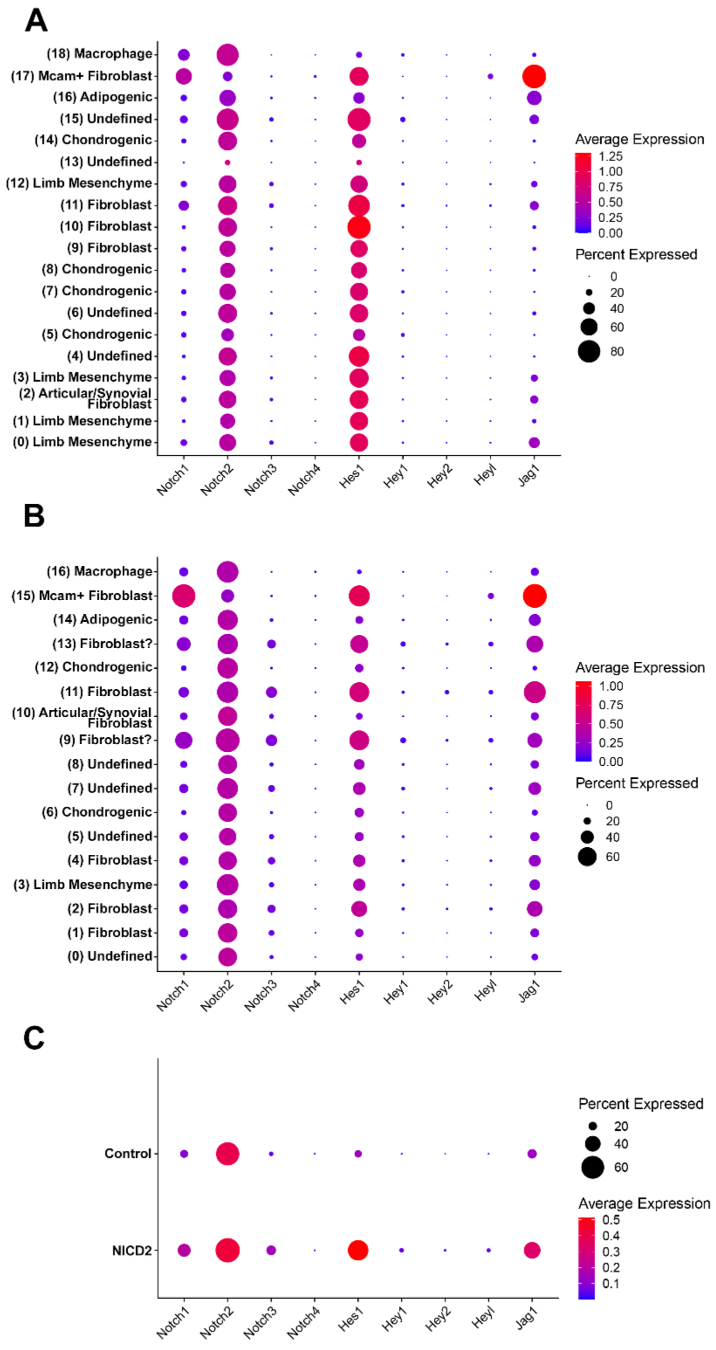

Figure S4. Ingenuity pathway analysis (IPA) of the articular/synovial fibroblast cluster from chondrocyte-enriched cells from *Notch2$^{tm1.1Ecan}$* and control mice. IPA of canonical pathways under the genes and chemical category was performed at a log2FC1, *p*<0.05, *p* adjusted value <0.1 and displayed for the top 30 pathways affected and shows induction of the osteoarthritis pathway in *Notch2$^{tm1.1Ecan}$* cells**.**

Figure S4

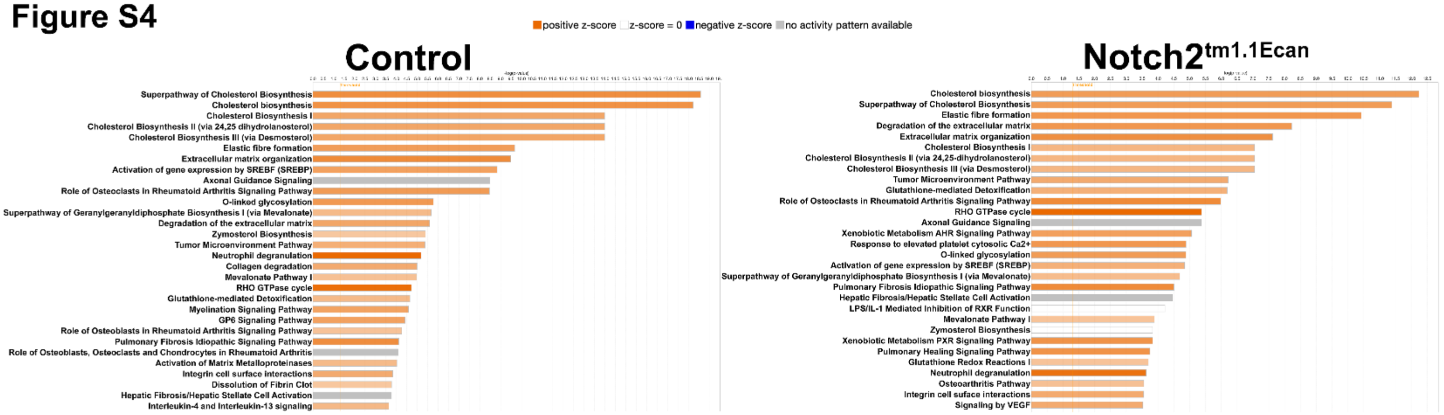

Supplement: Supplementary Material [file mmc1.pdf]
